# ESP Abstracts 2013

**DOI:** 10.1007/s00428-013-1444-y

**Published:** 2013-08-08

**Oral Free Paper Sessions**

Sunday, 1 September 2013, 08.30–12.00, Room 5C

**OFP-01 Oral Free Paper Session Breast Pathology**

**OFP-01-001**

**Fully automated FISH staining and digital analysis of HER2 in breast cancer: A validation study**

E. van der Logt ^*^, D. A. J. Kuperus, J. W. van Setten, M. C. van den Heuvel, J. E. Boers, E. Schuuring, R. E. Kibbelaar

^*^Pathologie Friesland, Dept. of Pathology, Noordeloos, The Netherlands

**Objective:** To validate the detection of human epidermal growth factor receptor-2 (HER2) status using the Leica HER2 fluorescent in situ hybridization (FISH) system and subsequent semi-quantitative analysis with the Menarini Benelux D-Sight digital imaging platform, in order to predict HER2-directed therapy in patients with invasive breast cancer.

**Method:** HER2 assessment was performed on 328 formalin-fixed/paraffine-embedded invasive breast cancer tumours on tissue microarrays (TMA) and 100 (50 selected IHC++ and 50 random IHC scores) full-sized slides of breast cancer resections biopsies obtained for diagnostic purposes. For digital analysis slides were pre-screened at 20× and 100× magnification for all fluorescent signals and semi-automated scoring was performed on at least two pictures with the D-Sight HER2 FISH analysis module. Results were compared to data obtained previously with the manual Abbott FISH test.

**Results:** The overall agreement with Abbott FISH data among TMA samples and in 50 selected IHC++ cases was 98.8 % (kappa = 0.94) and 93.8 % (kappa = 0.88), respectively. The results of 50 unselected IHC cases were concordant with previously obtained IHC and/or FISH data.

**Conclusion:** The combination of the Leica FISH system with the Menarini Benelux D-Sight digital imaging platform is feasible for the assessment of HER2 status in routine clinical practice in patients with invasive breast cancer.

**OFP-01-002**

**A new quantitative in-situ immunohistochemistry method**

H. Derand ^*^, J. Lohse, K. Petersen, K. Jensen, R. Jørgensen

^*^Dako A/S, Research and Development, Glostrup, Denmark

**Objective:** There is a general need to make pathologic examinations less subjective and to support the accuracy required for companion diagnostics. The aim of this study was to develop a new IHC method based on bright field technology combining morphological information with quantitative assessment.

**Method:** By chemical manipulation of a visualization system, single antibodies are visualized as dots instead of a conventional stain. The dots can easily be counted by image analysis, and the number of dot reflects protein expression levels. Initial assay performance was evaluated using Her2 as a test system; breast cancer cell lines as well as breast cancer tissue specimen were included.

**Results:** The assay generates highly reproducible results and gives a linear assay with a larger dynamic range than the conventional assays. No overlap was seen between the different breast cancer cell lines. Moreover, evaluation of HER2 status in a number of breast cancers by the use of this new method showed a strong correlation with established methods.

**Conclusion:** The data suggest enumeration of dots can be direct related to protein expression levels. This represents a new and standardized way of objectively determining protein amounts in cells and tissue in situ.

**OFP-01-003**

**Automated image analysis enables accurate enumeration of the Ki-67 labelling index of breast cancer**

A. Laurinavicius ^*^, A. Laurinaviciene, R. Meskauskas, I. Baltrusaityte, J. Besusparis, P. Herlin, B. Plancolaine, N. Elie, P. Belhomme, C. Bor-Angelier

^*^National Center of Pathology, Vilnius, Lithuania

**Objective:** Immunohistochemical Ki67 evaluation reflects proliferative activity and is regarded as important prognostic/predictive marker of breast cancer. However, its potential is hindered by lack of standardized and efficient methodologies to measure the Ki67 expression. Besides many other aspects, key element of the methodology remains accurate enumeration of Ki67-labelling index (LI). We investigated the accuracy aspect of automated image analysis (IA) approach.

**Method:** TMA (1 mm diameter spot per patient, *n* = 140) from invasive ductal breast carcinoma, stained for Ki67 and digitized by Aperio scanner, were used for the study. Reference values (RV) were obtained by counting the LI using stereological frame. IA was performed with Aperio Genie/Nuclear algorithms enabling automated selection of tumour tissue. The images were semi-quantitatively evaluated by 3 pathologists (P1,2,3).

**Results:** RV correlated strongly with IA (*r* = 0.95) and P1,2,3 (*r* = 0.86, *r* = 0.89, *r* = 0.93, respectively), *p* < 0.0001. Analysis of variance revealed no significant pairwise differences of the LI means of RV(40 %) versus IA(36 %), P2(43 %), or P3(44 %); however, the IA versus P2,3 differed, and P1(24 %) was significantly lower (*p* < 0.05). Regression analysis to predict RV revealed best performance for the IA results.

**Conclusion:** IA provided most accurate enumeration of the Ki67 LI. Validation against proper RV is a crucial step in setting up an IA tool.

**OFP-01-004**

**Prognostic impact of Ki-67 in breast cancer assessed on tissue microarrays, core needle biopsies and surgical specimens**

G. Knutsvik ^*^, I. M. Stefansson, K. Collett, L. A. Akslen

^*^University of Bergen, Dept. of Clinical Medicine, Norway

**Objective:** Tumor cell proliferation in breast carcinomas is strongly prognostic and may also predict response to chemotherapy. However, no consensus exists concerning counting areas or cut-off values. Here, our aim was to assess the prognostic value of Ki67 counts from surgical excision specimens as compared with core needle biopsies (CNB) and tissue microarrays (TMA).

**Method:** We examined a retrospective, population-based series of breast cancer (*n* = 547). The percentage of Ki67 positive nuclei was evaluated by manual counting on TMAs, CNBs, and whole sections (WSs).

**Results:** The three evaluation methods were significantly correlated. The median percentage of Ki67 expression was 18.2 % (WS, hot-spot areas), 16.6 % (estimated average across WS), 12.8 % (CNB) and 7.0 % (TMA). Increased Ki67 expression by all evaluation methods was associated with aggressive tumor features (large tumor diameter, high histologic grade, ER negativity) and reduced survival.

**Conclusion:** Ki67 is prognostic over a wide range of cut-off points. However, absolute Ki67 values are influenced by choice of specimen, and the cut-off levels derived from studies of TMA sections are different from those obtained using specimens from a clinical setting. Our findings indicate that specimen specific cut-off values should be established for practical use.

**OFP-01-006**

**Phenotypic and genotypic analysis of triple negative breast cancer with an emphasis on the identification of stem cells**

M. Comanescu ^*^, R. Ivan, G. Bussolati

^*^INCD Victor Babes, Pathology, Bucuresti, Romania

**Objective:** While targeted therapies have been developed for other types of breast cancers, triple negative breast cancers (TNBC) still represent a challenge for oncologists.

**Method:** We evaluated tumor specimens together with peritumoral tissue of 30 breast cancer patients. Besides the routine immunohistochemical analysis, other markers targeting stem cells (CSC) were used. In a first phase a gene array analysis was performes using Agilent Whole Human Genome 4 × 44 K Microarrays, which were further were scanned and signals were extracted using Feature Extraction 10.5.1. The data were analysed using the software GeneSpring GX. In a second phase, we investigated gene expression by real time PCR, using 3 types of 96-well plates: Breast Cancer Array, Human Stem Cell PCR Array and Human Stem Cell Signaling PCR Array from Qiagen. Interpretation of data was performed using Qiagen RT2 Profiler PCR Array data Analysis version 3.5.

**Results:** We identified significant differences in the immunophenotype of CSC between cases and together with the initial microarray based assay directed the study towards the investigation of stem cells.

**Conclusion:** Keeping in mind the need for improvement in the therapy of TNBC, we believe that the identification of genes involved in survival and self-renewal of cancer stem cells represents an area of interesrt Acknowledgments Project PN-II-RU-PD-2011-3-0248

**OFP-01-007**

**Sentinel lymph node macrometastasis in breast cancer- is axillary lymph node dissection mandatory?**

A. Figueiredo ^*^, A. Ribeiro, M. Martins

^*^Dept. of Pathology, Hospital Curry Cabral, Lisboa, Portugal

**Objective:** Axillary lymph node dissection (ALND) is the current procedure in breast cancer patients with sentinel lymph node (SN) macrometastasis. The aim of this study is to determine which factors could be associated with axillary non-SN metastasis.

**Method:** Between 2011 and 2012, 134 patients underwent SN analysis with One Step Nucleic Acid Amplification (OSNA), of which 28 (20 %) had macrometastasis. We assessed tumors in 27 of these 28 patients (10 with non-SN involvement and 17 without). For the statistical analyses the Mann–Whitney test and logistic regression models were performed. A significance level of 0,05 was considered.

**Results:** We verified a significant crude association between non-SN involvement and primary tumor size (OR = 5,6; CI95 %: 1,1;30,9), tumor multifocality (OR = 7,5; CI95 %: 1,1;51,5) and maximum number of CK19 copies (MNCK19) (OR = 7,3; CI95 %: 1,2;46,2). This group had also higher median values of the ratio between MNCK19 and the SN weight (*p* = 0,001) and the number of positive SN (*p* < 0,001). We found no statistically significant association with age, the histological grade and subtypes and lymphovascular invasion.

**Conclusion:** The results suggest that new criteria to perform ALND of breast cancer patients with SN macrometastasis can be developed.

**OFP-01-008**

**Immunohistochemical characterization of HER-2 tumors**

A. Cuesta ^*^, E. Honrado Franco, M. Baltasar Moreira, P. Castro Val, F. Izquierdo Garcia

^*^Hospital de Leon, Dept. de Anatomia Patologica, Spain

**Objective:** HER-2 positivity, defines a clinically challenging subgroup of patients with breast cancer with variable prognosis and response to therapy. The main aim of this study is to identify different immunohistochemical groups in HER-2 tumors and compare HER-2 amplified tumors with HER-2 polysomic or no amplified tumors.

**Method:** Tumors from 54 patients with IDC, 42 HER-2 amplified, 7 non-amplified and 5 polysomic were included in a tissue microarrays and stained for ER, PR, Ki67, p53, p63, ck5/6, Racemase, cyclin D1, p16 and for HER2 (immunohistochemistry and SISH silver in situ hybridization).

**Results:** HER-2 amplified tumors, are more frequently ER, PR, BCL2, P16 negative and do not express basal markers (ck5/6 and p63. P53, Ki67 and Racemase are positive. HER-2 polysomic tumors show overexpression of P53 and Racemase and PR negativity. On the other hand, HER-2 polisomic tumors, are similar to non-amplified tumors in the overexpression of basal markers (20 % versus 9 % in amplified tumors) and ER and the underexpression of Ki 67. We observed that all cases with polysomy of chromosome17 had an IHC score of 3+ except one 2+.

**Conclusion:** HER-2 amplified tumors, are a very homogeneous group with low expression of hormone receptors, and basal markers, and high expression of proliferation markers and Racemase. HER-2 polysomic tumors, might be a subgroup that share immunohistochemical characteristis of HER-2 amplified and non-amplified tumors, show HER-2 protein overexpression which probably has clinical differences.

**OFP-01-009**

**Altered expression of cell cycle regulatory proteins in different types of phyllodes tumors of the breast**

S. Bulimbasic ^*^

^*^Dubrava University Hospital, Dept. of Pathology, Zagreb, Croatia

**Objective:** Phyllodes tumors (PTs) are rare fibroepithelial tumors of the breast with wide biological potential, classified as benign, borderline or malignant.

**Method:** In this study we assessed the prognostic value of histopathologic characteristics and immunohistochemical features across a panel of antibodies against cell cycle regulatory proteins (cyclin D1, p21, p27 and p53) in a study set of 90 PTs. Additionally, immunohistologic features of benign, borderline, and malignant PTs are compared in order to ascertain whether malignant progression is associated with differential marker expression and whether such differences may be diagnostically useful.

**Results:** Stromal overexpression of Cyclin D1, p27, p53 correlated significantly with tumor grade. No immunostaining differences were detected for p21. The number of altered proteins in stroma increased with higher grade and was accompanied by increased proliferation and increased mean vascular density (MVD). Correlations existed between stromal overexpression of Cyclin D1, proliferation index and MVD as well as p53, proliferation index and MVD. Inverse correlation was noted between stromal expression of p27, proliferation index and MVD.

**Conclusion:** According to logistic regression model, p53, proliferation index and MVD were the best predictors of malignant phenotype and can be used as additional methods for grading.

**OFP-01-010**

**Breast tumours resembling Papillary Thyroid Carcinomas (PTC): Report of 10 cases with long term follow up**

S. Foreid ^*^, S. Popovska, V. Eusebi

^*^Hospital de Sao Jose, Dept. de Anatomia Patologica, Lisbon, Portugal

**Objective:** We report 10 cases of papillary carcinomas of the breast that histologically resemble PTC.

**Method:** These include 2 cases previously reported (Am. J. Surg. Pathol. 27, 1114–18, 2003) and 8 new cases.

**Results:** Patients were female of 54.7 years (mean age). All cases displayed neoplastic cells arranged in solid to papillary architectures and follicular structures resembling thyroid tissue. Cells evidenced eosinophilic granular cytoplasm and nuclei with clear chromatin with numerous grooves and occasional eosinophilic pseudoinclusions. All cases stained for mitochondria in >50 % of neoplastic elements. ER, PR, AR, Her-2, TTF1, Thyroglobulin were negative. Myoepithelial cells were absent in 8 cases. Cases were discovered mammographically, except for case 3 that had a lump present for 10 years. Sentinel nodes in 6 cases were negative. Case 3 had an intramammary metastatic lymph node. All patients are alive and well. No fewer than 3 had 10 year-FU.

**Conclusion:** Breast tumours resembling PTC have to be recognized to avoid misdiagnosis as metastatic PTC; they are triple negative tumours with indolent behaviour; the term tumour for these neoplasms seems appropriate as it is difficult, especially without immunohistochemistry, to establish their invasion.

**OFP-01-011**

**Improved malignancy prediction by B3 breast lesions subclassification**

F. de Beca ^*^, C. Rasteiro, A. Correia, I. Amendoeira

^*^Porto, Portugal

**Objective:** Core-needle biopsy (CNB) of breast lesions can be classified in 5 categories according to lesion type and risk of malignancy. B3 category constitutes a challenging problem in clinical decision with most ending in excisional biopsy. This study aims at assess if subclassification (in B3a and B3b categories) according to the presence of atypia in otherwise B3 lesions better predicts malignancy on excision.

**Method:** 48 cases with diagnosis of B3 lesion on CNB and matched surgical excision specimen were included and subclassified in B3a and B3b categories to evaluate positive predictive value (PPV) and odds of malignancy in CNB.

**Results:** B3 category overall PPV for malignancy was 12.5 % with a significant low odds of malignancy of 0.14 (95 % CI: 0.06–0.34). When subclassified, B3b (lesions with atypia) demonstrated a higher PPV for malignancy (36.36 %) with a non-significant odds. Inversely, B3a (lesions without atypia) demonstrated a PPV for malignancy of only 5.41 % and a significant low odds of malignancy of only 0.06 (95 % CI: 0.01–0.24).

**Conclusion:** The low rate of malignancy in some of B3 lesions additionally reinforces the practice of avoiding surgical excision in selected patients and provides data that further supports B3 lesions subclassification according to the presence of atypia.

**OFP-01-012**

**Adenoid cystic carcinoma of salivary glands and the breast: May their different clinical course be attributed to posttranscriptional divergencies?**

O. Kiss ^*^, J. Kulka, A.-M. Tokes, A. M. Szasz, S. Spisak

^*^Semmelweis University, 2nd Dept. of Pathology, Budapest, Hungary

**Objective:** Adenoid cystic carcinoma of the salivary glands and the breast (sACC and bACC) are histologically identical malignancies but their clinical course differ: sACC has a poor prognosis but bACC's clinical outcome is favourable. Our aim was to investigate sACCs and bACCs characteristics at the posttrascriptional level.

**Method:** Thirteen cases of sACCs and 10 cases of bACCs were investigated. Normal salivary gland (NS = 11) and normal breast tissues (NB = 9) were used as controls. Total RNA was isolated from FFPE tissues. Reverse transcription was carried out on each sample for 18 miRNAs. We performed quantitative PCR on 96-well plates.

**Results:** Relative expression of let-7b decreased in sACCs compared to NSs. In bACCs miR-20a decreased compared to NBs, while the expression of miR-17* and miR-27b was higher in bACCs than in NBs. The expression of miR-193b was higher in sACCs than in NSs but in bACC its expression was lower in bACCs than in NBs. The genes potentially regulated by the above mentioned miRNAs include CCND1, BCL2, VEGFA, and NOTCH1 which have central roles in the development, expansion and spreading of human malignant tumors.

**Conclusion:** The detected posttranscriptional differences might perhaps partially explain the diverse clinical course of bACC and sACC cases.

Sunday, 1 September 2013, 14.15–16.15, Room 5C

**OFP-02 Oral Free Paper Session Surgical Pathology and Other Topics**

**OFP-02-001**

**Effect of unbalanced immune reactions on myocardial remodeling of dilatative cardiomyopathy patients with left ventricular assist device implantation**

K. Wassilew ^*^, E. Potapov, T. Krabatsch, M. Dandel, B. Song, A. Mickley, C. Schmuttermaier, A. Gratchev, R. Hetzer, J. Kzhyshkowska

^*^Deutsches Herzzentrum Berlin, Inst. für Herzpathologie, Germany

**Objective:** We investigated effects of chronic inflammatory disorders (CID) such as asthma and autoimmune reactions on immunopathological markers supporting alternatively activated macrophages in dilatative cardiomyopathy (DCM) patients with LVAD implantation and compared levels of interstitial fibrosis (IF) in LV apex from the site of LVAD implantation with anterior LV wall of the explanted heart.

**Method:** Paraffin sections of LV myocardium from 13 patients (8–62 years, 2 female, 9 with CID) were stained for a macrophage marker (CD68) and a M2 marker (stabilin-1). Subpopulations of macrophages were examined using double immunofluorescence/confocal microscopy. Findings were correlated with IF on Sirius-red-stained slides.

**Results:** Whereas circulating macrophages were solely CD68+, tissue macrophages showed combined CD68+/stabilin-1+ and myofibroblasts were actin+/stabilin-1+. A higher CD68+/stabilin-1+ cell count in the LV apex was associated with elevated IF in all but 3 patients. In patients without CID, mean IF (8.5 %) was within normal limits (<9 %); in CID patients, total IF was elevated (mean 14.6 %). Generally, there was less fibrosis in the anterior LV wall than in apical myocardium.

**Conclusion:** CID appear to initiate inflammatory responses and influence the repolarization of resting resident macrophages into M2, which are associated with larger areas of IF and therefore participate in myocardial remodeling.

**OFP-02-002**

**Angiogenesis in symptomatic vascular malformations of skin and soft tissue relates to hemodynamic high flow features of the lesion**

L. Meijer-Jorna ^*^, C. M. van der Loos, O. J. de Boer, A. J. Horrevoets, J. R. Mekkes, C. M. van der Horst, A. C. van der Wal

^*^Symbiant/MCA, Dept. of Pathology, Alkmaar, The Netherlands

**Objective:** Vascular malformations are clinically categorized in high flow (HF) or low flow (LF) lesions. Episodic angiogenesis has been observed in arteriovenous malformations (AVMs) of skin and soft tissue. We investigated whether angiogenesis relates to different flow features.

**Method:** Resection specimens of 80 AVMs, of which 37 HF and 43 LF lesions were histopathologically screened for presence of angiogenesis, inflammation and/or thrombosis. Additional immunostaining was performed with anti-endoglin (CD105), a marker for endothelial activation, and anti-von Willebrand factor (vWF) for vascular leakage.

**Results:** 81 % of HF lesions versus 14 % of LF lesions showed angiogenesis (*p* < 0.005). In HF lesions, that were embolized prior to surgery (30 % of all), 88 % showed angiogenesis, 88 % inflammation and 33 % thrombosis. However, similar angiogenesis was also observed in AVMs without prior embolization (69 % HF versus 14 % LF, *p* < 0.005). Endoglin was more frequently expressed in HF lesions and extracellular vWF staining was found in nearly all HF and LF lesions.

**Conclusion:** Angiogenesis in AVMs appears to be strongly associated with HF features. This can be explained only to some extent by tissue damage initiated by previous embolization and/or (related) inflammation. Occurrence of angiogenesis in AVMs without prior embolization suggests that the high flow itself has angiogenic properties in AVMs.

**OFP-02-003**

**A new ligand for the endothelial receptor, TIE-2, that enhances barrier integrity and prevents vascular leakage**

C. Jackson ^*^, N. Minhas, P. Naik, M. Xue

^*^Kolling Institute, Sutton Laboratory, Sydney, Australia

**Objective:** Activated protein C (APC), a natural anticoagulant with strong cyto-protective properties, is FDA-approved for treatment of severe sepsis and acts by promoting endothelial barrier protection. The aim of this study was to investigate the immediate effects of APC through the Tie2 receptor on endothelial barrier integrity.

**Method:** Methods include biacore, bioinformatics, (competitive) binding assays, and a mouse model of sepsis

**Results:** APC rapidly activated Tie2 with the ratio of phosphorylated(P)-Tie2:total(T)-Tie2 increasing by ~4.5-fold after 60 min of APC treatment. Docking studies revealed intermolecular H-bonds between APC and Tie2 indicating binding between the two proteins. Protein binding assay using Biacore confirmed strong binding affinity (Kd = 0.003 nM) between APC and Tie2, which was only slightly less than Angiopoietin (Ang)1-Tie2 (Kd = 0.002) and surprisingly, ~11 times higher than Ang2-Tie2 (Kd = 0.032 nM). In a mouse model of sepsis, APC significantly reduced lipopolysaccharide-induced vascular permeability in lungs and kidneys and this was reversed by inhibiting the Tie2 receptor.

**Conclusion:** In summary, APC is a new ligand for Tie2. By binding to and activating Tie2, APC rapidly enhances endothelial barrier function and prevents vascular leakage, which could explain its therapeutic action in sepsis.

**OFP-02-004**

**Tumors and tumour-like lesions of the calcaneum: A study of 7 cases**

F. Khanchel-Lakhoua ^*^, K. Mrad, R. Dhouib, R. Doghri, M. Driss, L. Charfi, S. Sassi, I. Abbes, N. Ben Hamida, K. Ben Romdhane

^*^Salah Azaiez Institute, Pathology, Tunis, Tunisia

**Objective:** The calcaneum is an uncommon site for most bone tumors. The knowledge of the distribution of tumors in the calcaneus is important for an exact differential diagnosis and thereby for biopsy planning, diagnosis and therapy. The aims of our study is to evaluate tumors and tumor-like lesions of the calcaneum.

**Method:** We analyzed tumoral and tumour-like lesions of the calcaneum registered in the Department of Pathology Salah Azaiez Institut from 1992 to 2012.

**Results:** 7 cases were registered. There were 5 males and 2 females and their ages varied from 7 to 70 years with a median of 16. We registred 4 benign lesions, with one chondromyxoid fibroma, one angioma, one osteochondroma, one chondroblastomas. There were 3 malignant tumours (1 osteosarcoma, 1 Ewing sarcoma, 1 chondrosarcoma).

**Conclusion:** In our series, cartilaginous lesions were the main lesion of the calcaneum. In literature, cystic lesions such as simple bone cysts and aneurysmal bone cysts are the most common lesion. Other tumors in the calcaneus are mainly cartilaginous.

**OFP-02-005**

**Histone deacetylase inhibitors as potential therapeutic approaches for chordoma: An immunohistochemical and functional analysis in MUG-chor1**

S. Scheipl ^*^, B. Lohberger, E. V. Froehlich, B. Rinner, A. Beham, F. Quehenberger, P. P. Varga, A. Lazary, J. Haybaeck, A. Leithner, B. Liegl

^*^Medical University of Graz, Orthopaedic Surgery, Austria

**Objective:** Chordomas are rare malignancies of the axial skeleton. Therapy is mainly restricted to surgery. This study investigates histone deacetylase (HDAC) inhibitors as potential therapeutics for chordomas.

**Method:** Immunohistochemistry (IHC) was performed using the HDAC 1–6 antibodies on 50 chordoma samples (34 primary tumours, 16 recurrences) from 44 patients (27 male, 17 female). Pan-HDAC inhibitors Vorinostat (SAHA), Panobinostat (LBH-589), and Belinostat (PXD101) were tested for their efficacy in the chordoma cell line MUG-Chor1 via Western Blot, cell cycle analysis, caspase 3/7 activity, caspase-3, and PARP cleavage. *P*-values below 0.05 were considered significant.

**Results:** IHC was negative for HDAC1, positive for HDAC2 in most (*n* = 36; 72 %), and for HDACs 3 to 6 in all specimens available (*n* = 43; 86 %). HDAC 6 expression was strongest. SAHA and LBH-589, but not PXD101 caused a significant increase of G2/M phase cells and of cleaved caspase-3 (*p* = 0.0003, and *p* = 0.0014 after 72 h, respectively), and a peak of caspase 3/7 activity. PARP cleavage confirmed apoptosis.

**Conclusion:** The presented chordoma series expressed HDACs 2–6 with strongest expression of HDAC6. SAHA and LBH-589 significantly increased apoptosis and changed cell cycle distribution in vitro. HDAC-inhibitors should be further evaluated as therapeutic options for chordoma.

**OFP-02-006**

**Primitive mucosal melanoma: A review of 11 cases**

M. Njima^*^, S. Ben Abdelkrim, N. laabaied, T. Tlili, S. Korbi, S. Hmissa

^*^Farhat Hached Hospital, Dept. of Pathology, Sousse, Tunisia

**Objective:** Melanomas are essentially cutaneous tumors; their mucosal location is rare accounting for 1.3 % of melanomas. The pathologist is often confronted with two problems, positive diagnosis and confirmation of the primitive character of the tumor.

**Method:** This is a retrospective study of PMM seen over a period of 15 years (1993–2007). Epidemiological and clinicopathological features were recorded.

**Results:** 11 cases were diagnosed during this period. There were 5 males and 6 females patients. The mean age was 58 years (range 22–75). The most common location was the eye in 4 cases (36 %), then genital sites (vagina, clitoris) in 2 cases and digestive sites (rectum, anus) were involved in two other cases. The tumor was stage II or IV in 50 % of cases it was T3 or T4 in 55 % of cases. Lymph node metastasis and distant metastases were observed in 27 % of cases each. Treatment was surgical in 7 cases, radiotherapy or chemotherapy in 1 case.

**Conclusion:** PMM is a rare and aggressive tumor. Its symptomatology is non specific. Definitive diagnosis is achieved after pathological analysis. Diagnosis is often delayed.

**OFP-02-007**

**Quality control of deaths by pathologists: A systematic approach for improving death certificate completions, notification procedures and death statistics**

G. C. Alfsen ^*^, L. G. Lyckander, H. M. Eng, A. W. Lindboe

^*^Akershus University Hospital, Dept. of Pathology, Lørenskog, Norway

**Objective:** In order to improve the quality of death certificates and the notification procedures in deaths of presumed unnatural causes, all deaths at our institution have since 2008 been reviewed by two pathologists.

**Method:** Death certificates and medical records were examined continuously. Clinicians were contacted immediately if death certificates had illogical sequences of underlying death, or if cause of death was not in accordance with the clinical history. Changes were suggested and assistance in resubmittance offered. The Quality departement was notified if deaths by unnatural causes (of any kind) were suspected, and the clinicians contacted if notification procedures had not been followed.

**Results:** From 2008 to 2012, illogical death certificates decreased from 36 % to 20 %, certificates not in accordance with medical records from 24 % to 14 %. Unspecified “garbage” diagnoses were reduced by 79 %. Notification procedures in presumed unnatural deaths improved from 51 % to 86 %.

**Conclusion:** A centralized and systematic control of deaths improved the quality of death certificates and the handling of deaths by presumed unnatural causes. Being experienced in evaluating causes of death, having regular working hours and proximity to the morgue, the pathologists are best suited to perform this kind of quality control in hospitals.

**OFP-02-008**

**Choice of method for manual Ki-67 determination**

R. Røge ^*^, S. Nielsen, M. Vyberg

^*^Aalborg University Hospital, Institute of Pathology, Denmark

**Objective:** Ki67 is a proliferation marker important for classification of malignant tumours such as breast carcinomas. However, guidelines for determination of Ki67 proliferation indices are heterogeneous. The aim of this study was to examine the current practices and interlaboratory variability of Ki67 scoring.

**Method:** As part of the NordiQC international immunohistochemical external quality assessment scheme, 370 laboratories were asked to score proliferation indices on 20 Ki67 stained breast carcinomas on a virtual slide. Additionally, participants were asked to elaborate on the method used for obtaining the indices: Method (‘Eyeballing’ or ‘manual counting of X cells’), Area examined (‘Hot spot’ or ‘Overall average’) and whether moderate and weakly stained tumour cells were counted as positive.

**Results:** 139 laboratories participated. Estimated proliferation indices varied greatly. For tumours with low Ki67 proliferation index, estimated indices varied between 0 and 10 %, while results for high expressing tumours varied between 40 and 100 %. Eyeballing was the preferred method (65 %). Less than 40 % examined ‘hot spots’ as opposed to overall average.

**Conclusion:** The need for standardization of both staining and interpretation of Ki67 immunohistochemical stains is underlined. Digital image analysis may be an important tool to accomplish this.

**OFP-02-009**

**Initiation of a tumor tissue-bank in Malawi**

S. Berezowska ^*^, T. Tomoka, E. Borgstein, D. A. Milner, S. Kamiza, R. Langer

^*^Universität Bern, Inst. für Pathologie, Switzerland

**Objective:** Cancer in Africa has become a serious health problem with high mortality rates. Knowledge about epidemiology, pathogenesis and genetics is scarce. Pathology plays an important role in diagnostics and in cancer research, but the service is barely available. We initiated the project of building a tumor-biobank in Blantyre/Malawi, as a basis of structured, significant tissue-based research targeting cancer in sub-Saharan Africa.

**Method:** Routine diagnostics in Blantyre is based on formalin fixed paraffin embedded (FFPE) probes and allows diagnostic standard staining (HE, PAS, ZN). Application of further molecular analysis for routine or research is hampered by the lack of standardization of pre-analytic tissue handling (e.g. variable fixation times, unclear fixation agents).

**Results:** A basic approach for a biobank in the low resource setting encompasses: A) Structuring and standardization of tissue processing, allowing FFPE tissue to serve as a firm base for scientific projects. B) Evaluation of alternative fixation agents, which may offer alternatives for robust tissue preservation. C) Establishing a clinical data bank. D) Ethical issues (e.g. informed consent, cultural aspects).

**Conclusion:** Establishing a tissue-biobank in sub-saharan Africa is a valuable step towards tissue-based research for gaining insight into cancer in the African population, and a prerequisite for subsequent focused analyses (e.g. HPV-analyses).

**OFP-02-010**

**The preliminary results of a residency satisfaction survey: “To love and not to love Pathology”**

B. Pehlivanoglu ^*^, H. Hassoy, I. Nalbantoglu, C. Calle, A. Dendooven, A. F. Okuducu, B. Doganavsargil

^*^Ege University, Dept. of Pathology, Izmir, Turkey

**Objective:** Pathology tends to be a less known discipline among medical school graduates. Multiple factors contribute to career choices such as individual’s personality, skills, academic and socioeconomic expectations. Unfortunately, the number of the residents who are not satisfied with their job is considerable. We conducted a survey to evaluate pathology residents’ perspectives on Pathology.

**Method:** The 42 item-survey was delivered via a web-based link and questioned participants’ personal and institutional background, workplace, training conditions and job satisfaction.

**Results:** Survey was answered by 101 participants from Turkey (*n* = 76), Europe (*n* = 16) and continental America (*n* = 9), 78,2 % of whom were female. Mean age was 29,7 years-old (range 25–40). Mean daily work and weekly grossing-hours of the participants were 10.1 ± 1.7 and 12.9 ± 5.1 h, respectively. Eighty-two percent of the participants were happy that they chose Pathology and 72,2 % foresaw a bright future ahead. Overall working conditions of their department were dissatisfying for 8.9 % of the participants. Most of the participants wanted to have better structured residency training programs and to interact more with experienced pathologists.

**Conclusion:** The study aims to provide an insight to residency period and is presented to increase the philosophical interest on the subject as well as announcing for more participants.

Sunday, 1 September 2013, 17.00–19.00, Auditorium II

**OFP-03 Oral Free Paper Session Digestive Diseases Pathology I**

**OFP-03-001**

**Mutational analysis by next generation sequencing of preneoplastic intestinal metaplasia in patients with Barrett esophagus from endoscopic samples**

S. Lagana ^*^, Y. Yuan, T. Uehara, N. Jhala, T. Ganguly, Y. Liu, R. Brand, J. Sepulveda, G. Falk, A. Sepulveda

^*^New York Presbyterian-Columbia, Dept. of Pathology and Cell Biology, New York, NY, USA

**Objective:** To identify mutational biomarkers using next-generation sequencing (NGS) and endoscopic samples for stratification of Barrett Esophagus (BE) patients at increased risk of dysplasia.

**Method:** Ion Torrent AmpliSeq Cancer Panel was used to screen for mutations in 46 cancer genes. Sixteen samples were tested: Intestinal metaplasia from 6 patients (IM-P) with concomitant high-grade dysplasia (HGD)/adenocarcinoma (EAC) and HGD/EAC in 3 patients; IM of 7 patients followed for at least 2 years without any dysplasia (IM-N). Ion torrent suite software and ANNOVAR were used for analysis.

**Results:** The most frequent mutations in IM and HGD/EAC were detected in TP53 spanning codons 150 to 280. Four of 6 IM-P patients had TP53 mutations in IM samples and in HGD/EAC tested. None of the 7 IM-N patients had TP53 mutations. Sensitivity of TP53 mutation for presence of concomitant dysplasia was 67 % and specificity was 100 %.

**Conclusion:** DNA from routine endoscopic samples can be efficiently used to simultaneously detect multiple mutations by NGS. TP53 mutations were frequently detected in IM of patients with HGD/EAC but not in patients who did not progress to HGD/EAC, suggesting that TP53 mutational testing may be useful to identify IM-P patients who may benefit from closer surveillance.

**OFP-03-002**

**Amplification but not translocation of ALK is a frequent event in esophageal cancer**

P. Birner ^*^

^*^Medizin. Universität Wien, Inst. für Pathologie, Austria

**Objective:** Translocations of ALK have been demonstrated in a variety of human malignancies, and the corresponding fusion-proteins are potential therapeutical targets. Aim of this study was to investigate ALK gene status in a large cohort of esophageal squamous cell carcinoma (SCC) and adenocarcinoma (AC).

**Method:** ALK status was investigated in 117 SCCs and 136 ACs by fluorescence in situ hybridization (FISH), and ALK protein expression by immunohistochemistry. Data on expression of ALK downstream effector tyrosine −705 phosphorylated STAT3 (pSTAT3) was available from a previous study.

**Results:** FISH was successfull in 251 cases. No ALK translocations were found, while 14/135 (12.1 %) of SCCs and 14/116 (10.4 %) of ACs showed ALK amplifications. Concomitant EML4 amplifications were present in 27/28 cases with ALK amplifications. Three cases showed EML4 translocations not involving ALK. None of the tumors with ALK amplification showed ALK protein expression, and no correlation with clinical parameters, survival or pSTAT3 expression was observed.

**Conclusion:** While ALK translocations are not present in esophageal cancer, ALK amplifications are common events. Since ALK amplified breast cancer cells were shown to respond to ALK inhibitors, ALK amplified esophageal cancers might be considered as possible candidates for therapies targeting ALK.

**OFP-03-003**

**MALDI imaging mass spectrometry for proteomic segmentation of tumor heterogeneity in gastric cancer tissues**

B. Balluff ^*^

^*^Leiden University Medical Cent, The Netherlands

**Objective:** A high clonal diversity within a patient’s tumor is an important factor for the evolution of the disease and the clinical management of the patient with regard to cancer relapse and response to therapy (Marusyk, Nat Rev Cancer. 2012 Apr 19;12(5):323–34). The identification and molecular characterization of the tumor’s clonal diversity is therefore of high clinical relevance.

**Method:** In this study, matrix-assisted laser desorption/ionization (MALDI) imaging mass spectrometry was used to identify clinically relevant tumor subpopulations in gastric cancer. MALDI imaging allows the unlabeled in situ measurement of hundreds of molecules (like proteins) within their histomorphological context of tissue sections (Balluff, Gastroenterology. 2012 Sep;143(3):544–9.e1-).

**Results:** Spatially-resolved, tumor-specific proteomic data was acquired from 63 intestinal-type gastric cancer patients by MALDI imaging. The resulting data underwent a novel statistical procedure (Jones, PLoS One. 2011;6(9):e24913) which results in a spatial segmentation of areas within a tumor section based on their molecular similarity. Correlation of the molecular signatures of the segmented tumor areas with clinical data resulted in the identification of clinically relevant tumor subpopulation in terms of prognosis and metastasis.

**Conclusion:** Our results highlight the usefulness of MALDI imaging in combination with advanced statistical approaches for detecting novel and clinical relevant information from tumor tissues.

**OFP-03-005**

**Increased tumor-budding/CD8+ lymphocytes ratio is associated with metastasis and venous invasion in Pancreatic Ductal Adenocarcinoma (PDAC)**

E. Karamitopoulou ^*^, I. Zlobec, A. Perren, B. Gloor, A. Lugli

^*^University of Bern, Institute of Pathology, Switzerland

**Objective:** T-Lymphocytes can be a major part of tumor microenvironment, especially at the area of tumor-host interaction where tumor progression, reflected by epithelial-mesenchymal transition (EMT) and its hallmark tumor-budding is taking place. Our aim was to determine the role of CD8+lymphocytes in correlation with tumor-budding in PDAC.

**Method:** Double immunostaining for AE1-AE3/CD8 was performed on a multipunch tissue microarray of 120 well-characterized PDACs. Tumor-buds, CD8+ and tumor-budding/CD8+ lymphocytes indices were evaluated and associated with clinico-pathological features, follow-up and adjuvant therapy information.

**Results:** There was a strong negative correlation between the number of buds and CD8+ counts (*p* = 0.01). Increased numbers of tumor-buds were associated with venous invasion (*p* = 0.0272) and reduced overall survival (*p* = 0.0147). Low CD8+ peritumoral lymphocytes showed only a marginal association with metastasis (*p* = 0.0683). Tumor-budding/CD8+lymphocytes ratio was strongly associated with venous invasion (*p* = 0.0078) and metastasis (*p* = 0.0452). No intratumoral CD8+lymphocytes were detected.

**Conclusion:** Low counts of CD8+ peritumoral lymphocytes in the micro-environment of PDAC promote EMT as reflected by tumor-budding and facilitate tumor progression, since tumor-buds seem to display metastatic potential only when coupled by decreased counts of CD8+lymphocytes. However, unlike to other tumor types, no prognostic effect was found, probably reflecting the fact of the absence of intratumoral CD8+lymphocytes.

**OFP-03-006**

**Various carcinogenetic pathways may be involved in colorectal serrated polyposis**

E. Courcet^*^, A. Aline-Fardin, P. Cervera, J.-F. Fléjou

^*^Hôpital Saint-Antoine, Service d’Anatomie Pathologiqu, Paris, France

**Objective:** Serrated polyposis (SP) is considered as a model leading to colorectal cancer (CRC) through the serrated pathway. However, the precise morphological and molecular steps are still incompletely understood. We aimed to characterize the morphological and molecular abnormalities in a series of SP, in CRC and in precancerous lesions.

**Method:** Fifteen consecutive patients with SP were selected in our files. Polyps (*n* = 258) were classified according to the World Health Organization classification (conventional adenomas (CA), sessile serrated adenomas/polyp (SSA/P), traditional serrated adenomas (TSA), hyperplastic polyps (HP)). CRCs (*n* = 13) and polyps (*n* = 31) were evaluated for mismatch repair proteins (MMR), BRAF mutated V600E protein, p53 and beta-catenin expression using immunohistochemistry.

**Results:** Residual adenomatous lesions (4 tubulo-villous CAs, 4 SSA/Ps) were identified beside CRCs. Polyp number per patient ranged from 6 to 51, with 89 % of serrated polyps (44 HPs, 130 SSA/Ps, 47 dysplastic SSA/Ps). Eight CRCs and 1 SSA/P with high and low-grade dysplasia were MLH1-/PMS2-. One CRC was MSH2-/MSH6-. Five of 24 SSA/Ps were BRAF V600E+.

**Conclusion:** SP seems to be a possible although rare presentation of Lynch syndrome with MSH2 mutation. Serrated adenomas and conventional adenomas seem to be both involved in carcinogenesis in SP.

**OFP-03-007**

**Expression pattern of TrkB in colorectal cancer supports anoikis-resistance as a survival mechanism for tumor budding cells**

H. Dawson^*^, I. Zlobec, V. H. Koelzer, E. Karamitopoulou, A. Lugli

^*^Universität Bern, Inst. für Pathologie, Switzerland

**Objective:** Tumor buds in colorectal cancer represent an aggressive subgroup of non-proliferating, non-apoptotic cells. We hypothesize that survival of tumor buds is dependent on resistance to anoikis. Here we investigate the role of TrkB, a promoter of epithelial-mesenchymal transition (EMT) and anoikis-resistance in facilitating a pro-tumor budding phenotype.

**Method:** TrkB immunohistochemistry was performed on a multiple-punch tissue microarray of 211 colorectal cancer patients. Cytoplasmic (cTrkB) and nuclear (nTrkB) staining were evaluated in tumor and buds. KRAS/BRAF mutations were investigated. Correlation with a panel of EMT-related proteins was performed.

**Results:** cTrkB and nTrkB were strongly inversely correlated in tumor (*r* = −0.38; *p* < 0.0001) and tumor buds (*r* = −0.41; *p* < 0.0001). cTrkB was associated with high-grade tumor budding (*p* < 0.0001), KRAS mutation (*p* = 0.0008) and expressed frequently in tumor buds (100/154 cases; *p* < 0.0001). nTrkB was expressed in low-grade budding cases (*p* = 0.0073), BRAF wild-type tumors (*p* = 0.0519) and expressed infrequently in tumor buds (34/154; *p* < 0.0001). cTrkB and nTrkB protein profiles corresponded to pro- and anti-budding phenotypes, respectively.

**Conclusion:** These results underline functional differences of TrkB dependent on cellular localization with cTrkB promoting a pro-budding phenotype. Moreover, our findings support the notion of anoikis-resistance as a survival mechanism for budding cells in colorectal cancer.

**OFP-03-009**

**Primary Biliary Cirrhosis (PBC): A new histological scoring system allows a standardized and reliable evaluation of lesions with known prognostic significance**

D. Wendum ^*^, P.-Y. Boëlle, P. Bedossa, E.-S. Zafrani, F. Charlotte, M.-C. Saint Paul, S. Michalak, O. Chazouillères, C. Corpechot

^*^APHP-Hôpital St. Antoine, Dept. de Anatomie Pathologique, Paris, France

**Objective:** In PBC, an accurate evaluation of liver lesions predictive for progression or survival (fibrosis, lymphocytic interface hepatitis (LIH), ductopenia) is crucial. Presently there is no satisfactory system analyzing them reliably. We elaborated a semiquantitative scoring system and evaluated its intra/interobserver reproducibility.

**Method:** Fibrosis was classified into 4 stages (portal/periportal fibrosis, few septa, numerous septa, cirrhosis) and LIH into 4 grades. The bile duct ratio (BDR = portal tracts with ducts on total number of portal tracts) and Ludwig’s stage were also evaluated. 33 liver biopsies (HE, picrosirius red) were independently analyzed by 5 liver pathologists. Intra and interobserver agreement were assessed (multireader Light’s kappa, Washington intraclass correlation).

**Results:** The biopsies measured 23 mm [12–40 mm]. Five had numerous fibrous septa or cirrhosis and five had severe LIH. The mean BDR was 0.75. Intraobserver reproducibility was substantial for fibrosis (*k* = 0.78), LIH (*k* = 0.69) and BDR (ICC = 0.69). Interobserver reproducibility was moderate for fibrosis (*k* = 0.56), LIH (*k* = 0.59), BDR (ICC = 0.5). Ludwig’s staging had a fair intra and interobserver reproducibility (*k* = 0.26, *k* = 0.32 respectively).

**Conclusion:** This scoring system assesses the prognostic lesions with a substantial intraobserver and a moderate interobserver reproducibility. It is more reliable than Ludwig’s staging. It will likely improve the quality and robustness of the histopathological results in PBC.

**OFP-03-010**

**E2F-1 immunophenotype is an independent marker of poor prognosis in human hepatocellular carcinoma**

M. Palaiologou ^*^, J. Delladetsima, M. Karanikolas, E. Fatourou, D. Karandrea, E. Antoniou, E. Felekouras, J. Koskinas, D. Tiniakos

^*^NKUA Medical School, Lab. of Histology and Embryology, Athens, Greece

**Objective:** E2F-1 transcription factor induces expression of genes controlling G1/S phase transition, DNA synthesis/repair, and apoptosis. E2F-1 activation depends on pRb phosphorylation. We have shown that E2F-1 is overexpressed and pro-apoptotic in hepatocellular carcinoma (HCC), while others have suggested that increased apoptosis in HCC could be indirectly oncogenic*. The prognostic significance of E2F-1 immunoexpression in human HCC has not as yet been clarified.

**Method:** Immunohistochemistry for E2F-1 and phospho(Ser795)pRB was employed on 57 surgically resected HCCs (grade I:8,II:24,III:16,IV:9). Patients were followed for 39.7 ± 27.2 months.

**Results:** Nuclear E2F-1 (nE2F-1) immunoexpression was observed in 32/57(54.3 %) HCCs and correlated with phospho(Ser795)pRB immunoexpression (*p* = 0.045), indirectly suggesting E2F-1 transcriptional activity. E2F-1 cytoplasmic immunoexpression (cE2F-1) was observed in 46/57(80.7 %) HCCs. There was no significant correlation of nE2F-1 or cE2F-1 with clinico-pathological parameters. Portal vein thrombosis and nE2F-1 immunophenotype were correlated with poor overall survival (*p* = 0.05 and *p* = 0.0001, respectively), but not with recurrence-free survival. Stepwise Cox regression analysis highlighted nE2F-1 as an independent marker of poor prognosis in HCC (95 % CI = 2.55–52.43, *p* = 0.002).

**Conclusion:** E2F-1 is expressed and transcriptionally active in the majority of human HCCs. E2F-1 immunophenotype is an independent marker of poor overall patient survival in a cohort of Greek HCC. * Virchows Archiv 2012;460:439–46

Sunday, 1 September 2013, 17.00–19.00, Room 5B

**OFP-04 Oral Free Paper Session IT in Pathology**

**OFP-04-001**

**The Case Database of the European Congresses of Pathology: Current status and planned features**

M. Lundin ^*^, J. Szymas, J. Lundin

^*^Helsinki, Finland

**Objective:** The ECP Case database consists of Case presentations from the ECP/ESP congresses, years 2006–2012. The objective is to continuously expand and improve the contents and functionality of the database, and make it available for educational purposes.

**Method:** Slides of cases presented in the ECP slide seminars have been digitized and published online since 2006. The virtual slides together with case data (background, snapshots, diagnosis, handout) have been collected into a single repository. The database has been made available through a web site (http://www.webmicroscope.net/casedatabase) with interactive functions such as browsing by topic/working group, searching and a quiz/self test mode. The database is designed to be hosted on a network of campus servers

**Results:** After the ECP 2012 there were 634 cases with 3201 images included in the database. These cases have been contributed by 427 authors. The database will be expanded in 2013 with high-quality case presentations from the ECP in Lisbon.

**Conclusion:** The ECP Case Database is a significant educational resource, and continues to grow.

**OFP-04-002**

**The first results of Latvian national tissue biobank and the novel elaborated biobank information database system: Status report after the first 3 years of experience**

S. Isajevs ^*^, I. Liepniece-Karele, A. Kirshners, N. Jeshkevics, A. Ruskule, J. Eglitis, M. Leja

^*^University of Latvia, Dept. of Pathology, Riga, Latvia

**Objective:** The objectives and goals of the first national Latvian biobank project are to develop, build and utilize cutting edge tissue biobank and databasae in Latvia containing samples of common malignant tumours and elaborate a multifunctional easy to use biobank database system.

**Method:** 1,655 patients with oncological and premalignant diseases were enrolled in the study. Biobank information systems run on the operating system MS Windows 7 Professional. Server runs on MS Server 2008 R2 Standard environment, MS Windows SQL Server 2008 R2 Standard served as a database management system.

**Results:** In total 1,655 patients with oncological and premalignant diseases were enrolled in the study during the 2010–2012. Our database comprised a total of 1,655 cases covering all types of frequent human malignancies: breast cancer (30.64 %), gastric (22.78 %), colorectal (15.52 %), prostate (17.38 %) and thyroid (13.78 %). The novel biobank information database was developed which fulfils all major biobanking criteria and extends these. The advantages of novel biobank information system are easy entry, retrieval and storage of information.

**Conclusion:** The first Latvian biobank including malignant disease, premalignant conditions and autoimmune disease was elaborated. The novel easy to use and reproducible biobank information database system was developed and adapted.

**OFP-04-003**

**A Swedish government-funded project for distance diagnostics, consultative expert networks and multi-disciplinary team support**

J. Lindholm ^*^

^*^Djursholm, Sweden

**Objective:** A project aiming to create a system and workflow for distance diagnostics, consultative expert networks and multi-disciplinary team support. Three main scenarios: 1. Department that has long term or short term demand for analysis capacity. 2. Expert node with specific competence, serving a larger region. 3. Collaboration across sub-specialist areas in virtual multi-disciplinary teams to enhance the diagnostic quality and provide more effective treatment. The aim is complex as it transverse the organizational and sub-specialist boundaries that exists today enabling exchange of competence and capacity to provide the patient with the best care possible. The project also manages technic, regulations, reimbursement and patient security.

**Method:** 300 patient cases will be sent for pathology distance reading between two different counties in Sweden. Stockholm County Council—a large urban region and Blekinge County Council—a small region in the countryside. Much effort is being placed in risk-analysis and quality controls.

**Results:** The project is on-going. Distance reading is today being tested with real patient data between the two county councils. Simultaneously a prototype for multi-disciplinary team support is being put together.

**Conclusion:** Conclusions and results so far will be presented at ECP 2013 if abstract being accepted.

**OFP-04-004**

**Next-generation tissue microarray (ngTMA) strengthens pathology biomarker research: An exemplary study of the tumor microenvironment using CD3, CD8, and CD45RO in six different solid tumor types**

I. Zlobec ^*^, V. H. Koelzer, H. Dawson, A. Perren, A. Lugli

^*^Universität Bern, Inst. für Pathologie, Switzerland

**Objective:** We defined next-generation tissue microarrays (ngTMA) as the combination of strategic TMA planning, histological expertise, digital pathology and automated tissue microarraying. The aim is to test ngTMA focusing on the immune-score (CD3, CD8 and CD45RO) within the tumor microenvironment of six tumor types.

**Method:** Ten cases each of malignant melanoma, lung, breast, gastric, prostate and colorectal cancers were reviewed. One representative H&E slide was scanned and uploaded onto a digital slide platform. Using different colors, 1 mm TMA annotations were placed directly onto the digital slide. Selected regions of normal tissue (*n* = 1), tumor center (*n* = 2), tumor front (*n* = 2), and tumor microenvironment (*n* = 2) were annotated. Donor blocks were loaded into an automated tissue microarrayer. Using donor block images and annotated digital slides, transfer of 420 tissue cores created two ngTMAs. CD3, CD8 and CD45RO immunohistochemistry were performed.

**Results:** Scanning time was <10 h; annotation time was 1 h. Punching of tissue cores and transfer took 12 s/core. ngTMA construction took 1.4 h. Desired histological regions including tumor-stroma interaction captured the T-cell response.

**Conclusion:** ngTMA is a well-defined process based on planning and design, digital pathology, histological annotations and automated tissue microarraying. ngTMA is a promising approach that clearly supports the role of pathologists in translational research.

**OFP-04-005**

**Quantitative measurements of cell density and proliferative markers by image analysis**

A. Kudaybergenova ^*^

^*^RNCRCT, Pathology, St. Petersburg, Russia

**Objective:** Cell distribution and cell density are the main characteristic for tumor pathology. We do not yet understand how many cells inhabit a tumor from a 1 sq mm sample of histology slide. We analyzed a total number of tumor cells in breast cancer; renal cell carcinoma; lung carcinoma (NSC), and endometrial carcinoma and proliferative activity (% Ki67-positive cells) to establish the precise quantity of tumor cells per sq.mm of histological slide and the relations of this measure with proliferation.

**Method:** The study included 46 breast carcinomas; 44 RCC; 21 NSCLC, and 35 endometrial carcinoma.

**Results:** Mean absolute tumor cells in 1 mm2 of histology slide was for breast cancer: 4,160+/−251, Ki67–31 %; NSCL: 4,102+\\− 364 cells, Ki67–15 %; endometrial carcinoma: 7,073+\\− 614, Ki67–31.9 %; RCC 4,389+\\− 229, Ki67 11.62 %. There was moderate correlation between cell density and Ki67 for breast cancer *r* = 0.42 (*p* = 0.00018); for NSCL, *r* = 0,41 (*p* = 0,0,032), for endometrial carcinoma *r* = 0,48 (*p* = 0,0,059) and no correlation for RCC.

**Conclusion:** By analysis of these four types of cancer was established the quantity of tumor cells per mm2 and main the proliferative characteristics

**OFP-04-006**

**Automatic detection of tumor areas by applying homology method**

K. Nakane ^*^, Y. Tsuchihashi

^*^Osaka University, Dept. of Medicine, Suita, Japan

**Objective:** Computer assisted pathological diagnosis is now an issue of importance in the current situations of shortage of diagnostic pathologists in Japan. In the present study we propose a simple mathematical model to identify tumor tissues from their normal counterparts utilizing changes in the Betti numbers in tumorigenesis.

**Method:** The Betti numbers, which are consisting of two numbers (b1 and b0), can express the degree of the connection of the figure. When the number of contact points between individual components increases, irrespective of their shape, b1 and a ratio b1/b0 significantly change. We hypothesize these numbers can be used as indices to represent the cellular “accumulation” which is one of the characteristics of tumor tissue.

**Results:** Hematoxylin and eosin stained mucosal biopsy sections of colonic tumors are used as test samples. We compare the values, b1 and b1/b0, of colonic tumor tissues with those of normal tissues. As the values of b1 and b1/b0, we put color stones on unit area. The area of abnormal, it can be seen that the stone is placed

**Conclusion:** The results obtained in the present study clearly indicate that the difference in the Betti numbers can differentiate tumor tissues from their normal counterparts.

**Numerical Results:** 
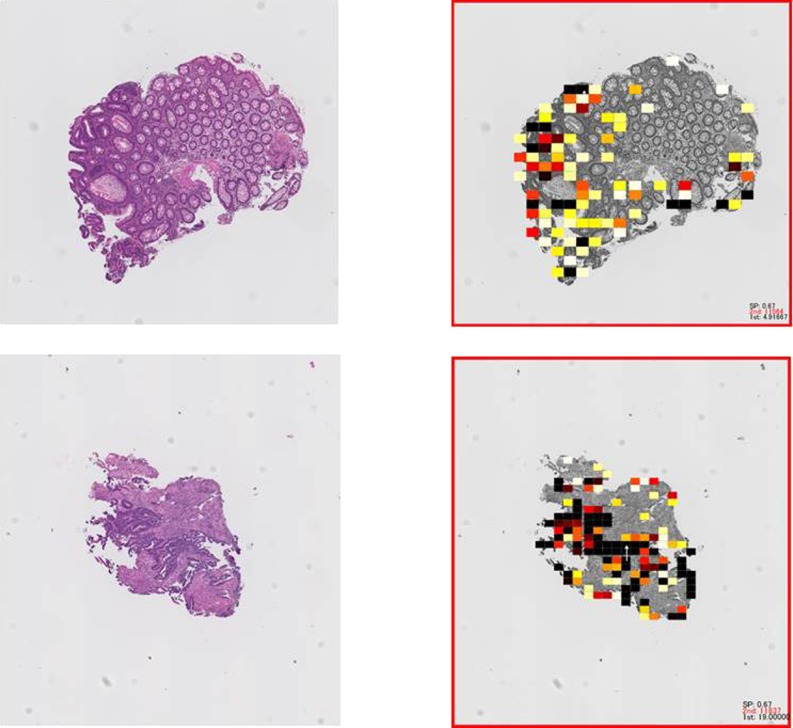


**OFP-04-007**

**Development of an automated 2D image reconstruction algorithm for multiple tissue blocks: Toward high-resolution whole organ 3D images**

N. Hashimoto ^*^, Y. Yagi

^*^Harvard Medical School and MGH, Pathology, Boston, USA

**Objective:** 3D image reconstruction from 100 s to 1,000 s of serial section Whole Slide Images (WSIs) from a single tissue block has been reported. Although the results were promising, high-resolution 3D images of whole organ offer more potential for new discoveries. There are a number of challenges in performing 3D image reconstruction of a whole tissue organ from WSIs. The objective of this study is to develop an algorithm to automatically reconstruct whole organ 2D images from WSIs of different tissue blocks, which were from the same slice of the tissue organ, since these WSIs may have different orientations and exact locations of tissue blocks are not known.

**Method:** We considered single slice of tissue organ. This slice is represented by WSIs of 5–15 blocks. Automatic 2D image reconstruction we have developed was implemented by finding the appropriate position and rotation of the images through template matching of low-resolution WSIs.

**Results:** Our 2D image reconstruction algorithm showed excellent results and the possibility to compare with gross or other modality images.

**Conclusion:** We developed the 2D image reconstruction method and confirmed its validity. It would be the important step to our goal “high-resolution whole organ 3D images”.

**OFP-04-008**

**Display systems for digital pathology**

T. Kimpe^*^, A. Xthona

^*^Barco, Healthcare Division, Kortrijk, Belgium

**Objective:** In digital pathology, pathologists make use of a display to look at scanned slides in order to come to a diagnosis. This paper presents clear guidelines of which specifications are needed for digital pathology displays.

**Method:** Image characteristics obtained when using current optical microscopes are examined. Special focus areas include luminance and color, stability and possible variation and instabilities among systems or over time. Characteristics of digital pathology scanners and display systems are also investigated and compared with current optical microscopes.

**Results:** When comparing characteristics of several displays, it has become clear that there is a very large variation in quality. Some (consumer) displays offer clearly lower image quality compared to optical microscope based systems, while other (typically medical) display systems specify similar or even clearly higher image quality compared to optical microscope based systems.

**Conclusion:** Pathologists make use of a display to look at scanned slides in order to come to a diagnosis. The display system therefore is a critical component, and it is important that certain minimum specification displays are used so that the same level of clinical performance can be obtained on digital systems as on optical microscopes.

**OFP-04-009**

**Larynx virtual microscopy validation study**

B. Sturm ^*^, S. Fleskens, F. Bot, L. van Velthuysen, E.-J. Speel, P. Slootweg, J. van der Laak

^*^Radboud University Nijmegen, Dept. of Pathology, The Netherlands

**Objective:** Whole Slide Imaging (WSI) is a technology in which histopathological slides are optically scanned to produce digital images. Aim of the present study is validation of WSI for tissue samples of pathologic lesions in which subtle differences could have major clinical implications.

**Method:** Larynx biopsies were used containing preneoplastic lesions. Glass slides were scanned using an Olympus dotSlide system with 40× objective. WSI were displayed at a calibrated monitor and were reviewed according to the 2005 WHO classification system by three pathologists with head and neck pathology as a field of interest. Diagnoses were converted into a three grade system relevant for clinical management. WSI diagnosis concordance rates were calculated. Kappa statistics were calculated to assess the degree of inter- and intra-observer agreement.

**Results:** The overall interobserver agreement was comparable for both WSI and glass slide diagnoses (unweighted kappa 0.38 and 0.39, respectively). The intraobserver (glass vs WSI) agreement ranges from 0.41 to 0.48 and 0.52–0.57 for unweighted and weighted *κ*-values respectively. WSI showed overall slightly less (although not statistically significant) concordance with the consensus diagnosis compared to glass slides.

**Conclusion:** This study shows that difficult diagnostic cases could be diagnosed using WSI, without compromising diagnostic quality.

**OFP-04-010**

**Students’ performance during practical examination on whole slide imaging using viewpath tracking**

J. Szymas ^*^, S. Walkowski, M. Lundin, J. Lundin

^*^University of Medical Sciences, Dept. of Clinical Pathology, Poznan, Poland

**Objective:** Introduction of whole slide imaging (WSI) to facilitate learning of oral cavity pathology by dental students has dramatically altered the way of teaching but little is known about students’ viewing behavior while interpreting WSI.

**Method:** We have decided to use tracking methods embedded in the WSI viewing system itself when viewing WSI by students during answering exam questions. Each time a student stopped panning and zooming for a for a time-period long enough for 100 % of the viewfield image data to be loaded, a record with coordinates in the virtual slide, timestamp and other metadata was sent to the database.

**Results:** Every student (*N* = 85) viewed 50 WSIs when answering 50 exam questions, generating in all more than 80,000 recorded viewfields. Mean number of viewfields/student was 1,600 and viewfields/WSI was 18.9. Correct students’ answers were 91.3 % (SD = 7.4 %). We calculated measures of total size of areas, dispersion of fragments and distribution of zoom levels viewed. Relationships were found between these measures and correctness of answers given by students.

**Conclusion:** The current tracking method during examination has provided us with a number of insights into the quality and effectiveness of WSI as a tool for learning and assessment.

Sunday, 1 September 2013, 17.00–19.00, Room 5C

**OFP-05 Oral Free Paper Session Soft Tissue Pathology**

**OFP-05-001**

**Expression of P16 in osteosarcoma as predictive neo-adjuvant therapy factor**

D. Borys ^*^, R. Canter, A. Horvai

^*^University of California, Dept. of Pathology, Davis, USA

**Objective:** Osteosarcoma (OS) is a common malignant primary tumor of bone affecting adolescent and young adults. Osteosarcomas are high grade sarcomas with aggressive behavior. There are few if any molecular markers to predict behavior and prognosis of osteosarcoma. The objective of this study is to investigate expression of p16 in correlation with neo-adjuvant chemotherapy response in osteosarcoma.

**Method:** A tissue micro array was created using paraffin embedded samples from 40 pretreatment osteosarcoma cases from two institutuions UC Davis and UCSF. Immunohistochemistry was performed with commercially available p16 monoclonal mouse antibody (mtm laboratories AG, Germany). Expression for p16 was defined as nuclear staining in at least 30 % of cells. Percent tumor necrosis was measured in post-chemotherapy resection specimens with good response set at >90 % necrosis.

**Results:** Patients age ranged from 9 to 75 years (mean 20). Most common locations were tibia and femur. 21 patients were female and 19 male. The clinical and p16 results are summarized in table 1. P16 expression correlated positively with median percent necrosis and fraction of cases with good chemotherapy response (*p* = 0.004 and 0.003, respectively).

**Conclusion:** Immunohistochemical expression of p16 significantly correlates with good chemotherapy response in osteosarcomas. p16 immunohistochemistry may be useful adjunctive marker of prognosis in osteosarcoma.

**OFP-05-002**

**RB1 and CDKNA2 gene deletions do not predict resistance to neoadjuvant chemotherapy in patients with osteosarcoma**

D. Borys ^*^, R. Canter, R. A. Schultz, A. Horvai

^*^University of California, Dept. of Pathology, Davis, USA

**Objective:** Pathologic response to neoadjuvant chemotherapy (NCH) predicts survival in osteosarcoma (OS) patients. The objective of the current study was to determine if copy number variations (CNV) of the CDKNA2 and Rb1 genes correlate with IHC expression of P16 and to determine whether CNV predict response or resistance to NCH.

**Method:** Pre-treatment genomic DNA was available from 31 pre-treatment cases of OS. CNV was determined by array comparative genomic hybridization (CGH). Clinical, pathologic, and copy number changes of the CDKNA2 and Rb1 genes were correlated for their association with good (>90 % necrosis)or poor (<90 % necrosis)response to chemotherapy.

**Results:** Array CGH was informative in 23 of 31 OS tumors. Among P16 negative tumors by IHC, CDKNA2 homozygous gene deletion was present in only 1 case (14 %), while the remainder had intact CDKNA2 or, in 2 cases, a gain, and LOH (loss of heterozygosity). Although homozygous Rb1 deletion was identified in 15 cases (65 %), there was no difference in the prevalence of Rb1 gene deletions between good (8/12) and poor responders (7/11) to chemotherapy (67 % vs. 64 %, *P* = 1.0).

**Conclusion:** Loss of P16 expression in OS is associated with resistance to chemotherapy, only a minority of cases result from deletion of the CDKNA2 gene. Rb1 gene deletions was not associated with response or resistance to NCH.

**OFP-05-003**

**Dissecting spindle and pure pleomorphic variants in undifferentiated soft tissue sarcomas by gene expression profile**

V. Canzonieri ^*^, E. Pivetta, B. Wassermann, T. Perin, P. Spessotto, A. Colombatti

^*^CRO-IRCCS Aviano NCI, Dept. of Pathology, Italy

**Objective:** Soft tissue sarcoma (STS) clusters a heterogeneous group of tumors: more than 50 subtypes are currently diagnosed by genetic and morphological criteria. Among STS, Undifferentiated Soft Tissue Sarcoma (USTS) is a matter of discussion both for etiologic and diagnostic criteria. The aim of this study was to identify a new biomarker profile that could distinguish prevalent spindle vs prevalent pleomorphic USTS variants, as defined on the basis of morphologic evaluation.

**Method:** We carried out a gene expression analysis on 40 different USTS samples using Affymetrix microarray slides. A supervised hierarchical analysis on obtained microarray data, qRT-PCR and immunohistochemical analyses were performed.

**Results:** The two USTS variants (spindle, sUSTS and pleomorphic, pUSTS) clearly emerged from expression gene analysis. The number of the genes differentially expressed belonged to different categories (from translation regulators to ATP-binding proteins). qRT-PCR confirmed the analysis and immunohistochemistry for four proteins validated the obtained results.

**Conclusion:** Despite the outcomes require to be confirmed with a greater case study, we identified four genes (IGF2BP1, FAM133A, ABCA13, and EYA4) helpful in discriminating sUSTS vs pUSTS. This short panel of biomarkers could identify patient subsets with an especially unfavorable prognosis, thereby allowing stratification to a more aggressive and effective therapy.

**OFP-05-004**

**TGFBR3 and MGEA5 rearrangements in pleomorphic hyalinizing angiectatic tumors of soft parts**

J. Carter ^*^, E. Montgomery, A. Folpe

^*^Mayo Clinic, Dept. of Pathology, Rochester, USA

**Objective:** Pleomorphic hyalinizing angiectatic tumor of soft parts (PHAT) is a rare, locally aggressive soft tissue neoplasm, typically occurring in the lower extremities. The putative precursor lesion of PHAT, termed “early PHAT” (EPHAT), shares many clinicopathological features with hemosiderotic fibrolipomatous tumor (HFLT). At the genetic level, HFLT, myxoinflammatory fibroblastic sarcoma (MIFS) and tumors showing hybrid features of HFLT and MIFS often show TGFBR3-MGEA5 rearrangements. To date, only a very small number of PHAT have been tested for this rearrangement; all have been negative.

**Method:** Four cases of PHAT, all containing EPHAT and lacking features of MIFS, were retrieved and evaluated for rearrangements in TGFBR3 and MGEA5 by FISH.

**Results:** The tumors occurred in adults (2M:2F; 37–55 years) and involved the foot, ankle and lower leg (2). Three of 4 cases were positive for rearrangements of TGFBR3 and MGEA5.

**Conclusion:** We report for the first time the presence of TGFBR3 and MGEA5 rearrangements in tumors showing mixed features of EPHAT and PHAT. The presence of such rearrangements, and the essentially identical morphological features of EPHAT and HFLT, strongly suggests that EPHAT/HFLT is related to both PHAT and MIFS, and that the latter two tumors may represent different morphological stages of a single entity in which only MIFS has acquired the capacity to metastasize.

**OFP-05-005**

**SMARCB1 mRNA expression is regulated by microRNAs in epithelioid sarcoma**

G. Papp ^*^, Z. Sápi

^*^Semmelweis University, 1st Dept. of Pathology, Budapest, Hungary

**Objective:** About 10 % of epithelioid sarcomas have biallelic mutation of the SMARCB1 gene resulting in lack of this nuclear protein. Our previous studies have shown that neither the gene promoter nor the histone methylation was responsible for the functional loss of INI1 in epithelioid sarcomas. Moreover there was no SMARCB1 mRNA detected in the laser microdissected tumor cells. It was speculated that miRNAs (microRNAs) may contribute to SMARCB1 gene silencing in this tumor.

**Method:** We identified SMARCB1-specific and upregulated miRNAs and examined their functional effects using in vitro experiments.

**Results:** 8 potential SMARCB1-specific miRNAs were collected from online databases. Upregulations of miR-206/381/671-5p/765 were determined by quantitative real-time polymerase chain reaction in the 25 SMARCB1 negative cases. For in vitro tests 3 cell cultures showing normal SMARCB1 expression (HT1080, CaCo-2 cell lines and primary culture of human fibroblasts) were transfected with Pre-miR miRNA Precursors of abovementioned 4 miRNAs using electroporation. Three miRNAs (miR-206/381/671-5p) decreased the level of SMARCB1 gene expression in the cultured cells.

**Conclusion:** Our results suggest that miR-206/381/671-5p overexpression seems to be one important factor in the oncogenesis and progression in epithelioid sarcomas through SMARCB1 downregulation.

**OFP-05-006**

**Preoperative intensity-modulated radiation therapy (IMRT) for high-risk soft tissue sarcomas of extremities and retroperitoneum: Histopathological response and molecular aspects**

M. Straub ^*^, K. Specht, H. Rechl, R. Eisenhart-Rothe von, B. Röper

^*^TU Munich, Pathology, Germany

**Objective:** Neoadjuvant radiotherapy is a promising therapeutic option for high-grade soft tissue sarcomas (hSTS). Histopathologic response following IMRT for hSTS was evaluated.

**Method:** Pretherapeutic biopsies and resection specimen of 41 hSTS (14 pleomorphic sarcomas, 9 myxofibrosarcomas, 5 synovial sarcomas, 7 dedifferentiated liposarcomas (DLS), 3 myxoid/roundcell liposarcomas, 1 epitheloid sarcoma, 1 rhabdomyosarcoma, 1 leiomyosarcoma) after preoperative IMRT were included. Percentage of vital tumor and resection margins were evaluated histomorphologically. P53 and p16 status was analyzed by methylation-specific PCR, sequencing and immunohistochemistry.

**Results:** Median age of patients was 57 year (24–89; M:F ratio 20/21). Anatomical locations were extremities (*n* = 36) and retroperitoneum (*n* = 5). Metastasis occurred in 11 patients, local relapse in *n* = 6. A clear resection margin (R0) was achieved in 31 patients, R1-resection in 6 patients (4 with retroperitoneal hSTS) and Rx-resection in *n* = 4. Percentage of vital tumor ranged from 5 to 80 % (median 45 %). In genomically highly complex sarcomas, median of vital tumor tissue was 30 % as opposed to sarcomas with intermediate genomic complexity (median 70 %) and translocation-associated sarcomas (median 60 %). Molecular and immunohistochemical analysis of p16 and p53 status is under way.

**Conclusion:** A high percentage of R0-resections can be achieved in STS of extremities after radiotherapy. Response rates vary widely, with certain subgroups (genomically complex sarcomas) displaying a better response.

**OFP-05-007**

**Absence of CHOP translocation and MDM2 amplification in a case of lipoblastoma-like tumor of the vulva**

C. A. Vásquez Dongo ^*^, C. Delbene Azanza, B. Ferrer Fabrega, P. Jiménez León, Y. Rodriguez Diez, D. Bodet Castillo, C. Valverde Morales, S. Ramón y Cajal Agüeras, C. Romagosa Perez-Portabella

^*^Vall d’Hebron U. Hospital, Anatomical Pathology, Barcelona, Spain

**Objective:** Lipoblastoma-like tumor of the vulva (LBLTV) is a rare mesenchymal neoplasm that can mimic myxoid liposarcoma. Molecular changes which are important in the differential diagnosis of these tumors, such as CHOP translocation and MDM2 amplification haven’t been reported before.

**Method:** We described the case of a painful and well-circumscribed mass in the vulva of a 28 year-old woman and the main clues for differential-diagnosis including morphology, immunophenotype and FISH.

**Results:** The lesion was mainly myxoid, with clusters of mature adipose tissue. The tumor cells were spindle and bland. Some lipoblasts and a chicken-wire vascular network were also observed. The differential-diagnosis was mainly done between myxoid liposarcoma, dedifferentiated liposarcoma, lipoblastoma and the myxoid vulvar lesions. Immunohistochemicaly, the tumor cells were negative for CD34, smooth-muscle actin, desmin, neurofilaments and S100. FISH didn’t show CHOP translocated cells or MDM2 amplification. Once previous diagnoses had been ruled out, the final diagnosis was LBLTV which was surgical removed with security margins being free of disease 2,5 years after.

**Conclusion:** Although LBLTV is a rare diagnosis, it should be considered in the differential-diagnosis of myxoid and adipose vulvar tumors. The absence of CHOP translocation and MDM2 amplification can help to differentiate these benign tumors from their main malignant differential-diagnoses.

Monday, 2 September 2013, 14.15–16.15, Room 5B

**OFP-06 Oral Free Paper Session Endocrine Pathology**

**OFP-06-001**

**Cellular retinoic acid binding protein I expression and cell proliferation in pancreatic neuroendocrine tumors**

V. Delektorskaya ^*^, G. Chemeris, N. Kozlov

^*^N.N. Blokhin Russian Cancer Res., Dept. of Pathology of Human Tumors, Moscow, Russia

**Objective:** The aim of the study was to investigate Cellular Retinoic Acid Binding protein type I (CRABP I) expression and its clinical significance in pancreatic neuroendocrine tumors (pNETs).

**Method:** Protein expression of CRABP I was investigated by immunohistochemical analysis in 71 pNETs of various histological subtypes, including well- and poorly differentiated neuroendocrine neoplasms. Relationship of CRABP I expression to clinical and pathological features, grading and cell proliferation (index Ki-67) was analyzed.

**Results:** Increased expression of CRABP I protein was detected in 43.7 % (31of 71) of cases, including 16.7 % (1 of 6), 42.1 % (24 of 57) and 75.0 % (6 of 8) of pNET G1, pNET G2 and pNEC G3, respectively. We found the significant relationship of increased CRABP I expression to histological differentiation (*p* = 0.003), primary tumor size (*p* = 0.024), lymph node and liver metastases (*p* = 0.017 and *p* = 0.024, respectively) and cell proliferation, evaluated on the basis of the Ki-67(MIB-1) antigen expression (*p* = 0.000). The protein expression was not associated with outcome and survival of pNET patients.

**Conclusion:** The increased protein expression and association with high-risk factors suggest the great potential prognostic and therapeutic interest of evaluation of CRABP I in pNETs. Further study is necessary to clarify the role of CRABP I protein in pNET pathogenesis.

**CRABP I expression in the cell cytoplasm of pNET G2 (Ki-67–9 %).:** 
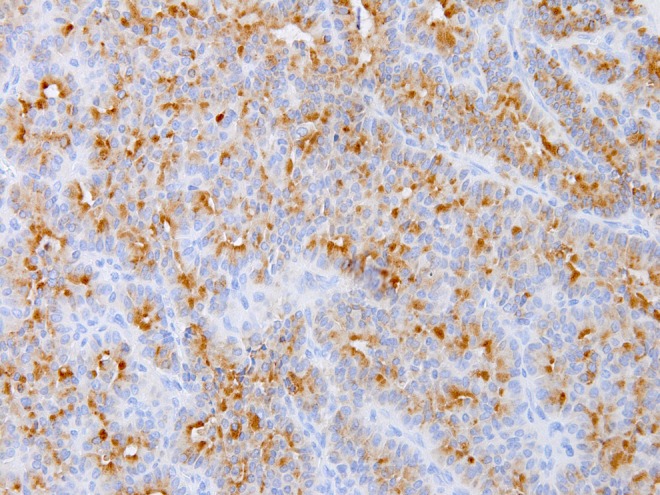


**OFP-06-002**

**Succinate dehydrogenase subunit B- mutation-related pheochromocytomas and sympathetic paragangliomas: Gene analyses and clinicopathological study**

N. Kimura ^*^, K. Takekoshi

^*^National Hakodate Hospital, Dept. of Pathology, Japan

**Objective:** The aim of this study was to examine the clinicopathological features of succinate dehydrogenase subunit B (SDHB) mutation-related pheochromocytoma/paraganglioma (PCC/PGL).

**Method:** A total of 15 patients with SDHB-mutation were analyzed genomic DNA, clinical data on family history, catecholamine types, tumor metastasis, patient prognosis, and histology of the tumors. Histologic analyses were performed using the new classification named Grading of adrenal pheochromocytoma and paraganglioma (GAPP) classification that was made based on a nationwide Japanese survey. All tumors were scored from 0 to 10 points, and were also graded according to three differentiation types depending on the total score: well (0–2 points), moderate (3–6) and poor (7–10).

**Results:** Mean age was 32.3 ± 14.2, and sex ratio was 10M: 5F. Mutation sites were exon 2 (R46Q) in 6, exon 5 (L157X) in 4, and exon 3, 6, 7, intron 2 in one patient, respectively. Five patients had a family history. Catecholamine phenotypes were 12 in norepinephrine-, and three in nonfunctioning type. Nine patients had metastases. Histological grading of the tumors was 4.87 ± 0.99, corresponding to moderate type differentiation.

**Conclusion:** Tumors with SDHB mutations were characterized by moderate type differentiation with 53 % of a pseudorosette pattern.

**OFP-06-003**

**Ki-67 antigen in pituitary adenomas: Role in the tumor behaviour prognosis**

L. Mitrofanova ^*^, U. Tsoy, P. Ryazanov, O. Beshchuk, V. Cherebillo, E. Grineva

^*^Almazov’s Centre, Dept. of Pathomorphology, St. Petersburg, Russia

**Objective:** To establish the relationship of the Ki-67 index with tumor size, hormonal activity and tumor behavior.

**Method:** We evaluated the expression of Ki-67 in 46 anterior pituitary adenomas. Tumors size and growth were verified by MRI. We used different criteria for invasive tumor definition: extrasellar growth, cavernous sinus infiltration, maximal diameter of the tumor >3 cm, tumor volume >5 ml.

**Results:** 14 adenomas were non-functioning and 32 functioning (25 growth hormone, 5 ACTH, 2 prolactin). In spite of applying different criteria for invasive tumor definition, there was no difference between the Ki-67 index in invasive and non-invasive adenomas. The Ki-67 index was higher in ACTH-secreting adenomas, though all of them were microadenomas, comparing with the rest ones (4,48 ± 1,54 % vs 1,05 ± 0,18 %, *p* < 0,002). In non-functioning adenomas positive correlation between the Ki-67 index and tumor volume was revealed (*r* = 0,64 *p* = 0,04). The Ki-67 index was significantly higher in adenomas which relapsed less than 2 years after the first operation (3,82 ± 1,8 vs 1,13 ± 0,19, *p* = 0,0013).

**Conclusion:** We did not get the data confirming evidence that the Ki-67 index can predict invasive growth of pituitary adenomas. Assessment of the Ki-67 index may be helpful in predicting tumor recurrence.

**OFP-06-004**

**A study of GATA3 immunohistochemistry in parathyroid and thyroid lesions**

G. Betts ^*^, E. Beckett, D. Nonaka

^*^Christie Hospital, Dept. of Histopathology, Manchester, United Kingdom

**Objective:** GATA3 (GATA binding protein 3) is a transcription factor involved in tissue differentiation in the breast, kidney, thymus and parathyroid. Whilst thyroid tumours have been reported to be negative for GATA3, no previous reports have documented expression of GATA3 in parathyroid lesions.

**Method:** Fifty five parathyroid lesions were assessed using immunohistochemistry (IHC) for GATA3 including parathyroid adenomas(35), parathyroid hyperplasia(9), atypical parathyroid adenoma(3), parathyroid cyst(2) and parathyroid carcinoma(5) with one case of recurrent hyperplasia after autotransplantation in the arm. A tissue microarray was also immunostained for GATA3 containing 58 evaluable thyroid lesions including; follicular adenoma(8), hurthle cell adenoma(8), follicular carcinoma(10), hurthle cell carcinoma(2), papillary carcinoma(14), medullary carcinoma(8), anaplastic carcinoma(1) and insular carcinoma(7).

**Results:** All of the parathyroid lesions were positive for GATA3, 96 % (53/55) of cases displayed strong, diffuse nuclear positivity. An atypical parathyroid adenoma showed weak diffuse nuclear positivity and a parathyroid carcinoma, focal strong nuclear positivity. None of the thyroid lesions displayed any nuclear positivity, two insular carcinomas showed weak cytoplasmic positivity.

**Conclusion:** Nuclear positivity with GATA3 IHC is highly specific for parathyroid tissue when compared with thyroid tissue. GATA3 IHC may be a useful adjunct for histological and cytological discrimination of parathyroid and thyroid lesions.

**OFP-06-005**

**Grading of Neuro Endocrine Tumors (NET): Mitosis and Ki-67 both essential?**

M.-L. van Velthuysen ^*^, T. Korse, A. van der Pol, M. Tesselaar

^*^AvL, Pathology, Amsterdam, The Netherlands

**Objective:** We investigated whether ki-67 and mitotic index assesment would lead to discordant grading and which of both is most informative to predict survival.

**Method:** Tumors of 252 patients with NET were analyzed for mitoses and Ki-67 index. Up till now survival data are available from 85 patients

**Results:** Of 252 cases, 88 showed discordance in grading between Ki-67 and mitotic index. In 37 cases this was due to a Ki-67 index ≥20, while mitoses were <20. In 25 of these cases mitotic index was <10, in 8 of these cases even <2. The ratio for biopsies and resection specimens was 1:1 for the cases with concordant mitosis and ki-67 index, while for the cases with discordant indexes this ratio was 7:3. Preliminary survival analysis of the 85 patients, showed that ki-67 index and mitotic index were both predictive for survival. However, for the patients with discordance between mitotic index and Ki-67 index (*n* = 31) the Ki-67 seemed to be the best parameter for survival.

**Conclusion:** Grading NET using Ki-67 and mitotic index results in conflicting data in 30 % of cases. Survival analysis suggests that Ki-67 is a better prognostic marker as compared to mitotic index. Correlation with survival analysis of all included patients is ongoing

**OFP-06-006**

**Prognostic and predictive role of MGMT immunoreactivity and promoter methylation in sporadic pancreatic endocrine neoplasms**

A. M. Schmitt ^*^, M. Pavel, T. Rudolph, H. Dawson, E. Vassella, A. Perren

^*^University of Bern, Institute of Pathology, Switzerland

**Objective:** To determine the value of MGMT protein expression and promoter methylation in pNEN regarding 1. prognosis and 2. response prediction to temozolomide therapy.

**Method:** Two independent cohorts of pNEN were analyzed: 1. Prognostic cohort: Patients did not receive TMZ. Methylation specific PCR of the MGMT promoter (*n* = 52) and MGMT immunohistochemistry (*n* = 130) were performed. Results were correlated with survival data. 2. Predictive cohort: Primer extension based quantitative PCR of the MGMT promoter and MGMT immunohistochemistry were performed in 15 progressive metastasized NEN receiving TMZ chemotherapy and results correlated with radiological tumour response according to RECIST 1.1.

**Results:** 1. Prognostic cohort: Loss of MGMT protein expression, but not MGMT promoter methylation correlated significantly with a shortened disease free survival (*p* = 0.0054) and decreased disease specific survival (*p* = 0.0322). 2. Predictive cohort: MGMT promoter methylation, but not loss of MGMT protein expression correlated significantly with response (*p* = 0.036). All tumours showing hypermethylation of the MGMT promoter were of pancreatic origin and showed either stable disease or partial remission.

**Conclusion:** 1. Loss of MGMT expression is an adverse prognostic marker in sporadic surgically resected pNEN. 2. MGMT promoter methylation in sporadic metastasized pNEN might serve as a predictor of response to TMZ.

**OFP-06-007**

**Application of molecular markers in cytological samples of thyroid nodules as pre-operative predictors of aggressiveness**

M. Volante ^*^, I. Rapa, J. Giorcelli, S. Aversa, P. Caraci, A. Fulcheri, A. Votta, F. Orlandi, M. Papotti

^*^University of Turin, Oncology at San Luigi Hospital, Orbassano, Turin, Italy

**Objective:** To test in thyroid cytology the role of molecular markers in predicting pathological features of potential impact in the pre-operative clinical management of thyroid neoplasms.

**Method:** A retrospective series of 201 consecutive FNA samples (from years 2004–2009) with a diagnosis of “thyroid neoplasm” (British Thyroid Association diagnostic categories: THY 3, 125 cases; THY 4, 27 cases; THY 5, 49 cases) with available histological diagnoses were selected. BRAF, N and H RAS mutations were screened using the pyrosequencing method onto DNA extracted from cell block preparations. Mutational profile was correlated with histological diagnosis and clinical-pathological characteristics of tumors at histology.

**Results:** BRAF mutations were detected in 37/201 (18 %) cases with overall sensitivity and specificity for malignancy of 31 % and 97 %, respectively. BRAF mutations were significantly associated with papillary carcinoma histotype, presence of multifocality and extra-thyroidal invasion but not with lymph node metastases. RAS mutations had low sensitivity and specificity and poor correlation with pathological features of aggressiveness, except for an inverse correlation with vascular invasion

**Conclusion:** the role of molecular markers in the pre-operative characterization of thyroid neoplasms at cytology is limited to BRAF, although the practical impact and cost-to-benefit ratio of such analysis still need to be addressed.

**OFP-06-008**

**Expression of DNA repair and synthesis enzymes and tyrosine kinase inhibitor(s) targets in poorly differentiated and anaplastic thyroid cancer**

M. Volante ^*^, V. Monica, I. Rapa, S. Busso, J. Giorcelli, G. Scagliotti, M. Papotti

^*^University of Turin, Oncology at San Luigi Hospital, Orbassano, Turin, Italy

**Objective:** To analyze the expression of enzymes involved in DNA repair and synthesis and of VEGF and PDGF receptors in aggressive thyroid cancers as potential biomarkers of response to chemotherapy and tyrosine kinase inhibitors

**Method:** 35 poorly differentiated (PD) and 19 anaplastic thyroid carcinomas were analyzed for gene expression of ERCC1, RRM1, Topoisomerase IIa (TOPOIIa), thymidylate synthase (TS), VEGF-R types 1-2-3 and PDGF-R using real time PCR, as compared to normal thyroid tissue. Gene expression levels were compared to tumor type, type of well-differentiated associated component, presence of oncocytic features and genotype (RAS vs BRAF mutations)

**Results:** All genes investigated were significantly over-expressed in tumors vs normal thyroid. TS and ERCC1 were significantly over-expressed in PD as compared to anaplastic carcinomas. TS and RRM1 were significantly down- and up-regulated in BRAF-positive tumors, respectively. All VEGF-R subtypes were significantly over-expressed in PD as compared to anaplastic carcinomas, whereas the reverse occurred for PDGF-R. VEGF-R1 and PDGF-R were also significantly up-regulated and down-regulated, respectively, in oncocytic vs non-oncocytic tumors

**Conclusion:** All markers investigated showed heterogeneous expression profiles partially associated to pathological or genotypic tumor characteristics that might potentially reflect differences in profiles of responsiveness to chemotherapy or to selective targeted therapies

**OFP-06-009**

**MiR-885-5p plays a causal role in the development of oncocytic phenotype in thyroid tumors**

M. Dettmer ^*^, M. Gandhi, M. Nikiforova, Y. Nikiforov

^*^Universität Bern, Switzerland

**Objective:** The molecular profile of oncocytic follicular thyroid carcinomas (oFTC) is largely unknown. We reported that miR-885-5p is upregulated in oFTC. The aim of this study was to explore the relationships between miR-885-5p overexpression and oncocytic phenotype of thyroid tumors.

**Method:** MiR-885-5p was stable overexpressed in human thyroid HTori-3 and TPC-1 cells. Cox4 gene is a part of the mitochondrial respiratory chain and was used to monitor the accumulation of mitochondria. Cells were stained with Cox4 antibody prior to FACS analysis (*n* = 5) and immunofluorescence and the amount of mitochondria was evaluated.

**Results:** Overexpression of miR-885-5p in both cell lines resulted in a significant increase of the Cox4 mitochondrial signal detected by the FACS analysis compared to controls (controls:58.2 ± 2.7 % vs. miR-885-5p:71.9 ± 1.2 %). Immunofluorescent analysis confirmed a significant increase in the Cox4 staining of the cytoplasm of the transduced cells, confirming the increase in the amount of mitochondria.

**Conclusion:** This study provides for the first time the experimental evidence that a single miRNA, miR-885-5p, is involved in the accumulation of mitochondria in thyroid cells, leading to the acquisition of the oncocytic features in human thyroid cells. Further studies are required to elucidate the target genes of miR-885-5p and the mechanisms behind this phenomen.

**OFP-06-010**

**Galectin-3 and HBME-1 expression in follicular thyroid tumors, especially in so called “follicular thyroid tumors of uncertain malignant potential” and determination of their histological features**

E. Tamponi ^*^, G. Faa, G. Senes, M. L. Lai

^*^AOU Cagliari, Dipt. di Anatomia Patologica, Italy

**Objective:** Investigate expression and diagnostic rule in follicular thyroid tumors, especially in so called “follicular thyroid tumors of uncertain malignant potential” of galectin-3 and HBME-1. Investigate histological behaviour of follicular thyroid tumors of uncertain malignant potential especially in term of capsule invasion mode and venous or lymph invasion.

**Method:** A total of 924 follicular thyroid nodules were collected from our institution including 315 follicular adenomas (FA), 117 follicular thyroid carcinomas (FTC), 394 follicular variant of papillary thyroid carcinomas (FVPTC), 82 differentiated carcinomas, not otherwise specified (DCNOS), 17 well differentiated tumors of uncertain malignant potential.

**Results:** About 4 % of FA expressed HBME-1 and galectin-3, weakly. About 50 % of FTC expressed HBME-1 and galectin-3 with weakly or moderate stain. About 80 % of FVPTC expressed HBME-1 and galectin-3 with strong stain. About 65 % of DCNOS expressed HBME-1 and galectin-3 with almost strong stain. About 26 % of DCNOS shows venous invasion and all of them show the same way of capsule invasion of FTC.

**Conclusion:** DCNOS show histological features (capsule invasion mode and venous invasion) very similar to FTC and expression of galectin-3 and HBME-1 very similar to FV-PTC. We strong believe that DCNOS in a new entity and its histological and clinical behaviour has to be assessed.

Monday, 2 September 2013, 14.15–16.15, Room 5C

**OFP-07 Oral Free Paper Session Head and Neck Pathology**

**OFP-07-001**

**Adult sinonasal sarcoma: A clinicopathologic analysis of 48 cases from the French Sarcoma Group (** **http://www.conticabase.org** **)**

V. Costes ^*^, V. Szablewski, A. Neuville, A. Gomez Brouchet, M. Lae, D. Ranchere Vince, P. Terrier, G. d. Pinieux, J. M. Coindre

^*^Hopital Gui de Chauliac, Dept. of Biopathology, Montpellier, France

**Objective:** The aim of this study was to determine the frequency of primary sinonasal adult sarcomas, to identify the different histological subtypes, and to analyze prognostic factors.

**Method:** Forty-eight cases of adult sinonasal sarcoma from the French Sarcoma Group database (conticabase) were reviewed with immunohistochemistry and molecular characterization.

**Results:** The most frequent tumor types were alveolar rhabdomyosarcoma (ARMS) (33.3 %), embryonal rhabdomyosarcoma (ERMS) (14,6 %), unclassified sarcoma (14.6 %) and leiomyosarcoma (12,5 %). All “round cells” sarcomas were rhabdomyosarcoma. A total of 41.7 % of cases were grade 3.10.4 % of patients had previous medical history of radiotherapy (PHR). The 5-year OS, MFS and LRFS were 62.3 %, 73 % and 88.8 %. Statistical analysis retained histological type for predicting OS, MFS and LRFS with the worst prognosis associated with rhabdomyosarcoma, whatever the subtype alveolar or embryonal. Grade influenced OS and MFS. The predictive factor for complete response was surgery, whatever quality of resection margins.

**Conclusion:** Rhabdomyosarcoma represented the most frequent sarcoma in this topography and was associated with the worst prognosis, without significant differences between alveolar and embryonal subtypes. These results suggest that paranasal sinuses and nasal cavity should be considered as an unfavorable site for ERMS. Moreover, surgery should be always considered in treatment even if wide resection cannot be obtained.

**OFP-07-002**

**T Lymphoma of the nasal cavities or paranasal sinuses (12 cases)**

F. Bougrine ^*^

^*^Military Hospital Tunis, Dept. of Pathology, Tunisia

**Objective:** T lymphomas of the nasal cavities or paranasal sinuses are unusual tumors, rarely seen in Occidental countries, where B cell lymphomas are the most common, They often lead to differential diagnosis problems with other destructive and necrotizing processes of the sinonasal tract.

**Method:** 12 cases of T lymphomas of the nasal cavities or paranasal sinuses have been retrieved at the department of pathology of Military hospital of Tunisia. during a period of 16 years, from 1995 to 2010. Follow-up was available for 10 patients

**Results:** The median age was of 36 year. Clinical presentation was dominated by nasal obstruction (11 patients), associated or not to nasal discharge (10 cases), epistaxis (3 cases), and nasal swelling (5 cases). The most frequent site of occurrence was the nasal cavity. The histopathological examination found: 4 cases of T/NK lymphomas and 8 cases of peripheral T lymphomas NOS. Detection of Epstein Barr virus was positive in10 cases. Outcome was marked by: Death occurrence (7 patients), a complete remission (3 cases).

**Conclusion:** T lymphomas of the nasal cavities are rare in Tunisia. They are agressive lymphomas, characterized by an heterogenous and little specific clinical presentation. Etiology is unknown, but there is an association between EBV and T lymphomas of the nasal cavities.

**OFP-07-003**

**Differential occurrence of sinonasal Intestinal Type Adenocarcinoma (ITAC) and sinonasal non-ITAC in Finland and France and their association with wood dust exposure**

H. Wolff ^*^, I. Leivo, R. Holmila, D. Luce, K. Husgafvel-Pursiainen

^*^Finnish Inst. of Occup. Health, Laboratory of Pathology, Helsinki, Finland

**Objective:** The WHO classification (2005) distinguishes two types of sinonasal adenocarcinomas (SNAC) the intestinal-type adenocarcinoma (ITAC) and the non-ITAC. In the pathology literature ITACs but not non-ITACs have been strongly associated with hardwood dust exposure. We have studied the distribution of adenocarcinoma types and wood dust exposure in tumors from France and Finland. Hardwoods are typically used in France while in Finland softwoods dominate.

**Method:** Paraffin-embedded tissue (PET) samples were obtained from SNAC:s in Finland for the period 1989–2002. In France, cases were from the areas of Isere, Somme and Doubs, 1990–2002, Patients or next-of-kins were interviewed using a structured questionnaire

**Results:** Immunohistochemical (IHC) positivity for either CDX2 or CDX2 was used as a criteria for ITAC:s. Of 34 SNAC:s from France 29 were ITACs (85 %). while only 43 % of the SNACSs(*n* = 21) from Finland were ITACs. 87 % of ITACs and 46 % of non-ITACs were assosiated with wood dust exposure

**Conclusion:** Qualitative differences in wood dust exposure is a probable explanation for the differential frequencies of ITACs and non-ITACs in these countries. The association of non-ITACs with wood dust exposure is lesser than for ITACs. Use of IHC is recommended for differentiating ITACs from non-ITACs.

**OFP-07-004**

**Diagnostic challenges of antrochoanal polyps**

N. Choudhury^*^, A. Hariri, H. Saleh, A. Sandison

^*^Charing Cross Hospital, London, United Kingdom

**Objective:** Antrochoanal polyps (ACP) are benign, solitary, unilateral lesions. It is accepted that they are accurately diagnosed clinically, but ACPs may infact mimic a variety of other benign and malignant conditions. Patients require a full diagnostic work up including nasal endoscopy, radiology and histological analysis of the resection specimen. Clinically atypical lesions merit referral to a tertiary centre.

**Method:** A retrospective review of all patients with a clinical and radiological diagnosis of ACPs made over a 7 years study period was conducted. Their histology was reviewed and patients with a diagnosis other than ACP were further evaluated.

**Results:** 60 patients with a provisional diagnosis of ACP, who underwent endoscopic sinus surgery, were identified. Following surgery, 12patients (20 %) with discrepant histology were identified (7males and 5 females; mean age of 51.0+/−16.5 years). Of these, 10 had benign pathology including inverted papilloma, oncocytic papilloma, capillary haemangioma, angiofibroma and fungal ball. There were also 2 malignant cases including malignant melanoma and cycindical cell carcinoma.

**Conclusion:** ACP may mimic a diverse range of nasal pathologies. Our series has identified 20 % of patients with presumed ACP to have a different underlying diagnosis, following biopsy. Although individually these pathologies may be rare, careful histopathological analysis, with appropriate referral for specialist treatment, is necessary.

**OFP-07-005**

**Insignificant differences in VEGF-C/D immunohistoexpression and lymphatic microvessel density between primary and recurrent salivary pleomorphic adenoma do not support the role of lymphangiogenesis in local spread of this tumor**

I. Stárek ^*^, R. Salzman, L. Kucerová, A. Skálová

^*^Medical Faculty Olomouc, ORL Clinic, Czech Republic

**Objective:** Recurrent pleomorphic adenoma (RPA) is typical by multiple nodules present far away from the primary site. One of the plausible explanation of this feature might be local lymphatic spread of tumor cells, promoted by lymphangiogenesis. In our study, immunohistochemical expression of lymphangiogenic factors VEGF-C and VEGF-D and lymphatic microvessel density (LVD) was tested in PA and RPA.

**Method:** 6 primary-non-RPA, 4 primary-to-recur PA and 10 RPA were tested. For the evaluation of VEGF-C and VEGF-D reactivity semiquantitative histoscore was used, LVD was determined as the number of D2-40 positive peritumoral lymphatic capillaries.

**Results:** No case showed VEGF-C positivity. VEGF-D reaction was found exclusively in epithelial tumor cells. VEGF-D histoscore and LVD in primary-non-RPA, primary-to-recur PA and RPA ranged from 4 to 6 (mean 5.17), 3–5 (4.25) and 4–5 (4.6), respectively, and 2–5 (3.17), 1–7 (3.75) and 1–7 (3.4), respectively. The differences were not stastically significant (*p* > 0.05). No correlation between VEGF-D histoscore and LVD was found (*p* > 0.56).

**Conclusion:** Our results suggest that lymphangiogenesis plays no role in local spread of RPA. Supported by the Instutitional Support of Ministry of Health, Czech Republic, Nr. 1RVO-FNOl.2013, and IGA Czech Republic, Nr. NT13701-4/2012.

**OFP-07-006**

**In-situ and minimally invasive neoplasia in a series of 49 cases of salivary carcinoma ex-pleomorphic adenoma**

M. Rito ^*^, I. Fonseca

^*^IPO de Lisboa Francisco Gentil, Serviço de Anatomia Patológica, Portugal

**Objective:** Carcinoma ex-pleomorphic adenoma (Ca-ex PA) is defined by the presence of malignancy in a pre-existing pleomorphic adenoma. This study aims to review its morphological characteristics in a large series of salivary Ca-ex-PA and to evaluate the impact on disease progression.

**Method:** Forty-nine cases, diagnosed from 1988 to 2013, were classified for the malignant component(s) subtypes and assessed for the surgical margins status and extension of invasion, mitotic counts, hemorrhage, necrosis, neural and vascular invasion.

**Results:** All patients were treated surgically, 53 % with incomplete excision. 45 % of tumors had criteria of in situ and minimal invasion. 14 cases had low-grade and 35 cases high-grade histology. The predominant malignant components were: salivary duct carcinoma (47 %), myoepithelial carcinoma (22 %) and epithelial-myoepithelial carcinoma (16 %). 40 cases had follow-up information: 13 patients had disease progression and 6 died, all presenting widely invasive tumours. 5-year survival was 85 %. There was significant association of overall survival with wide invasion, mitotic counts, necrosis and perineural invasion.

**Conclusion:** In situ and minimally invasive Ca-ex-PA incidence in this series was higher (45 %) than previously reported and associates better prognosis irrespective of the histological subtype. Wide invasion, necrosis, perineural invasion and mitotic counts significantly impact on prognosis.

**OFP-07-007**

**Clinicopathological characteristics and survival outcomes of patients with metastatic salivary gland tumors**

C. Nagano ^*^, C. Camillo-Coutinho, R. Iagñez, C. Lopes Pinto, I. Fonseca, S. Lourenço

^*^University of São Paulo, Stomatology (Dental School), Brazil

**Objective:** Malignant salivary glands neoplasms are rare and present a remarkable clinical, histological and biological diversity. Distant metastases are infrequent, mainly associated with high-grade neoplasms. We investigated metastatic salivary gland tumors in a Brazilian Institution, in order to identify metastatic risk factors.

**Method:** Medical records of all patients diagnosed with distant metastatic salivary gland tumors seen at Cancer Hospital A.C. Camargo, Sao Paulo–Brazil, from 1970 to 2012 were reviewed. Demographic, clinical, pathologic and treatment data were recorded.

**Results:** 37 patients were identified. 14 (37,8 %) were male and 23 (62 %) were female; median age was 48 years old. Lungs were the most common metastatic site (31 cases, 83,7 %). Adenoid Cystic Carcinoma was the most frequent histological type (24 cases, 64,8 %). Most primary tumors involved the parotid (18 cases, 48,6 %). 91,8 % (34 cases) of the patients underwent combined therapy (surgical resection, radiotherapy and/or chemotherapy) and 8,1 % (3 cases) were treated with non-surgical therapy (chemotherapy/radiotherapy). Factors significantly associated with poor survival were advanced stage, distant metastases, vascular invasion, and unresectable disease.

**Conclusion:** The histological type, tumor site and isolated treatment modalities are important factors associated with metastases and survival rate in patients with malignant salivary gland tumours.

**OFP-07-008**

**Stem-cell markers in head neck squamous cell carcinoma and association with clinicopathological characteristics**

C. Coutinho-Camillo ^*^, F. d. Moraes, S. Lourenço, R. Ianez, E. d. Sousa, M. Silva, L. Kowalski, F. Soares

^*^Hospital AC Camargo, Dept. of Pathology, São Paulo, Brazil

**Objective:** To investigate the presence of stem cell markers in head and neck squamous cell carcinoma (HNSCC).

**Method:** The immuno-expression of integrin-β1, CD24, CD44, ALDH1 and CD133 proteins was analyzed in 52 HNSCC patients. The results were semi-quantitatively analyzed considering the patterns of distribution and intensity of staining.

**Results:** Seven out of 52 cases of HNSCC (13.5 %) showed positive cytoplasmic staining of ALDH1; integrin-β1 was expressed in 45 out of 50 cases (90.0 %); 30 out of 52 cases (57.7 %) showed positive membranous staining of CD44; CD24 was expressed in 44 out of 50 cases (88.0 %) and 3 out of 52 cases (5.8 %) showed positive membranous staining of CD133. Expression of CD24 and CD44 was associated with well-differentiated tumours (*p* = 0.04 and *p* = 0.04, respectively). Expression of integrin-β1 was associated with advanced stage tumours (*p* = 0.04) and expression of ALDH1 was associated with vascular invasion (*p* = 0.04). Five-year cancer-specific survival rates differed significantly between patients with negative and positive expression of CD44 (*p* = 0.052).

**Conclusion:** Our study provides the evidence of the expression of putative stem-cell markers in head and neck squamous cell carcinoma and association of these markers with patient’s outcome.

**OFP-07-009**

**Activin A immunoexpression is useful to predict occult lymph node metastasis and overall survival in oral tongue squamous cell carcinoma**

R. Coletta ^*^, P. Rodrigues, N. Kelner, A. Bufalino, F. Fonseca, A. Santos-Silva, L. P. Kowalski, T. Salo, E. Graner

^*^UNICAMP, School of Dentistry, Dept. of Oral Diagnosis, Piracicaba, Brazil

**Objective:** Regional lymph node metastasis is the most impacting prognostic factor in patients with oral tongue squamous cell carcinoma (OTSCC). Approximately 30–50 % of OTSCC patients have regional metastasis at diagnosis, but the limited sensibility of the current diagnostic methods used for neck staging does not allow detection of all cases, leaving a significant number of undiagnosed metastasis (occult lymph node metastasis). Here we evaluated whether clinicopathological features and immunohistochemical detection of carcinoma-associated fibroblasts and activin A could be predictive markers for occult lymph node metastasis in OTSCC.

**Method:** One hundred and ten patients with primary TSCC, who were classified with early stage tumor and received surgical treatment with elective neck dissection, were enrolled in the study.

**Results:** Among all examined features, only activin A high expression was significantly associated with presence of occult lymph node metastasis (*p* = 0.006). Multivariate survival analysis showed that the activin A expression was an independent marker of reduced overall survival with a 5-year survival of 89.7 % for patients with low expression compared with 76.5 % for those with high expression (HR:2.44, 95 %CI:1.55–3.85, *p* = 0.012).

**Conclusion:** Our results demonstrate that activin A immunodetection can be useful for prognostication of OTSCC, revealing patients with occult lymph node metastasis and lower overall survival rates.

**OFP-07-010**

**Odontogenic ghost cell tumours: A single center review**

F. Menezes ^*^, A. Polonia, D. Felizardo, M. Jacome

^*^Portuguese Institute of Oncology, Dept. of Pathology, Porto, Portugal

**Objective:** Odontogenic ghost cell tumours (OGCT) are rare lesions. Derived from epithelial and ectomesenchymal elements of the tooth-forming organ, OGCT are neoplastic proliferations of ameloblastomatous epithelium, variable amounts of dysplastic dentin and the key diagnostic ghost cells, thought to be a product of keratinization or coagulation necrosis of epithelial cells. Classically grouped as Calcifying odontogenic cysts, posterior revisions led to the 2005 WHO classification, splitting Calcifying cystic odontogenic tumours (CCOT), solid Dentinogenic ghost cell tumours (DGCT), and extremely rare Ghost cell odontogenic carcinomas. OGCT classification remains controversial though: further group subdivisions have been proposed, but no significant behavior differences within each group’s histopathologic spectrum have been found. Association with other odontogenic lesions is common; if ghost cells are overlooked, confusion with ameloblastoma is most likely. Our aim is to review OGCT aspects, and potential diagnostic pitfalls.

**Method:** Our institute database was browsed for all patients diagnosed with OGCT. Histological and immunohistochemistry aspects, patient follow-up and recent literature were reviewed.

**Results:** 4 cases (3 CCOT, 1DGCT) have been diagnosed by our department so far. The DGCT case was first misdiagnosed as recurrent ameloblastoma.

**Conclusion:** The aspects reviewed are consistent with recent literature. OGCT should be a differential diagnosis not to overlook when facing odontogenic tumours.

Monday, 2 September 2013, 17.00–19.00, Auditorium VIII

**OFP-08 Oral Free Paper Session Joint Session–Neuropathology/Ophthalmic Pathology**

**OFP-08-003**

**Histological features and neo-angiogenesis in embolized meningiomas: Is there the risk of overgrading?**

V. Barresi ^*^, G. Branca, F. Granata, M. Caffo, C. Alafaci, G. Tuccari

^*^Policlinico G. Martino Pad D, Dip. Patologia Umana, Messina, Italy

**Objective:** Pre-operative embolization (POE) induces histological changes simulating malignancy in meningiomas. Our aims were to test for overgrading the scheme currently in use and to analyze whether the POE procedure may stimulate neo-angiogenesis in embolized meningiomas.

**Method:** The histological features of 41 embolized meningiomas were reviewed, compared with those observed in atypical non-embolized tumors, and considered for grading assessment. In the same cases, neo-angiogenesis was also quantified by the evaluation of microvessel density (MVD).

**Results:** In embolized meningiomas, necrosis and macronucleoli represented frequent findings. Nonetheless, differently from that observed in non-embolized meningiomas, necrosis mostly showed an abrupt line of demarcation from the viable tumour tissue, and macronucleoli were mainly restricted to peri-necrotic areas. Exclusion of these features resulted in increased specificity and positive predictive value of histological grading in the identification of recurring meningiomas. MVD showed a significant increase with the interval between POE and surgery and it was not correlated to malignancy in our cases.

**Conclusion:** The frequent evidence of necrosis and prominent nucleoli in embolized meningiomas may lead to overgrading, which may be avoided by excluding necrosis showing an abrupt line of demarcation and focal peri-necrotic macronucleoli in grading assessment. Also, as neo-angiogenesis may result from POE procedure, caution should be used in the interpretation of MVD as a prognostic factor in embolized meningioma.

**OFP-08-004**

**Solitary fibrous tumour of the meninges: A case series**

M. Cruz ^*^, M. Ferreira, M. Teixeira, F. Pardal, A. Silva

^*^Hospital de Braga, Serviço de Anatomia Patológica, Portugal

**Objective:** Meningeal solitary fibrous tumours (MSFTs) are uncommon and malignant cases are even rarer. We report four cases of MSFTs diagnosed in our department during a 15-year period, including a locally invasive malignant case.

**Method:** The patients (3 F, 1 M) ranged from 48 to 77 years (mean 60). Tumours were tentorial in one case and supratentorial in three cases, one of these showing invasion into the brain, parietal bone and subgaleal soft tissues. All patients underwent surgery as initial treatment. Follow-up was obtained.

**Results:** Tumours consisted of spindle cells disposed in fascicles with a dense collagenous stroma. Two supratentorial cases had rare mitoses and no significant nuclear pleomorphism or necrosis. The tentorial case showed high cellularity, nuclear pleomorphism and frequent mitoses, but no necrosis. The invasive supratentorial case showed identical atypical histological features with additional necrosis. This patient received postoperative radiotherapy with no recurrence after 4 years of follow-up. All cases were diffusely positive for vimentin, CD34 and bcl-2 but negative for EMA and S100 protein.

**Conclusion:** Data on outcome for patients with MSFT are limited. This case series suggests that MSFTs can have a locally aggressive behaviour. MSFTs should, therefore, be excised as completely as possible and followed carefully in the long-term.

**OFP-08-006**

**Sensitivity and specificity of IHC markers for diagnostics of variants of Non-small Cell Lung Carcinoma (NSCLC) in Brain Metastases (BM)**

D. Rotin ^*^, O. Paklina, O. Potapova, G. Kobyakov

^*^NN Burdenko Neurosurgery Institute, Dept. of Pathology, Moscow, Russia

**Objective:** Current modalities of target therapy in advanced Non-small cell lung carcinoma require diagnostic of exact variant of the tumor. Brain metastases are not rarely the first and only material in NSLC for histological evaluation. Aim: To determine on BM of NSCLC specificity and sensitivity of number of antibodies. To work out the optimal IHC panel for BM NSCLC

**Method:** Material from 96 patients (70 -male, 26- female), median age 58 (ranges 37–76) with BM NSCLC operated in NN Burdenko neurosurgery institute in 2004–201, was reviewed by two skilled pathologists and then stained with TTF-1, 1, CK7, CK5/6, CK18, p63, Napsin A etc. Specificity and sensitivity for each antibody was calculated.

**Results:** The highest rate of sensitivity and specificity for Adenocarcinoma was seen for Napsin A (94 %/91 %), following TTF-1 (79 %/86 %). For squamous cell carcinoma the best specificity and sensitivity demonstrated p63 and CK5/6–correspondently (91 %/98 %) and (87 %/94 %).

**Conclusion:** The optimal panel for diagnostic of the variant of NSCLC in BM includes 4 antibodies: TTF-1, Napsin A, p63, CK5/6.

**Table of sencitivity and specificity:**

**Table:** 

Antibody	TTF-1	Napsin A	p63	CK5/6
Sensitivity	79 %	94 %	91 %	87 %
Specificity	86 %	91 %	98 %	94 %

**OFP-08-007**

**Sebaceous gland carcinoma of the eyelid: Prognostic significance of nuclear survivin (BIRC5)**

K. Mulay ^*^, V. White, S. Shah, S. Honavar

^*^Center for Sight, Ophthalmic Pathology, Hyderabad, India

**Objective:** To evaluate the frequency of expression of nuclear survivin in sebaceous gland carcinoma ofthe eyelid and correlate its expression with histopathological features, clinical outcomes and expression of Ki-67 & p63.

**Method:** 55 cases of periocular sebaceous gland carcinoma with a minimum follow up of 1-year were retrieved from the archives. Patient files were assessed for clinical outcomes. Immunostaining for survivin, Ki-67 and p63 was performed on all cases. All tumours were assigned a nuclear survivin score (NS-Score) based on the intensity and percentage expression. The NS-score was correlated with the clinical outcomes, histopathological features and expression of Ki67 and p63.

**Results:** All tumours expressed survivin in their nucleus. 23 tumours (51 %) had a high NS score. A high NS-score was associated with a short disease free survival (*p* < 0.0001) and high Ki-67 activity (*p* < 0.001). No statistically significant association was seen with expression of p63 and histopathological features.

**Conclusion:** Survivin is expressed in the nucleus in sebaceous gland carcinoma of the eyelid. A high expression of survivin has a prognostic influence in sebaceous gland carcinoma of the eyelid.

**OFP-08-008**

**Amyioid-ß and age-related macular degeneration**

V. Ermilov ^*^, A. Nesterova, O. Makhonina

^*^Volgograd State Medical University, Dept. of Pathology and Forensic Medicine, Russia

**Objective:** Age-related macular degeneration (AMD) is the leading cause of severe visual impairment in the elderly. The appearence of amyloid in eye tissues makes it possible to see in a new light the problem of eye amyloidosis.

**Method:** Histological, immunohistochemical, and electron microscopic studies of the 111 eyes with AMD revealed amyloid-β in the drusen, Bruch’s membrane, and between the basal membrane of the retinal pigment epithelium (RPE) and the internal collagen layer of Bruch’s membrane.

**Results:** Comparative analysis of morphological changes in tissues of the macular and paramacular areas and the incidence of amyloid-β incorporations in them permit us to propose that accumulation of local senile amyloid is conductive to development and aggravation of AMD. A relationship between the degree of RPE degeneration and accumulation of amyloid-β was revealed. Ultrastructural studies of Bruch’s membrane have shown that amyloid fibrills are localised in it with fragments of degrading RPE cells closely attached to them. Amyloid-β deposition was specific to drusen from eyes with AMD.

**Conclusion:** The authors put forward a hypothesis of the pathogenesis of AMD, in which the principal role in the formation and deposition of abnormal protein–amyloid-β, is played by degenerative cells of RPE.

**OFP-08-009**

**The prognostic value of CXCR4-CXCL12 and CCR7 in uveal melanoma in patients with and without liver metastasis**

R. Verdijk ^*^, T. v. den Bosch, A. Koopmans, J. Vaarwater, A. d. Klein

^*^Erasmus MC University, Dept. of Pathology, Rotterdam, The Netherlands

**Objective:** To examine expression of the chemokine receptors CXCR4, CXCL12 and CCR7 in uveal melanoma with correlation to patient survival. Irrespective of primary treatment, nearly half of the patients develop liver metastases. Chemokine receptors are involved with different cell processes, including angiogenesis and trafficking of tumor cells in metastasis.

**Method:** Formalin-fixed paraffin-embedded primary uveal melanoma specimens were collected from non-metastatic (*n* = 30) and metastatic (*n* = 19) patients between years 1988–2008. Specimens have been examined for classical histopathological parameters and CXCR4, CXCL12 and CCR7 expression using immunohistochemistry. Single nucleotide polymorphism array were performed and chromosome fluorescence in situ hybridization of chromosomes 1,3,6, 8.

**Results:** CCR7 expression correlates with the epitheloid cell type (*p* = 0.006), tumor thickness (*p* = 0.000) and necrosis (*p* = 0.048). No copy number variations were found in the SNP array in the regions of the CXCR4 and CCR7 genes. In multivariate analysis CCR7 staining is inversely correlated to overall survival, and disease free survival whereas CXCR4 staining is not. SNP analysis showed no gains or losses in the regions of CCR7, CXCR4 or CXCL12.

**Conclusion:** CCR7 expression is correlated to adverse prognostic histopathological factors and is inversely correlated to overall survival, and disease free survival. CCR7 may be a potential target for therapeutic intervention.

**Overall survival for CCR7 expression:** 
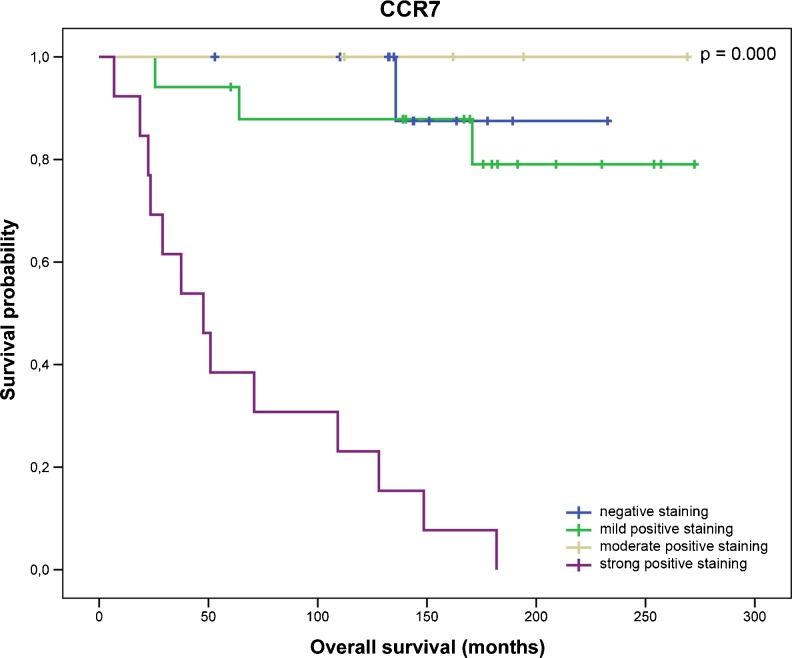


**OFP-08-010**

**Immune infiltration in choroidal melanomas**

D. Narasimhaiah ^*^, R. Remark, E. Becht, D. Damotte, C. Legrand, P. d. Potter, P. Coulie, M. Vikkula, C. Godfraind

^*^Dept. of Pathology, Université Catholique de Louvain, Brussels, Belgium

**Objective:** In choroidal melanomas, the presence of CD8+ T lymphocytes is associated with bad prognosis. This is in contrast to most other solid tumors, where CD8+ T cell infiltrate is related to good prognosis. The objective of this study was to better understand the immune microenvironment in choroidal melanomas.

**Method:** Primary, untreated tumors were analyzed for gene expression using Affymetrix U133 plus 2.0 array (*n* = 15) and immunohistochemistry (*n* = 89).

**Results:** Gene expression profile analysis led to the identification of a gene signature consisting of 39 upregulated genes in a subset of choroidal melanomas. These genes were associated with: antigen processing and presentation, cell adhesion molecules, interferon-gamma and chemokine signaling pathways. On immunohistochemistry, signature positive tumors displayed a dense intra-tumoral infiltrate of HLA-DRA+CD163+ macrophages and CD3+CD8+ T-cells. It was mild to moderate in tumors lacking the signature. On this basis, additional tumors were analyzed by immunohistochemistry. In total, 19 melanomas had high immune infiltrate and 70 low. Kaplan-Meier plots demonstrated tumors with high immune infiltrate had shorter disease-free survival (log-rank *P* < 0.001).

**Conclusion:** We identified in choroidal melanomas an interferon-gamma induced gene signature associated with infiltration of macrophages and CD8+ T lymphocytes. The presence of tumor-infiltrating immune cells correlated with higher risk of occurrence of metastases.

Monday, 2 September 2013, 17.00–19.00, Room 5A

**OFP-09 Oral Free Paper Session Paediatric and Perinatal Pathology**

**OFP-09-001**

**Increase of placental sensitivity to melatonin and the alteration to its local synthesis in hypertensive syndromes in pregnancy**

R. Corrêa ^*^, D. d. Resende Yamamoto, L. d. Resende Yamamoto, L. Penna Rocha, M. Antônia dos Reis

^*^Triângulo Mineiro Federal Universidade, ICBN, Uberaba, Brazil

**Objective:** To evaluate the relation between hypertensive syndromes and melatonin and its possible protective role against lesions due to hypertension.

**Method:** Placentas were classified into Gestational hypertension (GH), Chronic hypertension (CH), Pre-eclampsia (PE) and Pre-eclampsia superimposed on chronic hypertension (PSCH), and morphologically examined by Hematoxylin–Eosin and Periodic Acid Schiff methods. Immunohistochemistry was performed in order to detect tryptophan hydroxylase (TH) and melatonin receptor 1A (MR-1A).

**Results:** MR-1A expression was higher in all types of HSP, mainly in cases with GH, in Caesarean section delivery, preterm placentas and in the cases with alterations in the placental morphology, particularly those presenting inflammation. The expression of TH was higher in cases with CH when compared with the control. This expression was lower in primigestas, in cases of inflammation and with PE.

**Conclusion:** HSP therapies should be considered and studied, especially in cases of HSP associated with PE, in which the placenta is more sensitive as it has more receptors, but its synthesis ability is reduced. As for GH and CH, the possible benefits should be evaluated, since the local placental ability to produce melatonin still exists. Financial Support: CNPq, CAPES, FAPEMIG, FUNEPU.

**OFP-09-002**

**Neuronal apoptosis in the neonates born to preeclamptic mothers**

E. Ozer ^*^, H. Cosar, H. Topel, E. Özer

^*^Dokuz Eylul University, Dept. of Pathology, Izmir, Turkey

**Objective:** The aim of this study is to investigate the biological significance of neuronal apoptosis in an experimental model of preeclampsia.

**Method:** Two of four pregnant Sprague–Dawley rats (preeclamptic group) were given water containing 1.8 % NaCl on gestation day 15 and 22 in order to establish the model of preeclampsia whereas other two (non-preeclamptic group) received normal diet. A model of perinatal asphyxia was established on the postnatal 7th day to one preeclamptic and one non-preeclamptic dam. Overall 23 pups born to overall four dams were decapitated to assess neuronal apoptosis by the TUNEL assay.

**Results:** Mean body and brain weights were significantly lower in the pups born to the preeclamptic dams (*p* < 0,001). The number of apoptotic neuronal cells was significantly higher in the preeclampsia groups in comparison with the control group (*p* = 0.006 and *p* = 0.006, respectively). It was also significantly higher in the asphyctic/non-preeclamptic group than the count in the control group (*p* = 0.01). There was also significant difference between both asphyctic groups (*p* = 0.003).

**Conclusion:** We conclude that preeclampsia causes small babies for the gestational age and cerebral hypoplasia. Both preeclampsia and perinatal asphyxia can cause increased neuronal apoptosis in the neonatal brains. However the prognosis for neurological outcome is much worse when the perinatal asphyxia occurs in newborns born to preeclamptic mothers.

**Neuronal apoptotic cells labeled by the TUNEL assay.:** 
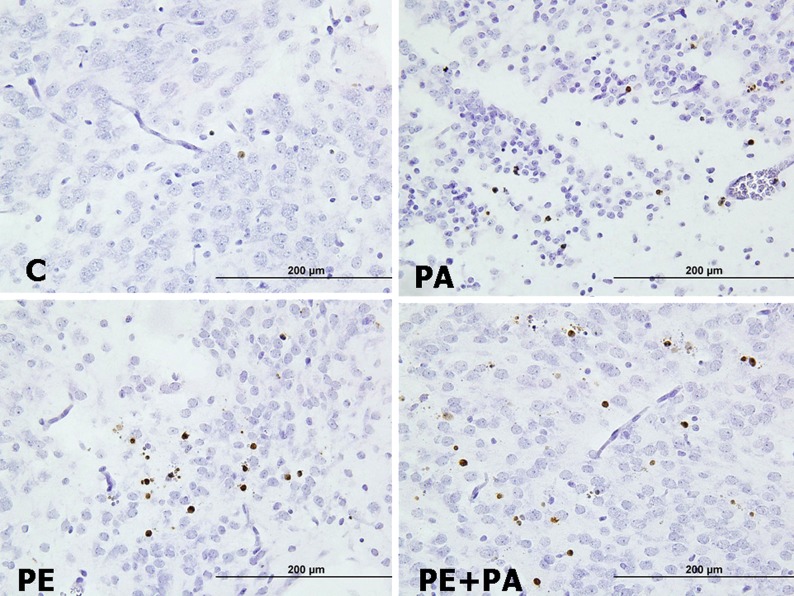


**OFP-09-003**

**The role of placental NOTCH signaling pathway disturbances in preeclampsia**

K. Pavlov ^*^, E. Dubova, A. Shchegolev, G. Sukhikh

^*^Research Center for Obstetrics, Dept. of Pathology, Moscow, Russia

**Objective:** Notch signaling pathway is necessary for placental development and plays a critical role in placentation, trophoblast invasion and placental angiogenesis. Our aim was to evaluate patterns of Notch receptor placental expression in preeclampsia (PE) complicated pregnancies.

**Method:** We performed complex morphological and immunohistochemical study of 9 term placentas from mild preeclampsia (mPE) cases (1st group), 6 term placentas from severe preeclampsia (sPE) cases (2nd group) and 10 term placentas from uncomplicated pregnancies (control group). Immunohistochemical study was performed with rabbit polyclonal antihuman Notch-1 antibodies (Spring Bioscience).

**Results:** We revealed decreasing of Notch-1 expression in all placental compartments in mPE and sPE in compare to control. Marked decreasing was noted in syncytiotrophoblast (mPE–38.6 %, sPE–39 % lower) and extravillous trophoblast (mPE–33.3 %, sPE–34 % lower) (*p* < 0.05). Notch-1 terminal villi endothelial expression was also decreased (both mPE and sPE–26 % lower) (*p* < 0.05). We also noted insignificantly decreased villous mesenchimal cell Notch-1 expression (*p* < 0.05).

**Conclusion:** Revealed patterns of Notch-1 expression reflect the role of this receptor in PE development.

**OFP-09-004**

**ACE mutation in the recurrent oligohydramniosis and neonatal death**

J. Laine ^*^

^*^TYKS-SAPA, Dept. of Pathology, Turku, Finland

**Objective:** Case: The girl was born at 32 weeks of gestation, with oligohydramniosis and severe lung hypoplasia. The ultrasound showed difference in the size of the kidneys. Despite intensive care the baby died at 7 h, with respiratory insufficiency and anuria. Previously the mother had had three deliveries at 32–33 weeks of gestation and all babies had died within 3 days, with similar symptoms as in the present case.

**Method:** Autopsy, immunohistochemistry and genetical testing were performed.

**Results:** The autopsy showed small lungs and urinary bladder. The fontanelles were large. In histology the kidney showed renal tubular dysplasia typical of that found in ACE mutation. The glomeruli were crowded and the cortical tubules were sparse, with narrow lumen and without brush border. In the immunohistochemistry the cortical tubuli were negative for CD10 and CD15 and positive for epithelial membrane antigen (EMA). Clusters of hemangiomatoid structures and thick-walled arteries were found in the cortex. The lungs showed findings in keeping with respiratory distress syndrome.

**Conclusion:** The findings are well in keeping with renal tubular dysgenesis that is a rare disease and due to ACE gene mutation (Lacoste ym. J Am Soc Nephrol 17: 2,253–2,263, 2006). The disease is autosomally recessively inherited and in this case has been unexpectedly manifested four consecutive pregnancies. The condition was confirmed by gene analysis.

**OFP-09-005**

**Placental pathology supports stratification of third trimester births into gestational age subgroups**

J. Stanek ^*^

^*^Cincinnati Children’s Hospital, Dept. of Pathology, USA

**Objective:** To find if placental pathology supports the new clinical concept of dividing preterm and term births into gestational age subgroups associated with varying perinatal mortality and morbidity.

**Method:** 27 clinical and 43 placental phenotypes were retrospectively analyzed in 4,617 third trimester pregnancies: 1,332 preterm pregnancies (28–33 weeks), 1,066 late preterm pregnancies (34–36 weeks), 940 near term pregnancies (37–38 weeks), and 1,279 term pregnancies (39+ weeks).

**Results:** The Figure depicts statistically significant clinical and placental differences between the preterm and late preterm, and near term and term births. The placental acute inflammatory pattern was characteristic for both gestational age sides of the 3rd trimester, while clinical conditions linked to in-utero hypoxia (preeclampsia, diabetes mellitus, fetal growth restriction) and their placental counterparts (atherosis, membrane chorionic microcysts, chorangiosis, intervillous thrombi) were associated with mid-3rd trimester. Frequency of acute fetal distress (abnormal fetal heart tracing, and clinical and histological meconium) increased with gestational age until term.

**Conclusion:** Differences in placental pathology among the four subgroups of third trimester pregnancy not only challenge the use of an arbitrary cutoff point of 37 weeks separating the preterm and term birth, but also further support separation of late preterm births from preterm births, and near term births from term births.

**Statistically significant differences in clinical and placental variables:** 
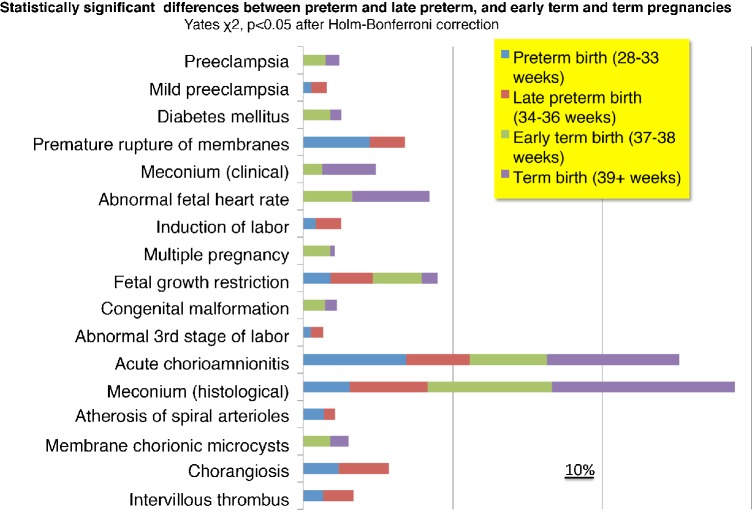


**OFP-09-006**

**Up-regulated TNFa placental terminal villi expression as a predictor of early-onset neonatal sepsis**

A. Shchegolev ^*^, E. Dubova, K. Pavlov, E. Balashova, O. Ionov, D. Degtiarev, G. Sukhikh

^*^Research Center for Obstetrics, Dept. of Pathology, Moscow, Russia

**Objective:** Early-onset neonatal sepsis (EONS) is one of the most dangerous neonatal pathology with extremely high mortality. Our aim was to study correlation between abnormal placental TNFα expression and EONS development.

**Method:** 28 newborns with EONS and 15 healthy newborns were included in a study. White blood cells (WBC) and platelets (PL) count, serum C-reactive protein (CRP) and procalcitonin (PCT) level and complex morphological study of the placenta with immunohistochemical detection of TNFα were performed.

**Results:** WBC count, CRP and PCT serum levels were significantly elevated in neonates with EONS, whereas PL count was significantly decreased. In 10 EONS cases we revealed villous tree maldevelopment (excessive syncytial knotting) and persistent villous immaturity. Signs of placental inflammation were revealed in 11 cases of EONS: acute chorioamnionitis (6 cases), acute funisitis (3 cases), acute basal plate deciduitis (3 cases). We also revealed significant elevation of TNFα expression in syncytiotrophoblast and terminal villi endothelial cells (41.1 % and 45.3 % in compare to control, accordingly).

**Conclusion:** Up-regulated TNFα placental terminal villi expression could be used as a prognostic marker of early-onset neonatal sepsis development.

**OFP-09-007**

**Fetal cardiovascular malformations: Clinical versus anatomopathological diagnosis**

P. Serra ^*^, A. Rodrigues, R. Pina

^*^CHUC- HG, Surgical Pathology, São Martinho Do Bispo, Portugal

**Objective:** Diagnostic correlation between fetal prenatal diagnosis (PD) results and autopsy findings on Cardiovascular malformations (CVM).

**Method:** We have analysed 772 fetal autopsies performed between January 2005 and December 2012. Only cases above 19 weeks gestation and whose CVM was not associated to cromossomopathies where selected, because these had PD with morphologic ultrasound and echocardiogram. We analysed the clinical records and autopsy diagnosis.

**Results:** There were 190 cases with CVM (24,6 %): 134 cases where above 19 weeks gestation but only 73 cases had a CVM not associated to a cromossomopathy. Our study was performed on the last group. The greatest results disparity was found on Ventricular septal defects (VSD) (7/14 cases), aortic arch coarctation (2/6), 2 cases of complete situs inversus, 1 Ebstein anomaly and 1 atrial septal defect. On complex cardiopaties, 8 cases showed minor disparity results. There was complete agreement on the hypoplastic left heart syndrome diagnosis (12 cases). On 1 case the CVM diagnosed on the echocardiogram was not found at the autopsy.

**Conclusion:** The CVM are a common indication for medical pregnancy termination and therefore they are common fetal autopsy findings. Only 17,8 % (13 cases) of CVM showed diagnostic disparities, mostly on VSD, because it were very small defects.

**OFP-09-008**

**Innate immune response and expression of melatonin receptors in the lung of children**

R. Corrêa ^*^, J. G. Pacheco Olegário, M. Vinícius da Silva, J. Reis Machado, L. Penna Rocha, M. Antônia dos Reis

^*^Triângulo Mineiro Federal Universidade, ICBN, Uberaba, Brazil

**Objective:** To analyze the cytokines of the innate immune pulmonary response and the capacity for local response to melatonin according to the perinatal stress.

**Method:** 49 cases of pediatric autopsies were evaluated, divided according to cause of death, perinatal stress, gestational age (GA), and birth weight. The percentages of IL-6, C-reactive protein (CRP), IL-1β, TNF-α, and melatonin receptor were evaluated by immunohistochemistry.

**Results:** The IL-6 expression was higher in the children showing chronic stress (CS), anoxia, and infection. The IL-6 expression showed a progressive increase according to the relation between weight and GA. There was no significant difference in the expression of IL-1β and TNF-α. The CRP expression was higher in the cases showing CS and premature cases. The expression of melatonin receptors was significantly higher in the cases showing CS, being more evident in the cases showing infection.

**Conclusion:** The cause of death and the type of stress influence the expression in situ of melatonin and cytokines of the innate immune pulmonary response. The greater sensitivity of the lung to melatonin in these cases may indicate an attempt at controlling the immunological response, in an attempt to diminish the harmful effects of stress. Financial Support: CNPq,CAPES, FAPEMIG, FUNEPU.

**OFP-09-009**

**Pediatric hepatocellular carcinoma of common type: Morphologic and immunophenotypic characterization of a monocentric series of 13 cases**

C. Mussini^*^, E. Gonzales, M.-J. Redon, S. Branchereau, H. Martelli, E. Jacquemin, C. Guettier

^*^Hopital Bicetre, Pathology, Le Kremlin Bicetre, France

**Objective:** Hepatocellular carcinoma (HCC) represents 0,4 % of pediatric malignant tumors. Anti-HBV vaccination modified epidemiology of common type HCC with an increasing incidence of constitutional liver diseases. To study epidemiologic, morphologic and immunophenotypic profiles of pediatric HCC of common type.

**Method:** Between 2006 and 2013, 13 children had surgery for common type HCC. Clinical and follow-up data have been collected. Immunochemical study has been performed on 18 tumor nodules with EpCAM, CK19, AFP, b-catenin and glutamin synthase antibodies.

**Results:** Median age at diagnosis was 8,5 years (range: 1–15,25). Average AFP level was 105,313 (range: 15–909,000) HCC occured in 2 normal livers, 1 HBV cirrhosis and 10 genetic liver diseases (5 Progressive Familial Intrahepatic Cholestasis-type 2, 3 tyrosinemia type 1, 2 mitochondrial cytopathies). WHO grade was more often 2 or 3. Eighty-nine per cent (16/18) of HCC had a progenitor-like profile EpCAM+, AFP+including CK19+ (5/18) and CK19- (11/18). Nuclear expression of b-catenin was observed in 66,7 % (12/18) HCC, mainly at the periphery of the tumor. Diffuse expression of glutamin synthase was present in only 3/18 HCC.

**Conclusion:** Most pediatric HCC occurs on genetic liver diseases and their phenotype suggests an origine from hepatic progenitor cells.

**OFP-09-010**

**Salivary gland tumours in children: A decade of experience and a review of current literature**

A. Joannou-Coetzee ^*^, A. Cohen, S. Whitaker, S. Di Palma

^*^Sheffield Children’s Hospital, Dept. of Histopathology, United Kingdom

**Objective:** Salivary gland tumours are rare in children. We describe our experience with these tumours from our institutions and review current literature.

**Method:** A search of the files from the Histopathology department at a paediatric referral hospital and from cases referred to one of the co-authors for histological review was performed between 2003 and 2013. The findings were compared to hitherto published evidence regarding diagnosis, prevalence and biology.

**Results:** We identified 5 pleomorphic adenomas; 3 mucoepidermoid carcinomas; 1 acinic cell carcinoma, 1 desmoid fibromatosis and 1 Burkitt’s lymphoma. In addition, cystic lesions that corresponded to a cystic hygroma (1 case) and a lymphatic malformation (2 cases) were also seen. Only 5 of these cases corresponded to a malignant neoplasia. In our experience, tumours arising in salivary glands of young patients have a high chance of malignancy (55.5 %).

**Conclusion:** Our results confirm the reviewed literature, that indicates that the incidence of malignant tumours in the salivary glands is much higher in children (55.5 %) than in adults (15 %–25 %). This may be the consequence of various genetic alterations in paediatric tumours. The rarity of salivary gland tumours at this age highlights the importance of the appropriate clinical and surgical management of these cases at the initial presentation.

Tuesday, 3 September 2013, 14.15–16.15, Auditorium VII

**OFP-10 Oral Free Paper Session Gynaecopathology**

**OFP-10-001**

**The role of All Trans Retinoic Acid (ATRA) in ovarian cancer therapy**

R. Januchowski ^*^, K. Wojtowicz, M. Zabel

^*^University of Medical Sciences, Dept. of Histology and Embriology, Poznan, Poland

**Objective:** Ovarian cancer represents the most common cause of death among gynecological malignancies. Chemotherapy in this cancers is still not satisfactory because of drug resistance. The main mechanism of drug resistance results from the ability of cancer cells to actively expel therapeutic agents via transport proteins of the ABC family. Recent investigations indicate that aldehyde dehydrogenase (ALDH1A1) can also be involved in drug resistance of ovarian cancer.

**Method:** A chemosensitivity assay MTT test was performed to assess drug cross-resistance. Quantitative real-time polymerase chain reaction and Western blot were performed to determine mRNA and protein expression of genes involved in chemoresistance.

**Results:** We observed overexpression of ALDH1A1 in primary ovarian cancer cell lines resistant do topotecan and paclitaxel at mRNA and protein level. Pre-treatment with all-trans retinoid acid (ATRA) sensitised these cell lines to topotecan and paclitaxel. Treatment with ATRA leads to downregulation of ALDH1A1, ABCB1 and ABCG2 at protein but not mRNA level.

**Conclusion:** Our results indicate that ATRA regulate expression of ALDH1A1 ABCB1 and ABCG2 at post-translational level. Downregulation of these proteins is very important from therapeutic point of view. ATRA could be considered as a therapeutic agent in combination with cytostatics in ovarian cancer therapy.

**OFP-10-002**

**Malignant ovarian epithelial tumors and coexistence with synchronous uterine carcinoma: A study of clinicopathological parameters and Her 2/neu protein expression**

M. El-Shaer ^*^, Z. S. Elalfy, Z. A. Shehab Eldin, F. Faten A. Ghazal, E. A. Omara

^*^National Research Centre Cairo, Dept. of Pathology, Egypt

**Objective:** To evaluate the oncogene Her2/neu in these ovarian tumors and its relation with synchronous uterine corpus carcinoma (SUC) and their association with different clinicopathologic parameters.

**Method:** Randomly selected retrospective 136 cases examined for the presence or absence of SUC,and all data were extracted

**Results:** Coexisting ovarian and SUC was found 11 % of malignant cases, 73.3 % of them were endometrioid ovarian adenocarcinoma with evident high percentage, 20 % of serous ovarian adenocarcinoma while only 6.7 % mucinous. The grades were found to be 13.3 % grade I,53.3 % grade II and 33.3 % grade III i.e. 86.6 % were moderately to poorly differentiated carcinomas. Staging was 41.7 % stage I, 0 % stage II, 50 % stage III & 8.3 % stage IV. About 27 % of ovarian tumors presented with SUC were stained positive for Her 2/neu, in comparison to only 6.3 % of isolated ovarian tumors without coexisting uterine primary tumor *P* value = 0.007.

**Conclusion:** The incidence of synchronous endometrial and ovarian cancer is not negligible, is commonly seen with malignant endometrioid ovarian tumors. Percentage of recurrence increased in the cases of ovarian tumor coexisting with SUC than in isolated ovarian tumor, ovarian tumors coexisting with (SUC) show a significant increase in Her2 over expression predicting an underlying molecular genetic defect causing this association.

**OFP-10-003**

**Ovarian metastasis of endocervical adenocarcinoma**

P. Caseiro Silverio ^*^, H. Kannuna, D. Huber, J.-C. Tille

^*^Hôpital Universitaire Genève, Switzerland

**Objective:** Ovarian metastasis of endocervical adenocarcinoma is uncommon and can mimic primary ovarian neoplasm.

**Results:** A 51-year-old woman was hospitalized for thromboembolic disease progression despite anticoagulation. Computer tomography showed a suspicious pelvic tumor and an elevated CA 125. Exploratory laparoscopy confirmed a 12 cm left adnexal mass with moderate ascite and no other intra-abdominal lesions. Peritoneal biopsies were negative and peritoneal cytology revealed an adenocarcinoma. A primary cytoreduction surgery was performed (hysterectomy with bilateral oophorectomy, appendectomy, omentectomy). The ovarian tumor showed an endometrioid-like pattern, with elongated nucleus associated with numerous mitosis, apoptoses, and focally mucinous differentiation. No macroscopic lesion was visible on the cervix, but a CIN3 and endocervical adenocarcinoma was present, with the same morphology of the ovarian tumor. No lympho-vascular invasion was observed. At immunohistochemistry, both tumors were positive for p16, CEA and in 50 % for estrogen receptors. The PCR for HPV shows the presence of type 16 HPV.

**Conclusion:** The reported frequency of ovarian metastases in cervical adenocarcinoma is ~5 %. In absence of macroscopic cervical lesion, the ovarian metastasis could be misdiagnosed as primary ovarian tumor. The hybrid pattern and detection of HPV is useful for identification an endocervical origin. A possible way of dissemination is perhaps through transtubal spread.

**OFP-10-006**

**Undifferentiated Connective Tissue Dysplasia Syndrome (uCTDS) and Genetically Determined Trombophyliae (GDT) influence on endometrium receptivity and reproduction function**

T. Demura ^*^, E. Kogan, D. Kolossovskiy, N. Fayzullina, A. Zanozin

^*^RCOGP, Dept. of Pathology, Moscow, Russia

**Objective:** Aim of investigation is a study of uCTDS and GDT influence on endometrium receptivity and pregnancy outcomes.

**Method:** Clinico-morphological analysis of 92 patients with diagnosis GDT was carried out. 54 (58.7 %) of these patients had GDT and uCTDS combination. Clinical study included analysis the following data: anamnesis, physical examination. Endometrium pipel-biopsies taken at 6-7th day after ovulation underwent a morphological investigation. Stepped paraffin sections were prepared. These sections stained by a hematoxiline & eosin and used for immunohistochemical determination the following markers: ER (DAKO, Denmark), PgR (DAKO, Denmark), PAI (Santa Cruz, USA), LIF (R&D Systems, USA), VEGF (DAKO, Denmark).

**Results:** In 83.1 % of all cases there was endometrium of either proliferation or early secretion phase in “implantation window”. In 84.3 % of all cases no mature pinopodies were found. Sclerosis and connective tissue disorganization occurred in 68.1 % cases with uCTDS and in 52.7 % cases without uCTDS; microcirculatory disturbances–in 48.9 % and in 38.9 % cases; mature pinopodies absence–in 87.2 % and in 80.6 % cases, abnormal ER/PgR ratio–in 80.9 % and in 69.4 % cases, consequently.

**Conclusion:** GDT and uCTDS combination can be considered as reproduction function disturbances negative prognosis factor.

**OFP-10-007**

**Identifying prognostically relevant subsets of endometrial cancer using unsupervised clustering of immunohistochemical staining data**

J. Huvila ^*^, D. Laajala, P.-H. Edqvist, L. Talve, S. Grénman, F. Pontén, T. Aittokallio, O. Carpén, A. Auranen

^*^University of Turku, Dept. of Pathology, Finland

**Objective:** Multiple studies have suggested that biomarkers, such as ER and PR have a prognostic significance in EC. However, these biomarkers are currently not utilized as prognostic markers in clinical practice. Our aim was to assess the immunohistochemical risk profile of EC patients by performing unsupervised clustering of eight prognostic factors.

**Method:** We collected clinical, histopathological and follow-up data on 230 EC patients operated at Turku University Hospital during 2004–2007. A TMA was constructed for immunohistochemical analysis and the results were entered in an unsupervised hierarchical clustering analysis.

**Results:** The unsupervised clustering produced a cluster with three major subgroups of which one subgroup consisting of 47 (20.4 %) cases had a higher risk for relapse [OR 14.3 (CI 5.91–34.7, *p* < 0.001)]. Apart from one clear-cell cancer, all non-endometrioid cancers clustered in the high-risk subgroup; furthermore, 36 (16.5 %) of the adenocarcinomas clustered in the high-risk subgroup and had a higher relapse risk compared to the endometrioid cancers in the non high-risk subgroups [OR 12.2 (CI 4.72–31.6, *p* < 0.001)]. The association sustained when adjusted for FIGO stage and grade [OR 8.3 (CI 2.88–23.95, *p* < 0.001)].

**Conclusion:** Our results indicate that using a panel of immunohistochemical stainings could be a useful tool for risk assessment in EC.

**OFP-10-008**

**eIF4E, a critical downstream target of the mTOR pathway, is overexpressed in high-grade dysplasia and carcinomas of the uterine cervix**

A. Asimomytis ^*^, M. Karanikou, A. Rodolakis, A. Vaiopoulou, P. Tsetsa, A. Antsaklis, E. Patsouris, G. Rassidakis

^*^University of Athens, First Dept. of Pathology, Greece

**Objective:** eIF4E, a critical regulator of the protein synthesis initiation, seems to recapitulate the oncogenic effects of the AKT/mTOR pathway in mouse models. In this study, we investigated the expression levels of eIF4E in precancerous lesions and cancer of the uterine cervix and their association with HPV infection.

**Method:** Uterine cervical biopsies from 73 patients were obtained including 40 fresh-frozen samples and 42 archival paraffin-embedded tissue specimens. eIF4E expression was analyzed by Western blot in whole protein extracts and by immunohistochemistry in archival tissues. HPV typing was performed using standard PCR methods.

**Results:** High expression levels of eIF4E was observed in all carcinomas and significantly correlated with the degree of dysplasia (*p* < 0.0005) in immunoblots. By immunohistochemistry, overexpression of eIF4E was seen in 17 of 21 (81 %) patients with high-grade dysplasia and carcinomas as compared to 2 of 20 (10 %) patients with low-grade lesions or normal histology (*p* < 0.0001). eIF4E expression did not statistically correlate with the presence of high-risk HPV or oncogenic HPV 16/18 types.

**Conclusion:** Our findings suggest that, eIF4E as a critical downstream effector of the mTOR pathway, may be involved in tumorigenesis of the uterine cervix, thus providing a novel target for investigational therapeutic approaches in these patients.

**OFP-10-009**

**Endometrial carcinoma: Developments during the last 5 decades in Portugal**

C. Bartosch ^*^, B. Gomes, J. M. Lopes

^*^IPO-Porto, Pathology, Portugal

**Objective:** To characterize consecutive endometrial carcinomas (EC) treated at a Portuguese tertiary hospital, and evaluate changes comparing our series with previous Portuguese studies.

**Method:** Clinical-pathological review of all EC diagnosed (2000–2010) at CHSJ; PubMed, Scielo, IndexRMP search (“Portugal” and “endometrial carcinoma”/“carcinoma”,“endometrio”); statistical analysis.

**Results:** 215 EC identified (>60 years of age: 70.2 %), with clinical-pathological features consistent with recent reports. Two previous studies (1977 Study 1: 260 EC, 55.0 % with >60 years of age; 1995 Study 2: 57 EC, 57.0 % with >60 years of age) disclosed less elderly patients, and less comorbidities, like obesity (Study 1: 44.2 %; Study 2: 36.4 %), than in our series (54.3 %). More accurate EC distribution by stage, grade, and histological types is observed over the years. There was a trend (Study1 vs. our study) to increased use of surgical staging (surgery 78.2 % vs. 90.4 %; lymphadenectomy 16.9 % vs. 27.7 %) and decreased use of radiotherapy (89.6 % vs. 52.7 %); our series overall 5-year survival (78.4 %) is better than in Study 1 (57.3 %; absent in Study 2).

**Conclusion:** Substantial developments in diagnosis, treatment, and survival of patients with EC ensued over last 5-decade, at least in part due to better healthcare, but with more morbidities possibly caused by women longevity/risk factors in Portugal.

**OFP-10-010**

**Tumor-free distance to serosa (TFD), depth of myometrial invasion (DOI) and 50 % cut-off of myometrial invasion (50 %MI) as predictors of metastasis at diagnosis and recurrence in endometrial carcinomas**

C. Bartosch ^*^, B. Gomes, J. M. Lopes

^*^IPO-Porto, Pathology, Portugal

**Objective:** To compare TFD, DOI, and 50 %MI as predictors of endometrial carcinoma (EC) metastasis at diagnosis/recurrence.

**Method:** Clinical-pathological review of all surgically treated EC at CHSJ during 2000–2010; univariate and multivariate analysis using logistic regression; and ROC curve analysis of single/model integrated prognostic parameters.

**Results:** 173 patients identified. Median age: 66 year. Tumour type: endometrioid: 80G1, 48G2, 22G3; non-endometrioid: 23. FIGO stage: 137I, 15II, 17III, 4IV. Median follow-up: 50.5 months; 25 recurrent; 25 deaths due to EC; 5 year survival: 83.3 %. TFD, DOI, and 50 %MI significantly associated to metastasis at diagnosis/recurrence/survival (univariate analysis). Prediction of metastasis was slightly better (not significant) for TFD (68.0 %AUC) compared to DOI (66.7 %AUC). Optimal cut-off values: 5 mmDOI and 8 mmTFD. In multivariate analysis of 8 mmTFD, 5 mmDOI, and 50 %MI separately, plus covariates (tumour type/grade/lymphovascular invasion), both 8 mmTFD (*p* = 0.01) and 50 %MI (*p* = 0.04), but not 5 mmDOI (*p* = 0.265) are independent prognosticators of metastasis. Prediction of metastasis at diagnosis/recurrence by the three multivariate models, showed slightly better (not significant) performance of 8 mmTFD (87.2 %AUC) vs. 5 mmDOI (86.2 %AUC) and 50 %MI (86.1 %AUC).

**Conclusion:** TFD and 50 %MI seem to be similar predictors of metastasis at diagnosis/recurrence of EC. However, tumor-free distance to serosa seems a simpler reproducible parameter in the diagnostic evaluation of endometrial carcinoma.

Tuesday, 3 September 2013, 14.15–16.15, Room 5A

**OFP-11 Oral Free Paper Session Uropathology**

**OFP-11-002**

**High RBM3 expression is an independent prognostic marker in operated prostate cancer and tightly linked to ERG activation and PTEN deletions**

K. Grupp ^*^, J. Wilking, K. Prien, C. Hube-Magg, H. Sirma, R. Simon, J. R. Izbicki, G. Sauter, S. Minner, T. Schlomm, M. Tsourlakis

^*^UKE Hamburg-Eppendorf, Allg. Chirurgie und Pathologie, Germany

**Objective:** The RNA-binding motif protein 3 (RBM3) has been suspected as a prognostic biomarker in several cancers.

**Method:** To explore the prevalence and clinical significance of RBM3 expression in prostate cancers.

**Results:** RBM3 expression was analyzed by immunohistochemistry on a tissue microarray containing 11,152 prostate cancers. RBM3 expression was more often detectable at various intensities in prostate cancers compared to benign prostate predominantly localized in the nucleus of the cells. RBM3 immunostaining was found in 64 % of the interpretable prostate cancers and was considered strong in 25.6 %. High RBM3 expression was linked to advanced tumor stage, high Gleason score, positive nodal involvement and positive surgical margin status (*p* < 0.0001 each). There was a remarkable accumulation of strong RBM3 expression in ERG positive prostate cancers and tumors harboring PTEN deletions (*p* < 0.0001 each). Moreover, RBM3 staining was tightly related to early biochemical recurrence if all tumors or subgroups of ERG negative and ERG positive cancers were analyzed (*p* < 0.0001 each). In multivariate analysis, including RBM3 staining, Gleason grade, pT stage, PSA, surgical margin status, and nodal status, the prognostic impact of RBM3 staining retained statistically significance (*p* = 0.0084).

**Conclusion:** Our observations indicate that high RBM3 expression is an independent prognostic marker in prostate cancer.

**OFP-11-003**

**Nephrogenic adenoma/metaplasia: A multicenter clinicopathological study of 106 cases**

A. Corominas-Cishek ^*^, M. Schiavo-Lena, L. Macías, A. Yagüe, R. Guarch, R. Tardanico, J. I. López

^*^Cruces University Hospital, Dept. of Pathology, Barakaldo, Spain

**Objective:** To analyze the clinicopathological characteristics of a large series of nephrogenic adenomas and metaplasias, lesions supposedly resulting from the displacement and implantation of renal tubular cells along the urinary tract

**Method:** Retrospective review of 106 cases collected in 4 different medical institutions in Spain and Italy. Sex, age, location, specimen type, histology, coexisting lesions, and main clinical settings were recorded. Immunostaining with PAX8, p63, PSMA, S100A1, CEA, CB1, CD117 and e-cadherin was performed in selected cases

**Results:** Males predominated in the series (84 M/22 F) with an average age of 65 (range 14–88). Bladder was the commonest location (66 %) but lesions were found from renal pelvis to urethra. Histologically, pure tubular (37.7 %), papillary (12.2 %) and flat (3.7 %) patterns, or combinations of them (46.8 %), were found. The most frequent concurrent lesions were urothelial carcinoma (16 %), cystitis (10.3 %), lithiasis (4.7 %) and stenosis (2.8 %). Urothelial carcinoma was the clinical antecedent in 88 % of the recorded cases. Average time between cancer resection and the diagnosis of nephrogenic adenoma/metaplasia was 16 months (range 1–168)

**Conclusion:** Nephrogenic adenoma/metaplasia is a underdiagnosed benign lesion with diverse morphology that may appear at any site along the urinary tract. The condition must be distinguished from urothelial carcinoma with which, moreover, is very frequently associated

**OFP-11-004**

**Significance of cribriform pattern of invasive prostate cancer**

G. Kir ^*^, B. Cosan, C. S. Topal, F. Zerenler, S. Cetin

^*^Umraniye Hospital, Dept. of Pathology, Istanbul, Turkey

**Objective:** There is now an increased understanding that invasive cribriform carcinoma is a relatively aggressive disease. With the revision of the Gleason system at an International Socitey of Urological Pathology (ISUP) consensus conference (2005), there was consensus that most cribriform glands should be classified as pattern 4. In some recent publications, recommendation is that all cribriform patterns be diagnosed as Gleason pattern 4 rather than Gleason pattern 3.

**Method:** We assessed the cribriform pattern associated with more definitive pattern 3, 4 and 5 elsewhere and evaluated the association of cribriform pattern with extraprostatic extension (EPE) and pathologic stage on the histopathologic evaluation of 185 radical prostatectomy specimens,

**Results:** Cribriform pattern was more frequently observed in cases with definitive patterns 4 and 5 than pattern 3. The cases with Gleason score 3 + 3 with cribriform foci, were more frequently associated with EPE and stage pT3 than the cases without cribriform foci.

**Conclusion:** Our results demonstrate that, to diagnose all cribriform patterns as Gleason pattern 4, would significantly impact on further therapeutic options and prognosis. However as many of these modifications are emprical and supported only by few sudies, long term follow-up studies with clinical end points are necessary to validate these recommendations.

**OFP-11-005**

**EGFR mutational status in patients with penile carcinoma**

I. Cunha ^*^, A. Silva, B. Lisboa, R. Rocha, S. Zequi, G. Guimarães, J. Vassalo, F. Soares

^*^Hospital A.C. Camargo, Dept. of Pathology, São Paulo, Brazil

**Objective:** Penile carcinoma (PC) is a worrisome issue in developing countries such as Brazil. Therapeutic options are limited, with poor prognosis, especially for patients with metastatic disease. Therefore, new therapeutic targets are warranted. The purpose of the present study was to search for mutations in EGFR tyrosine-kinase (TK) domain 18–21, frequently mutated in non-small cell lung carcinoma (NSCLC). This could have therapeutic implications with TK inhibitors

**Method:** Frozen tumor specimens from 29 patients with PC were submitted to direct sequencing of the EGFR-TK domain, using the polymerase chain reaction method. Sequences were analyzed with the CLC Bio software and compared with the EGFR sequence.

**Results:** None of the sequenced tumor samples showed relevant alterations in the four exons studied. However, 19 out of the 29 cases exhibited an alteration in exon 20 (p.Q787Q), which has been described as a single nucleotide polymorphism in other tumor types, without clinical significance.

**Conclusion:** In PC, unlike NSCLC, different alterations might play a role in EGFR expression and activation. As the knowledge on the molecular mechanisms of PC is still limited, because of the relative rarity of this tumor in wealthier regions of the globe, further studies are necessary to support new therapeutic alternatives.

**OFP-11-006**

**A modified point-count method as a practical approach to assessing tumor volume and percentage of prostate gland involvement by carcinoma**

D. Athanazio ^*^, P. R. Athanazio, A. Carvalho dos Santos, L. A. Rodrigues de Freitas

^*^Federal University of Bahia, Dept. of Pathology, Salvador, Brazil

**Objective:** To evaluate a modified point-count method for quantifying carcinoma in prostatectomy specimens (*n* = 142), adapted from Billis et al., Int Braz J Urol. 2013.29:113–120.

**Method:** The basal/apical margins were sampled using the cone method. The remainder of the gland was divided into 12 quadrant-shaped regions which had two slices sampled. Eight equidistant points were marked on the coverslip over each fragment. The points inside the tumoral areas were counted and expressed as the percentage of prostate gland involvement by carcinoma (PGI) and as the tumor volume (TV).

**Results:** A significant correlation between the preoperative PSA level and each of the three quantitative estimations was found, but it was higher for both values (PGI and TV) obtained using the point-count method, viz.: number of slices involved (NSI) (*r* = 0.32), PGI (*r* = 0.39) and TV (*r* = 0.44). When the data sets were stratified into three categories, all three methods correlated with Gleason scores ≥7, primary Gleason scores ≥4, perineural/angiolymphatic invasion, extraprostatic extension, seminal vesicle invasion and positive margins.

**Conclusion:** All three quantitative methods were associated with morphologic features of tumor progression. The results obtained using the modified point-count method correlated better with the preoperative PSA levels.

**Eight equidistant points were marked directly on the coverslip over each fragment.:** 
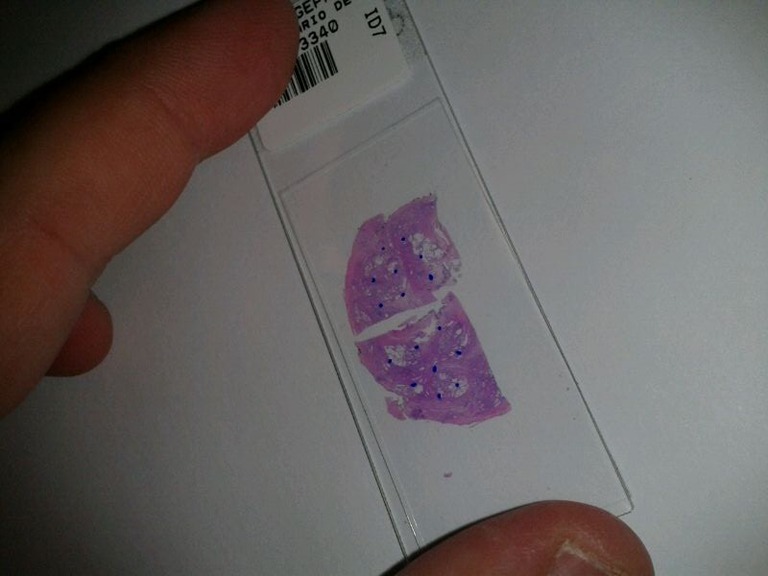


**OFP-11-007**

**Differential expression of genes related to tumor immunity in penile carcinomas with or without association to the human papillomavirus infection**

I. Cunha ^*^, M. Pavanello, A. Busso, S. Rogatto, G. Guimaraes, S. Zequi, R. Rocha, F. Soares, J. Vassallo

^*^Hospital A.C. Camargo, Dept. of Pathology, São Paulo, Brazil

**Objective:** Penile carcinoma (PC) presents a high incidence in Brazil. Around half of the cases are associated with human papillomavirus infection (HPV). Immunity to tumor seems to play a role in the pathogenesis of neoplasia. In spite of many studies on tumor immunity in other tumor types,no experience has been published on PC immunity. The present study aims to identify genes related to immunity in PC samples according to HPV status, based on a high throughput gene analysis approach.

**Method:** Four non-tumoral glans, 11 HPV positive, and 25 HPV negative PC were obtained from frozen tissue biorepository of our institution. Gene expression profiles (44 K, Agilent Tecchnologies) of the three groups were compared and those related to immunity were analyzed using the Ingenuity Pathway Analysis (IPA–http://www.ingenuity.com/).

**Results:** In silico functional characterization revealed higher expression of gamma-interferon (IFNG) gene (log2ratio = +2.40) in PC samples. In HPV positive cases, 3 out of 17 immune canonical pathways were significantly altered, namely, target cell apoptosis by T cytotoxic lymphocytes, chemokine signaling and Nur77 signaling in T lymphocytes.

**Conclusion:** Similar with other tumor types, including those related to HPV, INFG revealed to be higher expressed in PC. This result may support further studies, which might unravel its use as a therapeutic target.

**OFP-11-008**

**Analysis of alterations in the VHL gene and clinical significance in renal clear cell carcinoma cell type**

M. Alves ^*^, W. Costa, F. Silva, D. Carraro, I. Cunha, S. Zequi, G. Guimarães, F. Soares, R. Rocha

^*^AC Camargo Hospital, Anatomic Pathology, São Paulo, Brazil

**Objective:** Provide useful predictive or prognostic information in patients with renal clear cell carcinoma cell type (ccRCC) through VHL mutational status.

**Method:** VHL protein expression was analyzed by immunohistochemistry in a TMA containing 148 samples and validated in 62 cases of RNA by qRT-PCR. The mutation profile was assessed in 91 cases by Sanger sequencing.

**Results:** VHL was found mutated in 57.1 % of cases, being missense mutations present in 28.8 %, nonsense in 5.7 %, intronic mutations in 15.3 %, deletions in 44.2 %, indels in 9.6 %, duplication in 9.6 % and insertion in 1.9 % of cases. The prevalence of mutations by exon was: exon 1, 53.8 %; exon 2, 30.7 %; and exon 3, 15.3 %. Concerning VHL protein expression, our data showed high frequency of positivity (78.9 %) but no significance between protein expression, clinical data and survival were achieved. Importantly, 86.8 % of strong positive cases represented 45 muted cases of the 91 samples evaluated by sequencing.

**Conclusion:** According to our study, it is clear that a positive VHL immunostaining would not necessarily indicate a wild-type VHL status and that the examination of the VHL protein itself, downstream proteins/pathways and additional genes may provide more insights into the functional consequences of particular genetic changes and their clinical relevance.

**OFP-11-009**

**Handling of prostatectomy specimens: Simulation of partial embedding methods**

A. Alves ^*^, G. Borrecho, P. Luís, L. Correia, A. Costa-Silva

^*^Hospital de Santa Maria, Dept. de Anatomia Patologica, Lisbon, Portugal

**Objective:** Documenting staging parameters and margin status in radical prostatectomy specimens (RPS) is a key component in patient management. A variety of partial embedding methods are available with the aim of reducing costs and time constraints. Our goal is to determine the potential loss of prognostic information associated with different sampling protocols, by evaluating a commonly used tumor diagram associated with the pathology report.

**Method:** The tumor diagrams of 106 totally embedded (TE) RPS were retrospectively studied using an image editing software. Different protocols were simulated: alternate slice embedding, starting with the 1st (A1) or 2nd (A2) slice; total posterior embedding (PE); alternate slice+peripheral rim embedding (APR). Prostatic apex and base were always embedded. Tumor volume (TV), margin status and number of tissue blocks were determined.

**Results:** TV for each protocol was: 12,69 %-TE; 12,61 %-A1; 13,06 %-A2; 13,01 %-PE. In 49 cases with positive margins, the false-negative rate was: 22,5 %-A1; 26,5 %-A2; 12,24 %-PE (3 not evaluated). Block number reduction was: 42 %-A1; 48 %-A2; 36–58 %-PE; 36 %-APR.

**Conclusion:** Considering the importance of margin status, as compared to tumor volume, we think that the alternate slice+peripheral rim embedding is far superior to the other methods and is the most reliable partial embedding method, allowing prognostic information maintenance and saving of resources.

**OFP-11-010**

**Xp11.2 translocation renal cell carcinoma: Mutational analysis of KRAS and BRAF status and correlation between TFE3 immunostaining and fluorescent in situ hybridation**

M. Classe ^*^, F. Escande, V. Gregoire, J. Le Nobin, A. Villers, S. Aubert, X. Leroy

^*^CHRU Lille, Dept. of Pathology, Lille Cedex, France

**Objective:** Our aims were to study the KRAS and BRAF status of translocation renal carcinoma and to compare TFE3 expression by immunohistochemistry and gene rearrangement analysis by fluorescence in situ hybridation (FISH).

**Method:** 26 cases were studied. Immunohistochemistry was performed with TFE3 and TFEB antibodies and break apart dual color FISH assay was performed with TFE3 probe. KRAS and BRAF mutation status was analysed by PCR followed by pyrosequencing on DNA extracted from paraffin embedded samples.

**Results:** The 26 patients ranged from 7 to 76 years old (median: 31). 13 were staged pT1, 6 pT2, 4 pT3 and for 3 patients the information wasn’t available. In the follow-up 2 patients died of the disease. No KRAS or BRAF mutations were found. 22 tumors showed at least a weak (8), moderate (10) or intense (8) expression of TFE3 or TFEB (1/23) and all tumours for which FISH was interpretable, were proved to have a specific gene rearrangement in 10 % to 80 % of tumor cells.

**Conclusion:** It is the first work studying BRAF and KRAS genes in translocation renal carcinoma and reporting no mutations. We also observed a good correlation between TFE3 immunostaining and TFE3 rearrangement by FISH.

Tuesday, 3 September 2013, 17.00–19.00, Auditorium VI

**OFP-12 Oral Free Paper Session Joint Session–Digestive Diseases/Molecular Pathology**

**OFP-12-001**

**Is Src homology 2 domain of Cten essential for cell motility?**

M. Akhlaq ^*^, M. Ilyas

^*^University of Nottingham, Dept. of Molecular Medical Sciences, United Kingdom

**Objective:** Cten is a known oncogene in colorectal cancer. It is the smallest member of tensin gene family and has a role in enhancing cell motility. Cten is a fascinating protein because of its interaction with integrins and the presence of a Src Homology 2 (SH2) domain. The objective of this study is to investigate role of SH2 domain in Cten’s ability to enhance motility in cells.

**Method:** Site directed mutagenesis used to introduce a point mutation in an important Arginine residue (at site 474) in the SH2 domain of Cten. The effect of forced expression was evaluated by transwell migration and wound healing assay. Western Blot performed to evaluate levels of related proteins.

**Results:** Decrease in motility observed when transfection with SH2 mutant and Cten-GFP was compared to control Cten-GFP (*p* < 0.05). On western blotting, ILK and FAK levels were also decreased and N-cadherin and E-cadherin levels were affected showing an epithelial mesenchymal transition (EMT switch) with Cten-GFP but not with SH2 mutant Cten-GFP.

**Conclusion:** It is concluded that effect of Cten on cell motility is in part modulated through its SH2 domain. This leads to interaction with other proteins involved in signal transduction such as integrin linked kinase (ILK) and focal adhesions kinase (FAK).

**OFP-12-002**

**Failure of induction of the oncomiRic cluster miR-182/miR-96 in HCV-induced non-metastatic hepatocellular carcinoma tissues renders them as markers for metastasis**

R. Assal ^*^, H. Mohamed El Tayebi, K. Adel Hosny, G. Esmat, A. Ihab Abdelaziz

^*^German University in Cairo, Molecular Pathology Unit, New Cairo, Egypt

**Objective:** miR-182 and miR-96 are expressed as a single cluster with miR-183. They are intergenic and are located on chromosome 7q32.2. Both microRNAs were shown to be upregulated and induce metastasis in hepatocellular carcinoma (HCC). However they have never been investigated in non-metastatic HCC. We aimed at investigating the expression profile of miRNAs miR-182 and miR-96 in non-metastatic HCC.

**Method:** Total RNA was isolated from 23 Hepatitis C virus (HCV) induced non-metastatic HCC tissues and 12 healthy liver tissues. Expression of both miRNAs 182 and 96 was analyzed using qPCR after reverse transcription and normalization to RNU6B. The obtained Ct values were statistically analyzed using Mann–Whitney method.

**Results:** miR-182 and miR-96 share the same seed sequence (ACGGUU) which suggests that they can potentially target the same mRNAs thus regulating cellular functions. miR-182 and miR-96 screening in HCV-induced non-metastatic HCC liver tissues did not reveal any significant change when compared to liver tissues from healthy controls.

**Conclusion:** miR-182 & 96 are promising prognostic tissue markers for metastasis in HCC.

**OFP-12-006**

**Expression of histone modifying enzymes and histone post-translational marks in colorectal cancer**

A. Polónia ^*^, M. Coimbra, S. Reis, A. Tavares, A. Raimundo, L. Santos, C. Jerónimo, R. Henrique

^*^IPO Porto, Dept. of Pathology, Portugal

**Objective:** Characterization of the expression of histone modifying enzymes (HMEs) and their respective post-translational histone marks (HMs) in a series of colon cancer (CC) patients and determination of their value as biomarkers of prognosis and predictive of therapeutic response.

**Method:** A series of 98 CC cases were randomly selected from the archives of Portuguese Oncology Institute-Porto between March 2003 and November 2006. The expression of HMEs (EZH2, SMYD3, SETDB1 and LSD1) and HMs (H3K9me3 and H3K27me3) was determined by immunohistochemistry and assessed using a modified Histo-score.

**Results:** SETDB1 was overexpressed in left-sided CCs. EZH2, H3K9me3 and H3K27me3 were overexpressed in more invasive CCs as well as in CCs with regional lymph node metastases. LSD1 was overexpressed in less invasive CCs. Concerning response to treatment, we observed: higher survival rates in Stage I/II patients disclosing lower SETDB1 expression; higher survival rates in patients treated with Folfiri disclosing high LSD1 and H3K9me3 expression; and higher survival rates of patients treated with 5-FU/Leucovorine disclosing high H3K9me3 and H3K27me3 expression.

**Conclusion:** This work accomplished the identification of new biomarkers that might be useful in clinical practice to predict response to different treatment modalities and points to new directions in the study of colon carcinogenesis.

**OFP-12-007**

**New proteomic platform for utilization of formalin-fixed, paraffin-embedded samples in biomarkers search**

P. Ostasiewicz ^*^, J. R. Wisniewski, D. Zielinska, K. Dus, P. Ziólkowski, M. Mann

^*^Wroclaw University of Medicine, Pathology, WrocBaw, Poland

**Objective:** The aim of the study was to develop technology that would allow for effective exploration of tissue archives (paraffin blocks) with the modern proteomic tools in order to search for biomarkers.

**Method:** First, using newly developed protocol, we isolated proteins from fresh and from the formalin-fixed paraffin-embedded (FFPE) mouse liver and compared the results of the subsequent proteomic analysis. In second step we applied the protocol to the analysis of microdissected FFPE samples of human tissues. We compared the proteomes of colorectal carcinoma with patient-matched normal mucosa. All isolates were analyzed with tandem mass-spectrometry (MS/MS, Orbitrap-Velos) and raw data were evaluated with MaxQuant software.

**Results:** Protein identifications obtained for fresh and FFPE samples were the same with respect to both quality and quantity. It is also possible to analyze post-translational modifications in FFPE. The long-stored samples can be also analyzed. We quantified more than 6,000 proteins from carcinoma and paired healthy mucosa. Analysis of the data revealed that several proteins were expressed differently in normal versus neoplastic mucosa; many of them are not considered as potential biomarkers yet.

**Conclusion:** For the purpose of proteomic investigations FFPE material is equivalent to the fresh one in all aspects. Proteomics is a method of a great potential in biomarker search.

**OFP-12-008**

**Sensitivity and standardisation of multiplex molecular assays**

J. Frampton ^*^, A. Mulligan, C. Thorne, J. Goodall, K. Schmitt

^*^Horizon Discovery, Diagnostics, Cambridge, United Kingdom

**Objective:** As multiplex molecular assays are rapidly being integrated into genotype testing, it is critical to understand their sensitivity to ensure an accurate interpretation of results. Although multiplex assays offer significant advantages for mutation detection, there remain challenges as each platform presents an inherent chemistry bias and specific preparation steps. A method for standardisation and verification is therefore required.

**Method:** Quantitative Multiplex Genomic DNA and formalin fixed paraffin embedded (FFPE) cell line samples were used in this study. The multiplex standard covered the following mutations: B-Raf (V600E), K-Ras (G12D, G13D), cKIT (D816V), EGFR (G719S, ΔE746-A750, T790M, L858R and L861Q), N-Ras (Q61K), PI3KCA (E545K), PI3KCA (H1047R), and the allelic frequencies were verified using digital PCR.

**Results:** Using the multiplex reference standards, the sensitivities of the Sequenom, Ion Torrent and Mi-Seq platforms were analysed. The platforms were able to detect the majority of the mutations at the expected allelic frequencies. However, there were clear discrepancies when using the multiplex assays and some mutations were inconsistently detected.

**Conclusion:** These data illustrate both the benefits and challenges of deploying multiplex assays for complex applications such as tumor profiling. The workflow for multiplex analysis is broad and there are a number of areas which need optimization.

**OFP-12-009**

**STAT signaling is deregulated in malignant pleural mesothelioma: Are microRNAs the reason?**

L. Arzt ^*^, F. Quehenberger, I. Halbwedl, H. Popper

^*^Medizin. Universität Graz, Inst. für Pathologie, Austria

**Objective:** The STAT signaling pathway is totally deregulated in malignant pleural mesothelioma (MPM), an aggressive cancer due to former asbestos exposure. Protein expression is controlled by noncoding RNAs, e.g. microRNAs (miRNA) and we investigated the possible impact of miRNAs on the STAT signaling pathway in MPM.

**Method:** RNA was obtained from 52 formalin-fixed and paraffin-embedded tumor tissue samples. MiRNAs possibly regulating the STAT signaling pathway were identified via in silico target prediction tools. Quantitative real-time PCR was used to assess miRNA expression levels. Protein expression levels were detected by immunohistochemistry.

**Results:** STAT1 is upregulated whereas STAT3 is downregulated. The negative regulators SOCS1 and SOCS3 are totally missing. MiR-30d* regulates pSTAT1(Ser727) expression. MiR-19b, miR-30b, miR-30c and miR-222 are upregulated (target: SOCS1/SOCS3). MiR-21 (target: STAT3), a well-known oncomir, is extremely upregulated in MPM.

**Conclusion:** Absence or downregulation of proteins can be explained by upregulation of miRNAs. We currently investigate if SOCS1 and SOCS3 are expressed after downregulating the corresponding miRNAs. With these experiments we aim to answer if the STAT1/3 signaling cascade can be restored to a physiological level. Understanding the role of STATs in MPM could be the first step into the development of a targeted therapy for these tumors.

**OFP-12-010**

**MicroRNA portraits in human vulvar carcinoma**

B. Maia ^*^, A. Lavorato Rocha, I. Rodrigues, C. Coutinho, G. Baiocchi, M. Stiepcich, R. Puga, L. Lima, F. Soares, R. Rocha

^*^AC Camargo Cancer Hospital, Dept. of Molecular Morphology, São Paulo, Brazil

**Objective:** Our study aimed to characterize microRNA in vulvar tumors through an expression profile of 754 miRNAs, relating this with clinical and anatomopathologycal data, and presence of HPV infection.

**Method:** Twenty (20) HPV negative and 20 HPV positive samples, genotyped for high-risk HPVs (HPV16,18,31,33) and a pool of seven nomal vulvar skin samples were used for the identification of differentially expressed miRNAs by TLDA qRT-PCR.

**Results:** Twenty-five (25) differentially expressed microRNAs between HPV positive and HPV negative groups were obtained. A network between microRNA expression profiles and target mRNAs previously demonstrated as relevant in vulvar carcinomas, such as TP53, RB, PTEN, and EGFR was constructed. Downregulation of both miR-223-5p and miR-19-b1-5p were correlated with the presence of lymph node metastasis; downregulation of miR-100-3p and miR-19-b1-5p were correlated with presence of vascular invasion; overexpression of miR-519b and miR-133a were associated with advanced FIGO staging.

**Conclusion:** Our study demonstrates that microRNAs may be clinically important in vulvar carcinomas and our findings may help for further studies on functional implications of miRNA dysregulation in this type of cancer.

**Interactions network between differentially expressed microRNAs and their target mRNA, with relevance in vulvar carcinogenesis:** 
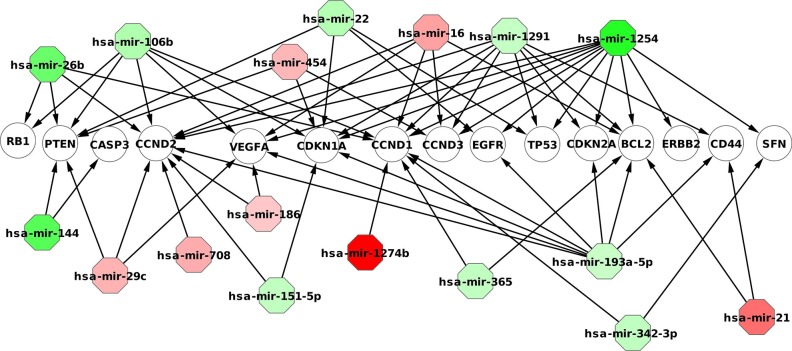


Tuesday, 3 September 2013, 17.00–19.00, Room 5A

**OFP-13 Dermatopathology**

**OFP-13-001**

**Cutaneous Melanoma: A case series of a Portuguese hospital**

R. Sampaio ^*^, J. Palla Garcia, C. Peixoto, J. Ramon Vizcaíno, T. Almeida

^*^Oporto’s Hospital Centre, Dept. of Surgical Pathology, Porto, Portugal

**Objective:** The latest data provided by Portuguese Regional Cancer Registry in 2005 placed the incidence of melanoma in 6–8 cases per 100,000 inhabitants and an increasing incidence was observed comparing with 2001. The aim of this study was to assess, clinical factors and histological predictors of survival, in skin melanoma cases of Pathology Department of Oporto’s Hospital Center.

**Method:** A retrospective review between years 2004 to 2011 was conducted. All cases of skin melanoma (*n* = 106), were evaluated in their clinical factors (age, sex, local, metastasis, lactate dehydrogenase, survival rate) and their histological factors (histological type, mitotic rate, ulceration, Clark’s level, necrosis, TNM staging, tumour thickness, regression and infiltrating lymphocytes). The association between survival and clinical/pathologic factors were analyzed using Cox regression. Predictive factors of distant metastasis were evaluated by logistic regression.

**Results:** The presence of distant metastasis was the only independent factor associated with survival (*p* = 0,022). Histological type, tumour thickness, Clark’s level, necrosis, ulceration were independent predictive factors of distant metastasis at presentation.

**Conclusion:** The purposes of pathologic examination are to provide an accurate diagnosis of melanoma and an useful prognostic information in the clinical management of the patient. In the near future, pathologic reports can also be used to predict responses to therapy.

**OFP-13-002**

**Correlation between diagnostic shave biopsies and therapeutic excisions done for Lentigo Maligna (LM) cases: A 4-year audit study**

R. Ramakrishnan ^*^, J. Lee, S. Fernandez, J. Weir

^*^Charing Cross Hospital, Dept. of Cellular Pathology, London, United Kingdom

**Objective:** Diagnostic shave biopsies are routinely performed at our centre for lentigo maligna bearing cosmesis in mind. The aim of the study was to evaluate the different categories of diagnostic biopsies for LM, look for sampling/diagnostic inadequacies related to procedure & correlate with post-excision histology.

**Method:** LM cases diagnosed at our hospital(s) over 4 years were retrieved. Information pertaining to nature of biopsy, primary diagnosis, diagnosis at excision and margin clearance (in mm) were noted.

**Results:** 70 clinical suspicious cases of LM who underwent biopsies were retrieved from database. 30 (43 %) were incisional ellipses, 8 (11.5 %) punch & 32 (45.5 %) shave biopsies. Histologically, 46 (65.5 %) were LM, 9 (13 %) LM with insitu melanoma (LMis), 6 (8.5 %) lentigo maligna melanoma (LMM) & 9 (13 %) solar/actinic lentigo/non-diagnostic of LM. Re-excision resulted in 3 cases being reclassified as radial growth phase melanoma in situ (4 %) and another 3 (4 %) as LMM. None of the 9 non-diagnostic cases had suspicious lesions on follow-up

**Conclusion:** Shave biopsies have an excellent diagnostic pick-up rate (almost 100 %) for LM or higher grade lesions with no false negativity recorded in our series. Subsequent re-excision biopsy showed a small proportion of high-grade lesions(6/70, 8 %) not seen in the original biopsy.

**OFP-13-003**

**Inflammatory infiltrate in melanoma with regression as prognostic parameter**

S. Zurac ^*^, G. Negroiu, R. Andrei, S. Petrescu, T. Tebeica, M. Petre, M. Neagu, C. Constantin, V. Chitu, C. Salavastru, F. Staniceanu

^*^Colentina University Hospital, Pathology, Bucharest, Romania

**Objective:** Regression in melanoma has unknown biologic significance. Our previous studies demonstrated different immunophenotype in areas of regression (RM) and nonregressed areas (NRM) in melanoma, the latter being similar to melanoma without regression (absence of regression–AR), thus identifying regression as expression of intratumor heterogeneity. This study characterized inflammatory infiltrate in different areas of melanoma.

**Method:** We analyzed inflammatory infiltrate in 102 cases of melanoma (in AR, NRM and RM) by immunohistochemical stains for CD3, CD5, CD7, CD4, CD8, CD25, FOXP3, CD20, CD23, CD138, CD1a, Langerin.

**Results:** The majority of tumor infiltrating lymphocytes (TIL) were T-cells, the morphologic estimation of TIL overlapping to IHC identification of T-cells (*P* < 0.001); slight predominance of CD4+ T-cells (CD4:CD8 > 1:1) was present, without correlation with pT level, ulceration or regression. B-cells (CD20+) and plasma cells (CD138+) were more frequent in advanced tumors, without statistic correlation. Langerhans cells (LCs) were more frequent in superficial spreading melanomas than nodular ones (*P* < 0.001); LCs were constantly associated with thinner tumors (*P* = 0.007); nodular distribution of LCs in RM associated frequent LCs in NRM (*P* = 0.038).

**Conclusion:** LCs infiltration is a promising parameter to be evaluated in melanomas for both predicting progression and therapeutic possibilities. Acknowledgments: project partially supported by Postdoctoral Program POSDRU/89/1.5/S/60746

**OFP-13-004**

**Fluorescence in Situ Hybridization (FISH) assay in melanoma prognostication**

G. Dyduch ^*^, A. Sinczak-Kuta, J. Szpor, M. Bialas, S. Demczuk, K. Okon

^*^Jagiellonian University, Dept. of Pathomorphology, Kraków, Poland

**Objective:** A 4-color fluorescence in situ hybridization assay (FISH) with probes targeting 6p25, centromere 6, 6q23 and 11q13 are being used to differentiate melanoma from melanocytic nevi. The significance of the assay in the discriminating melanomas with metastatic potential from those with an indolent course is a matter of controversy

**Method:** 50 cases of malignant melanoma with 5-year follow-up, including 13 with lymph node and 9 with distant metastases were analyzed using Melanoma Vysis FISH Probe Kit.

**Results:** Of 50 melanomas 40 were FISH positive according to the previously defined criteria (82 % sensitivity). Metastatic melanomas showed lower positivity in comparison with non-metastasic ones (77 % vs.82 %). In 10 FISH negative cases 4 were associated with distant and 1 with lymph node metastasis. There were no significant differences in patient survival between FISH-positive and FISH-negative cases. Among local prognostic factors ulceration was positively correlated with percentage of tumor cells with increased number of 6p25 copies.

**Conclusion:** Melanoma FISH assay seems to be of limited value in predicting the course of malignant melanoma, nevertheless further analysis of larger number of cases is needed to elucidate the issue.

**OFP-13-005**

**Melanoma MAPK pathway proteins and associated tumour suppressors: p16 is an independent prognostic biomarker by tissue microarrays**

J. Lade Keller ^*^, R. Riber-Hansen, P. Guldberg, H. Schmidt, T. Steiniche

^*^Aarhus University Hospital, Institute of Pathology, Aarhus C, Denmark

**Objective:** To produce a combined evaluation of changes in the immunohistochemical (IHC) expression of key melanoma drivers in order to assess their impact on progression and prognosis of melanoma.

**Method:** Tissue microarrays were constructed from a cohort of primary melanomas (*n* = 355) and evaluated for IHC expression of MAPK pathway activators (c-KIT; BRAF V600E oncoprotein; pERK; MITF) and related tumour suppressors (p16; p53). The results were correlated with clinicopathological parameters and clinical outcome. Median follow-up time was 9 years.

**Results:** In univariate analysis, absent p16 expression and reduced MITF expression were both associated with ulceration (*p* = 0.009 and *p* < 0.0001, respectively), tumour stage III (*p* < 0.0001 and *p* = 0.001, respectively), and increasing Breslow thickness (both *p* < 0.0001), as well as with a poor melanoma-specific survival (*p* = 0.002 and *p* = 0.05, respectively), overall-relapse-free survival (*p* < 0.0001 and *p* = 0.003, respectively), and distant-metastasis-free survival (*p* < 0.0001 and *p* = 0.02, respectively). In addition, absence of p16 expression predicted overall-relapse-free (*p* = 0.02) and distant-metastasis-free (*p* = 0.04) survival, independently of Breslow thickness, ulceration and tumour stage. Expression of c-KIT, BRAF V600E oncoprotein, pERK, and p53 did not consistently predict adverse prognosis.

**Conclusion:** IHC determined p16 expression is an independent prognostic marker of potential value in daily melanoma practice.

**OFP-13-006**

**An aggressive hypoxia related subpopulation of melanoma cells is TRP-2 negative**

D. Lenggenhager ^*^, A. Curioni-Fontecedro, M. Storz, O. Shakhova, D. Widmer, B. Seifert, H. Moch, R. Dummer, D. Mihic-Probst

^*^Kantonal Hospital St. Gallen, Inst. für Pathologie, Switzerland

**Objective:** TRP-2 is an enzyme involved in melanin biosynthesis and known as a melanoma differentiation antigen. In mice Trp-2 was shown to be expressed in melanocyte stem cells of the hair follicle; therefore it was also considered to be an indicator of stemness. The potential role in tumor progression suggested TRP-2 as a target for vaccination. Therefore, there is a need to further investigate on the role of TRP-2 in melanoma as a stem cell or differentiation marker.

**Method:** We analysed the expression of TRP-2 in over 200 melanoma biopsies and cell lines. In order to define if TRP-2 can be considered as an indicator of stemness, we analysed the mouse hair follicle for Trp-2 expression.

**Results:** We found an association between TRP-2 expression and differentiation. We identified a hypoxia related TRP-2 negative proliferative subpopulation which is associated with tumor thickness and disease progression, indicating a less favourable tumor specific survival. Also in mice we could show that Trp-2 is expressed in differentiated melanocytes.

**Conclusion:** Our findings underline that TRP-2 is a differentiation antigen whose expression decreases during tumor progression, therefore TRP-2 vaccination strategies would not target the hypoxia related proliferative, undifferentiated subpopulation of melanoma, indicating the need for combined approaches.

**OFP-13-007**

**CD8+ T-cells in invasive and in situ squamous cell carcinoma of the skin and actinic keratosis**

H. Kourea ^*^, A. Stravodimou, V. Tzelepi, H. Papadaki, C. Scopa

^*^Uniersity of Patras, Dept. of Pathology, Greece

**Objective:** CD8+ Τ-cells participate in tumor surveillance and impede tumor growth, a function extensively studied in cutaneous melanoma. We examined the presence of C8+ T-cells in actinic keratosis (AK), in situ (IS) and invasive (IN) squamous cell carcinoma (SCC) of the skin.

**Method:** CD8+ Τ-cells were identified using immunohistochemistry and recorded using image analysis in 25 cases of INSCC and 18 cases of IS (and their adjacent IS, AK or benign tissue (BN), when present). Statistical analysis was performed using the paired *T*-test. *p*-values < 0.05 were considered statistically significant.

**Results:** In INSCC, peritumoral (PT) CD8+ Τ-cells were more numerous than the intratumoral (IT) CD8+ Τ-cells (*n* = 25) (mean 277 vs. 129, *p* < 0,001) and the adjacent ΒN (*n* = 21) (mean 277 vs. 118, *p* < 0,001). Significant differences in INSCC, were not observed between PT CD8+ T-cells and those in the adjacent IS and AK. In ISSCC, PT CD8+ Τ-cells were more numerous around IS compared to the adjacent ΒN (*n* = 15) (mean 238 vs. 54, *p* < 0,001) and in AK compared to BN (*n* = 11) (177 vs. 54, *p* = 0,031), respectively.

**Conclusion:** CD8+ Τ-cell infiltration of the skin increases early from the precancerous AK, indicating early involvement of the immunologic response in the prevention of development of SCC.

**OFP-13-008**

**Early B cell differentiation in Merkel cell carcinomas indicates cellular ancestry**

A. Zur Hausen ^*^, D. Rennspiess, V. Winnepenninckx, E.-J. Speel, A. K. Kurz

^*^Maastricht University Medical Centre, Dept. of Pathology, The Netherlands

**Objective:** To assess the cellular origin of Merkel cell carcinomas.

**Method:** We tested 21 MCCs for the expression of MCPyV, TdT, PAX5, IgG, IgM, IgA, kappa and lambda by immunohistochemistry and assessed IgH and Igk rearrangement in all 21 MCCs.

**Results:** All of the MCCs revealed specific expression of PAX5 and 72.8 % of the MCCs expressed TdT. In addition, most of the MCC revealed specific expression of one or more Ig subclasses and kappa or lambda. One MCC did reveal monoclonal IgH and Igk rearrangement next to 2 other MCCs showing Igk rearrangement.

**Conclusion:** Since co-expression of TdT and PAX5 under physiological circumstances is restricted to pro/pre- and pre-B cells we propose–based on our results–that the cell of origin of MCC is a pro/pre- or pre-B cell rather than the postmitotic Merkel cells. MCPyV infection and transformation of pro-/pre- B cells is likely to induce the expression of simple cytokeratins as has been shown for SV40 in other non epithelial cells. This model of cellular ancestry of MCC might impact therapy and possibly helps to understand why approximately 20 % of MCC are MCPyV negative. zur Hausen A, et al., Cancer Res. 2013, in press.

Tuesday, 3 September 2013, 17.00–19.00, Room 5B

**OFP-14 Oral Free Paper Session Nephropathology**

**OFP-14-001**

**Preimplantation analysis of kidney biopsies from Expanded Criteria Donors (ECD)**

A. Sagasta Lacalle ^*^, A. Sánchez Escuredo, F. Oppenheimer, M. Solé

^*^Hospital Clinic Barcelona, Dept. de Anatomía Patológica, Spain

**Objective:** Preimplantation analysis of kidney biopsies in ECD is commonly used, with little consensus on management standards. Aim: To analyze the concordance between different observers and techniques for the parameters used in routine according to Remmuzzi score (Rs).

**Method:** Retrospective analysis of 92 biopsies (2000–2010). Frozen sections (FS) were blindly revised by a trained observer and compared with original reports (interobserver correlation). Frozen and paraffin sections (PS) analysis by the same observer were also compared (correlation between techniques). For correlation analysis Kendall’s Tau b (KTb) was calculated.

**Results:** Agreement between observers using FS was weaker than correlation between techniques in all examined parameters (KTb Rs 0.104 vs 0.306). Glomerulosclerosis was the parameter with better correlation (KTb FS vs PS 0.358), and tubular atrophy was the one with the worst (KTb FS vs PS 0.1579). Combined tubulo-interstitial score did not improve correlation (KTb IF/TA 0.346/0.15 vs combined 0.157) According to Rs, PS and FS analysis would have result in higher rate of organ rejection (12 and 14, respectively) than the original report (6).

**Conclusion:** Correlation between FS and PS is weak, but better than interobserver agreement. Given the relevance of the observed differences in organ acceptance, specific training is advisable irrespective of the technique used.

**OFP-14-002**

**Kidney allografts with biopsy features of chronic mixed rejection reflect poorer survival than those with pure chronic antibody-mediated rejection**

D. Dobi ^*^, Z. Bodo, K. Boda, E. Kemeny, E. Szederkenyi, B. Ivanyi

^*^University of Szeged, Dept. of Pathology, Hungary

**Objective:** The lesions of chronic antibody-mediated rejection (CABMR) and T-cell-mediated rejection (TCMR) can coincide. Since the clinicopathologic relevance of this pattern, termed chronic mixed rejection (CMR), has not been entirely elucidated, it was subjected to analysis.

**Method:** The lightmicroscopic, immunohistochemical (C4d, HLA-DR) and ultrastructural features of 61 consecutive biopsies displaying transplant glomerulopathy and/or peritubular basement membrane multilayering were re-evaluated, applying the Banff 09 categories. Arterial fibrointimal thickening with lymphocytes/macrophages received a distinct score (cv ly/ma). Hierarchical cluster analysis, Cox proportional hazards and Kaplan-Meier curves served to determine the significance of lesions relating to graft survival.

**Results:** 42.6 % of the cases exhibited CMR. The cluster analysis identified cv ly/ma and peritubular capillaritis as TCMR lesions; and glomerulitis as an antibody-mediated lesion. The median survival in CMR was significantly lower than that in pure CABMR cases (9 vs 26 months, respectively). On Cox regression, acute TCMR grades Ia/Ib, cv ly/ma, calcineurin inhibitor toxicity and tubular atrophy had significant negative effects on survival.

**Conclusion:** CMR was a common phenotype in our series; cv ly/ma represented chronic active TCMR, as indicated by Banff 09; CMR carried an inferior prognosis than pure CABMR, independently of the pattern of the T-cell-related component (supported by TAMOP 4.2.2.A-11/1/KONV-2012-0035 grant to B.I.).

**OFP-14-003**

**Chronic transplant glomerulopathy: Clinical and morphological characteristics**

A. Perkowska-Ptasinska ^*^

^*^Warsaw Medical University, Transplantology and Nephrology, Poland

**Objective:** Chronic transplant glomerulopathy (TG) is for a long time recognized type of kidney graft injury, but apart from glomerular capillary lesions the morphology of kidney grafts with TG has not been yet described. The aim of the study was to characterize the morphology of kidney grafts with TG and to analyze the impact of studied lesions on the graft survival.

**Method:** 159 TG cases were retrospectively compared with non-TG group comprising 85 recipients with IFTA and/or chronic arteriolo- or arteriopathy.

**Results:** TG group was characterized by a higher incidence of some of glomerular lesions (e.g. mesangiolysis, glomerulitis), tubulitis, C4d positivity, PTC-itis and interstitial inflammation. Among vascular lesions endarteritis, proliferative arteriopathy (arteriosclerosis without elastica multiplication), arteriolosclerosis, and hypertrophy/hyperplasia of arteriolar SMCs were significantly more common in TG group. Although the mean time interval between transplantation and TG recognition was 6 years, an active AMR with C4d deposition in PTCs was found in 43 %, and an acute T-cell rejection in 20 % of TG cases. Among lesions studied proliferative arteriopathy and glomerular thrombi were found to have an independent negative impact on graft survival.

**Conclusion:** TG is associated with a spectrum of inflammatory and structural changes in the renal transplant microvasculature, interstitium and arterial tree.

**Morphological characteristics of TG, and non-TG groups:**

**Table:** 

TG *vs* non-TG cases			
light microscopic evaluation Banff criteria + additional parameters	the incidence in TG group	the incidence in non-TG group	p
C4d in PTCs	42.86 %	1.3 %	<0.0001
PTC-itis	40.88 %	11.76 %	<0.0001
PTCs’ dilatation	35.85 %	10.59 %	<0.0001
acute interstitial inflammation (“i”)	30.82 %	10.59 %	<0.0001
total interstitial inflammation (“ti”)	81.76 %	68.24 %	0.02
the percantage of globally or segmentally sclerosed glomeruli	30.21 ± 21.71	19.59 ± 21.15	<0.0001
mesangiolysis	38.99 %	11.76 %	<0.0001
increase in mesangial celullarity	38.99 %	8.24 %	<0.0001
increase in mesangial matrix volume	48.43 %	27.06 %	0.002
*glomerulitis*	61.01 %	7.06 %	<0.0001
*tubulitis*	31.45 %	10.59 %	0.0002
*endarteritis*	9.79 %	0	0.003
arteriosclerosis without the multiplication of elastic lamina	18.88 %	3.61 %	0.0009
arteriosclerosis with the multiplication of elastic lamina	28.67 %	22.89 %	NS
arteriolar sclerotization	47.17 %	18.82 %	<0.0001
arteriolar SMCs hyperplasia	39.62 %	11.76 %	<0.0001
arteriolar wall hyalinization	86.79 %	77.65 %	NS

**OFP-14-004**

**Categorization of the diabetic nephropathy by Tervaert classification in clinical setting**

F. Moreno ^*^, A. Pinho, R. Dias, J. R. Vizcaino

^*^Centro Hospitalar do Porto, Dept. de Anatomia Patológica, Portugal

**Objective:** To assess the reliability and prognostic value of the Terveaert classification system in renal biopsies (RN), usually performed in type 2 diabetes mellitus patients with an atypical presentation of renal disease in clinical setting.

**Method:** Single-center study in a tertiary referral center for renal pathology. Three pathologists (1 senior reader: >10 year of experience; 1 intermediate reader: 3 year of experience; and 1 junior reader: first year of practice) evaluated retrospectively consecutive biopsies and categorized diabetic nephropathy (DN) blinded for inter-observer assessment and clinical outcome.

**Results:** Among 710 RN evaluated, there were 12 pureDN and 8 DN coexisting with other types of pathology (mixedDN). The mixedDN forms were associated to IgA nephropathy (3), transplant glomerulopathy (2), light chain disease (1), amyloidosis (1) or HIV-Associated Nephropathy (1). As it was previously reported, the inter-observer reproducibility for Terveaert classification was good (K = 0.82). The estimated 5-year renal survival rate was 98.4 % in ClassesII, 54.3 % in ClassIII, and 36.2 % in ClassIV (*p* = 0.04).

**Conclusion:** These findings corroborate the results from experimental centers: in fact, Tervaert classification seems to be user friendly, accurate and clinically useful in DN. Future studies are therefore recommended, in order to be consistently applied in clinical practice.

**OFP-14-005**

**Clinicopathological characteristics of segmental and global active subclasses of class IV lupus nephritis**

M. Wagrowska-Danilewicz ^*^, M. Danilewicz

^*^Medical University of Lodz, Dept. of Nephropathology, Poland

**Objective:** The prognosis of lupus nephritis is predicted by the class, activity and chronicity of the glomerular and interstitial pathology. The purpose of the study is to compare the clinical and laboratory data and the severity of glomerular active features in segmental active subclass of class IV lupus nephritis (IV-S/A) and global active subclass (IV-G/A)of class IV lupus nephritis.

**Method:** A retrospective analysis of 34 patients with IV class of lupus nephritis was performed. Clinical and laboratory data were available in all patients selected.

**Results:** Of 34 patients with class IV lupus nephritis, 25 were classified as having class IV-G/A lesions, and 9 patients class IV-S/A lesions. Nephrotic syndrome and hypertension were significantly more frequent in patients with IV-G/A lesions, whereas hematuria and low grade proteinuria were significantly more frequent in patients with IV-S/A. Class IV-S/A showed predominant mesangial deposits, and less prominent immune deposits on immunofluorescence in comparison to class IV-G/A. Fibrinoid necrosis of glomerular tufts were significantly more evident in class IV-S/A. Endocapillary hypercellularity and hyaline deposits on light microscopy were more frequent in class IV-G/A.

**Conclusion:** Results of the study point to the clinical and morphologic differences between class IV-S/A and class IV-G/A lupus nephritis.

**OFP-14-006**

**Role of renal biopsy in silent lupus nephritis**

M. E. Guerra ^*^, Y. Arce, M. M. Díaz, P. Moya, J. Ballarín, F. Algaba

^*^Hospital Central de Asturias, Dept. of Pathology, Oviedo, Asturias, Spain

**Objective:** To evaluate the frequency of silent lupus nephritis (SLN) in protocol renal biopsy (PRB) after 2 years of complete clinical remission

**Method:** Prospective and descriptive study of patients diagnosed of lupus nephritis with criteria of complete renal remission.

**Results:** 9 patients, 7 women and 2 men with a mean age 24.2 years. Results of first biopsies were: 1 patient was class II, 1 patient class III, 5 patients class IV and 2 patients class V with a mean actitivity and chronicity index of 5.5 and 0.87, respectively. In PRB 5 patients were class II, 1 patient class V and 3 patients a mixed pattern (II or III+V) with a mean activity and chronicity index of 0.62 and 1.12, respectively. Considering the result of PRB, mantainance treatment was decreased in 3 patients, in 5 patients remained and was increased in 1 patient.

**Conclusion:** After PRB no patients achieve complete histological remission: membranous pattern remains, endocapillary proliferation decreases and in all of them, mesangial proliferation holds. Activity index decreases, meanwhile chronicity index increases. SLN is highly prevalent in patients with systemic lupus erythematosus, being renal biopsy the gold standard for diagnosis. In our study, PRB implicates a change in therapeutic decision in 44.4 % of cases.

**Table 1.:**

**Table:** 

N	FIRST BIOPSY			PROTOCOL RENAL BIOPSY			THERAPEUTIC OUTCOME
	CLASS	ACTIVITY INDEX	CHRONICITY INDEX	CLASS	ACTIVITY INDEX	CHRONICITY INDEX	
1	V	0	0	V	0	3	no change
2	III	7	1	III+V	2	1	increase
3	IV	13	0	II+V	0	1	no change
4	II	1	2	II	0	2	decrease
5	II+V	0	0	II+V	1	1	no change
6	IV	9	4	II	2	0	no change
7	IV	5	0	II	0	0	no change
8	IV	9	0	II	0	1	decrease
9	IV	–	–	II	0	0	decrease

**OFP-14-007**

**Hypocomplementemic urticarial vasculitis syndrome in kidney biopsies**

A. Vizjak ^*^, J. Mraz, T. Avcin, A. Hocevar, J. Lindic, D. Ferluga

^*^Faculty of Medicine Ljubljana, Inst. of Pathology, Slovenia

**Objective:** Hypocomplementemic urticarial vasculitis syndrome (HUVS) is a rare, not yet fully explored immune-complex small vessel systemic vasculitis involving kidney in about 50 % of patients.

**Method:** Using light, immunofluorescence and electron microsopy, we studied histopathologic changes in 14 kidney and 8 skin biopsies of 8 HUVS patients.

**Results:** HUVS was diagnosed in 4 children, 2 were siblings (mean age 6.9 years) and 4 adults (mean age 50.3 years). In 3 patients, HUVS developed into SLE, manifesting with positive anti-DNA antibodies, after the first kidney biopsy. Light microscopy in the initial kidney biopsy showed mild mesangial glomerulonephritis (GN) (*n* = 3), focal proliferative GN (*n* = 3) combined with membranous GN in one case, and diffuse proliferative GN (*n* = 2) combined with membranous GN in one case. Granular full-house immune deposits in glomeruli, extraglomerular vessels and extensively in the tubulointerstitial compartment were seen in all cases by immunofluorescence, confirmed by electron microscopy. Fingerprint ultrastructure of electron dense deposits was noted in 5 cases. Skin biopsy revealed immune-complex leukocytoclastic vasculitis in all 8 cases.

**Conclusion:** Our study showed that light, immunofluorescence and electron microscopy histopathologic features of HUVS are very similar to SLE, in accordance with the not infrequent clinical and immunoserological overlapping of the two diseases.

**OFP-14-008**

**Pharmacological nephroprotection in a novel mouse model of hereditary nephrotic syndrome**

I. Simic ^*^, M. Tabatabaeifar, H. Denc, T. Wlodkowski, G. Mollet, C. Antignac, F. Schaefer

^*^University Children’s Hospital, Pediatric Nephrology, Heidelberg, Germany

**Objective:** Inducible knock-in mice carrying the R140Q podocin mutation develop nephrotic-range proteinuria and focal-segmental glomerulosclerosis. The aim of our study was to test the nephroprotective efficacy of RAS antagonists in this mouse model.

**Method:** C57BL/6 mice with Nphs2Flox/R140Q, Cre+genotype were injected with Tamoxifen for 5 days to induce hemizygosity for R140Q-mutant podocin. The animals were treated prophylactically with ramipril (R), candesartan (C), the combination of ramipril and candesartan (R+C), amlodipine (A) or remained untreated with either tamoxifen induction (sick controls) or vehicle injections (healthy controls). Histopathological changes were examined after 4 weeks.

**Results:** Animals treated with RAS antagonists scored lower glomerular sclerosis indices (R, 1.00; C, 1.13; R+C, 0.91; A, 1.20; sick controls, 1.47; healthy controls, 0.25). The average number of podocytes per glomerulus was reduced by 50 % in sick animals, but was preserved in R+C animals (R, 70; C, 72; R+C, 79; A, 62; sick controls, 39; healthy controls, 75). Despite preserved podocin mRNA expression, Western blot analysis showed subtotal loss of podocin protein in all induced animals irrespective of pharmacological treatment.

**Conclusion:** The administration of RAS antagonists markedly attenuates podocyte loss and delays glomerulosclerosis in mice carrying the most common human podocin mutation. Our findings suggest that RAS blockade provides effective pharmacological nephroprotection in this hereditary podocytopathy.

**OFP-14-009**

**Warfarin related nephropathy in human and experimental animals**

S. Brodsky ^*^, A. Satoskar, L. Hebert, T. Nadasdy

^*^Ohio State University, Dept. of Pathology, Columbus, USA

**Objective:** We had reported that excessive anticoagulation in patients on warfarin therapy (INR > 3.0) can result in acute kidney injury (AKI). Morphologic findings included glomerular hemorrhage and renal tubular obstruction by red blood cell (RBC) casts. The clinical outcome in these patients was unfavorable. We named this condition warfarin related nephropathy (WRN). The aim of this study is to reproduce WRN in an animal model.

**Method:** CKD was modeled by 5/6 nephrectomy (5/6NE) in rats. Control and 5/6NE were treated with different warfarin doses at different stages of CKD development (3, 9 and 19 weeks after the surgery). Serum creatinine and hematuria were measured; the kidney pathology was evaluated.

**Results:** Treatment with warfarin resulted in a dose-depended increase in prothrombin time and was associated with elevated Scr and hematuria in 5/6NE, but not control. SCr increase was correlated with CKD progression. Vitamin K prevented Scr and hematuria increase. Morphologically, RBC tubular casts were present, similar to those seen in humans (Figure 1).

**Conclusion:** WRN can be modeled in 5/6NE model of CKD. Excessive anticoagulation with warfarin results in increased SCr and hematuria, and RBC in tubules, resembling findings in humans. The pathogenesis of WRN can be studied in 5/6NE rats.

**Figure 1:** 
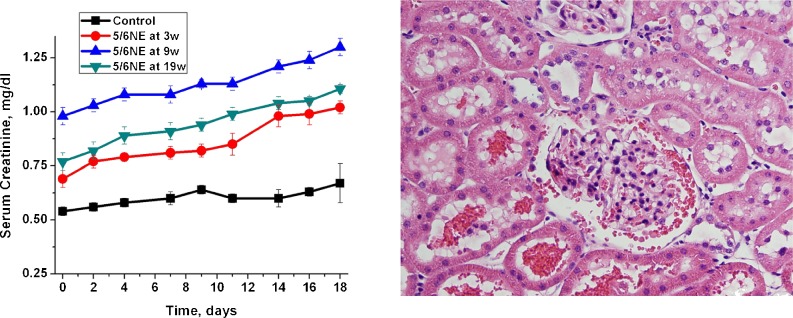


**OFP-14-010**

**A case of glomerular basement membrane lamellation associated with mutation in the MYO1E gene and not with Alport syndrome**

S. Leh ^*^, D. Brackman, I. Mujic, H. Boman, E. Svarstad, T. Fiskerstrand

^*^Haukeland University Hospital, Dept. of Pathology, Bergen, Norway

**Objective:** Glomerular basement membrane lamellation is the hallmark of Alport syndrome, but is occasionally also found in other hereditary renal diseases. This case was at first erroneously diagnosed as Alport syndrome due to pronounced basement membrane changes.

**Method:** A 10 year old boy developed hematuria and proteinuria. A kidney biopsy showed mesangial changes, segmental sclerosis in few glomeruli, interstitial foam cells and by electron microscopy thickening, lamellation and splitting of the glomerular basement membrane. A diagnosis of Alport syndrome was made. The boy had normal hearing, no eye abnormalities, and sequencing of the COL4A5 gene revealed no pathogenic sequence variants. There was no family history of kidney disease, but of parental consanguinity, which prompted us to investigate the possibility of an autosomal recessive disease. We performed a 250 K Affymetrix SNP-array on DNA, to look for areas of homozygosity.

**Results:** The largest region of homozygosity (20.8 Mb) on chromosome 15 contained one known podocyte gene MYO1E, encoding a membrane-associated non-muscle class I myosin. By RNA analysis, we detected homozygosity for a large deletion (c.1905_2049del145), encompassing exon 19 of MYO1E, and revised the diagnosis to MYO1E associated focal segmental glomerulosclerosis.

**Conclusion:** The differential diagnosis of glomerular basement membrane lamellation includes MYO1E associated focal segmental glomerulosclerosis.

**Basement membrane thickening, lamellation and splitting:** 
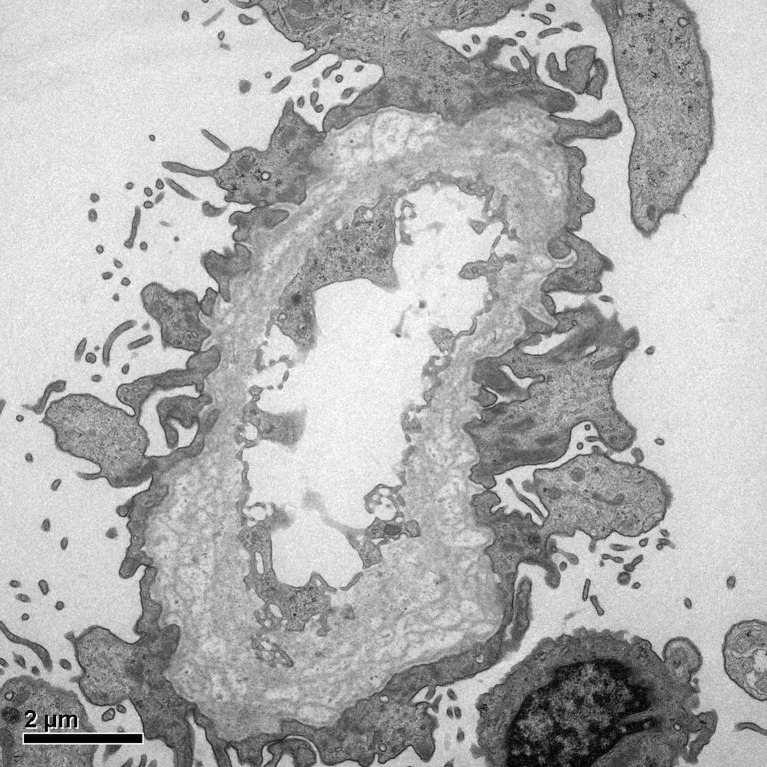


Tuesday, 3 September 2013, 17.00–19.00, Room 5C

**OFP-15 Oral Free Paper Session Cytopathology**

**OFP-15-001**

**Performance of ProEx C in detecting cervical squamous intraepithelial lesions among women with ASC cytology**

R. Alaghehbandan ^*^, S. Ratnam

^*^Memorial University of Newfoundland, Dept. of Anatomic Pathology, St. John’s, Canada

**Objective:** The clinical usefulness of the ProEx C test (BD) for the detection of cervical intraepithelial neoplasia (CIN) among women with atypical squamous cells (ASC) cytology was determined in a multicentre study carried out in Canada.

**Method:** The study population comprised of women representing five of the ten Canadian provinces referred to colposcopy for further assessment of cervical cancer risk and follow-up, and those routinely screened. Cervical specimens were collected in PreservCyt and cytology was performed using the ThinPrep method (Hologic). Histology confirmed CIN served as the disease endpoint to assess the test performance.

**Results:** The study analysis was based on a total of 354 women (mean age 30.6 years) with 75 (21.0 %) having CIN 2+. Overall, ProEx C was positive in 33.3 % (119/354). ProEx C detecting CIN was best found at CIN2+ with a concordance rate of 74.3 % (95 % CI: 73.1–75.5). The sensitivity of ProEx C in detecting CIN2+ lesions was 68.9 % (95 % CI: 67.7–70.2) while the specificity was 75.7 % (95 % CI: 74.6–76.9).

**Conclusion:** In conclusion, ProEx C was reasonably specific but less sensitive in detecting CIN2+ lesions. ProEx C may have the potential to serve as a useful adjunct test for the detection of CIN2+ lesions in cervical cancer screening.

**OFP-15-002**

**Accuracy of diagnosing pathological changes of glandular epithelium in cervical smears of primary screening**

Z. Pohar Marinsek ^*^, V. Kloboves Prevodnik, S. Uhan Kastelic

^*^Institute of Oncology, Dept. of Cytopathology, Ljubljana, Slovenia

**Objective: D**etermine accuracy of diagnosing pathological changes of glandular epithelium of the uterine cervix in primary screening.

**Method:** Study included 573 cervical smears diagnosed as atypical glandular cells (AGC), severly atypical glandular cells/adenocarcinoma in situ (SAGC/AIS) or invasive adenocarcinoma (IA) during primary screening between 2005 and 2009 at the Institute of Oncology, Ljubljana (IO). All smears were reviewed and final diagnoses obtained from histology or follow-up were compared to those from primary screening and to those from the review.

**Results:** Follow-up revealed 70 glandular neoplasias, 79 squamous neoplasias and 413 negative cases. 85 % of AGC and 33 % of SAGC/AIS from primary screening were negative on follow-up. 14 % of AGC, 36 % of SAGC/AIS and 8 % of IA from primary screening were squamous neoplasias. On review 61 % of true negative cases were corecty diagnosed and only 1.5 % of cases diagnosed as AGC or glandular neoplasia were in fact squamous neoplasia.

**Conclusion:** Between 2005 and 2009 there was a high procentage of cytological overdiagnosis concerning cervical glandular epithelium and poor distinction between glandular and squamous neoplasia in primary screening. Improved results on review are probably due to education of screeners and cytopathologists prior to organized cervical screening in Slovenia.

**OFP-15-003**

**Digital holographic microscopy as screening tool for cervical cancer**

H. Sevestre ^*^, N. Benzerdjeb, P. Camparo, C. Garbar

^*^CHU, Pathology, Amiens, France

**Objective:** To identify optic criteria of normal and abnormal cervical cells upon examination with a digital holographic microscopy (DHM) and software (Holocyt Intelligence System (HIS).

**Method:** Analysis performed with the DHM, vial “c-Box”, software (OZONE®) provided by HIS. Residual material of LBC from three labs, poured into a “c-Box” subjected to DHM. At least 20 normal or abnormal looking cells reconstructed selected per vial. Criteria: maximum height nuclear (MHN), normalized nuclear to cytoplasm height (NHN), nuclear diameter (ND) and nucleo-cytoplasmic ratio (NCR). Values expressed as mean ± SD. Mann–Whitney and ROC curves (threshold *p* < 0.05).

**Results:** 32 specimens with normal Pap test, 1,333 cells analyzed with DHM. 21 specimens with abnormal Pap test, 494 cells analyzed. For the three observers or each observer whatever criterion a significant difference between normal and abnormal specimens observed (except for MHN in PCA *p* = 0.56. Areas under the ROC curves (95 % CI): 0.82[0.79–0.84] for NHN, 0.85[0.82–0.87] for MHN, 0.94[0.93–0.96] for ND, 0.97[0.96–0.98] for NCR.

**Conclusion:** The criteria analyzed with DHM had good area under ROC. Large scale validation study will provide clear cut automated delineation of normal and abnormal cells without adulteration of fixed cells.

**OFP-15-004**

**Preliminary validation of a liquid based cytology technique (kabcyt) using conventional screening and imaging system**

H. Sevestre ^*^, T. Brun, B. Karkouche

^*^CHU, Pathology, Amiens, France

**Objective:** To describe the evaluation of a liquid based cytology technique (Kabcyt): confection of slides with Thinprep residual material, comparative screening by pathologists, conventional and assisted by the Thinprep Imaging System (TIS, Hologic).

**Method:** After Thinprep (TP) technique and screening, conventional and assisted by TIS, the residual material of 95 consecutive patients (excluding ASC-US and ASC-H) was used to perform the Kabcyt (KB) technique. KB slides were also double screened. The technique was performed in five rounds by a in-training medical student. The initial (TP) and second (KB) slides were classified separately by two pathologists according to Bethesda system.

**Results:** No difference of diagnostic categories observed in 92 pairs of cases (96.8 %). One pair: ASC-H and repair (TP) vs atrophic menopausal status (KB); on hysterectomy specimen: no cervical lesion. One pair: LSIL (TP) vs normal (KB). One pair: the reverse; after consensus evaluation, LSIL in these four slides. TIS evaluation was satisfactory in 100 % of TP and 74 % of KB slides.

**Conclusion:** The KB liquid based cytology method was succesful in this « real life» validation process. This manual technique was quickly learned by a medical student; it provided slides accepted by the TIS in most cases.

**OFP-15-005**

**Morpholgy versus flow cytometry of cerebrospinal fluids**

M. Lund-Iversen ^*^

^*^Oslo University Hospital, Dept. of Pathology, Norway

**Objective:** To compare morphological findings with flowcytometric phenotyping (FCP) of cells in cerebrospinal fluid (CSF).

**Method:** In 2010 we investigated, as part of rutine diagnostics, 165 CSF samples from 131 patients aged 7–86 years with the following clinical queries: Lymphoma [93 (56,3 %)], metastatic carcinoma [2 (1,2 %)], unspecific neurological or radiological findings [64 (38,8 %)] and none [6 (36,4 %)]. Within 4 h after collection, 100 μL were prepared as DiffQuick stained cytospin, and the remaining volume were phenotyped using antibodypanels according to morphological findings.

**Results:** Eight samples were excluded. Microscopy revealed malignant cells in 8 samples, 2 carcinomas and 6 lymphomas. Ep4+ cells and pathological lymphoid phenotype confirmed morphological findings respectively. Five samples contained monoclonal B-cells consistent with low grade B-cell lymphomas, and 8 samples had light chain restriction of uncertain nature. Atypical cells were not found in these samples.

**Conclusion:** Microscopy of cells in CSF can confirm cerebromeningeal malignant affection, but FCP is superior to microscopic evaluation in detection of cerebromeningeal affection of low grade lymphomas. FCP is both a confirmatory supplement and an independent diagnostic tool, but one have to be aware of false positivity due to blood contamination, as well as the fact that clonal expansion not necessarily is consistent with hematological neoplasia.

**OFP-15-006**

**Multiprobe FISH for the elucidation of equivocal pancreatobiliary cytology**

T. Vlajnic ^*^, G. Somaini, S. Savic, A. Barascud, L. Degen, E. Obermann, L. Bubendorf

^*^University Hospital Basel, Institute of Pathology, Switzerland

**Objective:** Endoscopic fine-needle aspiration is the standard method for the diagnosis of tumors of the pancreatobiliary tract. We explored the utility of FISH-improved diagnostic stratification between reactive and malignant cells in cases of equivocal cytological atypia.

**Method:** The multiprobe FISH assay UroVysion was used for copy number enumeration of chromosomes 3, 7, 17, and the 9p21 locus and applied to Papanicolaou-stained specimens with a diagnosis of equivocal atypia (*n* = 48), adenocarcinoma (*n* = 31), or no evidence of malignancy (*n* = 14). We captured images of the atypical cells and saved the coordinates on an automated stage prior to hybridization. A positive test was defined as increased copy number (>2) of at least 2 chromosomes or homo-/heterozygous loss of 9p21.

**Results:** FISH confirmed all 31 cytological diagnoses of pancreatobiliary adenocarcinomas and was negative in all 12 patients with no clinical evidence of malignancy. Among the 52 cases with equivocal atypia FISH detected 19/33 cases with a final diagnosis of adenocarcinoma and was negative in all 19 cases with no final evidence of malignancy (sensitivity 58 %, specificity 100 %, PPV 100 %, NPV 56 %).

**Conclusion:** Multiprobe FISH combined with automated relocation of atypical cells is a powerful technique to distinguish between reactive atypia and malignancy in pancreatobiliary cytology.

**OFP-15-007**

**Robinson’s cytological grading and correlation with Bloom Richardson histological grading on touch imprint cytology from core needle breast biopsy**

B. Babic ^*^, A. Guzijan

^*^Clinical Center Banja Luka, Pathology, Bosnia and Herzegovina

**Objective:** Cytological grading of malignant breast tumors is a useful method for making decision about the therapy and prognosis.

**Method:** 37 patients with breast cancer are cytological graded based on 6 morphological parameters by Robinson method: uniformity and size of the cells, nuclei membrane and chromatin, cell discohesion and nucleolus. For all of them, correlation was made with histological grade by Bloom Richardson methods. Cytological samples are performed by touch imprint cytology from core needle biopsy.

**Results:** Most of malignant tumors are cytological grade 2, 19 cases and the least grade 1,7 cases. Grade 3 is found in 11 cases. Histological grade 2 is found in 28 cases and grade 3 in 3 cases. Histological grade 1 is found in 6 cases. In grade 1 correlation with histological grade is nearly perfect. Discrepancy was found in only 1 case. Disagreement between grade 2 and 3 is found in 13 cases. Concordance between this two methods was 62,7 %.

**Conclusion:** Nuclei grading on cytological samples correlates with histological grading. Cytological grade is an essential predictive factor for histological grade and in combination with other diagnostic methods can give us useful data about size, grade and type of the tumor.

**OFP-15-008**

**Reliability of flow cytometric analysis on cytological samples in subclassification of small B cell lymphomas**

U. Klopcic ^*^, V. Kloboves Prevodnik, J. Lavrencak

^*^Institute of Pathology, Dept. of Cytopathology, Ljubljana, Slovenia

**Objective:** To establish if typical patterns of expression of antibodies CD10, CD5, CD23 and FMC7 enable correct classification of small B cell lymphomas (SBCL) by flow cytometry.

**Method:** We analysed flow cytometric measurments of 157 fine needle aspiration samples of primary SBCL: 19 marginal zone lymphomas (MZL), 55 follicular lymphomas (FL), 30 mantle cell lymphomas (MCL), and 53 chronic lymphocytic leukaemia/small lymphocytic lymphomas (CLL/SLL). Typical antigen patterns for differentiation of different types of SBCL were: FL (CD10+, CD5−, CD23−), MZL (CD10−, CD5−, CD23−), MCL (CD5+, CD10−, CD23−) and CLL/SLL (CD5+, CD23+, FMC7−).

**Results:** 88 % of SBCL in our series had typical immunophenotype (96 % of CLL/SLL, 63 % of MZL, 100 % of low grade FL, 76 % of high grade FL, and 93 % of MCL). The results are summarized in the Table 1.

**Conclusion:** Flow cytometry of FNAB samples enables precise classification in the great majority of SBCL. Correct classification is problematic in CD5+ MZLs, since the same immunophenotype can also be obtained in some CLL/SLLs and MCLs. Furthermore, the diagnosis of FL is difficult in CD10- cases, where immunophenotype overlaps with MZL. When morphology does not correlate with immunophenotype, and in doubtful cases, additional immunocytochemical markers (e.g. Cyclin D1, Bcl-2, Bcl-6) and/or molecular techniques for detecting cytogenetic abnormalities should be used to increase the accuracy rate.

**Table 1:**

**Table:** 

	CD10+	CD10−	CD5+	CD5−	CD23+	CD23−	FMC7+	FMC7−
MZL	0	19	6	13	2	17	8	11
FL1	12	0	0	12	2	10	6	6
FL2	22	0	0	22	6	16	16	6
FL3	17	4	0	21	4	17	15	6
MCL	0	30	28	2	0	30	25	5
CLL/SLL	0	53	53	0	52	1	1	52

**OFP-15-009**

**Lymphoplasmacytic lymphoma: A difficult diagnosis in lymph node fine needle aspiration cytology**

P. Luís ^*^, A. Alves, D. Lopéz-Presa, C. Ferreira

^*^Hospital de Santa Maria, Dept. de Anatomia Patológica, Lisboa, Portugal

**Objective:** Fine needle aspiration cytology (FNAC) is a powerful tool in the diagnosis of many conditions in lymph nodes it can be quite challenging to make a definitive diagnosis.

**Method:** We present a case of a 55-year-old male, with history of tuberculosis in 2011, which had recent cervical lymph node enlargement, asthenia and sweating. A FNAC of a cervical lymph node was made, and material was collected for smears and culture. Because the smears raised suspicion, FNAC was repeated, and material was collected for cell block and flow cytometry.

**Results:** The smears revealed a polymorphous population, with predominant small to intermediate sized lymphoid cells, some of them with plasmacytoid morphology, plasma cells with Dutcher bodies and histiocytes; no morphological features of tuberculosis were found. Flow cytometry identified a 68 % lymphoid population with a phenotype compatible with mature B-cell neoplasm. In cell block the lymphoid population showed immunoreactivity for CD20 and bcl-2 and negativity for CD3, CD5, CD10, CD23, CD43 and cyclin D1; the plasmacytic cells were monoclonal with restriction to kappa light chain and expressed cytoplasmatic IgM. Final diagnosis was Lymphoplasmacytic lymphoma, posteriorly confirmed by lymph node biopsy.

**Conclusion:** Lymphoma characterization by FNAC is sometimes difficult, but keeping in mind all the technical possibilities with FNAC material, more precise diagnoses can be made.

**Cell Block H.E. 400×:** 
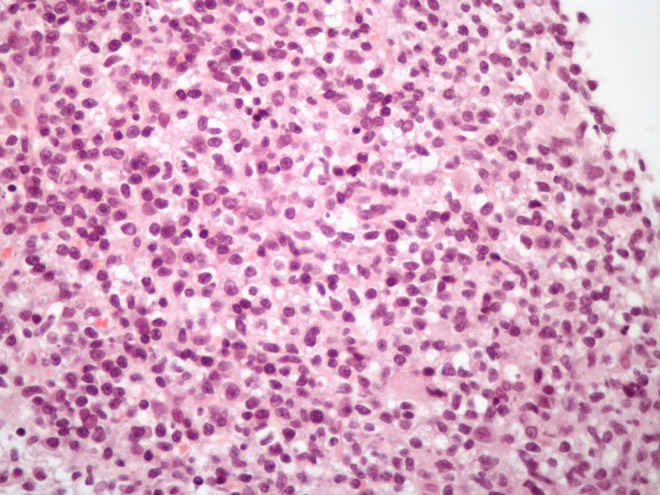


Wednesday, 4 September 2013, 08.30–12.00, Room 5C

**OFP-16 Oral Free Paper Session Joint Session—Pulmonary/Thymic and Mediastinal Pathology**

**OFP-16-001**

**Hyaluronidase, hyaluronan synthase, E-cadherin and TGF-ß profile in lung adenocarcinoma subtypes and squamous cell carcinoma of smokers/nonsmokers**

V. de Sá ^*^, L. Carvalho, A. Alarcão, P. Couceiro, V. Sousa, V. Capelozzi

^*^Faculdade de Madicina, Dept. of Pathology, São Paulo, Brazil

**Objective:** We examined Hyal1 and 3, HAS1,2 and 3, e-cadherin and TGF-β profile in lung adenocarcinoma (AD) subtypes and squamous cell carcinoma (SqCC) of smokers/nonsmokers.

**Method:** Fifty-six patients, mean age 64 years, who underwent lobectomy for AD (*n* = 31) and SqCC (*n* = 21) were included in the study. We used immunohistochemistry and morphometry to evaluate Hyal, HAS, e-cadherin and TGF-β expression.

**Results:** SqCC expressed Hyal3 predominant by stromal cells, whereas acinar predominant AD expressed strongly HAS3. In situ, papillary and solid patterns presented a weak expression of the immunomarkers. A positive association was found between smoking history and increased HAS1, HAS3 and Hyal1, while predominant acinar AD were negatively associated to HAS1, HAS3 and Hyal1. The following positive associations were found: TGF-β vs E-cadherin in stroma, TGF-β in tumor vs HAS2. Negative correlation occurred between TGF-β and HAS3 vs Hyal1, TGF-β vs HAS3 and Hyal1. E-cadherin was more frequent in the samples without lymphonode metastasis.

**Conclusion:** A profile of Hyal, HAS, E-cadherin and TGF-β has impact on AD subtypes and SqCC of smokers/nonsmokers, appearing as promise markers to guide treatment options.

**OFP-16-002**

**Detection of IGF1R in non-small cell lung carcinoma**

G. Vlacic ^*^, I. Kern

^*^University Clinic of Golnik, Dept. of Pathology and Cytology, Slovenia

**Objective:** The purpose of this study was to determine the presence of insulin-like growth factor-1 receptor (IGF1R) in tumour cells of non-small cell lung cancers (NSCLC).

**Method:** We collected 225 cases of NSCLC and used the paraffin-embedded blocks to perform immunohistochemistry to determine the expression of IGF1R and dual silver in situ hybridization (SISH) to quantitatively detect IGF1R genomic gain. IHC was assessed as positive or negative, SISH was analyzed in 40 nuclei and was regarded as positive if the ratio between IGF1R gene copy numbers and CEP 15 was higher than 2.

**Results:** In 34 cases there was not enough material to perform the study. There were 102 adenocarcinomas, 67 squamous cell carcinomas (SCC) and 22 NSCLC NOS. SISH was performed on 38 cases of which 13 were adenocarcinomas, 18 squamous cell carcinomas and 7 NSCLC NOS. Immunohistochemically we detected positivity in 85,1 % of SCC, in 79,4 % of adenocarcinomas and in 63,6 % of NSCLC NOS which gave us the overall result of 79,1 %. Gene amplification was detected in only 10,5 % of cases which were all SCC.

**Conclusion:** The expression of IGF1R in tumour cells of NSCLC can be detected in the majority of cases while gene amplification is rare.

**OFP-16-003**

**ALK rearrangement, EGFR, KRAS mutations and MET amplification are not exclusive in bronchial-pulmonary adenocarcinomas**

L. Carvalho ^*^, M. Silva, A. Alarcão, T. Ferreira, M. J. d’Aguiar, A. F. Ladeirinha, V. Sousa

^*^Faculty of Medicine Coimbra, Inst. of Anatomical Pathology, Portugal

**Objective:** ALK encodes a tyrosine kinase receptor and its rearrangement-ALK+was found in 2–7 % of lung carcinomas, resulting a constitutively active and oncogenic protein reported as exclusive of EGFR and KRAS mutations and associated with resistance to EGFR inhibitors but highly sensitive to treatment with ALK inhibitor crizotinib.

**Method:** Sections of 126 bronchial-pulmonary carcinomas surgical specimens of all histological types were screened for ALK positivity by FISH with break-apart dual color probe (Abbott) and immunohistochemistry(IHC)–monoclonal antibody clone 5A4 (Leica); EGFR and KRAS mutations were determined by DNA direct sequencing and EGFR and MET genes amplification, by FISH (Abbott).

**Results:** IHC was applied in FISH ALK+cases and of the 126 screened tumors, 9 adenocarcinomas (7 %) were FISH ALK +, 3+ and 2+ score in IHQ. Among these 9 FISH-ALK+cases, 3 had EGFR mutations and 2 had both EGFR and MET gene amplifications in FISH; KRAS was wild type.

**Conclusion:** IHQ correlates with FISH for ALK gene rearrangement, not excluding EGFR and MET alterations. ALK status has to be tested in advanced bronchial-pulmonary adenocarcinoma, as it could be responsible for TKI-resistence of EGFR mutated tumors that benefit from ALK-targeted agents. EGFR mutations search revealed again to be necessary in this context of targeted therapy.

**OFP-16-004**

**p53 mutated protein screened by p53 Ab-1 (Clone PAb 240) may be reliable to mutations search and targeted therapy in bronchial-pulmonary carcinomas**

L. Carvalho ^*^, A. Alarcão, P. Mota, J. R. Costa, J. Fonseca, A. Ladeirinha, T. Ferreira, M. J. d’Aguiar

^*^Faculty of Medicine Coimbra, Inst. of Anatomical Pathology, Portugal

**Objective:** DO-7 expression in neoplasias relates with p53 overexpression/non-functional protein synthesis; also p53 Ab-1-clone PAb 240-identifies mutant p53 protein in Pathology routine.

**Method:** DO-7 and PAb 240 were applied to 48 Bronchchial-Pulmonary Carcinoma sections: Adenocarcinomas(ADC)-31biopsies/17surgical specimens(SS); 53 Epidermoid Carcinomas(EC) -44 biopsies/9SS; 21 Small Cell Lung Carcinomas(SCLC)-biopsies and 26 cases of Polymorphic Carcinomas(PC)-Large Cell, Basaloid and Pleomorphic Carcinomas, Adenosquamous Carcinomas and Carcinomas CK7+/CK5.6+ 21 biopsies/5SS.

**Results:** Strict linear behaviour for PAb 240-positivity/DO-7positivity and negativity/positive–negative DO-7, grosso modo in the common score of 0, 1(<10 %), 2(10–50 %) and 3(>50 %) positive nuclei–DO-7 3/mutant p53 from 1 to 3: ADC: 21/48 positive, EC 28/53, SCLC 8/21 and PC 14/26 cases. DO-7 negativity/positivity together with mutant p53 protein negative expression results were similar: 27/48, 25/53, 13/21 and 12/26 respectively.

**Conclusion:** It is the first time to the best of our knowledge: PAb 240 was applied to BPC with DO-7 positivity and clear negativity independent of DO-7positive/negative expression and BPC typing, ready to be used isolated in routine to support trials of P53 mutations and targeted therapy.

**OFP-16-005**

**Membranous Filamin A protein expression is associated with poor overall survival in patients with non-small-cell lung cancer**

M. Gachechiladze ^*^, V. Kolek, J. Klein, L. Radova, Z. Kolek, J. Skarda

^*^Palacky University, Clin. and Molec. Pathology, Olomouc, Czech Republic

**Objective:** An actin-binding protein Filamin A (FLNA) connects the actin filament network to cell membrane receptors, and acts as a scaffold for various signaling pathways related to cancer growth and progression. Different cellular localization of FLNA protein, detected by immunohistochemistry, has been shown to have a different clinical significance. We aimed to investigate the correlation between differential expression of FLNA and clinicopathological factors in patients with non-small-cell lung cancer (NSCLC).

**Method:** We performed FLNA protein immunohistochemistry on FFPE tissue samples from 82 NSCLC patients, using EP2405Y antibody against C-terminus of FLNA (LSBio). Cytoplasmic, membranous and nuclear staining were evaluated semi-quantitatively and correlated with all available clinicopathological factors.

**Results:** Cox regression analysis of survival showed that overexpression of membranous FLNA negatively correlates with overall survival (HR = 0,9,972; 95 % CI (0,9945; 0,9998); *P* < 0,05), as well as disease free survival (HR = 0,9976; 95 % CI (0,9951; 1); *P* < 0,06) in all stages of NSCLC. No significant correlations were found between FLNA cytoplasmic and/or nuclear expression and other clinicopathological factors.

**Conclusion:** According to our study results FLNA membranous positivity might serve as useful prognostic marker in patients with NSCLC, although more extended study is necessary.

**OFP-16-006**

**Simultaneous KRAS and EGFR mutations: A single laboratory’s experience in 1,384 lung adenocarcinoma samples**

K. Éles ^*^, E. Tóth, J. Szoke

^*^National Institute of Oncology, Surgical & Molecular Pathology, Budapest, Hungary

**Objective:** KRAS mutation occurs in approximately 20–35 % and EGFR mutation occurs in 5–10 % of NSCLC. Previous reports have shown that EGFR and KRAS mutation are mutually exclusive. Our aim was investigate the frequency of simultaneous mutation of these two genes in lung adenocarcinoma.

**Method:** KRAS exon 2 and EGFR exon 19,21 mutations were tested on 1,384 lung adenocarcinoma samples between 2008 and 2012. The mutation analysis was performed by real- time PCR followed by Sanger sequencing of the EGFR exon 19,21 and KRAS exon 2 genes.

**Results:** KRAS mutations were detected in 36,4 % EGFR mutations were detected in 9 % of adenocarcinomas. Double mutations were found in 10 cases of 1384 patient (0,7 %). 7 patients had EGFR 19 exon deletion and 3 had EGFR exon 21 point mutation.

**Conclusion:** Our results show that KRAS mutation may coexist with EGFR mutation. There are few reports in the literature about concomitant EGFR and KRAS mutation in lung adenocarcinoma. It is important to use sensitive detection methods, as data shows that minor KRAS mutant tumour cell population may influence negatively the therapeutic effect of EGFR inhibitors even in the presence of tyrosine-kinase inhibitor sensitive mutations. Concomitant EGFR and KRAS mutation are most propably related to the multiclonal character of the tumour.

**OFP-16-007**

**Prognostic significance of p63, TTF-1 and maspin in non-small cell lung carcinomas**

B. Yaman ^*^, D. Nart, G. Çok, A. Veral

^*^Ege University Medical Faculty, Pathology, Izmir, Turkey

**Objective:** Antiapoptotic genes and protease inhibitors take an important role in lung cancers.

**Method:** 80 patients with non-small cell lung cancer were selected. Tumour characteristics, immunoreactivity with p63, TTF-1, maspin, and follow up datas were evaluated.

**Results:** Patients were predominantly male (*n* = 71, 88.8 %) and mean age was 59. Fourtyfive (56.3 %) tumours were adenocarcinoma (AC), 23 (28.8 %) squamous cell carcinoma (SCC), four (5 %) large cell carcinoma (LCC), six (7.5 %) large cell neuroendocrine carcinoma (LCNC), and two the others. The mean diameter of tumours was 4.06 cm (1.5–11). Patients with advanced TNM stage and ≥3 cm tumour had poor survival (*p* < 0.005). Immunohistochemically 87.5 % of SCCs, 4.3 % of ACs, 25 % of LCCs, and 16.7 % of LCNCs stained with p63 (*p* < 0.0001). The positivity with maspin in SCC was 66.7 %, and in AC 17.4 % (*p* < 0.0001). Immunoreactivity of TTF-1 in ACs was 84.8 %, whereas no staining in SCCs (*p* < 0.0001). The negativity of maspin in ACs has a positive prognostic effect (*p* = 0.048).

**Conclusion:** The results of this study indicate that p63 and TTF-1 are useful markers in differential diagnosis of SCC and AC, but they have no significance in survival. And maspin can be used as a prognostic marker in ACs.

**OFP-16-008**

**Has subtyping of mucinous adenocarcinomas of the lung any significance?**

H. Popper ^*^, U. Gruber-Moesenbacher, C. Manzl, A. Geles

^*^Medizin. Universität Graz, Inst. für Pathologie, Mol. Lung und Pleura Pathologie, Austria

**Objective:** Adenocarcinomas (AC) of the lung were reclassified in 2011, but mucinous ACs were all lumped together into one category. Mucinous AC can also be subtyped for predominant morphological pattern. This might have implications for molecular markers and subsequently for targeted therapy.

**Method:** We retrieved 76 mucinous pulmonary ACs from our biobank. KRAS mutation analysis was done in all cases. A TMA was created for molecular analysis, especially KRAS signaling pathways RAS-MAPK-ERK, RAS-RAL, and RAS-PI3K-AKT-mTOR. The type of mucin produced by the tumors was also analyzed and categorized. Clinical data were available for all patients.

**Results:** The majority of mucinous ACs presented as either predominant acinar or papillary variants, micropapillary was often combined with papillary, cribriform was associated with acinar pattern. Other variants were solid, signet ring cell, and colloid components, whereas pure forms of colloid, signet ring cell and micropapillary ACs were rare. Half of the cases showed mutation of the KRAS gene in codons 12, 13, and 61, double mutations did occur. KRAS mutation did not correlate with any of the subtypes. However, progression free survival correlated with the subtypes.

**Conclusion:** Subtyping of mucinous AC is encouraged, and might open new insights into the molecular signaling pathways of this group of AC.

**OFP-16-010**

**KRAS, EGFR, PDGFR-alpha, KIT and COX 2 status in Carcinoma Showing Thymus-like Elements (CASTLE)**

L. Veits ^*^, P. Hufnagel, R. Penzel, P. Ströbel, K. Kaserer, S. Schröder, N. Neuhold, K. W. Schmid, P. Schirmacher, A. Hartmann, R. J. Rieker

^*^Klinikum Bayreuth, Abt. Pathologie, Germany

**Objective:** CASTLE is a rare malignant neoplasm of the thyroid resembling lymphoepithelioma-like and squamous cell carcinoma of the thymus with different biological behaviour and a prognosis superior to anaplastic carcinoma of the thyroid. We retrospectively investigated 6 cases of this very rare neoplasm in order to investigate the mutational status of KRAS, EGFR, PDGFR-α and KIT, as well as the immunohistochemical expression pattern of CD117, EGFR and COX 2.

**Method:** The material was investigated using immunohistochemical staining as well as mutational analysis of EGFR, PDGFR, KIT and KRAS. Diagnosis was confirmed by a moderate to strong expression of CD5, CD117 and CK5/6, whereas Thyreoglobulin, Calcitonin and TTF-1 were negative in all cases.

**Results:** COX 2 expression was moderate to strong in almost all cases. In four cases single nucleotide polymorphisms (SNPs) could be detected in exon 12 of the PDGFR-α gene (rs1873778), in three cases SNPs were found in exon 20 of the EGFR gene (rs1050171). No mutations were found in the KIT and KRAS gene.

**Conclusion:** Nearly all tumors showed a moderate to strong COX 2 expression and a wild-type KRAS status. No activating mutations in the EGFR, KIT and PDGFR-α gene could be detected. Our data may indicate a potential for targeted therapies, but if these therapeutic strategies are of benefit in CASTLE remains elusive.

**OFP-16-011**

**Thymoma with molecularly verified “conversion” into T lymphoblastic leukemia/lymphoma over 9 years**

V. D. Ertel ^*^, M. Früh, A. Guenther, T. Cerny, C. Fretz, S. Cogliatti

^*^Cantonal Hospital, Pathology, St. Gallen, Switzerland

**Objective:** Thymoma is an epithelial tumor accompanied by non-neoplastic lymphocytes.

**Method:** Histological and molecular analysis of thymoma in a 62-year old male patient during a 9 years course.

**Results:** Initially a combinded B1/B2 thymoma was diagnosed in Masaoka stage IVa based on morphologic and immunophenotypic features. Over the years, suffering from recurrent disease, the patient developed combined B2/B3 thymoma. With sudden onset after 9 years, the disease switched dramatically to aggressive dynamics, when rebiopsies revealed at that time a T lymphoblastic leukemia/lymphoma. After dismal terminal course, the patient died within a few months by physical exhaustion. For verification of our hypothesis of secondary development of a precursor T lymphoblastic neoplasm following thymoma, we performed PCR analyses that revealed a clonal rearrangement of the T-cell receptor-gamma_genes in the V-gamma_10–12 and a biclonal peak in the V-gamma_1–8 primer setting. When we retrospectively analysed thymoma specimens, we found an identical biclonal peak in V-gamma 1–8 in combined B2/B3 thymoma and a polyclonal pattern in all previous tissue samples.

**Conclusion:** Our results support the assumption of a transforming process of polyclonality to oligoclonality in non-neoplastic cortical thymocytes in combined B1/B2 thymoma and B2/B3 thymoma, respectively and finally to monoclonality in secondary T lymphoblastic leukemia/lymphoma.

Wednesday, 4 September 2013, 14.00–16.00, Auditorium VI

**OFP-17 Oral Free Paper Session Haematopathology**

**OFP-17-001**

**The diagnostic value of miR17-92 microRNA cluster in diffuse large B-cell lymphoma**

A. Fassina ^*^, R. Cappellesso, F. Marino, M. Siri, F. Simonato, M. Benetti, M. Crescenzi, M. Trento, L. Ventura, M. Fassan

^*^University of Padua, Dept. of Medicine, Padova, Italy

**Objective:** Diffuse large B cell lymphoma (DLBCL) can present as de novo or can arise through the transformation of indolent lymphomas, including follicular lymphoma (FL). The morphological differentiation between germinal center-DLBCL (GC-DLBCL) and high grade (grade 3) FL could be challenging: new diagnostic tools are needed to assure the clinical-pathological follow-up of these patients. Recent expression profiling studies reported microRNAs (and miR17-92 cluster, in particular) as useful tools in differentiating DLBCL and FL.

**Method:** We investigated the expression profile of six members of the miR17-92 cluster (i.e., miR18b, miR19b, miR20a, miR92, miR93, and miR106a) by quantitative reverse transcription-polymerase chain reaction in 36 cases of GC-DLBCL and 18 cases of high-grade non-transforming FL, confirmed on clinical, histological, and immunohistochemical data.

**Results:** All the considered miR17-92 cluster miRNAs were significantly over-expressed in GC-DLBCL. The ROC estimated thresholds miR17-92 cluster miRNAs displayed a sensitivity level higher than 0.80 in achieving the GC-DLBCL diagnosis. The classification tree built on the six thresholds allowed the correct identification of 35/36 GC-DLBCL (97.2 %).

**Conclusion:** The miR17-92 cluster can represent a reliable, standardizable diagnostic tool for the sub-classification of large B cell lymphoid neoplasm for differentiating GC-DLBCL from high grade FL.

**OFP-17-002**

**The alteration of lipid metabolism in Burkitt lymphoma identifies a novel marker: Adipophilin**

G. De Falco ^*^, M. R. Ambrosio, P. P. Piccaluga, C. Doglioni, B. J. Rocca, M. Onorati, V. Malagnino, K. Naresh, S. Pileri, L. Leoncini, S. Lazzi

^*^University of Siena, Dept. of Medical Biotechnology, Italy

**Objective:** Recent evidence suggests that lipid pathway is altered in many human tumours. In Burkitt lymphoma this is reflected by the presence of lipid droplets which are visible in the cytoplasm of neoplastic cells in cytological preparations. These vacuoles are not identifiable in biopsy section as lipids are lost during tissue processing.

**Method:** We investigated the expression of genes involved in lipid metabolism, at both RNA and protein level in BL and in other B-cell aggressive lymphoma cases.

**Results:** Gene expression profile indicated a significant over-expression of the adipophilin gene and marked up-regulation of other genes (FASN, SCD5, USF1) involved in lipid metabolism in BL. These findings were confirmed by immunohistochemistry on a series of additional histological samples: 45 out of 47 BL cases showed strong adipophilin expression, while only 3 out of 33 not-BL category showed weak adipophilin expression.

**Conclusion:** Our preliminary results suggest that lipid metabolism is altered in BL, and this leads to the accumulation of lipid vacuoles. These vacuoles may be specifically recognized by a monoclonal antibody against adipophilin, which may be a useful marker for BL because of its peculiar expression pattern. Moreover this peptide might represent an interesting candidate for interventional strategies.

**OFP-17-003**

**Lymphoplasmacytic myeloma: A rare variant of plasma cell myeloma. A study of 14 cases**

M. Fameli^*^, A. Tasidou, E. Parasi, S. Giannouli, N. Stavrogianni, E. Stefanoudaki, G. Kokkini, E. Terpos, M. A. Dimopoulos, T. Papadaki, L. Marinos

^*^Evaggelismos, Hematopathology, Athens, Greece

**Objective:** Recently, two subgroups of cyclin D1+ plasmacytic myeloma (MM) are recognized: conventional (C-MM) and lymphoplasmacytic (LPL-MM) MM. LPL-MM is characterized morphologically by small plasma cells mimicking lymphoplasmocytoid lymphocytes and co-expression of both plasma cell (CD138) and B-cell (CD20, PAX-5) markers.

**Method:** 14 Bone Marrow Trephines (BMT) from 14 patients with cyclin D1+ (first diagnosis), with morphological and immunohistochemical (IM) features of LPL-MM. BMT study: morphology (Ε), IM: broad antibody panel.

**Results:** Among 686 new MM cases we identified 182/686 cyclin D1+ (~26,5 %). LPL-MM subgroup comprised ~2,04 % (14/686) of total MM cases and ~7.6 %(14/182) of cyclin D1+ cases (Table 1).

**Conclusion:** LPL-MM represents a rare MM subtype (2,04 % of new MM cases) and is characterized by Almost exclusive male predominance (13/14), “bipolar” (<30 % & >80 %) percentage of infiltration Exlusively γ-heavy chain and predominantly λ-light chain restriction. Difficulties in the differential diagnosis from lymphoplasmacytic lymphoma (2/14 cases had been previously diagnosed as such).

**Table 1. Results:**

**Table:** 

M/F	age	% infiltration		CD56+	CD20+	PAX-5+	Light chain only	Total κ/λ	Heavy chain
13/1	71.25	<30 %	5/14	2/14	100 %	14/14	6/14	5/9	γ = 8/14
	57–83	>80 %	8/14		9/14		κ = 1, λ = 5		α = 0
		fibrosis	1/14		60–90 %				
					5/14				

**OFP-17-004**

**Primary mediastinal large B-cell lymphoma: Immunohistochemical features and differential diagnostics**

A. Artemyeva ^*^, G. Frank

^*^Gerzen Institute, Dept. of Pathology, Moscow, Russia

**Objective:** Primary mediastinal large B-cell lymphoma (PMBL) may share common morphological and phenotypical features with mediastinal grey zone lymphoma (MGZL), Hodgkin lymphoma (HL) and diffuse large B-cell lymphoma (DLBL) with mediastinal involvement. Their differential diagnosis might represent a challenge requiring a specific immunohistochemical (IHC) analyses.

**Method:** Formalin-fixed paraffin-embedded tissue by 58 cases PMBL, 10 cases MGZL, 17–HL and 13–DLBL were examined for H&E staining and LCA, CD20, CD3, CD15, CD30, CD10, CD23, EMA, ALK, PAX-5, BOB.1, OCT-2, mum 1, p65, MAL, Ki67 IHC expression. The final diagnosis was established according to the WHO classification criteria.

**Results:** All cases were diagnosed as PMBL showing strong positive reaction with PAX-5, BOB.1 and OCT-2, 26 (45 %)–p65 nuclear expression, 40 (69 %)–exspressed CD23, 2 (3.4 %)–CD10, 39 (67 %) were MAL-positive. In cases of MGZL 8/10 showing CD23, PAX-5, BOB.1, OCT-2 and MAL expression, p65–only cytoplasmic staining. In 1 HL case was observed staining with MAL.

**Conclusion:** Only complex usage of such IHC markers like transcriptional factors (PAX-5, BOB.1, OCT-2), membrane associated protein MAL and p65 enables to give the diagnosis of PMBL. Estimation of p65 true nuclear expression complicated by background cytoplasmic staining, that restricted this marker usage.

**OFP-17-005**

**Combined inhibition of BCR/ABL- and CD27-signaling eradicates chronic myelogenous leukemia stem cells**

C. Schürch ^*^, C. Riether, A. Ochsenbein

^*^Medizin. Universität Bern, Inst. für Pathologie, Switzerland

**Objective:** Chronic myelogenous leukemia (CML) originates from leukemia stem cells (LSCs) harboring the oncogenic BCR/ABL tyrosine kinase. Tyrosine kinase inhibitors (TKIs) have revolutionized CML therapy; however, definite cure remains unachievable. LSCs are resistant to TKIs and there is a significant risk of disease progression to acute leukemia. Consequently, future therapies must aim at the elimination of LSCs. We recently documented in a murine CML model that LSCs express the costimulatory molecule CD27. Blocking CD27 on LSCs inhibited LSC proliferation and attenuated disease progression. Here, we investigated a combination treatment using TKIs and CD27-signaling blocking monoclonal antibody.

**Method:** Co-treatment was applied in vitro to BCR/ABL-expressing human leukemia cells and in vivo to mice harboring a CML-like disease induced by transplantation of BCR/ABL-transduced bone marrow.

**Results:** In vitro, co-treatment acted synergistically and significantly reduced leukemia cell growth compared to single treatments. In vivo, co-treatment significantly improved survival of CML mice and induced long-term cure in >50 % of animals, whereas mock- or single-treated animals all succumbed to CML. Analysis of bone marrow from CML mice and secondary transplantation experiments revealed that co-treatment specifically eradicated LSCs and leukemia progenitor cells.

**Conclusion:** Combining TKI treatment and blocking CD27-signaling may represent an attractive strategy to directly target LSCs in CML.

**OFP-17-006**

**Cytotoxic CD8+ T cells regulate myelopoiesis by cytokine signals to bone marrow stromal cells**

C. Schürch ^*^, C. Riether, A. Ochsenbein

^*^Medizin. Universität Bern, Inst. für Pathologie, Switzerland

**Objective:** Cytotoxic CD8+ T cells (CTLs) are crucial for protection against primary infection with intracellular pathogens. However, complete pathogen clearance and return to homeostasis requires a regulated interplay between innate and acquired immunity. CTL-secreted interferon-gamma (IFN-g) directly regulates innate immune cells. In addition, IFN-g has been shown to modulate hematopoiesis, but the exact mechanisms remain unclear.

**Method:** To assess the effect of CTL-secreted IFN-g on myelopoiesis, we either infected wild-type mice with lymphocytic choriomeningitis virus (LCMV) or adoptively transferred TCR-transgenic CTLs specific for LCMV-gp33 into mice ubiquitously expressing LCMV-gp33 on MHC class I. Hematopoietic stem (HSCs) and progenitor cells were analyzed at different time points.

**Results:** LCMV-infection or adoptive CTL transfer did not affect early HSCs but induced the proliferation of multipotent progenitors (MPPs), resulting in increased production of mature myeloid cells. IFN-g did not affect MPPs directly, but stimulated mesenchymal stromal cells of the bone marrow HSC niche to produce hematopoietic cytokines including interleukin-6.

**Conclusion:** Bone marrow stromal cells (BMSCs) have an important documented role in the regulation of HSC maintenance and quiescence. Our study now demonstrates that BMSCs are central in the regulation of myelopoiesis in response to infection and that CTLs exert an indirect positive feedback on myeloid progenitors during viral clearance.

**OFP-17-007**

**Retrospective description of previous biopsies in patients diagnosed of angioimmunoblastic T-cell lymphoma**

O. Balagué ^*^, D. de Jong

^*^NKI/AL, Pathology, Amsterdam, The Netherlands

**Objective:** Investigate the histopathological features of previous biopsies in patients diagnosed of angioimmunoblastic T-cell lymphoma(AITL)

**Method:** We have retrieved the 45 cases of AITL diagnosed between 2007 and 2012 in our region and selected those patients with previous biopsies to the diagnose of AITL. We revised the previous diagnoses and performed complementary analysis to investigate whether the lymphoma had been missed and why.

**Results:** 10 patients (22 %) had previous biopsies. The time between the first biopsy and the diagnose ranges from 3 to 36 months. AITL was suggested in 3 cases, but not concluding because of the presence of germinal centers,absence of dendritic expansion, lack of typical clinical features or clonality could not be demonstrated. Follicular hyperplasia was the diagnose in 4 cases (1 case with features of progressively transformed germinal centers). 3 patients were diagnosed of lymphoma (1 ATCL and 2 DLBCL). 9 of 10 cases were revised at the time of diagnose, 6 of them were then interpreted as AITL.

**Conclusion:** The diagnose of AITL is relatively often delayed. The absence of the typical clinial features, the presence of germinal centers and blastic B-cell populations seem to be reasons that difficult us an early diagnose.

**OFP-17-008**

**The therapeutic potential of targeted p53 activation by the MDM2-inhibitor nutlin-3a in anaplastic large cell lymphoma in relation to the ALK-expression status**

E. Drakos ^*^, V. Sinatkas, K. Psatha, A. Eliopoulos, H. Papadaki, G. Z. Rassidakis

^*^University of Crete, Medical School, Dept. of Pathology, Heraklion, Greece

**Objective:** p53 is rarely mutated in anaplastic large cell lymphoma (ALCL). We have shown that nutlin-3a, an MDM2-inhibitor, can induce cell cycle arrest and apoptosis in ALK+, ALCL cells harboring wt-p53. However, the therapeutic potential of nutlin-3a in ALK- ALCL, in comparison to ALK+ ALCL, has not been thoroughly studied.

**Method:** ALK+ ALCL (SUP-M2), and ALK-, ALCL (Mac2A and Mac1) cells harboring wt-p53, were treated with nutlin-3a. p53 activation and the biologic effects were investigated by western blot analysis, colony formation and MTT assays, trypan blue, annexin-V and PI staining. Also, combined treatments including mTOR, or BCL2-inhibitors were used.

**Results:** Nutlin-3a treatment induced activation of p53,and inhibition of growth and cell-cycle progession of both ALK+ and ALK−, ALCL cells. Nutlin-3a-induced apoptotic cell death of SUPM2 and Mac1 cells was accompanied by Puma upregulation. However, Mac2A cells, characterized by high levels of BCL2, were resistant to apoptosis. Combined treatment of Mac2A cells with nutlin-3a and the BCL2-inhibitor, YC-137, or rapamycin,or torin-2 resulted in dramatically decreased cell viability.

**Conclusion:** A subset of ALK- ALCL tumors, characterized by increased BCL2 expression levels, may be resistant to nutlin-induced apoptosis. Combined activation of p53 and BCL2, or mTOR inhibition may result in enhanced therapeutic potential in ALCL.

**OFP-17-009**

**HIV-related plasmablastic lymphoma of the head and neck: Proposal for a diagnostic algorithm**

S. Boy ^*^, P. Willem

^*^University of Pretoria, Oral Pathology and Oral Biology, South Africa

**Objective:** The literature concerning plasmablastic lymphoma (PBL) is incomplete and current knowledge mainly based on single case reports and small case series. This rare neoplasm is extensively investigated yet no specific diagnostic criteria currently exist. Distinguishing PBL from other high-grade B-cell and terminal B-cell neoplasms with plasmablastic features, especially in HIV/AIDS patients in third world circumstances is challenging and urges the development of a diagnostic algorithm. In South Africa more than 10 % of the population is HIV positive and PBL more commonly diagnosed than anywhere else in the world.

**Method:** Fifty eight cases diagnosed as PBL were subjected to a series of morphological and molecular investigations (Fish analysis: MYC BA, IGH BA, BCL6 BA, t(8: 14), t(11;14) and t(14;18); Immunohistochemistry: CD20, CD10, CD79a, Bcl6, CD45, CD38, CD138, IRF4/MUM1, PRDM1/BLIMP1, CD56, CD117, CCND1, Ki67, ALK, HHV8; In situ hybridisation: EBER, Immunoglobulin light chains). Available clinical features were noted. Results were compared to those of cases of high-grade B-cell lymphoma with plasmablastic differentiation and confirmed cases of multiple myeloma with plasmablastic morphology.

**Conclusion:** From the results we propose a diagnostic algorithm for HIV-related PBL to distinguish it from other high-grade B-cell lymphomas and plasma cell neoplasms with plasmablastic differentiation within the HIV setting.

**OFP-17-010**

**Pathological characteristics of 105 cases of HIV associated lymphoma included in the French ANRS CO16 LYMPHOVIR cohort study**

S. Prévot*, C. Besson, D. Costagliola, C. Chassagne-Clement, C. Delattre, B. Fabiani, C. Laurent, T. Lazure, B. Petit Jean, M. Raphaël

^*^Antoine Béclère Hospital, Pathology, Clamart Cedex, France

**Objective:** Introduction of combined antiretroviral therapy has reduced the incidence of NHL but not the incidence of HL in the HIV-infected population. This study analyzes the pathological characteristics of HIV-related lymphoma (L) collected in 4 years (2008–2012) in 32 centers among the prospective French national cohort of HIV-related L.

**Method:** Pathological material of 105 cases was reviewed according to the 2008 WHO classification with complementary techniques performed when necessary.

**Results:** 43 % were classical HL and 57 % B cell NHL. A predominance of mixed cellularity subtype HL was observed (33/45) and all but 3 were EBV positive. The distribution of the 60 NHL was: diffuse large B cell L 42 % (39 % EBV associated, 61 % of non GC subtype and 39 % of GC subtype), Burkitt L 28 %, plasmablastic L 12 %, marginal zone L 5 %, PTLD-like L 5 %, primary effusion L 5 %, anaplastic large cell L and unclassifiable because of necrosis (1 case of each).

**Conclusion:** This present study points out: (1) the high proportion of HL among HIV infection with L in the cART era, all of the classical type and the vast majority being EBV associated; (2) the heterogeneity of HIV-related NHL, all of B cell phenotype.

Wednesday, 4 September 2013, 14.00–16.00, Room 5B

**OFP-18 Oral Free Paper Session Digestive Diseases Pathology II**

**OFP-18-001**

**Limitations of the UICC/AJCC postoperative staging system for esophageal adenocarcinomas after neoadjuvant chemotherapy: Adding tumor regression grade enhances prognostic significance**

R. Langer ^*^, K. Becker, L. Sisic, M. Büchler, F. Lordick, J. Slotta-Huspenina, W. Weichert, H. Höfler, M. Feith, K. Ott

^*^Medizin. Universität Bern, Inst. für Pathologie, Switzerland

**Objective:** For tumors treated with neoadjuvant chemotherapy (CTX), postoperative staging classifications initially developed for non-pretreated malignancies may not accurately predict prognosis. We tested whether a TNM-based histopathologic prognostic score (PRSC), which additionally applies to tumor regression, may improve estimation of prognosis of esophageal adenocarcinomas treated by neoadjuvant chemotherapy, compared to the current UICC/AJCC TNM staging system (TNM7).

**Method:** 360 resection specimens were classified according to TNM7. For the PRSC, three factors were assigned a value from 1 to 3 (ypT0-2 = 1point; ypT3 = 2points; ypT4 = 3points; ypN0 = 1point; ypN1-2 = 2points; ypN3 = 3points; <10 % residual = 1point; 10–50 % residual tumor = 2points; >50 % residual tumor = 3points). TNM7 and the three-tiered PRSC basing on the sum value of these factors (group A: 3–4; group B: 5–7; group C: 8–9) were correlated with survival.

**Results:** Both TNM7 and PRSC showed highly significant prognostic impact (*p* < 0.001 each). However, TNM7 showed limitations especially in the lower staging groups while PRSC could accurately identify three prognostic different groups. Moreover, the PRSC showed better discrimination than TNM7 in multivariate analysis (for PRSC: *p* < 0.001; HR = 1.93; for UICC staging: *p* = 0.009; HR = 1.25).

**Conclusion:** The proposed PRSC clearly identifies subgroups with different outcomes and may be more helpful for guiding further therapeutic decisions than the current UICC/AJCC TNM staging system

**OFP-18-002**

**SYT and EWS abnormalities are recurrently occurring in poorly differentiated gastroesophageal cancers**

X. Sagaert ^*^, E. Van Cutsem, S. Tejpar, H. Prenen, P. Nafteux, K. Haustermans, T. Tousseyn, G. De Hertogh, S. Palmans, R. Sciot, M. Debiec-Rychter

^*^University Hospitals Leuven, Dept. of Pathology, Belgium

**Objective:** Being confronted with a case of a poorly differentiated gastroesophageal carcinoma with sarcoma-like features arising within high-grade Barrett-dysplasia, we investigated the immunohistochemical and molecular-genetic features in a (retrospective) series of this disease entity.

**Method:** 42 poorly differentiated gastroesophageal cancers were investigated by (1) immunohistochemistry for presence/absence of selected carcinoma/sarcoma markers (prekeratin, NCAM, vimentin, CD99) and by (2) FISH for presence/absence of abnormalities of the SYT and EWS genes, that are frequently rearranged in sarcomas. Twenty five well/moderately differentiated GE cancers were used as a control.

**Results:** 13/42 cases displayed an aberrant immunophenotype, with moderate/strong expression of NCAM, vimentin, and/or CD99. FISH did reveal a substantial number (14/42 = 33 %) of SYT/EWS-related genomic anomalies: 3 EWS-rearrangements, 3 SYT-rearrangements, 1 SYT and 1 EWS focal amplification, and 8 cases with SYT/EWS polyploidy. No immunohistochemical nor FISH abnormalities were found in the control group.

**Conclusion:** Our data show that SYT and EWS abnormalities are recurrently occurring in poorly differentiated gastroesophageal cancers. In addition, these abnormalities seemed to target cancers that are at risk of not responding well to the conventional chemoradiotherapy. Of interest, the one patient that started this study went into complete remission after being administered a sarcoma therapy, hence emphasizing the need for a prospective study.

**OFP-18-003**

**Resection margin status and lymph node yield in pancreaticoduodenectomy specimens at a single tertiary-care oncology centre**

M. Bal ^*^, M. Ramadwar, K. Deodhar, M. Goel, S. Arya, P. Patil, R. Engineer, S. Shrikhande

^*^Tata Memorial Hospital, Dept. of Pathology, Mumbai, India

**Objective:** To assess resection margin (RM) status and lymph node yield in pancreaticoduodenectomy (PD) specimens

**Method:** Data from all PD resections (2010–2011) were retrieved. A standardized pathology reporting protocol was used wherein transection [common bile duct (CBD), pancreatic neck (PN), proximal and distal enteric] and circumferential RMs (medial, retroperitoneal, posterior and anterior surfaces) were colour-coded and sampled. A 1 mm cut-off was defined as R1 (microscopic disease at margin).

**Results:** Study comprised 133 patients. Median age was 54 years. Tumours’ epicenter was in the periampullary (PA) region (88), pancreatic head (28), bile duct (5) and retroperitoneum (1); 11 had no tumour. Seven patients received neo-adjuvant chemo/radiotherapy (NACT/RT). R1 rate for pancreatic ductal adenocarcinoma (PDAC) and PA tumours was 71 % and 7 %, respectively; overall R1 rate was 17 %. R1 rate for posterior, retroperitoneal, PN, CBD, anterior, medial, proximal and distal enteric margins was 36 %, 30 %, 17 %, 9 %, 4 %, 4 %, 0 % and 0 %, respectively. Mean nodal yield was 13.6 (median, 11.5; range 0–34); following NACT/RT, mean nodal yield was 5.4.

**Conclusion:** Incidence of PA tumours was higher than PDAC in our PD specimens. R1 rate for PDAC resection was significantly greater than for PA tumours; posterior margin being the commonest involved. Neo-adjuvant therapy might influence nodal yield.

**OFP-18-004**

**Loss of Raf-1 Kinase Inhibitor Protein (RKIP) promotes Epithelial Mesenchymal Transition (EMT) and correlates with aggressive phenotype in Pancreatic Ductal Adenocarcinoma (PDAC)**

E. Karamitopoulou^*^, I. Zlobec, A. Lugli, B. Gloor, A. Perren

^*^University of Bern, Institute of Pathology, Switzerland

**Objective:** RKIP has emerged as a significant metastatic suppressor in a variety of human cancers and is known to inhibit the Ras/Raf/MEK/ERK signaling. By suppressing the activation of NFkB/SNAIL circuit, RKIP can regulate the induction of EMT. The aim of this study was to evaluate RKIP expression and to determine its association with clinic-pathological features, including EMT in form of tumor budding in PDAC.

**Method:** Staining for RKIP was performed on a multipunch Tissue Microarray (TMA) of 120 well-characterized PDACs with full clinico-pathological, follow-up and adjuvant therapy information. RKIP-expression was assessed separately in the main tumor body and in the tumor buds. Cut-off values were calculated by receiver operating characteristic curve analysis.

**Results:** RKIP expression was lost in 61.4 % of the PDACs and was significantly lower in the tumor buds compared to the main tumor body (*p* < 0.005). RKIP loss in the tumor was associated with higher tumor grade (*p* = 0.0389) and high-grade peritumoral budding (*p* = 0.0118). RKIP loss in the buds was associated with increased T stage (*p* = 0.035). No correlation with M- and N-stage or patient survival was found.

**Conclusion:** Loss of RKIP correlates with aggressive features in PDAC, especially characterized by the presence of EMT in form of tumor budding.

**OFP-18-005**

**Correlation of mitotic grade and Ki-67 index grade between histological and scrape cytological specimens in pancreatic neuroendocrine tumor**

K. Hirabayashi ^*^, G. Zamboni, G. Bogina, E. Bongiorno, L. Bortesi, P. Castelli, A. Pesci, N. Nakamura

^*^Tokai University, Dept. of Pathology, Isehara, Japan

**Objective:** The purpose of this study was to correlate mitotic grade (MG) and Ki67-index grade (KG) between histological and scrape cytological specimens in 61 resected primary pancreatic neuroendocrine tumors (P-NETs).

**Method:** Ki67 index was calculated as a percentage of 2000 cells in ‘hot spots’ on histological specimens and as a percentage of 100, 500, 1,000, and 2,000 cells in ‘hot spots’ on scrape cytological specimens. Mitotic count was calculated in the high-density area of mitoses per 10 high power fields by scanning 50 fields on histological specimens and 10, 20, or 50 fields on scrape cytological specimens. MG and KG were classified according to ENETS/WHO grading system.

**Results:** Kappa values of scrape cytological KG per 500, 1000, and 2000 cells showed a substantial agreement with histological KG, whereas KG per 100 cells only showed a fair agreement. Furthermore, when the cases were subdivided into KG1-2 and KG3, kappa value showed an almost perfect agreement. In contrast, kappa values of scrape cytological MG showed a fair agreement with histological MG.

**Conclusion:** Our results indicate that scrape cytological KG, determined in a small number of cells (e.g., 500 cells), accurately predicts histological KG, whereas scrape cytological MG does not predict histological MG.

**OFP-18-006**

**The CD8/CD45RO immunoscore in biopsy specimens of colorectal cancer predicts histopathologic features of the matched resection specimen and survival outcome**

V. Koelzer ^*^, I. Zlobec, H. Dawson, M. Borner, D. Inderbitzin, A. Lugli

^*^Universität Bern, Inst. für Pathologie, Switzerland

**Objective:** Reliable biomarkers based on pre-operative biopsies of colorectal cancer (CRC) are missing. We hypothesize that an immunoscore of CD8+ T-effector and CD45RO+ memory T-cell infiltrates in biopsies may predict histopathological features and survival outcome of CRC patients.

**Method:** Intraepithelial and stromal (s) CD8+ and CD45RO+ T-cells were quantified in 5 high-power-fields each of the highest density of infiltration in biopsy specimens of 130 well characterized CRC patients. Using Classification and Regression Tree (CART) analysis, CD8+ and CD45RO+ infiltrates in each zone were assessed for clinical relevance.

**Results:** High total (t) numbers of infiltrating CD8+ T-cells in biopsies strongly predicted the absence of nodal metastasis (*p* = 0.0182) and lymphatic invasion (*p* = 0.0201) in the matched resection specimen. High numbers of sCD45RO+ T-cells predicted earlier T-stage (*p* < 0.0001), lower tumor grade (*p* = 0.0223) and absence of distant metastasis (*p* = 0.0177). 100 % specificity and PPV for the prediction of pN was reached by combined analysis of tCD8 and tCD45RO. Strong immune infiltration in biopsies was highly prognostic [tCD8: HR (95 %CI) = 0.29 (0.1–0.7); *p* = 0.0061; sCD45RO: HR (95 %CI) = 0.43 (0.2–0.9); *p* = 0.0364].

**Conclusion:** An immunoscore of CD8 and sCD45RO in pre-operative biopsy specimens allows prediction of full TNM-stage of the matched resection specimen and survival outcome in the pre-operative setting.

**OFP-18-007**

**Hypermethylation of ZEB2 represents a novel mechanism of colorectal cancer progression and is a highly unfavourable prognostic factor in KRAS wild-type tumors**

I. Zlobec*, M. Helbling, H. Dawson, V. H. Koelzer, E. Diamantis Karamitopoulou, A. Lugli

^*^Universität Bern, Inst. für Pathologie, Switzerland

**Objective:** A majority of colorectal cancers exhibit de-regulation of WNT pathway signaling. This may include loss of E-cadherin expression through transcriptional regulation of ZEB1 and ZEB2. Here, we investigate the methylation status and prognostic effect of ZEB1 and ZEB2 in colorectal cancer.

**Method:** 77 primary colorectal cancers from patients with full clinicopathological and survival time data underwent methylation analysis for ZEB1 and ZEB2, and mutational analysis of KRAS and BRAF using pyrosequencing.

**Results:** ZEB1 was unmethylated in all cases. Frequent methylation of ZEB2 was observed (average 32.6 %; range 1–99 %). ZEB2 hypermethylation was significantly greater in patients with KRAS wild-type tumors (*p* = 0.0397) and BRAF mutated cancers (*p* = 0.055). Stratified by KRAS status, hypermethylation of ZEB2 in wild-type tumors was highly associated with more advanced pT stage (*p* = 0.0463), lymph node positivity (*p* = 0.0032), distant metastasis (*p* = 0.0192), venous invasion (*p* = 0.0193), lymphatic invasion (*p* = 0.0025) and unfavorable survival (*p* = 0.0155; HR (95 %CI): 3.2 (1.2–8.6)), but not with tumor budding. No associations were observed in KRAS mutated cases.

**Conclusion:** These findings underline a role for ZEB2 hypermethylation as a novel mechanism of colorectal cancer progression. Moreover, hypermethylation of ZEB2 identifies a highly aggressive subgroup of colorectal cancer patients in the context of a KRAS wild-type background only.

**OFP-18-008**

**Investigation of IL23 (p19, p40) and IL23R highlights nuclear IL23p19 expression as a marker of indolent tumor features and favorable outcome in colorectal cancer patients**

I. Zlobec ^*^, M. Helbling, V. H. Koelzer, H. Dawson, R. Langer, U. Nitsche, E. Diamantis Karamitopoulou, A. Lugli

^*^Universität Bern, Inst. für Pathologie, Switzerland

**Objective:** IL23 is involved in chronic inflammation but its role in cancer progression is not fully elucidated. Here we characterize IL23 subunits p40, p19 and IL23 receptor (IL23R) in the normal-adenoma-carcinoma-metastasis cascade of colorectal cancers and their relationship to clinicopathological and outcome data.

**Method:** Immunohistochemistry for IL23R, IL12p40, IL23 and IL23p19 (monoclonal) was performed on a multi-punch tissue microarray (*n* = 213 patients). Expression differences between normal-adenomas-cancers-lymph nodes were evaluated. Correlation with clinicopathological and outcome data was undertaken. Results were validated on an independent cohort (*n* = 341 patients).

**Results:** An increased expression from normal-adenoma-cancer was observed (*p* < 0.0001; all) followed by a marked reduction in lymph nodes (*p* < 0.0001; all). Cytoplasmic and/or membranous staining of all markers was unrelated to outcome. Nuclear IL23p19 staining occurred in 23.1 % and was associated with smaller tumor diameter (*p* = 0.0333), early pT (*p* = 0.0213), early TNM (*p* = 0.0186), absence of vascular (*p* = 0.0124) and lymphatic invasion (*p* = 0.01493) and favorable survival (univariate (*p* = 0.014) and multivariable (*p* = 0.0321) analysis). All IL23p19 positive patients were free of distant metastasis (*p* = 0.0146). Survival and metastasis results could be validated in Cohort 2.

**Conclusion:** The presence of nuclear IL23p19 is related to indolent tumor features and favorable outcome supporting a more ‘protective’ role of this protein in colorectal cancer progression.

**OFP-18-009**

**LGR5 positivity defines stem-like cells in colorectal cancer**

T. Gaiser ^*^, T. Ried, A. Marx, D. Hirsch

^*^Universität Mannheim, Inst. für Pathologie, Germany

**Objective:** Like normal colorectal epithelium, colorectal carcinomas (CRC) are organized hierarchically and include cells with stem-like properties. Leucine-rich-repeat-containing G-protein-coupled receptor 5 (LGR5) is highly expressed in these stem cells. However the precise function of LGR5 in CRC remains largely unknown.

**Method:** Here, we analyzed the functional and molecular consequences of RNA-mediated silencing of LGR5 in CRC cell lines SW480 and HT-29. Additionally, we exposed Lgr5-EGFP-IRES-Cre-ERT2 mice to azoxymethane/dextrane sodium sulfate (AOM/DSS) which induces inflammation-driven colon tumors. Tumors were then flow-sorted into fractions of epithelial cells that express high or low levels of Lgr5 and were molecularly characterized using gene expression profiling and array comparative genomic hybridization.

**Results:** Silencing of LGR5 reduced proliferation, migration and colony formation in vitro, and tumorigenicity in vivo. In accordance with these results, Notch signaling was down-regulated upon LGR5 silencing. In the AOM/DSS-induced mouse colon tumors Lgr5 high cells showed higher levels of several stem cell-associated genes and higher Wnt signaling than Lgr5 low tumor cells and Lgr5 high normal colon epithelial cells.

**Conclusion:** Our data elucidate mechanisms that define the role of LGR5 as a marker for stem-like cells in CRC.

**Poster Sessions**

Sunday, 1 September 2013, 09.30–10.30, Pavilion 2

**PS-01 Poster Session Digestive Diseases Pathology I: Upper Gastrointestinal Tract**

**PS-01-001**

**Morphological features of eosinophilic esophagitis in patients with asthma**

L. Mikhaleva ^*^, T. Barkhina, V. Golovanova, N. Shchegoleva, N. Gracheva

^*^Moscow City Hospital, Dept. of Pathology, Russia

**Objective:** To study the morphological features of the mucosa of esophagus in patients with eosinophilic esophagitis during bronchial asthma

**Method:** We used biopsies of esophageal mucosa of patients with asthma. Morphological study was performed by using hematoxylin and eosin staining, according to Mallory and by holding Schick-reaction. We studied a cellular composition of the inflammatory infiltrate by immunohistochemistry using monoclonal antibodies to CD4, to CD8, to CD16, to CD20, to CD68, a CD117. The histologic sections were examined in a light microscope. We conducted the morphometric study and the statistical analysis of the results.

**Results:** We identified an intraepithelial eosinophilic infiltration of the mucosa in the proximal part of esophagus (15 to 55 in a field of view), eosinophilic “microabscesses” and a significant subepithelial sclerosis. According to morphometry the ratio CD4/CD8 was significantly less than “1”. We confirmed by morphometric and statistical studies that the intraepithelial infiltrate consisted mainly in macrophages and “natural killer” cells. We noted an early formation of strictures and stenosis of esophagus in cases of eosinophilic esophagitis during asthma.

**Conclusion:** The study revealed morphological features of the esophageal mucosa, typical for cases of eosinophilic esophagitis with asthma. Further study of the pathogenesis of this comorbidity will help to optimize treatment strategy for these seriously ill patients.

**An intraepithelial eosinophilic infiltration of the mucosa in the proximal part of esophagus (15 to 55 in a field of view):** 
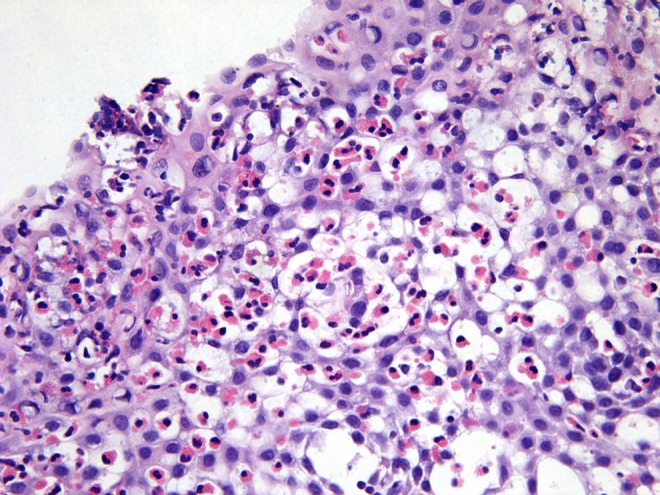


**PS-01-002**

**Interobserver reproducibility in interpretation of columnar lined esophagus**

F. Grillo ^*^, L. Mastracci, N. Piol, F. Pitto, L. Molinaro, R. Fiocca

^*^University of Genoa, DISC, Histopathology, Italy

**Objective:** Terminological confusion regarding the histological interpretation of columnar lined esophagus (CLE) has kept pathologists wading in murky waters for many years. Histology however is mandatory for the confirmation of endoscopically suspected esophageal metaplasia (ESEM). Aim of this study is to evaluate interobserver variability in the interpretation of CLE between pathologists.

**Method:** Thirty pathologists (10 gastrointestinal pathologists; 10 general pathologists; 10 pathology trainees) distributed nationwide and working in community and teaching hospitals were invited to review three 10-case sets of CLE. A first set was coupled with endoscopy with no precise evaluation of ESEM extent; the second and third sets were coupled with descriptive endoscopy (Prague CM criteria); national guidelines (Fiocca et al. DigLivDis 2011;43 Suppl 4:S319-30) were distributed to participants before evaluation of the third set. Agreement was statistically assessed using Randolph’s free-marginal multirater kappa.

**Results:** Concordance between pathologists was lower for the first set (K = 0.34) compared to the second (K = 0.44) and the third set (K = 0.63). Reproducibility was generally higher among GI pathologists compared to general pathologists and trainees. Interpretation of intestinal-type epithelium was less problematic than cardia and oxyntic-types.

**Conclusion:** Precise endoscopic description and use of guidelines increase consistency in interpretation and reporting of CLE in esophageal biopsies.

**PS-01-004**

**Selected molecular markers of disease progression in Barrett Esophagus**

J. Ehrmann ^*^, P. Luzna, V. Divoky

^*^Faculty of Medicine, Olomouc, Czech Republic

**Objective:** Barrett esophagus (BE) is an acquired preneoplastic condition that is characterized by replacement of a stratified squamous epithelium of the distal esophagus by a columnar epithelium. BE represents a permanent risk for developing esophageal adenocarcinoma (EAC). Thus, there is a strong need for specific molecular markers of BE progression that would enable early diagnosis of developing EAC.

**Method:** In our study we followed accumulation of gamm-H2AX, a marker of the DNA damage response (DDR) pathway activation, together with expression of active β-catenin and two inflammatory cytokines, IL-8 and IL-1β.

**Results:** Our results show that the activation of the DDR in BE increases with disease progression and that gamma-H2AX accumulates mainly in EAC. Active β-catenin is found in cytoplasm in all disease stages whereas its membrane localization is exclusively associated with EAC. While the level of IL-1β increased accordingly with tumor progression, IL-8 was expressed constitutively in all disease stages.

**Conclusion:** We conclude that genetic alterations associated with premalignant stages of BE do not have capacity to evoke DDR and that gamma-H2AX is a useful biomarker of disease progression of BE to EAC. Grant support LF_2012_016, LF_2012_019, NT 13585-3/2012

**PS-01-005**

**Unravelling novel mechanisms of Barrett’s esophagus progression: Centrosome amplification precedes the onset of invasion**

M. Mesquita ^*^, C. Lopes, A. Cunha, A. Pereira, P. Chaves

^*^Inst Português de Oncologia, Anatomia Patológica, Lisboa, Portugal

**Objective:** Barrett’s esophagus (BE) malignant progression involves major changes in cellular processes controlled by the centrosome, the primary microtubule-organizing centre in animal cells. Though centrosome dysfunction is associated with several neoplastic pathways, its role in BE tumorigenesis is still unknown. Our aim was to evaluate the centrosome profile in Barrett’s progression.

**Method:** Dysplasia-negative BE biopsies, esophagectomy specimens and established cell lines were analyzed. The percentage of cells with amplified centrioles (>4 centrioles/cell) was assessed by immunofluorescence.

**Results:** The percentage of cells with amplified centrioles increased from metaplasia to neoplasia: 0.0 % ± 0.0 %-dysplasia-negative BE biopsies, 0.46 % ± 0.69 %-BE associated with ADC, 2.46 % ± 1.73 %-ADC, 8.25 % ± 4.32 %-lymph node metastasis. Despite the high variability between patients, an increase of cells with amplified centrioles during progression was observed in all patients. These results were confirmed in cell lines: 1.7 %-metaplasia, 33.3 % ± 7.1 %-high-grade dysplasia, 10.8 % ± 10.5 %-ADC, 8.0 % ± 5.9 %-metastasis.

**Conclusion:** Our findings suggest that centrosome abnormalities are related to Barrett’s progression and precede the onset of invasion. Correlation between these changes and the well-known genetic profile of the cell lines provides new clues into the permissive genetic background for such abnormalities (like p53 loss) and may impact on BE diagnosis, prognosis and treatment.

**PS-01-008**

**Lymphoepithelioma-like carcinoma of the esophagogastric junction with microsatellite instability**

S. Gurzu ^*^, Z. Szentirmay, I. Jung

^*^University of Medicine and Pharmacy, Dept. of Pathology, Tirgu-Mures, Romania

**Objective:** To present an extremely rare tumor of the esophagogastric junction that can be associated with Epstein-virus infection

**Method:** A 60-year-old male presented with symptoms suggesting a gastric cancer and total gastrectomy was performed. The surgical specimen was submitted for histopathological examination.

**Results:** Microscopically, the ulcero-infiltrative tumor of the esophagogastric junction was a poorly differentiated adenocarcinoma intermingled with dense lymphoid infiltration predominantly composed of T-cell lymphocytes. The tumor cells infiltrated the submucosa, muscularis and subserosal layers of the stomach respectively the esophageal adventitia. No metastases were noticed in the 58 regional lymph nodes. Based on the histopathological features, the diagnosis was lymphoepithelioma-like carcinoma, pT3N0 stage. In situ hybridization for Epstein-Barr virus showed no nuclear signal in tumor cells. The p53 expression was observed in fewer than 10 % of the tumor cells. Real-time PCR analysis showed microsatellite instability without K-ras mutation in codon12. No recurrences or metastases were reported 2 years after surgical intervention. No adjuvant therapy was performed

**Conclusion:** Diagnosis of lymphoepithelioma-like carcinoma remains difficult, especially in those cases that lack glandular differentiation. Microsatellite instability seems to play an important role in its histogenesis and associates good prognosis even in late stages

**PS-01-009**

**Clear/glassy cell change of gastric mucosa: A diagnostic pitfall?**

J. Pinheiro ^*^, E. Rios, F. Carneiro

^*^Hospital de Sao Joao, Dept. de Anatomia Patologica, Vila de Cucujaes, Portugal

**Objective:** Glassy gastric cells (GGCs) were first described 27 years ago in gastric mucosa, characterised by a uniform glassy, eosinophilic or clear vacuole that pushes the nucleus towards the apical pole. Since then, the literature dedicated to this topic is quite scarce. GGCs constitute a diagnostic pitfall being frequently misinterpreted as malignant signet-ring cells.

**Method:** Two gastrectomy specimens and 12 endoscopic biopsies of stomach containing GGCs were retrieved from the files of our department. Slides were stained with haematoxylin-eosin and PAS-D.

**Results:** GGCs were identified in 14 patients (10M/4F; 50–85 years; mean age: 66.2 years), in the setting of chronic gastritis (*n* = 6), gastric carcinoma (*n* = 2), hyperplastic polyps (*n* = 1), fundic gland polyps (*n* = 2) and normal mucosa (*n* = 1). GGCs were observed in the antrum and body, in compact glands lined by a single row of cells. PAS-D staining was negative in the glassy/clear vacuoles, being restricted to a thin rim of apical cytoplasm.

**Conclusion:** GGCs can be found in the setting of a broad spectrum of gastric conditions and mimic malignant signet-ring cells (a diagnosis that is excluded by the negative PAS-D staining of the glassy/clear vacuoles). Awareness of the morphologic and histochemical features is necessary for proper interpretation.

**PS-01-010**

**An “unclassified” polyp of stomach**

E. Basar ^*^, B. Doganavsargil, R. Vardar, M. Sezak, M. Tuncyurek

^*^Ege University, Dept. of Pathology, Izmir, Turkey

**Objective:** Gastric polyps are a heterogeneous group of tumours including more common non-neoplastic fundic gland, hyperplastic or hamartomatous polyps and less frequent neoplastic polyps. However there also exist a considerable number of “reactive or unclassified” protrusions which are readily defined as “polyps” endoscopically but not as easily or reproducibly classifiable pathologically. We present a subgroup of such “polyps” which are not well documented in literature in detail.

**Method:** Twenty-one polyps of 18 patients which were reported as “polyp, not otherwise specified” in a 2 years period were included in the study.

**Results:** The mean age of the patients were 59 ± 12,6 years old, (Range: 32–80 y.o). Male/female ratio was 7/13, the localizations were antrum (85 %, 17/20), corpus (10 %, 2/20) and cardia (5 %, 1/20), The mean diameter was 1.14 ± 0.3 cm (Range: 0.5–1.7 cm). Identifiable common histological variations were marked foveoler hyperplasia (33,3 %), thick and irregular muscularis mucosa (23.8 %), prominent eosinophilic infiltration (38.0 %), intraepithelial lymphocytes (47.6 %), mild to severe stromal edema and congestion in all cases.

**Conclusion:** There exist a certain type of “unclassified/distal antral polyp” the most distinctive features of which are antral gland hyperplasia and hypertopy of muscularis mucosa. The cases were discussed in respect to nomenclature and differential diagnosis from other benign polyps.

**PS-01-011**

**Gastric Dieulafoy’s lesion or caliber-persistent artery: Report of two cases**

I. Jung ^*^, C. Copotoiu, S. Gurzu

^*^University of Medicine and Pharmacy, Dept. of Pathology, Tirgu-Mures, Romania

**Objective:** To present a vascular malformation of the gastrointestinal tract, incidentally diagnosed under microscope

**Method:** Two patients were hospitalized with hematemesis. Case 1 was a 62-year-old-male endoscopically diagnosed with chronic gastric ulcer for that laparoscopic suture was performed. Due to recurrent bleeding total gastrectomy was the treatment of choice but the patient died due to septic shock. Case 2 was a 75-year-old-male that underwent a Billroth-II gastric reconstruction for a peptic ulcer 38 years ago. At the present hospitalization, a stump gastric cancer was endoscopically diagnosed and total removal of the gastric remnant was performed, with favorable evolution

**Results:** In both cases 1 and 2, corresponding to the ulcerated lesion (case1), respectively near to the tumor, that was an early cancer (case 2), the histological examination revealed abnormally thick-walled enlarged vessels in the submucosa and muscularis propria, some of them being thrombotic. Focally, these oversized tortuous vessels protruded through the muscularis mucosae in the mucosal layer, some of them being eroded. Based on these characteristics and the hematemesis, the final diagnoses were fatal hemorrhage due to Dieulafoy’s lesion (case 1) respectively early gastric stump carcinoma associated with Dieulafoy’s lesion (case 2)

**Conclusion:** Dieulafoy’s lesion is a vascular malformation that still remains largely undiagnosed

**PS-01-012**

**Melatonin reduces intensity of morphogenetic signs of hyperacid gastritis, experimented on animals**

G. Mylovydova ^*^, G. Gubina-Vakulik, N. Kolousova

^*^Laboratory of Pathology, and Exp. Surgery, Kharkiv, Ukraine

**Objective:** Study microscopic features of a mucous coat of stomach of the rabbits staying in the conditions of continuous lighting within 5 months and estimate the effect of melatonin injections course on intensity of morphogenetic symptoms of hyperacid gastritis.

**Method:** For 5 months rabbits were under constant illumination. Then eight of them received 10 injections of melatonin 2.5 mg/kg. 5 animals received 10 injections of saline. 5 rabbits of the same age were intact. Their gastric mucosa microslides were stained by gallocyanine (by Einarsson) for total nucleic acids. Parietal cells and their nuclei sizes, as well as the optical density of the main cell cytoplasm (Axiostar-plus-Zeiss) were determined.

**Results:** Experiment led to formation of chronic atrophic-hypertrophic gastritis with erosions, parietal cells hyperplasia. Course of melatonin injections resulted in a significant decrease in the number of parietal cells. There were the signs of decreasing morphofunctional activity and enhanced apoptosis of parietal cells. A major cell cytoplasm contains an increased amount of RNA (0,406 ± 0,015 and 0,474 ± 0,018 conditional units of optical density, *p* ≤ 0,05).

**Conclusion:** Long permanent lighting leads to the development of chronic atrophic-hypertrophic gastritis in animals. The course of injections of night hormone melatonin causes improvement of morphofunctional pattern of mucous coat of stomach.

**PS-01-014**

***H. pylori*** **and chronic gastritis: A histopathologic study**

A. Kurt ^*^, S. A. Özmen, I. Çalik, I. Gelincik

^*^Bölge Egitim ve Arastirma Hastesi, Dept. of Pathology, Erzurum, Turkey

**Objective:** The aim of this study was to determine the level in the application of the Sydney Classification and to find out whether this grading system is reproducible in routine histopathological practice.

**Method:** Hematoxylen and Eosin, Alcian blue pH 2.5, PAS and Giemsa stained sections of 210 gastric biopsy specimens were evaluated. These were mucosa of antral biopsy, diagnosed as chronic gastritis during routine histopathological examination within April- May 2012. Each observer graded chronic inflammation, neutrophyl leucocyte, atrophy, intestinal metaplasia and Helicobacter pylorii density in the antrum on a scale 0–3. The measurement of on the histopathological grades was examined by “measures of agreement” and statistics respectively.

**Results:** It was found out that the proportion of overall agreement was on 19 % for atrophy, 17 % for lymphoid follicles, 67 % for neutrophyl leukocyte, 15 % for intestinal metaplasia in the antrum.

**Conclusion:** In this study, it was found out that there was a suitable relationship between lymphocyte inflammation, neutrophyl leukocyte and Hp. There was no relationship between Hp and glandular atrophy, intestinal metaplasia and lymphoid follicular formation. It was concluded that the results are in accordance with the referance generally. In conclusion, we are in the opinion that Sydney System is useful in diagnosing gastritis.

**PS-01-015**

**Interobserver agreement of gastritis staging by OLGA and OLGIM system between general pathologists**

S. Isajevs ^*^, I. Liepniece-Karele, D. Janciauskas, G. Moisejevs, K. Funka, I. Kikuste, A. Vanags, I. Tolmanis, M. Leja

^*^University of Latvia, Dept. of Pathology, Riga, Latvia

**Objective:** The aim of our study was to compare interobserver agreement in the staging of gastritis by OLGA and OLGIM system between the general pathologists.

**Method:** 839 patients undergoing upper endoscopy were enrolled in the study. Three general pathologists graded biopsy specimens according to the Sydney classification, OLGA and OLGIM staging system. Interobserver agreement was analyzed by kappa statistics.

**Results:** Overall, 280 (33.4 %) and 167 (19.9 %) patients were classified as stage I–IV according to OLGA and OLGIM, respectively. Interobserver agreement for atrophic gastritis was moderate in antrum and incisura angularis (respectively, kappa = 0.53 and 0.57, *p* < 0.0001), but fair for atrophic gastritis assessement in corpus (kappa = 0.38). However, interobserver agreement was almost perfect for intestinal metaplasia assessement both in antrum, incisura angularis and corpus (respectively, kappa = 0.82, 0.80 and 0.81, *p* < 0.0001). The interobserver agreement for displasia was moderate (kappa = 0.58, *p* < 0.0001).

**Conclusion:** Gastritis staging systems (both OLGA and OLGIM) convey prognostically important information on the gastritis-associated cancer risk. However, OLGIM staging system characterized with a highest interobserver agreement. Supported by ERDF Nr.2010/0302/2DP/2.1.1.1.0/10/APIA/VIAA/158.

**PS-01-016**

**What summons eosinophils to gastric mucosa?**

B. Doganavsargil ^*^, M. Sezak, B. Sarsik, B. Yaman, R. Vardar, M. Tuncyurek, S. Bor, DISPEN Study Group

^*^Ege University, Dept. of Pathology, Izmir, Turkey

**Objective:** The factors affecting eosinophil trafficking to Gastrointestinal tract, which shows a higher eosinophil density (ED) than many tissues, is not fully uncovered. We searched the relationship between ED and gastritis.

**Method:** One thousand cases were retrieved randomly from DISPEN-database and re-evaluated. Special forms of acute and chronic gastritis and biopsies which were unsuitable for atrophy assesment were excluded. ED was assessed as number of eosinophils per one high power field (HPF) in hot spots (by Olympus BX50 microscope, ×40 magnification:0,54 mm diameter, Ocular: ×10). Gastritis was evaluated according to modified Sydney classification and correlated by non-parametric tests.

**Results:** Distribution of cases with normal mucosa, atrophic and nonatrophic gastritis were 13.4 %, 7 %,79,6 % respectively. Chronic active gastritis, intestinal metaplasia and H. Pylori positivity observed in 35.4 %, 89.2 %, 68.2 % of cases. Mean antral and corporal ED’s were 3.88 ± 6.15 (Range: 0–70/HPF) and 2.32 ± 1.00 (Range:0–45/HPF). Sixty cases showed considerable (≥10/HPF) eosinophils. Increased ED was correlated with atrophy, H.pylori colonisation and active inflammation (*p* < 0.001, each). The difference between atrophic and nonatrophic gastritis was remarkable.

**Conclusion:** Eosinophils are responders of inflammation thus affected by active gastritis and H. Pylori colonisation. However, the relationship between eosinophil infitration and atrophy, deserves special attention and should be further investigated for possible pathogenetic aspects.

**PS-01-017**

**Solitary gastric hamartoma: A case report**

N. Abid^*^, R. Kallel, N. Gouiaa, H. Mnif, S. Ellouze, M. Ksentini, S. Makni, T. Boudawara

^*^Habib Bourguiba University Hospital, Pathology, Sfax, Tunisia

**Objective:** Gastric hamartoma is considered to be a rare entity and is frequently associated with polyposis syndromes. Sporadic hamartoma of the stomach is uncommon. We described a new case; our aim is to study the histological features of this entity and insist on its differential diagnosis.

**Method:** A 53-year-old man with a story of urinary lithiasis was proposed to be treated with extracorporeal shock wave lithotripsy. Pretreatment imaging by noncontrast computed tomography showed focal eccentric mural thickening at the wall of the greater curvature of the stomach. Esophagogastroduodenoscopy revealed a pedunculated polypoid mass with ulcerated mucosa measuring 1 cm in diameter. The polyp was endoscopically removed then fixed in formalin and embedded into paraffin. Serial sections stained with hematoylin-eosin were examined and immunohistochemical staining was perfomed for vimentin, desmin and smooth muscle actin

**Results:** Histological findings were consistent with hamartoma, containing glands with cystic formation, smooth muscle, vasoformative tissue and fat cells.

**Conclusion:** Gastric hamartoma is a rare occurrence in patients without familial polyposis coli. Clinically, solitary gastric hamartoma is asymptomatic and tend to be found incidentally,or may cause mild upper digestive symptoms. The final diagnosis depends on the pathological findings; however, gastric hamartomashould be histologically distinguished from gastritis cysticaprofunda.

**PS-01-018**

**Juvenile polyposis with prominent gastric involvement: Case report**

H. Baldaia ^*^, M. Guimarães, J. Magalhães, F. Carneiro

^*^Centro Hospitalar S. João, Dept. of Pathology, Porto, Portugal

**Objective:** Juvenile polyposis is a familial cancer syndrome characterized by multiple juvenile polyps throughout the gastrointestinal tract. Three types are described: juvenile polyposis of infancy, juvenile polyposis coli and generalized juvenile polyposis.

**Results:** We present the case of a 50-year-old male with no relevant family history, submitted to a proctocolectomy 7 years before for “Familial Adenomatous Polyposis”, APC negative. He presented with weakness and vomiting; the upper endoscopy revealed multiple polyps throughout the stomach. A total gastrectomy was performed which showed carpeting of gastric mucosa with sessile lobulated polyps, the largest 4,0 cm in diameter. Histologically, these polyps were hamartomatous, with a smooth or multilobulated surface, a prominent inflammatory stroma and tortuous and dilated glands. There were no dysplastic features. The histological evaluation prompted a review of the previous proctocolectomy specimen which revealed similar hamartomatous polyps, some with low grade dysplasia. A diagnosis of juvenile polyposis was confirmed by SMAD4 mutation.

**Conclusion:** This case documents a prominent gastric involvement in generalized juvenile polyposis, a condition that must be included in the differential diagnosis of polyposis syndromes affecting the stomach. The combination of clinical data and histological and molecular features are essential for the diagnosis.

**PS-01-020**

**Carcinoma in hyperplastic polyps of the gastrointestinal tract: A report of two cases**

J. Orlowska ^*^, D. Jarosz, J. Pachlewski, M. Rupinski, J. Kupryjanczyk, J. Regula

^*^Oncology Centre Warsaw, Dept. of Pathology, Poland

**Objective:** Hyperplastic polyps of the gastrointestinal tract (GIT) were considered innocuous for many years. At present malignant transformation of gastric hyperplastic polyps to carcinoma is well documented (WHO, 2010), however, its incidence is very low (2.1 %) 1. On the other hand, it is estimated that colorectal serrated lesions may be precursors of 15 %–25 % of colorectal carcinomas, particularly localized in the proximal colon.

**Method:** Two unusual cases of focal carcinoma in hyperplastic polyps (stomach and ascending colon) are described.

**Results:** CASE 1: Gastroscopy performed in 85-year-old woman due to anaemia, revealed a 13 mm pedunculated polyp of the corpus which was endoscopically removed. Histologically it was partially typical for hyperplastic polyp, and partially for conventional adenoma with focal tubular adenocarcinoma (G2) intermingled with signet ring cell carcinoma. CASE 2: Screening colonoscopy performed in a 60-year-old man revealed 16 pedunculated and semipeduculated polyps of 3 to 15 mm distributed throughout the colon which were endoscopically removed. Two of them were hyperplastic, and all but one remaining were tubular adenomas with low grade dysplasia. One 8 mm ascending colon polyp showed a structure typical for hyperplastic polyp with sharp transition to conventional adenoma showing focal invasive adenocarcinoma (G3) with signs of angioinvasion. 1. Orlowska J., et al.: Malignant transformation of benign epithelial gastric polyps. Am J Gastroenterol 1995;90:2,152–2,159.

**PS**-**01-021**

**Gastric adenocarcinoma associated with hyperplastic polyposis**

S. Rammeh^*^, N. Sabbegh Znaidi, A. Blel, R. Aloui, R. Zermani

^*^Charles Nicolle Hospital, Pathology, Tunis, Tunisia

**Objective:** We present a case report of a gastric adenocarcinoma associated with a diffuse hyperplastic gastric polyposis.

**Method:** A 56 years old tunisian male was investigated for dysphagia. The Esophago-gastro-duodenoscopy revealed a mass in the fundus and the antrum stomach associated with numerous gastric polyps filling the entire stomach. He had a total gastrectomy.

**Results:** Histological exmination showed a moderately differentiated adenocarcinoma associated with numerous hyperplastic type polyps.

**Conclusion:** The clinical and endoscopic features are important to consider in order to distinguish hyperplastic polyps from hamartomatous polyps, Coden disease and Cronkhite-Canada syndrom.

**PS-01-022**

**PTP4A3 (PRL-3) expression correlate with lymphatic metastases in gastric cancer**

K. Guzinska-Ustymowicz ^*^, A. Pryczynicz, K. Niewiarowska, W. Famulski, A. Kemona

^*^Medical University of Bailysto, Pathomorphology, Bialystok, Poland

**Objective:** Gastric cancers frequently metastasize to the nearest lymph nodes, involving retroperitoneal and left supraclavicular lymph nodes as well as distant organs. Early detection of metastases greatly improves the patients’ prognosis. Recent literature has proved that protein tyrosine phosphatase type IVA member 3 (PTP4A3, PRL-3) plays a major role in the metastasis of gastric cancer, especially to local lymph nodes. The objective of the current study was to assess the expression of PTP4A3 in gastric cancer in correlation with selected anatomoclinical parameters and patients’ survival.

**Method:** The study was conducted on a group of 71 patients treated surgically for gastric carcinoma and divided according to Lauren’s, Goseki’s, Bormann’s and Kubo’s classifications. The PTP4A3 expression was determined using the immunohistochemical method with mouse monoclonal anti-PTP4A3 antibody

**Results:** We demonstrated a statistically significant correlation between the expression of PTP4A3 and Kubo’s classifications (*p* = 0.0454) and on the verge of statistical significance with Lauren’s classification (*p* = 0.0503). The positive expression of the protein was associated with the poorly-differentiated mucoid carcinoma and diffused-type carcinoma (58 % of cases). Also a very significant correlation was found between local lymph node involvement and positive PTP4A3 expression in primary tumour (*p* < 0.001).

**Conclusion:** Our investigations seems to confirm that PTP4A3 may have a significant impact on the lymphatic spread of gastric carcinoma.

**PS-01-023**

**Gastric metastases of breast cancer may mimic primary gastric cancer**

A. Djikic Rom ^*^, J. Jotanovic, M. Andrejevic, O. Skrobic, M. Micev

^*^Clinical Centre of Serbia, Dept. of Histopathology, Belgrade, Serbia

**Objective:** The stomach is an infrequent site of breast cancer metastasis. Metastatic spread to the stomach may occur many years after the initial treatment for breast cancer and it’s very difficult to distinguish it from primary gastric cancer. It is important to make this distinction because of tretment differences between these two entities.

**Method:** Routine histological and ancillary immnohistochemical examination.

**Results:** We present a case of 66-years-old woman who had a diagnosis of breast cancer 12 years ago and had left radical mastectomy with subsequent radiochemotherapy. Last 2 years she had dysphagia and regurgitation. Endoscopic examination revealed stenosis of the upper thoracic esophagus and diffuse erosions of mediogastric region. Histologically gastric mucosal biopsy showed microfocal cancerous infiltration mostly in superficial propria which rarely could be seen in the deeper parts of the mucosa. Some of carcinomatous cells revealed obvious signet-ring cell type morphology. Imunohistochemically, most tumoral cells expressed steroid receptors but were negative for GCDFP-15 and mammaglobin. We concluded that tumoral infiltration was consistent with metastatic breast cancer infiltration.

**Conclusion:** Previous history of other malignancies should be always considered before histopathological examination.

**PS-01-024**

**Gastrointestinal lymphomas: 10 years experience of a single center**

F. Unal Yildirim ^*^, B. Doganavsargil, N. Ozsan, M. Sezak, M. Tunçyurek, M. Hekimgil

^*^Ege University Medical Faculty, Dept. of Pathology, Izmir, Turkey

**Objective:** One-third (30–40 %) of extranodal lymphomas occurs in gastrointestinal (GI) tractus. Although all histological types may develop, small and large B-cell lymphomas predominate, T- and extranodal NK/T- cell lymphomas are less common. Approximately 60–75 % of cases occur in the stomach. Frequency of small intestinal lymphomas shows geographic variation.

**Method:** We reviewed 177 cases diagnosed between 2002 and 2011, re-classified according to the recent World Health Organization classification.

**Results:** There was a slight male predominance (female/male ratio was 0,71; 73/104) The median age was 54 years old (Range: 3–90 y.o). Eighty-two percent (*n* = 145) of cases had only endoscopic biopsies, where 18 % (*n* = 32) also had resection materials. The most commonly involved site was stomach (*n* = 126; 71 %), followed by small intestine (*n* = 26; 15 %) colon (*n* = 16; 9 %), rectum (*n* = 6; 3 %), appendix (*n* = 2; 1 %) and esophagus (*n* = 1; 0.5 %). The most common subtypes were diffuse large B-cell lymphoma (*n* = 109; 62 %), MALT lymphoma (*n* = 31; 17 %) and Burkitts lymphoma (*n* = 18; 10 %). Rare subtypes were mantle cell lymphoma (*n* = 6; 3 %), T-cell lymphomas (*n* = 3; 3 %), follicular lymphoma (*n* = 1; 0.5 %), plasmablastic lymphoma (*n* = 1; 0.5 %) and anaplastic large cell lymphoma (*n* = 1, 0.5 %).

**Conclusion:** The distribution of histological types and localization were consistent with the literature. Despite geographic predisposition no immunproliferative small intestinal disease (IPSID) cases were identified.

**PS-01-025**

**Heterogeneities of HER2 expression and amplification in gastric carcinomas**

M. Bamba ^*^, H. Sugihara, S. Takemura, K. Fukuda, M. Masuyama, T. Shigematsu, G. Kato, R. Kushima

^*^Saiseikai Shiga Hospital, Dept. of Pathology, Ritto, Japan

**Objective:** Recently, HER2 becomes a molecular target for treatment of gastric carcinomas with a monoclonal antibody trastuzumab. However, evaluation of HER2 expression are not easy because of their intratumoral heterogeneities. In this study, we analyzed heterogeneities of HER2 expression and amplification in gastric carcinomas.

**Method:** This study was based on the analysis of 72 surgically resected gastric carcinoma cases. First, we performed fluorescence in situ hybridization to select HER2-amplified carcinomas. Then, H&E stain, immunohistochemical stain with HER2 antibody (4B5) and dual color in situ hybridization for HER2 gene were applied in selected carcinomas.

**Results:** Twelve out of 62 informative cancer lesions were HER2-amplified either homogenously (6 lesions) or heterogeneously (6 lesions). Of these 12 lesions, HER2 overexpression was homogeneous in 2 lesions, heterogeneous in 8 lesions and negative in 2 lesions. Although 3 out of 6 heterogenous HER2-amplified lesions included some non-expressed areas, its amplification was detected not only in virtually all the areas positive for its expression, but also in 18 out of 25 areas (from 10 cases) without its expression.

**Conclusion:** These findings suggest that even if HER2/neu overexpression cannot be detected anywhere in the lesion, it may possibly be valuable to examine HER2 amplification to extend the application for trastuzumab-therapy in gastric carcinomas.

**PS-01-026**

**HER2 amplification in gastric cancer is a rare event restricted to the intestinal phenotype**

A. Gamboa-Dominguez ^*^, C. Cruz-Reyes

^*^INCMNSZ, Dept. of Pathology, Mexico City, Mexico

**Objective:** Identify HER2 prevalence in gastric cancer and correlate it with location, phenotype and follow-up.

**Method:** Consecutive gastric cancer patients with tissue blocks, gross and follow-up data, were submitted to immunohistochemistry(IHC) with Herceptest. Chromogenic and fluorescent in-situ hybridization (CISH/FISH) was performed in IHC +ve tumors.

**Results:** 269 patients were included with a median age of 61 years. In 172 gastrectomized patients histotypes were diffuse (72/41.8 %), intestinal (63/36.6 %), and mixed (37/21.5 %). Her2 IHC expression was 0 in 167, 2+ in 2 and 3+ in 3 tumors. Only endoscopic biopsies were available in 97 patients and Her2 IHC expression was 0/88, 1+/3, 2+/4 and 3+/2 patients. 10/269 (3.7 %) had Her2 amplification. Amplified tumors were intestinal adenocarcinomas located throughout the different regions of the stomach. Heterogeneity was documented in four widely sampled tumors.

**Conclusion:** Her2 amplification was restricted to the intestinal phenotype; is a rare event and, its screening should be driven by gastric cancer histotype.

**PS-01-027**

**Automated HER2 expression and FISH detects all variables in gastric carcinoma for economical quickness targeted therapy**

L. Carvalho ^*^, M. R. Silva, A. Alarcão, A. Ladeirinha, T. Ferreira, M. J. d’Aguiar

^*^Faculty of Medicine Coimbra, Inst. of Anatomical Pathology, Portugal

**Objective:** Anti-HER2 transtuzumab benefit in Her2 overexpression and amplification in 10–20 % of gastric carcinoma(GC) implies testing. Two scoring systems for immunohistochemistry-IHC and FISH runs in the same fully automated staining system were compared to validate quickness and economical sparing.

**Method:** Sections of 49 biopsies of GC were either fully automatically stained with monoclonal antibody-based OracleHER2 Bond IHC kit and registered after the breast scoring for GC results and tested for amplification with dual-colour LSI HER2/CEP17 Dual Probe in the automated system BOND-MAX™.

**Results:** HER2 FISH amplification and overexpression–six cases (13 %) IHC 3+ also positive for FISH; of 2 cases (4 %) IHC 2+, one was FISH positive (HER2/Chr17 = 2.8) and the other was FISH negative (HER2/Chr17 = 1.0) due to polysomy of chromosome 17; 2 cases IHC 1+, were FISH negative (HER2/Chr17 = 1.6 and 1.5).

**Conclusion:** The automated BOND-MAX™ staining system for IHC and FISH demonstrated overall good quality in observed slides. Our study identified 6 cases with aberrant in situ hybridization polysomy of chromosome 17, negative for HER2 FISH amplification. Described heterogeneous tumor FISH patterns of chromosome 17 aneusomy appear as Her-2/CEP17 low ratio and were classified as negative. The applied methodology revealed to be economical with less manual discrepancy for routine in Pathology.

**PS-01-028**

**Minimum biopsy set for Her-2 evaluation in gastro-esophageal adenocarcinoma**

F. Grillo ^*^, L. Mastracci, I. Gullo, N. Piol, L. Molinaro, M. Fassan, M. Rugge, R. Fiocca

^*^University of Genoa, DISC, Histopathology, Italy

**Objective:** Patients with advanced or metastatic HER2-positive adenocarcinoma of the stomach or gastro-esophageal junction (GEC) can be treated with Trastuzumab. In these patients HER2 assessment is often based only on endoscopic biopsies; immunohistochemical HER2 expression in GEC has however been shown to be highly heterogeneous. The aim of the study was to identify the minimum endoscopic biopsy set required to confidently evaluate HER2-status in GEC.

**Method:** 103 cases of resected GECs were selected. For each case two different sections were immunostained for HER2 and slides were digitally scanned. By using digital image analysis 10 random virtual biopsies (VB) (2.6 mm diameter), were taken for each case from the luminal part of the sample. HER2-status as defined by biopsy sets composed of an increasing number of VBs was compared to the overall HER2-status.

**Results:** A minimum of 5 biopsies was shown to be the most accurate in predicting overall HER2-status (sensitivity: 91.89 %; specificity: 96.97 %). Fewer biopsies did not influence the level of specificity while sensitivity varied from 61.54 % for 1 biopsy to 89.19 % for 4 biopsies. Above 5 biopsies no increase in accuracy was seen.

**Conclusion:** A minimum set of 5 biopsies is required for maximum HER2 assessment accuracy in GEC.

**PS-01-029**

**Her2 expression in Turkish gastric carcinomas: Immunohistochemistry and dual-color single in situ hibridization**

S. Erdamar ^*^, C. Sonmez, E. Demirhan, N. Kepil

^*^Cerrahpasa Medical College, Dept. of Pathology, Istanbul, Turkey

**Objective:** A recent randomized controlled trial (Trastuzumab for Gastric Cancer [ToGA] study) established standard scoring criteria of human epidermal growth factor receptor 2 (HER2) for gastric cancer and demonstrated the efficacy of trastuzumab for treating metastatic gastric cancer. We aimed to evaluate the frequency of her2 positivity in advanced gastric carcinomas.

**Method:** A total of 218 patients were included in this retrospective study. All tumor samples were examined for HER2 expression by immunohistochemistry (IHC),and HER2 amplification by dual color in situ hybridization (DISH).

**Results:** HER2-positive tumors were identified in 47 patients (21.5 %). HER2-positive cases were more frequently found in the proximal localization and intestinal histological type (*P* = 0.04 and 0.030, respectively). There was 98 % concensus between IHC3+ cases and DISH positivity. Only 2 cases were positive in pure signet ring cell carcinomas. 78 % of IHC2+ cases were DISH+.

**Conclusion:** This study indicate that frequency of HER2 positivity in our series is similar with TOGA study. IHC3 positivity is strongly demonstrate DISH positivity, but the antibody chosen for HER2 IHC is important for analysis.

**PS-01-031**

**Does biopsy reliably identify grade in gastro-entero-pancreatic neuroendocrine tumors?**

F. Grillo ^*^, M. P. Brisigotti, M. Albertelli, C. Rossi, R. Fiocca, L. Mastracci

^*^University of Genoa, DISC, Histopathology, Italy

**Objective:** A new proliferation-based grading system (ENETs/WHO) has proved important in establishing prognosis and guiding therapy in gastro-entero-pancreatic neuroendocrine tumors (GEP-NETs). Biopsies are often performed for grading but little is known about their reliability in assigning grade. The aim of this study was to determine the accuracy of grade identification in virtual biopsies (VBs) of GEP-NETs.

**Method:** Twenty cases of resected GEP-NETs were selected. VBs of different lengths (3–20 mm) and widths (0.5–1.5 mm) were performed randomly. Number of neoplastic cells and Ki67 index were quantified in up to 10 different areas for each case and for different sizes of biopsies (classified as large, medium or small). Ki67 index in biopsies was compared with that assessed in the whole samples.

**Results:** Out of all (186) VBs performed, 50.7 % of them undergraded G2 lesions. In all 11 G1 cases all VBs of large size (>2,000 cells) correctly identified grade. In 3 G1 cases the small Vbs (containing <2,000 cells) overgraded the cases as G2.

**Conclusion:** Clinicians must be aware that grade identified on biopsy material can undergrade G2 cases (likely due to intratumor heterogeneity) or overgrade G1 cases (when little tissue is available for evaluation).

**PS-01-032**

**Grade increases in gastro-entero-pancreatic neuroendocrine tumor metastases compared to the primary tumor**

F. Grillo ^*^, M. P. Brisigotti, M. Albertelli, T. Borra, L. Mastracci, R. Fiocca, D. Ferone

^*^University of Genoa, DISC, Histopathology, Italy

**Objective:** The neuroendocrine tumor (NET) proliferation-based grading system (ENETs/WHO) has proved reliable for prognostic stratification. Whether grade changes between the primary site and metastases has never been investigated. Our aim was therefore to evaluate Ki67-index changes between the primary tumor and its metastases.

**Method:** A total of 256 GEP-NETs (1993–2011) were identified from the Histopathology archives; 45 of them had tissue from the primary tumor and from local/distant metastases (28 synchronous; 8 metachronous). Immunohistochemistry for Ki67 was performed on both primary and metastatic tumors.

**Results:** Out of 28 patients with primary NET and synchronous metastases, 22 (78,6 %) presented exactly the same grade. Six (21,4 %) cases showed discrepancy in Ki67-index and grade, 5 changed from G1 in the primary to G2 in the metastasis (nodal, hepatic and mesenteric) while 1 changed from G2 to G3 in the metastasis. Eight patients underwent surgical excision of metachronous metastases during follow up. Four (50 %–2 nodal; 2 hepatic) patients showed an increase in Ki67-index in the metastatic site and a change in grade, from G1 to G2.

**Conclusion:** Primary NETs and their synchronous/metachronous metastases not infrequently show differences in grade and evaluation of Ki67-index at all sites may be relevant for patient management.

**PS-01-033**

**Stromal tumors: GIST (about 13 comments)**

L. Beddar ^*^, K. Boudaoud, Z. TEBBI, M. Kout

^*^CHU Benbadis, Dept. of Pathology, Constantine, Algeria

**Objective:** GIST represent less than 1 % of all tumors of the gastrointestinal tract. Including the estimated impact is close to two new cases per 100,000 inhabitants per year. The purpose of our work is to study the pathological features and prognostic factors establish stromal tumors

**Method:** We report 13 cases whose average age is 56 years, with no particular medical history. The location is gastric in 80 % of cases. CT scan examination reveals the presence of large training with irregular contours, often compressing the vascular structures. The treatment is surgical.

**Results:** It is malignant mesenchymal tumors with cell fuso sometimes epithelioid differentiation, mitoses are always present from (<5 mitoses/50 + fields 10/50 fields at magnification 400). The immunohistochemical study in search of diagnostic certainty shows: -CD117positif in 90 % of cases, intense staining + + cells in 100 %. -Desmin-: negatif -Ki67: positive reaction.

**Conclusion:** GIST represent a specific nosological entity since the discovery of their relationship with “CAJAL cells,” they are often associated with a mutation in the gene Kit. Prognostic factors are essentially tumor size and mitotic index. Gastrointestinal stromal tumors usually occur in advanced stages because of their silence clinic before they can lead to local complications and sometimes metastasis.

**GX10 GIST: immunostaining shows: CD117positif:** 
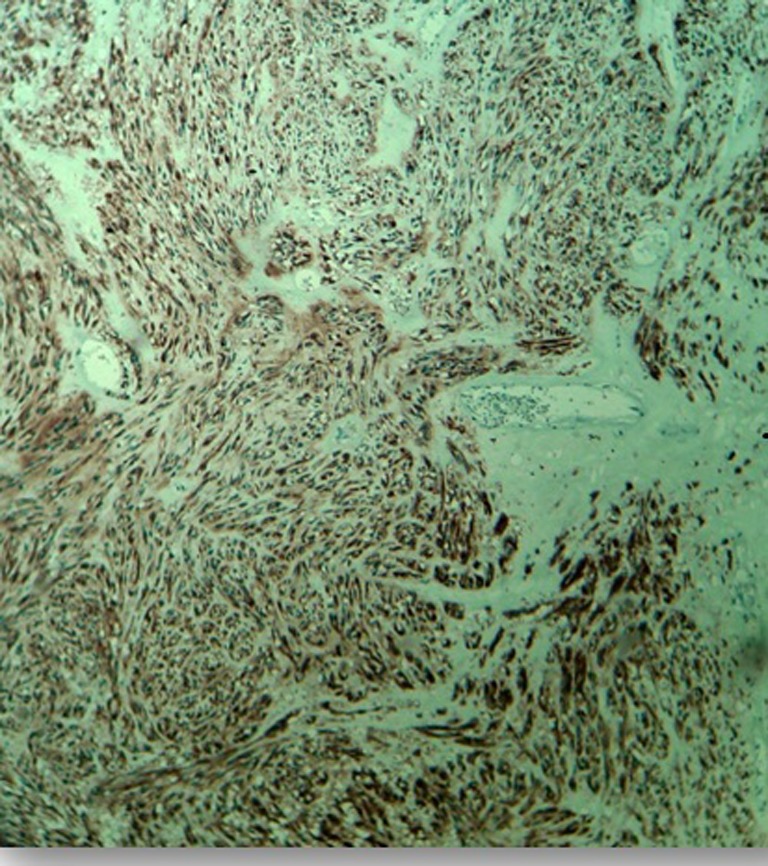


**PS-01-034**

**Differential diagnosis of gastrointestinal stromal tumors: Sampling cannot be overemphasized**

O. Daum ^*^, K. Peckova, M. Michal

^*^Faculty Hospital Plzen, Sikl’s Dept. of Pathology, Czech Republic

**Objective:** To highlight an important pitfall in the differential diagnostics of mesenchymal tumors of GIT.

**Method:** 79-years-old man underwent a right-sided hemicolectomy due to intestinal obstruction. The resection specimen was routinely processed for histological investigation. In the mesocolon, 17 lymph nodes were found.

**Results:** Grossly, the tumor sized 13 × 15 × 13 cm showed the typical “fish-meat” sarcomatous appearance. Histological investigation confirmed sarcomatous morphology of the tumor composed of spindle-shaped cells positive only for vimentin and focally for KIT. However, 2 lymph nodes were infiltrated by adenocarcinoma. Furthermore, both KIT and PDGFRA were wt. Therefore, the whole tumor mass was processed in 86 paraffin blocks and finally a focus of adenocarcinomatous differentiation (1 cm in diameter) was found.

**Conclusion:** Sarcomatoid carcinoma is a great mimicker of GIST. Extensive sampling, although primitive, is a more efficient tool in these cases, than modern sophisticated diagnostic methods.

**PS-01-035**

**Syncronous adenocarcinoma and gastrointestinal stromal tumor of the stomach: Report of a case**

C. I. Bassorgun ^*^, K. Balaban, M. A. Turkoglu, G. O. Elpek

^*^Akdeniz University, Pathology, Antalya, Turkey

**Objective:** “Collision” tumours consist of different neoplasms coexisting within a single lesion. We describe an exceptional case of a combined gastric adenocarcinoma and gastrointestinal stromal tumor(GIST).

**Method:** We report the case of a 66-year-old male patient who was diagnosed with the continius development of a GIST and adenocarcinoma of the stomach, in which the carcinoma cells focally invaded the GIST.

**Results:** On histopathologic examination, two completely different tumors were recognized in the stomach. One tumor was a moderatly differentiated adenocarcinoma characterized by glandular structures associated with lymphoid infiltration of the stroma. The other tumor was found to have proliferated in the wall of the stomach, with diffuse palisade spindle-shaped cells. It was diagnosed with the continius development of a GIST and adenocarcinoma of the stomach, in which the carcinoma cells focally invaded the GIST.

**Conclusion:** Gastric collision tumors are uncommon, and the combination of adenocarcinoma and GIST is extremely rare. According to the literature reviewed to our knowledge there are a few reported cases of synchronous occurrence of adenocarcinoma and gastrointestinal stromal tumors of stomach.

**PS-01-036**

**Gastrointestinal stromal tumor risk grade evaluation criteria relationship with disease progression**

L. Poskiene ^*^, D. Pangonyte

^*^LUHS, Pathology, Kaunas, Lithuania

**Objective:** To determine gastrointestinal stromal tumor (GIST) risk grade distribution based on tumor size and mitotic activity in different area (20, 22 and 50 high power fields (HPF)) and to set optimal area for mitosis counting.

**Method:** 131 of GIST were studied. Mitosis counted in the 20 HPF (5 mm2) according to WHO and ESMO recommendations; 22 HPF (5,3 mm2) according to data of Miettinen- Lasota/Armed Forces Institute of Pathology; 50 HPF (12,3 mm2) according to data of other studies and recommendations. Mitotic count were divided: I group ≤ 5, II group–6–10, III group > 10 mitosis. Risk grade was established. Outcome data consisted of recurrence or distant metastasis.

**Results:** The distribution of mitosis groups were identical in the 20 and 22 HPF. There were more cases in the III group of 50 HPF compared to 20 HPF (*p* = 0.03). While assessing the degree of risk, based on tumour size and mitoses, there were more patients that belonged to the high risk group, evaluating mitoses 50 HPF (*p* = 0.003), but the progression of the disease in both groups was the same.

**Conclusion:** The area of 20 HPF (5 mm2) is sufficient for mitotic assessment. One-third of the patients belong to high risk group and only in this group 31,7 % patients disease progressed (13 of 41).

**PS-01-037**

**Primary extra gastrointestinal stromal tumors**

A. C. Iorgescu ^*^, E. Stoica-Mustafa, C. Pechianu, V. Tomulescu, I. Popescu, V. Herlea

^*^Fundeni Clinical Institute, Pathology, Bucharest, Romania

**Objective:** Gastrointestinal stromal tumors (GISTs) are mesenchymal neoplasms of the gastrointestinal tract which express most frequently KIT protein detected by immunohistochemistry stain for CD117 antigen. Outside the gastrointestinal tract they are called extra-gastrointestinal stromal tumors (EGIST) and they bear the same histopatological and immunohistochemical aspects as their counterparts in the gastrointestinal wall. EGIST are rare neoplasms most of them being detected on the omentum, mesentery and retroperitoneal.

**Method:** We report a series of ten cases of extra-gastrointestinal stromal tumors, all diagnosed and treated in Fundeni Clinical Institute between 2005 and 2012.

**Results:** Our patients were 5 females and 5 males with ages between 34 and 74. Six tumors were located retroperitoneal, two in the mesentery and one peritoneal, most of them being high risk forms. IHC tests have been performed and several correlations have been made.

**Conclusion:** Primary EGISTs are rare, some of them with an aggressive behavior and a very poor prognosis. More data is needed for a proper characterization of the pathogenesis, prognosis and treatment options.

**PS-01-038**

**The prognostic value of angiogenesis in Gastro-Intestinal Stromal Tumors (GIST)**

R. Basilio-de-Oliveira^*^, V. L. Pannain, P. E. Portari, C. A. Basilio-de-Oliveira

^*^UNIRIO, Pathology, Rio de Janeiro, Brazil

**Objective:** To assessment the angiogenesis by immunohistochemistry with vascular endothelial growth factor (VEGF), intratumoral microvascular density (MVD) by CD105 and CD31 and the Ki67 proliferation index.

**Method:** The immunohistochemical study (CD34, CD105, KI67 antibodies) was performed in 54 GIST.

**Results:** GIST showed the following Miettinen’s risk: 3 no risk, 4 very low, 10 low, 14 intermediate and 23 high. Of the 23 tumors with Ki67 > 5 %, 19 showed a reduction in overall survival. VEGF strongly positive was associated with a reduction in overall survival and its absence was found mainly in the tumors with a good prognosis (18/33). Both MVD of CD31 (>2.5 %) and the CD105 (>1.2 %) were related to reduced overall survival. All factors studied showed statistical significance in univariate analysis. Undergoing index Jacard, MDV CD105 showed the best result.

**Conclusion:** The angiogenic factors showed prognostic ability, especially the CD105, which combined with others such as Ki67 can bring important contribution in understanding the prognosis of this tumor.

**PS-01-040**

**Plexiform fibromyxoma of the stomach**

K. Stevanovic ^*^

^*^LNS, Pathology, Luxemburg, Luxemburg

**Results:** Plexiform fibromyxoma, also known as plexiform angiomyxoid myofibroblastic tumour (PAMT), is a very rare and benign mesenchymal tumour of the antral part of the stomach. We report one case of plexiform fibromyxoma in Luxemburg. A 21-year-old woman presented with symptoms of nausea and stomach pain for a year and a half. An esophagogastroduodenoscopy was performed and revealed a submucosal antral mass. A computed tomography scan of the abdominal region described an invasive tumour suspicious for malignancy. The patient underwent a partial gastrectomy. On the gross examination, the tumour had a multinodular but well circumscribed aspect and measured 6,5 × 3 × 3 cm. Histologically it was composed of bland-looking spindle cells embedded in a myxoid matrix rich in small, thin-walled vessels. The tumour infiltrated the mucosa, the muscularis propria and the subserous tissue of the antrum. The tumour cells expressed alpha-smooth muscle actin and CD10. No expression of CD117 (c-KIT) or CD34 was observed. The proliferation index estimated by Ki67 was low (<2 %).

**Conclusion:** We present here an interesting case of plexiform fibromyxoma in a young female patient. The main differential diagnosis is the myxoid type of gastrointestinal stromal tumour.

**PS-01-041**

**Primary gastric synovial sarcoma: Case presentation and review of the literature**

H. E. Torres Rivas ^*^, M. S. Fernández García, M. F. Fresno Forcelledo

^*^HUCA, Pathology, Oviedo, Spain

**Objective:** Five to 10 % of the soft tissue sarcomas are Synovial Sarcomas (SS), and only 10 % of these have an unusual location without compromising the limbs/joints. To date, 14 gastric SS have been reported. We have added a case and reviewed the published data.

**Method:** A 45 year-old male with a large gastric tumor rising from lesser curvature. Hematoxylin-eosin stain revealed a solid neoplasia composed by spindle cells, with elongated nuclei and well-defined cytoplasm.

**Results:** The immunohistochemical phenotype was consistent with SS (Focal positivity: Cytokeratin AE1/AE3 and EMA. Negative: CD34, c-Kit, Desmin and S100) The molecular study for specific SS translocation X:18 was positive.

**Conclusion:** Uncommon gastric location represents a challenge to pathologists in order to exclude a gastro–intestinal stromal tumor (GIST), especially when dealing with the monophasic SS subtype, because of their histologic similarities. The number of reported cases of gastric SS still remains small.

**Gross features of gastrectomy and neoplasia. (a) Polylobulated yellowish tumor mass, emerging from the lesser curvature, (b) Post-fixation court observing the solid-fiber pattern of the neoplasm.:** 
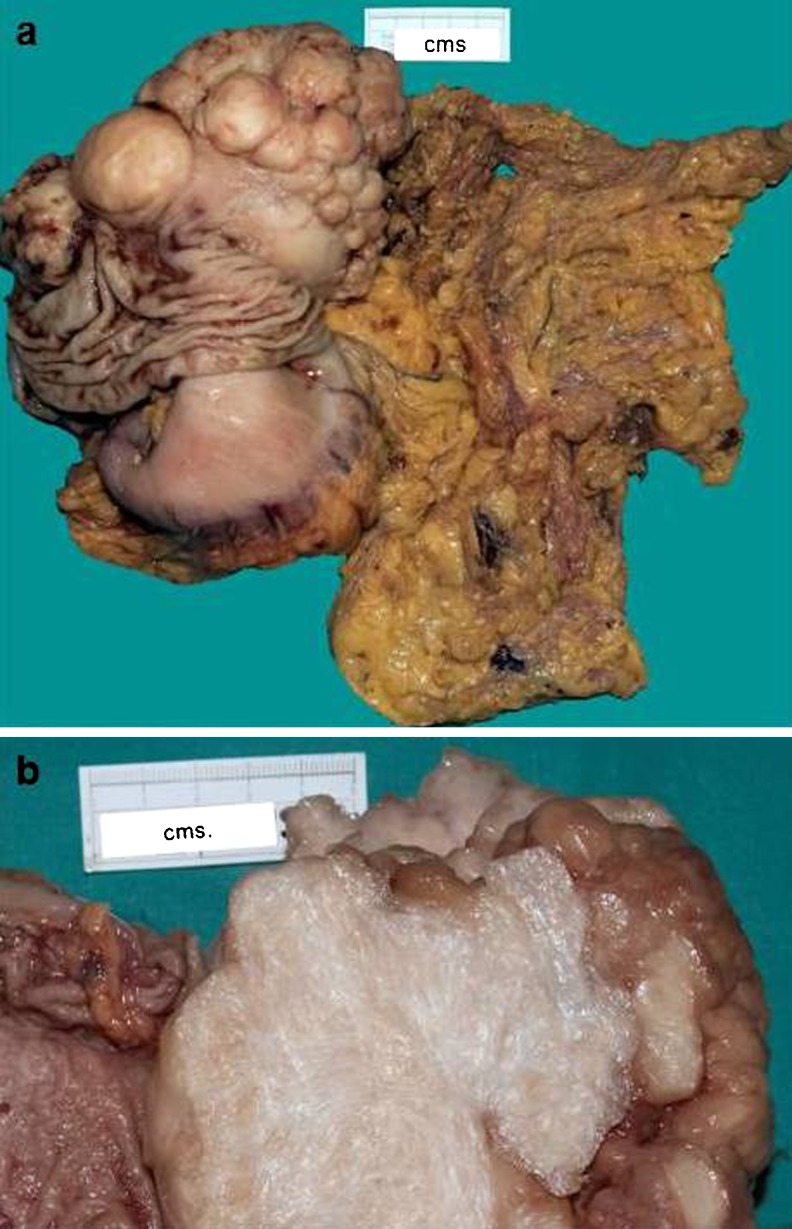


**PS-01-042**

**Gastrointestinal leiomyomas of muscularis mucosae–12 cases report with complete histological and immunohistochemical characterization**

F. Staniceanu ^*^, C. Popp, S. Zurac, G. Micu, A. Bastian, L. Nichita, E. Gramada, R. Andrei, C. Socoliuc, A. Stoica

^*^Colentina University Hospital, Pathology, Bucuresti, Romania

**Objective:** Gastrointestinal leiomyomas of muscularis mucosae (GILMM) are rare benign lesions usually representing incidental findings during endoscopic evaluation or surgical exploration of digestive tract. Although they are the most frequent type of digestive mesenchymal tumors, there are reported only small series of GILMM.

**Method:** We report 12 (male:female ratio 5:1) consecutive cases of GILMM (3 gastric and 9 colonic) with complete histological and immunohistochemical characterization. We performed routine hematoxylin-eosin and van Gieson’s stains and immunohistochemical assays for smooth muscle actin, S100, c-kit (CD117), CD34, Ki67, p53 and p21.

**Results:** Except one lesion, all were incidental findings: 2 necroptic findings (one woman with Cowden’s syndrome and multiple benign tumors and one man with cardiac disease and no other tumoral lesions); 2 surgical findings (one associated with an extranodal malignant large B cell lymphoma of the stomach) and 8 endoscopic findings (7 routine investigations and one for rectal bleeding associated with the leiomyoma).

**Conclusion:** Although rare, GILMM are an important entity, especially since the differential diagnosis with GISTs, leiomyosarcoma and neurofibromas of gastrointestinal tract has a great impact for the patient.

**PS-01-043**

**Mixed adenoneuroendocrine carcinoma of duodenum**

B. Andrejic ^*^, M. Živojinov, P. Miloševic, T. Boškovic

^*^Medical Faculty Novi Sad, Dept. of Histology and Embryology, Serbia

**Objective:** Mixed adenoneuroendocrine carcinoma (MANEC) is a rare, special type of tumour, characterised by an intimate mixture of two histologically different tumours, a neuroendocrine carcinoma (NEC) and adenocarcinoma.

**Method:** A 50-year-old man presented with a obstructive jaundice. An endoscopic retrograde cholangiopancretography (ERCP) with percutaneous transhepatic cholangiography (PTC) and implantation of endoprothesis was performed. Four days after ERCP/PTC a duodenum with tumor-like mass of the ampulla of Vater was resected along with liver metastases.

**Results:** Tumor showed two distinct, partly mixed components. The part of the tumor in the mucosal and submucosal layer was a well-differentiated adenocarcinoma. Tumot component, infiltrating mucosa, submucosa and muscular duodenal layer, consisted of a moderately to poorly differentiated round cells (about 60 % of entire tumor mass). Immunoprofile of this component was strikingly chromogranin-positive, moderately synaptophysin-positive with ki67 proliferation rate >50 %, confirming it as high grade NEC. Liver metastases had same immunoprofile. Staining for CK20 was strongly positive in adenocarcinoma and the negative in NEC. According to the current World Health Organization criteria the final pathological diagnosis was poorly differentiated MANEC of the duodenum.

**Conclusion:** Since follow-up data are scarce the prognosis of patients with MANECs is ill defined, and needs to be evaluated in further studies.

**PS-01-044**

**Metachronous metastized duodenal carcinoma in a Lynch syndrome patient: An unusual presentation**

J. Ferreira ^*^, R. Cabrera, C. Fidalgo, I. Rosa, P. Lage, M. J. Parreira, A. Bettencourt, I. Claro, A. Dias-Pereira, R. Fonseca

^*^IPO Lisboa, Serviço de Anatomia Patológica, Portugal

**Objective:** The prevalence of small intestine carcinoma in Lynch Syndrome, is higher than in the general population but duodenal carcinoma remains very rare. We report a case of duodenal adenocarcinoma in a patient with Lynch Syndrome.

**Method:** A 71-year-old-woman, with a family history of cancer, carrying a pathogenic mutation in the MSH2 gene, was followed at our institution since 2008. She had been previously diagnosed with an urothelial carcinoma (age 65) and an endometrial adenocarcinoma (age 69). In September 2012, as part of her annual follow-up, an upper gastrointestinal endoscopy revealed a fungating lesion in the duodenal ampulla. Abdominal CT showed a nodule in the nefrectomy space. The patient underwent a Billroth II gastrectomy and resection of the pararenal lesion. She is currently well.

**Results:** The surgical specimen contained a duodenal polypoid 3 cm tumor. Histology revealed a poorly differentiated adenocarcinoma arising in a tubulovillous adenoma. The tumor invaded the submucosa, had venous invasion and lymph node metastasis. The pararenal mass had identical histology. Immunohistochemical analysis revealed the loss of expression of MSH2 and MSH6 proteins in tumor cells.

**Conclusion:** According to medical literature, only 3 cases of duodenal adenocarcinoma in Lynch Syndrome have been reported to date.

**PS-01-045**

**Adenocarcinoma in solitary Peutz-Jeghers polyp in the duodenum**

L. Spasevska ^*^, V. Janevska, N. Jankulovski, B. Dukova, G. Ristovski, A. T. Zrmanovska

^*^Institute of Pathology, Skopje, Macedonia

**Objective:** Solitary Peutz-Jeghers-type hamartomatous polyps are rarely diagnosed in duodenum and have lower risk of malignancy. It is consider as a separate clinical entity from Peutz-Jeghers Syndrome (PJS) in relation with risk of cancer in other organ systems. There are few reported cases of focal adenocarcinoma within solitary Peutz-Jeghers-type hamartomatous polyps.

**Method:** The specimens from standard dissected operative material were formalin fixed and paraffin embedded, stained with hematoxylin-eosin, and immunohistochemically with smooth muscle actin.

**Results:** We present a case of 68 year old man with preformed pancreaticoduodenectomy (Whipple-resection) and finding of whitish polypoid lesion with nodular surface in duodenum measuring 5 cm in length. Routine dissection revealed malignant neoplasm infiltrating the wall of duodenum and the head of pancreas. Histopathology analysis showed polyp with branching smooth muscle bundles, which were immunohistochemically positive for smooth muscle actin, covered with hyperplastic mucosa and areas with adenomatous component. It also contained well differentiated adenocarcinoma with pushing border pattern involving pancreatic tissue. There was no lymphovascular invasion or metastasis in regional lymph nodes.

**Conclusion:** Duodenal solitary hamartomatous polyps although associated with low risk of malignancy, we have diagnosed a cancerous polyp. Patients who exhibit solitary Peutz-Jeghers-type hamartomatous polyps should be treated by surgical resection and need additional screening.

**PS-01-046**

**HepPar-1 and arginase-1 in small intestinal and ampullary adenocarcinoma**

S. Lagana ^*^, S. Hsiao, F. Bao, A. Sepulveda, R. Moreira, J. Lefkowitch, H. Remotti

^*^New York Presbyterian-Columbia, Dept. of Pathology and Cell Biology, New York, NY, USA

**Objective:** HepPar-1 and Arginase-1 are urea cycle enzymes used to distinguish hepatocellular carcinoma from other carcinomas. HepPar-1, but not Arginase-1, is known to stain normal small intestine. This study was performed to define the immunohistochemical staining patterns of HepPar-1 and Arginase-1 in adenocarcinomas of the small intestine and ampullary region.

**Method:** HepPar-1 and Arginase-1 immunostaining was performed on 20 non-ampullary small intestinal adenocarcinomas and 32 adenocarcinomas from the ampullary region. Ampullary adenocarcinomas were divided into intestinal morphology (15), pancreatobiliary (14), and unclassifiable (3). Non-neoplastic small intestinal mucosa and colorectal adenocarcinoma were also stained.

**Results:** HepPar-1 stained 12 of 20 non-ampullary adenocarcinomas with a median of 63 % of cells staining in positive cases. It also stained 11 of 15 ampullary carcinomas with intestinal morphology, median of 75 % of cells staining in positive cases. Two of 14 ampullary carcinomas with pancreatobiliary morphology were positive for HepPar-1. Arginase-1 was positive in two ampullary region carcinomas in 1 duodenal adenocarcinoma. Two of 22 colorectal carcinomas stained for HepPar-1 with none positive for Arginase-1.

**Conclusion:** HepPar-1, but not Arginase-1, is often positive in small intestinal adenocarcinoma and ampullary adenocarcinoma with intestinal morphology.

**PS-01-047**

**Histiocytic sarcoma of the small bowel**

R. Colling ^*^, J. Pawade, N. Wong

^*^University Hospitals Bristol, Dept. of Histopathology, United Kingdom

**Objective:** We present a case report of a 48 year old previously fit and well lady with right sided acute abdominal pain.

**Method:** Ultrasonography showed a 7 cm, highly vascular, soft tissue mass filling and compressing the lumen of the mid ileum. Computed tomography of this lesion was consistent with a gastrointestinal stromal tumour (GIST) and regional lymphadenopathy was also present. A small bowel resection was performed of a polypoid lesion with a fleshy and haemorrhagic cut surface.

**Results:** Histology revealed a solid tumour arising within the submucosa, invading through the muscularis propria and onto the serosal surface. The tumour was primarily composed of large ovoid cells with abundant eosinophilic cytoplasm and folded irregular nuclei. There were numerous giant cells present. The tumour cells showed positive immunostaining with CD163, CD68, lysozyme, CD45, HLA-DR, CD11c, vimentin and S100. The MIB-1 proliferation fraction was 40 %. There was negative staining for CD21, CD35, CD1a, langerin, HMB45, MelanA, EMA, ALK-1, CD56, CD30, CD3, CD2, CD20, CD79a, desmin, CD117, Dog1, and pan-cytokeratin. Seven lymph nodes were identified and all showed tumour involvement.

**Conclusion:** A diagnosis of histiocytic sarcoma was concluded and the patient is receiving radiotherapy, for which there has been a partial response so far. Histiocytic sarcoma is a rare, aggressive malignancy which may mimic a wide range of entities clinically and radiologically. The small bowel is an extremely rare site of origin, can be misdiagnosed as GIST radiologically and can present a challenging range of differential diagnoses to the cellular pathologist. The importance of accurate histological assessment and immunohistochemistry in these cases is therefore apparent.

Sunday, 1 September 2013, 09.30–10.30, Pavilion 2

**PS-02 Poster Session Endocrine Pathology**

**PS-02-001**

**Expression of Decoy receptor 3 in diffuse sclerosing variant of papillary thyroid carcinoma: Correlation with M2 macrophage differentiation and lymphatic invasion**

W.-C. Chang ^*^, J.-Y. Chen, C.-H. Lee, A.-H. Yang

^*^Mackay Memorial Hospital, Dept. of Pathology, New Taipei City, Taiwan

**Objective:** The diffuse sclerosing variant of papillary thyroid carcinoma (DSV-PTC) is a unique variant of papillary carcinoma characterized by extensive lymphovascular invasion of tumor cells. The lymphatic emboli contain tumor cells as well as macrophages. The aim of this study was to determine the relationship between the expression of Decoy receptor 3 (DcR3), recruitment of tumor-associated macrophages (TAMs) and lymphatic invasion in DSV-PTC.

**Method:** We retrospectively examined 14 cases of DSV-PTC using immunohistochemistry studies. The density of TAMs, lymphatic vessel density, lymphatic invasion, tumor emboli area and DcR3 expression were assessed. Statistical analyses were performed.

**Results:** The lymphatic tumor emboli contained relatively higher density of TAMs than stroma and classical PTC (CPTC) areas. In addition, the number of lymphatic invasion and tumor emboli area were positively correlated with the number of M2 TAMs. Moreover, DcR3 was expressed only in lymphatic tumor cells and squamous metaplastic tumor cells but not in macrophages and CPTC. In addition, the preferential expression of DcR3 in tumors was associated with higher levels of M2 TAMs and lymphatic invasion.

**Conclusion:** Our findings suggest that DcR3 expression in DSV-PTC tumor cells may promote the polarized macrophage differentiation towards the M2 phenotype. This phenomenon may further promote lymphatic invasion of DSV-PTC tumor cells.

**PS-02-002**

**Ectopic thyroid tissue in the adrenal gland: Report of two cases**

J. M. Cameselle Teijeiro ^*^, A. Romero Rojas, M. R. Bella Cueto, I. A. Meza Cabrera, A. Cabezuelo Hernández, D. García Rojo, H. Vargas Uricoechea

^*^Clinical University Hospital, Dept. of Anatomic Pathology, Santiago de Compostela, Spain

**Objective:** To describe two very unusual cases of ectopic thyroid tissue in the adrenal gland (ETTAG).

**Method:** We present the clinicopathologic, immunohistochemical, in situ hybridization, and molecular findings of 2 cases of ETTAG.

**Results:** The 2 cases presented as incidental cystic adrenal masses in adult females, one having a congenital hernia of Morgagni. The ETTAG was histologically indistinguishable from normal orthotopic thyroid tissue, and its follicular nature was confirmed by immunohistochemical positivity for thyroglobulin (Figure 1), thyroperoxidase, thyroid transcription factor-1, CK AE1/AE3, CK 7, pendrin, human sodium iodide symporter, paired box gene 8 and FOXE1, as well as positivity for the messenger RNA of the thyroglobulin gene by in situ hybridization analysis. No C cells (negativity for calcitonin, cromogranin and synaptophysin) were present. Neither BRAF nor KRAS mutations were detected with real-time polymerase chain reaction analysis. Further work-up did not show evidence of thyroid malignancy.

**Conclusion:** Although ETTAG pathogenesis remains unknown, the lack of C cells together with the coexistence of a congenital defect of the anterior diaphragm (hernia of Morgagni) in one of our patients could suggest an over-descent of medial thyroid anlagen derived cells in the origin of this heterotopia.

**PS-02-003**

**p40 immunoexpression in thyroid**

C. Eloy ^*^, R. Celestino, P. Soares, J. Cameselle-Teijeiro

^*^IPATIMUP, Dept. of Cancer Biology, Porto, Portugal

**Objective:** In thyroid, primary squamous cell carcinoma (SCC) and mucoepidermoid carcinoma (MEC) disclose squamous differentiation and p63 expression. SCC and MEC are thought to arise from solid cell nests (SCN) or from squamous metaplasia (SM) of the follicular epithelium. SCN and SM are often difficult to distinguish from each other and both express p63.p40 was reported to be more specific than p63 in the diagnosis of lung squamous cell carcinoma but its expression has not yet been reported in thyroid.

**Method:** We studied p40 immunoexpression in cases of incidentally found SCN (*n* = 10) and SM (*n* = 2), and cases of SCC (*n* = 2) and MEC (*n* = 2).

**Results:** p40 was expressed in all SCN, SM, SCC and MEC cases, and was not detected in the remaining follicular epithelium in any of the 16 samples.

**Conclusion:** In thyroid, p40 immunoexpression is, like p63 immunoexpression, a sensitive and specific tool for the detection of SCN and lesions with squamous differentiation; it does not help, however, in the distinction between SCN and SM, not even to understand their role in the histogenesis of SCC and ME. p40 expression is not exclusive from lung squamous cell carcinoma and appears to be a marker of differentiation rather than an organ specific marker.

**PS-02-004**

**Papillary microtumor of the thyroid gland: A validation of the Porto proposal**

J. M. Cameselle Teijeiro ^*^, M. J. Ladra González, R. M. Reyes Santías, I. Abdulkader Nallib, F. Barreiro Morandeira

^*^Clinical University Hospital, Dept. of Anatomic Pathology, Santiago de Compostela, Spain

**Objective:** Papillary microtumor (PMiT) was proposed in 2003 (Porto Cancer Meeting) to describe an apparently benign group of incidental thyroid carcinomas, ≤10 mm in adults. The aim of the Porto proposal was to avoid the word carcinoma as well as unnecessary treatments. We test this proposal.

**Method:** Clinicopathological study of 191 papillary microcarcinomas (PMiC) and 103 PMiT (2000–2008, Galicia, Spain), followed for 83.6(48–146) months.

**Results:** PMiC (191): 34 males, aged 49.37(18–84) years; tumor size 33(4–10) mm; thyroid capsular invasion 3.1 %, vascular invasion 3.7 %; total thyroidectomy 77 % and I131 39.3 %; lymph node metastasis in 14 patients (7.3 %), distant in 2(1.04 %), and 2 deaths. PMiT (103): 17 males, aged 51.72(20–80) years; size 33(3–10) mm; no capsular nor vascular invasion; total thyroidectomy 62.1 % and I131 4.9 %; no metastasis nor deaths. No sex differences comparing the proportion of PMiC/PMiT in relation to the number of thyroidectomies; no increase in prevalence regarding age. Rise in cancer thyroid incidence during period studied due to the PMiC.

**Conclusion:** PMiT, a clinically benign neoplasm, confirms the Porto proposal. Lack of sex differences in patients with PMiC/PMiT suggests biological differences with clinical thyroid cancer. PMiC is responsible for the increase in incidence of thyroid carcinomas.

**PS-02-005**

**A very unusual case of thyroid fat containing lesion: A nodule with papillary hyperplasia and focal atypia**

C. Cacchi ^*^, H. M. Arnholdt, A. Bartolazzi

^*^Klinikum Augsburg, Abt. Pathologie, Germany

**Objective:** Thyroid fat-containing lesions are not common. Sometimes however, cytological and architectural features suspicious for papillary thyroid carcinoma (PTC) can be detected in benign lesions.

**Method:** We report a case of 51 years old man referred to surgery for multinodular goiter, showing cystic nodules up to 3 cm. Formalin fixed paraffin embedded blocks were stained with routine Haematoxylin and Eosin and representative blocks were selected for immunohistochemistry.

**Results:** Microscopically one of them showed papillae with a delicate stroma with recognizable a prominent intralesional mature fat, having focal areas with enlarged, clear nuclei, with grooves and overlapping, without nuclear pseudoinclusions. The lesion didn’t show complete capsule and merges within the rest of thyroid tissue. The nodule expressed a positive reaction for Gal-3 and for HBME-1 immuostaining matching exactly the cells with cytomorphological atypicalitiy. On the basis of that we ruled out the diagnosis of PTC and concluded for an adenomatous nodule with fat-rich stroma and papillary atypical hyperplasia (focal cytological atypia) with molecular evidence of incipient transformation.

**Conclusion:** The presence of adipose tissue in the thyroid is rare but its presence doesn’t exclude malignancy per se. Therefore accurate integration of morphology and ancillary tests such immunohistochemistry are mandatory for a correct diagnosis.

**PS-02-006**

**VEGF, VEGFR1 and microvessel density in papillary thyroid cancer**

K. Ivanova ^*^, J. Ananiev, E. Onal, T. Vlaykova, M. Gulubova

^*^Trakia University, Faculty of Medicine, Dept. of Gen. and Clin. Pathology, Stara Zagora, Bulgaria

**Objective:** Vascular endothelial growth factor (VEGF) plays a key role in tumor angiogenesis. Up-regulated VEGF and VEGF receptor1 (VEGFR-1) expression in papillary thyroid cancer (PTC) have been shown to correlate with worse prognosis.

**Method:** Thirty nine PTC, 8 follicular (FTC) and 10 anaplastic (ATC) thyroid cancer samples were investigated immunohistochemically with antibodies against VEGF, VEGFR-1, CD31, CD1a and CD83-positive dendritic cells (DCs).

**Results:** We demonstrated that VEGF expression was significantly (*p* = 0.025) more prevalent in PTCs (92.3 %) than in ATC (60.0 %). CD31+ microvascular density (MVD) was statistically significantly higher (42.3+/−30.3) in PTCs than in FTCs (75.6+/−15.5) (*p* = 0.044) and in ATCs (34.3+/−20.8) (*p* = 0.407). PTCs with less CD31+MVD count showed better survival. In PTCs where VEGF expression in tumor cells was higher the immature DCs (CD1a+) were lower in number (2.53+/−4.0) in tumor stroma. Similarly, mature (CD83+) DCs were higher in number in tumor stroma in cases with negative VEGF expression in tumor cells (4.12+/−6.1) (*p* = 0.104).

**Conclusion:** In summary, the expression of VEGF and VEGFR-1 in tumor cells of PTCs was related to increased CD31+MVD, poor survival and with decrease of DCs. Therefore, except stimulating tumor angiogenesis VEGF might suppress DCs recruitment or proliferation in tumor stroma, and so to maintain the immune tolerance in thyroid cancer.

**PS-02-007**

**Tryptase- and chymase-positive mast cells in thyroid cancer**

E. Onal ^*^, K. Ivanova, J. Ananiev, A. Zdraveski, H. Stoyanov, T. Vlaykova, M. Gulubova

^*^Trakia University, Faculty of Medicine, Dept. of Gen. and Clin. Pathology, Stara Zagora, Bulgaria

**Objective:** The prognostic value of mast cells in the malignant tumors has not been yet demonstrated even though their hyperplasia was reported mainly at the periphery of neoplastic areas.

**Method:** We investigated immunohistochemically tryptase- and chymase-positive mast cells (MCT and MCC) in 31 papillary (PTC), 7 follicular (FTC), 7 anaplastic (ATC) and 8 oncocytic thyroid cancer (OTC). CD31+ microvessels (MVD) were also identified.

**Results:** We demonstrated that mast cell (MCT) density was higher in the stroma of ATC as compared to PTC and FTC. Converslly, in tumor stroma of FTC–MCC number was elevated, followed by ATC, PTC and OTC (FTC vs. PTC, *p* = 0.023). The MCC number in the stroma of PTC showed tendency for inverse correlation with CD31+ MVD (R = −0.309, *p* = 0.091) Patients with lower numbers of MCT in tumor stroma (*p* = 0.066) and in tumor border (*p* = 0.055) tended to have worse survival. In PTC, MCT and MCC numbers were significantly more as compared to the surrounding normal thyroid structure (*p* < 0.001). Mast cell role in VEGF expression and tumor development was discussed.

**Conclusion:** In conclusion, we may state that mast cells in thyroid cancer prevailed in those originating from folicular epithelium, and that their lower numbers may serve as a marker of worse prognosis.

**PS-02-008**

**Clinicopathologic features of primary thyroid lymphomas: A single center experience**

H. Aki ^*^, D. Uzunaslan, C. Saygin, H. Demir, A. Salihoglu, B. Ferhanoglu, N. Tuzuner

^*^Cerrahpasa Faculty of Medicine, Dept. of Pathology, Istanbul, Turkey

**Objective:** Analysis of clinicopathologic features of all PTL patients diagnosed at Cerrahpasa Faculty of Medicine.

**Method:** Pathology reports, hematoxylen-eosin and Bcl-2, Bcl-6, CD10, MUM1 and Ki-67 stained slides of seven patients diagnosed between years of 2000 and 2012 were retrieved and re-evaluated. Age, gender, year of diagnosis and follow-up data were recorded.

**Results:** Clinicopathologic features of 7 cases are summarized at Table 1. All of the patients had DLBCL as a histologic type. Three of them (3/7) were GCB subtype and four of them (4/7) were non-GCB. Male to female ratio was 2:5, and the median age at diagnosis was 52.8 years. Median follow-up was 40.8 months. Immunohistochemical features of all patients are summarized at Table 2. All specimens stained positively with CD-20, but none of them was Bcl-2 positive. Two patients were positive for CD 10, four had bcl-6, and three had MUM-1 positivity. All patients had a high proliferation index with Ki-67 immunostaining (mean, 92.14 ± 11.13).

**Conclusion:** Due to the rarity of PTL, current knowledge on this variant is limited to the data extracted from small case series. Large patient series and prospective clinical studies are necessary to elucidate their true clinical behavior and optimal treatment regimens.

**Tables:**

Table 1

**Table:** 

Case	Gender	Age	Age at diagnosis	Histologic type	Overall Survival
1	Female	47	2000	Peripheral Large B Cell	–
2	Female	82	2004	Diffuse Large Cell	22 month, exitus
3	Male	58	2005	Peripheral Large B Cell, aktive	7 year, alive
4	Male	34	2006	Diffuse Large B Cell	6 year, alive
5	Male	43	2007	B Cell Non-Hodgkin Lymhoma	1 month, exitus
6	Female	43	2007	Diffuse Peripheral Large B cell	5 year, alive
7	Female	63	2012	–	6 month, alive

Table 2

**Table:** 

Case	CD20	Bcl-2	Bcl-6	CD10	Mum-1	Ki67 (%)	Type
1	(+)	(−)	(−)	(+)	(−)	100	GMB
2	(+)	(−)	(−)	(−)	(−)	70	Non-GMB
3	(+)	(−)	(−)	(−)	(+)	100	Non-GMB
4	(+)	(−)	(+)	(+)	(−)	95	Non-GMB
5	(+)	(−)	(+)	(−)	(+)	100	Non-GMB
6	(+)	(−)	(+)	(−)	(+)	95	GMB
7	(+)	(−)	(+)	(−)	(−)	80–90	GMB

**PS-02-009**

**Metastatic tumors of the thyroid gland profile: A 15 years study**

D. G. Ciobanu Apostol ^*^, C. Preda, A. Grigorovici, R. Danila, L. Ionescu, C. Vulopoi

^*^UMF”Gr. T. Popa”, Dept. of Pathology, Iasi, Romania

**Objective:** To evaluate the clinical and pathologic impact of metastases to the thyroid gland (MT).

**Method:** We studied the pathologic features of 21 patients with MT over a 15-year period (1999–2013) diagnosed in the Department of Pathology of the St. Spiridon Hospital, investigated by fine-needle aspiration biopsy (FNAB), frozen section, routine histopathological exam and immunohistochemical staining.

**Results:** Eighteen (85.7 %) of the 21 patients presented with an enlarged mass in the thyroid. The average age was of 58.1 years, and sex ratio was F/M = 10/11. FNAB represented a method of preliminary diagnosis in a ratio of 71.42 %, and detected an 80 % malignancy rate. The MT could be identified macroscopically in 18 patients. Lesions were often solitary (9) rather than diffuse (5) or multiple (7). Microscopic examination revealed moderate or poorly differentiated tumors: squamocellular carcinoma (8), digestive and pulmonary adenocarcinoma (7), renal cell carcinoma (2), melanoma (1), and malignant lymphoma Hodgkin and nonHodgkin (3).

**Conclusion:** MT is uncommon and may be a diagnostic problem. The prognosis of a patient with a secondary malignant tumor to the thyroid is determined by the underlying primary tumor, and if metastatic disease is limited to the thyroid gland, surgery can prolong survival.

**PS-02-010**

**Cavernous hemangioma of the thyroid gland: A case report**

F. Yilmaz^*^, A. Kilitci, O. Büyükasik

^*^Abant Izzet Baysal University, Medicine Faculty, Bolu, Turkey

**Objective:** Hemangioma is a benign neoplasm of capillary proliferation and stroma that can be found in all organ systems. We report a case of a primary thyroid hemangioma, with nonspesific ultrasonographic(US) appearance. Cytologic dianosis was benign, final diagnosis was made at pathologic examination of he hemithyroidectomy specimen.

**Method:** Case report: A 24-year-old euthyroid female presented for evaluation of a symptomatic, slowly growing neck mass. She had no history of trauma, fine-needle aspiration(FNA) or other neck procedures. Ultrasound scan revealed a solitary hipoechoic nodule of the right thyroid lobe. Surgery in the form of right hemithyroidectomy was done

**Results:** At macroscopic examination, most of the right lobe was occupied by a reddish, solid, 3,8 cm lesion. Histologically, the tumor was encapsulated with fibrous tissue and contained many cavernous thin walled vessels, some which were thrombosed. The vascular nature of the structures was confirmed by immunohistochemistry: the cells were positive for the endothelial markers CD34, SMA, Factor8 related antigene and negative for TTF-1. The definitive diagnosis of a benign and isolated hemangioma of the right thyroid lobe was made.

**Conclusion:** Secondary hemangiomas of the thyroid after cytologic examination are possible, primary thyroid hemangiomas are rare. It is difficult to differentiate cavernous hemangiomas of the thyroid gland from other typical thyroid diseases due to the similar pattern they exhibit on ultrasound. An effort should be made to dissect the thyroid without rupture of these lesions in order to ensure a bloodless procedure.

**PS-02-011**

**Anaplastic Hürthle cell thyroid carcinoma with rhabdomyoblastic component: Case report**

S. Tatic ^*^

^*^Institute of Pathology, Belgrade, Serbia

**Objective:** Anaplastic carcinoma of the thyroid gland is highly malignant tumour. It may have sarcomatoid features and it can simulate many different sarcomas like fibrosarcoma, leiomyosarcoma or rhabdomyosarcoma

**Method:** We present the case of 49 years old male with anaplastic Hurthle cell carcinoma with rhabdomyoblastic component diagnosed after total thyroidectomy.

**Results:** Macroscopically, goitre, weighing 300 g, with a solid, partly cystic and haemorrhagic whitish node, 70 × 50 × 50 mm dimensions, occupying both, predominantly left lobe was described. Microscopically, tumour was composed of two components: epithelioid and mesenchymal. Oncocytic cells, in epithelioid component, were CK AE1/AE3+, CK7+, CK19+, TgA+, TTF−1 +, CK20−, CK18+, EMA +/− positive. Mesenchymal cells, with scant eosinofilic cytoplasm, showed intensive vimentine, desmin, INI-1 and myogenin positivity. Proliferative factor Ki67 was 10 time higher in mesenchymal than epitheloid component (50 % versus 5 %). Tumour infiltrates pseudocapsule and surrounding muscle and adipose tissue.

**Conclusion:** Hurthle cell thyroid carcinoma with anaplastic rhabdomyoblastic component is very rare and has a poor prognosis, which was confirmed in our case, where after total thyroidectomy a relapse occurred after 1 month.

**PS-02-012**

**Hyperfunctioning poorly-differentiated thyroid carcinoma: An extremely rare neoplasm**

D. Sakonlaya ^*^

^*^Thammasat University, Faculty of Medicine, Dept. of Pathology, Pathumthani, Thailand

**Objective:** To report a case of hyperfunctioning poorly-differentiated thyroid carcinoma with metastasis to bones.

**Method:** Case report of hyperfunctioning poorly-differentiated thyroid carcinoma with radiological and histological studies.

**Results:** A 43 year-old Thai female had suffered from hyperthyroidism with neck mass and significant weight loss for 1 year. Physical examination revealed multiple irregular, hard nodules involving right thyroid with right cervical lymphadenopathy. Thyroid function test showed thyrotoxicosis. Thyroid uptake was high normal. Functioning bone metastases at right scapula (size 9 × 8.7 × 8 cm) and right pelvis (size 12 × 7.7 × 7.5 cm) were demonstrated by thyroid scan with suspicious liver metastasis. Total thyroidectomy specimen revealed multiple, irregular, tan-white masses with extensive necrosis involving right lobe and isthmus. Histological examination showed multifocal infiltration of moderately to mildly dysplastic cells with endocrine features, predominantly arranging in trabecular with focal insular pattern and extensive necrosis. Vascular invasion and extrathyroidal extension was present. Scattered microfollicles with colloid were occasionally observed. Radical neck dissection specimen showed nodal metastasis and jugular vein invasion. Tumor cells were immunoreactive to: thyroglobulin(100 % of cells); TTF-1(100 %); AE1/AE3(80 %); Ki-67(30 %); cyclin D1(60 %); and Bcl2. After radioactive iodine treatment, patient still suffered from hyperthyroidism due to functioning bone metastases.

**Conclusion:** A case of hyperfunctioning poorly-differentiated thyroid carcinoma was reported.

**PS-02-013**

**Anaplastic thyroid carcinoma and primary thyroid lymphoma: The role of miRNA**

A. Fassina ^*^, F. Simonato, M. Siri, R. Cappellesso, F. Marino, L. Ventura, M. Crescenzi, M. Pivato, M. R. Pellizzo, M. Fassan

^*^University of Padua, Dept. of Medicine, Padova, Italy

**Objective:** Thyroid lymphoma (TL) and Anaplastic Thyroid Carcinoma (ATC) are rare, accounting for 5 % and 2 % respectively of thyroid malignancy. The majority of TL are of B-cell origin and present in elderly women; ATC are of the follicular origin, occur most frequently in men over 65, and are aggressive. The differential diagnosis of ATC and FL could be challenging and a precise diagnosis is therefore crucial. Recent studies evidenced a specific miR signature able to discriminate ATC from benign lesions.

**Method:** We retrieved cytological thyroid smears of 22 ATC and 14 TL and we extracted total RNA. We performed RT-PCR on the following miRNA panel: miR222, miR221, miR146b, miR26a.

**Results:** miR 221, miR 222 and miR26a were significantly over-expressed in ATC respect to TL (both *P* < 0.01). The ROC analysis estimated the optimal cut-off threshold for discriminating ATC vs TL, for all considered miRs, except for miR 146a, with a sensitivity level higher than 0.80. All the miR ROC thresholds were included in a classification tree analysis, and correctly identified 14/15 ATC (93 %).

**Conclusion:** Expression profiling of miR 146b, miR 26a, miR 221 and miR 222 is a promising tool for differential diagnosis of ATC and FL, also in scant cytological material.

**PS-02-014**

**BRAF V600E mutation is associated with aggressive behaviour in the papillary variant of papillary thyroid carcinoma**

A. Corominas-Cishek ^*^, M. Gonzalez, A. Pérez, V. Caamaño, G. Muñiz, N. Cerda, A. Gaafar, J. I. Lopez

^*^Cruces University Hospital, Dept. of Pathology, Barakaldo, Spain

**Objective:** To evaluate the impact of BRAF V600 mutations in the prognosis of papillary thyroid carcinoma.

**Method:** BRAF mutational status was analyzed in paraffin material of a series of 100 consecutive papillary carcinomas diagnosed by the same pathologist in our institution. The study was carried out using cobas® 4,800 BRAF V600 mutation test (Roche). Clinical and pathological data were obtained from the patient’s clinical histories.

**Results:** Thirty-one cases were mutated. Average age of patients with mutated tumors was 56 years (range, 45–75). Twenty-five mutated tumors displayed the conventional papillary phenotype (83 %), showed a tendency for extrathyroideal extension and presented a higher rate of loco-regional lymphoid involvement. Sixty-nine cases were non mutated, 45 (65 %) of them showing follicular histology. Non mutated group of tumors displayed a less aggressive clinical behavior, 89 % (62/69) of them being organ-confined lesions at the time of diagnosis (43 % were pT1 and 24 % were pT2).

**Conclusion:** BRAF V600 mutations are associated with conventional papillary variant of papillary thyroid carcinomas. In addition, this mutation is related with an aggressive clinical behavior.

**PS-02-015**

**Detection of Epstein-Barr virus-infected B lymphocytes with surface thyrotropin receptor antibodies in the peripheral blood from Graves’ disease patients**

K. Nagata ^*^, K. Higaki, Y. Nakayama, H. Miyauchi, Y. Kiritani, K. Kanai, M. Matsushita, I. Murakami, H. Kimura, K. Hayashi

^*^Tottori University, Dept. of Molecular Pathology, Yonago, Japan

**Objective:** Graves’ disease is an autoimmune hyperthyroidism caused by thyrotropin receptor antibodies (TRAbs). Because Epstein-Barr virus (EBV) persists in B cells and is occasionally reactivated, we hypothesized that EBV contributes to TRAbs production in Graves’ disease patients by stimulating the TRAbs producing B cells. Several reports have suggested a relationship between plasma cell differentiation of B cells and EBV reactivation. If EBV stimulates the antibody producing cell, the EBV must be present in that cell. We examined whether there were EBV-infected (EBV(+)) B cells with TRAbs on their surface as the membrane immunoglobulin (TRAbs(+)) in the peripheral blood of Graves’ disease patients.

**Method:** We developed a labeling system for detecting cell surface TRAbs. We stained the peripheral blood mononuclear cells from Graves’ disease patients by this labelling system and fluorescent EBER1 in situ hybridization. We sorted TRAbs(+) EBV(+) double positive (DP) cells by flow-cytometry and confirmed by confocal laser microscopy

**Results:** We observed granular signals of EBER1 in the nucleus, and red spot of TRAbs on the cell surface. TRAbs(+) EBV(+) DP cells really exist in peripheral blood from Graves’ disease patients.

**Conclusion:** These results suggested EBV had the possibility to stimulate the host cell’s TRAbs production

**PS-02-016**

**Clonal analysis of multifocal papillary thyroid carcinomas using human androgen receptor gene-based assay (HUMARA)**

T. Nakazawa ^*^, T. Kondo, N. Oishi, K. Mochizuki, T. Kawasaki, R. Katoh

^*^Yamanashi University, Dept. of Pathology, Chuo, Japan

**Objective:** Papillary thyroid carcinomas (PTCs) occasionally form non-continuous tumor foci in the same thyroid gland. It still remains controversial whether individual PTC nodules arise independently (multi-centric occurrence) or result from spread via lymph vessels (intra-glandular metastasis). To clarify which possibility gives an appropriate explanation, we investigated the clonal origin of multifocal PTC.

**Method:** We examined X-chromosome inactivation patterns of 32 PTC foci using a human androgen receptor gene-based assay (HUMARA) from 14 Japanese women. All PTC foci measured more than 3 mm and were macroscopically evident.

**Results:** HUMARA revealed to be informative in 13 cases and not in one. Of the 13 informative cases, 11 cases (84.6 %) showed inactivation of the same allele of the androgen receptor (AR) gene between two and three different PTC foci, suggesting a concordant X-chromosome inactivation pattern. Other two cases (15.4 %) revealed that inactivation of AR gene were not demonstrated in same allele, suggesting a discordant X-chromosome inactivation pattern. Statistically, these results indicated that the probability that each tumour shared the same progenitor cells was at least 69.2 % in this cohort.

**Conclusion:** The higher proportion of concordant X-chromosome inactivation pattern suggests that multifocal PTC can be made more frequently by intra-glandular metastasis than by multi-centric occurrence.

**PS-02-017**

**Implied risk of malignancy in a large series of thyroid fine needle aspiration cytology specimens based on the Bethesda system**

M. R. Bella Cueto ^*^, R. Orellana Fernandez, N. Combalia Soriano, M. Prenafeta Moreno, S. Barcons Vilaplana, S. Perez Aguilera, G. Marques Villacampa, M. R. Escoda Giralt, I. Capel Flores, M. Rigla Cros, M. Rey Ruhi

^*^Parc Taulí Sabadell H.U/UDIAT, Dept. of Pathology, Spain

**Objective:** To determine the risk of malignancy in a series of thyroid fine needle aspiration cytology (FNAC) specimens categorized by Bethesda system, with special interest in subdividing categories III and IV.

**Method:** In our Department we have been using for many years an equivalent system to Bethesda’s. We present the risk of malignancy obtained by cyto-histological correlation in a series of 896 satisfactory FNAC specimens obtained between September 1989 and March 2013, subdividing category III in Atypia of Undetermined Significance (AUS), Follicular Lesion of Undetermined Significance (FLUS) and Follicular Oncocytic Lesion of Undetermined Significance (FOLUS), and category IV in Follicular Neoplasm or Suspicious (FN), and Follicular Oncocytic Neoplasm or Suspicious (FON).

**Results:** Risk of malignancy has been: 1,26 % for category II; 20,17 % for category III; 48 % for category IV; 81,8 % for category V; and 100 % for category VI. Subdividing category III showed a risk of malignancy of 50 % for AUS; 16,90 % for FLUS; and 18,03 % for FOLUS; and subdividing category IV, the risk has been 55,76 % for FN, and 30,43 % for FON.

**Conclusion:** Implied risk of malignancy in our series has been higher than referred in reference literature except for category II, and there are significant differences in categories III and IV when they are subcategorized.

**PS-02-018**

**Can subclinical hypothyroidism influence lipid serum levels and thus increasing the cardiovascular disease risk?**

F. A. Oliveira ^*^, A. C. Silvestre Morais, H. S. Goetz, R. S. Goetz

^*^IPTSP/UFG, Setor de Patologia, Goiânia, Brazil

**Objective:** The present study described the prevalence of subclinical hypothyroidism (SCH) in a Brazilian population sample and the correlation between serum levels of Thyroid Stimulating Hormone (TSH) and lipid fractions.

**Method:** Medical records from a private practice in Goiania, Goias, Brazil, between February 2008 and October 2012, were retrieved and analyzed. Patients over 18 years old and who were not taking thyroid hormones, medications for thyroid diseases or to control lipid levels were included in the study. Data about age, sex, serum total cholesterol (CT), low density lipoprotein (LDL), high density lipoprotein (HDL), very low density lipoprotein (VLDL), triglycerides (TG) and TSH were collected.

**Results:** The prevalence of SCH found in the study sample was 8.4 %. No difference of prevalence between the sex groups or increase of TSH levels in the age ranges was observed. In the main group a significant and positive correlation was found between the TSH levels and the CT, LDL, VLDL and TG (*p* < 0.05). Comparing the euthyroid group with the patients with SCH a significant increase in CT, LDL and HDL was found in the latter group (*p* < 0.05).

**Conclusion:** This study showed that a higher level of TSH increases lipid levels resulting in risk of cardiovascular disease. Financial Support: FAPEG, IPTSP/UFG.

**PS-02-019**

**Cribriform-morular variant of papillary thyroid carcinoma in a patient with familial adenomatous polyposis. A case report**

A. Borda ^*^, E. Szasz, N. Berger, A. Loghin, R. Catana, M. Decaussin-Petrucci

^*^University of Medicine, Histology, Tirgu Mures, Romania

**Objective:** Cribriform-morular variant (CMV) is a rare subtype of papillary thyroid carcinoma (PTC) that usually occurs in the setting of familial adenomatous polyposis (FAP).

**Method:** Our patient, a 41-year-old woman, underwent a total colectomy for FAP 9 years ago. She presented at consultations for a bilateral thyroid mass, and mild swallowing disorders. Neck ultrasound identified multiple nodules in both thyroid lobes. Fine-needle-aspiration was refused by the patient. Consequently a total thyroidectomy was suggested.

**Results:** On macroscopy multiple whitish well-circumscribed nodules of various sizes were present both in left and right lobes. On microscopy the nodules showed a mixture of architectural features of which the cribriform and dense cellular morules were the most salient features. Most cells were elongated and their nuclei showed cytological and architectural features of PTC. The neoplasm did not show angioinvasiveness, extracapsular extension or lymph-node metastases. CK7+/CK20− immunohistochemical pattern excluded a colorectal origin. Although the thyroglobuline was negative, patient history, architectural/cytological and some immunohistochemical features (TTF1+, HBME1+ in the morules, galectine 3+, CK19+, oestrogen and progesterone positivity) guided the diagnosis to CMV of PTC.

**Conclusion:** This indolent variant of PTC should be considered in any situation in which a cervical mass appears in a patient with FAP.

**PS-02-020**

**Multinucleated giant cells significance: A study of 64 cases of papillary thyroid carcinoma**

N. Sabbegh Znaidi ^*^, S. Rammeh, A. Blel, Y. Zidi, F. Ferah, N. Kourda, R. Aloui, R. Zermani

^*^Hoptal Charles Nicolle, Pathology, Tunis, Tunisia

**Objective:** Multinucleated giant cells (MGC) are often detected in cases of papillary thyroid carcinoma (PTC). Their origin and significance, however, has not been established.

**Method:** A series of 64 cases of papillary thyroid carcinoma (PTC) including 33 cases of conventional CPT and 31 cases of follicular variant).

**Results:** Overall, we identified MGC in 21/33 (63 %) of the conventional CPT versus 8/31 (25 %) of the follicular variant (*p* = 0.0023). The mean age of patients having PTC with MGC was 39,4 ± 16,14 years versus 43,52 ± 15,86 (*p* = 0,32). The mean sizes of PTC in cases with MGCs was 29,85 ± 15,05 mm vs 43.05 ± 86,3 (*p* = 0.03). The cases of PTC with MGCs had higher multifocality rate 11/29 (37 %) vs 4/35 (11 %) (*p* = 0.012), nodal metastasis (13/23 (56 %) vs 8/23 (34 %) (*p* = 0.13) and extrathyroidal extension (12/29 (41 %) vs 5/35 (14 %) (*p* = 0,0001).

**Conclusion:** Cases of PTC with MGC are significantly more frequent in conventional CPT than in the follicular variant and have more extrathyroidal extension and multifocality.

**PS-02-021**

**Gastroenteropancreatic neuroendocrine tumors: A casuistic study**

F. Costa ^*^, A. Coelho, J. R. Vizcaíno

^*^Centro Hospitalar do Porto, Braga, Portugal

**Objective:** Gastroenteropancreatic neuroendocrine tumors (GEPNETs) represent a heterogeneous group of neoplasms with variable clinical findings and biological behavior, arising from several different neuroendocrine cell types. Many classification systems have been proposed for stratifying the malignant potential of GEPNETs. WHO updated its classification in 2010 and all GEPNETs, either G1, G2 and G3 (Neuroendocrine Carcinoma (NEC)) were categorized as malignant tumors.

**Method:** We collected and reviewed all the cases diagnosed as GEPNETs from the files of Centro Hospitalar do Porto from 2004 to 2012 and follow-up information was obtained. Immunohistochemical expression of Ki-67 labeling index was assessed and the tumors were recategorized according to 2010 WHO Classification.

**Results:** Fifty seven cases of GEPNETs were retrieved (age range 18–81 years, mean 55 years; male:female ratio of 1:1,28): esophagus 0 %, pancreas 21,1 %, stomach 26,3 %, small intestine 26,3 %, colorectal 12,3 % and appendix 14,0 %. The distribution of tumors according 2010 WHO Grading Classification was NET G1-70,2 %, NET G2-22,8 % and NEC-7,0 %. Malignant behavior was found in 15,0 % of NET G1, 69,2 % of NET G2 and 100 % of NEC.

**Conclusion:** Assessment of pathological parameters is crucial in understanding biological behavior of the tumor as well as predicting prognosis of patients with GEPNETs.

**PS-02-022**

**Shorter distance of Papillary microcarcinoma (Pmc) to the thyroid pseudocapsule and its relation with recurrence and metastases**

A. Gamboa-Dominguez ^*^, V. Rosas-Camargo, F. Candanedo-Gonzalez

^*^INCMNSZ, Dept. of Pathology, Mexico City, Mexico

**Objective:** Identify the effect of tumor distance to the thyroid pseudocapsule in microcarcinoma papillary (mcP).

**Method:** Tumor size, location and its distance to thyroid pseudocapsule was measured. Demographic, clinical, surgical, and 131I administration were obtained from charts.

**Results:** 1,343 thyroid specimens were evaluated and 511 PTC were identified. 59 (4.39 %) were mcP. Male/female ratio was 1:14 affecting mainly young patients, with multiple tumors (51 %) and involving bilateral lobes (30 %). Median tumor size was 7 mm and cervical metastases observed in 42 % without distant dissemination. Subtotal thyroidectomies were achieved in 50, 18(36 %) with lymph node dissection. All but four patients received 131I. Tumor contact with the pseudo capsule was documented in 13 patients. Ultrasonographic localization of a suspicious nodule was possible in 12. After 10 y follow-up, 55 patients had uneventful course; three presented local recurrences and one patient mediastinal/lung metastases. The four patients who had tumors in contact with the pseudo capsule and presented with local recurrences/distant metastases received 131I and underwent thyroidectomies with lymph node dissection (3 patients)/lobectomy(1 patient). mcP contact with pseudo capsule (*p* = 0.0016) and extra thyroidal disease at initial presentation (*p* = 0.02) were adverse factors.

**Conclusion:** mcP located close to the thyroid pseudo capsule is an adverse histologic finding, and should be explored in larger series using multivariate models.

**PS-02-023**

**The value of intra-operative frozen section in thyroid surgery. An analysis of 1110 cases**

R. Jouini ^*^, N. Abdessayed, W. Koubâa, A. M’rabet, M. Bel Haj Salah, I. Msakni, I. Harigua, O. Khayat, N. Labbene, E. Ben Brahim, A. Cahdli Debbiche

^*^Habib Thameur Hospital, Pathology, Tunis, Tunisia

**Objective:** To review our own experience with frozen section (FS) in thyroid surgery and to assess its value in the management of patients with thyroid disease.

**Method:** This retrospective study examined the results of 1,110 frozen sections of thyroid specimens analyzed over the 10 year period from 2003 through 2012and their correlations with the final histological examination. Deferred responses were not taken into account for statistical calculations.

**Results:** In our series, frozen section and final histopathological diagnosis agreed in 85,4 % and disagreed in 5,5 %. 9,1 % of the cases were deferred. The global specificity and sensitivity of frozen section analysis for all histological subtypes were 99,3 % and 64,7 % respectively. Its sensitivity for papillary carcinoma was 61,7 %, 83,3 % for follicular carcinoma and 100 % for anaplastic carcinoma. Discordances were due to 6 false-positive diagnoses and 55 false-negative (FN) diagnoses. 50 % of FN were represented by papillary microcarcinoma. The positive predictive value (PPV) of the frozen section examination was 94,4 % and its negative predictive value (NPV) was 93,9 %.

**Conclusion:** Our data supports the utility of intraoperative frozen section in the confirmation of malignancy of thyroid nodules. It’s correlated with a high degree of specificity and an acceptable rate of sensitivity. Most of the discordance between frozen section and final histopathological diagnosis was explained by papillary micocarcinoma.

**PS-02-025**

**Poorly differentiated carcinomas of the thyroid gland: A review of a series of 65 cases**

F. Z. Benserai ^*^, S. AIT Younes, F. Benserai, F. Asselah

^*^Mustapha Bacha Hospital, Algiers, Algeria

**Objective:** Controversial entity Spectrum heterogeneous of variants

**Method:** Review of 65 consecutive cases diagnosed at the departmentof Histopathology from 1997 to 2012 IHC selected cases (LSAB/Tg, CT,ChromogranineA, KI67,TP53, cycline D1, TTF1, Bcl2, Ecadherine)

**Results:** Invasive thyroid carcinomas 569 cases/Poorly differentiated carcinomas 65 cases (11,4 %) Clinical features: Rapidly growth masses or history of recent growth in a long multinodular thyroid. 44 womenF and 21 men, median age at the diagnosis (49 to 80 old years) The tumours measured 3 to 14 cm (m: 8 cm). Three different histologic patterns insular (37 cases) trabecular (15 cases) and solid (17 cases). Immunophenotypic profile All cases are negative for calcitonin, chromogranin and variable positivity for thyroglobuline.

**Conclusion:** A histologically poorly differentiated carcinoma subset Showing insular, solid or trabecular patterns predominant has distinct morphological and clinical features. This features often present as high stage tumours with a clinically aggressive course. No morphological or immunophenotypic parameters were of prognotic significance. This carcinomas can occur de novo or preexisting well differentiated carcinomas.

**PS-02-026**

**Solid variant of papillary carcinoma: Clinicopathologic features in the Algerian population retrospective study of 30 cases**

F. Z. Benserai ^*^, S. Ait Younes, R. Kassa, D. Chilla, F. Asselah

^*^Mustapha Bacha Hospital, Algiers, Algeria

**Objective:** Study of Characteristics: epidemiological, morphological, scalable and prognostic contribution of immunohistochemistry

**Method:** Revaluation of 30 cases of solid variant of papillary carcinoma collected from January 2005 to January 2013.

**Results:** 4 % of papillary carcinoma. This rare variant was found in 30- of our patients. These are 22 women and 08 men. The average age of patients was 29.5 years _G extreme ages of 14–75 years_G. Half of patient had presented pulmonary and bone metastasis. The tumor developed on a hyperthyroidism, with a toxic multinodular or Graves’ disease. The average tumor size was 4 cm in diameter _G 2.5 cm–8 cm _G with extra thyroidal extension for all patients The histological appearance is specific; characterized by solid sheets and typical nuclear of papillary cancer with wide areas of necrosis.

**Conclusion:** This variant of papillary carcinoma is dominated by solid sheets of tumor cells with typical nuclear features of papillary carcinoma, is a rare but aggressive.

**PS-02-027**

**Are RET and HRAS mutation status in medullary carcinoma and its metastasis always homogeneous? Preliminary results**

C. Cacchi ^*^, H. M. Arnholdt, C. J. Haas

^*^Klinikum Augsburg, Abt. Pathologie, Germany

**Objective:** The aim of this study was to investigate the prevalence of the mutational status (RET and HRAS) in medullary carcinomas (MTC) and their metastases.

**Method:** We retrieved 11 cases of MTCs (5 sporadic, 4 hereditary and 2 still unclear) with lymph node metastasis from 2000 to present. Microdissection of representative tissue was performed then DNA was extracted and, after PCR-amplification, the mutational Status of RET (p.M918T) and HRAS (codon 61) was determined by Sanger-sequencing.

**Results:** We found HRAS mutation neither in primary tumor nor in the lymph node metastases. One case of sporadic MTC and an unclear MTC showed a RET (p.M918T) mutation in both primary tumor and metastasis. In one case o RET wild type MTC the lymph node metastases showed a mutation. Morever in a case of multiple metastases by a still unclear MTC, two metastases were wild type and one of them was mutated.

**Conclusion:** HRAS mutation is reported to be common in sporadic MTCs lacking RET mutation, however our preliminary results showed no mutation in these cases. Interestingly, the detection of a discordant RET mutation status between primary tumor and metastases indicate that the possibility of heterogeneity may be observed.

**Medullary Carcinoma (H&E):** 
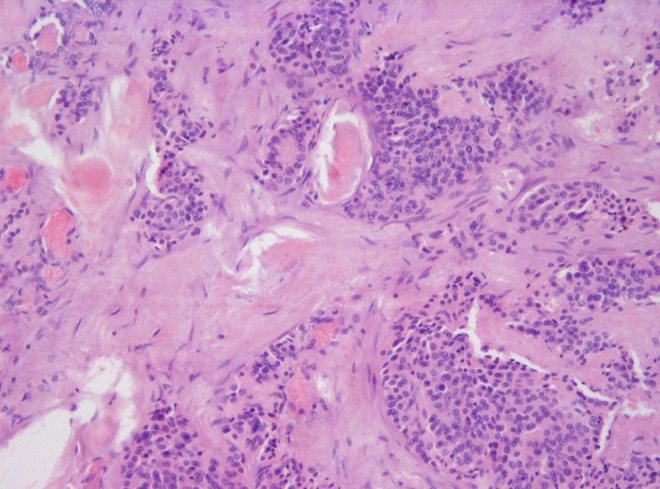


**PS-02-028**

**Predictive value of the Bethesda System Reporting for Thyroid Cytopathology (BSRTC): Should we keep categories III and IV?**

R. Silva ^*^, H. Barroca, I. Amendoeira, C. Eloy

^*^Centro Hospitalar de São João, Dept. de Anatomia Patologica, Porto, Portugal

**Objective:** BSRTC establishes 6 diagnostic categories associated with a determined risk of malignancy. Our aim was to determine the predictive value of BSRTC in Hospital de Sao Joao.

**Method:** We collected all the cases of thyroid cytology performed during 2011. The cases were classified according the BSRTC and the cytological diagnoses were compared with those made by the histological examination of the respective surgical specimen.

**Results:** Of the 1,384 identified patients, 307 underwent surgery. The thyroid cytology of the 307 patients was classified as: 37(12.0 %) I-Unsatisfactory; 125(40.7 %) II-Benign; 36(11.7 %) III-Atypia of Undetermined Significance/Follicular Lesion (AUS/FLUS); 30(9.8 %) IV-Follicular Neoplasm; 12(3.9 %) V-Suspicious for Malignancy; and 67(21.8 %) VI-Malignant. The percentages of cases in which a histological diagnosis of malignancy was made were the following for each diagnostic category: 18.9 % of the I-Unsatisfactory, 3.2 % of the II-Benign, 44.4 % of the III-AUS/FLUS, 40.0 % of the IV-Follicular Neoplasm, 83.3 % of the V-Suspicious for Malignancy and 100 % of the VI-Malignant.

**Conclusion:** In our series, the BSRTC had a positive predictive value of 1.00 and a negative predictive value of 0.97. The sensitivity was 0.94 and the specificity was 1.00. The similar malignancy risk for group III-AUS/FLUS and IV-Follicular Neoplasm reinforces the impression that it may prove useless keeping both categories.

**PS-02-029**

**Diffuse lipomatosis of thyroid: A case report**

J. Costa ^*^, J. Pardal, V. Máximo, F. Gonçalves, C. Eloy

^*^Centro Hospitalar S. João, Serviço de Anatomia Patológica, Porto, Portugal

**Objective:** Diffuse lipomatosis of thyroid (DLT) is a rare and benign condition characterized by infiltration of the gland by fat tissue.

**Method:** We report the case of a 46-year old male with hypothyroidism that underwent partial thyroidectomy at age 3. Now, he presented a slow growing mass in the neck. Ultrasound examination disclosed a solid, hypoechoic mass in the right thyroid region that measured 15.0 × 6.5 × 4.9 cm. Fine-needle aspiration biopsies were inconclusive. The patient underwent excision of the mass that weighted 250 g, was yellowish and displayed a lobulated appearance.

**Results:** Histologically, it was composed by thyroid parenchyma extensively infiltrated by mature adipocytes without atypia. Congo red staining was negative. SDHA immunostaining highlighted the mitochondrial content in follicular cells while SDHB was not expressed. Mitochondrial DNA was sequenced and no mutations were found in any of these genes. The diagnosis of DLT was based on the morphological similarity observed in the specimen of the first surgery (43-years ago) and in the last one.

**Conclusion:** DLT must be distinguished from others conditions with fat infiltration such as amyloid goiter, adenolipoma and papillary carcinoma. DLT may be related to non-structural molecular mitochondrial alterations at variance with multiple symmetric lipomatosis that can be associated with mitochondrial DNA mutations.

**PS-02-030**

**Sarcoid reaction in thyroid and regional lymph node diseases. A peculiar form of tumour and non-tumour response in four cases**

A. Hens Perez ^*^, M. Moreno, A. Calvo, M. Añón, G. Muñoz, M. García, Mª d. C. Vázquez, D. Martinez

^*^Hospital Puerto Real, UG A Patológica Bahía de Cádiz, Puerto Real (cádiz), Spain

**Objective:** Sarcoidosis is a multisystemic idiopathic disease with clinicopathologic criteria well defined that rarely expressed in the thyroid environment. Sarcoid reaction, as a granulomatous non-systemic lesion similar to those seen in sarcoidosis, exceptionally involves thyroid gland. We report four cases of thyroid and regional lymph node sarcoid reaction in the context of tumour and non-tumour thyroid lesions

**Method:** We reviewed 2,535 thyroid and regional lymph node surgical specimens registered in our service in the last 15 years, finding four cases of thyroid or regional lymph node with sarcoid reactions, along with a case of systemic sarcoidosis thyroid involvement, which information is presented,

**Results:** We report four cases of women aged 38–57 years, two of them with a history of papillary thyroid carcinoma, whose main clinicopathologic findings are shown in the table below. All of them enjoy good health with no evidence of tumour or granulomatous disease

**Conclusion:** Although the association between sarcoid reaction and malignancy is known, a very few cases with papillary thyroid carcinoma are mentioned in the literature, being also exceptional the coexistence with other thyroid lesions or perithyroid lymph node involvement, like the four reported cases

**Patient Dates:**

**Table:** 

CASE	SEX	AGE	THYROID SARCOID REACTION	LYMPH NODE SARCOID REACTION	PAPILLARY THYROID CARCINOMA	CHRONIC LYMPHOCYTIC THYROIDITIS	NODULAR HYPERLASIA
1	♀	57	+	+	−	−	+
2	♀	43	+	+	+	+	−
3	♀	38	+	+	+	−	−
4	♀	55	+	−	−	+	+

**PS-02-031**

**Single parathyroid adenoma in a patient with secondary hyperparathyroidism: A case report**

D. G. Ciobanu Apostol ^*^, C. Cristea, L. Ionescu, G. Savin, A. Covic, R. Danila

^*^UMF”Gr. T. Popa”, Dept. of Pathology, Iasi, Romania

**Objective:** We report the case of a 57 year old women, referred to surgery due to non-dialysis dependant renal hyperparathyroidism.

**Method:** The pathology diagnosis consisted of frozen sections and routine histopathologic exam. Tissue sections were fixed in 10 % buffered formalin, embedded in paraffin, sectioned at 4-μm, stained with Hematoxylin and Eosin (HE), and trichromic Van Gieson (VG).

**Results:** An thorough open surgery neck exploration revealed an 3/2/1.5 cm nodular enlargement of the right inferior PT while the other PT glands had a normal aspect. After frozen section confirmation the enlarged right inferior PT was removed and a biopsy was taken from the right superior PT. On multiple sections the first sample presented a thin incomplete capsule separating the adenoma from a rim of uninvolved atrophic gland. Inside the nodule the adipocytes were absent, the pattern of growth was diffuse, mostly with dark chief cells. The second one contained normal parathyroid tissue with chief, oyphil cells and scattered adipocytes. The value of PTH normalised postoperatively.

**Conclusion:** Although a diffuse hyperplasia involving all PT glands is a feature of renal hyperparathyroidism, in this rare case the macroscopic and microscopic aspects revealed a single PT adenoma, resembling primary hyperparathyroidism.

**PS-02-032**

**Clear cell parathyroid adenoma**

W. Rekik^*^, A. Heifa, H. Nfoussi, R. Ben Hammouda, A. Zehani, I. Chelly, A. Kdous, S. Haouet, N. Kchir

^*^Tunis, Tunisia

**Objective:** Water-clear cell hyperplasia is a rare but well-documented cause of primary hyperparathyroidism. Parathyroid adenomas of water-clear cell type are exceptionally rare, and only six case reports are available at present in the medical literature.

**Method:** We report an additional case and the differential diagnoses are discussed.

**Results:** We report histologic findings of a clear cell parathyroid tumor in a 60-year-old patient. Ultrasound examination of the neck showed a mass region in the posterior aspect of the left lobe of the thyroid. The excised parathyroid was a large mass measuring 3.8 × 2.8 × 1 cm in diameter. It was soft, covered with a thin capsule, did not infiltrate the thyroid parenchyma, and showed no evidence of malignant process. Histopathological examination showed large polyhedral cells with well-defined cell membranes and clear cytoplasm with a small amount of eosinophilic granular material. These clear cells were positive for pancytokeratin and PTH immunohistochemical stains. These results favored a diagnosis of parathyroid Clear Cell Adenoma.

**Conclusion:** Parathyroid adenomas composed exclusively of water-clear cells are exceptionally rare. Thus, in spite of its low occurrence, this diagnosis must be considered after rejection of the most frequent parathyroid clear cell hyperplasia and parathyroid carcinoma, or depending of the location, clear cell thyroid tumor and clear cell renal carcinoma metastasis.
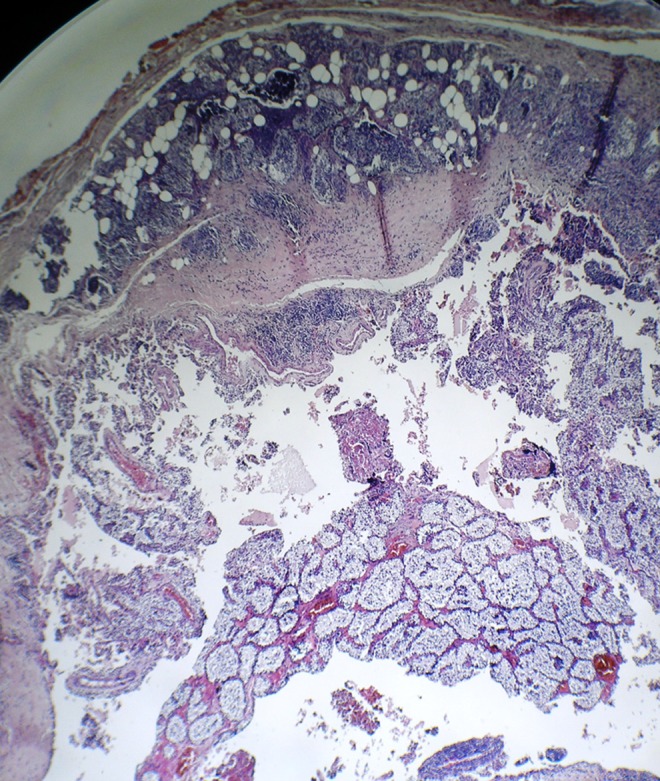


**PS-02-033**

**A case of ectopic Cushing’s syndrome: Neuroendocrine tumor of the cecum**

I. Voronkova ^*^, L. Gurevich, E. Marova, L. Rojinskaya

^*^Research Center of Endocrinology, Dept. of Pathomorphology, Moscow, Russia

**Objective:** Neuroendocrine tumors (NETs) of cecum are considered rare tumors. The prevalence of cecum’s NETs is 3,47 % of all gastrointestinal NETs. More rare these NETs have hormone’s hyper secretion. In the available literature, we did not find a case report about a NET of the cecum with ectopic ACTH secretion (EAS).

**Method:** A 52-year-old woman with EAS after computer tomography of the organs of the abdominal cavity the formation has been revealed in ilcocecal corner. Right hemicolectomy with the tumor and lymphadenectomy were made.

**Results:** Microscopic examination of the tumor showed NET of the cecum, with the infiltration of all its layers, valvula Bauhini and the mesenteric adipose tissue. The metastasis of a similar structure was found in 4 mesentery lymph nodes. The immunohistochemistry was positive for synaptophysin and chromogranin, ACTH, serotonin. There were 2 types of cells: one with the expression of serotonin, and the other ACTH. Tumor cells were negative for calcitonin, prolactin, corticotrophin-releasing, luteinizing and follicle-stimulating, parathyroid and growth hormones. Index of Ki-67 was equal to 0, despite the aggressive behavior of the tumor. That is why we have not shown the grade of the NET.

**Conclusion:** In the future we observed for the patient for 1 year with disease remission

**NET of the cecum with ectopic Cushing’s syndrome. Expression ACTH (Fig. A) and serotonin (Fig. B) in tumor cells ×20:** 
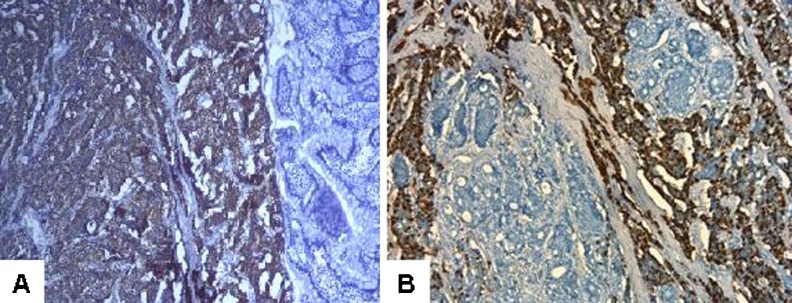


**PS-02-034**

**Galectin-3, cyclin D3 immunohistochemistry and tumour dimensions in oncocytic follicular lesions of the thyroid: Definitve results of retrospective study**

C. Cacchi ^*^, H. M. Arnholdt, C. J. Haas, M. Bruno

^*^Klinikum Augsburg, Abt. Pathologie, Germany

**Objective:** The differential diagnosis between oncocytic follicular adenomas (OAs) and oncocytic follicular carcinoma (OCs) may be challenging on the routine practice. The aim of this study was to evaluate the hypothesis that a combination of the factors tumour-diameter, immunohistochemical expression of Cyclin D3 and Gal-3 is helpful to distinguish between OCs and OAs.

**Method:** We retrieved, re-examined and reordered the diameter of 40 cases (14 OCs and 26 OAs) from1995 to 2007. Immuhistochemical analysis with Galectin-3 and Cyclin-D3 antibody was performed for each lesion.

**Results:** The mean value (in cm) of OCs was greater than in OAs (4.1 ± 2.3 vs. 2 ± 0. 8) (*P* < 0,001). OCs and OAs differ in the percent of positive cells for Galectin-3 25 ± 25 vs. 3 ± 8 (*P* < 0,001) and for Cyclin D3 46 ± 37 vs 8 ± 13 (*P* < 0,001). A combination of the two markers (Gal-3 + OR Cyclin D3+) demonstrated an excellent sensitivity (100 %) and a good specificity (85 %).

**Conclusion:** OCs and OAs differ from tumor dimension. Moreover, our study indicates that the association of Gal-3 and cyclin D3 has an excellent sensitivity and a good specificity, (100 % and 85 % respectively) and suggest these as helpful indicators to distinguish OCs from OAs.

**Cyclin D3 in Follicular Oncocytic Carcinomas:** 
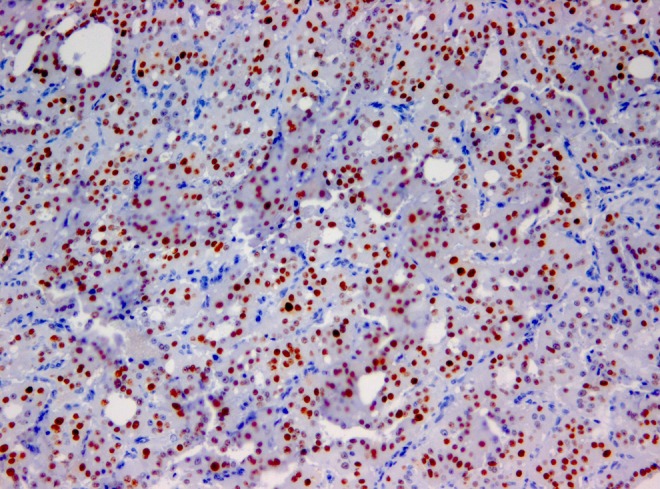


**PS-02-035**

**Sitagliptin treatment prevented pancreatic lesions evolution in a rat model of type 2 diabetes–proposal of antioxidant, antiapoptotic, anti-inflammatory and proproliferative mechanism**

C. Mega ^*^, H. Vala, J. Oliveira, F. Teixeira, R. Fernandes, E. Teixeira-Lemos, F. Reis

^*^Faculty of Medicine Coimbra, Lab. Pharmacology & Exp. Therap., Portugal

**Objective:** This study aimed to elucidate mechanisms underlying the protective effects of sitagliptin, a dipeptidyl peptidase-4 (DPP-4) inhibitor, against pancreatic lesions, in an animal model of T2DM.

**Method:** Male obese diabetic ZDF (fa/fa) rats, 20-weeks-old, were treated with vehicle or sitagliptin (10 mg/kg BW/day) for 6 weeks, and compared with lean control littermates (*n* = 8/each). Biochemical parameters and lipid peroxidation were evaluated in serum/blood/tissues. Pancreatic lesions were assessed semiquantitatively by routine histopathological and PAS staining methods. Expression in mRNA of apoptotic (Bax, Bcl2, caspase 9), inflammatory (TNFα, IL-1β, IL6), proliferative (PCNA) and angiogenic (VEGF) mediators was assessed by RT-qPCR. Immunohistochemical methods were used to confirm Bax/Bcl2 protein expression. Results are means ± s.e.m. ANOVA and Post-hoc tests were used (*P* < 0.05 was considered statistically significant).

**Results:** Sitagliptin treatment of diabetic ZDF (fa/fa) rats, ameliorated biochemical serum/blood parameters, pancreatic lipid peroxidation and diabetic lesions. Immunohistochemistry confirmed antiapoptotic effect observed by reduced expression of Bax/Bcl2 ratio by RT-qPCR. Caspase 9, IL-1β mRNA expression was decreased and proliferative and angiogenic factors overexpressed.

**Conclusion:** Sitagliptin, in this animal model of T2DM, may derive its protective pancreatic effect by antioxidant, antiapoptotic, anti-inflammatory and proproliferative/proangiogenic mechanisms.

**PS-02-036**

**Erythrocytes as target cells of diabetes types 1 and 2**

T. Pavlova ^*^, K. Prashchayeu, N. Pozdnyakova, V. Bashuk, A. Selivanova

^*^BelSU, Dept. of Pathology, Belgorod, Russia

**Objective:** Is to provide morphofunctional characteristics of erythrocytes s in case of diabetes mellitus (DM) (types 1 and 2).

**Method:** 37 patients from 28 to 71 years old. Light microscopy, scanning electron microscopy, scanning probe microscopy.

**Results:** In case of DM 1, only 10–15 % of discocytes were unchanged. 20 % discocytes had multiple excrescences, mulberry-shaped cells were also found among such discocytes. There were discocytes with crest ridges, spherical discocytes, including spherical erythrocytes with excrescences. About 5 % of red blood cells had the shape of deflated balls and were burned-out. In addition, both microcytes (5.49 μm) and macrocytes (8,36 μm) were observed, which was indicative of poikilocytosis. In average, cells (6.5 ± 0.5 μm) were smaller than those of the standard size (7.5). Erythrocytes of the patients with DM 2 were mostly in the form of dicocytes. Poikilocytosis appeared in a lesser degree than in the previous group and was indicated mostly by macrocytes (8.72 μm). Average size of cells was 7.0 ± 0.5 μm. Pic. 1

**Conclusion:** Erythrocytes are the target cells of DM. The data obtained about morphofunctional changes should be taken into account for therapy prescription, and their dynamics can serve as an option for therapy efficiency control.

**Erythrocytes Diabetes mellitus type 1. A. х 6,000 B.х 20,000 Scanning Electron Microscopy. C.D. Atomic force microscopy:** 
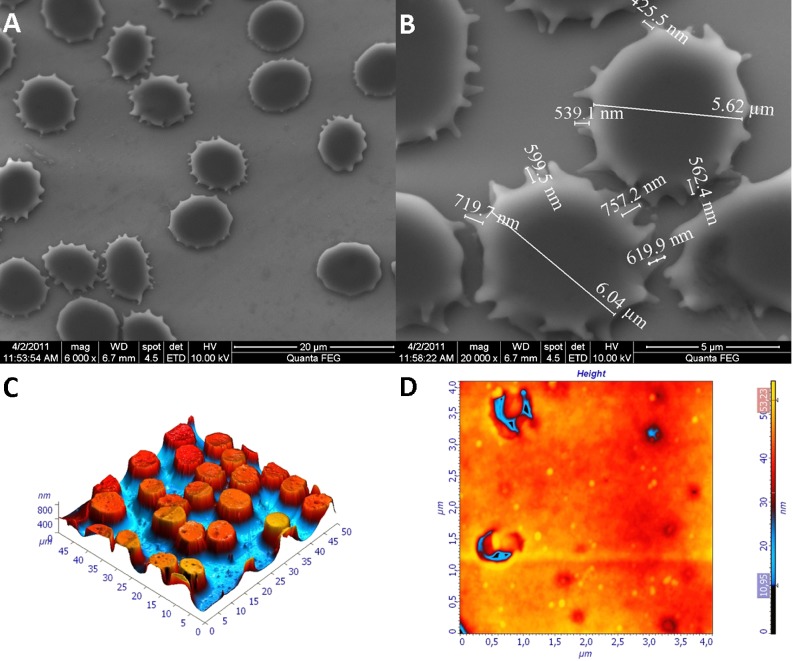


**PS-02-038**

**Ki-67 index assessment in Neuro Endocrine Tumors (NET): Image analysis and pathology routine compared**

M.-L. van Velthuysen ^*^, T. Korse, J. Sanders, F. Prins

^*^AvL, Pathology, Amsterdam, The Netherlands

**Objective:** We investigate the accuracy of the Ki-67 index assesment for grading NET.

**Method:** Ki-67 index of 82 tumor specimens of NET was assessed by two pathologists as in routine practice with the eyeballing method and twice by image analysis using ImageJ. For image analysis, photomicrographs of Ki-67 stained slides taken at a magnification of 20 and 40 were used.

**Results:** The correlation coefficient between pathologists was 0.897. Correlation for the measurements using image analysis was 0.956. Correlation of the pathologists with ImageJ, were 0.955 (20×) and 0.935 (40×) for one pathologist and 0.905 (20×) and 0.922(40×) for the other pathologist. If the Ki-67 index was translated to a grade (<3 % = grade 1, 3–20 % = grade 2 and ≥20 % = grade 3), pathologist 1 assigned a higher grade as compared with image analysis in 10 cases and pathologist 2 assigned a higher grade in 15 cases. Image analysis (20 vs 40) showed discordant grade in 5 cases. These discordances were due to differences in scoring around the cut-off values.

**Conclusion:** Assessment of Ki-67 index in routine practice by a pathologist correlates very well with Ki-67 index calculated by image analysis. However, grade assignment would be lower if image analysis is used.

**PS-02-039**

**Collagen-based dressings loaded with neurotensin repair ulcers in diabetic mice**

L. Carvalho ^*^, L. Moura, T. Ferreira, M. J. d’Aguiar, A. Dinis, E. Suesca, S. Casadiegas, M. Fontanilla Duque, H. C de Sousa

^*^Faculty of Medicine Coimbra, Inst. of Anatomical Pathology, Portugal

**Objective:** Diabetes foot ulcers (DFU) lead to amputations. Neuropeptides, substance P and neurotensin (NT) act as inflammatory modulators improving healing. Natural polymers, collagen and hyaluronic acid, have been used in wound healing applications. This work aimed to use collagen as a platform for the NT delivery into the wound site.

**Method:** Diabetes was induced by intraperitoneal injections of 50 mg/kg of streptozotocin during 5 days. Collagen, NT(50 ug/wound/per day), collagen loaded with NT(50 ug/wound/per day) or PBS were placed daily on two dorsal 6 mm excision wounds. Histological analysis(H&E and Masson’s Trichrome) of skin, at days 0, 3 and day of total wound clousure (df), was done.

**Results:** Day 0: hialin acellular collagen/higher number of cells in skin layers of diabetic mice compared to controls. Day 3: collagen+NT treatment did not contract skin in diabetics and collagen alone stimulated loose collagen synthesis. Day df: NT treatment stimulated PMN/lymphocytes to wound site while collagen dressing stimulated migration of fibroblast/higher deposition of loose collagen. NT+collagen treatment induced higher collagen deposition and recruitment of PMN in diabetics.

**Conclusion:** NT+ collagen dressing increased cellularization of skin tissue maintaining inflammatory status. Also, higher deposition of loose collagen was observed. In conclusion, NT+collagen dressing could be a possible wound dressing for human DFU treatment.

**PS-02-040**

**Expression of vascular endothelial marker CD 31 and carbonic anhydrase IX in carcinoid tumours**

K. Kuracinova ^*^, M. Štofíková

^*^Faculty of Medicine, Bratislava, Slovakia

**Objective:** Local hypoxia in tumour tissue and level of vascular supply are two interconnected dynamic factors playing a role in behaviour of the tumour. Neuroendocrine tumours in different locations were evaluated for density of vascular endothelial marker CD 31 and expression of carbonic anhydrase IX (CA IX), marker of tumor hypoxia.

**Method:** Immunohistochemical staining of differentiation antigen CD31 and CA IX membrane protein was performed on 25 neuroendocrine tumours with consequent morphometric analysis and comparison according to their location.

**Results:** Density of CD 31 positivity corresponded to level of vascularization of the tissue. The average vascularization in the gastrointestinal tract neoplasm was 3.52 %. Neuroendocrine tumours of lung tissue exhibit low degree of vascularity of the average value 1.15 %. From 11 lung carcinoids 81.8 % were positive for CA IX. Gastroenteropancreatic carcinoids expressed CA IX in a much lesser extent, from 14 examined specimens CA IX expression was detected in only 1 case.

**Conclusion:** Lung carcinoids have low density of vascularization and strong positivity of hypoxic marker. Gastrointestinal carcinoids have higher capacity to initiate neovascularization process than lung carcinoid. It is the task for future research to elucidate the causes of different CA IX expression and vascularization in carcinoids. Supported by grant UK/346/2013. Supported by ITMS 26240220052.

**PS-02-041**

**Neuroendocrine neoplasms of the Ampulla of Vater: Clinicopathological analysis of 9 cases**

L. Pérez Casanova ^*^, C. Meléndez, T. Serrano, C. Villabona, C. Valls, A. Teulé, J. Busquets, E. Condom

^*^Hospital de Bellvitge, Pathology, L’hospitalet de Llobregat, Spain

**Objective:** The aim of this study is to evaluate the prognostic significance of the ENETS grading, TNM staging system and the 2010 WHO classification in neuroendocrine neoplasms (NEN) of the ampulla of Vater (AV).

**Method:** Nine patients with NEN of AV treated with surgery were retrospectively analysed. Based on grading scheme with mitotic count and Ki67, the ENETS identifies three tumor categories: grade 1 (Ki67 <2 %), grade 2 (Ki67 2–20 %) and grade 3 (Ki67 >20 %). The 2010 WHO classification distinguish between well-differentiated neuroendocrine tumors (NET) grade 1 or 2, and poorly-differentiated neuroendocrine carcinomas (NEC) grade 3.

**Results:** Two cases showed areas with glandular differentiation and focus of adenocarcinoma were found in 3 cases. Seven patients had lymph nodes metastasis and only one case had liver metastasis. Six patients were classified as NEC and three patients as NET. Half of the patients with ampullary NEC (3/6) (two T2N1, one T3N1) died of disease (mean, 15 months). All three patients with ampullary NET had no evidence of disease after resection (mean follow up 4,6 years).

**Conclusion:** Both the ENETS grading system and the 2010 WHO classification are valid instruments for ampullary neuroendocrine neoplasms prognostic assessment but not TNM-based staging. The association with adenocarcinoma could suggest a common pathway in the carcinogenesis of these 2 neoplasms.

**PS-02-042**

**Tumor-to-tumor metastasis: Uterine leiomyoma with metastasis of neuroendocrine carcinoma**

A. Dema ^*^, M. Decaussin-Petrucci, S. Taban, D. Onet, N. Hrubaru, A. Vaduva, R. Cornea, D. Crstici, M. Derban, E. Lazar

^*^University of Medicine and Pha, Pathology, Timisoara, Romania

**Objective:** Metastasis of one tumor to another is a rare phenomenon with fewer than 200 reported cases. The aim of this paper is to describe the case of a 59-year-old woman with metastases of large cell neuroendocrine carcinoma (LCNEC) to a uterine leiomyoma.

**Method:** Nine months before the diagnosis, the patient underwent left lobectomy for thyroid nodules. During surgery preparation for thyroid nodules, a 3 cm pulmonary lesion was discovered, hepatic metastases and increased neuron-specific enolase, calcitonin and serotonin serum levels. While trying to identify the primary site of the tumor, a suspicious lesion was found in the uterus and the patient underwent hysterectomy.

**Results:** Gross exam revealed a uterine leiomyoma with poorly demarcated, hemorrhagic nodular areas. Microscopically, these areas were composed of undifferentiated large tumor cells, immunohistochemically positive for NE markers.

**Conclusion:** To our knowledge, this is the first reported case of metastases from LCNEC to a leiomyoma. The diagnosis of LCNEC can be very difficult and it requires additional clinical, laboratory, radiological and immunohistochemical information. This case emphasizes the difficulties in classifying a tumor without NE morphology but with serum and immunohistochemical markers of NE differentiation. Such a phenomenon (tumor-to-tumor metastasis) must be kept in mind to avoid diagnostic errors in unusual looking uterine tumors.

**PS-02-043**

**Renal cell carcinoma metastatic to pancreatic endocrine neoplasm: A second case described in world literature**

K. Bednarek-Rajewska ^*^, D. Breborowicz, A. Wozniak

^*^University of Medical Sciences, Chair of Clinical Pathology, Poznan, Poland

Metastatic tumors in the pancreas are rare but well documented events. The tumor- to- tumor metastasis within the pancreas is even more unusual possibility. We report a case of 64- year old male who presented with a tumor of the pancreatic head visualized in CT scan of the abdomen. He had nephrectomy 12 years prior the examination. The patient underwent partial pancreatectomy. Frozen section diagnosis of the pancreatic tumor was endocrine neoplasm. Histopathological examination of paraffin blocks revealed a tumor composed mainly of pancreatic endocrine neoplasm present mostly a the periphery. The central part showed lobules and tubules of clear cells surrounded by delicate branching vessels. The results of immunochistochemical stains were felt to be consistent with a metastatic renal cell carcinoma surrounded by pancreatic endocrine neoplasm and therefore representing an unusual case of renal cell carcinoma metastatic to pancreatic endocrine neoplasm.

**PS-02-044**

**Neuroblastoma in adults: A case series**

H. Sartelet ^*^, M. Decaussin, M. Latour, V. Combaret, R. Fetni, M. Peuchmaur

^*^CHU Sainte Justine, Dept. of Pathology, Montreal, Canada

**Objective:** Neuroblastoma is one of the most common malignancies in children. It most commonly originates in the adrenal medulla, but may arise anywhere within the sympathetic nervous system. Adult neuroblastoma is a exceptionaly rare and poorly recognized entity among tumors originating in the retroperitoneum and abdomen. We present a case series of four neuroblastomas in adults.

**Method:** The adult neuroblastoma group included two men and two women of 22–53 years of age (mean 34 years). Of the 4 cases, one tumor was situated in the retroperitoneum and three in the adrenal gland. One had stage 1 disease, one had stage 2 disease, and two had stage 4 disease at diagnosis.

**Results:** Two tumors were classified as poorly differentiated neuroblastoma, one as differentiated neuroblastoma and one as nodular ganglioneuroblastoma. All tumors presented a low mitosis-karyorrhexis index. These cases often showed immunoreactivity for multiple neural markers such as CD56, synaptophysin, neurofilament, and neuron-specific enolase. Fluorescence in situ hybridization analysis was also performed and showed no MYCN oncogene amplification.

**Conclusion:** Neuroblastoma should be considered as a diagnosis for adult patients with a suprarenal mass and, if confirmed, it should be classified and treated according to pediatric guidelines.

**PS-02-045**

**Algerian pediatric adrenal cortical neoplasms: An institutional retrospective study**

F. Z. Benserai ^*^, R. Kassa, D. Chilla, F. Benserai, F. Asselah

^*^Mustapha Bacha Hospital, Algiers, Algeria

**Objective:** Compare the histological criteria of malignancy in pediatric Adrenal Cortical Neoplasms used the Armed Forces Institute of Pathology (AFIP) score and Weiss’s score.

**Method:** An institutional retrospective review (2002 to 2013) identified 11 patients ˂ 19 years old with adrenocortical carcinoma (ACC) and adenomas (ACA).

**Results:** All the patients (6 girls, 5 boys) presented hormone-related symptoms. The tumors ranged in size from 3 to 15 cm (mean: 7.6 cm), the tumors ranged in weight from 25 to 990 g (mean:310 g). According to the Weiss’s score: 03 ACA, 08 ACC; According to the AFIP system: 02 benign, 02 uncertain malignant potential and 07 malignant tumors. There was reactivity with Vimentin, Melan A and Ki-67. All patients underwent adrenalectomy. The follow-up ranged from 10 years to 02 months.

**Conclusion:** Pediatric Adrenal cortical neoplasms are rare, Their biologic behavior are distinct. Pediatric ACA and ACC can exhibit overlapping morphologic features, which makes distinguishing benign and malignant adrenocortical tumors difficult. The AFIP proposed system may represents a useful method for assessing the behavior of adrenocortical neoplasms in pediatric patients.

**PS-02-046**

**A rare case of juxtadrenal schwannoma**

E. Souka^*^, E. Kyrodimou, Z. Kontogianni-Miller, A. Kostopoulou, E. Papaliodi

^*^G.H.A. G. Gennimatas, Dept. of Surgical Pathology, Athens, Greece

**Objective:** Schwannoma can rarely develop in the retroperitoneum (0.5–1.2 % of benign retroperitoneal tumors). We present an unusual case of juxtadrenal schwannoma and a mini review of the literature.

**Method:** A 68 year old asymptomatic female presented with a left adrenal incidentaloma, at abdominal ultrasonography. Physical and endocrinological examinations were unremarkable. She was diagnosed with a non-functioning left adrenal tumor on computed tomography and underwent left adrenalectomy. The macroscopic examination revealed an encapsulated, solid, fibroelastic, yellow-white tumor, in the periadrenal fat, measuring 4 × 3.6 × 3.3 cm. The adrenal gland was tumor free and normal in appearance. On microscopy the tumor was noninvasive and comprised of Antoni type A and B areas, a fibrous capsule and a lymphocytic cuff. The tumor cells were immunoreactive for S-100 and Vimentin. Ki-67 labeling (MIB-1antibody) of tumor cells was 1–2 %.

**Results:** The diagnosis was Juxtadrenal Schwannoma. A subgroup of the retroperitoneal schwannomas is located in the periadrenal fat (juxtadrenal schwannoma) and is often diagnosed preoperatively as non-functioning adrenal tumor. Macroscopically they are separate from the adrenal tissue, which appears normal. Microscopically and immunohistochemically, they are similar to the usual schwannomas. Differential diagnosis includes extra- gastrointestinal stromal tumor, solitary fibrous tumor, leiomyoma, neurofibroma, nodular fasciitis and schwannoma of the adrenal medulla.

**PS-02-047**

**Angiosarcoma of the adrenal gland: A case report**

A. Dimitriadi ^*^, E. Kyrodimou, C. Karabogias, G. Koutsonikas, T. Choreftaki

^*^General Hospital of Athens, Surgical Pathology, Greece

**Objective:** We report a rare case of primary adrenal epithelioid angiosarcoma.

**Method:** A 62 year old male patient presented with abdominal pain. Computed tomography and Magnetic Resonance Image revealed a large mass of right adrenal gland. The patient underwent adrenalectomy.

**Results:** Macroscopically, the mass had a brownish color with median diameter 10 cm, was cystic with extensive hemorrhage and central necrosis. Microscopically, neoplastic cells were recognized in limited areas with a diffuse pattern and sparsely formed tubules. The mitotic rate was up to 5/10 High Power Field. Immunohistochemically,these cells were reactive for vimentin,CKAE1/AE3,CK7,CD31. Differential diagnosis includes a variety of vascular neoplasms (primary and metastatic), carcinoma, melanoma, adenoma with extensive hemorrhage, as well as reactive processes with fibroblasts.

**Conclusion:** Angiosarcoma of the adrenal gland is a rare vascular that poses differential diagnostic dilemma with primary or metastatic tumor, especially when there is extensive hemorrhage in an adrenal mass.

**PS-02-048**

**Oncocytic adrenocortical tumors: Study of four cases**

E. Minaidou ^*^, A. Dimitriadi, E. Souka, E. Kyrodimou, T. Choreftaki, E. Papaliodi

^*^General Hospital of Athens, Surgical Pathology, Greece

**Objective:** We report four cases of oncocytic adrenocortical tumors (OACTs): two cases were malignant, one indeterminate and one was an adenoma, according to Weiss criteria (modified by Aubert).

**Method:** All patients, of age 50 to 62 years old, presented with non-functional “incidentalomas”. After resection, the tumors weighted 39–68 g with median diameter 5–9,5 cm.

**Results:** Microscopically, the neoplasms consisted of variably atypical cells with abundant granular eosinophilic cytoplasm and prominent nucleoli. Necrosis, vascular invasion, high mitotic index were not observed. Immunohistochemistry was performed for anti-mitochondrion, inhibin-A, Mart-1, SYP, CgA, Calretinin, Cam5.2, CKAE1/E2, EMA, Ki-67 (MIB-1), p53 and TopoII and histochemistry for reticulin.

**Conclusion:** OACTs are a very uncommon and heterogeneous group some of which may be overdiagnosed as carcinomas, as Weiss score 3 is attained easily given their innate morphology. In the last years, there is a big interest in these types of tumors as in their pure form are low grade tumors with a more indolent clinical course as compared with conventional adrenocortical carcinomas, while reticulin seems to help in non underestimating malignancy.

**PS-02-049**

**Adenomatoid tumor of the adrenal gland: Case report**

N. Chaleplidis^*^, C. Karabogias, E. Kyrodimou, G. Zografos, T. Choreftaki, E. Papaliodi, Z. Kontogianni-Miller

^*^General Hospital of Athens, Surgical Pathology, Greece

**Objective:** We present a case of an adenomatoid tumor (AT) of the adrenal gland.

**Method:** An incidental mass was found in the left adrenal gland of a 30-year-old male patient who underwent a Computed Tomography for abdominal pain. We received an adrenalectomy specimen, 6,5 cm in diameter, weighing 15 g. After sectioning, a circumscribed, whitish, soft, solid, focally micro-cystic tumor was recognized, 3,7 cm in diameter in the cortex. The rest of the adrenal gland retained its medullo-cortex architecture.

**Results:** Microscopically, the tumor consisted of anastomosing tubular, adenoid, solid, cystic and papillary formations, which extended to the peri-adrenal adipose tissue. They were lined by flattened or, mostly, epithelioid cells with abundant eosinophilic, focally micro-vesicular, cytoplasm. Cytological atypia, nuclear pleomorphism, or evident mitotic activity, were not observed. The tumor showed strong immunoreactivity for Calretinin, CKAE1/AE3, CK7, and Vimentin, weak and focal reactivity for CK5/6, and was negative for CD31, CD34, FVIII, CK20, EMA, CEA, Desmin, Inhibin, CD10, RCC, TTF-1, WT-1, p53, HBME-1. Proliferative index Ki-67 (MIB-1) was estimated at 2–3 %.

**Conclusion:** AT’s are benign tumors of mesothelial origin, rarely encountered extra-urogenitally, that have a broad differential diagnosis, including vascular neoplasms, mesothelioma and metastatic adenocarcinoma.

**PS-02-050**

**Ganglioneuroma of the adrenal gland: Report of two cases**

V. Theodorou ^*^, B. Christoforidou, P. Xirou, E. Goupou, N. Vladika, F. Patakiouta

^*^Katerini, Greece

**Objective:** Adrenal ganglioneuromas are rare, benign neoplasms of the primordial neural crest tissue.

**Method:** A 36-year old female presented with low-back pain and a 68-year old male with abdominal pain/constipation and weight-loss. Abdominal CT and MRI at both patients revealed a mass at left adrenal gland and adrenalectomy was performed. Grossly, both tumors (maximal diameter 12 cm and 6 cm) were well-circumscribed, encapsulated with homogenous, rubbery to gelatinous consistency and gray-white colour.

**Results:** Microscopically, both tumors consisted of spindle cells with wavy nuclei in a fascicular arrangement within variable amount of collagen admixed with single or small nests of mature ganglion cells. Myxoid stroma and mature fat tissue cells were also present. Immunohistochemically, in both tumors spindle cells were S-100 protein positive and ganglion cells were NSE, Synaptophysin and Neurofilament proteins positive.

**Conclusion:** Adrenal ganglioneuromas, due to their presentation as “incidentalomas” with non-specific clinico-radiological features, represent a pre-operative challenge. Surgical excision and histopathological confirmation are the gold standard for treatment and diagnosis.

**PS-02-051**

**Epitheliod angiosarcoma of the adrenal gland: A case report**

I.-P. Efstratiou ^*^, E. Pazarli, S. Pervana, D. Alataki

^*^Papageorgiou General Hospital, Dept. of Pathology, Thessaloniki, Greece

**Objective:** We report a rare case of a primary adrenal angiosarcoma.

**Method:** A 70-years-old woman presented with abdominal pain and sciatica leading to the discovery of an 11 cm diameter right adrenal mass. The tumor was compressing the renal pelvis resulting in hydronephrosis. She underwent right nephrectomy and adrenalectomy. The adrenalectomy specimen consisted of a well circumscribed ovoid mass with extensive hemorrhage surrounded by a rim of flattened adrenal tissue.

**Results:** Histological examination revealed epithelioid angiosarcoma arising in the adrenal gland. Immunohistochemically the large neoplastic cells showed positive reactivity for the vascular marker CD31,vimentin and pankeratin AE1/AE3. The ki67-proliferation index was 25 %. The postoperative course was uncomplicated. Three years after the initial surgery the patient is free of metastatic disease or recurrence. No chemotherapy was administered.

**Conclusion:** Primary angiosarcomas of the adrenal gland are very rare vascular tumours. Since the first description in 1988 about 30 cases have been published. The age range of the patients was 39–85 years with a mean of about 60 years. Because of cytokeratin positivity epithelioid angiosarcoma can mimic a metastatic carcinoma. Although generally angiosarcomas are considered as highly aggressive malignancies, angiosarcomas of the adrenal gland have a variable prognosis.

**PS-02-052**

**A case of giant myxoid adrenal cortical carcinoma with lipomatous metaplasia**

S. Gurzu ^*^, T. Bara, T. j. Bara, I. Jung

^*^University of Medicine and Pharmacy, Dept. of Pathology, Tirgu-Mures, Romania

**Objective:** To present a rare variant of adrenal cortical carcinoma diagnosed in a young women and the criteria used for its diagnosis

**Method:** A 36-year-old female presented with symptoms suggesting Cushing’s syndrome. The abdominal computed tomography revealed a tumor located in the left adrenal gland. Left adrenalectomy was performed

**Results:** Macroscopic examination of the surgical specimen revealed a 17 × 11 × 4 cm-sized encapsulated tumor weighing 1.2 kg, with a nodular structure, necrosis and hemorrhages on cut section. Histopathologic examination showed a heterogeneous aspect, the tumor cells being either arranged in clusters and cords or presenting diffuse architecture. Most of the cells were large, polygonal-shaped, with eosinophil cytoplasm. About 30 % of the tumor stroma had a myxoid aspect. Among the tumor cells, focal metaplastic lipomatous zones were evidenced. Immunohistochemically, the tumor cells were positive for Vimentin, Inhibin, CD56, and Melan A and negative for Chromogranin, Keratin AE1/AE3 and Epithelial membrane antigen. The molecular examinations did not detect K-ras or EGFR mutations. The final diagnosis was myxoid adrenocortical carcinoma with lipomatous metaplasia

**Conclusion:** In case of myxoid adrenocortical carcinoma, the diagnosis is mainly based on correlation of clinical aspects with hematoxylin eosin being only confirmed using immunohistochemical staining

**PS-02-053**

**Incidental adrenal neuroma: A case report**

N. Can ^*^, E. Tastekin, S. Isler, M. Azatcam, Z. Peglivanoglu

^*^Trakya University, Pathology, Edirne, Turkey

**Objective:** Neuroma represents proliferation of peripheral nerve fibers in which ratio of axons to Schwann cell fascicles approaches 1:1 and it is usually seen in soft tissues. Adrenal gland is a rare location of neuroma. Herein, we report an incidental adrenal gland located neuroma.

**Method:** A 65 years old male patient presented with abnormal kidney functions due to left sided grade 3 hydronephrosis and he was performed left simple nephrectomy. Grossly, nephrectomy specimen was surrounded by an adrenal tissue sized 5 × 3 cm along the upper pole. Microscopical examination of the renal tissue revealed chronic pyelonephritis but multiple unencapsulated spindle cell proliferations without Anthoni-A and Antoni-B areas were remarkable in the slides of adrenal medullary tissue. These spindle cell proliferations revealed S-100 and NSE positivity and there was no reaction with SMA, Melan A, Chromogranin A in immunohistochemical studies. So that, these unencapsulated spindle cell nodules were diagnosed as neuromas.

**Conclusion:** Neuromas are benign soft tissue tumors originated from peripheral nerve fibers. They are usually located in soft tissues and also mucosal neuromas can be seen, rarely. According to our knowledge soft tissue tumors such as Schwannoma can be found in adrenal tissue but neuroma is very rare. So that, we represent a rare localisation of neuroma and a rare soft tissue tumor originated in adreanal tissue in this report.

**PS-02-054**

**Crosstalk between pituitary adenomas blood vessels and their hormonal profile**

B. Balinisteanu ^*^, A. M. Cimpean, R. A. Ceausu, M. Raica

^*^Deta, Romania

**Objective:** To correlate pituitary blood vessels phenotype, microvessel density and the immunohistochemical hormonal profile as well as the hormone values in serum and cerebrospinal fluid.

**Method:** Thirty nine specimens obtained from patients with pituitary adenomas were included in our study. Immunohistochemical stains with specific antibodies against each of the pituitary hormones were performed on the selected specimens. The endothelial cells were labelled with anti CD34 antibodies and the perivascular cells with smooth muscle actin antibodies (SMA).

**Results:** Microvessel density was highest in GH secreting adenomas and lowest in prolactinomas. Based on a double immunostainig CD34/SMA, a predominance of immature and intermediate blood vessels was observed. We found a significant correlation between the immature blood vessels and the expression of tissue prolactin assessed by immunohistochemistry (*p* = 0,044) and partial correlations between serum (*p* = 0,036), cerebrospinal prolactin values (*p* = 0,006) and immature and intermediate blood vessels and between mature blood vessels and cerebrospinal fluid values of prolactin (*p* = 0,008).

**Conclusion:** Our study sustains the effect of prolactin on endothelial and perivascular cells, thus prolactin being directly involved in pituitary adenomas angiogenesis. The assessment of pituitary adenomas blood vessels phenotype correlated with their hormonal profile might partially explain the clinical and biological discrepancies found in these tumors.

Sunday, 1 September 2013, 09.30–10.30, Pavilion 2

**PS-03 Poster Session Head and Neck Pathology**

**PS-03-002**

**Carcinosarcoma arising in a pleomorphic adenoma of lacrimal gland: A rare tumor**

H. Rodrigues ^*^, C. Oliveira, M. J. Julião, M. F. Xavier Cunha

^*^CHUC, Anatomia Patológica, Pardilhó, Portugal

**Objective:** Tumours of the lacrimal gland are rare (<1/million individual year), half of them have epithelial origin. Pleomorphic adenoma is the most frequent (50 %) and the malignant transformation rarely reported.

**Results:** A 78 years male with retro-orbitary lesion in the left eye known since 2002 clinicaly suspicious of an “inflammatory pseudotumour”. In 2011 he presented with pain and sudden proptosis. Biopsy reveals a malignant mesenchymal neoplasia. Total exenteration of the left orbit was perfomed and a 4,3 cm tumour whitish and firm was found. Microscopic examination showed a neoplasm with areas of pleomorphic spindle and polygonal cells, areas of chondrosarcoma and carcinoma. Sections of the periphery of the tumour showed pleomorphic adenoma with transition to malignant areas and a small amount of lacrimal gland tissue. Epithelial component showed immunoreactivity to CK7, AE1/AE3 and Cam 5.2; Vimentina and focally SMA were positive in the mesenchymal component. Diagnosis was carcinosarcoma arising in a pleomorphic adenoma.

**Conclusion:** Carcinosarcoma of the lacrimal glands is an extremely rare tumour. We performed a literature review and to our knowledge only three cases are reported. Aggressive surgical excision is recommended with adjuvant radio and chemotherapy. Our patient died shortly after surgery.

**PS-03-004**

**Intranasal encephalocele: Presentation of two cases**

N. Can ^*^, E. Tastekin, E. Isler, A. Aslan, M. Azatcam

^*^Trakya University, Pathology, Edirne, Turkey

**Objective:** Nasal Encephalocele (NE) represents herniation of brain tissue and leptomeninges through bony defect of skull. NE is an uncommon diagnosis of nasal polyps and usually presents in older children and adults with equal gender distribution. Herein, we report two cases diagnosed as NE by histological, radiological and clinical evidence.

**Method:** Case 1: A 38 years old female patient presented with headache and rhinorrhea during the last 2 years. MR imaging revealed herniation of meningeal tissue through the wall of sphenoid sinus and polypoid lession in ethmoid sinus. The patient was performed anterior-posterior ethmoidectomy. Histological examination exhibited sheets of neuroglial tissue which revealed immunohistochemical reactivity with S-100 and GFAP. Case 2: A 56 years old female patient presented with rhinorrhea during the last month. MR imaging was reported as a right paramedian mass accompanied by fronto-nasal encephalocele. The patient was performed anterior-posterior ethmoidectomy and endoscopical excision of the mass. Neuroglial tissue which revealed immunohistochemical reactivity with S-100 and GFAP was seen in microscopical examination

**Conclusion:** Although NE is very rare the pathologists should be aware in the evaluations of nasal polyps.

**PS-03-005**

**Respiratory epithelial adenomatoid hamartoma in maxillary sinus: A case report**

K. Koulia ^*^, A. Tsavari, E. Moustou, E. Arkoumani, A. Zizi-Sermpetzoglou

^*^General Hospital Tzaneio, Dept. of Pathology, Piraeus, Greece

**Objective:** Respiratory epithelial adenomatoid hamartoma (REAH) is a rare lesion. Until today fewer than 50 cases have been reported in the literature. These lesions commonly site in the nasal cavity, but may occur in the nasopharynx and paranasal sinus. The exclusive involvement of the maxillary sinus is quite rare. This benign lesion originate from the Schneiderian epithelium, but not from the seromucous glands. Over 80 % of the patients with REAH are males, aging from third to ninth decate of life.

**Method:** A year-old male presented with a history of 2 years of cacosmia and recurrent rhinosinusitis. The patient underwent endoscopic sinus surgery to remove polyps. The speciment was sent for biopsy.

**Results:** Histologically, the lesion was composed of small to medium sized glands, separated by thickened, hyalinized stromal tissue. The glands were round to oval and were composed of multilayered, ciliated respiratory epithelium. A diagnosis of REAH was set.

**Conclusion:** Maxillary sinus localization is very rare, thus very important to distinguish a REAH from schneiderian pappilomas of the inverted type and adenocarcinomas. The treatment for REAH is complete local excision. Our patient is free of recurrence symptoms in 18-month follow-up.

**PS-03-006**

**Cell-cycle protein expression in respiratory epithelial adenomatoid hamartoma**

M. Rito ^*^, F. B. Santos, R. Fonseca, I. Fonseca

^*^IPO de Lisboa Francisco Gentil, Serviço de Anatomia Patológica, Portugal

**Objective:** Respiratory epithelial adenomatoid hamartoma (REAH) is a rare lesion, its putative neoplastic nature being recently suggested. Our objective is to review their clinicopathological profile and investigate immunophenotypic expression of proteins known to be involved in the tumorigenesis of other sinonasal tract neoplasms (p16, p53, MLH1 and MSH2).

**Method:** Eight cases of REAH were retrieved (2002 to 2010). Clinical data was obtained from the patients charts. Histological features were reviewed and p16, p53, MLH1 and MSH2 immunoexpression was evaluated.

**Results:** Patients mean age was 66 years with female predominance (*n* = 6). Lesions were unilateral in 5 cases and were associated with inflammatory polyps in 4 and with Schneiderian papilloma in 1. All patients underwent conservative local resections and no recurrences were recorded (mean follow-up time: 15 months). The 8 cases exhibited positive immunostaining for p16 (focal = 3, moderate = 4, diffuse = 1) and p53 (focal = 4, moderate = 4). MLH1 and MSH2 were normally expressed in all cases.

**Conclusion:** Our findings confirm REAH clinical features and benign course. Cell cycle-related proteins expression supports the previous evidence of the non-hamartomatous nature of this entity.

**PS-03-007**

**E-cadherin, b-catenin, p53, p16 and COX-2 expression in 98 intestinal-type sinonasal adenocarcinomas**

B. Vivanco ^*^, J. Perez-Escuredo, J. L. Llorente, M. Hermsen, F. Fresno

^*^HUCA, Dept. of Pathology, Oviedo, Spain

**Objective:** Intestinal-type sinonasal adenocarcinoma (ITAC) is an infrequent epithelial cancer. The aim of this work is to study the expression of several proteins in ITAC and in healthy tissue controls, and to figure out its prognosis implications.

**Method:** Immunohistochemical analysis of 98 ITAC was conducted on a tissue microarray and in healthy tissue samples.

**Results:** According histological subtype, p53 expression is less frequent in mucinous ITAC (*p* = 0,001) and weak E-cadherin expression is more frequent in this subtype (*p* = 0, 05). Loss of b-catenin expression and high p16 expression are associated with shorter overall survival (Fisher-exact test: *p* = 0,015 and 0,003/Multivariate Cox Regression: *p* = 0,011 [4,548HR (CI: 1,321–8,983)] and *p* < 0,001 [5,073HR (CI:2,044–12,594)]) and higher metastatic frequency (*p* = 0,008 and 0,044). P53 and COX-2 expression are related (*p* = 0,005) as well as b-catenin and E-cadherin expression (*p* = 0,012). Healthy tissue shows normal E-cadherin and b-catenin expression, and infrequent p53, p16 and COX2 expression.

**Conclusion:** Mucinous ITAC shows different immunohistochemical profile. Loss of E-caherin and b-catenin axis and high p16 expression are independent prognostic factors. P53 and COX2 are thought to be related to inflammatory-mediated carcinogenesis.

**PS-03-009**

**High-risk human papillomavirus is transcriptionally active in a subset of sinonasal squamous cell carcinomas**

L. Alos ^*^, B. Larque, S. Hakim, A. Nadal, A. Diaz, M. del Pino, L. Marimon, I. Alobid, A. Cardesa, J. Ordi

^*^Hospital Clinic Barcelona, Dept. of Pathology, Spain

**Objective:** The aim of the study was to assess the HPV active transcription in a series of sinonasal carcinomas, in correlation with the HPV DNA identification and the p16 immunohistochemistry.

**Method:** Seventy patients with squamous cell carcinomas of the sinonasal tract were included in the survey. All tumors were investigated for HPV through the HPV DNA detection by polymerase chain reaction (PCR), using the SPF10 primers and by in situ hybridization (ISH), using the high-risk GenPoint probe (Dako). HPV16 E7 mRNA transcripts detection was performed by PCR in 27 cases. The immunostaining for p16 was performed in all cases.

**Results:** Fourteen carcinomas (20 %) were positive for high-risk HPV by PCR: 13 HPV16 and one HPV35. ISH showed a dotted nuclear positivity in all these cases. HPV16 E7 mRNA was detected in 7 tumors harbouring HPV16; in the remaining HPV-positive cases RNA did not reach the quality for analysis. Strong, diffuse positivity for p16 was observed only in the HPV- positive cases.

**Conclusion:** We have shown that HPV is the etiological agent of a subset of sinonasal carcinomas demonstrating the transcriptionally-active HPV in these tumors. Immunostaining for p16 can be used as a surrogate marker to identify these tumors.

**PS-03-010**

**Expression of Pax5 and TTF-1 in Olfactory Neuroblastoma (ON)**

P. Czapiewski ^*^, P. Adam, J. Dzierzanowski, K. Okon, W. Biernat

^*^Medical University of Gdansk, Dept. of Pathology, Poland

**Objective:** Pax5 and TTF-1 play role in development of B-lymphocytes, and thyroid and lungs, respectively. They are expressed in human fetal brain, several brain tumors and some high grade neuroendocrine carcinomas. ON is an uncommon tumor of the nasal cavity that derives from olfactory neuroepithelium and shows features of neuroendocrine differentiation. The aim of our study was to identify expression pattern of Pax5 and TTF-1 in ON.

**Method:** We included 11 cases of ON (7M, 4 F, aged 46–86) into the study and analysed immunohistochemical expression of TTF-1 and Pax 5.

**Results:** TTF-1 was shown in 3/11 (27,3 %) cases as a nuclear pattern with mild to strong intensity, present in less than 1 % (*n* = 1) up to 30 % (*n* = 2) of cells. Pax5 was not identified in any of 9 analysed cases.

**Conclusion:** TTF-1 can be expressed in ON and, therefore, may present a diagnostic problem in metastasizing cases. Its clinical value requires further evaluation.

**PS-03-011**

**Dermoid cyst: A rare entity of the parotid gland**

S. Toprak ^*^, T. Ozgur, H. Gokce, C. Cevik, E. Akbay, E. Atik

^*^Mustafa Kemal University, School of Medicine, Dept. of Pathology, Hatay, Turkey

**Objective:** Dermoid cyts of the head and neck are benign lesions and they are extremely rare in the parotid gland. The clinical and radiologic presentation of dermoid cysts are ambiguous and preoperative diagnosis is difficult.

**Method:** A 53-year-old male presented with a 1-year-old swelling of the area of the right parotid gland to the Otorhinolaryngology Department outpatient clinic. Physical examination showed a soft, non-fluctuant mass in the inferior portion of the parotid gland. Computed tomography revealed a well-defined nodular mass with 2 × 2 cm diameter that was hypodense in the central region.

**Results:** The patient underwent right superficial parotidectomy and the material has been sent to pathology laboratory. In macroscobic examination there has been a soft 2.5 × 2 × 2 cm tan cystic mass holding on to the inner surface of the parotid gland with yellow oily material inside. Microscobic features included a cyst lined by stratified squamous epithelium and there has been skin appendages in the subepithelial stroma confirming the dermoid cyst diagnosis.

**Conclusion:** The differential diagnosis of dermoid cysts is large and includes mucous retention cyst, benign mesenchymal tumors, benign salivary gland tumors like pleomorphic adenoma, Hodgkin disease. To avoid recurrence dermoid cysts need careful surgical intervention.

**PS-03-012**

**Dermoid cyst of parotid gland: A case presentation**

S. Mocan ^*^, A. Iacob, D. Milutin, T. Mezei, D. Nekula, C. Petrovan

^*^Emergency Clinical Hospital, Dept. of Pathology, Targu Mures, Romania

**Objective:** Among different type of cystic lesion of the major salivary gland, dermoid cysts are described generally occurring midline in the floor of the mouth, with rare reported lesions in the parotid gland.

**Method:** We present a case of 26 years old women with a slow growing mass in the left parotid region. After a clinical and computed tomography evaluation a fine needle aspiration was performed. She underwent a superficial lobectomy of the left parotid gland and the lesion was surgically removed.

**Results:** Cytological specimen obtained from fine needle aspiration showed abundant eosinophilic debris some of which appeared to be keratin, with numerous squamous and foreign body giant cells. Also, fragments of hair were easily recognized. The resected specimen contained a collapsed, cystic lesion, with partially disrupted wall, of 3,5 cm diameter. Microscopic examination revealed a dermoid cyst with squamous epithelium on the surface and abundant skin adnexa. In the disrupted wall, the epithelium was replaced by many foreign body giant cells.

**Conclusion:** Although dermoid cysts can be easily recognized by microscopic examination, fine needle aspiration cytology can be helpful in recognizing this entity before surgical intervention. This case report shows the challenges in diagnosis and gives a short review of the literature.

**PS-03-013**

**Perineurioma of the parotid gland**

A. Yesilirmak ^*^, K. Kosemehmetoglu

^*^Hacettepe University, Dept. of Pathology, Ankara, Turkey

**Objective:** Mesenchymal soft tissue tumors are uncommon in the salivary gland. A 22-year old woman presented with a slowly growing unilateral parotid gland mass that was excised completely by partial parotidectomy.

**Method:** Routine pathological examination and immunohistochemistry were performed.

**Results:** Partial parotidectomy revealed 1,5 × 1 × 0,7 cm sized, grey-white, firm, well circumscribed, but unencapsulated, solitary nodule. Microscopically, tumor, arising in the parotid gland and extending to the peripheral fat tissue, was composed of spindle cells forming storiform pattern around peripheric nerve bundles and vascular structures. The tumor cells had eosinophilic bipolar cytoplasm and ovoid, bland nuclei without atypia and were associated with thick collagen fibers. Immunohistochemically, neoplastic cells were diffusely positive for EMA and focally positive for GLUT-1, collagen IV and SMA. S100, CD34, desmin and PR were completely negative. Differential diagnosis included perineuroma and meningioma. The absence of intranuclear inclusions, negativity for PR and GLUT-1 positivity supported the diagnosis of perineuroma, instead of meningioma. Electron microscopy was requested.

**Conclusion:** To our knowledge, this case represents the second reported case of soft tissue perineurioma in the salivary gland. Previous reports of parotid meningiomas should be reviewed for a possible misdiagnosis of perineurioma, although discrimination may be arbitrary.

**PS-03-014**

**Cytomorphology of mammary analogue secretory carcinoma of salivary glands: Report of two cases**

S. Tommola ^*^, I. Kholová, R. Karikoski

^*^Fimlab Laboratoriot Oy, Dept. of Pathology, Tampere, Finland

**Objective:** Mammary analogue secretory carcinoma is a recently described salivary gland neoplasm. Only small series and case reports on cytological features have been reported to date.

**Method:** Case 1: 56-year-old female presented with 1,5 cm parotid gland tumorous mass. Case 2: 34-year-old male revealed mass in submandibular gland of 1.2 cm diameter. Fine needle aspiration was performed twice under ultrasound control in both cases. Centrifuged material was stained with Papanicolaou stain. Additionally, immunocytochemistry was performed on cell-block material. The final histopathological diagnosis was revealed from resected gland.

**Results:** Aspirates were mildly to hypercellular. Cells formed tubular structures, smaller groups and nests. Cells varied slightly in size and shape. Cytoplasm was mildly vacuolated. Focally, oncocytic cytoplasm was detected in case 1. No myxoid material on background was noticed. Immunocytochemically, cytokeratin-7, EMA, vimentin, mammaglobin and S-100 were positive. MIB-1 proliferation index was 3 % (case 1) and less than 1 % (case 2). Low-grade salivary gland neoplasm was suggested diagnosis. The surgical specimen final diagnosis was mammary analogue secretory carcinoma.

**Conclusion:** Mammary analogue secretory carcinoma is a newly described salivary gland entity. Cytological differential diagnosis includes acinic cell carcinoma, mucoepidermoid carcinoma and pleomorphic adenoma.

**PS-03-015**

**Invasive carcinoma arising in sclerosing polycystic adenosis of the salivary gland**

R. Canas Marques ^*^, A. Félix

^*^Rio de Mouro, Portugal

**Objective:** Sclerosing polycystic adenosis (SPA) is a rare salivary gland lesion, recently regarded as a neoplastic lesion of low-grade malignant potential but to date no invasive carcinoma, metastasis or associated mortality has been reported. We report the first case of an invasive carcinoma arising in a recurrent SPA lesion.

**Results:** A 55-year-old man, with a past history of radiotherapy in childhood for a right lower lip lymphangioma, and three surgical excisions on the right parotid gland for recurrent lesions of SPA was submitted to a total right parotidectomy with ipsilateral neck dissection. Morphologically, the gland was occupied by multinodular lesions of SPA, with similar features as previous excised lesions. Arising in one of the nodules an invasive adenocarcinoma, with an in situ component was identified. Immunostains confirmed invasion by the absence of myoepithelial cells. The surgical margins were free of tumor and no nodal metastases were found. The patient was treated with additional local radiotherapy and, currently, is well and without recurrence, after 4 years of follow-up.

**Conclusion:** This case represents the first documented invasive carcinoma arising within a relapsing SPA highlighting the awareness of the potential malignant behaviour of this recently described neoplastic lesion.

**PS-03-017**

**Xanthogranulomatous sialadenitis: A potential mimicker of salivary gland malignancy**

A. Agaimy ^*^, B. Märkl, J. Zenk, M. Michal, A. Skalova

^*^Universität Erlangen-Nürnberg, Inst. für Pathologie, Germany

**Objective:** Xanthogranulomatous inflammation is an uncommon tumor-like subtype of chronic inflammation that has been mainly reported in kidney, gallbladder, testis and other less common sites. To date, only a few case reports exist on xanthogranulomatous sialadenitis (XGSA).

**Method:** We retrospectively reviewed our files for cases of XGSA. Inflamed cystadenolymphomas, cases post-FNA and re-resections with secondary inflammation were excluded.

**Results:** Nine patients were identified (6 males and 3 females aged 43–84 year; mean: 58 year). The parotid gland was affected in 8 cases and the submandibular in one. None was bilateral. Clinical diagnoses were mostly suspicious parotid gland tumor or swelling. Most lesions measured ≥2 cm and had poorly circumscribed margins. One lesion was confined to an intraparotid lymph node. Two cases showed no evidence of a preexisting salivary gland lesion/cyst (idiopathic XGSA). The remainder showed variably sized cystic ductal lesions suggesting cyst rupture as a trigger of secondary XGSA.

**Conclusion:** XGSA is probably significantly underreported. A thorough search for a pre-existing lesion is mandatory for appropriate classification. Idiopathic XGSA should be distinguished from other systemic granulomatous diseases and from salivary gland malignancy, particularly hematological neoplasms.

**PS-03-018**

**Epithelial predominant carcinosarcoma of salivary gland misdiagnosed as achromic melanoma**

F. Baderca ^*^, H. Urechescu, M. Pricop, E. Lazar, C. Solovan

^*^Timisoara, Romania

**Objective:** Carcinosarcoma of the salivary glands is a rare and aggressive tumor first described by Kirklin in 1951. We describe a case of a 52-y-o man who presented himself in January 2012 for an enlargement of the left parotid gland.

**Method:** The tumor appeared 1 month before and grew progressively. The clinical inspection revealed a 3 cm diameter mobile nodule, relatively well delimited.

**Results:** The microscopic evaluation showed islands of spindle and epithelioid malignant cells with pleomorphic vesiculous nuclei, eosinophilic macronucleolus and intranuclear inclusions in a mixoid stroma. Mitotic index was 45 mitoses/10HPF. Lack of a sarcomatous component and the development of tumor in proximity of skin, raised the suspicion of achromic melanoma. Immunohistochemical reactions sustained the diagnosis of carcinosarcoma versus melanoma. After 8 months, patient returned with a 1.5 cm diameter nodule at the site of surgical incision, with clinical suspicion of granuloma. The histopathology showed a tumor resembling the initial lesion but with areas of sarcomatous differentiation (condroid and osseous formation).

**Conclusion:** Even if the diagnosis of carcinoma of salivary gland is usually straightforward, the importance of immunohistochemical reactions in cases of epithelial predominant carcinosarcoma of salivary gland remains.

**PS-03-020**

**Isolated late metastasis from testicular seminoma presenting as a parotid gland mass: Case report and review of the literature**

J. Künzel ^*^, A. Agaimy, S. Krause, M. Vieth, C. Alexiou

^*^Universtätsklinik Erlangen, Germany

**Objective:** Parotid metastases from non head and neck cancers are rare and may represent a diagnostic and therapeutic challenge. A late metastasis from a seminoma to the parotid gland is presenting an unusual manifestation of disease.

**Method:** Case report and review of the literature

**Results:** A 45-year-old male patient with a history of testicular seminoma 5 years previously presented with a rapidly progressive parotid mass. Ultrasonography and computed tomography showed a space-occupying lesion at the angle of the right jaw. The mass was infiltrating into the parotid gland and into the parapharyngeal space. A primary parotid neoplasm was suspected, and panendoscopy with combined open biopsy was performed. Histological examination confirmed a seminoma metastatic to the parotid gland, and comparison with the primary tumour showed identical histology. The patient received chemotherapy for recurrent seminoma in accordance with the PEI protocol (cisplatin, etoposide, ifosfamide). After a total of four courses of chemotherapy, salvage radical parotidectomy with removal of all suspicious residual tumour tissue was then performed.

**Conclusion:** This case illustrates the difficulties that may be encountered in the differential diagnosis of parotid gland masses and underlines the necessity for a detailed clinical history and the need for strong interdisciplinary collaboration between oncologists and pathologists in order to correctly diagnose cases with such an unusual presentation.

**PS-03-021**

**Metastasis of Renal Cell Carcinoma (RCC) to large salivary glands: Description of 4 cases**

H. Majewska ^*^, K. Radecka, A. Skalova, D. Stodulski, C. Stankiewicz, W. Biernat

^*^Medical University of Gdansk, Dept. of Pathology, Poland

**Objective:** Metastatic tumors involving salivary glands arising from non-head and neck area are very rare. Renal cell carcinoma (RCC) is known for its high propensity for early metastasis. RCC metastasis to the maxillofacial area is a scarce event (16 %), however metastasis to the salivary gland are extremely rare. We report 4 cases of such pattern of dissemination.

**Method:** Four cases of metastatic RCC were selected from the historic files of salivary gland neoplasms operated between 1992 and 2012 in the Departments of Otolaryngology and Maxillofacial Surgery, Medical University of Gdańsk. The origin of the tumors was confirmed immunohistochemically, as vimentin, CD10 and RCC antigen were positive.

**Results:** The group included 3 females and 1 male. The age ranged from 66 to 97 years (mean 76,7 years). The tumours involved: parotid gland (3 cases) and submandibular gland (1 case). The size of tumours ranged from 2,6 to 5 cm. Total parotidectomy with selective neck dissection (2 cases) or simple resection of the tumor was performed (1 case). Histologically, the tumors were clear cell carcinomas. Differential diagnosis mainly included clear cell myoepithelial tumors, oncocytic lesions and clear cell adenocarcinoma.

**Conclusion:** The parotid was the initial manifestation of renal malignancy in 3 cases (1 status unknown). RCC, albeit rare, should be taken into differential diagnosis in clear cell tumors of the salivary glands.

**PS-03-023**

**Study of INI1 expression in four cases of myoepithelial carcinoma of the salivary glands**

M. Caldas ^*^, D. Felizardo, M. Jácome

^*^Ist. Português de Oncologia, Dept. de Anatomia Patológica, Porto, Portugal

**Objective:** Myoepithelial carcinoma of the salivary glands is defined as a neoplasm composed almost exclusively of cells with myoepithelial differentiation. A lesion with similar differentiation also occurs in the soft tissue. In myoepithelial carcinomas of soft tissue, INI1 expression loss is described in up to 40 % of cases in children and in up to 10 % of cases in adults and has not been detected in benign myoepithelial neoplasms. The aim of our study is to determine the pattern of INI1 expression in myoepithelial carcinomas of the major salivary glands.

**Method:** Cases of myoepithelial carcinoma of the major salivary glands diagnosed between 1998 and 2012 were retrieved from the files of the Department of Pathology, Portuguese Oncology Institute, Porto. Available HE and immunohistochemical slides were reviewed. Immunohistochemical staining for INI1 was performed on representative sections.

**Results:** 4 cases of myoepithelial carcinoma were retrieved, all located in the parotid gland. The patients were 3 male and 1 female, age range 76–85 years. All cases showed nuclear expression of INI1.

**Conclusion:** In our series, INI1 expression is preserved in myoepithelial carcinomas of the salivary gland.

**PS-03-024**

**Carcinoma ex pleomorphic adenoma: A clinicopathologic review of ten cases**

A. Yagüe Hernando*, J. M.ª Elizalde Eguinoa, P. Fernández Seara, E. Almudévar Bercero, A. Echegoyen Silanes, Y. Ruiz de Azúa Ciria, S. Rázquin Lizarraga

^*^Complejo Hospitalario Navarra, Surgical Pathology, Pamplona, Spain

**Objective:** Carcinoma ex Pleomorphic Adenoma (CEPA) develops from primary and recurrent Pleomorphic Adenoma (PA) and constitutes about 12 % of all malignant tumors of salivary glands. The benign mixed tumor component is often hialinized, whereas the malignant component can be of different histologic type, usually undifferentiated or adenocarcinoma NOS. We retrospectively review ten cases of CEPA diagnosed at our institution in the past 6 years.

**Method:** Of the ten patients aged between 42 and 80 years old, 7 were women and 3 were men. Primary PA was located in the parotid gland (6 cases), submaxilary gland (3 cases) and palate minor salivary gland (1 case).

**Results:** Adenocarcinoma NOS (4 cases) and Mioepithelial carcinoma (3 cases) were the most common histologic subtypes followed by salivary duct carcinoma (2 cases) and sarcomatoid (1 case). High histologic grade was found in 50 %. Half of the cases showed capsular invasion, whereas two of them where minimally invasive (<1.5 mm of capsular invasion) and three of them showed no capsular invasion.

**Conclusion:** The term Carcinoma ex Pleomorphic Adenoma is not sufficient as a stand-alone diagnosis. Recommendations for reporting these tumors should include: histologic type/grade, % of carcinoma and extend of invasion of the carcinomatous component (intracapsular, minimally invasive and invasive).

**PS-03-025**

**Carcinosarcoma ex-pleomorphic adenoma of the submandibular gland: Case report and review of the literature**

E. Borg*, V. Attard, B. Alexandra, P. M. Speight

^*^Mater Dei Hospital, Dept. of Histopathology, Msida, Malta

**Objective:** Carcinosarcomas of the submandibular gland are extremely rare. They can arise either de novo or from malignant transformation of a pleomorphic adenoma (carcinosarcoma ex pleomorphic adenoma).

**Method:** We report a case of an 89 year old lady who presented with a longstanding, slowly growing swelling in the right submandibular area. A fine-needle aspirate was suggestive of a pleomorphic adenoma. The submandibular gland was subsequently excised. The literature was searched for cases of carcinosarcoma of salivary glands and the data was tabulated.

**Results:** The resected mass was solid with a pale, firm cut surface. Microscopy and immunohistochemistry showed a carcinoma with foci or residual pleomorphic adenoma and areas of atypical spindle cell proliferation consistent with sarcoma. A diagnosis of carcinosarcoma ex pleomorphic adenoma was rendered. The literature search revealed 101 reported cases of carcinosarcoma with no sex predilection. Of these only 22 cases had arisen in the submandibular gland. Most cases arose de novo with only a small minority representing malignant transformation of a pre-existing pleomorphic adenoma.

**Conclusion:** Carcinosarcoma ex pleomorphic adenoma of the submandibular gland is an extremely rare tumour associated with a poor prognosis. Most patients will experience metastasis, recurrence and a poor prognosis despite optimal therapy.

**Macroscopic appearance of Carcinosarcoma ex pleomorphic adenoma Submandibular gland:** 
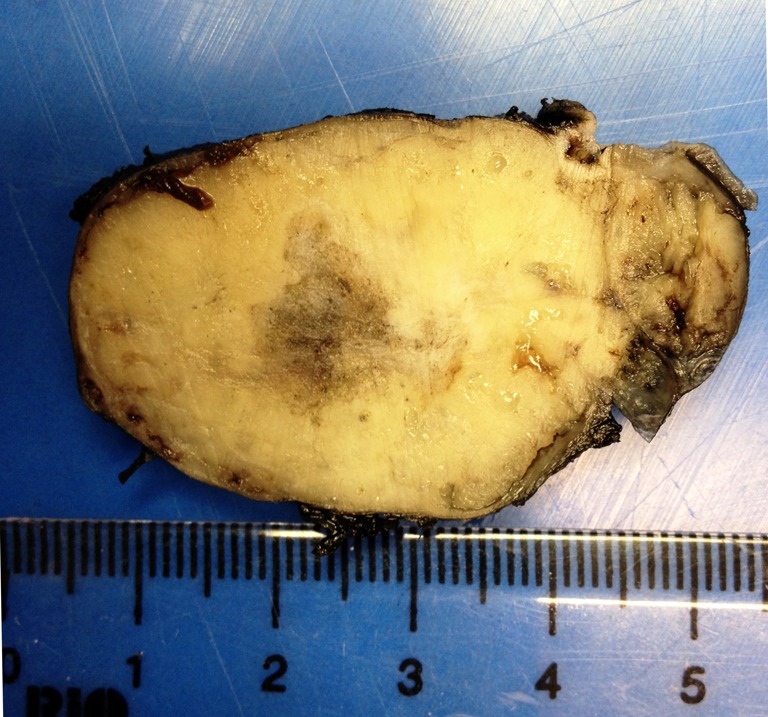


**PS-03-026**

**Genomic profile study of pleomorphic adenoma and carcinoma ex-pleomorphic adenoma by array-comparative genomic hybridization**

F. V. Mariano ^*^, L. P. Kowalski, O. P. de Almeida, R. D. Coletta, A. C. Victorino Krepischi, A. Altemani

^*^Faculty of Medicine—UNICAMP, Pathology, Campinas, Brazil

**Objective:** In this study we analyzed pleomorphic adenomas (PA) without malignant transformation and carcinoma ex-pleomorphic adenomas (CXPA) composed of tumors in different stages of carcinogenesis (intracapsular, minimally and frankly invasive), and classified according to histopathological subtypes by array-comparative genomic hybridization (array-CGH).

**Method:** Five PAs and eight CXPAs were evaluated. Tumor and reference DNA were hybridized to a 180k oligonucleotide array (Human CGH Microarray Kit 4 × 180K, Agilent).

**Results:** The PAs showed prevalence of gain in chromosome 8, loss in 17q and 19q. All alterations in PAs were maintained in CXPAs, with additional gain and losses. A different genomic profile were found in all CXPAs and within of same histological subtype. It was not observed progression of alterations with increase of tumor invasiveness. The most repeated alterations in CXPAs were gain and losses in chromosomes 2, 5, and 8.

**Conclusion:** In comparison to PAs, CXPAs showed an increase of number genomic alterations and the events in chromosomes 2 and 5 can be important for malignant transformation process. It was not possible to identify a particular pattern genotypic for each histological subtype and for stage of carcinogenesis. However more cases need to be investigated.

**PS-03-027**

**In vitro influence of extracellular matrix in myoepithelial cells from pleomorphic adenoma stimulated by epidermal growth factor**

V. Montalli ^*^, A. Altemani, V. C. Araújo, N. S. Araújo, E. F. Martinez

^*^State University of Campinas, Pathology, Brazil

**Objective:** The extracellular matrix-cell interactions as well as growth factors are known to be essential not only for the normal development but also its role in the tumorigenesis process.

**Method:** The present study aimed to in vitro evaluate the effect of EGF, in different concentrations (5, 10 and 20ŋg/mL) on cell morphology as well as the viability of benign myoepithelial cells, under the influence of different extracellular matrix proteins (matrigel, type I collagen, fibronectin). It was also evaluated the immunophenotype of these cells by α-smooth muscle actin and vimentin immunoexpression.

**Results:** The myoepithelial cells exhibited polyhedral morphology in all extracellular matrix conditions independently of the growth factor supplementation, except in the presence of EGF on polystyrene surface, where the cells exhibited a spindle-shaped morphology. In addition, no significant statistical alteration on cell proliferation was observed in the myoepithelial cells under influence of the studied doses of EGF. SMA was immunostained in the cells independently of EGF supplementation.

**Conclusion:** The extracellular matrix did not exert an important role on morphology, proliferation nor immunophenotype of the myoepithelial cells, even under the influence of EGF. These results may suggest that EGF might act in the neoplastic process (Grants from FAPESP 2011/10366-7, 2011/51112-8)

**PS-03-028**

**Polymorphous low-grade adenocarcinoma remains a challenging diagnosis: Our experience at a cancer institute**

D. Felizardo ^*^, A. Luis, F. Menezes, N. Coimbra, M. Caldas, A. Galaghar, M. Jacome

^*^Portuguese Oncology Institute, Dept. of Pathology, Matosinhos, Portugal

**Objective:** Polymorphous low-grade adenocarcinoma (PLGA) is a unique tumor which arises mainly in the minor salivary glands. Adenoid cystic carcinoma (ACC) is one of the most relevant differential diagnoses. Regrettably, discriminating value of immunohistochemistry remains controversial. Herein we report our experience, highlighting distinctive morphological and immunohistochemical characteristics.

**Method:** Cases of intra-oral PLGA and ACC diagnosed between 2006 and 2012 were reevaluated. All PLGAs were compared against an equal number of ACC cases. Key distinctive morphological aspects; ki-67 (using computer-assisted analysis) and calponin staining were assessed.

**Results:** In a total of 24 minor salivary gland malignant neoplasms, we retrieved 3 PLGA cases (13 %). One case was locally aggressive and had nodal metastasis. Ki-67 staining was lower in PLGAs (mean value 1.8 %; 9.1 % in ACCs). Calponin was focally positive in 1 and negative in 2 PLGAs and it was positive in all ACCs. Noteworthy histological distinctive features of PLGA were architectural diversity and bland cytology.

**Conclusion:** Most PLGAs are diagnosed in General Hospitals and only a small fraction arrives to specialized institutions. This would justify our lower percentage of PLGA (13 %) versus literature described proportion (26 %). Some PLGAs present unusual aggressiveness. Finally, immunohistochemistry might be helpful, but conventional histological evaluation remains crucial to formulate a correct diagnosis.

**PS-03-029**

**Evaluation of AKT pathway in polymorphous low-grade adenocarcinoma**

N. A. Silva Lascane ^*^, B. T. Sedassari, F. M. Martins, J. L. Saturno, S. C. O. Machado Sousa

^*^University of São Paulo, Oral Pathology, Brazil

**Objective:** The aim of this study was to investigate the role of AKT pathway in polymorphous low-grade adenocarcinoma (PLGA) of the salivary glands.

**Method:** PLGA were classified according to morphological characteristics as lobular, trabecular, tubular, cribriform and papillary. Immunohistochemistry was performed against antibodies pAKT, pmTOR and pS6 in 14 cases diagnosed as PLGA and scored as 0: negative cells, 1: 1–5 %, 2: 5–50 % and 3: >50 %.

**Results:** The most predominant morphological pattern were tubular and trabecular. pAkt and pmTOR were positive in 7 cases each one and 4 were scored as 2 and the other, 3 cases were scored as 3. Only 2 cases expressed pS6 positively, both scored as 1. pAKT and pS6 presented a nuclear expression and pmTOR showed expression in cell membrane, cytoplasm or both. Lobular was the subtype mostly positive followed by tubular subtype, 8 cases and 7 cases, respectively.

**Conclusion:** The results suggest a possible correlation of the proteins expression and PLGA subtypes lobular and tubular and showed that the AKT pathway plays a role in PLGA tumorigenesis, and this knowledge might benefit patients with target chemotherapy.

**PS-03-030**

**E-cadherin expression in mucoepidermoid carcinoma of oral & maxillofacial complex**

F. A. Oliveira ^*^, G. M. Carmo, B. C. Jham, A. C. Batista, E. F. Mendonça, E. C. Barroso Duarte

^*^IPTSP/UFG, Setor de Patologia, Goiânia, Brazil

**Objective:** The present investigation sought to evaluate E-cadherin immunohistochemical expression in mucoepidermoid carcinoma of oral and maxillofacial region.

**Method:** Thirty oral and maxillofacial mucoepidermoid carcinoma cases, diagnosed between 1996 and 2011, were retrieved from the medical files of the Head & Neck HAJ/ACCG, Goiania, Goias, Brazil. Immunohistochemistry study was performed on histological sections of formalin-fixed paraffin-embedded tissue samples (5 m-thick) using primary antibody anti-E-cadherin. Qualitative analysis of E-cadherin expression in the mucoepidermoid carcinoma was determined using a ×40 objective lens.

**Results:** In the present study around 73.3 % of Mucoepidermoid carcinoma cases presented a weak intensity of E-caderin expression, while a higher intensity expression was observed in 26.7 % of cases when compared with normal salivary glands. Patients presenting increased intensity of E-cadherin expression had better overall survival (93,4 mounths) when compared to those with low expression (55,5 mounths).

**Conclusion:** Our study demonstrated a correlation between E-cadherin intensity expression and overall survival. In most of cases the intensity of E-cadherin expression was reduced when compared with normal salivary glands that could be related to poor overall survival of patients. Financial Support: IPTSP/UFG, FAPEG.

**PS-03-031**

**Correlation among histologic grading, tumor size and lymph node metastasis in mucoepidermoid carcinoma of the salivary glands**

B. T. Sedassari ^*^, N. A. Silva Lascane, M. I. Fares Franco, S. C. O. Machado Sousa

^*^University of São Paulo, Oral Pathology, Brazil

**Objective:** The aim of this study was to correlate histologic grading, tumor size and nodal metastasis in mucoepidermoid carcinoma (MEC) of salivary glands.

**Method:** Seventeen cases diagnosed as MEC of salivary glands were selected from our files and data on topography and tumor size were analyzed. Slides stained with H&E were examined to confirm the diagnosis and histologic grading was performed according to WHO. Then, histologic grading was correlated with tumor size and nodal metastasis.

**Results:** Of the 17 cases, 11 were classified as low-, 3 intermediate- and 3 were high-grade. Tumor size ranged from 0,3 to 8,0 cm, with the smaller tumors classified as a low-grade and the largest as a high-grade carcinoma. Perineural invasion was detected in 7 cases, 2 low-, 2 intermediate- and 3 high-grade tumors. Angiolymphatic invasion was found in only one case, classified as a high-grade. Nodal metastasis was found in two cases, both low-grade.

**Conclusion:** Our study suggests that histologic grading may be correlated with tumor size, but not with perineural invasion and nodal metastasis, probably reflecting the genetic potential of the malignant neoplastic cells in invading and metastasizing, as well as events related to the tumor microenvironment may not correlate with morphological appearance.

**PS-03-032**

**Pulmonary metastasis of mucoepidermoid carcinoma synchronous with typical carcinoid tumor of the duodenum: An autopsy case report**

B. T. Sedassari ^*^, N. A. Silva Lascane, S. C. O. Machado Sousa, M. I. Fares Franco

^*^University of São Paulo, Oral Pathology, Brazil

**Objective:** Our aim is to report an autopsy case of metastatic mucoepidermoid carcinoma (MEC) synchronous with a typical carcinoid tumor (TCT) of the duodenum.

**Method:** A 76-year-old male patient experienced acute dyspnea, lung congestion and died 10 months after a parotidectomy for MEC. An autopsy was performed.

**Results:** On external examination, edema and neoplastic infiltration on the left side of the cervical region were observed associated with submandibular fistula. Generalized visceral congestion was observed upon the opening of the thoracoabdominal cavity. A mass in the lower lobe of the right lung was noted associated with chronic focal pneumonia, vesicular emphysema and edema. The aorta showed severe atherosclerosis. The GIT exam revealed duodenal wall thickening, diverticulosis with diverticulitis in the large intestine and polyp in colonic mucosa. The histological exam confirmed the diagnosis of metastatic MEC in the lung and TCT in duodenum. Acute pulmonary edema was considered the immediate cause of dead.

**Conclusion:** CME is the most common salivary gland carcinoma and its synchronic occurrence with a duodenal TCT has never been reported. Patients with MEC, as well as in other tumors, need to be monitored for a long period. Surgery and, whenever possible, adjuvant therapies should be employed in order to cure.

**PS-03-033**

**Morphological alterations in labial salivary gland in patients who underwent radiotherapy**

S. Bologna ^*^, T. H. N. Teshima, T. B. Nunes, M. D. Durazzo, M. M. Simonsen Nico, S. V. Lourenço

^*^HC-FMUSP, Dermatologia, São Paulo, Brazil

**Objective:** Radiotherapy is a modality of treatment largely used for head and neck malignancies. However, high doses of radiation in this area frequently results in permanent and severe salivary gland dysfunction which causes significant hyposalivation and xerostomia. Thus, the main purpose of this study is to evaluate the histopathological aspects of minor salivary glands affected by head and neck radiotherapy.

**Method:** Nineteen patients previously diagnosed with head and neck cancer submitted to radiotherapy were included in the study. Incisional biopsies of their labial minor salivary gland were performed pre and post-radiotherapy for histopathological analysis.

**Results:** Almost 80 % of all cases exhibited morphological alterations. Twelve cases showed acinar atrophy and three showed the presence of periductal hyalinization most probably due to radiotherapy. Other features as acinar fibrosis and replacement of glandular parenchyma by adipose tissue. All patients with these histopathological findings also demonstrated clinical signs and symptoms of xerostomia.

**Conclusion:** Association between radiotherapy and actinic damage in salivary glands result is xerostomia; further studies are necessary to develop radiation prevention and to prevent loss of function of salivary glands.

**PS-03-034**

**Sjogren’s syndrome: Histopathological evaluation of thrombosis in specimens of minor salivary glands**

S. Bologna ^*^, S. V. Lourenço, T. H. Teshima, S. Pasotto, M. M. S. Nico

^*^HC-FMUSP, Dermatologia, São Paulo, Brazil

**Objective:** Sjogren’s syndrome (SS) is an autoimmune exocrinopathy characterized by lymphocytic infiltration of exocrine glands in multiple sites. Progressive focal lymphocytic infiltration of salivary gland parenchyma with initial focal sialadenitis and late fibrosis are well described. Antiphospholipid antibodies (aPL) are observed in 2 % to 37 % of SS patients and may be responsible for thromboembolic events. Nevertheless, thrombosis may occur in the absence of aPL. Thomboembolic events have been described in organs such kidneys and central nervous system. Reports on the presence of thrombosis in salivary glands are still lacking. We investigated the presence of thrombosis in specimens of salivary glands from patients diagnosed with SS.

**Method:** Samples derived from 27 female patients with xerostomia, diagnosed with SS were histologically evaluated.

**Results:** Salivary gland alterations were detected in all cases. They included diverse levels of atrophy, acinar metaplasia and fibrosis. Presence of lymphocytic foci and linfo-myoepithelial ductal aggression were also observed; in 10 cases, it was associated with periductal hyalinization. In 17 cases, thrombosis of intra-glandular blood vessels was detected; from these case, 9 presented severe stromal fibrosis.

**Conclusion:** Salivary gland thrombosis is a histological feature that could be included as an additional criterion in the microscopic evaluation and diagnosis of SS.

**PS-03-035**

**The role of intrauterine growth retardation in parotid gland remodeling**

S. Morozov ^*^, O. Reshetnikova

^*^State Medical University, Lugansk, Ukraine

**Objective:** Salivary gland pathology results in the oral health status disturbances. Complicated pregnancy of mother may stimulate impaired salivation in their children later. The objective of this study was to determine the effects if experimental systemic intrauterine growth retardation (IUGR) in rats on their parotid gland remodeling.

**Method:** Sixty five samples of the newborn rats parotid glands, including 35 with experimental systemic growth retardation and 30 controls after uncomplicated pregnancies, were studied morphologically. Histological slides, stained with hematoxylin and eosin, were studied microscopically, and then their stereometric parameters were revealed.

**Results:** Results indicated morphological features of glandular acini and ducts immaturity. The area of pathologic changes in the glandular tissue increased. The volume fraction (VF) of parenchyma was significantly lower compared with controls (respectively-25,00 ± 6,96 and 51,72 ± 7,04 %,*P* < 0,05). The stroma VF in IUGR group increased to 35,67 ± 4,29 % (in control group-23,70 ± 3,25 %,*P* < 0,05).

**Conclusion:** Studies have confirmed the direct relationship between systemic IUGR in rats and delayed maturation of parenchymal components of the parotid gland. Theses may cause the insufficiency of the glands functional activity. The resultant oral homeostasis imbalance predispose to various dental pathologies, including caries, parodontitis, etc.

**PS-03-036**

**Clinicopathologic features associated to CD44/CD24 expression in benign and malignant salivary gland neoplasms**

A. Ribeiro-Silva ^*^, D. Soave, J. P. Oliveira-Costa, G. Silveira, R. Ianez, L. Oliveira, S. Lourenço

^*^Ribeirão Preto Medical School, Dept. of Pathology, Brazil

**Objective:** The present study aims to evaluate the CD44/CD24 expression in association with clinicopathologic features in benign and malignant salivary gland neoplasms.

**Method:** Immunohistochemical stains for CD44 and CD24 were performed on tissue microarrays containing SGN samples from 219 patients (128 pleomorphic adenomas, 22 Warthin tumors, 11 mucoepidermoid carcinoma, 34 adenoid cystic carcinoma, 4 acinic cell adenocarcinoma, 5 NOS adenocarcinoma, 3 polymorphous low-grade adenocarcinoma, 5 basal cell adenocarcinoma, 4 carcinoma ex-pleomorphic adenoma and 3 salivary duct carcinoma). The CD44, CD24 and CD44/CD24 expression phenotypes were correlated to patient clinicopathologic features and outcome.

**Results:** In benign neoplasms CD44 expression was associated with Warthin tumors when compared to pleomorphic adenomas (*p* = 0.0001). CD24 was associated with Warthin tumors when compared to pleomorphic adenomas (*p* = 0.0001). In malignat neoplasms CD44 expression was associated with the primary site of neoplasm (*p* = 0.046). CD24 was associated with clinical stage III/IV, T stage and lymph node (*p* = 0.008, *p* = 0,27, *p* = 0,001 respectively). The CD44/CD24 profiles were associated with the primary site of injury, lymph node and T stage (*p* = 0.005, *p* = 0.011, *p* = 0.023 respectively).

**Conclusion:** Our investigation demonstrates that CD44/CD24 expression is related to benign histologic types. CD44 and CD24 immunoexpression may provide prognostic information associated to clinicopathologic features in salivary gland malignant neoplasms.

**PS-03-037**

**Caspases involvement with ductal lumen formation during human salivary gland morphogenesis**

T. Teshima ^*^, S. Bologna, R. Iañez, C. Coutinho-Camillo, S. Lourenço

^*^University of São Paulo, Dept. of Oral Pathology, Sao Paulo, Brazil

**Objective:** Caspases represent a large family of proteases which possess an essential role in controlling apoptotic process. Although not completely understood, apoptosis can be related to the development of human salivary glands, mainly with the opening of the ductal lumen, and caspases would be possibly involved with that mechanism. Thus, the main purpose of this study is to elucidate a potential intervention of caspases 3, 6, 7 and 9 in human salivary gland morphogenesis, relating their expression to each stage of development.

**Method:** Samples derived from about 20 fetuses of natural miscarriages obtained from the Medical School of the University of Sao Paulo were submitted to immunohistochemical reactions for caspases 3, 6, 7 and 9.

**Results:** Only caspases 6 and 9 illustrated a positive expression during all stages of the gland development, being stronger in terminal phase. Nuclear staining of epithelial cells was observed in more than 65 % of the analyzed specimens. At the latest stage, cytoplasmic expression was also observed in epithelial and ductal portions.

**Conclusion:** Apoptotic proteins expressed in different stages of human salivary gland morphogenesis evidence an interaction among them during this process, especially guiding the formation of the ductal network, and the understanding of that mechanism will contribute to improve knowledge of salivary gland diseases.

**PS-03-038**

**Expression of PI3K/Akt pathway in adenoid cystic carcinoma and pleomorphic adenoma of salivary glands**

R. Ianez ^*^, C. Camillo-Coutinho, C. Pinto, A. Silva, F. Soares, S. Lourenço

^*^A.C. Camargo Hospital, Sao Paulo, Brazil

**Objective:** Pleomorphic adenoma (PA) and adenoid cystic carcinoma (ACC) are the commonest benign and malignant salivary gland neoplasms, respectively. They are originated from the intercalated duct region and are composed by luminal structures and myoepithelial cells. In a previous study we detected that protein c-kit is involved in the process of salivary gland morphogenesis and PA; this protein appeared to be related to pluripotent cells. Additionally, recent reports have shown that alterations in KIT gene are present in ACC. Based on this evidence, we further investigated the expression of PI3K/Akt pathway involved in the Kit signaling cascade in ACC and PA.

**Method:** The PI3K/Akt pathway was investigated in 50 cases of PA and 50 cases of ACC using immunohistochemistry

**Results:** In PA c-kit was positive in isolated luminal cells, and myopithelial cells were positive for alpha and beta PI3k, phospo-Akt and phosphor-mTor. In ACC, neoplastic luminal structures were positive for c-Kit and the other proteins showed positivity in myoepithelial cells especially in cribriform areas.

**Conclusion:** Expression of the downstream proteins of the c-kit cascade in myoepithelial cells in both PA and ACC may be indicative that these neoplastic cells share common proliferative pathways, despite being benign or malignant.

**PS-03-039**

**Advanced adenoid cystic carcinoma treated by imatinib mesylate: Report of five cases**

M. Vozmitel ^*^, J. V. Guljaeva, S. Rjabceva, T. I. Nabebina, A. Dubrovskij

^*^Minsk, Belarus

**Objective:** Adenoid cystic carcinoma (ACC) is a KIT-positive salivary glands tumor with frequent local recurrences and distant metastases. We report here our experience of using imatinib mesylate 400 mg/daily for treatment of 5 patients with unrespectable and/or extensively metastatic ACC.

**Method:** Clinical data, histologic features of tumors. Formalin-fixed paraffin blocks of 5 cases were available for immunohistochemistry (KIT; Dako) and RT-PCR (MYB-NFIB fusion and KIT gene mutations).

**Results:** Patient’s information: 3 man/2 women; median age 44 years. Disease status: multiple lung metastases (*n* = 3), massive liver metastasis (*n* = 1), wide local progression (*n* = 1). All tumors were KIT-positive. Exons 9, 11, 13 mutations of KIT were not revealed by PCR in all cases, whereas MYB-NFIB fusion transcript was detected in 2 of 5 tumors. Two patients had progressive disease, one of them dead of disease in 3 month after treatment start. Three patients had stabilization of disease for 1, 3 and 5 months, as the best response. In one of them after-treatment lesion established extensive degenerative changes of tumor tissue.

**Conclusion:** ACC shows doubtful treatment response to anti-c-kit targeted therapy. KIT gene mutations are not specific for ACC, while MYB-NFIB fusion is more frequent and may be a future candidate for targeted therapy.

**PS-03-040**

**Study of microRNA expression in malignant salivary gland tumours**

F. Passador-Santos ^*^, D. Soave, A. Silva, A. Soares, N. Araujo, V. Araujo

^*^São Leopoldo Mandic Institute, Pathology, Campinas, Brazil

**Objective:** Malignant salivary gland tumours comprise approximately 5 % of all head and neck malignancies. Adenoid cystic carcinoma (ADCC) is considered one of the most common malignancies of the salivary glands. Recently, a new class of small RNAs named microRNAs (miRNAs) has been described and applied for diagnostic and prognostic purposes. The aim of this study was to investigate the expression of miRNA 15a/16 and150 in ADCC

**Method:** Thirty-five cases ADCC of the salivary glands were retrieved from the files of the departments of Pathology of Sao Leoplodo Mandic Institute and Research Centre and Faculty of Medicine of the University of Sao Paulo (USP-Ribeirao Preto). Twenty-five cases from minor salivary glands, six cases from parotid and four cases from submandibular gland were collected. Total RNA was extracted using a standard Trizol method and expression of miRNAs 15a/16 and 150 was evaluated using real time PCR profiling

**Results:** Nineteen cases presented solid histopathologic pattern, 12 showed the cribriform histopathologic pattern while four cases had a tubular histopathologic pattern. Our preliminary results showed different expression of miRNAs 15a/16 and 150 in ADCC when compared to normal salivary glands

**Conclusion:** Our preliminary results indicate that deregulation of miRNA expression may be involved in the pathogenesis of ADCC

**PS-03-041**

**Cytokeratin immunoprofile of parotid region Squamous Cell Carcinoma (SCC): Is it possible to distinguish a salivary gland from cutaneous origin?**

N. A. Silva Lascane ^*^, B. T. Sedassari, M. I. Fares Franco, D. S. Pinto-Jr, S. C. O. Machado Sousa

^*^University of São Paulo, Oral Pathology, Brazil

**Objective:** The aim of this study was to compare the expression of cytokeratins (CKs) 7, 8, 13 and 14 in squamous cell carcinoma (SCC) from major salivary glands and from the parotid region (probably an infiltration of a cutaneous SCC).

**Method:** Twelve cases diagnosed as SCC in parotid gland were microscopically separated in primary SCC of the salivary gland (PSCC) and SCC of the parotid region (SCCPR). Immunohistochemistry was performed against CKs 7, 8, 13 and 14 in 8 cases of PSCC and in 4 cases of the SCCPR.

**Results:** All but one case of PSCC were negative for CK7. CKs 8 and 13 were positive in only two cases of PSCC, whilst CK14 was positive in all cases. One of these cases presented only a few cells positive to CK14 and this case was also positive to CKs 8 and 13. On the other hand, all cases of SCCPR showed positivity to CKs7 and 14, and 3 cases to CK8.

**Conclusion:** There is no correlation of the cytokeratins studied and the tumor site. The proximity of both lesions to excretory duct might explain the potential of these cells to differentiate in glandular-like or squamous-like or intermediate cells.

**PS-03-042**

**Profile of the lipid droplets in salivary gland carcinomas**

F. V. Mariano ^*^, H. T. Santos, V. A. Montalli, A. Altemani

^*^Faculty of Medicine—UNICAMP, Pathology, Campinas, Brazil

**Objective:** To verify the quantity of cytoplasmic lipid droplets (CLDs) in salivary carcinomas due to its association with tumor aggressiveness.

**Method:** In 68 salivary carcinomas (17 adenoid-cystic, 12 epithelial-myoepithelial, 11 mucoepidermoid, 10 acinic cell, 7 myoepithelial, 6 polymorphic low-grade and 5 salivary duct) the CLDs were stained for adipophilin. The quantity of CLDs in tumor cells was classified as absent (0 %–5 %), focal (>5 %–50 %) and diffuse (>50 %).

**Results:** 11.7 % of the carcinomas presented CLDs in more than 50 % of tumor cells as follows: myoepithelial (57 %), acinic cell (30 %) and epithelial-myoepithelial (8.3 %). In 53 % of the tumors, CLDs were not detected. In acinic cell carcinomas, the increased number of CLDs was associated with vacuolated appearance of the tumor cells.

**Conclusion:** In salivary carcinomas, CLDs are not a common event and its occurrence is not necessarily associated with high grade carcinomas. In acinic cell carcinomas, the upregulated lipogenesis reflects in the tumor morphology.

**PS-03-044**

**A 74–year-old caucasian woman with Kimura’s disease**

Y. Gorbacheva^*^, I. Kazantseva, A. Nikitin, E. Stepanova

^*^M.F. Vladimirsky Moscow Region, Dept. of Pathology, Russia

**Objective:** Kimura disease (KD) is a tumor-like chronic inflammatory disorder with angiolymphatic proliferation, usually affecting young men of Asian race, but it is rare in other races. The etiology of KD is still unknown.

**Method:** We report a rare case of KD involving the left salivary gland with surrounding lymph nodes and subcutaneous tissue. A 74-year-old woman presented with solid edema left parotid and orbital area, hearing and vision imparing.

**Results:** Computed tomography showed bilateral neck lymphadenopathy, subcutaneous tissue multiple infiltrations. Laboratory tests revealed that peripheral eosinophils (83 %) and serum IgE levels (400 ME/mL) were markedly increased. An excision biopsy of lymph node and salivary gland showed KD specific morphological features: lymphoid and eosinophil infiltration, the proliferation of high endothelial venules with slit-like lumina, eosinophillic microabscesses, fibrosis. The differential diagnosis between KD and leukemia with eosinophilia was performed: PDGFRA, PDGFRB and FGFR1 genes mutation was not revealed, bone marrow examination showed only eosinophilia.

**Conclusion:** Thus, such a clinical, instrumental, laboratory and morphological features combination allowed to provide KD diagnosis.
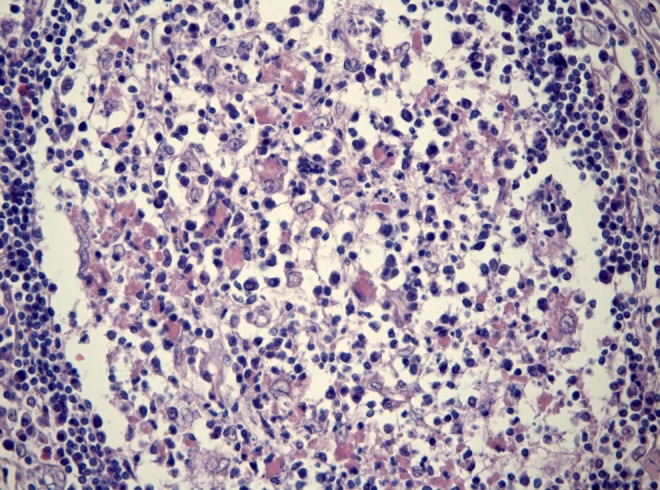
 
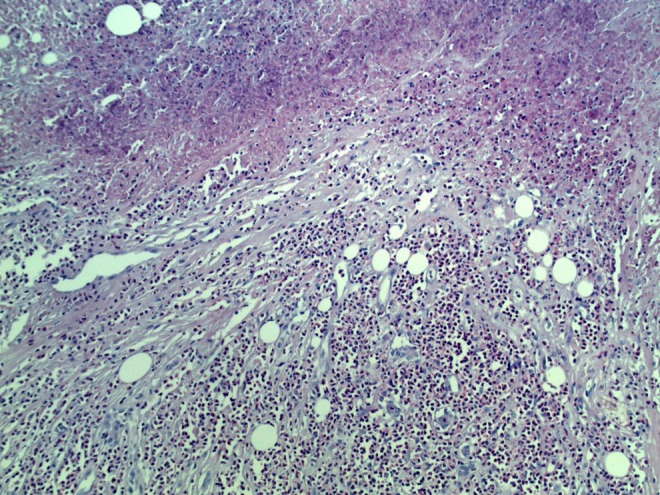
 
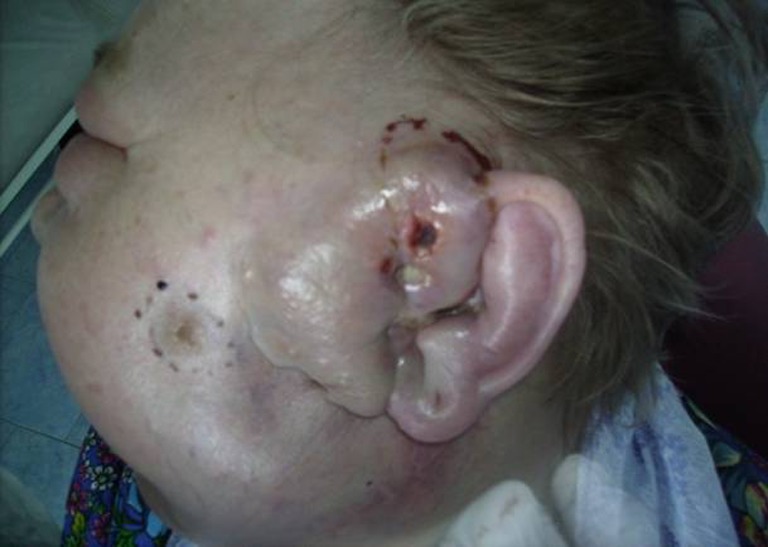
 
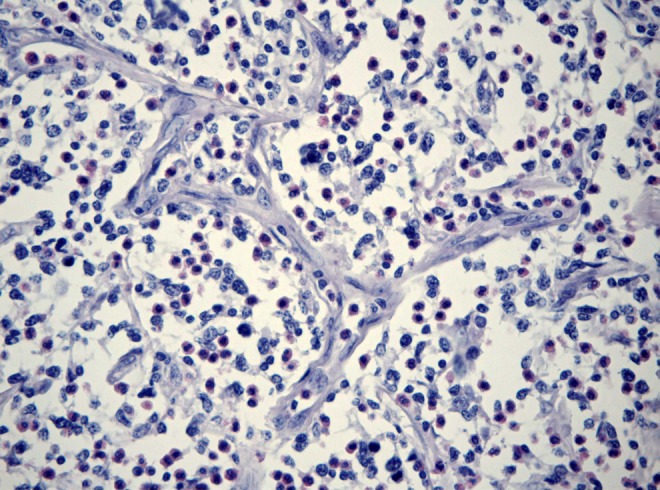


**PS-03-045**

**Parapharingeal solitary fibrous tumor in a 57-year-old female**

F. Moreno ^*^, R. Dias, A. Duarte

^*^Centro Hospitalar do Porto, Dept. de Anatomia Patológica, Portugal

**Objective:** Solitary fibrous tumor is an uncommon mesenchymal tumor that usually arises from the pleura. There are occasional reports of extrapleural sites, including the parapharingeal space. Most cases are benign and cured with complete resection but malignant potential was reported. Microscopically, they are well circumscribed but non-encapsulated tumors with a characteristic patternless arrangement of narrow spindle-cell cords interspersed by thick collagen bundles. Most have hypercellular and hypocellular areas and might show prominent haemangiopericytoma-like appearance. Immunohistochemically the cells are uniformly positive for CD-34 and Bcl-2 and negative for S-100 protein.

**Method:** We report the case of a 57-year-old woman proposed for study of a right parotid swelling, xerostomy, persistent right tinnitus and temporomandibular pain with 2 month evolution.

**Results:** The magnetic resonance study showed an expansive, well defined mass located in the right parapharingeal space. The patient underwent local surgical recession and the microscopic examination revealed a benign parapharingeal solitary fibrous tumor with the characteristic morphologic and immunohistochemical features described above. There is no evidence of local recurrence 1 year after surgery.

**Conclusion:** This case illustrates the typical histological and Immunohistochemical presentation of this type of tumors. The follow-up of these patients is mandatory, since there are reported cases of recurrent disease with malignant characteristics.

**PS-03-047**

**A case of imported paracoccidioidomycosis**

H. Coelho ^*^, J. Vaz de Castro, D. Brito, J. Oliveira e Neta, M. J. Aleixo, C. André, L. Antunes, M. J. Brito

^*^Hospital Garcia de Orta, Dept. de Anatomia Patológica, Almada, Portugal

**Objective:** The purpose of this study is to report a case of infection by Paracoccidioides brasiliensis, a fungus endemic in South America.

**Method:** A Brazilian 63-year-old man, with marked alcoholic and smoking habits, working in Portugal as gardener for the last 8 years, presented in the emergency department with odynophagia in the previous month associated with dysphagia in the last week. Observation of the oral and oropharyngeal cavities revealed an irregular mucosa with granular surface. On the left buccal mucosa, an ulcerated lesion was identified extending towards the inferior alveolar border, friable and painful on palpation. Endoscopy showed that the tongue base also harboured a lesion with the same characteristics, obliterating the left vallecula.

**Results:** Histologic examination identified microorganisms compatible with P. brasiliensis in the samples obtained from oropharyngeal mucosa.

**Conclusion:** Globalization and migratory patterns have increased the number of imported diseases. It is thus important that pathologists and clinicians, in Europe, become aware and familiarized with the clinical presentation of this rare disease, endemic to South America.

**PS-03-048**

**A malignant Perivascular Epithelioid Cell neoplasm (PEComa) of the carotid bifurcation in a 19-year-old man**

D. Koumoundourou^*^, T. Stathas, D. Mpatsoulis, V. Zolota

^*^University Hospital of Patras, Pathology, Greece

**Objective:** PEComas, are unique mesenchymal tumors characterized by a mixed myogenic and melanocytic phenotype. Examples of this neoplasm originating in the head and neck region are limited.

**Method:** A 19-year-old man was submitted to our hospital with a painless hard swelling of the right upper neck. The patient underwent a surgery with ipsilateral cervical lymph node dissection. A soft grayish tumor mass measuring 35 mm in greatest diameter was resected

**Results:** Microscopically the tumor consisted of solid sheets of medium sized spindle cells arranged in fascicles and nests with high cellularity, foci of coagulative necrosis and exceptional mitoses. The cytoplasm was either clear or acidophilic and tumor cells had a prominent eosinophilic nucleolus. An adjacent 30 mm lymph node was also affected. Immunohistochemically tumor cells were HMB-45+++, S-100+, MyoD1+, myogenin+. According to the criteria proposed for the classification of PEComas (Folpe) the diagnosis was a malignant PEComa. The patient received radiation therapy.

**Conclusion:** PEComas have been reported mainly in the abdominopelvic cavity and rarely in parenchymatous organs, skin, and soft tissues. None of the reported cases was situated in the carotid bifurcation. The present case serves to emphasize the potential of PEComa for aggressive behavior and the importance of distinguishing this tumor from other epithelioid neoplasms.

**PS-03-049**

**Metastatic epithelioid hemangioendothelioma of the palate region**

S. Mocan ^*^, S. Comisel, D. Milutin, T. Mezei, A. Iacob, C. Petrovan

^*^Emergency Clinical Hospital, Dept. of Pathology, Targu Mures, Romania

**Objective:** Metastatic tumors of the oral region are challenging because are very rare and frequently can mimic a benign lesion.

**Method:** We present a case of a 58 years old male, with a tumor in the palate region and multiple metastases. Three years previously, the patient was diagnosed with epithelioid hemangioendothelioma localized in the deep soft tissue of the lower extremity and he was treated by surgery and chemotherapy. After clinical evaluation, the tumor form the posterior region of the soft palate was removed.

**Results:** The tumor was localized deep in the mucosa, with an infiltrative growth pattern and extensive necrosis. The tumor was composed of short strands of small round epithelioid and slightly spindle cells set in a myxohyaline stroma, with abundant eosinophilic cytoplasm with prominent vacuolization. The number of mitoses was 4–6 mitoses/10 HPF. Immunohistochemically, the tumor cells stained positively for CD 31, CD 34 and Factor VIII-related antigen, and focally for Cytokeratin AE1/AE3.

**Conclusion:** Epithelioid hemangioendothelioma is considered as a borderline endothelial tumor. There aren’t strict criteria for malignancy that can be used to predict recurrence and metastases. The case illustrates the aggressive behavior of the tumor and the difficulties in diagnosis, especially because the tumor appears in an unusual location.

**PS-03-050**

**Peripheral Ossifying Fibroma (FOP): Clinico-pathological and immunohistochemistry findings in a series of 13 patients**

J. M. Suárez Peñaranda ^*^, H. Lázare, P. Gándara Vila, J. J. Carrera Álvarez, J. Caneiro, M. Pérez Sayáns, A. García García, J. M. Gándara Rey

^*^Hospital Clinico Santiago de, Compostela, Dept. de Patalogia, Santiago de Compostela, Spain

**Objective:** We report the findings of 18 biopsies from 13 patients with the diagnosis of FOP, including including the immunohistochemical study.

**Method:** Clinical data have been collected retrospectively from the files of the patients and the slides and paraffin blocks from the files of the Department of Pathology of the Clinical and University Hospital of Santiago de Compostela. Immunohistochemistry was performed for smooth muscle actin (SMA), CD68 and CD34 using the Dako Autostainer Link 48 and EnVision.

**Results:** 9 patients were females and 4 males. Ages ranged from 10 to 70 years. Most tumors were below 2 cm and only two were larger: 2,5 and 5 cm, respectively. Four patients presented relapses (30.7 %), from 1 to 8 years after surgery, one of them three times. Histopathological examination revealed the presence of a benign mesenchymal proliferation with variable degrees of bone formation: lamellar bone in 12 cases, woven bone in 11 cases and cementum in 11. Immunohistochemistry was performed in 14 biopsies: 10 showed stain for SMA and 3 for CD68, while CD34 was negative.

**Conclusion:** POF has showed a distinct predilection for women between the 2nd and 4th decades of life. The relapse rate has been 30 %, higher than expected according to the literature. The immunhistochemistry study confirms the myofibroblastic nature of the condition.

**PS-03-051**

**Primary central chondrosarcoma of craniofacial bones: Report of 5 cases**

W. Ouahioune ^*^, N. Moulai, N. Bettaz, N. Medjber, A. Mansour

^*^University of Blida, Dept. of Medicine, Algeria

**Objective:** Primary central chondrosarcoma (PCCS) accounts for about 20 % of malignant bone tumors. It’s characterized by the formation of cartilaginous matrix. The most common skeletal sites are the bones of the pelvis. PCCS is rarely found in craniofacial bones. The aim of this study is to report 5 new cases of PCCS of head and neck with analysis of the clinicopathological characteristics of this tumor.

**Method:** It’s a retrospective study of 5 PCCS collected over 10 years at our department. The follow up ranges from 02 to 10 years.

**Results:** On the 21 PCCS diagnosed in our service, 05 of them were localized at craniofacial bones. There were 03 males and 02 females; age ranged from 25 to 49 years. The sites of involvement were jaw bones (03 cases), temporal bone (01 case) and occipital bone (01 case). Radiologically, the diagnosis of PCCS was not raised in any case. The diagnosis of PCCS was established after histological examination. 4 of 5 patients were at first treated by a wide en-bloc surgical resection then they underwent radiotherapy. 03 of those who were treated had recurrences.

**Conclusion:** Although this tumor is rare in the craniofacial bones, PCCS must always be kept in mind.

**PS-03-052**

**Familial tumoral calcinosis: Clinico-pathological findings and confocal laser scanning microscopy of hard and soft tissues lesions**

E. Maiorano ^*^, L. Limongelli, A. Tempesta, A. P. Cazzolla, V. De Falco, D. Di Venere, G. Favia, A. Napoli

^*^Università degli Studi di Bari, Dipt. di Anatomia Patologica, Italy

**Objective:** We report on the clinico-pathological features of Familial Tumoral Calcinosis (FTC), a rare disease of childhood and adulthood, caused by mutations in FGF and GalNAc transferase 3, consisting in a bone metabolism disorder with abnormal phosphate and calcium (calcinosis) deposits around the joints, in viscera and soft tissues.

**Method:** A 17 year-old girl, complaining for long-standing leg pain, resistant to FANS therapy, was diagnosed with osteogenesis imperfecta and therefore undergoing bisphosphonates therapy. She was referred to our Dental Clinic for diffuse dental abnormalities, maxillary hypoplasia and tooth roots inclusions and underwent combined surgical and orthodontic treatment. The surgical samples were used for conventional and Confocal Laser Scanning Microscopic (CLSM) examination.

**Results:** Microscopically, several metaplastic micro and macro-calcification in soft and periodontal tissue location were detected, along with typical islands of homogenous, non tubular, dentino-osteoid calcified structures in dentinal tissues. Also, dentinal dysplasia with osteoid-like material, without incremental lines but with strong basophilia, intermingled with remnants of mature mucous connective tissue, were demonstrated. The diagnosis of FTC was confirmed by genetic analysis.

**Conclusion:** CLSM helps to demonstrate distinct odontoblast and osteoblast anomalies in FTC that lead to the accumulation of atypical calcified tissues, responsible for the several clinical signs detected in the patient and formerly attributed to osteogenesis imperfecta.

**CLSM features of Tumoral Calcinosis:** 
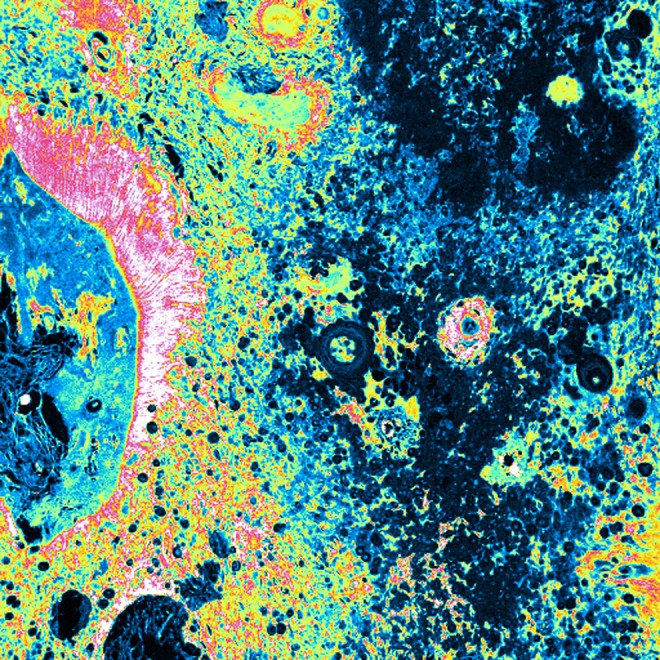


**PS-03-053**

**Podoplanin expression is enhanced in pseudoepitheliomatous hyperplasia associated with bisphosphonate osteonecrosis of the jaw**

J. Zustin ^*^, D. Reske, A. Koenemann, A. Assaf, H. Scheuer, R. Friedrich

^*^UKE Hamburg, Institute of Pathology, Germany

**Objective:** Pseudoepitheliomatous hyperplasia has been observed in bisphosphonate osteonecrosis of the jaw (BRONJ) lesions. Podoplanin expression has been linked with oral squamous cancer cell migration and invasion. Because podoplanin expression has further been reported in inflamed gingival epithelium, we examined podoplanin expression in pseudoepitheliomatous hyperplasia in BRONJ.

**Method:** Archival cases of BRONJ were re-reviewed in order to identify cases showing pseudoepitheliomatous hyperplasia. Six positive cases were simultaneously analyzed using immunohistochemical antibodies for cytokeratins (AE1/AE3) and podoplanin (D2-40).

**Results:** All six cases of pseudoepitheliomatous hyperplasia associated with BRONJ showed irregular epithelial proliferations both at the site of necrotic bone and within an inflamed granulation tissue. All lesions showed strong diffuse positive reaction with AE1/AE3 antibody. The pseudoepitheliomatous hyperplasia lesions revealed strong expression of D2-40 antibody in the basal epithelial cells and weak reaction in the parabasal cell layers.

**Conclusion:** We observed a D2-40 expression in benign pseudoepithelial hyperplasia associated with BRONJ. We suggest that podoplanin expression might be induced in benign pseudoepitheliomatous hyperplasia by chronic inflammation associated with BRONJ similar to chronic periodontitis. Even though malignancies have exceedingly rarely been reported in association with BRONJ, every precaution should be taken in the interpretation of the dignity of epithelial proliferations positive for podoplanin.

**Pseudoepitheliomatous hyperplasia associated with BRONJ:** 
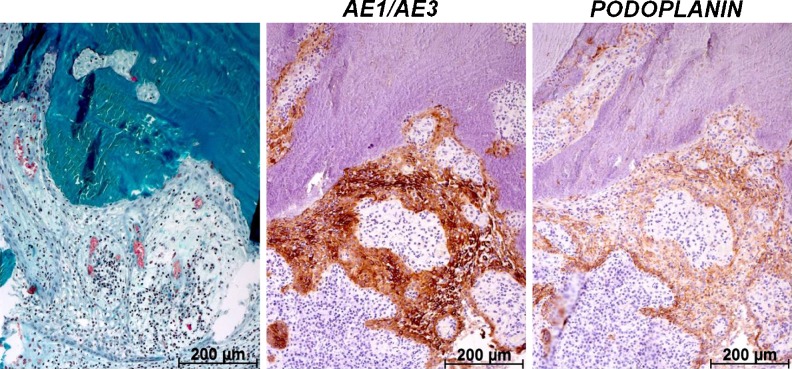


**PS-03-054**

**Recurrent squamous odontogenic tumor: A case report**

S. Manojlovic ^*^, J. Budimir, L. Manojlovic, D. Macan

^*^University Hospital Dubrava, Dept. of Pathology, Zagreb, Croatia

**Objective:** Squamous Odontogenic Tumor (SOT) is exceedingly rare benign odontogenic epithelial neoplasm. Recurrences are exceptional. The present case is the third report of recurrent SOT so far.

**Method:** Case report: A 50-year old female presented with a swelling in the mandible. Radiographic images disclosed a radiolucency in premolar area. Local excision was performed. Histopathological analysis revealed a squamous odontogenic tumor. Two recurrences arose at the same localisation,1 and 4 years later, respectively. Histopathological pattern was in both recurrences identical to the primary tumor.

**Results:** SOT is extremely rare tumor, around 45 cases having been reported to date. Most of them arose intraosseally, near the periodontal ligament. Recurrence has been described in two cases. The etiology and pathogenesis of the SOT are unknown. Differential diagnosis includes a variety of odontogenic cysts and desmoplastic ameloblastoma. The most important differential diagnosis is intraosseous squamous cell carcinoma (SCC). Conservative surgical procedures are considered adequate treatment for SOT.

**Conclusion:** The present case is, to our knowledge, the third report of the recurrent SOT. At least one case of SOT transformed into a carcinoma. Therefore, more extensive surgical excision and a long-term follow-up is essential for such lesions.

**PS-03-055**

**Podoplanin in ameloblastoma: Its relevance and distribution in different clinicopathologic subtypes**

C. H. Siar ^*^, K. H. Ng

^*^University of Malaya, Dept. of Oral Pathology, Kuala Lumpur, Malaysia

**Objective:** Human podoplanin, a specific lymphatic endothelial cell marker, is biologically related to cell migration and invasion. The ameloblastoma is a benign but locally-invasive odontogenc epithelial neoplasm. The aim of this study was to determine the distribution pattern of podoplanin in ameloblastoma and to speculate on its relevance.

**Method:** Immunohistochemical staining for podoplanin was performed on 18 unicystic(UA), 20 cases solid/multicystic(SMA), 4 desmoplastic(DA) and 20 recurrent ameloblastoma(RA).

**Results:** Podoplanin was detected in most ameloblastoma subtypes(UA = 18/18; SMA = 13/20; DA = 2/4 and RA = 17/20). Distribution pattern was heterogeneous, and indistinctive between tumour centre and periphery. Protein localization was pre-ameloblast-like>stellate reticulum-like cells, membranous and cytoplasmic. Stromal fibroblasts, lymphatic endothelium, nerves, osteoblasts and salivary glands were variably podoplanin-positive.

**Conclusion:** Although ameloblastoma subtypes are distinctive in their clinicopathologic presentations and behaviour, their podoplanin distribution patterns are not different. This suggests that this protein molecule might not be a major factor influencing the progression of these different subsets.

**Recurrent ameloblastoma and podoplanin expression:** 
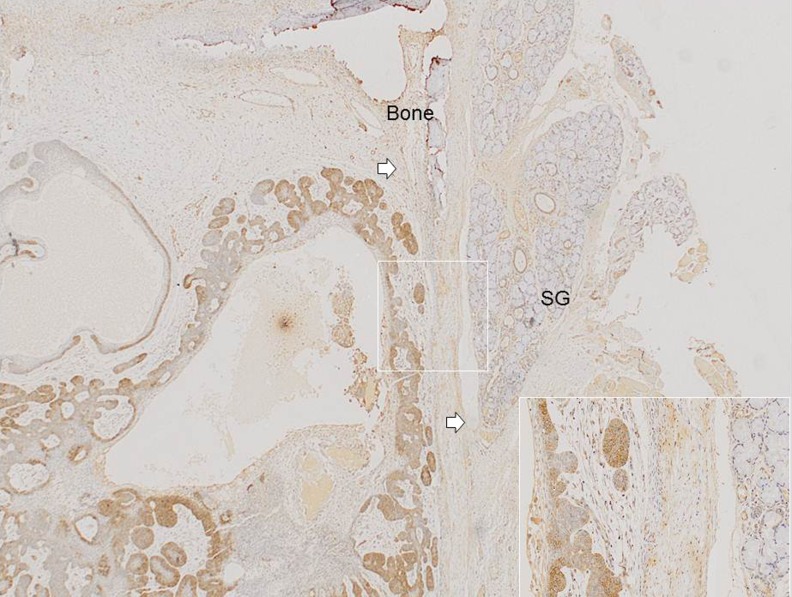


**PS-03-056**

**Ameloblastoma originated from maxillary sinus**

E. Dogan ^*^, Z. A. Abidin, O. Ipci, E. Akoglu

^*^Mustafa Kemal University, School of Medical Pathology, Hatay, Turkey

**Objective:** Ameloblastoma is a locally agressive tumor which arises from odontogenic epitelium. Origin of sinonasal tract is very rare and few cases have been reported.

**Method:** A 61-year-old male patient presented with epistaksis for 2 months. Hemorrhagic degenerative mass has seen in physical examination at the level of the left middle concha nasal cavity. CT scans has revealed a mass originated from left maxillary sinus. Multiple biopsies were taken from the mass.

**Results:** Macroscopically, the largest 1.5 × 0.6 × 0.5 cm in size 3 pieces covered with the mucous surfaces of irregularly shaped fragments were observed. Microscopically,in the form of islands, cords, which palizading at periphery, often monotonous, narrow oval-round nuclei and eosinophilic cytoplasm by the tumoral cells was observed.

**Conclusion:** Sinonasal ameloblastomas are rare tumors which arise from odontogenic epithelium. Small biopsies may be superficial and may not show typical histological findings. And this tumor could kept in mind in differantial diagnosis with aother tumor sharin similar morphology in small biopsies.

**PS-03-057**

**Activation of the AKT/mTOR pathway in dentigerous cysts, odontogenic keratocysts and ameloblastomas**

R. Chaisuparat ^*^, S. Yodsanga, S. Montaner, B. Jham

^*^Chulalongkorn, Dept. of Oral Pathology, Bangkok, Thailand

**Objective:** To investigate the activation of Akt/mTOR in dentigerous cysts (DC), odontogenic keratocysts (OKC) and ameloblastomas, and to correlate the findings with clinical and histopathological parameters.

**Method:** A total of 90 cases (30 DC, 30 OKC and 30 ameloblastomas) were studied. Patient records on age, sex, lesion location, symptoms, and radiographic and histopathologic features were collected. Immunohistochemical study of the Akt/mTOR pathway (p-Akt-S473, p-Akt-T308 and p-RPS6) was studied on tissue microarrays. Immunohistochemical reactivity was graded according to the percentage of positive tumor cells. Statistical significance was considered at *p* < 0.05.

**Results:** Over 90 % of OKC and ameloblastoma cases stained positive for p-Akt (Ser473), while 60 % of DC cases were found positive for p-Akt (Ser473). Ameloblastomas displayed the highest number of cases with positive p-Akt (Thr308) (73 %), followed by OKCs (40 %) and DC (20 %). Similarly, p-RPS6 was detected most frequently in ameloblastomas (83 %), followed by OKCs (76 %) and DCs (53 %). Statistical analysis showed significant differences in phosphorylation levels of all three proteins (*p* < 0.05). However, there was no significant correlation between those phosphorylation levels and the clinicopathologic features of the lesions.

**Conclusion:** We have shown that the Akt/mTOR pathway is upregulated in DCs, OKCs and ameloblastomas, suggesting a role for this pathway in the development and progression of the lesions.

**PS-03-059**

**Morphological evaluation of experimental temporomandibular joint disorder**

K. Semenov^*^, O. Reshetnikova

^*^Dnepropetrovsk Medical Academy, Ukraine

**Objective:** Temporomandibular joint disorders (TMJD) clinically presented with chewing muscles and joints pathology. Although it is known that physical stress on the structures around the joint may result in TMJD, in many cases the cause of TMJD remains unknown. The aim of present study was to find out morphological changes in chewing muscles and joint structures in case of experimental TMJD.

**Method:** Ten mature outbred 9-month male rats were the material for study. Five rats had standard diet with no pathology of the TMJ (controls). Other five rats were used for the TMJD modeling. The musculoskeletal blocks from the TMJ area of the injured side as well as from the intact TMJ area were taken for histology and morphometry.

**Results:** The results have shown the hypertrophy of the masseter muscle, foci of collagen fibers distruction with inflammatory infiltrates in tissues around the TMJ with experimental TMJD. The articular disk revealed small lesions. Morphometry showed the increased area, width, length and perimeter of the masseter muscle fiber section at the TMJD side.

**Conclusion:** These findings present experimental evidence of an association of TMJD symptoms with the hypertrophy of the chewing muscles, pathomorphological changes both in muscles and joint structures.

**PS-03-061**

**Morphogenesis of teeth and pathology of thyroid gland**

T. Pavlova ^*^, E. Peshkova, I. Goncharov, D. Kolesnikov, K. Prashchayeu, V. Markovskaya

^*^BelSU, Dept. of Pathology, Belgorod, Russia

**Objective:** Study of endocrinopathies is important in health care. The impaired production of thyroid hormones (thyroid gland-TG) affects bones’ mineral content, particularly, reticular tooth plate.

**Method:** The following combinations were studied: teeth with caries from the patients with hypo- or hyperthyreosis; teeth without caries from healthy patients. Molars were extracted from 18 to 50 years old patients. The scanning microscope was used to determine the structure and the microelements.

**Results:** The patients with TG pathology have more carious lesion than healthy people. Caries is localized in cervical region; the progression is faster. Nanoscale pictures of conditionally healthy teeth were examined and found caries before the appearance of external changes of enamel. In enamel with carious lesion, calcium decreased to one-fourth (10,52 %) of the normal enamel–40,37 %; in dentine, calcium decreased to one-sevenths (5,7 %). In enamel with caries, phosphorus decreases by a half; in dentine, phosphorus decreases to one-fourth. Due to caries, the amount of oxygen is increased: two times in enamel, 1,2 time in dentine. Pic.1.

**Conclusion:** We identified changes in the structure of hard tissues teeth in patients with pathology of TG.

**Pic.1. Tooth enamel in caries. A. Scanning Electron Microscopy x 1000. B. Atomic force microscopy:** 
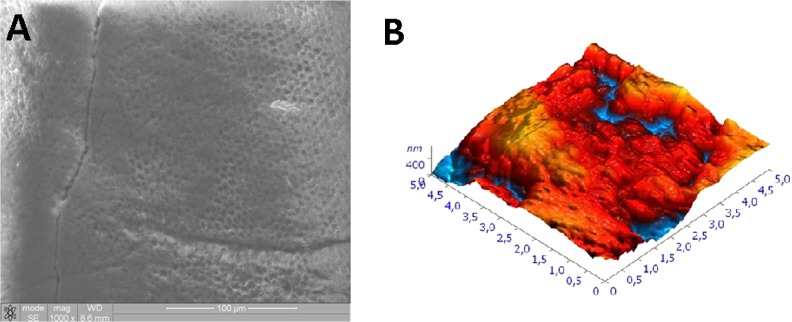


**PS-03-062**

**Nodal involvement in advanced laryngeal squamous carcinoma is associated with Ep-CAM but not E48 expression**

A. Nadal ^*^, C. Romeu, X. Farré, A. Cardesa

^*^Hospital Clínic Barcelona, Dept. of Pathology, Spain

**Objective:** The analysis of Ep-CAM and E48 expression and their relationship with differentiation and development of nodal metastases in laryngeal carcinoma.

**Method:** Conventional immunohistochemical analysis with monoclonal antibodies against Ep-CAM and E48 in a series of 66 laryngeal carcinomas in stage 3 or 4 and in adjacent non-neoplastic epithelia. Comparison with tumor histologic differentiation according to Broders grading and with presence of nodal metastases.

**Results:** Ep-CAM expression is higher in carcinoma than in normal epithelia. 96 % of carcinomas show Ep. CAM expression. High levels of expression (>50 % of cells) are associated with nodal metastases (*p* = 0.001) and poor differentiation (grades 1–2 vs 3–4, *p* = 0.003). E48 expression is higher in normal epithelia than in carcinoma. Poorly differentiated carcinomas had less positive cells than well to moderately differentiated carcinomas (*p* = 0.001).

**Conclusion:** Ep-CAM and E48 expression are associated with cell differentiation but in opposite ways. Ep-CAM could participate in the development of nodal metastases in laryngeal squamous carcinoma according to the association observed between expression and nodal metastases. Selection of satges 3 and 4 prevents a biass against nodal metastases caused by stages 1 and 2.

**PS-03-063**

**Is there any relationship between** ***Helicobacter pylori*** **and laryngeal carcinoma?**

I. Yilmaz ^*^, U. Berber, Z. Kucukodaci, G. Narli, A. Haholu, D. Demirel, E. Erkul

^*^Gata Heh, Dept. of Pathology, Istanbul, Turkey

**Objective:** Helicobacter Pylori (HP) infection is a major risk factor for the development of gastric cancer. Several studies have been done to investigate the role of HP in laryngeal disorders. The results of the studies on the presence of HP in benign and malignant laryngeal tissue are conflicting. The purpose of this study was to investigate the presence of HP in laryngeal squamous cell carcinoma by a sensitive method Real time polymerase chain reaction assay (RT-PCR).

**Method:** Formalin-fixed-paraffin-embedded tissue samples from 74 patients with laryngeal cancer who underwent total/partial laryngectomy in our hospital between 2005 and 2011 were used in this study. A sensitive RT-PCR assay was used to detect the presence of HP in the laryngeal carcinoma.

**Results:** HP was detected in only one case. The positive case was also investigated for the existence of HP with histopathologic evaluation and HP immunohistochemistry. However, we could not detect HP in this case with both methods. We suggested that HP in this case may contaminated from stomach to laryngeal tissue during surgery.

**Conclusion:** This study revealed that HP might not contribute to the pathogenesis of laryngeal carcinoma. Further studies with more patients are needed to clarify the role of HP in laryngeal carcinomas.

**PS-03-065**

**Detection of P 16 INK4a in laryngeal papillomatosis and its relationship with dysplasia**

E. Ben Brahim ^*^, A. Ben Salem, R. Jouini, W. Koubâa, I. Msakni, O. Khayat, M. Belhaj Salah, A. Souissi, A. Gharbi, C. Mbarek, A. Chadli

^*^Habib Thameur Hospital, Pathology, Tunis, Tunisia

**Objective:** Laryngeal papillomatosis (LP) is a rare and benign neoplasm, caused by human papillomaviruses (HPVs) especially HPV 6 and 11. The diagnosis of dysplasia in these tumors is still a source of pathologist’s disagreement. In cervical dysplasia, the use of anti p16INK4a (p16) antibody in immunohistochemistry allows having a better inter- pathologists reproducibility. We therefore sought to evaluate the diagnostic utility of anti P16 in detection of dysplasia in LP leading to better inter-pathologists reproducibility.

**Method:** A total of 17 samples of LP were analyzed for p16INK4a protein (p16) expression by immunohistochemistry and for human papillomavirus (HPV) infection using in situ hybridization (ISH) and polymerase chain reaction (PCR).

**Results:** Using ISH and PCR, 27 low risk HPV (LR-HPV) infections, 10 high risk HPV (HR-HPV) infection and one infection with an undetermined risk’s HPV were detected. The immunohistochemical study using anti P16 showed a non significant staining in all cases even the ones diagnosed histologically as dysplastic lesions or having a HR-HPV infection.

**Conclusion:** Our study find that anti P16 is a non specific marker of dysplasia in laryngeal lesions and that different molecular events, in comparison to the cervical ones, could be involved in progression of LP to the carcinoma.

**PS-03-066**

**Laryngeal laser excision mounted in cucumber. Our experience**

M. Giles Lima ^*^

^*^The James Cook University Hospital, Cellular Pathology, Middlesbrough, United Kingdom

**Objective:** Excisional biopsy is currently the preferred treatment option for early malignant laryngeal lesions. Assessment of margins is challenging in such small specimens. Using a mounting media can improve its accuracy. We present our experience using the described -cucumber technique-

**Method:** Cases of laser excision of vocal cord lesions were retrospectively retrieved from Head and Neck departmental database. Those whose specimens were placed and glued to a de-hydrated piece of cucumber were identified in the Pathology database and reports obtained. All specimens had been conventionally processed and stained.

**Results:** 44 cases were selected, 29 were reported as invasive squamous cell carcinoma, 13 as dysplasia or in situ carcinoma and 2 had no tumour conditions. 71 % of the cases of invasive squamous cell carcinoma, dysplasia or in situ carcinoma had the peripheral margin reported and often specified along with other prognostic factors as depth of invasion. In 7 % of the cases the margins were not assessable and 21 % of the cases were suboptimal but reportable.

**Conclusion:** The use of cucumber as mounting support allows a confident diagnosis of involvement of specific margins in small biopsies as laryngeal laser excisions.

**PS-03-067**

**Actinic cheilitis: Epithelial thickness changes and the presence of mutated p53 protein**

C. Fabiana Joca de Arruda ^*^, G. Sanchez Nagata, K. Lopez Ortega, M. Trierveiler Martins

^*^FOUSP, Oral Pathology, São Paulo, Brazil

**Objective:** Actinic cheilitis (AC) is a potentially malignant disorder caused by ultraviolet (UV) radiation which shows some specific epithelial characteristics, among them, variation in the epithelium thickness. It is known that the TP53 gene is a target of UV radiation. The objective of this research was to determine the variation of epithelium thickness in AC and to correlate it to the expression of mutated p53 protein.

**Method:** The presence of acanthosis, atrophy, extreme atrophy or normal epithelial thickness was evaluated in 456 AC cases. After this, 20 cases of each of these thicknesses were selected and submitted to the PAb240 antibody against mutated p53 protein by means of immunohistochemistry.

**Results:** Among the 456 cases studied, 38.8 % showed acanthosis, 34.8 % atrophy, 6.1 % extreme atrophy and 20.1 % normal epithelial thickness. The presence of the mutated p53 protein was seen in 40 % of the cases with acanthosis, 35 % with atrophy, 40 % with extreme atrophy and 24 % with normal thickness. The difference was not statistically significant.

**Conclusion:** Most of the cases showed epithelium thickness changes (acanthosis or atrophy) but this does not correlate to the genetic alterations in the TP53 gene caused by UV radiation.

**PS-03-068**

**Human papillomavirus detection in archived histology samples of oropharyngeal squamous cell carcinomas: Correlation of in situ hybridization and polymerase chain reaction**

K. Vesely ^*^, R. Tachezy, H. Binkova, Z. Horakova, E. Foltynova, M. Salakova, J. Klozar, R. Kostrica

^*^Brno, Czech Republic

**Objective:** Human papillomavirus (HVP) has been recognized as an etiologic and prognostic factor in head and neck squamous cell carcinomas (HNSCC). In our study we analyzed archival (2001–2010) formalin-fixed paraffin-embedded tissues from patients with primary oropharyngeal squamous cell carcinomas for comparison of different detection methods.

**Method:** HPV detection was done by means of in situ hybridization (ISH) and polymerase chain reaction (PCR), the detection of indirect marker of active viral infection p16 was evaluated by immunohistochemistry.

**Results:** In our set of samples ISH was more sensitive than PCR with general primers most likely due to the degradation of nucleic acids caused by less careful fixation procedures. The positivity for HPV as assessed by ISH was strongly correlated both with the results of p16 immunohistochemistry and PCR HPV DNA detection.

**Conclusion:** We concluded that ISH is a clinically relevant method for molecular testing of HPV infections in archived biopsy material of HNSCC.

**PS-03-069**

**Oropharygeal squamous cell carcinoma (OPSCC): A clinicopathological study of 102 cases**

M. C. Etxezarraga ^*^, G. Cancho, L. Ortega, J. C. Lopez Duque, N. Liaño, J. Bilbao, M. M. Ramirez, C. Ereño

^*^Hospital Universitario Basurto, Dept. de Anatomía Patológica, Bilbao, Spain

**Objective:** To study the prognosis significance of p16 in patients with OPSCC and to evaluate the relationship between p16 and human papillomavirus (HPV).

**Method:** A total of 102 of OPSCCs diagnosed during 10 years were randomly selected, their histological sections were revaluated and follow-up was retrieved from medical files. P16 and P53 immunostaining was performed. HPV status was determined by PCR and in situ hybridation. Kaplan-Meier (KP) log- rank test was performed using SPSS software.

**Results:** The median follow-up period was 4 years. The 53.9 % (55/102) results p16(+) and 46 % (47/102) were p16(−). The 92 % (51/55) of p16(+) cases were in males with an average of 59,4 years (range 38–84). 73 % of p16(+) tumors were well-moderately differentiated SCCs, mostly located in tonsillar fossae. Viral DNA was present in 40 % (22/55) of p16 (+) cases, being HPV 16 type the most frequent. Five years overall survival was 63 % for p16(+) versus 36 % for p16(−) patients: *p* < 0,05 by KP log- rank test.

**Conclusion:** In OPSCC p16 expression predict a better overall survival. In our series HPV positive cases rate was lower than other published series.

**PS-03-070**

**Human papillomavirus, EGFR mutation and loss of heterozygosity at 17p13 and 9q22 in tonsillar squamous cell carcinoma**

N. Kim ^*^, S. J. Cho, Y. S. Roh

^*^Gachon University, Gil Medical Center, Pathology, Incheon, Republic of Korea

**Objective:** Head and neck squamous cell carcinoma (SCC) is the sixth most common malignancy with an increasing incidence of tonsillar SCC in several countries, and epidemiologic evidences demonstrate that up to 50 % of head and neck SCCs harbor human papillomavirus (HPV). This study was performed to determine the clinical significance of microsatellite alterations at 17p13 and 9q22, mutations in the epidermal growth factor receptor (EGFR) along with their association with HPV infections in tonsillar SCCs.

**Method:** The HPV was detected by type-specific polymerase chain reaction. Loss of heterozygosity (LOH) was analyzed by markers flanking chromosome 17p13 and 9q22.

**Results:** Thirty-four male and six female patients of tonsillar SCCs were included. LOH was detected in 57.5 % (23/40) at 17p13 and 45 % (18/40) at 9q22 with EGFR mutation (17.5 %, 7/40) and HPV-positivity (45 %, 18/40) the most common genotype was HPV 16 and 18 (6/18 and 6/18, 33.3 % and 33.3 %, respectively). Ten percent of HPV-infected tonsillar SCCs were multiple infection by HPV 11, 18, 40, 45, 52, 58 and 70.

**Conclusion:** Albeit small series, our results showed EGFR mutation and LOH were not associated with HPV-status, which confers a better prognosis. Further investigation is needed to clarify its association.

**PS-03-071**

**Predictive clinicopathological factors and immunohistochemical markers of locoregional control in patients with early-staged tongue cancer treated by brachytherapy**

J. Laco ^*^, I. Sirák, L. Tucek, M. Hodek, P. Paluska, L. Kašaová, S. Paulíková, M. Vošmik, M. Cvanová, M. Halámka, J. Petera

^*^Faculty of Medicine, Dept. of Pathology, Hradec Kralove, Czech Republic

**Objective:** The aim of the study was to investigate predictive clinicopathological factors and immunohistochemical markers of locoregional tumor control in patients with early-staged squamous cell carcinoma of tongue treated by brachytherapy.

**Method:** Twenty-four patients treated during the years 2001–2010 were enrolled in the study. In every tumor, TNM status, grade, resection margin, and depth of invasion were recorded. Using immunohistochemistry, both expression (percentage of positive tumor cells) and expression intensity (mild, moderate or strong) of p16 protein, epidermal growth factor receptor, nuclear factor-kappaB, hypoxia-inducible factor-1alpha, human epidermal growth factor receptor-2, Ku-80, cyclooxygenase-2 and vascular endothelial growth factor (VEGF) were evaluated. Correlation between disease-free survival and abovementioned parameters was performed using the NCSS 2004 software.

**Results:** Median follows-up was 37 months. The estimated 5-year local control was 80 % and locoregional control was 62 %. Only depth of tumor invasion (median 4 mm; range 1–20 mm) and expression intensity of VEGR (mild in 6, moderate in 17, and strong in 1 case) were significantly predictive of worse disease-free survival in Cox multivariate analysis (*p* = 0.018; *p* = 0.016).

**Conclusion:** Strong expression of VEGF and deep tumor invasion may be significant negative predictive markers of disease-free survival in tongue cancer patients treated by brachytherapy alone.

**PS-03-072**

**Tumor budding and depth of invasion predict the prognosis of patients with early stage oral tongue squamous cell carcinoma**

A. Almangush ^*^, I. Bello, J. Hagström, Y. Soini, P. Koivunen, R. Grénman, I. Leivo, T. Salo

^*^Haartman Institute, Dept. of Pathology, University of Helsinki, Finland

**Objective:** Oral (mobile) tongue squamous cell carcinoma (OTSCC) is characterized by poor survival even in cases of low stage (T1-T2). This multicentre study examined the value of tumor budding, depth of cancer invasion, histologic risk score (HRS), and cancer-associated fibroblasts (CAFs) in predicting the disease-specific survival of T1N0M0 and T2N0M0 OTSCC.

**Method:** A whole population-based patient material from all five university hospitals in Finland was collected for this study. Hematoxylin and eosin-stained slides were used to assess tumor budding, depth of cancer invasion, and histologic risk score in 233 cases of OTSCC. For identification of CAFs, immunohistochemical staining for α-SMA was carried out.

**Results:** Tumor budding and depth of tumor invasion predict disease-specific survival (*p* = 0.009 and 0.02, respectively). On the other hand, HRS and CAFs did not classify patients with early OTSCC into low and high-risk groups. However, worst pattern of invasion (WPOI), a component of HRS proved to be an independent prognosticator (*P* = 0.005).

**Conclusion:** Tumor budding, depth of invasion and worst pattern of invasion demonstrated the best predictive value in early OTSCC. Our findings may provide an approach for individualized patient management of early OTSCC.

**Histological appearance of tumor budding:** 
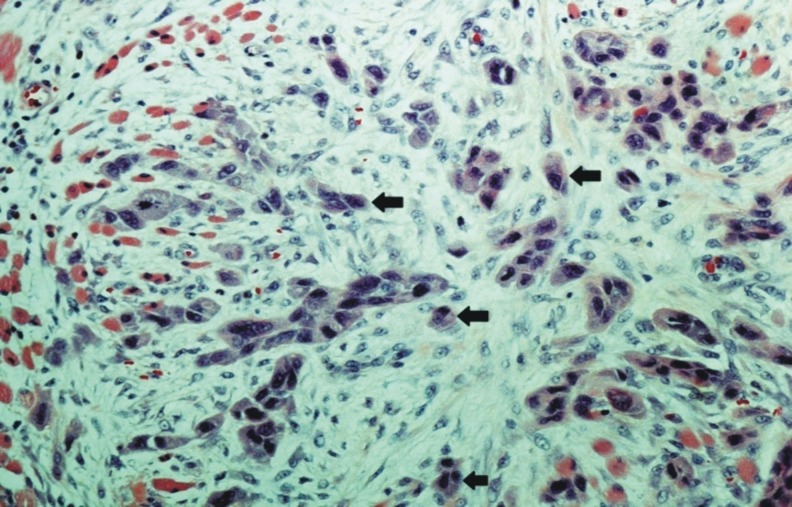


**PS-03-074**

**Stromal myofibroblasts in tongue squamous cell carcinoma of young patients**

A. R. Santos-Silva^*^, F. P. Fonseca, R. D. Coletta, M. A. Lopes, O. P. Almeida, R. Carlos, A. M. P. Soubhia, A. C. P. Ribeiro, K. D. Hunter, P. M. Speight

^*^Piracicaba Dental School, Dept. of Oral Diagnosis, Brazil

**Objective:** The increase of oral squamous cell carcinomas (OSCC) affecting young people has been demonstrated worldwide. However, little is known about the biological nature and course of the disease. Therefore, the aim of this study was to evaluate the presence of myofibroblasts in the stromal compartment of OSCC affecting young patients.

**Method:** In this international multi-centre study, 29 patients younger than 40 years old affected by tongue SCC were retrieved from 4 different oral pathology services and investigated for the presence of myofibroblasts by immunohistochemical reactions against αSMA. The results obtained were correlated to ploidy status of the tumors and compared to the expression observed in cases of OSCC affecting older patients previously matched for tumor site and AJCC grade.

**Results:** 58.6 % of the young patients presented stromal myofibroblasts, whereas 70 % of the older patients revealed positivity for αSMA. No significant difference could be seen when comparing both groups and no correlation could be obtained with higher tumor grades and DNA content.

**Conclusion:** No significant difference exist between the presence of stromal myofibroblasts of tongue SCC affecting young and old individuals, suggesting that this stromal component would not be responsible for the clinical behavior of OSCC in young people.

**PS-03-075**

**Usefulness of Human Papilloma Virus (HPV) status in Squamous Cell Carcinoma (SCC) of the Upper Aerodigestive Tract (UADT): A clinicopathological study of 406 cases**

M. C. Etxezarraga ^*^, M. Zufiaurre, J. A. Nieto, N. Liaño, J. C. Lopez Duque, J. Bilbao, M. M. Ramirez, C. Ereño

^*^Hospital Universitario Basurto, Dept. de Anatomía Patológica, Bilbao, Spain

**Objective:** To study the prognosis value of HPV status in patients with SCC of the UADT and try to establish the clinicopathological features and overall survival (OS) differences between positive versus negative patients.

**Method:** A total of 406 SCCs of UADT diagnosed during 10 years were randomly selected, were revaluated histologically and follow-up was retrieved from medical files. P16 and P53 immunostaining was performed. HPV status was determined by PCR and in situ hybridation. Kaplan-Meier (KP) log- rank test was performed using SPSS software.

**Results:** p16 expression was found in 50 % (204/406) of all UADT-tumors. HPV was detected in 75(36 %) cases, being types 16 and 33 the most frequent. Among HPV-positive cases, 35(46 %) present diffuse nuclear and cytoplasmic (DNC) staining. Koilocytosis was present in 8 (66 %). The more most common site was supraglottic larynx (12/35). Mean age was 60,1 years (20–91 range). Male predominate (360/406). The median follow-up period was 4,2 years. OS analysis between 75-HPV(+) vs. 129-HPV(−) patients was statistically significant: *p* < 0,01 by KP log-rank test.

**Conclusion:** Ours results prove the value of HPV-positive status to predict a better OS survival. The pattern of p 16 DNC immunostaining is associated with viral status.

**PS-03-076**

**Cytokine profile changes after oral squamous cell carcinoma cells and macrophages co-culture**

D. Antunes ^*^, D. M. Guimarães, M. B. Lima, F. D. Nunes

^*^São Paulo, Brazil

**Objective:** Inflammation as a key player in the development of different cancers, especially of epithelial origin, has been object of studies for several years. Macrophages are important components of this process and assume different roles in cancers as, for instance, increased cytokine production. Changes in the production of inflammatory cytokines may lead to altered cell survival and proliferation. Objective: to investigate differences in the expression pattern of inflammatory cytokines when macrophages are co-cultured with oral squamous cell carcinoma (OSCC) cells.

**Method:** One macrophage (U937) and two oral squamous cell carcinoma cells lineages (SCC4 and SCC9) were cultured alone and together. Analysis of the cytokine profile was performed by enzyme immunoassay using the Luminex® xMAP™ system.

**Results:** All studied cytokines were expressed in OSCC cells and macrophages at variable levels. IL-6, IL-8 and GM-CSF were expressed at very high levels in both lineages of OSCC. Macrophages also expressed very high level of IL-8. When both OSCC cell lineages were co-cultured with macrophages significant increase of IL-1β, IL-2, IL-4, IL-6, TNF-α and G-CSF was observed.

**Conclusion:** The results show a significantly modified expression profile of inflammatory cytokines when OSCC cells and macrophages are cultured together. The increased cytokines were previously associated with a modified neoplastic behavior.

**PS-03-078**

**Low prevalence of Human Papilloma Virus (HPV) infection in biopsies of oral leukoplasia**

J. M. Suárez Peñaranda ^*^, P. Gandara Vila, C. Aliste, M. Perez Sayans, J. M. Gandara Rey

^*^Hospital Clinico Santiago de, Compostela, Dept. de Patalogia, Santiago de Compostela, Spain

**Objective:** We have tested the presence of HPV DNA in biopsies from oral leukoplasias to evaluate its role as a contributor in the early steps of oral cancer development

**Method:** DNA was obtained from 43 biopsies and tested for the presence of HPV DNA with the method CLART HPV2 (Genomica). Histopathology evaluation of dysplasia was made independently by two pathologists, according to the WHO classification.

**Results:** HPV DNA was present in only 10 biopsies: HPV 16 in 5, HPV 6 in 2, HPV 51 in 2 and HPV 33, 43 and 56 in one each. Cases with positive for HPV showed moderate dysplasia (1), severe dysplasia/in situ carcinoma (2) or infiltrating carcinoma (2). Cases negative for HPV showed mild dysplasia (7), severe dysplasia/in situ carcinoma (3) or infiltrating carcinoma (2 cases). No statistical correlation could be found between the presence of HPV DNA and the degree of dysplasia or the presence of carcinoma.

**Conclusion:** The prevalence of HPV infection in oral leukoplasias is low in our patients and not related with the degree of dysplasia or the presence of carcinoma. HPV infection does not seem to be an important etiopathogenic factor in the early stages of development of oral squamous cell carcinoma.

**PS-03-079**

**Tumor deposits in head and neck squamous cell carcinomas**

S. Sarioglu ^*^, S. Iplikci, N. Akbulut, B. Aydin, E. Dogan, M. Unlu, H. Ellidokuz, E. Ada, F. Akman, A. O. Ikiz

^*^Dokuz Eylul University, Dept. of Pathology, Izmir, Turkey

**Objective:** Tumor deposits (TDS) were described for colorectal and gastric carcinomas with prognostic implications. The aim of this study is to evaluate TD in head and neck squamous cell carcinomas (SCC).

**Method:** Sections from neck dissection specimens from 84(60 %) laryngeal, 31(22,1 %) oro/hypopharyngeal, 25(17,9) oral SCC cases were reevaluated according to the criteria described for the TDs of colorectal carcinomas. The relation of TDs with clinicopathological findings were analyzed statistically.

**Results:** Among 140 cases (63,66+54,78 months follow up), 24(17,1 %) had tumor deposits [12(4,3 %) laryngeal, 5(20 %) oro/hypopharyngeal, 7(22,6 %) oral cases (*p* = 0,53)]. Disease recurrence was identified in 40(28,6 %) of the patients, this was more frequent in cases with TDs [13(13,4 %) versus 11(27,5 %) *p* = 0,048] and especially for cases with distant metastasis [15(12,9 %) versus 9(42,7 %) *p* = 0,003)]. Fifty one (36,4 %) died during follow up and of these 38 were related to disease. Death was more frequent in cases with TDs. At 12 and 24 months 94,7 % and 85,2 % of patients without TDs were alive but this was 64,1 % and 37,9 % for cases with TDs respectively (*p* = 0,005).

**Conclusion:** TDs may be very important prognostic factors in head and neck SCC.

**A tumor deposit and overall survival:** 
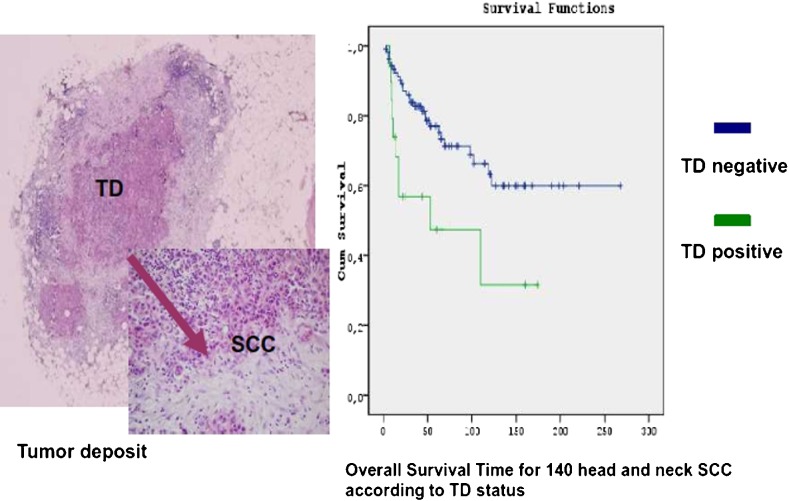


**PS-03-080**

**The role of HOX genes in Head and Neck Squamous Cell Carcinoma (HNSCC)**

K. Hunter ^*^, F. Hakami, L. Darda, D. Lambert, C. Murdoch, R. Morgan, P. Woll

^*^University of Sheffield, Oral Pathology, United Kingdom

**Objective:** Molecular alterations in HNSCC are variable and related to the site and stage of disease. Our previous HNSCC microarray data showed differential expression of a number of HOX genes. These are developmental patterning genes whose expression is altered in many cancers.

**Method:** Expression of all 39 HOX genes was assessed by qPCR in HNSCC cells. The 2 most differentially expressed were assessed by WB and IHC in oral cells and tissues. Expression was altered by transient over-expression and siRNA into low and high-expressing cells respectively. The phenotype was assessed by MTS, fibronectin adhesion and transwell migration assays. Agilent microarray analysis was performed to identify novel targets and differentially activated pathways.

**Results:** HOXD10 and HOXB9 were expressed at low levels in normal cells and precancerous cells and at high levels in most primary tumours. HOXD10 expression was very low in lymph node metastases. Over-expression or silencing affected proliferation, adhesion, and migration. Direct/indirect targets of HOXD10 identified by microarray analysis include miR146a and may explain some of these effects.

**Conclusion:** The elevated expression of HOXD10 and HOXB9 allows tumours to grow whilst HOXD10 loss in metastases may promote cell survival. These 5-prime HOX genes may represent novel therapeutic targets in HNSCC.

**PS-03-081**

**Adenoid dysplasia of the oral mucosa**

B. Bunn ^*^, K. Hunter, S. A. Khurram, W. van Heerden

^*^University of Pretoria, Oral Pathology & Oral Biology, Johannesburg, South Africa

**Objective:** To present the proposed in-situ component of the rare mucosal acantholytic variant of squamous cell carcinoma (ASCC). An immunohistochemical appraisal of intraoral lesions displaying features of “adenoid dysplasia” is reported. The immunoprofile is compared with that of invasive ASCC, typical dysplasia and conventional keratinising squamous cell carcinoma (SCC).

**Method:** Immunohistochemical staining for E-cadherin, β-catenin, EMA, AE1/3, p63, vimentin and SMA was performed on formalin-fixed, paraffin-embedded tissue sections of adenoid dysplasia, ASCC, conventional moderately differentiated, keratinising SCC and moderate epithelial dysplasia.

**Results:** The immunoreactivity in cases designated as adenoid dysplasia recapitulates the immunoprofile detected in invasive ASCC. E-cadherin and β-catenin were most useful in this regard with little to no E-cadherin staining present within the acantholytic cells and upregulation of β-catenin. This contrasted starkly with the surrounding epithelium and in cases of conventional SCC and dysplasia.

**Conclusion:** Cases of intra-oral adenoid dysplasia were morphologically and immunophenotypically similar to invasive ASCC. ASCC represented an aggressive variant of SCC with an increased propensity for distant metastasis. It is postulated that adenoid dysplasia be associated with a greater risk of malignant transformation.

**PS-03-082**

**EMA expression and DNA ploidy in oral epithelial dysplasia**

B. Bunn ^*^, W. van Heerden

^*^University of Pretoria, Oral Pathology & Oral Biology, Johannesburg, South Africa

**Objective:** Microscopic assessment of histological grade is the most reliable method of determining the malignant transformation potential of dysplastic lesions. This study aimed to determine whether a relationship exists between EMA expression, DNA ploidy and histological grade in oral epithelial dysplasia.

**Method:** EMA immunohistochemical staining and DNA ploidy analysis by means of high resolution image cytometry was performed on formalin-fixed, paraffin embedded tissue sections previously diagnosed as mild (*n* = 11), moderate (*n* = 11) and severe (*n* = 13) dysplasia. Lesions were classified as either diploid or aneuploid while EMA immunoreactivity was semi-quantitatively assessed. In addition, the staining pattern and distribution were also documented. Normal oral epithelium and invasive squamous cell carcinoma served as negative and positive controls for EMA expression.

**Results:** EMA immunoreactivity increased in dysplasia, the intensity and distribution of which correlated with histological grade and DNA ploidy. Basal cell and/or suprabasal positivity were more frequently noted in lesions of a high-grade nature and were associated with an increased incidence of aneuploidy. EMA positivity within the suprabasal cells correlated with a lower histological grade in lesions which were more frequently diploid.

**Conclusion:** The immunolocalisation, intensity and distribution of EMA staining correlated well with histological grade and DNA ploidy. Both EMA expression and DNA ploidy analysis represented useful adjuncts to histological grading for determining the malignant potential of dysplastic oral epithelial lesions.

**PS-03-084**

**Acetylsalicylic acid efficacy on in vitro proliferation inhibition of oral squamous cell carcinoma**

R. Moura ^*^, D. Santos Pinto Junior

^*^University of Sao Paulo, Oral Patology, Brazil

**Objective:** The aim of this study was to analyze the effect of the Acetylsalicylic acid on squamous cell carcinoma cell line (OSCC9) through analysis of the cell viability and death in OSCC9 cells treated with acetylsalicylic acid.

**Method:** The SCC9 cell culture was performed and the cells were plated when the cell monolayer reaches 70 % confluence. The Acetylsalicylic acid cytotoxicity was analyzed by [3-(4,5-dimethylthiazol-2-yl)-5-(4- sulfophenyl)-2H-tetrazolium (MTS) cell proliferation assay. Viable cells reduce tetrazolium compounds into colored formazan products that were detected as an absorbance change with a spectrophotometer. The amount of formazan color produced is directly proportion to the number of viable cells. The standard of apoptosis was evaluated using the TdT-mediated dUTP nick-end labeling (TUNEL) assay, detecting apoptotic cells that undergo DNA degradation during the late stages of apoptosis. Untreated cells were used as control.

**Results:** Dose–response curves were generated. Based on obtained IC50 (50 % inhibitory concentration) values, the Acetylsalicylic acid concentration were determined the in 40μM. TUNEL assay shows Acetylsalicylic acid causes apoptosis in dose of 40 μM in 24 h.

**Conclusion:** Acetylsalicylic acid can be an effective therapeutic agent since it has shown great efficacy in the cellular proliferation inhibition on the OSCC9 cell line.

**PS-03-085**

**Methylation profile and amplification of homeobox genes in oral squamous cell carcinoma**

F. Nunes ^*^, C. Esteves, M. F. Rodrigues, F. Xavier

^*^FOUSP, São Paulo, Brazil

**Objective:** Genetic and epigenetic alterations have an important role in carcinogenesis, especially in allowing the overexpression of oncogenes and silencing the tumor suppressor genes. It is well known that deregulation of genes controlling growth and embryonic development is implicated in carcinogenesis, including homeobox genes. The methylation profile and amplification of HOXD10, HOXD11, PROX1, ZHX1, IRX4, HOXC13, HOXA5, HOXA7, HOXA9, HOXB5 genes were studied in oral squamous cell carcinoma

**Method:** DNA methylation was evaluated in 40 fresh tumor and non-tumor samples by qPCR using a customized plate for quick and precise detection of CpG islands methylation. Gene amplification was analyzed on the same samples by qPCR.

**Results:** All homeobox genes showed a low percentage of methylation. However, when amplification was related to clinical parameters PROX1 and IRX4 genes were associated with an increased risk of death associated with disease, whereas HOXA5 was associated with lymph node involvement, and a low HOXB5 with the occurrence of metastasis. Patients who showed methylation of the latter gene presented short-term survival.

**Conclusion:** The findings presented in this study provide new information about homeobox genes as potential biomarkers for diagnosis and predictive of tumor behavior for oral squamous cell carcinomas.

**PS-03-086**

**Expression of adhesion molecules in an in situ model of tumorigenesis**

E. Martinez ^*^, I. de Souza, A. P. Demasi, N. S. Araújo, V. C. Araújo

^*^São Leopoldo Mandic Institute, Oral Pathology, Sao Paulo, Brazil

**Objective:** Cell-cell interactions are important regulators of both normal and abnormal biological processes. Catenins interact with an intercellular protein (Ecadherin) regulating a strong cell-cell cohesion. Both proteins, in concert with the extracellular matrix are involved in many cell-signaling pathways such as proliferation and migration during the neoplastic process. Thus, the aim of this study was to analyse cell-cell interactions on sites that mimic an in situ situation when malignant squamous cells are surrounded by benign myoepithelial cells from pleomorphic adenoma.

**Method:** Benign myoepithelial cells from pleomorphic adenoma were cultured with non-filtered malignant conditioned medium from squamous cell carcinoma (CAL27, ATCC) in fibronectin substratum. βcatenin and Ecadherin immunoexpression were examined in these in situ areas by indirect immunofluorescence.

**Results:** The carcinoma malignant cells strongly expressed βcatenin and Ecadherin in the cell surfaces, mainly in further periods. In contrast, βcatenin and Ecadherin were not immunoexpressed in the neoplastic benign myoepithelial cells from pleomorphic adenoma during the neoplastic process.

**Conclusion:** The present results suggested that βcatenin-Ecadherin complex, in the proposal in vitro model, has had an important role in the maintenance of the carcinoma malignant cell clusters, highlighting their importance in the tumoral process under the influence of the extracellular matrix. (Grants from FAPESP 2011/14053-3)

**PS-03-087**

**In vitro influence of substance P on squamous cell carcinoma**

I. Souza ^*^, V. Montalli, M. H. Napimoga, F. Passador-Santos, V. Araújo, E. Martinez

^*^São Leopoldo Mandic Institute, Oral Patology, Guarulhos, Brazil

**Objective:** In the neoplastic process, the tumor microenvironment plays a fundamental role in the cancer behavior. Among the components related to tumor progression, studies have been indicated substance P as an important factor contributing for tumoral behavior. Thus, the purpose of the present study was to evaluate the in vitro influence of substance P, at different doses (10–8M and 10–6M), in the cell proliferation, invasion and the tissue matrix inhibitors (TIMPs) expression on squamous cell carcinoma cells.

**Method:** Cells from squamous cell carcinoma of the tongue (ATCC, CAL 27) were used. For the invasion assay, the cells were cultured in transwell chambers and the lower surface was then counted. TIMP-1 and TIMP-2 levels were evaluated by ELISA.

**Results:** There was a reduction on cell proliferation in all studied concentrations. However, substance P influenced the cell invasion, as well as, the levels of TIMP-1 and TIMP-2. The number of invaded cells was higher and TIMP-1 and TIMP-2 levels decreased in the presence of substance P.

**Conclusion:** The present in vitro results demonstrated that substance P increased the invasive potential of squamous cell carcinoma cells indicating its influence on tumor behavior. (Grants from Fapesp 2011/14053-3)

**PS-03-088**

**Immunohistochemical expression of the PI3K-AKT-mTOR pathway in epithelial dysplasia, reactive epithelial lesions and squamous cell carcinomas**

F. Martins^*^, S. de Sousa, E. Santos, D. dos Santos Pinto Jr, S. B. Woo, M. Gallottini

^*^University of Sao Paulo, Oral Pathology, Brazil

**Objective:** To evaluate the immunohistochemical expression of the AKT-mTOR pathway in oral lesions diagnosed as low-grade (LGD) and high-grade dysplasia (HGD), squamous cell carcinoma (SCC), normal mucosa (NM) and frictional keratosis (FK).

**Method:** 186 cases, divided into 5 groups. Clinical information of the lesions was compiled. The labeling pattern of pAKT, pmTOR, PS6, and p4EBP1 antibodies were investigated by two pathologists and quantified by images using a photomicroscope (AxioCam®, Carl Zeiss MicroImaging GmbH, Germany). The variables were tested using chi-square, ANOVA F and univariate logistic regression analysis.

**Results:** Among all the cases of NM, positivity was found only pS6 (50 %); in FK cases there was positivity for pS6 (54.8 %) and 4EBP1 (22.6 %). In LGD, immunoreactivity was observed for pS6 (67.4 %), pAKT (56.2 %), p4EBP1 (41.7 %) and pmTOR (29.2 %), in the HGD group, positivity was found for pS6 (74 %), pAKT (68 %), p4EBP1 (44 %) and pmTOR (28 %). In SCCs, immunoreactivity was found for pAKT (83.3 %), PS6 (77.4 %), p4EBP1 (50 %) and pmTOR (50 %). Statistically significant differences were observed between all study groups and the proteins, except for pS6.

**Conclusion:** All the studied proteins are potential biomarkers to differentiate normal tissues from dysplastic lesions and SCC, but only pAKT and pmTOR proteins seem to be related to oral carcinogenesis.

**PS-03-089**

**Analysis of PTEN/AKT in the development of human oral tissue and oral squamous cell carcinoma: Role of malignant transformation**

M. Buim ^*^, S. Lourenço, F. A. Soares

^*^Hospital A C Camargo, Pathology, São Paulo, Brazil

**Objective:** Oral squamous cell carcinoma (OSCC) is a prevalent public health problem and is characterized by high degree of local aggression and lymph node metastasis. Signaling through the PTEN/AKT pathway is responsible for balancing survival and apoptosis cell. The goal of this study was to analyze the association of the PTEN and AKT expression with clinicopathological feature of the OSCC patients

**Method:** Expression of PTEN and AKT gene/protein was investigated in 68 cases of OSCC, obtained from the files of Anatomic Pathology Department from A.C. Camargo Hospital (Brazil), by qRT-PCR and IHC.

**Results:** Low PTEN gene and protein expression occurred in 66 % and 63 %, respectively, OSCC cases. However, no association was observed between this molecule and clinicopathological parameters. High phosphorylate AKT (pAKT) protein expression was associated with poor/moderated differentiated histological grade (*p* = 0.029); no associated was observed between pAkt protein expression and others clinicopathological parameters. AKT gene expression, by qRT-PCR, was associated with clinical stage (0.034), perineural infiltration (*p* = 0.014) and tumor size (*p* = 0.037).

**Conclusion:** Our results suggest that downregulation of PTEN can be important to OSCC tumorigenesis and can contribute to oncogene activation, and high pAKT expression might be an unfavorable prognostic marker in OSCC.

**PS-03-090**

**Metallothionein is not associated with metastatic behaviour in oral squamous cell carcinoma**

S. Cardoso ^*^, P. R. Faria, A. C. Campos, L. B. Muniz, A. M. Loyola

^*^Federal University of Uberlând, Pathology, School of Dentistry, Uberlândia, Brazil

**Objective:** Overexpression of metallothionein (MT) is associated to worst prognosis for many human cancers, including oral squamous cell carcinoma (OSCC). Recent papers reported association of MT with metastatic behavior of OSCC, but there are conflicting results from other works. The aim of this study was to compare the immunohistochemical reactivity of MT in samples of metastasizing or nonmetastasizing primary OSCC (PM and PNM, respectively), as well as in respective metastatic lymph node carcinomas (N) in order to clarify the role of MT in the biological behavior of OSCC.

**Method:** Fifteen PNM, 15 PM OSCC, and 14 N were submitted to immunohistochemistry to detect MT. It was semiquantified according to cytoplasmic and nuclear reactivity in different areas of adjacent non-neoplastic epithelium, invasive carcinoma, and metastatic implants. Statistical tests were used to compare these findings with metastatic behavior.

**Results:** None criteria for MT reactivity was not associated with the metastatic behavior of primary OSCC. It was also not associated with the primary or metastatic nature of the samples.

**Conclusion:** The present results do not indicate a role for MT on the metastatic phenotype of OSCC, nor support the usefulness of MT immunohistochemical analysis as a predictive tool for metastasis in this disease. Financial support: CNPq, FAPEMIG, CAPES.

**PS-03-092**

**Association of intrauterine growth retardation and fetal teeth germs structural remodeling in rats**

S. Morozov ^*^, O. Reshetnikova

^*^State Medical University, Lugansk, Ukraine

**Objective:** It is widely accepted that fetal intrauterine growth retardation (IUGR) results in various diseases in later life. Preterm and low birth weight newborns are at risk of severe childhood caries. But the relationships between growth restrictions and structural parameters of newborn’s teeth germs are still unclear.

**Method:** Morphologic and morphometric peculiarities of the newborns teeth germs in 65 rats (including 35- with experimental systemic growth retardation and 30–controls) have been analyzed. Histological slides, stained with hematoxylin and eosin, were studied microscopically, then they were analyzed by point count method and using the computer morphometry of the enamel thickness.

**Results:** Results have shown the delayed growth and maturation of the teeth hard tissues. Volume fractions of have shown the delayed growth and maturation of the teeth hard tissues. Volume fractions of dental papilla, enamel organ and dentin with enamel were reduced compared with control parameters (respectively 8,33 ± 0,66; 4,77 ± 1,09; 3,46 ± 0,75 % compared with 10,00 ± 1,02; 8,98 ± 1,60; 6,76 ± 0,88 % in controls, *P* < 0, 05). The average enamel’s thickness also decreased to 2,98 ± 0,19 mkm (in controls-4,24 ± 0,77; *P* < 0,05).

**Conclusion:** These findings present the evidence of an association between IUGR and impaired growth and maturation of the newborns teeth germs. Immature hard tissues of teeth germs may predispose to early development of the tooth decay.

**PS-03-094**

**Extramedullary plasmacytoma in tongue: A rare entity**

E. Tastekin ^*^, N. Can, M. Azatçam, F. Oz Puyan, A. Arslan

^*^Trakya University, Pathology, Edirne, Turkey

**Objective:** Extramedullary plasmacytoma is a variant of multiple myeloma and relatively rare. Approximately 80 % of them occur in the upper aerodigestive tract.

**Method:** Case: A 56-year-old female patient has been follow-up for an ulcerated lesion in tongue since 2010. The successive four incisional biopsies were performed. All biopsies were diagnosed as ulceration, acute inflammation and hyperemia. Before the fifth biopsy physical examination was shown severe granular appearance nodular lesion revealing severe granular apperance on oral side of tongue. Incisional biopsy was performed. Microscopically epithelial ulceration and subepithelial diffuse plasma cell infiltration were detected. These plasma cells were positive stained with CD138, kappa and IgG antibodies immunohistochemically. There was no reaction with lambda, other Ig antibodies and LCA. We diagnosed this case as “Atypical plasma cell infiltration” and we suggested performing of serum protein electrophoresis. There was no spike in any type of plasma proteins. These histomorphological and laboratory findings suggested to the diagnosis of “extramedullary plasmositoma in tongue”.

**Conclusion:** Surgical pathologists should evalute ulcerated and diffuse inflammatory lesions very carefully and must avoid from the only descriptive diagnosis. Extramedullary plasmositoma is an important entity for differential diagnosis.

Sunday, 1 September 2013, 09.30–10.30, Pavilion 2

**PS-04 Poster Session Ophthalmic Pathology**

**PS-04-001**

**Androgen receptor in sebaceous gland carcinoma of the eyelid: Does it have a prognostic significance?**

K. Mulay ^*^, V. White, S. Shah, S. Honavar

^*^Center for Sight, Ophthalmic Pathology, Hyderabad, India

**Objective:** To evaluate the frequency of expression of Androgen receptor (AR) in sebaceous gland carcinoma of the eyelid and correlate its expression with histopathological features and clinical outcomes.

**Method:** 55 cases of eyelid sebaceous gland carcinoma with a minimum follow up of 1-year were retrieved from the archives. Patient files were assessed for clinical outcomes. Immunostaining for androgen receptor, Ki-67 was performed on all cases. All tumours were assigned an androgen receptor score (AR-Score) based on the intensity and percentage expression. The AR-score were correlated with the clinical outcomes, histopathological features and expression of Ki-67.

**Results:** All tumours (100 %) expressed AR. A high AR-score was significantly associated with lower disease free survival (*p* < 0.0001), high Ki-67 activity (*p* < 0.001) and early recurrence (*p* < 0.001). There was no statistically significant association between AR-score and histopathological features.

**Conclusion:** Our results confirmed the expression of androgen receptor (AR) in sebaceous gland carcinoma. It also suggested a prognostic influence of AR expression in sebaceous gland carcinoma of the eyelid.

**PS-04-003**

**Choroidal extramedullary hematopoiesis as a chance finding in abusive head trauma**

R. Verdijk ^*^

^*^Erasmus MC University, Dept. of Pathology, Rotterdam, The Netherlands

**Objective:** To investigate the frequency of extramedullary hematopoiesis in post mortem eyeball specimens sent for evaluation of abusive head trauma.

**Method:** Extensive unilateral extramedullary hematopoiesis of the choroid was observed in an otherwise clear cut case of abusive head trauma with bilateral retinal hemorrhage, optic nerve sheath hemorrhage and peripapillary intrascleral hemorrhage. Subsequently, 60 consecutive cases of suspected abusive head trauma examined since 2005 were evaluated for the presence of extramedullary hematopoiesis.

**Results:** Review of 60 archival cases of suspected abusive head trauma identified two more examples of extramedullary hematopoiesis in abusive head trauma that initially had been diagnosed as uveitis.

**Conclusion:** Extramedullary hematopoiesis of the choroid may be observed in 5 % of post mortem histopathologic examinations of eyes sent for evaluation of abusive head trauma. It may be a chance finding or can have a relation to premature birth, possible anemia or may be a reaction to intraocular bleeding due to earlier trauma. Choroidal extramedullary hematopoiesis may easily be confused for choroiditis and interpreted as a sign of infectious disease. Awarenes of the possibiliy of extramedullary hematopoiesis of the choroid should prevent misdiagnosis.

**PS-04-004**

**Raised numbers of IgG4-positive plasma cells are a common histopathological finding in orbital xanthogranulomatous disease**

R. Verdijk ^*^, P. Heidari, R. Verschooten, P. L. van Daele, H. J. Simonsz, D. Paridaens

^*^Erasmus MC University, Dept. of Pathology, Rotterdam, The Netherlands

**Objective:** To determine the relation of orbital xanthogranuloma to IgG4-related disease. Xanthogranuloma is a benign, tumor that rarely affects the orbit. The response to immunosuppressive therapy and relapse rate may be dependent on as yet unknown immunological reaction patterns that may involve IgG4 related disease.

**Method:** We searched our charts for orbital xanthogranuloma diagnosed over the last 25 years. Patient files were reviewed for clinical and follow up data. Histopathological classification was re-assessed. Sixteen cases of orbital xanthogranuloma were evaluated. Immunohistochemical stains for IgG and IgG4 were performed.

**Results:** Eight out of 16 cases (50 %) showed raised numbers of IgG4-positive plasma cells in the tissues, scored as >50 per high-power field, with IgG4/IgG ratio >0,40. This seemed to be associated with recurrent disease. The groups were too small to reach statistical significance, however. The morphological features of the cases with high IgG4-positive plasma cell counts conforming to the Boston Consensus Criteria lacked the criteria of storiform fibrosis and phlebitis. None of the patients developed systemic manifestations of IgG4 related disease.

**Conclusion:** Raised numbers of IgG4-positive plasma cells are a common finding in histopathological specimens of xanthogranulomatous disease of the orbit and are not indicative for IgG4 related systemic disease.

**Clinical, histological (H&E) and immunohistochemical images of IgG and IgG4 staining of the different xanthogranulomatous diseases.:** 
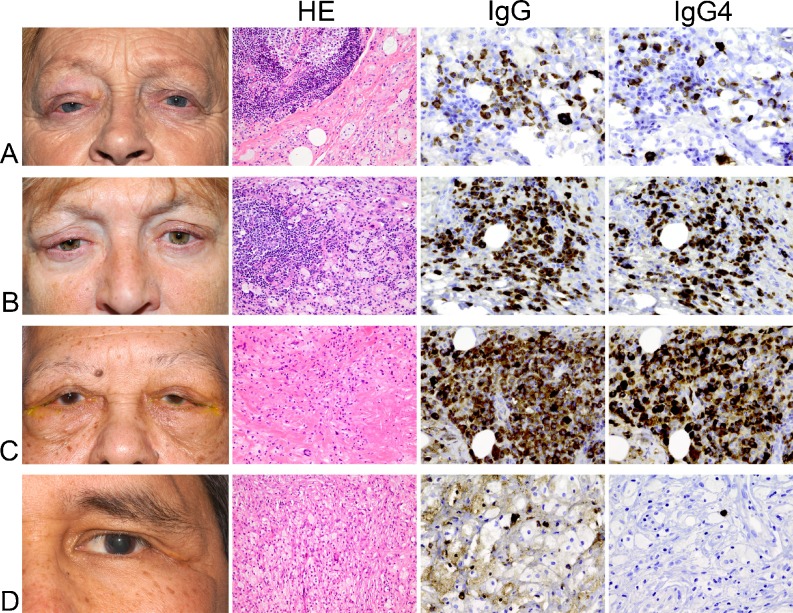


**PS-04-005**

**Atypical pleomorphic adenoma of the lacrimal gland: Report of a case**

M. Sofopoulos ^*^, N. Mylona, N. Koufopoulos, A. Vacarciuc, V. Petrou, N. Arnogiannaki

^*^Agios Savvas Hospital, Dept. of Pathology, Athens, Greece

**Objective:** We present a case of an Atypical Pleiomorphic Adenoma of the lacrimal gland.

**Method:** A 68 year old man with a small painless slow growing mass situated on his right lacrimal fossa. Clinical examination revealed a firm nodular, non pulsatile mass whose overlying conjuctiva was freely movable. Total excision was performed and a biopsy was sent to our laboratory.

**Results:** The specimen consisted of numerous irregular, ill-defined whitish pieces of tissue deprived of capsule. Histological examination revealed a neoplasm with intimate admixture of epithelial and mesenchymal elements. The latter is focally hyalinized and chondromyxoid. The epitelial component consists of cuboidal cells with enlarged nuclei, arranged in a trabecular fashion forming glands with an associated myoepitelial layer. These cells bear large, slightly atypical nuclei, with prominent nucleoli. Mitoses were rare and necrotic debris within the neoplasmatic lumen were found.

**Conclusion:** The diagnosis was atypical mixed tumor of the lacrimal gland. Atypical Pleiomorphic Adenoma is a rare premalignant condition which exhibits at least one of the following: capsule infiltration, hypercellularity, hyalinization, necrosis or cellular anaplasia. The treatment protocol recommends complete en-bloc excision of the tumor without a preliminary biopsy since incisional biopsy is related to an increased rate of recurrence due to incompletely excised mixed tumors.

**PS-04-006**

**Fine-needle aspiration biopsy as prognostic tool in uveal melanoma**

M. Mera ^*^, L. Blaga

^*^Univ. of Medicine and Pharmacy, Dept. of Pathology, Cluj-Napoca, Romania

**Objective:** Fine-needle aspiration biopsy (FNAB) becomes increasingly a valuable tool not only for diagnosis but for prognostication before treatment in an effort to avoid the much distressing enucleation. Our study is meant to ascertain whether sufficiently accurate prognosis information could be gleaned from fine needle aspiration biopsies as this is not used routinely yet in our area.

**Method:** We have included in our study 29 cases with FNAB prior to enucleation, including cytopathologic features as well as conventional histologic assessment.

**Results:** We have found that enough material for reliable cytodiagnosis by FNAB has been obtained in 27 (93 %) of cases. By means of appropriate statistical analysis we have found an excellent correlation between the presence of epithelioid melanoma cells observed on FNAB samples and the other histologic features obtained after enucleation. As further research we intend to add flow cytometry and molecular genetics workups to assess the prognostic reliability of a FNAB alone.

**Conclusion:** Fine-needle aspiration biopsy becomes increasingly compelling in order to assess a patient’s prognosis so that the most appropriate and the least distressing treatment can be applied. The ultimate goal is to improve the clinical practice in our area, where enucleation is still the procedure of choice.

**PS-04-007**

**Angiolymphoid hyperplasia with eosinophilia (epitheloid hemangioma) of the orbit**

A. Demirovic ^*^, I. Veliki-Dalic, R. Ivekovic, B. Kruslin, L. Pazanin

^*^University Hospital Centre, Dept. of Pathology, Zagreb, Croatia

**Objective:** Angiolymphoid hyperplasia with eosinophilia (ALHE) is the same condition as epitheloid hemangioma. It is an uncommon vascular tumor that occurs in the orbit extremely rarely. The main differential diagnosis is Kimura’s disease (KD). We present a case of an ALHE of the orbit and discuss main differences between ALHE and KD.

**Method:** Case presentation

**Results:** An 83-year-old patient presented with a subcutaneous mass of the left lower eyelid. Magnetic resonance imaging revealed a tumor in the left orbit and lower eyelid. An inferior orbitotomy was performed with tumor excision. Histopathological evaluation revealed a lobulated tumor measuring 2.5 × 2 × 0.8 cm, composed of proliferated blood vessels of varying caliber, lined by plump endothelial cells with large cytoplasmic vacuoles. The stroma contained numerous lymphatic follicles with prominent germinal centers and large proportion of eosinophils. The diagnosis of ALHE was made.

**Conclusion:** ALHE was first characterized by Wells and Whimster and the term epitheloid hemangioma was introduced by Enzinger and Weiss. ALHE shares some features with KD. However, they can be distinguished microscopically: ALHE has swollen, vacuolated endothelial cells; endothelial cells in KD are attenuated, without cytoplasmic vacuoles. Moreover, KD has systemic manifestations. ALHE is treated with surgical exision with approximately 33 % recurrence rate.

Sunday, 1 September 2013, 09.30–10.30, Pavilion 2

**PS-05 Poster Session Paediatric and Perinatal Pathology**

**PS-05-002**

**Morphological characteristics of thyroid gland of the fetuses from HIV-infected mothers**

I. Sorokina ^*^, S. Sherstyuk, T. Ospanova

^*^Nationa Medical University, Dept. of Pathologic Anatomy, Kharkiv, Ukraine

**Objective:** Thyroid diseases are now emerging in the first place among all endocrine diseases in children. Information on immunohistochemical features of thyroid gland in stillbirths from HIV-infected mothers, despite the steadily growing number of HIV-infected women of childbearing age, in the available literature has not been identified. The purpose of research is identification of immunohistochemical features of thyroid gland of the stillbirths from HIV-infected mothers

**Method:** In this research was used the thyroid glands of the fetuses from HIV-infected mothers. Immunohistochemical study was performed using monoclonal antibody to T3 and T4.

**Results:** Immunohistochemical study of the thyroid glands of the fetuses from HIV-infected mothers reveal the increased intensity of luminescence of the thyrocytes, compared to the control group, to T4 and T3 monoclonal antibody but light intensity of T4 was higher than T3. Apparently, the increase of the secretory activity of the thyroid gland of the stillbirths from HIV-infected mothers, compared to control group, is a manifestation of compensatory-adaptive mechanisms aimed to maintain homeostasis in the difficult conditions of maternal HIV infection.

**Conclusion:** In the thyroid gland of the stillbirths from HIV-positive mothers revealed a marked increase of secretory activity.

**PS-05-003**

**Autopsy findings in congenital somatic overgrowth consistant with Beckwith Wiedemann syndrome**

M. Kos ^*^, T. Lenicek

^*^KBC, Dept. of Pathology, Zagreb, Croatia

**Objective:** To make as accurate postmortem diagnosis as possible in a prematurely born neonate showing features of congenital somatic overgrowth.

**Method:** An autopsy was performed on a male neonate born at 32 weeks gestation after the lethal outcome in the 2nd day of life. The infant was mechanically ventilated, clinical informations were that he developed a right sided pneumothorax and respiratory distress syndrome. Except for acidosis, the laboratory findings also showed hypoglycemia, other results were unremarkable.

**Results:** The neonate weighted 3,030 g (normal 1,543+/−519 g), the lenght was 50 cm (normal 38.9+/−5.7 cm), at autopsy there was generalized visceromegaly. Microscopically, pancreas showed endocrine hyperplasia, both kidneys contained foci of nephroblastomatosis, both adrenals showed cytomegaly and cystic changes of the definitive cortex. An ectopic focus of adrenal tissue was found in the left lung. Other findings were consistant with prematurity; partial atelectasis, interstitial emphysema, hyaline membranes in the lungs and internal and external hematocephalus. The neonate also exhibited midfacial hypoplasia, macroglossia and ear lobe grooves.

**Conclusion:** There is a number of syndromes characterized with congenital somatic overgrowth. The findings in this case are most consistant with Beckwith-Wiedemann syndrome. The more sophisticated diagnostic methods (genetic analysis) were not available.

**PS-05-005**

**Case report: Congenital pulmonary lymphangiectasis in fetal autopsy**

G. Tasova Yilmaz ^*^, S. Toru, G. Ozbilim, C. Y. Sanhal

^*^Akdeniz University, School of Medicine, Dept. of Pathology, Antalya, Turkey

**Objective:** Congenital pulmonary lymphangiectasis (CPL) is a rare developmental disorder involving lung. CPL is a poorly documented disease, characterized by prominent lymhatic dilatation of septal-subpleural-peribronchial tissue of the lung.

**Method:** Here in we report a 17-week-gestation fetus with anhydramnios. In light microscopy there were marked dilatated channels in the subpleural -peribronchial-subseptal region of the lungs. The channels were lined with flattened cells which were expressing CD 31, negative for CD34. Although pulmonary interstitial emphysema (PIE) was considered an important differential diagnosis, a giant cell reaction surrounding the interstitial cystic lesions, a histologic hallmark of PIE. The morphological and immunohistochemical findings confirmed to a primary form of CPL, Noonan Group 3.

**Results:** CPL is characterized by dilatation of the pulmonary lymphatic vessels and occurs as a congenital anomaly. Noonan classified it into three goups. Primary developmental defect of pulmonary lymphatics is group 3. Group 3 is called also as congenital pulmonary lymphangiectasis, normal regression of the connective tissue elements fails to occur after the 16th week of fetal life, associated with an agressive clinical course, poor prognosis.

**Conclusion:** We report a rare developmental disorder diagnosed in fetal autopsy examination which was clinically and radiologically not detected.

**PS-05-006**

**Corticotropin releasing factor neuropeptides and binding sites in human fetal lung development and pathology**

M. Lambropoulou ^*^, E. Chouridou, M. Koureta, I. Balgouranidou, M. Simopoulou, N. Tsesmetzis, T.-E. Deftereou, V. Tsoulopoulos, N. Papadopoulos, E. Chatzaki

^*^Medical School, Democritus University of Thrace, Alexandroupolis, Greece

**Objective:** The CRF system (neuropeptides CRF, Ucn I, II, III and binding sites CRF1, CRF2, CRF-BP) is responsible for stress regulation and homeostasis of an organism. Here, we study CRF system expression in human normal and pathological fetal lungs.

**Method:** Lung tissues from 46 archival human fetuses were divided in Group A (normal), Group B (chromosomal abnormalities) and Group C (congenital disorders). Expression of the CRF system elements was evaluated using immunohistochemistry and was correlated to pathology, lung developmental stage and clinicopathological characteristics.

**Results:** Immunoreactivity for all antigens was found in both epithelial and mesenchymal lung cells of bronchi and alveoli. Ucn I and CRF1 were more frequently expressed in Group A. Ucns were more frequently expressed in the pseudoglandular stage. There was a positive correlation between the expression of the CRF neuropeptides and between CRF1 and CRF. Two fetuses with lung malformations showed no or low expression.

**Conclusion:** We demonstrated the expression of CRF system in human fetal lungs, being correlated to pathology and developmental stage. Our results comply with findings in experimental animal models, implicating the CRF system to fetal lung development, being more significant in the early stages.

**CRF1 expression in fetal lung X200:** 
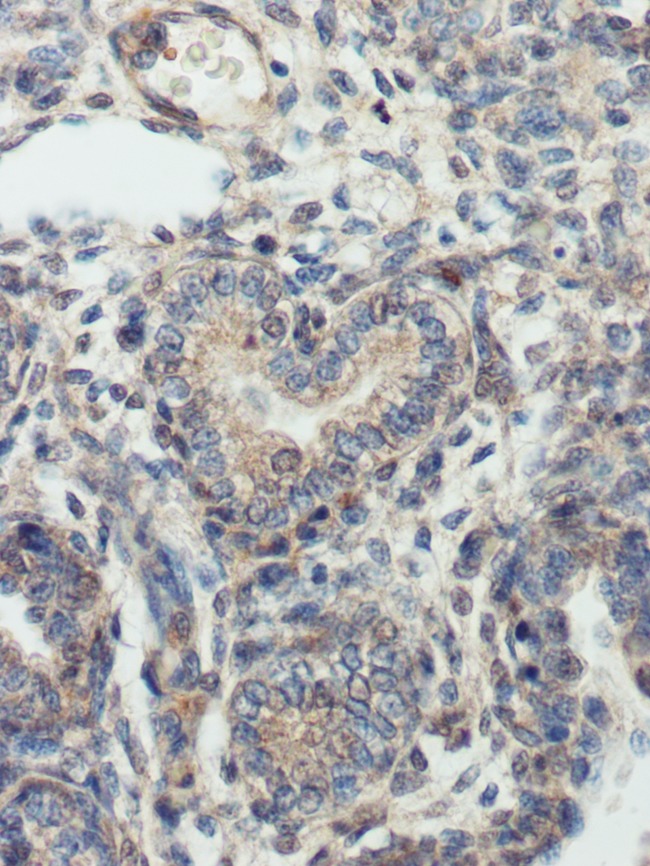


**PS-05-007**

**Cri du Chat syndrome with gingival fibromatosis: Histopathological analysis with confocal laser scanning microscopy**

G. Favia ^*^, S. Franco, S. Miccoli, V. De Falco, M. G. Lacaita, D. Di Venere, M. Falagario, E. Maiorano

^*^University of Bari, Dept. Odontostomatology and Surgery, Italy

**Objective:** Cri du Chat Syndrome (CCS) is a rare chromosomal disorder resulting from deletion of the short arm of chromosome 5, characterized by distinctive catlike cry. We report on still unreported clinic-pathological features CCS: diffuse Gingival Fibromatosis (GF) with Confocal Laser Scanning Microscopic (CLSM) examination.

**Method:** Case Presentation: A 21-year-old female, affected by CCS showed diffuse GF on the palatal gingiva in the molar region, which was surgically removed by Diode Laser, formalin-fixed, stained with hematoxylin-eosin and picrosirius red and analyzed atCLSM Nikon E-600 with double Laser inducing fluorescence (green and red).

**Results:** Microscopically, the lesion consisted in large parallel collagenous fibers, abundant blood vessels with plump endothelial cells and chronic inflammatory reaction, and large polygonal cells in the vascular interstitial spaces, with large nuclei, resembling undifferentiated totipotent mesenchymal cells (stem-like cells). At CLSM large and variably oriented collagenous fibers displayed intense fluorescence due to cross-linking between such fibers, which generally characterize fibromatosis, and the vascular structures. The latter showed lower fluorescence intensity, and were surrounded by loose collagenous fibers and trapezoidal large mesenchymal cells.

**Conclusion:** GF seems a characteristic feature of CCS, with typical fluorescent pattern at CLSM, in which purported totipotent mesenchymal cells may play a pathogenetic role.

**Confocal Laser Scanning Microscopy:** 
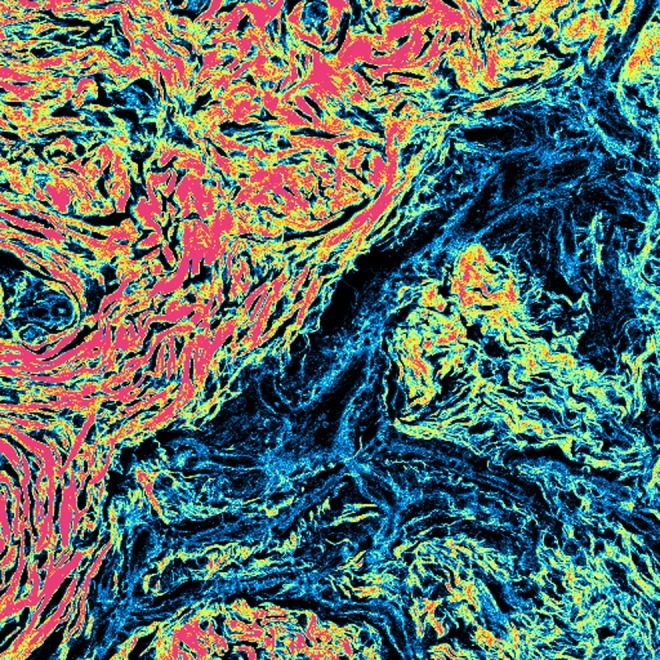


**PS-05-008**

**Tuberous sclerosis: Histological analysis with confocal laser scanning microscope of gingival angiofibromatosis**

M. L. Caruso ^*^, A. Tempesta, S. Miccoli, V. Lacarbonara, M. G. Lacaita, M. Corsalini, G. Favia, E. Maiorano

^*^IRCCS De Bellis Castellana, Pathology, Castellana Grotte Ba, Italy

**Objective:** We report on a case of Tuberous Sclerosis (TS) with gingival angiofibromatosis (GA), diagnosed by histopathological analysis with Confocal Laser Scanning Microscopy (CLSM) and treated with High-Power Diode Laser gingivectomy.

**Method:** Case presentation: The patient underwent gingivectomy and gingivoplasty with High-Power Diode Laser in pulsed modality and the surgical sample was formalin-fixed, paraffin-embedded and stained with hematoxylin-eosin and Pricrosirius red.

**Results:** Microscopically, thickened acanthotic epithelium with elongated rete ridges, densely packed, whorly collagen fibers, fibroblasts, variably sized vascular structures, and a few chronic inflammatory cells were detected. At CLSM examination, (Nikon Eclipse E-600 with green/red Laser inducing fluorescence) the collagen fibers, showing intense fluorescence, also manifested variable spatial orientation, due to cross-links among the bundles, ad typical of fibromatosis. Also, variably sized blood vessels and large and polygonal interstitial cells displayed fluorescence of lower intensity. The vascular component consisted of small groups of venous-like structures, frequently showing dilated lumina, thin walls and plump endothelial lining.

**Conclusion:** The histopathological analysis with CLSM of GA occurring in TS highlightes distinctive features, such as low fluorescence areas and a typical vascular component which may represent distinctive features of such lesion.

**Confocal Laser Scanning Microscopy:** 
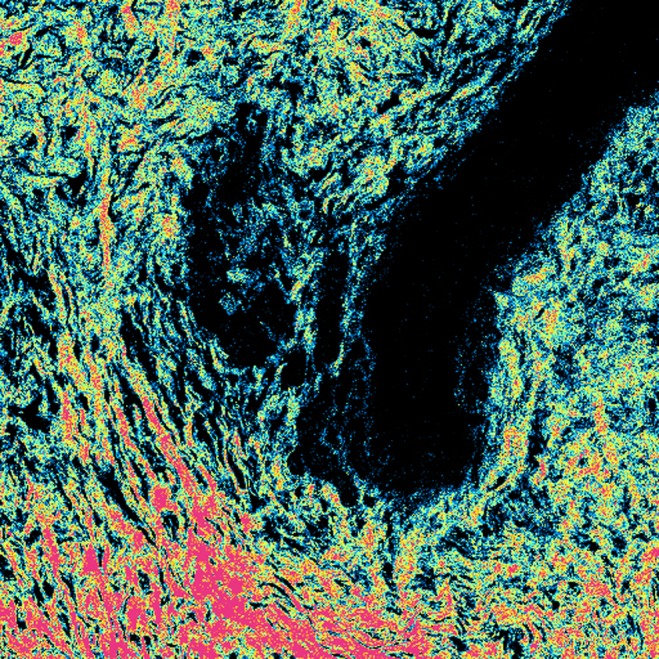


**PS-05-009**

**Correlation of computed tomography post-mortem imaging findings and autopsy findings regarding a case of congenital toxoplasmosis**

C. Tomikawa ^*^, R. Schultz, P. H. N. Saldiva

^*^FMUSP, Dept. of Pathology, Sao Paulo, Brazil

**Objective:** Post-mortem imaging is progressively used for autopsies, mainly in the forensic area, regarding this, we propose to correlate computed tomography (CT) post-mortem imaging findings and autopsy findings regarding a case of congenital toxoplasmosis.

**Method:** A female newborn with 33 weeks of pregnancy, admitted to emergency service with signs of intrauterine hypoxia,dying shortly after delivery, was submitted to the autopsy service. Whole-body CT and standardized autopsy examination were performed. An experienced clinical radiologist evaluated the imaging data without knowledge of autopsy findings.

**Results:** The CT and autopsy reports had few discrepancies. These were mainly regarding minor diagnosis not directly related to the cause of death. The autopsy report was, above all, more detailed with histological and immunohistochemical studies determining involvement extension and intensity on the organs.

**Conclusion:** Well established for forensic autopsies, the CT post-mortem imaging may be a complement to traditional autopsy also on the context of a newborn/stillborn–however, it is not yet capable of replacing it.

**Central nervous system calcifications on CT:** 
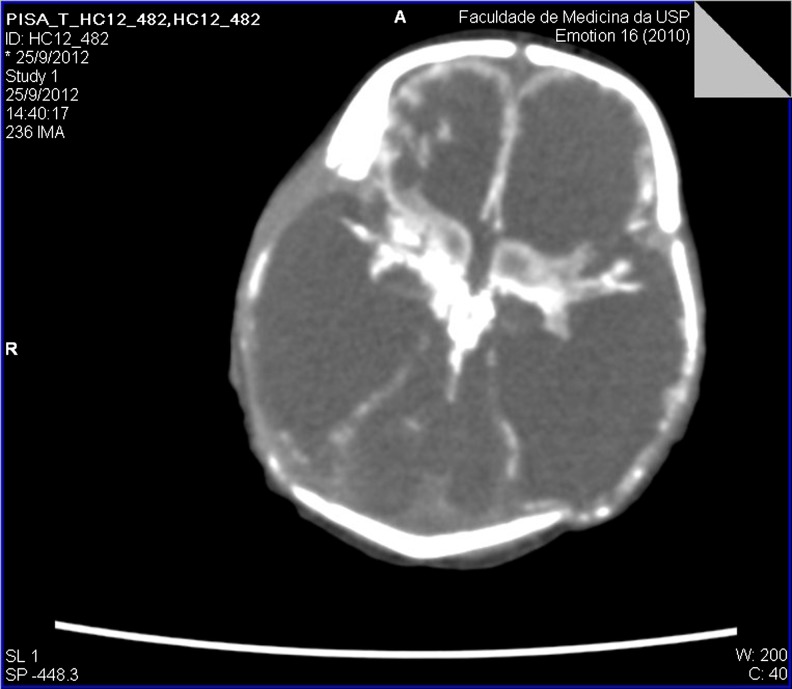


**PS-05-010**

**Prenatal diagnostic approach to fetal skeletal dysplasia**

H. S. Toru ^*^, G. Tasova Yilmaz, I. H. Ozbudak, B. Nur, C. Y. Sanhal, K. Karaali, O. Alper, I. Mendilcioglu, E. Mihci, S. Karaveli

^*^Akdeniz University, Pathology, Antalya, Turkey

**Objective:** Skeletal dysplasias (SDs) constitute a group of heterogeneous disorders affecting growth-morphology of the chondro-osseous tissues. Prenatal diagnosis of SD is a considerable clinical challenge due to phenotypic variability.

**Method:** The archive of fetal autopsies was reviewed between January 2006 and December 2012. Fifty-four cases were detected which were diagnosed by ultrasonography. Among them 17 cases underwent amniocentesis, one case underwent chorionic villus sampling and three cases underwent molecular analyses.

**Results:** One of the cases had a diagnosis of Trisomy 18, terminated at gestational age of 14thweek. Three cases had specific gene mutations as following: p. Arg248Cys, and p. Gly370Cys missense mutations at FGFR3 and homozygous c.1878delA/c.1878delA mutation at Dymeclin gene. In FGFR3 mutated cases, ultrasonographic findings were detected at gestational ages of 20th and 25th weeks. Case with dymeclin gene mutation had a sibling with Dyggve-Melchior-Clausen syndrome which was terminated at gestational age of 16thweek. Thirty-one of cases were associated with at least one congenital anomaly.

**Conclusion:** This study showed that SD is usually detected clinically after 20th gestational week. This gestational age could be considered as late to plan termination of pregnancy because of the psychological and clinical complications. Performing genetic analyses for SD can provide early diagnosis and management.

**PS-05-011**

**Twin reverse arterial perfusion syndrome: A case report**

S. Toprak ^*^, E. Atik, A. G. Okyay, O. Ipci, T. Ozgur, H. Gokce

^*^Mustafa Kemal University, School of Medicine, Dept. of Pathology, Hatay, Turkey

**Objective:** Twin Reverse Arterial Perfusion (TRAP) Syndrome is rarely seen in %1 of monochorionic twins. We aimed to present a rarely seen case.

**Method:** 28 year old woman at 28th gestation week with a history of two aborts and healthy triplet babies referred to clinic. Doppler USG revealed monochorionic plasenta and reversal flow in umbilical artery. Amorphous twin and plasenta send for autopsy.

**Results:** Macroscopically there were gastroschisis and two arteries,one vein and colon in edematous umbilical cord. Plasenta was monochorionic. At recipient twin deformed feet with oligodactyl, partially developed lumbar and thoracic spine was observed while the cervikal spine, head, heart, upper limbs and external genitalia was not observed. Abdominal organs were not well developed. TRAP syndrome was diagnosed.

**Conclusion:** TRAP syndrome is one of the rarest complications of monochorionic twins. Structurally normal, the pumping twin, perfuses acardiac twin. Reverse flow in the umbilical artery selectively to iliac vessels of the recipient twin is the key pathology. As the oxygenation of lower segment of the body is well, lower limbs grow better than upper segments. This leads to anomalies extending to amorphogenesis of upper segments of body. This case highligts the importance of prenatal diagnosis and planning treatment.

**PS-05-012**

**Neural tube defects and associated anomalies in fetal and perinatal autopsy series**

H. S. Toru ^*^, C. Uzun, G. A. Gokhan Ocak, C. Y. Sanhal, I. Mendilcioglu, S. Karaveli

^*^Akdeniz University, Pathology, Antalya, Turkey

**Objective:** Neural tube defects (NTDs) are heterogeneous and complex group of congenital anomalies. NTDs are major congenital malformations affecting the brain and spinal cord. NTDs often result in fetal death, death early in life, or in developmental disabilities among surviving infants and children.

**Method:** The archive of fetal autopsies was reviewed between January 2006 and June 2012. Sixty-two of them had NTD. For all fetuses we carried out macroscopic assessment, photographic records, gestational age, maternal age, clinical findings.

**Results:** Twenty-two of 62 cases with NTDs are associated with at least one anomaly. Thirty-two cases were chranioschisis, 28 cases were spina bifida and 2 cases were craniospinal rachischisis subtype. The most common anomalies associated with NTD is renal-urinary system anomalies(16.1 %), followed by skeletal (9.7 %), pulmonary (4.8 %)and cardiac anomalies(4.8 %).

**Conclusion:** The overall prevalence of associated malformations, observed in 35.5 %, emphasizes the need for a thorough investigation of infants with NTDs. Routine screening for other malformations, especially renal-urinary system, skeletal and pulmonary anomalies and cardiac defects may need to be considered in infants and fetus with NTDs, and referral for genetics evaluation and counseling seems warranted in most of these complicated cases. Prenatal early diagnose of the NTD may reduce the psychological and clinical complications of termination.

**PS-05-013**

**Fetal mediastinal teratoma- a rare cause of nonimmune hydrops fetalis. Report of one case**

A. Rodrigues ^*^, L. Monteiro, S. Ramos

^*^Hospital Egas Moniz, Pathology, Lisbon, Portugal

**Objective:** Fetal mediastinal teratomas are rare congenital germ cell tumors that can compress mediastinal structures and cause nonimmune hydrops.

**Method:** The authors present a case of a 36 years old woman, gravida 1, para 0111. Ultrasound evaluation at 21 weeks of gestation revealed fetal hydrops and suggested a diaphragmatic hernia with severe bilateral lung hypoplasia. An amniocentesis was performed and showed normal chromosomes, 46, XY. At 22 weeks of gestation a medical interruption of pregnancy was performed.

**Results:** The post-mortem examination revealed a massive tumor of the anterosuperior mediastinum with cardiac and bilateral lung compression and fetal hydrops. The tumor was solid, encapsulated, 34,59 g of weight and 5 × 4,5 × 3 cm. Histological examination revealed a solid teratoma composed of mature tissues admixed with immature neuroglial elements.

**Conclusion:** This rare condition, that could have an adverse outcome, should be included in the diagnostic evaluation of any case of second trimester nonimmune hydrops, associated with severe lung hypoplasia.

**PS-05-014**

**Heterotaxy syndromes: A case report of Ivemark syndrome**

C. Callé ^*^, A. Rodrigues, A. Teixeira, L. Caceres Monteiro

^*^IPO Lisboa, Anatomia Patológica, Portugal

**Objective:** The heterotaxy syndromes are rare diseases with an incidence of 1–1.5/10,000 live births. These complex entities are associated with numerous malformations, in particular, complex cardiac and extra-cardiac malformations that have an important impact on the prenatal and postnatal course. Right isomerism has a high postnatal mortality due to the more complex type of cardiac defects.

**Method:** We report a case of medical interruption of pregnancy at 25 week of gestation, due to severe cardiac abnormalities incompatibles with life. The echocardiography revealed a fetus with total anomalous pulmonary venous drainage, atrioventricular septal defect, and right side truncus arteriosus of aortic type. No other malformations were detected in morphologic ecography.

**Results:** The autopsy confirmed the complex heart defects and revealed bilateral trilobed lungs and asplenia, settling the diagnosis of Ivemark Syndrome.

**Conclusion:** The Ivemark complex (OMIM 208530) is a malformation with bilateral right-sidedness, with a Mendelian autosomal recessive inheritance, included in the group of Heterotaxy syndromes. The exact diagnosis of this entity is mandatory for adequate genetic counselling of the parents and for planning the postnatal care in a new pregnancy.

**PS-05-015**

**Contribution of fetal autopsy for diagnosis of Beckwith-Widemann Syndrome (BWS)**

M. E. Jo Velasco ^*^, I. Guerra, G. Perez de Nanclares, P. Morales, I. Garin, J. J. Aguirre, Z. S. Quintero, C. Gomez

^*^Hospital Txagorritxu, Dept. of Pathology, Vitoria-Gasteiz, Spain

**Objective:** Omphalocele is recognized as congenital malformation with a high mortality. It is associated with other congenital malformations.29 % has abnormal karyotype. The aim of the present study is to show the importance of perform the foetal autopsy in the legal interruption of the pregnancy with fetal diagnosis for scan of omphalocele.

**Method:** Fetal autopsy,histopathologic examination and molecular analysis were performed in a male fetus:46 XY karyotype,therapeutically aborted at 21 weeks of gestation with scan of omphalocele.

**Results:** Gross examination showed a male fetus with omphalocele and his weight corresponded to 24 weeks of gestation. Histologic showed adrenocortical cytomegaly which suggested the presence of BWS.We revised again grossly the fetus and we found that BWS was confirmed by the existence of his 3 criteria;macroglossia, omphalocele,macrosomy. Accompanied by minor signs: hemihypertrophyfacial,ear-creases, hepatomegaly. Molecular analysis revealed loss of methylation at IC2 (KvDMR); the most common genetic alteration associated with BWS.

**Conclusion:** BWS is an overgrowth disorder with variability in clinical manifestations and molecular causes. Their manifestations are highly variable with some cases lacking one or more of the hallmark features. In our case the existence of the 3 criteria help us in our diagnosis. The pathognomonic histologic feature in BWS is the adrenocortical cytomegaly. We present one BWS case diagnosis by a meticulous autopsy and our molecular diagnosis is consistent with that described thus far. We want to highlight the importance of the fetal autopsy and its histological study.

**PS-05-016**

**Microscopic features of kidneys in newborns born to mothers with preeclampsia**

I. Sorokina ^*^, V. Markovsky, M. Myroshnychenko, T. Ospanova, I. Korneyko

^*^Nationa Medical University, Dept. of Pathologic Anatomy, Kharkiv, Ukraine

**Objective:** The purpose of the study is to identify the microscopic features of kidneys in newborns born to mothers with complicated pregnancy by mild preeclampsia.

**Method:** The material for this study was the tissue of kidney, stained with hematoxylin and eosin according to van Gieson. All material was divided into two groups: 1 group–controls (3 cases) comprising the newborns from mothers with physiological pregnancy, 2 group-study group (7 cases) comprising newborns born to mothers whose pregnancy was complicated by mild preeclampsia.

**Results:** In kidneys of newborns of the second group embryonic glomeruli predominated; small areas of focal dysplasia and some glomeruli with the cyst formation were present; sclerotic processes in the glomeruli and interstitial tissue increased; the features of blood circulation disturbance characterized by hyperemia of peritubular and glomerular capillaries were noted. Mainly in the newborns of group 2, small foci of marked sclerosis with solitary lymphoid cell infiltrates were determined in cortical area of kidneys. Renal tubular epithelium of children from group 2 was characterized by focal degenerative changes.

**Conclusion:** Structural changes, identified in the kidneys of newborns born to mothers with complicated pregnancy may create the conditions for development of nephropathology in these children in future ontogenesis.

**PS-05-017**

**Placental pathology in cases of pregnancy, complicated with uterine leiomyoma**

O. Reshetnikova ^*^, O. Burgelo

^*^State Medical University, Lugansk, Ukraine

**Objective:** It is now known that uterine leiomyoma (UL) has a negative effect on women’s reproductive health contributing to a variety of gestational complications. It increases risk of perinatal lethality of a child. Pregnancy with this tumor is frequently accompanied by clinical and morphological manifestations of placental insufficiency. The aim of this study was to examine peculiarities of placental and fetal growth in case of gestation, complicated with UL.

**Method:** Thirty five placentas from pregnancies, complicated with UL and ten placentas of women with physiological pregnancy (controls) were studied by organometry, histology and computer morphometry.

**Results:** The results have shown that the weight of the placenta, maternal surface square and fetal- placental ratio in the main group of observations exceeded the results in control (*p* < 0.05). Placental volume for women with UL did not reach the control value (*p* < 0.05). The fetal weight did not have statistically significant differences. Placental pathology correlated with the place of placentation and neighborship to the leiomyoma.

**Conclusion:** Further study needed to evaluate the structural remodeling of the placental membrane depending of the tumor sites in the uterus and the area of placentation.

**PS-05-018**

**Villous trophoblastic expression of transcription factor FOXO1 in preeclampsia**

J. Stanek ^*^, R. Sheridan, C. Belludi, J. Khoury, S. Handwerger

^*^Cincinnati Children’s Hospital, Dept. of Pathology, USA

**Objective:** There is little information about FOXO1 (forkhead box protein O1) expression in pathologic placentas.

**Method:** Paracentral sections from grossly unremarkable areas of 10 placentas each from patients with mild preeclampsia (PE), severe PE and gestational age-matched controls were double immunostained for FOXO1 and E-cadherin, the latter distinguishing villous cytotrophoblasts (CTB) from syncytiotrophoblasts (STB). Cells counts were performed blindly on same ten 40× objective fields by three observers. Log transformed counts were analyzed using a generalized linear model.

**Results:** In mild PE, counts of FOXO1 positive STB were statistically significantly decreased and negative STB were significantly increased compared to controls. CTB positive cells were not statistically significantly different, while CTB negative cells were significantly increased. In contrast, FOXO1 positive and negative STB and CTB counts in severe PE were not statistically significantly different from controls (Figure).

**Conclusion:** Abnormal FOXO1 expression was observed in STB and CTB in mild but not severe PE placentas. Since FOXO1 is critical for placental cellular morphogenesis, abnormal FOXO1 expression may in part contribute to the abnormal trophoblast differentiation in mild PE. The differences in FOXO1 expression in mild and severe PE are consistent with other studies suggesting that the two forms of PE are different disease processes.

**FOXO1 expression in villous trophoblasts:** 
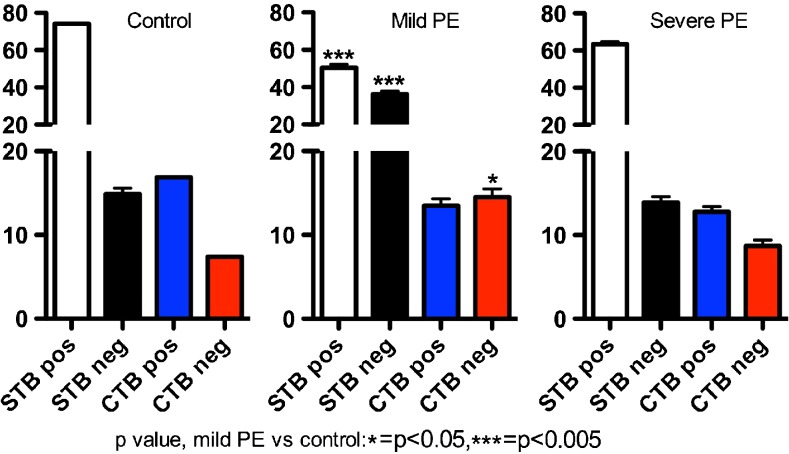


**PS-05-019**

**Increased apoptotic activity on human preeclamptic placentas: An immunohistochemical study**

M. Lambropoulou ^*^, N. Tsesmetzis, E. Chatzaki, P. Ypsilantis, O. Pagonopoulou, E. Kontomanolis, V. Tsoulopoulos, E. Chouridou, M. Koureta, E. Papadopoulos, N. Papadopoulos

^*^Medical School, Democritus University of Thrace, Alexandroupolis, Greece

**Objective:** The aim of this study was to assess the apoptotic activity in placentas of preeclamptic pregnancies.

**Method:** Paraffin-embedded placental tissue specimens collected from preeclamptic (group A, *n* = 174) and normal pregnancies (group B, *n* = 30) were examined by conventional histology and by immunohistochemistry using monoclonal antibodies against caspase 8, caspase 9, M30, and the DeadEnd Colorimetric TUNEL system.

**Results:** In preeclamptic placentas, the expression of all apoptotic markers was significantly higher compared to those from normal pregnancies (*p* < 0.05). Specifically, expression levels of Caspase 8, 9 and M30 in group A were significantly higher than in group B. TUNEL staining also confirmed that placental tissues from group A had significantly increased levels of apoptotic cells.

**Conclusion:** Overexpression of apoptotic markers was observed in placentas complicated with preeclampsia in comparison with normal ones, indicating that preeclampsia leads to increase of programmed cell death in placental tissue.

**Preeclamptic placenta, M30X200.:** 
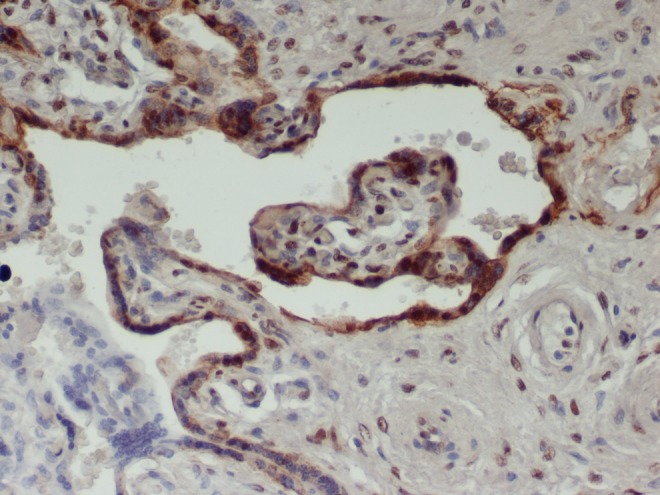


**PS-05-020**

**Umblical cord haemangioma: A case report**

T. Ozgur^*^, A. G. Okyay, H. Gokce, S. Toprak, E. Atik

^*^Mustafa Kemal University, School of Medicine, Hatay, Turkey

**Objective:** Umblical cord tumors are rare occurences with various pathologic features and prenatal differentiation is often very difficult. Haemangiomas are the most common of these lesions defined in the literature.

**Method:** 22 year-old woman gravida 1, para 0 was admitted at a gestational age of 38 weeks with urgent pelvic pain to Gynecology and Obstetrics outpatient clinic. Ultrasonography revealed a mass associated with umblical cord and there has been no other anomalities in fetus. The patient underwent cesarean delivery with high fetal weight and undefined umblical cord mass and gave birth to a female neonate with Apgar 9/9. The placenta has been sent to pathology laboratory.

**Results:** In macroscobic evaluation the umblical cord was long and contained a solid mass with 6 × 6 × 5 cm diameter and gray-brown cut sections. Sections of the mass had branching blood vessels with variable degrees of endothelial proliferation surrounded by loose myxomatous connective tissue. CD31 stain confirmed the vascular origin and the diagnosis was haemangioma.

**Conclusion:** Umblical cord haemangiomas should be remembered when an echogenic mass is visualized within the umblical cord which is located near the placental insertion site. Color doppler ultrasonography and serum AFP levels are helpful to distinguish from other lesions.

**PS-05-021**

**Placental insufficiency in placentas with viral infection**

A. Kolobov ^*^, E. Musatova, D. Niauri, V. Karev, V. Zinserling

^*^St. Petersburg, Russia

**Objective:** Infectious pathology plays important role in etiology of placenta insufficiency. HIV and herpes viruses may either cause primary or latent infection during pregnancy.

**Method:** The 63 placentas were collected from three groups of patients: Group 1–from women with herpes-virus infections (31 placentas–13 with HSV-1, 15 with CMV, and 3 with combined HSV-1 + CMV); Group 2–from HIV-infected women (12 placentas); Group 3–20 placentas from women without any infection and placental insufficiency. Expression of receptors was determined by monoclonal antibodies against CD68 (КР1 clone, Dako), CD31 (Novocastra), bFGF (Santa Cruz Biotechnology), TGF-β1 (Novocastra) HSV (I and II) and CMV (Diagnostic BioSystems), HIV-infection was confirmed antibodies against р24 (Dako).

**Results:** In all cases with viral infection was diagnosed placenta insufficiency. Expression of CD68 was the highest in Group 1 (13.07 ± 0.83 %), followed by group 2 (0.91 ± 0,02 %) when compared with Group 3 (2.02 ± 0.60 %, *р* < 0.05 for both comparisons). In placentas with HIV was noted increased expression of bFGF (5.49 ± 1.48 %) and TGF-β1 (11.2 ± 3.6 %). In placentas from Group 1 expression of bFGF was decreased–1.21 ± 0.5 %.

**Conclusion:** Thus, the mechanism of insufficiency in placentas with viral infection includes damage of vasculosyncytial membrane.

**PS-05-023**

**Placental metabolic markers expression in assisted reproductive technologies-achieved pregnancies**

A. Shchegolev ^*^, E. Dubova, K. Pavlov, N. Aleksandrova, O. Baev, G. Sukhikh

^*^Research Center for Obstetrics, Dept. of Pathology, Moscow, Russia

**Objective:** The placenta evolved to support development of the fetus, and so potentially plays a key role in the etiology of developmental programming through its impact on nutrient transfer.

**Method:** Our ami was to evaluate placental metabolism (transmembrane–SNAP 23, annexin 3; energy–Light Ferritin Chain–LFC, ATP-5J; carbohydrate–GLUT 1, GLUT 3) in women conceived after assisted reproductive technologies (ART). Immunohistochemical study with rabbit polyclonal antibodies SNAP 23, annexin 3, ATP-5J, GLUT1 and GLUT3; goat polyclonal antibodies LFC.

**Results:** Higher rate of obstetrical complications (OR = 1.1–2.33, *p* < 0.05 such as preeclampsia, GD, pregnancy loss, preterm delivery etc.) after ART (in comparison with spontaneous conception) is associated with positive somatic (OR = 1.4–3.22, *p* < 0.05), obstetric and gynecological history (OR = 1.14–4.0, *p* < 0.05) and also multiple pregnancy. In placentas of women conceived by ART the significant reduction of annexin3, ATP5J (markers of transmembrane and energy exchange) is revealed. Expression of GLUT3 is significantly lower in placentas after ART.

**Conclusion:** Finding data could be determined more by higher rate of previous extragenital and gynecological pathology than ART procedure itself. Future study is needed to understand the roles of the differentially expressed proteins in ART placentas.

**PS-05-025**

**The significance of acetylcholinesterase stain in diagnosis of Hirschsprung disease**

T. Koletsa ^*^, P. Pantelidou, G. Karayannopoulou, G. Karkavelas, I. Kostopoulos

^*^Medical School AUTH, Dept. of Pathology, Thessaloniki, Greece

**Objective:** The histologic diagnosis of Hirschsprung disease (HD) is challenging. A relatively reliable diagnostic method is the histochemical stain for acetylcholinesterase (AChE). The accuracy of the method is investigated in infants and neonates.

**Method:** 104 cases with clinical suspicion of HD were retrieved from the archives of our Department. In all cases, stain for AChE was performed in frozen sections of colon biopsies. Most cases were represented by at least two biopsies taken from different colon sites. There were also repeated biopsies in 18 cases. The histological findings were analyzed.

**Results:** Only eight patients were over 1 year of age. The male:female ratio was 2:1. Out of 104 cases, 20 cases (19.23 %) were diagnosed as HD. In all 20 cases marked staining of AChE-positive nerves in the lamina propria was observed. Moreover, 68 (65.38 %) cases were negative, whereas in 12 cases (11.54 %) the histological findings raised the suspicion of neuronal intestinal dysplasia. Four cases (3,85 %)were diagnosed as ultrashort HD. There was no false positive HD diagnosis.

**Conclusion:** AChE stain is considered to be an accurate method for diagnosing HD, restricting or eliminating false positive cases. The increase of AChE-positive nerves in the colonic mucosa confirms the diagnosis of HD.

**PS-05-026**

**Disseminated congenital melanoma associated with defective function of CD8 T-cells and NK cells**

E. Gradhand ^*^, R. Lucy, L. E. Newell, K. Neumann, P. Ramani

^*^University Hospitals Bristol, Dept. of Histology, United Kingdom

**Objective:** There are only 26 reported cases of congenital malignant melanomas (CMM) in the English scientific literature since 1925. The aim of this study was to characterise a CMM associated with an immunodeficiency by its histology, immunohistochemistry and molecular biology.

**Method:** A newborn boy presented with a large periorbital mass. He also had a generalised papular rash. We received skin punch biopsies to exclude molluscum contagiosum and a bone marrow trephine. In addition to routine histology and immunohistochemistry, we performed molecular genetic testing on the blocks of the primary lesion for Braf, cKit, Nras, Kras and Egfr mutations.

**Results:** The routine stains of the bone marrow and skin biopsy showed similar infiltrates of atypical cells of a CMM which stained positive for melanoma markers. The molecular testing revealed no mutations in any of the investigated genes. Immunological investigations demonstrated defective function of CD8 T-cells and NK cells.

**Conclusion:** Due to the disseminated metastases, we performed a molecular analysis to look for any treatment options and a possible unique mutation in CMMs. Unfortunately, the results were negative, but surprisingly, the child is now 2 years old showing a normal development and relatively stable health but with disseminated metastases of a CMM.

**PS-05-028**

**Brain pathology in three children with Leigh syndrome and different mutations found**

K. Jandova ^*^, H. Skalova, T. Honzik, M. Tesarova, J. Duskova

^*^General University Hospital, Institute of Pathology, Prague, Czech Republic

**Objective:** Leigh syndrome is an early onset, often fatal progressive neurodegenerative disorder caused by mutations in the mitochondrial or nuclear DNA. Until now, mutations in more than 35 genes causing Leigh syndrome have been reported, indicating an extreme genetic heterogeneity for this disorder.

**Method:** CNS lesions in three children with various mitochondrial disorders who died at the age of 8 months, 18 months and 6 years are described.

**Results:** In all three children defect of cytochrome C oxidase was proven. Subacute necrotizing encephalomyelopathy at different stages corresponding with the age of the patients was observed. Mutations in SURF1 gene in 6 years old girl, in AIMF1 gene in 18 months old boy and in COX10 gene in 8 months old girl were detected.

**Conclusion:** Irrespective of the different mutations found the morphology damage of the brain remains relatively monotonous reflecting rather the survival interval.

**PS-05-029**

**Cell-death factors expression in neuroblastoma**

L. Brcic ^*^, M. Bogovic, M. Misic, S. Seiwerth

^*^Univ. of Zagreb School of Med., Institute of Pathology, Croatia

**Objective:** Neuroblastoma is among the most common neoplasms in children usually diagnosed in higher clinical stages with uncertain outcome. Search for reliable prognostic and predictive factors is still underway.

**Method:** Apoptosis plays important role in tumor suppression/prevention. Fas and FasL are membrane proteins involved in one of several different ways to activate the cascade of reactions resulting in apoptosis. CTL (cytotoxic T lymphocytes) via the Fas/FasL system eliminate tumor cells, and vice versa, tumor cells can use this system to induce apoptosis of CTL. Another cytolytic activity of cells is mediated by perforin, released from the killer cells, creating pores in the membrane of target cells causing necrosis or apoptosis and independently through Hsp-70.

**Results:** We tested expression of Fas, FasL, perforin and Hsp-70 by immunohistochemistry in 50 tumor samples of children diagnosed between 1994 and 2013 at the University Hospital Center Zagreb and their correlation with molecular, histological and biological characteristics (favorable and unfavorable histology, MKI, N-myc status, INSS and INRGSS stage, risk groups based on POG criteria, metastases localization).

**PS-05-030**

**Metanephric stromal tumor: A pediatric renal neoplasm**

F. Khanchel-Lakhoua ^*^, R. Dhouib, L. Charfi, R. Doghri, I. Abbes, S. Sassi, M. Karima, M. Driss, D. Kacem, K. Ben Romdhane

^*^Salah Azaiez Institute, Pathology, Tunis, Tunisia

**Objective:** Metanephric stromal tumor (MST) of kidney is a pediatric benign stromal specific renal neoplasm. A few cases have been reported. In this communication we describe the gross and microscopic features of MST with emphasis on diagnostic criteria.

**Results:** We report a case of right renal tumor in a 3-year-old boy which was radiologically suspected of being a Wilms tumor. The patient had a neoadjuvant chemotherapy and a right radical nephrectomy was performed. Microscopically, on low power examination, alternating areas of high and low tumor cellularity were noted. Entrapped tubules and glomeruli were commonly seen within the lesion. The tumor cells were spindle to stellate in shape and arranged in a nodular pattern. Characteristically, there were onion skin-like concentric cuffs of tumor cells around entrapped tubules. The small intratumoral vasculatures showed irregular thickening (“angiodysplasia”).

**Conclusion:** MST is usually centered in the renal medulla. It has characteristic microscopic appearance which differentiates this lesion from congenital mesoblastic nephroma and clear cell sarcoma of the kidney. In most cases complete excision alone is curative. Recognition of this entity can spare a child potentially toxic adjuvant chemotherapy that might be used for lesions in its differential diagnosis.

**PS-05-031**

**Four cases with congenital pulmonary airway malformation**

H. S. Toru ^*^, G. Tasova Yilmaz, I. H. Ozbudak, I. Mendilcioglu, A. Aslan, G. Ozbilim

^*^Akdeniz University, Pathology, Antalya, Turkey

**Objective:** Congenital pulmonary airway malformations (CPAM) are a rare hamartomatous lesion of the lung, typically diagnosed in the antenatal period. CPAM is separated in to five major types based on clinical and pathological features.

**Method:** Here in we report four cases with CPAM. Three of them were postnatal lung segmentectomy material and one of them was fetal autopsy specimen. All of them were classified into histological subtypes according to five layered classification system of Stocker.

**Results:** Among four cases three of them were CPAM type II, one of them was CPAM type I. All of the cases were detected antenatal in ultrasonographic examination after gestational age of 22nd week. Only one family wanted termination, in the autopsy evaluation of this case CPAM type II is detected on the upper left lobe, compressing distal parts of the lung, lateralizing the heart to the right. Other three cases underwent surgical resection. Two of them were CPAM type II, one of them was CPAM type I.

**Conclusion:** Ultrasonography has been demonstrated to be highly useful modality in the in utero diagnosis of CPAM. Recognizing the imaging features of these abnormalities is necessary for prenatal counseling and appropriate prenatal and postnatal management.

**PS-05-032**

**Congenital salivary gland anlage tumor of nasopharynx in a neonate**

L. Mascarenhas Lemos ^*^, M. Mafra

^*^Lisbon Central Hospital, Dept. of Pathology, Lisboa, Portugal

**Objective:** Nasal and upper respiratory tract obstruction in the neonatal period is a potenctially life-threatening condition that can result from inflammatory conditions, hamartomas, congenital malformations and tumors. Salivary gland anlage tumor, also referred to as congenital pleomorphic adenoma, is a rare, benign congenital tumor of the nasopharynx, which may produce nasal obstruction.

**Method:** Case report with literature review.

**Results:** A male neonate presented with complaints of nasal obstruction and feeding difficulties. During the common clinical diagnostic approach was found a friable, pediculated mass with origin on the nasopharynx and protusion to the oropharynx. Radiographic evaluation demonstrated a circumscribed pediculated mass with 8 mm that did not communicate with the intracranial compartment. Pathology revealed a hamartomatous lesion with a biphasic nature demonstrated by the admixture of epithelial groups of duct-like and squamous structures and bland stromal cells.

**Conclusion:** Congenital salivary gland anlage tumor of nasopharynx is usually not suggested as a possible cause in the setting of nasal obstruction from a mass in the neonatal period because it is uncommon. The different clinical significance of other developmental masses and neoplastic lesions requires having congenital salivary gland anlage tumor considered on the differential diagnosis.

**PS-05-033**

**Analysis of the prevalence of Wilms’ tumor in Bulgarian children diagnosed with malignant tumors for a period of 10 years (2001–2010)**

D. Serteva ^*^

^*^N.I. Pirogov University Hospital, General and Clinical Pathology, Sofia, Bulgaria

**Objective:** The aim of this study is to show the prevalence of Wilms’ tumor (WT) in Bulgarian children, operated at University General Hospital for Active Medical Treatment and Emergency Medicine “N.I. Pirogov”, Sofia, Bulgaria, for a period of 10 years, compared to the total number of malignant tumors in children, diagnosed at the same hospital and the distribution of WT according to gender and age.

**Method:** Conventional histological staining–hematoxylin and eosin, immunohistochemical staining (WT antigen), archive of the department of pathology.

**Results:** A total number of 396 cases of malignant tumors in children were diagnosed and operated at the hospital for the 10-year-period, 49 of them have a confirmed histological diagnosis of WT (12.37 %). Number of males with WT–29/49 (59.18 %),number of females with WT–20/49(40.82 %). Mean age at diagnose: males–3 y10 m; females–3 y9 m; general age distribution: up to 2 year of age–11/49 (24.49 %), from 2 year to 5 year of age–24/49 (46.94 %), from 5 year to 15 year of age–14/49 (28.57).

**Conclusion:** WT makes a significant part of the malignancies in children, having a slight prevalence in males (59.18 % vs 40.82 %). The mean age at diagnose shows no gender difference. From the general age distribution–highest prevalence at the 2–5 year-of-age interval.

Sunday, 1 September 2013, 09.30–10.30, Pavilion 2

**PS-06 Poster Session Soft Tissue Pathology**

**PS-06-001**

**Malignant melanoma: Cutaneous metastasis frequency and survival rate**

C. Tamas ^*^, L. Popa, A. Lazar, C. Stanescu, C. Morosanu, D. Radulescu, D. Turliuc

^*^University of Medicine Iasi, Dept. of Plastic Surgery, Romania

**Objective:** We studied the variation in locoregional metastasis frequency and the relation between clinical form and survival in malignant melanoma (MM).

**Method:** We used the Kaplan-Meier survival curves and Cox proportional hazards model to calculate the survival rate for 263 patients with MM, operated between 2000 and 2010.

**Results:** Half of the patients (51 %) developped limbs MM. Patients with nodular melanoma(98) had a higher risk for cutaneous metastasis(61,22 %) than the rest and a low survival rate(36,73 %). There is a relation beween the number of metastatic limph nodes and the survival rate. This rate decreases with 13,64 % if more than 4 limph nodes are involved:from 16 months,in a single affected limph node,to 10 months for more than 4.

**Conclusion:** The presence of cutaneous metastasis in MM is associated to a very low survival rate. The patients with stage I MM had the longest survival period. In the third stage,the survival rate depends on the number of the metastatic limph nodes(57,14 % IIIA;43,50 % IIIB;12,50 %IIIC). In a small number of cases(4 %) the metastatic tumor was the first sign of MM. Key words: MALIGNANT MELANOMA,CUTANEOUS METASTASIS,LIMPH NODES,SURVIVAL

**PS-06-002**

**A retroperitoneal dedifferentiated liposarcoma with low-grade and high-grade heterogeneous components**

I. Jung ^*^, D. Milutin, S. Gurzu

^*^University of Medicine and Pharmacy, Dept. of Pathology, Tirgu-Mures, Romania

**Objective:** To present a retroperitoneal dedifferentiated liposarcoma with heterogeneous differentiation.

**Method:** A 50-year-old female presented with a 20-cm-sized tumor of the retroperitoneum. The surgical specimen was submitted for histopathological examination

**Results:** Macroscopic examination of the surgical specimen revealed an inhomogeneous aspect on cut section, the gelatinous areas being intermingled with solid zones with whirling-pattern, hemorrhages and necroses being also observed. Histopathologic examination confirmed the heterogeneous aspect. The areas of well-differentiated liposarcoma were admixed with tumor areas that presented histological features characteristics for pleomorphic malignant histiocytoma, osteosarcoma, fibrosarcoma, myofibroblastic and rhabdoid patterns being also seen. Immunohistochemically, the heterogeneous components were confirmed using specific antibodies such as Vimentin, Smooth Muscle Antigen, Desmin, S100, and CD68. Focal positivity was also observed for ALK, CD34 and CD105 but C-KIT antibody did not mark the tumor cells. No recurrences and/or metastases were reported 6 months after surgery.

**Conclusion:** Dedifferentiated liposarcoma seems to have a very heterogenous origin and the correct diagnosis is based on examination of several tumor sections

**PS-06-003**

**Possible predictive value of correlation between synovial angiogenesis and serum level of adiponectin in patients with rheumatoid arthritis**

I. Jung ^*^, R. Melinte, B. Dorcioman, S. Gurzu

^*^University of Medicine and Pharmacy, Dept. of Pathology, Tirgu-Mures, Romania

**Objective:** To present the particularities of synovial angiogenesis and serum level of adiponectin in patients with rheumatoid arthritis (RA) compared with those with osteoarthritis (OA).

**Method:** The immunohistochemical expression of the antibodies VEGF (Vascular endothelial growth factor) and CD31 was analyzed in 20 cases with RA and 10 with OA. The synovium specimens were obtained at the time of total knee joint arthroplasty. For a proper quantification of angiogenesis, the necrotic areas and those with high inflammatory activity were eliminated. In all patients the serum level of adiponectin was also preoperative determined using ELISA kits.

**Results:** The average serum level of adiponectin was significantly high (19718.45 UI) in patients with RA compared with those with degenerative OA (4836.25 UI). VEGF expression was diffusely observed in the synoviocytes of patients with RA but focal positivity was revealed in OA. The number of vessels was also significantly high in RA (9.03 vessels/HPF) compared with OA (4.75 vessels/HPF).

**Conclusion:** High adiponectin levels seem to be correlated with increased synovial VEGF expression. Intraarticular administration of antiangiogenic drugs associated with anti-inflammatory substances could inhibit both local angiogenesis and inflammation. It could prevent the severe synovial damages, usually observed in rheumatoid arthritis.

**PS-06-005**

**Reduced INI-1 expression in gastrointestinal stromal tumors**

G. Karagkounis ^*^, I. Themeli, M. Chantziara, T. Argyrakos, C. Vourlakou, K. Lariou, D. Rontogianni

^*^Evaggelismos Hospital, Dept. of Pathology, Athens, Greece

**Objective:** The loss of the SMARCB1/INI1 tumor suppressor gene protein characterizes malignant rhabdoid tumors, epitheliod sarcomas, myoepithelial carcinomas and renal medullary carcinomas while it is reduced in synovial sarcomas. We study the immunohistochemical expression of INI1 protein in Gastrointestinal Stromal Tumors (GISTs).

**Method:** Twenty GISTs (CD117+, CD34+) were classified according to the WHO/2010 criteria. Immunohistochemical detection of INI1 protein (clone MRQ-27) was performed. Staining was interpreted as: complete loss, partial loss (in more than 10 % of the tumor cells) and reduced (weak expression in more than 10 % of the cells). SPSS 17.0 was used for the statistical correlation of the results.

**Results:** 1/20 (5 %) of cases showed complete loss, 2/20 (10 %) showed partial loss, 4/20 (20 %) showed reduced expression while 13/20 (65 %) retained the expression of INI1. There was no statistically significant correlation between the reduced/lost expression of INI1 and 1. the possible biological behavior of the neoplasm, 2. the prognostic group as specified by WHO/2010, 3. the size of the tumor and 4. the mitotic count (*p* > 0.05).

**Conclusion:** 35 % of GISTs showed reduced expression or loss of INI1 protein. No correlation of the INI1 protein status with the prognostic characteristics of the tumors was proven.

**PS-06-006**

**Immunohistochemical evaluation of VEGF, its receptors and HIF1a expressions in Dupuytren’s disease**

A. Coer ^*^, L. A. Holzer, G. Holzer

^*^University of Primorska, Faculty of Health Sciences, Izola, Slovenia

**Objective:** Dupuytren’s disease (DD) is a benign progressive fibromatosis of the hand and fingers that leads to a permanent and irreversible flexion contracture of the fingers. We hypothesized that hypoxia and/or angiogenic factors are involved in the pathogenesis of DD, so the aim of our study was to analyse the expression of VEGF and its receptors VEGFR1 and VEGF2 in tissue samples obtained from patients with DD.

**Method:** An immunohistochemical study of tissue samples from 32 patients with DD was performed. Frozen tissuesections were analysed and expressions of VEGF, VEGFR1, VEGFR2, and HIF1α were determined immunohistochemically.

**Results:** Nodules in involutionary phase of DD with αSMA positive myofibroblasts were found in 15 samples. In 17 cases almost acellular tendon-like cords were present (residual phase of DD). At least one of the analysed angiogenic proteins was expressed in 9 out of 15 αSMA positive cases. In the involutionary phase, myofibroblasts showed overexpression of VEGF2 and HIF1α in comparison with samples from residual phase.

**Conclusion:** Overexpression of VEGFR2 and HIF1α in the αSMA positive fibroblast-rich nodules indicates that angiogenesis might play a role in the pathogenesis of DD.

**PS-06-007**

**Ossifying fibromyxoid tumor of soft parts: Histological, immunohistochemical and ultrastructural characteristics of the case presenting novel cytogenetic findings**

J. Rys ^*^, M. Iliszko, M. Vogelgesang, K. Knapczyk, A. Kruczak, B. Lackowska, J. Lasota, J. Limon, M. Miettinen

^*^Center of Oncology, Tumor Pathology, Cracow, Poland

Ossifying fibromyxoid tumor (OFMT) is a rare but morphologically distinctive soft-tissue tumor. Since 1989 approximately 450 cases of OFM were described, however cytogenetic characteristics of OFMT have been reported only in eight tumors (of six patients). In all of them clonal chromosomal aberrations have been detected by cytogenetic analysis and the short arm of chromosome 6, in particular 6p21, was frequently involved in OFMT. Notably, a balanced or unbalanced t(6;12)(p21;q24) translocation seemed to be characteristic for OFMT. We present a peculiar case of OFMT localized in the subcutaneous tissue of the thigh presenting the novel cytogenetic findings. The karyotype of the tumor cells exhibited the following numeric and structural aberrations: 44–48,X,−X[3],add(12)(p1?1)[3],der(12)t(12;15)(p13;q15)[5],−13[4],−15[3],−22[4],+r[6],inc[cp12]/46,XX[18] To our knowledge, this is the first reported case in which such complex karyotype with clonal chromosomal abnormalities including the unbalance translocation der(12) t(12;15)(p13;q15), ring chromosome and loses X, 13, 15 chromosomes has been shown.

**PS-06-008**

**Retroperitoneal leiomyosarcomas**

C. Pereira ^*^, C. Melendez, X. Sanjuan, J. Fabregat, L. Secanella, V. Romagosa

^*^Hospital Bellvitge, Anatomia Patologica, Hospitalet Llobregat, Spain

**Objective:** Leiomyosarcoma (LMS) is one of the most frequent retroperitoneal tumours, that appears as large mass at diagnosis. Complete surgical resection is often difficult and they are related with high rate of recurrence, metastasis and death.

**Method:** Were reviewed clinicopathologic and immunohistochemical features of retroperitoneal LMS treated at our institution between 1995 and 2012 and graded following French system.

**Results:** A total of 15 cases were reviewed (9 female, mean age: 60 years). Two were low grade (G1) and 13 high grade. Immunophenotype of G1 maintained all muscle markers, while G3 express actin and caldesmon but lose desmin. Vascular invasion was identified in 3 cases, all graded as 3 of mitosis. Of these, 2 had metastases and died shortly. The recurrence rate was 16.6 % while metastasis was 41.6 %.

**Conclusion:** 1) Caldesmon is more sensitive than desmin to identify high-grade LMS. 2) Desmin negativity does not exclude LMS. 3) LMS scored 3 of mitoses are associated with vascular invasion. 4) Recurrences and metastases occur in any grade, but surgical resection achieve prolonged survival in low grade. 5) The recurrence rate in our series is low and could be related to correct radical excision of primary tumor. 6) Death of progressive LMS is related with high-grade tumors.

**PS-06-009**

**Expression of epidermal growth factor receptor in soft tissue tumours**

K. Kuracinova ^*^, A. Janegova, P. Janega, P. Babal

^*^Faculty of Medicine, Bratislava, Slovakia

**Objective:** Epidermal growth factor receptor (EGFR) is member of the Her-family of transmembrane receptor tyrosine kinases. The expression of the EGFR protein in different subtypes of soft tissue tumours has not been known as thoroughly as in epithelial tumours.

**Method:** Immunohistochemical positivity of the EGFR in 69 samples of soft tissue tumours was evaluated by light microscopy and compared according to their localization.

**Results:** No staining was in liposarcomas, nodose fascitis, inflammatory myofibroblastic tumour, fibromyxoid sarcomas, malignant fibrous histiocytoma, angiosarcoma, chondrosarcoma, synoviosarcoma. Positive membranous staining for EGFR was seen in 23 cases. Ewing sarcoma (3/1), undifferentiated sarcoma (3/1), neurofibrosarcoma (5/5), malignant peripheral nerve sheath tumor (4/1), fibrosarcomas (3/1), leiomyosarcoma (8/2), embryonal rhabdomyosarcoma (18/12), alveolar rhabdomyosarcoma (7/1). Our measurement shows high intensity and positivity in embyonal rhabdomyosarcomas and neurofibrosarcomas in one case of Ewing sarcoma and in two cases of leiomyosarcomas from uterus. From alveolar rhabdomyosarcomas was one sample positive, but this tissue had the mutation in PAX/FKHR gene. These tumours behave more likely as embryonal rhabdomyosarcoma.

**Conclusion:** Our results demonstrated that IHC staining for EGFR shows membranous positivity in multiple subtypes of soft tissue tumours. The results open the possibility to evaluate the effect of EGFR blocking therapies. Supported by ITMS 26240220052.

**PS-06-010**

**Tumours and tumour-like lesions of the hip in the paediatric age**

F. Khanchel-Lakhoua ^*^, K. Mrad, R. Dhouib, L. Charfi, I. Abbes, S. Sassi, M. Driss, R. Doghri, R. Ben Ghorbel, K. Ben Romdhane

^*^Salah Azaiez Institute, Pathology, Tunis, Tunisia

**Objective:** Bone tumours and tumour-like lesions of the hip in children are rare. The aim of our study is to assess the histological type of tumors in children hip.

**Method:** We reviewed all tumors or tumor-like lesions occurred in the pelvis or proximal femur, involving the hip in patients less than 17 years old in Department of Pathology Salah Azaiez Institut from the 1st of January 1994 to 31st of December 2012.

**Results:** We registred 23 tumours or tumor-like lesions in 12 girls and 11 boys with a mean age of 13 years. Lesions occurred specially in the pelvis (78 %). In respectively 13 % and 8 % of cases they touched the sacrum and proximal femur. The majority of tumors (73 %) were malignant tumours, dominated by Ewing sarcoma (23 %). Only 21 % were benign tumour (8,6 % chondroma. 8,6 % chondroblastoma, 4,3 % osteoma). One tumor-like-lesion was registred (anevrysmal cyst).

**Conclusion:** Our results showed the large predominance of sarcoma, especially Ewing sarcoma. They are different from the Rizzoli institute series which is dominated by tumour-like lesions and benign tumours.

**PS-06-011**

**Retroperitoneal liposarcoma: The experience of a Tunisian cancer institute**

R. Hamrouni^*^, L. Charfi, R. Dhouib, K. Mrad, R. Doghri, I. Abbes, N. Boujelbene, M. Driss, S. Sassi, N. Ben Hmida, K. Ben Romdhane

^*^Salah Azaiez Hospital, Pathology, Tunis, Tunisia

**Objective:** The aim of this study was to analyse the clinicopathological features of different subtypes of retroperitoneal liposarcoma.

**Method:** A retrospective study about 20 cases of retroperitoneal liposarcoma (RLPS) diagnosed over a period of 18 years (1995–2012) to evaluate clinical results and prognostic factors for patient survival.

**Results:** We report 20 cases (12 men and 8 women, the mean age was 57.42 years) of RLPS. None of our patients had a percutaneous biopsy of his tumour. Surgery was indicated to all patients. Resection was performed in all cases (we performed a bloc resection in 17 cases and a simple tumour resection in 3 case). Maximum tumor diameter was 250 mm(range,65–350 mm). Histological classification included well-differentiated type in 11 cases, dedifferentiated type in 3 cases, myxoid/round cell type in 5 cases and pleomorphic type in 1 case. Resection of contiguous organs was required in 85 %. Three of patients had an adjuvant therapy. All patients had recurrent disease, and recurrence developed at a mean of 13.23 months after primary or repeated surgical treatment. The 3-year survival rate was 46.98 %

**Conclusion:** RPLS grow slowly and silently. Its prognosis is poor compared to the other histological subtypes of retroperitoneal sarcomas. Only complete excision provides a hope of a cure, this is often difficult.

**PS-06-012**

**Paraspinal juxta-articular myxoma: An unusual benign mesenchymal lesion readily mistaken for malignancy**

C. Beggan ^*^, J. Kinsella, M. Leader

^*^Carrickmacross, Ireland

**Objective:** We present a case of juxta-articular myxoma, a myxoma variant occurring around joints. The main histological differential is myxofibrosarcoma. For management and patient prognosis, separation of these is essential.

**Method:** A 55 year old female presented with a palpable neck mass. A poorly circumscribed cellular lesion, composed of ovoid and spindle cells within a myxoid stroma was observed. Given the absence of mitoses, necrosis, hyperchromasia or pleomorphism a diagnosis of juxta-articular myxoma was made. The cellularity, whilst unusual in a myxoma, is described in juxta-articular myxoma.

**Results:** Myxoma describes any bland hypocellular gelatinous neoplasm. Most are intramuscular and microscopy reveals a paucicellular lesion with round and stellate cells intermixed with abundant myxoid extracellular stroma containing sparse capillary sized blood vessels. No pleomorphism or mitoses are seen. Juxta-articular myxoma is a cellular lesion with increased vascularity. Distinction from a cellular myxoma is made based on location adjacent to joints and lack of GNAS1 mutation. Distinction from low-grade myxofibrosarcoma requires assessment of pleomorphism, hyperchromasia, mitoses and necrosis.

**Conclusion:** We describe the 1st reported case of paraspinal juxta-articular myxoma. Diagnosis requires excision rather than core biopsy, as atypical features may be focal. Complete excision is the mainstay of treatment. Local recurrences are described with incomplete resections.

**PS-06-013**

**Spinal hydatidosis: Report of four cases**

U. Aykutlu ^*^, B. Yaman, B. Doganavsargil, M. Argin, M. Sezak, M. Zileli, F. Öztop

^*^Ege University, Dept. of Pathology, Izmir, Turkey

**Objective:** Hydatidosis is a tapeworm infestation, caused by mainly Echinococcus granulosus (Cystic echinococcosis) or less commonly, Echinococcus multilocularis (Alveolaer echinococcosis). Liver (50–70 %) and lungs (20–30 %) are the primarily affected organs. Spinal involvement is seen in less than 1 % of cases.

**Method:** We present three cystic and one alveoler echinococcosis cases located in spine.

**Results:** Cystic echinococcosis cases were 2 men and 1 woman aged 61, 40 and 13 years-old respectively. The sites of involvement were T11-12, L4-5-sacrum with medullary cavity extention and T8 vertebrae. Alveoler echinococcosis was located in L10-L12 vertebrae of a 37 years-old woman from an endemic region of Turkey. Back pain and leg numbness were the presenting symptoms in all cases. Lytic, expansive, cystic masses and vertebra corpus destruction were observed radiologically. Histologically no protoscolices but prominent cuticular membranes were identified. Alveolaer echinococcosis case was initially misdiagnosed as granulomatous osteomyelitis due to extensive necrosis and palisating histiocytes and absence of cuticular membranes or laminated layers in the first biopsy. All cases recurred in the time.

**Conclusion:** The cases are presented because of their rarity and potential risk of miscibility with tumors radiologically or other types of spondylitis histologically especially when typical laminated layers are tenuous.

**PS-06-014**

**Parosteal osteosarcoma: Clinicopathological study of 10 cases**

W. Ouahioune ^*^, N. Bettaz, M. Guermi, S. Boukhalfa, L. Benrezki

^*^University of Blida, Dept. of Medicine, Algeria

**Objective:** Parosteal osteosarcoma (POS) is the most common type of osteosarcoma of the surface of bone. This rare tumor accounts for about 4 % of all osteosarcomas. POS involves the surface of long bones. We report 10 cases of POS diagnosed in our service during a period of 10 years.

**Method:** On the 81 osteosarcomas diagnosed in our department, 10 of them were POS.

**Results:** Age ranged from 14 to 68 years. The sex ratio was of 6 females/4 males. In 06 cases out of 10, the site of involvement was the femur. For the other cases, the sites were humerus (1 case), tibia (1 case) and the iliac fossa (1 case). Radiologically, diagnosis was raised in 03 cases. All the patients were treated by wide excision. Histologically, diagnosis was easily made in 07 cases. For the remaining cases, some difficulties were faced. 03 patients had recurrences.

**Conclusion:** Parosteal osteosarcoma is a malignant tumor, but still of good prognosis if diagnosed and excised on time.

**PS-06-015**

**Reticular soft tissue perineurioma**

M. Bel Haj Salah^*^, N. Abdessayed, I. Msakni, W. Koubaa, O. Khayat, A. Chadly-Debbiche, R. Jouini

^*^Habib Thameur Hospital, Pathology, Tunis, Tunisia

**Objective:** To describe clinical and histological features of reticular soft tissue perineurioma

**Method:** We report the case of a 60-years-old men who presented with a long lasting tumor of the thigh. Macroscopically, tumor was nodular and measured 14 cm in its greatest dimension. It was well limited with a firm whitish cut section. Microscopic examination showed a proliferation of variable cellularity made of slender fibroblast-like cells with small nuclei forming a net-like pattern and setting in a myxoid or occasionally denser collagenous matrix. Mitotic activity was absent. Tumoral cells were reactive with EMA and CD34. Diagnosis of reticular benign soft tissue perineurioma was made.

**Results:** Perineuriomas are very rare nerve sheath tumors that display pure perineurial cell differentiation. Two main groups are described: intraneural and soft tissue perineurioma. Soft tissue perineuriomas, although more common than their intraneural counterparts, is still relatively uncommon and probably underrecognized. Histologically, they display variable pattern and they often respond to a sclerosing perineurioma. Reticular perineurioma is extremely rare,it is characterized by a lace-like arrangement of cells resulting in the formation of microscopic cysts.

**Conclusion:** Although their histological distinctive features, the diagnosis of soft tissue perineuriomas is usually based on immunohistochemical demonstration of EMA and GLUT1 positivity.

Sunday, 1 September 2013, 09.30–10.30, Pavilion 2

**PS-07 Poster Session Thymic and Mediastinal Pathology**

**PS-07-001**

**Thymoma: Evaluation of clinical and pathological characteristics of 27 cases**

A. Lovrenski ^*^, D. Tegeltija, M. Panjkovic, Z. Eri, I. Jelicic

^*^Institute for Lung Diseases, Dept. for Pathology, Sremska Kamenica, Serbia

**Objective:** Analysis of patients demographic features, simptoms, morpholigical charasteritic any histological types of tumor.

**Method:** Retrospectively we studied 27 medical records of patients reffered to the Institute of pulmonary diseases of Vojvodina, in Sremska Kamenica between the January of 2005 and December of 2011.

**Results:** All types of thymoma more often occured in males, accept type B. The average age in patients with type A was 57 and with type B2 30 years. Patients with type A thymoma and B1 were mainly asymptomatic, while patients with type B2 and AB presented with dyspnea, dysphagia, pain and cough. Myasthenia gravis often was associated with type A (40 %). The most common histologic type was type B1. The largest number of patients, 14 of them (51 %) had a tumor diameter between 5 and 10 cm. Five patients had malignant thymoma, and the most common types of thymoma that showed signs of capsula invasion or pleural and pericardial implants were type AB (60 %) and B2 (50 %).

**Conclusion:** Thymomas occurs mainly in people older than 40 years and are slightly more frequent in females. There is no correlation between the incidence of malignant thymoma and patients age.

**Thymoma type A, HE x 20:** 
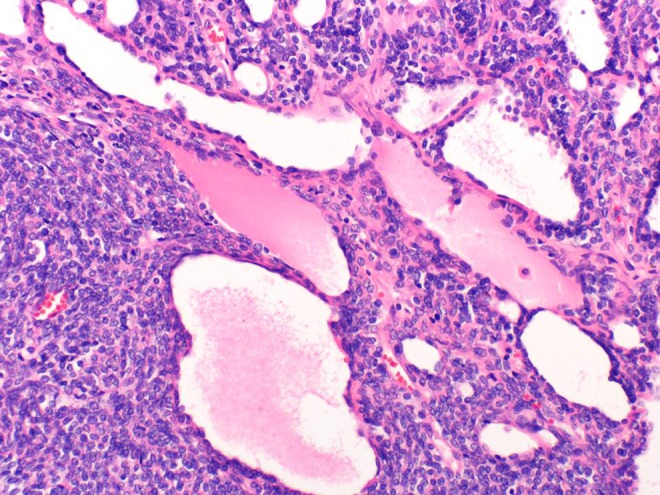


**PS-07-002**

**Primary mediastinal (thymic) large B-cell lymphoma: A case report and review of treatment modalities**

K. Diamantopoulou ^*^, P. Korkolopoulou, G. Piagkos, A. Stamatelopoulos, P. Mihos, H. Mahera

^*^Athens, Greece

**Objective:** Primary mediastinal large B-cell lymphoma (PMBL) is a rare subtype of diffuse large B-cell lymphomas. We present a case of PMBL with partly overlapping immunophenotype of classic Hodgkin lymphoma (CHL) and gray zone mediastinal lymphoma in an attempt to highlight potential therapeutic approaches.

**Method:** A 48-year-old female presented with superior vena cava syndrome, due to a large mediastinal mass with invasion of the right lung, and an enlarged supraclavicular lymph node. Formalin-fixed paraffin embedded tissue obtained from fine needle biopsy of the mass was examined with histology and immunohistochemistry.

**Results:** Microscopically, a diffuse lymphoid neoplasm was observed. Tumor cells were medium to large-sized with pale cytoplasm, round nuclei and occasionally prominent nucleoli. Immunohistochemically, neoplastic cells were CD20(+), CD79a(+), PAX-5(+), CD30(+), Bcl-2(+), Bcl-6(+), MUM-1(+), CD23(+/−), CD15(+ rarely), CD10(−), Ki67(30–40 %). Thymic islets were demonstrated with EMA and CK19. The diagnosis was PMBL, although CHL and gray zone mediastinal lymphoma also entered the differential diagnosis.

**Conclusion:** PMBL is an aggressive B-cell lymphoma (WHO 2008) with unique clinical and molecular features. Treatment approaches, such as R-CHOP, as our case received, with or without radiotherapy, could be implicated with high cure rates.

**PS-07-003**

**Mediastinal thymoma coexisting with intrapulmonary mature teratoma**

N. Mylona ^*^, M. Sofopoulos, S. Tsitsiou, N. Koufopoulos, S. Ntoumou, N. Arnogiannaki

^*^Saint Savvas Hospital, Dept. of Pathology, Athens, Greece

**Objective:** We report a case of a mediastinal thymoma and a simultaneous primary teratoma of the lung.

**Method:** A 72-year-old man with a lobulated mass 7 × 4 × 2,5 cm in the anterior mediastinum and a second lesion 4 mm in the upper left lobe of his lung.

**Results:** Histopathologic examination revealed a B3 thymoma as far as the anterior mediastinal mass and an intrapulmonary mature teratoma concerning the lung’s lesion. The first consisted of multiple nodules separated by fibrous tissue. The epithelial tumor’s cells had medium, oval shaped nuclei without prominent nucleoli. Atypia and mitotic activity were also observed. The lymphoid component comprised of immature T-lymphocytes. The latter lesion consisted of mature bone, adipose tissue and bone marrow, whereas the surrounding pulmonary parenchyma showed histological findings of bronchiolitis.

**Conclusion:** Thymomas and teratomas are two of the four most common anterior mediastinal tumors. However, primary intrapulmonary teratomas are rare and it is thought that these tumors originate from the third pharyngeal pouch which is the anlage of the thymus. Their presence may be due to displacement or separation of the thymus during early embryogenesis and they usually occur in the left upper lobe, as in this case. The treatment for teratomas is resection and the prognosis is excellent.

**PS-07-005**

**Typical carcinoid tumor arising in mediastinal mature teratoma**

M.-M. Koumpanaki ^*^, N. Vladika, P. Xirou, B. Christoforidou, F. Patakiouta

^*^Thessaloniki, Greece

**Objective:** Mature cystic teratomas (MCT) are common neoplasms of the anterior mediastinum. They are composed of mature tissues derived from two or three germinal layers. Malignant transformation is rare (1 %) and usually refers to squamous cell carcinoma. Primary carcinoid tumor occurring in MCT is extremely rare.

**Method:** We report a case of a 57-year old male presenting with a mediastinal mass showing teratomatous features in CT scan. No metastases or lymph node involvement were found. The tumor was excised and we received a cystic thick-walled lesion, sized 6.2 × 4 × 3 cm, filled with yellow-tan material.

**Results:** On microscopic examination, the neoplasm comprised of a variety of tissues from all three germinal layers. In certain areas of the cyst wall aggregations of small uniform cells were seen having morphologic and immunohistochemical features consistent with a typical carcinoid tumor.

**Conclusion:** Only four neuroendocrine tumors arising in mediastinal MCT have been reported. Complete surgical excision was curative in all cases. It is essential to differentiate typical carcinoid within MCT from other types of malignant non-germ cell components and immature neuroepithelial elements with known metastatic potential. In our case, 3 years later the patient is alive and well with no signs of metastases.

**PS-07-006**

**Soft tissue aneurysmal bone cyst in thoracic cavity**

T. Canpolat ^*^, F. Bolat, N. Bal

^*^Baskent University, Pathology, Adana, Turkey

**Objective:** Primary aneurysmal bone cyst is a benign, locally aggresive and expansile tumor that typically occurs in the long bone or vertebral bodies of children and young adults. It was believed to occur exclusively within bone. ABC devoloping in soft tissue is extremely rare but in resent years a few cases presented of soft tissue.

**Method:** We describe a case ABC of the soft tissue in thoracic cavity of 15 years old man. Although the tumor initially was radiologically considered terotoma, clinically cyst hidatic the microscopic features were identical to those faund in clasic ABC We report the imaging and pathological findings of soft tissue (STABC) and discuss its differantial diagnosis.

**Results:** 15- year-old man presented with chest pain and dyspnea. Chest X-ray showed a homogenous mass lesion in the right thorosic cavitiy. Computed tomography (CT) scan of the thorax was done, showed a large predominantly cystic, well-defined mass It had multible enhancing septations and irregular areas of calcification. The ribs were normal The mass was mixed with cystic solid and calcific components and associated without rib. It was adherent to lung parenchyma.

**Conclusion:** Many tumors in the thoracic cavity during childhood should be considered in the differential diagnosis of ABC.

**PS-07-007**

**Pure primary mediastinal yolk-sac tumor in an adult female patient: A case report**

M. Cardoso ^*^, A. Alves, C. Carvalheiro, N. Guerra, J. Caldeira, M. Ferreira

^*^Centro Hospitalar Lisboa Norte, Anatomia Patológica, Portugal

**Objective:** Pure primary mediastinal yolk sac tumor is a rare entity and occurs in male patients and prepubertal women. This is the first case report with histological confirmation in the surgical specimen in an adult fertile woman.

**Method:** A 28-year-old woman presented with an acute and intense retrosternal pain radiating to the left shoulder, dyspnea and a history of mild fever. Chest CT scan revealed a lobulated tumor in the left mediastinum (107 × 87 mm) and elevated levels of αFP (84505kU/L) were found. She was submitted to a mini-sternotomy with tumorectomy and left thymic lobectomy.

**Results:** Grossly, the tumor had a smooth surface with multiple firm white nodules and areas of hemorrhage. Histologically there were solid, microcystic and micropapillary patterns, Schiller-Duval bodies, occasional hyaline globules, extracellular eosinophilic bands and areas of necrosis. Immunohistochemically there was diffuse positivity for alpha-fetoprotein.

**Conclusion:** Extragonodal germ cell tumors are thought to derive from primitive germ cells that mismigrated along the urogenital ridge during embryogenesis. Histological, serological and cytogenetic characteristics of mediastinal yolk sac tumors are similar to their gonadal counter-parts, with major differences in clinical behavior. Although extremely rare, they can also occur in adult female patients, associated with other germ cell tumors or in a pure form.

**PS-07-008**

**The anatomoclinical particularities of thymomas in the center of Tunisia**

S. Hmissa ^*^, S. Korbi, N. Missaoui, S. Mestiri, H. Landolsi, S. Ben Abdelkarim, A. Ben Abdelkader, M. Mokni

^*^Farhet Hached Hospital, Dept. of Pathology, Sousse, Tunisia

**Objective:** Thymomas are rare tumors. Here, we studied their clinicopathologic features.

**Method:** We performed a retrospective study of 25 thymomas diagnosed in the Pathology Department of Farhet Hached Hospital of Sousse from January 1993 to December 2012.

**Results:** The mean age was 41.7 years (23–60 years). Clinically, we observed an alteration of the general state in 20 % of cases, a myasthenia (24 %), a chest pain (36 %), respiratory symptoms including cough, dyspnea, and sputum (60 %). The average time before diagnosis was 3 months. In biology, an inflammatory syndrome was observed in 52 % of cases. Chest computed tomography showed a solid mass located in the thymic in 24 cases. The histological diagnosis was a single case of thymoma A, 2 cases of thymoma AB, 5 cases of thymoma B1, B2 thymoma 3 cases, 5 cases of thymoma B3, and 8 cases of thymoma C. Stages III and IV were the most frequent (72 %). The evolution is marked by five deaths, two recurrences, and two recurrent myasthenic syndrome.

**Conclusion:** Thymomas were diagnosed as aggressive forms (5 cases of B3 thymomas and 8 cases of thymoma C) and at advanced stages (72 %) in the center of Tunisia.

**PS-07-009**

**Late recurrence of a type AB thymoma after primary resection**

J. S. Stadie ^*^, M. Pasic, T. Drews, N. Solowjowa, R. Hetzer, K. Wassilew

^*^German Heart Institute Berlin, Section Heart Pathology, Germany

**Objective:** Thymomas are rare epithelial tumors, but the most common neoplasms of the mediastinum. Case reports of local recurrence or distant metastasis of type AB thymomas are exceptional, even if they presented as Masaoka stage II or III tumors at time of diagnosis.

**Method:** We investigated a case of an 84-year old man with a history of thymectomy and myasthenia gravis, with incidental preoperative finding of a mediastinal tumor in context of an elective heart valve replacement.

**Results:** Radiologically, a peculiarly shaped centrally calcified tumor (80 mm in largest dimension) was noted right antero-laterally of the aorta and the pericardial reflection in the anterior mediastinum. Intraoperatively, the encapsulated tumor showed adhesions to both pleura mediastinalis and visceralis. Microscopically, the mass lesion consisted mainly of bland spindle-shaped epithelial cells arranged in primarily whorled, partly storiform pattern and rosettes with focal invasion of the mediastinal fat. Other fractions of the tumor showed a more lymphocyte-rich component, leading to the diagnosis of a minimally invasive Masaoka stage II type AB thymoma, which was confirmed by the immunophenotype.

**Conclusion:** The presented case highlights the need for long-term follow-up even after resection of a thymoma type tumor, which is considered to recur rarely.

Monday, 2 September 2013, 09.30–10.30, Pavilion 2

**PS-08 Poster Session Cardiovascular Pathology**

**PS-08-001**

**Mitochondrial cardiomyopathy**

S. M. Vesterkaer ^*^, U. Baandrup

^*^Sygehus Vendsyssel, Dept. of Pathology, Hjørring, Denmark

**Objective:** The mammalian mitochondrial genome (mtDNA) is a double-stranded DNA molecule, located in multiple copies within the mitochondria of each cell. Despite a contribution of less than 1 % of the total cellular nucleic acid content, it is fundamentally important for the function of every human tissue. The primary function of mitochondria is to support aerobic respiration and produce, by oxidative phosphorylation, the bulk of cellular ATP. Mitochondria also control cytosolic calcium concentration, regulate apoptosis and host other important biochemical pathways. Mitochondria are under the dual genetic control of both the mitochondrial and nuclear genomes, mutations within either DNA molecule may result in respiratory chain deficiency.

**Results:** I will focus on mtDNA mutations in human disease, by presenting and illustrating two case reports regarding Mitrochondrial myopathy, Encephalopathy, Lactic Acidosis and Stroke-like episodes (MELAS) and Maternally Inherited Diabetes and Deafness syndrome (MIDD), respectively. Mitochondrial diseases have a broad range of clinical manifestations and a highly variable course. Identical mutations may result in different diseases, and different mutations can cause identical disorders. This makes the diagnosis and counseling, of mutation carriers, challenging. Unexplained cardiomyopathy, together with multiple organ diseases, should however lead to a suspected diagnosis. Unfortunately no specific treatment is available.

**PS-08-002**

**Intravital and postmortem morphological studies and magnetic resonance imaging of 80 cases of arrhythmogenic right ventricular dysplasia**

L. Mitrofanova ^*^, N. Mitrofanov, D. Lebedev, M. Gordeeva

^*^Almazov’s Centre, Dept. of Pathomorphology, St. Petersburg, Russia

**Objective:** To improve verification of Arrhythmogenic Right Ventricular Dysplasia (ARVD)

**Method:** Morphometric, histological and immunohistochemical analysis were carried out on 47 endomyocardial biopsies (EMB) from the right ventricular (RV) and on the hearts of 33 patients with ARVD died unexpectedly. Intravital and post-mortem cardiac MRI scans were performed.

**Results:** All the cases showed the atrophy of muscle fibres, lipomatosis and fibrosis in the RV. The average residual cardiomyocyte area was 39,7 ± 12,8 %, the average cardiomyocyte diameter was 10,4 ± 1,5μm. The average thickness of RV anterior wall was 0,14 ± 0,07 cm. 44 % of the patients had myocarditis, 33 % of the patients had myocardial fibre disarray in RV and 60 % had focal γ-catenin expression in muscle fibres. Intravital MRI showed RV dissinchrony in 82 % of the cases, signs of adipose infiltration of RV in 65 % of the cases and mitral valve prolapse in 42 %.

**Conclusion:** To make the diagnosis of ARVD on EMB not only the residual area of RV muscle fibres should be determined but the average cardiomyocyte diameter as well. As to MRI the most important criterion of ARVD is the RV dissinchrony. In some cases we observed the combination of ARVD with mitral valve prolapse and myocardial fibre disarray in RV.

**PS-08-003**

**Left-sided arrhythmogenic ventricular cardiomyopathy with sudden-death in a young man: A case report**

L. De Carvalho ^*^, G. Pozzan, G. Monteiro, F. Bolfarini, R. Penny, G. Ciconelli Del Guerra

^*^Centro Universitário Lusíada, Pathology, Santos, Brazil

**Objective:** Arrhythmogenic ventricular cardiomyopathy is a clinically and genetically heterogeneous heart muscle disorder associated with ventricular arrhythmias and risk of sudden death. The classic form of the disease has an predilection for the right ventricle and is characterized by ventricular myocardial fibrofatty replacement. Biventricular arrhythmogenic cardiomyopathy and left dominant arrhythmogenic cardiomyopathy forms had been included in the spectrum of disease.

**Method:** 21-year-old male presented collapse during the sleep. The heart presented on gross examination a subepicardial, circumferential hemorrhagic lesion at left ventricle.

**Results:** The microscopy showed a myocardial replacement by fibrous tissue with capillaries and scattered lymphocytes. There were no remarkable changes in the wall thickness in both ventricles and coronaries abnormalities.

**Conclusion:** Arrhythmogenic ventricular cardiomyopathy/dysplasia is one of the leading causes of sudden-death in young people. The left-dominant subtype of the disease is still under-recognized and it may precede the onset of significant right ventricular changes,as we reported in this case.

**PS-08-004**

**Myocarditis associated sudden and non-sudden death: The testimony of a series**

R. Henriques de Gouveia ^*^, C. Cordeiro, B. S. Silva, F. Corte Real, D. N. Vieira

^*^INMLCF, I.P., Dept. of Pathology, Coimbra, Portugal

**Objective:** “Myocarditis” are still responsible for important morbility and mortality (5 %) worldwide, whether death setting is natural (sudden and unexpected or not) or non-natural. Authors report the experience of a National Institution.

**Method:** Demographic/circumstancial/toxicological and morphological data of 1,141 autopsies with anatomo-pathological examination (performed in 2011, territorial area = 29.206Km2, inhabitants = 2.595.540) were reviewed.

**Results:** Twenty-three autopsies (2,02 %) presented Myocarditis. They refered to 18 males with mean age of 53,8 years [21–75 years] and to 5 females whose mean age was 77,8 years [40–96 years]. Myocarditis was lymphocytic(*n* = 6), mixed cellularity (*n* = 14) and neutrophylic (*n* = 3). Etiology was infeccious, immunologic, toxic, hypersensitivity. Death occured at home, hotel, restaurant, prison, hospital, ambulance, river. It occured either suddenly (*n* = 16, 69,6 %); unexpectedly, of natural cause, but not suddenly (*n* = 1); complicating car accident with hospitalization (*n* = 4); complicating after explosion burn with hospitalazation (*n* = 1); or due to a fall (*n* = 1).

**Conclusion:** 1) Myocarditis remain an importante cause of Sudden Death. 2) Death due to myocarditis may have legal relevance in insurance disputes, medical negligence suspicion. 3) Knowing the real ‘Health Status’of a country, in what concerns pathologies, causes and death circumstances, both intra and extra-hospitalar data is required. 4) Promoting “Disease National Registries” is of utmost importance and demands multidisciplinary cooperation.

**PS-08-005**

**The structure of pre-transplant heart disease in Belarus**

S. Rjabceva ^*^, O. Yudina, A. Smolensky, Y. Ostrowskiy, P. Yudin, M. Vozmitel

^*^City Pathological Bureau, Pathology, Minsk, Belarus

**Objective:** The main indications for heart transplantation were cardiomyopathy (CMP) arising from end-stage chronic ischemic heart disease, dilatation of the heart cavities and other heart diseases.

**Method:** We examined 58 hearts of recipients which were operated in RSPC “Cardiology” from 2009 to 2011 years by macro- and microscopically.

**Results:** By clinical examinations our patients were with dilated cardiomyopathy (79.3 %), ischemic cardiomyopathy (15.6 %) and chronic rheumatic heart disease (5.1 %). The structure of pre-transplant heart disease has changed after the histological study of heart tissue. We diagnosed a dilated cardiomyopathy (67.2 %), ischemic cardiomyopathy (13.8 %), restrictive CMP (8.6 %). Mitochondrial CMP found in 5.2 % cases, a giant cell myocarditis–in 1.7 %, acute myocardial infarction–in 1.7 %, congenital disorders of connective tissue–in 1.7 % patients.

**Conclusion:** Morphological study showed that myocardial disease of recipients with a heart dilation are a heterogeneous group in Belarus, in which an important place occupied by patients with mitochondrial disorders (5.2 %).

**PS-08-006**

**Sudden death in the young due to myocarditis: The experience of two Iberian forensic centers**

R. Henriques de Gouveia ^*^, J. Lucena, J. Carvalho, B. S. Silva, F. Corte Real, D. N. Vieira

^*^INMLCF, I.P., Dept. of Pathology, Coimbra, Portugal

**Objective:** “Myocarditis” is responsible for 3.5 % of cardiac arrest in the young (1–35 years) occurring outside Health Institutions and eventually leading to Sudden Death. Two ‘Iberian Forensic Centers’ compared their 1-year experience based on the fact that Mediterranean countries share backgrounds, diets and lifestyles.

**Method:** Demographic, circumstantial, toxicological, microbiological and morphological data of autopsies with anatomo-pathological examination (performed in 2011) were reviewed. The ‘Spanish Center’, responsible for a population of 1.917.097 inhabitants, performed 850 autopsies. The ‘Portuguese Center’, serving a population of 2.595.540 inhabitants, performed 1141 autopsies.

**Results:** Both Centers had one case of Sudden Death in young persons due to Myocarditis. Spain: a 6 year-old female deceased of acute fulminant myocarditis, presenting positive microbiological analysis for Influenza B, Parainfluenza 4 and Rhinovirus. Portugal: a 33 year-old male unexpectedly died after diner due to acute Myocarditis. He was on anti-influenza therapy.

**Conclusion:** 1) Spanish and Portuguese experience, in these Centers, is similar concerning Myocarditis as a cause of Sudden Death in the Young. 2) It occurs both in the very young and in young adults. 3) A thorough postmortem examination is crucial for the correct diagnosis.

**PS-08-007**

**A rare case of epicardial involvement in Erdheim-Chester disease**

J. S. Stadie^*^, M. Pasic, K. Hauptmann, R. Hetzer, K. Wassilew

^*^German Heart Institute Berlin, Section Heart Pathology, Germany

**Objective:** Erdheim-Chester disease (ECD) is a rare non-Langerhans cell histiocytosis with heterogeneous systemic manifestations. Cardiovascular involvement is considered an important but rare, and often overlooked, finding. Cardiovascular involvement in ECD mainly occurs as periaortic ‘fibrotic change’, with pericardial involvement and pericardial effusion. Venous, myocardial or epicardial affections and involvement of the coronary arteries are noted less frequently.

**Method:** We report on a case of a 36-year old man with a tumorous cardiac lesion, diagnosed preoperatively as an incidental finding not related to the cause of his hospital admission.

**Results:** Radiologically, an ill-defined pericardially centered lesion, extending to the ascending aorta, the left pulmonary artery and the epicardial fat, encasing both left and right coronary arteries, was noted. Histological and immunhistological examination of the extensively sampled epicardial lesion and of samples from the gluteal tumor diagnosed later during the clinical course showed similar findings, namely diffuse infiltration of plentiful cytologically bland, S-100-negative, CD68-positive foamy histiocytes, admixed with multinucleate giant cells, set in a fibrous stroma.

**Conclusion:** The synopsis of clinical, pathological and radiological findings in this case allows the diagnosis of Erdheim-Chester disease with uncommon primary epicardial manifestation.

**PS-08-008**

**Autonomic innervation of pulmonary veins: Morphological substrate of atrial fibrillation**

I. Kholova ^*^, R. Leinonen, A. Mäkinen, O. Jääskeläinen, T. Paavonen

^*^Fimlab Laboratories, Dept. of Pathology, Tampere, Finland

**Objective:** Atrial fibrillation is the most common sustained tachyarrhythmia. Morphological substrates of atrial fibrillation were found also in myocardial sleeves around pulmonary veins. This study aims to examine immunohistochemically autonomic nerve distribution in pulmonary veins and venous-atrial junctions.

**Method:** Immunohistochemical staining was performed in 48 pulmonary veins and 9 atria samples using antibodies to tyrosine hydroxylase, choline acetyltransferase and GAP-43. The morphometrical analysis was performed with computer-assisted system (ImageJ).

**Results:** Adrenergic and cholinergic nerves and ganglia were often co-located. Their density varied at different compartments. The nerve structures were present both within myocardial sleeves and in surrounding fibro-fatty tissue.

**Conclusion:** Adrenergic and cholinergic nerves and ganglia may play the role in atrial fibrillation triggers from pulmonary veins. However, selective targeting of either vagal or sympathetic nerves is due to co-localization impossible.

**PS-08-009**

**Congenital vascular malformations: Cerebral lesions differ from extracranial lesions by their immune expression of the glucose transporter protein GLUT1**

L. Meijer-Jorna ^*^, E. Aronica, C. M. van der Loos, D. Troost, A. C. van der Wal

^*^Symbiant/MCA, Dept. of Pathology, Alkmaar, The Netherlands

**Objective:** Cerebral vascular malformations were investigated for the presence of the glucose transporter protein GLUT1, which is normally expressed in endothelial cells of pre-existing microvasculature of the brain and absent in vasculature of the choroid plexus and extracranial vasculature without a barrier function. Extracranial arteriovenous malformations (AVMs) are known to show an absence of GLUT1 expression which distinguishes them from infantile hemangiomas of skin and soft tissue. The expression of GLUT1 in cerebrovascular malformations is not systematically investigated.

**Method:** Paraffin sections of 12 cerebral AVMs, including one choroid plexus AVM, 10 cerebral cavernous malformations (CCMs) and 10 extracranial (facial) AVMs were immunostained with anti-CD31 and anti-GLUT1 in an immunodoublestaining procedure which was further analyzed with the use of spectral analysis software.

**Results:** All 22 cases of cerebral vascular malformations (AVMs and CCMs) showed co-localization of GLUT1/CD31 of endothelial lining of the vessels (malformed arteries and veins) within the malformation. In the 10 extracranial AVMs expression of GLUT1 was completely absent.

**Conclusion:** Cerebral AVMs differ from extracranial AVMs by their endothelial immunoexpression of GLUT1, indicating that the vessels of these malformations retain the endothelial phenotype of the local vascular beds from which they are derived during embryogenesis.

**PS-08-010**

**Rupture and dissection in pulmonary artery aneurysms: A case report**

S. Rjabceva ^*^, A. Gerasimovich, E. Adolf, M. Vozmitel

^*^City Pathological Bureau, Pathology, Minsk, Belarus

**Objective:** Pulmonary artery aneurysm (PAA) is an uncommon disorder. Dissection mostly develops in a pulmonary artery aneurysm associated with pulmonary hypertension and/or connective tissue disease. The aim of this study was to describe the histopathological features of PAA.

**Method:** We studied one case of a 44-year-old woman with PAA. We used Hart’s method for elastic fibers and van Gieson stain.

**Results:** A 44-year-old woman was admitted to hospital with chest pain and shortness of breath. She had some features similar to Marfan syndrome: arachnodactylia, pectus deformities and thoracolumbar scoliosis. She died from cardiac sudden death after 11 days. At necropsy, we found the enlarged main pulmonary artery (a diameter of 6.6 cm) with a dissection and a rupture of walls and hemorrhage in pericardial cavity with massive effusion. Coronary artery compression was a reason of myocardial infarct and death of patient. Histologically, there are myxoid degeneration of media, irregular arrangement elastin and collagen fiber in pulmonary artery aneurysm wall.

**Conclusion:** We diagnosed an idiopathic pulmonary artery aneurysm in this patient with Marfan-like phenotype. The diagnosis of PAA is often difficult, the natural history of the disease is not completely understood. Recognition of pulmonary artery aneurysms is important because of the high morbidity and mortality rates of rupture.

**PS-08-011**

**New reference tables for predicted heart weight**

J. Vanhaebost ^*^, M. Faouzi, P. Mangin, K. Michaud

^*^Curml, Lausanne, Switzerland

**Objective:** Knowledge of normal heart weight ranges is important information for pathologists. Comparing the heart weight to reference values is one of the key elements used to determine if the heart is pathological, as heart weight increases in certain cardiac pathologies. Current tables are outdated and were established on different populations. The purpose of this study is to establish new tables relevant to the local population and to determine the best predictive factor for normal heart weight. We also aim to provide technologic support to calculate the predictive normal heart weight.

**Method:** The reference values are based on a study including 288 adult patients without any obvious pathologies who were autopsied in western Switzerland from 2007 to 2011. The statistically analysed parameters were age, gender, height, body weight, BMI and body surface area.

**Results:** Heart weight is statistically correlated with all of the parameters studied. Body surface area is the best predictor of normal heart weight.

**Conclusion:** New references tables for predicted heart weight are presented as well a web application that enables the comparison of heart weights observed at autopsy with reference values. This application can also calculate the BMI and body surface area.

**PS-08-012**

**Postmortem CT-angiography of coronary arteries in cases of sudden cardiac deaths**

K. Michaud ^*^, S. Grabherr, P. Mangin

^*^University of Lausanne, CURML, Switzerland

**Objective:** Cardiac diseases are the most frequent causes of death in developed countries. The most common clinical finding associated with sudden cardiac death in general population is ischemic heart disease related to atherosclerotic coronary artery.

**Method:** At the University Center of Legal Medicine of Lausanne, postmortem CT-scanning and postmortem multiphase CT-angiography are routinely used since 2009 to investigate violent deaths but also natural deaths in which cardio-vascular lesions such as coronary artery diseases are suspected. This technique allows the visualization of the vascular system similarly to clinical investigations.

**Results:** In our experience, the exploration of coronary arteries by postmortem CT-angiography is very useful to analyze and document their anatomy and to detect stenoses, occlusions or calcifications. It also shows advantages in investigating deaths following cardiac surgery or vascular interventions.

**Conclusion:** Interpretation of post-mortem cardiovascular radiology is still a new field and we have to learn to interpret correctly the technique-related artefacts and those caused by post-mortem changes in order to distinguish them from pathological lesions (such as eroded plaques, coronary vasculitis). At the same time, post-mortem radiological examination followed by a complete autopsy by is very promising to further the understanding of interpretation of radiological examination in living patients.

**PS-08-014**

**Splenic lymphangioma with endothelial papillary proliferation in a patient with gastric carcinoma**

N. Dias ^*^, D. Oliveira, M. R. Silva, M. J. Julião

^*^Coimbra, Portugal

**Objective:** Lymphangiomas of the spleen are rare benign vascular tumours. They are classified as simple (capillary), cavernous and cystic (hygroma). Papillary endothelial proliferations in lymphangiomas have been described for long time. To our knowledge, this feature in the setting of splenic lymphangiomas is rare.

**Method:** We present a case of a 62-year-old woman with gastric cancer and a splenic nodule detected during staging CT, suspicious of metastasis.

**Results:** Total gastrectomy and splenectomy were performed. A 2 cm ulcerated tumour was found in the lesser curvature of the antrum and a 1,5 cm well-defined, grey-yellowish, hard nodule was found in the spleen. Histologically the gastric tumour was a moderately differentiated tubular adenocarcinoma invading the submucosa without lymph node metastasis. The spleen nodule consisted of luminal structures, without red blood cells, lined by a flat cell layer without atypia, with multifocal papillary proliferations into the lumen. These cells were CD34 and CD31 positive, as well as CAM5.2 negative, pointing towards an endothelial differentiation.

**Conclusion:** Splenic lymphangioma with papillary proliferations are rare lesions. This finding does not seem to represent a different subtype of lymphangioma nor have worse prognosis, and should not to be mistaken for a low-grade or malignant neoplasia.

**PS-08-015**

**A clinico-morphological review of tumors of heart**

K. Dikarev ^*^, L. Mitrofanova, I. Antonova

^*^Almazov Federal Heart, Blood, and Endocrinology Center, St. Petersburg, Russia

**Objective:** 200 cases benign and malignant tumors of heart have been investigated for 25 years (from 1988 to 2013).

**Method:** Conducted the statistical processing of the main clinical symptoms, microscopic examination of tumors with standard histological staining and immunohistochemical stain in some cases.

**Results:** The most number of tumors were benign (140 cases), predominantly myxoma (80 %). Other tumors were lipoma, papillary fibroelastoma, teratoma. The main symptoms were exertional dyspnea, edemas, arrhythmia, periodic pain of the chest. Sometimes tumors were identified by accident on clinical examination or autopsy. The surgical treatment benign tumors were effective. 2 patients without operation died from heart failure an arrhythmia. Among malignant tumors prevailed metastatic tumors (40 cases), among primary malignant tumors were different types of sarcomas. Prognosis is grave: the patients died during the term of 2 weeks to 1 year from metastases, progressive heart failure, pericardial tamponade. One patient who has received chemotherapy is alive for more than 1 year. Surgical intervention may reduce clinical symptoms and increase a term of life.

**Conclusion:** Prognosis cardiac tumors is difficult to predict, irrespect of their biological potential. There is not any TNM stading for malignant tumors yet. It is necessary to develop clinic-morphological criteria for optimal treatment and prognosis.

**PS-08-016**

**A single centre survey of cardiac tumours over a 16 year period from Singapore**

S. H. Lai ^*^

^*^Singapore General Hospital, Dept. of Pathology, Singapore

**Objective:** This study reviewed the pathology of cardiac tumours in a single tertiary institution in Singapore from 1997 to 2012.

**Method:** The departmental database was searched for cases of cardiac tumours during the period from January 1997 to December 2012 inclusive. Reports and slides of returned cases were reviewed. Demographical and pathological data was analysed and literature review was performed.

**Results:** here were a total of 61 cases, 53 were benign. The incidence was 3.8 per year. Age distribution ranged from 18 to 79 years, with a mean age of 55 years. The overall female to male ratio was 2.8:1. The eight malignant neoplasms included seven metastatic lesions and one primary lymphoma. Malignant lesions were lymphomas (2), renal cell carcinoma (1), sarcoma (1), hepatocellular carcinoma (1), mucoepidermoid carcinoma (1) and liposarcoma (1). Of the benign neoplasms, 48 were myxomas with annual incidence of 3 cases; a female to male ratio of 2.9:1; and occurred in the left atrium in 77 % of cases.

**Conclusion:** This is possibly the largest series of cardiac neoplasms reported from Singapore to date. The results provided an epidemiological and pathological description of cardiac neoplasms and are comparable to those described in the literature.

**PS-08-017**

**Metastasis of squamous cell carcinoma of the penis into a heart with pacemaker mimicking an endocardial vegetation**

C. Tomikawa ^*^, S. A. C. Siqueira

^*^FMUSP, Dept. of Pathology, Sao Paulo, Brazil

**Objective:** Metastasis of penile cancer to the heart is extremely rare and has been scarcely reported. Presented is an autopsy case of metastasis of squamous cell carcinoma of the penis to a heart with pacemaker mimicking an endocardial vegetation.

**Method:** Standardized autopsy examination was performed.

**Results:** A 79-year- old male patient with a permanent pacemaker underwent partial amputation of the penis due to a squamous cell carcinoma of the penis. Less than 1 year later, due to recurrence, emasculation was realized. After the surgery, the patient presented clinical features of sepsis and latter sudden discomfort, evolving to cardiac arrest. Autopsy was performed to elucidate the cause of death, being endocarditis the main hypothesis. The heart was augmented and on sectioning the right chambers a tumor was easily visible, from the atrium to the ventricle through the tricuspid valve, enclosing the pacemaker catheter and resembling an infectious endocardial vegetation. Histological examination showed extensively necrotic tumor, although some preserved areas displayed squamous cell carcinoma.

**Conclusion:** Penile cancer is an uncommon malignancy and his metastasis to the heart is even more. This case is presented to alert the physicians that metastatic tumor, albeit rare, may be a differential diagnosis of endocarditis.

**Metastasis of squamous cell carcinoma of the penis to the heart:** 
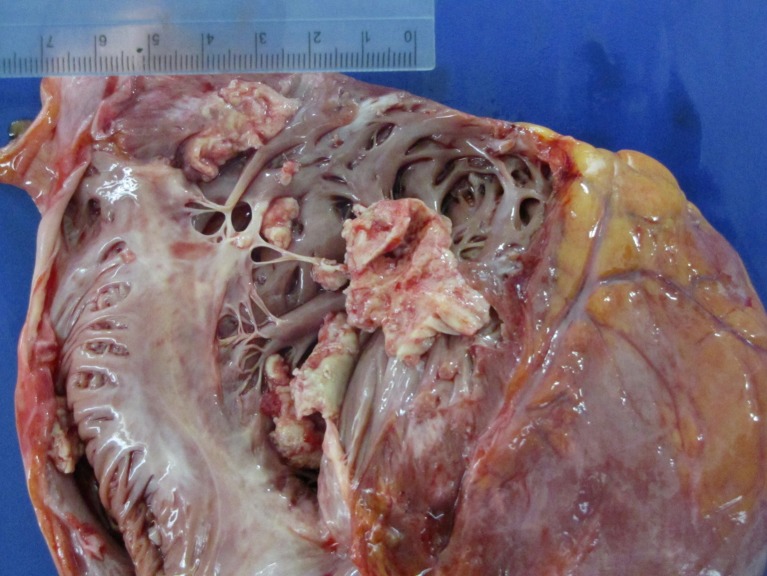


**PS-08-018**

**Intracardiac teratoma in a 7 year old boy: Case report**

A. Rodrigues-Pereira ^*^, E. Pinto, M. Ferraz de Oliveira

^*^Porto, Portugal

A 7-year-old male from Guine Bissau was diagnosed at birth with severe valvular stenosis and had two surgeries both at neonatal period and at 14 months old. He was followed by pediatric cardiology and the only symptom reported was fatigue after physical activity. The last transthoracic ultrasound (US) showed a multicystic mass measuring 6 × 3 cm in the right ventricle chamber adherent to the interventricular septum with US features suggestive of hydatid cyst. The magnetic ressonance confirmed these findings. The child had a third cardiac intervention to remove this mass. Macroscopically, two irregular white fragments were received measuring 4.5 × 3.5 cm and 3.5 × 2.5 cm. On section multiloculated cysts with a mucinous filling and intervening solid areas showing calcification were noted. Microscopically, they consisted in multicystic tumor lined by ciliated columnar epithelium and including structures such as bronchial glands, mature cartilaginous tissue, bone with bone marrow, adipose tissue, skeletal muscle and retina pigment cells. Since endodermal, mesodermal and ectodermal structures were identified, this lesion is classified as an intracardiac mature cystic teratoma. This is a benign rare primary tumor of the heart amenable to surgical cure.

**PS-08-019**

**Solitary fibrous tumor of the heart: An extremly rare neoplasm of the right atrium**

D. Galler ^*^, S. Reinold, D. Hoefer, M. Grimm, H. Popper, C. Ensinger

^*^Innsbruck, Austria

**Objective:** Cardiac solitary fibrous tumor (SFT) is a very rare entity with only 8 cases described in literature. We report on a case of a 68-year old man with a primary SFT developed in the right atrium.

**Method:** The tumor location was confirmed by CT and MRI after clinical symptoms of right atrial masses. Consecutively the well circumscribed 10 × 9 × 9 cm tumor was surgically resected and evaluated by intraoperative frozen sections wich revealed a partly myxoid, mostly fibrous tumor without signs of malignancy and tumor free resection margins. Formalin fixed histological slides showed variable cellularity with dense areas of spindle cells as well as pericytoma like vessels. Other parts revealed collagen bundles that are partly hyalinized.

**Results:** Tumor cells were reacting with CD34 as well as CD99 antibodies and were negative for S-100, desmin, actin, keratins and ALK. This reactivity excluded an inflammatory myofibroblastic tumor as well as neurofibroma from the differential diagnosis. In addition the tumor cells were strongly positive for IGF1R, typical for SFT and partly responsible for hypoglycemia of these patients.

**Conclusion:** Moreover, we found a PDGFR α and β reactivity in a large number of tumor cells, leading to an additional therapeutic option with imatinib in these tumors, especially important for the minority of malignant SFTs (5–10 %).

**PS-08-020**

**Expression of p53, p63 and Ki-67 in cardiac myxomas**

T. Canpolat ^*^, F. Kayaselcuk, A. Tünel

^*^Baskent University, Pathology, Adana, Turkey

**Objective:** Cardiac myxomas are the most common primary tumors of the heart, Although little is known about their etiology.

**Method:** We retrospectively evaluated 27 cases of myxomas and determined the role of p53, p63, Ki-67 in histogenesis.

**Results:** Nine patients (33.3 %) had immune staining in the nucleus for p53 in a limited number of cells. Similarly, nine patients (33.3 %) had immune staining for Ki-67. Especially the areas rich in cells were stained for Ki-67. P63 exhibited positive cytoplasmic and nuclear staining in the perivascular area or individual spindle- or star-like cells scattered on the myxoid stroma. Eight patients (29.6 %) had immune staining for P63 (+) and 12 patients (44.4 %) had immuhistochemical staining for p53 (++). Seven patients (25.9 %) were negative for P63. Tumor size, age, gender and presence of calcification were not correlated with immune staining for Ki-67 and p53 and p63 (p>0.05). Staining for P53 was significantly correlated with staining for Ki67 (*p* = 0.016).

**Conclusion:** We found immunoreactivity to p53 in few cells in 29.7 % of the cases of cardiac myxomas, which is consistent with the benign nature of cardiac myxomas. Extensive p53 and Ki-67 positivity especially in areas rich in cells was suggestive of cellular proliferation.

**PS-08-021**

**Valves damage in amyloidosis**

M. Rybakova ^*^, I. Kuznetsova, A. Goodkova, E. Semernin

^*^State Medical University, Dept. of Pathology, St. Petersburg, Russia

**Objective:** To identify the frequency of valve damage in amyloidosis.

**Method:** The operated valves from patients (150 cases) with different pathology. Clinical diagnosis was rheumatic heart disease in 59 % cases, valves myxomatosis in 14 % cases, atherosclerotic degenerative changes of the valves in 27 % cases. Average age of the patient was 58 years. Immunomorphological examination of biopsy specimens using hematoxylin and eosin, Congo red, van Gieson, polarizing microscopy and immunohistochemistry with antibodies (DAKO) κ and λ, AA, SAP, prealbumin were executed.

**Results:** Amyloidosis was diagnosed in 32 % of removed valves. Amyloid deposits were found in 42 % cases in valves with a clinical diagnosis of myxomatosis, in 30 % cases with a diagnosis of degenerative changes, in 18 % cases diagnosed as rheumatic heart disease, in 10 % cases diagnosed as atherosclerotic deformation of the valve. Amyloid deposits were located mainly along the elastic fibers of valve endocardium, breaking and fibering elastic beams. Around the amyloid and in the valve cusp were detected irregularly and diffuse sclerosis with small focuses of hyalinization and pulverulent calcification. Systemic amyloidosis is proven in 13 % cases on the basis of κ and λ imbalance (1:7).

**Conclusion:** The morphologist may suspect amyloidosis on the basis of disarrangement of collagen and elastic fibers in valve endocardium because of small fibrous eosinophilic deposits.

**PS-08-022**

**Hypoplastic left heart syndrome: Structural changes of the left ventricular myocardium**

V. Zakharova ^*^, T. Savchuk, O. Rudenko

^*^National Institute of CVS, Laboratory of Pathomorphology, Kyiv, Ukraine

**Objective:** to study the structural features of the LV myocardium at HLHS.

**Method:** fetal and newborn hearts: 18 with HLHS and and 20 without heart pathology. The thickness and the areas of LV compact and trabecular myocardium, and ventricular cavity narrow sizes at different levels of LV were determined on serial histological sections. The architectonic of myocardium was evaluated, the number and sizes of figures of cardiomyocytes disorientation were counted. Morphometric data of myocardium were compared with data of other elements of the HLHS.

**Results:** In 8 cases LV cavity was reduced as a result of its walls hypertrophy at a sharp stenosis or obstruction of the aortic valve and the presence of left atrioventricular connection. LV of 4 hearts was dramatically reduced. Its walls look as chaotic myocytic-vascular conglomerate. In 6 cases there were a sharp increased of amount of trabecules and decreased of narrow and other sizes of LV.

**Conclusion:** There were different versions of the LV hypoplasia at HLHS. Some of them are genetically determined, other one are result of intracardiac hemodynamic disturbances at changes in mitral and aortic valves.

**PS-08-023**

**Morphological comparison of electrocoagulation methods and biological tissues welding**

G. Mylovydova ^*^, V. Boyko, A. Lelytsia, I. Nalcha

^*^Laboratory of Pathology, and Exp. Surgery, Kharkiv, Ukraine

**Objective:** To investigate morphological changes in tissues under using the apparatus ЕK-300М1 (LigaShur analogue) and monopolar electrocoagulator for welding biological tissues.

**Method:** The research was conducted on the pig weighing 30 kg. Wide median laparotomy was executed under endotracheal general anesthesia. Next, electrocoagulation of vascular and parenchymal organs was performed through the use of medical welder EK-300M1 and monopolar coagulator. Estimated criteria were haemostasis degree, the extent of necrotic, necrobiotic and haemodynamic changes in the impact zones.

**Results:** When using monopolar electrocoagulator, coagulation was possible only when exposed to small vessels (up to 2 mm diameter). Upon that the area of coagulation necrosis was 450 μm. When using the ЕK-300М1 the diameter of vessels exposed to coagulation was over 3 mm. Complete hemostasis was thus achieved. Macroscopically the wound edges were whitish and practically did not go beyond the electrode edges. Area of coagulation necrosis did not exceed 250 μm. Microscopically, the area of welding represents denatured protein molecules of collagen and elastin forming “protein bridges” that help to hold connected surfaces of blood vessels.

**Conclusion:** Using medical welding unit ЕK-300М1 we can achieve quality haemostasis of vessels exceeding 3 mm in diameter and cause minimal morphological changes compared to monopolar electrocoagulation.

**PS-08-024**

**The heart as an unusual primary site of an unusual neuroendocrine tumor**

S. Ortiz ^*^, F. Tortosa, M. J. Correia, L. Rosário

^*^Hospital de Santa Maria, Dept. of Anatomia Patológica, Lisboa, Portugal

**Objective:** Cardiac paraganglioma tumors are rare neoplasms derived from the neural crest in the mediastinum, which account for 0,3 % of all mediastinal neoplasms. Primary cardiac paragangliomas are extremely rare, occurring in only 0,001 %–0,003 %, with female predominance. Although these tumors may be a cause of hypertension, they are the underlying cause of only about 0,01 % of cases of high blood pressure. Intracardiac paragangliomas have been found mostly in the left atrium. To our knowledge, only 6 previous cases of right atrial paraganglioma have been described in the world medical literature.

**Method:** A 53-year-old woman (with hypertension and diabetes) presented with a cardiac tumour detected by computed tomographic scan. A total tumorectomy was performed and revealed a 6,3 cm mass, with 89,1 g, located in the right atrial of her heart.

**Results:** Histologically, it had the usual “zellballen” growth morphology of a paraganglioma. The immunohistochemistry study showed positivity of chief tumour cells for chromogranin A and S-100 protein reveals the sustentacular cells. A diagnosis of paraganglioma was established.

**Conclusion:** Extra-adrenal paraganglioma is rare and those arising in right atrial are extremely rare. After surgery, the patient was free of clinical symptoms with normal blood pressure and without evidence of local recurrence or metastasis.

**PS-08-025**

**Immunohistochemical evaluation of allogeneic mesenchymal stem cells differentiation after different ways of transplantation in an experimental model of myocardial necrosis**

S. Rjabceva ^*^, O. Yudina, N. Zherdetskaya, O. Kardash, Y. Ostrowskiy, P. Yudin, M. Vozmitel

^*^City Pathological Bureau, Pathology, Minsk, Belarus

**Objective:** Experimental investigations on the model of myocardial necrosis (MN) showed better recovery of cardiac function after administration of mesenchymal stem cells (MSCs), but which way is preferable to use of MSCs has not been choose. The aim of this study is an immunohistochemical evaluation of MSCs differentiation after their transplantation by different ways in the experiment.

**Method:** MN formed by intramyocardial injection of ethanol. MSCs of bone marrow male rats were injected in the lateral tail vein (2*106 cells, *n* = 6, OG1) and into myocardium (5*105 cells, *n* = 6, OG2) of other rats (female, line Wistar, 22 ± 20 g) after 7 days. The control group (CG) were without MSCs (*n* = 6). The zone of MN was assessed by immunohistochemically after 21 days. We used such markers as CD31, CD34 and CD68.

**Results:** CD68 positive cells were not detected in groups. CD34 positive cells were higher than CD31 positive cells in OG2 and CG (*p* < 0.05). The number of CD31 positive cells were increased in group with intravenously injection of MSCs (0.58 ± 0.10 cells/1 mm2) than in group with intramyocardial transplantation (0.32 ± 0.10/1 mm2) and without MSCs (0.25 ± 0.09/1 mm2).

**Conclusion:** Intravenous transplantation of MSC showed better a development of angiogenesis than after intramyocardial way of injection.

**PS-08-026**

**‘Bone’ in the heart!…**

R. Henriques de Gouveia ^*^, J. Abecasis, T. Nolasco, S. Boshoff, S. Ramos, M. Mendes, J. P. Neves, D. N. Vieira

^*^INMLCF, I.P., Dept. of Pathology, Coimbra, Portugal

**Objective:** Cardiac Myxomas’ incidence is estimated in 0,5/1.000.000 inhabitants/year. Histological pattern may be classical or reveal assessory structuro-morphological elements, whose presence may prove to be of high clinical relevance.

**Method:** In a geographical area with 2.821.876 inhabitants, from the 10 cardiac myxomas surgically excised at our Institution in a time-span of 1 year and 11 months, the authors report 2 cases of clinicaly unexpected Ossified Myxomas.

**Results:** They were removed from the left atrium of a 60 year-old male and a 65 year-old female, both with signs of heart failure. They measured 3.5 cm and 5.5 cm, respectively; were ‘bony hard’ and required decalcification before technical handling of the specimens. Histological examination disclosed extensive areas of metaplastic bone, partialy obscuring the characteristic tumoral features.

**Conclusion:** Systemic and pulmonary embolization of myxoma fragments is not only a frequent complication (30–40 %), but is also associated to serious (stoke, …) or even fatal (sudden death) consequences. Thus, the presence of tumor Ossification, although rare, may 1) reduce the risk of embolization (due to less tumor friability) and 2) favour an early imagiologic diagnosis (due to its opacity/echogenicity/…), avoiding catastrophic outcomes from childhood to elderly.

**PS-08-027**

**Expression of matrix metalloproteinase-3 in ischemic myocardium**

D. Pangonyte ^*^, V. Buneviciene, I. Balnyte, L. Utkiene, L. Peciulyte

^*^Lithuanian University of Health, Dept. of Pathology, Kaunas, Lithuania

**Objective:** The aim of the study was to evaluate the expression of matrix metalloproteinase-3 in left ventricular myocardium in the presence of chronic myocardial ischemia.

**Method:** Heart specimens with persistent ischemia (*n* = 20, pre-infarction ischemic heart disease (IHD) group) and post-infarction scar (*n* = 20, post-infarction IHD group) from dissected males who had died suddenly and heart explants (*n* = 15, end-stage ischemic heart failure group) were studied. Heart specimens (*n* = 20) selected at autopsy from individuals who died from accidents were used as controls. The slides of myocardium were incubated with monoclonal antibody against the matrix metalloproteinase-3 (MMP-3) (clone 1B4, Santa Cruz).

**Results:** Expression of MMP-3 in the cardiomyocytes of pre-infarction IHD group did not differ from that of control group. It was higher (*p* < 0.05) in the myocardium of post-infarction IHD group and the highest in the myocardium of patients with the end-stage ischemic heart failure. Number of myocardial interstitial cells expressing MMP-3 increased already in the pre-infarction IHD group (*p* < 0.05) as compared to controls. It was higher (*p* < 0.05) in the myocardium of post-infarction IHD group and the highest in the myocardium of patients with the end-stage ischemic heart failure.

**Conclusion:** Expression of collagen-degrading MMP-3 is increasing in progression of ischemia-induced myocardial dysfunction.

**PS-08-028**

**ADAM10 is a major metalloproteinase involved in Antibody-mediated Rejection (AMR) in human cardiac allografts**

C. Toquet ^*^, A. Pabois, N. Gerard, S. Pattier, C. Laboisse, B. Charreau

^*^CHU Nantes, Dept. of Anapath. B, France

**Objective:** This study investigates the contribution of ADAMs and Notch signaling to vascular injury associated with AMR in cardiac allografts.

**Method:** Regulation of ADAM10, -15, -17, Notch receptors (Notch1, 2, 3, 4) and ligands (Jagged1, Dll4) and VCAM1 was analyzed by quantitative PCR and by immunohistochemistry in cardiac biopsies from patients with AMR (*n* = 9) or stable graft (*n* = 4), non failing heart (*n* = 8) and dilated cardiomyopathy (*n* = 9). ADAM10 proteolytic activity and Notch pathway were further investigated in cultured endothelial cells (EC) from donor transplants.

**Results:** We found that AMR induced by donor-specific anti-HLA is characterized by an upregulation of both ADAM10 and ADAM17 mRNAs (4.3 and 3.4-fold increase versus controls, respectively, *p* < 0.01). ADAM10 is located in graft EC and in macrophages and some T cells. AMR also associates with a significant increase in Notch ligands Jagged1 and Dll4 and drastic downregulation of the endothelial Notch4. In cultured EC, TNF recapitulates ADAM10-dependent Dll4/Notch4 imbalance. ADAM10 blockade also efficiently decreases the production of pro-inflammatory cytokines and chemokines.

**Conclusion:** ADAM10 is a major metalloproteinase driving proteolytic events involved in inflammatory responses and immune cell recruitment during AMR through the Notch pathway. ADAM10 should be considered as a potential therapeutic target.

**PS-08-029**

**Morphologic matrix of reocclusion after arteries reconstruction**

D. Maystrenko ^*^, K. Pozharisski, A. Kudaybergenova, M. Generalov, P. Tarazov, A. Bykovsky, F. Zherebtsov

^*^Rncrct, St. Petersburg, Russia

**Objective:** To determine a morphologic matrix of reocclusion of the artery after its reconstruction

**Method:** 9 objects were examined within the work: 2 areas of the superficial femoral artery in the zone of distal anastomoses with synthetic protheses and 2 areas of the popliteal artery in the zone of distal anastomosis with autovein being formed during surgery on femoral-popliteal bypass; 2 areas of reocclusioned superficial femoral artery and 1 area of the external iliac artery after loop-shaped endarterectomy; 2 fragments of material substrate occlusing the stent lumen in the superficial femoral artery. Material was taken from patients with peripheral artery disease of the II stage, during the repeated surgery. Standard and immunohistochemical (for collagen I, III, IV, VI, elastin, colpamine, as well as CD 3, 20, 31, 34, 45, 68,105) morphologic studies of the resected material were performed

**Results:** The substrate that blocked the vessel lumen in the occlusioned area consisted mainly of collagen IV. Endothelium was completely absent

**Conclusion:** Reocclusion of the areas of the arteries reconstruction is caused not by hyperproliferation of mythic neointima, but by formation of the matrix of connective tissue as a response for irritation of vascular wall structures.

**PS-08-030**

**Non infectious aortitis: A morphological analysis of 72 cases with clinicopathological correlation**

C. Ryan ^*^, A. Barbour, L. Burke, M. Sheppard

^*^Cork University Hospital, Histopathology, Ireland

**Objective:** Aortitis is an important cause of thoracic aortic disease. We describe its histopathologic patterns and ascertain associations with systemic connective tissue disease (CTD) and other aortic pathologies.

**Method:** Database searches of thoracic specimens over 17-years from two centres yielded 72 cases of non-infectious aortitis. Histologic verification of tunica media inflammation was required for inclusion. Histopathologic features were recorded on retrospective slide review. Clinical information was retrieved from reports.

**Results:** 13/72 had systemic disease known to be associated with aortitis, 59 were isolated/idiopathic. Three histologic patterns emerged–classic giant cell aortitis (GCA), diffuse band-like aortitis with/without giant cells and other. Histologic patterns corresponded with clinical features. 50/53 cases of classic GCA were isolated/idiopathic. 9/16 cases of diffuse aortitis had systemic CTD. Prominent cystic medial degeneration (CMD of equal/more significance than medial inflammation) occurred in 23/72 cases; all in the classical GCA category. One case of classical GCA associated with prominent CMD also had polymyalgia rheumatica.

**Conclusion:** Aortitis is mostly idiopathic/isolated but its varying histologic pattens have distinct clinical associations. The diffuse pattern may predict CTD. Prominent CMD was present only with the classical GCA pattern suggesting that one may cause the other. Knowledge of histopathologic patterns may guide patient management/follow-up.

**PS-08-031**

**Takayasu arteritis and aortic valve regurgitation: The histology of aortic valve of Takayasu arteritis at the time of valve replacement**

H. Ishibashi-Ueda^*^, K. Ohta-Ogo, T.-a. Matsuyama, Y. Ikeda, H. Ogino

^*^National Cardiovascular Center, Pathology, Suita, Japan

**Objective:** Takayasu arteritis (TA) is an inflammatory arterial disease of unknown etiology. Aortic valve regurgitation (AR) sometimes occurs in late stage. The histology of aortic valve of TA is not documented in literature.

**Method:** Ascending aortic walls and aortic valves surgically obtained from 69 patients with TA and AR between 1981 and 2010 were retrospectively reviewed. Three quarters of the patients were female. The average age was 50.4 years old.

**Results:** The 15-year survival rate was 76.l%. Late dilatation (>50 mm) of the residual ascending aorta after operation occurred in 8.8 %. Histologically, scar with persistent aortitis was seen in 35 cases (50.7 %). Active florid aortitis was recognized in 10 cases (14.5 %) and 4 prosthetic valve detachment occurred in these 10. Scar stage without inflammation was seen in 24 cases (34.8 %). Operated all aortic valves showed myxomatous degeneration. Only 3 cases of aortic valve (4.3 %) showed inflammatory or post-inflammatory change. Univariate analysis of background variables revealed active inflammation to be a risk factor for detachment (*P* = 0.0001; risk ratio 55).

**Conclusion:** AR in cases of Takayasu arteritis may not be correlated with inflammation involvement to valves, but may be associated with aortic root inflammation.

**PS-08-032**

**Thoracic aortic aneurysm with cystic medial degeneration: A rare complication of psoriatic arthritis**

I. H. Ozbudak ^*^, H. S. Toru, O. N. Tuncer, O. Erbasan, G. Ozbilim

^*^Akdeniz University, School of Medicine, Dept. of Pathology, Antalya, Turkey

**Objective:** Psoriasis is a chronic immunomediated inflammatory skin disease. A considerable proportion of these patients develop a form of inflammatory arthritis. Patients with psoriatic arthritis (PsA) have an increase risk of cardiovascular disease. Herein, we present a case with PsA and thoracic aortic aneurysms.

**Method:** A 37 year-old male patient was admitted with a sudden onset of severe back and chest pain, cough and hemoptysis. In his history, he had aortic aneurysm which was detected radiologically as 73 mm in diameter following the evaluation for unstable angina and mild hitting back pain. He had diagnosis of psoriasis and psoriatic arthritis. Regarding the history and sudden complaints, he underwent surgery. Ascending thoracic aorta aneurysm was found and the segment was changed with graft.

**Results:** The histopathological examination of the vessel wall revealed cystic medial degeneration and pseudocysts formation in the media, accompanied by extensive loss of elastic lamella. These changes result in the medial weakening that progresses to aneurysm.

**Conclusion:** Active PsA is a risk factor for cardiovascular disease, and so PsA patients should be also screened for thoracic aorta aneurysm. The case is reported for its rarity in occurrence and significant morbidity and mortality rate if not diagnosed and treated immediately.

**PS-08-033**

**The spectrum of prominent lymphoplasmacytic inflammatory infiltrates in the aorta and in periaortic tissue**

K. Wassilew ^*^, R. Hammerschmidt, M. Pasic, S. Buz, R. Hetzer

^*^Deutsches Herzzentrum Berlin, Inst. für Herzpathologie, Germany

**Objective:** Lymphoplasmacytic inflammatory infiltrates are the foremost incidental finding in routine examination of aortic specimens. Although recently attention has been given to inflammatory aortic wall changes, the diagnostic criteria of IgG4- related chronic periaortitis have not been clearly defined.

**Method:** We examined all aortic specimens taken in 1/2010–01/2013 for plasma-cell-rich inflammatory infiltrates. Positive cases were further characterized using immunohistochemistry (CD3, CD20, CD68, CD138, IgA, IgG, IgM, IgG4).

**Results:** The aortic wall of 907 patients was examined histologically: 25 (dissecting) aneurysms (2.75 % of studied cases) showed plasma-cell-rich inflammatory infiltrates. IgG4- positive plasma cells were present solely in the adventitia of the ascending aorta in six, mainly elderly patients (mean age 63 years). Moreover, IgG4 expression of plasma cells was observed not only in association with systemic autoimmune disease (*n* = 1, with known lupus erythematosus) but also in necrotizing (giant cell) aortitis (*n* = 7) and atherosclerotic aneurysms, mimicking vasculitis. In two of the 25 cases, aortitis was suspected clinically.

**Conclusion:** No isolated IgG4-related aortitis of the descending aorta could be identified in this large study cohort.

**PS-08-034**

**Histopathological changes of the aorta in patients with Marfan syndrome**

K. Wassilew ^*^, P. Gehle, R. Hammerschmidt, M. Bauer, R. Hetzer

^*^Deutsches Herzzentrum Berlin, Inst. für Herzpathologie, Germany

**Objective:** The morphological correlate of Marfan syndrome in the aorta is attributed to the mutation coding for fibrillin 1 gene. Fibrillin-1 protein functionally mediates interconnections between elastic fibers and between smooth muscle cells. In consequence, mutations in the fibrillin gene result in typical structural abnormalities of the aortic media as fragmentation and malalignment of the elastic lamellae, proliferation and/or depletion of smooth muscle cells and accumulation of mucopolysaccharids in the interstitial matter. Little is known about severity and distribution of these morphological features in the aortic wall.

**Method:** We searched our database for Marfan patients who had undergone reconstructive aortic surgery in 01/2011–10/2012. The H&E, EvG and AB/PAS stained slides were systematically reviewed.

**Results:** Nineteen of 27 patients (7 female, 10 genetically tested, 27–67 years old) presented with aneurysms. Histologically, the structure of the media was altered in all patients, with the full spectrum of Marfan-related changes in four of them. Nineteen showed at least focally moderate fragmentations of the elastic lamellae, eight proliferation of smooth muscle cells and 25 elevated levels of mucopolysaccharids (ranging from 50 to 70 %).

**Conclusion:** The severity and distribution of medial changes predisposing patients to development of aortic aneurysm vary considerably in this patient group.

**PS-08-036**

**Investigation of aortic allografts after different storage variants of cryopreservation (experimental study)**

S. Rjabceva ^*^, S. Spiridonov, O. Yudina, Y. Ostrowskiy, M. Vozmitel, S. Drik, O. Solodkaya, P. Yudin

^*^City Pathological Bureau, Pathology, Minsk, Belarus

**Objective:** Cryopreservation produces serious damage to cytosolic and mitochondrial functions of both endothelial cells and fibroblasts. The cryopreservation itself may cause the collagen metabolism to become degradable, which will lead to degeneration, aneurysm formation or rupture of aortic allograft after implantation. The aim of our study was to histological investigation of cryopreservation aortic allografts after different temperature storage.

**Method:** The 22 aortic allografts were treated with an antibiotic cocktail containing cefazolin, fluconazole and metronidazole. 12 aortic allografts (G1) were stored in liquid nitrogen at −197C and 10 allografts (G2) were stored in the vapor of liquid nitrogen at −150C, using dimethyl sulfoxide as a cryoprotectant. Thawed allografts were evaluated visually and histologically after 4 days of storage.

**Results:** Degenerative changes of aortic wall were not found in allografts of G2. Loss of endothelial cells and normal structural complexity of aortic wall was found in allografts of G1. Pathological changes included a fragmentation and homogenization of collagen and elastic fibers with myxoid degeneration of aortic media and focal cytolysis of smooth muscle cells.

**Conclusion:** The storage of aortic allografts in vapors of liquid nitrogen at −150C is optimal, as it contributes to the conservation of integrity and structures of allografts.

**PS-08-037**

**Case report: Pathological finding of mesenteric thromboangiitis obliterans lesion-like in a young male with systemic lupus erythematosus**

R. Sampaio ^*^, J. Palla Garcia, J. Ramon Vizcaino

^*^Oporto’s Hospital Centre, Dept. of Surgical Pathology, Porto, Portugal

**Objective:** Thromboangiitis obliterans (TO) or Buerger’s disease is an inflammatory occlusive disease occurring in young smokers that affects medium and small arteries and veins of the upper and lower extremities. Mesenteric involvement of TO is very rare. To date, 43 cases of visceral TO have been reported and none of them in the context of lupus or without clinical criteria of TO.

**Method:** We present a case of a 27-years-old, non smoker male patient, diagnosed with systemic lupus erythematous 15 years ago, who came to the emergency room with diffuse abdominal pain, rectal bleeding and vomit. After radiographic and clinical suspicion of intestinal perforation an exploratory laparotomy was performed. He then underwent low anterior ressection of the rectum with terminal colostomy.

**Results:** The histological examination of the received recto-colonic segment and epiplon revealed numerous vasculitic lesions involving arterial branches and veins without fibrinoid necrosis of the wall or calcifications, presence of perivascular fibrosis and preservation of the internal elastic lamina of arterial vessels.

**Conclusion:** Collagen disease was ruled out due to histological findings, the absence of immunoreactivity for IgG4 and because no autoimmune antibodies or anticardiolipin antibodies were evident at serologic testing.

Monday, 2 September 2013, 09.30–10.30, Pavilion 2

**PS-09 Poster Session Digestive Diseases Pathology II: Lower Gastrointestinal Tract**

**PS-09-001**

**Mesenteric veno-occlusive disease of terminal ileum and concomitant appendiceal mucinous cystadenoma with cytopenic pseudomyxoma peritonii**

C. Barbatis ^*^, F. Daglis, M. Kefala, E. Tsiakalou, V. Samaras

^*^Hellenic Red Cross Hospital, Dept. of Pathology, Athens, Greece

**Objective:** Mesenteric Veno-Occlusive disease (MVOD) comprise Idiopathic Myointimal Hyperplasia of Mesenteric Veins (IMHMV) and Mesenteric Inflammatory Veno-Occlusive Disease (MIVOD) without absolute acceptance that they either represent two separate entities or a spectrum of one disease with the IMHMV as the end stage of MIVOD. We present the first case of IMHMV type lesion in the terminal ileum of an 81 old man.

**Method:** A 66 cm segment of terminal ileum with 5 cm of caecum and appendix was examined and paraffin embedded tissue was studied with H+E and other histochemical and immunohistochemical stains. The patient, had a clinical history of ascites, weight loss, recurrent colicky abdominal pain and constipation.

**Results:** The appendix showed mucocele due to mucinous cystadenoma with cytopenic pseudomyxoma peritonii. The terminal ileum had a stricture with cobble-stone ulcerated mucosa, thick wall, matted intestinal loops and uninvolved caecum. Histologically the stricture had features of chronic ischaemia due to extensive myointimal hyperplasia of mesenteric veins without lymphocytic/necrotizing or granulomatous inflammation.

**Conclusion:** Our patient was not a young male with rectosigmoid disease but with exclusive myointimal hyperplasia of mesenteric veins without venulitis. We proposed a diagnosis rather of an End Stage MIVOD than IMHMV. The appendiceal lesion was most probably coincidental.

**PS-09-003**

**Malacoplakia of the colon**

E. Boudabous^*^, A. Heifa, H. Nfoussi, A. Zehani, I. Chelly, S. Haouet, N. Kchir

^*^Rabta Hospital, Dept. of Pathology, Tunisia, Tunisia

**Objective:** Malacoplakia, is a rare chronic, granulomatous inflammatory disease described at the first time in 1920.). Gastrointestinal tract is the second most common site for the occurrence of malakoplakia after the genitourinary system.

**Method:** We report one case of malacoplakia of the colon referred to our institution

**Results:** A 43-year- old man with 1 year history of intermittent diarrhea. On admission he presented with abdominal pain. No abdominal tenderness was noted on physical examination. Colonoscopy revealed pseudo-tumor yellow mass on the right colon. Biopsy was taken and he diagnosis of malacoplakia of colon was made on the histological analysis of the lesion, which showed an inflamed mucosa and submucosa with a dense cellular infiltrate composed of histiocytes(CD68+) having abundant cytoplasm and small round nuclei. The cytoplasm was granular and contained round laminated structures with central dark area and peripheral pale halo, These PAS positive structures were the Michaelis-Guttmann bodies.

**Conclusion:** Malacoplakia may be more common than usually suspected especially in immunodeficient patients and that a thorough study on the newly reported cases may eventually shed some light upon these disease.

**PS-09-004**

**Prevalence of subclinical amyloidosis in large intestine biopsy material**

M. Bialas ^*^, G. Dyduch, K. Milian-Ciesielska, S. Demczuk, K. Okon

^*^Jagiellonian University, Dept. of Pathology, Krakow, Poland

**Objective:** The aim of this study was to determine the prevalence of subclinical amyloidosis in two groups of patients: below and above 70 years of age based on large intestine biopsy taken for unrelated reasons in patients without the suspicion of amyloidosis.

**Method:** 200 cases of large intestine biopsy taken from patients without suspicion of amyloidosis were found in the files of Pathomorphology Department in Cracow. Each group of patients consisted of 50 males and 50 females, age respectively: 50 to 69 and over 70. Congo red stain was performed in every case. In cases with congophilic deposits immunohistochemistry with five most common amyloid directed antibodies was done.

**Results:** Deposits of AA type amyloid in intestinal submucosa and small blood vessels were found in a 58 years old female suffering from rheumatoid arthritis and in a 82 years old male operated for sigmoid cancer 5 years before.

**Conclusion:** 1. Subclinical amyloidosis was rare. No difference in the prevalence of asymptomatic amyloidosis in groups below and over 70 was found. 2. In our material amyloidosis in asymptomatic patients was of AA type. 3. No cases of senile amyloidosis were found.

**PS-09-005**

**The efficacy of digitally reinforced hematoxylin-eosin polarization technique in diagnosis of rectal amyloidosis**

G. Emiroglu Buberal ^*^, B. Doganavsargil, B. Sarsik, M. Sezak, S. Sen

^*^Ege University, Dept. of Pathology, Izmir, Turkey

**Objective:** We searched the efficacy of a recently described Digitally reinforced Hematoxylin-eosin polarization technique (DR-HEP) in detecting amyloid depositions in Hematoxylen-Eosin stained rectal biopsies.

**Method:** The details of the technique were described elsewhere (Turk Patoloji Derg. 2012;28(3)). It has been shown that amyloid deposits also show green birefringence on Hematoxylen-Eosin slides with polarised microscopy. The study group was composed of 75 Congo-red confirmed amyloidosis and 25 Congo-red negative control cases bearing amorphous eosinophilic amyloid-mimicking areas. Study set was designed by one of the researchers and evaluated by others in a blinded fashion. Digital evaluation was done by Olympus BX51 polarising microscope equipped with DP21,SAL camera on the 20×.

**Results:** Thirty-seven(49,3 %) of 75 Congo-red positive biopsies, showed green/yellowish green birefringence by DR-HEP. In 20 of those amyloidosis was dubious by Hematoxylen-Eosin alone. In 4 of negative-evaluated cases, the Congo-red positive vessel was not present in DR-HEP studied sections. No false positivity were found among control group. Overall the sensitivity, specificity, positive and negative predictive values were 49.3 %, 100 %, 100 % and 39.6 % respectively.

**Conclusion:** Congo-red is the gold standard in amyloidosis diagnose however, DR-HEP technique can be used as a fast search method for diagnosis of amyloidosis with higher specificity and considerable sensitivity.

**PS-09-006**

**Immunoexpression of matrix metalloproteinase-9 in colon mucosa of patient with Crohn’s disease complicated by AA amyloidosis**

S. Skuja ^*^, T. Zake, V. Groma

^*^Riga Stradins University, Laboratory of EM, Latvia

**Objective:** Involvement of matrix metalloproteinases (MMPs) in the bowel architecture remodeling in Crohn’s disease (CD) was emphasized. Neutrophils are the most important source of MMP9 in the acute phase of intramural inflammation. There is a marked increase in mucosal plasma cells in CD, although their precise role and ability to express MMPs are not well recognized.

**Method:** We aimed to range damage in the colonic mucosa and in CD complicated by AA amyloidosis with renal and gastrointestinal tract involvement, and estimate immunohistochemical expression of MMP9.

**Results:** The lesions found in the colonic mucosa and submucosa were compatible with CD: crypt alteration and destruction, lymphoplasmacytic infiltration, occasional occurrence of neutrophils and eosinophils, adipose tissue accumulation, granuloma formation, and vascular changes. The basal aspects of mucosa crypts and vessels showed Congo red positivity. MMP9 immunopositivity was observed in epithelial cells, fibroblasts and inflammatory cells including neutrophils and macrophages. MMP9 was expressed by most plasma cells, which number was notably increased. Vascular beds showed low staining intensity.

**Conclusion:** Supporting the role of MMPs in tissue remodeling in CD, MMP9 was expressed by variety of connective tissue and inflammatory cells. Intense MMP9 immunostaining demonstrated in plasma cells suggests their possible role in tissue destruction in CD.

**PS-09-007**

**Common variable immunodeficiency is characterized by inflammatory bowel disease-like presentation**

N. Comunoglu ^*^, N. Kepil, S. Kara, H. Aki, S. Erdamar Çetin, S. Dervisoglu

^*^Cerrahpasa Med1ial Faculty, Pathology, Istanbul, Turkey

**Objective:** Common variable immunodeficiency patients come with recurrent respiratory and gastrointestinal system infections. We present this case because of its inflammatory bowel disease-like presentation.

**Method:** 11 years old male person has been followed up with the diagnosis of CVID since he was 6 months old. A biopsy has been practiced after observing Crohn-like changes in colonoscopy, which is done because of abdominal pain and diarrhea complaints. Microscopy showed common epithelial damage, apoptotic bodies, crypt distortion, cyriptitis, severe decrease of plasma cells in the lamina propria and absence of plasma cells in some areas. Patient has been taken into operation after observing free air in abdominal graphy which is performed because of patient’s abdominal pain, vomiting and bloody diarrhea after 3 months. Macroscopy showed multiple perforation areas, superficial and deep ulcers in the 40 cm of small intestine resection material. Microscopy showed active enteritis with common aphthous ulcers, purulent peritonitis, a decrease in plasma cells and loss of primary follicules in lymph nodes.

**Results:** This has been described as inflammatory bowel disease-like CVID.

**Conclusion:** CVID disease can show variant histological patterns in gastrointestinal system. Some of these are lymphocytic colitis, collagenous colitis, Celiac disease, lymphocytic gastritis, granulomatous disease of the intestine, acute GVHD and inflammatory bowel disease.

**PS-09-008**

**Vascular expression CD34, CD31 (PECAM-1) in Inflammatory Bowel Disease (IBD) and Irritable Bowel Syndrome (IBS)**

A. Botina ^*^, O. Schykina, E. Kondrashina

^*^St. Petersburg Pavlov State, Medical University, Dept. of Pathology, Russia

**Objective:** To investigate of vessels in the intestinal mucosa from patients with IBD and IBS by immunohistochemistry.

**Method:** The subjects were 20 patients with IBD (10 with UC, 10 with Crohn’s disease) and 10 patients with IBS. Specimens from ileum (30) and colon (30) was taken by ileocolonofiberscopy. After dewaxing paraffin sections, specimens stained by immunohistochemistry with antibodies CD 31(PECAM-1, BioGenex) and CD 34 (BioGenex). The program VideoTesT 5.0 were used for analisis of CD31 positive vessels and ocular nest of Avtandilov–for CD34 positive vessels.

**Results:** In IBS group specific area of CD34 positive vessels in the small intestine was 12 error 1 %, in IBD group 15 error 2 %; in the colon mucosa–10 error 1 % and 14 error 2 %, respectively. In the small intestine in IBS area of CD31 positive vessels was 0.44 error 0,1 μm square (MS), in IBD–0,57 error 0,1 MS; in the colon mucosa area of CD31 positive vessels was–0,51 error 0.1 MS and 0,71 error 0,08 MS, respectively.

**Conclusion:** Thus, the tendency of increase of CD34, CD31 positive vessels, in IBD group was revealed. These findings suggested that the vessels change play a useful role in pathogenesis of chronic inflammation in patients with IBD.

**PS-09-009**

**Comparison of histological activity in patients with ulcerative colitis in clinical remission with versus without elevated fecal calprotectin**

A. Botina ^*^, O. Shchukina, E. Kondrashina, E. Markova, O. Orlov

^*^St. Petersburg Pavlov State, Medical University, Dept. of Pathology, Russia

**Objective:** Fecal Calprotectin (FC) assessed in stool samples of pts with UC is proved to be a good non-invasive marker of endoscopic activity. The aim of our study was to analyze histological activity in pts with UC in clinical remission with vs. without elevated FC.

**Method:** 19 pts with UC in clinical remission were included. FC was assessed by immunoassay method ELISA. 15 pts with normal FC level (<50 mkg/g) comprised group I, 4 pts with elevated FC (≥50 mkg/g)–group II. All pts were undergone total colonoscopy with biopsy. Cryptitis, crypt-abscesses, neutrophils, thickening of mucosal infiltrate, basal plasmocytosis were considered criteria of histological activity of UC. Absence of those criteria indicated of histological remission.

**Results:** Evidences of histological remission were found in 13 pts (92.9 %) vs. only in 1 pt (25.0 %) of group I and II respectively. Moreover, thickening of mucosal infiltrate, erosions and ulcers were detected more frequently in the samples of group II than in group I: 75.0 % and 7.0 % respectively.

**Conclusion:** Thus, FC should be considered as a reliable marker of histological activity in UC. Prospective controlled studies are needed to evaluate how this correlation can be utilized in clinical practice and whether FC level can guide treatment decision.

**PS-09-010**

**Apoptosis and proliferation in mycophenolate mofetil-induced colitis and inflammatory bowel disease**

I. Delladetsima ^*^, G. Liapis, J. Boletis, G. Bamias, K. Tsimaratou, E. Patsouris

^*^Medical School Athens, 1st Dept. of Pathology, Greece

**Objective:** Comparative analysis of crypt epithelium apoptosis and proliferation in mycophenolate mofetil (MMF)-induced colitis and active inflammatory bowel disease (IBD).

**Method:** The examined material consists of 29 colonic biopsies from renal transplant patients with MMF administration and persistent diarrhea, 10 cases with Crohn disease and 10 cases with ulcerative colitis. Apoptosis was examined by TUNEL-assay and proliferation by immunohistochemical detection of Ki-67. Apoptotic index (AI) and proliferation rate (PR) were defined as the absolute number of apoptotic and Ki-67 positive epithelial cells/100 crypts.

**Results:** In MMF-induced colitis the mean AI was 15.7 (range:2–30, median:15). Low AI (1–10) was found in 10, intermediate (11–20) in 9 and high (21–30) in 10 cases. In Crohn disease and ulcerative colitis the mean AIs [3.3 (range:1–8, median:3) and 3 (range 1–4, median:3)] were significantly lower compared with MMF-induced colitis (*p* < 0.01). No statistical differences were found regarding PR. Expansion of Ki-67 to mid and upper third of the crypts was noted in 22/29 cases of MMF-induced colitis in contrast to 8/20 cases of IBD (*p* < 0.05).

**Conclusion:** Apoptotic cell death seems to be a potential pathogenetic factor of mucosa injury in MMF-induced colitis and provides a tool in the differential diagnosis from IBD.

**PS-09-011**

**Colon mucosal schwann cell hamartoma with tactoid features**

F. Beça ^*^, J. Lopes, J. M. Lopes, F. Carneiro

^*^Porto, Portugal

**Objective:** Mesenchymal colorectal polyps are uncommon or rare, particularly those of neurogenic origin, and may occur in inherited syndromes. We describe a mucosal schwann cell hamartoma of the colon with tactoid features, so far only reported in peripheral nerve sheath tumors, and address its differential diagnosis and clinical implications.

**Method:** A 72-year-old male, without relevant medical history, underwent screening colonoscopy that displayed a 5 mm polypoid colon lesion submitted to histological and immunohistochemical evaluation.

**Results:** A poorly circumscribed lesion was observed in the lamina propria, comprising small round to oval structures with tactoid features, and uniform bland spindle cells with wavy nuclei, eosinophilic cytoplasm with indistinct borders, entrapping adjacent colon crypts. No ganglion cells were seen. The spindle cells displayed strong and diffuse expression of S-100 protein and vimentin, without expression of EMA, synaptophysin, CD34, CD117, neurofilaments, and smooth muscle actin.

**Conclusion:** Mucosal schwann cell hamartoma was recently recognized as a benign lesion distinct from common (submucosa) colorectal schwannoma, so far not associated to inherited syndromes. Thus it should be considered in the differential diagnosis of look-alike lesions (e.g., ganglioneuroma, neuroma and neurofibroma) which may occur in the setting of inherited syndromes such as Cowden syndrome, MEN 2B and NF1.

**PS-09-012**

**Neuromuscular and vascular hamartoma of small bowel: Report of two cases**

M. J. Martín Osuna ^*^, P. Florez Rial, M. C. Jironda Gallegos

^*^Hospital Carlos Haya, Malaga, Spain

**Objective:** Small bowel NMVH is an extremely rare disease. It consists of a mixture of disorganized intestinal tissue (smooth muscle bundles from the muscularis mucosa, unmyelinated nerve fibers, ganglion cells and angiomatous vessels). Macroscopic examination shows annular type intestinal strictures while the adjacent intestine appears normal. Hamartomatous’ nature has been questioned by many authors since identical characteristics can be identified in Crohn’s disease, ischemic enteritis, radiation enteritis and intestinal diaphragmatic disease related to NSAIDs.

**Method:** We report two cases of small bowel NMVH. Both patients had a history of recurrent pseudo-occlusive intestinal episodes, not related to taking NSAIDs and with favorable response to surgical treatment.

**Results:** First case: A young male with persistent weight loss. Pathological examination revealed annular strictures unevenly distributed throughout the intestinal segment, reducing light, ulcerating the mucosa and thickening the intestinal wall. Second case: An elderly woman with rectal bleeding. The intestinal mucosa was ulcerated and displayed cobblestone-looking areas.

**Conclusion:** We reviewed the limited literature on this entity. Based on the two cases described, we conclude that the HNMV can be considered an entity once Crohn’s disease and intake of NSAIDs have been ruled out.

**PS-09-013**

**One more morphological pattern of gastrointestinal neuroectodermal tumor**

B. Kokoskova ^*^, E. Kvicova, P. Grossmann, M. Michal, O. Daum

^*^Plzen, Czech Republic

**Objective:** To demonstrate a hitherto undescribed morphological pattern of EWSR1-rearranged gastrointestinal neuroectodermal tumor.

**Method:** A 60-years-old woman underwent surgery for intestinal obstruction. Tissue for light microscopy was fixed in 4 % formaldehyde and embedded in paraffin using routine procedures. FISH analysis was performed to detect possible rearrangements of EWSR1 gene.

**Results:** In the small intestine there was a perforation measuring 1,5 cm in diameter. Histologically, the wall of the intestinal perforation was infiltrated by diffusely growing tumor composed of round cells of “blastic” appearance. Imunohistochemically, the tumor was positive for vimentin and CD34, whereas results of staining for cytokeratins (AE1/3, CAM5.2), chloracetatesterase, S-100, HMB45, chromogranin, synaptophysin, CD20, CD30, desmin, NANOG, SALL4, CD31, CD117, CEA, CDX2, DOG1, EMA). FISH demonstrated a rearrangement of gene EWSR1.

**Conclusion:** We report on a case of malignant tumor with blastic features, “undifferentiated” immunophenotype, and rearrangement of EWSR1 gene. This rearrangement, in combination with intestinal origin, raises suspicion on a relationship of this tumor with a novel diagnostic category named “gastrointestinal neuroectodermal tumor”, formerly known as clear cell sarcoma of the gastrointestinal tract, which was found to display much broader spectrum of morphological patterns than the classical clear-cell morphology.

**PS-09-014**

**Primitive neurectodermal tumors: A case of extraosseous Ewing’s sarcoma of the small intestine**

A. Zehani-Kassar ^*^, I. Chelly, H. Azouz, H. Nfoussi, B. Chelly, k. Bellil, S. Haouet, N. Kchir

^*^La Rabta hospital, Pathology, Tunis, Tunisia

**Objective:** Ewing’s sarcoma/primitive neuroectodermal tumour (ES/PNET) is a rare, small, round cell malignancy with a specific (11;22) translocation. ES/PNET of the small intestine is extremely rare, only five cases have been reported.

**Method:** We report an exceptional case of Ewing’s sarcoma of the small intestine in a young adult.

**Results:** A 40-year-old man complained for severe abdominal pain. Computed tomography scan revealed a large mass located in the pelvic cavity. Segmental resection of the small intestine and omentectomy were performed. Macroscopically, a tumor measuring 10 cm was located in the mesentery of the ileum. The ileal wall was involved directly by the tumor. Microscopically, the lesion consisted of sheets of undifferentiated small round cells with uniform vesicular nuclei and scanty cytoplasms. Immunohistochemical studies revealed that tumor cells were positive for CD99 but negative for cytokeratin, leukocyte common antigen, synaptophysine, chromogranin, desmin, S-100 and MDM2. Fluorescence hybridization study revealed Ewing sarcoma breakpoint region 1 (EWSR1) gene rearrangement on chromosome 22q12.

**Conclusion:** Other small round cell tumors, including malignant lymphoma, desmoplastic small round cell tumor, undifferentiated carcinoma and small cell carcinoma, offer a differential diagnosis of the current lesion being discussed. Through histological, immunohistochemical and molecular methods, the lesion was meticulously examined to maintain distinction.

**PS-09-015**

**Neuroendocrine carcinoma in adenoma of the sigmoid**

T. Koletsa ^*^, S. Mavropoulou, E. Beretouli, T. Tziola, G. Karkavelas, G. Karayannopoulou

^*^Medical School AUTH, Dept. of Pathology, Thessaloniki, Greece

**Objective:** Neuroendocrine tumor in an adenoma is rare with only limited reported cases, so far.

**Method:** An 84 year-old woman underwent surgical removal of a tumor located in sigmoid, diagnosed as adenocarcinoma on previous biopsy. On gross examination, apart from the ulcerated tumor corresponding to adenocarcinoma, a polyp measured 2 cm in greatest diameter was also observed.

**Results:** Histologic examination of the polyp revealed the presence of a villotubular adenoma. In two sites of the adenoma solid nests of smaller cells with small amount of cytoplasm, round nuclei with finely stippled chromatin were observed. These cells located in the lamina propria and muscularis mucosa, without disturbing the polyp architecture, and showed the following immunophenotype: CK 8/18+, NSE+, chromogranin++/−, CK19-−/+, synaptophysin-−/+. Mitotic index Ki67/mib1 was >30 %. The histologic findings set the diagnosis of mixed adenoma and neuroendocrine carcinoma (NEC, G3) having a collision architecture pattern. Both adenoma and neuroendocrine tumor were MSH2, MSH6, MLH1 and PMS2 positive. The patient remained free of reccurence or metastasis 2 years ago.

**Conclusion:** The recognition of NEC in adenoma will help to avoid potential diagnostic pitfalls. Mixed benign adenoma-NEC is rare with uncertain histogenesis and biological behavior.

**PS-09-016**

**Multifocal small cell neuroendocrine carcinoma of jejuno-ileal with coeliac disease mimicking Burkitt’s lymphoma: A case report**

H. Belkralladi ^*^, K. T. Douidi, Y. Attar, F. Kermas, A. Tou

^*^Djilali Liabes University, Pathology, Sidi Bel Abbes, Algeria

Intestinal endocrine tumors are rare representing only 2 % of malignant tumors. Coeliac disease is common. The association of neuroendocrine carcinoma with coeliac disease is extremely rare. Although the significance of this association is unclear, to our knowledge, only a few cases of neuroendocrine carcinoma associated with coeliac disease has been described. We report a rare case of mutifocal small cell neuroendocrine carcinoma of jejuno-ileal in a 30 years old woman with coaliac disease who had on gluten free diet. The patient has been admitted for an acute small bowel obstruction. At emercengy laparotomy, a three tumors of the small bowel involving the jejunum and ileum. Histopathology, the tumor appeared similar and showed a pooly differenciation neoplasm. The tumor cells were arranged in solid sheets and trabecular pattern that infiltrated through the bowel wall to the serosal surface. The immunohistochemistry showed an intense reactivity to NSE and CK, focal positivity to chromogranin but not to CD3, CD10, CD20 and Bcl2. The aim of our study was to report on the clinical, histopathological, immunohistochemical characteristics and differential dignosis of this rare association.

**PS-09-018**

**Appendiceal mucinous neoplasms of low malignant potential: Clinicopathological features of five cases**

E. Kairi-Vasilatou ^*^, C. Dastamani, A. Melloy, A. Tsagkas, A. Kondi-Pafiti

^*^Aretaieion University Hospital, Dept. of Pathology, Athens, Greece

**Objective:** To investigate the clinicopathological features of mucinous neoplasms of low malignant potential of the appendix (MN-LMP).

**Method:** Five cases of appendiceal MN-LMP were diagnosed in our laboratory during a 6-year period, in a total of 193 appendectomy specimens examined (2,5 %).

**Results:** Patients’ age was 36–71 years (mean age 57 years). Four of them were female (80 %) and one was male (20 %). In 4/5 cases, the patients presented with an appendiceal tumor, whereas in 1/5 case the patient presented with clinical symptoms of acute appendicitis. The length of the appendix ranged from 6 to 8,5 cm (mean length 7.36 cm) and its maximum diameter measured from 0,7 to 4,7 cm (mean diameter 3 cm). On section, the appendiceal lumen of all specimens was filled with white to yellow mucinous material. In one case (20 %), there was a synchronous ovarian mucinous tumor, which was regarded as a metastasis from the appendiceal mucinous tumor. In all cases, no mucin deposits where identified to the peritoneum.

**Conclusion:** Appendiceal MN are considered tumors of upredictable biologic potential, which can potentially spread to the peritoneum and viscera in the form of gelatinous mucin deposits (pseudomyxoma peritonei) and should be sampled thoroughly to rule out malignancy.

**PS-09-019**

**COX-2 expression in benign serrated polyps of the colon**

M. Kiedrowski ^*^, A. Mroz, J. Orlowska, E. Kraszewska, A. Rembiszewska, A. Felisiak-Golabek

^*^Center of Oncology, Pathology, Warsaw, Poland

**Objective:** To assess and compare expression of cyclooxygenase-2 (COX-2) in benign serrated polyps of the large bowel.

**Method:** 119 serrated polyps of the large bowel removed at the Department of Gastroenterology and Hepatology during standard polipectomy were analyzed. There were 17 traditional serrated adenomas (TSA), 83 hyperplastic polyps (HP) and 19 sessile serrated polyps (SSP), according to WHO 2010 classification. The COX-2 expression was assessed semi-quantitatively (0–2) and each lesion was fully characterized in terms of anatomical location, size, histology, age and sex of patients. The multiple logistic regression model was used to asses the differences between the groups.

**Results:** The epithelial expression of COX-2 was found in 85/119 serrated polyps (71,43 %): 57/83 (68,67 %) HP, 12/17 (70,59 %) TSA and 16/19 (84,21 %) SSP. In HP and SSP it was predominantly of weak (49/83 HP, 12/19 SSP), whereas in TSA it was mainly of medium/strong intensity (8/17). The TSA category associated with more frequent COX-2 expression (OR = 7.00, 95 % CI 1.49–32.88, *p* = 0.014) than HP.

**Conclusion:** COX-2 expression was more prevalent in TSA than other serrated benign polyps. COX-2 staining could potentially serve as a marker in the differentiating serrated lesions of the colon.

**PS-09-020**

**Inter-observer agreement in histological assessment of dysplasia degree in conventional colorectal adenomas**

M. K. M’Farrej^*^, S. Rammeh, N. Sabbegh Znaidi, I. Smichi, W. Ajjouli, N. Kourda, F. Fareh, R. Zermani

^*^Charles Nicolle Hospital, Pathology, Tunis, Tunisia

**Objective:** Assessment of inter-observer reproductibility (IR) in determining the degree of dysplasia (DD) in conventional colorectal adenomas.

**Method:** Slides providing from 240 colorectal conventional adenomas, collected in our service over a period of 5 years (2007–2012) were reviewed separately by 2 pathologists. IR assessment was evaluated for the determination of DD. For this, statistical analysis was performed applying Kappa test (Κ).

**Results:** According to DD of adenoma, the first pathologist diagnosed 189 cases (78.8 %) as low grade dysplasia adenomas, and 51 cases (21.2 %) as high grade dysplasia. These DD features were reported by the second observer respectively in the proportions of 191 cases (79.6 %), and 49 cases (20.4 %). Thus, IR in assessing DD was evaluated as moderate (Κ = 0.55).

**Conclusion:** In addition to its biological importance, the value of a grading system depends on its reproductibility. Despite the adoption of enough relevant histological diagnostic elements, microscopic assessment of colorectal adenomas seems to need more efficient and accurate criteria for improving inter-observer reproductibility.

**PS-09-021**

**The serrated pathway: Systematic histological study and BRAF mutational analysis of precursor lesions**

L. Serrano ^*^, E. Musulen, C. Sanz, M. Lopez, R. Marginet-Flinch, L. Perez-Roca, D. Lopez-Alvarez, E. Martinez-Balibrea, A. Ariza

^*^Hospital Germans Trias i Pujol, Dept. of Pathology, Badalona, Spain

**Objective:** The serrated pathway accounts for about 90 % of sporadic microsatellite instability-high (MSI-H) colorectal carcinomas (CRCs) of the right colon (RC). Better knowledge of this pathway’s precursor lesions (PLs) is mandatory to understand the pathogenic mechanisms involved.

**Method:** MSI-H/MLH-1(−) RC CRCs were selected (Lynch syndrome and serrated polyposis were exclusion criteria), with microsatellite-stable (MSS) RC CRCs serving as controls. PL occurrence and features and BRAF mutational status were assessed in all cases and controls, which were classified according to BRAF status (BRAF-V600E vs BRAF-wild type).

**Results:** We identified 46 MSS and 65 MSI-H/MLH-1(−) RC CRCs. Of the latter 65 cases, 34 showed BRAF-WT and 31 showed BRAF-V600E. Eleven PLs were identified. Of the 7 PLs in the BRAF-V600E CRC group, 5 (2 adenomatous PLs, 3 serrated PLs) showed BRAF-V600E, while of the 4 PLs in the BRAF-WT CRC group, 3 showed BRAF-WT.

**Conclusion:** BRAF-V600E mutation is characteristic of MSI-H/MLH-1(−) RC CRCs and their PLs, which commonly show a serrated pattern. However, in some BRAF-V600E PLs the serrated pattern would be obscured by high-degree dysplastic changes giving rise to an adenomatous pattern.

**PS-09-022**

**Dome-type adenocarcinoma of the colon: A case report and review of the literature**

H. Kannuna ^*^, P. Caseiro Silverio, M. Girardin, L. Rubbia-Brandt, G. Puppa

^*^University Hospitals of Geneva, Switzerland

**Objective:** Presenting an additional case of dome type carcinoma of the colon (DC) to the 11 that have been already reported. This particular tumor is thought to derive from the specialized M-cells of dome epithelium, the epithelial component of the gut-associated lymphoid tissue, therefore its characteristic association with a prominent lymphoid stroma.

**Method:** The case is presented with clinical, endoscopic, histopathological and immunohistochemical features; a literature review is performed.

**Results:** A 57 years old female with no family history of colorectal cancer was hospitalized because of fever and abdominal pain. The computer tomography detected a cecal mass and endoscopy showed a large sessile polyp; a right hemicoloectomy was then performed. Histopathological examination showed a moderately differentiated adenocarcinoma developed in a tubulo-villous adenoma, invading the submucosa, in association with prominent lymphoid tissue. Eleven lymphonodes recovered were negative. Immumohistochemistry showed retained expression of the mismatch repair proteins. One year after the patient is recurrence free.

**Conclusion:** We report a case of DC of the colon sharing similar histopathological, immunohistochemical, staging and prognostic features with those already reported. This is further evidence that this histotype deserves being integrated into the classification of tumors of the colon and rectum.

**PS-09-023**

**The prognostic implications of percentage of tumor cells contacting surrounding stroma in mucinous colorectal carcinomas**

G. Akturk ^*^, S. Sokmen, H. Ellidokuz, A. E. Canda, M. Unlu, A. H. Sirin, O. Sagol, C. Terzi, M. Fuzun, S. Sarioglu

^*^Dokuz Eylul University, Dept. of Pathology, Izmir, Turkey

**Objective:** Colorectal mucinous carcinomas (MC) have a poor prognosis. The relationship of the cells to the stroma and extracellular mucin [percentage of tumour cells contacting surrounding stroma (PTCCSS)] was identified as a prognostic marker for salivary gland carcinomas. We evaluated the prognostic value of this feature in colorectal MC.

**Method:** In a series of 59 colorectal MC images from H&E stained tumor sections were captured by a camera. A grid with 140 points was laid over the computer screen. Points, falling on tumor cells floating in mucin and on cells contacting surrounding stroma were counted separately till a total count of 100 cells was achieved and the PTCCSS were analyzed statistically along with clinicopathological prognostic parameters.

**Results:** The median for PTCCSS was 70 % and 30 cases had lower values than this. There was significant difference between cases with higher and lower PTCCSS values for overall survival (*p* = 0.048). For the first group, 67,6 % of the patients were alive at first and 63,4 % at the second year, while this was 66,2 % % and 33,5 % for the other group.

**Conclusion:** In contrast to the salivary gland mucinous tumors, in colorectal MC, higher PTCCSS seems to be a poor prognostic marker.

**PS-09-024**

**Possible link between cancer stem cell like phenotype and apoptotic resistance in stage III and IV colorectal cancer**

B. Kleist ^*^, V. Seel, C. Kersten, C. Loland, M. Poetsch

^*^Soerlandet Sykehus HF, Dept. of Pathology, Kristiansand, Norway

**Objective:** Evidence and alterations of cancer stem cell (CSC) characteristics and their impact on key functions in colorectal carcinogenesis are still poorly understood. This study investigated genetic variation and expression of the putative CSC marker Lgr5 and its relation to apoptotic resistance and proliferation.

**Method:** Twenty-three single nucleotide polymorphisms (SNPs) of the Lgr5 gene and immunohistochemical expression of Lgr5, apoptosis inhibitor Bcl-2 and proliferation marker Ki-67 were analyzed in 88 stage III and IV colorectal carcinomas (CRC).

**Results:** Immunohistochemical Lgr5 expression was higher in CRC with the wild type Lgr5 genotype (*n* = 25; mean 8.94 %, median 6.1 %) compared to tumors with variant Lgr5 genotype (*n* = 63; mean 5.79 %, median 2.9 %). The CRC group with ≥15 % Lgr5 expression in the investigated tumor tissue harbored cases with the highest mean Bcl-2 expression (29.8 %), which was more than twice as high as in the group with 0–4 % Lgr5 expression (13.5 %). CRCs with ≥15 % Lgr5 expression showed also lower Ki-67 indices and higher percentages of non-responders to chemotherapy compared to the other groups.

**Conclusion:** Increasing evidence of a CSC like phenotype marked by Lgr5 is probably related to the wild type Lgr5 genotype and might be associated with apoptotic resistance of stage III and IV CRC.

**PS-09-025**

**Colorectal cancer: Morphological changes induced by neoadjuvant chemotherapy**

E. Lushnikov ^*^, A. Abrosimov, A. Nevolskikh

^*^MRRC, Dept. of Pathology, Obninsk Kaluga Oblast, Russia

**Objective:** Morphological study of tumors shows discrepancies in assessment of treatment effectiveness. Grades of tumor regression are variable by different authors and WHO criteria. These criteria are needed to be agreed.

**Method:** Colorectal cancer morphology was studied in two groups of patients: treated by irradiation and chemotherapy with 5-ftoruracil (56), by irradiation and chemotherapy with Xeloda (45). Mean age of patients in two groups was 58.5 and 57.3 year respectively. Moderate differentiated adenocarcinoma was diagnosed in most cases. Patients were operated 42–54 days after preoperative therapy.

**Results:** Similar morphological changes were observed: necrosis and apoptosis of tumor cells, inflammation, angiomatosis and stromal fibrosis. Grade 2 and Grade 3 of tumor regression were predominant in group 1 (36.5 % and 26.3 %) and in group 2 (57.8 % and 60.5 %). No remaining tumors diagnosed in one patient (group 1) and in 5 patients (group 2). Difficulties in assignment of Grades 2 and 3 allowed to combine them.

**Conclusion:** Neoadjuvant therapy of colorectal cancer induced regressive, inflammatory and fibrotic changes in tumors. Tumor changes depend on many factors and three grades of tumor regression could be proposed: no morphological changes in tumor; tumor has changed morphology; no remaining tumor diagnosed.

**PS-09-027**

**Grading of rectal carcinoma by quantifiying poorly differentiated cell clusters in endoscopic biopsy is predictive of nodal involvement and pTNM stage**

V. Barresi ^*^, R. Cardia, G. Branca, G. Tuccari

^*^Policlinico G. Martino Pad D, Dip. Patologia Umana, Messina, Italy

**Objective:** To evaluate the reliability and predictive value on nodal involvement and pTNM stage of a novel pre-surgical grading assessed by the count of cancer cell clusters composed of ≥5 cancer cells and lacking a gland-like structure (poorly differentiated clusters: PDC) in rectal carcinoma biopsies.

**Method:** Grading by quantifying PDC was assessed in 55 endoscopic rectal carcinoma biopsies and in the corresponding resection specimens. Then, the statistical correlation between the number of PDC in the biopsy and in the corresponding surgical specimen and those between PDC grade and nodal involvement (N+), number of involved nodes or pTNM stage were investigated.

**Results:** A significant positive correlation between the number of PDC in the biopsy and in the resection specimen was evidenced. Furthermore, the histological grade by quantifying PDC was significantly correlated with nodal involvement, number of involved nodes and pTNM stage of the surgical specimen.

**Conclusion:** Histological grading based on the count of PDC is feasible on endoscopic biopsy and it displays a good concordance with grading assessed on the surgical specimen. If validated, its use in routinary practice may provide significant pre-operative information on nodal involvement and pTNM stage, which may be taken into account in establishing the surgical management of this tumour.

**PS-09-028**

**Expression profile of maspin in colorectal cancer**

S. Gurzu ^*^, Z. Szentirmay, I. Jung

^*^University of Medicine and Pharmacy, Dept. of Pathology, Tirgu-Mures, Romania

**Objective:** To assess the possible prognostic value of the antiangiogenic and proapoptotic serine protease Maspin in colorectal carcinomas (CRC).

**Method:** The immunohistochemical Maspin expression was analyzed in 150 CRC, surgical specimens. It was correlated with the tumor stage, survival rate, tumor budding, p53 expression, microsatellite status and the intensity of angiogenesis evaluated with the antibodies VEGF-A, CD31, and CD105. Based on the percentage and intensity of Maspin expression in the tumor cells, the cases were grouped in four classes: negative, with cytoplasmic predominance, nuclear predominance, and cases with mixed (cytoplasmic-nuclear) expression.

**Results:** From the 150 CRC, 44 % of cases presented cytoplasmic predominance, 24 % showed a nuclear predominance, 23 % had mixed positivity and 9 % did not express Maspin in the tumor cells. The cytoplasmic predominance was correlated with p53 negativity, low rate of tumor budding and longer survival rate. The mixed Maspin expression was characteristic for MSI-cases with low intensity of angiogenesis. Maspin nuclear predominance was especially observed in VEGF-positive cases diagnosed in pT4 stage.

**Conclusion:** Evaluation of Maspin expression in CRC should take into account both nuclear and cytoplasmic pattern. The cytoplasmic predominance and mixed Maspin expression seems to associate parameters that indicate a better prognosis whereas nuclear predominance indicates high aggressivity.

**PS-09-030**

**Immunohistochemical evaluation of MMR proteins expression in colon adenocarcinomas: A laboratory’s experience**

C. Poulios ^*^, G. Raptou, E. Vrettou

^*^AUTH Medical School, Dept. of Pathology, Thessaloniki, Greece

**Objective:** Mismatch Repair (MMR) genes play a significant role in the repair of point mutations during DNA replication. The aim of this study was to evaluate the expression of MMR proteins in colorectal cancer in comparison with tumor histological features, sites of involvement and patient’s age.

**Method:** 94 specimens from patients with colorectal cancer stage II–III were examined for MMR protein expression. Sections were stained with monoclonal antibodies: MSH2(25D12), MSH6(PU29), MLH1(ES05) and PMS2(MOR4G).

**Results:** In 23 out of the 94 cases absence of at least one MMR protein nuclear expression was observed. These 23 cases regarded 13 men and 10 women with an average age of 65 years. Most patients (19/23) had carcinoma of the right colon. 16 cases regarded the simultaneous absence of MLH1 and PMS2 proteins expression and 3 cases the absence of both MSH2 and MSH6 protein expression. In 4 cases various combinations of expression absence were observed. The rest 71 cases showed positive expression for all MMR proteins.

**Conclusion:** Immunohistochemistry although may not replace MSI testing, owing to its simplicity and availability, should be as one of the first line screening measures in the evaluation of MMR genes status.

**PS-09-031**

**Adenocarcinomas with Microsatellite Instability (MSI)–correspondence between IHC and PCR examination**

J. Nemecková^*^, I. Kaspercík, T. Prikryl, M. Nemecek, R. Andelová, L. Kudelka, A. Bóday, S. Tavandzis, K. Horka, J. Rychnovsky

^*^Laboratore AGEL a.s., Pathology, Czech Republic, Czech Republic

**Objective:** Microsatellite instability (MSI) is a condition which occurs due to defects in the repair system in gene defects, coding repair mismatch proteins that modify the length of microsatellites (i.e. sequence of repeating nucleotide units) during DNA replication. Around 15 % of sporadic colorectal adenocarcinomas and almost all colorectal adenocarcinomas in HNPCC are formed on an MSI basis. Adenocarcinomas with this defect manifest a different response to chemotherapeutic treatment.

**Method:** We examined 208 colorectal adenocarcinomas in patients without gender, age or illness stage restrictions. These adenocarcinomas were subject to immunohistochemical examination for the evidence of expression of protein products in MLH1, PMS2, MSH6 and MSH2 genes and subsequently to molecular genetic examination, which determined, using the polymerase chain reaction (PCR), the state of microsatellite stability (MSS) or microsatellite instability (MSI) and MLH1 methylation status of the tumour.

**Results:** From the total number of 208 adenocarcinomas, 7,9 % manifested MSI according to IHC, molecular genetic examination revealed 10,8 % MSI carcinomas. Correspondence between IHC and PCR was found in 73,1 %.

**Conclusion:** Due to a different response of microsatellite unstable colorectal adenocarcinomas to chemotherapeutic treatment, it is necessary to separate patients with these tumours from patients with microsatellite stable tumours. Immunohistochemical analysis seems to be a fast and relatively cheap screening method suitable for this purpose.

**PS-09-032**

**Double immunostaining for all four mismatch repair proteins: An easy screening method for microsatellite instability in colorectal cancer**

A. Aline-Fardin ^*^, A. Scriva, J.-F. Fléjou

^*^Hôpital Saint-Antoine, Service d’Anatomie Pathologiqu, Paris, France

**Objective:** Microsatellite Instability (MSI) in colorectal cancer (CRC) can be diagnosed by PCR or by immunohistochemistry (IHC). The sequence and the number of antibodies against MisMatch Repair (MMR) proteins are discussed. We aimed to evaluate MSI in CRC by double immunostaining of four MMR proteins.

**Method:** We identified 30 CRCs who had at the time of surgery simple IHC (MLH1 and MSH2) and PCR for MSI. Ten consecutive cases were studied in 3 categories: MLH1+/MSH2+ (9 MSS, 1 MSI), MLH1−/MSH2+ (all MSI), MLH1+/MSH2− (all MSI). Double IHC was performed: MLH1 (DAB chromogen)/MSH2 (Fast Red chromogen), and PMS2 (DAB chromogen)/MSH6 (Fast Red chromogen). Double blind analysis was made by two observers.

**Results:** MMR protein expression was correctly diagnosed by both observers in all cases. The normal mucosa served as positive control. In 29 cases, MLH1/MSH2 and PMS2/MSH6 pattern of expression was identical. The only MLH1+/MSH2+ MSI CRC, showed loss of PMS2, detected on the PMS2/MSH6 stained slide.

**Conclusion:** Double immunostaining is feasible to detect MSI in CRC. This method allows in a simple manner the study of all four MMR proteins.

**PS-09-033**

**Immunohistochemical detection of BRAF V600E mutation in colorectal cancer and its use to identify Lynch syndrome patients**

A. Ristimäki ^*^, A. Thiel, M. Heinonen, J. Kantonen, L. Lahtinen, J.-P. Mecklin, A. Gylling, P. Peltomäki

^*^Haartman Institute, Dept. of Pathology, Helsinki, Finland

**Objective:** Aim of the study was to detect BRAF V600E mutation using immunohistochemistry (IHC) in colorectal cancer (CRC) specimens, and to use this information to identify Lynch syndrome (LS) patients.

**Method:** Consecutive cases of primary CRC (*n* = 137) were analyzed for MLH1 protein expression using IHC and promoter methylation using MethyQESD and MS-MLPA. BRAF V600E mutation was detected by IHC (VE1 antibody) and by qPCR analysis. Selected cases were subjected to microsatellite instability assay and MLH1 gene sequencing.

**Results:** Of the 137 CRC specimens 18 were negative for MLH1 protein expression. These 18 cases were confirmed to be microsatellite instable (MSI-H). Loss of MLH1 expression was due to methylation of the MLH1 promoter in 15 cases. In the MSI-H group 14 cases (77.8 %) presented with a BRAF V600E mutation whereas in the microsatellite stable (MSS) group only 9 patients (7.6 %) were mutated. Two of the three MLH1 IHC negative, non-MLH1 methylated and wild-type BRAF cases were found to be Lynch syndrome patients by MLH1 sequencing. Finally, 11 previously confirmed LS cases served as a control group, and none of them had a BRAF V600E mutation.

**Conclusion:** In conclusion, detection of BRAF V600E mutation can be used as part of the algorithm to indentify LS patients.

**BRAF V600E IHC in CRC:** 
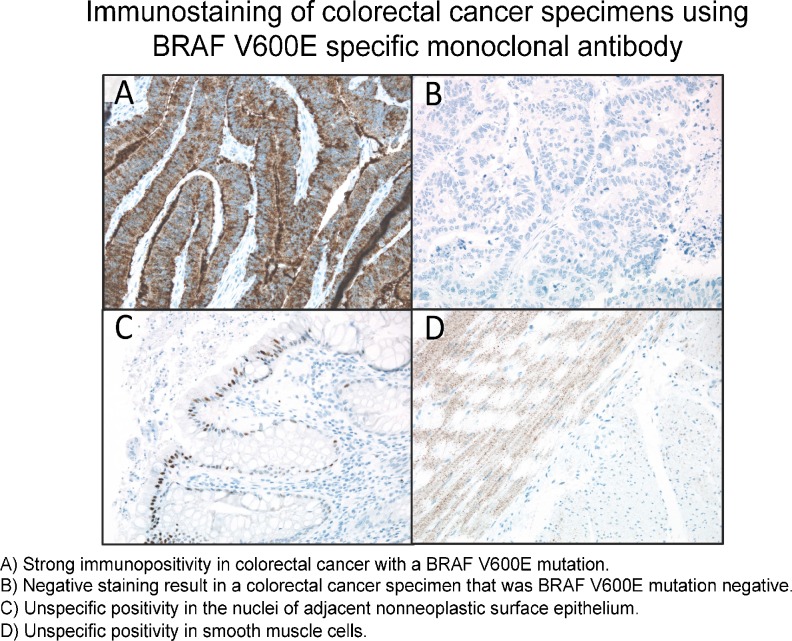


**PS-09-035**

**Comparison of CD133 and CD44 expression between microsatellite stable and microsatellite unstable colorectal cancers**

A. Aline-Fardin ^*^, A. Scriva, A. Duval, J.-F. Fléjou

^*^Hôpital Saint-Antoine, Service d’Anatomie Pathologiqu, Paris, France

**Objective:** Relation between stem cell (SC) marker expression and microsatellite instability (MSI) has not been studied in colorectal cancer (CRC). We aimed to compare the expression of two SC markers, CD133 and CD44, in a series of MSI and microsatellite stable (MSS) CRCs.

**Method:** Consecutive MSI CRCs (*n* = 102) were matched with 148 MSS CRCs (one or two MSS for each MSI, matched for gender, age, tumour localization and stage). CD133 and CD44 immunohistochemistry was performed. A score was established: 0 for ≤50 %, 1 for > 50 % expression on tumour cells. Double blind analysis was performed by two observers.

**Results:** 59 % of MSI CRCs and 41 % of MSS CRCs had a score 1 for CD44 (*p* = 0.0045). 73.5 % of MSI CRCs and 43 % of MSS CRCs had a score 0 for CD133 (*p* = 2.17E-6). Patients with score 1 CD44 had a better 5-year disease free survival than patients with score 0 (*p* = 0.026). There was no relation between CD133 expression and survival.

**Conclusion:** Differential expression of SC marker (CD133, CD44) could participate to the morphological and molecular characteristics of MSI CRCs.

**PS-09-036**

**DNA methylation in colorectal carcinomas**

M. Comanescu ^*^, M. Dobre, C. Ardeleanu

^*^INCD Victor Babes, Pathology, Bucuresti, Romania

**Objective:** DNA methylation alterations have been described as one of the most common consistent findings in human tumors, including colorectal carcinomas and both hypo- and hypermethylation have been described.

**Method:** Our study group included both formalin fixed parraffin embedded (FFPE) surgical specimens and blood. All cases of CRC had confirmed histopathological diagnosis. A basic immunohistochemical profile was performed on all FFPE cases. DNA methylation alterations were studied using the kit from Qiagen, Epitect Methyl II Pcr Array.

**Results:** We studied 16 cases of primary colorectal carcinomas and 3 metastasis with colorectal origin. For 11 cases peritumoral tissue was aslo included in the study and blood was obtained only from 6 of these patients. We used blood from 3 healthy persons as control group. The genes studied were APC, CDH1, CDKN2A, DKK2, DKK3, HIC1, HNF1B, HS3ST2, MGMT, MLH1, OPCML, PCDH10, RASSF1, RUNX3, SFRP1, SFRP2, SFRP5, SPARK, TMEFF2, UCHL1, WIF1, WT1 and different patterns of hypo- and hypermethylation were identified.

**Conclusion:** The study of DNA methylation in CRC is important as it could lead to the development of new biomarkers of prevention and detection by identyfing other genes that are aberrantly methylated in the initiation and progression of colon cancer. Acknowledgments PERSOTHER–SMIS-CSNR: 549/12.024.

**PS-09-037**

**Relationship between Indolamine 2, 3-DiOxygenase (IDO) activity and lymphatic invasion propensity of colorectal carcinoma**

I. Isik Gonul ^*^, A. Engin, B. Engin, A. Karamercan, A. Sepici Dincel, A. Dursun

^*^Gazi University Medical School, Dept. of Pathology, Ankara, Turkey

**Objective:** Aim of this study is to evaluate whether serum and tumor indoleamine 2, 3-dioxygenase (IDO) activities can predict the lymphatic invasion or lymph node metastasis in colorectal carcinoma.

**Method:** The study group was consisted of 44 colorectal carcinoma patients and 43 cancer-free subjects without any metabolic disturbances were included in the control group. Serum tryptophan (tryp) and kynurenine (kyn) concentrations of all patients were determined by high performance liquid chromatography and kyn/tryp was calculated to estimate the serum IDO activity. The tumor tissues of study group were re-examined for tumor grade, pathological stage, lymph node status, the presence of peri-intratumoral lymphocytic infiltrate and lymphatic invasion and were stained with IDO by immunohistochemistry.

**Results:** Serum kyn/tryp ratio was significantly higher in colorectal carcinoma patients compared to the control group (*p* = 0.006). Lymphovascular invasion (LVI) was present in 23 of 44 patients, of which only 12 patients had lymph node metastasis. 11 patients with lymphatic invasion had no lymph node metastasis. IDO expression correlated both with the presence of lymphatic invasion and lymph node metastasis (p: 0,014; p: 0,043 respectively).

**Conclusion:** IDO expression may predict the presence of an unrecognized LVI and lymph node metastasis and may be included in the histopathological evaluation of colorectal carcinoma cases.

**Strong IDO expression in well-differentiated adenocarcinoma of colon, streptavidine-biotin peroxidaseX200:** 
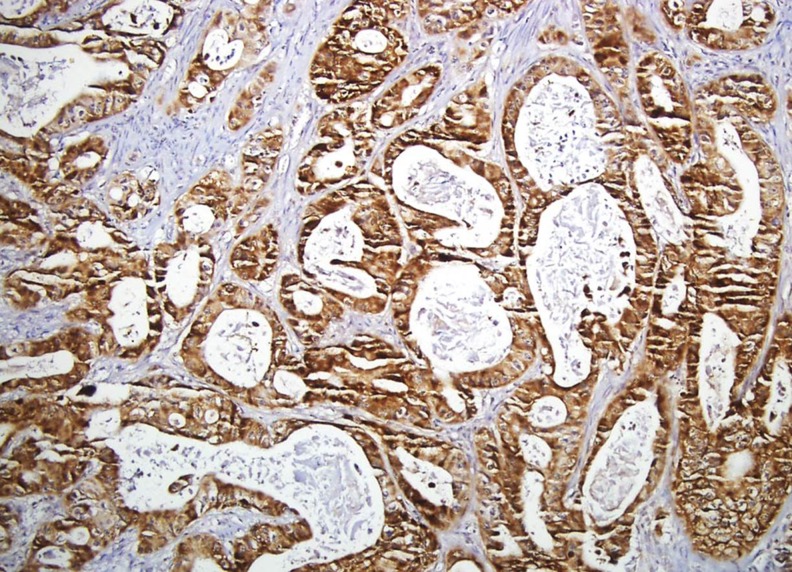


**PS-09-038**

**Prognostic value of tissue expression of matrix metalloproteinase -2, -7 and -9 in patients with colorectal cancer**

V. Janevska ^*^, L. Spasevska, V. Janevski, E. Kostova, B. Dukova, R. Jovanovic, E. Trajkovska

^*^Faculty of Medicine, Institute of Pathology, Skopje, Macedonia

**Objective:** The aim was to investigate the expressions of matrix metalloproteinase, MMP-2, MMP-7 and MMP-9 in tumour tissue and their relation to: disease stage, T and N parameters from pTNM classification, serum levels of MMP-2, MMP-7, MMP-9 and tissue inhibitors of MMPs; TIMP-1 and TIMP-2 in patients with colorectal cancer (CRC), preoperatively and at 3, 6, 9 and 12 month postoperatively.

**Method:** Specimens of cancer and surrounding normal tissue of 82 patients were immunohistochemically stained for MMPs. Expression of MMPs was analyzed in epithelial and stromal cells. Signal intensity and quantity of positive cells were determined. Results were correlated with the clinical and pathologic parameters.

**Results:** Expression of MMP-2 was more frequent in patients with higher serum levels of MMP-2, MMP-9, in patients with lymph node metastasis and advanced stage of the disease. Expression of MMP-7 was more frequent in patients with elevated serum levels of MMP-7, MMP-9 and deeply invasive neoplasms. MMP-9 cell expression was in a positive correlation with elevated serum levels of MMP-2, MMP-9 and depth of CRC invasion. There was no correlation between immunihistochemical expression of MMPs and serum levels of TIMPs.

**Conclusion:** Immunohistochemical expression of MMPs is a useful indicator for disease progression in patients with CRC.

**PS-09-039**

**S100A8 and S100A9 positive cells in colorectal carcinoma**

C. I. Bassorgun ^*^, A. Ozluk, N. Erin, O. C. Uzun, G. O. Elpek

^*^Akdeniz University, Pathology, Antalya, Turkey

**Objective:** S100A8 and S100A9 (S100A8/A9) are members of the S100 family of calcium-binding inflammatory proteins secreted by neutrophils and activated monocytes. Recent data indicated that, besides their role in inflammation, these proteins might play an important role in tumor development, progression and metastasis. Although, the influence of inflammatory cells on the behavior of colorectal carcinomas (CRC) has already been established previously, the relationship between the presence of S100A8/A9 expressing inflammatory cells to clinicopathological parameters has not been fully evaluated.

**Method:** Eighty patients diagnosed with colorectal adenocarcinoma (30 cases with distant metastasis, 30 cases with lymph node metastasis and 20 cases without metastasis) were included in the study. Quantitative evaluation was performed on tissue sections stained immunohistochemically with anti-S-100A8 and anti-S100A9 monoclonal antibodies. Peritumoral and intratumoral S100A8/A9 expressing inflammatory cells were counted in primary tumors and their metastasis.

**Results:** The number of peritumoral and intratumoral S100A8/A9 expressing cells was correlated with grade and tumor size (*p* < 0.05). Moreover, their number was significantly higher in primary tumors with metastasis when compared to tumors limited to colon (*p* < 0.05).

**Conclusion:** Our findings demonstrate that the number of peritumoral and intratumoral S100A8/A9 expressing inflammatory cells might be a reliable marker of metastatic potential of CRC.

**PS-09-040**

**High expression of IL-13 Receptor a2 in colorectal cancer is associated with invasion, liver metastasis, and poor prognosis**

J. I. Cornejo ^*^, M. Abengózar, R. Barderas, R. Bartolomé, M. J. Fernández-Aceñero, S. Torrés, J. I. Casal

^*^Fundación Jiménez Díaz, Pathology, Madrid, Spain

**Objective:** Autocrine secretion of cytokines by metastatic colorectal cancer cells has been poorly characterized. Interleukin 13 (IL-13) is a non-glicosilated cytokine produced by T lymphocytes, involved in lymphocyte growth and differentiation. The objective of our work is to establish the prognostic role IL-13R alpha2 overexpression in patients with colorectal cancer.

**Method:** Eighty patients diagnosed and treated of colorectal adenocarcinoma between 2001 and 2006 in Fundacion Jimenez Díaz (Madrid, Spain) were enrolled for the study. We have reviewed the clinical records and performed immunohistochemical detection of IL-13 and IL-13R alpha2. The intensity of the membrane and cytoplasmic staining was graded as absent, weak, moderate or intense. In all cases, sections from normal colonic mucosa distant from the tumor site were used as negative controls.

**Results:** IL-13R alpha2 was intensely overexpressed in 53 patients (66.3 %).

**Conclusion:** This high expression showed a clear association with late stages in human cancer and poor outcome. This worse prognosis could be attributed to the increased invasiveness and homing capacity of cells overexpressing IL-13R alpha2.

**PS-09-041**

**Systematic evaluation of intratumoral heterogeneity of EGFR, HIF1α and Ki-67 expression in primary colorectal carcinomas reveals a possible regulating effect by hypoxia**

A. M. Szasz ^*^, E. Agoston, K. Dede, K. Fekete, T. Krenacs, J. Timar, L. Harsanyi, J. Kulka

^*^Semmelweis University, 2nd Dept. of Pathology, Budapest, Hungary

**Objective:** To analyze EGFR, HIF1A and Ki67 expression in selected regions of CRCs and their lymph node metastases.

**Method:** Fifty-six patients were enrolled in the study. Regions of the CRCs were evaluated using TMAs: normal tissues (NN), tumor-normal border (TN), main tumor mass (TT), invasive front (TI), lymph node metastases (TL). Immunohistochemical EGFR, Ki67, HIF1A expression was evaluated digitally for intensity and frequency.

**Results:** Twenty-eight tumors harboured activating KRAS mutations, 28 were KRASwt. Expression of EGFR was highest in NN (average: 9.3 ± SD:1.4), followed by TL (8.0 ± 2.4), TI (7.7 ± 2.3), TT (7.5 ± 2.8), TN (7.2 ± 2.9). HIF1A showed similar gradient (TN: 3.6 ± 2.67, TT: 3.7 ± 2.2, TI: 5.3 ± 1.8, TL: 5.3 ± 2.4), while Ki67 displayed an opposite expression gradient (TN: 5.5 ± 1.7, TT: 4.8 ± 2.1, TI: 4.4 ± 2.5, TL: 3.6 ± 2.1). Significant difference was observed regarding EGFR expression between the NN and regions of the primary CRC (p/TN/ = 0.002, p/TT/ = 0.002, p/TI/ = 0.004). Lower HIF1A expression was noted in NN vs. TI (*p* = 0.011), and the expression of TL (*p* = 0.022); TT also displayed lower HIF1A levels than TI (*p* = 0.031).

**Conclusion:** EGFR and HIF1A expression correlates suggesting a regulating effect of hypoxia on EGFR expression.

**PS-09-044**

**Phosphoproteomic analysis of liver metastasis from colorectal cancer**

V. Canzonieri ^*^, E. Parasido, M. Pierobon, E. Pin, C. Belluco, L. Memeo, E. Petricoin

^*^CRO-IRCCS Aviano NCI, Dept. of Pathology, Italy

**Objective:** Liver metastasis is the main cause of death in Colorectal Cancer patients. To improve patient survival it is essential to identify signaling pathway derangements and to identify druggable targets for new therapies.

**Method:** We conducted a phosphoproteomic analysis on a multicentric collection of colorectal cancer liver metastasis samples. Twenty-eight samples have been analyzed from 14 patients with resectable metastasis and the rest with non-resecable metastasis. For each of them, metastasis biopsies were collected and microdissected for tumor enrichment. Pathways activation mapping analysis was performed using Reverse Phase Protein Microarray and 160 endpoints chosen among drug targets and downstream molecules were evaluated. Unsupervised hierarchical clustering analysis and mean comparison analysis were used to compare resectable and non-resectable patients.

**Results:** We revealed distinct clustering based on tumor resectability. Mean comparison identified the activation of candidate drug targets and downstream kinase substrates of MAPK and AURORA kinase signaling in the non-resectable compared to resectable population. On the base of these data some commercially available Phase II–III drugs could be considered for prospective clinical trial.

**Conclusion:** Based upon these results, a prospective multi-center validation set will be collected to confirm data and exclude the presence of any bias associated to sample collection techniques.

**PS-09-045**

**The value of CDX2 and cytokeratins 7 and 20 expression in identifying colorectal metastases**

R. Jouini ^*^, W. Koubâa, N. Abdessayed, I. Msakni, M. Bel Haj Salah, A. Bouhafa, O. Khayat, N. Labbene, E. Ben Brahim, A. Chadli Debbiche

^*^Habib Thameur Hospital, Pathology, Tunis, Tunisia

**Objective:** The purpose of this study is to determine the value of CDX2 expression and CK7/CK20 phenotype in predicting the colorectal origin of metastatic adenocarcinomas of the the liver and peritoneum.

**Method:** CK7/CK20 staining pattern and CDX2 expression were evaluated in 22 biopsies of metastatic adenocarcinomas of the liver or peritoneum. The primary tumor localisation was established in all cases by a combination of clinical, radiological and histological data.

**Results:** The CK7-/CK20+ immunophenotype was expressed by 5/8 adenocarcinomas of colorectal origin, 2/5 adenocarcinomas of gastric origin and by only 1/9 adenocarcinomas of pancreatic origin. The CK7+/CK20-expression pattern was observed in only one case of colorectal metastases, in 8 of 9 cases of pancreatic metastasis and in one case of gastric metastases. The CK7+/CK20+ immunophenotype was expressed in 2 cases of colorectal metastases but not in gastric or in pancreatic metastases. CDX2 expression was observed in all cases (8/8) of adenocarcinomas of colorectal origin, in 2 of 5 cases of gastric origin. In metastases of pancreatic origin, CDX2 was not expressed.

**Conclusion:** The CK7-/CK20+ phenotype is very helpful in predicting colorectal origin of liver or peritoneal metastases. CDX2 should be a useful adjunct for the diagnosis of colorectal primary localisation when the CK7 and CK20 yield equivocal results.

**PS-09-046**

**Gastrointestinal tract metastasis as first manifestation of primary lung malignant tumors**

S. Gheorghita ^*^, E. Gramada, C. Popp, L. Nichita, R. Chirculescu, A. Bastian, M. Gafton, A. Oniga, F. Staniceanu

^*^Colentina Universitary Hospita, Pathology, Bucharest, Romania

**Objective:** First manifestation of a primary lung malignant tumor as a gastrointestinal (GI) metastasis is very rare and represents a late-stage disease sign. Adequate microscopic diagnosis offers the best choice of treatment, improving the poor prognosis of these patients.

**Method:** Between 2010 and 2012 we identified 3 cases of metastasis from lung carcinoma in gastrointestinal tract out of a total of 1050 cases of gastrointestinal malignancies routinely diagnosed in our department.

**Results:** Our patients were 2 men and 1 woman, with a mean age of 69.3 years. One case involved the small bowel, one the colon, and one patient had two tumors in both small bowel and colon. All cases had histopathological aspect of poorly differentiated carcinoma with peculiar immunohistochemical phenotype: (CK7 +, CK20 +/−, 34βE12 +/−, p63 −/+, TTF1 +).

**Conclusion:** Lung carcinoma presenting as GI-tract metastasis represents a diagnostic challenge, thus adding 34βE12, p63, and TTF1 in antibody panel for imunohistochemical characterization of poorly differentiated carcinoma of the GI-tract proves helpful in selected cases.

**PS-09-047**

**Factors affecting the number of dissected lymph nodes in rectum tumor resections after neoadjuvant therapy**

A. Gunal ^*^, B. Kurt, M. Oztas, N. Ersoz, Y. Karslioglu

^*^Gülhane Askeri Tip Akademisi, Patoloji AD, Ankara, Turkey

**Objective:** In colorectal resections, to use the number of metastatic lymph nodes as a reliable criterion, the total number of dissected lymph nodes must be above a certain threshold due to guidelines. However, achieving the required number of lymph nodes can be difficult in rectum resections after neoadjuvant treatment.

**Method:** 102 rectum resections with neoadjuvant treatment were reviewed. The relation with the total number of dissected lymph nodes and the criteria related to patient, tumor and pathologic evaluation were valuated.

**Results:** The total numbers of dissected lymph node were greater in high grade tumors from younger patients showing mucinous component, perineural invasion, positive radial surgical margin, and lymph node metastases at the time of diagnosis. Inadequate tumor regression, extramural tumor depth, and being resected in recent years are also correlated with increased number of total lymph nodes. We noticed that the diameter of the largest lymph node has an impact on the total number of lymph nodes, too. Among these criteria, the patient’s age, tumor depth and the diameter of the largest dissected lymph node were more significant ones.

**Conclusion:** Here, we evaluated the rectum resections received neoadjuvant therapy as a unique model. Factors that should kept in mind while evaluating the required number of lymph nodes were discussed.

**PS-09-048**

**The effect of methylene blue assisted lymph node mapping in colorectal radical resection specimens to establish lymph node number and size**

G. Kir ^*^, H. Seneldir, C. S. Topal, M. H. Karabulut, M. S. Yilmaz

^*^Umraniye Hospital, Dept. of Pathology, Istanbul, Turkey

**Objective:** Adequate lymph node evaluation is very crucial for appropriate lymph node staging in colorectal carcinoma.

**Method:** In this study we performed a prospective study comparing lymph node recovery of 54 colorectal resection specimens with (21 cases) and without (33 cases) postoperative methylene blue lymph node mapping.

**Results:** Otal lymph node number and the number of lymph nodes which are smaller than 0.5 cm were significantly higher in the first group than in second group (*p* < 0.05).

**Conclusion:** Using postoperative methylene blue lymph node mapping in colorectal radical resection specimens provided us to evaluate higher number lymph nodes and smaller lymph nodes. We think it is highly effective and simple method that will improve lymph node yield in colorectal resection specimens.

**PS-09-049**

**Does GEWF solution improve lymph node harvest in colorectal cancer specimens with and without neoadjuvant therapy?**

A. Börretzen ^*^, F. Pfeffer, S. Leh

^*^Haukeland University Hospital, Dept. of Pathology, Gades Insitut, Bergen, Norway

**Objective:** Accurate assessment of lymph node status in colorectal cancer resections has both prognostic significance and therapeutic consequence. We examined the effect of GEWF solution (glacial acetic acid, ethanol, water and formaldehyde) on overall lymph node yield, positive tumor lymph node yield and possible upstaging.

**Method:** Colorectal cancer specimens (*n* = 456) between January 2010 and December 2012 were fixed either in formalin (*n* = 146) or GEWF solution (*n* = 310) and lymph node yield was compared.

**Results:** Mean lymph node count per colonic and rectal specimen without neoadjuvant therapy increased from 17.1 to 22.3 and 16.2 to 20.6 respectively by fixation in GEWF solution (*p* < 0.05). There was no significant difference in mean lymph node count regarding rectal specimens with neoadjuvant therapy fixed either in formalin (15.1) or GEWF solution (16.4). Mean positive lymph node count did not differ significantly between formalin and GEWF fixation in colonic (2.0 vs. 1.4) and rectal specimens without neoadjuvant therapy (1.4 vs. 1.1) or rectal specimens with neoadjuvant therapy (1.2 vs. 1.0). GEWF solution did not lead to upstaging.

**Conclusion:** Lymph node count was adequate using conventional formalin fixation. Though generally increasing mean lymph node harvest, GEWF solution did not increase positive lymph node count and did not lead to upstaging.

**PS-09-050**

**Molecular lymph-node upstaging in colon cancer using One-Step Nucleic Acid Amplification (OSNA): Preliminary results from a Spanish multicenter study**

M. Cuatrecasas ^*^, R. Ortiz, J. D. Sardon, L. Bernet, M. C. Mendez, K. Elorriaga, J. Tarragona, T. Pereda, C. Villar, C. Ibarrola, J. Zamora

^*^Hospital Clinic Barcelona, Dept. of Pathology, Spain

**Objective:** Colon Cancer (CC) is the second cause of death from cancer in developed countries. 2–5μm HE lymph-node sections used for lymph node staging (pN) represent <1 % of the entire node. Aproximately 25 % stage I–II patients (pN0) will have local recurrence or distant metastases within 5 years of surgery. The sensitivity of HE for detecting micrometastases is very low, and does not identify those patients that could benefit from adjuvant therapy. Aims: To assess the rate of stage I–II CC patients understaged with standard HE, compared to the OSNA molecular analysis for detecting lymph-node metastases.

**Method:** Fresh lymph-node dissection from 106 stage I–II CC patients (3 pT0, 8pTis, 24pT1, 22 pT2, 38pT3, and 10pT4) selected from 9 Spanish hospitals was done. Half of the node was analysed by HE and the other half using OSNA. All patients were staged pN0 with HE.

**Results:** Of the 106 pN0 patients staged with HE, 48(45.3, 95 % CI 35.6–55.2 %) were up-staged using the molecular analysis (OSNA); 44(42.4 %) were micrometastases and 10(9.4 %) macrometastases.

**Conclusion:** Molecular lymph-node staging using OSNA is more sensitive than HE staging and may be useful to select stage I–II CC patients that could benefit from adjuvant therapy.

**PS-09-051**

**Lymph-node molecular staging of early colon cancer (pT1) and malignant polyps using One-Step Nucleic Acid Amplification (OSNA)**

M. Cuatrecasas ^*^, R. Ortiz, M. Pellise, F. Balaguer, D. Monblan, S. Delgado, I. Aldecoa, L. Herrero, R. Almenara, A. Castells, A. de Lacy

^*^Hospital Clinic Barcelona, Dept. of Pathology, Spain

**Objective:** Early colon cancer (ECC) is a pT1 adenocarcinoma. A malignant polyp is an endoscopically benign polyp with a pathologic diagnosis of infiltrating adenocarcinoma. After a malignant polyp diagnosis, either surgical resection of the colonic segment, or a conservative approach is done, depending on the presence of high-risk histologic factors. Aims: To determine the presence of lymph-node metastases using the molecular method OSNA in pN0 by HE colectomy samples, from ECC or performed after a malignant polyp diagnosis.

**Method:** Fresh dissection of 301 lymph-nodes from 25 colectomies (4 to 23 lymph nodes) was done from 21 patients with previous malignant polyp, and 4 ECC patients. One half of the node was analysed by HE, and the other half by OSNA. Lymph-node staging with HE and OSNA were compared.

**Results:** All patients were pN0 with HE. OSNA found lymph-node metastases in 11 patients (7 pN1a; 4 pN1b). Haggitt and Kikuchi levels of submucosal invasion in malignant polyps did not completely correlated with the OSNA results.

**Conclusion:** Molecular lymph-node staging with the OSNA method is highly sensitive and reveals the presence of lymph-node metastases at early stages of colon cancer. Long-term follow-up of these patients will allow determining the prognostic value of metastases detected by molecular methods.

**PS-09-052**

**Feasability of molecular detection of lymph node metastasis in colorectal adenocarcinomas by One-Step Nucleic Acid Amplification (OSNA) in daily routine practice**

F. Forest ^*^, A. Petcu, C. Habougit, S.-A. Berremila, A. Clemenson, J. Porcheron, J.-M. Phelip, M. Peoc’h

^*^CHU De Saint Etienne, Anatomie Pathologique, France

**Objective:** In colorectal cancer, the lymph node status is an important parameter determining the therapy, especially neo-adjuvant chemotherapy. The evaluation of the lymph node status is usually made by histologic technique. Since OSNA is a reliable technique for the evaluation of metastasis in breast cancer, detecting more metastasis than histological technique, we wondered if its application is feasable in colorectal adenocarcinomas in daily routine practice. The objective of our study is to assess the feasability of this technique for lymph nodes in colonic adenocarcinomas.

**Method:** We evaluated the parameters necessary for the dissection of lymph node and molecular analysis, and we compared them to the parameters of “classical” processing (macroscopic and histologic evaluation.

**Results:** Our study shows that implementation of OSNA for the routine evaluation of lymph node status in colorectal cancer was feasable but was slightly more time-consuming than classical histological evaluation.

**Conclusion:** OSNA seems to be a feasable technique for the evaluation of lymph node status in colorectal adenocarcinomas. Since it seems to detect a higher rate of lymph node metastasis, it could upgrade the clinical staging.

**PS-09-053**

**Primary large adenosquamous carcinoma of the cecum and ascending colon**

V. Leontara^*^, E. Minaidou, T. Zogka, C. Karamveri, Z. Kontogianni-Miller, T. Choreftaki

^*^General Hospital of Athens, Surgical Pathology, Greece

**Objective:** Primary adenosquamous cell carcinomas (Ad-SCCs) of the colon are rare and accounts for less than 0.1 % of all colorectal malignancies. The clinical presentation and gross findings of Ad-SCC of the colon are similar to those of adenocarcinoma of the colon, but Ad-SCC has a more aggressive clinical course and a poorer prognosis.

**Method:** We report a case of primary large Ad-SCC of the cecum and ascending colon. The patient was a 75 year old male, who presented diarrhea, right lower quadrant abdominal pain and loss of weight during the last period. No history of existed malignancies or Inflammatory Bowel Disease (IBD), neither on the patient or his family. Abdominal Computed Tomography scan and colonoscopy reported a large mass occupy the cecum and part of ascending colon. A right hemicolectomy was performed.

**Results:** Histopathological examination revealed 13 × 8 × 2.5 cm sized collision-type Ad-SCC. Four out of 38 lymph nodes contain metastasis. The postoperative recovery was uneventful and adjuvant chemotherapy was administrated.

**Conclusion:** Histogenesis of colorectal Ad-SCC is not clearly understood but different hypotheses have been suggested. According to them malignant squamous cells can originate from transformation of ectopic squamous cell in colonic mucosa, undifferentiated or reserve cells in colonic epithelium, normal glandular cells or directly from adenocarcinoma in situ.

**PS-09-054**

**Primary adenosquamous carcinoma of the colon and upper rectum: Report of three cases**

S. Haraoka ^*^, A. Iwashita, I. Iwashita

^*^Fukuoka University, Pathology, Chikushi Hospital, Chikushino-Shi, Japan

**Objective:** Primary adenosquamous carcinomas of the colorectum are rare, accounting for about 0.1 % of colorectal cancer. We report three cases of primary adenosquamous carcinoma of the colon and upper rectum.

**Method:** Three patients are 67-year-old man, 54-year-old man, and 68-year-old woman. The involved sites of the tumor included transverse colon in two cases and upper rectum in one case. The radical resection of the tumor was performed. The size of three tumors was 5 cm, 9 cm, 5.8 cm in the greatest diameter, respectively. Lymph node metastasis and distant metastasis were not found at operation.

**Results:** Histological examination confirmed the advanced carcinomas composed of intimate admixtures of tubular adenocarcinoma, squamous cell carcinoma, and poorly differentiated element. Immunohistochemically, the adenocarcinoma component was positive for CAM5.2, CK18, and CEA. The squamous cell carcinoma component was positive for 34βE12 in two cases and P63 in one case. The carcinoma cells were occasionally positive for CDX-2 and CK20 in one case, whereas negative for those markers in two cases.

**Conclusion:** Adenosquamous carcinoma of the colorectum consists of both carcinoma components and a transitional area. The histologic and immunohistochemical features suggest a hypothesis about the histogenesis that squamous metaplasia occurs during the process of adenocarcinoma development.

**PS-09-055**

**Anal margin melanoma: About one case with review of literature**

S. Mameri ^*^

^*^Beni-Messous Hospital, Dept. of Pathology, Algiers, Algeria

**Objective:** We report a case of anal melanoma. This tumor is rare in this localization accounting for only around 0.2–0.3 % of all melanoma.

**Method:** This case concerns a 46-years-old woman who is followed for hemorrhoids causing bleeding and pain. She undergoes resection of these hemorrhoids. The surgical specimen sent to our Unit pathology realizes a 2 cm reddish mass showing an ulceration by a little nodule of 1 cm,whitish and brown, ferm. Two samples of this nodule are embedded in paraffin and stained with hematoxylin-eosine. Secondarily, an immunostaining using HMB45 antibody is practiced.

**Results:** The histopathological exam reveals an authentic melanoma, nodular with round cells and large amounts of melanic pigment. The HMB45 immunostaining shows a diffuse and intense positivity.

**Conclusion:** Several studies confirm that melanoma is a very rare neoplasm in sun-protected regions like anal mucosae. It generally affects patients between 58 and 71 but pediatric and adolescent cases are reported in literature. Our case concerns an adult younger than the age bracket. Furthermore, all studies reveal that anal melanoma is often confused clinically with prolapsed rectal polyp, hemorrhoids, fissures or perianal abcesses leading sometimes to a delayed diagnosis. A case report mentions the occurrence of a melanoma on a perianal fistula in a 38-years-old man after 4 years of evolution.

Monday, 2 September 2013, 09.30–10.30, Pavilion 2

**PS-10 Poster Session Electron Microscopy**

**PS-10-001**

**Is electron microscopy an effective tool in the practice of fine needle aspiration?**

E. A. Turbat-Herrera ^*^

^*^LSU Health, Department of Pathology, Shreveport, USA

**Objective:** Comparison of immunohistochemistry(IHC) with electron microscopy(EM) as a diagnostic tool for the practice of fine needle aspiration(FNA)cytology.

**Method:** 113 cases submitted to the Cytopathology Service, 25 went to the EM in Pathology at LSUHSC(to author)during 9/2012–3/2013 for diagnosis. 90 % of the cases were obtained at the time of FNA, the rest of the cases were obtained from paraffin and submitted for EM. All cases were fixed in gluteraldehyde

**Results:** Of the 113 cases, only 108 included since 5 cases were insufficient. A total of 24(22 %) cases were submitted for EM of which only 20 (19 %)cases had sufficient cells for ultrastructural studies. 13 required IHC(12 %), 10 cases were submitted for Histochemistry(9 %) and 3 cases were submitted for FISH(3 %).

**Conclusion:** Understanding the limitations of the ancillary technique of choice is paramount. Favoring a technique to the exclusion of others places pathologists at a disadvantage. A judicious combination of ancillary techniques often is the best approach.

**PS-10-004**

**Encephalopathy-related changes in the white matter evidenced by immunoreactivity of superoxide dismutase and metalloproteinase-9**

S. Skuja ^*^, V. Groma, M. Tarasovs, G. Karelis, A. Stepens, O. Teteris, M. Murovska

^*^Riga Stradins University, Laboratory of EM, Latvia

**Objective:** Encephalopathy has been reported to be developing due to metabolic alcohol-, drug-related factors and infections. Metalloproteinases (MMPs) have been implicated in a number of neurological conditions. Cu/Zn superoxide dismutase (SOD1) overexpression leads to MMP9 activation and the white matter changes. We evaluated impact of MMP9 and SOD1 in alcohol addiction associated encephalopathy.

**Method:** Immunoexpression was detected by conventional technique on sections obtained from subcortices. Statistics was performed using semi-quantitative scoring and non-parametric tests; results were presented as medians.

**Results:** Subcortical white matter of chronic alcohol addicts demonstrates statistically significant differences for SOD1 and MMP9 immunolabeling, respectively, in myelinated axons 0.80 (0.60; 1.00), 0.35 (0.30; 0.40); astrocytes 0.25 (0.20; 0.40), 0.00 (0.00; 0.10); oligodendrocytes 0.40 (0.20; 0.80), 0.10 (0.10; 0.28). Ultrastructurally, axons showed loss of myelin compaction, split of lamellae, crossing and ballooning of paranodal loops.

**Conclusion:** Subcortical affection of myelination mediated by glial contribution, metalloproteinase implication, and protective action of SOD1 were evidenced in alcoholics.

**PS-10-005**

**Renal hydroxichroloquine toxicity and Fabry disease: How to differentiate in EM?**

R. M. Costa ^*^, E. Martul, J. Reboredo

^*^CHTMAD, Dept. of Nephrology, Vila Real, Portugal

**Objective:** Presence of myeloid bodies in electron microscopy from renal biopsies may result from hydroxichloroquine toxicity and differentiation for Fabry Disease was only possible after genetic/enzimatic analysis. However, recent studies revealed curvilinear bodies in renal cells in hydroxichroloquine toxicity cases, never described in Fabry’s nephropathy. We report a 31-year-old patient with Systemic Erithematous Lupus under long term therapy with hydroxychloroquine. The presence of zebra bodies in electron microscopy lead to initial interpretation of Fabry disease but genetic analysis showed no mutation. Further reevaluation revealed curvilinear bodies in renal cells, supporting hydroxycholoroquine induced renal phospholipidosis.

**Conclusion:** The presence of curvilinear bodies seems to be an crucial feature to distinguish hydroxychroloquine induced lesion from Fabry’s nephropathy.

**Curvilinear bodies:** 
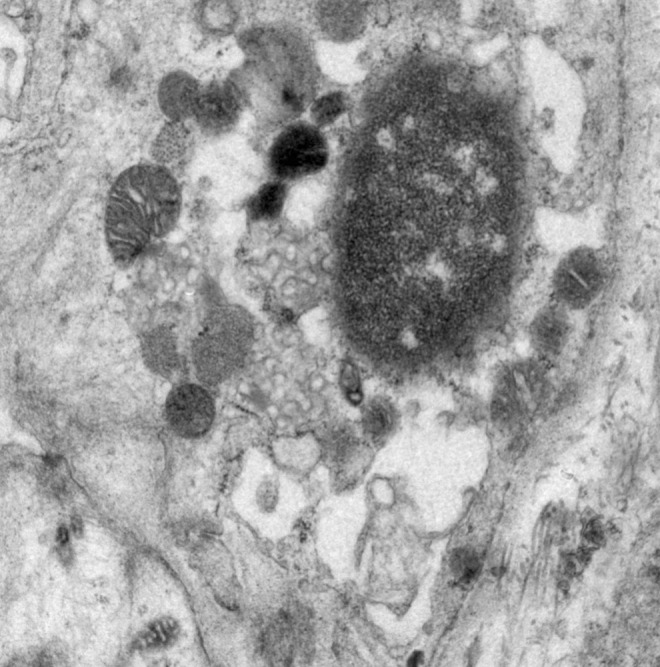


**PS-10-006**

**Colocalization of F-actin and SATB1 after induced cell death by using phalloidin-based method for F-actin and immunogold technique for SATB1 labelling at the level of TEM**

D. Grzanka ^*^, M. Gagat, M. Izdebska

^*^Collegium Medicum UMK, Clinical Patomorphology, Bydgoszcz, Poland

**Objective:** Quantum dots (QDs) are fluorescent nanocrystals whose unique properties are fundamentally different from organic fluorophores. Moreover, their cores display sufficient electron density to be visible under transmission electron microscopy (TEM). Together with widely used immunogold method for protein labelling, the usage of QDs make excellent material for multi-labelling of protein at the level of TEM.

**Method:** Here, we report a technique for TEM colocalization of F-actin and SATB1. The ultrastructural reorganization of F-actin after doxorubicin treatment was estimated using a combination of pre- and post-embedding techniques with biotinylated phalloidin and QD-streptavidin conjugates but SATB1 was localized using post-embedding immunogold method.

**Results:** TEM studies showed different patterns of QD-labeled F-actin after doxorubicin (DOX) treatment. In the case of colocalization technique using QD-based and nanogold (AU) methods, there were seen a different distribution and dependence of F-actin and SATB1 in 750 nM DOX treated Jurkat cells.

**Conclusion:** Our studies showed that combination of QDs and AU nanoprobes are useful in high-resolution colocalization studies at the level of TEM.

**PS-10-007**

**Enlarged platelets with abnormal morphology in congenital asplenia: A case report**

T. Martinovic ^*^, D. Ciric, O. Markovic, D. Trpinac, T. Kravic

^*^School of Medicine, Inst. of Histology, Belgrade, Serbia

**Objective:** We report a case of 52-year old woman with low platelet count (54 × 109/L) in congenital asplenia. Prothrombin time, aPTT and bleeding time were normal. The objective was to determine the ultrastructural characteristics of platelets in this patient.

**Method:** Full blood sample was fixed in 3 % glutaraldehyde, rinsed in cacodylate buffer, dehydrated in ethanol and propylene oxide, and processed for embedding in EPON. Ultrathin sections were stained with uranyl acetate and lead citrate, and examined by transmission electron microscope (Morgagni 268D).

**Results:** Platelets of abnormal morphology, sugestive of their activation, can be seen on TEM. They show spheroid forms with pseudopodia and the centralization of granules. Marginal band of microtubules cannot be spoted. However, unlike the usual appearance of activated platelets, there is no dilatation of open canalicular system. Platelets were enlarged on conventional blood smear, and on TEM the largeset mesured diametar of platelets were 6.7 μm.

**Conclusion:** Findings are very unusual since in patients with asplenia common finding is that of small thrombocytes. This could be explained by a peripheral platelet consuming process in this patient which may share underlying causes with other clinical findings. This would be consistent with platelets abnormal appearance, indicating possible aberrant activation.

Monday, 2 September 2013, 09.30–10.30, Pavilion 2

**PS-11 Poster Session Haematopathology**

**PS-11-001**

**c-Jun N-terminal kinase is activated and contributes to proliferation of Hodgkin and Reed Sternberg cells of classical Hodgkin lymphoma**

E. Drakos ^*^, V. Leventaki, M. Karanikou, K. Psatha, V. Sinatkas, A. Eliopoulos, H. Papadaki, L. J. Medeiros, E. Patsouris, G. Z. Rassidakis

^*^University of Crete, Medical School, Dept. of Pathology, Heraklion, Greece

**Objective:** Activated c-Jun N-terminal Kinase (JNK), a member of the mitogen activated protein kinase (MAPK) superfamily, activates c-Jun, a member of the activator protein-1 (AP-1) family, and it is implicated in cell transformation. Previous studies have shown that c-Jun is activated and overexpressed by the neoplastic cells (HRS) of classical Hodgkin lymphoma (cHL). However, the activation status and the functional significance of JNK in cHL are unknown.

**Method:** We investigated JNK activation/phosphorylation (p-JNK) in 58 cHL tumors by immunohistochemistry. The biologic effects of pharmacologic inhibition of JNK by SP600125 in cHL cells (MDA-V, KM-H2, L1236, L428) were investigated in vitro by MTT and colony formation assays, trypan blue and PI-staining and western blot analysis.

**Results:** Most cHL tumors (52/58, 89.6 %) expressed p-JNK, assessed using a 10 % cutoff for positivity. The percentage of p-JNK-positive HRS cells ranged from 5 to 95 %, with a median of 70 %. Pharmacological inhibition of JNK activity in cultured HRS cells resulted in significant decrease of cell growth and G2 cell cycle arrest associated with de-activation of c-Jun and upregulation of its known gene target, p21.

**Conclusion:** The data suggest that JNK is highly activated in HRS cells of cHL tumors, and may contribute to proliferation of HRS cells.

**PS-11-002**

**Expression of c-Met/HGF (hepatocyte growth factor) and tumour associated macrophages markers CD163 and CD68 in Hodgkin Lymphoma (HL): Correlation with progression free survival (PFS)**

J. Martín López ^*^, D. García, M. J. Coronado, Y. Vicente, L. Kilany, I. Krsnik, C. Bellas, P. Martín

^*^Hospital Univ Puerta de Hierro, Pathology, Majadahonda (Madrid), Spain

**Objective:** We aim to investigate the expression of c-Met and HGF in HL patients, correlation with EBV infection and its prognostic value.

**Method:** Biopsies from 60 HL patients were included in this study. By using the tissue microarray technique, we have performed an immunohistochemical study of c-Met, HGF, CD68, CD163 and Ebers. FISH was performed for c-met amplification. PFS was used for survival analysis.

**Results:** Histological distribution was 39 nodular sclerosis (NS), 13 mixed cellularity (MC), 1 lymphocyte depleted, 3 lymphocyte rich and 4 PLNHL. c-Met was positive in 16 cases (25 %) of the total series and in 46 % (6/13) of MC subtype. 9 cases (14 %) were HGF+ and 13 cases EBV+. There was no association between c-Met/HGF expression and EBV infection. Tumour-associated macrophages markers showed 16 cases CD163+ and 10 cases CD68+. FISH analysis showed polyploidy but not c-Met gene amplification. CD163+ is associated with shortened PFS.

**Conclusion:–**c-Met is expressed in 25 % of HL, mainly in MC subtype and this expression is independent of EBV infection–No association has been found between c-Met/HGF and PFS. c-Met expression is not associated with gene amplification–CD163 is more specific marker than CD68 and its expression is associated with shortened PFS

**PS-11-003**

**Hodgkin lymphoma: A clinicopathologic study of 85 cases**

G. Benkhedda ^*^, N. Khelifi, Y. Lamouti

^*^Saad Dahleb University, Dept. of Medicine Pathology, Blida, Algeria

**Objective:** Hodgkin lymphoma (LH) is characterized by the presence of Reed-Sternberg (RS) cell, although other abnormal cell types, in a particular cellular environnement. It is relatively rare. It is highly curable disease (70 %) whose forecast and treatment depend on the stage of extension of the disease. The aim of this study is to assess the pathological features, clinical and epidemiological from our series and to compare our results with those of the literature.

**Method:** We exploited 85 cases of LH followed in our department. All cases are diagnosed in the lymph node. Thin sections of tumor specimen were stained with usual techniques, and study immunohistochemical using the CD15 and CD30

**Results:** The LH accounts for 36 % of the whole of the lymphoma. The distribution from the age is bimodal with 2 peaks of frequency 10–20 and 50–70 years. The female sex was prevalent(sex-ratio = 1,12). It was almost about disease of traditional Hodgkin (99 %)with nodular sclerosis(76 %) and mixed cellularity(17 %). The cells of RS are marked by CD30 in 100 % and CD15 in 84 %.

**Conclusion:** Our series is distinguished from the other series by the frequency and the sex. The results of histological types and immunohistochimy were similar to those reported in the literature apart from the type of lymphocyte depletion which was not found in our series.

**PS-11-004**

**Glut-1, a diagnostic marker of Reed-Sternberg cell**

H. Sevestre ^*^, C. Attencourt, J.-F. Ikoli, J.-P. Marolleau

^*^CHU, Pathology, Amiens, France

**Objective:** To test Glut-1 expression by Reed-Sternberg and Hodgkin cells in Hodgkin lymphoma, in order to identify a marker of RS and H cells.

**Method:** Archival cases of Hodgkin lymphoma retrieved from our files. Diagnosis confirmed by two of us in 65 cases. Full size recut immunostained in a Benchmark Ultra instrument (Ventana, Roche Diagnostics) using a monoclonal antibody directed against Glut-1. Slides were independently scored by one resident and two pathologists, in order to recognize and classify RS and/or H cells expression of Glut-1 in a semi-quantitative mode.

**Results:** RS and H cells were easily recogniz by a membrane staining in most cases. Staining was described as strong in 67 %, weak to moderate in 30 % and null in 3 % of cases respectively.

**Conclusion:** Glut-1 proved to be very sensitive to identify RS and H cells in this series of Hodgkin lymphomas. Its specificity remains to be demonstrated. Some lymphocytes, were stained to a lesser extent and intensity.

**PS-11-005**

**Breast involving Burkitt’s lymphoma**

L. Santos^*^, D. A. Portela, J. O. Gomes Santos, J. O. Ibiapina, A. C. de Oliveira Lima, C. M. Oliveira, J. B. Parentes Vieira, T. Carvalho

^*^Federal University of Piaui, Dept. of Pathology, Teresina, Brazil

**Objective:** Burkitt’s lymphoma is a mature B-cell neoplasia highly aggressive that impacts primarily children and young adults. Endemic, sporadic and immunodepression associated forms have been described. The sporadic form presents itself mostly as an abdominal mass with an involvement of nodal and extranodal sites. We hereby present a Burkitt’s lymphoma case with a simultaneous involvement of the digestive system and the breast.

**Method:** Case Report: MBSN, 25 years old, with a complaint of abdominal pain, weight loss and vomiting. Abdominal and pelvic CT and US showed pelvic lesion of poorly defined limits and multiple hepatic nodules. Endoscopy with biopsy showed diffuse infiltration of the gastric mucosa by small hyperchromatic lymphoid cells, positive for CD20 and CD10 and negative for CD3, cyclin, Bcl-2 and TdT with a cell proliferation index of 100 %, confirming the diagnosis of Burkitt’s lymphoma. In a posterior investigation, the patient presented a hypoechoic nodular lesion in the upper outer quadrant of the left breast, which a needle biopsy confirmed a lymphomatous infiltration. The patient was referred to clinical oncology for chemotherapy.

**Conclusion:** Breast involvement has been described in all clinical forms of Burkitt’s lymphoma, being usually a bilateral and diffuse infiltration. This case presented itself only as an isolated nodule simulating a lesion of benign nature.

**PS-11-006**

**Tissue quality alters correlation between MYC protein immunoexpression and MYC locus rearrangement detected by fluorescence in situ hybridisation in “aggressive” B-cell non-Hodgkin lymphoma**

O. Ramuz ^*^, J. Skerman, F. Tang, L. Wockner

^*^Pathology QLD Central Laboratory, Dept. of Anatomical Pathology, Herston, QLD, Australia

**Objective:** Detection of MYC by immunohistochemistry (MYC IHC) has been described as possible surrogate to fluorescence in situ hybridisation (FISH) in “aggressive” B-cell non Hodgkin lymphomas (ABCL). We assessed whether MYC IHC could be used to screen ABL cases eligible for FISH.

**Method:** We applied MYC IHC on 75 formalin-fixed-paraffin embedded (FFPE) specimens of ABCL with known FISH status. MYC IHC was scored as percentage of positive tumour cells by two pathologists following two different methods and in a blinded manner. Results were there analysed for inter-observer reproducibility and correlation with FISH results. Receiver operating curves (ROC) were drawn for each method to find ideal cut-off values. Influence of the quality of the specimens on correlation between MYC IHC and FISH was then studied.

**Results:** Both methods showed good inter-observer correlation. MYC IHC correlated with FISH status, but the two methods appeared to show significantly different specificities at high sensitivity levels. Noticeable improvement of the predictive value of MYC IHC was observed when specimens of poor quality were excluded.

**Conclusion:** MYC IHC is reproducible but is affected by the quality of FFPE tissue specimens, which may become an issue when assessing suboptimal material.

**PS-11-007**

**Diffuse large B-cell lymphoma, primary of the central nervous system (DLBCL-PCNS) harbours frequent translocations of the BCL6 but not of the MYC or the BCL2 genes**

G. Tapia ^*^, A.-M. Muñoz-Mármol, M.-J. Baptista, A. Gaafar, M. Puente-Pomposo, C. Sanz, R. Marginet-Flinch, M. Lopez-Peña, L. Perez-Roca, J.-L. Mate, A. Ariza

^*^Hospital Germans Trias i Pujol, Pathology, Badalona, Spain

**Objective:** MYC gene translocations occur in 10–15 % of diffuse large B-cell lymphomas and are associated with bad prognosis, especially if accompanied by BCL2 or BCL6 translocations. Our objective was to evaluate DLBCL-PCNS immunophenotype and molecular profile, focusing on MYC, BCL6 and BCL2 translocations.

**Method:** DLBCL-PCNS cases from our two institutions were collected. Immunophenotype was re-evaluated and cases were classified as GC-like or non-GC (Hans algorithm). The status of MYC, BCL2 and BCL6 genes was evaluated by FISH using break-apart probes.

**Results:** The study included 49 cases (median age, 59 years). Immunophenotype: CD10, BCL6 and MUM1 were positive in 3/49 (6 %), 34/48 (71 %) and 48/49 (98 %) cases, respectively. Four instances were GC-like. The mean proliferative index (Ki67) was 72 %. MYC translocation was demonstrated in 1/48 cases (2 %) and BCL6 translocation in 19/47 (40 %). BCL2 translocation was not demonstrated in any case. Moreover, copy gains or losses, with no MYC, BCL2 or BCL6 gene translocations, were observed in 17 %, 36 % and 11 % of the cases, respectively.

**Conclusion:** DLBCL-PCNS belongs to the non-GC subtype and undergoes frequent translocations of BCL6, but not of BCL2 or MYC genes. These findings indicate that DLBCL-PCNS molecular profile is unlike that of its nodal counterpart.

**PS-11-008**

**Clinical impact of immunohistochemical subtyping in patients with de novo diffuse large B-cell lymphoma treated with R-CHOP immunochemotherapy**

M. Melachroinou ^*^, E. Plakoula, P. Lampropoulou, C. Sirinian, A. Symeonidis

^*^University of Patras, Pathology, Greece

**Objective:** Hans algorithm has been widely used as standard to subclassify diffuse large B-cell lymphoma (DLBCL) into germinal center B-like (GCB) and nonGCB subtype. However, several studies have shown conflicting results regarding its prognostic significance. We retrospectively examined the prognostic value of Hans algorithm and the individual immunohistochemical biomarker in patients with DLBCL treated with R-CHOP.

**Method:** Fifty DLBCL cases [25 nodal, 25 extranodal] were immunostained for CD20, CD10, BCL6, MUM1, CD138 and BCL2. The cases were subclassified into GCB and nonGCB subtype according to the Hans algorithm.

**Results:** Fifteen cases (30 %) were classified as GCB group and 32 (64 %) as nonGCB group [3 unclassified]. Overall survival (OS) was significantly improved in the GCB group compared to the nonGCB group (*p* = 0.003). CD10 and BCL6 overexpression (cut off 30 %), as well as MUM1 hypoexpression (cut off 30 %) were significantly correlated with better OS (*p* < 0.05). In addition, high levels of BCL6 were significantly associated with longer disease free survival (DFS) (*p* = 0.042).

**Conclusion:** Our results support the prognostic value of GCB and nonGCB immunohistochemical profiles in DLBCL and they suggest the prognostic significance of CD10, BCL6 and MUM1 immunoexpression. In addition, the correlation of BCL6 with DFS indicates its predictive role.

**PS-11-009**

**Anaplastic lymphoma kinase-positive large B cell lymphoma with plasmablastic differentiation in the oral cavity: Report of a case**

A. Galzerano ^*^, M. Chorão, M. Silveira, J. Cabeçadas

^*^CHLO, Pathology, Lisbon, Portugal

**Results:** We report a case of an Anaplastic lymphoma kinase (ALK)-positive diffuse large B-cell lymphoma with plasmablastic differentiation of the oral cavity, in a 60 year-old man. ALK-positive diffuse large B-cell lymphoma is a rare variant of diffuse large B-cell lymphoma with distinctive phenotypic and cytogenetic characteristics, that represents less than 1 % of all B-cell lymphomas; most are lymph node based, with few reported in the oral cavity. The patient presented with foreign body sensation and loss of sensibility at deglutition for 1 year, without B symptoms. A mass in the right tonsil was clinically detected and biopsied. Histological analysis showed diffuse infiltration by large plasmablastic cells with expression of CD79a, CD138, CD30 (10 %), cIgA and a granular cytoplasmatic positivity for ALK but with no expression of CD20, PAX-5, CD3, BCL-2, EMA, CD56 and EBER. FISH showed a break in the ALK gene. Clinical and imaging staging failed to show dissemination.

**Conclusion:** Although the majority of cases thus far described are high stage lesions, ours seems to be low stage. The diffuse growth pattern and the plasmablastic morphology cause difficulties in the differential diagnosis with plasmablastic lymphoma in this extranodal location.

**PS-11-010**

**Extracavitary variant of Primary Effusion Lymphoma (PEL): A case report with nodal involvement**

D. Gonçalves ^*^, R. Ilgenfritz, J. Cabeçadas, M. J. Brito

^*^Hospital Garcia de Orta, Serviço de Anatomia Patológica, Almada, Portugal

**Objective:** Rare human herpesvirus type 8 (HHV8)-positive lymphomas with features similar to PEL can present as tumor masses in the absence of cavity effusions. These neoplasms are considered to represent an extracavitary or solid variant of PEL, and occur more commonly in extranodal sites. Here, we report a case of extracavitary PEL involving lymph nodes.

**Method:** A 37-year-old HIV-positive man with AIDS and a pre-existent Kaposi sarcoma of the skin presented with generalized lymphadenopathy of recent onset. A cervical lymph node biopsy was performed with histological, immunohistochemical and in situ hybridization studies.

**Results:** The lymph node biopsy showed a partial infiltration of cells with blastic morphology. These cells were positive for CD45, CD38 and HHV8, while they were negative for CD20, CD79a, CD138, CD2, CD3, CD5, CD30 and EMA. Epstein-Barr virus (EBER in situ hybridization) was also positive. These findings supported the diagnosis of an extracavitary variant of PEL. The patient passed away 2 months after the confirmation of the diagnosis.

**Conclusion:** The extracavitary variant of PEL is a rare lymphoma similar clinically, morfologically and immunophenotypically to classic PEL. Awareness of the existence of this variant and assessment for HHV8 infection are essencial for correct diagnosis.

**PS-11-013**

**Transformation of follicular lymphoma into CD30 large B-cell lymphoma: Study of 3 cases**

C. Meléndez ^*^, F. Climent, M. R. Taco, M. Araya, S. Mercadal, H. A. Florez, E. Condom

^*^Bellvitge Hospital, Pathology, L’hospitalet De Llobregat, Spain

**Objective:** The histologic transformation of follicular lymphoma(FL) to a higher-grade lymphoma, most commonly diffuse large B-cell lymphoma, is a well-described phenomenon. However, although the transformation to CD30+ large-cell lymphoma, it’s rare.

**Method:** The clinical, pathological, immunophenotypical and molecular findings of FL cases with transformation to CD30+ large-cell lymphoma were reviewed.

**Results:** Our series comprised 1 man and 2 women with a median age of 69 years(range: 62–77). One patient had FL grade 1–2 and 2 patients had grade 3A. The pattern of FL was follicular in 2 cases and follicular and diffuse in 1 case. The transformation was found in sequencial biopsy specimen in 1 case and coexisted in 2 cases. The transformation pattern was diffuse(2/3), follicular(2/3) and sinusoidal(3/3). The lymphocytes were anaplastic in the transformation and were CD30+(3/3), CD20+(2/3), CD79a+(3/3), CD10+(2/3), Bcl-6+(2/3) and Bcl-2+(2/3). T-cell markers, CD15 and EBER were negative. Translocations (t[14;18] [q32;q21]) was present in 2/3, the other one had wins/translocation of IGH gen but normal bcl2.

**Conclusion:** This unusual form of transformation must be recognized and indicates the need to consider antecedent FL in any B-cell lymphoma with anaplastic cytologic features.

**PS-11-014**

**EBV-positive extranodal marginal zone lymphoma of mucosa-associated lymphoid tissue in a HIV-positive paediatric patient**

A. Carstens ^*^, G. Pillay

^*^AMPATH, N1 City, South Africa

**Objective:** The case report describes a 7 year HIV-positive girl presenting with a MALT lymphoma in the antrum of the stomach. EBER positivity can be demonstrated in numerous lymphoma cells. Malt lymphomas have been described in patients with HIV infection, but are typically EBV negative.

**Method:** All immunohistochemical studies were performed on a Dako autostainer Link 48 using Dako antibodies. EBER was preformed on a Ventana Benchmark XT using a Ventana Inform EBER (Ebstein Barr Virus Early RNA) probe

**Results:** HIV viral load at time of the biopsy–36 876 copies/ml. Bone marrow not involved by MALT lymphoma. A diffuse infiltrate of lymphocytes with the appearance of centrocytes with scattered centroblasts are focally present in the biopsy. Numerous lymphoepithelial lesions are present. The adjacent mucosa shows slight chronic inflammation and a fibrinopurulent exudate consistent with ulceration. No Helicobacter pylori can be found. Immunohistochemistry shows diffuse CD20 and BCL-2 positivity in the lymphoma cells with scattered CD3 and CD5 T-cells. The lymphoma cells do not stain with CD10 or CD5.

**Conclusion:** Gastric MALT lymphomas are normally associated with H.pylori infection. EBV-positive MALT lymphomas have been described in a posttransplant setting. This case illustrates an EBV-positive gastric MALT lymphoma in a paediatric HIV- positive patient.

**Diffuse infiltrate of centrocyte like cells with lymphoepithelial lesions:** 
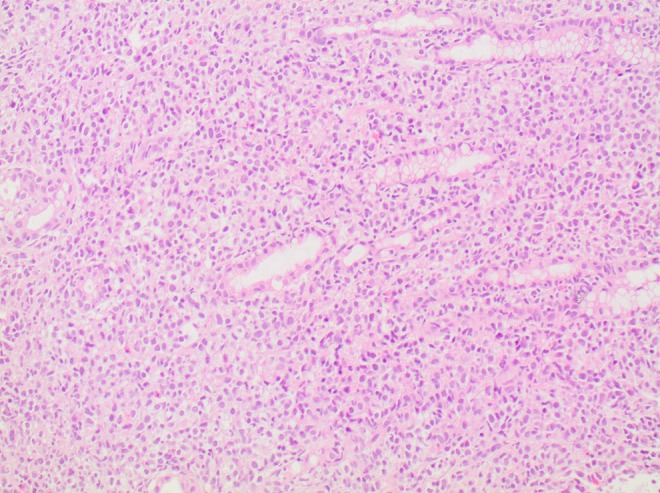


**PS-11-016**

**Primary B lymphoma of the uterine cervix**

L. Santos^*^, D. A. Portela, J. O. Gomes Santos, J. O. Ibiapina, A. C. Cunha

^*^Federal University of Piaui, Dept. of Pathology, Teresina, Brazil

**Objective:** Diffuse large B-cell lymphoma is the most common type of non-Hodgkin lymphoma, with about 31 % of incidence. It is an aggresive neoplasia that, in most cases, does not present any known risk factors.

**Method:** The authors hereby present a rare case of diffuse large B-cell lymphoma (CD20+) involving primarily the female genital tract.

**Results:** MGSV, 41 years old, asymptomatic, presented a bleeding tumoral lesion in the cervix during routine gynecological exam. Oncotic colpocytology demonstrated ASCUS and incisional biopsy showed HSIL. Conization was indicated, and it showed extense infiltration by malignant neoplasia constituted by diffuse of small hypercromatic cells, positive for CD20 and CD45, confirming that was a diffuse CD20+ B lymphoma. Howewer, there was a significant worsening of the clinical state with major bleeding, so urgent hysterectomy was indicated. The study of the surgical specimen (Fig. 1) showed solid compact tumoral lesion infiltranting the entire cervix wall and compromising parametriun and vaginal wall without lymph nodes disease. At post-op, the patient evolved well and the MRI didn’t show any lymphadenopathy. The patient was referred to oncologic treatment.

**Conclusion:** Extra-nodal lymphomas are poorly understood entities and the involvement of unusual site such as the female genital tract, specially the cervix, without nodal disease is even rarer.

**Macroscopy view of lymphoma cervix:** 
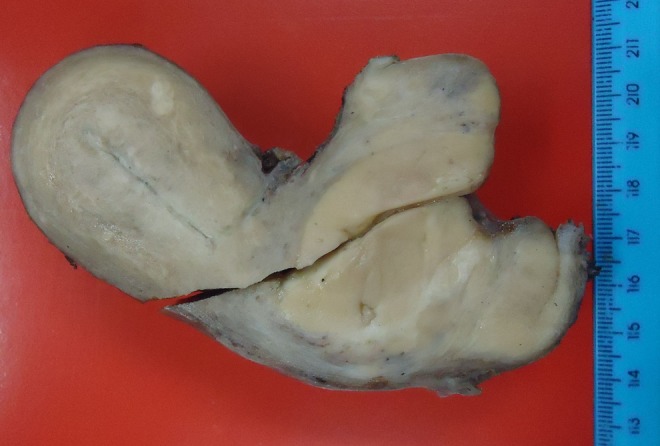


**PS-11-018**

**B and T lymphocyte attenuator expression in mature B cell lymphomas**

P. Trougouboff ^*^, H. Kreizman-Shefer

^*^Ha’emek Medical Center, Dept. of Pathology, Afula, Israel

**Objective:** B and T lymphocyte attenuator (BTLA) is a lymphocyte inhibitory receptor mainly expressed on several subsets of T and B lymphocytes. This study assess the BTLA expression in mature lymphoma B cells

**Method:** BTLA expression is examined for the first time on formalin fixed and paraffin embedded tissue sections of mature B cell non-Hodgkin’s lymphomas (253 cases), using a newly developed monoclonal antibody. The sections were studied by single immunochemistry and selected positive and negative cases were also examined by double immunochemistry with BTLA and BCL6 or PAX5 and double immunofluorescence with BTLA and CD20

**Results:** BTLA is highly expressed in chronic lymphocytic leukemia/small lymphocytic lymphoma. In contrast, BTLA expression is nearly absent in mantle cell lymphoma and is not expressed in any of the follicular lymphoma cases of all grades. In marginal zone lymphoma, diffuse large B cell lymphoma, and Burkitt’s lymphoma, a minority of the cases exhibit expression of BTLA.

**Conclusion:** This study demonstrates the value of BTLA expression as an additional tool in the panel of immunochemical markers used for the differential diagnosis of mature B cell lymphomas in FFPE tissue sections.

**BTLA expression in CLL/SLL and in MCL.:** 
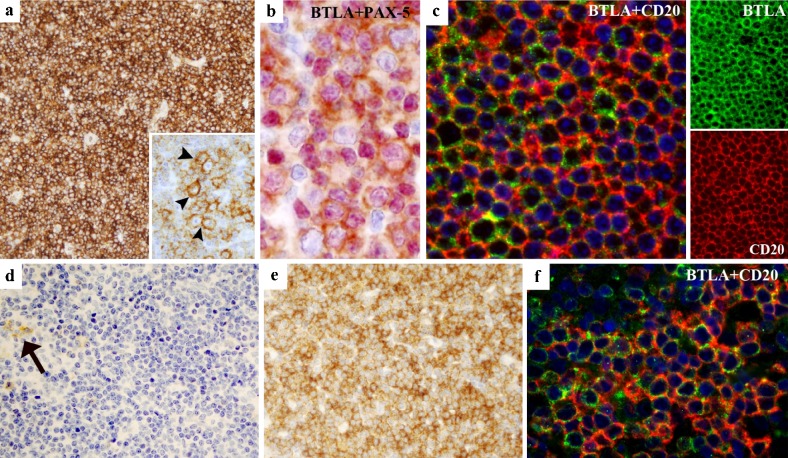


**PS-11-020**

**Focal adhesion kinase is expressed in a subset of mantle cell lymphoma and contributes to survival and growth of mantle cell lymphoma cells**

E. Drakos ^*^, K. Psatha, V. Sinatkas, A. Eliopoulos, H. Papadaki, G. Z. Rassidakis, E. Patsouris, E. N. Stathopoulos

^*^University of Crete, Medical School, Dept. of Pathology, Heraklion, Greece

**Objective:** Focal adhesion kinase (FAK), a non-receptor tyrosine kinase mediating scaffolding signaling, is involved in tumor progression of various carcinomas. Recently, FAK has been shown to be expressed in various types of B-cell non Hodgkin lymphomas, including mantle cell lymphoma (MCL). However, the functional significance of FAK in MCL is unknown.

**Method:** We investigated FAK expression in MCL cell lines and tumors by western blot analysis and immunohistochemistry. The biologic effects of pharmacologic inhibition of FAK in MCL cells were investigated in vitro by MTT assay, trypan blue and Annexin-V staining.

**Results:** FAK was expressed in 65 % of MCL tumors (15/23), and one out of 4 MCL cell lines. Treatment with the FAK-specific inhibitor PF-573 228 resulted in significant reduction of cell growth and apoptosis induction, specifically in MCL cells expressing FAK, while had minimal effects on FAK-negative MCL cells. In addition, nongenotoxic activation of p53, a known transcriptional regulator of FAK, accomplished by nutlin-3a treatment, resulted in downregulation of FAK in MCL cells. Combined p53 activation and FAK inhibition resulted in enhanced cytotoxicity of MCL cells expressing FAK.

**Conclusion:** FAK is expressed in a subset of MCL tumors and may contribute to survival and growth of MCL cells.

**PS-11-021**

**Composite mantle cell lymphoma and Hodgkin lymphoma in two different anatomic locations**

T. Koletsa ^*^, A. Cheva, L. Sakkas, G. Karkavelas, I. Kostopoulos

^*^Medical School AUTH, Dept. of Pathology, Thessaloniki, Greece

**Objective:** Coexistence of mantle cell lymphoma and Hodgkin lymphoma is an uncommon event with only limited reported cases in the literature.

**Method:** A 69-year-old male patient presented with lumbal pain, cervical and maxillary lymphadenopathy. MRI showed a paravertebral tumor on T3/T4. Neurosurgical tumor excision was performed and the specimen along with a left maxillary lymph node was sent for histologic examination.

**Results:** Hematoxylin and eosin stained sections of both specimens revealed infiltration by neoplastic population. This population consisted of medium-sized lymphoid cells with scant cytoplasm and round or ovoid nuclei with indistinct nucleoli, having the following immunophenotype: CD45+, CD20+, CD79a+, PAX5+, BCL2+, cyclinD1+, CD5−, CD3−, CD23−, BCL6−, CD10−, MUM1−, CD30−, CD15−, EMA−, ALK-1−, LMP1−. Among these neoplastic cells, large cells were diffusely observed, having the morphology and the immunophenotype of Hodgkin and Reed-Sternberg cells (PAX5+, CD30+, CD15+, CD45−, CD79a−). PCR analysis revealed IGH monoclonality. On FISH investigation the translocation t(11;14) was detected. No bone marrow involvement was observed.

**Conclusion:** The interesting of this case rests in the presence of the double neoplastic population in two different anatomic locations. The question raises in such cases is whether this coexistence is coincidental or it is due to a clonal relationship.

**PS-11-022**

**An extra-ordinary case of composite B-cell chronic lymphocytic leukemia and mantle cell lymphoma in cervical lymph node**

I. Iacovidou ^*^, G. Chinari, G. Mitropoulou, G. Galanopoulos, V. Chatziantoniou, M. Kotsopoulou

^*^Metaxa Cancer Hospital, Dept. of Pathology, Piraeus, Greece

**Objective:** We present a very rare case of B-cell chronic lymphocytic leukemia (B-CLL) and mantle cell lymphoma (MCL) ina cervical lymph node biopsy specimen.

**Method:** A 72-year-old Caucasian woman, with a 10 years medical history of B-CLL Binet stage A, without treatment, presented with generalized lymphadenopathy and splenomegaly. Histological examination revealed complete infiltration of the lymph node by a composite neoplasm with diffuse architecture. 40 % of the of the neoplastic population was composed of small lymphocytes mature type, while the rest consisted of medium and large size cells arranged either in solid nests or in diffuse pattern with prominent nucleoli, dense nuclear chromatin and frequent mitotic figures.

**Results:** Both cell populations were immunoreactive for LCA, CD5, CD20, CD79a, BSAP and TCL1 while they were negative for EMA, BCL6, CD3 and CD30. The small cell size neoplastic population was also CD23(+), CyclD1(−) and MIB1<10 %, immunophenotype compatible with B-CLL. The large size population was also CyclD1(+), CD23(−) and Mib1>50 %, immunophenotype compatible with MCL of pleiomorphic variant.

**Conclusion:** The combination of B-CLL and MCL is extremely rare with only four cases reported in the literature.

**PS-11-023**

**Infectious mononucleosis mimicking lymphoma: A trap for the unwary**

P. Dabir ^*^, S. Hamilton-Dutoit

^*^Aarhus N, Denmark

**Objective:** The clinical diagnosis of Epstein-Barr virus (EBV) associated infectious mononucleosis (IM) is clear-cut, rarely necessitating biopsy. Atypical cases may, however, closely resemble lymphoma, both clinically and histologically—an important potential pitfall for the unwary pathologist. We report 2 cases of IM mimicking lymphoma.

**Method:** Two male patients (17 and 21 years) presented with systemic symptoms and large, ulcerating masses in the pharyngeal tonsils and the nasopharynx, respectively. Clinically, lymphoma was suspected in both.

**Results:** Histological examination revealed similar polymorphic atypical lymphoproliferations with extensive soft tissue infiltration, including large bizarre Reed-Sternberg-like cells. Immunohistochemistry revealed a mixture of highly proliferating B and T cell lymphocytes including many CD30-positive cells. In spite of the malignant morphology, a diagnosis of acute EBV-associated infectious mononucleosis was made. This was confirmed by in situ hybridization for EBV-encoded RNA (EBERs) and by immunohistochemistry for EBV antigens LMP1-4 and EBNA2 (EBV latency III pattern). Serology confirmed acute EBV infection and both patients recovered well.

**Conclusion:** Care should be taken, especially by the novice pathologist, when asked to confirm a clinical diagnosis of lymphoma in young patients, particulary when faced with malignant looking lymphoproliferations occurring in unexpected locations. Once the possibility of acute IM is considered, the histological diagnosis is straightforward.

**PS-11-024**

**Mouse xenograft models of human aggressive non-Hodgkin lymphomas using primary cells**

R. Jaksa ^*^, P. Klener, M. Klanova, L. Lateckova, M. Vokurka

^*^UK 1st Faculty of Medicine, Institute of Pathology, Prague, Czech Republic

**Objective:** Aggressive non-Hodgkin lymphomas comprise diverse B- and T-cell malignancies. Animal xenograft models of aggressive NHL using primary cells are scarce.

**Method:** 5–40 × 106 primary lymphoma cells obtained from peripheral blood, bone marrow, malignant ascites or infiltrated lymph node of patients with NHL were tail-vein injected into 2Gy irradiated immunodeficient NSG mice (NOD-SCID-Gamma). Infiltration of selected murine organs (bone marrow, spleen, liver, kidneys and brain) was analyzed by immunohistochemistry using anti-human antibodie CD45, CD20,cyclin D1,CD3,CD7,CD30 and Ki-67.

**Results:** Tumor engraftment was achieved in 3 out of 4 primary DLBCL, 7 out of 12 primary MCL, and 1 out of 1 primary tCTCL samples. The mice were analyzed since day 17 to day 60 after xenografting. The principal site of engraftment for all tested samples appeared to be the spleen with infiltration with human lymphoma cells. Proliferation rate of engrafted lymphoma cells ranged from 30-near 100 %. Inconstant infiltration of other murine organs with human lymphoma cells was observed, including bone marrow, liver and kidneys, but not brain.

**Conclusion:** Mouse models of human aggressive NHL provide a valuable tool for the study of disease biology and for preclinical assessment of experimental treatment approaches including agents that cannot be properly tested in vitro.

**PS-11-025**

**Primary anaplastic large cell lymphoma, ALK negative, of the liver: A case report**

K. Koulia ^*^

^*^General Hospital Tzaneio, Dept. of Pathology, Piraeus, Greece

**Objective:** Primary extranodal lymphomas of the liver are notably rare. A proportion of cases are associated with infection with hepatitis C,B,HIV,EBV or primary biliary cirrhosis.

**Method:** We report a case of a 45 year old man who presented with abdominal pain and weight loss. Physical examination revealed an enlarged liver but ascites, jaundice, splenomegaly and peripheral lymphadenopathy were absent. Laboratory studies showed elevated hepatic enzymes such as alkaline phosphatase,LDH and SGOT. Tumor marker CEA and AFP were normal. Abdominal Computed Tomography (CT) revealed multiple hypodense lesions in both lobes of the liver but no sign of lymphadenopathy.

**Results:** Liver biopsy examination confirmed a diagnosis of non Hodgkin’s Anaplastic large cell lymphoma of T phenotype. The tumour consisted of a population of medium to large sized cells with irregular nuclei. Immunohistochemically the neoplastic cells were positive for CD43, CD4, CD2, CD3, UCHL1, CD30, CD5, granzyme B and negative for CD20, CD79a, CD10, EBV, CD15, CEA, EMA, CD56, CD57, TIA-1 and ALK-1. Among the tumour cells there were non neoplastic cells consisted of small lymphocytes and histiocytes. Bone marrow biopsy did not reveal lymphomatous involvement.

**Conclusion:** Primary lymphoma of liver is rare. The diagnosis is accomplished by immunohistochemical studies that allowed the accurate identification of the disease. Since the prognosis relates to the specific disease entity.

**PS-11-026**

**Cutaneous intravascular anaplastic large T-cell lymphoma: A case report and literature review**

F. Menezes ^*^, R. Azevedo, A. Rodrigues, R. Henrique

^*^Portuguese Institute of Oncology, Dept. of Pathology, Porto, Portugal

**Objective:** Report a case of a 59-year-old male, with multiple small cutaneous nodules in the trunk evolving for several months. Extensive staging investigation disclosed that the disease was limited to the skin. Biopsy of the lesions was performed.

**Method:** Hematoxylin-eosin stain and immunohistochemistry was performed to characterize the neoplastic lesion. Clinicopathological features of this case, and relevant literature, were reviewed.

**Results:** Histologically, neoplastic cells formed nodular, solid, poorly cohesive structures, with central comedo-like necrosis, lined by endothelial (CD34+) cells, limited to the dermis. Neoplastic cells were immunoreactive for CD45, CD2, CD3, CD5, CD30 and CD4, and lacked cytokeratins, EMA, pS100, ALK, CD20, CD8, CD56 and PAX5. In situ hybridization for EBV was negative.

**Conclusion:** These findings are consistent with primary cutaneous intravascular anaplastic large T-cell lymphoma (CIV-ALTL). Whereas intravascular T-cell lymphomas are mostly of cytotoxic-T/NK phenotype (often EBV-related), rarely present cutaneous involvement and follow an aggressive course, CIV-ALTL, in contrast, is circumscribed to the skin and follows an indolent clinical course. This exceptionally rare entity, with only a handful of cases reported thus far, poses a diagnostic challenge with cutaneous metastases or adnexal carcinoma. Its recognition as a distinct clinicopathological entity should allow for adequate diagnosis and therapy.

**PS-11-027**

**Alk-positive anaplastic large cell lymphoma presenting as an endobronchial tumour**

E. Mejia ^*^, G. Tapia, P. Zuluaga, S. Marti, M. Martin-Cespedes, J. L. Mate

^*^Hospital Germans Trias i Pujol, Pathology, Badalona, Spain

**Objective:** ALK-positive anaplastic large-cell lymphoma (ALCL) is a T-cell lymphoma that appears more frequently in childhood. Most patients present in advanced stages, with generalized lymphadenopathy and extranodal involvement, especially of the skin. Presentation of ALCL as an endobronchial tumour is extremely rare.

**Method:** A 26-year-old male presented with respiratory symptoms, anorexia and popliteal and gluteal pain. A parahilar tumour was seen on chest X-ray. Flexible bronchoscopy demonstrated an endobronchial mass, which was biopsied. Computed tomography revealed multiple lymphadenopathies in the retroperitoneum, pelvis and femoral region. A magnetic resonance demonstrated a diffuse lesion involving the gluteus and semitendinous muscle.

**Results:** The endobronchial tumour consisted of a diffuse proliferation of large, discohesive cells with abundant cytoplasm and irregular, eccentric nuclei, some of them kidney-shaped. Mitotic figures were frequent. There was positivity for CD4, CD30 and ALK, whereas CD3, CD20, CD79, CD15, cytokeratins and EBER were negative. FISH showed an ALK translocation.

**Conclusion:** ALCL may appear in atypical locations and, therefore, must be considered in the differential diagnosis of undifferentiated tumours of any site in children and young adults. A high degree of suspicion is necessary, since ALCL may be negative for the most used immunohistochemistry panels, including T-cell and B-cell markers.

**PS-11-028**

**Angioimmunoblastic T-cell lymphoma: Report of four cases and review of possible diagnostic problems and pitfalls**

K. Kamaradova ^*^, M. Nova, M. Cegan

^*^Teching Hospital, Fingerland’s Dept. of Pathology, Hradec Kralove, Czech Republic

**Objective:** Angioimmunoblastic T-cell lymphoma (AITL) is a distinct type of peripheral T-cell lymphoma recognized separately in current WHO classification. It has quite distinguishing clinical background and hallmark histological features which, if not considered, can lead to a misdiagnosis.

**Results:** We report four cases of AITL showing effaced lymph node architecture with features of typical AITL that includes neoplastic cells of follicular helper T-cell (TFH) origin with hyperplasia of high endothelial venules and follicular dendritic cells with attenuated B-zone and dispersed large atypical EBV-positive cells.

**Conclusion:** In spite of histological criteria there are possible diagnostic pitfalls. First, the initial or interfollicular involvement can lead to an incorrect diagnosis of paracortical hyperplasia or Castleman disease. Cases with prominent EBV+ cells can be mistaken for classical Hodgkin lymphoma. Phenotype of TFH cells can mislead to the diagnosis of follicular lymphoma, or it can be classified as a T-zone and follicular variant of PTCL, U. The latter two entities belong to a recently described group of T-cell lymphomas with TFH phenotype and can show overlapping features with AITL. Since AITL is an aggressive T-cell lymphoma with survival less than 3 years its accurate diagnosis is important. Supported by Charles University project PRVOUK P37/11

**PS-11-029**

**Extranodal T/NK-cell lymphoma, nasal type, associated with a pseudo-carcinomatous epithelial hyperplasia: A case report and review of the literature**

A. Ben Lakhdar ^*^, O. Casiraghi, J. Bosq

^*^Gustave Roussy, Pathology, Villejuif, France

**Objective:** Extranodal NK/T-cell lymphoma, nasal type, is an extranodal lymphoma with a cytotoxic phenotype associated with EBV, which has a broad morphologic spectrum with frequent necrosis and angioinvasion. It most commonly involves the midfacial region, especially the nasal cavity but also other extranodal sites.

**Method:** We present the case of a 62-year-old man with a necrotic and ulcerative polypoid mass of the nasal cavity.

**Results:** A biopsy was performed which showed a diffuse infiltrate of medium to large-sized lymphoid cells, with slightly irregular nuclei and moderate polymorphism, associated with a pseudo-carcinomatous epithelial hyperplasia. The immunophenotype of tumor cells was CD45+, CD56+, CD4+, TiA1+, CD30+, CD20−, CD3−, CD5−, CD8−, and LMP1−. A diagnosis of Extranodal T/NK-cell lymphoma, nasal type was therefore established. CT of the chest, abdomen and pelvis as well a bone marrow biopsy showed no other localisation.

**Conclusion:** The patient received combination chemotherapy and sequential radiotherapy initially and when a local recurrence occured 5 years later. The patient is still followed in our center without sign of disease for 5 years.

**PS-11-030**

**Primary cutaneous gamma-delta-T-cell lymphoma mimicking granulomatous panniculitis**

T. Koletsa ^*^, A. Patsatsi, E. Georgiou, D. Sotiriadis, G. Karkavelas, I. Kostopoulos

^*^Medical School AUTH, Dept. of Pathology, Thessaloniki, Greece

**Objective:** Primary cutaneous -T-cell lymphoma (−TCL) is a rare lymphoma that can be accompanied by granulomatous reaction on histological grounds, raising diagnostic problems.

**Method:** A 25 year old female presented with an 1 year history of subcutaneous nodules on the arms, thighs and lower legs. The old lesions remained palpable and evident due to hypopigmentation, while newer lesions showed superficial erosions. Along with the presentation of new nodules, resistant fever and leucopenia raised the suspicion of a hematological disorder.

**Results:** A biopsy specimen from a lesion revealed epithelioid histiocytes forming several glanulomas on dermis and subcutis, positive to CD68 antigen. Among them there were atypical medium-sized lymphoid cells. Immunohistochemical analysis revealed the following immunophenotype: CD45RO+, CD3+, CD2+, CD56+, TIA-1+, Granzyme B+, perforin+, MUM1-−/+, CD20−, CD45RA−, PAX5−, CD5−, CD7−, CD4−, CD8−, CD30−, CD15−, CD68−, LMP1−, TCR−. In situ hybridization for Epstein-Barr virus was negative. Polymerase chain reaction showed TCRB and TCRG rearrangements. The diagnosis of primary cutaneous -TCL was made. No bone marrow involvement was detected. The patient had a rapidly progressive clinical course.

**Conclusion:** In such cases, clinicopathologic correlation, extensive immunohistochemical and even molecular analysis are essential in order to distinguish lymphomas from inflammatory or reactive disorders.

**PS-11-031**

**Expression profiling of miRNAs and their target genes in peripheral T cell lymphoma, not otherwise specified**

J. Zhang ^*^, M. Li, H. Liu, N. Lv

^*^306 Hospital of PLA, Dept. of Pathology, Beijing, People’s Republic of China

**Objective:** To study the expression profile of microRNAs (miRNAs) in peripheral T cell lymphoma, not otherwise specified (PTCL-NOS) and to explore the molecular characteristics.

**Method:** TaqMan low density array was used to assess the expression level of 754 miRNAs in 6 cases of PTCL-NOS and 3 cases of reactive lymphoid hyperemia as control. We predicted target genes for significantly and differentially expressed miRNAs with Targetscan and miRanda software. Expression patterns of 3 miRNAs were further analyzed in 15 cases of PTCL-NOS and 10 benign reactive lymphoid hypeplasia.

**Results:** Eight miRNAs showed statistically significant difference in PTCL-NOS and benign reactive lymphoid hyperemia. miR-886-3p,miR-511,miR-1291,miR-572,miR-27a-3p,miR-25-3p and miR-886-5p were significantly over-expressed in PTCL-NOS while miR-182-5p was significantly under-expressed (*P* < 0.05). Target genes prediction showed that 1,646 candidate genes involved in the pathogenesis and progression of PTCL-NOS. Further GO and Pathway analysis found these genes significantly focused on 63 GO terms and 61 pathways.

**Conclusion:** Eight miRNAs are aberrantly expressed in PTCL-NOS, which might be involved in the molecular regulation in the pathogenesis of PTCL-NOS; the underlying mechanism is relevant to many target genes and complicated signaling pathways.

**PS-11-032**

**POEMS syndrome suggested by 2 biopsies: A case report**

M. Rito ^*^, J. Costa Rosa, J. Cabeçadas

^*^IPO de Lisboa Francisco Gentil, Serviço de Anatomia Patológica, Portugal

**Objective:** We report a case of POEMS syndrome (polyradiculoneuropathy, organomegaly, endocrinopathy, monoclonal plasma cell disorder and skin changes), a rare paraneoplastic syndrome due to an underlying plasma cell neoplasm, in a 51-year-old man. Its diagnosis is based on clinical and laboratory features and if not considered is easily missed. Its importance relies on the fact that treatment and overall survival differs from standard plasma cell dyscrasias.

**Method:** A 51-year-old man was investigated for a latero-cervical mass, odynophagia and hypoacusis, suggesting an occult neoplasm. CT scan revealed skull base lytic lesions and cervical adenopathies.

**Results:** Biopsy of a lytic lesion was consistent with multiple myeloma and together with a nodal biopsy showing a Castleman disease prompted us to suggest the presence of a POEMS syndrome. Further investigation revealed polyneuropathy, organomegaly (spleen, liver, lymph nodes), bone marrow involvement with plasma cells, thrombocytosis, arterial thrombosis and weight loss, fulfilling the diagnostic criteria. The patient was treated with radiotherapy of the skull lesions with clinical improvement.

**Conclusion:** POEMS syndrome is a rare syndrome whose diagnosis is challenging, requiring awareness and a good clinical and laboratory investigation. As the diagnosis is based on several apparently unrelated features, the pathologist can have an important role suggesting the diagnosis.

**PS-11-033**

**Simultaneous occurence of low-grade B-cell lymphoproliferative disorders and monoclonal plasma cell proliferations: A report of three cases**

L. Marinos ^*^, G. Kanellis, E. Pouliou, T. Asimakopoulou, G. Kokkini, T. Papadaki

^*^Agios Stefanos, Attiki, Greece

**Objective:** To present three such cases in order to alert pathologists about this rare occurrence.

**Method:** Bone marrow biopsies(BMB) from three (3) patients (one male and two females with median age 74 years) with mantle cell lymphoma(MCL)(×2) and chronic lymphocytic leukemia(CLL)(×1)were studied morphologically(H&E stain) and immunohistochemically with a broad panel of antibodies.

**Results:** All three cases showed a variable percentage(10–70 %)of neoplastic B lymphocytic infiltration(BLI) in the context of MCL [CD20+PAX-5+CD5+CD23-Cyclin D1+SIg/CIgMD(κ or λ)+)]orCLL[CD20+PAX-5+CD5+CD23+Cyclin D1-SIgMD(λ)+],associated with an additional plasma cell monoclonal population(5–15 %) with a distinct from the BLI heavy and/or light chain expression [CD56+(2 cases)CD20-PAX-5-CD138+CIgA(κ)+(*x*2)orG(κ)+(x1)]

**Conclusion:** 1) The rare coexistence of low-grade B-cell lymphoproliferative disorders and monoclonal plasma cell proliferations emphasizes the need for a detailed immunophenotypic approach of BMB even in cases where involment by a NHL is straightforward. 2) ancillary studies(i.e. sequencing,interphase FISH) might be necessary to delineate the true nature of these entities. 3) a long follow up is required for determining the clinical significance of the MPC.

**PS-11-035**

**Multiple myeloma with involvement of the urinary bladder. A case report**

P. Xirou ^*^, D. Minotakis, G. Panselinas, B. Christoforidou, E. Katodritou, F. Patakiouta

^*^Theagenion Cancer Hospital, Pathology, Thessaloniki, Greece

**Objective:** We report a case of multiple myeloma (MM) with involvement of the urinary bladder.

**Method:** Our patient, a 74 years old man, was diagnosed with IgA/lamda MM 10 years ago. Iliac crest biopsy revealed a bone marrow infiltration of 60 % by moderately differentiated plasma cells. The patient received chemotherapy and responded well. Five and 7 years later he had two relapses, the latter manifesting with myeloma cast nephropathy (creatinin: 4.5). He continued chemotherapy and was stable until recently.

**Results:** Six months ago he presented with gross haematuria. Cystoscopy showed a 2 cm pedunculated nodule, protruding into the lumen. Biopsy was performed and showed infiltration of the bladder mucosa by moderately differentiated plasma cells with the same monotypic immunoglobulin.

**Conclusion:** MM is a systemic disease located mainly in the bone marrow in 90 % of cases. During the course of the disease, extramedullary spread is seen in 66 % of patients, whereby any organ may be affected. Urinary bladder involvement is extremely rare. The patient received one cycle of bortezomid/desamethasone and is doing well so far.

**PS-11-036**

**Littoral cell angioma: Morphologic and immunohistochemical features**

P. Katoch ^*^, P. Johansen

^*^Aalborg University Hospital, Patologisk Institut, Denmark

**Objective:** Littoral cell angioma(LCA) is a rare primary vascular tumor, unique to the spleen. It is believed to be derived from the lining cells of the splenic red pulp sinuses- the littoral cells, which show both endothelial and macrophage characteristics. We wish to present the morphologic and immunohistochemical features of 2 cases of LCA.

**Method:** Histopathological evaluation of splenectomy specimens

**Results:** Macroscopy: multiple cystic,spongy,& blood-filled relatively well circumscribed round nodules ranging from 0.5 cm to 4 cm. Microscopy: Spleen parenchyma was replaced by cystic areas showing anastomosing network of vascular spaces of varying sizes. The cells lining these spaces had features of tall endothelial like cells with a large nucleus and focally a small but distinct nucleolus. Mitotic figures and atypia in lining cells was not seen. Immunohistochemistry: The vascular nature of the lining cells was confirmed by CD31+ cells. CD34 was negative, while CD68(histiocytic marker) was positive. Contrary to normal sinus endothelium, the lining cells were CD8 negative. CD21 was focally positive.

**Conclusion:** Splenic vascular tumors may pose a diagnostic challenge. LCAs are benign tumors and should be differentiated from splenic hemangiomas, hamartomas and most importantly angiosarcomas. Features which help support diagnosis of LCA over angiosarcoma are lack of cellular atypia & mitosis, no complex anastomosing channels and immunohistochemistry as depicted in the image.

**Table:**
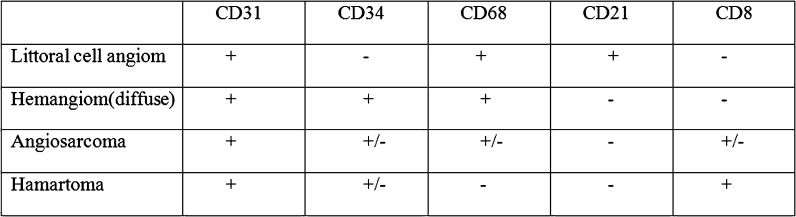

**PS-11-037**

**Systemic mastocytosis: Case report**

E. Kimiloglu ^*^, Z. Ozcan, N. Erdogan, A. Akyildiz Igdem, O. Mavis, T. Elibol

^*^Taksim’s Hospital, Dept. of Pathology, Istanbul, Turkey

**Objective:** Systemic mastocytosis is a rare disease of unknown etiology characterized by abnormal proliferation of tissue mast cells. The cutaneous manifestations were first described by Nettleship in 1869,in the form of urticeria pigmentosa. The skin is by far the most frequent site of involvement. Other organs commonly involved include the liver, bone marrow,spleen and lymph nodes, but virtually any organ or system can be affected.

**Method:** 49 years old male patient presented with hepatomegaly,massive splenomegaly, pancytopenia and diffuse cutaneous maculopapuler pigmentation. The bone marrow aspiration was dry. Tru-cut biopsy from liver and bone marrow were performed. Cutaneous punch biopsy was taken from the patient.

**Results:** There were diffuse mast cell infiltration more than 90 % of the bone marrow. Immunohistochemically the infiltration stained positive with both CD68 and CD 117. Histochemically the mast cells were positive by Giemsa and Toluidine Blue. The tru-cut biopsy from liver also showed mast cell infiltration in portal areas (more than 60/40HPF). There was mast cell infiltration (more than 40 mast cells/40 HPF) in the cutaneous punch biopsy. As a result the case was diagnosed as systemic mastocytosis.

**Conclusion:** We report the case here because of rarity and to remind the pathologists this entity.

**PS-11-038**

**A peculiar case of multiple neoplasia: Ovarian carcinoma, Systemic Mastocytosis and Hematologic Non-Mast Cell Disease (SM-AHNMD)**

I. Iacovidou ^*^, I. Kasselaki, F. Katis, M. Papazian, D. Papagiannopoulou, K. Megalakaki

^*^Metaxa Cancer Hospital, Dept. of Pathology, Piraeus, Greece

**Objective:** We present an extra-ordinary case of multiple neoplasia: a 65 year old female with Ovarian Carcinoma and metachronous Splenic Marginal Zone Lymphoma and Systemic Mastocytosis.

**Method:** A SMZL was diagnosed at the age of 50 after splenectomy. After several chemotherapy cycles she quits on 5th 2011. Three months later she presented with numerous painful subcutaneous nodules located in the abdominal wall, lumbar spine and lower limps, generalized lymphadenopathy and hepatomegaly. Laboratory control revealed anemia, leukocytosis, eosinophilia, monocytosis and thrombocytopenia. A femoral lymph node was sampled and histological examination showed infiltration by a Diffuse Large B-Cell Lymphoma (Transformation of MLZ to DLBCL).

**Results:** A bone marrow sample, 9 months later, was infiltrated by a neoplastic cell population [CD2(+), CD25(+), CD(68+), CD117(+), with numerous metachromatic cytoplasmic granules (GIEMSA)]. The diagnosis was systemic mastocytosis.

**Conclusion:** Systemic mastocytosis is characterized by clonal proliferation of abnormal mast cells that accumulate in one or more extracutaneous organs. A synchronous or metachronous AHNMD is diagnosed in 5–40 % of cases. The prognosis is variable, generally poor, due to the associated disorder, with a median survival of 2 years overall.

**PS-11-039**

**A case report of an extranodal (mesenteric) follicular dendritic cell sarcoma**

E. Moustou ^*^, K. Manoloudaki, E. Arkoumani, A. Tsavari, K. Koulia, D. Myoteri, T. Vasilikaki

^*^General Hospital Tzaneio, Dept. of Pathology, Piraeus, Greece

**Objective:** Follicular dendritic cell sarcoma (FDCS), is a rare neoplasm defined by neoplastic proliferation of follicular dendritic cells (FDC), and it is grouped with the histiocytic and dendritic cell neoplasms in the WHO classification. About 30 % of the tumors are found in extranodal sites including head and neck area, gastrointestinal tract, liver and spleen.

**Method:** We report a case of a 64 year old female who presented with a paraneoplastic oral pemphigus and an intraabdominal/mesenteric mass.

**Results:** Grossly, the radically resected tumor was multilobulated, encapsulated measuring 17 × 12 × 8 cm, gray to brown yellow in color and partially hemorrhagic and necrotic. Microscopically composed predominantly of spindle and ovoid cells with low mitotic rate in a whorled, storiform, fascicular and vaguely nodular pattern with a lymphocyte rich stroma. Focal mild pleomorphism, occasional multinucleated cells, areas of necrosis and hemorrhage was included. Immunohistochemistry was positive for vimentin CD21, CD23, HLA-DR, CD68, ki67 (0–20 %). Lack of expression of pankeratin CD1a, desmin, S100p, CD45, CD117, CD34, HMB45 allowed the differential diagnosis from other neoplasms and a diagnosis of FDC sarcoma was made.

**Conclusion:** Extranodal FDCS is a rare tumor. An accurate diagnosis is established based on tumor morphology and immunoexpression of one or more FDC associated markers.

**PS-11-040**

**A rare case of follicular dendritic cell sarcoma localised in mesentery: A case report**

I. Iacovidou ^*^, K. Manoloudaki, P. Giagkazoglou, E. Paliouri, G. Chinari, A. Chatzimarkou

^*^Metaxa Cancer Hospital, Dept. of Pathology, Piraeus, Greece

**Objective:** Follicular dendritic cell sarcoma (FDCS) is a rare malignant neoplastic condition with origin the dendritic cells of germinal centers of lymphoid follicles. Extranodal lesions occur in sites such as head, neck and gastrointestinal tract. Primary mesentery location is extremely uncommon.

**Method:** We report a case of mesentery FDCS, an extremely rare condition, which can easily be misdiagnosed, due to the scarcity of the lesion. A 66 year old female presented with a large, movable, painless, palpable mass in her left abdomen. Interoperative findings revealed a tumor originated from the small intestinal mesentery.

**Results:** Histopathology revealed ovoid and spindle cells with clear nuclei arranged in fascicles and whorls formating vague nodules. The dedritic cell origine was confirmed by the use of IHC stains such as CD21, CD23, CD35, CD68, HLA-DR and S-100. There was no evidence of metastatic disease. The tumor was radicaly resected and the patient received additional chemotherapy.

**Conclusion:** Mesentery FDCS can be easily under-recognized and misdiagnosed. The knowledge of this extraordinary entity and the performance of IHC establishes the diagnosis. It has to be treated as a high grade soft tissue sarcoma: radical resection followed by radiation and adjuvant chemotherapy.

**PS-11-041**

**Histiocytic sarcoma in a 56-year-old woman submitted to kidney transplant**

R. Dias ^*^, J. Palla Garcia, P. Aguiar, J. R. Vizcaíno

^*^Hospital de Santo António, Porto, Portugal

**Objective:** Histiocytic sarcoma is a rare neoplastic disease. To date a few hundred cases have been reported, with a wide range of clinical presentations and manifestations.

**Method:** We report the case of a 56-year-old woman, with history of HCV infection, and chronic kidney disease, submitted to kidney transplant in 1984 and since then under immunosuppression.

**Results:** The patient came to hospital with complains of a relenting growing, painless mass on her right front thorax. Ecography revealed a heterogenous highly vascularized mass (8 × 9 cm), with a necrotic centre and PET scan showed a large axillary mass, as well as multiple thoracic and abdominal nodules. Histological examination of the axilary mass showed a neoplasm composed of sheets of large, round to oval, cells with abundant eosinophilic cytoplasm, in an inflammatory background. The neoplastic cells had large pleomorphic nuclei with fine chromatin and prominent nucleoli. The immunohistochemical study was positive for CD45, CD68, CD163 and CD4 (+−) in tumour cells, thus suggesting a histiocytic sarcoma.

**Conclusion:** We present the case of a rare neoplasm in a transplant recipient. Although phenotypic features of histocytic sarcoma are relatively uniform, it is important to note that the diagnosis requires exclusion of more common neoplasms by immunophenotypic studies.
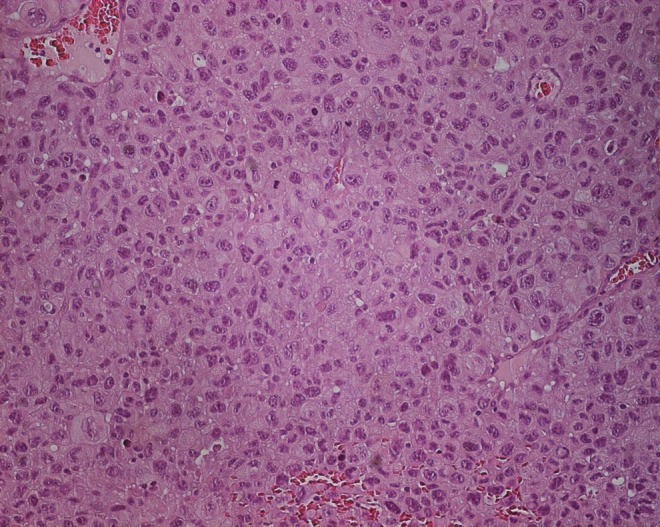


**PS-11-042**

**Involvement of uterine cervix as initial presentation in blastic plasmacytoid dendritic cell neoplasm lacking cutaneous lesion**

V. Cemerikic-Martinovic ^*^, N. Drndarevic, M. Dencic-Fekete, T. Martinovic, N. Andjelkovic, S. Mitrovic, P. Djurdjevic

^*^Beo-lab, Dept. of Pathology, Belgrade, Serbia

**Objective:** Blastic plasmacytoid dendritic cell neoplasm (BPDCN) is a rare and aggressive haematological malignancy derived from plasmacytoid dendritic cell precursors. Almost all patients show cutaneous manifestations, and >60 % show bone marrow involvement at initial presentation. It is exceedingly rare to diagnose BPDCN without a cutaneous lesion.

**Method:** We report a 66-year-old female who was diagnosed with BPDCN in the absence of cutaneous symptoms. Clinically she was presenting with uterine bleeding.

**Results:** Histopathology of a cervical biopsy specimen demonstrated a monomorphous infiltrate of small to medium-sized cells with blastoid morphology. Tumor cells predominantly occupy the lamina propria, sparing the epithelium. By immunohistochemistry tumor cells were positive for CD4, CD43, CD56 and CD123. Immunohistochemistry revealed minimal bone marrow involvement (CD123+; CD56+, CD4+). Cytogenetics analysis showed abnormal karyotype with monosomy of chromosome 16 and tetrasomy of chromosome 2. The patient refused chemotherapy and died of bone marrow failure few months after diagnosis.

**Conclusion:** This case is significant for rare BPDCN presenting with initial involvement of uterine cervix and bone marrow in the absence of characteristic cutaneous manifestations.

**PS-11-044**

**Concomitant Squamous Cell Carcinoma (SCC) and Acute Myelogenic Leukaemia (AML)in skin lesion**

I. Iacovidou ^*^, C. Valavanis, G. Galanopoulos, B. Chatziantoniou, O. Tzaida, M. Kotsopoulou

^*^Metaxa Cancer Hospital, Dept. of Pathology, Piraeus, Greece

**Objective:** SCC is the second most common skin neoplasia while myeloid sarcoma appears only in 10 % of AML patients. We present the simultaneous occurrence of these two histogenetically distinct neoplasms within the same skin lesion of the right cheek.

**Method:** An 84 year old male patient with an unclassified myelohyperplastic/myelodysplastic syndrome, diagnosed on bone marrow biopsy, presented with a brownish ulcerated skin lesion of the right cheek, 2.5 cm wide. The histology revealed an epitheloid and spindle cell neoplastic population with solid, trabecular and diffuse architecture, infiltrating the reticular dermis in composition with a second neoplastic population of medium to large sized cells, with vesicular nucleus, abnormal nuclear membrane, single or multiple nucleoli and intense mitotic activity.

**Results:** The epitheloid and spindle cell neoplasm expressed CKAE1-3 and CK 5–6, compatible with squamous invasive carcinoma, while the second neoplastic population expressed LCA, MPO, CD117, HLA DR, consistent with myeloid sarcoma-AML and the context of the acquainted hematological disease of the patient.

**Conclusion:** The concomitant occurrence of SCC and myeloid sarcoma -a tumor in tumor- is extremely rare. There is only a single case in the literature of such a composite tumor localized on the conjunctiva.

**PS-11-045**

**The chemical structure of erythrocytes in clinical models of early ageing**

T. Pavlova ^*^, K. Prashchayeu, N. Pozdnyakova, V. Bashuk, A. Nesterov

^*^BelSU, Dept. of Pathology, Belgorod, Russia

**Objective:** The aim is to provide the chemical structure of erythrocytes in clinical model of early aging.

**Method:** 46 patients from 38 to 54 years old with diseases associated with early aging (diabetes mellitus 2, arterial hypertension, coronary heart disease), 26 patients from 64 to 70 years old with same disease. Scanning electron microscopy, scanning probe microscopy.

**Results:** In the middle age the correlation (*p* < 0.05) between concentration of C (carbonium) and Cl (chlorum) and the level of polymorbidity take place: the proportional concentration of C in erythrocytes is increasing (from 45,90+0,11 % to 58,06+0,16 %) and the proportional concentration of Cl is decreasing. (from 5,55+1,00 % to 0,69+0,01 %). In the elders the situation is more catastrophically. Besides the changes same in middle age, in elders the negative trend of proportional concentration of O (oxygen) take place (*p* < 0,05). The level of proportional concentration of C is higher than in same situations in the middle age (*p* < 0,05).

**Conclusion:** As ageing as early ageing correlate with disbalance of C- and O-proportional concentration in erythrocytes. One part of this changes present pathogenic mechanisms, other part present sanogenic mechanisms.

Monday, 2 September 2013, 09.30–10.30, Pavilion 2

**PS-12 Poster Session History of Pathology**

**PS-12-001**

**The problem of causality in pathology**

A. Ivantsov ^*^, M. Kleshchev

^*^Petrov Research Institute, Dept. of Pathology, St. Petersburg, Russia

**Objective:** To identify the causes of unilateral examination of the problem of causality in pathology.

**Method:** A dialectical analysis of the world-view of “researcher”.

**Results:** The last two centuries medicine develops based on the philosophy of positivism, which has dignity (criterion validity of knowledge is not based on blind faith, but to the facts, which can be seen in the objective reality) and disadvantages (in the opinion of positivists the cause of science collect and organize facts, not to explain the cause-effect relationships in their set). Also, K. Gödel in 1931, proved that logically impeccable, rationally can prove everything that will be ordered, but will be true only that, which comes from the perfect objective true (incompleteness theorem).

**Conclusion:** The need to be free from the power of positivism in general. The study of causality is possible on the basis of subjective dialectic (a method of knowing the truth by asking leading questions, combining evidence and descriptive methods of developing new knowledge). The essence of the concept of “disease” (adaptation or failure of adaptation) can not be proved logically, but can be realized as a result of moral judgment, that practically confirmed in everyday life.

**PS-12-002**

**Infanticide or stillbirth? Forensic pathology in Norway 100 years ago**

G. C. Alfsen ^*^, L. Hernæs, C. L. Ellingsen

^*^Akershus University Hospital, Dept. of Pathology, Lørenskog, Norway

**Objective:** This year, Norway celebrate the 100th anniversary of womens suffrage. Everyday life for women 100 years ago was characterized by lack of control of reproduction. Clandestine childbirths, abandonment and infanticide was a large problem. Forensic examinations of suspected mothers and dead infants was a regular task for pathologists and general practitioners.

**Method:** 735 files from 1910 to 1912 in the archives of the National Forensic Commission were studied.

**Results:** Out of 350 forensic autopsies, 131 cases (37 %) dealt with the question of infanticide or abandonment (125) or illegitimate abortions (6). Of 136 clinical examinations, 28 (20 %) were performed on women suspected of illegitimate births. In autopsies, the hydrostatic lung test was used indiscriminately as proof for live birth, and 46 out of 62 positive floating tests were performed on partially decomposed bodies. Only one of the positive tests was assessed as false positive.

**Conclusion:** Due to birth control, legalized abortions and improved social conditions, the pattern of forensic examinations has changed dramatically since the early 1900s. Despite knowledge of the limitations of the hydrostatic test, the use was never questioned by the experts in the National Forensic Commission. Many innocent women were imprisoned for infanticide due to peremptory use of tests now recognized as obsolete.

**PS-12-003**

**“Omnium Anatomicorum Exactissimus”: The man who described “Glisson’s capsule”**

R. Henriques de Gouveia ^*^, G. Tralhão, G. Castanheira, C. Cordeiro, B. S. Silva, S. Ramos, L. Carvalho, F. Corte Real, D. N. Vieira

^*^INMLCF, I.P., Dept. of Pathology, Coimbra, Portugal

**Objective:** The eminent Dutch physician Boerhaave described “FRANCIS GLISSON” as the “Omnium Anatomicorum Exactissimus”. But who was this man? What does Science/Medicine owe him?

**Results:** Born in 1597 (Rampisham-Dorset,UK), admited at Cambridge Caius College (1617), got an Arts degree in 1620. Accepted at Oxford Medical Academy (1627), studied physics/medicine, getting the degree in Cambridge (1634). After admission at Physicians’ College (1634), became “fellow” (1635) and then “regius professor of physics” (1636) until his death. During the Civil War, moved to Colchester, practicing medicine. Afterwards, goes to London, where he develops important scientific activity at the Physicians’ College. He died on October 15th, 1677. His masterpieces are: 1) “De Rachitide” (1650); 2) “Anatomia Hepatis” (1654)–where he describes the membrane that covers the liver (Glisson’s Capsule) and hepatic internal anatomy -; 3) “Tractatus de Natura Substanti¦ Energetica” (1672); 4) “Tractatus de Ventriculo et Intestinis” (1676).

**Conclusion:** Being known the importance of “Glisson’s Capsule” in the mantainance of hepatic integrity, bleeding prevention, as an obstacle to liver abcess formation, …; and in an era where the preassure to abolish eponims from the nomina anatomica and the clinical practice is far from neglectable, the authors pay homage to the person who, with the XVIIth century technico-scientific resources, left us an anatomo-physiological legacy highly useful to medical and surgical daily practice.

Monday, 2 September 2013, 09.30–10.30, Pavilion 2

**PS-13 Poster Session Infectious Diseases Pathology**

**PS-13-002**

**Histopathological autoptic findings in 17 patients with novel influenza A (H1N1) pneumonia**

H. Skalova ^*^, C. Povysil, B. Goldova, R. Jaksa, J. Galko, L. Bauerova, K. Jandova, K. Nemejcova, M. Pirhalova

^*^General University Hospital, Inst. of Pathology, Prague, Czech Republic

**Objective:** In the years 2009 and 2010 a novel influenza A (H1N1) caused the first influenza pandemic after 41 years. We report 17 autoptic cases of patients who died between 2009 and 2013 with confirmed H1N1 influenza and underwent a post mortem examination. This group differs from the others reported in literature by having a higher age as well as a higher percentage of patients with pre-existing severe comorbidities. All patients developed atypical pneumonia with subsequent respiratory failure.

**Results:** We focus primarily on the findings in the respiratory tract. The most prominent feature observed was diffuse alveolar damage accompanied by intraalveolar haemorrhages and inflammatory infiltrate of variable extent. Less frequent features included cytopathic changes of pneumocytes, reactive changes of bronchial epithelium, intraalveolar fibrinous exudate, minor necroses, necrotizing bronchitis, focal granulation tissue and fibrosis in patients with longer survival. Some cases were complicated by superimposed bacterial and mycotic bronchopneumonia. In one case we found an extraordinary vascular change of uncertain origin.

**Conclusion:** Our group of patients slightly differs from those in other studies and case reports. By reporting them we wish to extend the number of described cases, which may contribute to a better understanding of the pathogenesis of novel influenza infection.

**Novel influenza A (H1N1) pneumonia:** 
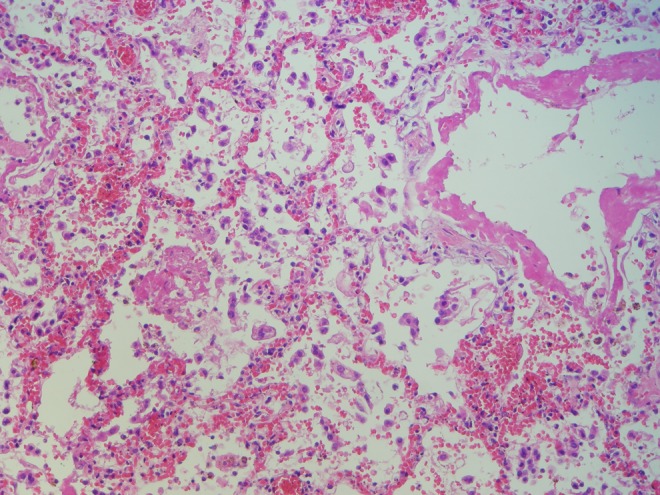


**PS-13-003**

**Anal squamous intraepithelial lesions: A HPV genotype study of 83 cases**

J. E. Moro-Rodriguez ^*^, B. García Espinosa, E. Álvarez Fernández

^*^Universidad Rey Juan Carlos, Histology & Anatomical Pathology, Alcorcón, Madrid, Spain

**Objective:** Human Papillomavirus (HPV) genotype distribution and co-infection occurrence was studied in anal specimens.

**Method:** A total of 83 abnormal specimens, from the Hospital General Universitario Gregorio Maranon of Madrid, were studied. These included 4 specimens with benign lesion (BL), 52 specimens with low-grade squamous intraepithelial lesion (LSIL), 24 specimens with high-grade squamous intraepithelial lesions (HSIL) and 2 specimens with invasive anal carcinoma (IC). HPV genotyping was performed using PCR amplification and reverse dot blot hybridization.

**Results:** We detected 33 different HPV types: 17 carcinogenic high-risk HPV types (HR HPVs), 2 probably carcinogenic high-risk HPV types (PHR HPVs) and 14 carcinogenic low-risk HPV types (LR HPVs). In 2 specimens the presence of an uncharacterized type (HPV X) were detected. The most frequent HPV genotypes found in all specimens were HPV 16 (9.8 %), 52 (8.9 %) and 43/44 (7.6 %). HPV 18 was only detected in 0.9 % of total number of virus detected in all lesions. Co-infections were found in 67.5 % of all kind of lesions. In the majority of cases were detected other concomitant infections mainly by the human immunodeficiency virus (HIV) in 90.4 %.

**Conclusion:** The presence of carcinogen high-risk genotypes in anus pathological samples is remarkable.

**PS-13-004**

**Macrophages in children: They do not impact AIDS progression more than CD4+ T cells**

F. Staniceanu ^*^, L. Nichita, S. Gheorghita, R. Chirculescu, A. Bastian, S. Zurac, C. Socoliuc, G. Micu, C. Rosculet, A. Streinu-Cercel

^*^Colentina University Hospital, Pathology, Bucuresti, Romania

**Objective:** Studies in animal models have suggested that high monocytes turnover may predict the rate of AIDS progression in HIV positive patients. Turnover of monocytes may be identified in tissue based on macrophages depletion.

**Method:** We analyzed 28 lymph nodes from HIV iatrogenically infected children in the first year of life who died between 1993 and 2000; the lymph nodes were harvested during autopsy; CD68 immunohistochemical stain was performed; we compared the number of macrophages counted on a microscope counting grid in lymph nodes from 15 children aged 1–5 years and from 13 children aged 6–12 years in correlation with blood CD4+T level; no patient had antiretroviral therapy

**Results:** Depletion of macrophages was not correlated with CD4+T cells level. In 6–12 years group 69.2 % of the patients had moderate CD4+T cells depletion (AIDS stage being classified based on recurrent/persistent bacterial infections) so macrophages could be incriminated in disease progression; in 1–5 ys group CD4+T cells depletion was severe (in 86.6 % of the patients) and macrophage number higher compared with 6–12 ys group (*p* < 0.03) suggesting a subsidiary role of macrophages in HIV progression.

**Conclusion:** Conflicting results suggest that, unlike animal models, macrophage depletion cannot be considered a direct marker of AIDS progression. Stăniceanu and Nichita have equally contributed

**PS-13-005**

**Morphological changes in tuberculosis, caused by different genotypes of mycobacteria**

V. Zinserling ^*^, V. Svistunov

^*^Med. Academy St. Petersburg, Research Institute of Phtysiop., Dept. of Pathology, Russia

**Objective:** Last decades are characterized by increase of lethality from tuberculosis and qualitative changes of its structure. Generalized forms with atypical microscopic picture are predominating. Their appearance is usually relied with increased number of patients with AIDS. Decreased number of CD4+ lymphocytes is considered to be crucial. The role of other factors with probable influence upon structural changes, such as virulence of the pathogen is underestimated.

**Method:** We studied 163 autopsy cases of deceased from tuberculosis in Irkutsk during 2008–2012. Genotype of mycobacteria was determined by study of tissues by MIRU-VNTR genotyping on 12 locuses. Identification of received profiles was provided according open data base MIRU-VNTR plus.

**Results:** 7 different genotypes of mycobacteria were determined: Beijing-108, LAM-17, T-14, Orphan-11, Ural-6, Harlem-5, S-2. According to character of pathological changes we distinguished two groups: 1 group–53 cases with typical for tuberculosis exudation and granuloma formation. Among them 12 HIV infected. Genotypes: LAM -14, T-12, Orphan-8. Ural -5, Harlem-4, S-2, Beijing -8 2 group–110 cases with alterative changes without typical cellular reactions. Among them 53 HIV-infected. Genotypes: Beijing -100, LAM-3, Orphan- 3, T-2, Ural-1, Harlem-1.

**Conclusion:** Thus, we can suppose that the genotype of Mycobacteria tuberculosis may influence upon the histological pattern in tuberculosis.

**PS-13-006**

**An exceptional case of fulminant hepatic failure caused by tuberculosis**

R. A. Cioca ^*^, M. Mihaela, F. Ana Maria, C. Ovidiu, C. Anca, R. Marius

^*^Emergency Clinical Hospital, Pathology, Cluj-Napoca, Romania

**Objective:** Miliary tuberculosis is a virulent form of tuberculosis that may affect any organ of the body. It is well established that it can affect the liver, but tuberculosis presenting as fulminant hepatic failure is extremely rare. Only 3 cases have been reported in the literature since 1994.

**Method:** A 54 year-old female underwent a left nephrectomy for a renal tumour which later, on histologic examination, proved to be tuberculosis. After surgery she developed a hepatocytolisis syndrome. As the clinical and biochemical hepatic picture continued to deteriorate antituberculose treatment could not be administered. Because of an unknow aetiology of hepatic failure, serologically tests were performed in order to exclude viral causes. The patient condition continued to deteriorate and she died after 16 days.

**Results:** Postmortem examination showed multiple caseating granulomas in the liver, lungs, spleen and myocardium. More than 50 % of the liver parenchyma was destroyed by granulomatos infiltration. Brain edema and perivascular lymphocytic inflammation were other significant findings. Ziehl Neelsen staining demonstrated the presence of acid fast bacilli in the brain tissue.

**Conclusion:** We presented an autopsy case of military tuberculosis with rapid evolution and severe outcome.

**Poorley formed granulomas in liver:** 
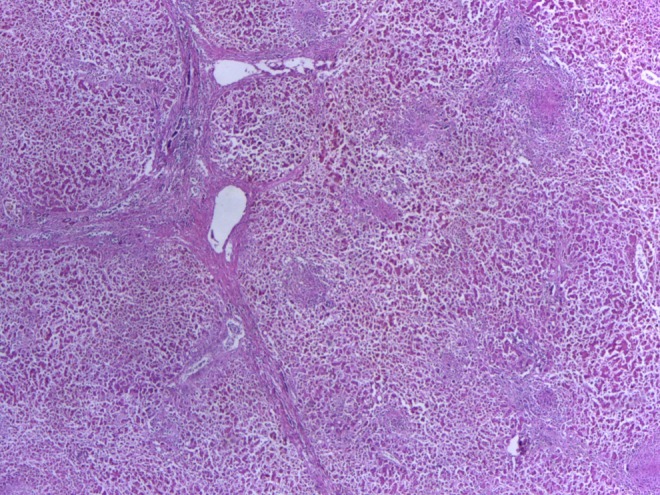


**PS-13-007**

**Whipple’s disease in cervical lymph node: An unusual presentation**

M. Dwyer ^*^, N. Rooney

^*^NBT, Dept. of Histopathology, Bristol, United Kingdom

**Objective:** To find out the cause of lymphadenopathy in a lady who presented with cervical and abdominal lymphadenopathy and weight loss. The CT scan showed abdominal lymph adenopathy with foamy appearance

**Method:** The case was worked up as a lymph node biopsy was sent to the Lymphoreticular team for assessment of lymphoma. The histology revealed granulomatous lymphadenitis with foamy macrophages and giant cells. Alcian blue Pas showed that the macrophages were packed with PAS positive bacilli. Result: A diagnosis of Whipple’s disease was made and confirmed by PCR and subsequent duodenal biopsy. Conclusion: Lymphadenopathy can be an unusual presenting feature of Whipple’s disease. Ultrasound guided lymph node core may be done in view of the fact and therefore pathologists should be aware of this unusual presentation. Whipples disease is curable with long term antibiotic therapy.

**Results:** Ultrasound guided lymph node core may be done in view of the fact and therefore pathologists should be aware of this unusual presentation. Whipples disease is curable with long term antibiotic therapy.

**Conclusion:** Lymphadenopathy can be an unusual presenting feature of Whipple’s disease

**PS-13-008**

**Dirofilariasis in pathology specimens**

Y. Rogov ^*^, E. Parhovchenko, E. Grigorieva

^*^Belarusian Medical Academy of Pathology, Minsk, Belarus

**Objective:** Dirofilariasis affects dogs, cats, wild predators and rarely people. The disease is mostly common in warm, humid climates. In Belarus in recent years a few cases of the disease in humans were revealed and published by ophthalmologists. The aim of the study is to analyze the morbidity rate of dirofilariasis in Belarus in biopsy and surgery samples for the last 10 years.

**Method:** The results of biopsy and surgery investigations from Minsk and regional pathology departments were studied for the period 2002–2012 years taking into account the age and the gender of the patients.

**Results:** For this period 9 cases of dirofilariasis were found in the Republic of Belarus. The age of the patients was between 26 and 58 years. Women (6) were affected more often. Some of the patients were the owners of dogs and cats. Tumor-like lesions in different parts of the limbs and body dominated in the clinical picture of disease. They were not connected with orbits. In some cases a cancer was suspected clinically. Among the affected areas there was one breast. The diagnosis was based on the typical parasite structures and inflammation in tumor-like tissues.

**Conclusion:** According to the published clinical data and original research there is some increase in the spread of dirofilariasis in Belarus over the last decade.

**PS-13-009**

**Gangrene of the large intestine caused by enterobius vermicularis**

S. Bozanic ^*^, D. Lalosevic, N. Solajic, V. Lalosevic

^*^Institut za Onkologiju, Odeljenje za Patologiju, Sremska Kamenica, Serbia

**Objective:** Enterobiasis is a common parasitic infestation worldwide. Enterobius vermicularis is most commonly encountered in the lumen of large intestine. Only very rare cases of enterobiasis are invasive.

**Method:** We present a case of 59 years old male who was submitted to the hospital as an emergency case due to acute abdomen. Explorative laparotomy revealed partly gangrenous large intestine with obvious perforation and acute peritonitis and therefore total colectomy was performed.

**Results:** On pathologic examination, there was a multifocal thick purulent exudate on the serosal surface with one grossly visible perforation in the right colon. The intestine was dilated and filled with fecal masses containing numerous white, 4–13 mm long round worms some of which were adhered to the mucosal surface. The mucosa was thin, multifocally eroded and ulcerated. Microscopically, adult worms and ova were identified as Enterobius vermicularis and were found in both intestine lumen and wall. Moreover, the worms were found in some of the thrombosed mesocolonic blood vessels.

**Conclusion:** Invasive enterobiasis in extremely rare compared to its usual non-invasive form which is relatively commonly encountered in general population. Our case shows that it can cause a life-threatening condition.

**PS-13-010**

**Echinococcus alveolaris: 73 new cases**

A. Kurt ^*^

^*^Bölge Egitim ve Arastirma Hastesi, Dept. of Pathology, Erzurum, Turkey

**Objective:** Erzurum, Turkey was the most E. Alveolaris reported center.

**Method:** We present 73 E.alveolaris materials that we have seen in the last 27 years.

**Results:** In 51 patients (mean age 37.6) it was localized in the liver:29 women (mean age 36.2) and 22 men (mean age 39.2), 5 women (mean age 34.3) and 8 men (mean age 33.5), a total of 13 patients (average age 34) were localized in the brain. In 9 patients with other organ involvement (lung, parietal peritoneum, kidney, pleura, abdominal cavity, gall bladder). 38 of the patients were female, and mean age was 35.6, and 35 were male and the mean age was 39.4. The incidence heaped up at 4th, 5th, and 3rd decade respectively. The youngest patient was 14 years old, male (liver), the oldest patient was 68 years old, male (liver).

**Conclusion:** In recent years, two important differences were seen; While the vast majority was in the liver in 1985–1995, over recent years the localization of the brain was increased. Almost all of the previous cases were at only an organ, 5 patients who came in the last 4 years, E. alveolaris materials which were extracted at least 2 separate sessions were seen.

**PS-13-011**

**Anisakiasis (Anisakidosis): A health problem in the context of increasing popularity of Japanese cuisine and traditional consumption of pickled anchovies in Southern Europe**

L. Baron ^*^, M. Postiglione, P. Alfano, F. Borriello, C. Trombetta, F. Quarto

^*^Ospedale S. Leonardo ASLNA 3, Dipt. Anatomia Istologia Patologica, Castellammare di Stabia, Italy

**Objective:** Human may be accidental hosts for nematode parasites in which they do not progress in their life cycle, but can cause gastrointestinal diseases. Anisakiasis is nematode infection due to consumption of raw or undercooked fish. The highest incidence is found in Eastern Asia, where raw fish (sushi, sashimi) is usually eaten. In the Mediterranean, globalization associated with traditional dishes at risk of contamination (pickled anchovies) can justify the increase, often unrecognized, of new cases of Anisakiasis in seaside towns.

**Method:** In our institution, during the past 2 years, 5 cases had a diagnosis of eosinophilic enteritis compatible with Anisakiasis. Patients presented abdominal pain and signs of intestinal obstruction when submitted to laparotomy, followed by resection of ileal segments.

**Results:** Histological examination reveals transmural inflammation, marked infiltration of eosinophils, histiocytes, lymphocytes and plasmacells until periintestinal fat, mucosal erosions, oedema, submucosal abscess and granulomatous reaction surrounding the parasite cyst, when found. In one case the larva came out from eroded mucosa. Another case presented blastic lymphocyte proliferation, T type, suspicious for T cell lymphoma.

**Conclusion:** Globalization of traditional food habits can explain why Anisakis musk be taken into account in differential diagnosis in cases of acute abdomen in our geographical location.

**Anisakis, encysted in small bowel wall:** 
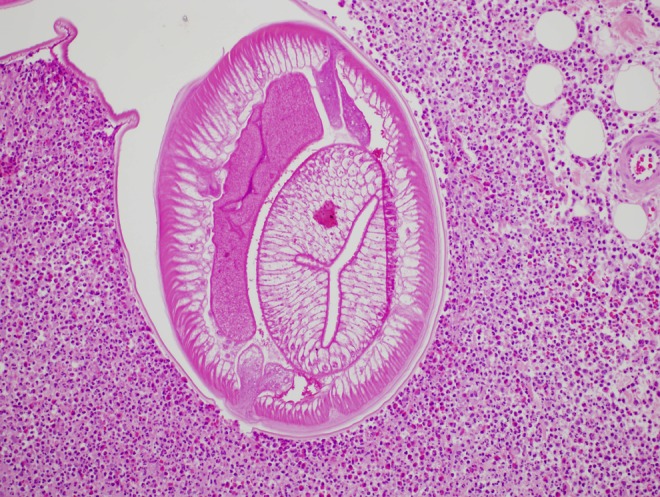


**PS-13-012**

**The immunopathological aspects in experimental trypanosoma cruzi reinfections depends on infecting strains**

J. Machado^*^, M. Reis, M. Silva, D. Borges, C. Silva, L. Ramirez, V. Rodrigues, D. Rodrigues

^*^University Triângulo Mineiro, Laboratory of Immunology, Uberaba, Brazil

**Objective:** Chagas disease is caused by Trypanosoma cruzi infection. Besides the host-related factors, such as immune response and genetics, factors related to the parasite, such as starter strain and occurrences of reinfection, may have an influence in the development of clinical forms and outcome of the disease.

**Method:** Experimental study

**Results:** Our results show that both the primary infection and the reinfection with the Colombian strain are connected with lower survival rate of the animals. After reinfection the levels of parasitaemia were approximately ten times lower than in primary infected animals. Only Colombian, Col/Col and Y/Col Groups showed amastigote nests in cardiac tissue. Moreover, the mice infected and/or reinfected with the Colombian strain had more T. cruzi nests, intense inflammatory infiltrate, and significantly higher in situ expression of TNF-α and IFN-γ. After spleen cell culture, the groups infected and/or reinfected had higher production of TNF-α, IFN-γ and IL-10, especially in antigen-stimulated culture.

**Conclusion:** Our results suggest that a higher mortality rate, tissue parasitism, inflammatory infiltrate and the expression of pro-inflammatory cytokines, in situ and in vitro, are influenced by the presence of the parasite, and are more visible in the infection and reinfection with the Colombian strain. Financial support: CNPq, Capes, Fapemig, Funepu, UFTM

Monday, 2 September 2013, 09.30–10.30, Pavilion 2

**PS-14 Poster Session IT in Pathology**

**PS-14-001**

**Diagnostic challenges and advantages of international telepathology between two medical institutions**

D. Borys ^*^, C. DiLoreto, M. Gambarotti

^*^University of California, Dept. of Pathology, Davis, USA

**Objective:** Digital pathology is used primarily as an educational or research tool, however application in daily pathology practice has already been implemented in areas with limited access to pathologists. The aim of this study is to demonstrate the diagnostic telepathology used in orthopedic pathology consultation between two medical centers from different countries and time zones.

**Method:** Over a 25 day period, slides for bone and soft tissue subspecialty cases were captured with the Aperio ScanScope CS whole slide scanner at UCDavis. The slides for each case were viewed virtually by a orthopedic pathologist at Rizzoli Orthopedic Institute, Bologna, Italy using the Aperio Spectrum WebScope. The pathologist had secure access to the UCD Laboratory Information System (LIS), electronic medical records, and radiology images. Case discussions were accomplished by secure hospital email. The glass slides were later reviewed by light microscopy.

**Results:** Fifty-two cases were scanned and evaluated virtually. The mean time for scanning cases and reporting results was 2.29 days. The majority of cases (69.2 %) were reported within 1 day. One histologic discrepancy (1.9 %) was identified upon light microscopic review.

**Conclusion:** Our international telepathology experience has shown that digital pathology is adequate for primary diagnosis and consultation and can be included in daily pathology practice without delaying diagnosis. However, image quality should be closely monitored to ensure

**PS-14-002**

**E-modules on histopathology: A valuable online tool for students, researchers and professionals: The HIPON project**

L. Brcic ^*^, A. C. Lazaris, S. Seiwerth, H. van Krieken, G. Agrogiannis, A. Smeets, S. Themistokleous, G. Armenski, M. Gushev

^*^University of Zagreb School of Medicine, Institute of Pathology, Croatia

**Objective:** One of the major challenges in medicine today is acquiring the basic knowledge deriving from the huge amount of available information and transforming it to medical experience. A new project under the title above, co-financed by the Lifelong Learning Program of the Education, Audiovisual and Culture Executive Agency of the Commission of the European Union, has been launched at the beginning of this year.

**Method:** HIPON intention is to transmit professional experience through a learning website platform using modern technology that will stimulate users. Basic concept is to follow mixed learning pathways (microscopic images, educative videos, virtual slides) supported by a rich variety of components of the ICT System (virtual portfolio, e-modules, online game, histo-book) and a mass amount of case data in order to provide practical experience, bridging essentially the worlds of education and professional practice. HIPON users will be able to send questions to the instructor of the subject, and collaborate on-line with peers. It will be a multilingual platform for multinational training and vocational activities, providing flexibility to both users and instructors.

**Results:** By accomplishing all this, HIPON will improve the efficiency of training and enhance creativity at all levels of education, fulfilling the aims set by “The EU strategic framework for education”.

**PS-14-003**

**A simulation of digital pathology in a clinical workflow: Facts and figures**

P. Branders ^*^

^*^University Hospital Leuven, Dept. of Pathology, Belgium

**Objective:** Five different scanners were evaluated in order to see the impact of digital pathology on the timeline, data storage and standard operating procedures of a clinical workflow.

**Method:** 114 randomly selected biopsy slides containing hematoxylin-eosine, immunohistochemistry and special stainings were scanned at the standard 40× magnification profiles. The mean scanning time and amount of data needed per slide were determined. Based on those results, five separate simulations of a real clinical workflow was made containing 384 slides ready to be scanned between 8:00 AM and 4.30 PM, in mean batches of 17 slides and always using two identical scanning systems.

**Results:** Mean scan speed was 5,3 min (SD 2,6 min) per slide. The mean amount of data created by the systems was 1,6 GB (SD 0,7 GB). Implementing digital pathology in the tested clinical workflow, would lead to an average delay of 8:09 h (SD 05:25 h).

**Conclusion:** It can be concluded that not all digital pathology systems are ready to be used in a clinical workflow due to the lost of time. Large differences in terms of scanning speed and data collection were measured between the systems. Testing several systems to find the correct set up for the specific application is necessary.

**PS-14-004**

**Use of Hypothesized Interaction Distribution (HID) analysis in follicular lymphoma automatically identifies prognostically important patterns of cellular spatial distribution**

L. Nelson ^*^, J. Mansfield, C. v. der Loos, R. Lloyd, K. Linton, J. Radford, R. Byers, C. Rose

^*^Manchester, United Kingdom

**Objective:** Histopathological prognostication relies on morphological pattern recognition, but as biomarker numbers increase the ability for human prognostic pattern recognition decreases. We have developed an automated method, hypothesized interaction distribution (HID) analysis, for characterizing the statistical distribution of spatial patterns of multiple cellular markers. Follicular lymphoma (FL) has a variable outcome, partly determined by FOXP3 and CD69 positive T-cells. We used HID analysis to determine whether their spatial pattern was prognostically significant.

**Method:** A tissue microarray comprising 40 FL samples was used in triplex immunohistochemistry for FOXP3, CD3 and CD69. Multispectral imaging was used to determine the spatial locations of FOXP3 and CD69 positive T-cells. HID analysis was then used to assess prognostic significance of cellular pattern, quantified by Shannon entropy.

**Results:** HID analysis identified two groups with higher (2.42) and lower (0.36) median entropy. Higher entropy corresponded to longer median survival (142 months) compared to lower entropy (35.5 months) (*p* = 0.0025). Since higher entropy reflects a more diffuse cellular pattern a diffuse pattern of FOXP3 and CD69 positivity was associated with better outcome.

**Conclusion:** These results demonstrate the ability of HID analysis to automatically identify prognostic patterns of cellular distribution, demonstrating favourable outcome of a diffuse pattern of FOXP3 and CD69 cells in FL.

**PS-14-005**

**Estimation of Ki-67 labelling index using systematic random sampling of microscopic fields: An unbiased way to appraise proliferation on breast cancer biopsies**

N. Elie ^*^, M. Oger, M. Allaoui, P.-E. Brachet, J. Marnay, C. Augé, C. Lebeau, B. Plancoulaine, P. Herlin, C. Bor-Angelier

^*^Normandie University, UNICAEN, CMABIO-HIQ Facility, Caen Cedex 5, France

**Objective:** Estimation of the proliferating fraction of epithelial cells on breast cancer biopsies is recommended for improving patient care. The most popular method is the enumeration of immunolabeled and unlabeled cell nuclear profiles on subjectively chosen microscopic fields on histological sections. Nevertheless the standardisation of the counting method used and the representativity of the results obtained should be proved before being recommended and used in routine practice. The aim of the present work is to evaluate if optimal sampling methods and counting rules of Stereology could help in this context.

**Method:** Estimation of Ki67 was performed by image analysis on whole sections of 92 breast cancer biopsy virtual slides and compared to the results obtained by the same approach on systematically sampled fields using the “forbidden line” rule of Stereology. Finally, results were confronted to distribution marker heterogeneity, visually appraised by the senior pathologist.

**Results:** The Ki67 labelling index obtained from sampled fields and from whole sections are correlated (*r* = 0.96, *p* < 0.0001). There is no significant difference between heterogeneous and homogeneous tumours.

**Conclusion:** Systematic random sampling of microscopic fields could be a simple, fast and reliable approach to provide a representative assessment of Ki67 labelling index on histological sections.

**PS-14-006**

**A complete framework for automatic grading of H&E stained images of follicular lymphoma**

T. Koletsa ^*^, E. Michail, E. Kornaropoulos, K. Dimitropoulos, I. Kostopoulos, N. Grammalidis

^*^Medical School AUTH, Dept. of Pathology, Thessaloniki, Greece

**Objective:** Follicular lymphoma grading based on the number of large cells in high-power field is subject to intra-observer variability. The development of a computer assisted method may improve reproducibility.

**Method:** A complete methodology has been developed for automatic grading of H&E stained images acquired from tissue biopsies of follicular lymphoma. The proposed methodology consists of three main steps; image segmentation, cell splitting and cell classification. The image segmentation algorithm used to detect the cells in the image is based on intensity thresholding. A novel cell-splitting method is introduced in this presentation to segment the so called “touching cells”. The proposed method is based on Minimum Description Length (MDL) and Expectation Maximization (EM) that help to segment cluster of cells into sub-cells. After the elimination of touching cells, candidate centroblasts (CBs) are selected for classification, according to their size. Candidate cells are classified into CBs and non-CBs by using a supervised classification algorithm.

**Results:** The proposed cell-splitting technique was seen to improve automatic grading results, by properly resolving many situations with merged cells, which were originally misidentified as CB cells.

**Conclusion:** Experimental results with H&E stained images have already shown the great potential of the proposed method.

**PS-14-007**

**Digital HER2 IHC-evaluation is equal to the semi-quantitative method**

T. Micsik ^*^, E. Turányi, Z. Sápi, L. Krecsák, G. Kiszler, T. Krenács, B. Molnar

^*^Semmelweis University Budapest, Ist Dept. of Pathology, Hungary

**Objective:** Quantification of HER2 expression of breast cancers became an inevitable part of the histopathological report which determines patient selection for targeted anti-HER2 therapy. Perfect targeting of trastuzumab therapy needs precise and standardized evaluation and documentation, for which purpose the digital pathology offers high definition tools. Our objective was to validate digital evaluation in a retrospective study on numerous cases.

**Method:** HER2 immuno-slides of 186 breast cancer cases were evaluated manually on glass slides and semi-automated on digitized slides by 3 pathologists and then compared by calculating Cohen’s kappa (CK) and Quadratic weighted kappa (QWK) in each interobserver (between pathologists) and intermethod (manual versus semi-automated) setting.

**Results:** HER2 interobserver CK with manual reading ranged 0,712–0,779 and QWK ranged 0,925–0,942, HER2 interobserver CK with semi automated reading ranged 0,698–0,722 and QWK ranged 0,912–0,916. HER2 intermethod CK comparing manual and semiautomated reading ranged 0,579–0,820 and QWK ranged 0,876–0,951.

**Conclusion:** Our data validated that evaluation of membranous HER2 immunostaining on glass and digitized slides results the same data without any adverse effect of digitization, but offering more convinient and flexible method. This data will gain increasing importance with the continuous improvement of digitization platforms using fully-automated evaluation algorithms.

**PS-14-008**

**Validation of digital immunohistochemical evaluation of hormone-receptors in breast cancer**

T. Micsik ^*^, E. Turányi, Z. Sápi, L. Krecsák, G. Kiszler, T. Krenács, B. Molnar, A. Matolcsy

^*^Semmelweis University Budapest, Ist Dept. of Pathology, Hungary

**Objective:** Estrogen (ER) and progesteron (PR) receptor expression of breast cancers is crucial for hormontherapy. The wide Allred-index assumes lower reproducability of scores in histopathological reports influencing patient selection. Thus standardized evaluation and documentation offered by digital pathology can be very beneficial. Our retrospective study aimed to validate digital nuclear-immunohistochemistry.

**Method:** ER and PR immunostained slides of 186 breast cancer cases were evaluated on glass and on digitized slides by 3 pathologists. Cohen’s kappa (CK) and Quadratic weighted kappa (QWK) were calculated in each interobserver (between pathologists) and intermethod (glass versus digitized) setting.

**Results:** Manual interobserver CK ranged 0,456–0,645 and QWK ranged 0,917–0,956. Digital interobserver CK ranged 0,532–0,640 and QWK ranged 0,930–0,962. Intermethod CK ranged 0,329–0,781 and QWK ranged 0,894–0,982.

**Conclusion:** Digitized evaluation of nuclear immunostaining was as precise as the manual evaluation of routine glass slides. Furthermore, digital reading has improved CK and QWK-values, especially with PR-immunoslides. Given the wide range of Allred-index, standardization by digital evaluation might improve patient selection for hormonal treatment.

**PS-14-010**

**Portable virtual microscopy: Is it possible?**

L. Alfaro ^*^, M. Roca, A. Paradis, F. Sevilla, M. Hernandez

^*^Valencia, Spain

**Objective:** Virtual microscopy with whole slide imaging is a complex technology in which the use of expensive devices for scanning microscopic glass slides combines with sophisticated software to serve images and allow remote access. We tried to develop alternatives to handle this digital files in a way they could be easily moved, transported into different computers, accessed from anywhere, and reviewed without installing specific software.

**Method:** Three scanners were employed to obtain whole slide images: a Leica CN400 (.scn files), a 3D-Histech Mirax Midi (.mrxs files), and a Dako ACIS III (exported .tif files). Aperio free viewer was used to read and export mrxs files and tif files into plane jpg files. A portable Java viewer for tif slides and a portable Flash/HTML5 viewer for jpg files were use to handle virtual slides from DVD and USB memory devices

**Results:** Virtual slides generated to be hold with Java and Flash/HTML portable viewers could be accesses from any computer form the Internet without installing additional software, hosted in any conventional server and reviewed directly from DVDs and USB memories.

**Conclusion:** Portable virtual microscopy is feasible with free viewers, requiring only some knowledge to export and transform the original file formats from different scanners.

**PS-14-011**

**A taste of Romania**

M. Comanescu ^*^, G. Butur, C. Ardeleanu

^*^INCD Victor Babes, Pathology, Bucuresti, Romania

**Objective:** The need for standardized quality in the histological and cytological preparations gave rise to an internation project focused on the use of Telepathology for the Assessment of Histopathological Techniques at European level (TASTE), by building-up an ICT environment TASTE System. The present study investigates the impact the development of the TASTE system had on different professionals all over Romania.

**Method:** The TASTE platform was presented to different workshops and participants were asked to give their written feedback.

**Results:** The assessment with real users eliminated some problems as well as participated to the improvement of the system.

**Conclusion:** This system created a local web-based community aimed to improving quality of histopathological and cytopathological preparations. Acknowledgement Project TASTE–Multilateral projects, project number: 519108–LLP-2011-IT-KA3-KA3MP, Grant Agreement number: 2011-4018/001-001 KA3–With the support of the Lifelong Learning Programme of the European Union. This project has been funded with support from the European Commission.

**PS-14-012**

**3** **dimensional digital reconstruction of the murine coronary system for the evaluation of chronic allograft vasculopathy**

L. Fonyad ^*^, K. Shinoda, EA Farkash, M. Groher, DP Sebastian, RB Colvin, Y. Yagi

^*^1st. Dept. of Pathology and Experimental Cancer Research, Semmelweis University, Budapest, Hungary

**Objective:** Chronic allograft vasculopathy (CAV) is a major mechanism of graft failure of transplanted organs in humans. Morphometric analysis of coronary arteries enables the quantitation of CAV in mouse models of heart transplantation. However, conventional histological procedures using single 2­dimensional sections limit the accuracy of CAV quantification. The aim of this study is to improve the accuracy of CAV quantification by reconstructing the murine coronary system in 3­dimensions and using virtual reconstruction and volumetric analysis to precisely assess neointimal thickness.

**Method:** Mice tissue samples, native heart and transplanted hearts with chronic allograft vasculopathy, were collected and analyzed. Paraffin embedded samples were serially sectioned, stained and digitized using whole slide digital imaging techniques under normal and infrared lighting. Sophisticated software tools were used to generate and manipulate 3­dimensional reconstructions of the major coronary arteries and branches.

**Results:** The 3­dimensional reconstruction provides not only accurate measurements but also exact volumetric data of vascular lesions. This virtual coronary arteriography demonstrates that the vasculopathy lesions in this model are localized to the proximal coronary segments. In addition, virtual rotation and volumetric analysis enabled more precise measurements of CAV than single, randomly oriented histologic sections, and offer an improved readout for this important experimental model.

Monday, 2 September 2013, 09.30–10.30, Pavilion 2

**PS-15 Poster Session Neuropathology**

**PS-15-001**

**Amyloidosis restricted to meninges: A rare phenotype of familial amyloidosis associated to Ala25Thr**

T. Ribalta ^*^, I. Aldecoa, L. Herrero, M. Solé, F. Graus

^*^Hospital Clinic of Barcelona, Pathology, Spain

**Objective:** To report the features of a patient with the Ala25Thr variant of familial amyloidosis discovered through a spinal cord meningeal biopsy. Leptomeningeal amyloidosis (LA) is a rare phenotype of hereditary transthyretin (TTR) amyloidosis, characterized by preferential amyloid deposition in the leptomeninges. Several mutations in TTR genes are known to cause LA with predominant central nervous system manifestations, but only the Ala25Thr variant is associated with pure leptomeningeal involvement.

**Method:** Review of the patient chart, routine pathological studies, immunohistochemical labelling with anti-TTR antibody, and genetic testing.

**Results:** The patient was a 53-y.o.-woman without familial history of amyloidosis, with a 4-y history of progressive paraparesis and ataxia. Her mother died at the age of 60 y with similar symptoms. Head and spinal MRI revealed contrast enhancing thickened meninges and siderosis. On histological examination, the leptomeninges were thickened by heavy deposits of amyloid with scattered hemosiderin-laden macrophages. Immunohistochemistry identified the amyloid as TTR. Genetic studies revealed a Ala25Thr mutation. In the following search for systemic amyloidosis, no amyloid deposits were observed in the biopsed specimens.

**Conclusion:** The Ala25Thr variant of TTR presents as highly selective LA. In the absence of a familial history, a high index of suspicion is required to consider LA in the diferential diagnosis of rare entities with meningeal thickening and siderosis.

**PS-15-005**

**P53 and Ki-67 expression can predict drug-refractory epilepsy**

M. Aizpurua ^*^, M. Toledo, F. Martinez Ricarte, A. Ortega Aznar, A. Navarro, R. L. Palhua, S. Ramon y Cajal, E. Martinez Saez

^*^Vall d’Hebron Hospital, Dept. of Pathology, Barcelona, Spain

**Objective:** Diffuse astrocytomas, specifically glioblastoma (GBM), are the most frequent malignant brain tumours. GBM is a WHO grade IV neoplasm, characterized by necrosis and vascular proliferation. Two kind of GBM are recognized: primary (pGBM) GBM (LOH10q, EGFR amplification, p16 deletion as major molecular alterations) and secondary GBM (sGBM) arising from a lower grade astrocytoma (LOH10q, TP53 mutation). Clinically they present with a short history of raised intracranial pressure, but also with personality change, stroke-like symptoms or epileptic seizures. Our aim is to correlate proliferation index (Ki67) and p53 expression with epilepsy refractoriness.

**Method:** From a cohort of 28 GBM we sought to determine predictors of drug-resistant epilepsy, including Ki67 and p53 expression.

**Results:** Mc-Nemar test showed the higher the p53, the higher the likelihood of drug-resistant epilepsy; in counterpart to Ki67 values. The ROC curve established the best cut-off points as 45 % for p53 and 25 % for Ki67. All diagnosed sGBM (*n* = 5) were drug resistant.

**Conclusion:** Higher p53 expression along with lower Ki67 index correlates with medical refractory epilepsy. P53 overexpression is a well known phenomenon in sGBM, although no cut-off point has been established yet. Herein, we found similar behaviour between diagnosed sGBM and GBM with p53>45 %.

**PS-15-008**

**Pilocytic astrocytomas: Immunohistochemical and molecular study**

S. Hmissa ^*^, S. Ben Abdelkarim, S. Trabelsi, N. Missaoui, M. Achour, S. Korbi, M. Njima, M. T. Yaacoubi

^*^Farhet Hached Hospital, Dept. of Pathology, Sousse, Tunisia

**Objective:** Pilocytic astrocytomas are the most common paediatric brain tumours. In this study, we have assessed immunohistochemical and molecular parameters in order to determine a potential diagnosis of pilocytic astrocytoma.

**Method:** 12 cases of pilocytic astrocytoma were collected in the period between 2006 and 2012 occurring in patients aged between 2 and 40 year old. The microscopic examination of these sections is increasingly supplemented by the application of immunohistochemistry using GFAP, Vimentin, NF, NSE, Synaptophysin, bcl2, Ki67, P53, CD99, and CD117 antibodies, and molecular tests using MLPA technique (IDH mutations, BRAF amplifications, CDKN2A gene).

**Results:** The immunohistochemical showed a GFAP expression in all the cases, Vimentin was expressed in 10 cases, NSE was positive in 7 cases, bcl2 was positive in 3 cases, whereas ki67, P53, CD99 and CD117 were negative. The MLPA test showed IDH gene mutation in 3 cases, amplification of BRAF in 3 cases, amplification of CDKN2A gene in 1 case.

**Conclusion:** These results argued that immunohistochemistry is useful in the diagnosis of the glial origin of the tumour and the pilocytic type is supported by the BRAF anomalies detected by molecular tests, whereas, this is not compulsory to do a diagnosis of pilocytic astrocytoma.

**PS-15-009**

**Transformation of atypical pilocytic astrocytoma to anaplastic astrocytoma: A case report**

J.-H. Lee ^*^, K.-B. Kim, D.-H. Shin, K.-U. Choi

^*^PNUYH, Dept. of Pathology, Yangsan, Republic of Korea

**Objective:** A case of a 27-year-old female Vietnamese with headache is reported.

**Method:** A multi-cystic, 4.6 cm-sized, brain tumor was present in the right frontal lobe on the brain MR image. The tumor seemed to be an extra-axial tumor, such as atypical meningioma. It was found during the surgery that the tumor was located in the brain parenchyma rather than the dura mater. The tumor was near completely removed.

**Results:** Microscopically, the lesion had paucicellular, fascicular, and spongy tissue with Rosenthal fibers and eosinophilic granular bodies. There were also more pleomorphic areas and some with scattered mitotic figures, a maximum of 3/10 HPFs. Given the relatively low level mitotic activity, however, the tumor was diagnosed as pilocytic astrocytoma with adjective of “atypical”.

**Conclusion:** The follow-up MR imaging showed tumor recurrence in the right frontal area of the brain 9 months later. The histologic section revealed very mitotically active lesion with no pilocytic features. The lesion was to us anaplastic astrocytoma transformed from atypical pilocytic astrocytoma.

**PS-15-010**

**Papillary ependymoma: its differential diagnosis from choroid plexus papilloma**

A. Zehani-Kassar ^*^, I. Chelly, H. Nfoussi, H. Azouz, B. Chelly, E. Boudabous, S. Haouet, N. Kchir

^*^La Rabta hospital, Pathology, Tunis, Tunisia

**Objective:** Ependymomas are heterogeneous group of tumours including cellular, papillary, clear cell and tanacytic histology. The papillary ependymoma is an unusual variant of ependymoma characterized by distinct morphology resembling other papillary tumours and corresponding to WHO grade II malignancy. It often gives rise to confusion with choroid plexus papilloma because of topographic and light microscopic.

**Method:** We report a retrospective case series of 12 patients with papillary ependymoma, regarding their unique clinicopathologic features and differential points from choroid plexus papilloma.

**Results:** The patient population consisted of 6 females and 6 males. The mean age was 20 years with extreme ranging from 3 to 70 years. Magnetic resonance imaging of the brain revealed a mass in the spinal cord (7 cases), in the 4th ventricle (3 cases) or in infratentoriel (2 cases). A total excision of the tumor was performed in all cases. Pathological examination showed a tumor with a neuroglial stroma. It was was characterized by tubules and papillary structures with single or multiple layers of cuboidal, uniform tumour cells, without pleomorphism.

**Conclusion:** Histopathologically, the papillary ependymoma is distinguished from the choroid plexus papilloma by the frequent arrangement of cells in multiple layers, formation of tubules and the presence of the neuroglial stroma.

**PS-15-011**

**GFAP (−) anaplastic ganglioma of the temporal lobe**

E. Minaidou ^*^, G. Koutsonikas, C. Zorzos, E. Papaliodi, P. Korkolopoulou

^*^General Hospital of Athens, Surgical Pathology, Greece

**Objective:** Anaplastic ganglioglioma is a rare type of central nervous system (CNS) having an aggressive clinical course. (Grade III, WHO Classification)

**Method:** A 22 year old female was admitted for evaluation of long standing headaches. No previous personal medical history. Magnetic Resonance Image showed a circumscribed solid, partly cystic mass in the left temporal lobe, 8 × 4 × 3 cm in size which was excised surgically.

**Results:** Histologically the neoplasm was consisted of a dimorphic cell population: irregular groups of neurons often with dysplastic features {synaptophysin (+), NSE (+)} and glial neoplastic cells {GFAP (−), Olig2 (−) and IDH (−)}with increased cellularity and high proliferative activity. No obvious necrosis, vascular hyperplasia, eosinophilic bodies or Rosenthal fibers were seen. Many neoplastic glial cells were CD34 (+) demonstrating, interlacing cytoplasmic processes.

**Conclusion:** GFAP (−), CD 34 (+) anaplastic gangliogliomas rise a differential diagnostic dilemma from another primary myelohyperplastic,or even metastatic CNS neoplasm. Histological identification of the biphasic cell population may be helpful in the initial diagnostic approach. When there is no recognizable vascular hyperplasia or necrosis, diagnosis should be confirmed by more that one pathologists.

**PS-15-015**

**Immunohistochemical evaluation of meningiomas in respect to human herpes virus-6 infection markers**

S. Roga ^*^, I. Strumfa, A. Pavars, U. Berkis, S. Chapenko, S. Rasa, M. Murovska

^*^Riga Stradins University, Dept. of Pathology, Latvia

**Objective:** Our group has demonstrated the presence of human beta herpesvirus-6 (HHV-6) infection markers in human brain meninges. The present study investigates the presence of HHV-6 infection markers in adult meningioma tissues.

**Method:** The study group comprised 30 cases of meningiomas (aged 37–72 years) in operation materials. The meninges and brain tissue, obtained in adult (aged 41–71 years) autopsies, were included in the control group. The control group (*n* = 30) had no brain or meningeal pathology. Tissues were submitted for routine histology including haematoxylin-eosin staining. The presence of HHV-6 antigens in tissues was studied by immunohistochemistry (IHC); the presence of HHV-6 genome (DNA) was verified by PCR in the control group.

**Results:** By immunohistochemistry, unequivocal presence of HHV-6 antigens expression was identified in 23 cases of studied meningiomas. In the control group, presence of HHV-6 antigens expression was found by IHC in meningeal tissues (13 cases), including dura mater (3 cases); in brain tissues (9 cases), and verified by finding of HHV-6 DNA sequence.

**Conclusion:** Presence of HHV-6 is a frequent finding in meningiomas and human brain meninges. The data are consistent with the wide spread of the virus in population and chronic latent course.

**PS-15-016**

**Choroid plexus meningioma: Report of two cases**

M. Milanka ^*^, V. Blazicevic, G. Blagus, B. Splavski, D. Muzevic

^*^University Hospital Osijek, Inst. for Pathology, Croatia

**Objective:** Our intention is to show our experience with common tumor found in an unusual and rare location.

**Method:** Case 1. A 58 years old female patient was admitted to the hospital for acute psychosis. CT and MR showed an oval tumor process, about 3 cm in diameter, situated at the trigone of the right lateral ventricle, with dilatation of occipital horn of the ventricle. Case 2. 71 years old female patient, for the last 5 years in psychiatric treatment for psychosis, passed through neurologic and radiologic diagnostics, due to suspicion of brain trauma, which discovered an expansive process at trigone of left lateral ventricle, 4 cm in diameter with dilatation of occipital horn. During postoperative course the patient developed the deep vein thrombosis and died because of massive pulmonary thromboembolism.

**Results:** Fibroblastic type of meningioma was diagnosed in both cases. Immunohistochemical staining for EMA, Vimentin, S-100 and progesteron receptors showed positivity in both cases while immunohistochemical staining for GFAP, CKAE1/AE3, CKMNF 116 and CEA were negative. Estrogen receptors showed weak and focal nuclear positivity in the first case.

**Conclusion:** Choroid plexus meningioma in this location becomes diagnostically the most challenging because certain variants of meningioma may mimic other tumors found in this location and meningioma may be forgotten in the differential.

**PS-15-017**

**A recipient of tumor-to-tumor metastasis: Meningiomas**

M. Bagirzade ^*^, A. Ersen, M. G. Durak, N. Karabay, E. Demirtas, T. Canda

^*^Dokuz Eylul University, Medical Faculty, Dept. of Pathology, Izmir, Turkey

**Objective:** “Tumor-to-tumor” metastasis is a rare phenomenon and meningioma has been reported as the most common intracranial tumor for metastases. Such a case is presented in order to discuss this rare phenomenon.

**Results:** We describe a case of a 52-year-old female patient who underwent radical modified mastectomy for a breast lobular carcinoma in 2005. In 2010, diffusion MRI revealed a 3 × 2.5 × 2.5 cm dura based mass considered as dural metastasis. The patient recieved adjuvant chemotherapy. Three years after, she underwent a resection of the progressively enlarging intracranial masses. Microscopically, the larger mass revealed features of breast lobular carcinoma within the dura, with also invasion of adjacent brain parenchyme. The smaller mass was a typical meningioma of transitional type. Within the meningioma we found multiple foci of lobular carcinoma metastasis. By immunohistochemistry the metastatic foci was ER, PR, GCDFP-15 positive and cerb-B2 negative.

**Conclusion:** Our case had both a transdural and adjacent brain parenchmal metastasis in addition to a metastasis to an existing meningioma from the same primary tumor. This rises the question if all the metastatic tumors in meningiomas are actually only seeding to dura and co-incidentally ending up in a meningioma, which is a relatively common neoplasm.

**PS-15-018**

**Medulloblastoma: Immunohistochemical and molecular study**

S. Hmissa ^*^, S. Ben Abdelkarim, N. Missaoui, S. Trabelsi, A. Rebai, S. Korbi, L. Jaidaine, M. T. Yaacoubi

^*^Farhet Hached Hospital, Dept. of Pathology, Sousse, Tunisia

**Objective:** Medulloblastoma (MB) is a common tumour of the posterior fossa in children. Histopathological and genetic studies seem to be necessary to establish correct diagnosis. The aim of our study was to determine the criteria of positive diagnosis

**Method:** A retrospective study including 12 patients aged from 18 months to 36 years (sex-ratio: 5).

**Results:** Tumour were localized in the vermis (5 cases), in the fourth ventricule (4 cases) and in the cerebellar hemispheres (3 cases). There were 5 classic MB, 3 desmoplastic/nodullar MB, 2 nodular extensive MB and 2 large cell MB. The GFAP staining was positive in 9 cases and negative in 3 cases (1 classic MB, 1 desmoplastic/nodular MB, and 1 large cell MB). Immunohistochemical study showed synaptophysin expression in all cases, Vimentin expression in 6 cases, proliferation index Ki67 ranged from 40 to 90 %, weak P53 expression in all cases (positivity from 10 to 80 %) and cytoplasmic β-catenin staining in all cases. Molecular analysis showed chromosome 6p amplification in 4classic MB, chromosome 16p amplification in 8 tumours, GPR56 (16q) amplification in 8 tumours.

**Conclusion:** These results confirm the immunochemistry role in the establishment of the positive MB diagnosis. Molecular results are heterogeneous and cannot determine molecular subgroups of cases.

**PS-15-019**

**Neuroblastoma type supratentorial PNET**

V. Moldovan ^*^, M. Lisievici, C. Cocosila, S. I. Bedereag

^*^Bucuresti, Romania

**Objective:** We present the difficulties encountered in diagnosing a rare case of neuroblastoma type supratentorial PNET with focal glial differantiation in a 5 years old female.

**Method:** We had use squash technique (blue toluidine) for the intraoperatory diagnosis, the usual Hematoxylin-eosin; vanGieson staining and finally immunohistochemistry (monoclonal antibodies) to confirm the diagnostic.

**Results:** In the same year, the patience addressed three times intraoperatory specimens to our laboratory. The initial aspect was of an oligodendroglioma with numerous calcifications. In few months the tumor relapsed with an aspect of a neuroblatoma type PNET with small areas of glial differentiation. The second relapse exhibited a neuroblatoma type PNET. Immunohistochemistry showed positivity for NSE, synaptophysin, S-100 and focally for GFAP. Ki67 was estimated at 12 %.

**Conclusion:** The PNET imature pattern may exhibit neuroepithelial cells with divergent differentiation along neuronal, glial or mezenchymal lines. The particularitie of the case is the focal acquisition of glial differentiation making the initial presentation to resemble an oligodendroglioma. The instability of the supratentorial PNET phenotype could transform them into pitfalls for diagnostic.

**PS-15-020**

**Germinomas of the central nervous system–Case series**

M. Skender-Gazibara ^*^, S. Raicevic, E. Manojlovic Gacic, S. Pekic, D. Grujicic, V. Popovic

^*^Institute of Pathology, Belgrade, Serbia

**Objective:** Germinomas of the central nervous system are uncommon tumors with predilection for midline structures. We present a series of geminomas.

**Method:** From 2002 to 2012 18 cases of germinomas were diagnosed. Surgically removed tumor tissue was stained with hematoxylin-eosin. For immunohistochemistry following antibodies were used: PLAP, CD117, CK, GFAP and D2-40.

**Results:** Among 18 cases, 15 were male and 3 female (M/F ratio 5:1). The mean age of patients was 21,16 years (range, 10 to 47 years). Pineal region was the most common site (50 %), followed by sellar/suprasellar region (22,2 %), third ventricle (11 %), thalamus (5,6 %), subependymal seeding of lateral ventricles (5,6 %) and spinal cord (5,6 %). Grossly, tumor tissue was grayish and fragmented with soft consistency in 15 cases and tough consistency in other three. Microscopically, tumor was composed of large cells with vesicular nuclei, prominent nucleoli and pale cytoplasm with intervening stroma infiltrated by lymphocytes. In two cases of sellar and one of pineal germinoma, very dense inflammatory infiltration with or without noncaseating granulomas obscured tumor cells. Tumor cells were positive for PLAP, CD117 and D2-40.

**Conclusion:** According to our series, germinomas are predominantly present in pineal region of young males. Immunohistochemistry is essential for correct diagnosis.

**PS-15-021**

**Papillary tumor of the pineal region**

V. Moldovan ^*^, M. Lisievici, C. Cocosila, S. I. Bedereag

^*^Bucuresti, Romania

**Objective:** Case presentation of 25 years male with Papillary Tumor Of The Pineal Region (PTPR). PTPR remain a rare diagnostic, till date few cases have been published.

**Method:** We had use squash technique (blue toluidine) for the intraoperatory diagnosis, Hematoxylin-eosin and vanGieson stainings and immunohistochemistry (monoclonal antibodies) for the final diagnostic.

**Results:** The squash technique showed nonhomogeneous hypercellularity, no glial network, with a branched papillary appearance. They were covered by tumor cells arranged into single or bilayer. The cells were medium sized, ovoid with less obvious membrane. Vacuolated cells and signet ring cell were identified. With MRI and CT, assumption of a papillary tumor of the pineal region has been raised. Hematoxylin-eosin and vanGieson staining showed a pseudopapillary architecture covered by epithelioid cells, with abundant cytoplasm and a nucleus located centrally. Hyalinization of vascular walls, a low mitotic activity without necrosis, orientated for a grade II tumor. Immunohistochemistry showed positivity for AE1/AE3, CK18, vimentin, S-100 and focally for GFAP and synaptophysin. Estimated Ki67 was 2 %. Complete resection fallowed by radiotherapy has been tempted.

**Conclusion:** The patients have survived without recurrence till date confirming the grade II tumor. PTPR remains rare and little known. Intraoperatory diagnosis with squash may be feasible tool.

**PS-15-022**

**Primary central nervous system lymphoma: Deadly phenomenon exhibiting a distinct biologic phenotype–case report**

P. Donizy ^*^, A. Halon, M. Dziegala, A. Zimny

^*^Wroclaw Medical University, Dept. of Pathomorphology, Poland

**Objective:** Primary central nervous system lymphoma (PCNSL), is a rare and aggressive form of non-Hodgkin lymphoma. This is the disease where prognosis is markedly worse than in most other localized extranodal lymphomas. PCNSL often involves cerebrospinal meninges, eyeballs and spinal cord. Although PCNSL is sensitive both to radiotherapy and chemotherapy, the recurrences are very frequent.

**Method:** This is the case report of PCNSL in an immunocompetent 73-year-old woman.

**Results:** Imaging examination revealed diffused infiltration of brain, corresponding to TBC, sarcoidosis, fungal infection or lymphoma. Stereotactic biopsy was proceeded. Histopathological examination was inconclusive. Double analysis of cerebrospinal fluid did not demonstrate any pathological features. PCR test did not confirm Mycobacterium tuberculosis infection. The patient died after 3 months from admission to the hospital without established diagnosis. Autopsy revealed PCNSL, confirmed by immunohistochemistry (CD20+, CD5+, Bcl-2+, Bcl-6+, p53+, Ki67+, CD3−) and did not show extranodal lymphoma.

**Conclusion:** PCNSL is a relatively rare neoplasm, but its incidence has increased in recent years and should be taken into account in daily clinical practice. The proper diagnosis of PCNSL could be really difficult to establish. In this case even stereotactic biopsy did not reveal PCNSL. Sometimes only the autopsy studies may give us the good answer.

**PS-15-023**

**Primary histiocytic sarcoma of the central nervous system limited to the brain ventricles**

P. Cermakova ^*^

^*^Faculty Hospital Hradec Kralove, Dept. of Pathology, Czech Republic

**Objective:** Histiocytic sarcoma presenting as a primary lesion of the CNS is exceedingly rare with only 6 cases reported so far. A case of 31-year-old woman with a history of aseptic meningitis complicated with hydrocephalus, repeatedly treated by drainage 3 and 2 years before death is presented. The patient was finally hospitalized for a new attack of hydrocephalus, but her condition progressed rapidly and she died 14 days after beenig admitted. MRI revealed slight dilation of brain ventricles. Cytological evaluation of cerebrospinal fluid (CSF) proved large LCA positive cells suspicios of lymphoma.

**Method:** At the autopsy, all brain ventricles were dilated and difusely lined by 3–4 mm thick layer of pinkish gelatinous material, focally with bleeding. Histologically, this layer was composed of large, non-cohesive cells with abundant cytoplasm and large lobulated and pleomorphic nuclei with low mitotic activity.

**Results:** They were immunohistochemically positive for histiocytic markers (CD 68, CD 163, CD 11c) and expressed neither B and T cell markers nor myeloid markers. Dendritic cell, epithelial and melanocytic markers were also negative.

**Conclusion:** As herein demonstrated, despite its extreme rarity, histiocytic sarcoma of the central nervous system can be cytologically diagnosed from CSF. Unfortunately, despite correct diagnosis, the prognosis of this malignancy is very poor.

**PS-15-024**

**Malignant melanoma in a patient with leptomeningeal melanocytosis: A case report**

B. Pehlivanoglu ^*^, Y. Ertan, B. Yaman, T. Turhan, T. Akalin

^*^Ege University, Dept. of Pathology, Izmir, Turkey

**Objective:** Diffuse leptomeningeal melanocytosis is characterized by diffuse melanocytic infiltration of leptomeninges and derived from supra- and infratentorial leptomeningeal melanocytes. Malign transformation may occur and prognosis is poor in these cases.

**Results:** An 8-year old girl was referred with dizziness, vomiting and seizures. Physical examination revealed pigmented hairy nevi in various sizes. Cranial computered tomography showed multiple hyperdense lesions in brain and cerebellum, therefore brain biopsy was performed. Histopathological examination showed round tumor cells with prominent nucleoli invading brain parenchyma. Some of the tumor cells were pigmented. Immunohistochemically, tumor cells were stained positive for S100, melan A and HMB45. Ki67 proliferation index was 18 %. No other primary melanoma focus was detected and the case was considered as malignant melanoma transformed from leptomeningeal melanocytosis. The patient died 2 months after the diagnosis as being evaluated for neurocutaneous diseases.

**Conclusion:** Primary central nervous system melanoma is rare (incidence: 0,005/100.000) and it may develop due to diffuse leptomeningeal melanocytosis. Cases of leptomeningeal melanocytosis accompanying neurocutaneous syndromes have been reported. Prognosis is poor in cases of malignant transformation and most patients die before age 10. Here, we present an 8-year-old malignant melanoma case arising in leptomeningeal melanocytosis.

**PS-15-025**

**Case presentation: Intra- et parasellar chordoma**

L. Parascan ^*^

^*^Emmergency Institue, for C.V. Disease, Dept. of Pathology, Bucharest, Romania

**Objective:** Chordomas represent 1 % of all malignant bone tumors. The patient was a 64 years old women without pathological antecedents that was admitted for headache, progressive decrease of visual acuity, of right eye, double vision installed over the past 2 months.

**Method:** Cerebral MRI revealed a saddle area tumor measured 28/32/34 mm in its greater diameter, with hyper-signal in T1 and T2-weighted images, homogenous, with slight inside calcification, polycyclic outlined. Histology and immuno-histo-chemistry (IHC) tests are made: HE, VG, PAS, vimentin, EMA, CEA.

**Results:** Microscopic criteria: lobular pattern, more important in central part of the tumor, with different shapes, fibrous septa of variable thickness, more important at the tumor periphery, containing vessels. IHC analysis of the chordoma cells showed focal positivity for vimentin, EMA. Negative for CEA was noted. Ultrastructurally the tumor was composed of large cells containing irregular nuclei with multiple indentations and prominent nucleoli. Analysis of DNA revealed a diploid histogram.

**Conclusion:** Chordomas are middle slow-growing tumors that arises from proximal extreme remnants of the notochord, are locally invasive, may metastasize and are characterized by local recurrence. A gross total resection followed by radiotherapy has been the accepted treatment for schedule chordomas.

**PS-15-027**

**Spindle cell oncocytoma of the adenohypophysis**

B. Chelly^*^, A. Zehani, H. Azzouz, H. Nfoussi, I. Chelly, S. Haouet, N. Kchir

^*^Tunis, Tunisia

**Objective:** Spindle cell oncocytoma (SCO) of the adenohypophysis is a rare tumour recently reported by Roncaroli et al. in 2002. Our aim is to report a new case of SCO and to describe its histological and immunohistochemical features supporting the theory of a possible common origin with pituicytoma.

**Method:** We describe what is, to the best of our knowledge, the 17th case of its kind in the literature.

**Results:** We report the case of a 46-year-old woman with medical history of diabetes and asthma who presented with intermittent decrease of visual acuity and headache. Cranial magnetic resonance imaging (MRI) revealed a solid adenohypophysis mass of 36 × 33 × 29 mm with suprasellar extension and enlargement of the sella. This mass showed contrast enhancing in T1-weighted MRI scans. The diagnosis of nonfunctioning pituitary macroadenoma was suspected, and the tumour was completely resected via transsphenoidal surgery. Microscopic findings consisted of a solid spindle cell neoplasm with increased cellularity. Tumour cells were spindled to epithelioids organized in interlacing fascicles. The tumour cells had eosinophilic and oncocytic cytoplasm. Nuclear atypia and pleomorphism were absent.

**Conclusion:** SCOs of the pituitary gland are rare tumours whose pathogenesis and management remain debated because of the few numbers of reported cases. These tumours are considered to have a good prognosis despite the early recurrences reported in some cases.

**PS-15-029**

**Olfactory groove schwannoma: Case report**

M. Cruz ^*^, M. Ferreira, M. Teixeira, F. Pardal, A. Silva

^*^Hospital de Braga, Serviço de Anatomia Patológica, Portugal

**Objective:** Intracranial schwannomas preferentially arise from the vestibular branch of acoustic nerve, and rarely from the trigeminal nerve, facial nerve, and lower cranial nerves. Schwannomas arising from the olfactory groove are extremely uncommon and only 40 cases have been reported in the literature. We describe the case of an olfactory groove schwannoma (OGS) in a 24-year-old-woman.

**Method:** The patient presented with a 2-months history of headaches. MRI study showed a left subfrontal cystic extra-axial mass, involving the cribriform plate and contiguous to the left olfactory bulb. Total excision of the tumour was performed.

**Results:** The tumour was composed of spindle cells arranged in a palisading fashion with Antoni type A and Antoni type B cellular patterns. Tumor cells were diffusely positive for S-100 protein and CD57 (Leu-7) and negative for epithelial membrane antigen. The patient remained asymptomatic and without evidence of disease within a follow-up of 17 months.

**Conclusion:** The pathogenesis of anterior cranial fossa schwannoma has been a matter of controversy and there are several hypotheses concerning their putative origin. Recently, it was proposed the concept of olfactory ensheathing cell tumour which shares with OGS several radiological and histological similarities.

**PS-15-030**

**Evaluation of micro satellite instability in brain metastases of colorectal carcinoma**

D. Rotin ^*^, O. Paklina, V. Krivopuskov

^*^NN Burdenko Neurosurgery Institute, Dept. of Pathology, Moscow, Russia

**Objective:** Colorectal carcinoma (CRR) is considered to present different entities. One of CRR variants is an adenocarcinoma with micro satellite instability (MSI), which has specific approaches for treatment and prognostic significance. Aim: To evaluate and investigate MSI in Brain metastases (BM) of CRR.

**Method:** The material obtained from 45 patients with BM of CRR operated in NN Burdenko Institute in 2004–2010 (28- female, 17- male), median of age 55 (ranges 34–76), was stained H&E and IHC with 4 antibodies MSH, PMS After that results were re-checked with PCR.

**Results:** Among 45 patients only 6 had primary CRR located in right part of colon. Only in one case we IHC revealed typical MSI phenotype, which was confirmed with PCR. All other cases belonged to micro satellite stabile tumors.

**Conclusion:** MSI CRR tumors have extremely slight tendency to give arise to BM, comparing with MS stabile variants. MSI negative Tumor originated from left part of colon is risk factor for BM in CRR

**PS-15-031**

**Isolated intracranial Rosai-Dorfman disease: A pathological challenge**

M. A. Saco Alvarez ^*^, A. Sagasta Lacalle, B. Gonzalez, M. L. Cabañas, E. Campo, T. Ribalta

^*^Hospital Clinic Barcelona, Dept. of Anatomía Patológica, Spain

**Objective:** Rosai-Dorfman disease (RDD) is a idiopathic form of non-Langerhans-cell histiocytosis that occurs primarily in lymph nodes. Isolated intracranial involvement is extremely rare. We reviewed our files in search for cases and discuss their clinical and pathologic features.

**Method:** Three cases (women, 50–62 year-old) diagnosed at our institution between 2005 and 2012. Hematoxylin-eosin and immunohistochemical stains, using standard techniques and antibodies.

**Results:** We identified three patients with intracranial RDD without evidence of lymphadenopathies or extraneural abnormalities. Imaging studies favoured meningioma in two cases and metastatic disease in one. Microscopically, the masses consisted of dense infiltration of small lymphocytes and plasma cells, and groups of histiocytes scattered in fibrotic background. Emperipolesis suggested the diagnosis of RDD. Histiocytes were strongly immunoreactive for S-100 protein and CD68, and negative for CD1a. Treatment was surgical excision followed in one patient by combined conventional radiotherapy and chemotherapy. One patient died of a postoperative complication. The other two remain alive and free of disease.

**Conclusion:** Rosai-Dorfman disease should be added in the list of differential diagnosis for intracranial masses. A high index of diagnostic awareness is required to recognise isolated intracranial RDD.

**PS-15-032**

**Isolated intracranial Rosai-Dorfman disease: Case report and review of the literature**

A. Kurt^*^, S. A. Özmen, S. Zorludemir, F. Erdogan, H. Ö. Okay, O. Tanriverdi, I. Çalik

^*^Bölge Egitim ve Arastirma Hastesi, Dept. of Pathology, Erzurum, Turkey

**Objective:** Although extranodal involvement is known to occur, isolated intracranial Rosai-Dorfman Disease is very rare. To the best of our knowledge, there have been 80 cases with central nervous system (CNS) involvement reported in the literature, only 5 were in children.

**Method:** A 16-year-old female admitted to our neurosurgery clinic with a complaint of unbalance while working.

**Results:** In neurologic examination, cerebellar ataxi and disdiadocyhnesia were detected. MRI showed the solid mass assessed as meningioma and indicating intensive contrasting after IVKM injection followed izointense in T1 weighted sequences, hypointens in T2 weighted sequences. The lesion measured nearly 20 × 18 × 17 mm dimensions and located in neighboring sites of inferior sagital sinus and rooted from tentorium at slightly right of the middle line at the neighboring of right occipital lobe in tentorium. At the operation, cerebellar and supratentorial component of the mass was removed subtotally. As supratentorial component was adhesive, it couldn’t be excised exactly. Histological analysis revealed numerous histiocytes admixed lymphocytes and plasma cells. Well-preserved lymphocytes were observed within the cytoplasm of histiocytes, indicating emperipolesis. Immunohistochemistry for CD68, S-100 protein were positive for the histiocytic cells, CD1a and GFAP were negative.

**Conclusion:** Histological and immunohistochemical findins were consistent with Rosai-Dorfman Disease.

**PS-15-033**

**Sudden death from ruptured choroid plexus vascular malformation**

R. A. Cioca ^*^, M. Mihaela, G. Dan, C. Ovidiu, C. Anca, R. Marius

^*^Emergency Clinical Hospital, Pathology, Cluj-Napoca, Romania

**Objective:** Central nervous system vascular malformations are recognized as having potential to produce fatal hemorrhage. Arteriovenous malformations may be situated in any region of the brain but very rarely they can be restricted to choroid plexus.

**Method:** A 9-year-old boy previously healthy was admitted to the Emergency Room with no history of headache, migraine or epilepsy. At admission the patient was comatose with Glasgow coma scale of 3 points. Computer tomography of the brain revealed difuse subarahnoidian hemorrhage and extensive intraventricular hemorrhage without traumatic lesions. He died shortly after admission to hospital and an autopsy was performed next day.

**Results:** Gross examination of the brain showed edema with scattered subarahnoidian hemorrhage and with abundant blood filling the cerebral ventricles. Multiple histology sections were taken from the areas containing the most hemorrhage. Microscopic examination revealed a vascular malformation that involved the choroid plexus. Morphologically, there was an irregular proliferation of blood-filled arteries and arterialized veins with abundant sorrounding hemorrhage and focal ischemic changes.

**Conclusion:** We report an interesting case of sudden death caused by a ruptured vascular malformation with an unsual location.

**Choroid plexus vascular malformation (panoramic picture):** 
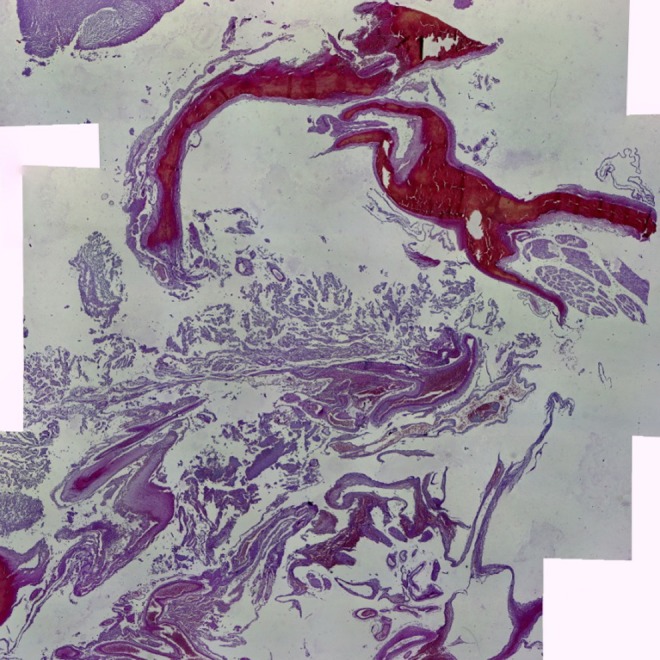


**PS-15-034**

**Role of alpha synuclein aggregation and mitochondrial dysfunction in neurodegeneration: A rat animal model**

G. Stoica ^*^, G. Lungu, N. Bjorklund, M. D’Amelio

^*^Texas A&M University, Dept. of Veterinary Pathobiology, College Station, USA

**Objective:** This study investigated the impact of alpha-synuclein (α-syn) aggregation and accumulation in the target areas of the brain, which impaired the mitochondrial function and contribute to neurodegeneration in an autosomal recessive rat animal model for Parkinson’s disease.

**Method:** In this study we employed a protein aggregate filtration (PAF) assay and a paraffin-embedded tissue (PET) blot for sensitive and selective detection of α-syn aggregates in tissue slides and brain homogenates. Confocal microscopy was used for co-localization of α-syn with mitochondria, and western blot assay for quantitative analyses.

**Results:** Affected rats demonstrated increased accumulation and aggregation of α-syn in brain mesencephalon (MS), putamen (P), and olfactory bulb (OB) at different postnatal time points (dpn) when compared with control littermates. Confocal microscopy showed co-localization of α-syn with mitochondria. Mitochondrial lysate WB analysis showed a significant increased in α-syn and also a significant decreased in complex 1 and membrane potential in affected rats at 25 dpn. In addition, an increased in LC3 conversion (as biomarker of autophagosome formation) was found in the brain target areas in affected rats.

**Conclusion:** Taken together our results provide evidence that increased α-syn accumulation and aggregation in this rat model impaired mitochondrial function induces oxidative stress and contributes to neurodegeneration.

**PS-15-035**

**4R Tauopathy with cognitive and motor impairment, not associated with Alzheimer Disease**

E. Evgeneva ^*^, J. Salas Felipe, M. Soriano Navarro, M. Vaquero Perez, J. Forteza Vila

^*^Hospital de Denia, Dept. de Anatomía Patológica, Spain

**Objective:** Tauopathy is a condition frequently associated with abnormal protein deposits in the cerebral cortex in the context of Alzheimer-type dementia. Our hypothesis is that the manifestations within the tauopathy concept can range from predominant bulbar and motor alterations to Parkinsonism or parkinsonism-plus clinical features.

**Method:** We present the case of a 76-aged female patient with 7-month-course progressive neurologic impairment, predominantly bulbar (gait impairment, postural tremor and bilateral hand bradykinesia, motor slowness, facial, chewing and swallowing muscle weakness, cervical extensor muscle weakness), and no response to levodopa (Sinomet). Final fulminant course with coma and death. The brain CT showed triventricular dilatation, without any other alteration. Final clinical differential diagnosis: Parkinsonism plus syndrome, brain neoplasia/lymphoma, paraneoplastic syndrome or viral infection.

**Results:** Partial postmortem examination (cranial cavity) is carried out and no remarkable macroscopic findings are observed. Immunohistochemistry showed 4R phosphorylated tau protein deposits at the substantia nigra and in scanty bridge neurons. Phosphorylated tau (AT8), 3T tau, ubiquitin, beta-amyloid were negative and absence of immunoreactive inclusions for crystalin and alpha-synuclein was found. At the structural level, neurofibrillary tangles (paired helical filaments) were found in the same area.

**Conclusion:** Final pathologic diagnosis: 4R tauopathy with substantia nigra involvement. Possible progressive supranucleary palsy (Steel-Richardson-Olszewski syndrome).

**Substantia nigra (immunohistochemistry):** 
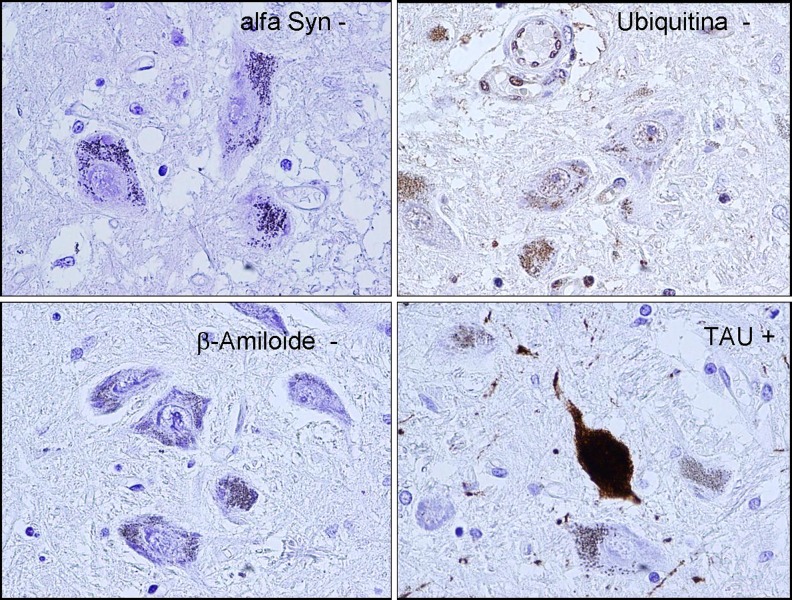


**PS-15-036**

**Transforming growth factor beta immunoexpression in cerebral cortex and basal ganglia regions in case of chronic alcoholism**

S. Skuja ^*^, V. Groma, K. Ravina, O. Teteris

^*^Riga Stradins University, Laboratory of EM, Latvia

**Objective:** Strongly varying data exist on distribution of transforming growth factor beta (TGFβ1) and its potential role. Elevated TGFβ1 expression was observed in ischemia, trauma, inflammation and neurodegeneration; decreased–in altered axonal transport, neuronal injury. We aimed to assess TGFβ1 expression in different CNS regions in case of addiction.

**Method:** Immunohistochemical performance on 39 alcoholics and 12 controls was studied by semiquantitative counting of TGFβ1 expression and non-parametric tests.

**Results:** Results on TGFβ1 expression (median (range)): in the neuropil, somata and white matter of substantia nigra 0.2 (0.0; 0.8), 1.0 (0.0; 6.0), 1.0 (0.6; 1.5) and 0.4 (0.0; 1.0), 0.0 (0.0; 1.0), 1.4 (0.8; 2.1) in alcoholics and controls, respectively. Corpus striatum neuronal expression in alcoholics was 8.5 (2.0; 19.0) compared with control 1.0 (0.0; 12.3), in small tracts expression was 0.0 (0.0; 0.6) and 0.7 (0.4; 0.9), whereas in subcortical white matter–0.9 (0.6; 1.0) and 1.6 (0.9; 2.0), respectively.

**Conclusion:** Quantitation of TGFβ1 expression in different CNS regions is a useful tool ranging TGFβ1 involvement in cellular adaptation and defense mechanisms in case of alcoholism.

**PS-15-037**

**Analysis of histopathological findings in Romanian limb girdle muscular dystrophy 2A patients**

F. Staniceanu ^*^, A. Bastian, G. Gaina, E. Manole, S. Zurac, G. Micu, C. Socoliuc, E. Gramada, C. Popp

^*^Colentina University Hospital, Pathology, Bucuresti, Romania

**Objective:** Our study correlates detailed clinical data with morphological aspects and calpain 3 protein expression on muscle biopsies and immunoblotts in patients suspected to have limb girdle muscular dystrophy (LGMD), to establish a better diagnostic strategy prior to current genetic testing of the CAPN 3 gene.

**Method:** We performed opened biopsies of clinically selected skeletal muscles and applied histological, histochemical, histoenzimological stains on fresh frozen tissue, followed by immunohistochemical techniques and western blotting for several proteins known to be involved in LGMD.

**Results:** Our study revealed that primary calpain 3 partial/complete deficiency appears to be the most frequent form of LGMD in the Romanian population, with high phenotypic variability both in adult and infantile cases. The identified morphological changes in the muscle varied from mild nonspecific myopathic aspects to severe dystrophic picture, but unusual neurogenic-like or focal pseudo-inflammatory patterns were identified.

**Conclusion:** Calpainopathy should be included on the differential diagnosis list of many muscular diseases; muscle biopsy from patients with suggestive clinical phenotype allows the assessment of primary and secondary protein reductions and is the fastest preliminary guide for further genetic testing. Stăniceanu and Bastian have equally contributed.

**PS-15-038**

**Aprikalim a K+-ATP channel opener prevents ischemic spinal cord injury in rabbits through inhibition of apoptosis and modulation of nitric oxide synthase expressions**

G. Agrogiannis ^*^, V. Lozos, I. Toumpoulis, E. Patsouris

^*^University of Athens, 1st Dept. of Pathology, Greece

**Objective:** To determine the effects of aprikalim, a K+-ATP channel opener, on apoptosis and on the expressions of nitric oxide synthase (NOS) in a rabbit model of spinal cord ischemic injury.

**Method:** Fifty-four rabbits were randomly assigned to three groups: group I (*n* = 18, sham operation), group 2 (*n* = 18, 30 min of normothermic aortic cross-clamping) and group 3 (*n* = 18, aprikalim 100 μg/kg was administered 15 min before 30 min of normothermic aortic cross-clamping). Six animals from each group were sacrificed at 24, 48 and 168 h postoperatively. The lumbar spinal cords were harvested and immunohistochemistry was performed to determine the expression of apoptosis inducing factor (AIF), caspase-3 and the expression of neuronal, endothelial and inducible NOS (nNOS, eNOS and iNOS).

**Results:** Aprikalim exhibited improved effect on clinical and histologic neurologic outcome when compared to group II (*P* < 0.05). Aprikalim inhibited apoptosis via caspase-dependent and -independent pathways (*P* < 0.05 vs. group II). In addition, aprikalim inhibited the expression of iNOS and increased the expression of eNOS at 24 and 48 h (*P* < 0.05 vs. group II) and showed no influence on nNOS expression when compared to group II.

**Conclusion:** The results indicate for the first time that aprikalim provides neuroprotection against ischemic spinal cord injury through inhibition of apoptosis and iNOS and overexpression of eNOS.

**PS-15-040**

**Progressive multifocal leukoencephalopathy in an immunocompromised patient treated for diffuse large B cell lymphoma**

G. Karagkounis ^*^, T. Argyrakos, D. Riga, P. Constantinou, S. Korfias, G. Stranjalis

^*^Evaggelismos Hospital, Dept. of Pathology, Athens, Greece

**Objective:** Progressive Multifocal Leukoencephalopathy (PML) is a demyelinating disease of the Central Nervous System caused by the John Cunningham (JC) virus. It is encountered exclusively in patients with deficits in cellular immunity (HIV/AIDS, chemotherapy, transplantation or patients with Crohn’s disease receiving monoclonal antibodies like natalizumab). We describe the case of a 56-year-old man with a history of mixed connective tissue disease and DLBCL who presented with multiple lesions in the cortex and white matter.

**Method:** A stereotactic biopsy was performed and the tissue was studied with histochemical and immunohistochemical stains.

**Results:** Histology revealed a CD68+ macrophage-rich lesion containing many reactive astrocytes with bizarre nuclei and oligodendrocytes with ground glass nuclei and homogenous amphophilic intranuclear inclusions. The atypical cells coexpressed GFAP, p53 and ki-67 and exhibited positivity for SV-40 T antigen which reacts with JC virus as well as other members of the polyoma virus family (BK, KI, WU and Merkel virus). Kluver-Barrera and MBP stains revealed loss of myelin sheaths in demyelinating areas and myelin debris inside the histiocytes.

**Conclusion:** PML resembles histologically astrocytoma or oligodendroglioma. JC virus binds and stabilizes protein p53. The numerous macrophages as well as the SV40-Τ/ki-67/p53 positivity of the atypical glial cells verifies the diagnosis of PML.

**PS-15-041**

**Cerebral sparganosis**

L. Daoud ^*^, J. Fortes, C. Medina, E. Gavín, F. Manzarbeitia

^*^Fundación Jiménez Díaz, Pathology, Madrid, Spain

**Objective:** Case report: We are reporting the case of a 29 year old patient from Bolivia with history of seizures for about 2 years. The MRI finding was a multicystic cortical lesion in the left temporal lobe with smooth ring enhancement. The surgical excision revealed an white encapsulated cyst with 2 cms, filled with a transparent fluid. Histological study showed a multicystic lesion with parietal granulomatous reaction containing the larval form of Sparganum mansoni. The body wall of the worm consisted of a tegument with microvilli, layers of smooth muscle and tegumental cells. In the parenchyma we observed loose stroma, calcareous bodies and smooth muscle.

**Conclusion:** Sparganosis is a rare parasitic infection in humans by a larval cestode of the genus Spirometra. It is an uncommon condition, particularly in Europe. It is most frequently seen in Asia but may be found anywhere in the world. Differential diagnosis must be done with other parasitic cystic cerebral infections such as cerebral cysticercosis, hydatidosis and gnathostomiasis.
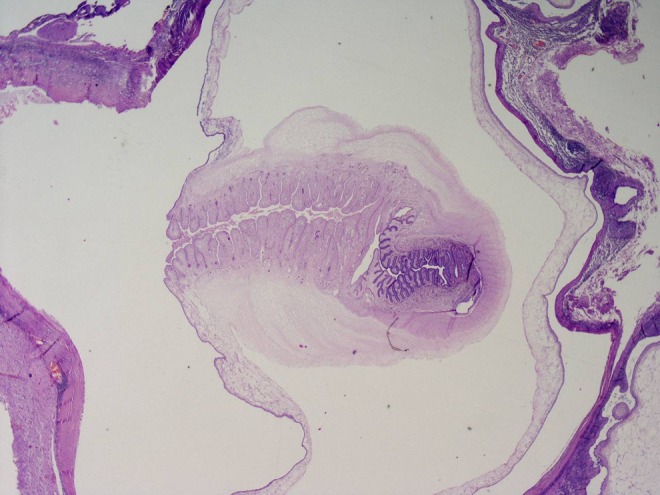


Monday, 2 September 2013, 09.30–10.30, Pavilion 2

**PS-16 Poster Session Pulmonary Pathology**

**PS-16-001**

**Epidermal growth factor receptor mutations in primary and metastatic adenocarcinomas from a tertiary hospital in São Paulo, Brazil**

V. de Sá ^*^, E. Nascimento, S. Meireles, V. Capelozzi

^*^Faculdade de Madicina, Dept. of Pathology, São Paulo, Brazil

**Objective:** Recently, the epidermal growth factor receptor (EGFR) mutation emerges promise as target for molecular therapy in Adenocarcinomas of the lung. However, a number of clinical features are associated with EGFR mutations: women, never-smokers than former or current smokers and Asians than other ethnic groups. The aim of this study was to evaluate the frequency and distribution of EGFR mutations in 100 consecutive patients with surgically excised primary and metastatic Adenocarcinomas.

**Method:** Direct bidirectional sequencing evaluated EGFR gene mutations on exons 18 to 21 and was correlated with ethnia (East-Asian or European), gender, age, tobacco history, primary (*N* = 75) or metastatic (*N* = 25) and histologic subtypes.

**Results:** Twenty-eight tumors (28 %) exhibited EGFR mutation. The most frequent EGFR mutation detected was a deletion in exon 19 (50 %), followed by multiple mutations in the exon 20 (28 %) and an L858R amino acid substitution in exon 21 (21,4 %). EGFR mutation was significantly associated with men (*N* = 59), older patients (>60 year), smokers, non-East Asian or non-European origins, primary tumor and acinar predominant histologic subtype.

**Conclusion:** Our results indicate that if the current clinical features were strictly followed as the criteria for selecting patients for EGFR testing, a substantial number of patients who might benefit from treatment will be excluded.

**PS-16-002**

**Precised diagnosis of histological type of non-small cell lung carcinoma on small-sized biopsy samples: The first step to the individual target therapy**

J. Stojsic ^*^, J. Markovic, I. Jovanic

^*^Clinical Centre of Serbia, Service of Pathology, Belgrade, Serbia

**Objective:** Target therapy increases survival rate and quality of life of lung cancer patients but requests precise subtyping of non-small cell lung carcinoma (NSCLC). The aim is to evaluate 6 monoclonal antibodies in differential diagnosis of NSCLC on small tissue samples.

**Method:** 50 small tissue samples obtained on bronchoscopy or fine needle aspiration. According to morphology on routinely fixed and H&E stained small-sized biopsies 2 squamous cell carcinomas (SCC), 6 adenocarcinomas (AC), 9NSCLC-probably SCC, 11NSCLC-probably AC and 22 unclassified NSCLC were diagnosed. TTF-1, cytokeratin5/6, cytokeratin7, p63, CD56 and synaptophisin were used in differentiation NLCLC according to manufacturer prescription.

**Results:** After immunohistochemistry 13(26.0 %) SCC, 27(54.0 %) AC, 3(6.0 %) NSCLC with neuroendocrine differentiation (LCLC-NE) and 7(14.0 %) NSCLC- unclassified was diagnosed. 22NSCLC- unclassified were diagnosed as 7SCCs and 7ADCs, 2LCLC with neuroendocrine differentiation and 6NSCLC–unclassified. Significant difference was found between finally diagnosed 7NSCLC-unclassified and 15AC (20.5 % versus 38.5 %, *p* = 0.008). TTF-1 and Cytokeratin7 were expressed in 85.2 % (23/27) AC, respectively and Cytokeratin5/6 and p63 in 100 %(13/13)SCC, respectively. Positivity of CD56 and synaptophisin in 3LCLC determinate as LCLC-NE.

**Conclusion:** Combination of TTF-1, cytokeratin7, p63, cytokeratin5/6, CD56 and synaptophisin allows differentiation of NSCLC.

**PS-16-004**

**Lung adenocarcinomas in Algerians**

Z.-C. Amir-Tidadini ^*^, F. Asselah

^*^CHU Mustapha, Dept. of Pathology, Algiers, Algeria

**Objective:** To delineate the clinicopathological features of lung adenocarcinomas in Algerians using the two histological classifications (WHO 2004, IASLC 2011)

**Method:** Review of consecutive cases of lung adenocarcinomas (AD) from paraffin archived material (Jan. 2008 to Dec. 2011): usual histology (WHO 2004/IASLC 2011) and immunohistochemistry (IHC) (TTF-1, CK7, CK20, p63 or CK5/6…), molecular testing for 20 patients.

**Results:** 415 lung cancer: 92,2 % of NSCLC including 206 AD (54 %): patients aged 31–87 years (m: 55 years, pick: 50–70 y), M/F = 6, 84 % smokers; tumour size: 3–13 cm (m: 7 cm); 82 % mixed AD versus 47 % acinar and 43 % solid predominant invasif AD. 2/20 patients had EGFR mutation, 1/7 patients with ALk translocation.

**Conclusion:** Primary lung AD is prevalent in Algeria; more of them are of the mixed type versus acinar and solid, IHC can differentiate them from metastasis; most are diagnosed at an advanced stage. Identification of driving mutations (EGFR, KRAS, EML4-ALK) achieved in a few cases, is crucial for individualized treatment of patients. Challenges include collecting sufficient tissue from little biopsy specimen for analysis, and performing accurate profiling with validated biomarker assays in our department.

**PS-16-005**

**Napsin A is a useful marker for metastatic adenocarcinomas from the lungs**

M.-Y. Kim ^*^, H.-J. Go, J.-M. Koh, H.-S. Min, M.-A. Kim, Y.-K. Jeon, H.-S. Lee, K.-C. Moon, S.-Y. Park, W.-H. Kim, D.-H. Chung

^*^Seoul National University Hospital, Dept. of Pathology, Republic of Korea

**Objective:** Napsin A (NapsA) has been known to be an excellent marker for pulmonary adenocarcinomas. However, it is unclear whether NapsA is useful to determine metastatic adenocarcinomas of pulmonary origin. In this study, we addressed this issue.

**Method:** Using tissue microarray, 54 cases of metastatic adenocarcinomas from the lungs and 1,776 cases of adenocarcinomas in various organs (breast, colon, endometrium, kidney, liver, ovary, prostates, stomach, and thyroid) were immunohistochemically stained for NapsA, TTF-1, CK7, CK20, and CDX2. The scores were calculated by adding expression intensities and proportions of NapsA or TTF-1-positive tumor cells.

**Results:** Among metastatic adenocarcinomas from the lungs, NapsA, TTF-1, and NapsA/TTF-1 were positive in 87.0 %, 81.5 %, and 90.7 %, respectively. There was no statistically difference between NapsA and TTF-1 as a single marker for pulmonary adenocarcinomas (*p* = 0.157), although mean scores for NapsA staining (4.02) was higher than TTF-1 (2.89) (*p* = 0.000). All extrapulmonary adenocarcinomas were negative for NapsA except 13.0 % of renal cell carcinoma, 7.1 % of ovarian adenocarcinoma, and 14.5 % of endometrial adenocarcinoma. Interestingly, clear cell adenocarcinomas in the ovary (68.8 %) and endometrium (66.7 %) highly expressed NapsA.

**Conclusion:** NapsA may be a useful immunohistochemical marker to determine metastatic adenocarcinomas with pulmonary origin.

**PS-16-006**

**Intestinal type of lung adenocarcinoma in younger adults**

J. Stojsic ^*^, M. Kontic, D. Subotic, M. Popovic, D. Tomasevic, J. Lukic, J. Markovic

^*^Clinical Centre of Serbia, Service of Pathology, Belgrade, Serbia

**Objective:** Intestinal type of lung adenocarcinoma (ILADC) is a rare entity, diagnosed in patients from 50 to 80-years. Tha aim is to alert with ILADC, diagnosed in young adults, in which histologic and immunohistochemical findings were indistinguishable from colorectal adenocarcinoma.

**Method:** ILADC was diagnosed on operative tissue sections, routinely fixed and H&E stained and immunohistochemicaly confirmed by: Cytokeratin20, CDX-2, Villin, MUC-1 and MUC-2 and to exclude usual type of lung adenocarcinoma: Cytokeratin7, TTF-1, Napsin-A and SurfactantB. PCR primers were designed to amplify 2. exon of K-ras and 18,19,20,21. exon of EGFR gene. PCR products were sequenced by bidirectional dye-terminator fluorescent sequencing. Colorectal endoscopy was performed to exclude intestinal origin.

**Results:** 25-year female and 27-year-old male patient with radiologicaly detected lung tumor went under lobectomy. In both patients ILADC was diagnosed and confirmed immunohistochemicaly. In both patients mutations in EGFR gene were not detected. In male patient we detected mutation in 2. exon 12. codon of K-ras GGT->GAT,Gly->Asp. Colonoscopy in the both patients did not reveal intestinal origin of adenocarcinoma.

**Conclusion:** More experience is needed for further understanding of ILADC specially in younger adults. We suggest introducing of EGFR and KRAS mutation status analysis in the purpose of personalized lung cancer therapy of ILADC.

**Detected mutation in 2. exon 12. codon of K-ras:** 
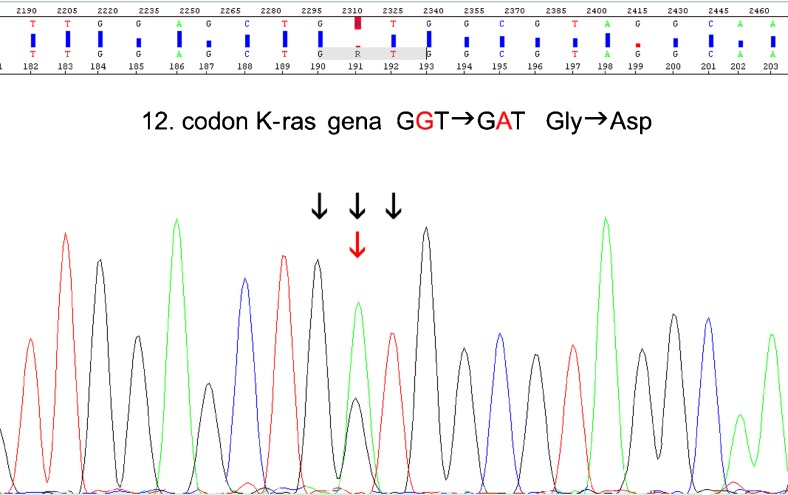


**PS-16-007**

**Frequency and immunohistomorphological profile of the lung adenocarcinomas with activating mutations of the Epidermal Growth Factor Receptor (EGFR) gene in 435 Non-small Cell Lung Carcinomas (NSCLC)**

J. Nieslanik ^*^, J. Dvorackova, M. Uvírová, D. Ziak, D. Konvalinka, J. Zmolikova, S. Laciok, R. Ondrussek, V. Zidlik

^*^CGB Laboratory a.s., Dept. of Pathology, Ostrava-Vítkovice, Czech Republic

**Objective:** Currently, activating mutations are the best predictive markers of responsiveness to EGFR tyrosine kinase inhibitors in NSCLC. The most commonly observed aberrations are deletions in exon 19 and point mutation L858R in exon 21, which are associated with response rates to EGFR tyrosine kinase inhibitors of approximately 70 %.

**Method:** DNA is isolated from biopsy and cytology specimens with verified histological diagnosis of NSCLC. Mutation is performed by real–time PCR, fragment analysis and mutant enriched PCR. Standard histological and immunohistochemical stains (HE, PAS, TTF 1, CK 7, CK 5/6, P 63,) were used.

**Results:** Since 10/2010 till 03/2013 in 435 patients with histological diagnosis of NSCLC their DNA samples were analysed. Out of these 25 patients (6 %) were found positive for activating mutations within EGFR gene. Out of these 16 patients (60 %) suffered from the lung adenocarcinoma. Detailed analysis of histological pattern of the above mentioned 16 cases of lung carcinomas is stated in the table.

**Conclusion:** Determination of tumor mutational status and histological typing can provide powerful tool for setting up strategy and therapeutic protocols in NSCLC.

**PS-16-008**

**Heat shock protein 27 immunohistochemical expression as a prognostic factor in patients operated for small cell lung carcinoma or large cell neuroendocrine carcinoma**

D. Marinova^*^, Y. Slavova, N. Trifonova, V. Maksimov, D. Petrov

^*^UHPD, Dept. of Pneumology, Sofia, Bulgaria

**Objective:** To study the prognostic significance of heat shock protein 27 (Hsp27) expression in operated for small cell lung carcinoma (SCLC) or large cell neuroendocrine carcinoma (LCNEC) patients.

**Method:** Surgically resected specimen from 51 SCLC and 15 LCNEC patients were studied (men *n* = 51; women *n* = 15, age 59 ± 7). The Hsp27 expression was detected by immunohistochemistry. Statistical methods: Kaplan-Meier, Cox regression analysis.

**Results:** Hsp27 positive immunoreaction in the cytoplasm was observed in 45 (88 %) SCLCs and 14 (93 %) LCNECs. The median survival time was significantly longer in cases with >65 % Hsp27 cytoplasmic positivity rate (percent tumor cells) compared to cases with ≤65 % rate (*p* = 0.035). The Cox regression multivariate analysis showed a significant survival advantage for >65 % Hsp27 rate positive cases compared to ≤65 % cases (Hazard ratio[HR]2.00; *p* = 0.04). А combination of cytoplasmic with nuclear Hsp27 expression was observed in 28 SCLCs and 14 LCNECs. The median survival time was significantly longer in cases with Hsp27 nuclear positivity compared to negative cases (*p* = 0.035). The Cox regression multivariate analysis showed a significant survival advantage for Hsp27 nuclear positive cases compared to negative cases (HR 2.4;*p* = 0.014).

**Conclusion:** Hsp27 expression in >65 % of the tumor cells and positive nuclear Hsp27 expression may represent favorable prognostic factors in SCLC and LCNEC.

**PS-16-009**

**Pulmonary giant cell carcinoma with lepidic growth pattern and hemophagocytosis in a patient with hemoptysis and aspergillosis**

U. Gruber-Moesenbacher ^*^, E. Wenzl, P. Cerkl, H. Popper

^*^Landeskrankenhaus Feldkirch, Abt. Pathologie, Austria

**Objective:** Diffuse lung infiltrates with partial destruction of the lung usually are regarded as an inflammatory process. Continuing hemorrhage can be suspicious of malignancy. Mainly mucinous adenocarcinomas grow diffusely, not tending to bleed.

**Method:** A 72 years old male cigarette smoker suffered from recurrent hemoptysis, increasing pulmonary infiltrates in the right upper lobe, not responding to antibiotic therapy. Pneumonectomy was performed because of persistent hemoptysis and biopsy-diagnosis of malignancy

**Results:** Intended lobectomy was extended to pneumonectomy because of multifocal diffuse tumor infiltrates in the whole right lung. Histology: large, often multinucleated tumor cells, frequently stuffed with erythrocytes, growing in a lepidic manner. Invasive growth only in 10 % of tumor blocks, lymphnode metastases in two nodes less than 8 mm. The upper lobe contained aspergillus fumigatus, surrounded by organizing pneumonia with hemorrage and disseminated tumor cell groups. Final diagnosis: predominantly lepidic multifocal giant cell carcinoma with pleural and vascular invasion and hilar lymph-node metastases (G3 pT4 N2 (2/21) R0 V1). KRAS-mutation: p.G12A. 18 days after surgery the patient died of pulmonary embolism.

**Conclusion:** The etiology of hemophagocytosis by epithelial tumor cells needs further investigation with regard to possible therapy. Lepidic growth of giant cell carcinoma has neither been mentioned in the 2004 WHO classification nor in the ATS-ERS IASLC classification 2011.

**Lepidic growth and hemophagocytosis:** 
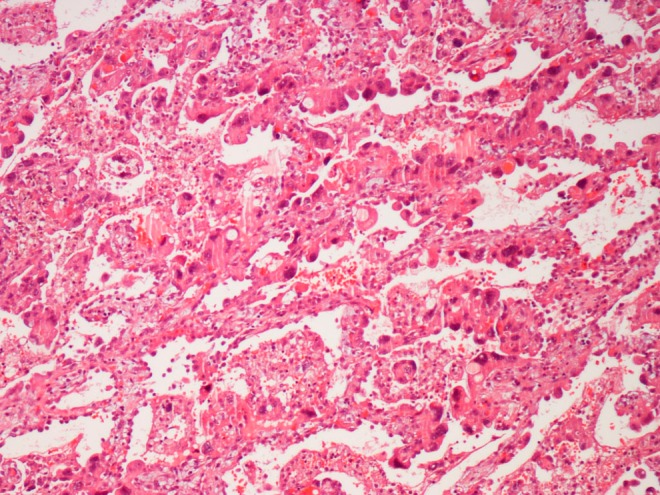


**PS-16-010**

**Unusual cause of hemoptysis in a 49-year old female patient**

U. Gruber-Moesenbacher ^*^

^*^Landeskrankenhaus Feldkirch, Abt. Pathologie, Austria

**Objective:** Expectoration of fresh blood has to be investigated stepwise for exclusion of malignancy and to avoid overtreatment. Beside highly vascularized benign and malignant tumors, infarcts, vascular malformation and granulomatous infection can cause localized pulmonary hemorrhage.

**Method:** A 49 years old light female smoker with COPD and DM, was admitted to the hospital after ORL finding of fresh blood on the larynx. Cytology of the bronchial secretion, microbiological cultures, bronchoscopy, spiral CT and 18FDG-PET did not result in a final diagnosis, so wegde resection was performed.

**Results:** In frozen section of 5 cm measuring subpleural lung parenchyma we found focal intraalveolar hemorrhage, corresponding to radiological ground glass opacity, no tumor. In serial sections a 2 mm focus of endometrium was found in the lung parenchyma beside an isolated focus of lambertosis, which was positive in TTF1. CD10 decorated the endometrial stroma. Hemoptysis was terminated by the resection for months.

**Conclusion:** Pulmonary endometriosis is an etiologically unknown process, mostly affecting the pleura, associated with uterine surgery. Hemoptysis caused by endometrial foci in the lung parenchyma can be reduced by hormonal ablatio.

**Focus of endometrium in lung parenchyma:** 
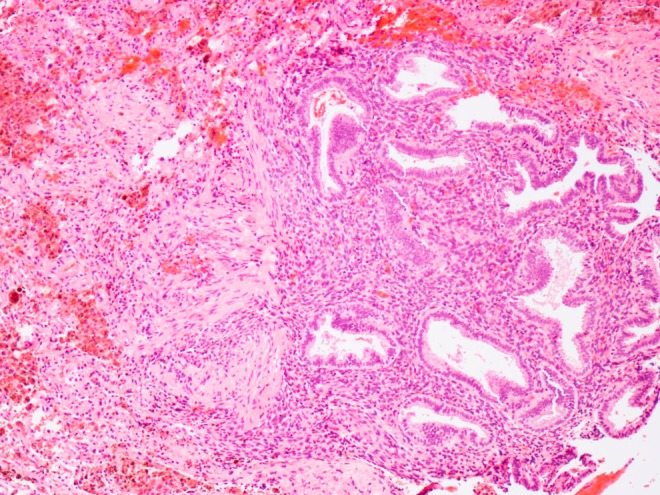


**PS-16-011**

**Molecular diagnosis of sentinel lymph nodes for lung cancer**

A. Cremades Mira ^*^, A. Zuñiga Cabrera, J. M. Galbis Caravajal, M. Estors Guerrero, A. Tembl Ferrairó, M. Navarro Hervás

^*^Hospital Universitario Alzira, Dept. de Anatomía Patológica, Spain

**Objective:** Sentinel lymh nodes (SLN) mapping and molecular staging to improve the detection of lymph nodes (LN) micrometastases in order to evaluate an improvement of staging in early non-small cell lung cancer (NSCLC) patients.

**Method:** Forty-two patients diagnosed cT1-2aN0M0 (stage I) NSCLC were enrolled in the study between March and December 2012. Reverse transcriptase-polymerase chain reaction (RT-PCR) analysis for cytokeratin 7 (CK7) and 19 (CK19), CEACAM5 and PLUNC was used to identify tumor-derived material in LN. Samples from dissected SLN were sectioned and conventionally examined using haematoxylin and eosin staining and immunohistochemical analysis. In addition, one section of each SLN was snap-frozen to −80 C for RNA-detection of CK7, CK19, CEACAM5 and PLUNC.

**Results:** Of 42 patients, 13 were excluded (2 benign tumors, 3 metastatic tumors, 8 patients with stage II). SLN mapping was performed in 29 patients. Overall, 7 SNLs (24 %) were positive in molecular study. It was found a positive SNL in 4/11 adenocarcinomas and 3/15 squamous. Affected LN were: station 5 (1/3), station 7 (0/6), station 9 (0/1); station 10 (5/11); station 11 (1/1).

**Conclusion:** RT-PCR of CK7, CK19, CEACAM5 and PLUNC can estimate the presence of metastatic cells with greater precision than current staging methods, and may be useful for stratifying histologically N0 patients.

**PS-16-012**

**A useful guideline for determining histological subtypes of non-small cell lung cancers that show equivocal expression of p40 and Napsin A in small biopsies**

J.-M. Koh ^*^, D.-H. Chung, Y.-K. Jeon, H.-J. Go, M.-Y. Kim

^*^Seoul National University, Dept. of Pathology, Republic of Korea

**Objective:** Napsin A (NapsA) and p40 have been introduced as sensitive and specific markers of pulmonary adenocarcinomas (ADCs) and squamous cell carcinomas (SCCs), respectively. However, there is lots of trouble in histologic distinction of nonsmall cell lung cancers (NSCLCs) which show ambiguous NapsA and p40 expressions on small biopsies.

**Method:** Twenty two cases were included that the tumor (1) had both biopsy and subsequent resection specimens, (2) diagnosed as NSCLC on biopsies and (3) showed NapsA/p40 (−/−) (DN) or (+/+) (DP). Histologic features were confirmed with resections. Immunohistochemistry for TTF-1, CK7, CK5/6 and p63 was additionally performed.

**Results:** Fourteen cases were DN in biopsies (6 ADCs, 5 SCCs and 3 others) and 8 cases were DP (4 ADCs, 2 SCCs and 2 others). Of 6 DN ADCs, TTF-1 and CK7 was positive in 3 and all cases, respectively and, of 4 DP cases, p63 and CK5/6 were negative in 2 same cases. Of 5 DN SCCs, p63 and CK7 was positive in 4 cases each. TTF-1 and CK7 were all negative in 2 DP SCCs.

**Conclusion:** These findings suggest that expression patterns of CK7/TTF-1 and p63/CK5/6 may be useful for determining histological subtypes of NSCLCs, which equivocally express NapsA and p40 in small biopsy.

**PS-16-013**

**Comparison of lung adenocarcinoma developed in genetically engineered mouse to human adenocarcinoma subtypes–similarities and differences**

H. Popper ^*^, B. Grabner, S. Rao, E. Casanova

^*^Medizin. Universität Graz, Inst. für Pathologie, Mol. Lung und Pleura Pathologie, Austria

**Objective:** Lung adenocarcinoma (AC) induced by carcinogen inhalation never came close to the human counterpart. This has changed with the creation of genetically engineered models.

**Method:** Different types of mouse AC were studies, all of them with a KRASV12 background, but with additional hits (ATG5, PTEN, PI3K, p53). Development of AC was followed for 28 weeks. Human lung AC subtypes and precursor lesions served for comparison.

**Results:** AC starts with papillary growth at the bronchoalveolar junction (baJ). Tumor spreads into the alveolar periphery. Due to different cell to alveolar size alveoli are filled almost completely, simulating solid growth. At certain size hypoxic necrosis is seen in centers, followed by neoangiogenesis and stroma formation. Finally invasion of tumor cells into stroma occur. Metastasis is rare, due to high tumor load, causing early death. Vascular invasion is seen, if second carcinogenic hits are applied.

**Conclusion:** Dissimilarities with human precursor and AC types are: no mucinous AC, predominant papillary or solid AC, high incidence of focal signet ring cell formation, requirement of large tumor size before invasion and metastasis. To understand AC development knowledge of the anatomy and histology of mouse and human lung is necessary, but these models open a new way of investigating lung AC.

**PS-16-014**

**Agreement between size of resected specimen and preoperative tumor measurement on CT in non-small-cell lung cancer**

C. Callé ^*^, J. Ip, S. Esteves, N. Abecassis, I. Duarte, M. T. Almodovar, F. Cunha

^*^IPO Lisboa, Anatomia Patológica, Portugal

**Objective:** To evaluate the agreement of pathology gross-specimen with CT-scan tumor measurements in non small-cell lung cancer(NSCLC).

**Method:** 88 patients with primary NSCLC CT-staged T1 or T2, submitted to surgery (Jan2008–Dec2011). Largest tumor diameter was reviewed from pathology gross reports and CT-scan images (PACS caliper segmentation algorithm adjusted with radiologist input). A paired *t*-test was used to evaluate the differences between pathology and CT measurements. Agreement between pathology and CT measurements and T-staging were evaluated using Bland-Altman Methods and Cohen-Kappa.

**Results:** The mean of pathology measurement was 30,63 mm and of CT was 30,27 mm. The mean difference between pathology and CT measurements was −0,35 (95 % Confidence Interval −2,15; 1,45; lower and upper 95 % limits of agreement were −17,33 mm and 16,62 mm). The pathology T-staging (T1A = 30, T1B = 22, T2A = 27, T2B = 9) and clinical T-staging CT based (T1A = 21, T1B = 34, T2A = 20, T2B = 13) had moderate agreement (Cohen-Kappa = 0,491, *p*-value < 0,001). Stage agreement was seen in T1A = 17/30 (57 %), T1B = 16/22 (73 %), T2A = 14/27 (52 %) and T2B = 8/9 (88 %).

**Conclusion:** There was moderate agreement between Pathology and CT measurements and between pathological and clinical T-stage. These results may have prognosis implications in patients with early stage of NSCLC based on clinical staging only.

**PS-16-015**

**Non-small-cell lung cancer: Morphologic features in CT-scan cannot predict histological type: Revision of 77 cases**

C. Callé ^*^, J. Ip, S. Esteves, N. Abecassis, I. Duarte, M. T. Almodovar, F. Cunha

^*^IPO Lisboa, Anatomia Patológica, Portugal

**Objective:** To evaluate the association between CT-scan features with lung cancer histological type: adenocarcinoma(ADC) and squamous cell carcinoma(SCC).

**Method:** 77 patients, submitted to pulmonary lobectomy between January 2008 and December 2011. Histological diagnosis was recorded: 46 ADC and 31 SCC. Margins and opacity were reviewed in CT-scans. Margins were classified as: regular margins (RM), irregular margins (IM), or spiculated margins (SM). Opacity as ground glass opacity (GGO), mixed opacity (MO) or solid opacity (SO). To test the association between histological type with opacity and margins Chi-Square test was used.

**Results:** RM were registered in 10 cases (7ADC, 3SCC), IM in 16 (6ADC, 10SCC) and SM in 51 cases (33 ADC, 18 SCC). GGO was documented in 19 cases (10ADC, 9SCC), MO in 30 cases (19ADC, 11 SCC) and SO in 28 cases (17ADC, 11SCC). The Chi-Square test showed no association between histological type and CT-scan density, (*p*-value = 0,75), neither between histological type and margins (*p*-value = 0,15).

**Conclusion:** CT-scan is the most used image method to evaluate and to stage non-small-cell lung cancer. However the morphological features evaluated in this exam do not allow it to predict histological type. Histological diagnosis is mandatory for therapeutic decision.

**PS-16-016**

**P53 and RAS genes expression and correlation with proliferation index in squamous cell lung carcinomas**

P. Mikou ^*^, N. Kalogeropoulos, A. M. Athanassiadou, M. Veslemes, P. Athanassiades, I. Iordanoglou

^*^Laiko Hospital, Dept. of Cytopathology, Athens, Greece

**Objective:** Oncosuppresor gene p53 and ras oncogene are important for carcinogenesis, as they are frequently detected in human tumours. Our aim is to investigate their immunohistochemical expression and their correlation with PCNA index and major clinicopathologic prognostic parameters in squamous cell lung carcinomas.

**Method:** We have included 85 cases of surgically resected squamous lung tumours in male patients, stage I to IIIA. P53 and ras protein and PCNA expression were detected by immunohistochemistry. Statistical analysis of the results was performed by ×2 and ANOVA test.

**Results:** There was positivity for p53 in 34,1 % and ras in 57,7 % of the cases. No correlation between p53 expression and grading, tumour size, lymph node involvement, stage and proliferation index. P53 and age showed negative correlation. No correlation between ras expression and age, grading, lymph node involvement, local infiltration, stage and proliferation index. Ras showed borderline negative correlation with tumour size.

**Conclusion:** P53 and ras protein are expressed in squamous cell lung carcinomas reflecting their important role in carcinogenesis. Correlations of p53 and ras observed in our study imply the involvement of p53 and ras mutations in early phases of tumorigenesis. Lack of correlation with major prognostic parameters signifies independent prognostic significance of these markers.

**PS-16-017**

**Correlation between EGFR expression, gene amplification, mutation and KRAS mutation in lung adenocarcinomas**

G. Bulbul Dogusoy ^*^, N. Bassullu, I. Coban, V. Hancer, M. Buyukdogan, E. Namal, A. Toker, R. Yasar, G. Demir

^*^FLORANCE NIGHTINGALE, Istanbul, Turkey

**Objective:** It was aimed to investigate EGFR protein expression, gene amplification and EGFR and KRAS mutations in lung carcinomas.

**Method:** EGFR status was analyzed in 6 bronchoscopic biopsies, 9 fine needle aspiration cell blocks, 8 surgically resected lungs and 33 paraffin blocks of consultation. Carcinoma samples of 61 patients were examined by three methods: protein expression (*n* = 25) by standardized immunohistochemistry (IHC), gene amplification (*n* = 36) by fluorescence or chromogen in situ hybridization (FISH, CISH), and mutation analysis by real-time polymerase chain reaction (*n* = 50 for EGFR, 9 for KRAS).

**Results:** The results showed that 48 % of the carcinomas were adenocarcinomas, 18 % were others and 34 % were nonsmal cell carcinomas NOS. 7.1 % of samples were positive by IHC, 10.7 % positive by FISH-CISH, and 17.9 % contained activating kinase domain mutations. EGFR and KRAS mutations were more frequent in patients with adenocarcinomas (*p* = 0.00), and these mutations were not associated with FISH positivity. When using H score, EGFR protein expression was not correlated with EGFR FISH positivity and mutations.

**Conclusion:** EGFR mutation was correlated with adenocarcinoma type however not showed any relationship with EGFR protein expression and EGFR gene amplification in our study. Standard methods and interpretation criteria need to be established.

**PS-16-018**

**Angiogenesis in stages I, II and IIIa of lung squamous cell carcinoma**

L. Vuckovic ^*^, F. Vukmirvic, T. Nenezic

^*^Clinical Center Montenegro, Pathology, Podgorica, Montenegro

**Objective:** Currently the best prognostic factor for operable lung squamous cell carcinoma (LSCC) is the TNM staging system. Despite potentially curative surgical resection, patients with the same pathological stage of disease display marked variablity in reccurence and survival. Increasing knowledge of the pathohistological prognostic factors of tumors may allow us to predict the outcome for the individual patient.

**Method:** A retrospective study included 120 patients with LSCC. All of the patients were in stage I, II or IIIa. Pathological stage of each tumour was recorded using TNM staging system. For immunohistochemistry was used CD105 antibody.

**Results:** Mean microvessel density (MVD) in the study was 30,10 ± 7,65. According to that value, patients were separated in 2 groups: with MVD less then mean average and with MVD over the mean average. Statistical analysis were done. According to results of the analysis there is no significant statistical difference of microvessel density in groups of patients in stage I and II. Analysis of microvessel density in stages I or II with IIIa stage of disaese shows significant statistical difference, with higher microvessel density in group of patients in stage IIIa.

**Conclusion:** Microvessel density is higher in advanced stages of lung squamous cell carcinoma.

**PS-16-020**

**Quantitative measurements of cell density and proliferative markers by image analysis of lung (non small-cell) carcinoma**

A. Kudaybergenova ^*^, L. Stelmakh, I. Dvoravskaya

^*^RNCRCT, Pathology, St. Petersburg, Russia

**Objective:** We analyzed a total number of tumor cells in NSCLC and % Ki67-positive cells to establish absolute quantity of tumor cells per sq.mm of histological slide, and the relations of this measure with proliferation.

**Method:** The study included 21 patients with NSCLC. After whole slide scanning by Mirax scanner (3DHistech, Budapest) of Ki67 stained slides, we located the areas within the tumors with maximal Ki67 levels and calculated the precise quantity of tumor cells per sq.mm, which was the sum of negative and Ki67 positive cells. Morphometric analysis was performed using the Pannoramic Viewer software (3DHistech, Budapest). For each case we analyzed a total number of tumor cells in a 1 mm2 sample and Ki67 was evident in at least 10 fields of view.

**Results:** Mean tumor cells in 1 mm2 of histology slide was 4,102 +\\− 364 cells, median 4,163 cells among them 1,294 +\\− 166 (15 %) were positive for Ki67. There was moderate correlation between cell density and Ki67 *r* = 0,41 (*p* = 0,0032). Survival analysis snows 40 % survival rate in groups with high levels of cell density (more than median) compared with 10 % in groups with low levels of cell density, although this data is not significant statistically.

**Conclusion:** By analysis of NSCLC was established the total quantity of tumor cells per mm2 and the main proliferative characteristics.

**PS-16-021**

**Detection of ALK gene rearrangements in non small cell lung cancers**

A. B. Oz ^*^, S. Batur, N. Comunoglu, H. Kumusoglu

^*^Istanbul University, Cerrahpasa Medical Faculty, Turkey

**Objective:** The EML4 gene is commonly associated with ALK in non-small cell lung cancers. Detection of an ALK rearrangement is critical for treatment as an ALK targeted therapy. The aim of this study was to determine the frequency of ALK rearrangement in the Turkish population.

**Method:** ALK rearrangement was identified by using FISH and the value of immunohystochemistry (5A4 clone-Novacastra, Ventana) was explored. 201 formalin fixed paraffin embedded non-small cell lung cancer samples sent to laboratory for determination of ALK rearrangement status were assessed using the Abbott LSI ALK break apart rearrangement FISH probe. Fifty tumor cells were enumerated for the presence of a break apart signal which was considered as present when at least one set of orange and green signals were 2 or more signal diameters apart or when a single orange signal without a corresponding green signal was observed in more than 15 % of the tumor cells.

**Results:** 201samples tested. 12 samples demonstrated an ALK rearrangement and 153 had no detectable alteration. All 153 tumors assessed as ALK negative by FISH showed no expression of ALK protein.

**Conclusion:** The prevalence of ALK rearrangement is 5.9 % in a Turkish population of NSCLC, consistent with the reported literature.

**PS-16-022**

**Diffuse Idiopathic Pulmonary NeuroEndocrine Cell Hyperplasia (DIPNECH) in association with a sclerosing hemangioma: A case report**

B. Yaman ^*^, D. Nart, U. Çagirici, A. Veral

^*^Ege University Medical Faculty, Pathology, Izmir, Turkey

**Objective:** Diffuse idiopathic pulmonary neuroendocrine cell hyperplasia (DIPNECH) is a neuroendocrine proliferative process associated with carcinoid tumors. This extremely rare entity is being diagnosed more frequently with the increasing productivity of advanced imaging and histopathology.

**Method:** Here we report the case of a 32 year old male who coincidently have a lesion in the chest radiography on routine screening.

**Results:** Thorax computed tomography scans revealed a well- circumscribed lesion measuring 1.8 cm in its widest diameter in the right lung. Additionally, several milimetric nodules as large as 0.6 cm were evident in the same lung. The patient underwent a video-assisted thoracoscopic wedge resection for diagnostic workup. Pathological processing of the specimen revealed a sclerosing hemangioma. Besides this main lesion, there were multiple small lesions revealed several foci less than 5 mm in diameter and also one focus with 6 mm in diameter with a different trabecular and nest-like neuroendocrine morphology highlighted by staining for the neuroendocrine markers.

**Conclusion:** Clinical diagnosis of DIPNECH is extremely challenging and often delayed because of the insidious clinical course, noncharacteristic presentations, and lack of effective noninvasive diagnostic tests. Pathologic evaluation is the gold standard in making a definitive diagnosis.

**PS-16-023**

**Pulmonary mucinous cystic tumour of borderline malignancy: A case report**

B. Yaman ^*^, A. Veral, U. Çagirici, D. Nart

^*^Ege University Medical Faculty, Pathology, Izmir, Turkey

**Objective:** Pulmonary mucinous cystic tumours of borderline malignant potential are extremely rare. These tumours have a very good prognosis and as such should be distinguished from usual type of pulmonary adenocarcinoma.

**Method:** A 63 year old male patient with cough that failed to improve with antibiotic treatment presented here.

**Results:** Bilateral smooth margined masses with homogeneous density were performed at computed tomography. Bronchoscopy revealed no evidence of an endobronchial lesion. A wedge resection was performed to right lung. Gross pathologic evaluation revealed two cystic foci with 3 cm and 1.5 cm in greatest diameters. Microscopically, the tumour comprised tall columnar mucin secreting cells with minimal- moderate cytological atypia and stratification in patches. No solid tumour areas were identified. Tumour cells expressed cytokeratin 20 (CK20) strongly and CK7 weakly. There was no expression of TTF-1. Ki-67 proliferation index was 5 %. After 5 year follow-up the patient is still alive without disease.

**Conclusion:** Pathologists should use the term borderline for a tumour of low malignant potential, rather than a tumour of no malignant potential. We need to recognize this distinct and rare entity and distinguish it from other primary mucinous lesions of the lung and metastatic mucinous tumors from other sites.

**PS-16-024**

**Non-typical EGFR mutations in NSCLC patients: Incidence and implications for molecular diagnosis and therapy**

C. Otto ^*^, A. Csanadi, P. Fisch, S. Schmid, J. Rawluk, M. Werner, G. Kayser

^*^UMC Freiburg, Institute of Pathology, Germany

**Objective:** Small molecules targeting EGFR in lung cancer are especially effective if activating mutations are present. Therapeutic approval is given is for specific deletion in exon 19 or point mutations in exon 21. But other mutations in the EGFR gene exist. We here present incidence and molecular characteristics of non-typical EGFR mutations in NSCLC.

**Method:** EGFR mutation analyses at the Institute of Pathology, University Medical Center Freiburg, between January 2008 and July 2012 were retrospectively reevaluated. Typical mutations were defined as deletions in exon 19 between aminoacids 745 and 750 as well as L858R mutation in exon 21. Other genetic aberrances of exon 18 to 21 were defined as non-typical EGFR mutations.

**Results:** 108 (10.3 %) out of 1,050 analyzed tumors showed mutations in the EGFR gene 108 (10.3 %). 17 (15.7 %) tumors were bearing non-typical mutations. Molecular modeling analyses reveal potential electrostatic changes in the ATP-binding pocket similar to typical mutations in only a subset of non-typical EGFR mutations.

**Conclusion:** Non-typical mutations represent 16 % of all EGFR-mutations and occur in 1.6 % of NSCLC patients. With respect to molecular changes, careful evaluation of EGFR mutation analyses needs to be performed with complete sequencing of the mutation hotspots.

**PS-16-025**

**Comparison of the histological growth patterns in pulmonary adenocarcinomas and their distant metastases**

P. Czapiewski ^*^, A. Gorczynski, M. Skrzypski, W. Biernat

^*^Medical University of Gdansk, Dept. of Pathology, Poland

**Objective:** In 2011 IASLC/ATS/ERS proposed a new classification of invasive pulmonary adenocarcinomas (PAC) based on their architectural pattern. However, until now, no published study has appeared on the morphological predominating pattern in the primary PAC and their respective metastasis.

**Method:** The architectural pattern of 19 metastatic lung adenocarcinomas from 14 male and 5 female patients aged 40 to 69 years was analyzed. Subsequently the grading of histological components of primary tumor and metastasis was performed according to the IASLC/ATS/ERS classification. This was followed by assessment of grade 3 content in the tumor bulk.

**Results:** Proportion of unfavorable tumor content (micropapillary/solid) decreased in metastases compared with tumor primary(49 % and 59 %, respectively; student’s *T*-test, *p* = 0.172). Predominant more favorable tumor architecture (acinar/papillary) increased in 21 % of metastases (Wilcoxon, *p* = 0.068), whereas no case with acquisition of less favorable pattern of growth in the metastasis was observed.

**Conclusion:** Our results strongly suggest that a portion of PAC metastases show shift in the morphological appearance from less favorable (micropapillary/solid) into more favorable (acinar/papillary). Further studies are required to confirm our initial results and explain its biological background and consequences.

**PS-16-028**

**Solitary fibrous tumor of the pleura: Series of cases**

A. Lovrenski ^*^, M. Panjkovic, Z. Eri, D. Tegeltija, G. Samardzija

^*^Institute for Lung Diseases, Dept. for Pathology, Sremska Kamenica, Serbia

**Objective:** Analysis of 13 cases of solitary fibrous tumor of the pleura (SFTP), especially in differentiating benign and malignant SFTP.

**Method:** Retrospective analysis of clinical datas from 13 patients with pathohistologically diagnosed SFTP in period from 2005. to 2011. year was performed.

**Results:** The average patient age was 54 years. 61.5 % of patients were female. Most common clinical manifestations were pain in the hemithorax (46.1 %), cough (38.5 %) and general infectious syndrome (23 %). The tumor was localized in 61.5 % of patients in the right hemithorax, and in 38.5 % in the left. The smallest diameter of the tumor was 2 cm, and the largest 15 cm. Five tumors (38,5 %) had a visible stalk which were tied to the pleura. 11 tumors were associated to visceral and one to parietal pleura. In only one case SFTP presented as malignant and infiltrative and in one, due to the presence of necrosis, increased cellularity and size of 13 cm, as tumor of uncertain malignant potential. Vimetin, CD34 and bcl-2 proved to be a reliable positive antibodies and CD31, EMA, desmin and panCK as reliable negative.

**Conclusion:** The line between benign and malignant SFTP is not entirely clear. The definitive diagnostis must include all of the demographic, clinical, morphological, histological and immunohistochemical data.

**PS-16-030**

**Diffuse pulmonary meningotheliomatosis diagnosed by transbronchial lung biopsy: A case report**

E. Alcaraz Mateos ^*^, R. Bernabeu Mora, M. Rodriguez Rodriguez, A. Gimenez Bascuñana, C. Hu, J. M. Sanchez Nieto, A. Chaves Benito

^*^Hospital Morales Meseguer, Dept. de Anatomia Patologica, Murcia, Spain

**Objective:** Diffuse pulmonary meningotheliomatosis is a rare entity, generally asymptomatic characterized for single or multiple lesions involving one or both lungs, defined histologically by intraparenchymatous nodular proliferation of cells of meningothelial origin. We present a case report.

**Method:** A 59-year-old woman with a past medical history of endometrial adenocarcinoma 17 years ago was referred following the observation of multiple lung nodules on an abdominopelvic computed tomography (CT) scan performed to investigate chronic diarrhea.

**Results:** A chest CT scan confirmed the presence of multiple disseminated minute nodules, distributed randomly throughout both lungs. Bronchoscopy showed normal mucosa and transbronchial biopsy demonstrate interstitial proliferation of epithelioid cells with oval monomorphic nuclei, without atypia or mitosis, arranged in irregular clusters with poorly defined borders. Immunohistochemical staining showed positivity for vimentin, EMA, progesterone receptors, and CD56, leading to a pathological diagnosis of pulmonary meningothelial-like nodule.

**Conclusion:–**Minute pulmonary meningothelial-like nodules (MPMNs) are rare lesions of uncertain origin.–They mostly occur in women, are most common in the sixth decade of life, and are generally asymptomatic.–In most cases, MLNs occur as single lesions. When they occur as multiple lesions, they tend to affect one or several lobes of the same lung. Diffuse, bilateral involvement, known as diffuse pulmonary meningotheliomatosis, is less common and can simulate metastatic disease, as occurred in our case.

**Diffuse pulmonary meningotheliomatosis–Histopathology:** 
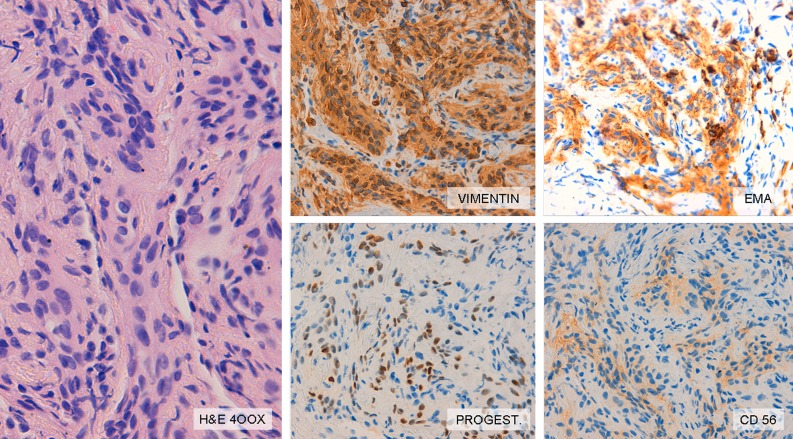


**PS-16-031**

**A rare case of biphasic pulmonary blastoma: Case report**

M. Papazian ^*^, G. Mitropoulou, E. Paliouri, O. Batsi, I. Lekka, P. Arapantoni-Dadioti

^*^Peiraias, Greece

**Objective:** We present a case of a biphasic pulmonary blastoma a rare malignant neoplasm of the lung in a 45-year-old man.

**Method:** A 45-year-old Caucasian male presented with a 1 month history of non-productive cough and low-grade fever. A computed tomography (CT) of the thorax showed a 6.5 cm solitary solid mass in the right upper lobe of the lung. Fine needle biopsy (FNB) was interpreted as malignant spindle cell neoplasm with immunohistochemical findings compatible with sarcomatoid carcinoma and a right thoracotomy was performed.

**Results:** Histologically the tumor consisted of both epithelial and mesenchymal elements. The epithelial component entailed a well differentiated fetal adenocarcinoma as well as a mucinous carcinoma and it was immunoreactive with epithelial markers. The mesenchymal element was a sarcoma with embryonic appearance and it was immunoreactive mainly for vimentin and CD99. A biphasic pulmonary blastoma was diagnosed and the patient died 8 months after surgery.

**Conclusion:** Our case is about a biphasic pulmonary blastoma, which is a rare lung tumor and a subgroup of pulmonary blastoma, characterized by an exceptional histological heterogeneity intermixing both mesenchymal and epithelial components that mimic morphologically an embryonal pulmonary entity.

**PS-16-032**

**Primary undifferentiated pleomorphic sarcoma of the lung: A case report**

A. Alves ^*^, M. Cardoso, A. Pignatelli, I. Lourenço

^*^Hospital de Santa Maria, Dept. de Anatomia Patologica, Lisbon, Portugal

**Objective:** Radiation-induced sarcoma is a very rare form of presentation that can arise following breast and thyroid cancer treatment. Undifferentiated pleomorphic sarcoma is known to follow breast radiotherapy but a primary location in the lung has not been previously described.

**Method:** We present a case of a 65-year-old woman, followed in another institution, with a personal history of breast cancer treated with chemo and radiotherapy (7 years ago), and thyroid cancer treated with radioactive iodine (6 years ago), with no histological information. On follow-up a pulmonary nodule with 2,5 cm was detected on CT scan and a wedge resection was performed. No other tumors were found on clinical investigation.

**Results:** Histologically, the tumor was composed of a highly cellular proliferation, grouped in intersecting bundles of highly pleomorphic cells, giant cells and a high mitotic rate, including atypical mitosis. Immunohistochemistry only revealed positivity for vimentin and CD68. The diagnosis given was of undifferentiated pleomorphic sarcoma, primary to the lung. There was a disease-free interval of 2 years with death following 3 years after treatment.

**Conclusion:** Undifferentiated pleomorphic sarcoma is a diagnosis of exclusion following thorough sampling and an extensive morphological and immunophenotypic study that should be considered in patients with previous radiation therapy.

**PS-16-034**

**Unusual variant of Erdheim Chester disease or new histiocytosis?**

H. Popper ^*^, J. Loeffler-Ragg

^*^Medizin. Universität Graz, Inst. für Pathologie, Mol. Lung und Pleura Pathologie, Austria

**Objective:** Erdheim Chester Disease (ECD) is a rare multisystemic histiocytosis characterized by histiocytic infiltrations. We encountered an unusual variant in a young male patient with multifocal brain infiltrations treated and responded under high-dose corticosteroids and cyclophosphamide for 10 years. Due to bone marrow insufficiency treatment was stopped, followed by relapse of the neurological disorder and newly arising diffuse lung infiltrations.

**Method:** Lung and brain biopsies were investigated by immunohistochemistry and clonality analysis to rule out lymphoma.

**Results:** Histology showed a mixed lymphocyte and histiocyte infiltration of the lung and brain nodules. Whereas the brain lesions were nodular, the lung lesions were mixed nodular and diffuse composed of macrophage-like cells and lymphocytes. Lymphoma could be excluded by immunohistochemistry and clonality analysis, the histiocytic cells were positive for CD68, CD163, but negative for CD1a and Langerin, and also negative for CD35 and CD83, as well as myeloperoxidase, but positive for CD14 ruling out dendritic cell tumors and myeloid leukemia cells.

**Conclusion:** Based on the morphologic analysis a diagnosis of ECD was rendered. However, the clinical onset with a CNS involvement and the lymphocyte-rich background infiltration is unusual, and has not been recognized in ECD. It might represent a variant of ECD or a new disease.

**PS-16-035**

**ABCA 3 mutation in a Portuguese female infant with Respiratory Distress Syndrome (RDS): A case-report**

M. Ferreira ^*^, J. Gonçalves, M. Cruz, A. Silva, M. Teixeira, F. Pardal

^*^Hospital de Braga, Serviço de Anatomia Patológica, Portugal

**Objective:** To describe the histological findings in surfactant deficiency associated with the first ABCA3 gene mutation in a Portuguese female infant. Surfactant is a mixture of lipids and proteins that plays an important role in lung function by lowering surface tension at the air-liquid interface. Gene mutations affecting SP-B, SP-C, the major surfactant proteins and ABCA3 cause surfactant deficiency and consequent RDS.

**Method:** A female infant was hospitalized with RDS that started 4 h after birth. She needed mechanical ventilation and received multiple doses of exogenous surfactant. A lung biopsy was performed because congenital alveolar proteinosis was suspected.

**Results:** Microscopically, pulmonary parenchyma had preserved and collapsed areas. The alveolar septum had fibrosis and, in the alveolar luminae, we observed hemorraghic infiltration. Alveolar macrophages number was greater than normal and small amounts of eosinophilic and amorphous material could be seen. No extensive inflammatory reaction was detected. Alveolar proteinosis was excluded but not SP-C and ABCA3 deficiency. Peripheral blood was sent for genetic analysis and revealed a previously undescribed mutation of the ABCA3 gene.

**Conclusion:** ABCA3 gene deficiency was first described in 2004. It has been identified in full-term neonates and, like our case, it has been proved to be fatal.

**PS-16-036**

**The critical look at the “optical biopsy” of the pulmonary pathology: Dispelling clinical prejudice**

A. Sorokina^*^, F. Zabozlaev, O. Danilevskaya, D. Sazonov, A. Averyanov

^*^FRCC FMBA Russia, Pathology, Moscow, Russia

**Objective:** Nowadays there are many clinical studies performed with the confocal laser endomicroscopy in lungs (“optical biopsy” of cancer, sarcoidosis and alveolar proteinosis), but all of them have been done in vivo, which is not the adequate way to investigate the diagnostic patterns of these diseases. Our aim was to study the same lung pathology ex vivo and to compare received images with histological specimens.

**Method:** 20 cases of lung cancer, 3 alveolar proteinosis cases and 5 sarcoidosis cases have been studied with confocal laser endomicroscopy ex vivo, the examined areas were marked and then the histological specimens from the marked areas were prepared. The comparison analysis has been done.

**Results:** For each kind of studied lung pathology we found the particular specific features, which were different from patterns previously found by clinicians in other in vivo research.

**Conclusion:** Our recent pre-clinical study showed the specific patterns of the lung cancer, sarcoidosis and alveolar proteinosis, which are different from previously stated by clinicians. Thus nowadays the confocal laser endomicroscopic features of the lung pathology should be investigated ex vivo for making the more accurate diagnosis.

**A–Alveolar proteinosis, h&e, ×200, B–Alveolar proteinosis, pCLE. C–Pneumocystis pneumonia, h&e, ×40, D–Pneumocystis pneumonia, pCLE. E–Pulmonary adenocarcinoma, h&e, ×40, F–Pulmonary adenocarcinoma, pCLE. G–Pulmonary squamose cell carcinoma:** 
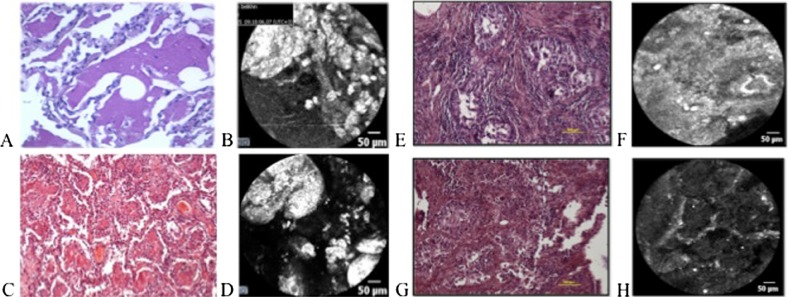


**PS-16-037**

**Endobronchial hamartoma: Case report**

A. Giannouli ^*^, V. Theodorou, I. Michalopoulou-Manoloutsiou, N. Vladika, N. Barbetakis, A. Nikolaidou

^*^Cancer Hospital Theagenion, Pathology, Thessaloniki, Greece

**Objective:** Pulmonary hamartomas are benign mesenchymal neoplasms, usually peripheral and only 10 % arising endobronchially. Patients are usually males, with a peak in the sixth decade. Typically hamartomas are asymptomatic, but endobronchial lesions tend to cause symptoms due to bronchial obstruction.

**Method:** We report a case of an endobronchial hamartoma of a 54 year-old male. The patient had dyspnea, weakness and fever for 2 months. The chest x-ray revealed a mass, sized 5,5 × 4,5 cm on the left upper lobe of the lung and the bronchoscopy showed an exophytic lesion with glistening surface on the left bronchial tree.

**Results:** The mass was surgically removed and we received the left upper lobe of the lung, with a gray-white area sized 7 × 5 cm inside which, a polypoid yellow-white, solid tumour was found. Microscopically the tumour composed of mature adipose tissue, mature cartilage and bone with hematopoietic elements in a fibrous and myxoid background. Sections from adjacent sites showed obstructive bronchiolitis and organizing pneumonia. Numerous epithelioid granulomas with central necrosis and Langhans cells were also found.

**Conclusion:** Differential diagnosis of this benign neoplasm should include monomorphic benign soft tissue tumours, chondrosarcoma, pleuropulmonary blastoma and bronchopulmonary chondromas.

**PS-16-039**

**Unexpected death of mother in labour after caesarean section**

R. Rozboril ^*^, J. Benko, M. Igaz, H. Gergisakova, P. Zon, K. Kubisova

^*^Lekárska Fakulta UK, Forensic Medicine, Bratislava, Slovakia

**Objective:** Undetected illness of mother in labour, which during the demanding situation, pregnancy, resulted in death at young age. The patient was, due to the uterus bleeding caused by placental abruption, urgently taken to hospital and immediately to birthing room where the baby was born by caesarean section. Postoperative patient period was without complications. An internal examination was conducted on the 4th. day after the caesarean section due to patients abdominal pain. Patient was consequently transferred from Obstetric -Gynaecological department to internal clinic due to collapse and suspicion of pulmonary artery embolization. The patient was transferred to anaesthesiology and intensive care department due to the right-sided heart failure symptoms, where she after several days, during morning toilet, collapses and dies.

**Method:** Histological and immunochemical methods.

**Results:** The cordial hypertrophy, dilatation of the right atrium of heart and presence plaques on the lining of the pulmonary artery was detected during the autopsy. Histological and immunochemical examination showed intimal thickening and luminal stenosis of the arteries and the presence of plexiform lesions. During the diagnostic conclusion the following immunohistochemical stainings were used namely immunohistochemical stainings for vimentin, antibody staining CD34 for endotel and immunohistochemical staining for SM–actin.

**Conclusion:** The case was, after the autopsy and following laboratory testing, diagnostically closed as right heart failure in idiopathic primary pulmonary hypertension, plexogennes pulomary arteriopatiae.

**PS-16-040**

**Primary pulmonary paraganglioma versus carcinoid tumor**

L. Serrano ^*^, J. Alvaro, A. Fernandez-Vasalo, M. A. Serrano-Muñoz, I. Ojanguren

^*^Hospital Germans Trias i Pujol, Dept. of Pathology, Badalona, Spain

**Objective:** Although paraganglioma has been described in virtually every organ, the occurrence of primary pulmonary paraganglioma (PPP) is controversial. Histological and immunohistochemical overlap between PPP and pulmonary carcinoid tumour (PCT) significantly contributes to this controversy. Prompted by the diagnosis of a recent PPP case, we decided to compare it with our PCT cases.

**Method:** The PPP case and 19 PCT cases retrieved from our archives were studied. H&E slides were reviewed and immunostains for keratins AE1/AE3, CAM 5.2 and S100 protein were performed.

**Results:** The PPP case was negative for CAM 5.2 and keratins AE1/AE3 and positive for S100 protein. Of the 19 PCT cases, all were positive for CAM 5.2, while only 9 were positive for keratins AE1/AE3. S100 protein immunoreactivity showed accompanying sustentacular-like cells in 13 of the 19 PCT cases.

**Conclusion:** The case herein described supports the occurrence of paraganglioma as a primary lung tumour. CAM 5.2 immunoreactivity, steadily positive in PCT and almost always negative in PPP, would be a helpful discriminator between the two. On the other hand, S100 protein expression in sustentacular-like cells may be shown by both tumour types.

**PS-16-041**

**Pleural myxofibrosarcoma: Case report**

I. H. Ozbudak ^*^, G. A. Gokhan Ocak, S. Akdeniz, A. Demircan, G. Ozbilim, H. S. Coskun

^*^Akdeniz University, School of Medicine, Dept. of Pathology, Antalya, Turkey

**Objective:** Myxofibrosarcoma is a common connective tissue neoplasm of malignant fibrocytes in a myxoid matrix favoring the extremities. We present a patient with myxofibrosarcoma of the pleura.

**Method:** A 58 year-old woman was admitted to hospital for evaluation of shortness of breath, chest pain and recurrent pleural effusion in the left hemithorax. The patient underwent pleural biopsy.

**Results:** The histopathological examination of the pleural biopsy revealed distinctive histologic features included nodular growth pattern, a myxoid matrix containing elongated, curvilinear capillaries, and fusiform, round or stellate tumor cells with indistinct cell margins, slightly eosinophilic cytoplasm, and hyperchromatic atypical nuclei. The lesion varied from a hypocellular, mainly myxoid, and purely spindle-cell appearance to intermediate-grade with moderately atypical tumor cells. Immunohistochemistry revealed only vimentin positivity.

**Conclusion:** To our knowledge, this is the first report of primary myxofibrosarcoma of the pleura. Sarcomatoid neoplasms of the pleura are rare tumors that present a complex differential diagnosis, making them challenging for surgical pathologists and myxofibrosarcoma should be considered in the differential diagnosis.

**PS-16-043**

**Epithelioid haemangioendothelioma of the lung: A case report**

A. Giannouli ^*^, G. Panselinas, B. Christoforidou, S. Barbanis, N. Barbetakis, F. Patakiouta

^*^Cancer Hospital Theagenion, Pathology, Thessaloniki, Greece

**Objective:** Pulmonary epithelioid haemangioendotheliomas (PEH) are rare low-to-intermediate-grade vascular tumors.

**Method:** We report a case of PEH in a 74 year-old male. The tumor was found incidentally on chest x-ray, performed for respiratory infection.

**Results:** The patient underwent right upper lobectomy. Gross examination showed a circumscribed gray-white nodule sized 2 cm. Microscopically the nodule composed of a central sclerotic hypocellular zone and a cellular peripheral zone, composed of oval cells with eosinophilic cytoplasm and sharply defined cytoplasmic vacuoles. Immunohistochemically, the neoplastic cells were positive for Vimentin, CD31, CD34, Factor VIII, focal positive for KerLMW and S-100 and negative for KerAE1/AE3, EMA, SMA, Desmin, HMB45, TTF1, CD68, chromogranin and calcitonin. Differential diagnosis includes a variety of benign conditions such as granulomatous disease, amyloid nodules and malignant neoplasms such as adenocarcinoma, angiosarcoma, chondrosarcoma and leiomyosarcoma.

**Conclusion:** We present this rare case of lung tumor in order to emphasize the importance of antibody panels to distinguish these vascular tumors from carcinoma. Additionally, the possibility of lung metastasis should be excluded, since EH can also arise in the liver, soft tissue and bone.

**PS-16-044**

**P16 and p53 protein expression in malignant mesothelioma and mesothelial proliferative processes**

M. Valladares ^*^, D. R. Luján, R. Ávila, A. Santos, L. Gómez

^*^Hospital Virgen del Rocío, Anatomy Pathology, Sevilla, Spain

**Objective:** Pleural endoscopic biopsy is a challenge in relation to defferential diagnosis between reactive epithelial proliferation and malignant mesothelioma (MM). Expression of immunohistochemical markers such as EMA, GLUT-1 or p53 are associated with MM as well as with homozygous deletion of p16 gene, but none of these are specific for MM. However, genetics technics are not available in all hospitals. We aim to investigate whether the combination of p53 and p16 immunostaining could be useful for the differential diagnosis of MM.

**Method:** 34 endoscopic biopsies from MM were subjected to immunohistochemical technics and genetics technics for p16 deletion (FISH) in some cases, and were subsequently diagnosed. 16 cell blocks were made to perform p16 and p53 immunostaining.

**Results:** Malignant epithelial mesothelioma: 28 (82.3 %). 24 showed expression of p16 (85.7 %) and 28 showed p53 protein (100 %). Malignant no epithelial mesothelioma (bifasic and sarcomatoid): 6 (17.6 %). 4 showed expression of p16 (66.6 %) and 6 p53 protein (100 %). Reactive mesothelium: p53 protein wasn’t found and 10 showed expression of p16 protein (62.5 %).

**Conclusion:** The combined use of p16 and p53 immunostaning could be helpful in malignant mesothelioma differential diagnosis on endoscopic pleural biopsy.

**PS-16-045**

**Overexpression of IL-17 pathway in allograft rejection of rat orthotopic lung transplantation**

F. Calabrese ^*^, N. Nannini, F. Lunardi, M. Vadori, E. Cozzi, F. Rea

^*^University of Padoava, Italy

**Objective:** The outcome of lung transplantation depends mainly on both acute and overall chronic rejection (obliterative bronchiolitis, OB). IL-17 pathway seems to have a role in the pathogenesis of alloimmune-induced autoimmunity. However, there is no evidence of specific cellular immunolocalization in lung allograft rejection. We aimed to investigate this pathway expression across specific cellular types.

**Method:** Lungs from Lewis rats were transplanted into Fisher rats (33 cases). Histology and tissue IL-17 pathway (IL-17/IL-23R) expression were assessed in the grafts distinguishing different cell types.

**Results:** Acute rejection and OB, developed by day 30, were detected in 57 % and 14 % respectively. At day 90, all lungs showed severe allograft dysfunction with evidence of OB in 53 % of cases. IL-17 expression was only found in rats that developed acute rejection and OB. IL-17 macrophagic immunoreactivity was significantly increased in grafts with higher grade of acute rejection. Both epithelial and endothelial IL-17 pathway expression was higher in OB cases.

**Conclusion:** Our study demonstrated that IL-17 pathway is crucial in lung rejection. Airway and endothelial other than inflammatory cells play a key role in this process.

**PS-16-046**

**Quantitative characterization of alveolar epithelial type II cells in acute pneumonias**

R. Zibirov^*^, D. Kozlov

^*^Smolensk Institute of Pathology, Russia

**Objective:** Comparative quantitative characterization of alveolar epithelial type II cells in acute bacterial pneumonias and investigations without pneumonia.

**Method:** We examined 60 observations of pneumonia with bacteriological and histological examination. Histological examination included counting the number of alveolar epithelial type II cells in ten different visual fields (*n* = 600) in focus of pneumonia, uninvolved perifocal zone (up to 0.5 cm) in the first group investigation and number of same cells in second group (28 observations, *n* = 280) investigation without pneumonia. After hematoxylin and eosin staining we counted type II cells around capillaries at magnification of the microscope to 1,000×. The data is presented as means +/− SD.

**Results:** Number of type II cells in focus of pneumonia were 2,49 +/− 1,93; number of type II cells in uninvolved perifocal zone were 5,50 +/− 3,32; number of type II cells in control group of investigation without pneumonia were 5,91 +/− 2,83.

**Conclusion:** Increasing number of alveolar epithelial type II cells with increasing distance from the source of inflammation and the maximum number of type II cells in group without pneumonia shows that inflammation slows down the process of epithelial regeneration.

**PS-16-048**

**Effects of flavonoids synthesis derivative on a rat ovalbimine sensitization model of asthma**

C. L. Zamfir ^*^, C. Lupusoru, F. Baderca, E. Stefanachi, R. Folescu

^*^University of Medicine, Dept. of Histology, Iasi, Romania

**Objective:** This study intends to reveal the possible flavonoids sinthesis derivatives involvement in modulation of hiperresponsiveness of the respiratory airways in asthma.

**Method:** Samples were prelevated from the three groups of Wistar rats: control group versus group treated with intraperitoneally injected ovalbumin (10 mcg/o,5 ml) day 1, day 14 versus group treated with the same ovalbumin sequence, associated with F1 flavonoid derivative,170 mg/kgbody weight/day, intraperitoneally administered,for 30 days. The last two groups were exposed to inhaled ovalbumine 10 mg/ml, for 20 min each day, on days 28, 29, 30. Samples of pulmonary tissues(specifically treated for micrpscopic exam-paraffin embedded, sectioned and HE stained) and bronchoalveolar lavage(BAL) fluid were prelevated from each group

**Results:** Pulmonary specific inflammation and vascular congestion together with high density of infamatory cells in BAL fluid observed in the second group were significantly diminished in the third group

**Conclusion:** We hypothesize that F1 flavonoid derivative exhibits anti inflammatory effects, suggesting a possible therapeutic potential in asthma treatmnet.

**PS-16-049**

**The 5 gestational week as the onset time of pulmonary sequestration: A case report**

Y. Kimula ^*^

^*^Ifuki Clinic, Nagasaki, Japan

**Objective:** A 38-year-old woman visited a clinic because of the general malaise. The unusual shadow of the lower right lung field was found out by the chest XP.

**Method:** Since that the aberrant artery from a thoracic aorta to a shadow was clarified by CT, the patient was diagnosed with intralobar pulmonary sequestration and underwent surgery. The anomalous artery was divided, and the sequestered segment was completely resected by video-assisted thoracic surgery.

**Results:** It was diagnosed as the pulmonary sequestration of Pryce type 2. Her mother had fallen from a motorbike when she was 5 gestational week.

**Conclusion:** This case suggests that pulmonary sequestration occurs in the early stage of lung generating. After the operation her symptoms disappeared and the complications of the operation do not appear for 5 years. Reference: Pryce DM, Lower accessory pulmonary artery with intralobar sequestration of lung; a rep ort of seven cases. Pathol Bacteriol. Jul;58(3):457–67, 1946.

**PS-16-050**

**Adaptive immune response in COPD patients with and without a1 antitrypsin deficiency**

F. Calabrese ^*^, N. Nannini, F. Lunardi, S. Baraldo, E. Bazzan, E. Balestro, M. Schiavon, F. Rea, M. Saetta

^*^University of Padoava, Italy

**Objective:** COPD is characterized by variable degree of lung damage in response to cigarette smoke. An elastase/antielastase imbalance, along with innate immunity, is believed to account for lung destruction in α1-antitrypsin deficiency (AATD). We aimed to study adaptive immunity in COPD patients with AATD in comparison to COPD patients with normal AAT-levels.

**Method:** Immunohistochemistry for B and T-cells, neutrophils and macrophages were quantified in lung tissue of COPD patients with (10) and without (22) AATD undergoing lung transplantation, as well as smoker (16)and non-smoker (8) controls.

**Results:** The two groups of patients with COPD, with and without AATD, had similar numbers of B-lymphocytes [median (range): 1.9 (0–4.4) and 1.1 (0–5)cells/mm], CD8 [3.4 (0.6–6.8) and 4.1 (3.1–6.8)] and CD4 lymphocytes[5.5(1–10.8) and 6.0 (1.6–11.9)] that were higher when compared to the controls(*p* < 0.05). Lymphocytes in alveolar walls, particularly B-lymphocytes, correlated with lymphoid follicle number (*r* = 0.59 *p* < 0.0001) and with FEV1 (*r* = −0.49 *p* < 0.001).

**Conclusion:** Immune response in COPD with AATD is similar to that found in COPD without AATD, suggesting that the adaptive immune response is an important pathogenetic factor in both.

**PS-16-051**

**Diffuse parenchymal lung osteodystrophy–Idiopathic Interstitial Lung Disease (ILD) with Bronchiolar Alveolar Transition Zone (BAPZ) involvement**

E. Kogan ^*^, S. Demura, V. Paukov, D. Fligil, U. Ziuzya

^*^RCOGP, Dept. of Pathology, Moscow, Russia

**Objective:** Case report of the diffuse parenchymal lung osteodystrophy (DPLO). DPLO is a rare lung pathology with unknown etiology and unclear pathogenesis.

**Method:** 37 year old patient with bilateral DPLO lesions of the lower lobes suffered from COPD. He had transitor Micobacteria Fortunata infection. Open lung biopsy with histological and immunohistochemical (monoclonal antibodies to SMA, Desmin, Vimentin, Oct4, CD34, Pan CK) examinations were performed.

**Results:** We found the formation of the bone foci with bone marrow tissue in the BAPZ and interstitium involvement with mesenchimal cells positive for SMA, Vimentin, Oct4, CD34. Background lung pathology–hemosiderosis and COPD–was proved.

**Conclusion:** DPOL may be considered as idiopathic ILD, that development is associated with the BAZP damage and is characterized by chronic inflammation and impaired differentiation of mesenchymal stem cells during repair probably of the bone marrow origin. BAZP damage in ILD, where stem cell differentiation is disturbed, may lead to fibrosis and in some cases to the bone marrow tissue formation.

Tuesday, 3 September 2013, 09.30–10.30, Pavilion 2

**PS-17 Poster Session Breast Pathology**

**PS-17-001**

**Solid neuroendocrine carcinoma of the breast: A rare tumor**

A. Tsavari ^*^, E. Moustou, E. Arkoumani, D. Myoteri, K. Koulia, A. Zizi-Sermpetzoglou

^*^General Hospital Tzaneio, Dept. of Pathology, Piraeus, Greece

**Objective:** Solid NeuroEndocrine Carcinoma of the Breast (SNECB) is a subtype of primary neuroendocrine carcinoma (NEC) of the breast with several distinctive features.

**Method:** A 84-year old woman was admitted in the hospital with a lump in her right breast. Mammography revealed a well-defined nodule in the upper quadrant of her right breast. She underwent lumpectomy and sentinel lymph node biopsy, which showed no metastasis.

**Results:** The histological diagnosis was solid neuroendocrine carcinoma of the breast. Microscopically, the tumor cells are arranged in nests or trabeculae and separated by scant connective tissue. Immunohistochemical staining demonstrates strong positivity for NSE, Chromogranin, Synaptophysin, ER and PR.

**Conclusion:** NEC of the breast is rare, accounting for less than 2 % of all breast cancer and less than 1 % of all neuroendocrine tumors. According to the WHO SNECB is one type of NEC, the other types are: small/oat cell carcinoma and large NEC. SNEB is considered to be a well-differentiated tumor and has an overall good prognosis. Because of the rarity of this disease there is no standard treatment protocol and a large variety of chemotherapy protocols have been employed in treating this disease.

**PS-17-003**

**Distribution of CD10 and other myoepithelial/basal cell markers in sub areolar breast ducts**

S. Shousha ^*^, D. Saeed

^*^Charing Cross Hospital, Dept. of Histopathology, London, United Kingdom

**Objective:** We have previously noted that the most superficial parts of mammary ducts are devoid of CD10 positive myoepithelial cells and that in situ lobular neoplasia (ILN) does not appear in these areas, suggesting a link. We aim to confirm the absence of CD10 myoepithelial cells in the superficial parts of mammary ducts and asses if other immuno markers show a similar distribution

**Method:** Thirty consecutive cases of mastectomy nipple specimens were studied. New sections were cut and stained for EGFR, SMA, p63 and CD10.

**Results:** The ducts displayed a clear pattern of CD10 positive myoepithelial cells becoming less prevalent then absent as the ducts become more superficial. EGFR, SMA and p63 were positive in most cases with no consistent pattern. The myoepithelial cells surrounding the only case with ductal carcinoma in situ underlying Paget’s disease of the nipple was negative for CD10, p63 and SMA but positive for EGFR.

**Conclusion:** Our findings confirm the absence of CD10 positive myoepithelial cells from the most superficial parts of mammary ducts. If ILN is linked to the presence of CD10 positive myoepithelial cells, this may explain the rarity of ILN in association with Paget’s disease and in the superficial part of the breast.

**PS-17-004**

**Utility of cytokeratin immunostaining in the diagnosis of occult metastasis in Sentinel Lymph Node Biopsies (SLNB) from patients with breast cancer: A 2-year audit study**

R. Ramakrishnan ^*^, S. Parekh, S. Shousha

^*^Charing Cross Hospital, Dept. of Cellular Pathology, London, United Kingdom

**Objective:** The aim was to identify number of positive SLNB done for breast cancer patients at our centre & number of morphologically negative SLN detected as positive after cytokeratin immunostaining (CK-IHC)

**Method:** Breast cancer cases with SLNB were retreived. Information pertaining to number of SLNs evaluated, positive nodes, type and size of metastasis and CK-IHC results were noted. The number of non-SNLBs & positive non-SLNB were also recorded.

**Results:** 223 cases had SLN biopsies, 62/223 cases(28 %) showed 1 positive node atleast. 161/223 cases were negative for metastasis on H&E. CK-IHC performed on 145 H&E negative cases showed 5 additional cases with tumour; 1 micrometastasis, 3 ITCs & 1 macrometastasis. Review of H&E in the latter confirmed tumour, missed on H&E. All patients with macrometastasis except 2 had further axillary surgery. None of the patients with ITC/micrometastases had axillary surgery.

**Conclusion:** The detection rate of occult metastasis by CK-IHC in H&E negative node is very low, (3 %); 4/5 ITCs and micrometastasis & 1/5 macrometastasis. None of the patients with micrometastases had axillary surgery. Chemotherapy was considered for patients with micrometastasis. 71 % cases with a positive non-SLNB had atleast 1 positive SLN whereas 29 % cases with positive non-SLNB showed no metastasis in SLN.

**PS-17-005**

**Toker cells of the nipple are commonly associated with underlying sebaceous glands**

D. Saeed ^*^, S. Shousha

^*^London, United Kingdom

**Objective:** To study the incidence and distribution of Toker cells in a consecutive series of nipple specimens.

**Method:** Forty five consecutive cases of mastectomy nipple specimens were retrieved from our archive. New sections were cut and stained for cytokeratin 7 (CK7), a Toker cell marker.

**Results:** Five out of the 45 (10 %) cases were positive for CK7. Surprisingly, it was noted that in all five cases, the Toker cells were most concentrated in the regions overlying the sebaceous glands. We then examined three random cases, one of which was positive for Toker cells and showed a similar distribution. However, sebaceous glands with no associated Toker cells were commonly seen. The basal layer of sebaceous glands was commonly CK7 positive whether or not Toker cells were present. Lactiferous ducts were not a consistent feature in association with Toker cells.

**Conclusion:** Our findings strongly suggest that Toker cells of the nipple are commonly associated with underlying sebaceous glands. This raises interesting questions regarding the origin of Toker cells.

**Toker cells overlying sebaceous gland:** 
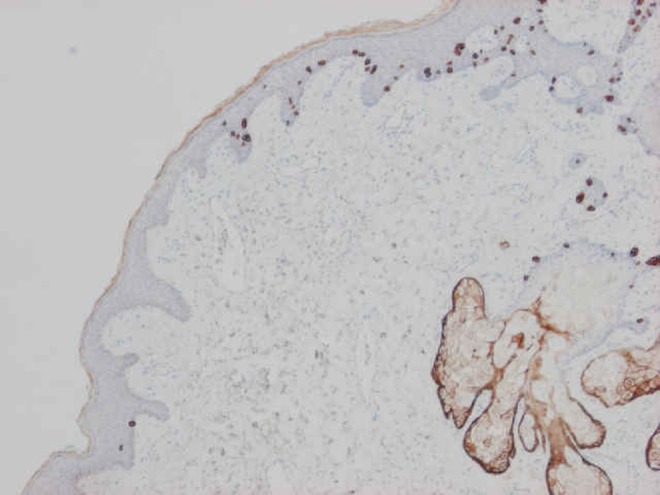


**PS-17-006**

**Estrogen Receptor Negative (ERN) solid & intracystic Papillary Carcinoma (PC) of the breast: A clinicopathological and immunohistochemical study**

R. Ramakrishnan ^*^, S. Shousha

^*^Charing Cross Hospital, Dept. of Cellular Pathology, London, United Kingdom

**Objective:** Solid (SPC) & intracystic (IPC) papillary carcinoma are circumscribed, low grade tumours with a good prognosis and usually oestrogen receptor positive (ERP). We report 7 oestrogen receptor negative (ERN) PCs of a series of 27 consecutive PCs reported at our hospital, with details of the nomenclature, clinical & immunophenotypical features and follow up (FU). To our knowledge, this is the first report on ER negative PCs.

**Method:** 27 breast PCs (locally treated and referral) were identified from the database. Pertinent demographic, clinical and follow up information was obtained. H&E slides were reviewed. ER, PR, Her2 and CK5-IHC staining was evaluated. 7 cases were ERN-PCs & 20 were ERP-PCs.

**Results:** Median age of patients was 68 years. Median size was 19 mm & 16 mm respectively in ERP & ERN groups. Of ERP-PCs, 6 were intracystic & 14 solid/cribriform types. Of ERN-PCs, 3 were intracystic & 4 solid/cribriform types. 19/20 ERP cases were Her2-. 6/7 ERN cases were Her2-. 11/20 ERP & 6/7 ERN cases showed foci of invasive disease. 2/4 ERN-PCs had distant metastasis. 3/4 patients received chemotherapy. 1/4 patient died due to co-morbidities.

**Conclusion:** ERN-PCs may be a distinct subgroup with specific clinico-pathological features, aggressive behaviour and poor prognosis with increased risk of metastasis.

**PS-17-007**

**Rat mammary cancer chemically-induced as a model to study cancer cachexia**

H. Vala ^*^, C. I. Teixeira-Guedes, A. I. Faustino-Rocha, A. Andrade, D. Talhada, M. J. Pires, P. A. Oliveira, R. Ferreira

^*^Escola Superior Agrária de Viseu, Instituto Politécnico de Viseu, Portugal

**Objective:** Cachexia is characterized by a severe weight loss, muscle wasting and anemia. It has been associated with cancer, being responsible by up to 20 % of all cancer deaths. The main goal of this study was test if mammary cancer model induced by N-methyl-N-nitrosourea (MNU) is adequate in the study of cachexia.

**Method:** In this sense 25 female Sprague–Dawley female rats were randomly divided into two groups: control (*n* = 10) and MNU (*n* = 15). All procedures were performed in accordance to European Directive 2010/63/EU. At 50 days-old animals from MNU group received a single dose of MNU (50 mg/Kg). After 8 months animals were sacrificed. Body and organs’ weights were measured and blood samples were collected to evaluate hematological and biochemical parameters.

**Results:** We observed that animals from MNU group exhibited a lower body and gastrocnemius weights and a higher spleen and liver weights. Concerning to blood analysis, MNU group evidenced a lower hematocrit, total protein, cholesterol, low density lipoprotein, triglycerides and glucose, and a higher levels of creatine kinase. Immunoblotting also showed higher activity of interleukin-6, C-reactive protein and myostatin.

**Conclusion:** The observed alterations in hematocrit and biochemical parameters were compatible with cachexia. These results are similar to those described in human cachexia associated with cancer.

**PS-17-008**

**miR-125b and miR-145 are downregulated in MUC1 positive invasive breast ductal carcinomas**

A. Ribeiro-Silva ^*^, J. Zanetti, P. Darin, F. d. Lucca

^*^Ribeirão Preto Medical School, Dept. of Pathology, Brazil

**Objective:** Mucin 1 (MUC1) may act as an oncogene with an important role during breast cancer development. Recently, our research group showed that MUC1 is overexpressed in a subset of invasive breast ductal carcinoma (IDC) and correlated with important clinicopathological features. In cell cultures, some miRNAs, such as miR-125b and miR-145, have the gene MUC1 as one of their targets. In this work, we investigated the expression of miR-125b and miR-145 in invasive ductal carcinomas that overexpress MUC1.

**Method:** Using RT-PCR, the expression of miR-125b and miR-145 was assessed in 10 formalin-fixed paraffin-embedded (FFPE) samples from invasive ductal carcinomas that overexpressed MUC1, and in 5 normal breast samples, used as controls.

**Results:** RT-PCR assay showed a decreased expression of miR-125b and miR-145 in all samples of ductal carcinomas in which MUC1 was overexpressed, as well as and an increased expression in all controls (normal breast samples).

**Conclusion:** The demonstration that these miRNAs are associated with MUC1 expression may contribute for a better understanding of the molecular mechanisms involved in the development of MUC1 positive invasive breast ductal carcinomas.

**PS-17-009**

**Bilateral Ewing sarcoma/primitive neuroectodermal tumor of the breast: A very rare entity and review of literature**

M. Amrani ^*^, A. Haddan, N. Majid, S. Mesmoudi, A. Moumni, I. El-Ghissassi, H. Hachi

^*^Faculté de Médecine, Dept. de Pathologie, Rabat, Morocco

**Objective:** The aim of this work is to demonstrate that although rare, the possibility of PNET should be kept in mind while evaluating a palpable breast abnormality in a young female.

**Method:** A 30-year-old woman presented with painless and progressively growing lumps in the right than in the left breast for 10 months duration. Examination revealed firm, fixed, painless and palpable retromammary bilateral masses measuring 7 and 4 cm in the right and left breast respectively associated with skin retraction and bilateral axillary lymph node metastases. Mammography and ultrasonography identified suspicious multiple bilateral masses. The pathology report showed a proliferation of small, round to oval cells having unconspicuous nucleoli and scanty cytoplasm with thickened nuclear membrane. Tumor cells were strongly positive for vimentin and CD99, but were negative for AE1/AE3 and CD56. A staging work-up revealed a superior mediastinal mass extending to the para-aortic area with metastases in the right lung and a pleural effusion;

**Results:** Based on these findings metastatic ES/PNET was the final diagnosis but the patient died after 2 cycles of chemotherapy.

**Conclusion:** Prognosis of metastatic disease is generally poor and it doesn’t seem to make a difference whether the ES/PNET is primary or metastatic to the breast. Histopathological confirmation is mandatory especially in cases of unusual locations.

**PS-17-010**

**Preliminary results of Mouse Mammary Tumor Virus (MMTV) detection in 35 cases of breast cancer in Moroccan women**

M. Amrani ^*^, M. Slaoui, M. Attaleb, A. A. Zouré, H. Arias-Pulido, M. El-Mzibri

^*^Faculté de Médecine, Dept. de Pathologie, Rabat, Morocco

**Objective:** To find out if MMTV virus is present in moroccan breast cancer specimen in the aim to contribute in the prevention and personnalized care of breast cancer in Morocco.

**Method:** 29 Inflammatory Breast Cancer (IBC) cases and 6 Non Inflammatory Breast Cancer (NIBC) cases were included in this preliminary retrospective study. Normal tissue for comparison was available in 8 IBC and 2 NIBC cases and were at least 2 cm away from the tumoral tissue. All corresponding Hematoxyline and Eosine slides were reviewed to make sure that the tumoral and normal tissues were represented at a percentage higher than 30 % of the cut surface. After deparaffination and DNA extraction, DNA amplification was done using the thermocycler GeneAmpR PCR System9700 (AB/Applied Biosystems, Foster City, CA) and beta-globine gene was amplified for the quality test. MMTV DNA was detected by PCR using MMTV65R/MMTV489Fsa amorces which amplify a 171 bp MMTV fragment at the Env (envelop) gene. Positive and negative controles were used for each PCR.

**Results:** MMTV was detected in 23/35 cases (67.7 % of the cases): 20/29 IBC and 3/6 NIBC.

**Conclusion:** We intend to work on a larger specimen size to find out if there is any statistically significant correlation with breast cancer prognostic factors.

**PS-17-011**

**Association between BMI-1 and homologous recombination markers in human breast cancer**

A. Ribeiro-Silva ^*^, G. Silveira, M. R. Celes, C. Camillo, F. Soares

^*^Ribeirão Preto Medical School, Dept. of Pathology, Brazil

**Objective:** B-cell-specific Moloney murine leukemia virus integration site 1 (Bmi-1) is a Polycomb group protein that is able to induce telomerase activity, enabling the immortalization of epithelial cells. Immortalized cells are more susceptible to double-strand breaks (DSB), which are subsequently repaired by homologous recombination (HR). BRCA1 is among the HR regulatory genes involved in the response to DNA damage associated with the RAD51 protein, which accumulates in DNA damage foci after signaling H2AX, another important marker of DNA damage. Topoisomerase IIIB (topoIIIB) removes HR intermediates before chromosomal segregation, preventing damage to cellular DNA structure. Our objective is to evaluate the relationship between BMI-1 and these regulatory proteins of homologous recombination in human breast carcinomas.

**Method:** We analyzed protein expression by immunohistochemistry in 239 cases of primary breast tumors. For gene expression, we performed Real-Time PCR reactions in MCF-7 cell line.

**Results:** BMI-1 immunohistochemistry overexpression was related to p53 (*p* = 0,003), BRCA-1 (*p* = 0,003), H2AX (*p* = 0,024) and TopIIIB (*p* < 0,001). Real-Time PCR assay showed that BMI positive cells have high expression of H2AX and P53.

**Conclusion:** Our results point to a relationship between BMI-1 and homologous recombination markers, suggesting that the presence of BMI-1 is an important event in breast cancer homologous recombination.

**PS-17-012**

**PIK3CA and EGFR mutation in triple negative breast carcinoma: Comparison with hormone receptors positive and HER2 positive breast carcinomas**

H. E. Pestereli ^*^, M. Ozcan, G. Erdogan, F. S. Karaveli

^*^Akdeniz University, School of Medicine, Dept. of Pathology, Antalya, Turkey

**Objective:** We aim to investigate PIK3CA and EGFR mutation in triple negative breast carcinomas and compare the results with hormone receptor positive and HER2 overexpressed breast carcinomas.

**Method:** Genomic DNA was isolated from formaline fixed parafin embedded tumor samples. 38 with Triple negative breast cancer (TNBC), 10 patients with hormone receptors positive, HER2-negative tumors, 10 patients with hormone receptors positive, HER2-positive tumors, 10 patients with hormone receptors negative, HER2-positive tumors. EGFR gene (Exon 18, 19, 20, 21) and PIK3CA gene (Codon 540–546 and Codon 1,042–1,049) were analyzed by pyrosequencing method (Qiagen).

**Results:** No mutations in Exon 18, 19, 20 and 21 of EGFR gene were detected in all groups. PIK3CA mutations were detected in 9 of TNBC (2: E542K, 1: E54K, 5: H1042R and 1: M1043I mutation), 3 of hormone receptors positive, HER2-positive tumors (1: E545K, 2: H1047R mutation), 5 of hormone receptors positive, HER2-negative tumors (2: E545K, 1: H1047L, 1: H1047R and 1 had both E542K and M1043I mutations). In HER2-positive tumors, no mutations were detected in PIK3CA gene.

**Conclusion:** PIK3 CA mutation is mostly occured in hormone positive breast cancers; only 23.7 % of triple negative tumors have PIK3CA mutation and as no EGFR mutation has benn detected; EGFR amplification seen in TNBC might occur by other mechanisms.

**PS-17-013**

**Clinicopathological features and prognosis of pregnancy associated breast cancer: A matched case control study**

L. Madaras ^*^, K. A. Kovács, A. M. Szász, A.-M. Tökés, B. Székely, Z. Baranyák, O. Kiss, I. Kenessey, M. Dank, J. Kulka

^*^Semmelweis University, 2nd Dept. of Pathology, Budapest, Hungary

**Objective:** Pregnancy Associated Breast Cancer (PABC) manifests during pregnancy or within a year following delivery. We sought to investigate differences in management, outcome, histopathology and immunohistochemistry (IHC) characteristics of PABC and control cases.

**Method:** PABC and matched control patients were selected from breast cancer cases of women ≤45 years, diagnosed in our institution between 1998 and 2012. Histopathology of invasive and associated in situ lesions, ER, PgR, HER2, Ki67, p53 expression data, IHC-based -subtype, clinical, management and outcome information were analysed.

**Results:** Thirty-one women had PABC. Clinical, management data, histopathology of disease at presentation was not significantly different, but Nottingham Prognostic Index (NPI) assessed the PABC group as of poor prognosis, while controls as of intermediate prognosis. The associated in situ lesion was mainly high grade Extensive Intraductal Carcinoma component (EIC) in PABC. Triple negative and LumBprol tumors predominated in PABC. Post-partum patients’ both disease-free and overall survival, while pregnant patients’ disease-free survival was inferior to that of controls. PABC patients with LumBprol and Triple negative tumors had worse prognosis. On multivariate analysis inferior prognosis of PABC was associated with pregnancy.

**Conclusion:** Our study has demonstrated difference in tumor biology and inferior outcome of PABC compared to controls.

**PS-17-015**

**Immunohistochemical expression of some markers of invasivity in breast cancer**

L. Danihel ^*^, V. Sisovsky, Z. Cierna, M. Palkovic, I. Fridrichova, V. Repiska

^*^Comenius University, Faculty of Medcine, Dept. of Pathology, Bratislava, Slovakia

**Objective:** Breast carcinoma (BCa) is the most common neoplasia in women that is caused by progressive accumulation of genetic and epigenetic abnormalities. During the tumorigenesis many tumour suppressor genes are inactivated with following decreasing of relevant protein expression and function. This study is focused on immunohistochemical evaluation of proteins in normal and tumor breast tissues that are responsible for self-sufficiency in growth signals and inhibition of cell invasion and metastases forming.

**Method:** The total of 5 normal breast tissue samples and 20 biopsy specimens with metastatic duct and lobular BCa were evaluated by light microscope semiquantitatively for SOCS1, CDH1, TIMP3 and ADAM23. These proteins are responsible for invasivity and metastasis regulation of tumor cells.

**Results:** In normal breast tissue we found intensive expression of these proteins. In invasive breast carcinoma we found a decreased expression of evaluated markers.

**Conclusion:** Next study will investigate the role of epigenetic inactivation of relevant genes in correlation with protein expression changes in BCa. Causal DNA methylation profiles in promoters of evaluated genes could be utilized for identification of new biomarker for invasivity and metastatic potential of breast cancers. This study was supported by the grant APVV-0076-10.

**PS-17-016**

**Rectal metastases originating from occult lobular breast carcinoma**

V. Filipovski ^*^, K. Kubelka-Sabit, D. Jasar, B. Dukova

^*^Acibadem-Sistina, Dept. of Pathology, Skopje, Macedonia

**Objective:** We present a case of a patient complaining of hemorrhoid bleeding that underwent colonoscopy. The clinician took biopsies from a lesions in the mucosa of the rectum under a clinical suspicion of amyloidal deposits.

**Method:** The biopsies were routinely formalin fixed, paraffin embedded and stained with hematoxyllin eosin. Additional immunohistochemical analysis was performed using the following antibodies: Cytokeratin 7, Cytokeratin 20, E-cadherin, Gross cystic disease fluid protein 15, Estrogen receptor.

**Results:** Neoplastic cells were observed in the mucosa infiltrating in a discohesive manner sparing the epithelial lining. Typically some of the cells showed a signet ring appearance. The initial assumption was that it was a case of metastatic diffuse gastric carcinoma. The clinician found out that the patient had similar findings 5 years ago in the stomach and there was a suspicion of primary metastatic breast cancer but a primary lesion was never found. However the patient received antiestrogen therapy and the deposits receded. The tumor cells were positive for CK7, ER and GCDFP-15. Additional imaging techniques failed to present a primary lesion.

**Conclusion:** Minute metastatic deposits with discohesive growth in the gastrointestinal tract should exclude a primary breast lobular carcinoma since primary breast cancer can easily be overlooked.

**Estrogen receptor positivity of tumor cells in rectal mucosa:** 
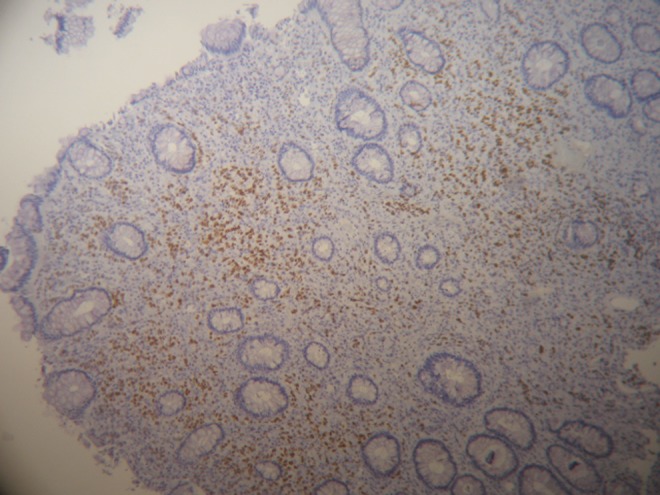


**PS-17-017**

**Paget disease of the nipple: Co-expression of E- and P-cadherin**

M. Lacerda ^*^, A. S. Ribeiro, J. Paredes, F. Schmitt

^*^Coimbra, Portugal

**Objective:** Paget disease of the nipple (PD): a model to study the co- expression of E- and P-cadherin as a putative mechanism of migration of the malignant cells to the epidermis.

**Method:** 45 cases of PD and DCIS and 26 biopsies of PD. P- and E-cadherins immunostaining using the streptavidin-biotin-peroxidase method. The P- and E-cadherin results were based on a semi quantitative evaluation. Paget cells (PC) and DCIS cells with membranous staining in at least 10 % of the neoplastic cells were scored as positive.

**Results:** Our series show in all the cases, in the epidermis, singly or closely packed clusters of PC. P and E cadherins were co-expressed in PC in all the cases except 2. PC closely packed: P-cadherin membrane expression is weaker among the neoplastic cells than with the surrounding cells; E-cadherin expression was homogenous. E-cadherin is expressed both in DCIS and in the PC. P-cadherin was expressed in DCIS and PC in all the cases except 2

**Conclusion:** Motility is the most common accept pathogenic mechanism in PC. Our findings reinforce the recently described role of P-cadherin in the impairment of E-cadherin function (J Pathol 229: 705–18, 2013).

**PS-17-018**

**Blood vessels inside lymphatic vessels in the human breast: An unrecognized morphological and functionally significant structure or an artificial phenomenon?**

S. Popovska ^*^, I. Ivanov

^*^University Hospital Pleven, Head of Pathology, Bulgaria

**Objective:** Arteries situated in the lumen of veins are known to exist and can be found in uterine tissue and rarely in breast tissues. Recently, we reported the presence of arteries inside lymphatic vessels. The aim of the present work is to present the morphological appearance and main features of a vascular complex composed of blood and lymphatic vessels that can be found in breast.

**Method:** Ten benign biopsy tissue specimens from breast (five from male and five from female patients) were analyzed. Immunostaining with D2-40 and anti-CD34 was performed. A partial three-dimensional reconstruction of an arterial-lympho-vascular complex was made.

**Results:** The presence of arteries situated inside lymphatic vessels was seen in all studied cases (sometimes after revising several tissue samples per patient). The arterio-lympo-vascular complex described is not an artificial phenomenon since it can be followed on series of tissue sections.

**Conclusion:** Arteries situated inside lymphatic vessels are an existing morphological structure, which can be found in breast tissues from male and female individuals. Their functional role is still unknown, but we assume that it may support lymph propulsion inside the initial collecting lymphatic vessels with an incomplete smooth-muscle layer.

**PS-17-019**

**Additional immunohistochemical features in triple negative breast cancer**

V. Barresi ^*^, A. Ieni, R. Cardia, G. R. Ricciardi, V. Adamo, G. Tuccari

^*^Policlinico G. Martino Pad D, Dip. Patologia Umana, Messina, Italy

**Objective:** To evaluate the immunohistochemical expression of androgen receptors (AR), E-cadherin (E-CAD) and Ki-67 in 45 formalin-fixed paraffin-embedded samples of triple negative breast cancer (TNBC).

**Method:** AR positivity was defined as >10 % positive neoplastic cells with nuclear staining, while E-CAD was semi-quantitatively analyzed as the percentage of cells showing membrane positivity. Ki-67 was scored by counting the positively stained nuclei per 1,000 malignant cells. Correlations between immunohistochemistry and clinico-pathological characteristics of TNBC or overall survival of the patients were investigated.

**Results:** Histological types were ductal (35; 77.7 %), lobular (7; 15.5 %), medullary (3; 6.6 %). 29 cases (64.4 %) showed a G3 tumor. AR were positive in 12/45 tumours (26.6 %). E-CAD was negative in 24/45 (53.3 %) cases. The Ki-67 index was ≥ 25 % in 17/45 (37.7 %).

**Conclusion:** The absence of AR expression and high Ki-67 index display a significant correlation with the ductal histotype and high histological grade (G3) (*p* < 0.001) in TNBC. Moreover, the patients with negative AR and E-CAD and high Ki-67 expression showed significantly (*p* < 0.001) worse overall survival than those with positive AR and E-CAD and low Ki-67 expression.

**PS-17-020**

**Nodular pseudoangiomatous stromal hyperplasia of the breast: A case report**

J. Pinto ^*^, T. Amaro, M. Honavar

^*^ULS Matosinhos, Dept. de Anatomia Patológica, Rio Tinto, Portugal

**Objective:** Pseudoangiomatous stromal hyperplasia (PASH) is a benign proliferating lesion of the mammary stroma that may rarely present as a localized mass. A case of a large palpable nodular PASH is reported.

**Method:** A 22-year-old woman presented with a painless mass in her left breast. She underwent ultrasonography and core-needle biopsy and subsequent surgical excision of the mass.

**Results:** The gross specimen was a well-defined, encapsulated nodular mass measuring 12 × 8.5 × 4 cm and weighing 233 g, with a solid, opaque white cut surface. Histologically, scattered and slightly dilated terminal ductal lobular units, with simplified branching, were observed and the interlobular stroma was hypercellular with dense collagen. Anastomosing slit-like spaces lined by spindle cells with uniform and hyperchromatic nuclei were present within and between the breast lobules. No atypia or mitoses were noted. The endothelium-like spindle cells were immunopositive for CD34, smooth muscle actin and bcl-2, and negative for CD31, estrogen and progesterone receptors and cytokeratin AE1/AE3.

**Conclusion:** Nodular PASH has overlapping clinical and radiologic features with fibroadenoma. The most important differential diagnosis on histopathology is low-grade angiosarcoma. It is treated by local surgical excision with clear margins and has minimal risk of recurrence.

**H&E–40× augmentation:** 
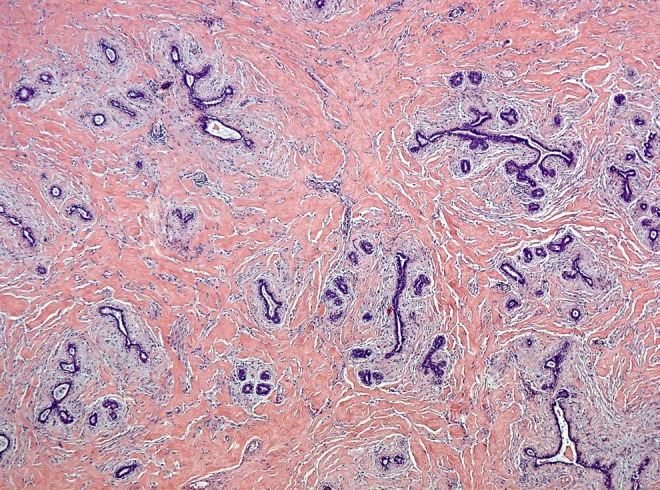


**PS-17-021**

**Features of vimentin and Ki-67 expression in basal-like breast cancer**

S. Sazonov ^*^, A. Brilliant, Y. Zasadkevich

^*^Institute of Cell Technology, Dept. of Pathology, Ekaterinburg, Russia

**Objective:** Basal-like breast cancer (BLBC) stands out for the poorest prognosis due to a high risk of distant metastases development and the ability to recurrence. Epithelial-mesenchymal transition (EMT) is thought to play a significant role in tumor progression and metastasis. One of EMT features is appearance of expression of mesenchymal markers such as vimentin on tumor cells. The aim of the research was to study features of the relation between a level of proliferation and EMT realization in BLBC.

**Method:** 68 breast carcinomas related to BLBC according to immunohistochemical (IHC) classification (ER-,PR-,HER2-) were studied. Research was performed by IHC method with use of monoclonal antibodies Anti-Swine Vimentin (DAKO, clone V9) and Mouse Anti-Human KI-67 Antigen (DAKO, clone MIB-1).

**Results:** We found that an average proliferative activity index in the BLBC group was 70.0 ± 0.8 %. Positive expression of vimentin was detected in 75 %. A strong positive correlation was found between a proliferative activity level and vimentin expression (*r* = 0.86, *p* < 0.05).

**Conclusion:** BLBC is characterized with a high proliferative activity of tumor cells as well as a high percentage of cases with positive vimentin expression that speaks about dedifferentiation of tumor cells and EMT realization that is one of the mechanisms providing metastatic potential of the tumor.

**PS-17-023**

**Characterization of immunohistochemical markers in triple negative breast carcinomas from Turkey**

E. Dogan ^*^, M. Guray, T. Ozgur, T. Canda

^*^Mustafa Kemal University, School of Medical Pathology, Hatay, Turkey

**Objective:** Triple negative breast carcinomas constitute 15–25 % of all breast carcinomas and has been correlated with aggressive behaviour and poor prognosis. Our aim is to characterize the immunphenotypes of these tumors in a group of patients from Turkey

**Method:** We applied immunohistochemical markers; CK 5/6, CK14, EGFR, E-Cadherin, p53 and androgen. Formalin-fixed parafin embedded tissues from 51 breast carcinoma; 36 triple- negative and 15 non-triple negative patients were included into the study. Mann Whitney U and Kruskal Wallis tests were performed for statistical analyses.

**Results:** The distribution of immunohistochemical markers have been evaluated and mean value was CK 5/6 78.4 %, CK14 was 84.8 %, EGFR was 87.2 %, e-cadherin was 96.9 %, p53 was 87.3 % and androgen was 89.5 % in triple negative group. CK 5/6 was 5.3 %, CK14 was 8.0 %, EGFR was 8.0 %, e-cadherin was 53.2 %, p53 was 7.3 % and androgen was 33.3 % in non-triple negative group. CK 5/6 stained significantly different in Grade 2 and Grade 3 cases (*p* = 0.035) in triple negative group. The other markers demonstrated no significant differences between two grades.

**Conclusion:** Triple-negative breast carcinomas from Turkish population also express markers of basal cytokeratins, have high levels of p53 compared to non-triple negative breast carcinomas.

**PS-17-024**

**FoxP3 expression in luminal A, luminal B, HER2 overexpressors and Triple Negative Breast Cancers (TNBC): A clinical & immunohistochemical correlation study**

J. Vidak ^*^, N. Ali, P. Trivedi, R. Ramakrishnan

^*^St. Mary’s Hospital, Dept. of Cytopathology, London, United Kingdom

**Objective:** a) To evaluate FoxP3 expression in tumour infiltrating lymphocytes (TILs) in ER+ [luminal A & B] & ER- [Her2 overexpressors & triple negative] breast cancers & b) correlate with Ki-67 proliferation index and other clinical parameters.

**Method:** FoxP3 & Ki67 expression was evaluated in a pilot set of 39 cases; 10 luminal A, 10 luminal B, 8 Her2 over-expressors & 11 TNBCs. H&E sections of tumours were reviewed to chose block for immunostaining. FoxP3 expression was counted in 100, 200 &500 TILs and expressed as %. Ki-67 index was calculated in 100 tumour cells. 200 cases (50 from each group) will be eventually evaluated for final analysis.

**Results:** In the ERpositive category, luminal A group showed median FoxP3 of 7.3 % & Ki67 index of 15.7 % while luminal B group showed median FoxP3 of 10 % & Ki-67 index of 23 %. In the ERnegative category, Her2 over-expressors showed median FoxP3 of 9.2 % & Ki-67 index of 45.2 % while in TNBCs it was 11.8 % & 38.7 % respectively.

**Conclusion:** In the ER+ tumours, increased FoxP3 expression in TILs showed a trend towards higher tumour Ki-67 index, while the trend was reversed in the TNBC group.

**PS-17-026**

**Immunohistochemical and molecular profiling of invasive apocrine carcinoma of the breast**

S. Vranic ^*^, I. Castellano, C. Marchiò, C. Botta, F. Tondat, P. F. Di Celle, M. S. Scalzo, A. Sapino

^*^University of Turin, Italy

**Objective:** Invasive apocrine carcinoma of the breast is a rare, special type of breast carcinoma, characterized by the lack of consistent data of its immunohistochemical and molecular features. The aim of the present study was to perform an immunohistochemical (IHC) and molecular profiling of the invasive apocrine carcinomas diagnosed in a single institution.

**Method:** Fifty-five invasive apocrine carcinomas were diagnosed from 1997 to 2012. For IHC profiling a tissue microarray was constructed. FISH was performed for HER-2/neu and MET gene evaluation. The gene copy number alterations were analyzed using multiplex ligation-dependent probe amplification (MLPA) probes P078-C1 Breast Tumor and P004-C1 ERBB2.

**Results:** The majority of apocrine carcinomas were ER and PR negative (84 %) and AR positive (86.7 %). Thirteen out of 52 cases (25 %) showed HER-2 overexpression. MLPA data indicate that apocrine carcinomas frequently loss BRCA1 and BRCA2 genes along with the TP53 gene. HER-2/neu amplification was confirmed in all Her-2/neu overexpressing apocrine carcinomas. Gains of MYC, FGFR-1, CCND1 and ZNF703 genes were also seen in a subset of tested apocrine carcinomas.

**Conclusion:** Apocrine carcinomas of the breast appear to harbor distinct genetic alterations that involve the most prominent oncogenes and tumor suppressors with a significant potential for the tailored therapy.

**PS-17-027**

**Stem-associated genes expression is detectable in homogenized SLN from OSNA positive breast cancer cases**

T. Perin ^*^, M. Santarosa, R. Maestro, V. Canzonieri

^*^CRO-IRCCS Aviano NCI, Pathology, Italy

**Objective:** The One-step nucleic amplification (OSNA) assay, based on Cytokeratin19 (CK19) mRNA quantification, allows a rapid intra-operative detection of metastases in the sentinel lymph node (SLN) in breast cancer patients. Here, we investigated the feasibility to probe putative prognostic markers in the homogenized SLN used for OSNA.

**Method:** 604 Patients with operable CK19 positive breast cancer and clinically negative evaluation of the axilla, were operated between October 2010 and December 2012. 199 were positive, 97 macrometastases, 71 micrometastases and 31 inhibited. Total RNA was purified from homogenized lymph-nodes and investigated by means of qRT-PCR for the expression of known stem-associated genes and luminal transcription factors.

**Results:** To demonstrate the feasibility of our approach, we proved that stem-associated genes (e.g. SOX2 and POU5F1) and luminal transcription factors were not expressed in the homogenized SLN of negative cases. Hence, we are investigating the differential expression of these cancer specific genes in a homogeneous series of CK19 positive samples in order to probe their putative prognostic value.

**Conclusion:** We consider that the homogenized SNL,used for OSNA analysis, might represent a clinical resource to identify cancer specific genes of prognostic value in the at-risk of recurrence patients among CK19 positive cases.

**PS-17-029**

**Are routine immunohistochemical studies recommended for histopathological assessment of excised sentinel nodes in breast cancer patients?**

E. Srutek ^*^, T. Nowikiewicz, W. Zegarski

^*^Centrum Onkologii, Bydgoszcz, Poland

**Objective:** In this publication we attempted to determine the usefulness of immunohistochemical studies in postoperative histopathological SLN assessment.

**Method:** Retrospective analysis was conducted on 1584 patients after SLN excision due to non-advanced breast cancer. Frozen tissue sections of SLN were dyed with hematoxylin and eosin during intraoperative assessment. Final examination was performed on paraffin sections. If metastatic lesions could not be identified, IHC staining with antibodies for cytokeratin detection (CK7, AE1/AE3) was additionally performed.

**Results:** SLN involvement was diagnosed in 351 patients (macrometastases-276, micrometastases-72, ITC-3. Use of IHC allowed for detection of metastatic changes in SLNs of 65 patients, macrometastases–24, micrometastases and ITC-41). It led to an increase in the total number of diagnoses of SLN involvement by 22.7 % (9.5 % for macrometastases and 111.8 % for micrometastases). In most patients (92.7 %) SLN histopathological examination was conducted ad hoc. The acquired sensitivity of intraoperative lymph node assessment was 63.8 % (12.5 % for micrometastases, 77.5 % for macrometastases) and specificity of this method amounted to 100 %.

**Conclusion:** Use of IHC studies in the diagnosis of SLN metastases in breast cancer patients constitutes a valuable supplementation to standard histopathological assessment.

**PS-17-030**

**Pathologic response to inducation therapy as prognostic factor in patients with locally advanced breast cancer: Results of a 6-year-long follow-up**

E. Srutek ^*^, T. Nowikiewicz, M. Piatkowska, W. Zegarski

^*^Centrum Onkologii, Bydgoszcz, Poland

**Objective:** The aim was to assess the significance of pathologic response to induction systemic treatment in our own material.

**Method:** Analysis included 75 patients with breast cancer who received induction chemotherapy. Median patient follow-up time after surgery was 56.5 months.

**Results:** Complete pathologic response pCR was identified in 8 patients, partial response pPR-17, minimal residual disease MRD-9, no response pNR- 41. Among patients with pCR there was one case of distant metastases, 4 cases among patients with MRD and 8 patients with pPR. There were 3 cases of local recurrence and 15 cases of distant metastases among patients with pNR. Median survival time in patients with pCR was 70 months, with pPR- 57.2, with pNR- 49.5 and with MRD- 61.4. Median time to recurrence in patients with pCR was 16.1 months, pPR- 18.4, pNR- 16.8, MRD- 26.8. The differences were not statistically significant (*p*-value for total survival was 0.107 and 0.941 for time to disease recurrence).

**Conclusion:** Despite not demonstrating statistical significance of the influence of type of tumor cell pathologic response to induction treatment on long-term treatment outcomes, it may be taken into consideration as an additional prognostic factor for therapeutic management.

**PS-17-031**

**Specificity analysis of estrogen receptor antibody clones using a novel high density protein microarray**

N. Ryan^*^, G. McIntosh, J. Reed

^*^Leica Biosystems, Newcastle-upon-Tyne, United Kingdom

**Objective:** The assessment of estrogen receptor (ER) status by immunohistochemistry (IHC) remains vitally important in directing the treatment of patients with breast cancer. Positivity rates can vary between laboratories due to many factors such as population demography and testing protocol sensitivity. Despite this variation there is no recognized gold standard ER IHC assay. The ASCO/CAP guidelines for IHC testing of ER identifies a number of antibody clones that have well-established specificity and sensitivity demonstrating good correlation with patient outcomes. This study aimed to use a novel protein microarray to assess the specificity of a number of commercially available ER antibody clones.

**Method:** Blinded samples of Estrogen Receptor antibody clones 6F11, SP1 and EP1 were assessed for specificity using the 10K high density protein microarray chip developed by OriGene Technologies Inc. Results from the chip analyses were verified by Western Blot.

**Results:** 6F11 was shown to be specific for ESR1. SP1 was shown to recognize ESR1 and two variants of the protein EDARRAD. EP1 recognised ESR1 and four other proteins.

**Conclusion:** The results of the OriGene 10K protein chip assay suggest that 6F11 is a more specific antibody for Estrogen Receptor than SP1 and EP1.

**PS-17-032**

**Sentinel lymph nodes with isolated tumour cells and micrometastases in breast cancer: Clinical relevance and prognostic significance**

S. S. Ahmed ^*^, A. A. Thike, J. Iqbal, P. H. Tan

^*^Singapore, Singapore

**Objective:** To determine the prognostic significance of isolated tumour cells and micrometastases to the sentinel lymph nodes of breast cancer patients.

**Method:** We performed a retrospective review of total of 1,044 patients with a diagnosis of invasive carcinoma of the breast who underwent surgical treatment including the sentinel lymph node biopsy procedure from July 2004 to October 2009

**Results:** In 710 (68 %) patients, no metastasis was seen to sentinel lymph nodes. Isolated tumour cells were detected in 22 (2.1 %) patients, micrometastasis in 52 (5.0 %) and macrometastases in 260 (24.9 %). With a median follow up of 28.8 months, in macrometastasis group, disease recurrence was seen in 38 (3.6 %) patients and 15 (1.5 %) patients died of disease. No disease recurrence or deaths were recorded in women with isolated tumour cells in sentinel lymph nodes. In the micrometastasis group, 2 patients suffered disease recurrence and both died of disease.

**Conclusion:** We conclude that isolated tumour cells to the sentinel lymph nodes did not adversely impact disease free and overall survivals. Although only 2 recurrences with subsequent death occurred in the micrometastasis group, it may suggest a propensity for presence of micrometastases to augur a worse outcome, and may justify segregation of isolated tumour cells from micrometastasis.

**PS-17-033**

**Ferroportin expression and its association with other prognostic factors in breast cancer**

M. Kadivar ^*^, A. Joulaee, A. Mojtahedzadeh

^*^Tehran University of Medical Sciences, Dept. of Pathology, Iran

**Objective:** Breast cancer is the most common malignancy in women. Recent studies described a marked decrease in the levels of ferroportin in breast cancer. More importantly, the presented results demonstrate convincingly the incredible diagnostic and prognostic value of ferroportin expression in breast cancer. This study was undertaken to define the presence of ferroportin in breast cancer and its association with breast cancer subtypes and tumor prognostic indicator.

**Method:** The Ferroportin immunohistochemical evaluation was carried out using surgical specimens from 100 cases of breast carcinoma and scored semiquantitatively on a score of 0 to 2. The overall intensity of staining and its association with tumor subtype, and tumor characteristics, such as size, grade, lymph-node involvement, and classic prognostic factor including ER, PR, Her2/neu and Ki67 were investigated using Chi-square, analysis of variance.

**Results:** Twenty eight percent of cancer samples received staining intensity score of 1. No staining Was seen in the remaining 72 % of cases. Lower Ferroportin expression was significantly seen in cases with classic prognostic factor of poor outcome including, absence of estrogen receptor, lack of progesterone receptor, positive Her2/neu and basal like molecular subtype (*P* < 0.05)

**Conclusion:** Measurement of Ferroportin expression might help aid prognosis and predict the clinical outcome of breast cancer.

**PS-17-034**

**Mixed ductal-neuroendocrine carcinoma of the breast with metastases to both adrenal glands: Case report**

L. Gurevich ^*^, I. Kazantsheva, N. Korsakova, G. Plyakova

^*^Moscow Regional Clinical, Research Institute, Dept. of Pathology, Germany

**Objective:** Primary neuroendocrine carcinomas of the breast (BC) are difficult to identify during the standard morphological examination. Little is known about mixed ductal-endocrine BC, their clinical course and prognosis.

**Method:** 48-year-old woman underwent surgery for the BC. Diagnose was “infiltrating lobular carcinoma”, and standard chemotherapy was applied.

**Results:** Four years later metastases were found in patient’s adrenal glands (AG). Metastasis in the left AG (7.5 cm) composed of large spindle cells complexes. Cells were synaptophysin (Syn) and E-cadherine-positive, PR, ER, cytokeratin 7 (CK7), mammaglobin (MG) and vimentin negative, Ki67 proliferation index (IP) was 44,5 %. Metastasis in the right AG (6 cm) composed of small clear cell complexes, Syn, E-cadherin positive, PR, ER, MG focal positive, CK7, vimentin negative. Ki67-IP was 28 %. We retrospectively investigated the primary BC and found two components in it: in the first one the tumor cells were PR, ER, CK7, MG, E-cadherin positive, Syn negative, in the second component–Syn positive and CK7, MG, PR, ER negative. Finally mixed ductal-endocrine BC was diagnosed

**Conclusion:** Use of the standard chemotherapy in this case contributed to the survival and progress of only poorly differentiated neuroendocrine component. Prognosis for mixed ductal-endocrine BC is most likely to depend on the correct diagnosis and individual therapy

**Metastases of the breast cancer in the adrenal glands (A) and lymph node (B). Expression E-cadherin in tumor cells (B). Synaptophysin (C) and Ki-67 (D) expression in tumor cells of the metastasis of the left adrenal gland.:** 
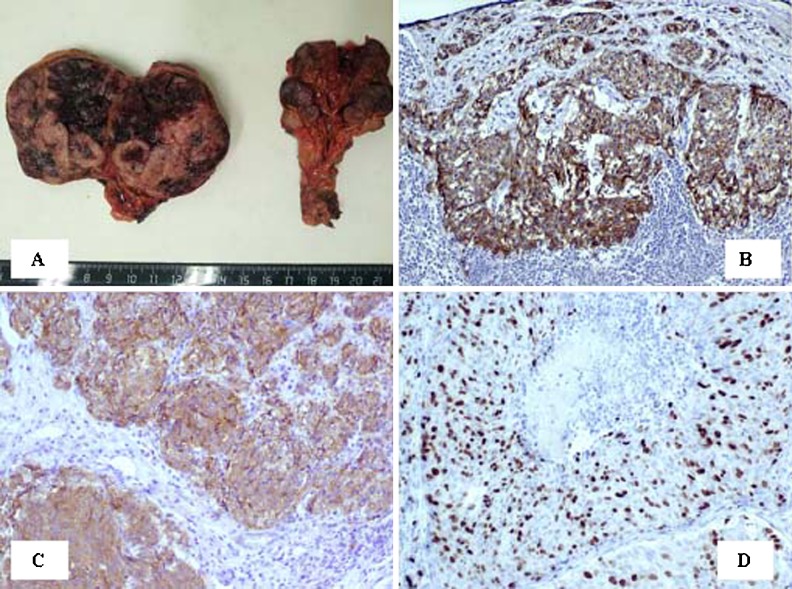


**PS-17-035**

**Breast cancer stromal elastosis: Association with mammography screening detection and favourable prognostic markers**

Y. Chen ^*^, T. A. Klingen, K. Liestøl, H. Aas, Ø. Garred, J. Mæhlen, L. A. Akslen, J. Lømo

^*^Akershus University Hospital, Dept. of Pathology, Lørenskog, Norway

**Objective:** Screening-detected cancers have a better prognosis than predicted from established prognostic markers. A search for additional criteria that are characteristic for screening cancers and a favourable prognosis is needed. Here, we have investigated whether the presence of elastosis in the tumours may serve as such a criterion.

**Method:** We performed a population based retrospective study of breast cancers detected in the Norwegian Breast Cancer Screening Programme in Vestfold county from 2004 to 2008. 202 invasive screening-detected cancers and 45 interval cancers in age group 50–69 years were compared with regard to standard clinic-pathologic parameters and several additional histological parameters. In particular, the presence of elastotic material in tumours was graded on a 4 tiered scale.

**Results:** Screening-detect cancers had a significantly higher content of stromal elastosis than interval cancers (*p* < 0.001). The degree of elastosis showed a strong covariation with low histological grade, ER positive and HER-2 negative status, stellate tumor shape and tumor with high stroma content.

**Conclusion:** There is a strong correlation between the presence of tumour elastosis and mammography detection of breast cancers. Presence of elastosis seems to be associated with a good prognosis.

**PS-17-036**

**A case of breast angiosarcoma associated with pregnancy diagnosed by fine needle aspiration**

R. N. Demirbag ^*^, B. Öztürk, Ö. Odabas, G. Saygi, A. Küçük

^*^Avrasya Hospital, Pathology, Istanbul, Turkey

**Objective:** Primary angiosarcoma of the breast is a rare tumor that account for fewer than 0,05 % of all malignant mammary tumors. It is more frequent in the second and third decade. Between 6 and 12 % of cases are diagnosed during pregnancy or shortly after. Diagnosis prior to surgery, either by fine needle aspiration (FNA) or core biopsy is difficult.

**Method:** We present a case of breast angiosarcoma associated with 7 months of pregnancy in a 26-year-old woman diagnosed by FNA cytology. She had a 87 × 66 mm painfull, hemorrhagic mass at her left breast.

**Results:** In smears, there were cellular sheets of atypical spindle cells some forming vasculer like spaces. There was mitotic activity. Immunhistochemistry was performed on cell blocks. The atypical spindle cells showed strong posıtıvıty with CD31. Ki-67 index was about 10 %. Ostrogen and progesteron receptors were negative. She refused the surgery before the delivery. After 6 months, she came back with disseminated bone and lung metastases. Then, the mass was 15 cm in diameter. The breast skin was ulcerated and bleeding. The diagnosis was confirmed by histopathology after the palliative mastectomy and the patient was lost 2 weeks after the surgery.

**Conclusion:** The diagnosis of angiosarcoma can be made by FNA if the smears are satisfactory and a cell block can be prepared.

**Spindle cells forming vascular spaces, ×400:** 
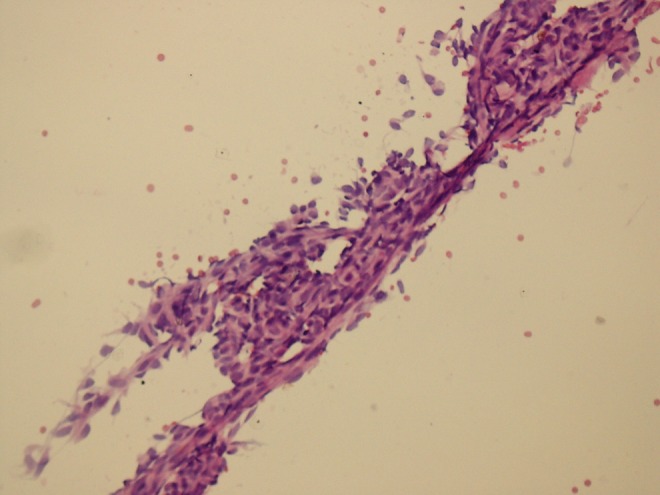


**PS-17-037**

**Breast carcinoma with choriocarcinomatous features: A case report**

O. Tzaida ^*^, K. Ntatsis, P. Giagkazoglou, I. Kasselaki, I. Lekka, P. Arapantoni- Dadioti

^*^Metaxa Anticancer Hospital, Dept. of Pathology, Piraeus, Greece

**Objective:** Breast carcinoma with choriocarcinomatous features (BCMF) is a rare subtype of breast cancer characterized by highly atypical cells morphologically similar to choriocarcinoma cells, immunoreactive to human chorionic lactogen (HCL) as to human chorionic gonatropin (HCG). A theory of metaplastic process in the development of this special, usually aggressive malignancy, has been proposed.

**Method:** A 73 year old woman, presented with a solitary, palpable left breast tumor measuring 1,8 × 1,5 × 1 cm. A quadrectomy with sentinel axillary lymph node dissection was done. Histopathology revealed an invasive, Grade 3, breast carcinoma with metaplastic features (areas of squamous and spindle cell morphology) in a hemorrhagic and necrotic background. A distinct choriocarcinomatous component, consisted of markedly atypical multinucleated cells, immunohistochemically positive for HCL and HCG, was demonstrated. The tumor cells, negative for ER, PR and HER2 were locally immunoreactive to CK5/6, p63 and EGFR. A diagnosis of a T1cSN0M0 metaplastic, triple negative BCMF was established. Adjuvant local radiotherapy and hormonotherapy (Stephaplex) was provided. Two years after diagnosis, the patient is without evidence of disease.

**Conclusion:** BCMF is a rare, special subtype of metaplastic breast carcinoma. This malignant entity implies differential diagnostic problems with metastatic choriocarcinoma to the breast and requires appropriate treatment modalities.

**PS-17-038**

**Phyllodes tumors with a malignant phenotype: A clinicopathological study of 25 cases**

O. Tzaida ^*^, G. Galanopoulos, E. Paliouri, I. Iakovidou, K. Ntatsis, D. Kondylis, P. Arapantoni-Dadioti

^*^Metaxa Anticancer Hospital, Dept. of Pathology, Piraeus, Greece

**Objective:** Depending on histological appearance of their stromal component, phyllodes tumors with a malignant phenotype (PTMP) display a morphological spectrum ranging from low grade fibrosarcoma (borderline phyllodes tumors: BFT) to high grade sarcoma (malignant phyllodes tumors: MPT).

**Method:** Twenty-five cases of PTMP diagnosed during 20 years from a total of 4,726 mammary malignancies (0.4 %), are reviewed. Immunohistochemical analysis with several tumor markers was performed.

**Results:** Six (24 %) were classified as BFT and 19 (76 %) as MPT with a mean tumor size of 5.4 cm and 8,26 cm and a median age 53.8 and 54.4 years, respectively. In six cases an heterologous stromal component was detected. One case had also developed a carcinomatous element. All patients underwent surgery (wide local excision or simple mastectomy) while four received postoperative, adjuvant radiotherapy or chemotherapy. Follow-up, during 3 months to 18 years, was possible for 15 patients. One patient experienced local recurrence and another one developed distal metastases in lungs, both of them with MPTs.

**Conclusion:** PTMP are rare breast neoplasms with a good correlation between histological classification and patient clinical outcome. Surgery is the appropriate first line therapy. Because these tumors can recur, close follow-up is usually recommended.

**PS-17-039**

**Assosiation of primary adenocarcinoma of large bowel and colonic metastasis from lobular breast cancer: A case report**

I. Iacovidou ^*^, D. Papagiannopoulou, G. Mitropoulou, H. Trihia, I. Lekka, P. Arapantoni- Dadioti

^*^Metaxa Cancer Hospital, Dept. of Pathology, Piraeus, Greece

**Objective:** We present a rare case of co-occurrence of primary adenocarcinoma of the colon and metastasis of a lobular breast cancer in pericolic fat and regional lymph nodes.

**Method:** The patient is a 75 year old Caucasian woman with lobular breast carcinoma Grade 2 diagnosed in 1998. Eleven years later a gastric biopsy by a private pathologist diagnosed diffuse gastric cancer followed by gastrectomy. In December 2012 she was operated for colonic cancer in our hospital and on this occasion a histological re-evaluation in our department revealed metastatic infiltration of the stroma and not a primary gastric cancer. Bone marrow biopsy showed also massive infiltration by lobular mammary cancer.

**Results:** Pathological examination revealed indeed primary adenocarcinoma of the colon T2N0Mx and the presence of metastatic lobular breast carcinoma in pericolic fat and two regional lymph nodes. Immunohistochemical expression of metastatic cancer cells was positive for CK7, ER, PR, GCDFP-15 and P120, and negative for CK20, CDX2, E-cadherin and PGM1.

**Conclusion:** Pathologists and clinicians should be aware to rule out the possibility of mammary metastatic cancer, a tumor with a great risk of misdiagnosis especially in patients with an acquainted diagnosis.

**PS-17-040**

**A comparative study of the ER, PR, cERB2 and other parameters in 154 women who were operated with breast cancer in Theageneion Hospital of Thessaloniki**

P. Eskitzis ^*^, B. Tarlatzis, A. Papanikolaou, M. Zafrakas, I. Dimitriadis, M. Gkoutzioulis, E. Panagopoulou, G. Simpilidis

^*^Ptolemaida, Greece

**Objective:** The study was conducted in Theageneio Hospital among 154 women, aged 30–80 years, who had partial breast excision and lymphadenectomy.

**Method:** For the study were used (ER),(PR) receptors, and CERB2/oncoprotein, to evaluate the histological and immunohistochemical phenotype of the tumors and associate it with the size, the grade of differentiation, the age and the lymph node metastases.

**Results:** The histological grade was associated with the size of the tumors. The ER,PR were expressed independently of the grade. The expression of CERB2 was negative in 65 % of the tumors. Mitotic activity was related to the size of the tumors and the infiltration of lymph nodes.

**Conclusion:** The increase in the grade of modulation is related to the size of tumors and the infiltration of lymph nodes but not to the expression of ER,PR,CERB2. According to the results, the predominant immunophenotype was the ER(+)PR(+)CERB2(−), witch was expressed in 48 % of tumors in the study.

**Table:** 

GRADE	Tumor size (average)	ER(−)	ER(+)	PR(−)	PR(+)	CERB2(−)	CERB2(+)	Age (average)	Lymph Node Metastases
I	1.4	3(0.019 %)	5(0.032 %)	5(0.032 %)	3(0.019 %)	5(0.032 %)	3(0.019 %)	53.8(8 patients)	3 patients(0.019 %)
II	1.8	9(0.058 %)	77(0.500 %)	17(0.110 %)	69(0.448 %)	63(0.409 %)	23(0.146 %)	60.5(86 patients)	30 patients(0.194 %)
III	2.3	21(0.136 %)	39(0.253 %)	28(0.181 %)	32(0.207 %)	32(0.207 %)	28(0.181 %)	59.7(60 patients)	26 patients(0.168 %)

**PS-17-041**

**Audit of adequacy of lymph node sampling on axillary samples in breast carcinoma and the additional yield following GEWF fixation**

J. Thorne ^*^, M. Harrison

^*^Dublin, Ireland

**Objective:** To quantify the nodes retrieved in axillary specimens received Sept 2011–Feb 2012, ensuring our findings were compliant with international guidelines. To determine the impact of GEWF fixation on nodal yield.

**Method:** All axillary samples received in the Mater Private Hospital for the designated 6 month period were included. Following normal sampling the specimens were fixed in GEWF for 24 h. Each specimen was then re-examined and any remaining lymph nodes grossly identified were embedded. Total lymph node count was documented for each patient and the presence or absence of metastatic disease was determined.

**Results:** In total 20 samples were received from 18 patients. 247 nodes were identified following normal sampling. The mean number of nodes identified following routine fixation was 12.35 (range 2–35). Following GEWF fixation extra nodes were found in 6 specimens (1 extra node in 3 cases, 2 extra nodes in 2 cases and 3 extra nodes in 1 case). All were <3 mm and one showed a metastasis. Pathological staging was not altered in any case.

**Conclusion:** The mean number of nodes retrieved per specimen was 12.35, within international guidelines. Improved node yield was noted following GEWF fixation but no impact on pathological stage was seen.

**PS-17-042**

**Ductal carcinoma arising within a mammary fibroadenoma: A case report**

H. Trihia ^*^, E. Paliouri, G. Galanopoulos, G. Vecchini, K. Kalogerakos

^*^Metaxa Cancer Hospital, Dept. of Pathology, Piraeus, Greece

**Objective:** Fibroadenoma is the most common benign breast tumor in women under 30 years of age. However, carcinoma arising within a fibroadenoma is unusual. We report a case of an invasive ductal carcinoma arising within a fibroadenoma.

**Method:** A 50-year old woman was admitted for the investigation of mammographically-detected microcalcifications. Histological examination revealed the presence of microcysts, papillomatosis, ductal epithelial hyperplasia, sclerosing adenosis and multiple fibroadenomas. The larger one, of 1,4 cm diameter, was occupied partly by an invasive ductal carcinoma, grade I.

**Results:** A carcinoma arising in a fibroadenoma is mostly an incidental finding. Azzopardi et al. states that there are three possibilities: carcinoma arising in the adjacent breast tissue engulfing and infiltrating a fibroadenoma; in the crevices of a fibroadenoma as well as in the adjacent breast tissue; and carcinoma restricted entirely, or at least dominantly, to a fibroadenoma. Our case corresponded to the third criteria. The biological behavior of carcinoma arising within a fibroadenoma does not differ from that of a breast carcinoma alone.

**Conclusion:** We present this case to increase awareness of this entity of the rare association of two common breast diseases and stress the need for histological evaluation for the diagnosis of malignancy.

**PS-17-043**

**Pagetoid spread of lobular carcinoma cells in a blood vessel wall. A unique phenomenon of tumor dissemination, not previously described: A new mechanism of metastasis?**

H. Trihia ^*^, I. Kasselaki, D. Kondylis

^*^Metaxa Cancer Hospital, Dept. of Pathology, Piraeus, Greece

**Objective:** The majority of breast cancers metastasize via lymphatics and blood vessels, as tumor emboli. A unique phenomenon of intra-endothelial spread of carcinoma cells of a lobular carcinoma of the breast is presented for the first time.

**Method:** A 42-year old woman was diagnosed with metachronous bilateral lobular carcinoma of her breasts. There were also tumor emboli and lymph node metastases. In between there was intra-abdominal spread to both her ovaries and the omentum.

**Results:** Intra-endothelial spread of malignant cells was noted in a blood vessel wall. Tumor cells were positively stained with KerAE1/AE3 and Ker7. An attenuated layer of endothelial cells was maintained on the luminal surface of the blood vessel, while carcinoma cells were ‘running’ underneath them, above the basement membrane.

**Conclusion:** Although pagetoid spread is commonly seen in LCIS and invasive lobular carcinoma of the breast, affecting ducts and nipple skin, pagetoid spread in a blood vessel wall has not been described in the past. This is obviously another form of tumor dissemination, which could also serve as an explanation for ‘dormant carcinoma’ cells, could partly explain the low sensitivity of current techniques for the detection of circulating tumor cells and could also been taken into consideration regarding new therapeutic strategies.

**PS-17-044**

**Extensive axillary lymph node metastases with focal DCIS and microinvasion in the breast in a patient with a 3 years history of continuous breast feeding**

A. Arnaout ^*^, E. St John, L. Alarcon, C. Pogson

^*^Croydon University Hospital, Dept. of Histopathology, United Kingdom

**Objective:** A 42 year old lady presented with right axillary lymphadenopathy and 3 years history of continuous breast feeding. Clinically no breast lump was encountered.

**Method:** Triple assessment of the axilla and breast was performed.

**Results:** Mammography and ultrasound of the breast were normal but highlighted the enlarged axillary nodes. The axillary lymph node biopsy and immunocytochemical stains showed histological features in keeping with metastatic ductal carcinoma G3 ER and PR negative but HER2 +3 positive. Subsequently MRI of the breast showed ill-defined features and an MDT decision for a Right mastectomy made and performed. Meticulous gross inspection showed a central firm and fibrotic area measuring 10 mm which on Histology revealed a high grade DCIS with focal microinvasion ER and PR negative HER2 +3 positive similar to the metastatic lymph node expression surrounded by a heavy lymphocytic inflammatory infiltrate and fibrosis consistent with regression changes. Eight macro metastatic axillary nodes out of 16 were identified.

**Conclusion:** The findings were unusual and although we have no proof of a heterogeneous tumour an antioestrogenic effect induced by breast feeding resulting in regression of the hormone sensitive tumour component should be considered.

**PS-17-046**

**Audit of lymph node retrieval in axillary sampling in breast cancer: Correlation with specimen weight**

J. Thorne ^*^, M. Harrison

^*^Dublin, Ireland

**Objective:** To quantify lymph nodes from axillary specimens in breast carcinoma and compare the number retrieved with the weight of the specimen to determine a correlation.

**Method:** All axillary specimens received in the department from 01/09/2010 to 31/08/2011 were included. The histology reports were reviewed; the specimen type (sentinel node, axillary clearance, axillary node, axillary sample, axillary dissection) and weight were documented along with the total nodal count in each case.

**Results:** A total of 153 specimens were received from 108 patients. 72 patients had sentinel node only. Weight was recorded in 84 % of cases (*n* = 128). The mean weight for sentinel nodes was 4.6 g with a mean nodal count of 1.6. The mean weight of the axillary samples, dissections and clearances was 40.8 g with a mean nodal count of 6.2. The mean weight for axillary clearances was highest at 89.65 g with a mean nodal count of 12.9.

**Conclusion:** A range of axillary specimens was received with many patients having multiple specimens. The mean number of nodes from clearance specimens was above the minimum expected as per international guidelines. A positive correlation between the weight of the specimen and the number of nodes retrieved was identified.

**PS-17-048**

**Diabetic mastopathy: A case report**

F. Gursoy^*^, G. Kir, S. Cetin, M. Segmen Yilmaz

^*^Umraniye Research Hospital, Dept. of Pathology, Istanbul, Turkey

**Objective:** Diabetic mastopathy is a rare benign entity and is typically observed as a self-limiting fibro-inflammatory disease of the breast. It occurs in premenopausal women affected by longstanding type I. It clinically presents as multiple palpable painless breast masses. Diabetic mastopathy and breast cancer’s clinico-radiological findings are very similar and can be confusing.

**Method:** We report a case of a 48-year-old female, with an 4-year history of type 1 insulin-dependent diabetes, presented with a painless palpable lump in her left breast.

**Results:** Ultrasound showed an irregular, slight asymmetric with dense glandular tissue and a hypoechoic mass that measured 14 × 18 × 10 mm. Microscopilly, keloid like fibrosis, lymphocytic lobulitis and ductulitis with glandular atrophy and perivascular lymphocytic infiltration were observed.

**Conclusion:** The diagnosis of diabetic mastopathy is based on histological examination and these findings are characteristic. The patients do not need to undergo a surgery however it is necessary to plan an adequate long-term care of these patients.

**PS-17-049**

**Complex metaplastic carcinoma of the breast expressing SOX2: Travelling the full spectrum of breast ductal neoplasia**

A. Sagasta Lacalle ^*^, A. Saco, A. B. Larque, L. Rodriguez Carunchio, A. Ruiz, A. Carrió, R. Falcón Escobedo, P. L. Fernández

^*^Hospital Clinic Barcelona, Dept. de Anatomía Patológica, Spain

**Objective:** Metaplastic breast cancers with mesenchymal metaplasia are rare and account for less than 1 % of invasive breast cancers. We report a rare case of a 45-year-old woman with a 22 cm, lobulated, ulcerated mass in her right breast.

**Method:** Immunohistochemical analysis included various CK, vimentin, CD68 p63, p53, Ki67, RE, RP, cerb2, ZEB1 and SOX2. FISH for p53 and chromosomes 17 and 8 completed the study.

**Results:** Light microscopy showed a metaplastic carcinoma with complex histology including extensive areas of squamous and undifferentiated carcinoma as well as pleomorphic sarcoma and osteosarcoma. Isolated foci of high grade ductal in situ and infiltrating carcinoma were found. Mitoses were abundant and often bizarre. The epithelial component was negative for hormone receptors, Her2, and positive for HMW keratin and keratin 5 in squamous areas. Pleomorphic cells expressed vimentin, Zeb1, P53, P63 and CD68. Interestingly, SOX2 was strong and diffusely positive in the majority of the pleomorphic cells. FISH analysis demonstrated multiple copies of P53 both in pleomorphic mesenchymal and squamous cells.

**Conclusion:** We hypothesize that this case represents a variant of metaplastic neoplasms enriched in cancer pluripotent cells conferring great plasticity, leading to a complex phenotypical pattern which encompasses the full spectrum of breast ductal neoplastic transformation.

**Pleomorphic components:** 
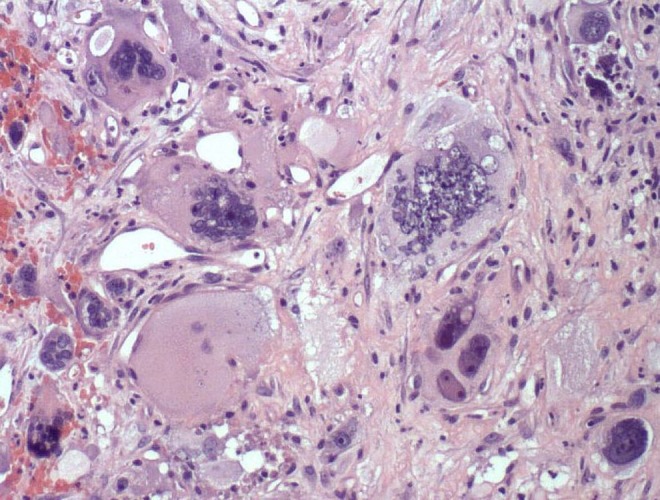


**PS-17-050**

**A pathologic evaluation of the sentinel node in breast cancer: A biomolecular protocol compared with a histological method**

V. Canzonieri^*^, T. Perin, A. Carbone

^*^CRO-IRCCS Aviano NCI, Dept. of Pathology, Italy

**Objective:** We compared the histological protocol used from June 2000 to December 2005 to assess the status of the sentinel node (SLN), with the biomolecular OSNA protocol used from October 2010 to December 2012.

**Method:** The histological protocol was based on the microscopic examination of serial sections, every 50 μm, of the entire SLN. OSNA procedure relies on the liquid phase evaluation of the expression of CK19 mRNA for the rapid detection of metastases

**Results:** The histological protocol was applied to 731 women, mean age of 54 years (range 30–84) (1170 SNL/1,6 SLN/pt). 289 were metastatic, (39.5 %,130 macro-metastases and 159 μ-metastases). 270 underwent axillary dissection (81 positive LNs, 30 %). The biomolecular method was applied to 604 women, mean age 58.75 y (range 28–82) (940 SNL/1.55 SLN/pt). 199 were metastatic (33 %, 97 macro-metastases and102 μ-metastases). 186 underwent axillary dissection (68 positive lymph nodes: 36.5 %).

**Conclusion:** Histological and biomolecular protocols were applied in two large groups of patients matched for age and clinical stage. No differences emerged between the biomolecular and histological protocols in ascertaining the status of the SLN. OSNA procedure demonstrates better results, reducing the time of examination and warranting axillary dissection during quadrantectomy or mastectomy.

**PS-17-051**

**Breast cancer bone metastases: The impact of decalcification in Hormone Receptor (HR) and HER-2 status**

D. Felizardo ^*^, N. Coimbra, T. Sarmento, J. Vieira, N. Afonso, C. Leal, C. Bartosch, M. Afonso

^*^Portuguese Oncology Institute, Dept. of Pathology, Matosinhos, Portugal

**Objective:** Reassessment of HR/HER-2 status in relapsing disease is necessary for patient management because differences between primary and metastatic tumor have been reported. Although bone is a preferential metastatic site in breast cancer, the majority of data comes from studies in non-bone metastases. We aimed to assess the impact of decalcification in HR/HER-2 status on bone biopsies.

**Method:** Retrospective review of 56 biopsies of breast cancer bone metastases (2010–2011). Clinicopathologic reevaluation of all cases and comparison with primary tumor. Semi-quantitiative evaluation of specimen quality and quantity/intensity HR expression. HER-2 was scored when available. Statistical analysis (McNemar’s, Wilcoxon and Fisher’s exact test).

**Results:** Table 1.

**Conclusion:** Although bone is traditionally considered inadequate for HR/HER-2 reevaluation, the changes we observed in bone metastases are similar to those reported for non-bone metastases. HR/HER-2 expression does not seem to be substantially influenced by decalcification procedures and thus reassessment of biomarkers may be feasible in bone biopsy. Careful evaluation of discrete staining is mandatory and caution must be taken when interpreting negative results due to potential decalcification artifacts. Since bone may be the first and/or only breast cancer metastatic site, larger studies are needed to address the real impact of decalcification process in bone biopsy.

**Table 1.:**

**Table:** 

Table 1. Change in HR/HER-2 status between primary tumor and bone metastases							
ER		PR		HER-2			
+ → −	− → +	+ → −	− → +	+ → +	+ → −	+ → Eqv	− → −
4/52 (7.7 %)	1/4 (25 %)	24/45 (53.3 %)	1/11 (9.1 %)	2/7 (28.6 %)	3/7 (42.9 %)	2/7 (28.6 %)	38/38 (100 %)
■ Conversion between primary and metastatic tumor was significantly more frequent in PR (*p* < 0.001) than in ER.							
■ In ER-positive cases, there was a significant (*p* = 0.046) reduction in the number of ER-positive cells in bone metastases.							
■ In the PR-positive cases, there was no significant (*p* = 0.338) change in the number of PR-positive cells in bone metastases.							

**PS-17-052**

**Histopathological and immunohistochemical findings in four primary papillary breast tumours, without mammaglobin expression**

N. Petre ^*^, F. Pop, D. M. Pop

^*^Medcenter Bucharest, Dept. of Pathology, Romania

**Objective:** In females, less than 2 % of breast carcinomas are papillary carcinomas. Breast cancer in males accounts for only < 1 % of all cases of breast cancer and invasive carcinoma of no special type (NST) is the most common type. Invasive papillary carcinoma is more common in males than in females accounting for 2–4 % of cases. We present 4 cases (2 males and 2 females) with papillary invasive breast carcinoma, three with solid papillary carcinoma with invasion and one with encapsulated papillary carcinoma with invasion.

**Method:** One case of solid papillary carcinoma with invasion was thought to be a breast metastasis of an fallopian tube carcinoma, but the lack of p 53, WT1, CA 125 expression and the GCDFP-15 focal positivity confirmed the breast origin. We have studied for all cases the immunohistochemical expression of GCDFP-15+ Mammaglobin, CK 7, ER, PgR, c-erbB-2, Ki 67, ChromograninA, Synapthophysin and CD 56.

**Results:** All cases was focally positive for GCDFP-15 and CK7 but negative for Mammaglobin. Only one case of solid papillary carcinoma with invasion expressed the neuroendocrine markers.

**Conclusion:** Our findings suggest that the lack of expression of Mammaglobin observed in the cases we have studied may be a special features of solid papillary and encapsulated invasive breast neoplasm.

**PS-17-053**

**Comparison of histological grade, hormone receptor status and Her-2 status in breast cancer pre- and post-neoadjuvant chemotherapy**

I. Jovanic ^*^, Z. Milovanovic, O. Zivkovic, J. Stojsic

^*^IORS, Dept. of Pathology, Belgrade, Serbia

**Objective:** Changes in tumor markers between biopsies performed before and after neoadjuvant chemotherapy (NAC) are controversial. The aim of study is to compare histological grade and immunohistochemical (IHC) expression of estrogen, progesterone and Her2/neu in breast cancer before and after treatment and to correlate the expression of tumor markers with response to NAC.

**Method:** Retrospecively analysis of 49 patients with locally advance breast carcinoma diagnosed on core needle biopsies and surgical specimens was performed. IHC staining for ER, PR, Her2/neu were available before and after NAC.

**Results:** Changes between histological grade pre- and post-NAC were statistically significant (*p* = 0,006). Pre-chemotherapy IHC revealed 37 (75,51 %) ER+, 30 (61,22 %) PR+ and 12 (24,49 %) Her2+ cases. Three (7,14 %) ER+, two (4,76 %) PR+ and one (2,38 %) Her2+ patients lost positivitu after NAC. Initially three (7,14 %) PR- and three (7,14 %) Her2- tumors were PR + and Her2+ after NAC. No correlation was found between marker expression before NAC and pathological response.

**Conclusion:** No significant changes were seen in steroid receptors and Her2 status before and after NAC, but retesting of these markers in residual tumor shoud be considered in certain situations to optimize adjuvant systemic therapy.

**PS-17-054**

**Prognostic value of matrix metalloproteinase 2 (MMP-2, gelatinase A), matrix metalloproteinase 9 (MMP-9, gelatinase B) and E26 transformation-specific-1 transcription factor (ETS-1) in breast cancer patients**

V. Puzovic^*^, I. Brcic, J. Jakic-Razumovic

^*^General Hospital Dubrovnik, Dept. of Pathology, Croatia

**Objective:** The aim of this study was to analyse expression and coexpression of ETS-1 protein and two gelatinases in sense of their possible prognostic value in breast cancer patients.

**Method:** The expression of MMP-2, MMP-9 and ETS-1 was immunohistochemicaly analysed in 121 primary breast carcinomas. Expression and coexpression of ETS-1, MMP-2 and MMP-9 were correlated with other clinicopathological parameters and patient’s survival.

**Results:** The ETS-1 protein, detected as nuclear and cytoplasmatic staining, was found positive in 56,3 % breast carcinoma patients. MMP-2 and MMP-9 were expressed in cytoplasm of tumour cells, and were found positive in 77,7 % and 90 % breast carcinoma patients respectively. The observed MMP-2, MMP-9 and ETS-1 coexpression in tumour cells was more common in tumours larger than 2 cm, histological grade III, steroid receptor negative tumours, and were not associated with lymph node positivity, vascular invasion, HER-2 status or Ki-67 proliferation index. Statistically significant impact on overall survival and disease free survival was found in patients with coexpression of ETS-1, MMP-2 and MMP-9 in tumour cells.

**Conclusion:** Our results suggest that MMP-2, MMP-9 and ETS-1 coexpression might be used as a poor prognostic predictor in breast cancer patients.

**PS-17-055**

**Large tumor forming pseudoangiomatous stromal hyperplasia of the breast: A case report**

S. Bozanic ^*^, N. Solajic, S. Knezevic Usaj

^*^Institut za Onkologiju, Odeljenje za Patologiju, Sremska Kamenica, Serbia

**Objective:** Pseudoangiomatous stromal hyperplasia (PASH) is a benign mesenchymal proliferation with numerous slit-like spaces lined by spindle cells. This is not an uncommon finding showing small microscopic foci, but tumor forming PASH is rare, especially in postmenopausal women.

**Method:** A 73-years old woman presented with a painless tumorous lump of the left breast. Physical examination revealed about 80 × 80 mm, round, easily movable mass of upper quadrants, with no paplpable axillary lymph nodes. Mammographic finding pointed to a well circumscribed oval, non-calcified mass. Ultrasonography showed a well-defined hypoechoic mass. Surgical excision was done with pathologic frozen section examination.

**Results:** Gross examination revealed a 80 × 75 × 45 mm, well demarcated, rubbery-hard mass with fibrous grayish–white cut surface. There were a few small cysts filled with a tan fluid. Microscopic examination showed anastomosing slit-like, empty spaces, lined by spindle cells with bland nuclei lacking both pleomorphism and mitotic figures. Immunohistochemically, the spindle cells were positive for CD34, calponin and weakly for actin, but negative for CD31, cytokeratins, estrogen and progesterone receptors.

**Conclusion:** A tumorous PASH is a rare benign entity which has to be distinguished from vascular lesion, especially from low grade angiosarcoma. The recommended treatment is wide local excision.

**PS-17-056**

**Evaluation of breast cancer after preoperative chemotherapy**

A. Batistatou ^*^, S. Kamina, A. Ntoulia, A. Zioga, A. Goussia, V. Malamou-Mitsi

^*^University of Ioannina, Medical School, Dept. of Pathology, Greece

**Objective:** The aim of the study was to evaluate the effects of preoperative chemotherapy (PCT) on breast cancer morphology and biomarker expression.

**Method:** The material consisted of formalin-fixed paraffin-embedded tissue from 12 breast cancer patients treated with PCT (pre-treatment biopsy and post-treatment surgical material). Histological sections were examined for detailed morphological features. Furthermore, immunohistochemical expression of ER, PgR, HER2 and Ki67 was evaluated.

**Results:** One of the patients exhibited complete pathological response, one residual disease only in the lymph nodes, while the remaining ten had residual invasive carcinoma. Those cases with significant regression exhibited considerable morphologic changes. Changes in the expression of ER were noted in 4/11 (36 %) cases, with change in status in only one (negative to positive). Changes in PgR expression were observed in 4/11 (36 %) cases, with change in status in only one (negative to positive). HER2 overexpression was lost in one case. In 3/11 (27 %) cases there was reduction in Ki67 expression. All biomarker changes were unrelated to morphological changes.

**Conclusion:** PCT induces changes in the morphology of remaining neoplastic cells as well as in biomarker expression. Both negative to positive and positive to negative conversion has been noted, thus evaluation of post-therapy biomarkers is necessary.

**PS-17-057**

**Expression of cyclin E and epidermal growth factor receptor in invasive breast cancer**

A. Mariamidze ^*^, O. Khardzeishvili, M. Gudadze, G. Dzagnidze, I. Kiladze, I. Sikharulidze, G. Burkadze

^*^Tbilisi, Georgian

**Objective:** The aim of study is to reveal the features of expression of Cyclin E and EGFR in breast cancer with different Grade and phenotype.

**Method:** We have assessed the post-operative specimens of 362 patients.

**Results:** The study has revealed Er/Pr/Her2 negative (triple negative) cases in 14,6 %, Er/Pr/Her2 positive cases in 5,8 %, Er/Pr positive/Her2 negative cases in 62,1 % and Er/Pr negative/Her2 positive cases in 17,4 %. The most of Cyclin E positive cases were found in triple negative cancers (54,6 %), and maximum was noted in triple negative/CK5 positive cases (57,7 %). Also, high rate of expression was noted in Er/Pr negative/Her2 positive cases (33,2 %) and Er/Pr/Her2 positive cases (28,5 %). The rare Cyclin E positive cases were detected in Er/Pr positive/Her2 negative tumors (7,9 %). The most cases of triple negative cancer express EGFR in 39,5 %, maximum number of EGFR positive cases were noted in triple negative/CK5 positive cases (49,9 %). Er/Pr/Her2 positive (38,5 %).

**Conclusion:** The number of Cyclin E positive cases are different in cancers with different phenotype and it increases with aggressiveness of cancer. EGFR positive cases has phenotype specific features and does not progress with aggressiveness.

**PS-17-058**

**Astronomy-based image processing for quantification of breast tumour histopathology**

H. Ali ^*^, A. Dariush, M. Irwin, E. Provenzano, F. Blows, P. Pharoah, N. Walton, J. Brenton, C. Caldas

^*^CRUK Cambridge Institute, Functional Genomics, United Kingdom

**Objective:** To investigate methods for the quantitative interrogation of breast cancer histopathology using image-processing techniques adapted from astronomy. We are developing a high-throughput image analysis platform to rapidly analyse samples from large clinical studies.

**Method:** Tumour samples represented in tissue microarrays (TMAs) from the SEARCH population-based study were immunostained for oestrogen receptor (ER) and progesterone receptor (PR). Digital images of tissue-cores were subjected to image-processing algorithms adapted from those used in astronomy for the analysis of large, high throughput, sky surveys of the night sky. The degree of staining in tumour cells, the cellular composition of tissue cores, cell-morphology and spatial relationships between cell types were evaluated.

**Results:** Automated readouts of staining, cell types and relational features were generated. These data will be correlated with clinical, histopathological and molecular characteristics using over 3,000 tumour samples. The pattern of associations for different image-features will be presented.

**Conclusion:** Astronomy-based image processing techniques represent a viable method for the quantitative analysis of histopathological features of large collections of tumour samples including novel cellular and spatial characteristics.

**PS-17-059**

**Ki-67 labeling index as a predictive factor of chemosensitivity in breast cancer HR+HER2-: Advantage of systematic sampling and counting using stereological rules**

M. Oger ^*^, M. Allaoui, P. E. Brachet, E. Coquan, J. Marnay, N. Elie, C. Levy, B. Plancoulaine, P. Herlin, C. Bor-Angelier

^*^CLCC Francois Baclesse, Calvados, Caen, France

**Objective:** Estimation of proliferation in breast cancers is mandatory, especially for luminal subtypes, by Ki67 Labeling Index(LI). Accurate quantification methods of this immunohistochemical marker are still needed by pathologists and oncologists in order to have a reliable biomarker reflecting aggressivity of a tumor and chemosensitivity.

**Method:** Study was performed on a series of 92 women with the same neoadjuvant chemotherapy for invasive breast carcinomas, positive for estrogen receptor and negative for HER2. Evaluation of pathologic complete response(pCR) was made on surgical specimens. Estimation of Ki67LI was performed on pretherapeutic core-biopsies using a standardized immunochemistry procedure. We compare three methods of quantification done by visual count under microscope by a pathologist: eyeball, then counting 400 cells whose the first 100 was designated as the hotspot and by manual counting on corresponding digitized slides using stereology box grids.

**Results:** Best correlation for mean value is obtained between eyeball and stereology count(*r* = 0.87, *p* < 0.0001). There’s no correlation between Hotspot and pCR. Correlation is found between Ki67 stereology value and pCR (*p* = 0.003). 100 % of pCR has LI>12 %.

**Conclusion:** Strength of sampling and counting using stereological rules to determine Ki67LI is highlighted. KI67LI mean value seems to be a reliable biomarker of chemosensitivity for luminal breast cancers HR+HER2-.

**PS-17-060**

**Lymphangiogenesis as a negative prognostic factor of breast cancer metastasis into regional lymph nodes: Morphometric analysis of chosen vasculature parameters in tumor and regional lymph node surroundings**

A. Stanek-Widera ^*^, J. Nozynski, E. Zembala-Nozynska, D. Lange

^*^Institute of Oncology, Pathology, Gliwice, Poland

**Objective:** Lymphangiogenesis is induced by tumor progression and accompanies metastatic process. In here I investigated the relationship between increased vessel density in tumor as well as lymph node margins and incidence of metastasis in ductal breast carcinoma cases. The goal of the study was to assess lymphangiogenesis in breast cancer as a potential prognostic marker of metastatic spread incidence to regional lymph nodes.

**Method:** We examinated lymphatic and blood vessel (immunohistochemical staining CD34 and podoplanin) in 60 patients by using image analysis system.

**Results:** Lymphangiogenesis was compared between infiltrated and cancer-free lymph nodes. The obtained results allow to make a link between metastatic spread occurrence and increased peritumoral vessel density. The results obtained show that average density of lymphatic vessels in breast carcinoma is substantially higher in cases involving cancer spread.

**Conclusion:** This may mean that intensity of lymphangiogenesis in breast cancer constitutes a negative prognostic factor. In addition, presence of metastases in lymph nodes was accompanied by statistically significant increase in average density of lymphatic vessels.

**PS-17-061**

**Neuroendocrine cell hyperplasia of the breast: Potential precancerous lesion of mammary neuroendocrine carcinoma**

T. Kawasaki ^*^, G. Bussolati, I. Castellano, C. Marchiò, L. Daniele, L. Molinaro, R. Katoh, A. Sapino

^*^University of Turin, Biomedical Sciences, Italy

**Objective:** The developmental mechanisms of breast neuroendocrine carcinoma (B-NEC) have not been sufficiently analyzed and are not as yet well understood. This study aimed to clarify the presence of a precursor lesion of B-NEC.

**Method:** We serially investigated 32 totally-resected mammary tissues with a B-NEC by immunohistochemistry for specific NE markers (chromogranin A/synaptophysin). Thirty-two mastectomy specimens harboring a mammary non-NE carcinoma were similarly examined.

**Results:** Extensively-distributed NE cells were immunohistochemically identified in the background mammary ducts/lobules of 7 (22 %) of 32 B-NECs. These NE cells were classifiable into three emerging patterns: isolated/scattered, clustered and circumferential. Their distributions were intermingled and were not clearly related to B-NEC foci. NE cells were morphologically polygonal, oval or columnar with sometimes eosinophilic and/or fine-granular cytoplasm and round-to-ovoid nuclei lacking atypia. Some cells were located between epithelial and myoepithelial cells. Apical snouts were occasionally observed in NE cells forming luminal structures. In contrast, 32 non-NE carcinomas had no NE cells in the cancer lesion background.

**Conclusion:** Benign-appearing NE cells in the parenchyma of a breast with NEC could be regarded as hyperplastic based on their emerging patterns and distribution; this NE cell hyperplasia may be associated with the histogenesis of some B-NECs as a premalignant condition.

**PS-17-062**

**Apocrine adenoma of breast in a 7 year-old patient: Case report**

A. Ribeiro ^*^, A. Figueiredo, M. M. Martins

^*^Centro hosp lisboa central, Amadora, Portugal

**Objective:** Breast mass lesions in children are rare, usually benign, and may arise from abnormal breast development, infection or trauma. In male patients they usually represent gynecomastia and malignancy is rare. Tubular apocrine adenomas are benign lesions, described in the breast and on the skin.

**Method:** We present a case of a 7 year old male patient with no relevant clinical past history, apart from recent history of trauma (dog bite) in the areolar region, he now presents with a painless subareolar lesion with 4 cm in largest dimension. Endocrinology assessment was performed and concluded he was pre-puberal (Tanner 1). Ecography showed a hypoechogenic partially cystic lesion. The clinical differential diagnosis were: haemangioma, lymphangioma or haematoma. On gross examination it represented a well defined mass with multiple cysts.

**Conclusion:** We believe this lesion to represent an apocrine adenoma with low grade intraductal neoplasia. The origin of this lesion, whether from skin or breast glands, is questionable although being areolar based and the presence of normal breast ducts argues in favour of being a breast lesion. This lesion in males is very rare and we are not aware of any other reports in the English literature in such young patients.

**PS-17-063**

**Fibromatosis of the breast: A case report**

H. Trihia ^*^, M. Papazian, G. Galanopoulos, D. Papagiannopoulou, K. Kalogerakos

^*^Metaxa Cancer Hospital, Dept. of Pathology, Piraeus, Greece

**Objective:** Fibromatosis of the breast is rare and most patients present with radiologic findings that closely mimic invasive carcinoma. A minority of cases are associated with prior trauma or have a history of prior breast surgery.

**Method:** A 63-year old woman with a history of breast cancer was diagnosed with a radiologic ill-defined, partly nodular density, of the upper outer quadrant of her left breast, during her regular follow up.

**Results:** A 4,2 × 2,5 × 1 cm mammary specimen was excised which showed an ill-defined, 1,4 cm solid area of elastic consistency, close to the surgical margin. Microscopically, there was a mildly cellular area consisting of long fascicles of spindle cells, without cellular atypia or mitotic activity, merging focally with more collagenized areas. In areas there was infiltration around ductules with epithelial hyperplasia. Cells were positive for SMA and negative for KerAE1/AE3, desmin, CD34 and p63. There were no inflammatory cells, haemosiderin-laden macrophages, or foreign body giant cell reaction.

**Conclusion:** Mammary fibromatosis is a neoplastic proliferation of fibroblasts that is locally infiltrative but which does not metastasize. It can mimic invasive carcinoma both radiologically and macroscopically. Fibromatosis should be considered in the differential diagnosis of a suspicious mammographic finding in women with history of prior breast surgery.

**PS-17-064**

**Hypersecretory DCIS with multiple microscopic foci of invasive pure mucinous carcinoma of the breast in a young woman: An interesting case**

H. Trihia ^*^, M. Papazian, G. Stanc, C. Farfarelos

^*^Metaxa Cancer Hospital, Dept. of Pathology, Piraeus, Greece

**Objective:** Hypersecretory carcinoma is characterized by the presence of eosinophilic secretory material. Majority of cases are in situ carcinomas. Rare associated invasive carcinomas are poorly differentiated and have solid growth pattern. We present a case of a young woman with hypersecretory type DCIS, accompanied by multiple foci of invasive mucinous carcinoma.

**Method:** A 34-year old woman was diagnosed with microcalcifications of her left breast. She had given birth in 2008 and 2010. She was submitted to partial mastectomy for diagnostic purposes.

**Results:** The mastectomy specimen measured 8,5 × 7 × 1,5 cm. On cut surface there were white foci of elastic-hard consistency, approximately 5,8 cm. Microscopically, it was mostly occupied by ductal in situ carcinoma, of hypersecretory type, with clear cell change and intraluminal eosinophilic secretion. There were also foci of micropapillary and comedo type. PAS and PAS-D stains revealed the presence of abundant intracellular glycogen and rare intracytoplasmic mucin droplets. Some of the intraluminal secretion was also positive for Alcian blue stain. There were also foci of mucocele-like changes, due to the spillage of mucin in the stroma, as well as three independent microscopic foci of invasive mucinous carcinoma. There were no lactational changes found.

**Conclusion:** The combination of hypersecretory DCIS with mucinous carcinoma has not been described before.

**PS-17-065**

**Discordance of estrogen and progesterone receptors expression between cells of primary lesions and locoregional metastases in breast cancer**

K. Konyshev ^*^, A. Brilliant, S. Sazonov

^*^IMCT, Laboratory of Pathomorphology, Yekaterinburg, Russia

**Objective:** The research was performed to reveal and characterize changes in estrogen and progesterone receptor (ER, PgR) status of locoregional metastatic cells in comparison with primary breast cancer cells.

**Method:** Tumor tissue of primary carcinoma and local lymph nodes metastases from 73 patients with breast cancer was assessed by immunohistochemistry for ER and PgR expression using the monoclonal mouse antibodies clone ER 1D5 and clone PgR 636 (Dako). Samples were rated using the Allred scoring guideline.

**Results:** From 73 examined cases of primary breast cancer with metastatic lesions of regional lymph nodes 7 (9,6 %) switches of estrogen status and 10 (13,7 %) switches of progesterone status were revealed. 3 cases (4,1 %) of positive to negative changes and 4 cases (5,5 %) of negative to positive changes for ER; 2 cases (2,7 %) and 8 cases (11,0 %) respectively for PgR were identified.

**Conclusion:** Possibility of discordance of ER and PgR status between primary breast cancer cells and locoregional metastatic cells was found. Changes of receptor status in metastatic process may occur both in negative and in positive primary lesions. These results put under the question the sufficiency of ER and PgR expression examination only in primary breast cancer lesion for adequate therapeutic management.

**PS-17-066**

**Male breast invasive lobular carcinoma: Report of two cases**

R. Canas Marques ^*^, F. Cunha, P. Machado, F. Vaz, S. André

^*^Rio de Mouro, Portugal

**Objective:** Male breast cancer (MBC) is infrequent and occurs in older age than female breast cancer (FBC). Invasive lobular carcinoma (ILC) is the second most frequent subtype of FBC (with high rate of multicentricity and bilaterality and increased incidence since 1980s) but is exceptional in men. In FBC, the occurrence of ILC and invasive carcinoma NOS was not different between carriers of BRCA2 mutations and controls. BRCA2-related MBC has been reported at earlier age compared with non-BRCA2-related, with no differences in other clinicopathological features. Our aim is to report the clinicopathological characteristics of two cases of male ILC, one with BRCA mutation.

**Method:** Clinical data was obtained from medical records. Histopathological characteristics were recorded.

**Results:** Table 1 summarizes the clinicopathological data. Table 1 FH: familiar FBC history; Ie: in evaluation; NED: no evidence of disease.

**Conclusion:** The two cases of ILC in males occur in relatively young patients with familiar FBC history, being one BRCA2 related. Multicentricity or bilaterality were not present. The immunoprofile is the usual in ILC of FBC.

**Table:**
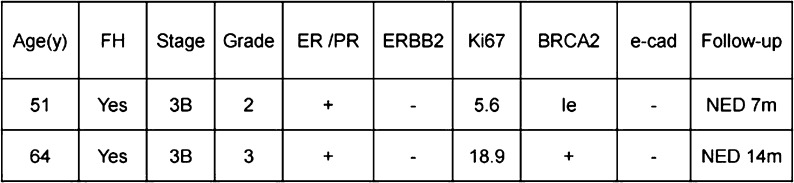

**PS-17-067**

**Breast schistosomiasis japonica**

T. Kawasaki ^*^, G. Bussolati, I. Castellano, C. Marchiò, L. Daniele, L. Molinaro, R. Katoh, A. Sapino

^*^University of Turin, Biomedical Sciences, Italy

**Objective:** Schistosomiasis japonica is an important colonic and hepatic parasitosis but is exceptionally rare in the breast. Herein, we describe four cases of breast schistosomiasis japonica detected by screening mammography.

**Method:** All four patients were postmenopausal Japanese women, 63–81 years old (mean 70.3), from the Kofu basin, in Yamanashi prefecture, an area previously endemic for schistosomiasis japonica.

**Results:** Screening mammography revealed numerous unilateral micro-calcifications of the breast in all cases. These amorphous calcifications were distributed in segmental regions and, in addition, fine linear calcifications were also seen in one case. All patients were clinically suspected to have breast ductal carcinoma in situ (DCIS) based on these mammographic findings. Histologically, needle biopsy specimens showed elliptical calcifications, 50 to 80 μm in length, around terminal ducts and lobules. These calcified bodies had miracidium- and shell-like structures, indicating calcified ova of Schistosoma japonicum. No cancers, such as DCIS, or other breast diseases were identified.

**Conclusion:** It is noteworthy that, while extremely rare, schistosomiasis japonica can occur in the breast and may be a potential mammographic pitfall since its calcification patterns mimic those of DCIS. Therefore, it is worth considering this parasitic disease if a patient with breast micro-calcifications resides or has traveled in an endemic area.

**PS-17-068**

**High prevalence of neuroendocrine carcinoma in breast lesions detected by the clinical symptom of bloody nipple discharge**

T. Kawasaki ^*^, G. Bussolati, I. Castellano, C. Marchiò, L. Daniele, L. Molinaro, R. Katoh, A. Sapino

^*^University of Turin, Biomedical Sciences, Italy

**Objective:** Bloody nipple discharge (BND) is an important clinical symptom in breast disorders, especially cancers. However, the association between this symptom and breast neuroendocrine carcinomas (NECs) has not been sufficiently investigated or well understood.

**Method:** We clinicopathologically studied 89 cases using biopsy and/or resection in 144 patients who came to the hospital for a thorough examination of symptomatic BND.

**Results:** Of these 89 cases examined histologically, 24 (27 %) were neuroendocrine carcinomas (NECs) in which >50 % of cells immuno-expressed chromogranin A and/or synaptophysin. Moreover, NECs made up 44 % (24/55) of the mammary cancers found because of the BND. The 24 NECs were subclassified into neuroendocrine ductal carcinoma in situ (NE-DCIS) (9 cases) and microinvasive (7 cases) and invasive (8 cases) NECs with extensive NE-DCIS components. The frequency of diagnosing malignancy preoperatively in 24 NECs was 4 % by nipple discharge cytology, 40 % by fine needle aspiration cytology, 62 % by core needle biopsy and 67 % by mammotome biopsy.

**Conclusion:** NECs predominantly with NE-DCIS lesions, often under-diagnosed preoperatively, accounted for an important share of breast conditions associated with BND. It is, therefore, worth keeping this type of breast cancer in mind when performing medical examinations on patients with BND.

**PS-17-069**

**Retinoic Acid Receptor-alpha (RAR-α) is an independent prognostic marker in breast cancer and it plays different roles in ER-positive and HER2 tumours**

R. Abduljabbar ^*^, E. Rakha, D. Jerjees, C. Nolam, C.-F. Lai, L. Buluwela, A. Green, S. Ali, I. Ellis

^*^University of Nottingham, Academic Oncology, United Kingdom

**Objective:** Retinoic Acid Receptor alpha (RAR-α) is a nuclear receptor (NR) that localises to oestrogen receptor (ER)-binding regions to modulate the expression of many oestrogen-related genes. Gene expression profiling studies have identified RAR-α to be associated with luminal ER-positive breast cancer (BC)

**Method:** This study investigates RAR-α protein expression using immunohistochemistry in a large (*n* = 1,150) cohort of BC patients to assess its biological and clinicopathological significance

**Results:** RAR-α expression is mainly nuclear with 85 % of the cases (*n* = 966) showing high expression (H-score >130). High RAR-α expression is positively correlated with ER, progesterone and androgen receptor and LRH-1 protein. Its expression is reduced or absent in Triple negative (TN) BC while the highest expression is observed in low-grade tumours with tubule formation. Outcome analysis showed that RAR-α expression is associated with longer survival (*p* = 0.005) in the whole series and in ER-positive but not in TN tumours. In HER2-positive tumours, RAR-α shows an association with shorter survival independent of ER expression. Multivariate analyses demonstrate RAR-α as an independent predictor of outcome

**Conclusion:** These findings confirm the relationship between RAR-α and ER and demonstrate that RAR-α plays different roles in ER-positive and HER2 positive tumours highlighting the complex interaction between ER, HER2 and RAR-α.

**PS-17-071**

**Oestrogen receptor and HER2 differentially activate ERK/MAPK signaling pathway in breast cancer: A large cohort study**

D. Jerjees^*^, R. Abduljabbar, C. Nolan, A. Green, I. Ellis, E. Rakha

^*^Nottingham, United Kingdom

**Objective:** The extracellular signal-regulated kinase (ERK)1/2 is a key member of ERK/MAPK pathway that plays important roles in breast cancer (BC) growth and different cellular fates.

**Method:** Aassessing phosphorylated(p)-ERK1/2expression in a large well-characterized series of early stage BC(*n* = 1,150)using immunohistochemistry.

**Results:** p-ERK1/2 is expressed in cytoplasm and nuclei of malignant cells. ER is associated with overexpression and nuclear localization of p-ERK1/2 whilst HER2 is associated with down-regulation of nuclear p-ERK1/2 expression. In ER+tumours,HER2 retained its negative association with p-ERK1/2. However, in HER2+cases,the association with ER is lost. Differentiation associations between p-ERK1/2and other variables are obtained when data were stratified according to ER and HER2. Importantly,in ER+/HER2-BC,p-ERK1/2 is associated with lower-grade,smaller size positivity for BRCA1,E-cadherin,Bcl2,luminal enriched proteins (MUC1,FHIT),androgen and other ER-related proteins including FOXA1,TFF1,CARM1,CCNBI,BEX1,XBP1,PELP1 and GATA3. However,P-ERK1/2 shows an inverse association with HER4,ck5 and KI67. Outcome analyses reveal better outcome with nuclear and worst outcome with cytoplasmic expression alone

**Conclusion:** ER and HER2 differentially activate ERK/MAPK signaling pathway. Moreover, subcellular localization of p-ERK1/2 alone contrary to nuclear was associated with worst outcome

**PS-17-072**

**Metaplastic breast carcinoma: Clinicopathologic features of 12 cases**

F. Bolat ^*^, T. Canpolat, A. Tarim

^*^BASKENT UNIVERSITESI, Adana, Turkey

**Objective:** Metaplastic carcinoma (MC) is a rare tumor showing high histological grade and low hormone receptor expression. It has pure epithelial and epitelial-mesencymal types. Herein we present histopatological characteristics and hormone receptor status of 12 MC cases of the breast.

**Method:** Twelve cases of MCs were reviewed for their histopathologic features. Histopathologic features evaluated for each case included the age of the patient, tumor grade, tumor size, tumor growth pattern, histological subtypes, the extend of axillary lymph node involvement, estrogen/progesterone receptors and HER-2/neu status.

**Results:** The mean patient age was 57.6 years and the mean tumor size was 4.06 cm. Histological grade was 3 for all patients. Average mitotic count was 36/10 high power fields. Five patients had axillary nodal metastases; the metastatic component was epithelial only in 4 cases. Microcopically, 4 cases were mixed type (carcinosarcoma) and 8 cases were pure epithelial type (7 were adenosquamous cell carcinoma, 1 was squamous cell carcinoma). Nine of the 12 cases were triple-negative for hormone receptors (estrogen receptor-, progesterone receptor -, HER2/neu -).

**Conclusion:** Metaplastic carcinoma of the breast is usually a triple-negative carcinoma, with large size, high histological grade and frequent mitotic activity.

**PS-17-073**

**Expression and potential involvement of connexins in breast cancer progression**

I. Teleki ^*^, M. A. Szász, J. Kulka, N. Meggyesházi, G. Kiszner, B. Balla, T. Krenács

^*^Semmelweis University, 1st Dept. of Pathology, Budapest, Hungary

**Objective:** Connexin (Cx) channels (gap junctions) are involved in cell homeostasis and their deregulation is linked to carcinogenesis. New data suggest their context-dependent roles during tumor progression.

**Method:** We have analyzed 4 Cx isotypes using in silico mRNA expression based on gene expression data of HGU133A and HGU133+2 microarrays (Affymetrix, Santa Clara, CA) and survival information of 1,809 patients from gene expression omnibus (GEO) and tested the their expression in 252 paraffin embedded breast cancer samples in tissue microarrays using fluorescence digital microscopy (3DHistech Ltd, Budapest, Hungary).

**Results:** High (>median) Cx43 mRNA levels were linked with improved relapse free survival (RFS) in the ER positive subgroup (*p* = 0.036) and also linked with significantly better distant metastatic disease free survival (*p* = 0.002) in the whole patient cohort), in the grade 2 subgroup (*p* = 0.00038) and in the lymph node negative (*p* = 0.004) subgroup. Patients with decreased Cx32 and elevated Cx26 and Cx43 protein level had a significantly better RFS (pCx32 = 0.013; pCx43 = 0.023; pCx32 = 0.027). Cx43 and Cx26 proved to be a significantly better prognostic factor than vascular invasion and necrosis. Cx26 was found as good a prognostic factor as hormone receptor status or Ki67 expression.

**Conclusion:** Connexin expression may serve as a useful prognostic factor in breast cancer diagnostics.

**PS-17-074**

**Dermatofibrosarcoma protuberans arising in a mastectomy scar: A case report**

A.-M. Fericean ^*^, G. Kalodimos, L. Mavrogiannis, A. Kerasioti

^*^General Hospital of Volos, Pathology, Greece

**Objective:** Dermatofibrosarcoma protuberans (DFSP) is a cutaneous tumour of low malignant potential with high rates of local recurrence. It has been reported to arise within scars.

**Method:** We report the case of a 65-year old Caucasian female who presented with a 4.5 cm firm mass beneath the scar of a right mastectomy she had undergone 22 years ago. Microscopic examination revealed a spindle cell neoplasm of low to medium cytologic atypia and few mitoses. Immunohistochemistry was positive for vimentin, CD34 and factorVIII (focally), while being negative for pancytokeratin, desmin, S100, SMA, HHF35 and HHV-8. Ki67 labelling index was 20–25 %.

**Results:** Based on the above the neoplasm was classified as DFSP. It had developed at the site of scar tissue in the dermis. Investigation of the patient’s history revealed a mastectomy 22 years ago with a diagnosis of malignant phyllodes tumour, the stromal component of which had been diagnosed as both liposarcomatous and leiomyosarcomatous. A year later a dermal recurrence of the leiomyosarcomatous component had occurred. After this the patient had been in good health for over 20 years.

**Conclusion:** The case is reported because of the problems in differential diagnosis it raises (DFSP versus recurrent phyllodes tumour) and because of its rarity.

**PS-17-075**

**Metaplastic breast carcinoma with chondroid differentiation: Cost-effectiveness of fine-needle aspiration biopsy and needle-core biopsy in a 18 cases series**

M. T. Soler Monsó ^*^, L. Perez-Casanovas, I. Morilla, M. Terricabras, A. Petit, F. Climent, R. Ortega, A. Garcia-Tejedor, I. Catala

^*^Hospital de Bellevitge, Pathology, L’hospitalet De Llobregat, Spain

**Objective:** Fine-needle aspiration biopsy (FNAB) and needle-core biopsy(NCB) allow diagnostic approach in the majority of breast neoplasias. Cost-effectiveness biopsy in metaplastic carcinoma with chondroid differentiation (MCCD) has not been studied.

**Method:** We selected pure and mixed MCCD. We evaluated the correlation between the diagnosis in FNAB/NCB and the resection specimen

**Results:** There were 13 pure and 5 mixed cases of MCCD. FNAB was performed in 4 cases. Six patients had NCB and 8 patients had both FNAB and NCB. None of the FNAB was diagnostic of MCCD but myxoid material was detected in 3/12(25 %). MCCD was diagnosed in 7/14(50 %) with NCB. Neither FNAB nor NCB was able to diagnose MCCD when chondroid component was ≤10 %. When chondroid component was>10 %, MCCD was suspected in 3/7 FNAB(42,8 %) and diagnosed in 7/10 NCB(70 %).

**Conclusion:** The mesenchymal component in MCCD is difficult to identify in FNAB/NCB. The possibility to suspect or diagnose MCCD in FNAC and NCB is 25 % and 50 % respectively. The percentage of chondroid component and experience in identifying myxoid material, could be related factors in its detection in FNAB and NCB.

**PS-17-077**

**The protein expression of nestin in invasive breast carcinomas**

C. Magkou ^*^, A. Tampaki, I. Theohari, E. Tsaldari, I. Giannopoulou, A. Keramopoulos, L. Nakopoulou

^*^Evaggelismos General Hospital, Pathology, Cholargos, Athens, Greece

**Objective:** Nestin, an intermediate filament protein, was initially described as a marker for neural stem cells and gliomas. In recent years its expression has been studied in various cancer types and has been correlated with an aggressive phenotype. Concerning breast cancer, few studies have been performed. The aim of our study was to determine the prognostic value of nestin expression in invasive breast carcinomas.

**Method:** Immunohistochemistry was applied on 280 paraffin sections with invasive breast cancer for the detection of nestin. Univariate and multivariate statistical analyses were used for the determination of nestin relation to clinicopathologic parameters, breast cancer molecular subtypes, molecular markers (p53, MMP2) and patients survival.

**Results:** Nestin expression was detected in the cytoplasm and membrane of cancer cells in 7.9 % (22/280) of the cases and was positively related to histologic and nuclear grade (*p* = 0.021 and *p* = 0.002, respectively), p53 (*p* = 0.009) and MMP2 (*p* = 0.011). Nestin was more frequently expressed in triple-negative breast carcinomas (14/22), though this relation was not statistically significant. Nestin expression was not correlated with other parameters or patients survival.

**Conclusion:** It appears that nestin expression is related to less differentiated breast carcinomas and it seems to be involved in the invasive and metastatic process.

**PS-17-078**

**Bilateral male breast cancer and BRCA2 mutation**

F. Santos ^*^, F. Cunha, P. Machado, F. Vaz, S. André

^*^IPO Lisboa, Anatomia Patológica, Lisbon, Portugal

**Objective:** Bilateral Male Breast Cancer (BMBC) is exceptionally rare. Several risk factors have been proposed, including BRCA2 mutations. In women, BRCA1/2 mutation carriers have a high risk of developing bilateral breast cancer, but in men this risk is unknown. Also, BRCA2 mutation is associated with increased risk of other malignancies. The BMBC of our institution were reviewed.

**Method:** We report 4 cases of BMBC. Clinical data was obtained from medical records. Histopathological characteristics were recorded.

**Results:** First breast malignancies presented at a mean age of 60 years (median 57.5 years). One patient had synchronous tumors (ST). All patients underwent modified radical mastectomy. All tumors were invasive carcinomas of no special type, positive estrogen receptors (ER). The ST was both ERBB2 positive; all the others were ERBB2 negative. All patients had a family history of breast cancer and all carried BRCA2 mutations. Other malignancies developed in all cases: prostate cancer occurred in three, one of them also had vesical urothelial carcinoma, and neuroendocrine gastric neoplasia in the patient with ST.

**Conclusion:** All the BMBC had BRCA2 mutations and a positive family history. Also, all developed other malignancies. BMBC data relies on institutional case series, and multiinstitutional investigation may improve the current knowledge.

**PS-17-079**

**Nuclear-cytoplasmic PARP-1 expression predicts poor clinical outcome in lymph node-negative early breast cancer–15-year follow-up**

P. Donizy ^*^, G. Pietrzyk, A. Halon, C. Kozyra, P. Surowiak, R. Matkowski

^*^Wroclaw Medical University, Dept. of Pathomorphology, Poland

**Objective:** The aim of the study was to examine the expression of PARP-1 in breast cancer (BC) patients and to assess the relationship between the sub-cellular localization of this protein and clinicopathological characteristics.

**Method:** Reactivity of PARP-1 was analyzed by immunohistochemistry in a homogeneous group of 83 stage II ductal BC patients with 15-year follow-up.

**Results:** Different pattern of expression was noted: 58 % of tumors with cytoplasmic expression (CE) and 42 % with co-expression within nucleus and cytoplasm (NCE). NCE was correlated with higher probability of disease recurrence, especially in lung and liver (*P* = 0.011). NCE was associated with shorter overall survival, which was not statistically significant during first 10 years follow-up and became statistically significant after 10 years of observation–during 15-years follow-up (*P* = 0.009). Analysis performed in subgroups of patients with (N+) and without (N−) nodal metastases showed, that NCE was associated with poor clinical outcome in N- patients (*P* = 0.017). It was not observe any significant correlations between parameters of PARP-1 expression and survival of N+ patients. Multivariate analysis confirmed significant impact of NCE on unfavorable prognosis in N(−) early BC.

**Conclusion:** Presence of PARP-1 NCE may be a new potential unfavorable prognosticator in lymph node-negative early BC.

**PS-17-081**

**HER2/neu positive status, estrogen receptor, progesterone receptor, and their association with clinico-pathological parameters in North Tunisian breast carcinomas**

Z. Nour^*^, L. Charfi, K. Mrad, R. Sellami, R. Doghri, N. Boujelbène, I. Abbes, M. Driss, S. Sassi, K. Ben Romdhane

^*^Institut Salah Azaiz, Pathology, Tunis, Tunisia

**Objective:** To measure the frequency of estrogen receptor (ER), progesterone receptor (PR), Her2Neu status and to investigate their association between tumor characteristics in breast carcinoma

**Method:** A retrospective study in the department of pathology of Salah Azaiz Institute in Tunisia. 147 cases of confirmed breast carcinoma were reviewed. The following factors were evaluated: tumor size, tumor grade, lymph node status, ER, PR, Her2neu status, and Ki67 index. The association of ER, PR and Her2neu status with clinico-patholigical parameters was determined with Chi2 test.

**Results:** Mean patient age was 50,90. ER, PR and Her2neu were positive, in respectively 74,8 %, 69,4 %, 18,4 % cases. Ki67 index was≥ 20 in 53,1 % cases. Her2neu positive was inversely related to ER expression (*P* = 0,011) and to PR expression (*P* = 0,002). Her2neu was highly represented in grade 3 tumors (*P* = 0,707). Her2neu positivity was more important in ki67 ≥ 20 % with 20,5 % compared to 13,2 % in ki67<20 without significant difference (*P* = 0,334). ER and PR was related to tumor grade1–2 (*P* < 10–3). ER positive tumors, Her2neu positive status was reduced in PR positive tumors than in PR negative tumors (28,6 % versus 11,5 %).

**Conclusion:** ER and PR were inversely correlated to Her2neu positive status. A negative association was reported concerning hormonal receptors and tumor grade

**PS-17-082**

**Comparative histopathology of benign mammary disorders in woman and dog**

V. Deckwirth ^*^, P. Kronqvist, M. Lintunen

^*^University of Helsinki, Dept. of Veterinary Biosciences, Finland

**Objective:** Female dog has been proposed as model for human mammary tumor research. However, benign mammary disorders (BMD) have been omitted. Many BMD are recognized in woman as premalignancy or marker of increased risk for invasive breast cancer (IBC). Our objective was to map comparable BMD in female dog.

**Method:** Formalin-fixed, paraffin-embedded mammary gland tissue samples from canine patients (*n* = 771) submitted for tumor diagnosis in 1998–2012. Histological comparison to woman (literature based data) was performed with HE-stained sections and immunohistochemistry (antibodies for e.g. Ki-67, ERα, HER2, E-cadherin). Canine epidemiological data was analyzed.

**Results:** Several comparable BMD were identified from the categories non-proliferative lesions and proliferative lesions without/with atypia. These include such as duct ectasia, cysts, adenosis, fibroadenoma, usual/atypical ductal hyperplasia, DCIS and lobular neoplasia. Canine epidemiological results show shared characteristics with woman.

**Conclusion:** Our results indicate canine BMD as an option for translational research of premalignancies and markers for IBC risk in woman. However, their occurrence in normal non-cancerous canine mammary gland should be evaluated.

**PS-17-083**

**Correlation of HER-2 status with immuhistochemical markers and histopathologic features in invasive breast carcinomas**

M. Aschie ^*^, A. Mitroi, I. Poinareanu, M. Enciu

^*^Pathology, Constanta, Romania

**Objective:** Our study investigates the relationship of HER-2 status with immunohistochemical expression of estrogen and progesterone receptors and Ki-67 and histopathologic features of invasive breast carcinoma.

**Method:** Thirty cases diagnosed with invasive breast carcinoma were selected from the Pathology Department of the Emergency Clinical County Hospital of Constanta. These cases were pathological revaluated. HER-2 status was determined on formalin fixed paraffin embedded sections by immunohistochemistry with HER-2 antibody followed by identification of gene status using HER-2 gene probe by chromogenic in situ hybridization (CISH). Immunohistochemical analysis for estrogen and progesterone receptors and for Ki-67 was also performed.

**Results:** Our study revealed a strong positive correlation between HER-2 protein expression determined by immunohistochemistry and HER-2 gene status determined by CISH (*r* = 0.78, *p* = 0.0006). HER-2 overexpression or amplification was limited to grade 2 and 3 carcinomas. Among HER-2 positive tumors the rate of estrogen and progesterone receptors was significantly decreased in high grade tumors compared with intermediate grade tumors. Ki-67 expression is significantly increased in tumors with HER-2 amplification and high grade.

**Conclusion:** The study emphasizes the role of the above markers in characterization of invasive breast carcinomas.

**HER2 normal status:** 
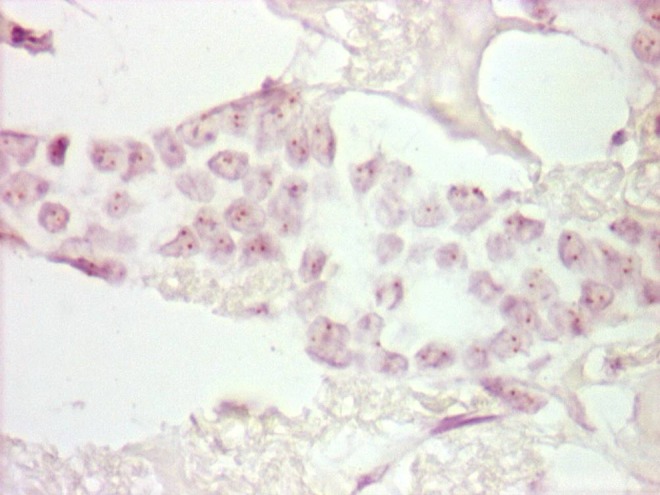


**PS-17-084**

**Idiopathic granulomatous mastitis: Clinicopathological study of four cases**

N. De La Ossa Merlano ^*^

^*^HUGTiP, Pathology, Badalona, Spain

**Objective:** Idiopathic granulomatous mastitis (IGM), an uncommon disease with clinical and radiological features mimicking malignancy, may be initially difficult to identify. Definitive diagnosis is achieved by a careful histopathological examination that rules out an infectious aetiology, among others. We report four IGM cases diagnosed and treated at the HUGTP Breast Pathology Unit with excellent clinical and aesthetic results.

**Method:** Four female patients (age, 29–38 years) consulted because of a breast lump. Bilateral lumps were present in one case and a solitary ulcer with mastitis was seen in another. X-rays showed features suspicious (BI-RADS 4) or highly suspicious (BI-RADS 5) for malignancy and, consequently, biopsies were performed.

**Results:** Microscopic study revealed similar findings in all four cases, namely acute and chronic inflammation with multinucleated giant cells, fat necrosis, non-caseating granulomas, and loss of acinar structures. Acid-fast bacilli, periodic acid-Schiff and Gram stains were all negative. Following steroid treatment the breast lesions progressively improved, with an excellent outcome.

**Conclusion:** Histopathological study is essential to determine the non-malignant nature of IGM lesions and rule out infectious conditions associated with granulomatous reactions. Although the aetiology of IGM is currently unknown, the excellent outcome obtained with steroid treatment is very suggestive of an autoimmune basis.

**PS-17-085**

**Microcalcifications as presenting feature of mammary amyloidosis**

F.-X. Sáenz Sardà ^*^, E. Catellà, G. Palomar Centena, M. Gomez-Plaza, A. Gonzalez-Nuñez

^*^HUGTiP, Surgical Pathology, Badalona, Spain

**Objective:** Mammary amyloidosis (MA) is very rare and its presentation as first manifestation of amyloidosis is even rarer. MA usually appears in patients with chronic amyloid-related diseases and often presents as a mass that is easily mistaken for carcinoma both mammographically and clinically. We describe a peculiar MA case presenting as microcalcifications.

**Method:** We present the case of a 66-year-old female with seronegative polyarthritis and a previous tumorectomy for right breast invasive duct carcinoma. A left breast mammogram showed a 3-cm area of microcalcifications (BI-RADS 4B). A core biopsy and a subsequent tumorectomy were carried out.

**Results:** Both the core biopsy and surgical specimen showed amyloid deposits around ducts, lobules and blood vessels, as well as multiple microcalcifications. Congo red stain positivity, apple-green birefringence and AA protein immunoreactivity were shown by the deposits.

**Conclusion:** MA, a deft clinical and mammographic mimicker of breast carcinoma, usually appears as a mass following amyloid deposition in other organs. Nevertheless, as illustrated by this case, MA may be the first amyloidotic manifestation and may present as microcalcifications in the absence of a breast mass. These rare associations of MA should be kept in mind, particularly when examining breast lesions of patients with amyloid-related diseases.

**PS-17-086**

**Correlation of androgen receptor expression in histologic and molecular subtypes and clinicopathologic parameters of breast carcinoma**

I. A. Park ^*^, H. W. Baek, H.-C. Lee

^*^Seoul National University, Dept. of Pathology, Republic of Korea

**Objective:** To provide a clue in discovering effective targeted therapy for breast carcinoma patients with limited therapeutic options, we studied AR expression in breast carcinoma.

**Method:** We performed an immunohistochemical study using tissue microarrays in 377 cases of invasive carcinoma. We classified cases into “high AR” and “low AR” according to positivity equal to or above 10 % or below, and compared according to histologic and molecular subtypes and clinicopathologic parameters. Chi-square method was used for analysis.

**Results:** A total of 249 cases (66.0 %) were high AR (+). On histologic type, a total of 219 cases (67 %) of IDC-NOS were high AR (+). Invasive lobular carcinoma shows high AR (+) in 81.8 %. Metaplastic and medullary type shows high AR (+) in 16.7 and 0 % in each. In molecular subtypes, 87.2 %, 83.0 %, 47.4 % and 21.0 % of luminal A, luminal B, HER2 and triple negative type were high AR(+). High AR cases were statistically relevant with low nuclear grade & histologic grade, hormone-receptors (ER, PR) positive, p53 negative (<50 %), bcl-2 positive and ki-67<14 %, and not significant with T, N stage or HER2 status.

**Conclusion:** The finding of High AR expression was not relevant with HER2 status may suggest some patients may have benefit from AR target therapy.

**PS-17-087**

**Clinical relevance of the reappraisal of negative hormone receptor expression in breast cancer patients diagnosed in the 90’s**

A. E. Pinto^*^, F. Areia, M. Ferreira, T. Pereira, P. Cardoso, M. Aparício, G. L. Silva, S. André

^*^IPO Lisboa, Serviço de Anatomia Patológica, Portugal

**Objective:** Accurate assessment of estrogen (ER) and progesterone (PR) receptors is critical in predicting the response to endocrine therapies in breast cancer. We re-evaluated, on the same tumour material, ER and PR immunohistochemical analyses (IHC) performed in the 90’s to assess their clinical impact.

**Method:** From a series of 360 patients with breast carcinoma, we re-analysed cases previously considered negative (*n* = 164), i.e., ER-/PR- (*n* = 95), ER+/PR- (*n* = 63) and ER-/PR+ (*n* = 6), and 16 of 196 ER+/PR+ with unfavourable outcome. The concordance between IHC performed in 90’s and the current one (BenchMark ULTRA) was assessed by McNemar’s test. Disease-free (DFS) and overall survival (OS) were estimated by Kaplan-Meier method.

**Results:** From 101 ER- and 158 PR- cases, 38 (37.6 %) and 58 (36.7 %) became positive, increasing ER and PR expression from 71.9 % to 82.5 % and from 56.1 % to 72.2 % (*P* < 0.001), respectively. All 16 ER+/PR+ cases maintained their co-positivity. All ER-/PR+ tumours changed to ER positive. Kaplan-Meier curves showed significant differences related to DFS and OS for PR, either in the whole series or in the subset submitted to hormonal treatment. The subgroup with ER+/PR- tumours exhibited the worst prognosis.

**Conclusion:** IHC technical evolution improved the clinical usefulness of ER/PR assessment by decreasing false-negative results.

**PS-17-088**

**The expression of trimethylation at lysine 27 of histone H3 is correlated with less aggressive phenotype of invasive breast carcinomas**

C. Magkou ^*^, E. Souka, I. Giannopoulou, I. Theohari, K. Tzelepis, A. Keramopoulos, L. Nakopoulou

^*^Evaggelismos General Hospital, Pathology, Cholargos, Athens, Greece

**Objective:** The tri-methylation at lysine 27 of Histone H3 (H3K27me3), a core protein of chromatin, is associated with repression of gene transcription. Contradictory associations with outcome of patients with various carcinomas have been reported. Our aim was to investigate the prognostic impact of H3K27me3 in a series of breast cancer specimens.

**Method:** Immunohistochemistry was applied on 285 paraffin sections with invasive breast cancer for the detection of H3K27me3. Univariate and multivariate statistical analyses were performed for the correlation with established clinicopathologic parameters, the molecular subtypes of breast cancer (luminal, triple negative, Her-2), molecular markers (Ki-67, p53), as well as overall and disease free- survival.

**Results:** Nuclear expression of H3K27me3 was observed in 134 of 285 cases (47 %). Positive immunoreactivity was inversely correlated with tumor size (*p* = 0.012), nuclear grade (*p* = 0.003), stage of disease (*p* = 0.003), Ki-67 (*p* = 0.008) and p53 (*p* < 0.001). Patients with positive H3K27me3 demonstrated longer overall and disease-free survival time (*p* = 0.015 and *p* = 0.031, respectively). There was no significant correlation with the molecular subtypes of breast cancer (*p* = 0.278).

**Conclusion:** According to our results, the expression of H3K27me3 seems to be a favorable prognostic marker in breast cancer through its correlation with tumor size, nuclear grade, stage of disease, overall and disease-free survival.

**PS-17-089**

**Microglandular adenosis as a precursor of invasive carcinoma: An under-recognised entity**

A. Patil ^*^, A. Khan, S. Desai, T. Shet

^*^Tata Memorial Hospital, Pathology, Mumbai, India

**Objective:** Microglandular adenosis (MGA), a rare proliferative breast lesion, shows significant association with atypical MGA (AMGA) and invasive carcinoma (IDC). Previously considered as benign lesion, recent evidence points to clonal nature of MGA, suggesting its role as a precursor of IDC. We studied clinicopathological features of IDC developing in MGA.

**Method:** Histopathological features of four cases of IDC with MGA, retrieved from pathology database (2005–2012), were studied. Clinical data was available in electronic medical records for two cases treated at our institute, while others were referral cases.

**Results:** Of four cases (age 34–60 years), tumour size was known in two in-house cases (3.9, 5 cm). IDC with MGA was identified in both, with one case showing transition from MGA to AMGA to DCIS and IDC. Two referral cases showed predominantly IDC with focal MGA pattern. Tumour grades observed were I (1), II (2) and III (1). All the four tumours were triple negative, expressing S-100. Margins were revised in both in-house cases, with revised margin free of tumor in one, while other showed residual carcinoma in revised margin, with completion mastectomy showing residual MGA.

**Conclusion:** MGA is a rare precursor lesion of IDC that remains under-recognised and warrants a complete excision.

**PS-17-090**

**Male mammary Paget disease: Ten cases from a single institute**

X. Duan ^*^, A. Gullett, N. Sneige, E. Resetkova, C. Ersahin, Y. Wu, I. Bedrosian, C. Albarracin

^*^Loyola University Med. Ctr, Pathology, Maywood, USA

**Objective:** Examining the clinicopathologic features of this rare entity: male mammary Paget disease (MPD).

**Method:** We identified 10 cases of male MPD in our database in a 25 year period and studied their clinical and histopathologic features.

**Results:** Age of these patients at presentation ranged from 50 to 90 years and all of them had underlying breast carcinomas of ductal origin including one bilateral. Sizes of invasive carcinoma (IDC) ranged from 0.25 to 4.3 cm. Six patients with IDC were treated with modified radical mastectomy or total mastectomy, followed by chemotherapy, radiation and/or hormonal therapy. Remaining two patients with DCIS and two with IDC were treated with modified radical mastectomy only. Follow-up time ranged from 44 to 156 months. Three patients with IDC had lymph node metastasis. Two of them died of disease at 66 and 84 months and one was alive with metastatic cancer at 56 months. Six patients without axillary lymph node involvement and one with isolated tumor cells were all alive without disease at their last follow-up (44 to 156 months).

**Conclusion:** Male MPD, similar to female MPD, is associated with underlying breast cancer and lymph node involvement is a main factor associated with worse prognosis.

**PS-17-092**

**Comparison of pathological and prognostic factors between pure and mixed mucinous breast carcinomas**

G. Ayranci^*^, F. Gerin, I. Erbarut Seven, M. U. Ugurlu, H. Kaya

^*^Marmara University EAH, Dept. of Pathology, Istanbul, Turkey

**Objective:** Mucinous carcinoma is a rare subtype of breast carcinoma, which is known to be associated with favorable prognosis. The aim of this study is to compare the prognosis of pure and mixed subtypes and to correlate the prognosis with histopathological parameters.

**Method:** Retrospective review of 29 cases of mucinous carcinoma diagnosed between 2002 and 2012 was conducted in 993 surgical breast specimens. In this study we compared grade, stage, lymphovascular invasion, peritumoral DCIS between pure mucinous and mixed groups. We also evaluated estrogen receptors (ER), progesterone receptors (PR), HER-2 and Ki-67 proliferating index. The patient follow up was between 6 months to 11 years.

**Results:** In pure mucinous group, ER, PR, and HER-2 positivity was found to be 94 %, 88 %, and 11 % respectively. While in the mixed group, ER and PR were positive in 83 % and HER2 in 8 %. There was no recurrence, except for one case of mixed mucinous carcinoma with signet ring cells. The difference between two groups was not statistically significant, for both prognostic and pathologic factors. There was a positive correlation between the tumor grade and lymphovascular invasion.

**Conclusion:** Both groups had similar pathological and clinical characteristics. Signet ring cell component was revealed as a marker for poor prognosis.

**PS-17-093**

**Interlaboratory variability of Ki-67 labeling index in breast cancer tissue microarrays**

C. M. Focke ^*^, D. Gläser, K. Finsterbusch, H. Bürger, E. Korsching, K.-H. Berghäuser, B. Hinrichs, J. Packeisen, P. J. van Diest, T. Decker

^*^Dietrich Bonhoeffer Klinikum, Dept. of Pathology, Neubrandenburg, Germany

**Objective:** Our aim was to investigate interlaboratory variance of Ki67 labeling index (Ki67-LI) results using breast cancer tissue microarrays (TMAs) and centralised assessment.

**Method:** Six pathology laboratories (5 German and one Dutch) performed Ki67 staining of a TMA slide according to its routine in house protocol. 34 samples per lab were centrally analysed. The Ki67-LI was calculated after counting first all tumour cells and subsequently all Ki67 positive tumour cells of each sample regardless of staining intensity. For each tissue sample we evaluated the maximum variance of Ki67-LIs between different labs. Another 20 labs will participate in 2013.

**Results:** Maximum variances of Ki67-LIs between the labs were: 1–5 % in 7 (20,6 %) samples, 6–10 % in 4 (11,8 %), 11–15 % in 4 (14,7 %), 16–20 % in 2 (5,9 %), 21–25 % in 7 (20,6 %), 26–30 % in 4 (11,8 %), >30 % in 3 (8,8 %), >50 % in 1 (2,9 %), and >70 % in 1 (2,9 %) samples, respectively. Thus, in 34 TMA specimens, 47 % of the results of the 6 labs differed in Ki67-LI more than 20 %.

**Conclusion:** In a setting strictly standardised in terms of preanalytic influences by using TMA and postanalytic variance by centralised quantification, Ki67-LI seems to be heavily influenced by laboratory-specific analytic variables.

**PS-17-094**

**Ki-67 labeling index is not suitable to reproduce mitotic count score groups of the Nottingham Grading System**

C. M. Focke ^*^, D. Gläser, K. Finsterbusch, T. Decker

^*^Dietrich Bonhoeffer Klinikum, Dept. of Pathology, Neubrandenburg, Germany

**Objective:** We examined whether the three mitotic count scores (MCScores) of the Nottingham Grading System (NGS) can be reproduced by Ki67-Labeling Index (Ki67-LI).

**Method:** MCScores of 421 breast cancers (76 G1, 177 G2, and 168 G3) were determined according to the NGS at 10 high-power fields (1.59 mm2) in the tumor periphery. Ki67-LI (MIB1 antibody) was quantified independently of staining intensity using three protocols: 1) 100 tumor cells in “hotspot” (Ki67100); 2) 1020 tumor cells in three HPF in “hotspot” and tumor periphery (Ki671020periphery); 3) 1020 tumor cells in three HPF in “hotspot”, “coldspot” and an intermediate area (Ki671020spectrum).

**Results:** Of 421 tumors 160/421 (38 %) showed MCscore 1, 119/421 (28 %) MCscore 2, and 14/421 (34 %) MC-score 3. They came out with following median Ki67-LIs: MCscore 1: 22 SD14.1 (Ki67100); 12.5 SD17.5 (Ki671020 periphery); 9.2 SD7.8 (Ki671020spectrum), MC-score 2: 34 SD13.9 (Ki671020spectrum); 22.7 SD12.7 (Ki671020periphery); 16.5 SD9.8 (Ki671020spectrum), MC-score 3: 52 SD23.3 (Ki67100); 41 SD24.2 (Ki671020periphery); 32.7 SD19.6 (Ki671020spectrum).

**Conclusion:** Independent of the method employed Ki-67-LI did not allow a selective discrimination of the three NGS MCScores. Therefore, Ki67-LI seems not to be a substitute for standardised mitotic counting in grading of breast cancer.

**PS-17-095**

**Mitotic index and Ki-67 index: Are they correlated?**

R. Hamrouni^*^, K. Mrad, L. Charfi, R. Dhouib, N. Boujelbene, R. Doghri, S. Sassi, I. Abbes, M. Driss, K. Ben Romdhane

^*^Salah Azaiez Hospital, Pathology, Tunis, Tunisia

**Objective:** Both mitotic index (MI) and KI67 index (KI67I) are prognostic factors in breast cancer. The Aim of the study is To compare the results of MI and KI67I

**Method:** We compared the value of MI and KI67Ia in a series of 100 cases of breast cancers. Mitotic index was defined as low (<5mitoses/10highpower fields(×400) = 10HPF), moderate (5–10/10HPF) and High (>10/10HPF). Ki67 (novocastra, 1/50, DAB) index was estimated in 3 hot spots (×400). We combined the results in the following categories: Low MI and: KI67I = 0–20 (A1), KI67I = 21–30 (A2), KI67I = 31–50 (A3), KI67I = 51–1OO (A4); moderate MI and: KI67I = 0–20 (B1), KI67I = 21–50 (B2), KI67I = 51–1OO (B3); high MI and: KI67I = 0–20 (C1), KI67I = 21–50 (C2), KI67I = 51–1OO (C3).

**Results:** The different categories was distributed as follows: A1 : 24; A2 : 9; A3:5; A4:13; B1:2, B2:8; B3:6; C1:3; C2:3 and C3:24.

**Conclusion:** Results could be influenced by observer interpretation and technical quality. As expected, the more coherent categories were the most represented (A1 and C3). Ki67index could be high with low MI (category C4), on the contrary, low Ki67 with elevated MI is infrequent (B1, C1).

**PS-17-096**

**The effect of neoadjuvant chemotherapy on hormone receptor and HER2 profile of primary breast carcinoma**

P. Xirou ^*^, N. Vladika, S. Barbanis, D. Gerasimidou, B. Christoforidou, R. Iosifidou, E. Lalla, F. Patakiouta

^*^Theagenion Cancer Hospital, Pathology, Thessaloniki, Greece

**Objective:** To compare immunohistochemical expression of estrogen and progesterone receptors and HER2 status of primary breast carcinoma before and after neoadjuvant chemotherapy.

**Method:** A total of 90 female patients with primary breast carcinoma were analyzed retrospectively. Patients received neoadjuvant chemotherapy after needle core biopsy and underwent subsequent surgery. Estrogen and progesterone receptors (ER, PR) were assessed immunohistochemically, whereas HER2 status was evaluated by IHC and, in case of equivocal result, by SISH.

**Results:** Of the 90 cases, 64 (71,1 %) were ER positive and 47 (52,2 %) were PR positive pre-treatment. In 2 patients (2,2 %) the ER profile changed after treatment; both cases switched to a negative status. The PR profile changed post-treatment in 10 patients (11,1 %); 5 cases switched to a negative and 5 to a positive status. In 5 patients (5,5 %) there was a change in HER2 profile after treatment; 3 cases changed to a negative and 2 to a positive status.

**Conclusion:** In the present study there were no significant differences in ER, RR and HER2 profiles of breast carcinoma following neoadjuvant chemotherapy. Due to the conflicting reports in the literature, further studies are needed in order to evaluate the incidence and the prognostic value of the above changes.

**PS-17-097**

**Granular cell tumour of the breast**

I. Exposito Afonso ^*^, R. Méndez Medina, M. d. Carmen Martín Corriente, L. Martínez Blanco, A. Vega Falcón, S. García Hernández, A. Brito García, M. D. Ravina Cabrera, C. Manzano Sanz, C. García Castro, A. Martín Herrera

^*^La Laguna (Tenerife), Spain

**Objective:** Granular cell tumours are uncommon and can arise nearly anywhere in the body, having been described for the first time in the tongue in 1926 by Abrikossoff. It is believed to have a neural origin in Schwann cells based on its positivity for S-100 protein and its ultrastructural similarities. It is uncommon in the breast, suggesting the recent literature that the prevalence is 6,7:1,000 cases of breast malignancies.

**Method:** We report the case of a 61 years old woman with an irregular 10.3 mm nodule of spiculated margins, located in the tail of left breast (upper outer quadrant) on ultrasound and NMR which suggested malignancy. FNA was carried out and stained with Pap. Lumpectomy was performed. The lesion was solid, yellow-whitish and measures 1 × 0.9 cm. HE, PAS and IHC for S100, CD68, calretinin and CKAE1/AE3 were made.

**Results:** An irregular breast lesion was studied, consisting of compact clusters of large cells and small granular cytoplasm generally with a central core, sometimes involving breast ducts and lobules. It showed positivity for S100, CD68 and calretinin and negativity for CKAE1/AE3. The diagnose was Granular cell tumour of the breast.

**Conclusion:** We present a rare case of granular cell tumor of the breast, with ultrasound and NMR features, misleading to a radiological diagnosis of malignancy.

**PS-17-099**

**WT1 immunoreactivity in mucinous breast carcinoma**

S. Barbanis^*^, N. Vladika, P. Xirou, B. Christoforidou, D. Gerasimidou, F. Pavlidou, E. Michalopoulou, G. Galaktidou, F. Patakiouta

^*^The agenion Cancer Hospital, Pathology, Thessaloniki, Greece

**Objective:** To access the extent of WT1 immunoreactivity in mucinous breast carcinomas, including pure and mixed subtypes.

**Method:** A total of 50 cases of mucinous breast carcinomas were examined for WT1 immunoexpression. Of these cases 28 were pure mucinous carcinomas and 22 were mixed. All cases were immunostained for WT1 (clone 6F-H2, Dako) on the Benchmark XT automated stainer. Only nuclear staining was scored similar to hormone receptors and the positive staining was subdivided into weak (score 11–50), moderate (score 51–199) and strong (score ≥ 200).

**Results:** Twenty nine (58 %) cases were positive. Nineteen (65.5 %) of them were pure (5 with strong positivity) and 10 (34.5 %) were mixed mucinous carcinomas (2 with strong positivity). In the mixed group similar degree of WT1 expression in the mucinous and non mucinous component was observed in most cases.

**Conclusion:** Our results show that WT1 expression is frequent in mucinous breast carcinomas (more common in pure forms) and are consistent with the recent literature. Moreover, a similar degree of WT1 expression in the non mucinous component of mixed mucinous breast carcinomas should be kept in mind in the differential diagnosis of an unknown primary, since WT1 is characteristic in carcinomas of mullerian origin, especially ovarian serous carcinoma.

**PS-17-100**

**Androgenic receptor as a complementary marker in the evaluation of breast carcinoma molecular subtypes**

I.-D. Caruntu ^*^, C. Popescu, R. Balan, S. E. Giusca, R. Avadanei, L. Lozneanu, M. Raica

^*^U.M.F., Morpho-functional Sciences, Iasi, Romania

**Objective:** The present study aimed to investigate differences in the androgen receptor (AR) expression in the molecular subtypes of breast carcinomas.

**Method:** The study included 42 cases of breast carcinomas that were initially investigated immunohistochemically (IHC) using a panel of antibodies for diagnostic tumor in compliance with the molecular classification and then by evaluating the AR immunoexpression according to a standardized working protocol.

**Results:** The assessment of AR profile, reflected by nuclear positivity revealed a positive expression in 32 cases and negative in 10 cases. From the point of view of IHC reaction intensity, it was predominantly moderate (17 cases), followed by low levels of staining (9 cases) and strong levels of staining (6 cases).

**Conclusion:** The identification of positive AR expression predominantly in the molecular subtype luminal A and B, somewhat parallel with the ER and PR expression, clearly contributes to broadening the role of hormonal dependence applicable to breast cancer. The heterogeneity of AR expression in HER2 molecular subtype and in triple-negative group suggests their differentiation potential in distinct subclasses.

**PS-17-101**

**Metaplastic breast carcinoma: Cruces University Hospital experience**

N. Cerda ^*^, A. Pérez, G. Muñiz, A. Corominas-Cishek, V. Caamaño, M. González, L. Andrés

^*^Cruces University Hospital, Anatomia Patologica, Barakaldo, Spain

**Objective:** Metaplastic carcinomas (MC) comprise a heterogeneous group of breast tumours characterized by differentiation of the neoplastic epithelium into squamous and/or mesenchymal-looking elements. They are frequently triple receptor-negative and tend to have a poor prognosis. The aim of this study was to report the clinicopathologic features of 19 MC treated in our center.

**Method:** The authors retrospectively analyzed the medical records and archival tissue sections of patients with MC diagnosed in Cruces University Hospital between 1990 and 2012.

**Results:** 19 MC cases were retrieved, with only 1 identified before 2000. All patients were women, average age 60.9 years, all palpable mass with a median size of 4.84 cm, located in the right breast (55.6 %) and external quadrants (77.8 %). High grade was seen in (78.9 %), mesenchymal differentiation (42.1 %) and squamous (36.8 %). 15 cases had lymph node dissection, 9 demonstrating nodal metastases. The most common metastatic organs were lung (21 %) and liver (21 %). Immunohistochemistry profile was negative for oestrogen receptors (94.4 %), progesterone receptors (89.5 %) and HER2 (84.2 %). 6 patients with metastatic disease (MD) have died and another one did due to recurrence. 10 patients are free of disease and 2 were lost to follow-up (one of them with MD).

**Conclusion:** MC variants appear to rarely overexpress HER2 and hormone receptors.

**PS-17-102**

**Neoadjuvant chemotherapy in breast cancer: Experience of a Brazilian cancer hospital with pathological standardized protocol analysing residual cancer burden**

I. Cunha ^*^, C. Toledo, M. Chaves, L. G. Lima, F. Soares

^*^Hospital A.C. Camargo, Dept. of Pathology, São Paulo, Brazil

**Objective:** Breast cancer (BC) residual neoplasm after neoadjuvant chemotherapy treatment has relationship with patient outcome and can be pathologically evaluated. The present study analyzed the results of the residual cancer burden (RCB) in a cancer center hospital.

**Method:** In AC Camargo Hospital, the breast surgical specimens were evaluated for the RCB by a standardized protocol based on Symmans et al. It gives a score of residual disease, graduated as pCR (absence), RCB-I (minimal), RCB-II (moderate) or RCB-III (extensive). Neoplasm immunophenotype was classified in the pre-chemotherapy biopsy.

**Results:** In 182 cases, the most common immunophenotype was Luminal B (47,3 %; *N* = 86/182). The pCR was more common in basal-like (43,3 %; *N* = 13/30), triple-negative (36,3 %; *N* = 4/11) and HER-2 (41,1 %; *N* = 7/17) groups, and RCB-II and III were more common in Luminal A (39,2 %; *N* = 33/84) and B without (38,3 %; *N* = 33/86) and with (71,4 %; *N* = 5/7) the expression of basal-like markers, respectively.

**Conclusion:** Individual and different clinical protocols of treatment can explain the differences obtained in the same immunophenotype group. The uneven groups (a massive prevalence of Luminal B group) contributed for the unexpected better response of the worst prognosis groups.

**PS-17-106**

**Silver-enhanced In Situ Hybridization (SISH) detection assay and Inform HER2 Dual ISH DNA probe cocktail assay (DSISH) for HER2 gene status determination in breast carcinoma: A new experience**

F. Patakiouta ^*^, V. Theodorou, M.-M. Koumpanaki, E. Triantafillidou, D. Minotakis, C. Fotiou, P. Papakotoulas, C. Andreadis, G. Sibilidis

^*^Theagenion Cancer Hopsital, Pathology, Thessaloniki, Greece

**Objective:** Determination of HER2 status in breast cancer is important in the clinical management of the patients and can be identified by a number of methods. In this study we compare the results of the SISH and DSISH technique used in our laboratory.

**Method:** 398 cases of invasive breast carcinoma, including 41 core biopsy specimens were analyzed by SISH technique whilst 136 cases, including 22 core biopsy specimens were processed by DSISH technique. All cases were previously characterized immunohistochemically (CB11) as equivocal on a protein level (HER2 2+). Evaluation was made by bright-field microscope using the automated Ventana Benchmark XT machine.

**Results:** Out of the 398 cases, HER2 gene was amplified in 82 cases (20.6 %) using SISH, whilst 14 cases (3.5 %) were characterized as equivocal. Out of the 136 cases studied using the DSISH technique HER2 gene was amplified in 31 cases (22.8 %).

**Conclusion:** There is a statistically significant difference in the results of the two techniques. DSISH appears to be more accurate, time and cost-efficient and easier to interpret compared to SISH. Furthermore, it eliminates equivocal cases, which optimizes patient treatment.

**PS-17-107**

**First phase of breast cancer screening: Insight into the screening unit of Aachen, Germany**

S. Westphal^*^, M. Müller, P. Keulers, B. Wein, R. Knüchel

^*^Uniklinikum Aachen, Dept. of Pathology, Germany

**Objective:** Starting in May 2005, nationwide breast cancer screening was implemented in Germany. Mammographic screening detects signs of breast cancer in primarily asymptomatic women aged 50–69 and performs biopsy and histologic diagnostics. End of 2007, mammographic screening was introduced in the region of Aachen.

**Method:** We analyzed 526 cases of biopsy and consecutive surgery specimen from 2010 to 2011 regarding radiological BI-RADS classification, pathological B-classification, diagnosis and grading of biopsy compared to tumour specimen and lesion size assessed by mammography and ultrasound.

**Results:** Younger women (50–55) are biopsied relatively more frequently (1.3:1) and show more benign lesions (1:1.3) compared to older women (1:3.5). Biopsy diagnosis is in most instances consistent with tumour biology of the surgery specimen. Variants of invasive breast carcinoma are found, such as lobular (5 %), mucinous (0.8 %), tubular (0.8 %) and mixed (0.6 %). Tumour size differs between radiology and histology especially in some cases of extensive lobular invasive carcinoma and DCIS. Mean tumour size of invasive breast cancer detected by screening is smaller in comparison to regular health care.

**Conclusion:** Consistent results of biopsy and surgery specimen show good performance of regional mammographic screening within two consecutive years and encourage further interdisciplinary effort including more detailed evaluation of larger cohorts.

**PS-17-109**

**Comparison of Ki-67 expression using five different clones in core biopsy samples of breast cancer patients undergoing neoadjuvant chemotherapy**

A. M. Szasz ^*^, J. Kulka, E. Agoston, I. Teleki, T. Krenacs, A.-M. Tokes, C. Riemenschnitter, R. Keszthelyi, G. Szekeres, H. Moch, Z. Varga

^*^Semmelweis University, 2nd Dept. of Pathology, Budapest, Hungary

**Objective:** To compare 5 Ki67 antibodies in breast cancer cases in the neoadjuvant setting to clarify which clone would predict pCR best

**Method:** TMAs were composed of 84 pre-treatment core-biopsies of neoadjuvant-treated breast cancer patients from 1998 to 2009, pathological response was classified according to the EWGBSP and CPS-EG system. Automated immunohistochemical staining for 5 clones of Ki67 (Ventana:30–9, Histopathology:B56, IOT: MIB1, DAKO:polyclonal, Histopathology:SP6) was performed. Digital slides were evaluated using a liear scale.

**Results:** There were 4 TR1a, 1 TR1b, 10 TR2a, 27 TR2b, 24 TR2c, 9 TR3 (9 unclassified) cases according to the EWGBSP system (*p* = 0.003), while 2 I, 19 II, 18 III, 12 IV and 5 V (28 unclassified) cases according to the CPS-EG system (*p* = 0.068). Fourty-five cases were available for further statistics. Each clone displayed tendency to predict overall survival (15 % threshold, p>0.225,for all). Higher expression of each clone was seen in pCR, lower scores were detected in major response, but higher scores were characteristic in minor response. No clones correlated with the EWGBSP (p>0.121,for all) or the CPS-EG (p>0.385,for all) system.

**Conclusion:** Different frequency of Ki67 positivity was observed with the 5 different clones, but none was superior to predict pCR.

**PS-17-110**

**Interval detected breast cancers are associated with tumor cells invading blood vessels**

T. A. Klingen ^*^, I. Stefansson, K. Collett, T. Aas, A. L. Abrahamsen, H. Aas, Y. Chen, L. Akslen

^*^Vestfold Hospital Trust, Pathology, Tønsberg, Norway

**Objective:** Breast cancer presentation during mammography screening intervals is associated with markers of aggressive tumors and adverse prognosis.

**Method:** Here, we compared the frequency of tumor cell invasion into blood vessels or lymphatic vasculature according to mode of presentation. We studied 437 carcinomas in two independent series from the population-based Norwegian Breast Cancer Screening Program. By immunohistochemical staining, tumor cells invading lymphatic or blood vessels were examined on sections of paraffin embedded tumor samples using D2-40 and CD31 antibodies, respectively.

**Results:** In Series I (*n* = 247), interval cases demonstrated both more frequent blood vessel invasion (OR = 6.9; *p* < 0.0001) and lymphatic involvement (OR = 3.1; *p* = 0.001) than screen-detected cases. In the size-matched Series II (*n* = 190), interval cases demonstrated more frequent blood vessel invasion (OR = 4.7; *p* = 0.001), whereas lymphatic invasion was not significantly different (OR = 1.8; *p* = 0.13).

**Conclusion:** Our findings indicate that blood vessel invasion by tumor cells, as a morphologic indicator of hematogenous spread, is strongly associated with interval breast cancer presentation.

**PS-17-111**

**Interobserver variability in evaluation Ki-67 index in invasive breast cancers**

K. Mrad ^*^, I. Charfi, R. Dhouib, R. Doghri, N. Boujelbene, K. Ben Romdhane

^*^Salah Azaiez Institute, Pathology, Tunis, Tunisia

**Objective:** To estimate interobserver variability of Ki67 evaluation in invasive breast cancer

**Method:** Ki67 index was evaluated in 100 cases of breast invasive carcinoma by two independent observers with the following intervalls: (A) 0–20 %, (B): 21–49 %, (C): 50–100 %. The value estimated by observer 1 (obs1) was compared to the value estimated by observer 2 (obs 2). We categorized results as follows: (A1) : Obs1: A, Obs2 : A; (A2) : obs1 : A, Obs2 : B; (B1): obs1:B, obs2:B; (B2) : obs1:B, obs2:C, (B3) : obs1:B, obs2:A; (C1): Obs1: C, Obs2 : C; (C2) : Obs1: C Obs2 : B.

**Results:** The results was distributed as follows: A1:24, A2:7; B1:29, B2:3, B3:3; C1:33, C2:1. We noticed that the index value estimated by observer 1 was generally inferior to that estimated by observer 2.

**Conclusion:** There was good concordance in the 3 categories as defined by A, B and C since A1, B1 and C1 represented together 76 % of all the cases. The discrepancy decreased as the index increased.

**PS-17-112**

**Histological aspects of lymph node metastases in multiple breast carcinomas with different morphology: A study of 806 consecutive cases**

M. Boros ^*^, A. Ilyes, A. Boila, C. Marian, C. Moldovan, C. Dobos, S. Stolnicu

^*^UMPh Targu Mures, Dept. of Pathology, Romania

**Objective:** Numerous studies reported a higher percentage of axillary lymph node metastases in multiple carcinomas comparing to unifocal ones. Our study aims to analyze the histological features of lymph node metastases in multiple tumors in which primary tumor foci differ morphologically. No other similar studies have been previously published in English literature.

**Method:** We reassessed slides from multifocal/multicentric breast carcinomas surgically treated in Targu Mures in which complete axillary lymph node dissection was performed. We selected only cases with multiple tumor foci having different morphology.

**Results:** 178 (22.08 %) of 806 cases had multifocal/multicentric breast cancer and of these, 22 (12.36 %) had foci with different morphology. 17 (77.27 %) out of the 22 cases had axillary lymph node metastases. In most of these cases (82.35 %) the metastases had the same histological features as the more “aggressive” tumor foci. In 6(35.29 %) cases, metastases had the features of the smallest tumor focus, as this was the one that metastasized.

**Conclusion:** The morphologic features of axillary lymph node metastases in multiple breast carcinomas resemble the tumor focus with the most aggressive histological type, which is not necessarily the largest focus. As such, we strongly emphasize the necessity of reporting and assessing each tumor focus in multifocal/multicentric breast carcinomas.

**PS-17-113**

**Encapsulated papillary carcinoma of the breast in a male patient**

M. C. Aydin ^*^, E. A. Saglam

^*^Hacettepe University, Dept. of Pathology, Ankara, Turkey

**Objective:** Tumors of the breast are uncommon in males. Encapsulated papillary carcinoma of the breast in a male patient is an rare tumour.

**Method:** Paraffin embedded sections were examined.

**Results:** We present a 72 year old man with a mass in his right breast. Ultrasonography showed a capsulated cyctic lesion 5 × 4 cm in diameter with a solid component. Gross examination revealed a cystic tumor 6 cm in diameter with a gray-white cut surface and solid- papillary texture. On microscopic examination a cyctic lesion harbouring a papillary tumor was identified. The papillary neoplasm was characterized by cores of sclerotic fibrovascular stroma covered by a population of monomorphic cuboidal or columnar epithelial cells with low grade atypia. SMA and CK5/6 immunohistochemical studies showed absence of sorrounding myoepithelial cells and hence the neoplasm was classified as an encapsulated papillary carcinoma. Estrogen receptor and progesterone receptor were positive, C-erbB2 was negative.

**Conclusion:** Encapsulated papillary carcinoma of the breast is a rare entity. Tumors of the breast are rare in males. Cyctis masess are usually associated with papillary lesions. This case once again reminds us that encapsulated papillary carcinoma should be in the differential diagnosis in male patients with cystic breast masses.

**PS-17-114**

**Characteristics of stroma in normal breast, in situ and invasive ductal carcinoma**

B. Andrejic ^*^, T. Ivkovic-Kapicl, S. Kneževic-Ušaj

^*^Medical Faculty Novi Sad, Dept. of Histology and Embryology, Serbia

**Objective:** Evidence imply that stroma actively participate in normal and pathological processes in breast. Our objective was assessment of morphological and immunohistochemical analysis of stroma in normal breast (N), in situ (DCIS) and invasive ductal carcinoma (IDC), in regard to clinicopatohological parameters.

**Method:** Normal breast tissue (*n* = 20), DCIS (*n* = 30) and IDC (*n* = 30) samples were stained with hematoxilyn-eosin, ki-67, PDGFRα, CD8, CD4, ER, PR and Her2. Clinicopathological data were taken from patients records.

**Results:** Normal tissue showed rare lymphocytes, stromal fibroblast proliferative index (PI) and PDGFRα-positivity <5 %. Irrespective of grade, DCIS stroma was infiltrated predominantly with CD4+ lymphocytes, PI ranged from 10 to 15 % and PDGFRα-positivity was detected in 27 %. IDC stromal PI increased by four times (20 %), followed by PDGFRα-positivity increase (39 %) and predominance of CD8+ lymphocytes over CD4+. Increase in fibroblast PI and PDGFRα-positivity in DCIS and IDC correlated with parameters of poor prognosis (high grade and extensive DCIS, and older age, larger diameter, pT3/T4, ER negative and Her2/neu positive tumors in IDC). Frequent CD8+ lymphocytes inversely correlated with PI and PDGFRα-positivity in IDC, indicating significant role of immunity.

**Conclusion:** Differences in stromal compartment can explain different progression and outcome in patients with breast carcinoma, and possibly provide new therapy targets.

**PS-17-115**

**Invasive carcinoma and ductal carcinoma coexisting with Paget disease of the nipple**

G. Emiroglu Buberal ^*^, K. Acar, L. Yeniay, M. Kapkaç, R. Yilmaz, O. Zekioglu, N. Özdemir

^*^Ege University, Dept. of Pathology, Izmir, Turkey

**Objective:** Paget disease of the nipple is characterized with malignant glandular epithelial cells within the squamous epithelium of the nipple. In this study it was evaluated invasive cancers or carcinoma in situ coexisting with Paget disease.

**Method:** Seventy one cases of Paget disease diagnosed with excisional biopsies and mastectomies between 2000 and 2012 were reevaluated.

**Results:** Ductal carcinoma in situ coexisting with the Paget disease was present in %32.4 of cases. Invasive ductal carcinoma was %22.5, both invasive ductal carcinoma and ductal carcinoma in situ was seen in %24 of cases. The other tumors that was seen with Paget disease were microinvasive carcinoma (%8.5), both invasive ductal and lobular carcinoma (%4.2), micropapillary carcinoma (%4.2), and mucinous carcinoma (%2.8). In one case (%1.4) there was no underlying carcinoma. Estrogen and progesterone receptors were expressed in %18.8 and %5.7 of cases respectively. C-erb-B2 immunoreactivity was present in %74.8 of cases.

**Conclusion:** Paget disease usually coexists with invasive carcinoma and carcinoma in situ. Paget disease without underlying carcinoma is rare, with a reported incidence of 1.4–13 of all cases. The results of this study were consistent with the literature.

**PS-17-116**

**Comparison of hormonal receptor expression and HER2 status between primary and metastatic breast cancer**

A. Alves ^*^, S. Ortiz, P. Luís, L. Correia

^*^Hospital de Santa Maria, Dept. de Anatomia Patologica, Lisbon, Portugal

**Objective:** In breast cancer, the different therapeutic options take into consideration prognostic and predictive markers such as estrogen (ER) and progesterone receptor (PR) expression and HER2 status. Currently hormone receptor (HR) and HER2 status determination in metastasis is only recommended when negative in primary tumor (PT) or unknown. Our objective was to compare HR and HER2 status between PT and metastasis.

**Method:** 251 metastasis, from which 102 had paired PT, were retrospectively studied. Tumor histological characteristics and data from the Portuguese regional oncological registry were obtained. Her2 status was assessed by immunohistochemistry (IHC) and/or CISH. HR status was evaluated by IHQ. Cytology specimens, “in situ” and bilateral cancer cases were excluded.

**Results:** Of the PT: 90,4 % were invasive carcinoma, NST; 68,3 % were HR positive and 25,5 % were HER2 positive. The most frequent metastatic locations were: liver (27 %), bone (25 %), lung (23 %) and skin (7,5 %). For paired metastasis, HER2 status was altered in 7 % (3 positive to negative, *n* = 59), ER in 24,64 % (13 positive to negative, *n* = 69) and RP in 40,9 % (18 positive to negative, *n* = 66).

**Conclusion:** As described in other papers, change in HR and HER2 status between PT and metastasis was observed. This can have implications in treatment options for metastatic breast cancer.

**PS-17-117**

**Growth Hormone-releasing Hormone (GHRH) receptor in breast cancers: An immunohistochemistry study revealing its presence in apocrine epithelium and carcinomas**

B. P. Kovari ^*^, G. Cserni, Z. Kahán, O. Rusz

^*^University of Szeged, Dept. of Pathology, Hungary

**Objective:** Growth hormone-releasing hormone (GHRH) and its receptors (GHRH-R) have been implied in carcinogenesis acting through auto-/paracrine mechanisms. Little is known about the distribution of GHRH-R in breast cancers. In this pilot study, GHRH-R expression was correlated with different classes of breast cancers, as no systematic evaluation of such tumors has been performed to date.

**Method:** 64 small primary breast carcinomas were retrospectively evaluated for GHRH-R expression by immunohistochemistry using a polyclonal antibody and a cut-off value of 10 % staining.**Results:** GHRH-R positivity was detected in 62 % of all the cases, 23/26 (88 %) of invasive lobular carcinoma and 19/37 (51 %) of ductal carcinomas (IDC). A higher positive proportion was seen in grade 2 tumors (90 %), in contrast to those with grade 1 (40 %) or grade 3 (48 %). Apocrine epithelium, all 5 apocrine carcinomas and 7/8 (88 %) IDCs with casting-type calcifications on the mammogram showed positivity for the GHRH-R.

**Conclusion:** These preliminary results suggest a greater than average GHRH-R expression in lobular carcinomas, and seemingly characteristic positivity of apocrine carcinomas and IDCs with casting-type calcifications. Whether these findings could indicate a potential role of GHRH-antagonists in targeted treatment of these types of breast cancer requires further study. (Supported byTÁMOP-4.2.2.A-11/1/KONV-2012-0)

**PS-17-118**

**Invasive Paget disease of the breast: A rare entity. Report of two cases and review of the literature**

J. Pardal ^*^, M. Pinto, J. Magalhães, I. Amendoeira

^*^Hospital São João, Dept. de Anatomia Patologica, Porto, Portugal

**Objective:** Mammary Paget disease is a rare entity first described in 1874 by James Paget. It is in most cases associated with an underlying mammary carcinoma (invasive carcinoma or ductal carcinoma in situ-DCIS). Very rarely epidermal Paget cells invade into the dermis–so called Invasive Paget Disease (InvPD). This was first reported in 2 cases in Rosen’s textbook and, since then, 11 more cases have been described. The clinical significance and management of InvMPD are unknown. Our aim is to call attention to this rare entity, discussing the criteria of invasion and prognosis.

**Method:** We describe two cases of InvMPD from our institution and compare their clinicopathological features with the other cases reported.

**Results:** Both patients were treated with radical mastectomy and sentinel lymph node evaluation. The depth of invasion was 1,5 and 1,9 mm. Both cases were associated with DCIS. In Case 1 there were lymph node metastases, not present in Case 2. The patient in Case 1 received chemotherapy and radiotherapy. At follow-up (4 and 13 months, respectively) there is no evidence of disease.

**Conclusion:** In summary we report two cases of InvMPD and compare them with the cases published recently in the literature.

**PS-17-119**

**A case of secretory carcinoma of the breast expressing gross cystic disease fluid protein-15**

M. Caldas ^*^, A. Polónia, J. Vieira, M. Teixeira, C. Leal

^*^Ist. Português de Oncologia, Dept. de Anatomia Patológica, Porto, Portugal

**Objective:** Secretory carcinoma of the breast (SC) is rare (<0,15 % of all breast cancers). Mammary analogue secretory carcinoma of the salivary glands (MASC) has similar histologic features and the same recurrent translocation t(12;15). SC has been reported to express S-100 protein and carcinoembryonic antigen (CEA) and to be negative for hormone receptors, Her-2 and gross cystic disease fluid protein-15 (GCDFP-15), a marker of apocrine differentiation. Nevertheless, focal positivity for GCDFP-15 has been reported in MASC. We herein report a case of SC expressing GCDFP-15.

**Method:** Case report

**Results:** 56-year old asymptomatic female, presenting with a lobulated nodule in the upper-outer quadrant, right breast, detected on screening mammography. Core needle biopsy: carcinoma with secretory features. Excisional biopsy: 15 mm SC with predominant microcystic pattern. Immunohistochemistry revealed staining for CEA, S-100 protein and estrogen receptor in 1–10 % of neoplastic cells. GCDFP-15 was strongly expressed in the cytoplasm of neoplastic cells and in the luminal secretions. No staining was observed for progesterone receptor or Her-2. The diagnosis was confirmed by interphase fluorescent in situ hybridization using an ETV6 break apart dual color probe, showing ETV6 gene rearrangement.

**Conclusion:** To our knowledge, this is the first report of GCDFP-15 expression in secretory carcinoma of the breast.

Tuesday, 3 September 2013, 09.30–10.30, Pavilion 2

**PS-18 Poster Session Cytopathology**

**PS-18-001**

**Endoscopic ultrasound guided fine needle aspiration in pancreatic lesions: A center experience**

J. Nogueira ^*^, P. Pinto Marques, M. J. Brito

^*^Hospital Garcia de Orta, Serviço Anatomia Patológica, Cête, Portugal

**Objective:** Endoscopic ultrasound guided fine needle aspiration (EUS-FNA) has increasingly been incorporated in the diagnostic and staging algorithms of benign and malignant diseases of the gastrointestinal tract and adjacent organs. This technique allows the possibility of obtaining cytological and/or histological samples. In cases where sampling of pancreatic masses is indicated, EUS-FNA is recommended as a first-line procedure. In this study the objective was to validate EUS-FNA in our center as a diagnostic and staging tool in pancreatic mass assessment.

**Method:** Between January 2008 and June 2012 in our center, 229 smear/biopsy core EUS-FNA samples were obtained from pancreatic masses. The clinical and imaging data were consulted and correlated with the pathology report.

**Results:** In our series, 187 samples provided a diagnosis (66,8 %): 38 % were positive and 28,8 % negative for malignancy, (with an 85 % concordance between the clinical data and the pathology report) and 14,8 % were classified as suspicious for malignancy. 18,3 % of the samples were non-diagnostic.

**Conclusion:** EUS-FNA at our center gives a valuable contribution for the diagnosis of pancreatic masses with great impact in patient management avoiding more invasive techniques and allowing further analysis with other techniques, such as immunocytochemistry and molecular biology.

**PS-18-002**

**Pancreatobilliary IPMN in both biliary and pancreatic ducts**

P. Lazari^*^, P. Zafiriadou, I. Serafetinidis, V. Samaras, G. Sgourakis, I. Karoumpalis, C. Salla, C. Barbatis

^*^Athens General Hospital, Dept. of Cytopathology, Greece

**Objective:** A pancreatobiliary type IPMN in both biliary and pancreatic ducts.

**Method:** A 73 year old woman presented in our hospital with a cystic lesion in the body of the pancreas. The patient had a history of left hepatectomy,8 months before,due to a probable cholangiocarcinoma. EUS revealed multiple small cysts (up to 5 mm) communicating with the main pancreatic duct, a feature suggestive of IPMN. From the EUS-FNA aspirated material conventional and liquid based slides were prepared stained with Papanicolaou, but also cell block preparations were made for both H+E and immunohistochermical studies.

**Results:** Architecture: trabecular,papillary branching clusters and cribriform glandular pattern on the cell block. Cytomorphology: no cytoplasmic mucin, sometimes oncocytic features and intranuclear inclusions. Although the cytomorphologic and EUS features were suggestive of IPMN, immunohistochemical investigation (MUC1, MUC5AC, MUC2) was performed to identify the covering epithelium as pancreatobiliary, oncocytic or tubulopapillary type (excluding only the last one). Partial pancreatectomy was performed and pancreatobiliary type IPMN was confirmed. A retrospective study of the hepatectomy has also proved a well differentiated cholangiocarcinoma on the grounds of a pancreatobiliary IPMN of the intrahepatic bile ducts.

**Conclusion:** Concomitant IPMNs of the pancreatic and bile ducts can occur and may be covered by the same epithelial type.

**PS-18-003**

**Endoscopic ultrasound guided fine needle aspiration cytology: Contribution in malignancy staging**

J. Nogueira ^*^, D. Gonçalves, P. Marques, M. J. Brito

^*^Hospital Garcia de Orta, Serviço Anatomia Patológica, Cête, Portugal

**Objective:** Endoscopic ultrasound guided fine needle aspiration (EUS-FNA) has become an increasingly requested technique being incorporated in the diagnostic and staging algorithms of benign and malignant diseases of the gastrointestinal tract and adjacent organs. With the possibility of obtaining cytological and/or histological samples it allows an objective and definitive diagnosis. The objective was to analyze the usefulness of EUS-FNA in malignancy staging in our center.

**Method:** From 431 EUS-FNA performed between January 2008 and June 2012, 121 aspirates were reviewed using the clinico-imagiological data of the patients and their final cytology reports. The samples were obtained from different locations such as lymph node (*n* = 76), pancreas (*n* = 16), liver (*n* = 9), adrenal gland (*n* = 5), stomach (*n* = 2), small bowel (*n* = 3), large bowel (*n* = 1), peritoneum/retroperitoneum (*n* = 5) and mediastinum (*n* = 4).

**Results:** In 86 % of the aspirates a definitive diagnosis was provided (negative or positive for malignancy), therefore contributing to the staging of malignant diseases. 8 % were considered suspicious for malignancy and only 6 % were insufficient for diagnosis.

**Conclusion:** EUS-FNA at our center has shown to be a powerful method for the diagnosis of metastatic disease allowing staging of the tumors with great impact in patient management avoiding more invasive techniques.

**PS-18-004**

**Endoscopic ultrasound-guided fine needle aspiration (EUS-FNA) of mediastinal and abdominal lymph nodes: A useful technique in daily practice**

D. Gonçalves ^*^, J. Nogueira, P. Pinto-Marques, M. J. Brito

^*^Hospital Garcia de Orta, Serviço de Anatomia Patológica, Almada, Portugal

**Objective:** EUS-FNA is a safe and accurate method of sampling mediastinal and abdominal lymph nodes. In this study we analyzed the results of EUS-FNA of lymph nodes performed at our hospital between 2008 and 2012 (5 years).

**Method:** In this period 119 EUS-FNA of mediastinal and abdominal lymph nodes were performed in 112 patients. Patient demographics, indications for the procedure and final cytologic diagnoses were recorded.

**Results:** The indications included diagnostic purposes (54 %) and cancer staging (46 %), mainly for gastric (14 %), lung (10 %) and pancreatic (8 %) primary tumours. The locations of the lymph nodes were mediastinal in 41 % and abdominal in 59 % of the cases. In 109/119 (92 %) of cases, adequate specimens could be obtained. The final cytologic diagnoses were metastatic disease (40 %), granulomatous disease (4 %), lymphoma (2 %) and benign/reactive lymph nodes (54 %).

**Conclusion:** EUS-FNA has a high diagnostic yield in the case of mediastinal and abdominal lymphadenopathy. For cancer staging, it has major implications in the management of patients, allowing more accurate selection of patients for immediate surgery, neoadjuvant therapy or palliative treatment.

**PS-18-006**

**An 8-year retrospective survey of sputum cytology**

D. Sakonlaya ^*^, L. Yanagihara

^*^Thammasat University, Faculty of Medicine, Dept. of Pathology, Pathumthani, Thailand

**Objective:** To survey cytological diagnoses, quality, and clinical resources of sputum cytology specimens at Thammasat University Hospital.

**Method:** An 8-year retrospective study of sputum cytology specimens from Year 2004–2011 was performed. Adequacy of specimens, cytological diagnoses and clinical resources of specimens were reviewed from the request forms and cytology reports.

**Results:** All 119 sputum cytology specimens obtained in the 8-year period were reviewed. Cytological diagnoses were: malignancy (3 %), cytological atypia (5 %), benign (64 %), and unsatisfactory sample (28 %). All malignancy cases were adenocarcinomas. 94 % of clinical resources of sputum specimens were non-respiratory physicians and the remaining 6 % of cases were received from respiratory physicians. All cases of malignancy and cytological atypia were obtained from non-respiratory physicians. In the group of unsatisfactory samples, resources of most cases (94 %) were non-respiratory physicians. Among these, there were two specimens obtained by endotracheal tube suction by non-respiratory physicians.

**Conclusion:** Diagnostic rate of malignancy by sputum cytology in this study was 3 %. Unsatisfactory rate was 28 %. Most common clinical resources of specimens were non-respiratory physicians.

**PS-18-007**

**Diagnostic accuracy of fine-needle aspiration cytology in small pulmonary nodules with semi-solid or ground glass radiologic pattern**

R. L. Palhua Flores ^*^, M. Alberola, C. Iglesias, C. Dinarès, O. Persiva, E. Pallisa, A. Navarro, M. Aizpurua, S. Ramon y Cajal, N. Tallada

^*^Vall d’Hebron Hospital, Pathology, Barcelona, Spain

**Objective:** Fine-needle aspiration cytology (FNAC) has proven to have high diagnostic yield in relatively large solid nodules. Our purpose is to evaluate the diagnostic accuracy when nodules are small and with semi solid or ground glass radiologic pattern.

**Method:** A total of 24 patients (ages,57–90; men/women:16/8) underwent CT-guided percutaneous FNAC. Nodules measured between 11 and 39 mm all of them with semi solid or ground glass radiologic pattern. Diagnostic accuracy was determined by biopsy in the resected specimen.

**Results:** A total of 20(83.3 %) cytologies were adenocarcinoma with a good correlation with biopsy (sensitivity: 80 %, PPV:100 %). In Situ adenocarcinoma was found in 6 biopsies, 3 of them with positive cytology (sensitivity:50 %, PPV:100 %). There were 4 false-negative cytologies, 3 of them In Situ subtype. They were reexamined and reclassified as well differentiated adenocarcinoma (cytological features observed includes columnar-cuboidal shape, monolayer arrangement, fine chromatin structure and prominent nucleoli). Correlation for radiology revealed high sensitivity(100 %), all with malignancy diagnosis (4 of them only cytological).

**Conclusion:** FNAC is an accurate method of establishing diagnosis for these nodules whose radiological features suggest early cancer. FNAC is a good diagnostic method even in In Situ adenocarcinomas and give the possibility to better treatment options with a potential for cure.

**PS-18-008**

**Correlation of bronchial brushing cytology with bronchial biopsy in diagnosis of lung cancer**

I. Jelicic ^*^, D. Tegeltija, A. Lovrenski, M. Panjkovic, Z. Eri

^*^General Hospital of Vrbas, Dept. of Pathology, Novi Sad, Serbia

**Objective:** To evaluate bronchial brush cytology with histology in our case study.

**Method:** Our study inculuded 727 patients during 2 years who underwent brush and forceps biopsy through a fibreoptic bronchoscope.

**Results:** Out of 727 patients the mean age was 60.5 years and the male:female ratio was 2,4:1. The 304 were found to be malignat and 410 were inflammatory lesions. Only brush cytology was positive in 24 patients and only bronchial biopsy in 144. Cytohistology correlation was found in 136 case of malignacy (and 416 of non malignant lesions). Different findings of brush cytology and bronchial biopsy were found in 13 cases which showed other or more specific histologic type of tumor. The most common histological type was adenocarcinoma (45,40 %), followed by squamous cell carcinoma (37,5 %), small cell carcinoma (8,88 %), large cell carcinoma (2,63 %), non small cell carcinoma (2,63 %) and other histological type (2,96 %). The bronchial brush cytology showed sensitivity of 49 %, specificity of 94,5 %. Pre-test probability was 39 %. Positive and negative predictive value were 85 % and 74 % respectively.

**Conclusion:** Bronchial biopsy has higher detection rate than brush cytology in this study, but combination of these methods gives us much more information when we suspect on malignancy.

**PS-18-009**

**Basaloid salivary gland tumors and FNA: A challenge diagnostic? Our institution experience (2000–2013)**

A. Navarro ^*^, R. Campos, M. Ochoa, C. Iglesias, C. Dinarès, M. Aizpurua, R. Palhua, S. Ramón y Cajal, N. Tallada, M. Alberola

^*^Vall d’Hebron Hospital, Pathology, Barcelona, Germany

**Objective:** Basal cell tumors (BCT) of the salivary gland are among the most diagnostic challenge areas of salivary gland FNA cytopathology. The primary tumors included are basal cell adenoma, basal cell carcinoma and the solid variant of adenoid cystic carcinoma. In addition, cellular pleomorphic adenoma can also exhibit basaloid features. The purpose of study was reviewed the FNA/biopsy correlation.

**Method:** In this study, 31 BCT in FNA with histologic correlation were reviewed.

**Results:** Correlation diagnostic FNA/biopsy: 15 concordant (48.3 %), 6 discordant (19.36 %) and 10 descriptive diagnoses (32.2 %). The discrepancy found was due to misinterpretation in 2/6 (33.33 %) and poor sampling in 4/6 (66.66 %). The diagnosis of malignancy/benignity was made in 26/31 cases(83.87 %). From these last cases, 16/26 cytology and biopsy were malignant, in 7 both benign, 1 benign cytology and malignant biopsy, and 2 malignant cytology and benign biopsy. The diagnosis was not conclusive in 5/31(16.12 %). The overall results are 94.1 % sensitivity, 77.7 % specificity, 88.8 % PPV, 87.5 % NPV.

**Conclusion:** In our experience, FNA allows the diagnosis of BCT with 94.1 % sensitivity and a 77.7 % specificity. Different distinctive morphologic patterns of each entity (type/distribution of basal membrane and architectural pattern) determine the diagnosis. Sometimes cytomorphological features only allow a descriptive diagnosis.

**PS-18-010**

**Nodal Langerhans cell histiocytosis presenting as an isolated enlarged lymph node: Report of a case diagnosed by FNA and review of the literature**

E. Tejerina González ^*^, F. Pérez Rodriguez, M. Miralles, E. Sanz

^*^HM Universitario Torrelodones, Anatomía Patológica, Madrid, Spain

**Objective:** To describe the cytologic findings of a case of nodal Langerhans cell histiocytosis diagnosed by FNAC and review previously reported cases.

**Method:** FNAC under ultrasound guidance was made in a 4-year old boy who presented with an enlarged and painful inguinal lymph node. After the cytologic diagnosis the lymph node was resected and examined histologically with hematoxylin-eosin and immunohistochemistry.

**Results:** Smears were polymorphous, highly cellular and consisted of medium-size histiocytic cells singly or in small, non-cohesive groups. Nuclei were irregularly round or oval, indented or lobulated with chromatin folding images. Binucleated forms were seen. The cytoplasms were well-defined, finely vacuolated or with small granules in some, with ocassional cytoplasmic processes. Atypical mitotic figures and nuclear pleomorphism were observed. The histiocytic cells were accompanied by many eosinophils,small lymphocytes, a few neutrophils and plasma cells, together with multinucleated giant cells. A scant amount of necrotic debris was identified in the background. The histologic and immunohistochemical examination confirmed the cytologic diagnosis.

**Conclusion:** The cytologic features of Langerhans cell histiocytosis seem to be characteristic enough as to achieve a diagnosis even in cases of unusual presentation. Other benign and malignant lymphadenopaties must be considered in the differential diagnosis of the rare cases limited to the lymph nodes.

**PS-18-011**

**Cyto-morphological features of blastic plasmacytoid dendritic cell neoplasm on fine needle aspiration and cerebrospinal fluid cytology: A review of 5 cases**

J. Ferreira ^*^, G. Gasparinho, R. Fonseca, S. André

^*^IPO Lisboa, Serviço de Anatomia Patológica, Portugal

**Objective:** Blastic plasmacytoid dendritic cell neoplasm (BPDCN) is a rare hematopoietic tumor, once thought to be derived from natural killer cells and now recognized as originating from precursors of plasmacytoid dendritic cells. It generally involves the skin and has an aggressive clinical course. Due to its highly malignant behavior, a fast and correct diagnosis of this condition is of utmost importance.

**Method:** Cytology specimens from five patients diagnosed with BPDCN were reviewed as well as their medical records.

**Results:** Two exfoliative cytology specimens (cerebrospinal fluid) and three fine-needle aspiration(FNA) specimens (two lymph nodes and one cutaneous) were reviewed. The cyto-morphological aspects were similar in all cases. The smears were hypercellular with a monotonous population of intermediate-sized cells, dispersed singly or arranged in loose aggregates. The cells had round to oval nuclei, with fine chromatin and prominent nucleoli; the cytoplasm was generally scant, without visible granules. All cases were also characterized by flow cytometry, which revealed expression of CD4, CD56 and CD123. FNA was used in 2 cases for primary diagnosis. Histological confirmation was available in all cases.

**Conclusion:** BPDCN is a highly malignant neoplasm with a poor outcome. Cytology, in association with flow cytometry, is a reliable method to establish the diagnosis.

**PS-18-012**

**MALT lymphoma of bronchus diagnosed by Fine Needle Aspiration (FNA): A case report**

H. D. Quiceno Arias ^*^, M. D. Lozano, J. L. Solórzano Redón, R. A. Carias Calix, T. Labiano, J. I. Echeveste

^*^Clinica Universidad de Navarra, Dept. de Anatomia Patologica, Pamplona, Spain

**Objective:** Extranodal marginal zone lymphoma of mucosa-asscociated lymphoid tissue (MALT lymphoma) is an extranodal lymphoma that comprises 7–8 % of all B-cell lymphomas. Most cases occur in adults with a median age of 61 and a slight female preponderance. The gastrointestinal (GI) tract is the most common site of MALT lymphoma, comprising 50 % of all cases and between 10 % and 19 % corresponds to bronchus associated lymphoid tissue (BALT).

**Method:** A 65 year old men with a personal history of hypertension, diabetes, dyslipidemia, gastroesophageal reflux and smoking. With 6 months of severe cough and dyspnea. Spirometry show a moderate ventilatory obstruction and the TC with generalized thickening of the walls of the trachea and the left and right main bronchus. Thickening is more significant at the level of the bifurcation of right upper lobe bronchus. video bronchoscopy was performed with pathologist in the room, cytologic smears were obtained and mucosal biopsies.

**Results:** The cytologic smears and mucosal biopsies showed bronchial epithelium with a preserved mucosal below an extensive lymphocytic infiltrate erasing all normal structures of the bronchial wall, lymphocytes are small destroying some bronchial glands. immunohistochemical study was performed where the cells described were positive for ALC, CD20 and Bcl2. CD5 and CD3 was positive only in population of nonneoplastic lymphocytes. cyclin D1, CD10 and Bcl6 were negatives. Molecular studies of the translocation t(14;18) of DNA extracted from cytologic material was not found.

**Conclusion:** 1. The diagnosis of lymphoma BALT can be done through Cytologic smears of transbronchial biopsies by videobronchoscopy, leaving invasive procedures in selected cases. 2. Immunohistochemical and molecular techniques in cytologic smears can prevent interventions with more morbidity and mortality.

**PS-18-013**

**Thyroid paraganglioma: Report of a case with fine needle aspiration biopsy diagnosis and histopathologic correlation**

S. Cetin^*^, G. Kir, M. Segmen Yilmaz, F. Gursoy

^*^Umraniye Research Hospital, Dept. of Pathology, Istanbul, Turkey

**Objective:** Thyroid paragangliomas are rare neuroendocrin tumors. Fine needle aspiration biopsy(FNAB) diagnosis is difficult and can be misdiagnosed as other types of thyroid diseases. This case appears to be the third on FNAB of primary thyroid paraganglioma.

**Method:** We report a case of a 66-year-old woman presenting with multinoduler guatr. Ultrasound guided FNAB was performed from the 3 cm diameter nodule in the superior portion of the right lobe of thyroid. The cytological smears contained single cells or loose clusters of round to ovoid cells with oval granular nuclei. Cell block material of the FNAB was stained immunohistochemically with thyroglobulin, calcitonin, cytokeratin(CK), thyroid transcription factor-1 (TTF-1), synaptophysin, chromogranin A, neuron-spesific enolase(NSE) and S-100.

**Results:** The immunohistochemical staining was positive for synaptophysin, chromogranin A, neuron-spesific enolase(NSE) and S-100 and negative for thyroglobulin, calcitonin, cytokeratin(CK) and thyroid transcription factor-1 (TTF-1). Follicular neoplasm, paraganglioma, parathyroid adenoma and other neuroendocrine neoplasms were considered in the differantial diagnosis. Total thyroidectomy was performed and final diagnosis of paraganglioma was made.

**Conclusion:** Paraganglioma is a rare neuroendocrine tumor of the thyroid and should be considered in the cytologic differantial diagnosis of the thyroid neuroendocrin tumors.

**PS-18-014**

**Analysis of 4490 thyroid fine needle aspiration cytology, reported according to the Bethesda System for Reporting Thyroid Cytopathology**

A. Borda ^*^, I. Pascanu, I. Budan, J. Balazs, C. Gliga, A. Fisus, S. Voidazan, N. Berger

^*^University of Medicine, Histology, Tirgu Mures, Romania

**Objective:** The aim of this retrospective study is to evaluate the distribution of thyroid lesions in fine-needle aspiration (FNA) specimens and to assess the accuracy of the method using cyto-histological correlations.

**Method:** 4490 FNA performed between 2007 and 2012 were analyzed. The Papanicolaou staining was used, and all samples were reviewed and/or reported according to the Bethesda System for Reporting Thyroid Cytopathology (BSRTC). The results of adequate samples were compared to the histological diagnoses, when surgery was performed. The sensitivity, specificity, positive predictive value (PPV), and negative predictive value (NPV) were calculated.

**Results:** Histology was available for 634 FNA cases. 437 (67.6 %) were benign lesions, 6 (1.1 %) were atypia of undetermined significance/follicular lesions of undetermined significance (AUS/FLUS), 120 (18.6 %) were follicular neoplasm/suspicious for follicular neoplasm (FN/SFN), 82 (12.7 %) were malignant/suspicious for malignancy, and 197 (4.38 %) were non-diagnostic. The sensitivity was 66.30 %, the specificity 96.48 %, the PPV was 82.7 % and the NPV was 88.1 % (*p* < 0.0001). Of 120 FN/SFN, 94 (78 %) were benign and 26 (22 %) were malignant lesions.

**Conclusion:** FNA is a useful tool in selecting malignant and benign thyroid lesions, and preventing unnecessary surgery. BSRTC is an excellent tool for classifying thyroid FNA, although the AUS/FLUS category is heterogeneous in terms of usage.

**PS-18-016**

**Diagnostic performance of fine needle aspiration in major salivary glands tumors: Comparative study of parotid and submandibular gland**

S. Cardoso ^*^, P. R. Faria, L. B. Muniz, J. V. Rezende, S. J. Silva, S. Moraes, A. M. Loyola

^*^Federal University of Uberlând, Pathology, School of Dentistry, Uberlândia, Brazil

**Objective:** To compare diagnostic performance of fine needle aspiration (FNA) of tumors in parotid and submandibular gland (SmG), in a retrospective study.

**Method:** Cytopathology reports obtained from FNA of 66 consecutive MSGT (45 parotid and 21 SmG) were compared to histopathological reports from respective resection samples. Diagnostic performance was calculated for identification of neoplastic lesions (NL) and malignant tumors (MT).

**Results:** Regarding evaluation of neoplastic nature, exams with non-informative results were more common in submandibular (29 %) than parotid (20 %) tumors, but it was very similar for the evaluation of malignancy (8 % and 9 %, respectively). The following performance was observed: A) NL in parotid: accuracy and positive predictive value (PPV) of 100 %, there was no false-negative result; B) NL in SmG: accuracy and PPV of 100 %, NPV of 50 %; C) MT in parotid: accuracy of 93 %, VPP of 97 %, NPV of 82 %; D) MT in SmG: accuracy, PPV and NPV of 100 %.

**Conclusion:** These results corroborate the usefulness of FNA in the diagnosis of salivary gland tumors. Non-informative result is a major problem. A result denying neoplasia in SmG must be viewed with greater caution. Financial support: CNPq, FAPEMIG, CAPES.

**PS-18-017**

**Cytologic identification of neuroendocrine differentiation of breast ductal adenocarcinoma**

P. Lazari^*^, G. Koutsonikas, Z. Kontogianni-Miller, M. Kefala, E. Anastasopoulou, E. Asimis, M. Vlachou, I. Nikas, P. Panagouli, C. Salla

^*^Athens General Hospital, Dept. of Cytopathology, Greece

**Objective:** Identification of neuroendocrine differentiation of breast ductal adenocarcinoma (invasive and in situ) from cytological specimens.

**Method:** A retrospective study was carried out in the Cytology Department of Athens General hospital during the period from January 2010 to December 2012. Eight patients with an age ranging between 59 and 89 years old underwent breast surgery (lumpectomy or mastectomy) with a final histological diagnosis of invasive ductal adenocarcinoma with neuroendocrine differentiation. The FNA smears of the above mentioned women were re-examined to identify morphologic neuroendocrine features. Chromogranin-A was also performed on destained conventional alcohol fixed smears.

**Results:** 3 cases had eccentric nuclei, 4 salt and pepper chromatin pattern and only 2 granular cytoplasm. 4 of them showed focal immunoreactivity for chromogranin-A.

**Conclusion:** The Cytopathologist should be aware of the probable neuroendocrine differentiation of breast ductal adenocarcinoma and search for the morphologic and immunohistochemical features, but also make the differential diagnosis from lymphoplasmacytoid lymphoma or plasmacytoma,that can form deposits in soft tissue and breast. Prognosis of breast adenocarcinomas with neuroendocrine differentiation is still a matter of debate.

**PS-18-018**

**Audit of urine cytology of the upper urinary tract**

P. Mikou ^*^, F. Malta, E.-A. Trigka, I. Leotsakos, I. Adamakis, K. Stravodimos, E. Patsouris

^*^Laiko Hospital, Dept. of Cytopathology, Athens, Greece

**Objective:** Urine cytology is used for the detection of urothelial cancer and tumour recurrence. In the upper urinary tract, cytologic morphologic evaluation has to overcome cellular alterations due to catheterization and is quite critical, as biopsy may be difficult to obtain. Our objective is to define cytology performance on this field in our laboratory.

**Method:** Laboratory database was used for the retrieval of cytology and corresponding histopathology reports in 2 years period. All samples were processed by routine and liquid based cytology technique. In cases without histology, clinical follow up data were gained from the files of the urology unit.

**Results:** Our study included 85 cytology reports corresponding to 64 different cases, from which 34 were negative, 17 were atypical and 11 were suggestive of urothelial carcinoma. In 2 cases the sample was inadequate. There were 29 corresponding pathology reports. Sensitivity of 73 %, specificity of 86 % and positive predictive value of 82 % were estimated.

**Conclusion:** Cytology of the upper urinary tract in our laboratory is within acceptable performance standards, thus a significant contributory method in monitoring urology patients.

**PS-18-019**

**Cytology of cerebrospinal fluid in unrecognized extracranial malignancies with neurology symptoms**

J. Duskova ^*^, I. Vítková, P. Fuccillo, P. Prokopova

^*^1st Institute of Pathology, Dept. of Cytopathology, Prague 2, Czech Republic

**Objective:** Known, but especially clinically silent extracranial malignancies can be accompanied with neurology symptoms suggestive of many differential diagnoses. Cerebrospinal fluid (CSF) cytology represents a morphology contribution to the solution & correct diagnosis. The CSF cytology can exhibit various findings:–many–a few–no neoplastic cells

**Method:** Three cases with unclear neurology symptoms & different CSF findings were investigated using both non-morphological and morphological tests.

**Results:** Case 1: Neoplastic pleocytosis in the CSF of woman 39 with immunocytochemistry employed resulted into the diagnosis of cryptogenic carcinoma–stomach primary subsequently identified. Case 2: Neoplastic oligocytosis in the CSF of man 56 was suggestive of possible malignancy; the melanoma of unknown primary location with brain micrometastases was confirmed at autopsy. Case 3: Non-neoplastic pleocytosis in a woman 59 accompanied for 9 months cryptogenic bronchogenic carcinoma diagnosed together with paraneoplastic encephalitis at autopsy.

**Conclusion:** CSF cytology contribution to the diagnosis is dependent not only on the amount of neoplastic cells available for analysis but also on the broad differential diagnostic thinking and employment of additional methods.

**PS-18-020**

**Intramuscular metastases as the initial presentation of non-small cell pulmonary carcinoma: Report of three cases diagnosed by fine needle aspiration cytology**

E. Tejerina González ^*^, A. López García, J. Martín López, R. Pérez Arangüena

^*^HM Universitario Torrelodones, Anatomía Patológica, Madrid, Spain

**Objective:** To describe three cases of non-suspected pulmonary carcinomas, non-small cell type, diagnosed by fine needle aspiration cytology (FNAC) of intramuscular masses as a sole manifestation.

**Method:** Three cases of intramuscular masses with a clinical and radiological diagnosis of sarcoma were evaluated by FNAC. Masses were located within the muscle of the upper portion of the thigh in two and inside the buttock muscles in the third. Immunocytochemical study was performed in two cases.

**Results:** In two cases smears were highly cellular and composed of neoplastic cells with high nuclear/cytoplasmic ratio, arranged singly or in tridimensional clusters in a necrotic background. Nuclei were irregularly round, hyperchromatic, with prominent nucleoli and chromatin foldings. The cytoplasms had low density and appeared occasionally vacuolated. The third case consisted of epithelial cells with large, irregular and hyperchromatic nuclei and dense cytoplasms variabily keratinized. Immunocytochemical study showed TTF1 positivity in two cases. A diagnosis of metastatic pulmonary carcinoma was rendered in all cases and subsequently confirmed clinical and histologically.

**Conclusion:** Intramuscular metastases of non-small cell pulmonary carcinoma are very unusual, moreover as its initial manifestation. Fine needle aspiration cytology is a useful tool in order to achieve a correct diagnosis, excluding the possibility of sarcoma in such cases.

**PS-18-021**

**Fine Needle Aspiration (FNA) diagnosis of metastatic intracranial tumors. Report of 3 cases**

P. Jiménez León ^*^, C. Delbene, C. Dinares, E. Martínez, S. Ramon y Cajal, N. Tallada Serra

^*^Vall d’Hebron Hospital, Pathology, Barcelona, Spain

**Objective:** Systemic metastases from intracranial tumors are infrequent and few reported cases have been diagnosed by FNA. We present 3 cases recorded in our Department over a period of 30 years.

**Method:** Case 1: 51 year-old woman diagnosed at 22 with malignant ependymoma, presenting liver metastasis assessed by FNA: cohesive plaques of ill-defined mid-sized cells with round-oval nuclei and occasional filiform cytoplasm. Immunohistochemistry on cell block: GFAP(+), EMA(+) and CK(−). Diagnosis was metastatic malignant ependymoma. Case 2: 58 year-old man diagnosed 7 years ago with atypical meningothelial meningioma. Pulmonary nodule (1.8 cm) was detected and oriented as hamartoma/metastasis. Smears showed a medium-large size population of atypical, frequently isolated cells with dense cytoplasm and intranuclear pseudoinclusions. Immunohistochemistry on smears: PR(+), TTF1(−). Diagnosis was metastatic anaplastic meningioma. Case 3: 23 year -old woman diagnosed with Glioblastoma Multiforme (GMF). 3 years later a pleuro-pulmonary mediastinal node is detected and assessed by FNA: abundant, atypical, medium-large sized cellularity with lysate, inconspicuous cytoplasm. Immunohistochemistry on smears showed PGFA(+), TTF1(−). Diagnosis was metastasis of high-grade tumor consistent with GMF, later confirmed by biopsy.

**Conclusion:** FNA is a quick and minimally invasive technique that is useful in the diagnosis of metastatic intracranial tumors, but knowledge of clinical history is critical.

**PS-18-022**

**An analytic study of the recommendations formulated on 311 cervico-vaginal smears as to dysplasia cancerous lesions diagnosed in The Dakar Grand Yoff General Hospital (An African experiment)**

C. Kammoun ^*^, D. Kwame, F. Pyrrhus, F. Sellami, C. Dial

^*^Dakar, Senegal

**Objective:** The authors propose a retrospective study on the therapeutic recommendations made by pathologists as to the dysplasic lesions of the cervix of the uterus on the detection cervical smears.

**Method:** The summaries of 3,417 smears were studied. They are taken according to the conventional method. On each woman two laminas are taken, an excocolite one and an endocolite one. The Bethesda classification is used for all the smears.

**Results:** On such sample of 3,417 women, 9,1 % showed all degrees of dysplasic lesions. The low-degree dysplasias are more frequent with 5,4 % of the. In 68 % of the cases a cytological surveillance instruction is indicated. The High-degree dysplasias are diagnosed in 0,9 % of the cases. Colposcopy is recommended in 87 % of such lesions. For the rest, cytological surveillance is recommended. The ASCUS are noticed with 2 % of the. Colposcopy and biopsy are recommended for 54 women (77.1 %). Epidermoid carcinoma and the AGCUS represent 0,4 of the cases. Biopsy is indicated in 77 % of the cases.

**Conclusion:** In our study, 70 % of the therapeutic indications have complied with the recommendations of Bethesda. In 30 % of cases, the therapeutic indication is not adequate or absent. It is important for pathologists to mention adequate therapeutic indication.

**PS-18-023**

**Cervista HPV test for cervical cancer screening: A comparative study**

L. Garrote ^*^, F. Alameda, C. Sousa, V. Alba, S. Mojal, M. Muset, B. Lloveras, B. Bellosillo, C. Saldanha, S. Serrano

^*^Hospital del Mar, Dept. of Pathology, Barcelona, Spain

**Objective:** The aim of the study is to investigate the Cervista HPV HR test for its application in the cervical screening. The study will be done following the recommendations of Meijer et al. guideline.

**Method:** For the study of HPV test sensitivity we included 64 samples of women with a biopsy CIN2 or more. For the study of HPV test specificity we incorporated 811 samples of women with cytology result of negative, ASCUS, LSIL o ASCH. The intra-laboratory and inter-laboratory reproducibility were determined by evaluation of 507 and 513 samples, respectively.

**Results:** Clinical sensitivity was 100 % for HC2 and 98.4 % for Cervista. Specificity was 86.4 % for HC2 and 85.2 % for Cervista. The agreement between HC2 and Cervista was 91.7 % with a kappa value of 0.743. Discordant results were 32 HC2+/Cervista- and 41 HC2-/Cervista+. The intra-laboratory and inter-laboratory reproducibility showed a kappa value of 0,886 and 0,907.

**Conclusion:** Our results demonstrated that Cervista has at least the same clinical benefit as HC2. Some laboratory benefits of Cervista are the internal control, the lower requirements of sample material and the autonomy time. For improve the screening methods, it is very important to promote the validation of new HPV tests.

**PS-18-024**

**Frequent and uncommon findings in Fine Needle Aspiration (FNA) of Warthin tumor**

R. Orellana Fernandez ^*^, L. M. Palacio Arteaga, N. Combalia Soriano, M. R. Bella Cueto, M. R. Escoda Giralt, C. Blazquez Maña, R. A. Posada Caez, M. M. Rey Ruhi

^*^Parc Taulí Sabadell H.U/UDIAT, Pathology, Spain

**Objective:** Identify the uncommon cytomorphological findings associated with Warthin tumor (WT).

**Method:** Between 2000 and 2012, in our institution were made 369 FNA of parotid gland lesions. We studied 45 specimens with WT diagnosis, in which there were cytologic correlation. The background elements of the smear (proteinaceous, mucoid, crystalloids, calcifications, corpora amylacea,…) and the cellular components (oncocytic cells and their morphology, lymphocytes, degenerated oncocytic cells, squamous cells, spindle cells, mast cells, histiocytes, osteoclastic type giant cells, papillary structures or fragments of fibrovascular tissue) were evaluated.

**Results:** The cytological diagnosis of the 45 cases was: WT (27), oncocytic cells tumor (2), cystic component (7), polymorph lymphoid cellularity (2), pleomorphic adenoma (1), and material insufficient for diagnosis (6). We confirmed that the majority of cases present with the characteristic features that allow the diagnosis of WT.

**Conclusion:** The term oncocytic tumor of the salivary gland includes several entities, such as WT. To differentiate them, a good diagnostic algorithm has to be made. The FNAs of WT do not represent a diagnostic difficulty when all the characteristic cytomorphological features are present. When the cellular components are observed isolated or associated to uncommon findings, the diagnosis can be difficult.

**PS-18-025**

**Evaluation of high-risk human papilloma virus prevelance in cervical sample with known cytology results utilizing cerrvista HPV HR test in high-risk patients from the Department of STI Control (DSC) Clinic**

S. S. Ahmed ^*^, G. S. Thoe, W. X. Ting, S. Minto, Jacqueline S. G. Hwang, S. A. Eng Chang, P. Sen

^*^Singapore, Singapore

**Objective:** Due to strong association with cervical cancer, 14 sexually transmitted HPV oncogenic genotypes are consider high-risk. Early detection of high-risk HPV may help in early intervention and clinical management by adjunctive screening with cervical cytology.

**Method:** Cervical smears of 255 women recruited from the Department of STI Control Clinic from April 2012 to November 2012 for cervical screen were evaluated and reported according to 2001 Bethesda System using ThinPrep 2000 processor. Using Genfind DNA Extraction Kit, DNA was extracted from 101 cases of ASCUS and above, incubated with Cervista HPV HR and evaluated for HR HPV. Cervista HPV-16/18 was performed on DNA samples positive for HPV HR.

**Results:** 60 (23.5 %) smears were positive for HPV HR, of these 33 (35.9 %) were negative for both HPV 16/18. For HPV 16 positive, 7 (7.6 %) were reported ASCUS, 5 (5.4 %) LSIL and 1 (1.1 %) HSIL. For HPV 18 positive, 3 (3.3 %) were reported LSIL. 3 (3.3 %) with both HPV16/18 positive were reported as LSIL. 4 (4.3 %) indeterminate results were reported ASCUS and LSIL.

**Conclusion:** Cervista HPV HR test enhances detection of high-risk HPV in ASCUS/LSIL. In conjunction with Cervista HPV HR test, ASCUS with high-risk HPV can be diagnosed early and aid in early clinical management.

**PS-18-027**

**Imaging system: Evaluation for cervical cancer screening in a university hospital**

H. Sevestre ^*^, N. Benerdjeb, J.-F. Ikoli, C. Attencourt, M. Sockeel

^*^CHU, Pathology, Amiens, France

**Objective:** To evaluate the adherence of pathologists to the Thinprep Imaging System (TIS), (Hologic) 18 months after its implementation.

**Method:** Six pathologists and four pathologist-in-training were asked to answer a questionnaire: number of cases per day, expectations from TIS (faster, better, archival, other), weekly range of slides, feeling on the TIS (not satisfied, poorly, moderately, very satisfied), difficulties met (fear of diagnostic error, waste of time, access to the microscope, other), the advantages of the TIS (gain of time, better diagnosis, archival, other).

**Results:** Four pathologists and four “Internes” read between 5 and 10 slides and one pathologist more than 10 slides per day. One pathologist never used TIS, complaining of waste of time. The felt advantages are: better diagnosis (56 %), archival (22 %), gain of time or other reason (11 %). Satisfaction is moderate for six, high for two and null for one. The main dis-satisfaction causes are: access to the TIS (78 %), waste of time (22 %). no image capture (3/4), lack of multi-station system (1/4).

**Conclusion:** In comparison with our previous six-month satisfaction study, we noticed a significant improvement of satisfaction. The main dis-satisfaction is the limited access to the TIS.

**PS-18-028**

**Validation of fixcytol, a liquid based cytology medium, for HPV genotyping using the papillocheck method (greiner bio-one)**

H. Sevestre ^*^, B. Karkouche, S. Tolleron

^*^CHU, Pathology, Amiens, France

**Objective:** To describe the process of validation of a liquid cytology fixative for HPV genotyping (Papillocheck, Greiner Bio-One).

**Method:** Process conducted in three rounds. First: ability of Fixcytol to preserve HPV DNA in residual material: eight cases (LSIL or HSIL) tested; Index cytology slides blindly re-screened. Second: residual material from ten cytologically normal cases tested; Index cytology slides blindly re-screened. Third: voluntary women (25–35 years), at risk of HPV infection, enrolled; cervexbrush sampling plus Quiagen cervical sampling (QCS) performed. HPV genotyping from the residual material in the three rounds and from the QCS.

**Results:** -First: all cases positive. Cytology diagnoses confirmed. -Second: nine cases negative; HPV 31/39 in one case: HSIL at cytological re-evaluation; biopsy: CIN3. -Third: 38 of 48 pairs concordant: in 36 cases the same types in both, an extra type twice from QCS; in two cases vial content negative and QCS positive, the reverse in two cases; in six cases HPV at Relative Light Unit inferior to 100.

**Conclusion:** This three step method was conducted independently. It was easy to perform. Fixcytol proved: ability to maintain HPV DNA; excellent specificity; as accurate as QSC to type HPV DNA.

**PS-18-029**

**Carcinosarcoma–A challenging diagnosis in cervical cytology smear**

H. Rodrigues ^*^, A. Deus, G. Fernandes, M. F. Xavier Cunha, P. Agapito, A. Figueiredo

^*^CHUC, Anatomia Patológica, Pardilhó, Portugal

**Objective:** Although rare, uterine carcinosarcoma is the most common subtype of mixed mullerian tumours, and a challenging diagnosis in cytology. We report a difficult case whose diagnosis was raised in cervical smear confirmed later in the surgical specimen.

**Method:** A 60 years old woman presented to routine cervical carcinoma screening and the cytology diagnosis was of epidermoid carcinoma with endocervical glandular invasion. The patient was then sent to Cervical Pathology Unit of her home Hospital and the clinical study revealed an endocervical inflammatory polyp retrieved and reported. Six months later the patient was back to Hospital due to abnormal gynecological bleeding and ascites.

**Results:** The ascitic liquid showed malignant cells interpreted as belonging to metastatic carcinoma of the ovary. The patient underwent hyterectomy with bilateral adnexectomy. A 7 cm diameter tumour covered by endometrium and raising in the uterine bottom occupied the whole cavity and the final diagnosis was of a uterine carcinosarcoma with bilaterally involvement of the ovaries and peritoneal implants.

**Conclusion:** Uterine carcinosarcoma usually presents with base implantation in the uterine cavity bottom as the case here reported. The particularity of malignant cells in a routine cervical smear understood as epidermoid carcinoma and needing 6 months till final diagnosis, is important to retain.

**PS-18-030**

**Cytohistological correlation of cervical squamous lesions**

P. Luís ^*^, A. Alves, D. Lopéz-Presa, M. Mendes-Almeida

^*^Hospital de Santa Maria, Dept. de Anatomia Patológica, Lisboa, Portugal

**Objective:** Pap test is an important tool in the detection of cervical lesions, mainly squamous lesions. In literature, the concordance rate (CR) between a positive diagnosis for squamous lesion (ASCUS, ASC-H, LSIL, HSIL and Squamous cell carcinoma (SCC)) and the histologic findings in biopsy and/or conization varies greatly. Our goal was to evaluate the incidence of squamous lesions and the CR.

**Method:** 5,682 liquid-based smears from 2007 were retrospectively revised; 330 (5,8 %) had squamous lesions; in 219 of these a histological evaluation (HEV) was possible, whether simultaneous or in a 6 month period; further follow-up, up to 5 years, was analyzed.

**Results:** 83 (1,4 %) were ASCUS, 36 (43 %) with HEV (15 revealed lesion (CIN 1 to CIN 3)); 5 (0,1 %) were ASC-H, 4 (80 %) with HEV (1 with lesion (CIN 1)); 170 (3,0 %) were LSIL, 113 (66,6 %) with HEV, the CR was 53 % (60 cases); 67 (1,2 %) were HSIL, 62 (93 %) with HEV, the CR was 76 % (47 cases); 5 (0,1 %) were SCC, 4 (80 %) with HEV, the CR was 100 %.

**Conclusion:** The CR in our experience was similar to those found in the literature. Further analysis of discrepant cases will possibly allow contribution to a better CR.

**PS-18-032**

**The composition that restores the hepatocytes in case of toxic liver damage**

S. Roga ^*^, J. Voicehovska, G. Orlikovs, I. Ivanovs

^*^Riga Stradins University, Dept. of Pathology, Latvia

**Objective:** The liver is susceptible to the toxic effects of drugs and toxins; it inevitably suffers the risk of adverse reactions.

**Method:** In vivo study was designed to investigate protective effect of composition, containing metoprolol, essential phospholipids and emulsifier against toxic liver damage. Essential phospholipids are known to benefit in case of liver damage. Acetaminophen-induced liver damage (AILD) was developed; further histology of liver biopsy samples was made. Five-week-old male C57Bl/6J mice (*n* = 24) were divided into 3 groups: A–control group: AILD. B–experimental group: AILD damage receiving a new composition. C–experimental group: AILD receiving essential phospholipids. Haematoxylin-eosin and Sirius red staining were performed using standard protocols. Survived hepatocytes number in light microscopy assesses hepatocytes damage. Laboratory animals were handled according to European Union Regulation guidelines.

**Results:** Group B compared to group A demonstrated less hepatocytes damage: enhanced by 30 % hepatocytes regeneration, reduced by 25 % liver parenchyma fibrosis. Group C compared to group A demonstrated improvement in liver parenchyma by 10 %, number of damaged hepatocytes in group C was 30 % higher compared to group B.

**Conclusion:** The developed composition might be effective in toxic liver damage.

Tuesday, 3 September 2013, 09.30–10.30, Pavilion 2

**PS-19 Poster Session Dermatopathology**

**PS-19-002**

**MMP-9 in lichenoid cutaneous disorders: Overlap and deviation of expression**

I. Legusa^*^, S. Skuja, V. Groma

^*^Daugavpils Nov., Latvia

**Objective:** Lichen ruber planus (LRP) and benign lichenoid keratosis (LK) are similar, sometimes confused, cutaneous disorders. Clinicopathologic spectrum of LK is broad and encompasses several unrelated entities. Action of matrix metalloproteinases (MMP) and collagen-degradation products along with mediation of infiltration has been studied in several skin diseases.

**Method:** We investigated expression of MMP-9 in LRP and LK distinguishing epidermal, interface, dermal infiltrate, and glandular compartment, and correlating changes with clinical parameters.

**Results:** The male/female ratio was 1:2; the average age–66.6 years. Plasma cells, eosinophils, and neutrophils as well as epidermal parakeratosis were demonstrated in LK distinguishing this from LRP. The expression of MMP-9 was greatly varying throughout epidermis and interface regions, and among different variants of LRP being universily strongly expressed in lymphocytic infiltrates and sweat glands. By contrast, weak immunostaining was revealed in parakeratotic loci.

**Conclusion:** LK is a rare disease in Latvia that does not coincide with the word’s literature data. Keratinocytes, glandular and vascular cells expressed MMP-9 in studied lichenoid disorders. Upregulation of MMP-9 in keratinocytes may be influenced by the architectural changes at the interface region; lymphohistiocytic, and in a lesser extent, neutrophilic/eosinophilic infiltrates are essential in pathogenesis.

**PS-19-003**

**Granuloma faciale: A clinicopathological study**

J. Alves ^*^, D. Matos, E. Bártolo

^*^Hospital Garcia de Orta, Dermatology and Venereology, Almada, Portugal

**Objective:** To describe the clinicopathological features of a series of patients with granuloma faciale diagnosed in our Department.

**Method:** A retrospective study from 2001 to 2012, evaluating the demographic and clinico-histopathological features of granuloma faciale was performed.

**Results:** Seven granuloma faciale (5 women and 2 men) were diagnosed. The mean age was 56 years. Five patients had a single while 2 had multiple lesions. All of them were located on the face. In 6 cases a Grenz zone was identified. The inflammatory infiltrate was mixed, predominantly lymphocytic (6 cases) and extended to the reticular dermis (4 cases). In all cases it was also composed by eosinophils and neutrophils. Leukocytoclasia, vascular ectasia and fibrinoid necrosis were detected in 6, 5 and 4 cases, respectively. Fibrosis, hemosiderin and extravasated erythrocytes were found less frequently, in 3, 2 and 1 cases, respectively.

**Conclusion:** The results support most of the published data. Contrary to the literature, in our experience granuloma faciale was more common in women. The detailed characterization of this disease is important to its clinical and histopathological recognition. Furthermore it is essential to establish the differential diagnosis with other more common dermatoses.

**PS-19-004**

**Epidemiology and aetiology of chondrodermatitis nodularis chronica helices**

Y. Yuyucu Karabulut^*^, Y. Dölek, D. Kankaya, B. A. Kirmizi

^*^Cankiri State Hospital, Dept. of Pathology, Çankiri, Turkey

**Objective:** Chondrodermatitis nodularis chronica helicis is a painful nodule affecting the pinna. The aetiology of the disease is unknown. We suggest a possible explanation based upon pathophysiological treatment correlations to histopathological evidence.

**Method:** This study included 17 patients with CNH and the other lesions of the ear that incoming differential diagnoses of CNH a total of 131 patients that were diagnosed between 2011 and 2012 in the clinic of pathology of Cankiri state hospital.

**Results:** With 17 patients 12.97 % of the 131 patients were CNH. The mean age of the patients was 47.41 and the age range of the CNH patients was 32–79 years, with a mean age of 54.9 years. The mean diameter of the lesions was 4.57 mm for CNH patients. On histologic examination epidermal acanthosis associated with a horny, partly parakeratotic, plug, ulceration and crust was the most common finding.

**Conclusion:** This is the first report that research the patients’ cardiovascular problems which can lead perichondrial arteriolar changes as the possible cause of underlying cartilage necrosis resulting in CNH. In the study we also searge the frequency of the lesion amoung all other ear lesions and detected that 12.97 % of the lesions were CNH

**PS-19-005**

**Tattoo and generalised lymphadenopathy: A case report**

G. Narli^*^, Z. Kucukodaci, I. Yilmaz, U. Berber, A. Haholu, D. Demirel

^*^GATA HEH, Pathology, Istanbul, Turkey

**Objective:** We present a patient with generalised lymphadenopathy secondary to multiple, wide, whole body tattooing. The clinical and pathological significance of this case is discussed.

**Method:** 21 years old male patient was admitted to our hospital for multiple palpable nodules in his neck, bilateral groins and axillar region. After 3 weeks antibiotic treatment there was no regression in size of nodules. The biggest lymph node in the cervical region was removed surgically and sent to pathologic examination.

**Results:** Lymph node was measuring 1.5 × 1 × 1 cm. Microscopically, there were benign histiocytes with dark pigment material within subcapsular and sinusoidal areas of the lymph node, but nodal architecture was preserved. Immunohistochemical analysis showed no evidence of lymphoma or metastatic melanoma. We have questioned whether patient has tattooing in his body or not; and we found out that patient has multiple large tattoos in whole body but he did not have any skin lesion. The case was diagnosed as benign reactive lymphadenopathy with pigment accumulation due to tattooing.

**Conclusion:** Tattoo pigment can migrate to the regional lymph nodes and can cause lymphadenopathy. Tattooing should always be considered in differential diagnosis of pigmented lymphadenopathy. Especially, when examining a lymph node of a melanoma patient, tattoo history should be inquired to avoid radical surgery.

**PS-19-006**

**Calcinosis cutis**

P. Constantinou ^*^, I. Themeli, D. Riga, M. Chantziara, N. Poulianitis, C. Vourlakou

^*^Athens, Greece

**Objective:** Calcinosis cutis (also known as cutaneous calcification) is a rare manifestation of systemic calcinosis, characterized by precipitation and deposition of calcium and phosphate deposits in the dermis and subcutaneous tissue.

**Method:** A 48-year-old woman presented with multiple, firm, erythematous and ulcerated nodules on the anterior and lateral surfaces of the lower extremities, with gradual but accelerating enlargement within a few months and excreted a white, chalky material. The last 5 years the patient was undergoing chronic dialysis for the treatment of end-stage-renal-failure of unknown cause and revealed a history of acute intermittent porphyria, 25 years ago without skin lesions.

**Results:** Histopathology revealed amorphous, dystrophic, calcium deposits in the dermis and subcutaneous tissue as well as foreign body reaction. A diagnosis of calcinosis cutis was made. No evidence of calciphylaxis was observed.

**Conclusion:** Calcinosis cutis occurs in approximately 1 % of patients with end-stage renal disease undergoing chronic dialysis, usually after a short period of dialysis (median, 4 years). It is associated with elevated levels of plasma calcium-phosphate product and increased serum phosphate concentration. Metastatic calcification occurs primarily in patients with chronic renal failure with primary or secondary hyperparathyroidism, Vitamin D intoxication and milk-alkali syndrome.

**PS-19-007**

**Peculiar variants of primary cutaneous anaplastic large-cell lymphoma: Signet ring cell and ALK-1 positive**

G. Pérez Esteve ^*^, A. Sologaistoa, M. Turell, D. Sitjas, E. Llistosella, F. Pérez-Bueno

^*^Hospital Dr. Josep Trueta, Dept. of Pathology, Girona, Spain

**Objective:** To highlight the clinical, light microscopic, and phenotypic features of two cases of the least common types of Primary Cutaneous Anaplastic Large-Cell Lymphoma (PCALCL).

**Method:** Skin biopsies were taken from both patients and stained with H&E and immunohistochemically with CD20, CD79a, CD3, CD4, CD8, CD43, CD45R0, CD30, CD56, ALK-1 and bcl-6 antibodies.

**Results:** Patient 1: 70-year-old man with a rapidly growing 6 cm. ulcerated tumour on the left leg. Biopsy revealed extensive infiltration of the dermis and subcutis by large anaplastic cells that expressed CD8 (focal), CD43, CD45R0, CD30, CD56 and AKL-1. He had no history of nodal disease and a negative staging. Surgical excision and radiation therapy resulted in complete remission. No local relapse or systemic disease was seen during a follow-up of 20 years. Patient 2: 45-year-old woman with 3-weeks history of a 3.5 cm ulcerated plaque on the left forearm. Punch biopsy showed a superficial dermal infiltrate of signet-ring type cells with anaplastic morphology that were diffusely positive for CD3, CD4, CD5, CD30 and CD43. The tumour showed spontaneous regression. Follow-up for 13 years showed no local relapse.

**Conclusion:** Recognition of these rare variants of PCAALCL is important because of their favourable prognosis related to their differential diagnosis.

**PS-19-008**

**Blastic plasmacytoid dendritic cell neoplasm**

E. Mejia Urbaez ^*^, M. C. Yus Gotor, C. Hörndler Argarate, C. Salvador Osuna, J. Alfaro Torres, S. Vicente Arregui, D. S. Rosero Cuesta, M. Alastuey Aisa, N. Torrecilla Idioipe, A. Valero Torres, C. Muñoz Montano

^*^Zaragoza, Spain

**Objective:** Rare entity that was recently classified as an acute myeloid leukemia related to precursor neoplasms in the WHO classification. Overall incidence is extremely low, accounting for 0,44 % of all hematologic malignancies and 0,7 % of cutaneous lymphomas.

**Method:** 68 year old man with 2 months chest, right mandibular and right leg red macules, itchy and rapidly growing. We received cuts of skin with subcutaneous tissue.

**Results:** A nodular infiltrated monomorphic blastoid cells, occupying the entire dermis and subcutaneous tissue, without destroying, not necrosis, were observed. Papillary dermis and epidermis were unaffected. The cells are of medium size, with irregular and indented nuclei, granular chromatin and one or more small nucleoli. The cytoplasm is priceless and abundant mitosis, are accompanied by reactive lymphocytes and eosinophils. IHC: positivity for CD4, CD56, CD43, Bcl2, and Bcl6 (weak), was consistent with the result of flow cytometry in the bone marrow. Blastic Plasmacytoid dendritic cell neoplasm was our final diagnosis.

**Conclusion:** BPDCN has a highly aggressive clinical course with poor diagnosis, showing a high incidence of cutaneous and bone marrow involvement and risk of leukemia dissemination. The medial survival period of 14 months and 2–5 years overall survival rates of 33 % and 6 % respectively.

**PS-19-009**

**A rare case of chronic eosinophilic pneumonia associated with mycosis fungoides**

T. Koletsa ^*^, F. Delli, I. Lefaki, G. Karkavelas, I. Kostopoulos

^*^Medical School AUTH, Dept. of Pathology, Thessaloniki, Greece

**Objective:** There are several reports describing the association of chronic eosinophilic pneumonia (CEP) with lymphomas. We report a unique case of Mycosis Fungoides (MF) diagnosed 3 years after full clinical resolution of CEP.

**Method:** A 60-year-old woman was hospitalized with a generalized pruritic dermatitis-like skin lesions resistant to antihistaminic therapy and topical corticosteroids for the last 3 months. A skin biopsy from an infiltrated abdominal plaque was sent for histologic examination.

**Results:** Hematoxylin and eosin stained sections revealed lymphoid infiltration of the cutis with neoplastic cells having irregular nuclei. These cells were also apparent in the epidermis, forming Pautrier abscesses or having a linear basal infiltration pattern. On immunohistochemical analysis the neoplastic cells expressed CD45RO, CD3, CD2, CD5, CD4 and CD99 antigens, while they were negative to CD8, CD20, CD45RA, CD56 and Tdt. A small number of the cells being positive to CD7 and CD30 antigens. These histological findings in correlation with clinical picture are compatible with MF diagnosis.

**Conclusion:** It must be kept in mind that CEP may precede lymphoma. Thus, when patients present with skin lesions and refer a history of eosinophilia it is important to include cutaneous lymphoma in differential diagnosis.

**PS-19-010**

**Dendritic cells in cutaneous lymphomas: Are they clinically relevant?**

G. Dyduch ^*^, J. Szpor, M. Bialas, K. Okon

^*^Jagiellonian University, Dept. of Pathomorphology, Kraków, Poland

**Objective:** Primary cutaneous lymphomas often feature different clinical course and prognosis compared to systemic lymphomas. Dendritic cells may play a role in the pathogenesis of cutaneous lymphomas, but their prognostic significance was not extensively studied. The aim of this work was investigation the occurrence and number of dendritic cells in cutaneous lymphomas.

**Method:** The analysis included 118 cases of cutaneous lymphomas. Dendritic cells demonstrating expression of S-100, CD1a, CD207 and CD68 were evaluated within tumors’ infiltrate.

**Results:** This study demonstrated in indolent cutaneous lymphomas a statistically significant higher number of dendritic cells and Langerhans cells within tumors’ infiltrate compared to intermediate and aggressive lymphomas.

**Conclusion:** The results obtained from this work might indicate the impact of the number of dendritic cells in cutaneous lymphomas on their clinical behavior.

**PS-19-011**

**Langerhans cells distribution may discriminate between early stage mycosis fungoides and inflammatory dermatosis**

M. Petre ^*^, S. Zurac, R. Andrei, T. Tebeica, A. Birceanu, R. Chirculescu, C. Popp, A. Evsei, F. Staniceanu, A. Bastian

^*^Colentina University Hospital, Pathology, Bucharest, Romania

**Objective:** Langerhans cells (LCs) are professional antigen-presenting cells with important role in regulating immune responses against pathogens and skin tumors. The differentiation between inflammatory dermatosis and patch stage mycosis fungoides (MF) is difficult since they share similar histology, no specific immunophenotype and/or genetic alterations allowing proper discrimination. However, LC density and distribution within dermal infiltrate might offer insights about neoplastic or reactive nature of the lymphoid population.

**Method:** We studied 56 patients, 28 with patch/plaque stage MF, 28 with spongiotic dermatitis (SD); positive diagnosis was established based on histopathologic examination corroborated with clinical data, independently confirmed by three pathologists. Immunohistochemical tests for CD1a and Langerin were performed and density and distribution of LCs were recorded in each case.

**Results:** The majority of cases presented numerous LCs in the dermis (82.14 % in each group); LCs form clusters with arachnoid or nodular appearance. Arachnoid pattern of LCs distribution within lymphoid infiltrate was more frequent in MF group (89.29 %) than in SD group (67.86 %) while nodular pattern predominates in SD group (32.14 %) than in MF group (10.71 %). Arachnoid pattern of dermal LCs clustering was significantly associated with MF (*P* = 0.05).

**Conclusion:** Immunohistochemical characterization of LCs distribution may help in differentiation between early stages of MF and SD.

**PS-19-012**

**Various patterns of CD3+ cells distribution in cutaneous B-cell lymphoid infiltrates as histopathologic parameter in differential diagnosis between malignancy and pseudolymphoma**

A. Birceanu Corobea ^*^, S. Zurac, R. Andrei, T. Tebeica, M. Petre, S. Gheorghita, C. Socoliuc, G. Halcu, F. Staniceanu, L. Nichita

^*^Colentina Universitary Hospital, Pathology, Bucuresti, Romania

**Objective:** Cutaneous B-cell lymphoid infiltrates can be difficult to diagnose especially in those cases with dense cellularity. Differentiation between cutaneous B-cell lymphomas (CBCL) and pseudolymphoma on histopathologic level imposes corroboration between routine microscopy, immunohistochemical and, sometimes, molecular workup.

**Method:** We analyze T-cell infiltration (density and pattern) (based on immunohistochemical testing for pan-T marker–CD3) in 13 cases of CBCL (5 marginal zone B-cell lymphomas, 3 follicle center lymphomas, 4 diffuse large B-cell lymphomas and one B-cell small lymphocytic lymphoma) and 18 cases of cutaneous pseudolymphomas.

**Results:** Two main patterns of T-cell distribution were identified: diffuse pattern (relatively uniform distributed CD3+ cells without any area of focal conglomeration within lymphoid infiltrate) and polarized pattern (areas of dense CD3+ cells delineating nodular areas of lymphoid infiltrate with rare–if any–T-cells). All the CBCL have diffuse pattern of CD3+ cells (irrespective of their density within the tumor mass) while all but 3 cases of pseudolymphomas have polarized pattern of CD3+ T-cells (*P* < 0.001). In pseudolymphomas with diffuse pattern of T-cells, there was a relatively low density of lymphoid cells within the lesion in comparison with the other cases.

**Conclusion:** Distribution of CD3+ cells within cutaneous B-cell lymphoid infiltrates may facilitate differential diagnosis between CBCL and pseudolymphoma.

**PS-19-014**

**Atypical FibroXanthoma (AFX): A clinicopathologic and inmunohistochemical study**

S. Fernandez ^*^, A. Fernandez de Larrinoa, G. Cancho, M. Zufiaurre, M. C. Etxezarraga

^*^H.U. Basurto, Dept. of Pathology, Bilbao, Spain

**Objective:** The recognition of morphological variants of AFX.

**Method:** Morphological features analyzed in 25 cases: localization, ulceration, circumscription, vascular/perineural invasion, and histological pattern. The antibodies used were: vimentin, CD68, CD10, CKAE1/AE3, SMA, desmin, CD31, CD34, HMB-45, Melan-A, S100 and EMA.

**Results:** There were 22 males and 3 females, aged 56–97. All lesions were developed in the sun exposed region. Ulceration was found (56 %). The most frequent morphological pattern was an admixture of spindle/epithelioid cells (44 %), followed by predominantly spindle (36 %), and less frequent pure spindle (12 %) and predominantly epithelioid (8 %). The majority of lesions were localized in dermis (68 %). The deepest margin was expansive (44 %) and infiltrative (48 %). No vascular/perineural invasion. Additional histological changes were hemorrhagic/pseudo-angiomatous areas (28 %), osteoclast-like giant cells (24 %), keloid-like areas (24 %), granular cell change (12 %), stromal myxoid degeneration (4 %) and absence of clear cell change. These can be found focally or can represent the predominant component. All tumors are vimentin, CD68, CD10 and EMA (weak and focal) positive, and negative for the other markers.

**Conclusion:** Numerous histological morphology features can be encountered. Diagnosis is still made by exclusion. Thus inmunohistochemistry remains crucial to establish the diagnosis.

**PS-19-015**

**Atypical fibrous histiocytoma: A case report**

F. Costa ^*^, A. Coelho, A. Duarte

^*^Centro Hospitalar do Porto, Braga, Portugal

**Objective:** Atypical Fibrous Histiocytoma (AFH) is a rare variant of cutaneous fibrous histiocytoma, first described in 1983 by Fukamizu. It presents as a solitary firm cutaneous nodule in a broad age range (5–79 years) and a roughly equal gender distribution. AFH is most commonly observed on the lower limb/girdle, followed by the upper limb/girdle and trunk.

**Method:** We report a case of a 50-year-old man with an unremarkable medical history who presented to our institution with a solitary cutaneous lesion described as a dark pigmented, slightly elevated plaque on the left shoulder, associated with pain and pruritus.

**Results:** Macroscopically, there was a poorly demarcated dermal nodule of 1,2 cm in diameter, whitish cut surface and firm consistency. Histologically, it demonstrated the usual cytoarchitectural features of conventional fibrous histiocytoma but, in addition, there were larger cells with bizarre, mainly vesicular nuclei and variably prominent nucleoli and rare atypical mitotic figures, rendering the diagnosis of AFH.

**Conclusion:** AFH represents a distinctive variant of FH that has a tendency to recur locally and a capacity to metastasize, albeit very rarely. No histological features were found to be predictive for local recurrence or development of metastatic disease. The differential diagnosis includes atypical fibroxanthoma, dermatofibrosarcoma protuberans and malignant fibrous histiocytoma.

**PS-19-016**

**Variants of dermatofibroma**

J. Alves ^*^, D. Matos, E. Bártolo

^*^Hospital Garcia de Orta, Dermatology and Venereology, Almada, Portugal

**Objective:** Dermatofibroma is one of the most common benign cutaneous soft tissue lesions with several variants described in the literature. The objective of this study is to review the clinical and histopathological features of variants of dermatofibroma diagnosed in our department in a 5-year period.

**Method:** A retrospective study of skin biopsies and tissue excisions of dermatofibromas carried in our Department between May 2007 and April 2012 was performed. The clinical and histopathological features were evaluated.

**Results:** 192 dermatofibromas were diagnosed in 181 patients. They were more common in women with a median age of 48. The most common location was the limbs (74 %). In 78 % of cases, the suspected clinical diagnosis was dermatofibroma. The histopathological types that have been found were the common fibrous histiocytoma (80 %) and the aneurismal (5,7 %), haemosiderotic (5,7 %), epithelioid (2,6 %), cellular (2,1 %), lipidized (2,1 %), atrophic (1,0) and clear cell (0,5 %) variants.

**Conclusion:** This review focuses on the clinical and histological features of the several variants of dermatofibroma, regarding principally its clinical presentation, distinct histopathologic features, differential diagnosis and prognosis. This study ultimately pretends to facilitate the exact identification of the several dermatofibromas variants.

**PS-19-019**

**Diffuse neonatal haemangiomatosis: Report of a case**

A. Diaz-Lagama ^*^, S. Vázquez Navarrete, R. Jiménez Peña, M. Espinel Vázquez

^*^Hospital de La Línea, Dept. of Pathology, Cádiz, Spain

**Objective:** This study aimes to examine clinical and follow-up data, together with histopathological features of a rare disorder, diffuse neonatal haemangiomatosis (DNH).

**Method:** This is a descriptive retrospective case of a 5 months infant female with progressive appearing cutaneous hemangiomas, which were present at birth. Until now, the miliary haemangiomatosis counts up to 70 lesions, ranging from 0.2 to 2 cm. Spontaneous regression of some of them recently occurred.

**Results:** Multiple cutaneous haemangiomas may occur with or without disseminated visceral haemangiomas. In those cases with visceral involvement, any organ may be affected, and the morbidity and mortality rates are high. The most commonly affected organs are liver, brain, digestive tract, lungs, eyes, mouth and kidneys. This case has been diagnosed as a purely cutaneous form of DNH.

**Conclusion:** Appropriate evaluation and accurate diagnosis of neonatal haemangiomas is imperative to allow an early treatment.

**PS-19-020**

**Verrucous haemangioma**

Y. Lorenzo Mahìa ^*^, B. Iglesias Rodriguez, M. San Martin Alonso, N. Alfonsin Barreiro

^*^Hospital Meixoeiro, Dept. de Anatomìa Patológica, Mos, Spain

**Objective:** Verrucous hemangioma is a rare vascular malformacion. It is presents at birth or in early childhood. It is typically unilateral and localized to the lower extremities. Bleeding or scratching is frequent. Surgery is the gold-standard treatment.

**Method:** An 12 year-old female, has a painful verrucous lesion on the right lower leg. The lesion were removed and diagnosed as angiokeratoma.

**Results:** After relapsed, it was diagnosed as verrucous hemangioma.

**Conclusion:** Verrucous hemangioma usually appears at birth or in early childhood on the lower extremities as a unilateral isolated condition. Clinically it may be single or grouped. Distribution is linear or serpiginous and satellite lesions are typical. Histopathologically, it shows prominent acanthosis, papillomatosis and hyperkeratosis with a proliferation of dilated and congested vessels in the upper dermis with vascular capillary-like in the deep dermis and subcutis. Epidermal tongues encircled the vessels in the papillary dermis. The diagnosis is made by histopathological examination, because clinical findings can mimic others lesions. The histological appearance closely resembles an angiokeratoma. Actually verrucous hemangioma is considered by most a malformation rather than an authentic hemangioma. The gold standart treatment is surgery, and requires a large, deep excision with adequate margins. Incomplete excision leads to recurrence.

**PS-19-021**

**Lymphangioma/hemangioma-like Kaposi sarcoma: A case report**

A. Papanastasiou ^*^, Z. Stamou, P. Aroukatos, D. Nakas, M. Repanti

^*^Patras General Hospital, Dept. of Pathology, Greece

**Objective:** Lymphangioma- and/or hemangioma-like variants of Kaposi sarcoma (LLKS and/or HLKS) are rare comprising fewer than 5 % of all reported cases. LLKS was first described by Ronchese but its histologic characteristics were reported in 1979 by Gange. HLKS was first mentioned by Ackerman in 1988.

**Method:** We report on a 78-year-old man who presented with two bluish bullous dermal nodules on his left leg.

**Results:** Histologic findings were consistent with LLKS/HLKS mixed with classic KS areas. LLKS areas consisted of ectatic, irregularly shaped vascular spaces lined by mildly atypical endothelial cells. HLKS areas consisted of dilated vessels, stuffed with erythrocytes. All tumour cells, including those associated with LLKS/HLKS areas, showed strong HHV8, CD34, CD31 and podoplanin positivity.

**Conclusion:** LLKS and/or HLKS are rare morphologic variants of KS that have the potential to mimic benign and malignant vascular tumors. The differential diagnosis relies on the recognition of typical KS areas, although such areas have been absent from some cases. HHV8 and podoplanin positivity play an important role in establishing definitive diagnosis. Podoplanin expression in LLKS/HLKS as well as in classic Kaposi sarcoma areas provokes consideration as to whether the disease originates from vascular and/or lymphatic endothelial cells.

**PS-19-022**

**Evaluation of c-Kit expression in classic Kaposi’s sarcoma in Greek patients**

G. Panselinas ^*^, I. Michalopoulou Manoloutsiou, A. Giannouli, D. Minotakis, V. Theodorou, G. Theodoropoulos, A. Nikolaidou

^*^Theageneio Anticancer Hospital, Pathology, Thessaloniki, Greece

**Objective:** Kaposi’s sarcoma (KS) is an angioproliferative disorder associated with human herpesvirus 8 (HHV8). It is classi_ed into four clinico-epidemiological types: classic, endemic, iatrogenic and AIDS-associated. HHV8 is related with all epidemiological types of KS and has been shown to induce the tyrosine receptor kinase c-Kit in infected cells. Our aim is to evaluate the expression of c-Kit in cases of classic KS.

**Method:** In total, 10 cases of classic KS at various histological stages and different sites were included in the study. Age and gender of the patients, location and histological stage of the tumours were recorded. Formalin-fixed, paraffin wax-embedded tissue sections were stained immunohistochemically with antibodies to c-Kit and HHV8 antigen. C-kit cytoplasmic and/or membranous staining reaction in >1 % of tumour cells was considered positive.

**Results:** c-Kit immunoreactivity was found in 5 cases (50 %) and HHV8 immunoreactivity was present in all cases (100 %).

**Conclusion:** Our study shows that in cases of classic KS there is a high rate of c-Kit expression. These data demonstrate an essential role for C-kit in KS tumorigenesis and reveal a target for pharmacological intervention.

**PS-19-023**

**Clear cell/balloon cell dermatofibroma of skin: A rare variant**

C. Fiandesio ^*^, C. Santonja-Garriga, C. Saus, L. Requena

^*^Dept. of Surgical Pathology, Fundación Jiménez Díaz, Madrid, Spain

**Objective:** Cutaneous fibrous histiocytomas (dermatofibromas) are frequent benign neoplasms usually arising on the legs of young women. Zelger et al. have described clear cell dermatofibroma as a rare “minor histologic variant” of benign fibrous histiocytomas. Only a handful of cases have been reported, some of them with a balloon cell morphology. The differential diagnosis includes cutaneous clear cell myomelanocytic tumor, a member of the PEComa family, with morphologic similarities and a distinct immunophenotype.

**Method:** We report two leg lesions in two women (39 and 48 years), one polypoid and one forming a plaque. A wide immunohistochemical study was performed.

**Results:** Histologically both consisted of solid dermal proliferations of clear/balloon cells with smooth margins. Both cases express CD68, Vimentin and Factor XIIIa, and were negative for Melan A, HMB45, AE1/AE3, S100 and Smooth Muscle Actin. No cytoplasmatic PAS positive material was found.

**Conclusion:** Knowledge of this rare variant is important, so as not to misinterpret this lesions as a malignancy. Lack of HMB45 on immunohistochemical study allows exclusion of cutaneous PEComa. Differential diagnosis with dermatofibrosarcoma protuberans, clear cell sarcoma, clear cell squamous-cell carcinoma, or balloon cell melanoma is mandatory.

**PS-19-024**

**Verruciform xanthoma of the genital skin: Report of two cases**

J. Cassis ^*^, I. Viana

^*^Caxias, Portugal

**Objective:** Verruciform xanthoma is a rare entity that occurs most commonly in the oral mucosa. Very few cases have been reported in the skin namely in the anogenital area.

**Method:** We report two cases: one of a white verrucous lesion in the left labia minora of an 80-year-old patient, the other of a pediculated papule in the scrotum of a 58-year-old patient.

**Results:** Both lesions had verruca-like configuration with foamy xanthoma cells in the papillary dermis. The diagnosis was verruciform xanthoma.

**Conclusion:** The etiology of verruciform xanthoma is unknown. Although HPV has been proposed as a causative agent this hasn’t been confirmed by most studies. Other dermatological conditions can be associated with verruciform xanthoma such as lichen planus or pemphigus vulgaris. The importance of these two cases concerns the rarity of these lesions in the skin and the differential diagnosis with other lesions including carcinomas.

**PS-19-025**

**Digital pacinian neuroma: A distinctive hyperplastic lesion**

B. Chelly^*^, I. Chelly, H. Azzouz, A. Zehani, H. Nfoussi, K. Bellil, S. Haouet, N. Kchir

^*^Tunis, Tunisia

**Objective:** Neural tumours composed solely of Pacinian corpuscles or showing focal Pacinian differentiation are extremely rare and have only occasionally been reported in the literature. Our aim is to report a new case of Pacinian neuroma and to describe its clinical and histological features.

**Method:** We report a case of digital Pacinian neuroma diagnosed in the Department of pathology, Rabta hospital, Tunis, Tunisia.

**Results:** A 30-year-old man presented with a tender area in the pulp of his left index fingertip. All symptoms were localized to the tip of the index finger. Physical examination revealed only an exquisitely tender left index fingertip: no definite mass was palpable and there were no skin or nail abnormalities. We decided to explore the finger. Histopathological findings revealed pacinian corpuscles to be increased in size and number and occasional sweat glands. Individual corpuscles consisted of a central nerve fiber surrounded by 35 to 60 concentric lamellae. Many small nerves were associated with the Pacinian corpuscles.

**Conclusion:** Hyperplasia of Pacinian corpuscles is an unusual cause of digital pain. This diagnosis should be considered in the differential diagnosis of painful lesions of hand along with glomus tumor, neuroma of digital nerve and subungual exostosis.

**PS-19-026**

**A rare case of plexiform epithelioid schwannoma**

A. Coelho ^*^, F. Costa, J. R. Vizcaíno

^*^Centro Hospitalar do Porto, Anatomia Patológica, Portugal

**Objective:** Epithelioid Schwannoma (ES) is a rare tumor, and the plexiform variant is extremely unusual, with only one case described. It is a diagnostically challenging, and not yet fully characterized tumor.

**Method:** We report a case of a 53-year-old woman with a recurrent cutaneous lesion described as an asymptomatic irregular nodule on the left thigh. A review of the primary lesion, excised 3 years before, was also made.

**Results:** Macroscopically, there was a well-defined dermal nodule of 1,0 cm in diameter, whitish cut surface and firm consistency. Histologically, the lesion demonstrated a plexiform multinodular pattern, consisting of clusters of uniform epithelioid S100 protein-positive cells with round to oval nuclei and prominent nucleoli. Scattered thin-wall vessels were noted. No cytologic atypia, invasion or necrosis were observed, but the mitotic activity was high (6 mitoses per 10 high power fields (HPF)), with no atypical mitotic figures. The primary tumor was identical, with the exception of a much lower mitotic activity (1 mitosis per 10 HPF).

**Conclusion:** Overall, ES tumors with bland cytologic features are thought to act in a benign fashion. Our case, however, showed local recurrence, with an increased mitotic activity in the recurrent lesion.

**PS-19-027**

**A case of ectopic hamartomatous thymoma (branchial anlage mixed tumour)**

B. A. Kirmizi ^*^, A. Okcu Heper, S. Yuksel, H. Akgül

^*^Ankara University, Pathology, Turkey

**Objective:** Ectopic hamartomatous thymoma (EHT) is a rare benign tumour of the lower neck showing an admixture of spindle cells, epithelial islands, and adipose tissue. We wanted to present our case, because of its rarity (with about 50 reported cases) and diagnostic challenge.

**Method:** Our case was a 36-year-old woman presenting with subcutaneous mass on the left lower neck area. Ultrasound imaging of the neck revealed a 10 cm well-circumscribed nodule with cystic areas and lipomatous echogenicity. A total excision was performed.

**Results:** The tumor was well-circumscribed, elastic-firm nodule with lipomatous areas and small cysts. Microscopically, it was predominantly composed of delicate, spindle cells arranged haphazardly or in fascicules, adenomatoid-syringomatoid epithelial areas, small cysts and mature adipose tissue. Immunohistochemically spindle cells were both positive with epithelial and myoepithelial markers.

**Conclusion:** The differential diagnoses of EHT include benign and malignant tumours with a biphasic pattern, such as adnexal cutaneous tumours, salivary gland tumours, sarcomatoid carcinoma, synovial sarcoma and teratoma. The diagnostic accuracy mainly depends on awareness of this very rare peculiar tumour with characteristic lower neck localization, distinctive histomorphological and immunohistochemical features.

**PS-19-028**

**Genital melanocytic naevus, with invasion of lymphatic vessels: A case report**

J. Palla Garcia ^*^, R. Sampaio, R. Dias, A. Duarte

^*^Oporto’s Hospital Centre, Dept. de Anatomia Patológica, Porto, Portugal

**Objective:** Melanocytic lesions on the genital area are rare, they arise mainly on the vulva, less frequently they may also occur on the perineum, pubic region, and male genitalia. Genital melanocytic naevus (GMN) generally exhibit characteristics similar to other parts of the body, where the invasion of lymphatic vessels by melanocytes is an infrequent finding. Most GMN are compound or intradermal. There is a subgroup known as atypical genital naevus with unsual histologic features.

**Method:** We present a case of a 28-year-old female, pregnant with a 10 mm tan-brown papule in the perineum that was detected by her gynecologist, after the delivery. She underwent excision of the lesion.

**Results:** Grossly was a skin fragment centered by a raised brown papule. Histologically was an intradermal melanocytic naevus, with a large cell type, some mitotic activity probably pregnancy-related, and with groups of naevus cells bulging into lymphatic channels. The lesion was regarded as benign. The surgical resection was complete.

**Conclusion:** Regardless of the infrequent findings of this case, GMN differ from melanoma by circumscription, maturation and symmetry. The invasion of the lymphatic vessels is a rare finding that associated with the mitotic activity could lead to a misdiagnosis of malignancy.

**PS-19-029**

**Desmoplastic melanoma: A clinico-pathological study of 18 cases**

A. Konstantinov ^*^, K. Shelekhova

^*^Research Institute of Oncology, Dept. of Pathology, St. Petersburg, Russia

**Objective:** Desmoplastic melanoma (DM) is an uncommon type of melanoma that is characterized by diffusely infiltrative malignant spindle cells within prominent fibrocollagenous stroma. It may be mixed up with nonmelanocytic lesions, both at clinical and histopathological levels, what is associated with an inadequate excision and high rate of local recurrence.

**Method:** We have studied the clinico-pathological features of 18 retrospective cases of DM.

**Results:** The mean age of the patients was 67 years; 61.2 % were males and 38.8 % were females. The neoplasm was located on the head and neck region in 39 %, on the trunk in 28 % and on the lower limbs in 33 % of the cases. Malignant melanoma was the clinical diagnosis in only 66 % of the cases. Pure DM was in 61 % of primary tumors and combined DM was in 39 % cases. The follow-up information was obtained in all cases: 39 % patients had repeatedly recurrent tumors (disease-free interval range 12–28 months), in 5 %–lymph node metastasis. On the basis of morphology two lines of tumor progression were observed. The first line is neural transformation and the second one is an increase of malignancy.

**Conclusion:** Our results demonstrate peculiar histological properties in recurrences of DM. Further investigations will define its relevance.

**PS-19-030**

**Primary pulmonary melanoma: Case report**

L. Carvalho ^*^, N. Dias, M. Pontes, P. Rodrigues, I. Coutinho, M. L. Coelho, M. J. Martins

^*^Faculty of Medicine Coimbra, Inst. of Anatomical Pathology, Portugal

**Objective:** Primary melanoma of the lung is a rare entity, accounting for 0.01 % of lung tumors and the diagnosis is one of exclusion. The therapeutic approach is not consensual, but complete resection of the primary lesion appears to give better control of the disease; yet, the prognosis is still uncertain.

**Method:** Our case concerns a 74 year old lady, followed for COPD, who presented a lower left lobe unique nodular lesion in a thoracic radiography, and later in a PET scan, suggesting a probable 2 cm primary pulmonary carcinoma.

**Results:** After transthoracic biopsy, an excisional surgical biopsy was performed, which revealed a pigmented tumor with large epithelioid cells, exhibiting anisokaryosis and immunohistochemical positivity to vimentin, S100 protein, HMB45, NSE and synaptophysin. Extensive clinical studies confirmed the pulmonary primary origin of the melanoma.

**Conclusion:** Five months after surgical resection the patient survives, but developed multiple pulmonary metastatic nodules, having started chemotherapy with dacarbazine. The mutational study was negative for BRAF. Different theories try to explain the origin of a primary pulmonary melanoma; one of them hypothesizes that it derives from melanocytes that migrated to the lung during embryogenesis; another theory emphasizes that melanoma cells may derive from a pluripotent stem cell, with or without neuroendocrine differentiation.

**PS-19-032**

**Melanoma metastasis in parotid gland: Fine-needle aspiration diagnosis as a clinical presentation**

H. Rodrigues ^*^, C. Oliveira, D. Oliveira, M. J. Julião, G. Fernandes, M. F. Xavier Cunha

^*^CHUC, Anatomia Patológica, Pardilhó, Portugal

**Objective:** Parotid gland is a common site for metastasis for any tumor. Salivary glands lodge metastasis from head and neck tumors especially melanoma and squamous cell carcinoma.

**Method:** A 76 years old male presented with a right parotid nodule with 8 months evolution, mobile, painless and well circumscribed that the CT scan of head and neck revealed with 2,5 cm. A fine-needle aspiration was performed and reported as consistent with melanoma metastasis to parotid gland. The patient underwent parotidectomy and the diagnosis was confirmed together with clinical study: a skin lesion with 0,6 cm was found in scalp, removed for diagnosis and corresponding to an achromic melanoma with 3,4 mm.

**Results:** Metastatic disease as the first manifestation of a malignant tumor is not rare and runs with poor prognosis. This case reveals the importance of fine-needle aspiration in the diagnosis of salivary glands nodules.

**Conclusion:** Diagnosis of melanoma in cytology maybe again difficult when based on morphological criteria such as intracytoplasmatic pigment, intranuclear inclusions and binucleation, needing immunochemistry for confirmation.

**PS-19-033**

**CD44s expression in melanomas**

V. Samaras^*^, P. Lazari, S. Kalantzakis, O. Pantzartzi, C. Barbatis

^*^Athens, Greece

**Objective:** CD44s represents a major cell-adhesion molecule implicated in the progression of various tumors. Melanomas are characterized by their aggressive behaviour and by their poor response to chemotherapeutic agents. Appropriate immunohistochemical and molecular markers that could accurately predict the progression of these neoplasms have not been identified so far. We studied the expression of CD44s, S-100 protein and Ki67 in a series of melanoma patients attempting to examine any association with tumor Breslow’s thickness and Clark’s stage.

**Method:** Formalin-fixed and paraffin-embedded tissues from 24 cases of melanomas [17 of the skin, 3 of the nasal cavity, 2 ocular, 1 nasopharyngeal and 1 metastatic to stomach] were immunohistochemically tested for the expression of CD44s, S-100 and Ki67.

**Results:** CD44s was diffusely expressed in all cases, though with variable intensity. A primarily membranous pattern of immunoreactivity was recognized. Statistical analysis did not reveal any significant association among CD44s, S-100, Ki67, Breslow’s thickness and Clark’s stage.

**Conclusion:** Our results indicate that CD44s expression is common in melanomas and unrelated to site, Breslow’s thickness or Clark’s level of invasion. Nevertheless, larger series are needed in order to elucidate the exact role of cellular adhesion within malignant melanomas.

**PS-19-034**

**Cutaneous side effects associated with BRAF-inhibitor therapy: Report of two cases**

M. Vozmitel ^*^, J. M. Bogdaev, T. I. Nabebina, A. Dubrovskij

^*^Minsk, Belarus

**Objective:** Vemurafenib (Zelboraf) is a chemotherapeutic BRAF-inhibitor, which is used for treatment of metastatic BRAF V600E mutation-positive melanoma. It is known, that BRAF-inhibitors are linked to the development of cutaneous squamous cell carcinoma and keratoacanthomas.

**Method:** We evaluated the clinical and histologic features of skin side effects developing on vemurafenib therapy in 2 patients from our institution.

**Results:** First patient developed one squamous cell carcinoma, second patient formed benign vascular skin lesion–lobular capillary hemangioma. Side effects appeared as early as 1 and 2 months after therapy beginning, respectively.

**Conclusion:** Cutaneous vascular changes as side effect of BRAF-inhibitor therapy were not mentioned earlier. It is known, that squamous cell proliferations on the background of BRAF-inhibitor therapy probably connected with RAS mutations, similar, the RAS gene family encodes membrane-associated proteins involved in the control of cell proliferation, differentiation and organization of endothelial cells into highly organized networks. Awareness of potential adverse effects of BRAF inhibitor therapy is necessary.

**PS-19-035**

**Human Telomerase Reverse Transcriptase (hTERT) expression in actinic keratosis, Bowen’s disease and invasive squamous cell carcinoma of the skin**

M. Stojanovic ^*^, D. Brasanac, S. Cirovic, R. Jankovic

^*^University of Belgrade, Faculty of Medicine, Inst. of Pathology, Serbia

**Objective:** Human telomerase reverse transcriptase (hTERT) enables elongation of chromosomal ends and unlimited cell replication. Our aim was to determine hTERT expression pattern in invasive squamous cell carcinoma of the skin (SCC) and in situ lesions, actinic keratosis (AK) and Bowen’s disease (BD), and to analyse it in relation to SCC differentiation, diameter and thickness.

**Method:** Immunohistochemical staining was performed on 53 AK, 14 BD and 38 SCC. hTERT expression was analysed semiquantatively in the whole lesion. Labeling index (LI) was determined in areas with the most pronounced immunopositivity.

**Results:** High hTERT expression was more frequent in AK than in BD (*p* = 0.041) or SCC (*p* = 0.002). Similar difference was found between in situ lesions (AK and BD together) and SCC (*p* = 0.002). High immunopositivity was associated with SCC thicker than 4 mm (*p* = 0.043), but not with its differentiation and diameter. Higher LI was associated with poorly differentiated SCC (*p* = 0.015), and with SCC smaller than 2 cm (*p* = 0.032). No association of LI with the type of lesion or SCC thickness was found.

**Conclusion:** Different methods of hTERT expression analysis provided various associations with in situ and invasive keratinocytic lesions characteristics.

**PS-19-036**

**Activation of Akt/mammalian target of Rapamycin/4E-BP1 signaling pathway in the pathogenesis of Merkel cell carcinoma**

T. Iwasaki ^*^, M. Matsushita, D. Nonaka, I. Murakami, M. Kato, K. Nagata, K. Hayashi

^*^Fukuoka, Japan

**Objective:** *Merkel cell polyomavirus* (MCPyV) infects up to 80 % of patients with Merkel Cell Carcinoma (MCC). In this study, we investigated the association of MCPyV infection with the activation of the AKT/mTOR/4E-BP1 signaling pathway in MCCs to elucidate the mechanisms involved in molecular pathogenesis in MCCs.

**Method:** We analyzed 39 MCPyV-positive and 26 -negative MCCs determined by qPCR. *PIK3CA* gene was sequenced directly and SNaPshot assay was performed. mRNA expressions and phosphorylation statuses of AKT/mTOR/4E-BP1 signaling pathway were analyzed.

**Results:** *PIK3CA* mutations were found in 6/34 MCPyV-positive MCCs and in 2/28 -negative MCCs. Heatmap analysis of mRNA expressions in the Akt/mTOR/4E-BP1 signaling pathway revealed different in patterns between MCPyV-positive and -negative MCCs. *TSC1*, *TSC2*, and *mTOR* mRNA expression levels were significantly higher in MCPyV-negative MCCs than in MCPyV-positive MCCs (*p* < 0.05). Phosphorylated Akt and 4E-BP1 levels were high in MCC tumor cells, being highest in mitotic cells. No significant differences in phosphorylation levels were demonstrated between MCPyV-positive and -negative MCCs.

**Conclusion:** These results suggest that MCPyV-positive and -negative MCCs may have different tumorigenic pathways and that Akt/mTOR/4E-BP1 signaling pathway signals may be novel targets for targeted therapy in MCC treatment.

**PS-19-037**

**Clinicopathological characterization of Merkel cell carcinomas in Japan and United Kingdom: RT-PCR & ISH analyses of MCPyV-infection**

M. Matsushita ^*^, T. Iwasaki, D. Nonaka, S. Kuwamoto, K. Nagata, I. Murakami, Y. Kitamura, K. Hayashi

^*^Yonago, Japan

**Objective:** Merkel cell polyomavirus (MCPyV) is a novel polyomavirus that is monoclonally integrated into genomes of up to 80 % of Merkel cell carcinomas (MCC). The aims of this study are to clarify the clinicopathological and molecular differences between Japanese and UK MCCs.

**Method:** We analyzed 43 Japanese [women:32, men:11] and 22 Caucasian (UK) [women:17, men:5] MCC cases. To detect MCPyV-DNA and mRNA expression of Small T (ST) and Large T antigen (LT), we performed real-time PCR. In situ hybridization (ISH) was also done using RNA probes targeting ST mRNA.

**Results:** Prevalence of MCPyV-infection in UK MCCs (7/22) was significantly lower than that in Japanese MCCs (34/46) (*p* = 0.001), and combined MCC and SqCC was significantly more frequent in UK than Japan (*p* = 0.0058). Overall survival was significantly longer in Japanese MCCs than in UK ones (*p* = 0.004) but MCC-specific survival was not. MCPyV-DNA quantity and LT, and ST mRNA expression levels in MCPyV-positive cases were neither different nor associated with prognosis in Japanese and UK MCCs. The details of ISH data will be shown in the congress.

**Conclusion:** We elucidated differences between Japanese and UK MCCs and established a sensitive and specific ISH method. This may be useful in practical diagnosis.

**PS-19-038**

**Aberrant expression of TTF-1 in Merkel cell carcinoma combined with squamous cell carcinoma**

P. Czapiewski ^*^, H. Majewska, P. Adam, W. Biernat

^*^Medical University of Gdansk, Dept. of Pathology, Poland

**Objective:** TTF-1 expression is a hallmark of thyroid and pulmonary epithelium and tumor developing at these sites. However, it is also present in a subset of neuroendocrine tumors. Merkel cell carcinoma (MCC) uncommonly expresses TTF-1. We attempted to evaluate TTF-1 expression in large cohort of MCCs using tissue microarray (TMA) technique.

**Method:** Twenty-three cases of MCC, including one combined with squamous cell carcinoma (SqCC), were stained with antibody against TTF-1 (Dako, IR056). Nuclear expression of TTF-1 was evaluated as positive (>1 %) or negative (0 %).

**Results:** Strong nuclear TTF-1 expression in >90 % of cells was observed in the combined MCC/SqCC tumor. The remaining 22 cases of pure MCCs did not show TTF-1 expression. Pax5 expression in this combined case was negative, what is in contrary to frequent expression of Pax5 in Merkel cell carcinoma.

**Conclusion:** We described an unusual expression of TTF-1 in combined MCC/SqCC. This expression may be an indicator of distinct pathway of neuroendocrine differentiation in combined and pure Merkel cell carcinoma. It also indicates that expression of TTF1 in cutaneous anaplastic neuroendocrine tumor may not exclude MCC diagnosis. Our study requires verification on a larger group of patients.

**PS-19-039**

**Melanocytic matricoma: Clinicopathologic features of a recently described entity**

D. R. Lujan Rodriguez ^*^, M. Moreno Valladares, O. Mateo Vico, T. Zulueta Dorado

^*^Hospital Virgen del Rocío, Dept. de Anatomía Patológica, Sevilla, Spain

**Objective:** Melanocytic matricoma (MM) is a recently described entity, this tumor is composed of matrical cells and dendritic melanocytes that mimics a normal anatomic process that takes place in the healthy bulb of an early anagen hair follicle.

**Method:** Comparison of the clinicopathologic features of MM in a new case recently diagnosed in our service with the previously reported in the literature.

**Results:** Our findings are similar to those described in all the case reports of MM published to date. It presents clinically as a small (less than 1 cm) well-circumscribed purple to black papule on sun-damaged skin in the elderly (seventh to ninth decade). Affected sites have included the nose, cheek, preauricular area, chest, back, hand, and forearm. MM presents histologically as a well-circumscribed dermal tumor showing asymmetrical pigmentation, is composed of a dual cell population including admixed epithelial matrical and supramatrical cells with shadow cell formation and pigmented dendritic melanocytes. Immunohistochemical studies for cytokeratin and β-catenin highlighted the epithelial component and studies for Melan A, HMB-45, and S-100 protein confirmed the melanocytic component.

**Conclusion:** Although clinical follow-up is quite limited, it seems that MM does not behave in a clinically aggressive manner. Further studies, however, will be necessary to determine this lesion's true biological potential.

**Melanocytic Matricoma: Clinicopathologic features:** 
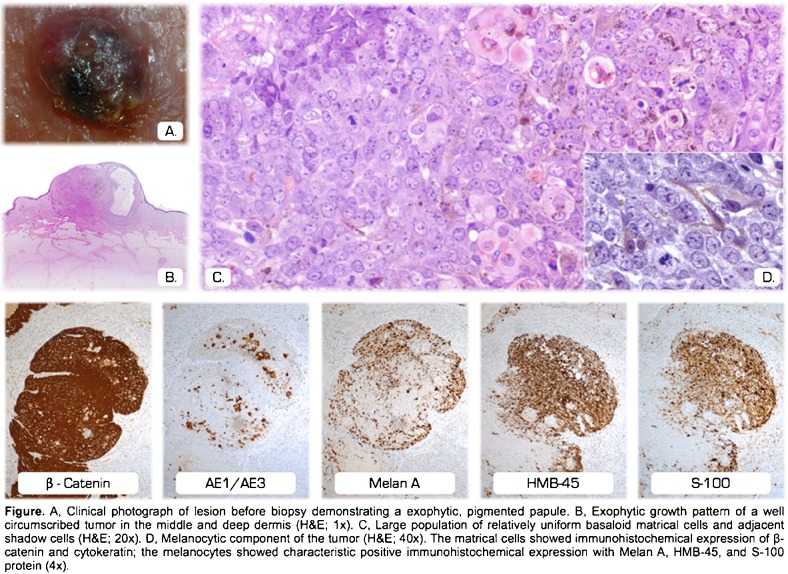


**PS-19-040**

**Melanocytic matricoma: Case report**

R. de Mora Féria ^*^, M. C. Alves, M. Campo, S. Loureiro dos Santos

^*^Hospital Dr. Fernando Fonseca, Anatomia Patológica, Lisbon, Portugal

**Objective:** In 1999 Carlson et al. proposed a new entity, the melanocytic matricoma, which would recapitulate the anagen phase of the hair cycle. Since that publication, 13 cases have been reported (one being in a dog). As far as we know, none was ever registered in Portugal.

**Method:** Case: We present a case report of a 76 years old man, with a pigmented lesion measuring 1,7 cm, localized on the skin of the forehead. On histopathologic examination it was a well-circumscribed tumour, localized within superficial dermis, with a biphasic population of melanocytes and epithelial matrical cells, with shadow cells formation. Immunohistochemistry studies with S100, HMB45 and melanA emphasized the dendritic structure of the melanocytes that were asymmetrically distributed within the lesion. The epithelial population were positive to cytokeratin AE1:AE3 and E-cadherin. There were no granulomas, calcifications or connection to epidermis or infundibula. However, it did have cysts.

**Conclusion:** There is still no consensus about the diagnostic criteria that should be used, and even on the validity of this entity being separated from matricoma, despite intense debate. The authors discuss these findings in the context of the published literature.

**PS-19-041**

**Familial ocurrence of desmoplastic trichoepitheliomas and multiple seborrheic keratoses**

A. Diaz-Lagama ^*^, R. Jiménez Peña, S. Vazquez Navarrete, M. Espinel Vázquez

^*^Hospital de La Línea, Dept. of Pathology, Cádiz, Spain

**Objective:** Desmoplastic trichoepithelioma is a uncommon benign cutaneous neoplasm which is usually present as a solitary lesion, familial desmoplastic trichoepithelioma is extremely rare.

**Method:** We describe a family in which multiple seborrheic keratoses and multiple desmoplastic trichoepitheliomas have been transmitted in some members with dominant autosomic inheritance pattern.

**Results:** The genetic predisposition of seborrheic keratoses is universally accepted. However few reported cases exist of early, numerous and family occurrence with dominant inheritance.

**Conclusion:** From our review, composite tumors associating trichoepitheliomas and seborrheic keratoses were reported, but no familial ocurrence of both tumors.

**PS-19-042**

**Tubular apocrine adenoma in the axillary area**

B. Chelly^*^, H. Azzouz, A. Zehani, I. Chelly, H. Nfoussi, S. Haouet, N. Kchir

^*^Tunis, Tunisia

**Objective:** Tubular apocrine adenoma (TAA) is a very rare benign sweat gland tumor demonstrating apocrine differentiation that typically occurs on the scalp region. Axillary location is rare. The aim of our work is to remind the clinical and pathological features of this uncommon lesion.

**Method:** We report a case of Tubular apocrine adenoma on the axillary area, diagnosed in the department of pathology, Rabta hospital, Tunis, Tunisia.

**Results:** We report a case of a 45 years old women presenting a painless tumor of the left axilla, which had slowly enlarged since he first noticed it six months earlier. Physical examination revealed a slightly soft, dome shaped tumor. The tumor was resected and histological study concluded to a tubular apocrine adenoma with tubular structures that have a double layered epithelial lining. The peripheral epithelial layer consists of cuboidal cells and the luminal layer of columnar cells that demonstrate decapitation secretion.

**Conclusion:** Tubular apocrine adenoma is a benign dermal adnexal neoplasm commonly occurs on the scalp region. Axillary localization is rare. Some neoplasm may occur in association with a syringocystadenoma papilliferum and can also arise within an organoid naevus.

**PS-19-045**

**Immunohistochemical maspin expression in skin appendage tumors**

T. Ranchal ^*^, M. J. Fernandez-Aceñero, J. P. Alameda, M. Llanos Casanova, S. Chaves, J. I. Cornejo

^*^Fundacion Jimenez Diaz, Surgical Pathology, Madrid, Spain

**Objective:** Maspin is an important metastasis suppressing gene, whose expression plays an important role in the acquisition of metastatic potential of the cells. The aim of the present study is to analyze immunohistochemical expression of Maspin in trichoepitheliomas, cylindromas and eccrine spiradenomas, showing a lack of CYLD gene.

**Method:** We have performed immunohistochemical detection of maspin in paraffin-embedded tissue from 5 spiradenomas, 5 cylindromas and 5 trichoepitheliomas retrieved from our files. We have also performed cytokeratins 5, 14 and 10 in the same tumors.

**Results:** We have found a strong reduction in Maspin expression in the three kind of tumors compared with normal epidermis and hair epithelium. Also keratin 5 and 10 expression was reduced in all the tumors compared with adjacent normal epithelia.

**Conclusion:** Our results are consistent with the experimental findings in cell culture models and confirm the reduction in Maspin expression in cylindromas and related tumors, that seems related to the lack of the deubiquination function of CYLD.

**PS-19-046**

**Aggressive patterns in basal cell carcinoma: Underdiagnosed enemies in small incisional biopsies**

S. Zurac ^*^, C. Popp, R. Andrei, T. Tebeica, C. Socoliuc, A. Birceanu, A. Stoica, V. Chitu, D. Boda, S. Tiplica, F. Staniceanu

^*^Colentina University Hospital, Pathology, Bucharest, Romania

**Objective:** Basal cell carcinoma (BCC) represents a group of malignant cutaneous tumors with a significant microscopic heterogeneity. Some patterns are considered to have more aggressive behavior. Since the new emerging non-surgical treatments of BCC, the impact of microscopic pattern of a biopsy-diagnosed BCC on therapeutical decision is increasing. This study is evaluating how probable is to miss an agressive pattern of a BCC if the diagnosis is performed on a small biopsy before an unsurgical treatment.

**Method:** We studied 150 consecutive BCCs diagnosed in our department during 6 months (106 surgical specimens and 44 biopsies): at least two different pathologists evaluated each case and noted all patterns. Infiltrative, micronodular, morphoeic and metatypical patterns were considered aggressive.

**Results:** 18.18 % of the biopsies and 30.19 % of the surgical specimens. (*p* = 0.04) had aggressive patterns. The sensitivity of biopsy was 60 %, while the negative predictive value was of 85 %. The patient with higher risk of having an undiagnosed aggressive pattern is an older patient with large, ulcerated lesion on non-photo-exposed areas.

**Conclusion:** The possibility of underestimate the aggressiveness of a BCC on a biopsy specimen is significant, and should always be kept in mind during treatment decisions and long-term surveillance. Zurac and Popp have equal contribution.

**PS-19-047**

**Primary cutaneous sarcomatoid carcinoma**

B. A. Kirmizi ^*^, A. Okcu Heper, H. Ozakinci, B. Kaya

^*^Ankara University, Pathology, Turkey

**Objective:** Primary cutaneous sarcomatoid carcinoma or carcinosarcoma is a rarely diagnosed skin cancer, with fewer than 70 reported cases in the literature. It is a biphasic tumour with malignant epithelial and mesenchymal components.

**Method:** Here we present, a 53-year-old man with a 2- year history of 5 cm mass at the scalp, growing rapidly in the past 3 months. The lesion was totally excised.

**Results:** Histological examination revealed a tumour ulcerating the epidermis and filling the dermis. Superficial part of the tumour was compromized of basaloid solid epithelial islands, admixed with large, bizaar, mitoticaly active, oval-spindle shaped cells arranged in solid sheets and extending deep throughout the dermis. Immunohistochemistry revealed that pleomorphic cells were diffusely vimentin, focally p63 positive, and keratin negative. Whereas, the basaloid cells were diffusely keratin positive, vimentin negative.

**Conclusion:** In cutaneous carcinosarcoma the epithelial component is typically basal or squamous cell carcinoma. The mesenchymal component may have heterolog components as osteosarcoma or chondrosarcoma. Immunohistochemistry can be crucial for differantiating these tumours from, spindle squamous cell carcinoma, malignant melanoma or atypical fibroxanthoma with pseudoepitheliomatous hyperplasia. Also the epithelial component can be so small that extensive sampling is required for the correct diagnosis.

**PS-19-048**

**Exploring connexin expression in melanocytic tumors**

G. Kiszner ^*^, I. Teleki, N. Meggyeshazi, P. Balla, M. E. Maros, I. B. Nemeth, E. Varga, I. Korom, T. Krenacs

^*^Semmelweis University, 1st Dept. of Pathology, Budapest, Hungary

**Objective:** Connexins (Cx) form transmembrane channels which can transport small hydrophilic molecules between adjacent cells or communicate with the extracellular environment. They also interact with intracellular regulatory proteins. Connexins contribute to the maintenance of multicellular homeostasis. Their altered expression can be observed in many neoplasms, however, melanocytic tumors were rarely examined.

**Method:** We tested expression of connexin isotypes in cutaneous melanocytic tumors using immunohistochemistry in tissue microarrays of 68 benign nevi and 77 malignant melanomas as well as in human melanoma cell lines.

**Results:** Cx43/GJA1 appeared as punctuate membranous plaques in 60 % of nevi and were weakly expressed or missing in most melanomas. Cx30.3/GJB4 reaction localized to cell membrane in the superficial regions of 70 % of nevi and the reaction was cytoplasmic in 28 % of melanomas. Cx36/GJD2 immunostaining was observed in 54 % of nevi and 29 % of melanomas. All melanocytic tumors expressed Cx26/GJB2. The reaction was diffuse and cytoplasmic in 33 % of melanomas and paranuclear in most nevi (77 %). All these expression differences between nevi and melanomas were statistically significant (*p* < 0.001).

**Conclusion:** Our results demonstrate that several previously undetected connexin isotypes can be found in melanocytic tumors. Most tested connexins are significantly down-regulated or delocalized in melanomas, which possibly contribute to their malignant phenotype.

Tuesday, 3 September 2013, 09.30–10.30, Pavilion 2

**PS-20 Poster Session Digestive Diseases Pathology III: Liver/Biliary**

**PS-20-001**

**Mucinous cystic neoplasm of the liver with ovarian-type stroma: A challenging diagnosis**

E. Chelbi^*^, S. Sassi, G. Sahraoui, F. Khanchel, E. Ben Brahim

^*^Med Tahar Maamouri Hospital, Pathology, Nabel, Tunisia

**Objective:** Mucinous cystic hepatic neoplasms are rare cystic multilocular lesions lined by cubo-columnar epithelium and associated with an ovarian-type stroma. The epithelium can be minimally atypical or highly dysplasic or carcinomatous. Here we describe a case of hepatic mucinous cystadenoma with an ovarian-type stroma mimicking cystic leiomyosarcoma.

**Method:** The patient, a 45-year-old-woman, presented 7 years ago with abdominal pain and an impression of hydatic cyst measuring 15 cm on abdominal ultrasound. She undergo a needle aspiration and a surgical biopsy.

**Results:** Microscopic examination revealed a dense fusiform cell proliferation with mitotic activity and no epithelium. The cells express caldesmon, actin, desmin, vimentin but not cytokeratins nor CD117. A cystic leiomyosarcoma was suspected. The patient was then lost of view. Currently, the patient presents abdominal pain related to a multicystic hepatic mass compressing the inferior vein cave. A microscopic review and further immunohistochemical study showed strong stromal expression of oestrogen receptors and progesterone receptors with the emergence of a cuboidal epithelium on immunohistochemical sections confirming thus mucinous cystadenoma diagnosis.

**Conclusion:** Mucinous cystic neoplasm can radiologically mimic hydatid cyst mainly in endemic areas. The pathological diagnosis can be difficult in surgical biopsy showing exclusively dense and mitotic stroma without epithelium. Immunohistochemstry is than very helpful.

**PS-20-002**

**Calcifying nested stromal-epithelial tumor of the liver: An unusual tumor of uncertain histogenesis**

N. Dias ^*^, P. Rodrigues, C. Oliveira, M. A. Cipriano, M. F. Xavier Cunha

^*^Coimbra, Portugal

**Objective:** Calcifying Nested Stromal-Epithelial Tumor (CNSET) is rare and recently recognized; it has uncertain histogenesis and behavior.

**Method:** A 20 years-old woman (1997) presents with irresectable solid tumor in the right lobe and short history of weight loss, abdominal pain, jaundice, portal hypertension and progressive hepatic failure requiring liver transplantation; died from surgical complication. She had history of Fallot’s trilogy corrected at age 5, when a 5 cm liver nodule was diagnosed by ultrasound as hemangioma. Macroscopy: 22 cm well-circumscribed, hard, whitish tumor. Histology: nests of round/oval/spindled bland cells with psammoma calcification, osseous metaplasia, surrounded by cellular/loose stroma with bile ductules. The diagnosis was “probably mixed epithelial-mesenchymal hepatoblastoma (MEM)–unusual teratoid features”.

**Results:** The entity was first reported in 2001 with average 30 published cases. It occurs more commonly in females within 2–14 years; the size varies from 2 to 20 cm. Described associations: Cushing and Beckwith-Wiedemann syndromes, Wilms tumor. Differencial diagnosis with MEM and Desmoplastic Small Round Cell Tumor is crucial due to prognostic/therapeutic implications. A relationship to mixed blastomatous pediatric tumours is uncertain; like hepatoblastomas, the epithelioid nests have nuclear positivity for beta-catenin suggesting a pathogenic role for this gene.

**Conclusion:** Low-grade malignancy with characteristic diagnostic features: neoplastic nests with spindle/oval cells, variable calcification/ossification and stroma.

**PS-20-003**

**Primary malignant mesothelioma of the liver**

T. Zogka^*^, C. Zorzos, I. Margaris, G. Koutsonikas, G. Kakiopoulos

^*^General Hospital of Athens, Surgical Pathology, Greece

**Objective:** We report a case of primary hepatic mesothelioma in a 68–year -old man, with no known history of hepatic or neoplastic pathology.

**Method:** Computed tomography showed an ill defined, intraparenchymal tumor obliterating most of the right lobe of the liver. Surgical excision of the diseased lobe was performed. At our laboratory lobectomy specimen was massively infiltrated by a brown partly hemorrhagic tumor, with soft consistency,with soft consistency, measured 18 × 15,5 × 8 cm, extending to the capsular surface.

**Results:** Microscopically the tumor was composed of tubular or cord like arrangements of epithelioid cells with eosinophilic cytoplasm and atypical nuclei. Immunohistochemically, tumor cells were positive for EMA, CK-7, Vimentin (weakly), Calretinin and CK 5/6, but negative for other hepatocellular, neuronal, lymphoid or germ-cell markers.

**Conclusion:** These findings were consistent with our diagnosis of primary malignant epithelioid mesothelioma of the liver (epithelioid type). To the best of our knowledge, this is the third reported case of localized malignant primary mesothelioma arising in the liver.

**PS-20-004**

**Primary hepatic solitary fibrous tumour with malignant transformation: A case report and review of current literature**

A. Silvanto ^*^, N. Karanjia, I. Bagwan

^*^Royal Surrey County Hospital, Dept. of Histopathology, Guildford, United Kingdom

**Objective:** Extrapleural solitary fibrous tumour (SFT) is an uncommon mesenchymal neoplasm, presenting most commonly in the lung, but which has been reported at numerous extrathoracic locations. The majority of SFTs are benign, but 10–15 % behave aggressively. We report a case of primary hepatic SFT with malignant transformation.

**Method:** The authors present a case of primary hepatic SFT in a 65-year old man, describing the clinical presentation and histopathological features, highlighting features predictive of a more aggressive/malignant behaviour. The authors also review the current literature regarding hepatic SFTs.

**Results:** This case of primary hepatic SFT was diagnosed incidentally during clinical examination and a subsequent CT scan demonstrated a large liver mass. Histology following a hemihepatectomy showed a solitary fibrous tumour with morphological patterns ranging from benign to areas of malignant transformation, including pleomorphism, increased cellularity, herringbone pattern, necrosis and a raised mitotic count. On review of literature, only an occasional case report of malignant transformation in a hepatic SFT was identified.

**Conclusion:** This case highlights that SFT should be included in the differential diagnosis of a hepatic spindle cell lesion, and that on rare occasions, malignant transformation can occur in this already uncommon neoplasm.

**PS-20-005**

**Atypical biliary adenofibroma of the liver: An unusual tumor with malignant potential**

V. Trak-Smayra ^*^, C. Kesrouani, V. Paradis, G. Abadjian, C. Sader-Ghorra

^*^Hotel Dieu de France Hospital, Dept. of Pathology, Beirut, Lebanon

**Method:** A 69 year old female patient was admitted to our hospital for the surgical resection of a 16 cm hydatid cyst of the right liver lobe. She was known to have cervical squamous cell carcinoma, in remission. During surgery, an unexpected lesion of the left liver lobe was discovered and excised.

**Results:** Gross examination of this lesion revealed a relatively well-delineated nodule, with white, microcystic cut surfaces, measuring 1.3 cm. Microscopic examination showed a proliferation of tubulo-cystic structures, some with cribriform pattern, surrounded by a fibrous stroma and lined by a biliary epithelium showing cytonuclear atypia and few mitotic figures. The lesion was globally well circumscribed, with no evidence of infiltration of the surrounding liver parenchyma. Immunohistochemical stains revealed positivity of the tumor cells for cytokeratin 7 and 19, CEA and Filamin A and focally for CDX2 and p53. The proliferation index was high. The diagnosis of atypical biliary adenofibroma was retained.

**Conclusion:** Biliary adenofibroma is a rare neoplasm of the biliary ducts with malignant potential. Cases of malignant transformation and metastasis have been reported. Cytonuclear atypia, high proliferative index and immunoreactivity for p53 and Filamin A, observed in this case, are features of aggressive behavior and warrant close follow up of the patient.

**PS-20-006**

**Acute alcoholic liver disease presenting as an hepatic pseudotumor: A case report**

A. Figueiredo ^*^, A. M. Carvalho, A. Lázaro, A. Murinello

^*^Dept. of Pathology, Hospital Curry Cabral, Lisboa, Portugal

**Objective:** Alcoholic liver disease is very prevalent, being acute alcoholic hepatitis (AAH) a potentially life-threatening situation. The presentation of the latter as a hypervascularized liver mass on computed tomography (CT) has been reported, but less than 10 case reports are found in the literature. The aim of this case report is to describe a pseudotumoral liver presentation of AAH.

**Results:** A 43 year-old man with heavy daily alcoholic intake was admitted at the Emergency Department due to loss of body weight, ascites, edema of the lower limbs, coluria and hepatomegalia. His blood tests revealed increased liver parameters and the CT showed two liver masses measuring 5,3 and 18 cm, hypodense and hypocaptating. A diagnosis of hemangioendothelioma was suggested. Liver biopsy revealed cirrhosis and acute alcoholic hepatitis. He was successfully treated with corticosteroids.

**Conclusion:** AAH presenting as a liver mass is a very unusual but possible situation, so that it must be considered when assessing patients and biopsies.

**PS-20-007**

**A case of primary mucoepidermoid carcinoma of the liver with the CRTC1-MAML2 fusion transcript detected**

J. Watanabe ^*^, A. Deguchi, N. Mizukami, M. Hiraki, H. Yamamoto, H. Yano

^*^Yame General Hospital, Dept. of Pathology, Japan

**Objective:** Primary mucoepidermoid carcinoma (MEC) of the liver is a rare type of intrahepatic cholangioma. We examined a case of MEC of the liver with the CRTC1-MAML2 fusion transcript detected.

**Method:** A 79-year-old female. On dynamic study, a 5-cm tumor showed ring enhancement on CT and MRI. Since no primary lesion was detected by PET, the diagnosis was intrahepatic cholangioma. Lobectomy was performed.

**Results:** The tumor had a whitish cut surface. Histologically, the tumor had no capsule. It was composed of a proliferation of atypical epidermoid cells forming solid and nest growth patterns with little fibrous stroma. Focal keratinization and intercellular bridge were observed. The tumor also contained cells with intracytoplasmic mucin and ductular elements with mucin secretion. Immunohistochemically, the tumor cells were positive for cytokeratin (CK)5/6, CK7, CK19 and involucrin, but negative for CK8 and hepatocyte. Furthermore, CREB-regulated transcription coactivator 1 (CRTC1) at 19p13 and Mastermind-like 2 (MAML2) fusion gene transcript at 11q21 were detected by RT-PCR in paraffin-embedded tissue. Based on these findings, we diagnosed this case as mucoepidermoid variant of intrahepatic cholangioma.

**Conclusion:** It was the first case of primary MEC of the liver in which the CRTC1-MAML2 fusion transcript was detected.

**Histology:** 
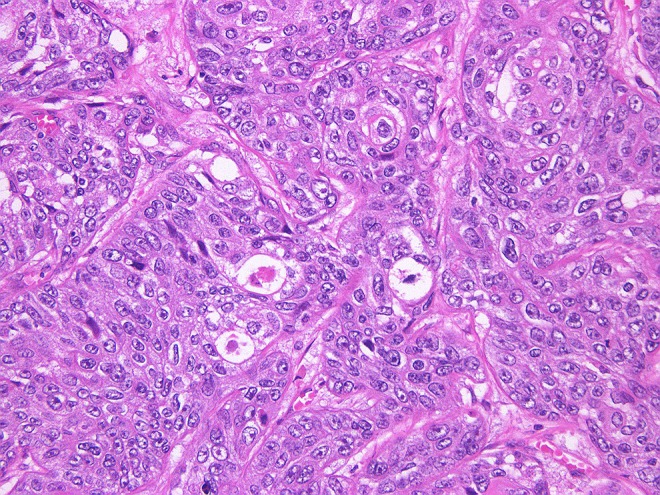


**PS-20-008**

**The invisible side of liver ischemia**

I. Delladetsima ^*^, S. Sakellariou, E. Mahera, D. Tiniakos

^*^Medical School Athens, 1st Dept. of Pathology, Greece

**Objective:** To investigate parenchymal changes in ischemic liver conditions.

**Method:** We studied 7 cases of hepatic venous congestion, 5 of which with additional nodular regenerative hyperplasia and 3 cases with non-specific changes exhibiting areas of plate atrophy (biopsies and surgical specimens). Metaplastic changes, hepatocyte proliferation, stellate cell activation, and capillarization of sinusoids were examined immunohistochemically with antibodies to keratins (K) 7 and 19, Ki67, αSMA and CD34, respectively.

**Results:** In venous congestion, K7 was expressed in zone 3 hepatocytes, either segmentally in mild cases or diffusely in more severe cases. In two cases, a portal-central distribution was additionally observed, analogous to acinar zone 3 topography. K7 immunoexpression was accompanied by capillarization of sinusoids. Diffuse stellate cell activation, highlighted by prominent αSMA expression, was noted in K7-positive areas. Similar results were obtained in isolated plate atrophy and at the periphery of regenerative nodules. Ki67 was negative in K7-positive areas.

**Conclusion:** Ischemic parenchymal changes are characterized by hepatocyte K7 immunoexpression, sinusoidal capillarization, stellate cell activation and lack of cellular proliferation, indicating a new microenvironment associated with a more resistant hepatocellular phenotype. K7 immunohistochemistry may prove of value in confirming lesions of hepatic ischemia.

**PS-20-009**

**Multiple sclerosis and drugs: Dangerous association for the liver?**

P. Rodrigues ^*^, N. Dias, C. Oliveira, M. J. Martins, M. R. Silva, M. A. Cipriano, M. F. Xavier Cunha

^*^CHUC, Dept. de Anatomia Patológica, Coimbra, Portugal

**Objective:** Drug induced liver injury (DILI) is a frequent and underestimated cause of acute hepatitis that sometimes lead to liver failure.

**Method:** Report of two female patients, with multiple sclerosis (MS) aged 34 and 39 years, the first with 8 months evolution, ANA+, polymedicated, treated with methylprednisolone (MT) pulses for disease exacerbations. She experienced two episodes of severe hepatitis, the last under MT alone. The second had disease history of 8 years, in the last one treated with interferon (IFN)-β1a, paroxetine and diazepam, developed hepatic liver failure (ALF) requiring liver transplantation. Both showed dense lymphoplasmocytic inflammation, periportal and lobular/perivenular with bridging necrosis.

**Results:** MS is an autoimmune disease with exacerbations that can be treated with MT pulses and IFN, sometimes in a set of polymedication. MT pulses are linked with severe hepatotoxicity occasionally with prolonged latency suggesting immune rebound phenomenon. The immunomodulating effects of IFN-β1a could trigger an autoimmune response, but evolution to ALF is uncommon.

**Conclusion:** Patients with MS should be checked for liver function tests and autoantibodies and, under treatment with corticosteroid and IFN, screened for potential liver injury until several weeks after drug exposure.

**PS-20-010**

**Acute hepatitis E virus infection as a cause of graft hepatitis**

M. Kollár ^*^, E. Sticová, S. Franková, E. Honsová

^*^IKEM, Dept. of Pathology, Prague, Czech Republic

**Objective:** Hepatitis E virus (HEV) infection is common in developing countries, but the incidence in the US and Europe is considered low. Progressive chronic infection has been identified almost exclusively in immunocompromised persons, mainly organ transplant recipients.

**Method:** We present 2 cases of acute HEV infection in liver transplant recipients. Both patients underwent a liver graft biopsy due to abnormal liver tests. The first patient underwent liver transplantation (OLT) for hepatitis B associated cirrhosis, and elevated level of transaminases appeared 4 years after OLT. The second patient underwent OLT because of hepatocellular carcinoma in alcoholic cirrhosis, and liver test abnormalities occurred 2 months after transplantation

**Results:** Both biopsy samples showed similar morphological features, predominantly with lobular necroinflammatory activity, which were descriptively classified as “nonspecific hepatitis” and a laboratory search for infection was recommended. Both patients had IgM and IgG anti-HEV antibodies and positive HEV RNA tests (genotype 3f and 3 g). Treatment with ribavirin led to normalization of liver tests and a non-detectable level of HEV RNA.

**Conclusion:** Acute HEV infection extends the differential diagnosis of “nonspecific” hepatitis in immunocompromised patients. Early diagnosis followed by treatment can lead to the eradication of the virus, and prevent progressive chronic disease.

**PS-20-012**

**Clinical implication of variations in the Liver Fibrosis index (LF-index) as assessed by tissue elastography within each pathomorphological stage in patients with chronic hepatitis C**

K. Furuya ^*^, T. Maeda, K. Kito

^*^Ehime Prefectural Hospital, Dept. of Pathology, Matsuyma, Japan

**Objective:** Tissue elastography is a non-invasive method for chronic hepatitis staging; however, LF-index values within each pathomorphological stage vary widely. We evaluated the clinical implications of the range of LF-index values.

**Method:** Liver elasticity, represented by the LF-index, was evaluated using tissue elastography (FibroScan) in 57 patients with or without chronic hepatitis C. Patients then underwent liver biopsy or resection, and microscopic staging was performed using Mallory stain slides.

**Results:** The mean values of LF-index were 1.9 ± 0.4 for normal liver, 2.3 ± 0.5 for stage F1, 2.5 ± 0.4 for stage F2, 3.5 ± 0.8 for stage F3, and 3.9 ± 1.0 for stage F4. The range of values were 1.9−3.2 for stage F1, 2.1−3.1 for stage F2, 2.6−5.4 for stage 3, and 2.9−5.8 for stage 4. The overlap of values between consecutive stages was related to the staging definition; fibrosis was staged according to the pathomorphological pattern, not the absolute amount of scar tissue.

**Conclusion:** Variability in LF-index values within each stage, which reflects at least in part the difference in the absolute amount of scar tissue, may be useful for predicting the effectiveness of scar matrix degradation therapy.

**PS-20-014**

**Detection of Langerhans cell infiltrate in liver biopsies for primary biliary cirrhosis and chronic viral hepatitis**

E. Ben Brahim ^*^, R. Jouini, M. Sabbah, N. Abdessayed, W. Koubâa, I. Msakni, O. Khayat, M. Belhaj Salah, A. Souissi, D. Gargouri, A. Chadli

^*^Habib Thameur Hospital, Pathology, Tunis, Tunisia

**Objective:** Langerhans cells (LC) play a role in the pathogenesis of primary biliary cirrhosis (PBC) and chronic viral hepatitis (CVH), by initiating specific T-cell responses. The aim of this study is to evaluate the presence of these cells in 26 liver needle biopsies using a monoclonal antibody, CD1a.

**Method:** Liver biopsy from adult patients diagnosed with PBC (*n* = 9), CVH-C (*n* = 8) and CVH-B (*n* = 9) were reviewed. An immunohistochemical stain for CD1a was used to detect LC, and their distribution was recorded as lobular, portal, and intraepithelial.

**Results:** Distribution of LC was heterogenous and sparse in most cases. Intraepithelial LC were more readily identified in CVH-C (*n* = 4) and were rare in other conditions. Portal LC were most frequent in PBC (*n* = 8). A similar distribution was evident for lobular LC in PBC (*n* = 5) and CVH-C (*n* = 4), although it tended to be rare in CVH-B (*n* = 2).

**Conclusion:** The detection of LC by CD1a is more frequent in CVH-C and PBC, where they play an important role in initiating specific T-cell response. Their high level in portal infiltrate in PBC could support hypothesis of their role in pathogenesis of this disease, but their diagnosic value was not evaluated needing more cases and similar studies.

**PS-20-015**

**Hepatocarcinogenesis in a cirrhotic microenvironment: Experimental model for the study of human hepatocellular carcinoma**

T. Lima ^*^, C. Portela, C. Araujo, M. Dagli, B. Cogliati, F. Blazquez, J. Silva

^*^Biomedical Sciences Institute, Cell and Developmental Biology, São Paulo, Brazil

**Objective:** The objective of this study was evaluate the role of thioacetamide (TAA) as an induction agent for hepatocarcinogenesis leading to tumor development in a cirrhotic environment.

**Method:** Adult male Wistar rats were injected i.p. with TAA 3 times/week by 14, 21, and 35 weeks. Liver tissues were pretreated with anti-GST-P immunohistochemistry followed by HE staining methods. Preneoplastic lesions were identified by GST-P positive areas and classified as persistent or remodeling. Apoptosis was quantified by number of apoptotic bodies/1,000 cells using optical microscope (40×).

**Results:** As expected, animals treated with TAA showed an increased number of apoptotic bodies [22.8 ± 15.7] when compared to control groups [0.5 ± 0.77]. Furthermore, 21 weeks of TAA injections led to an increased number of apoptotic bodies when compared to 35 weeks (*P* < 0.01); however, this increase was not significant compared with 14 weeks. Despite remodeling lesions and surrounding tissue have shown high amount of apoptotic cells, apoptosis was more pronounced within persistent lesions (*P* < 0.001).

**Conclusion:** These data show that 21 weeks of TAA injections induce cellular injuries that trigger the initiation of hepatocarcinogenesis a fact that was inferred by both activation of apoptosis in liver cells and because the presence of preneoplastic lesions.

**PS-20-016**

**Characteristics of steatohepatitic hepatocellular carcinoma in a series of predominantly virus-related cirrhosis**

U. Aykutlu ^*^, A. Uguz, Ö. Ünalp, D. Nart, Z. Karasu, M. Sözbilen, F. Yilmaz

^*^Ege University, Dept. of Pathology, Izmir, Turkey

**Objective:** Steatohepatitic hepatocellular carcinoma (SH-HCC) is described as a subtype of hepatocellular carcinoma (HCC) related with cirrhosis due to metabolic syndrome and HCV. We document the characteristics of SH-HCC in a series of predominantly viral etiology.

**Method:** Explanted livers of 90 cases with cirrhosis+HCC were retrospectively reviewed both histopathologically and clinically. Tumors were classified as either classical HCC (C-HCC) or SH-HCC based on their predominant (>50 %) histopathological pattern.

**Results:** The SH-HCC variant represented 20 % (18/90) of cases. Male: female ratio was 3,5 in SH-HCC (versus 7 in C-HCC), whereas median age was similar in both groups. Etiology of cirrhosis in SH-HCC cases were as follows; HBV and/or HDV (78 %), HCV (11 %), alcohol(5,5 %) and HBV+NASH (5,5 %). Among the SH-HCC cases, mean body mass index (BMI) was 26,24, median BMI was 27,05 (range, 19,40–32,50) and diabetes mellitus(DM) was present in 50 %. Tumor was macroscopically unifocal nodular, multifocal <5 nodules and multifocal >5 nodules in 17 %, 66 % and 17 %, respectively. Tumor size was larger SH-HCC versus C-HCC cases(5,46 cm versus 4,63 cm).

**Conclusion:** SH-HCC is not rare among HBV-related HCC, but high mean BMI and frequent occurence of DM in this series supports the combination of metabolic and viral mechanisms.

**PS-20-017**

**miR expression profile in Sorafenib treated hepatocellular carcinomas**

B. Gyöngyösi ^*^, É. Végh, G. Bodoky, B. Járay, E. Székely, Z. Schaff, A. Kiss

^*^Semmelweis University Budapest, 2nd Dept. of Pathology, Hungary

**Objective:** We aimed to investigate pretreatment miR expression profile of fine needle aspiration biopsy (FNAB) hepatocellular carcinoma (HCC) samples followed by Sorafenib treatment in comparison to HCCs without Sorafenib.

**Method:** 15 HCC patients with Sorafenib treatment until progression and not treated 10 HCC patients were analyzed. Sorafenib patients were devided into slow (treated >9 months) and fast (treated <9 months) progression subgroups. Expression of miR-17-5p, mir-18a, miR-21, miR-34a, miR-122, miR-195, miR-210, miR-214, miR-221, miR-222, miR-223, miR-224, miR-140, miR-328 and U6 was analyzed.

**Results:** Pretreatment miR-195 and miR-214 expressions were significantly higher in Sorafenib treated patients in comprison to not treated ones. Fast progressing HCCs under Sorafenib did not show significant difference in miR expression when compared with not treated patients, except for miR-195. Expression of miR-195 was significantly higher in fast progressing Sorafenib treated group in comparison to not treated HCCs. Interestingely, Sorafenib treated patients with high pretreatment miR-224 expression showed slow progression (*p* = 0.04) and higher overall survival (*p* = 0.03).

**Conclusion:** Our results suggest that pretreatment mir-122 expression does not predict sensitivity to Sorafenib treatment. High pretreatment miR-224 expression was associated with slow progression and longer overall survival suggesting that miR-224 might predict the success of Sorafenib treatment.

**PS-20-018**

**Challenging even rarer intrahepatic cholangiocarcinoma: Epstein-Barr virus-associated lymphoepithelioma-like carcinoma. Report of a case**

L. Dimitrova ^*^, A. Vlahova, S. Ivanova, S. Hristova

^*^Medical University Sofia, Dept. of General and Clinical Pathology, Bulgaria

**Objective:** Intrahepatic cholangiocarcinomas (ICC) are hepatic malignancies with biliary differentiation. They represent 5–15 % of primary liver cancer and are relatively infrequent in European population. Several histologic subtypes of ICC are currently WHO 2010 recognised. We present a case of a rare variant of this tumor.

**Method:** A 61-year-old female patient presented with intrahepatic mass measuring 6.5/4/3.5 cm, infiltrating the wall of the gallbladder and retroperitoneal metastatic lymph nodes. The partial hepatectomy surgical specimen was found to contain ill-defined, white-tan, necrotic tumor tissue. Microscopically the tumor was composed of polymorphic cells with vesicular nuclei arranged in trabecular and syncitial pattern dispersed in abundant, lymphocyte-rich fibrous stroma.

**Results:** Imunohistochemically tumor cells stained positively for CKAE1-AE3, EMA, CK19 and EBV-coded transcripts by ISH; the stromal component turned out to contain predominantly CD3+ T-lymphocytes, fewer CD20+ B-lymphocytes and histiocytes. Ancillary studies showed reciprocal expression of E-cadherin and HER-2 with strong p53 expression in cytokeratin-positive component of the tumor.

**Conclusion:** We report this case of lymphoepithelioma-like ICC as a diagnostic challenge because of its rarity (only 13 cases are described in English literature) and offer further discussion about prognosis and treatment.

**PS-20-019**

**Clinicopathological and immunohistochemical evaluation of cholangiocarcinoma, cholangiolocarcinoma, hepatocellular carcinoma and mixed hepatocellular carcinoma and cholangiocarcinoma**

N. Bassullu^*^, I. Turkmen, I. Coban, N. Guler, M. Dayangac, M. Akyildiz, O. Yaprak, Y. Yuzer, Y. Tokat, G. Bulbul Dogusoy

^*^Istanbul Bilim University Medi, Dept. of Pathology, Turkey

**Objective:** We aimed to investigate histopathological, immunohistochemical (IHC) features and survival of cholangiocarcinoma (CC), cholangiolocellular carcinoma (ColC), hepatocellular carcinoma (HCC) and mixed HCC-CC (m-HCC-CC).

**Method:** Histopathologic features were assessed and after immunohistochemistry (CK7, CK19, CD56, Hep Par 1, p53 and CD117) the diagnosis of CC was given in 8 cases, ColC 2 cases, m-HCC-CC 3 cases (a total of 13 cases). These cases were correlated with 50 cases of HCC (totally 63 patients). The correlations between expressions of these markers and pathologic characteristics, survivals were analyzed statistically.

**Results:** After IHC, 46 cases (73 %) remained unchanged as HCC. However 17 cases (5 cases m- ColC-CC, 2 ColC, 4 CC, 3 m-HCC-CC, 2 m- HCC-ColC-CC and 1 mixed and synchronous HCC-ColC-CC) were evaluated as non-HCC (27 %). There are significant differences between the two groups with histopathological and immunohistochemical findings (*p* < 0.05), but no significant difference in survival was observed.

**Conclusion:** There are significant differences HCC and other liver carcinomas except survivals so we recommend for the correct diagnosis of liver carcinomas, if the dominant pattern appears to be glandular and multiple tumors show different pattern, not only Hep Par 1, but IHC panel including CK7, CK19, p53, and CD56 should be applied to these cases.

**PS-20-020**

**MMP-9 and TIMP-1 in liver metastases**

E.-R. Avadanei ^*^, I.-D. Caruntu, C. Amalinei, D. Ciobanu, S.-E. Giusca

^*^UMF Grigore T. Popa Iasi, Dept. of Histology, Romania

**Objective:** The present study aims to assess the immunohistochemical expression of MMP-9 and TIMP-1 in secondary liver tumors and to correlate the expression with the clinical profile, focusing on the post-therapy survival (overall survival, disease free survival).

**Method:** The study included 24 cases diagnosed with liver metastases analyzed by routine histopathology and by immunohistochemistry. The expressions of MMP-9 and TIMP-1 were quantified as scores obtained from the percentage of positive cells (P) and the staining intensity (I). The data obtained were statistically analyzed using SPSS software.

**Results:** MMP-9 and TIMP-1 expressions were detected in 66.66 % and 70.83 % of the specimens, respectively. The correlation analysis between MMP-9 and the two time-dependent tracked variables showed statistically significant values (*p* = 0.041 and *p* = 0.030, respectively). The correlation analysis between TIMP-1 and two time-dependent tracked variables showed statistically significant values of post-therapy survival in metastatic disease (*p* = 0.05) and without statistical significance of disease-free interval (*p* = 0.22).

**Conclusion:** Our results support the microenvironmental influences on metastatic host site and the value of MMP-9 and TIMP-1 immunohistohemical expression as unfavorable prognosis factors.

**PS-20-021**

**Pediatric liver tumors: Anatomo-clinical study about 20 cases**

S. Ait-Younes ^*^, D. Chilla, F. Z. Benserai, F. Asselah

^*^Mustapha Bacha Hospital, Alger, Algiers, Algeria

**Objective:** Determine the anatomoclinical profile of pediatric liver tumors in our population and compare our results with literature database.

**Method:** Reevaluation from the paraffin material and clinical files of 20 cases of pediatric liver tumors collected in our department over 6 years; we used usual histology and IHC (selected cases).

**Results:** 20 cases reported; 11 benignant tumors and 9 malignant tumors; -Benignant lesions: hemangioma:*n* = 1, mesenchymal hamartoma:*n* = 9, Focal Nodular Hyperplasia: *n* = 1; mean age: 4 years, range: 11,5–10 y; sex-ratio = 1,5–Malignant tumors: hepatoblastoma:*n* = 8, mean age = 2,6 y range: 1–4 y, sex-ratio = 5; one case of indifferenciated tumor (male, 8 years)–Most children present with abdominal distension, a palpable abdominal mass,or both; ultrasonography and tomodensitometry were used to determinate the location, size, extent and to characterize the consistency as cystic or solid, alpha fetoprotein (AFP) levels are elevated in 50 %.

**Conclusion:** Hepatic tumors and pseudotumors offer a diagnostic challenge to the clinician and pathologists seeing only occasional cases, in this series the most common tumors were hamartomas and hepatoblastomas; the contribution of the pathologist is to determine the diagnosis and evaluate the quality of the resection. Our results regarding anatomo-clinical features are in concordance with litterature database.

**PS-20-022**

**Pancreatoblastoma in an adult: A case report**

J. Jotanovic ^*^, A. Djikic Rom, A. Karamarkovic, M. Andrejevic, M. Micev

^*^Clinical Centre of Serbia, Dept. of Histopathology, Belgrade, Serbia

**Objective:** Pancreatoblastoma is a rare pediatric neoplasm which can exceptionally occur in adults. The first case of adult pancreatoblastoma was reported in 1986 and less than 30 cases have been published in literature since then. We present a case of pancreatoblastoma in a 32-year-old man with one-month history of abdominal pain and discomfort.

**Method:** Routine histological and ancillary immunohistochemical examination were performed.

**Results:** Solid tumor measuring 46 mm in diameter in the tail of pancreas was detected on CT scan as well as enlarged liver with numerous metastatic deposits. Biopsy of liver metastasis was performed and examination of the tumor histomorphologically revealed nested and trabecular organization of primitive-appearing cells with prominent zones of squamous differentiation, sometimes with overt keratinization. Most tumoral cells expressed CAM 5.2 and CK19 immunopositivity, but CK7, CK17 and CK20 immunonegativity with Ki-67 protein labelling index 95 %. The second population of squamous differentiated cells showed strong CK5, antichymotrypsin and N-cadherin immunopositivity. Focal moderate cytoplasmic synaptophysin immunopositivity in 30 % of cells was also observed. All cells were chromogranin A, antitrypsin and p63 negative.

**Conclusion:** Although pancreatoblastoma is extremely rare in adult population, it should be considered in the differential diagnosis of pancreatic mass in young adults.

**PS-20-023**

**Neurofilament and CD31 expression in pancreas tissue in chronic pancreatitis**

V. Klopova ^*^, I. Samsonova

^*^Vitebsk State Medical University, Dept. of Pathological Anatomy, Belarus

**Objective:** Neurofilament (NF) proteins are the major structural proteins of neurons. CD31 expression is restricted to cell of the vascular system, namely platelets, monocytes, neutrophils, selected T cell and endothelial cell. Study of this expression in pancreas at chronic pancreatitis (CP) will help to characterize the neuropathia development and its role in clinical manifestations. To study NF expression in pancreas at CP depending on fibrosis.

**Method:** We investigated pancreas head of 43 patients with CP, who were operated. Control group included 5 pancreas head specimens of people, died due to accident and having no pathology of pancreas. Statistic analysis included Kruskal-Wallis’s and Spearman’s tests.

**Results:** Depending on fibrosis area all patients were subdivided into 3 groups: I group–<25 %, II group–25–50 %, III group–>50 %. Increase of fibrosis degree was accompanied with increase of pancreas nerve tissue (PNT) total area [from 27451,118 to 1105858,974 mkm2] and the decrease of positive expressed nerve elements (PPENE) percent [from 100 % to 0 %] (*r* = −0,3 р < 0,05). Increase of CD31expression was accompanied with decrease of PNT total area and PPENE (*r* = −0,3 р < 0,05).

**Conclusion:** Diffuse degenerative changes of pancreas at CP were accompanied with severe fibrosis and involvement of nerve component, increase of its total area. At the same time the area of mature and viable nerve elements was decreased.

**PS-20-024**

**Lipid-rich cell variant of pancreatic neuroendocrine neoplasm**

Y. Goto ^*^, M. Higashi, S. Yonezawa

^*^Kagoshima University, Dept. of Human Pathology, Japan

**Objective:** Pancreatic neuroendocrine tumor (PNET) is a rare tumor (less than 5 % of pancreatic tumors). We report a case of clear cell variant which is particularly rare in PNETs.

**Method:** 64 years old Japanese woman pointed out the distention of common bile duct by chance in abdominal ultrasound at medical chekup. A tumor (32 mm in diameter) was detected in the pancreatic tail by CT. Serum hormone levels are normal. Lipid component in the tumor was detected by MRI. Laparoscopic distal pancreatectomy was performed.

**Results:** Grossly, the tumor showed yellow-tan color. Histologically, tumor cells with uniform round nuclei are proliferated in cord-like structure. Coagulated necrosis is seen in the center of tumor. Some parts showed aggregation of foamy cells with abundant microvesicular cytoplasm which are lipid-rich cells positive for Oil red O staining. The lipid-rich cells area is coincided with the MRI finding. Venous invasion is pointed, and Ki67 labeling index is 12 %. Immunohistochemically, the tumor cells are diffusely positive for CD56 and synaptophysin, and partially positive for chromogranin A and S100.

**Conclusion:** This is the first report of lipid rich cell variant of PNET carrying lipid component could be detected by a presurgery MRI.

**PS-20-025**

**Ki-67 scoring in neuroendocrine tumors of the pancreas: Alternative solutions**

E. Çinar ^*^, U. Aykutlu, E. Basar, A. Uguz, H. Uluer, F. N. Ipek, A. Coker, F. Yilmaz

^*^Ege University, Dept. of Pathology, Izmir, Turkey

**Objective:** Proliferation index (PI) determined by nuclear Ki-67 positivity is important for staging of neuroendocrine tumors (NET). Traditionally Ki-67 index is determined by counting 2000 cells in the most intensely stained areas. This method is time consuming, there is a risk for intra/interobserver variability and image analysis is not available widely. We compared three different methods for Ki-67 counting and sought their correlation with the clinicopathological parameters.

**Method:** PI was scored in automatically stained slides of 25 pancreatic NET patients as follows: i) proportion of positive cells in 2000 cells (Ki-67-traditional), the number of positive cells in ii) 10HPF (Ki-67-10-HPF), and iii) 50 HPF (Ki-67-50-HPF). Correlation of clinico-pathological parameters (mitotic count, retroperitoneal, perineural, vascular, capsular invasion, recurrence, exitus, prognosis) and PI were analyzed statistically.

**Results:** Correlation rates between Ki-67-traditional vs Ki-67-10-HPF and Ki-67-50-HPF were 0.738 and 0.68, respectively, and Ki-67-10-HPF vs Ki-67-50-HPF was 0.935. No significant correlation was found between Ki-67 scores and mitotic count, exitus, recurrence, perivascular, perineural and capsular invasion. Ki-67-10HPF and Ki-67-50HPF were correlated positivly with retroperitoneal invasion and negatively with prognosis.

**Conclusion:** Ki-67 scoring with alternative manual methods provide significant results for prognosis. These novel counting methods, which are similar to mitotic count can be used for staging of NET.

**PS-20-026**

**O6-MethylGuanine DNA MethylTransferase (MGMT) expression and Ki-67 index predict response to temozolomide in patients with Well Differentiated Pancreatic NeuroEndocrine Tumors (WDPNET)**

J. Cros ^*^, O. Hentic, V. Rebours, M. Zappa, F. Maire, P. Hammel, P. Levy, P. Ruszniewski, A. Couvelard

^*^Beaujon University Hospital, Dept. of Pathology, Clichy, France

**Objective:** Temozolomide (TEM) is an orally administered drug with very encouraging results in WDPNET in a recent retrospective study (Strosberg et al., 2011). We aim to assess MGMT expression and its correlation with the efficacy of TEM-based chemotherapy in patients with WDPNET.

**Method:** Patients with WDPNET treated with TEM between 2006 and 2012 were included. The expression of MGMT was assessed by immunochemistry.

**Results:** 43 patients (21 men, 58(27–84) years) with WDPNET, grade 1 (6 pts) or 2 (37 pts) (WHO 2010 classification) were treated with TEM in Objective response, stable disease and progression rates were 37.2 % (16pts), 44.2 % (19pts) and 18.6 % (8pts) respectively. Median (range) MGMT score was 49 (0–300). The MGMT was correlated with the radiological response (Objective response, *p* = 0.02) and the progression free survival (PFS) (*p* = 0.003). Progression was associated with a high Ki67 (mean 16 %, *p* = 0.01) and most of them had low MGMT level. Objective response was associated with a low MGMT level (*p* = 0.06). Tumors of patients with NEM1 almost always harbored an elevated MGMT regardless of the clinical response.

**Conclusion:** Temozolomide-based chemotherapy is highly efficient in WDPNET. MGMT level can predict objective response and PFS in TEM treated patients with sporadic low Ki67 index WDPNET.

**PS-20-027**

**Genetic aberrations in solid pseudopapillary tumor of the pancreas**

E. Gordienko ^*^, I. Chekmareva, O. Paklina, D. Rotin

^*^Vishnevsky Surgery Institute, Moscow, Russia

**Objective:** To investigate the presence of-Myc and N-Myc amplification in SPT of the pancreas

**Method:** Seventeen cases of SPT and seven cases of control pancreatic tissue were included in our study. Dual-color interphase fluorescence in situ hybridization (FISH) was performed on 4-μm thick sections of formalin-fixed paraffin-embedded (FFPE) tumors. To validate loci 8q24/MYC and 2p24/N-MYC locus specific and centromere DNA probes (CEP8 and CEP2) (Vysis, Abbott Lab) were used.

**Results:** Macroscopically SPT presented single or multiple units ranging in size from 0.6 to 17 cm (mean size 6.2 cm). Grossly solid architecture pattern of the tumor prevailed–12 cases (57.1 %) following by cystic-solid–8 (38.1 %), and cystic–1 (4.8 %). Normal tissue nuclei showed balanced profile in all seven control cases. FISH analysis of SPT revealed the gain of N-MYC gene copy number in 41 % (7/17), while in two cases an aggressive clinical course with metastatic development ocurred. Profile C-Myc in all cases of SPT was balanced.

**Conclusion:** Amplification of N-Myc gene in SPT can be one more important confirmation of its neurogenic origin. However, the tumor has a low malignant potential and seldom metastasizes or recurs in contrast to the aggressive course of high grade poorly differentiated of the nervous system neoplasms

**PS-20-028**

**Solid-pseudopapillary and endocrine pancreatic tumor cells: Nuclear ploidy comparative study**

E. Dubova ^*^, O. Mishnev, M. Nagovitsyna, A. Shchegolev

^*^Research Center for Obstetrics, Dept. of Pathology, Moscow, Russia

**Objective:** Endocrine (ET) and solid-pseudopapillary (SPPT) tumors of the pancreas are rare neoplasms account up to 1–2 % of all pancreatic tumors. Our aim was to study nuclear ploidy of SPPT and ET cells.

**Method:** 12 cases of ET and 6 cases of SPPT (5 females, 1 male) were studied. We performed histological, morphometrical and ploidy analysis of all specimens.

**Results:** In all SPPT and ET cases cell nuclear perimeter and size were higher than in normal pancreatic acinar and endocrine cells. This sign could be used to cell atypy assessment. Maximal nuclear perimeter and size were observed in SPPT cases being higher than in normal endocrine cells (*p* < 0.05). Mean nuclear ploidy (MNP) of normal acinar cells was 2.4c, of endocrine cells–2.21c. Most of the SPPT cells were diploid (43 %), whereas ET cell were predominantly triploid (38 %). MNP of ET was 3.49c, SPPT–2.59c. Proliferation index (PI) of SPPT cell was 3 times higher than of acinar cells, PI of ET cell was 7 times higher than of endocrine cells. Aneuploidy coefficient of SPPT was 0.04 and of ET–0.78.

**Conclusion:** Nuclear morphometric parameters, nuclear ploidy, aneuploidy coefficient and proliferation index could be used as the additional criteria for SPPT and ET differential diagnosis.

**PS-20-029**

**Intraductal papillary mucinous neoplasms of the pancreas: Report of seven cases**

E. Kairi-Vasilatou ^*^, C. Dastamani, A. Tsagkas, E. Carvounis, A. Kondi-Pafiti

^*^Aretaieion University Hospital, Dept. of Pathology, Athens, Greece

**Objective:** Intraductal papillary mucinous neoplasms (IPMNs) are neoplasms of the pancreatic duct epithelium characterized by intraductal papillary growth and thick mucin secretion, arising in the main pancreatic duct or its branches. They are usually located in the head of the pancreas and can display various degrees of dysplasia.

**Method:** Over a 5-year period, seven cases of pancreatic IPMNs were diagnosed in our laboratory in a total of 238 pancreatectomy specimens examined (2.9 %).

**Results:** Patients’ age was 57–74 years (mean age 68.8 years). Six were male (85 %) and one was female (15 %). Two patients (28.5 %) underwent a total pancreatectomy, and five (71.4 %) had a partial pancreatectomy. Invasive carcinoma was present in one patient (14.2 %) In 4/5 cases of partial pancreatectomy (80 %), the pancreatic transection margin was involved with atypia. The main pancreatic duct was involved in 6/7 cases (85 %). Microscopically, the pancreatic duct was cystically dilated and displayed complex papillary fronds of mucin-producing epithelial cells with variable atypia. The surrounding pancreatic parenchyma showed mild to moderate fibrosis and acinar atrophy, typical of obstructive chronic pancreatitis.

**Conclusion:** Though IPMNs without an associated invasive carcinoma have excellent prognosis, the prognosis for IPMN-associated invasive carcinomas is worse, but overall better than that of pancreatic ductal adenocarcinomas.

**PS-20-030**

**MUC2 and MUC6 glycoproteins expression may be useful for differentiate the intestinal and gastric histological types of intraductal papillary-mucinous neoplasm and may indicate different pathways in pancreatic carcinogenesis**

S. Teresa ^*^, L. Perez-Casanova, M. Terricabras, C. Villabona, C. Valls, J. Busquets, E. Condom, M. Paules

^*^Hospital De Bellvitge. Idibell, Pathology, Barcelona, Spain

**Objective:** Intraductal papillary-mucinous neoplasm (IPNM) are classified into main duct and branch duct and non invasive IPMNs were mostly intestinal or gastric types. Mucins could play a key role in pancreatic carcinogenesis.

**Method:** We examined 22 consecutive cases of non invasive IPMNs. Immunohistochemistry studies for MUC2, MUC 6 and MUC5a mucins, CK7, CK20 and CDX2 were evaluated.

**Results:** Sixteen of the 22 non invasive IPMNs were main duct IPMNs. Seven were intestinal-type and were positive for MUC2, CK20 and CDX2 and negative for MUC6 and CK7, and 6 were gastric type and positive for MUC 6 and CK7 and negative for MUC2 and CK20. Six of the 22 non invasive IPMNs were categorized as branch duct subtype. Four were intestinal type small lesions and were positive for MUC2 and showed variable expression of CK20, and 2 were gastric type multilocular cyst lesions and were positive for MUC6 and negative for MUC2 and CK20.

**Conclusion:** The immunohistochemistry expression profile may help to separate the intestinal MUC2+/MUC6- from the gastric MUC2-/MUC6+ type of non invasive IPMNs and supports the notion that there is an intestinal pathway distinct from the gastric pathway in IPMN carcinogenesis.

**PS-20-031**

**Intraductal tubulopapillary neoplasm of the pancreas: Part of the spectrum of intraductal neoplasms of the pancreatobiliary system**

H. Rodrigues ^*^, C. Oliveira, N. Dias, D. Oliveira, M. A. Cipriano, M. F. Xavier Cunha

^*^CHUC, Anatomia Patológica, Pardilhó, Portugal

**Objective:** Intraductal tubulopapillary neoplasm (ITPN) is a recently recognized rare variant of pancreatic intra-ductal neoplasm.

**Method:** A 54 years old male presented with epigastric pain, steatorrhea, diabetes mellitus and 5Kg weight loss in 2 months. CT scan: solid, nodular, heterogeneous mass in the cephalic pancreas. Gross examination showed a multinodular solid well circumscribed tumour with 5.7 cm involved by a firm stroma. Histologically was represented by intraductal monotonous growth, composed of tightly packed small tubules lined by cuboidal cells with high-grade dysplasia, without mucin production. The main differential diagnoses are intraductal papillary mucinous neoplasms and other tumours with intraductal growth pattern: acinar cell carcinoma, neuroendocrine tumours, and ductal adenocarcinoma (DAC).

**Results:** ITPN has an indolent course. No correlation was found between invasion and prognosis, and even with lymph nodes or liver metastasis the prognosis is better than DAC. One of the major problems is to assess invasion due to the complexity of the intraductal pattern. Common molecular findings: DPC4 retained in 90 %; p53 overexpressed in 20 % and p16 in 50 %.

**Conclusion:** ITPN is a malignant neoplasm with protracted clinical course. Extensive sampling is necessary to exclude invasion. Diagnostic key features: macroscopic solid multinodular tumour, tubulopapillary growth pattern and absence of mucin secretion and acinar differentiation.

**PS-20-033**

**Morphological evaluation of margins and R-status in ductal pancreatic cancer**

O. Paklina ^*^, G. Setdikova, O. Potapova

^*^Burnazyan FMBC of FMBA Russia, Dept. of Pathology, Moscow, Russia

**Objective:** To systematize the line of “resection margin” in PDA and evaluate prognostic significance of regional lymph nodes involvement and perineural invasion for the patients with PDA.

**Method:** 55 patients with PDA located in the head of pancreas. R1 was stated in cases with the tumor complexes presented at the distance from the resection margin < or = 1 mm.

**Results:** R1 status in PDA was determined in 69.1 % (38/55). Most frequent affection was seen in the anterior and middle margins–14.5 % and 9 % (8/38 and 5/38). This type of tumor spreading we associated with the direct extension. Intrapancreatic PNI developed in 76.4 % (42/55), extrapancreatic PNI was found in 29.1 % (16/55). Regional lymph nodes involvement was found in 67.3 % (37/55) of the series. Both types of PDA spreading should be classified as locoregional spreading. Thus, only in 7.3 % (4/55) of cases neither tumor spreading beyond the pancreas. Most cases, 50.9 % (28/55), developed the mixed type of tumor invasion.

**Conclusion:** In PDA the allocated locoregional and mixed types of spreading should be kept in mind along with the direct spread of the tumor. The aim to increase the proportion of R0 resections is considered to be impossible without improvement and standardizing of pathological examination protocols.

**PS-20-034**

**Loss of PTEN expression in tumor microenvironment correlates with aggressive behaviour and detects synergistic roles of tumor and stromal cells in Pancreatic Ductal Adenocarcinoma (PDAC)**

E. Karamitopoulou ^*^, I. Zlobec, A. Lugli, B. Gloor, A. Perren

^*^University of Bern, Institute of Pathology, Switzerland

**Objective:** PTEN is a major tumor suppressor in PDAC. Our aim was to determine the role of PTEN in PDAC progression and correlate PTEN alterations with clinico-pathological features of the patients.

**Method:** PTEN-Immunohistochemistry was performed on a multipunch Tissue Microarray (TMA) of 120 well-characterized PDACs with full clinico-pathological, follow-up and adjuvant therapy information. PTEN-immunoreactivity was evaluated in the tumor- and the stromal cells. Another 3 TMAs including precancerous lesions (PanINs), matched lymph node metastases and normal pancreas were additionally stained.

**Results:** We found a significant progressive PTEN loss from normal ductal epithelia (21.5 %) to PanINs (13 %) and to PDAC (6.2 %) (*p* < 0.0001). PTEN loss in tumor cells was associated with higher tumor grade (*p* = 0.0338) and marginally with higher T-stage (*p* = 0.0596). PTEN loss in the stromal cells was strongly associated with distant metastasis (*p* = 0.0045) and showed a trend towards worse overall (*p* = 0.0791) and disease-free survival (*p* = 0.066).

**Conclusion:** PTEN loss in PDAC may play an important role in the neoplastic progression and seems to correlate to a more aggressive phenotype. Moreover, loss of PTEN in the neoplastic stroma shows a strong association with distant metastasis, suggesting that the stromal cells are actively participating in the process of pancreatic cancer dissemination.

**PS-20-035**

**MYC gene abnormalities in ampulary carcinoma and ductal adenocarcinoma of the pancreas**

O. Paklina ^*^, G. Setdikova

^*^Burnazyan FMBC of FMBA Russia, Dept. of Pathology, Moscow, Russia

**Objective:** MYC amplification in pancreatic ductal adenocarcinoma (PDA) and ampulary carcinoma (AC).

**Method:** 74 cases of PDA, 22 cases of ampulary carcinoma (AC). Age of patients ranged from 30 to 78 years. We examined the expression of MUC1, 2 and 5AC types in PDA and AC and fluorescent in situ hybridization.

**Results:** Most of the PDA have been reported as–MUC1 +/MUC5AC + and accounted for 42 % (31/74), the smallest was a group with the intestinal mucin phenotype (MUC2 +) (7 % (5/74). AC in most cases have been reported as MUC2 + and accounted for 63 % (14/22), 37 % (8/22) of cases the tumor presented as MUC1 +/MUC5AC +. FISH analysis of PDA revealed of MYC gene copy number in 21/74 (28 %) cases. 8/74 pancreatic tumors (10,8 %) revealed MYC gain in association with an increased chromosome 8 copy number. AC profile MYC gene is diploid.

**Conclusion:** MYC gene amplification should be considered as a marker of a more aggressive behavior in cancers biliary pancreaticduodenal region. Diploid profile MYC gene in ampulary carcinomas indicates a more favorable prognosis compared with ductal adenocarcinomas of the pancreas.

**PS-20-036**

**Mucin expression in pancreatic ductal adenocarcinoma: Immunohistochemical examination in EUS-FNAB specimens**

M. Higashi ^*^, Y. Goto, S. Yonezawa

^*^Kagoshima University, Dept. of Human Pathology, Japan

**Objective:** We aimed to detect predictive markers in EUS-FNAB specimens of pancreatic ductal adenocarcinoma (PDAC).

**Method:** Expression of mucins (MUC1/DF3, MUC2, MUC4/8G7, MUC5AC, MUC6, MUC16/M11) and labeling of MIB-1/Ki67 were examined by immunohistochemistry of the EUS-FNAB specimens from 109 patients. Mucin expression of PDAC was considered “positive” if more than 10 % of carcinoma cells were stained. MIB-1/Ki67 labeling was considered “high” if more than 50 % of carcinoma cells were labeled. The mucin expression and Ki67 labeling were compared with clinico-pathological features in each patient.

**Results:** The expression rates of each mucin were as follows: MUC1/DF3, 88.0 %; MUC2, 0.9 %; MUC4/8G7, 94.4 %; MUC5AC, 78.0 %; MUC6, 24.7 %; and MUC16/M11, 67.0 %. The prognosis of patients with MUC16 positive was significantly poorer than those with MUC16 negative. The prognosis of patients with high Ki67 labeling was significantly poorer than those with low Ki67 labeling.

**Conclusion:** MUC16 expression and Ki67 labeling are predictive factors of PDAC.

**PS-20-037**

**Undifferentiated carcinoma of the pancreas with osteoclast-like giant cells: Report of two cases**

S. Chaves ^*^, M. J. Fernández-Aceñero, A. Cazorla, T. Ranchal, J. I. Cornejo

^*^Fundacion Jimenez Diaz, Surgical Pathology, Madrid, Spain

**Objective:** To report two cases of pancreatic undifferentiated carcinoma with osteoclast-like giant cells.

**Method:** Both patients were women (aged 62 and 71). One consulted on vague abdominal discomfort and the other was asymptomatic. Imaging studies revealed an abdominal mass.

**Results:** In the first patient diagnosis was made in a gastric endoscopic biopsy, but surgical pancreatoduodenectomy revealed that the tumor arose in the pancreas and measured 17 × 15 × 10 cm. The second patient had a preoperative diagnosis of pancreatic carcinoma and the tumor was a 5 × 4.2 × 3, partially cystic mass in the pancreatic head. Histology revealed undifferentiated tumors composed of pleomorphic to spindle cells and osteoclast-like giant cells, with areas of necrosis and haemorrhage. Spindle cells expressed CK 7, AE1/AE3; giant cells, EMA, CD 45, CD 68, CD 56; and both components, CD 117, p53 and p16. Diagnosis was undifferentiated carcinoma with osteoclast-like giant cells.

**Conclusion:** Undifferentiated carcinoma of the pancreas with osteoclast-like giant cells is a rare neoplasm. The mean age is 60 years. The mean survival was 12 months, a more favourable prognosis than usual ductal adenocarcinoma. This histological type is rare outside the pancreas and its presence in any location should awake suspicion of this primary origin.

**PS-20-038**

**Metastasis to the pancreas and the spleen: An increasing diagnostic and therapeutic challenge**

S. Chaves ^*^, M. J. Fernández-Aceñero, M. Abengózar, P. Wolfgang, T. Ranchal, J. I. Cornejo

^*^Fundacion Jimenez Diaz, Surgical Pathology, Madrid, Spain

**Objective:** The objective of the present report is to review the cases of pancreatic and splenic metastasis diagnosed at a single center between 1998 and 2010.

**Method:** We have reviewed the electronic biopsies database files of the Department of Surgical Pathology, Fundación Jim’nez Daz in Madrid (Spain).

**Results:** We have found four pancreatic metastasis and five splenic metastasis. Two of the pancreatic metastasis were originated in clear cell renal cells carcinomas. The other pancreatic metastasis were from a malignant cutaneous melanoma diagnosed and treated 8 years before and from a cervical squamous cell carcinoma treated with surgery and radiotherapy. Three of the splenic metastasis were diagnosed during abdominal surgery for primary therapy of the tumor (two ovaries and one endometrium), while the remaining two corresponded to metastasis from a lung and colonic tumors. The patients with splenic metastasis died on the short term with progression of the disease despite the resection of the splenic lesions, while the patients with pancreatic metastasis have survived longer.

**Conclusion:** Our experience seems to fit to the reported literature regarding tumor types and outcome of the patients. According to the present literature metastasectomy is indicated to improve prognosis of these patients.

**PS-20-039**

**Inflammatory polyp of the gallbladder containing intestinal metaplasia**

F. Yilmaz^*^, A. Kilitci, N. Sengül

^*^Abant Izzet Baysal University, Medicine Faculty, Bolu, Turkey

**Objective:** Inflammatory polyp of the gallbladder is a rare variant of benign gallbladder polyp. Inflammatory polyps histologically consist of vascular connective tissue containing inflammatory infiltrates and there is hyperplastic epithelial invagination. These polyps appear to be the result of enlargement and fusion of villi due to chronic inflammation.

**Method:** Case report: A 56-year-old male presented with gallstone and a polypoid lesion(PL) that was found incidentally. Ultrasonography (USG) revealed the PL measuring 0,8 cm in diameter in the fundus and a stone measuring 0,7 cm in diameter. Laparoscopic cholecystectomy was performed. Resected specimen revealed a smooth-surfaced polyp and a gallstone.

**Results:** Histological study revealed a nonneoplastic polyp with edematous stroma and infiltration of lymphcytes and plasmacytes. Intestinal metaplasia (IM) was found in the polyp. Alcian blue/PAS was used to define and characterize IM. Acid mucin stained the goblet cells in glands and at the surface epithelium of the polyp. This lesion was diagnosed as an inflammatory polyp with IM, of the gallbladder.

**Conclusion:** Availability and wide use of USG has increased the incidental detection of ‘PLs of the gallbladder’(PLG). The majority of them are benign neoplastic/nonneoplastic in nature, although they can rarely be malignant. As our case, surgery may be advised for PLGs of 5 mm or greater, patient over 50-years, symptomatic or in whom follow-up cannot be completed should be considered for cholecystectomy. The histological signs of inflammatory polyp of the gallbladder should guide the examination of USG findings.

**PS-20-040**

**Multiple adenomyomas of the gastrointestinal and biliary tract in the same patient: Case report**

A. Pop ^*^, S. Ohja, L. Jiao, P. Cohen

^*^Imperial College Healthcare Trust, London, United Kingdom

**Objective:** Adenomyomas of the gastrointestinal and biliary tract are benign tumour-like lesions, most frequently found in the gallbladder. Cases have also been described in the extrahepatic biliary tract, stomach and small bowel.

**Method:** We describe a very unusual case of a 69-year-old woman who underwent duodenopancreatectomy and cholecystectomy for a suspicious ampullary mass causing obstruction.

**Results:** The specimen revealed four simultaneous adenomyomas: ampullary, fundal gallbladder, duodenal (presenting as a sessile polypoid lesion) and a common hepatic duct adenomyoma (microscopic incidental finding). To our knowledge this is the first report of four adenomyomas in the same patient. All four lesions showed glandular structures lined by a single-layer of bland cuboidal to columnar epithelium and interlacing hyperplastic smooth muscle bundles surrounding the glandular lobules. The aetiology of the gastrointestinal adenomyomas is still controversial, most authors suggesting that they represent heterotopic pancreatic tissue lacking acinar and endocrine cell elements. Our immunohistochemical findings of diffuse CK7 and MUC6, focal MUC5AC and very focal MUC2 positivity and absence of CK20 and MUC1 staining are not definite for a specific origin, though they suggest pancreatobiliary type epithelium.

**Conclusion:** The presence of multiple similar lesions, which is the particularity of this case, with the immunostaining pattern described could constitute an argument for the hypothesis mentioned.

**PS-20-041**

**Combined adenoneuroendocrine carcinoma of the gallbladder**

G. Benkhedda ^*^, H. Ghernaout, K. Bendjebbar, Y. Lamouti

^*^Saad Dahleb University, Dept. of Medicine Pathology, Blida, Algeria

**Objective:** Primary neuroendocrine (NE) carcinoma of the gallbladder is extremely rare. Mixed tumors are even more rare, with few cases reported

**Method:** We report a rare case of tumor combined with neuroendocrine carcinoma and adenocarcinoma of the gallbladder in a 73-year-old woman.

**Results:** The gallbladder revealed a (5 × 5 × 5) cm white exophytic mass located at the fundus and growing into the lumen. Histologically, the tumor consisted of two components. The endocrine cell carcinoma was composed of solid or cribriform nests of medium-sized cells, with amphiphilic or clear cytoplasm, hyperchromatic moderately pleomorphic nuclei and hight mitotic rate. The adenocarcinoma was a well differentiated adenocarcinoma and included goblet-type cells. Additionally, there was focal necrosis, fibrosis, extension for the adjacent liver tissue, and vascular invasion. Immunohistochemical studies of the cells neuroendocrine were positive for chromogranin A and synaptophysin. Strongly positivity with Ki67, estimated at 80 %.

**Conclusion:** Primary carcinoma of the gallbladder is mostly adenocarcinoma and NE tumor of the gallbladder is rare. The origin of neuroendocrine carcinoma of the gallbladder is still unclear. The histogenesis of carcinoid tumors is that they originate from multipotential stem cells that have the ability to differentiate towards neuroendocrine and glandular cell types.

**PS-20-042**

**AFP secreting gallbladder carcinoma: A case report**

G. Akturk ^*^, O. Sagol, T. Unek, M. Ozbilgin, T. Egeli, S. Karademir, F. Obuz, I. K. Astarcioglu

^*^Dokuz Eylul University, Dept. of Pathology, Izmir, Turkey

**Objective:** AFP producing carcinomas other than hepatocellular carcinoma, hepatoblastoma, yolk sac tumors; are rarely reported and they are known to have a poor prognosis. The typical general histologic appearance of these tumors are reported as hepatoid carcinomas but there are also other rarely reported histologic types like tubular/papillary adenocarcinomas and poorly differantiated medullary carcinomas. The most well defined AFP producing tumors are known to occur in stomach and they are rarely reported in gallbladder. Here we report a case originated from gallbladder with high levels of AFP production.

**Method:** An AFP secreting gallbladder carcinoma with gross and microscopic features was presented.

**Results:** 45 year old woman with a tumor mass in the liver related to the gallbladder was performed a liver biopsy. The biopsy revealed a papillary type adenocarcinoma morphology. Serum AFP level was extremely high. The immunohistochemical stains revealed AFP and Hepatocyte A positivity at the tumor although hepatoid morphology was not observed. The patient was operated and tumor mass originating from the gallbladder was diagnosed as papillary adenocarcinoma with AFP production.

**Conclusion:** These rare tumors were discussed in terms of nomenclature and morphologic features.

Tuesday, 3 September 2013, 09.30–10.30, Pavilion 2

**PS-21 Poster Session Nephropathology**

**PS-21-002**

**Renal graft injury: Overlapping features between transplant glomerulopathy and de novo thrombotic microangiopathy**

R. M. Costa ^*^, E. Martul, J. Reboredo, C. Rivera

^*^CHTMAD, Dept. of Nephrology, Vila Real, Portugal

**Objective:** Characterize pathological relationship between transplant glomerulopathy (TG) and de novo thrombotic microangiopathy (TMA) in renal allograft biopsies.

**Method:** Retrospective analysis based on demographic, clinical, laboratorial, histological and outcome features.

**Results:** A total of 39 TG(6.2 %) and 14 (2.2 %) de novo TMA cases were identified. TMA presented diffuse glomerular lesions when compared to TG (100 % vs 45.9 % *p* = 0.001). Both groups presented equally glomerular basement membrane (GBM) double contours, glomerulitis, tubulitis, capilaritis and ischemic features. Capillary congestion(100 vs 35.1 %, *p* < 0.001),microthrombi(50 % vs 5.4, *p* = 0.001), schistocytes (42.8 % vs 7.7 %, *p* = 0.009)and mesangiolysis (85.7 % vs 29.7 %, *p* < 0.001) were associated to TMA. TG presented advanced glomerulosclerosis, IF/TA and interstitial plasma cells(30.8 % vs 0 %, *p* = 0.018). Positive C4d staining in de novo TMA cases was similar to TG (71.4 % vs 53.8 %, *p* = 0.252) but arteriolar C4d deposition was predominantly seen in these cases (35.7 % vs 8.7 %, *p* = 0.042). Donor Specific Antibodies detection was equally found in both groups (TG: 41.6 %; TMA: 57.1 %, *p* = 0.500). After ultrastrutural analysis only TG cases presented GBM multilayering (75 % vs 0 %, *p* = 0.044)

**Conclusion:** The similarities between TG and de novo MAT suggest that both are different manifestations of endothelial lesion.

**PS-21-003**

**Non-diabetic renal disease in patients with type 2 diabetes mellitus: A single center experience**

M. Wagrowska-Danilewicz ^*^, M. Danilewicz

^*^Medical University of Lodz, Dept. of Nephropathology, Poland

**Objective:** The prevalence of diabetes, especially type 2 diabetes, is experiencing a rapid growth. The aim od the study was to evaluate the incidence of non-diabetic renal disease (NDRD) in type 2 diabetic patients.

**Method:** From January 2006 to February 2012, 76 patients with type 2 diabetes mellitus underwent renal biopsy in Nephropathology Center in Lodz. Based on the biopsy findings, patients were grouped as Group I- isolated NDRD, Group II- NDRD with underlying diabetic glomerulosclerosis and Group III- isolated diabetic glomerulosclerosis (DG).

**Results:** Of the 76 patients studied 54 were males, and 24 were females. Mean age at the time of biopsy was 57.4 ± 9.4 years. Group I included 38 patients (50 %), Group II-11 patients (14.5 %), and Group III- 27 patients (35.5 %). The most common lesion found in Group I was focal segmental glomerulosclerosis, followed by membranous glomerulopathy, IgA nephropathy, and glomerulonephritis with crescents. The duration of diabetes was significantly less in Group I compared with Group II and III. There were no statistically significant differences between groups in relation to age, nephrotic syndrome and hypertension. The presence of hematuria was significantly more frequent in Group I than in Group III.

**Conclusion:** NDRN is a common feature in patients with type 2 diabetes with renal involvement.

**PS-21-004**

**Linear glomerular IgA deposition**

S. Sarioglu ^*^, S. Ozkal, M. Bagirzade, M. Unlu, Y. Sahin, A. Celik, O. G. Sevindik, I. Alacacioglu

^*^Dokuz Eylul University, Dept. of Pathology, Izmir, Turkey

**Objective:** Glomerular lineer IgA deposition is rarely encountered in renal biopsies. Such a case is presented in order to discuss the differential diagnosis.

**Results:** A-56 year old male patient presented with vasculitic symptoms and microscopic hematuria. Renal biopsy revealed membranous linear IgG, IgA and kappa chain deposition with mild mesangial matrix expansion. Electron microscopy revealed mild basement membrane thickening but electron dense deposits weren’t identified. The case was reported as nonspecific DIF findings, further evaluation for light chain disease and anti-GBM nephropathy was suggested. Without any plasma cell dyscrasia signs, bone marrow aspiration was performed and revealed 40 % plasma cells with some bizarre shaped cells which proved to be monoclonal and staining predominantly with kappa. Serum protein electrophoresis showed monoclonal gammopathy which was identified as IgA kappa in immunofixation. Although the patient didn’t meet active (symptomatic) myeloma criteria as he had complaints of bone pain and his PET results revealed multiple lytic-sclerotic lesions at costal, vertebral and femoral regions. Because of the documented bone lesions patient was put on to combination chemotherapy.

**Conclusion:** IgA kappa monoclonal gammopathy should be considered if glomerular lineer IgA deposition along with positive kappa and negative lambda staining is observed in a renal biopsy.

**PS-21-005**

**Biopsy series of acute kidney injury from a tertiary care referral centre in South India**

S. Siddappa ^*^

^*^Institute of Nephrourology, Laboratory, Bangalore, India

**Objective:** High prevalence of acute kidney injury in developing countries, hence detection of AKI in its early and potentially reversible stages to prevent disease progression to kidney failure necessitating renal replacement therapy.

**Method:** Our Institute is a premier non-profit tertiary care referral centre for Nephrology and Urology funded by government of Karnataka. A retrospective analysis of patients who underwent renal biopsies in our institute was reviewed. A total of 243 renal biopsies from Jan 2012 to March 2013 to our centre were included.

**Results:** Out of 243 biopsies, 130 cases fulfilled criteria of acute kidney Injury. The mean age was 38.8 ± 10.02 years with male preponderance (73 males to 57 females). 10 patients were transplant recipients while 11 patients had succumbed to kidney injury. In five patients AKI was drug related and four females presented with acute renal failure due to postpartum. Renal failure was common clinical presentation followed by acute nephritis. Histopathologically acute interstitial nephritis was the most common finding in 33.1 % patients followed by postglomerular nephritis (19.2 %) and ischemia causes (6.9 %).

**Conclusion:** A high mortality of 8.46 % in our referral centre; Is renal support underutilized or delayed? This is the question of the hour.

**PS-21-006**

**The incidence of non-lupus „full house“nephropathy: Review for the past 10 years**

M. Zivotic ^*^, J. Vjestica, S. Cirovic, M. Stojanovic, J. Markovic-Lipkovski, V. Brkovic

^*^University of Belgrade, School of Medicine, Inst. of Pathology, Serbia

**Objective:** Glomerular “full house” immunofluorescence (IF) staining commonly indicates lupus nephritis (LN). Some “full house” glomerular entities can be present in the absence of other clinical features of systemic lupus erythematosus (SLE) and in this way can mimic LN. Our goal was to analyze a 10-year incidence of the non-lupus “full house” nephropathy and its possible significant increase within our investigation period.

**Method:** During this period, 131 kidney biopsies with “full house” IF pattern were observed. We analyzed 86 (61 %) cases with clinically developed SLE and 45 (39 %) cases without any evidence of SLE.

**Results:** Among non-lupus “full-house” cases we diagnosed 27 membranous glomerulonephritis (GN), 9 membranoproliferative GN, 4 mesangioproliferative GN, 3 membranous/membranoproliferative GN and 2 rapidly progressive GN. In 2005, 2006 and 2011 we noticed an increased incidence of non-lupus “full-house” nephropathy. Usually, the incidence of non-lupus “full-house” GN was betwen 10 % and 30 %, however, in three previously mentioned years, the incidence was about 45 %.

**Conclusion:** The incidence of non-lupus “full house” GN had two peaks during the last 10 years, indicating that some environmental factor could be the cause of this type of GN. Patients with non-lupus “full-house” nephropathy should be closely followed up in order to see whether they will develop SLE.

**PS-21-007**

**FOXP3 immunohistochemical detection in patients with Lupus Nephritis (LN)**

G. Hariklia ^*^, L. Maria, K. Evangelia, L. Georgia, S. Anastasios, P. Korkolopoulou, J. Boletis, P. Efstratios

^*^Athens University, Pathology, Greece

**Objective:** Recent studies have revealed that the forkhead transcription factor FOXP3, a key element in the development and homeostasis of CD4+CD25+ regulatory T cells (Tregs), plays an important role in the pathogenesis of SLE. The aim of this study was to investigate FOXP3 immunohistochemical expression in a series of SLE patients in relation with histological parameters, activity and chronicity index.

**Method:** Thirty renal biopsies from patients with various classes of LN were investigated with respect to FOXP3 expression. A standard immunohistochemistry was performed using a monoclonal antibody against FOXP3. Immunohistochemistry was evaluated semiquantitatively in interstitial tissue, renal tubules and glomeruli.

**Results:** FOXP3 staining demonstrated a nuclear intense localization. It was mainly observed in interstitial inflammation of hyperplastic cases (classes III and IV) showing a periglomerular predilection. In cases with crescent formation, rare FOXP3 positive cells were also observed inside crescents. Although the number of cases is still too restricted to permit statistical analysis a higher FOXP3 labeling index seemed to coincide with a higher activity index.

**Conclusion:** FOXP3 immunohistochemical staining seems to be associated with histological disease activity in LN which is in keeping with studies showing FOXP3 m-RNA upregulation in the urine of patients with active LN

**PS-21-008**

**Renal diseases in patients with Human Immunodeficiency Virus (HIV) disease**

P. Constantinou ^*^, I. Themeli, D. Riga, N. Poulianitis, T. Argyrakos, C. Vourlakou

^*^Athens, Greece

**Objective:** We report our experience that illustrates the heterogeneous nature of HIV-associated renal diseases. We conducted a retrospective review of the renal biopsies in HIV-positive patients in order to describe both “classic HIV Associated Nephropathy- HIVAN” and non-HIVAN pathologies.

**Method:** Retrospective review of six HIV patients from 2010 to 2012 with renal diseases confirmed by percutaneous renal biopsy.

**Results:** Four patients were African or Asian and two Caucasian. Two patients had classical HIVAN with proteinouria, rapidly progressive renal failure and features of focal and segmental glomerulosclerosis (collapsing variant of FSGS), with associated tubulo-interstitial nephritis (TIN)–pan nephropathy. Two patients had IgA Nephropathy and one of them with associated features of FSGS and tubular changes suggestive of HIVAN. One patient had only interstitial nephritis and one patient membranoproliferative nephropahy with associated significant TIN.

**Conclusion:** 1. HIVAN and IgAN were the commonest renal diseases found in this group of the patients; however, a variety of other pathologies were seen. 2. To distinguish HIVAN from other forms of renal diseases, patients who are HIV-seropositive require a renal biopsy when their daily protein excretion is greater than 1 g, 3. The commonest finding is a collapsing form of FSGS with tubulointerstitial scarring, atrophy and marked microcystic dilatation of the tubules.

**PS-21-009**

**Intrarenal plasma cells may predict clinical outcome in ANCA associated necrotising glomerulonephritis**

S. Brix^*^, E. Herden, M. Noriega, G. Stege, U. Kneissler, G. B. Wolf, W. Jabs, U. Helmchen, U. Panzer, R. A. Stahl, T. Wiech

^*^University Hospital Hamburg, Internal Medicine–Nephrology, Germany

**Objective:** The potential role of intrarenal plasma cell infiltration and the higher organisation of lymphocytic infiltrates in antineutrophil cytoplasmic antibody (ANCA) associated glomerulonephritis has not been studied in greater detail.

**Method:** We prospectively analysed the localisation and organisation of CD3+, CD20+ and plasma cells in kidney biopsies of patients with ANCA associated renal disease (*n* = 49, *n* = 14 respectively). Glomeruli were categorised for different degrees of damage. Periglomerular and tubulointerstitial compartments were defined and tubulointerstitial lymphocytes were divided in scattered, nodular and lymph follicle like structures. Plasma cells were counted using immunohistochemical stains (Immunoglobulins A, G and M).

**Results:** At the time of renal biopsy, patients with high organised lymphocytic infiltration had the same renal function as patients with scattered intrarenal lymphocytes. During follow up, they were more likely to reach end stage renal disease (*p* < 0.05). Patients with plasma cell rich biopsies were in a greater percentage dialysis dependent at time of renal biopsy (*p* < 0.05), but their renal function seemed to improve after 6 months compared to patients with less intrarenal plasma cells.

**Conclusion:** An increasing organisation of lymphocytic infiltration might account for refractory disease, whereas plasma cells could be a predictive marker for treatment response.

**PS-21-010**

**A clinico-pathological comparison of adult-onset Henoch-Schönlein Purpura Nephritis (HSPN) and IgA Nephropathy (IgAN)**

M. Janicka-Jedynska ^*^, A. Wozniak, J. Zurawski, I. Piechocka-Idasiak, E. Kaczmarek, J. Bulak

^*^Uniwersity of Medical Sciencie, Patomorphology, Poznan, Poland

**Objective:** HSPN is considered a systemic form of IgAN, both are very similar in immunopathological changes, and therefore considered one disease entity. The present study aimed to characterize their relationship through clinico-pathological comparison. We also used immunohistochemical markers in an attempt to find prognostic indicators of poor outcome.

**Method:** Sixty-four adults with IgAN and HSPN were enrolled in this study. Their clinical manifestations and follow-up data were analyzed. Renal pathological findings (a modified histological scoring system to evaluate chronicity), IF and electron microscopy (EM) were also compared. Immunohistochemistry for Ki-67, PCNA and p27 was performed.

**Results:** Clinical classification demonstrated that more severe conditions were found in the IgAN. The renal pathological investigation showed the higher degree of glomerulosclerosis and tubulointerstitial fibrosis in IgAN, but endothelial proliferation was more prominent in HSPN (*p* < 0.01). Thin basement membrane was found in IgAN, but not in HSPN. The location of IgA deposits in IF and EM also differed. There was a correlation of proliferation index and p27 expression with glomerulosclerosis and deterioration of renal function.

**Conclusion:** Significant clinico-pathological differences were found, which does not support the one disease entity hypothesis. HSPN and IgAN are probably two diseases with similar immune abnormalities.

**PS-21-012**

**Focal segmental glomerulosclerosis tip-lesion variant: Clinicopathological analysis of 20 cases**

D. Baydar ^*^, C. Aydin, S. Mungan, E. Turkmen, M. Altindal

^*^Hacettepe University, Pathology, Ankara, Turkey

**Objective:** Tip-lesion variant is known to be one subtype of focal segmental glomerulosclerosis (FSGS). In this study we have aimed to analyze clinicopathological features of this entity in our cohort.

**Method:** Pathology archives of our institution were reviewed. Twenty-cases of primary FSGS tip variant were found between 1998 and 2013. Their detailed histomorphological features were recorded. The clinical and follow-up information were gathered from hospital files.

**Results:** Patients had equal gender distribution (age:21–82). The mean proteinuria, serum albumin and creatinine at presentation were 5,5 g/day, 2,3 g/dl and 0,95 mg/dl respectively. Ratio of glomeruli with tip-lesion was between 2 and 100 %. There was focal mesangial proliferation in 5/20, variable interstitial fibrosis/tubular atrophy in 5/20, patchy inflammation in 2/20. All were treated with steroids, six with additional azothioprin. Follow-up data were available in 16 (3–136, mean 35.7 months). Fourteen-patients (87.5 %) showed complete, 2 (12.5 %) showed partial remission. One (1/14) relapsed after a year. Median proteinuria was 211 mg/day, mean serum creatinine was 0,9 mg/dl at the last control. None of the clinical/histomorphologic parameters correlated with outcome.

**Conclusion:** This study confirms favorable prognosis of FSGS tip-variant. This is unrelated to morphological findings and presenting clinical features.

**PS-21-013**

**Comparation of histological findings in renal biopsies of the patients with non-lupus „full house“nephropathy and patients with lupus nephritis**

J. Vjestica ^*^, M. Zivotic, S. Cirovic, R. Naumovic, S. Simic-Ogrizovic, D. Dundjerovic, J. Markovic-Lipkovski

^*^University of Belgrade, School of Medicine, Dept. of Pathology, Serbia

**Objective:** Renal involvement in systemic lupus erythematosus (SLE) commonly includes lupus nephritis with “full house” immunofluorescence (IF) pattern, but also could involve kidney diseases with various IF findings. However, the “full house” pattern can be occasionally found in non-SLE patients. Our aim was to analyze type of glomerulonephritis (GN), activity index (AI) and chronicity index (CI) of renal lesions, in patients with “full house” nephropathy in absence of any clinical and/or serological evidence of SLE, and compare them to those characteristics in SLE patients.

**Method:** Renal biopsies from 2002 until 2012 year were reviewed. Among 2151 patients, 51 patient had “full house” nephropathy without SLE, 41 SLE patient had “full house” GN, while 40 SLE patients had GN without “full house” IF pattern.

**Results:** Patients with non-lupus “full house” mainly had membranous GN 27 (52,9 %). Diffuse- proliferative GN had 28 (68,3 %) SLE patients with “full house” IF and 23 (57,5 %) SLE patients without “full house” pattern. AI was statistically lower in non-lupus “full house” nephropathies, while there was no difference in CI among analyzed groups.

**Conclusion:** Patients with non-lupus “full house” nephropathy should be monitored regarding the possible progression of active renal lesions and developing of underlying clinically silent immune mediated condition, including SLE.

**PS-21-014**

**Nine year registry of native renal biopsy from the Department of Pathology of Dubrava University Hospital of Zagreb**

J. Bacalja^*^, S. Bulimbasic, P. Šenjug, A. Pacic, I. Horvatic, M. Knotek, K. Galešic, D. Galešic Ljubanovic

^*^University Hospital Dubrava, Dept. of Pathology, Zagreb, Croatia

**Objective:** To show the epidemiology of renal diseases.

**Method:** We performed a retrospective statistical analysis of native renal biopsies diagnosed in Dubrava University Hospital Zagreb, during period April 2003–December 2012.

**Results:** During analyzed period a total of 1981 native renal biopsies were found. Patient’s age range 1–88 years (average 47.1). Biopsies originate from 11 Croatian hospitals and Mostar University Hospital (Bosnia and Herzegovina). Men had 1.3× more biopsies than women (M:F = 1,085:806). Glomerular diseases were the most frequent diagnosis (70.9 %) followed by diseases of the blood vessels (8.8 %) and tubulointerstitium (5.0 %). Primary glomerular diseases were 1,4× more frequent than secondary. IgA nephropathy was the most common disease diagnosed in 18.9 % biopsies, followed by focal segmental glomerulosclerosis (12.1 %) and membranous glomerulonephritis (8.4 %). Pauci-immune glomerulonephritis was the most common secondary glomerular disease in our sample (6.9 %), followed by lupus nephritis (5.6 %) and diabetic nephropathy (5.5 %). Some diseases were more frequently diagnosed in women than in men and vice versa.

**Conclusion:** At present time, two-thirds of renal biopsies performed in Croatia are processed by our laboratory. We believe that presented data are representative and provide detailed information about incidence, prevalence and trends in epidemiology of renal diseases in Croatia.

**PS-21-016**

**Computer-assisted automated quantification of Tubulo-Interstitial Fibrosis (TIF) in chronic glomerulopathies with relation for predicting long-term prognosis**

A. Wozniak ^*^, J. Bulak, M. Janicka-Jedynska, I. Idasiak-Piechocka, L. Lapaj, A. Gorna

^*^University of Medical Science, Clinical Pathomorphology, Poznan, Poland

**Objective:** Chronic kidney disease at a certain advanced stage inevitably progresses to ESKD. Glomerular diseases enter a progressing course after encroaching onto the tubule leading to TIF. Numerous reports have demonstrated the strong association between the extent of TIF and renal function. The aim of our study was computer assisted semiautomated (SADA) and automated (ADA) analysis of TIF.

**Method:** The study group consisted of 49 cases: FSGS (*N* = 22), IgAN (*N* = 6), membranous glomerulopathy (*N* = 6) and membrano-proliferative glomerulonephritis (*N* = 5). The clinical manifestations and follow-up data were analyzed. %TIF was determined by quantitation of red material in Sirius Red-stained sections by computerized digital analysis (Olympus DP12, ×40, ImageJ program).

**Results:** The comparison between two methods showed no significant differences (*p* < 0.001), when ADA was much less time consuming. Extent of TIF correlated significantly with the clinical parameters (proteinuria, creatinine levels). The majority of patients (*N* = 36) were in CRF at final review (10 years). They had significantly higher values of %TIF as compared to patients who did not progress to CRF (*p* < 0.001).

**Conclusion:** ADA has proven to be a useful tool in every day nephropathologist practice. The close correlation between %TIF and clinical parameters suggested that quantitative measurement of %TIF may predict functional consequences of glomerulopathies.

**PS-21-017**

**Study on the progression of renal failure in a model of fetal reprogramming using markers of epithelial mesenchymal transition**

R. Corrêa ^*^, K. R. Martins Pucci, C. D. Pereira Júnior, P. B. Idaló, L. P. Rocha, C. S. Oliveira Guimarães, A. C. Rogélis, L. B. Rocha, M. A. dos Reis, N. O. Saraiva Camara

^*^Triângulo Mineiro Federal Universidade, ICBN, Uberaba, Brazil

**Objective:** We aimed to evaluate the progression of folic acid (FA)-induced renal failure in the renal tissue of young offspring of diabetic mothers.

**Method:** We divided 2- and 5-month-old (mo) offspring into 4 groups: CC (controls, receiving vehicle); DC (diabetics, receiving vehicle); CA (controls receiving FA solution, 250 mg/kg); and DA (diabetics receiving FA solution). Renal function and morphological changes were evaluated. Gene and protein expression of epithelial mesenchymal transition (EMT) markers was analyzed by immunohistochemical tests and qPCR.

**Results:** Glomeruli number was reduced in the diabetic groups. Creatinine, urea, TGF-β1 levels and urinary space were increased in the CA and DA groups. TGF-β3 expression was higher in the DC than in the CC group at 5 month, and in 2- and 5-months CA and DA offspring, with a significant increase in DA compared to CA 2-month offspring. Actin expression patterns were similar to that of TGF-β3. Vimentin expression at 5 month was higher in DC than in the CC group.

**Conclusion:** Thus, fetal reprogramming promotes remarkable changes in the kidney morphology and function in offspring. Further, the expression of EMT markers demonstrated that renal failure progression may be faster in younger offspring of diabetic dams. Financial support was provided by CNPq, CAPES, FAPEMIG, FUNEPU and FAPESP.

**PS-21-018**

**Histological kidney changes associated with high rhEPO doses in a rat model of chronic renal failure**

H. Vala ^*^, P. Garrido, S. Ribeiro, J. Fernandes, B. Parada, R. Alves, L. Belo, E. Costa, A. Santos-Silva, F. Reis

^*^Escola Superior Agrária de Viseu, Instituto Politécnico de Viseu, Portugal

**Objective:** Recombinant human erythropoietin (rhEPO) is extensively used to treat anaemia of chronic kidney disease. This study aims to evaluate kidney damage associated to high rhEPO doses in a rat model of chronic renal failure (CRF).

**Method:** Three groups (*n* = 7) of male Wistar male (280 g) were studied during 12 weeks: Sham; CRF: (5/6) nephrectomy; CRF+rhEPO (200IU/kg/week of beta-EPO, Recormon®, sc). In different times, hematological data and renal function was assessed. Anti-EPO antibodies were measured by ELISA. Kidneys lesions were analyzed with H&E and PAS staining.

**Results:** CRF rats developed anaemia, which was corrected by rhEPO until the 9 week, when rats developed anaemia due to production of anti-rhEPO antibodies. CRF group showed a significant increase in BUN and creatinine, which were not prevented by rhEPO therapy. The renal impairment found in CRF rats was accompanied by kidney tubulointerstitial, glomerular and vascular lesions. rhEPO therapy aggravated the tubulointerstitial lesions.

**Conclusion:** In our model, rhEPO therapy was associated with correction of anaemia but there was a negative impact on the kidney, viewed by aggravation of tubulointerstitial lesions. Whether this profile is related to the high doses and/or the development of resistance induced by anti-rhEPO antibodies deserves further elucidation.

**PS-21-019**

**Immunosuppressive therapy optimization applied in transplantation: Renal benefits of cyclosporine dose reduction and conversion to Sirolimus in a rat model**

H. Vala ^*^, J. Sereno, P. Rodrigues-Santos, B. Parada, F. Teixeira, F. Reis

^*^Escola Superior Agrária de Viseu, Instituto Politécnico de Viseu, Portugal

**Objective:** Sirolimus (SRL) have been pointed as a feasible option for minimize the use of Cyclosporin A (CsA), especially because of putatively less nephrotoxicity. This study aimed to characterize the histological lesions and the molecular pathways implicated in CsA-dose reduction followed by SRL conversion.

**Method:** The following 3 groups (*n* = 6) were tested during 9 week: Vehicle, CsA 5 mg, and reduction+conversion (CsA-30 mg/3 week + CsA-5 mg/3 week + SRL-1 mg/3 week). BP and HR were monitored. Blood and urine were collected for renal evaluation and kidney for gene expression and histology: H&E, PAS and Gordon & Sweets staining.

**Results:** After 3 week, only CsA-30 mg treatment promote hypertension (*p* < 0.001) with tachycardia (*p* < 0.001), increase serum levels of creatinine and urea (*p* < 0.001, both) and decreased GFR (*p* < 0.05), accompanied by important kidney lesions: arteriolosclerosis and arteriosclerosis, nodular sclerosis, vascular pole hialinization and Bowman’s capsule thickening, tubular calcification, interstitial fibrosis and hyaline cylinders. Dose reduction only partially ameliorated kidney damage, while SRL conversion produced remarkable improvement in glomerular and tubulointerstitial lesions, accompanied by reduction of oxidative stress, proliferation and angiogenesis markers.

**Conclusion:** CsA nephrotoxicity is dose dependent and moderate dysfunction could be ameliorated/prevented by SRL conversion, if replacement is performed before severe dysfunction.

**PS-21-020**

**The 5/6 nephrectomized rat as a good model for the study of anaemia associated with functional and structural kidney damage**

H. Vala ^*^, P. Garrido, S. Ribeiro, J. Fernandes, B. Prada, R. Alves, L. Belo, E. Costa, A. Santos-Silva, F. Reis

^*^Escola Superior Agrária de Viseu, Instituto Politécnico de Viseu, Portugal

**Objective:** The anaemia associated with chronic kidney disease (CKD) is frequently characterized by functional iron deficiency. This study intended to characterize a rat model of CKD concerning the anaemia/iron deficiency as well as the kidney dysfunction/structural damage.

**Method:** Two groups (*n* = 7) of male Wistar rats (280 g), Sham and CRF, were studied during 12 weeks. In different times, blood was collected to assess renal function, hematological data and iron metabolism markers. Kidneys were collected for histomorphological analysis and liver for gene expression of iron metabolism markers by RT-qPCR.

**Results:** The CRF model showed a sustained increase of creatinine and BUN, which was accompanied by anaemia, as well as hypertension and dyslipidaemia. Additionally, a significant decrease of serum iron and transferrin levels was obtained, and reduced liver mRNA expression of genes for hepcidin, transferring receptor, hemojuvelin, haemochromatosis, bone morphogenetic protein 6 and erythropoietin receptor. The CRF rats showed renal hypertrophy and structural damage, represented by a) glomerular: mesangial expansion; b) tubulointerstitial: hyaline cylinders formation and interstitial fibrosis and tubular atrophy.

**Conclusion:** This rat model of remnant kidney disease is a good model to study functional iron deficiency anaemia associated with functional and structural kidney damage.

**PS-21-021**

**Xanthine oxidase and NO synthase expression in kidney tissue of streptozotocin-induced diabetic rats**

S. Isajevs ^*^, J. Sokolovska, N. Sjakste

^*^University of Latvia, Dept. of Pathology, Riga, Latvia

**Objective:** The present study investigated the role of endothelial and inducible nitric oxide synthase (eNOS and iNOS) and xanthine oxidase (XO) in the pathogenesis of streptozotocin-induced diabetic changes in the kidney tissue.

**Method:** Diabetes mellitus was induced in rats by a single injection of streptozotocin (STZ) at a dose of 50 mg/kg. The XO, eNOS and iNOS protein expression in the kidney was studied by immunohistochemistry.

**Results:** Obtained results showed that STZ administration incresed the numbers of XO-positive cells in kidney tissue compared to control group (27 ± 7 vs. 8 ± 2 cells/mm2; *p* = 0.002). XO was expressed predominantly in kidney cortical tissue in proximal tubules epithelial cells. The expression of eNOS and iNOS was mainly localized in the endothelium of preglomerular and postglomerular vessels and glomerular capillaries, and was increased in the diabetic kidneys. At contrast, the eNOS expression was also observed in medullary collecting tubules, and it was decreased in STZ-treated rats (3 ± 2 vs. 9 ± 3 cells/mm2; *p* = 0.013).

**Conclusion:** The present study suggests that increased XO in proximal tubules and decreased eNOS expression in medullary collection tubules, with concomitant increased eNOS and iNOS expression in the microvasculature plays a pivotal role in the kidney damage in diabetes mellitus.

**PS-21-022**

**Losartan improves kidney function and decreases oxidative stress and tubular injury in spontaneously hypertensive rats during acute renal failure**

J. Markovic-Lipkovski ^*^, M. Ivanov, Z. Miloradovic, N. Mihailovic-Stanojevic, J. Grujic-Milanovic, D. Jovovic, D. Karanovic, U. J. Vajic

^*^University of Belgrade, Faculty of Medicine, Dept. of Pathology, Serbia

**Objective:** Acute renal failure (ARF) is a vicious illness, especially when it is combined with arterial hypertension. We investigated effects of losartan in spontaneously hypertensive rats (SHR) with ARF in order to determine the role of angiotensine II.

**Method:** The right kidney was removed and the renal ischemia was performed by clamping the left renal artery for 40 min. SHR groups received losartan (competitive antagonist of type I angiotensine II receptor) or vehicle in the femoral vein 5 min during and 175 min after the period of ischemia. All biochemical parameters were measured and kidney tissue was analysed morphologically and immunomorphologically applying Bax and Bcl-2 antibodies 24 h after ischemia.

**Results:** Treatment with losartan induced significantly increased urea clearance, decreased creatinine, improved oxidative stress parameters, increased HDL cholesterol and decreased tubular damages vs. ARF. High expression of pro-apoptotic Bax protein in proximal tubules of SHR with ARF was lower in losartan-treated animals. Anti-apoptotic Bcl-2 protein was decreased in distal tubules of losartan-treated postischemic SHR, indicating lower degree of apoptotic damage in them.

**Conclusion:** Treatment with losartan can improve kidney function, decrease oxidative stress in SHRs with ARF. Angiotensine II receptor blockade may have beneficial effects on tubular injury of postischemic SHR.

**PS-21-023**

**Effect of chaethomellic acid on renal function in rat model of chronic renal failure**

A. Nogueira^*^, C. Mega, E. Fonte, P. Oliveira, B. Colaço, J. M. López-Novoa, A. Colaço, M. J. Pires

^*^Dep of Veterinary Science, Center Study Animal Sciences, Vila Real, Portugal

**Objective:** To study the effect of chronic treatment with chaethomellic acid (CA), a highly specific inhibitor of ras farnesyl-protein transferase, on the renal function of rats with renal failure induced by renal mass reduction.

**Method:** Male Wistar rats were subjected to 5/6 nephrectomy (RMR) or sham-operated (SO). One week after surgery, rats have been placed in four experimental groups: RMR: rats without treatment (*n* = 13); RMR+CA: rats treated with CA (*n* = 13); SO: rats without treatment (*n* = 13); SO+AC: rats treated with CA (*n* = 13). CA was intraperitoneally administered in a dose of 0.23 μg/Kg three times a week for 6 months. Creatinine, blood urea nitrogen (BUN) and protein were measured in serum and/or urine by routine laboratory techniques.

**Results:** BUN, creatinine, and proteinuria were significantly lower and creatinine clearance was significantly higher in SO and SO+AC groups when compared with RMR and RMR+AC groups. There were no differences in creatinine, proteinuria and creatinine clearance between RMR and RMR+AC groups. Anyway, RMR+AC group showed significant lower BUN and lower creatinine and proteinuria, and higher creatinine clearance than RMR group.

**Conclusion:** In a model of renal failure induced by RMR, 6 months of treatment with CA may have some beneficial effect on renal function.

**PS-21-024**

**Apoptosis as a prognostic marker in prediction of renal injury, after acute bleeding and volume replacement with HES 130/0.4 or Ringer solution, in a pig model**

H. Vala ^*^, R. Cruz, A. Machado, C. Venâncio, J. Mesquita, A. Silva, A. Liza ortiz, D. Ferreira

^*^Escola Superior Agrária de Viseu, Instituto Politécnico de Viseu, Portugal

**Objective:** The aim of this study is identify and quantify apoptosis in renal tissue, using a biochemical marker (TUNEL) in a pig haemorrhagic model, after intravascular volume replacement with Ringer’s lactate or Hydroxyethylstarch 130/0.4) solutions.

**Method:** 18 Large White pigs underwent total intravenous anaesthesia with propofol and remifentanil. 25 ml/kg of arterial blood were removed from the femoral artery. Volume was replaced, RL, in group1 (*n* = 6) and HES 130/0.4, in group2 (*n* = 6), 20 min after bleeding. The control group did not face bleeding and volume reposition. One hour after volume replacement, pigs were euthanized with intravenous KCl, and renal fragments were taken for several studies, including immunohistochemically with TUNEL method for apoptosis detection. ANOVA was used to compare data between groups.

**Results:** Apoptosis was, detected in all groups, mainly in epithelial tubular cells. The number of apoptotic cells per mm2 was lower in group 1 (11.94cells/mm2), when compared with group 2 (67.94cells/mm2) and with the control group (146.34cells/mm2). Levels of apoptosis were significantly lower in group1(RL), comparing with the control group(*P* < 0.05)

**Conclusion:** The median apoptotic levels were significantly lower in pigs, subjected to fluid replacement with RL, when compared with HES 130/0.4. Ringer lactate might promote better renal perfusion in the presence of severe hypovolaemia following acute haemorrhage.

**PS-21-026**

**Renal malakoplakia in a transplanted kidney: A case report and review of the literature**

E. Borg ^*^, J. Degaetano

^*^Mater Dei Hospital, Dept. of Histopathology, Msida, Malta

**Objective:** Malakoplakia is a rare chornic inflammatory condition which is presumed to be due to impaired killing of bacteria by macrophages. Renal malakoplakia in transplanted kidney is very rare.

**Method:** A 22 year old female on immunosuppressive therapy for a transplanted kidney presented with fever and rapid deterioration in renal function. Initial biopsy showed unremarkable looking renal parenchyma. In view of further clinical deterioration, nephrectomy of the transplanted kidney was performed.

**Results:** The nephrectomy specimen showed numerous yellowish-white soft, well defined nodules. The rest of the kidney looked unremarkable. The literature was searched for cases of renal malakoplakia in transplanted kidneys. The data obtained was tabulated. Malakoplakia of a transplanted kidney is very rare. Only 14 cases have been reported since 1977. All patients were on immunosuppressive therapy and experienced at least one preceding episode of urinary tract infection. There was deterioration in renal function needing dialysis, and transplant nephrectomy in the majority of cases.

**Conclusion:** Renal malakoplakia in kidney transplants is a very rare condition and should be included in the differential diagnosis of unusual histological features in post transplant biopsies.

**Renal malakoplakia in a transplanted kidney:** 
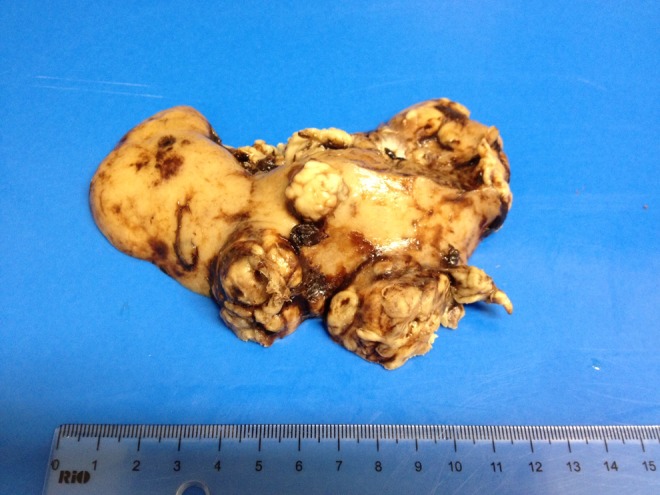


**PS-21-027**

**TREX1 mutations–one of the genetic causes for renal vascular diseases in younger patients**

T. Menter^*^, D. Winkler, G. Isimbaldi, H. Hopfer, M. Mihatsch

^*^Universitätsklinik Basel, Inst. für Pathologie, Switzerland

**Objective:** In most cases, vascular pathology (VP) in renal biopsies is due to hypertension, diabetes mellitus or more rarely inflammatory processes. However, there are also cases in which VP can be related to specific genomic alterations.

**Method:** We present a Caucasian male patient who had suffered from migraine-like headaches for years and experienced neurologic complications and progressing failure of other organ systems. A renal biopsy was performed at the age of 40. This biopsy demonstrated severe stenosing arteriolohyalinosis, intimal fibrosis of interlobular arteries and glomerular abnormalities showing irregular basement membranes. The findings were considered to be due to thrombotic microangiopathy.

**Results:** Eight years later, the symptoms of the patient could be attributed to hereditary systemic angiopathy (HSA), as a mutation in the TREX1-gene was found. HSA is part of the family of syndromes linked to alterations on chromosome 3p21.1–3p21.3 such as cerebroretinal vasculopathy (CRV), hereditary endotheliopathy with retinopathy, nephropathy and stroke (HERNS) and hereditary vascular retinopathy (HVR). Similarities and differences of these entities will be discussed.

**Conclusion:** VP in renal biopsies based on distinct genomic alterations should be kept in mind by nephropathologists, especially in younger patients and might have major impacts on the further treatment of the patients.

**PS-21-028**

**Ultrastructural deposits appearing as–zebra bodies–in renal biopsy: Fabry’s disease?**

M. Reis ^*^, P. Menezes, J. Machado, S. Iwamoto, M. Ferreira, M. Freire, F. Custodio

^*^University Triângulo Mineiro, Nephropathology Service, Uberaba, Brazil

**Objective:** Cases reports: Fabry’s disease (FD) is a genetic disorder caused by a deficiency of alpha-galactosidase A. Some drugs, such as hydroxychloroquine, can produce renal deposits that mimic the morphological findings seen in FD, characterizing a type of drug-induced renal phospholipidosis.

**Method:** Kidney biopsy: Light microscopy, immunofluorescence and electron microscopy.

**Results:** Case 1: A woman with systemic lupus erythematosus who had been using hydroxychloroquine for 14 months presented with proteinuria. A renal biopsy revealed deposits compatible with FD, but alpha-galactosidase A activity was normal. These deposits were no longer detected in a second renal biopsy after discontinuation of the medication. Case 2: A man presented with acroparesthesia, angiokeratomas, cornea verticillata, cardiac alterations and proteinuria. Deposits compatible with FD were detected upon renal biopsy and measurement of alpha-galactosidase A showed no activity.

**Conclusion:** Clinical investigation is necessary in the case of a suspicion of FD upon renal biopsy for confirmation of the diagnosis and drug-induced renal phospholipidosis should be included in the differential diagnosis. Financial support: CNPq, Capes, Fapemig, Funepu, UFTM.

**PS-21-029**

**The case of fibronectin glomerulopathy (pathomorphological study)**

V. Sipovskiy ^*^, V. Dobronravov, A. Smirnov

^*^Research Institute of Nephrology, Dept. of Pathology, St. Petersburg, Russia

**Objective:** Differential diagnosis of fibronectin glomerulopathy. is one of the most difficult tasks in the field of non-tumor renal disease.

**Results:** Patient S. 27 years, with symptoms of nephrotic syndrome: Daily proteinuria 11.4 g/day., Albumin-21.0 g/l, total protein-45.0 g/l, creatinine 0.60 mmol/l, urea 2.9, total cholesterol–10, 43 mmol/L. Light microscopy: Glomerular basement membranes were thickened by the accumulation of homogeneous eosinophilic, PAS-positive and Jones-negative masses. Segmental zone the doubling and splitting of basement membranes were revealed. Lumen of the capillary loops were narrowed and focally obliterated. Congo-red stain was negative. Immunofluorescense (cryostat sections): A linear deposits IgA, (2 +), IgM (2 +), IgG (2 +), C3 (2 +), lambda (+)contoured the capillary loops on the outer side. Immunohistocemistry: (paraffin-embedded sections): The deposits of the fibronectin were identified in the connective tissue of tubulointerstitium, tubular basement membranes and into the subendothelial and mesangial PAS-positive masses (Fig.1d). Electron microscopy: Multiple subendothelial deposits of various sizes, merging between themselves and occluded the lumen were defined. in the mesangium and capillary loops. Deposits had small granular structure with focal, short fibrillar substructures, but without the formation of a “cotton-like” structures.

**Conclusion:** We suppose that we have idetified fibronectin glomerulopathy

**D-Immunohistochemistry: Products reaction to fibronectin was located in the tubulointerstitium, subendothelial glomerular capillary walls and mesangial matrix (black arrow). Magnification x 40.:** 
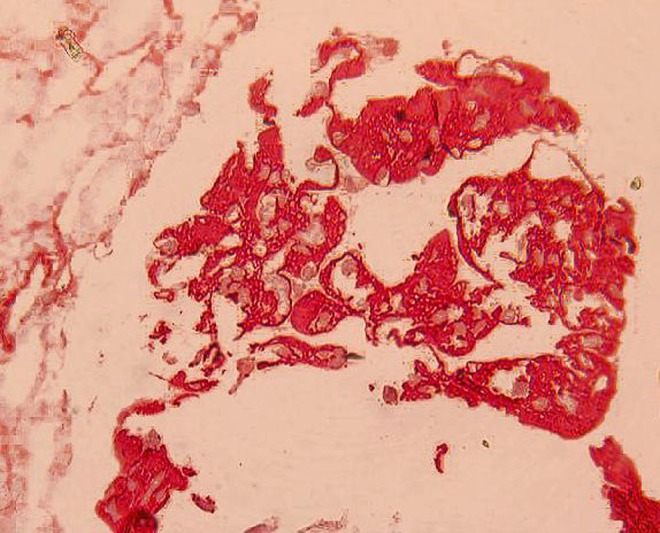


**PS-21-030**

**Alport’s syndrome or thin basement membrane nephropathy–Electron microscopy and study of collagen IV alpha chains**

M. Reis ^*^, V. Laterza, J. Machado, M. Silva, L. Araújo, F. Oliveira, R. Corrêa

^*^University Triângulo Mineiro, Nephropathology Service, Uberaba, Brazil

**Objective:** To diferenciate Alport’s Syndrome (AS) and Thin Basement Membrane Nephropathy (TBM) though electron microscopy and type IV collagen alpha chains (1, 3 and 5) Immunohistologic staining.

**Method:** 22 renal biopsies of AS or TMG patients were analysed using: 1. light and electron microscopies with basement membrane (BM) morphometry; 2. immunofluorescence: a) direct–immunocomplex–IgA, IgG, IgM, kappa, lambda, C1q C3 and fibrinogen and b) indirect: type IV collagen alpha1, 3 or 5 chains.

**Results:** Groups: 1. AS-EM–typical alterations under electron microscopy (EM) with thickening and irregular contours of the glomerular BM and splitting of the lamina densa, (*n* = 9); 2. AS–alpha 5 chain (collagen IV) absent (*n* = 8) and TBM (*n* = 5). Group AS-EM showed significantly: larger number of male patients, lower age average, more proteinuria, BM larger thickness and positive correlation between age and levels of serum creatinine.

**Conclusion:** AS and TBM are genetical diseases due to a collagen IV alpha chain gene mutation. AS-EM group showed earlier and more severe symptoms than the other groups. In some cases there is only decrease in GBM thickness in both diseases (AS and TBM). In these cases the research of collagen IV alpha chains is essential to diferenciate one disease from the other. Financial support: CNPq, Capes, Fapemig, Funepu, UFTM

**PS-21-031**

**Renal transplant in HIV-2 patient: Case report**

J. Cassis ^*^, A. Natário, A. Weigert

^*^Caxias, Portugal

**Objective:** 54 years old black male patient was transferred to Portugal from Guinea Bissau for renal replacement therapy. The cause of ESRD was believed to be hypertension. The patient was diagnosed with HIV-2 when started haemodialysis. After 13 years the patient underwent renal transplantation. He developed delayed graft function which recovered with thymoglobulin. Immunosupression was reduced 14 months later due to high levels of BK virus in plasma and urine. The patient then developed graft dysfunction; a renal biopsy was performed.

**Method:** Chronic humoral rejection (diffusely positive C4d) with no signs of polyomavirus nephropathy (SV40 negative) was observed.

**Results:** Plasmapheresis, IVIG and rituximab were given to the patient with good response. The follow-up renal biopsy showed no signs of humoral rejection or polyomavirus nephropathy.

**Conclusion:** HIV infection is no longer a contraindication for renal transplantation and graft survival rates can be similar to non-HIV transplanted patients. However this is a new territory to explore as there are no definite guidelines regarding the management of these patients and the existent data comes from small cohort studies in patients with HIV-1. This is the first case in literature of a renal transplantation in a HIV-2 infected patient.

**PS-21-032**

**Focal crescentic glomerulonephritis in a patient with lymphoplasmacytic lymphoma involving the kidney: A case report and review of literature**

J. A. Merino Garcia ^*^, J. Gimeno Beltrán, L. Comerma Blesa, B. Bellosillo Paricio, T. Baró Tomas, A. Berrada, M. A. Orfila-Gornés, S. Serrano Figueras

^*^Consorci Mar Parc de Salut, Barcelona, Spain

**Objective:** Thirty-six-year-old patient diagnosed of a nodal lymphoplasmacytic lymphoma showing an acute deterioration in renal function.

**Method:** Renal biopsy was performed, 14 glomeruli were evaluated, 5 globally sclerosed and 4 with cellular crescents. No hyaline thrombi were detected in capillaries neither immune deposits/organized deposits under fluorescence and electronic microscopy examination. Moreover, there was a dense intersticial lymphoid infiltrate, consisting of small-sized B-cells with CD5 co-expression, admixed with plasma cells harboring light chain restriction. A clonal rearrangement of IgH gene was detected, identical to the one detected in a nodal sample.

**Results:** In summary, we present the case of a low grade B-cell lymphoma involving the kidney associated with a focal crescentic glomerulonephritis without glomerular deposits.

**Conclusion:** Low-grade lymphoproliferative disorders do often affect the kidney. Kidney failure can be related to direct infiltration and destruction of the renal parenchyma, or to indirect mechanisms, such as chemotheraphy nephrotoxicity or renal deposition of immunoglobulins producing true glomerulonephritis. Crescentic glomerulonephritis has been rarely described in association to lymphoproliferative diseases. This could be related to paraprotein or cryoglobulin production by lymphomatous cells. Nevertheless, in most of the cases, the cause cannot be demonstrated, suggesting activation of the immune system by lymphoma cells with consequent glomerular damage.

**PS-21-033**

**Papillary renal cell neoplasia as an incidental finding in allograft kidney biopsy**

G. Hariklia ^*^, T. Eleni, V. George, K. Evangelia, S. Anastasios, P. Erasmia, P. Efstratios

^*^Athens University, Pathology, Greece

**Objective:** To investigate the cause of gradual deterioration of renal function in a 31 year old woman who received a cadaveric renal transplant in 1996.

**Method:** Renal biopsy was evaluated by means of standard histochemical stains, immunofluorescence and immunohistochemistry.

**Results:** The renal biopsy showed advanced chronic lesions (ci3-ct3-cv3) and 50 % global glomerulosclerosis. This was more or less expected. The unexpected finding was the focal demonstration in both cores of papillary formations, covered by cells without significant nuclear atypia or mitotic activity, immunoreactive for CD10, keratin-7 & racemase, showing a low Ki-67 labeing index and accompanied by the presence of psammoma bodies. The two foci measured 0,25 cm and 0,05 cm in each core respectively. These findings were consistent with a papillary renal cell neoplasm the discrimination between adenoma and carcinoma depending on the subsequent determination of its total greatest diameter. The MRI revealed an exophytic mass of 1.6 cm with marked enhancement after gadolinium injection. The lesion was peripheral and it was treated using percutaneous radiofrequency ablation (RFA). No macroscopic lesion was left after RFA.

**Conclusion:** Incidental neoplastic lesions are occasionally found in renal allograft biopsy specimens. When an incidental neoplasm is found, the pathological type should be defined, and imaging should be performed. Management is still controversial.
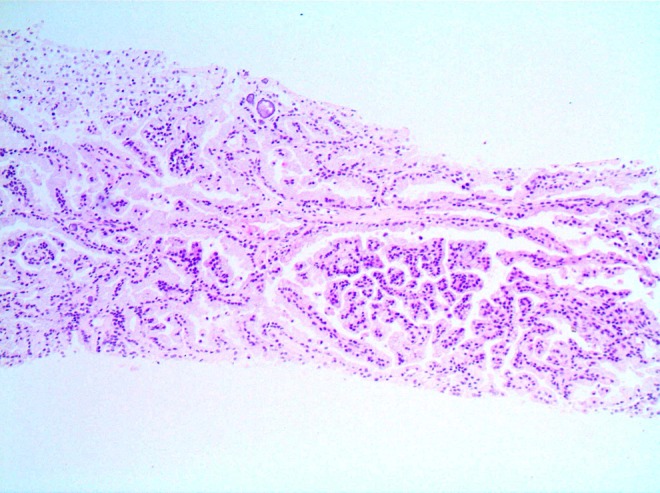


**PS-21-034**

**A case of C3 glomerulopathy: Diagnosis and differential diagnosis**

G. Hariklia ^*^, S. Konstadinos, K. Evangelia, S. Anastasios, L. Maria, D. Eugene, P. Efstratios

^*^Athens University, Pathology, Greece

**Objective:** The reclassification of membranoproliferative glomerulonephritis has led to the emergence of a new spectrum of diseases called C3 glomerulopathies. We present a case of C3 glomerulopathy in a young postpartum woman presenting with nephritic syndrome, normal renal function, hypocomplementemia and high ASTO titers. Of note, the patient has a positive family history of glomerulonephritis.

**Method:** Renal biopsy was evaluated by means of histochemistry, immunohistochemistry, immunofluorescence and electron microscopy.

**Results:** Light microscopy showed a glomerulonephritis with membranoproliferative pattern with diffuse and very strong C3 staining on immunofluorescence. Immunoglobulins, other complement factors and light chains were negative. Electron microscopy revealed mesangial, intramembranous, subendothelial and subepithelial deposits. Thus, a diagnosis of complement mediated C3-glomerulopathy, most probably C3-glomerulonephritis was supported.

**Conclusion:** C3 glomerulonephritis belongs along with Dense Deposit Disease in the newly described spectrum of C3 glomerulopathies characterized by abnormalities of the alternative complement pathway. Differential diagnosis between the two entities relies mainly on electron microscopy. However, an evaluation to detect abnormalities of the alternative pathway is indicated regardless of whether electron microscopic examination shows dense deposit disease or C3 glomerulonephritis.

Wednesday, 4 September 2013, 09.30–10.30, Pavilion 2

**PS-22 Poster Session Gynaecopathology**

**PS-22-001**

**Uterine cervical tubulosquamous polyp resembling a penis**

M. Fukunaga ^*^

^*^Jikei University, Daisan Hospital, Dept. of Pathology, Tokyo, Japan

**Objective:** This case report describes a case of tubulosquamous polyp resembling penis in the uterine cervix.

**Method:** This unique lesion was clinicopathologically analized.

**Results:** The polyp had a penis-like appearance; the tip portion looked like glans penis and the mid-portion resembled the shaft of the penis. Its surface was covered by squamous epithelium, and urethra-, corpus spongiosum penis-, and external orifice urethra- and foreskin-like tissues were observed although corpus cavernosum penis was not seen. Skene’s glands and Cowper glands were also observed. Immunohistochemically, Skene’s glands and the urethra-like epithelium were focally positive for prostate-specific antigen and/or prostatic acid proteins.

**Conclusion:** Histologically and immunohistochemically, the polypoid lesion overlapped with tubulosquamous polyp of the vagina and ectopic prostatic tissue of the uterine cervix and encompassed these lesions in the lower female genital tract. The most likely theory of histogenesis is a developmental anomaly and misplacement of Skene’s glands.

**PS-22-002**

**Ectopic complete hydatiform mole presenting as an adnexal tumor in a postmenopaused patient**

S. Stolnicu ^*^, A. Ilyes, E. Quiñonez, F. F. Nogales

^*^University of Medicine, Dept. of Pathology, Targu Mures, Romania

**Objective:** Hydatidiform mole (HM) is rare in postmenopause, with 7 cases reported. The occurrence of ectopic (HM) is also rare, with 26 fully documented tubal cases. We are not aware of any reported cases of ectopic HM in a postmenopausal patient.

**Method:** A 51-year-old patient with 3 years amenorrhea was admitted for pelvic pain and nausea. Ultrasound examination revealed a 7 cm right adnexal mass. Surgery revealed a necrotic, hemorrhagic mass involving the peritubal space. Total hysterectomy with bilateral salpingo-oophorectomy, omentectomy and appendectomy was performed.

**Results:** Macroscopically, the irregular hemorrhagic right adnexal mass surrounded the fallopian tube and the right ovary showed a corpus luteum of pregnancy. Microscopically, chorionic villi were seen within the haemorrhagic mass accompanied by circumferential trophoblast hyperplasia. The basophilic mesenchyme of the villi exhibited cisterns and trophoblastic inclusions. Immunohistochemically, p57kip2 positive nuclei were prominent in the extravillous (intermediate) trophoblast, but absent in the cytotrophoblast. The HER2 FISH expression was diploid. A diagnosis of an early complete HM was made.

**Conclusion:** The occurrence of HM in the menopause may originate from incomplete, transitional loss of ovarian function with sporadic ovulatory cycles, hypothesis that is supported in this case, due to the presence of a well-formed gestational corpus luteum, confirming a recent ovulation.

**PS-22-003**

**Types of Vulvar Squamous Cell Carcinoma (VSCC): Pathological and clinical characteristics**

S. Fernandez ^*^, A. Fernandez de Larrinoa, L. Ortega, J. A. Nieto, O. Rodriguez

^*^H.U. Basurto, Dept. of Pathology, Bilbao, Spain

**Objective:** Two different aetiopathogenic patways for the development of VSCCs. One associated-HPV and other independent-HPV

**Method:** A total of 21 cases of VSCCs. Morphological features analyzed: size, VSCC-type, VIN-type, inflammatory dermatological conditions, margins, and TNM. The antibodies were used p16 and p53.

**Results:** The study included 21 patients. Aged 33–87 years. Tumor size 0.5–9 cm. VSCCs types were: basaloid (9.5 %), warty (14.3 %), keratinizing (71.4 %) and only one verrucous. All basaloid and warty types were usual VIN associated. All but one of keratinizing types, arise of differenciated VIN and most were associated with chronic dermatological diseases. VIN affected margins in 2 cases. All but one were considered pT1b. Nodal metastasis were 14.3 %. Basaloid and warty: were diffuse p16 positive, and showed weak-moderate and focal positivity for p53 (66.7 %). Keratinizing: were completely negative for p16 and intense and diffuse p53 positive, only one case showed moderate-diffuse positivity for p16 and p53 negativity. The verrucous type was p16 and p53 negative.

**Conclusion:** The majority of VSCCs keratinizing type were p53 positive and p16 negative, and that p16 and p53 tended to be mutually exclusive. But VSCCs warty and basaloid types exists in more the half of the cases co-expression of two markers.

**PS-22-005**

**PLA identifies glycopeptides (T/ST) carried by MUC16 and MUC1 as putative biomarkers discriminating different subgroups of serous ovarian tumours**

L. David ^*^, S. Ricardo, L. Silva, R. Pinto, R. Almeida

^*^IPATIMUP, Porto, Portugal

**Objective:** To evaluate expression of T and ST antigens and T/ST linked to MUC1 or MUC16 as biomarkers of malignancy in serous ovarian tumours.

**Method:** Immunohistochemistry and in situ proximity ligation assay (PLA).

**Results:** MUC16/MUC1 were expressed in 100 % of malignant/borderline lesions and less frequently in cystadenomas. T-antigen was more frequent in adenocarcinomas (44 %) than in borderline and benign lesions (13 % and 14 %) contrasting with ST that was present in 100 % of all sub-groups. Despite that ST is expressed in 100 % of cases from all sub-types, PLA pairs ST/MUC16 and ST/MUC1 have a decreasing gradient from malignant (87 % and 74 %), to borderline (76 % and 56 %) to benign lesions (72 % and 35 %). Expression of PLA pairs carrying T antigen (MUC16 and MUC1) was more restricted and PLA defining T/MUC16 separated malignant and borderline (30 % and 32 %) from benign tumours (3 %), whereas PLA defining T/MUC1 separated malignant (35 %) from borderline and benign tumours (8 % and 7 %).

**Conclusion:** Our results show that PLA for T/ST and MUC16/MUC1 identifies true interactions carbohydrate-mucin. Despite that each marker per se was not informative for discriminating ovarian tumours, all glycopeptides identified by PLA have a putative role in discriminating different subgroups of serous ovarian tumours.

**PS-22-006**

**Metastatic non gestational choriocarcinoma in a postmenopausal woman. A case presentation and a review of the literature**

A. Abuomar ^*^, R. Duran, A. Sonia

^*^Elda University Hospital, Dept. of Pathology, Alicante, Spain

**Objective:** Choriocarcinoma is a highly malignant epithelial tumor originating in trophoblast. it primarily occurs during the fertile period and is extremely rare after menopause. Choriocarcinoma is a biphasic proliferation of trophoblast and syncytiotrophoblast without chorionic villi

**Method:** A 63-year-old woman presented with severe lower limbs edema and ocasional vaginal bleeding. Abdominal CT scan showed a large uterine tumour sugestive to leiomioma, enlarged abdominal, retroperitoneal and mediastinal adenopathy, and bilateral pulmonary noduls. Abdominal lymphadenectomy was performed for diagnostic purpose

**Results:** We recieved a lymph node of 2 × 2 × 1 cm with a necrotic/hemorrhagic aspect on gross section. microscopically it showed necrosis, hemorrahgia and infiltration with a highly pleomorphic malignant cells with clear cytoplasma, atypical nuclei with uneven chromatin and numerous nucleoli. Multinucleated cells showing enlarged, bizarre nuclei, granular, chromatin, numerous nucleoli and with abundant atypical mitoses were observed. Human beta HCG immunopositivity demonstrated the presence of syncytiotrophoblast. Malignant cells were also positive for CKAE1-3, CK7 and EMA. AFP, Vimentina, R Estrogen, CK20, S100, P63 and CA125 were negative.

**Conclusion:** Choriocarcinoma is a highly malignant tumor. Though Nongestational choriocarcinoma has worse prognosis than gestational choriocarcinoma, Early histopathological diagnosis and laboratory and immunohistochemical confirmation of choriocarcinoma in postmenopausal women is very important, as an effective chemotherapy regimens exist.

**Choriocarcinoma:** 
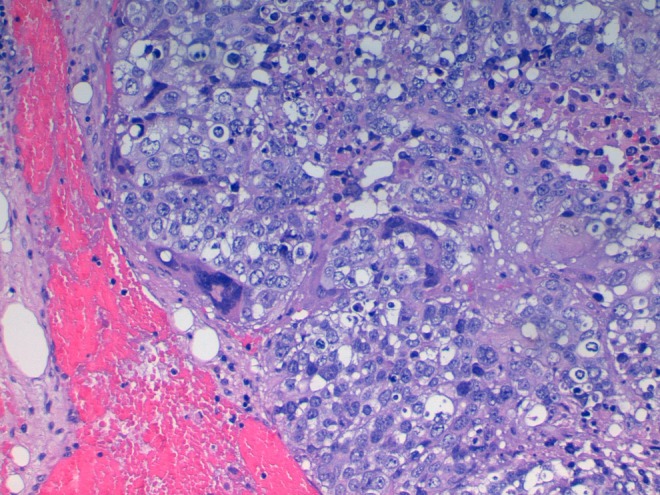


**PS-22-007**

**Prognostic factors in endometrial carcinoma**

D. Radulescu ^*^

^*^Apollonia University, Dept. of Morphopathology, Iasi, Romania

**Objective:** The aim of this study was to present the prognostic factors for endometrial carcinoma. Microscopic type and grade, presence of tumor receptors in the tumor cells, infiltration of uterine serosa, cervical invasion, presence of lymph node and intraperitoneal metastases have been considered as prognostic factors. Ki67, mean nuclear volume, and PTEN protein also provided prognostic information.

**Method:** We evaluated histologically 26 specimens.

**Results:** The histologic parameters, Ki67, mean nuclear volume, PTEN protein, and mitotic index were significantly correlated to survival and recurrence.

**PS-22-009**

**A case of giant uterine lipoleiomyoma**

F. Yilmaz^*^, A. Kilitci, B. Duran

^*^Abant Izzet Baysal University, Medicine Faculty, Bolu, Turkey

**Objective:** Uterine lipoleiomyoma is composed of various mixtures of smooth muscle and mature fat tissue, and it is a rare benign tumor. They may arise in any part of the uterus including the cervix. Its occurence in the uterine corpus in a postmenopausal woman reported.

**Method:** Case report: A 50-year-old woman presented with lower abdominal pain. Pelvic and radiographic examinations revealed an enlarged uterus, suggesting leiomyoma. To confirm the pathologic diagnosis, the patient underwent total abdominal hysterectomy and bilateral salphingo-oophorectomy. Macroscopically, the uterus, measuring 9 × 6 × 4 cm and weighing 496 g, revealed a subserosal mass measuring 12 × 9 × 8 cm. The cut surface of the mass had a lipoma-like appearance. It was a well-demarcated, lobulated, yellowish soft mass with scattered elastic hard whitish areas. There was no continuation between the mass and the pelvic wall.

**Results:** Histologic examination revealed that the tumor was composed of mature lipocytes and smooth muscle were present, and this confirmed the diagnosis of lipoleiomyoma.

**Conclusion:** In cases where the radiological appearance and physical examination of a large uterine tumor in a patient is a suspicious for leiomyoma/leiomyosarcoma/liposarcoma, the possibility of a benign lipoleiomyoma should be considered. Careful consideration of the patient’s preoperative condition and familiarity with uterine fat-containing tumors are essential for determining an optimal therapeutic approach.

**PS-22-010**

**Malignant mixed müllerian tumor (teratoide carcinosarcoma) of the ovary with primitive neuroectodermal differentiation**

S. Bachurska-Yovcheva ^*^, D. Staykov, D. Tashova, N. Koev, V. Belovezhdov

^*^Medical University Plovdiv, General and Clinical Pathology, Bulgaria

**Objective:** Malignant mixed Mullerian tumor (teratoide carcinosarcoma) of the ovary is a rare neoplasm, consisting of malignant epithelial and mesenchimal components. There are only few reported cases of this tumor with primitive neuroectodermal differentiation, resembling nasopharingeal carcinosarcomas.

**Method:** We report a case of Malignant mixed Mullerian tumor (teratoide carcinosarcoma) of the ovary in 80-year-old female.

**Results:** Grossly the tumor was presented as a solid, brownish mass, measuring 8 sm in diameter. Microscopical examination showed a mixted structure of the tumor, consisting of squamous carcinoma, adenocarcinoma (positive for cytokeratin 7); leomyosarcoma (positive for actine and myosine), chondrosarcoma with osseous methaplasia and areas with neuroendocrine (synaptophysin positive) and glial (GFAP positive) differentiation. All the components were extremely immature without presence of organoide structures.

**Conclusion:** We report the sixth case of the teratoide carcinosarcoma with neuroectodermal differentiation of the ovary, filling with the additional clinical and morphological data about this rare and agressive neoplasm.

**PS-22-011**

**Acquired vulvar lymphangioma circumscriptum: Series of cases in three hospitals and literature review**

M. San Martín Alonso ^*^, E. Carballo Núñez, J. M. Suárez Peñaranda, J. A. Ortiz Rey

^*^Hospital do Meixoeiro, Dept. of Pathology, Vigo, Spain

**Objective:** We present a series of cases of acquired vulvar lymphangioma circumscriptum (ALVC) from three hospitals, with emphasis on predisposition factors and on the need of a good knowledge of this entity and its clinical differential diagnoses.

**Method:** Six ALVC cases were recorded and clinical and histopahologically studied, including immunohistochemical determinations and therapeutical follow-up, in order to know eventual relations between characteristics of the diagnosed cases, their previous clinical record (three of them had undergone surgical or radiotherapeutical precedures and the other three arose from benign conditions) and their evolution (with or without treatment, depending on the case), integrating our findings into the information about this entity provided by the literature.

**Results:** All of the biopsies showed consistent histological findings: dilated lymphatic vessels in papillary dermis. The endothelium showed flat nuclei, without atypia or proliferative changes. The content was amorphous and eosinophilic, mainly without red blood cells. Dermis showed no inflammation. No findings of human papillomavirus or dysplasia were found. Immunohistochemically lymph vessels endothelium were podoplanin+ and CD31+. CD34 positivity was weak and discontinuous. P16 was negative.

**Conclusion:** Histopathologic study of this entity cannot stablish a histogenetic subtype, but is very important for the patient’s medical record and her later management, being necessary a good vulvar biopsy.

**Hematoxilin and eosin ALVC:** 
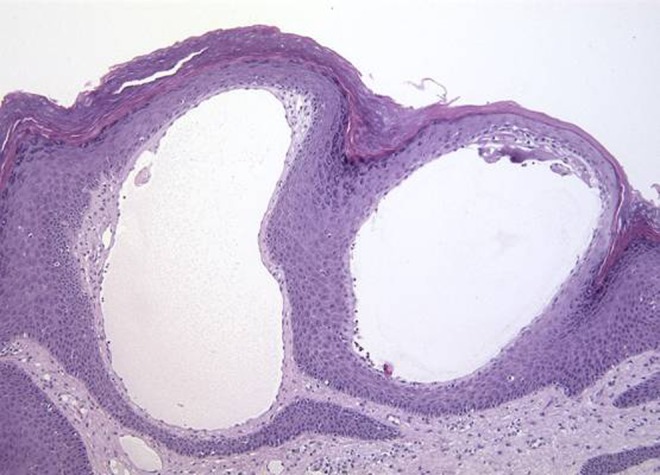


**PS-22-012**

**Primary melanoma of the uterine cervix: A case report**

A. Dolzhikov ^*^, T. Mukhina, V. Dmitriev

^*^Regional Pathology Bureau, Dept. of Oncopathology, Belgorod, Russia

**Objective:** Primary melanoma of the uterine cervix is a rare neoplasm. We report the case of a 57 -year-old woman with amelanotic melanoma of the cervix not diagnosed before radical hysterectomy.

**Method:** A 57-year-old postmenopausal woman presented with vaginal bleeding of 1 month duration. Examination of the genital tract showed a non-pigmented tumor of the anterior lip of the cervix measuring 7 × 6 cm. The clinical diagnosis was suspicious for cancer of the cervix. The patient received radical hysterectomy.

**Results:** Histological examination revealed diffuse infiltration of large polymorphic cells in the cervical stroma. The cervical epithelium did not show epitheliotropism by malignant cells. The tumor cells had large polymorphic vesicular nuclei with prominent nucleoli, eosinophilic cytoplasm without any pigment granules. The tumor was initially considered as a poorly differentiated malignant tumor. Histogenetic immunohistochemical analysis (CD45, cytokeratin AE1/AE3, S-100, vimentin) revealed that the tumor cells were strongly positive for vimentin and S-100. The strong immunoreactivity for melanoma-cocktail (HMB-45+thyrosinase+melanA) and HMB-45 was also detected. Immunohistochemical findings supported the diagnosis of epithelioid amelanotic melanoma.

**Conclusion:** Cervical melanoma is extremely rare neoplasm and less than 50 cases have been published in the literature. Amelanotic variants may be easily misdiagnosed as undifferentiated carcinomas or lymphoid tumors. Immunohistochemistry is essential since cervical melanoma is a tumor with poor prognosis.
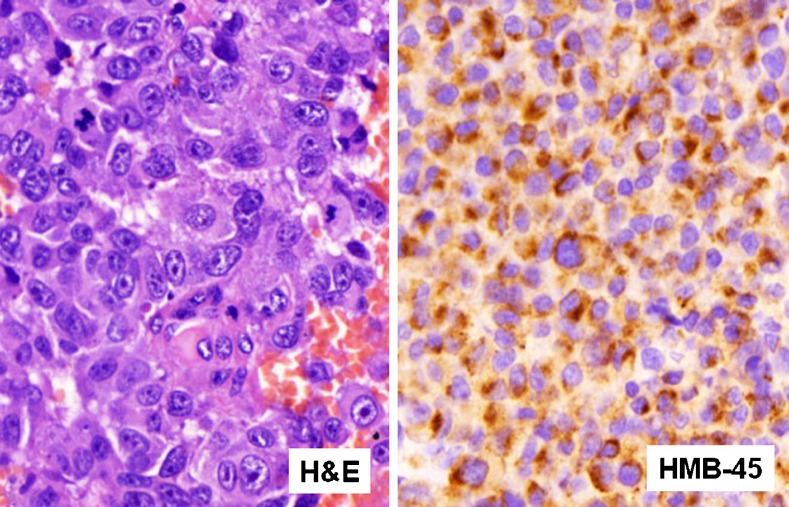


**PS-22-016**

**A rare case of yolk sac tumor of the vagina**

G. Tasova Yilmaz ^*^, G. Erdogan, C. Uzun, F. Çelik, S. Karaveli

^*^Akdeniz University, School of Medicine, Dept. of Pathology, Antalya, Turkey

**Objective:** Malignant germ cell tumors are rare tumors of childhood accounting less than %3 of pediatric malignancies, and yolk sac tumor(YST) is the most common histological subtype. The vagina is an extremely rare site for germ cell tumors.

**Method:** A 12-month old girl presented vaginal bleeding, starting 2 months prior to diagnosis. An ultrasound test revealed uterus was larger. Magnetic resonance imaging showed 3 × 1.5 cm vaginal mass. The serum alpha-fetoprotein(AFP) was elevated.

**Results:** An incisional biopsy of the tumor mass showed a loose meshwork of microcystic spaces, anastomosing glands and pseudopapillary structures with a fibrovascular core lined by columnar cells projecting into the cystic spaces. Few hyaline globules were seen. Immunohistochemical analysis revealed that the tumor cells were positive for AFP, negative for CK7 and EMA. Based on these findings, the diagnosis of YST of the vagina was concluded. The patient was given six cycles of chemotherapy and continues to be disease-free on follow up

**Conclusion:** YST is locally aggressive and capable of metastasis Therefore, in pediatric patients with a history of vaginal bleeding YST must be considered. So, we encourage consideration of chemotherapy as a sole modality to treat this rare tumor.

**PS-22-017**

**Coexistent atypical polypoid adenomyomas of the uterus and adenocarcinoma**

M. Sotiropoulou ^*^, P. Markoulis, N. Thomakos, I. Koutroumpa, A. Rodolakis, D. Zacharakis, D. Haidopoulos, G. Vlachos, A. Antsaklis

^*^Alexandra Hospital, Dept. of Pathology, Athens, Greece

**Objective:** Atypical polypoid adenomyoma (APA) of the uterus is uncommon and usually occurs in women of reproductive age who have abnormal vaginal bleeding. It arises most frequently in the lower uterine segment or endocervix and presents as pendunculated or sessile polypoid mass. The differential diagnosis with endometrioid adenocarcinoma, which is of great clinical importance, can be difficult in small endometrial biopsy or curettage.

**Method:** We present the clinicopathological features of 11 APAs diagnosed in endometrial biopsy or curettage. The tumors occurred in women aged 25–68 years (premenopausal 9, postmenopausal 2).

**Results:** Histologically they were composed of marked complexity branching glands with prominent morules separated by intersecting fascicles of fibromuscular stroma. Epithelial atypia was mild to moderate and mitotic figures were rare. Two cases coexisted with endometrioid adenocarcinoma grade I with minimal myometrial invasion in hysterectomy specimen. The mild or moderate atypia, the more cellular active smooth muscle component, the lack of desmoplastic reaction and the absence of CD10 expression are the major diagnostic features in the differential diagnosis. All patients are alive and well 3 to 117 months after diagnosis (mean 61 months).

**Conclusion:** The differential diagnosis between APA and endometrial adenocarcinoma, based chiefly on morphological features, is of great clinical importance in biopsy material due to reproductive conservation in young women.

**PS-22-018**

**Mucinous carcinoma of the ovary: Significance of prognostic factors in clinical outcome**

M. Sotiropoulou ^*^, P. Markoulis, N. Thomakos, A. Rodolakis, I. Koutroumpa, D. Zacharakis, D. Haidopoulos, G. Vlachos, A. Antsaklis

^*^Alexandra Hospital, Dept. of Pathology, Athens, Greece

**Objective:** Τhe aim of the study was to correlate pathological and surgical factors in patients with primary mucinous carcinoma of the ovary with clinical outcome and overall survival.

**Method:** We retrospectively examined all surgically staged cases of mucinous ovarian carcinomas from 1998 to 2008. All available histologic slides were reviewed and clinical information was obtained from hospital files and through telephone communication.

**Results:** Forty-two cases were retrieved and subclassified into 19 expansile and 23 infiltrative subtypes. Thirty-four (80 %) tumors were stage I, three II and five III. Twenty one patients (77,7 %) were alive and free of tumor at a mean FU interval of 6,2 years, while the rest died of the disease. 94 % of patients with expansile type of invasion were stage I and none had a recurrence. On the contrary, two of 16 patients with stage I infiltrative pattern carcinomas had a fatal recurrence. Four of six patients (66,5 %) who died had carcinomas with nuclear grade 3. Neither the mitotic count nor the necrosis had any influence on survival.

**Conclusion:** Clinical outcome of mucinous ovarian carcinomas depends mainly on their stage, which is largely related to the histologic features. Carcinomas with expansile growth pattern and low nuclear grade usually present as stage I tumors and have a good prognosis.

**PS-22-019**

**Granulosa cell tumors of the ovary: A clinicopathologic and immunohistochemical study of 21 cases**

E. Kairi-Vasilatou ^*^, C. Dastamani, P. Mavrigiannaki, E. Carvounis, A. Kondi-Pafiti

^*^Aretaieion University Hospital, Dept. of Pathology, Athens, Greece

**Objective:** To study the clinicopathologic and immunohistochemical features of ovarian granulosa tumors (GCTs).

**Method:** A retrospective study of all cases of GCTs diagnosed in our laboratory over a 10-year period was performed and the archival blocks were stained for inhibin, vimentin, cytokeratin, Ki-67 and p53.

**Results:** Twenty-one cases (15 of the adult and 6 of the juvenile type) were retrieved. All patients were FIGO Stage I at the time of diagnosis. Recurrent disease was detected in four patients (19 %) during a median follow-up of 36 months (range 2–26 years). Pathology revealed a concomitant theca-cell component in three cases, a Sertoli-Leydig component in one case, and a thecoma in one case. Archival tissue material was available in 12 cases. Immunohistochemistry was positive for: beta-inhibin in 12/12 cases (100 %), vimentin in 11/12 cases (91.7 %), cytokeratin in 3/12 cases (25 %), CD34 in 0 cases (0 %), and p53 in 2/12 cases (16.7 %). The Ki-67 index was <5 % in 12/12 cases (100 %). No significant correlations were observed between the pathologic and immunohistochemical parameters examined and the clinical outcome.

**Conclusion:** Despite the relatively indolent nature and favorable prognosis of most GCTs, late recurrences are not a rare event even in Stage I patients, necessitating a close and long-term follow-up.

**PS-22-020**

**Immunopathological features of 30 cases of Brenner ovarian tumors**

E. Kairi-Vasilatou ^*^, C. Dastamani, A. Paraskeva, A. Melloy, E. Carvounis, A. Kondi-Pafiti

^*^Aretaieion University Hospital, Dept. of Pathology, Athens, Greece

**Objective:** To investigate the immunopathological features of Brenner ovarian tumors.

**Method:** Thirty cases of Brenner ovarian tumors were examined in our laboratory among 1,680 cases of ovarian tumors, representing 1.5 % of all tumors examined. Blocks of paraffin-embedded tumor tissue were stained for Uroplakin-III, Chromogranin, WT1, NSE, CK20 and CK7.

**Results:** The mean age of the patients was 51.4 years, ranging from 16 to 82 years. Seventeen cases (56 %) were pure Brenner tumors and 13 cases (44 %) were mixed tumors consisting of a Brenner tumor element and a mucinous ovarian tumor (10/13 cases) and a germ cell tumor (3/13 cases). The immunoprofile of Brenner tumor cell was CK-7 positive (30/30 cases), CK-20 negative in the Brenner cell element, but positive in the mucinous component of mixed Brenner tumors, focally WT-1 positive (5/30 cases), NSE negative (0/30 cases), focally chromogranin positive (6/30 cases) and Uroplakin-III positive in 23/30 cases, with faint cytoplasmatic or luminal distribution.

**Conclusion:** Brenner ovarian tumors express some urothelial differentiation, showing positivity to Uroplakin-III, but in contrast to the urothelial epithelium are not positive to both CK-7 and CK-20.

**PS-22-021**

**Ovarian carcinomas associated with endometriosis: A clinicopathologic study of 18 cases**

E. Kairi-Vasilatou ^*^, C. Dastamani, S. Stasinopoulou, A. Kondi-Pafiti

^*^Aretaieion University Hospital, Dept. of Pathology, Athens, Greece

**Objective:** To present the clinicopathological features of ovarian carcinomas associated with endometriosis.

**Method:** A retrospective study of 18 cases of ovarian carcinomas associated with endometriosis, diagnosed over a 10-year period in our laboratory is presented.

**Results:** Patients’ age was 27–76 years. The tumor size ranged from 2 to 24 cm in maximum diameter. In 12/18 cases, the tumor presented as a solitary mass of the ovary, in 4/18 cases it affected both ovaries and in 5/18 cases the tumor affected the ovary and the fallopian tube. In one case, the tumor affected both ovaries and endometrium. Ten cases (55.5 %) were classified as clear cell carcinomas (CCC), seven (38.8 %) were endometrioid adenocarcinomas (EAC) and one case (5.5 %) was serous carcinoma associated with endometriosis. Endometriosis (both ectopic endometrial glands and stroma) was documented in 9/18 cases (3 cases of CCC, 5 cases of EAC and one case of serous adenocarcinoma). In the remaining 9 of 18 cases, the presence of fibrotic tissue with pseudoxanthoma cells and a few residual stromal cells [CD10 (+)] were considered as strong evidence of endometriosis–“presumptive endometriosis”.

**Conclusion:** Endometriosis might be viewed as a precursor lesion of endometrioid and clear cell carcinoma of the ovary, via intermediary atypical borderline lesions.

**PS-22-022**

**Cell cycle markers and hormone therapeutic targets in uterine smooth muscle tumors, an immunohistochemical approach**

N. Santonja Lopez ^*^, S. Navarro Fos

^*^Pathology-Manises Hospital, Labco, Valencia, Spain

**Objective:** To analyse the immunohistochemical expression of some cell cycle markers and hormone therapeutic targets in uterine muscle neoplasm.

**Method:** Four tissue microarrays (TMA) were constructed including 29 leiomyomas, 33 special leiomyomas, 16 leiomyosarcomas, and 13 no neoplastic adult uterus myometrium. 4 μm-thick sections were obtained for H/E staining as well as for several immunohistochemical staining: p53 (DAKO), p16 (CINTEC), p21 (DAKO), and estrogen and progesterone receptors (NOVACASTRA).

**Results:** We observed a significant correlation (*p* < 0.005) between the protein expression and the pathological diagnosis. P53 was expressed only in leiomyosarcomas, whereas p16 and p21 were more frequently expressed in leiomyosarcomas, being focal in leiomyomas. There was also a correlation with hormone receptor; both estrogen and progesterone were more frequently expressed in leiomyomas.

**Conclusion:** Immunohistochemical expression of cell cycle proteins is useful for diagnostic and pronostic in leiomyosarcomas, whereas hormone receptors do not seem as useful for treatment like they are in benign counterparts.

**PS-22-023**

**A rare case of vulvar malignant fibrous histiocytoma/pleomorphic sarcoma**

E. Moustou ^*^, K. Manoloudaki, E. Arkoumani, A. Tsavari, K. Koulia, D. Myoteri

^*^General Hospital Tzaneio, Dept. of Pathology, Piraeus, Greece

**Objective:** Primary sarcomas of the vulva comprise a rare group of malignant neoplasms consisting 1,5–3 % of all vulvar malignancies. However, Malignant Fibrous Histocytoma (MFH) is the second most frequent sarcoma of this region.

**Method:** We report a case of a 73 year old woman with a polypoid ulcerated tumor in the labium major, measuring 7,5 × 6 × 4,5 cm.

**Results:** Grossly, the tumor was multilobulated, firm or soft-elastic and gray white or reddish in color. Histologically, showed a high–grade cytologic features of spindle, pleomorphic and multinucleated cells in a storiform, fascicular and diffuse pattern, high atypical mitotic rate, fibromyxoid stroma, necrosis, hemorrhage and an inflammatory inflitrate. Immunoreactivity was positive for vimentin, CD68/kp1,a1–antitrypsin NSE and P16, focal for SMA, CD10 myoglobin, rare for S100p and negative for cytoceratins EMA, CD34 as well as the rest of myoid, neurogenic, vascular, lymphoid, and melanocytic markers. According to the morphology and immunohistochemistry (lack of any lineage specific marker) and after excluded other pleomorphic malignancies, a diagnosis of MFH/pleomorphic sarcoma was decided.

**Conclusion:** MFH arising in the vulva is rare with a few reference in literature. The diagnosis is one of exclusion and presuppose excellent tumor sampling, current microscopic evaluation and appropriate use of immunohistochemistry.

**PS-22-024**

**Clinicopathological and immunohistochemical analysis of 23 cases of ovarian cellular fibroma**

N. Basheska ^*^, B. Ognenoska-Jankovska

^*^UCRO, Faculty of Medicine, Dept. of Histopathology, Skopje, Macedonia

**Objective:** To define clinicopathological characteristics and immunohistochemical markers helpful in differentiating between cellular fibroma (CF) and mitotically active cellular fibroma (MACF).

**Method:** Patient records and archival pathology specimens of 23 ovarian cellular fibromas diagnosed and followed between 2000 and 2012, were reviewed and immunohistochemistry was performed.

**Results:** The mean age of patients with CF (*n* = 15) and MACF (*n* = 8) was 55 and 37 years, respectively. All tumours were unilateral, with a mean tumour size of 8.6 cm for CFs and 12.9 cm for MACFs. In all tumours, most of the cells showed mild or moderate nuclear atypia. The mean highest mitotic count was 2.0 MFs/10 HPFs for CF, and 7.2 MFs/10 HPFs for MACFs. The majority of the tumours were immunoreactive for vimentin, alpha-SMA, WT-1, inhibin-alpha, calretinin, CD56, melan-A, PR, and bcl-2, and negative for pan-cytokeratin, EMA, CD117, ER, and p53. A few tumours were also positive for S100, desmin, CD10, and CD99. In addition, the MIB-1 labeling index (LI) in MACFs was higher (mean 16.9 %, range 12–25 %), than that in CFs (mean 5.9 %, range 3–9 %).

**Conclusion:** Our results confirm the clinicopathological differences and the immunophenotypic similarity between ovarian fibromas and cellular fibromas, and suggest that the use of MIB-1 LI may help in differentiating between CF and MAFC.

**PS-22-025**

**HR HPV in situ hibridization, p 16 INK 4A and survivin expressions in uterine cervix carcinomas and the evaluation of these expressions with prognostic factors**

E. Kimiloglu ^*^, F. Demir, Y. T. Ayanoglu, N. Erdogan

^*^Taksim’s Hospital, Dept. of Pathology, Istanbul, Turkey

**Objective:** Cervix Carcinoma(CC) is one of the most important health problems of women in developping countries. CC is the second most common carcinoma of women in the world. Because of the prooved effect of the load of the disease, efficient screening and early therapy programmes, the disease has been one of the vital area of action.

**Method:** We studied 44 cases of cervical intraepithelial neoplasms(CIN), 9 cases of squamous carcinomas(SCC). Hr HPV DNA ISH, p16 INK 4A and survivin ımmunohistochemical expressions were analysed in these 53 cases.

**Results:** We determined that the presence, density and the nuclear detection form of Hr HPV DNA had a diagnostic and prognostic importance in CIN and CCs(*p* < 0.05). Positive staining of p16 and survivin signalled progressive oncogenic events, therefore p16 and survivin were persistent HPV infection markers (*p* < 0.001 for p16) and (*p* < 0.01 for survivin). The episomal pattern which is nonassociated guest of HrHPV DNA with the host cell DNA signalled early HPV infection (*p* < 0.001). When it integrated the host cell DNA, especially if the density and widespread of HPV DNA increase, this signals persistent HPV infection(*p* < 0.001).

**Conclusion:** With the light of these findings we determined that in CIN I lesions, HPV is infectious, in CIN II-CIN III lesions, HPV is neoplastic.

**PS-22-026**

**Morphological characteristics of ovaries in case of different kinds of surgical treatment**

O. Reshetnikova ^*^, V. Simrok, O. Teleshova, D. Simrok-Starcheva

^*^State Medical University, Lugansk, Ukraine

**Objective:** Polycystic ovarian syndromes (PCOS) are found in 5–10 % of women of reproductive age. In 60–75 % the ovarian pathology results in endocrine infertility. Surgical treatment of PCOS is one of the most important methods of treatment. Therefore, there is a need in study of less traumatic ways of surgical technique in gynecology.

**Method:** Twenty of ovaries were studied morphologically. Ten samples experienced the exposure to holmium laser (main group) and other ten-thermo cauterization (group of comparison). Tissue samples were immersion fixed in 10 % buffered formalin solution, embedded in paraffin wax. Histological slides were studied microscopically; computer morphometry was conducted. The data were compared with group of comparison by Student *t*-test.

**Results:** It has been found that under the action of a bipolar electrode during cauterization of the ovarian tissue expressed pathological changes occur. Microscopy revealed a broad band of coagulation necrosis with areas of hemorrhage. The severity of pathological changes under the holmium laser influence was far less significant, the necrotic zone was two times narrower than under the action of a bipolar electrode.

**Conclusion:** The research results are considered in the aspect of the structural basis for optimistic prognosis for the course of regeneration and recovery of the ovaries reproductive function.

**PS-22-027**

**Aberrant promoter methylation of p16 gene and HPV16/18 status in adenocarcinoma of the uterine cervix and precursor lesions**

N. Danilova ^*^, T. Kekeeva, L. Zavalishina, Y. Andreeva, G. Frank, P. Malkov

^*^Moscow Oncology Institute, Dept. of Pathology, Russia

**Objective:** This study was conducted to investigate the aberrant promoter methylation status of the p16 gene in cervical glandular lesions and to determine the correlation with HPV 16/18 infection status. We studied p16 methylation in primary cervical adenocarcinoma, cervical glandular intraepithelial neoplasia (CGIN) and in surrounding stroma in order to estimate it’s role in cervical carcinogenesis.

**Method:** Aberrant promoter methylation was evaluated using a methylation-sensitive PCR in 11 cervical cancer tissue specimens accompanying with CGIN. CGIN, tumor and stromal components were investigated separately using laser capture microdissection. HPV 16/18 was detected by CISH using CenPoint HPV DNA Probe Cocktail, Biotinylated (Dako, Denmark).

**Results:** The frequencies of aberrant promoter methylation of p16 in tumor, CGIN and in stromal compartment were 2 %, 3 %, 95 %, respectively. HPV 16/18 was detected in 9/11 cases (81 %) in adenocarcinoma and in 9/11 (81 %) of CGIN.

**Conclusion:** We detected low level of the promoter methylation and high level of HPV16/18 in adenocarcinoma and CGIN. In contrast the high level of the p16 methylation and absence of HPV 16/18 were observed in stromal component.

**PS-22-028**

**ACA protein expression and localization in smooth muscle uterin tumors**

E. Kogan ^*^, Z. Becker-Kojic, T. Demura, N. Fayzullina, N. Nizyaeva, S. Askolskaya, U. Popov, G. Sukhikh

^*^RCOGP, Dept. of Pathology, Moscow, Russia

**Objective:** АСА protein is GPI-anchor protein playing role in regulation stem cells activity in different processes including embryogenesis, inflammation, tumor genesis. It may launch signal molecular way PI3K-AKT-mTOR. ACA role in smooth muscle uterus tumors (SMUT) development is unknown. The aim of study was to investigate ACA expression and localization in (SMUT).

**Method:** Immunohistochemical analysis was performed on 31 cases of leiomyomas (LM) and 3 leiomyosarcomas (LS) from patients of the mean age 34yy LM and 51yy LSs with antibody against ACA, SMA, desmin, ER, PR, CD117, Ki-67. LMs were simple (16), cellular LM (10), mitotically active (5).

**Results:** Results of the study show АСА dysregulation in LMs and LSs, which is characterized by increasing of ACA expression in cytoplasm of tumors cells (especially in malignant tumors), and losing of normal membrane staining. All tumors cells were highly positive for desmin and SMA. ACA, CD117 and Ki-67 positive tumors cells were revealed in perivascular zones in LMs.

**Conclusion:** Particularities of ACA expression and localization prove its significance in growth and progression of LMs and LSs.

**PS-22-030**

**Intraepithelial pathology of fallopian tube and serous ovarian tumors**

A. Asaturova ^*^, L. Ezhova, E. Kogan, N. Faizullina

^*^NCAG, Dept. of Pathology, Moscow, Russia

**Objective:** Recently scientists pay great attention to the role of intraepithelial pathology of fallopian tube (FT) in serous ovarian tumors pathogenesis.

**Method:** 35 patients (47 ovaries and 70 FT with intraepithelial pathology of FT) were observed histologically, immunehistochemically and statistically.

**Results:** We revealed 16 serous ovarian tumors (4 serous benign tumors, 8 borderline serous tumors (BST), 4 serous carcinomas (SC)), 11 FT with papillary tubal hyperplasia, 20 FT with epithelial proliferation and 5 FT with atypical epithelial changes (accompanied only with BST and SC). High p53 expression was in 25 % of cases (only in patients with serous ovarian tumors), high Ki-67 expression–in 50 %. Based on the algorithm for intraepithelial pathology of FT diagnosis (R. Kurman, 2013) we diagnosed normal/reactive changes in 50 % of FT, papillary tubal hyperplasia in 8 %, serous tubal intraepithelial lesion (STIL) in 8 %, serous tubal intraepithelial carcinoma (STIC) in 17 %, while STIL and STIC were associated only with SBT and SC.

**Conclusion:** Intraepithelial pathology of FT was associated with serous ovarian tumors in 50 %. Papillary tubal hyperplasia is more frequently associated with benign serous tumors, STIC and STIL were found only in patients with BST and SC. Thus, we suppose that there is a pathogenetic relationship between STIL/STIC and BST and SC development.

**PS-22-031**

**Nonneoplastic endometrial signet-ring cells: A diagnostic challenge for morphology**

L. Alfaro ^*^, S. Tena, E. Gonzalez

^*^Valencia, Spain

**Objective:** Signet-ring cells are associated with neoplastic lesions and with a ominous prognosis especially when diagnosed as metastasis. Gastric adenocarcinomas are the most common source of this cells but also colon, appendix, breast can harbor this characteristic cell type. Rarely signet-ring cells appear as metastatic adenocarcinoma infiltrating endometrium. A few cases of reactive benign cells with signet-ring cell morphology have been described in endometrium.

**Method:** A endometrial curettage was received in our hospital from a 35-years-old woman. She had underwent several spontaneous abortions, and a endometrial biopsy was indicated previously to be included in a in vitro fertilization program. Sample was taken in a 26 day, having the patient regular 28-days-long cycles.

**Results:** Biopsy showed hypersecretory glands and marked pseudodecidual stromal changes with slight granulocytic infiltration. Numerous cells with typical signet-ring cell morphology were seen. Mitosis were easily recognized among stroma. Immunohistochemistry was performed and signet-ring cells were negative for cytokeratins and EMA.

**Conclusion:** Nonneoplastic signet ring-cells in endometrium can be morphologically indistinguishable from malignant cells. The described cases also with decidual change was seen in postmenopausal woman with no mitosis in endometrial cells a worrisome feature for late secretory phase. Immunohistochemistry should be mandatory in cases as ours to rule out malignancy.

**PS-22-032**

**Distinction of primary and metastatic mucinous tumors involving the ovary: Morphological and immunohistochemical features**

L. d. Angelo Andrade ^*^, P. B. Cardoso Pinto, S. F. Derchain

^*^University of Campinas, Dept. of Pathology, Brazil

**Objective:** Primary ovarian mucinous carcinomas are uncommon and the most important differential diagnosis is metastatic adenocarcinomas, mainly from gastrointestinal origin. Besides immunohistochemical profile, an algorithm determines, with high accuracy, that unilateral and >13 cm tumors are primary and all others, metastatic. Objective: to describe clinical and histopathological aspects of mucinous carcinomas, assessing algorithm accuracy and immunohistochemical markers contributory to diagnosis.

**Method:** 76 mucinous carcinomas from our files (1993–2009) were revised; immunohistochemical reactions for CK7, CK20, Ca125, hormonal receptors (ER, PR), SMAD4, beta-catenin, CDX2 were performed by TMA.

**Results:** 35 were primary ovarian tumors (group 1) and 41 metastases (group 2), most from colorectal cancer (54 %). Mean survival differed between the groups (129 × 33 months; *p* < 0.0001). In group 1, 82 % were unilateral >13 cm; in group 2, 58 % were bilateral and 14 % unilateral tumors <13 cm. Markers in groups 1 and 2 were: CK7: 97 %X46 %; CK20: 62 %X61 %; SMAD4: 54 %X56 %; Ca125: 40 %X22 %; CDX2: 26 %X63 %; beta-catenin: 0 %X10 %; ER: 11 %X7 %; PR: 20 %X5 %. Different from group 1, common features in group 2 were infiltrative pattern and vascular invasion. Agreement with the algorithm was 78 %.

**Conclusion:** Algorithm and immunohistochemistry are useful, but there is no gold-standard marker. Only multidisciplinary evaluation can achieve reliable anatomo-clinical diagnosis in this challenging situation.

**PS-22-033**

**Microcystic stromal tumor of the ovary with mutation in exon 3 of ß-catenin: A case report**

S.-Y. Kwon ^*^, H.-R. Jung, I.-S. Hwang, M.-S. Choe, Y.-N. Kang, C.-H. Cho, K.-Y. Kwon, S.-S. Lee, S.-P. Kim, S.-J. Shin, S.-H. Kwon

^*^Keimyung University, Dept. of Pathology, Daegu, Republic of Korea

**Objective:** Microcystic stromal tumor of the ovary is a very rare ovarian tumor with distinctive microcystic histologic features and characteristic immunophenotype of stromal tumor. However, its origin, tumor pathogenesis or prognosis has not been well established till now.

**Method:** We report a unusual case of microcystic stromal tumor of the ovary with mutation in exon 3 of the β-catenin (CTNNB1) gene.

**Results:** Macroscopically, the fragmented ovarian tumor showed diffuse solid mass. Microscopically, tumor had unique pattern of solid and cellular area with microcysts and fibrous hyalinized strom. Immunohistochemical staining of CD10, vimentin, CD99, and β-catenin was positive expression. However, α-inhibin and e-cadherin showed negativity. Mutational analysis revealed a point mutation in exon 3 of β-catenin (CTNNB1) gene

**Conclusion:** In the current study, a case of MCST of the ovary was demonstrated the mutation of β-catenin gene in company with distinctive histological and immunohistochemical finding.

**PS-22-035**

**Müllerian adenosarcoma of the cervix with sarcomatous overgrowth and heterologous elements with ovarian metastasis: A case report and the review of the literature**

M. Koyuncuoglu ^*^, B. Saatli, Y. Sahin, N. Yildirim

^*^Dokuz Eylul University, Dept. of Pathology, Izmir, Turkey

**Objective:** Mullerian adenosarcoma of the cervix is relatively a rare type of mixed epithelial and mesenchymal tumors of the uterus. It is usually composed of a benign epithelial component and a low grade stromal-sarcomatous component. The epithelial component may be atypical and sarcomatous overgrowth (SO) and heterologous elements may be seen infrequenly in stromal component. The SO and myometrial invasion are the most relevant prognostic factors. Treatment strategy is stil unclear.

**Method:** Case: A 32 year-old woman has had abnormal vaginal bleeding history for a couple of years. The pelvic examination revealed a 3–4 cm polypoid mass protruding from the cervix to the vagina. MRI showed a lesion starting from the endocervical canal lying to the vagina. In the operation, there were metastatic implants over the bilateral ovary. Total abdominal hysterectomy and bilateral salpingoopherectomy was performed

**Results:** Histopathological examination revealed, the lesion was mullerian adenosarcoma with sarcomatous overgrowth and cartilaginous foci as heterologous elements. There was no endometrial involvement. The tumor were invaded the ovaries. The gynecologic oncology board decided to give her chemotherapy.

**Conclusion:** In young women with cervical polyp, mullerian adenosarcoma must be considered and should be excluded by careful histopathological exam.

**PS-22-036**

**Uterine lipoleiomyoma: A histopathological review of 22 cases**

A. Kurt ^*^, Y. A. Karakaya, D. Ayaz, S. Sayhan, N. Dicle

^*^Bölge Egitim ve Arastirma Hastesi, Dept. of Pathology, Erzurum, Turkey

**Objective:** Uterine neoplasms composed of an admixture of smooth muscle and adipose(SMA) tissue are relatively uncommon and have been designated as lipoleiomyomas.

**Method:** At the Pathology Laboratory of Izmir Aegean Obstetrics and Gynecology Training and Research Hospital, between 2009 and 2012, a series of 22 patients who were diagnosed lipoleiomyom were examined in terms of the clinical and pathological aspects.

**Results:** Patients were between the ages of 26–77 and the mean age was 49. A 26-year-old patient was pregnant and diagnosed angiomyolipom. A 77-year-old case was diagnosed with endometrioid adenocarcinoma. In one case,the tumor was in the endocervical localization. 14 cases were in post-menopausal period, 8 cases were in intermediate age. Immunohistochemical examination of the SMA patients (+), desmin (+), S-100 (−), CD 34 (−) was observed. There was virtually no mitotic activity. There were no lipoblasts, no atypia in adipocytes or smooth muscle cells, and no necrosis.

**Conclusion:** The differential diagnoses of similar uterine tumors with adipose tissue and spindle cells include spindle cell lipoma, angiolipoma, angiomyolipoma, leiomyoma with fatty degeneration, atypical lipoma, and well-differentiated liposarcoma. In these series, all lipoleiomyomas were comprised of mature adipocytes and smooth muscle cells. Our cases of this rare entity are presented in the light of the literature.

**PS-22-037**

**Histopathological changes that mimic cervical superficially invasive squamous cell carcinoma**

H. Gutnik ^*^, M. Strojan Fležar

^*^Faculty of Medicine Ljubljana, Inst. of Pathology, Slovenia

**Objective:** Histopathological diagnostics of cervical superficially invasive squamous cell carcinoma (SISCCA, FIGO stage IA1) can be a difficult task. Criteria for its evaluation are clearly determined, however difficult to implement in many cases. On the other hand many histopathological changes exist that can mimick early invasion and lead into overdiagnosing of SISCCA.

**Method:** 64 of 230 cervical cone biopsies with the original diagnosis of SISCCA were reevaluated as overestimated and were subsequently analysed for the probable reasons of overdiagnosing. The histopathological changes that could mimick SISCCA were categorised into several groups created by our own experience and according to data accessible in the literature.

**Results:** The most frequent single histopathological change to mimic SISCCA was complex cervical intraepithelial lesion grade 3 (CIN 3) with extensive gland crypt involvement followed by inflammatory changes with blurred epithelial-stromal interface and tangentially sectioned CIN 3.

**Conclusion:** Evaluation of SISCCA is difficult because some histopathological changes can mimick it. The data about this problem in the literature are sparse, but reported histopathological mimics are comparable to our findings. In doubtful cases when the criteria of SISCCA are not clearly expressed, the morphological changes should be carefully examined to exclude the possible mimics of early invasion.

**PS-22-038**

**Correlation of hyperspectral hysteroscopy with histopathological features in endometrial pathology**

A. Batistatou ^*^, A. Ntoulia, F. Gkrozou, M. Paschopoulos, V. Malamou-Mitsi

^*^University of Ioannina, Medical School, Dept. of Pathology, Greece

**Objective:** The aim of this study was to correlate the histologic features of various endometrial pathologies with the conventional hysteroscopic and hyperspectral imaging findings.

**Method:** Eleven women underwent conventional and hyperspectral hysteroscopy and excisional biopsies were taken. The pathological diagnoses were: polyps, (hyperplastic, 4; functional, 1; isthmus, 2), simple hyperplasia (1), leiomyoma (1), adenomyoma (1) and endometrial adenocarcinoma (1). In histological sections the immunohistochemical expression of ER, CLA, Ki67, CD31 and CD34 was evaluated in the stroma and correlated with the imaging findings.

**Results:** Pathological diagnoses were correlated with the hyperspectral imaging data presented as pseudocolour maps. Lymphocytic infiltration correlated with pseudocolour maps, but not with the specific histological diagnosis. The density of the small vessels in the stroma varied, being lower in functional and isthmus polyps, medium in the hyperplastic polyps and carcinoma and higher in hyperplasia, correlating with the pseudocolour map. Stromal expression of ER was considerably lower in the carcinoma compared to all other pathologies. Increased expression of Ki67 in the functional polyp stroma was noted.

**Conclusion:** Histopathological examination of the endometrial pathologies is the gold standard. Novel imaging, such as hyperspectral generated pseudomaps can aid in accurate sampling and possibly in identification of lesions with increased vascular bed.

**PS-22-039**

**Leiomyoma of the ovary: A report of a rare case**

H. Trihia ^*^, D. Karagianni, G. Mitropoulou, I. Kasselaki, N. Kalinoglou

^*^Metaxa Cancer Hospital, Dept. of Pathology, Piraeus, Greece

**Objective:** Although most primary ovarian tumors are of surface epithelial, sex cord-stromal, or germ cell origin, a variety of rarer neoplasms are of other or uncertain lineage. Leiomyoma of the ovary is one of the rarest solid tumors of the ovary. Approximately 70 ovarian leiomyomas have been reported worldwide. We report one such rare case.

**Method:** A 35-year old woman was admitted with left abdominal pain. During U/S examination, a circumscribed solid mass of the left ovary was detected. Values of Ca125 and CEA were 7,5 and 0,40 respectively. She was submitted to resection of her left adnexa.

**Results:** Grossly, the ovary measured 6,5 × 4,5 × 5 cm and on cut surface there was a circumscribed solid tumor of 5 × 4,5 × 3,5 cm. The appearances were consistent with leiomyoma. Tumor cells were strongly positive for VIM, SMA and showed low MIB-1 expression.

**Conclusion:** Leiomyomas of the ovary are incidental findings in most of the cases and they resemble their uterine counterparts. They may arise from smooth muscle in the hilus or blood vessel walls or from foci of smooth muscle metaplasia of the ovarian stroma. The diagnosis is usually straightforward. Because of its rarity, it appears justified to report an additional case and focus some attention on this type of neoplasm.

**PS-22-042**

**Abnormal placentation and post partum hemorrhage: The pathologist and the accurate diagnosis**

S. Prévot ^*^, A.-E. Mas, M. Laroudie, L. Nomenjanahary, A. Benachi, M. Tassin

^*^Antoine Béclère Hospital, Pathology, Clamart Cedex, France

**Objective:** The indication of emergency peripartum hysterectomy has changed in recent years from uterine atony to abnormal placentation whose main risk factors are previous cesarean section and scarred uterus. This study analyzes the pathological characteristics of hysterectomy specimens collected over a period of 10 years because of post-partum hemorrhage.

**Method:** 27 hysterectomy specimens (and placenta in 19 cases) with abnormal placentation were reviewed with analysis of the placental site and search for uterine abnormalities.

**Results:** The 2 types of abnormal placentation were: abnormal involution of the placental site with uterine atony (1 case), placenta accreta (26 cases). In 1 case, the accreta implantation was focal and diagnosed because of peri menopausal hemorrhage. Placenta praevia was isolated in 2 cases. In the 23 remaining cases, placenta accreta was associated with placenta praevia in 10 cases. An uterine scar was present in 75 % of these 23 cases (because of cesarean section or surgery for malformative uterus). In 25 % adenomyosis or leiomyoma were observed.

**Conclusion:** This study allows to better describe the pathological and sometimes quite focal lesions of accreta placentation which can be search with the help of immunohistochemistry on products of curettage for abortion or delayed post partum hemorrhage.

**PS-22-043**

**Immunohistochemical expression of luteinizing hormone/human chorionic gonadotropic hormone in pregnant fallopian tubes**

H. Çetiner^*^, G. Kir, E. Kaygusuz, S. Ayaz

^*^Istanbul, Turkey

**Objective:** The etiology of ectopic pregnancy is uncertain but tubal transplantation is probably due to retension of the embryo owing the impaired embryo-tubal transport. Both cilial and myosalpingeal activity, are believed to be necessary for successful tubal transport. Number of studies have shown that the activation of Luteinizing hormon/human chorionic gonadotropic hormone (LH/hCG) receptors in Fallopian tube resulted in numerous changes that are required for pregnancy. We investigated whether Fallopian tubes bearing an ectopic pregnancy differs from a normal tube during the menstruel cycle in expression of LH/hCG hormon receptor.

**Method:** Thirty Fallopian tubes from women who diagnosed with ectopic pregnancy and 20 Fallopian tubes from women who had operation for benign diseases not effecting the tubes, were obtained. All the samples were immunohistochemically stained with LH/hCG hormon. To see that whether there are significant differences among patient groups Fisher’s exact test was applied.

**Results:** Number of cases with LH/hCG hormon receptor seen in myosalpinx is very high for ectopic pregnancy group (28/2) than control groups (follicular and luteal phase groups)(2/18). This difference is statistically significant (*p* value = 0.000)

**Conclusion:** Increased LH/hCG hormon expression in human Fallopian tube may play an important role in influencing tubal functions and embryo transport.

**PS-22-045**

**MSI in an Asian series of primary uterine endometrioid carcinoma**

I. Busmanis ^*^, T. Lim, M.-H. Tan

^*^Singapore General Hospital, Pathology, Singapore

**Objective:** a) To determine, by immunohistochemical(IHC)means,the incidence of microsatellite instability (MSI) in an Asian series of primary uterine endometrioid carcinoma (EC) b) To ascertain any clinico-pathologic associations.

**Results:** 6/20 cases demonstrated mismatch repair protein(MMR)deficiency(30 %) These comprised 4 Chinese,and 2 Malay patients, of average age 51.8 years Average tumour size was 5.6 cm;none were isthmic 4 cases were Grade II, 1 each were Grade I and III 2/6 were associated with endometrial hyperplasia. No significant lymphocytic infiltrate was seen. 2 patterns of MMR were identified: MSH2 MSH6 MLH1 PMS2 + + − − 4/20 (20 %) − − + + 2/20 (10 %)

**Conclusion:** Prevalence of IHC MMR deficiency in this Asian series of primary sporadic EC is 30 %, slightly higher than the average quoted for Caucasian populations. The MSI cases were large tumours (>5 cm)and none were located in the isthmus. Patients were younger (51.8 years) than the non-MSI group (62.6 years) The majority of EC were intermediate to high grade (83 %), and advanced stage (83 %). There was no noteworthy endometrial hyperplasia or lymphocytic infiltrate. Both the MSH2/MSH6- MLH1/PMS2+ cases were Stage III. The findings suggest that these sporadic MSI +ve EC cases may be expected to be associated with a poorer prognosis.

**PS-22-047**

**Metadherin and NF-kappa-B (p50/p65) expression in ovarian epithelial neoplasms**

I. Giopanou*, D. Papachristou, I. Lilis, P. Aroukatos, A. Papanastasiou, S. Kounelis, E. Papaspyrou, H. Papadaki

^*^University of Patras, Greece

**Objective:** Metadherin (MTDH) is a primary mediator of tumor progression, metastasis and chemoresistance in many human cancers and can activate several pathways, including the NFκB.

**Method:** We investigate, using immunohistochemistry, the expression of MTDH and NFkB (p65/p50) in formalin-fixed, paraffin embedded tissues from 76 patients with epithelial ovarian neoplasms (15/76 bordeline, 30/76 malignant, 31/76 benign). The relationship of MTDH/AEG1 with NFκB (p65/p50) and clinicopathological parameters such as tumor grade, stage, and patient age were evaluated. Statistical analysis was performed using SPSS 9.0 for Windows.

**Results:** Adjacent normal ovarian tissue was MTDH, and NFκB (p50, p65) negative. MTDH and NFκB (p50, p65) demonstrated immunopositivity in 1/31, 3/31 and 3/31 benign ovarian neoplasms respectively. MTDH and NFκB p50 immunoexpression was detected in malignant neoplasms and was statistically significantly higher compared with borderline tumors (*p* < 0.001). NF-κB p65 expression shows no significant differences between malignant and borderline neoplasms. Statistical significant correlation was observed between MTDH and NF-κB (p50, p65) expression in malignant neoplasms (*p* < 0.001). No statistical correlation was noticed between MTDH, NFκB (p50, p65) and clinicopathological parameters.

**Conclusion:** Our findings suggest that MTDH may play a role in the pathogenesis of human ovarian cancer possibly through the activation of the NFκB signalling pathway.

**PS-22-048**

**CAPRIN 1 expression in ovarian adenocarcinomas**

G. Erdogan ^*^, M. Özcan, Z. Çetin, E. Pestereli, S. Karaveli

^*^Akdeniz University, Patholgy, Antalya, Turkey

**Objective:** Caprin1 is a protein that encoded by cytoplasmic activation/proliferation-associated protein-1 gene located in 11p13 chromosomal region. Previous studies indicated that Caprin1 is associated with cell proliferation and might be overexpressed in some of the tumor types The aim of this study is to investigate caprin 1 expression in ovarian adenocarcinomas.

**Method:** Caprin1 expression was evaluated by immunohistochemistry in tissue microarrays of 26 ovarian adenocarcinomas.

**Results:** There were 14 cases of serous, 7 cases of endometrioid, 5 cases of mucinous carcinomas and 4 cases Stage I+Stage II, 22 cases Stage III. Strong positive cytoplasmic and membranous staining was observed in the tumour sections. Caprin 1 was positive in 21 of 26 ovarian adenocarcinomas. No statistically significant difference between caprin expression and grade, stage, tumor type, lymp node metastasis, ER, PR, cerbB2, p53 staining.

**Conclusion:** In our study, the number of cases was limited, and than our result was not evaluate the role of caprin 1 in progression of ovarian carcinomas. Further studies are needed to clarify the importance of caprin 1 expression.

**PS-22-049**

**Endometrial glandular dysplasia: A case report**

G. Erdogan ^*^, F. Çelik, E. Pestereli, S. Karaveli

^*^Akdeniz University, Patholgy, Antalya, Turkey

**Objective:** Based on morphologic and molecular findings, lesions that bridge between the resting endometrium and serous endometrial intraepithelial carcinoma(EIC) were designated as ‘Endometrial glandular dysplasia(EmGD)’ in 2004. EmGD may represent the earliest change in the development of endometrial serous carcinoma(ESC). Recently reported that about 20 % of women with ESC had an earlier history of breast cancer and the incidence was higher in patients who were younger age

**Method:** A 50-year-old woman who had an history of breast cancer and taking tamoxifen for several years was referred. Hysterectomy was performed.

**Results:** The microscopic examination showed an atrophic endometrium with endometrial glands and surface epithelium lined by atypical cells showing nucleomegaly, nuclear hyperchromasia, rare nucleoli, and no significant stratification. Rare papillae formation and atypical mitotic figures were present. p53 expression were evaluated by immunohistochemistry in these cells.

**Conclusion:** EmGD can be diagnosed by routine microscopic evaluation and requires the careful exclusion of morphologic mimics, such as metaplastic processes and EICs. Characteristics of p53 and MIB-1 immunostains of EmGD may be of diagnostic usage in surgical pathology practice. Recognition of EmGD potentially offers the opportunity to prevent the development of the associated malignancy and may provide an opportunity to improve the management of uterine serous carcinoma

**PS-22-050**

**Characterization of tumoral immune response in cervical carcinomas (CC) of pregnant women**

J. Ferreira ^*^, A. Felix

^*^IPO Lisboa, Serviço de Anatomia Patológica, Portugal

**Objective:** Cervical carcinomas are the second most common neoplasm in pregnancy (1-12/1,0000pregnancies). Being pregnancy a unique immunological situation, we evaluated the inflammatory infiltrate in CC diagnosed during this period.

**Method:** Twelve cases of CC in pregnant women were retrieved and a control group of non-pregnant CC patients with similar clinical-pathologic features was used. Counting of CD4, CD8 and CD68 was performed in 5 high-power fields(HPF) in stromal and intraepithelial compartments.

**Results:** Seven tumours were squamous cell carcinomas, three adenocarcinomas and two glassy cell carcinomas. All women were stage I or II and four, five and three patients were diagnosed in the 1st, 2nd and 3rd trimester, respectively. CD4+ and CD68+ cells numbers were similar in pregnant and non-pregnant patients, in stromal and intraepithelial lymphocytes. The mean number of total and intraepithelial CD8+ lymphocytes was higher in pregnant than in non-pregnant patients (total and intraepithelial CD8+ cells/HPF MD:37,4(*p* = 0,057) and 25,96(*p* = 0.052)), mainly during the first trimester (total and intraepithelial CD8+ cells/HPF MD:71,22 and 37,92).

**Conclusion:** Glassy cell carcinoma incidence is higher in pregnant women. In cervical carcinomas diagnosed during pregnancy, tumoral immune response is not significantly different from non-pregnant patients; although a slight increase of intraepithelial CD8+ lymphocytes was found, mainly during the first trimester.

**PS-22-051**

**Bilateral gonadoblastomas with unilateral dysgerminoma in a case of 46 XY pure gonadal dysgenesis (Swyer syndrome)**

F. Bolat ^*^, T. Canpolat, E. Kilicdag

^*^Baskent Universitesi, Adana, Turkey

**Objective:** Gonadoblastoma is a rare gonadal tumor, almost always arising from a dysgenetic gonad with a Y chromosome. Patient with 46 XY pure gonodal dysgenesis (Swywer Syndrome) are at higher risk of developing gonadoblastomas and other germ cell tumors.

**Method:** A 17 a-year-old patient with female phenotype presented with delayed puberte and primary amenorrhea. On physical examination, secondary sexual characteristic were poorly developed but normal external genitalia. An endocrine evaluation was showed hypergonodotropic hypogonadism. Laparoscopy showed normal uterus and fallopian tubes with streak gonads. Bilateral gonadectomy was done.

**Results:** Histopathologic examination of both gonads showed germ cell proliferation and sex cords derivates frequently surronding small round deposits containing amorphous hyaline material resembling Call-Exner bodies. Thus, a diagnos, of gonadoblastoma was made. The right gonad also showed a dysgerminoma extensively overgrowing the gonadoblastoma. On immunohistochemistry, the overgrowing tumor cells were positive for placental alkaline phosphatase and C-kit but negative for CD30, inhibin, cytokeratin. A final diagnosis of bilateral gonadoblastoma with overgrwon dsygerminoma of the right gonad in case of XY pure gonadal dysgenesis (Swyer Syndrome) was made.

**Conclusion:** In this report we describe clinical and histopathological features of bilateral gonadoblastoma with coexisting disgerminoma in a girl with a 46 XY karyotype.

**PS-22-053**

**An immunohistochemical study of CD10 and h-caldesmon in uterine atypical polypoid adenomyofibroma and myoinvasive endometrial adenocarcinoma**

V. Ivanova ^*^, M. Karaivanov, T. Dineva, I. Ivanov, S. Popovska

^*^Medical University, General and Clinical Pathology, Sofia, Bulgaria

**Objective:** Atypical polypoid adenomyofibroma (APA) is a rare uterine tumor that generally occurs in women of reproductive age. The major differential diagnostic problem presented by APA is the exclusion of well-differentiated endometrial carcinoma invading the myometrium, especially in curettage material. The aim of this study is to investigate the expression of CD10 and h-caldesmon in APA and compare them with those present in myoinvasive well-differentiated endometrial adenocarcinoma.

**Method:** Curettage and hysterectomy materials from 10 patients with APA and 5 with polypoid adenomyofibroma, along with 15 cases of grade I myoinvasive endometrioid adenocarcinoma were studied. All cases consistently expressed Smooth Muscle Actin and were further evaluated by CD10 and h-caldesmon (DAKO).

**Results:** The myometrium invaded by endometrioid carcinoma showed diffusely positivity for h-caldesmon, while CD10 was expressed only in fringe-like pattern around neoplastic glands. Stromal elements in all adenomyofibroma cases revealed CD10 positive signals. In 8 out of 15 specimens, especially at the basis of polypoid formations, CD10 was focally underexpressed and emergence of h-caldesmon expression was noted.

**Conclusion:** Different staining patterns for CD10 and h-caldesmon in APA and grade I myoinvasive endometrioid adenocarcinoma may be useful tool in differential diagnostic process.

**PS-22-054**

**Hydatidiform mole oncogenesis: Immunohistochemical study**

S. Hmissa ^*^, H. Landolsi, N. Missaoui, A. Essakly, S. Korbi, M. Njima, S. Ben Abdelkarim, M. T. Yaacoubi

^*^Farhet Hached Hospital, Dept. of Pathology, Sousse, Tunisia

**Objective:** Hydatidiform moles (HM) are gestational trophoblastic diseases with a potential of malignant transformation involving multiple genetic alterations. Here, we investigate the expression of Cerb-2 and bcl-2 oncoproteins, p53, p21 and p63 tumour suppressor genes, and cell proliferation marker (Ki-67) in non-molar hydropic abortions (HA), partial HM (PHM) and complete HM (CHM).

**Method:** We used immunohistochemistry on 140 CHM, 41 PHM and 39 HA.

**Results:** Positive staining for Cerb2 was found in 13 cases (10 CHM and 3 PHM). The expression of bcl-2 was significantly higher in HM than in HA (*p* < 0.05) but there was no significant difference for this protein expression between CHM and PHM. The expression of p21 was significantly higher in HM than in HA (*p* < 0.05) but there was no significant difference for this protein expression between CHM and PHM. p53 was significantly higher in CHM than PHM and HA (*p* < 0.05). The expression of ki-67 and p63 was significantly higher in CHM than HA (*p* < 0.05), but the difference between CHM and PHM and between PHM and HA was not significant.

**Conclusion:** Altered expression of oncoproteins and tumour suppressor genes may be important in the pathogenesis of HM. Quantitative techniques should be more useful for the evaluation of the HM prognosis.

**PS-22-055**

**Pleomorphic uterine leiomyosarcoma with heterologous chondrosarcomatous differentiation: A case report**

V. Caamaño Villaverde ^*^, M. Saiz Camin, A. Corominas Cishek, G. Muñiz Unamunzaga, A. Perez Zabala, L. Andrés Alvarez

^*^Cruces University Hospital, Pathology, Barakaldo, Spain

**Objective:** Malignant change in a leiomyoma or uterine fibroid tumor is termed leiomyosarcoma. It arises from smooth muscle of the uterus and is a rare tumor that accounts for 2–5 % of all uterine malignancies. The molecular events that underlie the genesis remain unknown, but the emerging lines of evidence suggest that some leiomyosarcomas have the ability to evolve from benign to more aggressive lesions.

**Method:** 47 years old female patient G2C2 with several months history of hypermenorrhea. The patient’s uterus was enlarged to 20 weeks gestational size, she underwent subtotal abdominal hysterectomy with right salpingoophorectomy. Gross examination showed a mass resembling a leiomyoma, distorting uterine cavity. Tube, ovary and cervix were not involved.

**Results:** Histopathologic examination showed a cellular sarcoma with necrosis, prominent pleomorphic atypical nuclear features and high rates of mitosis (> 7/10 hpf). Heterologous chondrosarcomatous differentiation was seen in some fields. Immunohistochemistry showed positivity for smooth muscle markers and focal positive staining with CD10. ER, PR, betacatenin and inhibin were negative.

**Conclusion:** Uterine leiomyosarcoma is a rare neoplasm, considered to be highly malignant due to high metastatic potential and recurrence rates even if diagnosed at early stages. Heterologous differentiation in leiomyosarcomas is an extremely rare phenomenon, with few cases reported to date.

**PS-22-056**

**Coexistence of ovarian mature cystic teratoma and endometriosis with smooth muscle metaplasia: Case report**

V. Leontara^*^, A. Dimitriadi, N. Chaleplidis, S. Antoniou, E. Papaliodi

^*^General Hospital of Athens, Surgical Pathology, Greece

**Objective:** Smooth muscle is a frequent component of endometriotic lesions in pelvic locations, like ovary. Mature cystic teratoma is also a frequent neoplasm of the ovary. What is the role of coexistence of these two entities in the same ovarian mass, and how often is this encountered?

**Method:** We report a case of a 29-year-old woman submitted to our hospital complaining about pain at the left pelvic area, with personnal history of right oophorectomy. Radiological examination showed a cystic mass of the left ovary with different possible diagnosis between dermoid and endometriotic cyst. Ca-125 in plasma was >1000 U/ml. We received a multilocular cystic mass with smooth outer surface measuring 13 × 9.5 × 5 cm.

**Results:** Histologically, a mature cystic teratoma was recognized with the presence of epidermis, sebaceous glands, hair and fat tissue. However, the biggest part of the mass represented an endometriotic cyst with smooth muscle metaplasia.

**Conclusion:** There have been numerous theories trying to explain the pathogenesis of smooth muscle metaplasia in endometriotic lesions. In the international literature there is only one case of endometriosis with smooth muscle metaplasia, with a synchronous ovarian thecoma. To our knowledge, this is the first reported case of coexisting ovarian mature cystic teratoma and endometriosis with smooth muscle metaplasia.

**PS-22-057**

**Adenosarcoma of the uterine corpus with rhabdomyosarcomatous differentiation: A case report**

E. Goupou ^*^, I. Michalopoulou-Manoloutsiou, D. Gerasimidou, B. Christoforidou, F. Chatzinasios, F. Patakiouta

^*^Theagenion Cancer Hospital, Pathology, Thessaloniki, Greece

**Objective:** Adenosarcoma of the uterine corpus is a rare, biphasic neoplasm, containing a benign epithelial component and a sarcomatous mesenchymal component.

**Method:** We report a case of a 74 year old female patient, whose MRI showed an enlarged uterus with widened endometrial cavity and the presence of a large endometrial tumor. Total hysterectomy was performed.

**Results:** Gross examination showed an exophytic, polypoid mass, 6 cm in diameter, that extended into the uterine cavity. Microscopically, the tumor was biphasic, consisting partially of a spindle cell stromal sarcoma and simultaneous presence of numerous rhabdomyoblasts with eosinipholic fibrillar cytoplasm and cross-striations. Among them, there were scattered isolated, often dilated or compressed benign glands. Immunohistochemically, the spindle cells were positive for CD10, SMA, CD34 and negative for CD117, EMA and S100. The rhabdomyoblasts were positive for desmin and sarcomeric actin.

**Conclusion:** The differential diagnosis includes adenofibroma in adults and embryonal rhabdomyosarcoma in children. Adenosarcoma is considered a low grade neoplasm, but recurs in approximately 25–40 % of cases. Rhabdomyosarcomatous differentiation, as in our case, is an adverse prognostic factor in some series. Patient received adjuvant chemotherapy and remains well, 6 months after diagnosis.

**PS-22-059**

**PSMAD2 expression in cervical lesions and its relation to high risk HPV types**

D. Koumoundourou^*^, E. Nikolatou, H. Geropoulou, P. Ravazoula

^*^University Hospital of Patras, Pathology, Greece

**Objective:** It is well established that in cervix, high risk HPV viruses play an important role in the malignant transformation procedure. On the other hand pSmad2 is a TGF-beta intracellular mediator which translocates to the nucleus and triggers TGF-beta-dependent gene transcription thus usually having a tumor-suppression role. The purpose of the present study was the assessment of pSmad2 and HPV status in cervical lesions and their correlation with their grade.

**Method:** pSmad2 and HPV expression was evaluated in seven CIN I, 11 CIN II, 21 CIN III and 10 invasive carcinomas using immunohistochemistry

**Results:** The percentage of pSmad2 positive cells was significantly reduced in high grade lesions (*p* = 0,065) and was strongly correlated with the presence of high risk HPV types (*p* = 0,022). HPV expression was significantly higher in premalignant lesions (*p* = 0,035)

**Conclusion:** Our results indicate that pSmad2 deletion is a critical step in cervical carcinogenesis, while the correlation between TGF-beta pathway and HPV- induced malignant transformation seems quite challenging.

**PS-22-060**

**PTEN, PAX2 and PINCH protein expression in normal endometrium, endometrial hyperplasia and endometrioid adenocarcinomas: Correlation with clinicopathologic parameters**

O. Erdem ^*^, E. Karakus, C. Taskiran, A. Onan, R. Karakus

^*^Gazi University, Pathology, Ankara, Turkey

**Objective:** The objective of this study is to investigate the role of PTEN; PAX and PINCH expressions in endometrial carcinogenesis.

**Method:** 27 proliferative endometrium, 24 complex atypical hyperplasia, 10 complex hyperplasia without atypia, 30simple hyperplasia without atypia and 89 endometrioid type endometrial adenocarcinoma were selected. PTEN, PAX2 and PINCH antibodies were applied immunohistochemically to all cases.

**Results:** Immunohistochemical staining revealed that during progression from normal to malignancy, staining distrubition and intensity of both PTEN and PAX2 were found the decrease significantly. Expession of PINCH was found to be increased distinctly during progression from normal to malignancy which correlated reversly with PTEN and PAX 2 expressions. When PTEN, PAX2 and PINCH expressions in endometrioid adenocarsinoma were compared with histopathologic prognostic parameters, a relation between histopathologic grade, angiolymphatic invasion, lymph node metastasis, stage, myometrial invasion was not detected.

**Conclusion:** It is assumed that PTEN, PAX2 and PINCH could be involved in endometrial carcinogenesis. However to define the prognostic importance of these markers, studies in larger series and with long term follow up could be performed.

**PS-22-061**

**Sex Cord Tumours (SCT) of the ovary: Incidence, clinical behaviour and morphological study of 26 cases during a 18-year period in Vall d’Hebron hospital in Barcelona**

R. L. Palhua Flores ^*^, J. Temprana Salvador, J. Castellví, A. Navarro, M. Aizpurua, S. Ramon y Cajal, A. García

^*^Vall d’Hebron Hospital, Pathology, Barcelona, Spain

**Objective:** SCT account for less than 3 % of ovarian tumours and their behaviour is unpredictable. Our goal is to determine several immunohistochemical markers with predictive significance.

**Method:** We reviewed our archives (1994 to 2013) finding 26 cases of a total of 1835 ovarian tumours, accounting for 1,4 % of our series. All cases were reexaminated and immunohistochemical stains for p16, HER2, p53, ki67, oestrogen and progesterone receptors were performed.

**Results:** Age ranged from 6 months to 85 years with an average of 52. Histologically, there were different subtypes: granulose cell tumours 12 (46.2 %) (2 juvenile type), Sertoli/Leydig: 7 (26.9 %), sex cord tumour with annular tubules: 2 (7.7 %) and unclassifiable: 5 (19.2 %). As far as their behaviour was concerned: 23 cases (88.5 %) remained free of disease, 2 (7.7 %) relapsed locally after treatment and 1 (3.8 %) died. Oestrogen and progesterone receptors were positive in 100 %. p16 was expressed in 60 % and HER2 in 40 %. Ki67 and p53 expression were lower than 10 and 20 % respectively. No correlation was found amongst different subtypes and their behaviour.

**Conclusion:** Apart from morphology, no other specific immunohistochemical prognostic factors have been proved so far, thus it remains important to continue the study.

**PS-22-062**

**Krukenberg tumor: About of 40 cases**

R. Hamrouni^*^, R. Dhouib, R. Doghri, L. Charfi, N. Boujelbene, I. Abbes, S. Sassi, M. Driss, K. Mrad, K. Ben Romdhane

^*^Salah Azaiez Hospital, Pathology, Tunis, Tunisia

**Objective:** To determine the pattern of presentation of Krukenberg tumor(KT), microscopic diagnosis, primary origin and clinicopathologic correlation and survival of patients.

**Method:** 40 patients diagnosed with KT of the ovary, in our pathology department, who underwent surgical treatment between 1997 and 2012 were retrospectively evaluated.

**Results:** Among the 40 cases of KT due to GI cancers, there were 10 cases synchronously diagnosed, 2 cases with primary tumor identified first and 28 cases with ovarian tumor identified first. Of the 40 patients, the median age at diagnosis of KT was 46.7 years (range, 17–65). Stomach is the most common primary site. It was confirmed in 5 cases (12.5 %), followed by colon in 3 cases (7.5 %) and appendicular in 2 cases (5 %). Most of the patient had bilateral ovaries involved (82.5 %). The frozen section was done in 29 cases (72.5 %). The largest dimension was 16 × 11 cm. Métastases to uterine were found in 2 cases (5 %) and peritoneal carcinomatosis in 15 cases (37.5 %).

**Conclusion:** Patients with KT from colorectal cancer experience a better prognosis than those from gastric cancer and benefit more from metastasectomy.

**PS-22-063**

**Squamous cell carcinoma arising from mature cystic teratoma of the ovary: Report of a case**

E. Kairi-Vasilatou ^*^, E. Carvounis, C. Dastamani, A. Tsagkas, A. Kondi-Pafiti

^*^Aretaieion University Hospital, Dept. of Pathology, Athens, Greece

**Objective:** Dermoid cyst or mature cystic teratoma (MCT) is one of the most common tumors in females. Malignant transformation is rare and occurs in about in 1–2 % cases, typically in postmenopausal women.

**Method:** A case of squamous cell carcinoma (SSC) arising in a MCT diagnosed in our laboratory is presented.

**Results:** The patient was a 56-year old postmenopausal female, who presented with a cystic mass in the anatomic space of the right ovary. A tumorectomy was performed. The ovarian mass had a smooth external surface and measured 10,5 × 8,5 × 7,5 cm in size. It was filled with white greasy sebaceous material intermixed with hair. Microscopically, keratinising squamous epithelium with underlying skin adnexa, respiratory type epithelium, cartilage and neural tissue were identified. Focally, the cyst lining consisted of non-keratinising squamous epithelium with marked acanthosis and atypia, reminiscent of differentiated VIN-III and superficially invasive SCC. The lesion measured 1,2 cm. The external surface of the cyst wall was intact.

**Conclusion:** SCCs arising in MCTs occur rarely and are not usually diagnosed preoperatively as there are no specific symptoms or signs associated with malignant transformation.

**PS-22-064**

**Epithelioid leiomyoma, clear cell variant, of the cervix: A case report**

P. Karabagli ^*^, S. Bastoklu

^*^Selcuk University, Medical Faculty, Pathology, Konya, Turkey

**Objective:** Epithelioid leiomyomas of the cervix are extremely uncommon neoplasms and unlike ordinary leiomyomas, show substantial epithelial differentiation.

**Method:** We report a case of the epithelioid leiomyoma, clear cell variant of the cervix in a 51-year-old women.

**Results:** The patient had abdominal pain and vajinal bleeding. The major axis of the tumor was 22 cm, and the tumor was brown, soft and homogeneous. Histologically, the tumor was composed of round to polygonal shape, epithelioid and clear cells. Nuclear atypia, pleomorphism and necrosis were not seen, Mitotic index was 2 per 10 high power fields. The tumor cells were immunoreactive for SMA and Desmin, and focally immunoreactive for EMA. CD34 and S-100 were immunonegative.

**Conclusion:** We presented the rare case of epithelioid leiomyoma, clear cell variant of the cervix and reviewed the current literature in this report.

**PS-22-065**

**The evaluation of the reliability and contribution of frozen section pathology to staging endometrioid adenocarcinomas**

P. Karabagli ^*^, B. Yilmaz, C. Celik, S. Ugras

^*^Selcuk University, Medical Faculty, Pathology, Konya, Turkey

**Objective:** The objective of this study is to evaluate the reliability and accuracy of intraoperative pathological findings, compared to permanent section (PS) and to understand the contributions of frozen section (FS) to final staging in patients with endometrioid carcinomas.

**Method:** This is a retrospective analysis of 79 patients with intraoperative FS of endometrioid adenocarcinomas. FS findings of cases were compared with PS as to grade, depth of myometrial invasion (MI), cervical involvement, lymphovascular space invasion (LVSI) and stage. Whether staging procedures such as metastasis of lymph nodes, peritoneal cytology and extension out of uterus, and tumor diameter were suggestive of FS findings or not were also analysed. Staging was based on the FIGO 2009 system. SPSS 15.0 was used for statistical analysis.

**Results:** FS results were agreement with PS in 89.9 % for the grade, 88.6 % for MI, 100 % for cervical invasion, and 92.4 % for LVSI. Final pathology upstaged 12 %, 16.6 % and 44.4 % of FS stage IA, IB and II specimen, respectively. On FS, 3.4 %, 16.7 % and 44.4 % of patients in stages IA, IB and II had lymph node metastases, respectively.

**Conclusion:** FS is a useful procedure to identify poor prognostic pathological factors as well as to diagnose endometrial cancer.

**PS-22-066**

**High proliferation potential in Ovarian Surface Epithelium (OSE) From BRCA mutated patients: Evaluation of Ki-67 expression and identification of a possible new condition in these patients**

P. Jiménez León ^*^, B. Majem Cavaller, J. Camacho Soriano, J. Temprana, C. Delbene, A. García, M. Rigau, S. Ramon y Cajal, J. Castellvi

^*^Vall d’Hebron Hospital, Pathology, Barcelona, Spain

**Objective:** We have observed that primary cultures (PC) from OSE samples of prophylactic bilateral salpingo-oophorectomy (PBSO) from BRCA mutation carriers grow faster than the PC from control ovaries. Our goal was to determine whether this high proliferation potential can be reflected by immunohistochemical techniques.

**Method:** Twenty PBSO specimens obtained between 2010 and 2013 in our hospital, corresponding to 20 women with BRCA mutation were collected. Ki67 expression in OSE, and Ki67 and p53 in tubes were evaluated.

**Results:** Only one case showed a continuous expression of Ki67 in an otherwise normal OSE; the remaining cases were negative. In this case P53 was performed, which was similarly positive. The fallopian tubes and the contralateral ovary showed no abnormalities. The remaining samples showed no morphological abnormalities in the fallopian tubes except one case which showed a “P53 signature” lesion.

**Conclusion:** The high proliferative rate of PC with BRCA mutation apparently cannot be explained by the expression of Ki67. The alteration detected in the sole positive case has not been previously described and raises the possibility of the existence of carcinoma in situ in the OSE, in the same manner that occurs in fallopian tubes. Further studies are necessary to determine the significance of these findings.

**PS-22-067**

**Evaluation of MisMatch Repair (MMR) protein immunohistochemical expression in endometrial carcinomas**

C. Bartosch ^*^, S. Relvas, C. Jeronimo, J. M. Lopes

^*^IPO-Porto, Pathology, Portugal

**Objective:** To evaluate immunohistochemical MMR expression in endometrial carcinomas.

**Method:** Patients with both synchronous/metachronus neoplasias plus family history, or >1 relative with neoplasia in consecutive endometrial carcinomas (*n* = 188) diagnosed (2000–2010) at CHSJ; MMR immunohistochemical expression (present/absent/weak): MLH1, MSH2, MSH6 and PMS2; statistical analysis.

**Results:** 51 (27 %) identified. Median age: 51 years; type I tumor (24G1, 14G2, 7G3); type II: 6; FIGO stage: 36I, 7II, 8III. 31 MMR normal expression; 20 MMR loss of expression (LOE): 8 MLH1/PMS2 (4 %); 8MSH2/MSH6 and 4MSH6 (6.3 %); 19 type I (9G1, 7G2, 3G3) vs 1 type II (MSH6). No significant differences for age, obesity,grade,tumor type and FIGO stage, when comparing cases with vs without LOE. Cases with LOE showed higher (not significant) frequency of lymphovascular invasion (45 % vs 29 %), worst overall outcome (5-year: 88.7 % vs 68.4 %, *p* = 0.107)and disease-free survival (5-year: 88.6 % vs 61.9 %, *p* = 0.036). So far, five patients with LOE displayed MMR germline mutations (3MSH2/MSH6; 2MSH6); the others are under genetic counseling.

**Conclusion:** Our frequency of MMR LOE fits with literature, namely MSH2/MSH6 and MSH6 patterns that are usually caused by MMR germline mutations. Immunohistochemical screening of MMR is useful for the identification of Lynch syndrome in endometrial carcinoma patients with personal/family history of neoplasia.

**PS-22-068**

**Serous tumors of the ovary: Diagnostic challenges at frozen section and clinical implications**

A. Dhaoui^*^, L. Charfi, K. Mrad, R. Sellami, R. Doghri, N. Boujelbene, M. Driss, I. Abbes, S. Sassi, K. Ben Romdhane

^*^Salah Azaeiz Institut, Pathology, Tunis, Tunisia

**Objective:** Frozen section (FS) diagnosis of serous tumors of the ovary can be difficult due to the size of these tumors, heterogeneity and potential risk of metastasis from peritoneal or endometrial neoplasms. Few data are provided on this topic in the pathology literature explaining our objective to try to study the reliability of FS diagnosis in ovarian serous tumors.

**Method:** A retrospective review of 120 ovarian serous tumors submitted for(FS)evaluation between August2010 and April2013 was conducted. FS and final pathology results were collected.

**Results:** The average tumor size was 16,3 cm(1–32,5 cm). The FS and final pathology diagnosis were concordant in 91,6 %(110/120) of the cases. Of the 10(8,3 %) discordant cases, three(30 %) was downgraded,two(20 %) was upgraded and 5 cases(50 %) have a different histological type. Of the 6 tumors interpreted as borderline serous tumors(BST) on FS, 2(30,3 %) were malignant at final diagnosis, 2(30,3 %) remain as BST and 2(30,3 %) was benign. Of the73(60 %) benign tumors on FS, one case(1,3 %) were upgraded to serous carcinoma at final diagnosis. Tumors with a malignant diagnosis on FS were 100 % concordant with final diagnosis.

**Conclusion:** Our study showed a 8,3 % rate of discordance between FS and final diagnosis mainly in the histological type hardly defined on FS.

**PS-22-070**

**An unexpected tumor of the adnexial region: Adrenocortical carcinoma**

I. Isik Gonul ^*^, O. Ekinci, B. Cetin, M. A. Inan, U. Coskun

^*^Gazi University Medical School, Dept. of Pathology, Ankara, Turkey

**Objective:** The aim of this presentation is to introduce the clinicopathological features of a 52 year-old female patient presenting with a right adnexial mass. The patient was operated with a clinical diagnosis of ovarian carcinoma.

**Method:** The right salpingo-oophorectomy material was examined by frozen section. There was a solid, encapsulated mass located in the right adnexial region. The ovary and the tuba uterina could clearly be seperated from the mass on gross examination. The frozen diagnosis of “undifferentiated high-grade malignant tumor” was given.

**Results:** The permenant sections revealed a high-grade poly-phenotypic tumor with multifocal necrotic foci. In the surrounding areas, cells with clear cytoplasm similar to adrenal cortex were identified. Light microscopy and large-panel immunohistochemistry confirmed the diagnosis of adrenocortical carcinoma.

**Conclusion:** Abdomen and pelvis are relatively common sites for the presence of aberrant adrenal tissue. However, development of adrenocortical carcinoma arising from this tissue is extremely rare. Its radiological and clinical diagnosis is almost impossible in non-functioning cases.

**Encapsulated, solid mass with yellow-orange coloured cross-section.:** 
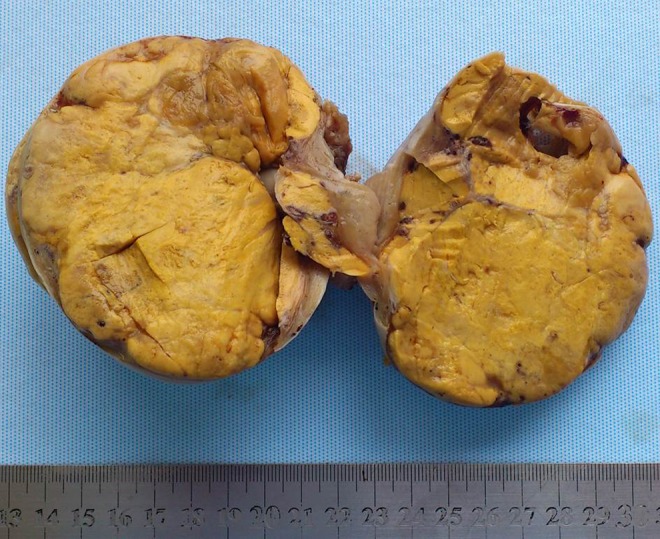


**PS-22-071**

**Simultaneous Serous Tubal Intraepithelial Carcinoma (STIC) and serous carcinoma of the uterine corpus within a carcinosarcoma (malignant müllerian mixed tumor): Divergent expression patterns for Wilms-tumor 1**

M. Igaz ^*^, H. Brustmann

^*^Landesklinikum Baden/Moedling, Pathology, Austria

**Objective:** We report the case of a patient who underwent Wertheim’s operation for a biopsy- proven uterine carcinosarcoma (malignant Müllerian mixed tumor (MMMT)). Histological workup of the specimen revealed a serous papillary adenocarcinoma as the epithelial component of a heterologous MMMT. Applying the SEE-FIM protocol a serous intraepithelial carcinoma (STIC) was noted in the right Fallopian tube. This is an unusual finding, since STICs are frequently associated with serous carcinomas of the ovary or peritoneum.

**Method:** Case report.

**Results:** Serous neoplasia at both locations showed crisp nuclear p53 immunoexpression. However, immunostaining for Wilms-tumor 1 (WT-1) was exclusively observed in the lesion found in the FT in cytoplasmic and membranous patterns. In contrast, WT-1 is known to be expressed in such tumors of tubal as well as ovarian sites. Both tumors were reactive for AE1-AE3 cytokeratin. The rate of cycling cells as determined by MIB1 was about 50 %.

**Conclusion:** We conclude that serous neoplasia may develop through different pathways in different parts of the female genital. This obviously rare pathway may be alternating to the more commonly observed one in STICs and associated ovarian serous carcinomas. A customary application of the SEE-FIM protocol may yield higher frequencies of such lesions and should be considered by pathologists.

**PS-22-072**

**Collision primary tumor of the endometrium- small cell neuroendocrine carcinoma and endometrioid adenocarcinoma. A case report**

D. Crisan ^*^, M. Oltean

^*^Pathology, Cluj-Napoca, Romania

**Objective:** Small cell neuroendocrine carcinomas of the endometrium are rare tumors, which may present as distinct tumors or may be associated with other histological types of endometrial cancer.

**Method:** A 57-year-old postmenopausal woman was admitted at the Department of Gynecology for vaginal bleeding. Clinical investigations revealed an endometrial tumor suspected of malignancy and a total hysterectomy with bilateral adnexectomy was performed. The macroscopic examination of the specimen showed two separated tumor masses, located at the fundus, filling the entire uterine cavity and infiltrating more than one half of the uterine wall. Hematoxylin-eosin stain and immunohistochemistry were used for the histologic diagnosis.

**Results:** Microscopically, there were two types of endometrial carcinoma: a well differentiated endometrioid adenocarcinoma, and a small cell neuroendocrine carcinoma (with three positive neuroendocrine markers). The tumors were sharply delineated from one another. The neuroendocrine component was more aggressive, showing transmural and angiovascular invasion.

**Conclusion:** The classic aspects of the neuroendocrine carcinoma of the endometrium and the main differential diagnoses are discussed. Collision tumors of the endometrium are rare and small cell neuroendocrine carcinoma may be a part of such associations of tumors.

**PS-22-073**

**Age related morphological features and hormone receptor expression in the human clitoris**

A. Luís ^*^, C. Meireles, C. Bartosch

^*^Portuguese Oncology Institute, Pathology, Porto, Portugal

**Objective:** To evaluate the morphological features and hormone receptor expression of the human clitoris.

**Method:** Histological evaluation of 11 representative slides collected from vulvectomy specimens. Qualitative evaluation of morphology using H&E and Masson trichrome stains. Semiquantitative evaluation of estrogen receptor (ER) and progesterone receptor (PR) immunohistochemical expression.

**Results:** Case age distribution: group A:3 cases <60 years, group B: 4 cases 60–70 years, group C: 4 cases >80 years. There were no obvious morphological differences in vascular and nervous structures when comparing the 3 groups. Cavernous bodies (CB) showed more extensive fibrosis in C. ER expression was found in connective tissue of CB, tunica albuginea (TA) and periclitorian tissue (PC). ER expression was diffuse in A and B, and multifocal in C. PR expression was multifocal in group A, including CB, TA and PC. Focal PR expression was found in TA and PC in B, while in group C it was limited to PC. CB smooth muscle showed focal ER expression in all groups and focal PR expression in A.

**Conclusion:** Clitoris hormone receptor expression and fibrosis extension appear to change with age. This most probably relates to hormonal changes after menopause, as it has been suggested by animal model studies.

**PS-22-074**

**Rosai-Dorfman disease: Vaginal case report**

F. Filho ^*^, C. Alves, J. Alves, F. V. Ferreira

^*^Universidade Federal do Ceara, Dept. of Pathology, Fortaleza, Brazil

**Objective:** Comparative report on a vaginal case and the general data of the Rosai-Dorfman disease (RDD). RDD was first described in 1969 and is characterized by atypical histiocytes showing emperipolesis, being a special reactive condition. Although RDD shows a predilection for affecting cervical nodes, there are reports of extra-nodal sites. Some authors calls these extranodal and no cutaneous forms as atypical and suggests drug treatment (corticoids, metrotrexate). Cases may involve the genitourinary tract, however there aren’t cases in PubMed or Cielo citations of vaginal involvement

**Method:** Case presentation: woman 46 y old was submitted in Dec 2012 to exéresis of vaginal introitus nodular mass large than 2.0 × 1.5 × 1.4 cm, without capsule.

**Results:** Routine Histopathology revealed RDD affecting vaginal mucosae and muscular wall. Imunomarkers for gian emperipolesis cells shows CD68+ and vimentin +, CD1a negative Desmin and Actin enhances clear muscular fibers diruption. The patient are disease free by clinical and Pet Scan image four months after excision, despite no special drug treatment; she remains under medical periodical care

**Conclusion:** Vaginal RDD have pathological parameters as elsewhere and in owners previous skin, nodal and laryngeal cases. Evolution too, despite muscular invasion in this atypical one

**PS-22-075**

**Reproducibility of Squamous Cervix Lesions (CSL) diagnosis by pathologists and pathology residents in Portugal**

E. Rios ^*^, C. Bartosch, J. M. Lopes

^*^Centro Hospitalar São João, Dept. of Pathology, Porto, Portugal

**Objective:** Classification of CSL remains controversial. We evaluated inter-observer and intra-observer reproducibility of CSL diagnosis by pathologists and pathology residents (5-year training) from several pathology departments.

**Method:** Online quiz with representative H&E images of 50 CSL selected from two pathology departments. Pathologists and residents answered anonymously, assigning one out of six options (metaplasia, condyloma, CIN1, CIN2, CIN3, and invasive carcinoma) in two rounds 1-year interval; inter-observer and intra-observer agreement evaluated with Kappa coefficients.

**Results:** 35 pathologists and 22 residents participants (19 also in the second round): overall inter-observer reproducibility fair (*k* = 0.31), highest in CIN3 (*k* = 0.44) and invasive carcinoma (*k* = 0.75), lowest in CIN2 (*k* = 0.20); intra-observer agreements: fair (*k* = 0.21) to good (*k* = 0.62); inter- and intra-observer agreement higher among pathologists (*k* = 0.34, *k* = 0.47) than residents (*k* = 0.28, *k* = 0.43), with an improving tendency in older than younger (≤3 years) residents; reproducibility increases to moderate (*k* = 0.42) in pathologists when grouping lesions as no dysplasia, low grade, high grade, and invasive carcinoma.

**Conclusion:** Our results fit with reported literature. Reproducibility regarding classification of CSL falls short of required, particularly in ≤CIN2 lesions which may benefit from ancillary methods (e.g., p16 IHC) for adequate clinical management of patients. Implementation of national proficiency testing is warranted to improve diagnostic accuracy of CSL in Portugal.

**PS-22-076**

**Two examples of tubulo-squamous polyps of vagina and cervix, one of them with a minimal glandular component overgrown by squamous epithelium**

Á. Ilyés ^*^, S. Stolnicu, E. Quiñonez, F. F. Nogales

^*^Emergency Country Hospital, Dept. of Pathology, Targu Mures, Romania

**Objective:** The tubulo-squamous polyp (TSP) of the cervix and vagina is a recently described, but unusual, entity displaying an acinar prostatic immunophenotype that may derive from developmentally misplaced Skene’s (paraurethral) glands. Histologically, it usually is a nodular lesion composed of narrow tubular or cystically dilated glands with areas of eccentric, massive, squamous metaplasia.

**Results:** Two 76 and 55 year-old patients were admitted for vaginal bleeding due to 1.5–2.5 cm masses situated in the cervix and vagina. Microscopically, the one occurring in the older patient consisted of two independent lesions and had standard TSP histology, whilst the second one, which originated in the posterior vaginal wall only showed confluent non-keratinizing squamous epithelium nodules, often with central necrosis, resembling infiltrating squamous carcinoma. However, TSP was finally diagnosed in step sections when scarce tubular structures were identified at the periphery of epithelial nests that were positive for PSA and other acinic prostatic markers (prostatic acid phosphatase and basal cell markers). Estrogen receptors were positive in both glands and squamous cells while androgen receptors were only found in tubular glans. Ki67 index was <1 %.

**Conclusion:** It is concluded that in some TSP, extensive squamous component may obliterate the prostatic-type glandular component inducing confusion with squamous cell carcinoma.

Wednesday, 4 September 2013, 09.30–10.30, Pavilion 2

**PS-23 Poster Session Molecular Pathology**

**PS-23-001**

**PI3K/AKT pathway analysis in breast cancer: Association with clinicopathological features**

A. Saetta ^*^, A. Tserga, I. Chatziandreou, N. V. Michalopoulos, P. Kontogianni, P. Korkolopoulou, E. Patsouris

^*^University of Athens, 1st Dept. of Pathology, Greece

**Objective:** To analyse PIK3CA, AKT1, PTEN mutations simultaneously with mRNA relative expression levels of PIK3CA and AKT2 in Greek breast cancer patients

**Method:** Mutations in PIK3CA, AKT1 and PTEN genes were analysed in 75 breast carcinomas using: HRMA, pyrosequencing and sequencing. Relative quantitation of PIK3CA and AKT2 mRNA expression levels was performed for 50 cases and 10 corresponding normal samples using RT-PCR.

**Results:** PIK3CA exon 20, exon 9 mutations were detected in 14.6 % and 13.3 % of the cases, respectively. AKT1 mutation p.E17K was present in 4 % of the samples. PTEN p.G132V (1.3 %) and p.Q214* (1.3 %) were identified in exons 5 and 7 respectively. Exon 20 mutations were correlated with larger tumor size (*p* = 0.028) whereas exon 9 mutations with low to moderate tumor grade (*p* = 0.012). A statistically significant difference (*p* < 0.001) of AKT2 mRNA expression levels (ΔCt) were observed between breast carcinomas and normal tissues.

**Conclusion:** Mutations in the examined genes, were detected in 36 % of breast carcinomas. PIK3CA p.M1043I mutation as well as PTEN p.G132Vand p.Q214* mutations are detected for the first time in breast cancer. Our findings along with previous studies underline the importance of PI3K/AKT pathway components as potential biomarkers for breast carcinogenesis.

**PS-23-002**

**Differential expression of miR-21 and PDCD4 in breast lesions**

B. Schnack Nielsen ^*^, L. Christensen, T. Møller, K. Holmstrøm

^*^Bioneer A/S, Molecular Histology, Hoersholm, Denmark

**Objective:** To establish multiplex assays that help characterization of molecular pathways in cancer biology, here the control of protein biosynthesis by microRNA (miR). MiRs target complementary sequences in the 3′UTR of mRNAs to control translation. MiR-21 reportedly targets and down-regulates programmed cell death 4 (PDCD4), which is seen in the nuclei of proliferating cancer cells.

**Method:** A multiplex fluorescence assay (DAPI, FITC, Cy3 and Cy5) that combines immunohistochemistry with miR in situ hybridization. Seven high-grade CIS/early invasive cancers were examined.

**Results:** We noted that miR-21 (generally stromal) also can be expressed in cancer cells of high-grade breast tumors, allowing evaluation of potential differential expressions of PDCD4 and miR-21. Two cases showed miR-21 expression in tumor cells, one confined to cell clusters and one within all tumor cells. PDCD4 was prevalent in cytokeratin-positive malignant and benign epithelial cells, but in the case, where all cancer cells were miR-21-positive, these were negative for PDCD4, and in the case, where only focal clusters of malignant epithelial cells were miR-21-positive these were often weakly stained or negative for PDCD4. The other 5 tumors showed PDCD4-positive tumor and miR-21-positive stromal cells.

**Conclusion:** Our findings illustrate that multiplex assays allow confirmatory evaluation of expression of miRs and their target protein in vivo.

**PS-23-003**

**Assessment of HER2 status in breast tumours using droplet digital PCR**

S. Di Palma ^*^, B. Ping, N. Collins

^*^Royal Surrey County Hospital, Dept. of Histology, Guildford, United Kingdom

**Objective:** Droplet digital PCR (ddPCR) is a new technique that accurately quantifies the number of copies of a gene.

**Method:** We evaluated this technology for assessment of the HER2 gene in breast tumour samples. We compared it with our routine single probe silver in situ hybridisation (SISH) assay in which only equivocal cases require chromosome 17 (C17) assessment and calculation of HER2:C17 ratio.

**Results:** Forty five samples generated a result; 44 results were concordant with previous SISH results (97.8 %). One sample was previously reported as HER2 not-amplified by SISH but found to be ddPCR amplified. Re-evaluation of the SISH slide showed there was concordance between ddPCR and HER2 copy number but the HER2:C17 ratio had classified the sample as ‘not-amplified’.

**Conclusion:** This preliminary data indicates good concordance between ddPCR and single probe SISH analysis. Numerical cut-off points must be established, samples falling into an ‘equivocal’ category could be reflexed to SISH. This approach corresponds well with the movement towards HER2 evaluation in terms of absolute copy number rather than a ratio. The lack of histological information is a disadvantage compared with SISH, however, the significant reduction in reagent costs and staff time along with this encouraging early data warrant further investigation of this technology.

**PS-23-004**

**Correlation between PARP-1 immunoreactivity and cytomorphological features of parthanatos: Specific cellular death in breast cancer cells**

P. Donizy ^*^, A. Halon, P. Surowiak, C. Kozyra, R. Matkowski

^*^Wroclaw Medical University, Dept. of Pathomorphology, Poland

**Objective:** Parthanatos is a PARP-1-mediated cell death which is different from other types of cellular deaths, such as apoptosis and necrosis. In the parthanatos dissipation of mitochondrial membrane potential, large DNA fragmentation and chromatin condensation were observed. In contrast to apoptosis, it does not cause apoptotic bodies formation. Although PARP-1-mediated cell death presents loss of membrane integrity similar to necrosis, it does not induce cell swelling. The purpose of the study was to correlate the immunohistochemical parameters of PARP-1 reactivity and the selected cytomorphological features of parthanatos: presence of apoptotic bodies and necrosis in breast cancer (BC) specimens.

**Method:** Immunohistochemistry (IHC) for PARP-1 was performed on 83 paraffin-embedded BC specimens. Correlations between parameters of PARP-1 expression and sub-cellular localization and presence of apoptotic bodies and necrosis were evaluated.

**Results:** High expression of PARP-1 (IRS≥6) was associated with lack of apoptotic bodies (*P* = 0.011) and with absence of necrosis (*P* = 0.001). Moreover, presence of apoptotic bodies was correlated with re-distribution of PARP-1 from nucleus to cytoplasm in BC cells (*P* = 0.034). Additionally, it was observed a tendency between necrosis and loss of nuclear PARP-1 expression (*P* = 0.053).

**Conclusion:** Our study suggests that PARP-1 may play a crucial role in induction and regulation of specific type of cellular death–parthanatos.

**PS-23-005**

**Paraoxonase 1 and graft vascular disease of transplanted myocardium**

V. Repiska ^*^, I. Varga, E. Gonçalvesova, H. Gbelcova, D. Bohmer, L. Danihel, L. Halcak, P. Sykora

^*^Comenius University, Faculty of Medicine, Dept. of Medical Biolology, Bratislava, Slovakia

**Objective:** Coronary vasculopathy is obliterating affection of coronary arteries of transplantate myocardium and at present it is great effort to define significant parameters correlating with its early stage. Paraoxonase 1 (PON1) is an esterase affecting the development of atherosclerosis and imunological reaction of organism. There were described SNP polymorphisms (codons 55(L/M) and 192(Q/R)) affecting its enzymatic activity. Aim of study was to compare PON1 activity with respect to DNA polymorphisms depending on incidence of atherosclerotic changes on coronary arteries in patients after heart transplantant.

**Method:** It was collected 120 samples of blood from patients after heart transplant (20 samples of all were from patients with atherosclerotic changes on coronary arteries). PCR-RFLP analysis was used to detect DNA polymorphisms. All samples were divided in 3 subsets–“strong genotype”(RR/LL, QR/LL), „middle genotype“(QR/LM, QR/MM, QQ/LL, QQ/LM) and „weak genotype“(QQ/MM). PON1 paraoxonase and arylesterase activities were detected spectrophotometrically.

**Results:** We found out no significant differences in paraoxonase and arylesterase activities in patients with coronary vasculopathy.

**Conclusion:** PON1 has probably no patophysiologic relationship with incidence or severity of atherosclerotic changes on coronary arteries of transplantantate myocardium. Supported by the grant VEGA 1/0186/11 and grant CZ.1.07/2.3.00/30.0060.

**PS-23-006**

**BRAF mutation in desmoplastic melanoma**

I. Cunha ^*^, M. Macedo, M. Silva, J. Duprat, B. Lisboa, M. D. Begnami, L. D. Brot Andrade, F. Mello, C. Pinto, D. M. Carraro, F. Soares

^*^Hospital A.C. Camargo, Dept. of Pathology, São Paulo, Brazil

**Objective:** Desmoplastic melanoma (DM) is rare. Data is scarce concerning DM molecular findings. We report a single institution casuistic of DM with clinical and molecular aspects.

**Method:** Patients were from A.C. Camargo Cancer Hospital from years 2003–2012. BRAF mutation was tested both by pyrosequencing (PyromarkQ24) and direct sequencing (ABI3500) from paraffin embedded tissue DNA sample.

**Results:** Nine patients with DM were retrieved, eight males and one female. Sites were scalp (5), upper extremities (2) and trunk (2). Age range 42–82 years, with a 67 years mean. 1 patient died 6 years after diagnosis, 7 are alive with a 5 years follow up mean. Seven patients had BRAF analysis. None showed a V600E mutation. One showed a p.D594N (c.1780G>A), a 82 years old men, with a scapular lesion, negative node and metastasis to the back region skin. This patient is alive with no disease after 2 year.

**Conclusion:** DM is found in elderly, mostly in scalp, with a good overall outcome. None of the cases showed a V600E BRAF mutation, a data consistent with the scarce literature. We found mutation in another region of the gene (D594N). Rare reported cases showed this mutation in melanoma, colorectal and hematological neoplasm. This is the first report in DM.

**PS-23-007**

**Testing for KRAS and BRAF in colorectal cancer? A comparison of three methods**

A. Oniscu*, J. Fairley, K. Walsh, S. Camus, D. Stirling

^*^Royal Infirmary of Edinburgh, Pathology, United Kingdom

**Objective:** The widespread use of targeted therapies has generated the need for accurate and cost-effective methods of mutation detection. Several platforms are currently being used in molecular pathology services for KRAS and BRAF testing in colorectal cancer. Our centre offers KRAS and BRAF testing using pyrosequencing. We wished to investigate the utility of two alternative testing platforms.

**Method:** 64 colorectal adenocarcinomas, formalin-fixed paraffin-embedded, were analysed for KRAS codon 12 13 and 61 mutations and BRAF V600E mutations using pyrosequencing. The results were compared to those of two new platforms: the COBAS 4800 System (Roche Diagnostics), which is real-time PCR based, and the Randox (Evidence Investigator)which uses multiplex biochip technology.

**Results:** Similar KRAS mutation results were obtained for 63 tumours when using pyrosequencing and COBAS (98.4 % concordance rate). However, a false positive result (p. Gly60Gly silent mutation), confirmed by sequencing was obtained by COBAS. False positive and false negative KRAS results were obtained when comparing Randox with pyrosequencing (89 % concordance rate). There was 100 % concordance for BRAF testing using all three platforms.

**Conclusion:** Our results show good concordance for KRAS and BRAF testing using these three methods. However, false positive or negative KRAS results should not be underestimated due to treatment implications. Pyrosequencing is a reliable and cost-effective method to test for KRAS and BRAF mutations.

**PS-23-008**

**HER2 and HSP90 overexpression in respect to KRAS gene status in colorectal cancer**

J. Bar ^*^, A. Lis-Nawara, A. Lebioda, A. Jonkisz, P. Grelewski, P. Gajdzis, T. Dobosz, M. Jelen

^*^Medical University Wroclaw, Dept. of Pathomorphology, Poland

**Objective:** The personalized target therapy based on the molecular characterization of malignant tumor might be needed for more effective treatment strategies for patients with metastatic colorectal cancer.

**Method:** Fifty three paraffin-embedded colorectal cancer specimens were analyzed for KRAS mutation, HSP90 expression and HER2 overexpression/amplification. A high-resolution melting (HRM) assay and single-nucleotide polymorphisms (SNPs) were used to detect somatic mutation in exon 2 notably codons 12 and 13 of the KRAS gene. The HSP90, HER2 proteins expression was examined using immunohistochemistry. HER2 overexpression was confirmed by fluorescence in situ hybridization (FISH).

**Results:** KRAS mutations were identified in 19/53 (35.8 %) of colorectal cancers. KRAS mutation was independent of tumor grade. HER2 was observed in 66.0 % and HSP90 in 72.2 % of colorectal cancers and associated with moderate tumor grade. HSP90 expression was associated with sex and observed mainly in women (*p* = 0.01). HER2 and HSP90 immunoreactivity was independent of KRAS gene status. HER2 overexpression was accompanied by amplification of HER2 gene. The association between the level of HSP90 and HER2 protein overexpression was revealed (*p* = 0.001). The KRAS/HER2/HSP90 immunophenotype was associated with moderately differentiated colorectal cancers.

**Conclusion:** Our results indicate that HSP90/HER2-targeted therapies might be considered in KRAS positive/negative colorectal cancers

**PS-23-009**

**Angiogenic factors profiling of lymph node negative and distant metastasis positive colorectal cancers**

N. Jákob ^*^

^*^Budapest, Hungary

**Objective:** In the past decades, colorectal cancer became one of the main foci of cancer research, and colorectal cancer is one of the most serious problems of the public health.

**Method:** In our study, angiogenic proteome profiler array was carried out to investigate the expression of lymph-node metastasis negative and distant metastasis positive colon cancer tissues compared to the adjacent non-cancerous mucosa from surgical resections, in order to improve our understanding of the genetic mechanism of angiogenesis in human colorectal cancer and to identify new potential tumor markers useful for clinical practice. We validated our results on an expanded cluster of patients with tissue microarray. After the linearization of the data we can use the integrated density values for the appropriate exposition time based on the intensity.

**Results:** During the comparison of the proteins of the tumor (*n* = 8) and the corresponding normal mucosa (*n* = 8) we found significant differences in the following proteins: Serpin E1, uPA, VEGF, MMP-8, MMP-9 and endoglin.

**Conclusion:** We found that only the serpin B5 (maspin) correlated with the stage of colon cancer. Our data gives new insights into the angiogenic mechanisms in colorectal cancer.

**PS-23-010**

**KRAS and BRAF mutational status in colon cancer from Albanian patients**

L. Memeo^*^, R. Costanzo, D. Martinetti, S. Kadare, M. Alimehmeti, C. Colarossi, V. Canzonieri

^*^Mediterranean Inst. of Oncology, Dept. of Experiemenal Pathology, Catania, Italy

**Objective:** Many studies have indicated that KRAS mutations occur in about 30–35 % of colorectal cancer (CRC), while BRAF mutations in 5–10 % of CRC and that KRAS and BRAF mutations are mutually exclusive. No data are present in the literature about the percentage of mutations in Albanian patients.

**Method:** We have evaluated KRAS and BRAF status in 159 CRC samples obtained from a collaboration with the University of Tirana. Mutational status was evaluated by direct sequencing. 90 patients were male (57 %) and 69 female (43 %); the patients’ ages ranged from 17 to 85 (median 61.7). 24 patient were stage I, 36 stage II, 84 stage III and 15 stage IV.

**Results:** Out of the 159 cases, 27 (17 %) showed KRAS mutation (13 G12D, 4 G12C, 4 G12V, 3 G12A, 2 G13 D and 1 G12S), and 10 (6,3 %) showed BRAF mutation (all V600E).

**Conclusion:** This is the first report of KRAS and BRAF status in Albanian patients with CRC and though the relatively small sample size might not provide enough statistics power, the reported incidence of KRAS mutation is slightly less frequent in Albania when compared with the European data. The percentage of BRAF mutation seems in line with the data present in the literature.

**PS-23-011**

**-509C>T SNP in TGF-beta gene in the development and prognosis of CRC**

M. Gulubova ^*^, E. Aleksandrova, J. Ananiev, T. Vlaykova

^*^Trakia University, Faculty of Medicine, Dept. Gen. and Clin. Pathology, Stara Zagora, Bulgaria

**Objective:** TGF-b is a multifunctional cytokine that suppresses the recruitment and proliferation of dendritic cells in tumor microenvironment, which are involved in the development and progression of colorectal cancer (CRC). Growing evidence suggests that the commonly studied -509C>T polymorphism in the promoter region of the TGF-b gene has been associated with the transcription activity of the gene, but its role in development and progression of CRC remains controversial.

**Method:** In our current case–control study 114 patients with CRC and 176 non- affected controls were enrolled. The genotyping was performed by PCR-RFLP.

**Results:** There was a statistically significant difference in the genotype and allele frequencies between cases and controls (*p* = 0.046, *p* = 0.031). The carriers of TT genotype appeared to have 2.74-fold lower risk for CRC compared to CC genotype. The genotype frequency was associated to the survival of the patients: median survival of TT genotype–14.4 month.; CT–24.4 month, and CC–54.0 month (*p* = 0.049). The variant -509T allele tended to correlate with lower number of CD1a+ dendritic cells in tumor stroma.

**Conclusion:** The results of our study suggest that -509T allele associated with higher transcription of TGF-b gene may play a role as a protective factor for CRC and might determine a worse prognosis.

**PS-23-012**

**Evaluation of KIT and PDGFRA mutations in Gastrointestinal Stromal Tumors (GIST)**

D. Barcelos ^*^, R. Artigiani, K. Funabashi, A. Comodo, G. Landman, R. Stilhano, S. Han, E. Iwamura

^*^UNIFESP, Dept. of Pathology, Guarulhos, Brazil

**Objective:** To evaluate the KIT exon 11 and PDGFRA exon 18 mutations in the samples of formalin-fixed and paraffin embedded (FFPE) in the Brazilian population sample.

**Method:** For carrying out this study were used samples FFPE of stomach GIST tumors (*n* = 22). The analysis were performed after the DNA extraction, using QIAamp DNA Mini Kit, the PCR by appropriate primers and using Big Dye Terminator® Kit in the ABI 3100 Genetic Analyzer (Applied Biosystems).

**Results:** The KIT exon 11 has revealed difficulties, especially in the amplification procedure, while only 6/22 (27 %) were successfully sequenced. Only one out of 6 samples which was sequenced had only one mutation (deletion at codon 579). The sequencing of exon 18 of PDGFRA was possible in 59 % (13/22) of samples. We found no mutations that alter the amino acid sequence, however we observed in 6/13 (46 %) samples and mutations of the type Val824Val and 7/13 (54 %) no mutation in this exon.

**Conclusion:** The sequence analysis performed in FFPE materials have required standardization of all procedures from extraction to the final ones of the process and however, such material can have factors that affect the results.

**PS-23-013**

**Molecular markers analysis for hereditary nonpolyposis colorectal cancer in Belarusian population**

M. Vozmitel ^*^, J. Guljaeva, R. Smolyakova

^*^Minsk, Belarus

**Objective:** Hereditary non-polyposis colon cancer (HNPCC/Lynch syndrome) is one of the most common cancer predisposition syndromes representing 3–5 % of all colon cancer. HNPCC lacks strict clinical features, so diagnosis is based on the family history, genetic testing for mutations of one of the mismatch repair genes. There are 5 genes associated with HNPCC: MLH1, MSH2, MSH6, PMS2, EPCAM. MLH1 and MSH2 alterations account for the majority of Lynch Syndrome families.

**Method:** 53 patients with family history of HNPCC and fulfilling the Amsterdam criteria. Microsatellite instability (MSI) was assessed at eight loci (BAT25, BAT26, D2S123, D5S346, D17S250, NR-21, NR-24 and NR-27) from paraffin embedded specimens and blood of patients on ABI PRIZM 3130. Immunohistochemistry (IHC) for MLH1 and MSH2 proteins were performed on paraffin embedded tissue.

**Results:** High level MSI (MSI-H) was detected in 13 (24.5 %) patients; low level of MSI (MSI-L) was detected in 7 (13.2 %) patients. Among the 24 MSI-negative tumors 22 showed an intact protein expression.

**Conclusion:** The definition of MSI has a high diagnostic efficacy in detecting predisposition of HNPCC. IHC offers an alternative method for assessment of MSI.

**PS-23-014**

**PI3K/AKT/mTOR activation in esophageal carcinomas in Greek population**

A. Saetta ^*^, K. E. Tasioudi, S. Sakellariou, G. Levidou, N. V. Michalopoulos, P. Kontogianni, D. Theodorou, P. Korkolopoulou, E. Patsouris

^*^University of Athens, 1st Dept. of Pathology, Greece

**Objective:** To perform PIK3CA, AKT-1 mutational analysis in relation with pAKT, pmTOR, p-p70S6K and p4EBP1 protein expression in ECs from Greek patients.

**Method:** 44 specimens (30 adenocarcinomas, 12 squamous cell carcinomas and 2 adeno-squamous cell carcinomas) were analysed for mutations (PIK3CA exons 9,20/AKT-1 exon4) by HRM/Sanger or Pyro-sequencing. p-AKT, p-mTOR, p-p70S6K and p-4EBP1 expression was assessed immunohistochemically (29 cases).

**Results:** No PIK3CA, AKT-1 mutations were found. 97.7 % of cases coexpressed nuclear p-mTOR along with p-p70S6K and p-4EBP1 and 90 % of them with p-AKT, showing activation of p-AKT/mTOR pathway in the majority of the examined esophageal cancers. Average cytoplasmic p-mTOR positivity was correlated with tumor stage (*p* = 0.05), marginally with grade and lymph node metastasis, also when the adenocarcinomas subgroup was considered separately in which pAKT cytoplasmic expression was negatively correlated (marginally) with tumor stage (*p* = 0.0526). p-4EBP1 immunoreactivity was negatively correlated with tumor histological grade (*p* = 0.0427).

**Conclusion:** We observed activated AKT, mTOR, p70S6K and 4EBP1 despite the absence of PIK3CA, AKT-1 mutations. Our findings support the hypothesis that mTOR activation may be a rather late event, contributing to the acquisition of a more aggressive phenotype, whereas AKT and 4EBP1 activation appears more crucial during early stages in esophageal adenocarcinomas.

**PS-23-015**

**Tumour suppressor gene PTEN in normal endometrium and endometrial carcinoma**

V. Repiska ^*^, Z. Lasabova, V. Sisovsky, L. Straka, M. Telkova, B. Rychly, H. Gbelcova, P. Bakes, L. Danihel

^*^Comenius University, Faculty of Medicine, Dept. of Medical Biolology, Bratislava, Slovakia

**Objective:** Endometrial carcinoma (ECa) is the most common neoplasia of the female genital system. There are two basic types of ECa, endometrioid (estrogen related, indolent behavior) and non-endometrioid (unrelated to estrogen, aggressive). The phosphatase and tensin (pten) homolog is a tumor suppressor gene (10p23) encoding a phosphatase, 47 kDa. The primary target is the phosphatidylinositol 3,4,5-triphosphate (PIP3) that is involved in a signal transduction pathway that regulates cell growth, migration and apoptosis. The study evaluates an association between the morphological appearance of normal endometrium and ECa, and the degree of pten alterations.

**Method:** A total of 40 archived paraffin-embedded biopsy tissue specimens with normal proliferative endometrium, endometrioid Ca G1 and G3 and serous (SC) subtype of ECa were evaluated by molecular biology methods for the exons 1–9 pten DNA sequence alterations in nuclei of endometrial epithelial cells.

**Results:** The pten mutations (insertions/deletions/substitutions) were related to all types of ECa tested. Some specimens harbored more then one mutation.

**Conclusion:** The presence of certain pten mutations is associated with individual type of ECa. Evaluation of pten in ECa by molecular biology method could be relevant component useful in biomedical research as well as in clinical practice. Supported by the grant 2007/28-UK-05-MZSR.

**PS-23-016**

**Spheroid structures in HPV-associated cervix uteri pathology progression**

E. Kogan ^*^, N. Fayzullina, T. Demura, G. Sukhikh

^*^RCOGP, Dept. of Pathology, Moscow, Russia

**Objective:** Cellular spheroid structures (CSS) play an important role in repair processes and tumorogenesis and are described in stem cell cultures in vitro and in breast and colon cancer in vivo. We found OCT4 and p16 positive CSS in CIN 1–3 (Kogan E. et al., 2012). The goal of the study was to analyse CSS morphology in HPV-associated cervical pathology with characteristic of cell immunophenotype and HPV DNA localization.

**Method:** Biopsy material (paraffine sections) from 28 women with chronic cervetites (7 patients), CIN 1 (7), CIN 2–3 (14) was investigated. Immunohistochemical detection of OCT4, CD34, Vimentin, CD44, E-cadherin,p16, Ki67, CK7, SMA and in situ hibridization analyses of HPV DNA (16, 18, 33, 54, 56 types) were performed.

**Results:** It was found that CSS are formed in basal-parabasal layers of squamous epithelium and adjacent myofibroblasts transitional zone. HPV DNA was detected in epithelial and endothelial cells of CSS.

**Conclusion:** There are two structural and functional types of CSS–repair CSS and tumor CSS, that differ by capillary content and oncomarkers expression. OCT4 and Vimentin positive cells localize in peripheral regions in colonies of CSS.

**PS-23-017**

**Immunophenotype of the neoplastic spheroids in liquid-based cytology of cervical lesions**

E. Kogan*, N. Fayzullina, T. Demura

^*^RCOGP, Dept. of Pathology, Moscow, Russia

**Objective:** Сell spheroid structures previously have been described in stem cell and tumour cultures, as well as in primary tumour tissues of breast and colon carcinomas. We investigated the structural features of the spheroids in histological preparations CIN II-CIN III. (Kogan E. at al. 2012). The aim of our study was to investigate the immunophenotype of tumor spheroids in cervical liquid-based cytology.

**Method:** Samples from 21 patients with L-SIL (7 women) and H-SIL (14) were analyzed by immunocytochemical method to detect expression of p16INK4a, Ki-67 (CINtec PLUS Cytology Kit, Roche), Oct4 (Spring Bioscience,USA).

**Results:** Cell sheroids were detected in PAP smears by typical morphology and Oct4 due to positive staining of this marker. Positive p16INK4a and Ki-67 cells were found in 20–25 % of epithelial cells in L-SIL spheroids and in 90–95 %–H-SIL spheroids.

**Conclusion:** Obtained data prove neoplastic nature of spheroid structures in L-SIL and H-SIL. Oct-4, p16INK4a and Ki-67 immunocytochemical analysis of cell spheroid structures in liquid-based cytology may be used in the diagnostics of cervix uteri lesions.

**PS-23-018**

**Localization of high-risk HPV DNA in CIN I–CIN III by In situ Hybridization (ISH)**

N. Fayzullina ^*^, E. Kogan, T. Demura, A. Kozachenko

^*^Research Center of Obstetrics, Dept. of Pathology, Moscow, Russia

**Objective:** High-risk human papilloma viruses (HR-HPV) are the main etiological factor in development of cervical cancer. The aim of this study was to determine the localization of HR-HPV DNA in CIN I–CIN III by ISH. ISH combines detection of HPV DNA with morphology, thus permitting the localization of HPV in specific cells and tissues.

**Method:** 12 patients with CIN I, 10–CIN II, 17–CIN III were included in the study. The knife cone biopsies of the cervix were in cases with CIN II–III. ISH was performed on paraffin sections using the ZytoFast PLUS (ZytoVision, German) to detect high-risk (HR)-HPV (types 16, 18, 31, 33, 35) according to manufacturer protocol.

**Results:** In cases with CIN I–III epithelial cells of the basal, parabasal and superficial layers of epithelium had HPV DNA. In cases with CIN II–III HPV DNA was detected also in the cells of the glandular epithelium, endothelium, subbasal myofibroblasts and cells of inflammatory infiltrate.

**Conclusion:** HR-HPV affect to niche of stem cells in cervix uterus including myofibroblasts and epithelial precursors and cannot be considered as a local infection.

**PS-23-022**

**Detection of the EBV-encoded Bart-6-3p in sera from endemic Burkitt lymphoma patients and malaria-exposed healthy controls: A possible biomarker?**

G. De Falco ^*^, S. Gazaneo, L. Mundo, J. Volule, L. Leoncini, A. Moormann

^*^University of Siena, Dept. of Medical Biotechnology, Italy

**Objective:** Burkitt lymphoma is associated with oncogenic Epstein-Barr virus (EBV) and Plasmodium falciparum malaria co-infections. We have recently performed microRNA profiling and characterized viral Bart6-3p, which is highly expressed by eBL tumors. In this study we compared eBL cases to malaria-endemicity matched controls (healthy children) from western Kenya and correlate Bart6 levels with EBV load to evaluate its utility as a risk biomarker for eBL.

**Method:** microRNAs were extracted from serum and viral Bart6-3p was quantified by qRT-PCR. The proportion of samples containing Bart6-3p and its level for eBL was compared to healthy controls.

**Results:** We found viral Bart6-3p in both eBL patients and healthy malaria-exposed children who are at risk for developing eBL. In the healthy controls, Bart6-3p was more likely to be positive when the number of EBV copies was below the threshold limit of detection by conventional qPCR. In eBL patients, Bart6-3p was positive prior to induction of chemotherapy but became negative during remission (6 month post-diagnosis).

**Conclusion:** A readily measured biomarker for eBL risk would greatly facilitate interventions aimed at prevention. Viral Bart6-3p might serve as a predictive biomarker that correlates with treatment response and become a target for therapy aimed at improving outcomes after diagnosis of eBL.

**PS-23-023**

**EBV-Bart-6-3p induces cell proliferation and escape of immunosurveillance in BL cell lines and primary tumours**

G. De Falco ^*^, M. Navari, M. R. Ambrosio, L. Di Lisio, A. Onnis, N. Martinez, E. A. Leon, C. Bellan, M. A. Piris, L. Leoncini

^*^University of Siena, Dept. of Medical Biotechnology, Italy

**Objective:** More than 90 % of endemic Burkitt lymphoma carry latent Epstein-Barr Virus (EBV), though still no satisfactory explanation of how EBV participates in its pathogenesis is provided. The aim of this study was to compare EBV-pos and EBV-neg BL cases for miRNA expression profile, and to assess the role of viral miRNAs in Burkitt lymphomagenesis.

**Method:** microRNA profiling identified viral miRNAs expressed in EBV-pos cases. Target genes of viral miRNAs were searched and classified according to the Gene Ontology (GO), revealing their involvement in cell cycle, proliferation, signal transduction, apoptosis and differentiation.

**Results:** We focused on viral BART6-3p and on two predicted target genes, PTEN and IL6ST (gp130). The role of the former as a tumor suppressor is widely assessed, whereas IL6ST is the receptor beta of the Interleukin 6 family of cytokines. Ectopic expression of a synthetic miRNA determined a strong reduction of the expression level of both PTEN and gp130, whereas inhibition of BART6-3p resulted in marked upregulation of both genes, suggesting that BART6-3p is able to regulate the expression of PTEN and gp130.

**Conclusion:** Our results identified a subset of viral miRNAs expressed in EBV-pos BL cases, which may affect the function of key cellular pathways, through dysregulation of cellular genes.

**PS-23-024**

**Importance of c-MET pathway as a predictor of cetuximab-based therapy outcome in head and neck recurrent or metastatic squamous cell carcinoma**

S. Zazo ^*^, C. Chamizo, N. Perez-Gonzalez, C. L. Auz, L. Daoud, C. Medina, E. Gavin, N. Carvajal, J. Madoz, J. García-Foncillas, F. Rojo

^*^Fundacion Jimenez Diaz, Pathology, Madrid, Spain

**Objective:** c-MET, which promotes invasion and metastasis, is activated as a compensatory pathway in the presence of EGFR blockade, resulting in resistance to EGFR-inhibitors in lung and colorectal carcinoma. We investigated the impact on cetuximab sensitivity of HGF, c-MET, p-c-MET and c-MET gene status in metastatic squamous cell carcinoma of head and neck (HNSCC).

**Method:** 50 HNSCC patients were assayed for HGF, c-MET by immunohistochemistry and c-MET gene by SISH. Overexpression were defined by ROC curves and amplification was defined by >2 copies in at least 70 % of tumor.

**Results:** c-MET overexpression was detected in 26patients, c-MET amplification in 15 and p-c-MET in 12. Amplification was associated with c-MET overexpression (*p* = 0.004). HGF overexpression was observed in 8(12 %) associated with c-MET phosphorylation (*p* = 0.001). c-MET overexpressing patients showed worse outcome for progression-free(*p* = 0.002) and overall-survival(*p* = 0.045); p-c-MET was correlated with worse PFS (*p* = 0.014) and OS (*p* < 0.001). In multivariate logistic regression analysis, p-c-MET was an independent prognostic factor for PFS(HR 6.5; 95 % CI 1.5–8.9).

**Conclusion:** HGF/c-MET pathway correlated with worse outcome in cetuximab-based treated HNSCC patients, acting as a resistance for EGFR inhibitors and blocking HGF/c-MET and EGFR.

**PS-23-025**

**06-methylguanine-DNA-methyltransferase gene promoter region methylation pattern and its effect on survival, recurrence and chemosensitivity in laryngeal cancer**

O. Onerci Celebi^*^, E. Sener, S. Hosal, I. H. Gullu, M. Cengiz, A. B. Sözeri, G. Guler Tezel

^*^Hacettepe University, Otolaryngology, Ankara, Turkey

**Objective:** The aim of his study was to examine the methylation pattern of the promoter region of the MGMT (06-methylguanine-DNA-methyltransferase) gene and determine whether there is a relationship between the methylation pattern of MGMT and tumor stage, survival, recurrence and chemosensitivity in patients with laryngeal cancer.

**Method:** Paraffine blocks of 69 primary laryngeal cancer patients who received alkyllating chemotherapy for treatment were retrieved. Blocks were selected from diagnostic laryngeal specimens before the patients received any therapy. DNA was extracted and submitted to bisulfite treatment followed by pyrosequencing. Pyrograms were analyzed.

**Results:** 85 % of the patients were found to be methylated according to pyrosequencing. MGMT methylation pattern was not found to be associated with survival, chemosensitivity, recurrence, T stage, N stage, Stage or histopathological diferentiation.

**Conclusion:** This study showed that promoter methylation is a frequent event in laryngeal cancer, but methylation patern was not found to be associated with the studied clinical and pathological parameters. Additional studies are needed to support these results and to determine the relationship between MGMT promoter region methylation and survival and recurrence.

**PS-23-026**

**Extra-brain expression of tau-protein: New possibilities for life-time diagnosis of Alzheimer’s disease**

I. Kvetnoy ^*^, M. Paltsev, T. Kvetnaia, V. Polyakova, S. Konovalov, G. Gurko, S. Mursalov

^*^Ott Institute of Obst. Gynecology, Dept. of Pathology, St. Petersburg, Russia

**Objective:** Taking into account the hypothesis that Alzheimer’s disease (AD) might be a systemic disease that affects several tissues in the body, the aim of this study was to try to detect the expression of phosphorylated tau-protein in human peripheral blood lymphocytes (PBL) as well as in buccal epithelium (BE) in patients with AD.

**Method:** Blood samples and smears of BE were obtained from patients with AD and from volunteers without psychoneurological pathology of same age. PBL were isolated on Ficoll-Paque gradient centrifugation. Immunocytochemical detection of tau-protein was carried out using tau monoclonal antibody (1:100, clone TAU-2, ICN) and universal immunostaining kit IMMU-MARK (ICN).

**Results:** The expression of tau-protein was shown in PBL and in the smears of BE in absolute majority of AD patients studied. Only in five healthy volunteers a single lymphocytes and BE cells demonstrated a very weak-positive immunostaining to tau-protein.

**Conclusion:** This first demonstration of clear difference in expression of tau-protein in blood lymphocytes and BE cells between healthy and sick people testifies to the fact that tau-protein could be considered as a promising marker and PBL as well as BE cells as suitable samples for life-time diagnosis of AD.

**PS-23-027**

**Molecular analysis of IDH1/2 mutations and MGMT promoter methylation in gliomas and meningiomas**

A. Saetta ^*^, A. Gioti, I. Chatziandreou, P. Tsioli, R. F. Soldatos, S. Sakellariou, V. Samaras, A. Sepsa, A. Zisakis, P. Korkolopoulou, E. Patsouris

^*^University of Athens, 1st Dept. of Pathology, Greece

**Objective:** IDH mutations induce hypermethylation of CpG islands through inhibition of demethylases. MGMT promoter methylation is a proposed as a favorable 3prognostic marker predictive for temolozomide response in glioblastoma. We searched for IDH1/IDH2 mutations in gliomas and meningiomas simultaneously with the presence of MGMT promoter methylation.

**Method:** MGMT promoter methylation, and IDH1, IDH2 mutations were examined in 34 gliomas and 22 meningiomas by MS-HRMA, sequencing and Pyrosequencing

**Results:** Grade II, III and IV gliomas with absence and low levels of MGMT promoter methylation displayed IDH1 and IDH2 wild type.75 % of grade II/III gliomas with high MGMT methylation carried the R132H mutation, while one case was also IDH2 mutant. Three grade IV gliomas with high MGMT methylation were IDH2 wild type, while one (25 %) harbored an R132H IDH1 mutation. All grade II meningiomas with unmethylated MGMT promoter had normal IDH1 and IDH2. On the contrary 1/4 and 2/4 of grade II meningiomas with heterogeneous MGMT methylation harbored mutations in IDH1 and IDH2 respectively. Two grade III meningiomas showed low MGMT methylation, normal IDH2 and IDH1 mutations in one case.

**Conclusion:** IDH1 and IDH2 mutations were observed in gradeII/III gliomas displaying high MGMT methylation and heterogeneous methylated meningiomas, implying an early event in brain tumorigenesis

**PS-23-028**

**Genomic instability and characteristic DNA methylation pattern in chordoma**

B. Rinner ^*^

^*^Medizin. Universität Graz, Zentrum für Medizin. Forschung, Austria

**Objective:** Chordomas are rare mesenchymal tumors occurring in the midline from clivus to sacrum. Early tumor detection is extremely important as these tumors are resistant to chemotherapy and irradiation. Despite continuous research efforts surgical excision remains the main treatment option. Because of the often challenging anatomic location early detection is important to enable complete tumor resection and to reduce the high incidence of local recurrences. The study was performed on tumor samples from ten chordoma patients

**Method:** Affymetrix GeneChip Human Mapping SNP 6.0 arrays were performed. The methylation changes were done by AITCpG360 microarray and confirmed by high throughput quantitative PCR.

**Results:** We found significant genomic instability, all chordomas showed a loss of 3q26.32 (PIK 3CA) and 3q27.3 (BCL6) thus underlining the potential importance of the PI3K pathway in chordoma development. By using the AITCpG360 methylation assay we elucidated 20 genes which were hyper/hypomethylated compared to normal blood. The most promising candidates were C3, XIST, TACSTD2, FMR1, HIC1, RARB, DLEC1, KL, and RASSF1.

**Conclusion:** In summary, we have shown that chordomas are characterized by a significant genomic instability; furthermore we demonstrated a characteristic DNA methylation pattern. These findings add new insights into chordoma development, diagnosis and potential new treatment options.

**PS-23-029**

**Antiangiogenic treatment with bevacizumab in non-squamous Non-Small Cell Lung Cancer (NSCLC): The role of VEGF-A 165 family of isoforms**

F. Rojo ^*^, S. Zazo, C. Chamizo, L. Daoud, C. Medina, N. Carvajal, N. Perez-Gonzalez, C. L. Auz, J. Madoz, M. Domine, J. García- Foncillas

^*^Fundacion Jimenez, Pathology, Madrid, Spain

**Objective:** Bevacizumab is a monoclonal antibody against vascular endothelial growth factor (VEGF) that improves Time to Progression (TTP) in advanced non-squamous NSCLC with a doublet of platins, but no proven predictive markers exist. The VEGF-A165 splice variant has been described as the most abundant and active isoform in cancer. Exon 8 distal splice site modifications of VEGF165 generates the VEGF-A 165a isoform (pro-angiogenic effect), and the VEGF-A 165b isoform (antiangiogenic activity). This study aims to explore the role of VEGF165a and VEGF165b isoform expression as predictive biomarkers in non-squamous NSCLC treated with platin plus bevacizumab.

**Method:** From 22 patients, 5 received carboplatin-taxol-bevacizumab, 14 carboplatin-taxotere-bevacizumab and 3 cisplatin-gemcitabine-bevacizumab. RNA was isolated byRNeasy FFPE. VEGF165a and VEGF165b expression was analyzed by RT-qPCR. Individual VEGF165a and VEGF165b isoforms expression was calibrated to normal tissue and the ratio VEGF165a/b was calculated.

**Results:** VEGF165a overexpression was detected in 14 (63.6 %) and VEGF165b overexpression in 15 (68.2 %). Ratio VEGF165a/b was associated with TTP, correlating a predominant expression of VEGF165a with a significant benefit compared to predominant VEGF165b expression cases (15 vs. 8 months, *p* = 0.005).

**Conclusion:** VEGF165a/VEGF165b ratio >1 correlated with benefit to anti-angiogenic therapy in this cohort, supporting use as predictive biomarkers for bevacizumab in stage IV non-squamous NSCLC.

**PS-23-030**

**Pemetrexed sensitivity in advanced cancer patients: Is thymidylate synthase expression a reliable predictive biomarker?**

C. Chamizo ^*^, F. Rojo, S. Zazo, C. Medina, E. Gavin, N. Perez-Gonzalez, C. L. Auz, N. Carvajal, J. Madoz, M. Domine, J. Garcia-Foncillas

^*^Fundación Jiménez Díaz, Pathology, Madrid, Spain

**Objective:** High level of thymidylate-synthase (TS)expression in malignant tumours has been suggested to reduce sensitivity to pemetrexed, but no evidence has been demonstrated in samples from patients treated with pemetrexed. This study evaluates TS-expression in tumor cells as predictor of efficacy in patients with non-small cell lung cancer, small cell lung cancer and mesothelioma treated with pemetrexed.

**Method:** 54 patients were included in this study: 40 NSCLC, 3 SCLC and 11mesothelioma. 21 patients received platins-pemetrexed as first line NSCLC, 20pemetrexed in monotherapy as second and further lines and 3 carboplatin-pemetrexed for extensive disease SCLC. RNA was isolated by RNeasy FFPE procedure. TS-expression was analyzed by RT-qPCR and calibrated using normal tissue.

**Results:** From 54 cases, TS-expression data were available in 32, detecting overexpression in 23 (71.8 %) and low expression in 9 (28.2 %). Response rate with low expression was 0.63 vs 0.15 in patients with overexpression (*p* = 0.015). A significant benefit in time to progression was observed in patients with low expression (12 vs. 2 months, *p* = 0.002), but no impact on overall survival (20 vs. 19 months, *p* = 0.595).

**Conclusion:** TS overexpression in tumor cells is correlated with reduced response to pemetrexed-containing chemotherapy and might be used as predictive biomarker in advanced lung and mesothelioma cancer patients.

**PS-23-031**

**Combinated study of KRAS and EGFR gene copy status as superior predictive markers of response and time to progression in stage IV non-squamous Non-Small Cell Lung Cancer (NSCLC) treated with EGFR tyrosine-kinase inhibitors (TKI)**

E. Gavin ^*^, S. Zazo, C. Chamizo, L. Daoud, J. Madoz, N. Perez-Gonzalez, N. Carvajal, C. L. Auz, J. García-Foncillas, M. Dómine, F. Rojo

^*^Fundación Jiménez Díaz, Pathology, Madrid, Spain

**Objective:** KRAS mutations on codons 12, 13 and 61 may render tumor independent of EGFR and resistant to TKI therapy in NSCLC patients. We studied the associations of KRAS and EGFR copy alterations and mutations with response and time to progression (TTP) in EGFR TKI treated patients

**Method:** 84 samples from NSCLS patients with gefitinib or tarceva-containing therapy were analyzed for KRAS and EGFR mutation status. EGFR copy number was determined by FISH. Amplification was defined with ≥3 gene copies.

**Results:** KRAS mutation was detected in 15 cases, EGFR mutation in 27 and EGFR amplification in 8. Significant differences were detected in response for wild-type (0.2) and mutant-KRAS (0.0)(*p* = 0.023), wild-type (0.12) and mutant-EGFR (0.39)(*p* = 0.007), and non-amplified(0.18) and EGFR-amplified (0.71)(*p* = 0.005). Benefit from TKI was observed for KRAS-WT versus KRAS mutated (TTP 7 vs. 3 months, *p* = 0.001), EGFR-mutated versus wild-type (14 vs. 4 months, *p* = 0.004) and EGFR-amplification versus non-amplified (11 vs. 5 months, *p* = 0.001). Combined analysis of status of three biomarkers strongly predicted benefit to TKI therapy (TP 15 vs. 3 months, *p* < 0.001)

**Conclusion:** Combined analysis of KRAS mutation, EGFR mutation and amplification in EGFR TKI treated NSCLC provides superior predictive information than single study.

**PS-23-032**

**Determination of Epidermal Growth Factor Receptor (EGFR) mutation in lung adenocarcinomas using biofilm chip-based microarray technology**

Ö. C. Uzun ^*^, G. Özbilim, M. Özcan, A. Sarper

^*^Akdeniz University, Dept. of Pathology, Antalya, Turkey

**Objective:** Advanced non-small cell lung cancer (NSCLC) patients with gene encoding epidermal growth factor receptor (EGFR) mutations may respond to drugs that include EGFR tyrosine kinase inhibitors (EGFR-TKI) such as Gefitinib. The aim of this study was to determine the expression of EGFR mutation in Turkish patients and the relationship between patient’s sex, smoking habbits, stage of the tumor.

**Method:** In this study, 58 resection materials of lung adenocarcinomas were used. 44 patient were male and 14 patient were female. Tissue samples were taken from parafin-embedded blocks. EGFR mutation was detected using biofilm chip based microarray technology (Infiniti Autogenomics®).

**Results:** EGFR mutation was detected in 14 patients (%24.1), out of 58 patients studied. EGFR mutation was more pronounced in female patients with no smoking history. However, there was no correlation between EGFR mutation and the size of tumor, lymph node metastasis, distance metastasis and stage of the cancer.

**Conclusion:** Patients with advanced NSCLC have been increasingly treated with EGFR-TKI. We also observed the frequency of EGFR mutation in Turkish population. We also evaluated the reliability of the biofilm chip based microarray study in EGFR gene mutation determination. Our results were consistent with the literature.

**PS-23-035**

**Prevalence of EGFR mutations in patients with Non-Small Cell Lung Cancer (NSCLC) in Turkish population**

E. Sener ^*^, K. Kosemehmetoglu, G. Guler Tezel

^*^Hacettepe University, Dept. of Pathology, Ankara, Turkey

**Objective:** The detection of EGFR mutation is critical for management in patients with non-small cell lung cancer (NSCLC). We determined the prevelance and pattern of EGFR mutations in NSCLC in Turkish population.

**Method:** We retrospectively reviewed molecular pathology reports of 371 cases (252 male, 119 female) with lung cancer analysed for EGFR mutation in Department of Pathology, Hacettepe University. Speciemens diagnosed as adenocarcinoma (285 cases,76.8 %), non-small cell carcinoma-NOS (77 cases,20.8 %), and squamous cell carcinoma (9 cases,2.4 %) were included.

**Results:** 69 mutations (18.6 %) were found which were; deletions in exon 19 (31/69, 44.9 %), point mutations in exon 21 (27/69, 39.1 %) and exon 18 (5/69, 7.2 %), and insertions in exon 20 (4/69, 5.8 %). 2 cases showed double mutation in exon 19 and exon 20. In mutated cases, 71 % were female and 29 % were male. The distribution of mutated cases according to diagnosis were 65/285 (22.8 %) adenocarcinoma, 4/77 (18.9 %) NSCLC- NOS and 0/9 (0 %) squamous cell carcinoma. Mutations were detected in 52/261(19.9 %) of primary and 17/110 (%15.5) of metastatic pulmonary tumor samples.

**Conclusion:** Prevalence of EGFR mutation was similar to Caucasian population and lower than East Asian patients. Mutation patterns were similar to literature.

**PS-23-036**

**Morphological and molecular analysis of lung cancer biopsies fixed in RCL2**

V. Costes ^*^, J. Solassol, L. Khellaf, M. Larrieux, I. Serre

^*^Hopital Gui de Chauliac, Dept. of Biopathology, Montpellier, France

**Objective:** The treatment of lung cancer requires molecular tests on adenocarcinoma small biopsies. However, formaldehyde causes methylenic bridges between nucleic acids that render it inappropriate for high quality DNA extraction. Among alternative alcohol-based _xatives, RCL2 has been reported to highly preserve histomorphology and the integrity of nucleic acids.

**Method:** We prospectively collected lung-tumour specimens from 22 patients: one part was FFPE, the second was snap-frozen in liquid nitrogen and the last was fixed overnight at 4°C in RCL2 and paraffin-embedded (RFPE).

**Results:** Morphology quality and immunochemistry results were comparable between FFPE and RFPE. DNA extraction obtained from FFPE, RFPE and frozen tissue specimens yielded on average 1041, 495, and 192 μg/ml, respectively. All fresh frozen and RFPE specimens yielded amplicons up to and including 600 bp in length. By contrast, DNA degradation was evident in the FFPE specimens. EGFR and KRAS genotypes (respectively two and four mutated cases) were similar in RFPE and frozen specimens. Finally, ALK rearrangement was evaluated by FISH in both NBF-fixed and RCL2-fixed, paraffin-embedded H3122 cell pellets. The image qualities were identical for the both specimens without adjustment of the FISH protocol.

**Conclusion:** RCL2 has great potential for morphological and molecular analyses to be performed on the same sample.

**Assessment of DNA quality of adenocarcinoma biopsy specimens:** 
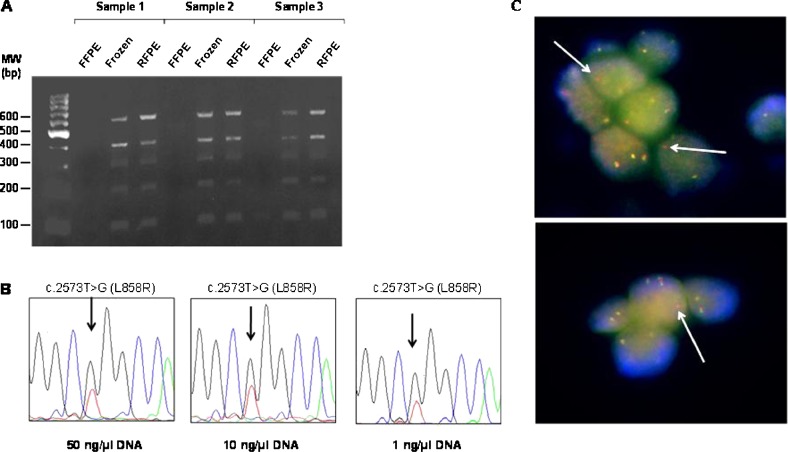


**PS-23-037**

**Epigenetic modulation of the androgen receptor gene expression**

B. Fialova ^*^, K. Smesny-Trtkova, L. Paskova, K. Langova, Z. Kolar

^*^Palacky University Olomouc, Czech Republic

**Objective:** DNA methylation and histone deacetylation are epigenetic mechanisms which can contribute to regulation of androgen receptor (AR) expression in prostate cancer (CaP). DNA methyltransferase and histone deacetylase inhibitors might be useful tools for the CaP treatment. We analysed the 5-aza-2′-deoxycytidine (Aza) and sodium butyrate (NaB) effects on androgen-insensitive human prostate cancer cell lines which do not express AR protein (PC3 and DU145).

**Method:** Cell lines were treated with Aza and NaB. PCR of bisulfite-modified DNA and RT-PCR with bisulfite-sequencing were used for AR gene analysis. Conventional PCR of immunoprecipitated DNA was applied to detection of re-acetylated histones.

**Results:** The co-treatment by Aza and NaB resulted in significant decrease of cell viability in both cell lines and increase of DNA demethylation in DU145. Site-specific demethylation of the AR gene promoter induced the gene re-expression and increased acetylation in histones H3 and H4 in DU145 cells.

**Conclusion:** AR gene promoter could be a target of the AR gene inactivation during androgen deprivation therapy of patient with CaP.

**PS-23-038**

**Immunohistochemical assessment of HDAC1 in prostate cancer: Correlation with transcript levels and ERG status**

L. Santos ^*^, J. Ramalho-Carvalho, F. Q. Vieira, J. D. Barros-Silva, P. Paulo, A. T. Martins, M. R. Teixeira, C. Jerónimo, R. Henrique

^*^Portuguese Institute of Oncology, Dept. of Pathology, Porto, Portugal

**Objective:** Histone deacetylases (HDAC) exert mostly a repressive role in transcription. In PCa, HDAC1 mRNA is overexpressed and this has been correlated with ERG+ tumors. HDAC1 may constitute a prognostic biomarker and a therapeutic target. We sought to determine the method of imunohistochemical assessment of HDAC1 expression which better correlates with mRNA levels.

**Method:** Immunohistochemistry for HDAC1 was performed in 48 PCa, previously characterized for ERG by FISH, and 8 morphologically normal prostate tissues (MNPT). Staining intensity, percentage of positive cells and H-score were assessed using a semi-automated image analysis software (GENASIS). Transcript levels were determined by quantitative RT-PCR in paired frozen-tissue samples. Results were correlated with transcript levels.

**Results:** ERG+ PCa displayed higher transcript levels compared to ERG- PCa and MNPT. Higher HDAC1 transcript levels associated with higher pT stage. Concerning HDAC1 protein expression, staining intensity, but not percentage of positive cells or H-score, was significantly higher in ERG+ tumors. No correlation was found between any of the protein expression variables and clinical parameters.

**Conclusion:** The association between ERG and HDAC1 upregulation in PCa was confirmed, although most PCa displayed HDAC1 overexpression, at transcript and protein level. For routine use, immunohistochemical assessment of HDAC1 should be based on staining intensity evaluation.

**PS-23-039**

**The dynamics of hypoxia-angiogenesis relation as reflection of evolution of tumor microenvironment**

L. Maruscakova ^*^, Z. Poljak, O. El-Hassoun, M. Kopani, J. Jakubovsky, I. Hulin

^*^Comenius University, Dept. of Pathological Physiology, Bratislava, Slovakia

**Objective:** Monitoring the dynamics of hypoxia-angiogenesis relations allow as to describe the evolution of tumor microenvironment and its importance in tumor growth.

**Method:** During experimental tumorigenesis on a rat model using BP6 fibrosarcoma cell line injected intraperitoneally we measured and analysed the levels of glucose, LDH and CD34+ cells in peritoneal lavage acquired weekly through a period of 4 weeks.

**Results:** We observed the following changes of measured parameters: Glucose level gradually increased until 3rd week, then fell down. LDH had the opposite course. The increase in CD34+ cells in 2nd week was followed by gradual decrease.

**Conclusion:** Glucose levels reflect nutrition supply, LDH levels indicate cell necrosis caused by hypoxic microenvironment, CD34+ progenitor endothelial cells reflect angiogenesis. Changes of these parameters illustrate the dynamic relation between hypoxia and angiogenesis as two characteristics of the tumor microenvironment. Hypoxia induces tumor angiogenesis, which is imperfect and leads again to hypoxia. They are in constant duel. Their antagonic effect is reflected by the opposit course of measured glucose and LDH levels. We assume that microenvironment parameters are suitable information about progression of tumours. Acknowledgements: This study was supported by the VEGA grant no. 1/1163/11.

**PS-23-040**

**First external quality assessment of molecular pathology disease-based gene panels**

J. Fairley ^*^, Z. Deans

^*^NHS Lothian, Laboratory Medicine, Edinburgh, United Kingdom

**Objective:** UK NEQAS for Molecular Pathology provided external quality assessment for EGFR in non-small cell lung cancer; KRAS in metastatic colorectal cancer; and BRAF in melanoma. To reflect more closely the workflow of testing carried out by diagnostic laboratories these have been extended to gene panel based testing schemes for lung cancer, colorectal cancer and melanoma.

**Method:** Three clinical case scenarios and FFPE tumour sections were distributed for each scheme. Each case was validated by 2 independent laboratories; lung cancer cases for EGFR, KRAS, BRAF and ALK; colorectal cancer cases for KRAS, BRAF and PI3K; and melanoma cases for BRAF, NRAS and KIT. Participants tested according to normal procedures and issued clinical interpretative reports.

**Results:** 55**–**65 laboratories participated in each scheme. Anonymised EQA results and reporting styles along with the mutations tested by different labs will be presented.

**Conclusion:** EQA schemes play an important role in the development of molecular pathology and delivery of a high quality service. With the rapid introduction of new tests, a panel based approach should help to reflect the clinical scenarios encountered by laboratories. It also acts as an educational tool for the setting up of new assays.

**PS-23-041**

**Five years of external quality assessment of molecular pathology testing indicates an improvement in the standard of testing**

Z. Deans ^*^

^*^UK NEQAS for Molecular Genetic, Laboratory Medicine, Edinburgh, United Kingdom

**Objective:** External quality assessment (EQA) has been provided by UK NEQAS for Molecular Genetics and UK NEQAS for Immunocytochemistry and In Situ Hybridisation since 2008 for a range of molecular pathology gene tests in colorectal cancer, gastrointestinal stromal tumours, melanoma and non-small cell lung cancer.

**Method:** Laboratories were required to test tumour sections according to normal protocols and report the genotyping results in the context of the clinical scenarios supplied for each case. Submissions were marked for genotyping accuracy, interpretation of the result and clerical accuracy of the report by peer assessors and feedback comments were provided to each laboratory.

**Results:** A marked improvement in the standard of molecular testing was observed with a decline in the number of genotyping errors. The standard of reports improved with clearer patient related clinical interpretation and inclusion of relevant information which helped the report to convey an unambiguous result e.g. patient identifiers, methodology, sensitivity of the test, assessment of tumour content and correct mutation nomenclature.

**Conclusion:** Participation in EQA not only provides laboratories with an external measure of their service but helps with the development of the quality of the testing and improves the reporting of the result leading to a high standard of patient care.

**PS-23-042**

**MicroRNA-17-5p promotes tumorgenesis through the repression of IGFBP-3 and enhancement of insulin like growth factor signaling**

D. Habashy ^*^, H. Mohamed El Tayebi, K. Adel Hosny, G. Esmat, A. Ihab Abdelaziz

^*^German University in Cairo, Molecular Pathology Research, Egypt

**Objective:** MiR-17-5p is a member of miR-17-92 cluster and was found to be significantly upregulated in metastatic hepatocellular carcinoma(HCC). On the other hand, Insulin-like growth factor binding protein-3(IGFBP-3)decreases the bioavailability of Insulin-like growth factor-II (IGF-II),thus decreasing cell proliferation in HCC. Therefore, reduced expression of IGFBP-3 contributes to the development of HCC. Our bioinformatics analysis revealed that IGFBP-3 is a downstream target for miR-17-5p. Therefore we aimed to study the impact of miR-17-5p on inducing carcinogenesis through targeting IGFBP-3.

**Method:** Total RNA was extracted from 23 HCC and 8 healthy liver tissues. MiR-17-5p expression was mimicked and antagonized in Huh-7 cell lines. MiR-17-5p & IGFBP-3 were quantified by Quantitative RT-PCR.

**Results:** IGFBP-3 mRNA was significantly upregulated in non-metastatic HCC tissues (*p* = 0.0189). Moreover,it showed an inverse correlation with miR-17-5p (Spearman *r* = −0.3850, *p* = 0.0697).By enhancing the expression of miR-17-5p in Huh-7 cells; IGFBP-3 mRNA expression was decreased with the recovery of its expression upon miR-17-5p antagonizing.

**Conclusion:** miR-17-5p and its downstream target IGFBP-3 are potential players in promoting cell growth through IGF-II bioavailability.

**PS-23-043**

**Cten localisation and its impact on cancer metastasis**

M. Akhlaq ^*^, S. Al-Ghamdi, M. Ilyas

^*^University of Nottingham, Dept. of Molecular Medical Sciences, United Kingdom

**Objective:** Cten is a member of the tensin gene family and we have previously shown that it acts as an oncogene in colorectal, pancreatic and lung cancer. The Cten protein is classically located at focal adhesions and has a role in regulating cell motility. However, by immunohistochemistry, we observed the presence of Cten within the nucleus of colorectal tumours and found that high nuclear expression correlated with metastasis.

**Method:** In order to investigate this further, we tested for nuclear Cten expression cell lines derived from colorectal, pancreatic and lung tumours by western blot. Cten was tagged with nuclear localisation signal (NLS) through PCR and Topo- Cloning technique. Effect on cell motility was tested through transwell and wound healing assays.

**Results:** Cten was detectable by Western blotting in the nuclear protein fraction of all three tumour types. The NLS-Cten construct when forcibly expressed in two colorectal cancer cell lines resulted in increased migration compared to the usual Cten expression vector.

**Conclusion:** We conclude that Cten may stimulate cell motility through both unlinking focal adhesions from the actin cytoskeleton and from as yet undetermined activity in the nucleus.

**PS-23-044**

**Validation of pharmacodynamic biomarkers for a phase I clinical trial with a multi-inhibitor drug directed to cMET, FGFR, and AXL pathways (S 49076)**

M. Akhlaq ^*^, S. Al-Ghamdi, M. IlyasL. Prudkin*, P. Martinez Perez, C. De Montrion, V. Cattan, M. Burbridge, A. Jacquet-Bescond, P. Nuciforo

^*^VHIO, Molecular Pathology Lab., Barcelona, Spain

**Objective:** Identification, development, and validation of pharmacodinamic (PD) biomarkers to determine effect and pathway inhibition induced by a multi-inhibitor drug directed to cMET, FGFR, and AXL pathways (S 49076).

**Method:** Sixteen antibodies and 5 probes of c-MET, FGFR and AXL pathways related PD biomarkers were screened in cell pellets by immunohistochemistry (IHC)/fluorescent in-situ hybridization (FISH). Those successfully optimized were then tested and validated on formalin-fixed and paraffin-embedded xenograft controls treated with the drug. Baseline levels of the validated biomarkers were then verified in human clinical samples.

**Results:** From the 16 antibodies initially tested on cell-pellets, 13 showed high specificity and were selected for analysis in xenografts models generated with cell lines having different cMET, FGFR, and AXL dependency levels. Amplification/Non amplification status of cMET, FGFR1, and FGFR2 was confirmed by FISH in the models. After treatment with the pan-inhibitor drug, selective targets modulations were observed detected by decrease of protein phosphorylation levels upon treatment. Analysis of archival human clinical samples confirmed the suitability of the selected targets to be used in samples from clinical trials.

**Conclusion:** We successfully developed and validated PD assays protocols by immunohistochemistry/FISH to be used in the context of a Phase I clinical trial using S 49076.

**PS-23-046**

**Improved RNA quality using cold phosphate buffered formalin and routine procession in FFPE tissue specimens**

S. Loeschke ^*^, C. Ankerlund Matthiesen, N. S. Knak Kristensen, T. Lisbeth Hansen, B. Nielsen

^*^Sygehus Sønderjylland, Patologisk Institut, Sønderborg, Denmark

**Objective:** Better RNA quality out of FFPE tissue is very much wanted. To find out the necessary routine conditions for good RNA preservation during fixation and procession.

**Method:** 20 fresh Tonsils were cut in 6 pieces. One was snap frozen, another fixed in HOPE solution at 2° Celsius and 4 in Phosphate buffered Formalin (PbF), 2 of them at room temperature and 2 at 2 °C for 24 +/− 4 h. After fixation HOPE tissue was processed and embedded following the manufacturers protocol and FFPE tissues were processed with routine conditions using either conventional or microwave assisted processing and paraffin embedding. All the different tissues were sectioned the same day and RNA was eluted and afterwards analyzed on an Agilent 2100 Bio analyzer. RNA-Integrity-Number (RIN) was calculated for each tissue specimen.

**Results:** Tissues fixed at 2° in PbF, processed routinely, showed best results (RIN 5.2 mean), fresh frozen was second best (4.6 mean), room temperature formalin conventionally third (3.9 mean) and room temperature with microwave and HOPE were least effective (3.2 for both).

**Conclusion:** Fixation in 2° cold PbF and conventional processing yields best RNA quality, as good as, or better than fresh frozen tissue after sectioning. In our hands the other methods resulted in various degrees of degradation.

**PS-23-047**

**Vacuum-based preservation of colorectal cancer specimens: What about RNA and DNA?**

P. Cervera^*^, S. El-Naderi, M. Jobar, R. Houdart, E. Tiret, J.-F. Fléjou

^*^APHP/Paris VI University, Dept. of Pathology, France

**Objective:** Preanalytical phase is a crucial step of pathological examination of tissues, especially for samples that need molecular analysis. The standard procedure comprises immediate fixation in neutral buffered formalin (NBF). An alternative based on vacuum sealing and cooling (VSC) that allows delayed formalin fixation, has to be evaluated.

**Method:** RNA and DNA preservation was compared in a series of colorectal cancer, either conserved with VCS for 24 to 96 h before fixation, or fixed in NBF within an hour delay. RNA was extracted from frozen material in 20 cases, to compare the VCS group to NBF 1 h group and quantified with Spectrophotometer Nanodrop (Shimadzu) and after reverse translation with real time PCR (LightCycler® 480, Roche) of Bcr-Abelson gene. DNA was extracted from paraffin embedded tissue of all the 151 cases and equally quantified with Nanodrop and after screening KRAS mutation by real-time PCR.

**Results:** In all cases, no correlation was found between fixation delay or fixation duration and DNA or RNA concentration, CT or copies number. No significative differences were identified between the VCS and NBF groups for DNA or RNA evaluation.

**Conclusion:** Vacuum sealing and cooling can be considered as a reasonable alternative to immediate formalin fixation for molecular analyses.

Wednesday, 4 September 2013, 09.30–10.30, Pavilion 2

**PS-24 Poster Session Other Topics**

**PS-24-003**

**Connexin expression in Giant Cell Tumour of Bone (GCTB)**

P. Balla ^*^, M. Maros, I. Teleki, Z. Sapi, M. Szendroi, L. Kopper, P. Picci, M. S. Benassi, T. Krenacs

^*^Semmelweis University, 1st Dept. of Pathology, Budapest, Hungary

**Objective:** Intercellular communication through connexin (Cx) channels is known to regulate cell homeostasis and play crucial roles in promoting signals and nutrients between osteoblasts and osteocytes. Giant cell tumour of bone (GCTB) is an aggressive osteolytic lesion, in which neoplastic stromal cells drive osteoclastogenesis. Here we studied Cx expression in GCTB compared to normal fibroblasts (HDFa) cell line.

**Method:** Tissue microarrays, immunohistochemistry and western blot were used for protein detection. Flow cytometry was performed for functional dye transfer in cell cultures.

**Results:** Cx26, Cx30.2, Cx43 and Cx46 were found primarily in the mononuclear cells. Double labelling revealed connexins both stromal and monocytic lineages. Most Cx isotypes were mainly localized in ER/Golgi complex both in primary GCTB stromal cells and HDFa cell line. Cx43 expression and membrane localisation decreased in GCTB stromal cells compared with HDFa cells. Accordingly, flow cytometry revealed low levels of communication between GCTB neoplastic stromal cells in culture. Concerning clinico-radiological correlations, Cx26 showed significantly elevated (*p* < 0.05) expression in aggressive compared to active GCTB cases.

**Conclusion:** Several connexin isotypes can be detected in GCTB. Of these, cell membrane Cx43 and cell coupling are reduced in primary GCTB stromal cells, while Cx26 levels seem to correlate with more aggressive tumor phenotype.

**PS-24-004**

**Bone regeneration and host defense in rabbit bone after the implantation of pure and mixed hydroxyapatite and tricalcium phosphate**

J. Vamze ^*^, M. Pilmane, A. Skagers

^*^Riga, Latvia

**Objective:** Investigation of the appearance and distribution of bone morphogenetic protein 2/4, osteopontin, osteocalcin, osteoprotegerin, Interleukins-1, -8, -10 and β defensin2 protein in the lower jaw of rabbits after the implantation of pure and mixed hydroxyapatite and tricalcium phosphate.

**Method:** Material was obtained from the lower jaw tissue of Californian rabbits after 3 months of the implantation of hydroxyapatite/tricalcium phosphate (HAP/TCP) and tricalcium phosphate (TCP) burned under 1000C and 1150C, hydroxyapatite (HAP) burned under 1000C. Bone tissue were processed for routine morphological study and immunohistochemical detection of the bone regeneration and immune profile proteins. Quantification of immunohistochemical positive osteocytes was done using semi–quantitative evaluation method.

**Results:** The highest expression of osteocalcin and osteopontin and moderate expression of osteoprotegerin was detected in tissue with pure HAP. Expression of osteopontin, osteocalcin and osteoprotegerin was less extent, but equally with control tissue in bone tissue by use HAP/TCP burned under 1000C, while expression of bone morphogenetic protein 2/4 was higher. Study showed low variability of distribution of β defensin2 and Interleukins.

**Conclusion:** Prevalence of osteopontin, osteocalcin and osteoprotegerin containing osteocytes in the experimental tissue 3 months after implantation indicates possible bone regeneration stimulated by pure HAP implant.

**PS-24-006**

**The influence of activated macrophages combined or not with adjuvants in experimental sarcoma cell proliferation**

F. A. Oliveira ^*^, M. S. Gomes, L. C. Alves, A. C. Silvestre Morais, H. S. Goetz, D. F. Magalhães, D. F. Santos, E. C. Barroso Duarte, L. A. Silveira, A. d. Moraes Costa Crespo, C. R. Oliveira

^*^IPTSP/UFG, Setor de Patologia, Goiânia, Brazil

**Objective:** Compare the neoplastic growth and describe the histopathological analysis of experimental sarcoma in BALB/c mice subjected to different treatments with LPS-activated macrophages, associated or not with adjuvant.

**Method:** Animals were divided into seven treatment groups using adjuvants aluminum hydroxide or Freund, associated or not with activated macrophages and a control group. Density of cell proliferation was performed by immunohistochemistry, using anti-cyclin D1. Slides were scanned using Aperio ScanScope XT and then, 10 randomly selected images were captured from each slide per group and analysed. Positive cell couting for cyclin D1 was performed using the software Image J with the ×20 objective lens.

**Results:** The mean densities of cell proliferation in all treated groups were significantly lower than that of the control group (*p* < 0.05). Also, mice treated with activated macrophages in association with Freund’s adjuvant showed a lower density of cell proliferation as compared to the other groups. Presence of inflammatory infiltrate, central necrosis, pleomorphic cells, evident nucleoli and multiple mitoses were the main morphological features observed in all groups.

**Conclusion:** The treatment with immunemodulatory adjuvants and activated macrophages led to a significant decrease in cell proliferation of sarcoma 180 in BALB/c mice. Financial Support: FAPEG, IPTSP/UFG.

**PS-24-007**

**Angiosarcoma: A clinicopathological study of 10 cases**

P. Franco Câmara ^*^, A. F. Capelinha, M. Smit Cordeiro, M. A. Cavaleiro, B. Jardim, R. Freitas, F. Baptista, J. Vieira, J. Camacho

^*^Hospital Central do Funchal, Portugal

**Objective:** The purpose of this study was to examine a series of angiosarcoma cases of Hospital Central Funchal and to characterize their clinicopathological features and prognosis.

**Method:** Between 2006 and 2013, 10 cases of angiosarcoma were diagnosed and the clinical and histological features assessed, including arquitecture, mitotic rate, necrosis, cell morphology and immunohistochemistry.

**Results:** Age at diagnosis ranged from 38 to 93 years. Tumors were located in the skin (7), breast (1), mesentery (1) and mediastinum (1). Three patients had a history of prior radiotherapy for breast carcinoma and one had lymphedema secondary to previous mastectomy. Tumor size ranged from 1,2 cm to 15 cm. Arquitecture: vascular (6) and solid (4). Morphology: mixed (4), epithelioid (4) and spindled (2). All cases stained for CD31 and factor VIII and nine for CD34. Five patients died of disease and two were alive without disease. Survival ranged from less than 1 month to 49 months.

**Conclusion:** Cutaneous angiosarcoma (scalp, face, breast with prior radiotherapy or lymphedema) occurs in older patients when compared with primary mammary angiosarcoma. The patients with non-cutaneous angiosarcoma had metastases at diagnosis and survived less. Patients had favorable prognosis if the tumor was completely excised and less than 5 cm.

**PS-24-008**

**Lean management in histopathology**

P. Branders ^*^, R. Sciot, L. Pintelon

^*^University Hospital Leuven, Dept. of Pathology, Belgium

**Objective:** The objective was to create a state of the art histopathology workflow based on the existing expertise, equipment and quality system. A reduction in turn around time, while maintaining quality, was needed. We wanted to create a continuous workflow in order to decrease the arrears.

**Method:** We used lean management principles to improve the workflow. We started with an analysis of the as-is workflow using a value stream map to reduce the non-value added steps. We used the principles of 5S, just-in-time and muda in a multidisciplinary workgroup to define the to-be workflow in a new value stream map. Each step in the process was standardized to become a well-defined continuous workflow.

**Results:** After implementation we saw a significant decrease of the mean turn around time with 0,8 weekdays(*p* < 0,01). We could also establish a more stable turn around time in the technical process with a standard deviation of 0,2 in comparison with 0,5 before lean management. We measured a significant reduction of the mean arrears after the implementation of lean management principles from 259 to 44 paraffin blocks a day (*p* < 0,01).

**Conclusion:** The implementation of lean management in histopathology has led to a significant decrease and sustainable turn around times and arrears.

**PS-24-010**

**Optimizing tissue samples quality and procedures in a cancer center biobank**

V. Canzonieri ^*^, T. Perin, S. Cervo, P. De Paoli, A. Steffan

^*^CRO-IRCCS Aviano NCI, Dept. of Pathology, Italy

**Objective:** The cancer biobank of the National Cancer Institute, Aviano (Italy) (Cro-Biobank) is a core facility aiming at collecting and managing tissue and blood samples for research purposes. Several parameters have to be monitored and analyzed in order to reduce the potential negative effects imparing the quality of stored tissue samples: environmental factors (like contamination, humidity, and temperature), materials, human factors, methods, and instruments.

**Method:** Since its foundation in 2006, the CRO-Biobank has monitored quality control procedures for the Optimal Cutting Temperature (OCT) tissue-embedding process to prevent hot/cold tissue ischemia. We also implemented aliquoting of stored tissues by frozen sample sectioning.

**Results:** Specific analyses have been routinely performed with positive quality control results (H&E, immunohistochemistry and biomolecular tests). In details, histological and biomolecular characteristics are preserved in aliquoted samples despite temperature rising from −80°C to −20°C, that is the optimal cryostatic cutting temperature to obtain multiple sections.

**Conclusion:** A part from high quality standards for frozen stored samples, we explore alternative scenarios of biobanking based on paraffin embedded samples, as inestimable resources of Pathlabs. We are, then, evaluating a novel tissue preservative that, based on preliminary results, allows both good morphology for histological diagnosis and optimal feasibility of molecular studies.

**PS-24-011**

**Pathology websites for undergraduate education: A review and new resource**

T. Helliwell ^*^, U. Azram, C. Emelle

^*^University of Liverpool, Pathology, United Kingdom

**Objective:** To review general pathology websites and assess their suitability to support undergraduate education in pathology and to provide additional resources for students in Liverpool.

**Method:** A search engine was used to identify ‘pathology revision sites’. Additional sites were identified from those used by residents. Sites were assessed for content, usability and credibility.

**Results:** On line resources could be grouped into: 1. portal sites that provided rapid links to other sites but with little original content; 2. sites that were predominantly image based, often using virtual microscopy, and accompanied by short explanatory text, and 3. sites with longer text and informative images. Preference for content depends on whether one is looking for a revision aid or a primary learning tool. There was little on-line content that engaged students in interactive learning. To assist students in our course we have started to develop a resource that facilitates the understanding of a range of cellular pathology reports through decrypting the jargon and providing general understanding of the implications of the reports‘content.

**Conclusion:** Generally accessible websites that provide information at an appropriate level for undergraduate students are few and of variable quality. There is scope for improving the understanding of pathology by students through interactive resources.

**PS-24-013**

**Intraoperative consultation: Quality assessment**

A. Costa Braga ^*^, M. A. Campo, S. Loureiro dos Santos

^*^Hosp Prof Dr Fernando Fonseca, Serviço Anatomia Patológica, Amadora, Portugal

**Objective:** An accurate intraoperative consultation (IC) is essential for performing appropriate surgical procedures. For this purpose quality control and quality assurance programs should be included in every Pathology Departments. Our objective was determine IC diagnostic concordance (DC) and average turn-around time (TAT) in Hospital Professor Doutor Fernando da Fonseca, EPE’s Pathology Department.

**Method:** We reviewed all 2012’s IC specimens at our Pathology Department, made the correlation between intraoperative (gross examination–GE, frozen sections–FS and/or cytology–CT) and final diagnosis (paraffin blocks), evaluated disagreements and deferrals and determined TAT (overall and technical).

**Results:** We performed IC in 173 surgical specimens: GE = 43, FS = 76, CT = 54. The most common assessments requested were: presence/typing of neoplastic cells (56), characterization of neoplasia (47) and margin’s status (40). Percentage diagnostic agreement was 96.9 %; disagreements (5 cases) were most frequently due to gross assessment; 10 cases were deferred. The average overall TAT was 19.81 min (technical was 10.28 min).

**Conclusion:** In our department IC is an excellent diagnostic test; the DC and average TAT of are consistent with those reported in literature.

**PS-24-014**

**Incidental findings with revelatory character in otherwise unremarkable specimens**

M. Petre ^*^, S. Zurac, A. Birceanu, E. Gramada, G. Micu, L. Nichita, A. Stoica, B. Mastalier, F. Stanicanu, M. Gafton

^*^Colentina University Hospital, Pathology, Bucharest, Romania

**Objective:** Most of the departments of pathology are confronted with a work overload; to ease pathologists efforts, surgeons are tempted to “sort” so-called “not interesting” specimens (lipomas, hernia sacs, hemorrhoids or even appendixes or gall bladders) and not to send them for histopathologic examination.

**Method:** We conducted a study of incidental findings in these unremarkable specimens over a 5 years period of time, in order to establish the frequency of potential life-threatening lesions if any.

**Results:** Twelve previously clinically unsuspected lesions where identified in a total of 1778 “not interesting” specimens (0.67 %): 5 appendiceal endocrine tumors (4 carcinoids and one neuroendocrine carcinoma with lung metastases); one cholecystic carcinoid; one cholecystic metastasis from an unsuspected pulmonary adenosquamous carcinoma; one pericystic metastasis from a signet-ring cell gastric carcinoma; one metastasis from a colonic adenocarcinoma within a hernia sac; invasive ductal mammary carcinoma within a supramammary lipoma; Paget disease in hemorrhoids; carcinomatous embolus within hemorrhoids from a previously unsuspected squamous cell carcinoma of the uterine cervix.

**Conclusion:** Even low in frequency, the incidental findings in otherwise unremarkable specimens have revelatory character for previously unrecognized severe diseases, thus imposing the necessity of histopathologic examination of any tissue, no matter how trivial it looks at a first glance.

**PS-24-015**

**A new ultra-rapid immunohistochemical staining technique using an alternating current electric field: Its values for intraoperative consultation**

M. Endo ^*^, A. Molohashi, J.-i. Akahira, Y. Akagami, Y. Minamiya

^*^Sendai Kousei Hospital, Dept. of Pathology, Japan

**Objective:** A new ultra-rapid immunohistochemical staining technique using an alternating current (AC) electric field has been developed by Akagami Y. and Minamiya Y. et al. We tried to prove its values for intraoperative consultation with frozen sections.

**Method:** Resected 20 metastatic pulmonary tumor and one lymph node with carcinoma metastasis obtained our hospital, from April 2012 to March 2013, provided for intraoperative consultation were chosen for this study. After removal, the specimens were immediately processed into a standard method of frozen section. Frozen sections were cut at 5 μm and placed on slides, air-dried for 30 s, fixed in acetone for 2 min at room temperature, air-dried for 15 s at room temperature, and subjected to the staining procedure. The sections were incubated under the novel technique; high-voltage, low-frequency AC electric field, for 2 min, with nine antibodies respectively, such as CK7, CK20, TTF-1, Napsin A, p63, CDX-2, CD10, vimentin and thyroglobulin according to primary tumor sites. After incubation, the sections were stained ordinary IHC-DAB procedure.

**Results:** All of the specimens were stained within 40 min, and were obtained positive signals for respective origins such as colo-rectal, renal and thyroidal carcinomas.

**Conclusion:** The novel ultra-rapid technique was considered to become a valuable and beneficial method for intraoperative consultation.

**PS-24-016**

**Small intestine apoptosis evaluation after haemorrhage followed by volume replacement with colloid and crystalloid solutions in a pig model**

H. Vala ^*^, A. Ortiz, R. Cruz, C. Garcia, A. Silva, C. Venâncio, J. Mesquita, D. Ferreira

^*^Escola Superior Agrária de Viseu, Instituto Politécnico de Viseu, Portugal

**Objective:** Ischemia may induce apoptosis in the small intestine and seems to be exacerbated by reperfusion. The aim of this study was to compare apoptosis in the small bowel after haemorrhage, followed by volume replacement with hydroxyethyl starch (HES) 130/0.4 and Ringer lactate (RL) solutions.

**Method:** 25 ml/kg of arterial blood were removed from the femoral artery in 18 Large White pigs under general anaesthesia with propofol and remifentanil. Volume was replaced using RL in group1 (*n* = 9), and HES130/0.4 in group2 (*n* = 9), 20 min after bleeding. 1 h after volume replacement, pigs were euthanized. Segments of duodenum, jejunum and ileum were studied immunohistochemically with in situ TUNEL method for apoptosis detection. Data is expressed as apoptotic index (AI).

**Results:** Group1: Duodenum (AI = 41.7 %), jejunum (AI = 33 %) and ileum (AI = 32.2 %); Group2: Duodenum (AI = 24 %), jejunum (AI = 24 %) and ileum (AI = 33.6 %). AI was significantly higher in group1 for duodenum and jejunum (*P* = 0.006 and *P* = 0.046, respectively).

**Conclusion:** HES130/0.4 administration was associated with a smaller percentage of apoptosis in duodenum and jejunum, when comparing with RL solution. These results may be related with the better tissue perfusion and improved microcirculation usually associated with the administration of HES130/0.4. Funding: FEDER funds through COMPETE Program, and by Portuguese FCT (COMPETE: FCOMP-01-0124-FEDER-009525)

**PS-24-017**

**The influence of silver nanoparticles on proliferation and apoptotic activity of glioblastoma multiforme cultured on in ovo model**

K. Urbañska ^*^, J. Sokolowska, M. Szmidt, P. Sysa

^*^WULS-SGGW, Dept. of Morphological Sciences, Warsaw, Poland

**Objective:** Due to ability to cross the blood–brain barrier and affinity for acidic environment, silver nanoparticles (AgNPs) can be a useful tool in anticancer therapy of CNS tumors, especially those of neuropeithelial origin. Thus we evaluated the influence of AgNPs on proliferation and apoptotic activity of glioblastoma multiforme (GBM) cultured on in ovo model.

**Method:** GBM cells line U-87 were placed on the chorioallantoic membrane of the chicken embryos at day 7th. At day 14th tumors were divided into three groups: control (*n* = 20), AgNPs (*n* = 20)- tumors treated with colloidal AgNPs (40 ppm) and placebo (*n* = 15)- tumors supplemented with aqua pro injectione. Four days later tumors were isolated and processed by common paraffin technique. The proliferation activity of GBM was established on the basis of mitotic and Ki-67+ cells index. The number of TUNEL positive cells were calculated to assess apoptotic index.

**Results:** The mean mitotic index for control group was 8,54, proliferation index 28,72 % and apoptotic index was 1,12 %. The same examined parameters for AgNPs and placebo groups were: 5,62, 20,93 %, 2,05 % and 7,88, 27,29 %, 1,07 % respectively. The mean values for all examined parameters differ significantly between AgNPs and other groups.

**Conclusion:** AgNPs can influence tumor’s growth inhibition, however it required more studies.

**PS-24-018**

**Linkers for rare cancer cells isolation**

J. Budna ^*^, K. Sterzynska, E. Frydrych-Tomczak, H. Maciejewski, M. Zabel

^*^Wroclaw Research Centre EIT, BioMed, Poland

**Objective:** The high mortality of different cancer patients is due to a late diagnosis at an advanced stage. This is why screening of high risk patients became a crucial issue. Methods of detection and evaluation of cancer cells (i.e. circulating tumor cells, CTCs) are expected to be accurate, selective and efficient. The aim of this study was to create the silica linkers for effective antibodies binding on the surface of a device for selective CTCs isolation.

**Method:** The system was based on glass slides coated with silica linkers containing several functional groups–amino, epoxy, thiocyanate and isocyanate. The binding of target cells was mediated by the antibody against epithelial cell adhesion molecule (EpCAM)–common for carcinomas, bound with the silica linker. Experiments were conducted using suspension of colon, breast cancer and control cell lines.

**Results:** We successfully isolated EpCAM-positive colon and breast cancer cells using our system. The best results were achieved using silica linkers with amino groups comparing to other tested, what means that effectiveness of antibodies binding was the highest in this case.

**Conclusion:** The system has a potential to become an important device to enrich CTCs in vitro (and after improvements in vivo) increasing prognostic accuracy.

**PS-24-019**

**Photobiomodulation on collagen remodeling and MMP activity during muscle repair process**

R. Mesquita-Ferrari ^*^, A. N. Alves, K. P. Santos Fernandes, C. A. Viana Melo, R. Y. Yamaguchi, C. M. França, D. d. Fátima Teixeira, S. K. Bussadori, F. D. Nunes

^*^UNINOVE, Rehabilitation Sciences, São Paulo, Brazil

**Objective:** Evaluate the effects of Low Level Laser Therapy (LLLT) on collagen remodeling and activity of matrix metalloproteinase 2 (MMP-2) in rat skeletal muscle following acute injury.

**Method:** Wistar rats were divided into five groups: 1) control group; 2) sham group; 3) LLLT group; 4) non-treated injury group and 5) injury + LLLT group. Cryoinjury was performed on the belly of the tibialis anterior (TA) muscle. LLLT was performed daily with an AlGaAs laser (780 nm; beam spot of 0.04 cm2, output power of 40 mW, power density of 1 W/cm2, energy density of 10 J/cm2 and 10-s exposure time). Animals were euthanized at 1, 3 and 7 days. The TA muscles removed and the amount and distribution of collagen fibers were evaluated by picrosirius staining. Characterization and activity of MMP-2 were evaluated by zymography and Western blot techniques, respectively.

**Results:** LLLT induced an increase in MMP-2 gelatinase activity and a greater organization of collagen bundles arranged in a compact form, especially in the region of the perimysium after 7 days.

**Conclusion:** LLLT has a positive effect on MMP2 activity and collagen organization and distribution in the repair process of rat skeletal muscle.

**PS-24-020**

**Clinicopathological correlation in clinical autopsy: Descriptive study of 308 cases from 2006 to 2011 and their comparison with 1991–1992**

L. Garrote ^*^, I. Vazquez de las Heras, R. Albero Gonzalez, J. Corominas Torres, J. Lloreta Trull, S. Mojal Garcia, F. Alameda Quitllet

^*^Hospital del Mar, Dept. of Pathology, Barcelona, Spain

**Objective:** This report aims to provide a descriptive study of clinical autopsies (CA) from 2006 to 2011 and compare it with the 1991–1992 results.

**Method:** A retrospective collection of clinicopathological information of CA from 2006 to 2011, correlation analysis by department and days of stay, and compare it with data in 1991–1992. The Fisher and Mann–Whitney *U* test was applied throughout.

**Results:** The total number of CA at 2006–2011 is 308. The most frequent causes of death were cardiovascular (24.3 %) and respiratory disease (23.9 %). The clinicopathological correlation with the main diagnosis is 66.2 % and with the cause of death is 65.8 %. In 1991–1992 the correlation with the main diagnosis was 78.8 % and with the cause of death was 70.6 %. Only the difference in the main diagnosis correlation is statistically significant (*p* = 0.009).

**Conclusion:** The confidence of the medical staff in the new technologies could have produced a tendency to request autopsies with more complex diagnoses, resulting in lower agreement with the main diagnosis. A tendency for higher percentage of agreement is noted in hospitalization of over 30 days. The emergency department gets the most discrepant results, explainable by the short stay and the characteristics of the patients. This study reaffirms the value of the CA as quality control tool.

**PS-24-021**

**Cystic lymphangioma of the spleen: A pathologico-anatomic and immunohistochemical study of two cases**

E. Kairi-Vasilatou ^*^, C. Dastamani, A. Melloy, A. Tsagkas, S. Stasinopoulou, A. Kondi-Pafiti

^*^Aretaieion University Hospital, Dept. of Pathology, Athens, Greece

**Objective:** To present the pathologico-anatomic and immunohistochemical features and the differential diagnosis of two cases of spleenic cystic lympangioma, a benign neoplasm usually observed in children, in two adult females (41 and 85 years old).

**Method:** In the first case, the splenectomy specimen measured 23 × 17 × 17 cm and weighed 2.133 g, consisting of multiple cysts measuring 0,2–6 cm in maximal diameter, filled with yellow seromucinous fluid. In the second case, the splenectomy specimen measured 16 × 10,5 × 7 cm and weighed 828 g. On its surface, a nodular exophetic tumor was observed, consisting of multiple cysts measuring 0,2–4,5 cm in maximal diameter, filled with serous clear or gelatinous fluid.

**Results:** Microscopically, in both cases, small cystic spaces were observed, lined by one column of flat endothelial cells [Factor VIII (+), CD-31 (+), CD-34 (+), Pan-CK (−)] without atypia, filled with eosinophilic material.

**Conclusion:** Cystic splenic lymphangioma consists of cystic vascular spaces with thin walls, lined by one column of endothelial cells and filled with eosinophelic proteinaceous fluid, which occasionally contains lymphocytes, red blood cells and histiocytes. The differential diagnosis includes mesothelial and epithelial cysts of the spleen, lined by cells positive to cytokeratins, and splenic hemangioma, who has a similar immunophenotype, but consists of cysts filled with blood.

**PS-24-023**

**Correlation of immunohistochemically-identified lymphatic and/or blood vessel tumor invasion with lymph node and distant metastasis in cancer patients**

G. Kir ^*^, A. Ihvan, C. S. Topal

^*^Umraniye Hospital, Dept. of Pathology, Istanbul, Turkey

**Objective:** Lymphovascular invasion is known as an independent predictor of lymph node and distant metastasis in cancer patients. However, the diagnosis of lymphovascular invasion is sometimes diffucult by hematoxylin-eosin (H&E) staining.

**Method:** Immunostaining using CD31 and D2-40 was performed to analyze the correlation of immunohistochemically-identified lymphatic and/or blood vessel tumor invasion with lymph node and distant metastasis in a series of 213 cancer resection specimens (87 colon, 52 breast, 35 stomach, 14 endometrium, 7 cervix, 8 ovary, 10 larynx).

**Results:** Sensitivities for lymph node and distant metastasis by the lymphovascular invasion diagnosed by H&E, CD31, D2-40 and CD31+D2-40 were 72.41 %, 57.47 %, 65.52 %, 79.31 % and 68.18 %, 68.18 %, 59.09 %, 77.27 % respectively.

**Conclusion:** These results suggest that, the sensitivities for the prediction of lymph node and distant metastasis from the lymphovascular invasion status in primary tumor increase by using immunohistochemistry with D2-40 and CD 31.

**PS-24-024**

**Histiocytic necrotizing lymphadenitis (Kikuchi-Fujimoto disease) with atypical locations**

R. Chirculescu ^*^, E. Gramada, A. Bastian, L. Nichita, S. Gheorghita, G. Micu, M. Gafton, A. Dumitru, F. Staniceanu

^*^Colentina Universitary Hospital, Pathology, Bucharest, Romania

**Objective:** Histiocytic necrotizing lymphadenitis (Kikuchi-Fujimoto disease–KFD) is a rare, benign and self-limited disease frequently reported in Asian countries. It affects young adults most commonly in cervical location. The etiology and pathophysiology of KFD are unknown.

**Method:** We studied 9 patients diagnosed in our department between 2008 and 2012; we analyzed clinical and histopathological data.

**Results:** Our patients were mostly females (78 %) between 19 and 34 years. The involved lymph nodes were less than 3 cm in greatest diameter, surprisingly located frequently in inguinal areas (44,44 % inguinal, 44,44 % cervical, 11,12 % axillar). All the cases included altered lymph node architecture; irregular paracortical areas of coagulative necrosis with extensive karyorrhectic debris were present; granulocytes were absent; plasma cells were either absent, or very scanty. No granulomas, giant cells, atypical lymphoid cells or Reed Sternberg cells were seen. Immunohistochemicaly, lymphoid malignancies were ruled out in all cases; characteristically CD15 + macrophages lined necrotic areas. Special stainings (Ziehl-Neelsen, PAS, Giemsa) were negative in all cases. Gomori revealed preserved reticulin network.

**Conclusion:** Although the lymphadenopathy in our cases of KFD had an atypical location, we should consider this diagnosis in young patients; differential diagnosis should include lymphoma, systemic lupus erythematosus lymphadenitis, or infectious processes like tuberculosis.

**PS-24-025**

**Skin and subcutaneous tissue involvement in one case of extranodal Rosai-Dorfman disease**

E.-I. Vana ^*^, M.-M. Petrescu, M. G. Weber, R. Buiga

^*^County Emergency Hospital, Pathology, Cluj-Napoca, Romania

**Objective:** Rosai-Dorfman disease is a rare, non-neoplastic disorder of unknown etiology, frequently located in lymph nodes throughout the body. Extranodal involvement is even more rare. We present the case of a 59-year-old woman with skin and subcutaneous tissue involvement.

**Method:** The patient presented multiple subcutaneous nodules, located on right hand and arm, anterior torax and right breast. Only the lesion located on the right hand was painful and it was removed by surgical excision. After 1 year it recurred in the same place. This and another one located on the right arm were also removed by surgical excision. For the lesion located on the right breast it was performed fine-needle biopsy. Histologically, the lesions were relatively circumscribed, located in the dermis and subcutaneous tissue and presented the same aspects. The tumor was composed of sheets of medium sized, polygonal cells, with abundant pale, foamy, granular cytoplasm and oval nuclei with nucleoli. The tumor cells were displaying lymphocytophagocytosis and were scaterred in a polymorphous background of mature lymphocytes, plasma cells and isolated eosinophils.

**Results:** Tumoral cells were S100, CD68 and VIMENTIN strongly positive; CK and HMB45 -negative.

**Conclusion:** This is a case of pure cutaneous form of extranodal Rosai-Dorfman disease.

**PS-24-026**

**Solitary intraparenchymal splenic metastasis from ovarian carcinoma: A case report**

E. Goupou ^*^, I. Michalopoulou-Manoloutsiou, A. Giannouli, V. Theodorou, A. Kiziridoy

^*^Theagenion Cancer Hospital, Pathology, Thessaloniki, Greece

**Objective:** Solitary intraparenchymal splenic metastasis from epithelial ovarian carcinoma is extremely rare, representing late dissemination disease and is usually asymptomatic. Visceral metastases represent hematogenous spread, capsular involvement resulting from serosal and peritoneal seeding is more common.

**Method:** We report a case of a 73-year-old woman with a history of bilateral serous ovarian cancer 4 years ago. After adjuvant chemotherapy, a year ago, an abdominal CT scan revealed a splenic mass of 2 cm, additionally with an elevated serum CA-125 and CEA. Splenectomy was performed. To exclude peritoneal carcinomatosis, open laparotomy was performed with negative gross and microscopic findings.

**Results:** Grossly, an intraparenchymal solid papillary tumor of 3,5 cm was found. Microscopically, the tumor was compatible with the primary ovarian serous adenocarcinoma. Immunohistochemically, neoplastic cells were positive for Keratin 7, ER, WT-1 and negative for Keratin 20, CDX-2, TTF-1, Hep-Par-1.

**Conclusion:** Splenectomy is the proper therapeutic procedure, if recurrence is located only in spleen. Abdomen should be carefully examined, to exclude intraabdominal dissemination. This case is a reminder of one of the less likely differential diagnosis of an intraparenchymal solid splenic mass and to always keep the medical history of the patient in mind.

**PS-24-027**

**Sclerosing angiomatoid nodular transformation of the spleen: A new IgG4-related sclerosing disease?**

J. Pardal ^*^, S. Guimarães, E. Fonseca

^*^Hospital São João, Dept. de Anatomia Patologica, Porto, Portugal

**Objective:** Nonlymphoid primary tumours of the spleen are rare. The majority correspond to vascular tumors. In our Institution, for a period of 23 years we diagnosed 23 vascular tumors (including hamartomas) and 4 inflammatory pseudotumors. Two of the 23 vascular tumours corresponded to “sclerosing angiomatoid nodular transformation of the spleen” (SANT), an entity described in 2004 by Rosai et al. Recently, because SANT shares some histopathological features with so-called IgG4-related sclerosing diseases, their relationship with IgG4 has been questioned.

**Method:** We report two cases of SANT diagnosed in our Institution and describe their clinicopathological and immunocytochemical features, including the presence of IgG4 positive plasma cells in these lesions.

**Results:** Both cases had the same macroscopic, histologic and immunocytochemical features. Besides the vascular proliferation we noticed the presence of marked stromal sclerosis in both lesions. Plasma cells proved to have a high IgG4/IgG ratio. Splenectomy was performed in both patients and they are well with no evidence of disease. None developed any already known Ig4G-related disease.

**Conclusion:** Although extensive research remains to be carried out, the fact that both cases had obvious increase in IgG4+ plasma cells compared with control lesions support the possibility that SANT may be an IgG4-related sclerosing lesion.

**PS-24-028**

**Unilateral malignant Leydig cell tumour of the testis: An unusual case**

E. Skafida ^*^, T. Vasilakaki, D. Myoteri, K. Koulia, E. Arkoumani, E. Moustou

^*^General Hospital Tzaneio, Dept. of Pathology, Piraeus, Greece

**Objective:** Leydig cell tumour is a benign testicular non germ cell tumour and malignant transformation if present is rare.

**Method:** We report a case of a 35 year old man who presented with a painless left testicular mass. He had no gynecomastia. Blood concentrations of chorionic gonadotrophin,fetoprotein and human placental lactogen were within the reference range. Physical examination and ultrasonography revealed a testicular mass measured 1,2 × 1 cm. A left radical orchidectomy was performed.

**Results:** Microscopic examination revealed malignant leydig cell tumour. The cells had acidophilic cytoplasm, intranuclear inclusions and increased mitotic activity (>3/10 hpt). Many cells with large atypical pleomorphic nuclei were also evident. There was no angiolymphatic invasion, foci of necrosis or extension beyond the capsule of the testis. The immunohistochemical study showed that the tumour cells were positive for Vimentin, Melan A and Inhibin and negative for CKAE_1_, CKAE_3_, S100p, P63, CEA, AFP and Actin. Ki67 was expressed in 10 % of the malignant cells. Retroperitoneal lymph node dissection was suggested but the patient declined further surgery.

**Conclusion:** The diagnosis of a malignant leydig cell tumour is not always easy, because no definite histological criteria exist for malignancy.

**PS-24-029**

**Environmental prints of air pollution on children’s nasal epithelium living along Kifissos river in Attica**

P. Nicolopoulou-Stamati ^*^, A. M. Athanassiadou, A. S. Partsinevelou, P. Stamatis, C. Kotampasi, L. Evrenoglou

^*^University of Athens, Medical School, Filothei Athens, Greece

**Objective:** To investigate the effects of air pollution exposure on the nasal mucociliary respiratory epithelium in healthy children, living and attending school in three municipalities along the banks of the Kifissos River in Attica.

**Method:** Children’s age in the study ranged between 11 and 12 years. Samples of nasal mucosa were obtained from 30 children living in two municipalities designated as urban areas (U). The histology of nasal epithelium of each sample was assessed based on histological criteria that were compared with nasal samples from 19 children living at the third municipality which is characterized as a sub-urban non industrial area (SU) findings where correlated with ozone levels. Pearson’s Chi-square test was used to compare nasal epithelium changes. A *p* value < 0.05 was considered as significant.

**Results:** The important findings in this study were that none of the 49 exposed children to the Athens air environment within similar ozone exposure had normal nasal epithelium. No statistically significant differences were observed between the histopathology parameters in the different municipalities.

**Conclusion:** As the environment through air pollution may affect the histology of nasal mucosa, awareness of the pathologist should be raised on observing environmental impact on tissue and cellular level.

**PS-24-030**

**Multiple accessory tragi without genetic syndromes and other abnormalities**

Y. Yuyucu Karabulut^*^, E. Senel, Y. Dölek, D. Kankaya, B. A. Kirmizi

^*^Cankiri State Hospital, Dept. of Pathology, Çankiri, Turkey

**Objective:** Accessory tragi are relatively common congenital abnormalities, with an incidence of between 1 and 10 per 1000 live births. Multiple accessory tragi, a rare entity, comprises the syndromes, namely Goldenhar (oculo-auriculo-vertebral spectrum), Treacher-Collins, Townes-Brocks, Vacterl, Wolf-Hirschhorn, Delleman, and Haberland (encephalocraniocutaneous lipomatosis). Nonetheless, multiple accessory tragi have not been described in a healty child without a syndrome so far

**Method:** A 9 year-old boy, presented to our department with asymptomatic skin-colored raised lesions in the right preauricular area. According to the mother, the lesions had been present since birth. History of pregnancy was unremarkable with no history of alcohol, tobacco, or drug abuse. The child was born of an uneventful normal vaginal delivery. There was no significant family history. Examination revealed small, soft to firm, skincolored papules in the right preauricular region. Systemic examination was normal.

**Results:** The lesions were excised and on histologic examination a prominent connective tissue framework, subcutaneous fat, with the presence of a cartilaginous component was observed.

**Conclusion:** As is widely believed multiple accessory tragi have been associated with a number of genetic syndromes and other abnormalities, our case showed that multiple accessory tragi can be performed in a healty person.

**PS-24-031**

**Influence of therapy on biomarkers of oxidative stress in patients with Parkinson’s disease**

G. Nikolova ^*^, B. Grigorov, D. Georgieva, Y. Karamalakova, A. Zheleva, V. Gadjeva

^*^Trakia University, Faculty of Medicine, Chemistry and Biochemistry, Stara Zagora, Bulgaria

**Objective:** Parkinson’s disease takes a leading place among contemporary frequent diseases of the central nervous system. This is a slow, progressive disease characterized by loss of dopaminergic neurons in the Substantia Nigra (SN). The aim of the present study is to evaluate influence of Madopar (with active components L-dopa/benzeraside) on levels of some biomarkers of oxidative stress which are stable products of free radical damage of lipids, proteins and nucleic acids.

**Method:** The study was performed in blood samples of PD patients with/without drug therapy and controls. The products of lipid peroxidation were measured spectrophotometrically as malondialdehid (MDA), the amount of 8-hydroxy-2′-deoxyguanosine (8-OHdG) and protein carbonyl (PC) content were measured with ELISA.

**Results:** By the present study we report that the therapy with Madopar lead to decrease of MDA levels and significant increase of PC and 8-OHdG levels comparing with PD patients without drug therapy and controls.

**Conclusion:** In view of these facts we can conclude that administration of Madopar results in a much greater degree of oxidative stress than is induced by PD itself. Further studies are needed to clarify the effect of the combined therapy with antioxidants, which would have a protective effect on Madopar–induced oxidative toxicity.

**PS-24-032**

**Extranodal histiocytic sarcoma primarily arising in the central nervous system in a case of a 9-year-old boy**

E.-I. Vana ^*^, M.-M. Petrescu, M. G. Weber, R. Buiga, I.-S. Florian, A. Baritchii

^*^County Emergency Hospital, Pathology, Cluj-Napoca, Romania

**Objective:** Histiocytic sarcoma is a rare neoplasm occuring mostly in adults, median age 46 years, frequently located in lymph nodes, skin and intestinal tract. We present the case of a 9-year-old boy with central nervous system involvement.

**Method:** The patient presented headache, vomiting and psychomotor agitation. Computer scanning showed 2 masses located in the left frontal lobe and left parieto-occipital area, respectively. The tumors had infiltrative margins. No adenopathies were found. Craniotomy with suboptimal surgical excision of both lesions was performed. Histologically, the tumor was composed of large sheets of epithelioid discohesive cells with abundant eosinophilic cytoplasm and oval to irregular nuclei, often excentrically placed, ‘rocket shaped’. The chromatin was vesicular with large nucleoli. Spindling, binucleation and giant tumoral cells were focally noted. Hemophagocytosis was identified focally. Mitotic index was 10 mitoses/10 HPF. Necrosis and a striking inflammatory infiltrate were also present.

**Results:** Tumoral cells were CD68 and VIMENTIN strongly positive, LCA weakly positive; CD 20, CD3, S100, GFAP, EMA, CK, SMA, DESMIN, CD30, ALK, CD15 -negative. The tumor recurred locally as a giant mass leading to the death of the patient within 2 months.

**Conclusion:** This is a rare case of histiocytic sarcoma occuring in childhood, presenting as a central nervous system primary tumor.

**PS-24-033**

**Diffuse glosis as risk factor of subacute subdural haematoma extension**

A. Enache^*^, N. Vladika, D. Koutsoukis, F. Chatzinikolaou

^*^Thessaloniki, Greece

**Objective:** We studied gliosis in cases with subacute subdural hematoma.

**Method:** The study group contained 14 autopsied cases which presented subacute subdural haematomas, for which H&E stains have been made.

**Results:** Between 2008 and 2012 115 autopsied cases were in relation with dead trauma. Of these, 14 cases were diagnosed with subacute subdural haematoma, the initial CT exam having shown minimum cerebral damage. Between day 21 and day 38, there were subdural bleedings of different sizes, with compressive effects. Demographics: 11 males, 3 females, between the ages of 19 and 62, 7 cases were traffic accidents, 3 were falls and 4 were aggressions. Microscopically, we observed extremely discreet contusive areas, but with cerebral edema and numerous glial cells. These were mostly microglial cells diffusely laid within the white matter and less with a perivascular position.

**Conclusion:** In cerebral trauma with minimal contusive lesions which are initially non-severe, we observed that the microglial cells localized in the white matter and diffusely can be associated with a subacute subdural haematoma with lethal consequences, despite the evacuation of the haematoma and the decompression.

**PS-24-035**

**A rare case of liver epithelioid hemangioendothelioma presented as ischemic stroke**

C. Magkou ^*^, E. Papagianni, P. Constantinou, D. Riga, P. Zis, A. Assi, V. Sevastianos

^*^Evaggelismos General Hospital, Pathology, Cholargos, Athens, Greece

**Objective:** Liver hemangioendothelioma is a rare neoplasm of vascular origin. Apart from asymptomatic patients (42 %), it may present with abdominal pain, jaundice and ascites. We report a case of a young patient presented with an ischemic stroke of the cerebellum as the only manifestation of a liver hemangioendothelioma.

**Method:** A 38-year-old obese woman was admitted to our hospital with acute occipital headache, vomiting and rotational vertigo lasting for 8 h. Brain MRI scan indicated an acute ischemic infarct in the right cerebellum whereas liver MRI showed multiple lesions in both hepatic lobes. An ultrasound guided liver biopsy was performed.

**Results:** Microscopically, liver biopsy revealed a vascular neoplasm with an epithelioid appearance composed of round polygonal cells with glassy pink cytoplasm with prominent intracytoplasmic lumina and intraluminar red blood cells. Immunohistochemistry showed factorVIII, CD34 and CD31 positivity. The diagnosis of liver epithelioid hemangioendothelioma was established.

**Conclusion:** With the unanswered question of whether the ischemic stroke was an early onset manifestation of liver localized hemangioendothelioma, the patient was referred for liver transplantation.

**PS-24-036**

**Esophagoatrial fistula: Report of a case**

V. Henriques ^*^, M. Chorão, A. C. Rodrigues

^*^CHLO, Lisboa, Portugal

**Objective:** Nontraumatic esophagoatrial fistulas are extremely rare and may result from chronic peptic esophagitis or cancer.

**Method:** Authors report a case of an 83 years old man with known history of myocardial infarction, COPD, chronic renal disease and myelodisplastic syndrome. He presented to the emergency department (ED) with abdominal pain, lower limb paresis and worsening dyspnea and received treatment as for acute myocardial infarction, bronchitis and severe anemia. Three days after discharge, he presented with lipotimia and was readmitted on ED with respiratory distress. Nasogastric intubation showed abundant outflow of blood. An oesophageal bleeding ulcer was confirmed with upper gastrointestinal endoscopy. Patient died during procedure.

**Results:** Post-mortem examination revealed a chronic peptic ulcer in anterior oesophageal wall perforated to left atria and an abcess in right thalamus.

**Conclusion:** Due to the anatomical localization esophagic peptic ulcers may mimic other entities such as myocardial infartion, pericarditis, atrial fibrillation and stroke due to embolization of food or septic necrotic debris. Esophagoatrial fistulas are more commonly diagnosed post-mortem, after fatal hematemesis. A proposed diagnostic triad of chronic dysphagia, hematemesis and acute neurologic signs should raise suspicion and prompt investigation.

**PS-24-037**

**Modulated electrohyperthermia causes caspase independent programmed cell death in HT29 colon cancer xenografts**

N. Meggyeshazi ^*^, G. Andocs, S. Spisak, G. Kiszner, P. Balla, L. Balogh, T. Krenacs

^*^Semmelweis University, 1st Dept. of Pathology, Budapest, Hungary

**Objective:** Modulated electro-hyperthermia (mEHT) is a capacitive impedance-coupled modulated radiofrequency, selectively accumulates in the tumor tissue. We have studied the molecular mechanism of action of mEHT related tumor damage.

**Method:** HT29 human colorectal carcinoma cells xenografted into BalbC (nu/nu) mice, were treated with a single shot mEHT for 30 min. 3 parallel samples were collected at 0, 1, 4, 8, 14, 24, 48, 72 h post-treatment. 4 h post-treatment mRNA expression was compared to untreated samples. Human Apoptosis Arrays were also used 8, 14 and 24 h after treatment. Histomorphological, immunohistochemical and TUNEL assay results were analyzed in digital slides.

**Results:** The treatment induced DNA fragmentation (24–48 h) tested by TUNEL assay. In line with this, we observed the mitochondrial translocation of Bax (8–14 h), cytochrome c release from mitochondria to the cytoplasm (8–14 h) and concomitant nuclear translocation of AIF (14–24 h). Furthermore, apoptosis assay revealed the treatment related elevated expression of TRAIL-R2 and FADD (8 h). The mRNA chip detected significant changes in the expression of 48 genes upon treatment, including several heat shock proteins. Immunohistochemistry confirmed elevated hsp70 expression (14–24 h) in the morphologically intact peripheral parts of treated tumors.

**Conclusion:** In HT29 xenografts, mEHT caused programmed cell death utilizing a caspase independent subroutine.

**PS-24-038**

**The accuracy of intra-operative scrape cytology in diagnosis of ovarian tumors**

F. Abbasi ^*^, F. Noroozinia, A. Moradi, S. Oshnoi

^*^Urmia Medical University, Dept. of Pathology, Iran

**Objective:** To evaluate the accuracy of scrape cytology in intraoperative diagnosis of ovarian tumors and to compare it with frozen section (F.S) and permanent section

**Method:** In this prospective study we compared the accuracy of intraoperative scrape cytology with F.S in 75 cases of ovarian tumors received in Urmia, Imam Khomeini hospital during 2010 to 2012.

**Results:** Overall accuracy of scrape cytology was 96 % and of F.S, 97.34 %. Sensitivity, specificity, positive and negative predictive values of scrape cytology were 92.30 %, 96.77 %, 85.71 % and 98.36 % respectively. These figures were 92.30 %, 98.38 %, 92.30 % and 98.38 % for F.S. Youden index of scrape cytology was 89.07 % and of F.S. 90.68 %.

**Conclusion:** It seems that scrape cytology can be used as a simple, inexpensive method for intraoperative diagnosis of ovarian tumors as an alternative to F.S, however, combination of both methods is more reliable.

**PS-24-039**

**Inflammatory macrophage cytokines production is altered by phototherapy**

K. Fernandes ^*^, R. Ferrari, N. Souza, L. Rocha, S. Bussadori, F. Nunes

^*^UNINOVE, Biofotônica, Sao Paulo, Brazil

**Objective:** To analyze the effects of low level laser therapy (LLLT) on the production of cytokines by activated macrophages because the inflammatory process although important for tissue repair is frequently exacerbated compromising tissue function and causing pain and inflammatory macrophages and cytokines are an important component of this process

**Method:** J774 macrophages were activated to simulate an inflammatory situation and received two different laser irradiation parameters (780 nm, 70 mW, 3 J/cm2 and 660 nm, 15 mW, 7.5 J/cm2). TNF-α and IL-6 gene and protein expression were analyzed by RT-qPCR and ELISA, respectively, after 4, 24, 48 and 72hs. Non-irradiated cells were used as controls. Experiments were performed in triplicate and statistically analyzed

**Results:** After 24hs, IL-6 mRNA and protein expression were positively modulated by 660 nm laser irradiation, while 780 nm laser reduced only IL-6 protein production. At 72hs there was no difference in IL-6 expression for both laser parameters. The TNF-α mRNA and protein expression was also reduced by both lasers after 24hs

**Conclusion:** 660 and 780 nm lasers are able to affect macrophage cytokines production and can be a useful tool to modulate the inflammatory process.

Wednesday, 4 September 2013, 09.30–10.30, Pavilion 2

**PS-25 Poster Session Uropathology**

**PS-25-001**

**Histopathological, molecular and genetical aspects in collecting duct renal carcinoma: Case report**

O. Tica ^*^, E. Rosca, O. Pop, O.-A. Tica, D. Rahota

^*^Country Hospital of Oradea, Dept. of Pathology, Romania

**Objective:** The aim of this study is to reveal the histopathological details of Collecting Duct Renal Carcinoma (CDC), known as Bellini Duct Carcinoma, combined with immunohitochemical panel and genetical aberations.

**Method:** We analized 3 specimens of radical nephrectomy from patients with primary renal cell carcinoma (1 woman & 2 men) with ages between 72 and 88 years old. We used classical Hematoxilin–Eosin stain, immunohistochemistry and genetical studies.

**Results:** All three cases were confirmed with CDC and were presenting 5 major criteria (location in a medulary piramid for small tumours; typical histology with irregular tubular arhitecture and high nuclear grade; inflammatory desmoplastic stroma with numerous granulocytes; reactive with antibodies to high molecular weight cytokeratin; absence of urothelial carcinoma) and 3 minor criteria (central location for large tumours; papilary arhitecture with wide, fibrous stalks and desmoplastic stroma; intratubular epithelial atypia adjacent to the tumour) for positive diagnose.

**Conclusion:** Histopathological examination is the “gold standard” in positive diagnosis of these tumours. Use of immunostains in diagnose has its limitations. Although we can diagnose these tumours, the oncological treatment is the same for all renal cell carcinomas and that means that we are a step forward of fellow oncologists.

**PS-25-004**

**Prostate carcinogenesis after the Chernobyl accident in Ukraine**

A. Romanenko ^*^, A. Chekalova, A. Yurakh, P. Harkonen

^*^Kyiv, Ukraine

**Objective:** The present study was conducted to evaluate the development of radiation-dependent lesions in benign prostate hyperplasia (BPH) patients living in 137Cs-contaminated areas of Ukraine.

**Method:** BPH samples from 30 patients from nocontaminated areas (control group 1) and from 90 patients living in 137Cs contaminated areas (group 2). These BPH samples were examined histologically and immunohistochemically (IHC). γH2AX, iNOS, Ki67, p53, p63, p27Kip1 and Bcl2 proteins were IHC investigated in BPH samples from all patients.

**Results:** A pattern of chronic proliferative atypical prostatitis (CPAP) accompanied with large areas of sclerotic stromal connective tissue with increased angiogenesis in association with dramatic increase in the incidences of areas of proliferative inflammatory atrophy (PIA), basal-cell hyperplasia (BCH) with cellular atypia as well as with the areas of prostatic intraepithelial neoplasia (PIN) were detected in group 2 BPH patients.

**Conclusion:** Our data support a strong relationship between long-term low-dose 137Cs radiation exposure of BPH patients who lived about 26 years in radio-contaminated areas and development of CPAP, a possible preneoplastic condition in humans. Our study suggests the alteration of cell cycle transition and apoptotic regulatory molecules in association with γ-H2AX and iNOS overexpression at the areas of PIA and BCH with cellular atypia which could be also crucial early molecular events in the pathogenesis of the radiation induced prostate carcinogenesis.

**PS-25-005**

**Carcinoma of the collecting ducts of Bellini**

L. Santos^*^, D. A. Portela, R. M. Souza-Filho, L. G. de Deus-Sousa, L. F. Braz Lima, J. O. Gomes Santos, J. O. Ibiapina, A. M. Rebelo, T. C. B. Carvalho, C. M. Oliveira

^*^Federal University of Piaui, Dept. of Pathology, Teresina, Brazil

**Objective:** The Bellini duct tumour is rare, accounting for <1 % of renal cells carcinomas and displays metastases by about 1/3 of cases the time of diagnosis. We present a case of advanced carcinoma of the collecting ducts of Bellini.

**Method:** A 34-years-old male presenting with a low back pain, hematuria, and loss weight for 2 months. Radiological examinations (US, CT and MR) showed right renal lesion, heterogeneous, solid and infiltrating, affecting the hilar structures and retroperitoneal and interaortocaval lymphadenopathy.

**Results:** Indicated that percutaneous biopsy of the lesion suggested that it was of Bellini duct carcinoma, which was confirmed by histopathology of the surgical specimen of right nephrectomy (Fig. 1), which revealed carcinoma with tubule-papillary growth pattern in oedematous desmoplastic stroma permeated by inflammatory cells. Metastases to ipsilateral adrenal, right colon and duodenal margin were present. Two months after surgery, patient developed subcutaneous metastatic nodule.

**Conclusion:** The Bellini duct carcinoma is a rare renal neoplasm whose clinical and radiological presentation can be atypical, however histopathological diagnosis can be straightforward in experienced hands. The prognosis is usually poor and 70 % of patients die 2 years after diagnosis.

**Fig. 1. Gross appearance of surgical specimen:** 
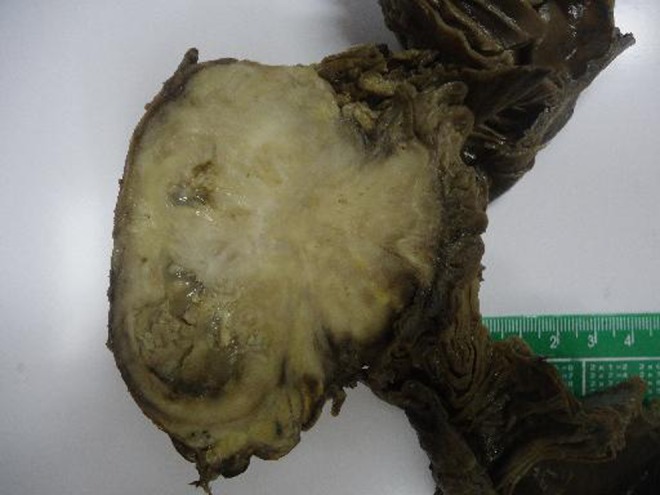


**PS-25-006**

**Alterations of sonic hedgehog pathway components in urinary bladder carcinomas: Possible role in early invasiveness**

B. Kleist ^*^, V. Seel, C. Protzel, C. Kakies, M. Poetsch

^*^Soerlandet Sykehus HF, Dept. of Pathology, Kristiansand, Norway

**Objective:** The sonic hedgehog (SHH) signaling pathway controls proliferation and differentiation during human urinary tract development, but is normally inactivated in adult tissue. In this study, we investigated, whether alterations in functional components of the SHH pathway are associated with the pathogenesis of urinary bladder cancer.

**Method:** Allelic imbalance in nine microsatellite loci (around PTCH1) and the mutation status in the promoter region and five exons of the PTCH1 gene were analyzed in 93 transitional cell carcinomas (TCC) of the urinary bladder. Immunohistochemical SHH expression was determined in a part of these tumors. Results of these analyses were compared with staging and grading characteristics of the tumors.

**Results:** Allelic imbalance was found in all tumor stages, but most frequently in pT1 carcinomas (81.5 %). Only four mutations of PTCH1 were detected in tumor tissue. No differences in the frequency of allelic imbalance were seen after stratifying the carcinomas according to their histological grade. Genetically instable tumors with available immunohistochemical data showed more frequently overexpression than low or no expression of SHH.

**Conclusion:** Our results indicate that alterations of SHH components are involved in the pathogenesis of urinary bladder carcinomas and may play a role in early invasiveness of this cancer type.

**PS-25-008**

**Urothelial lesions and urine zymography of samples obtained from rats exposed to N-butyl-N-(4-hydroxybutyl) nitrosamine**

H. Vala ^*^, D. Talhada, C. Teixeira-Guedes, A. Andrade, A. I. Faustino-Rocha, C. Vasconcelos-Nóbrega, R. Ferreira, P. A. Oliveira

^*^Escola Superior Agrária de Viseu, Instituto Politécnico de Viseu, Portugal

**Objective:** Experimental urinary bladder tumours chemically induced by N-butyl-N-(4-hydroxybutyl) nitrosamine (BBN) has been proposed as a useful model for the study of urinary bladder cancer in humans. The purposes of this study were to analyze in an animal model the spectrum of urinary bladder lesions chemically induced and to evaluate urine proteolytic activity of matrix metalloproteases (MMPs.

**Method:** In this experimental protocol were used 23 female Wistar rats. All procedures were performed in accordance to European Directive 2010/63/EU. Animals were randomly divided into two groups: control (*n* = 10) and BBN (*n* = 13). BBN was administrated in drinking water in a concentration of 0.05 % during 12 weeks. After euthanasia, urinary bladders were collected, sectioned and processed. Zymography technique was performed to analyze MMP activity in urine.

**Results:** The spectrum of BBN-induced urothelial lesions was similar to those observed in humans in their morphological characteristics (simple hyperplasia, nodular hyperplasia, papillary hyperplasia, dysplasia, papilloma, papillary carcinoma and invasive carcinoma). We reported the increase of MMP-2 and MMP-7 proteolytic activity in urine samples.

**Conclusion:** BBN induces the development of urothelial lesions similar to those described in man and the MMP-2 and MMP-7 proteolytic activity identified in rat urine samples is similar to those also described in man.

**PS-25-009**

**Nodular adenocarcinoma of the prostate with leiomyomatous stroma: Report of 3 cases of an underecognized histological phenotype**

V. Caamaño Villaverde ^*^, G. Muñiz, A. Corominas-Cishek, N. Cerda, A. Perez, M. Gonzalez, J. I. López

^*^Cruces University Hospital, Pathology, Barakaldo, Spain

**Objective:** To call the attention on the distinct histology of some prostate adenocarcinomas with nodular architecture and leiomyomatous stroma.

**Method:** Case 1: 77 year old man presenting with urinary obstruction refractory to catheter removal. Serum PSA: 16. Digital exam showed a grade III prostate. Transrectal biopsy (TB) was negative. Retropubic simple prostatectomy was performed. Case 2: 62 year-old man without previous clinical history. Serum PSA: 7.3. TB showed multifocal adenocarcinoma GI 3+3. Radical prostatectomy (RP) was performed. Case 3: 51 year old man with a history of hepatitis C. Serum PSA: 7.58. TB showed monofocal left-side adenocarcinoma, GI 3+3. RP was performed.

**Results:** Prostate adenocarcinoma in the three cases showed on low power view a striking nodular architecture with sharp margins. Each nodule was composed of a pure leiomyomatous stroma with Gleason grade 3 glands. Smooth muscle fascicles were highlighted by desmin immunostaining.

**Conclusion:** The combination of nodular architecture, pure leiomyomatous stroma and low-grade glands may constitute an underecognized morphological variant of prostate adenocarcinoma.

**Prostate adenocarcinoma with leiomyomatous stroma:** 
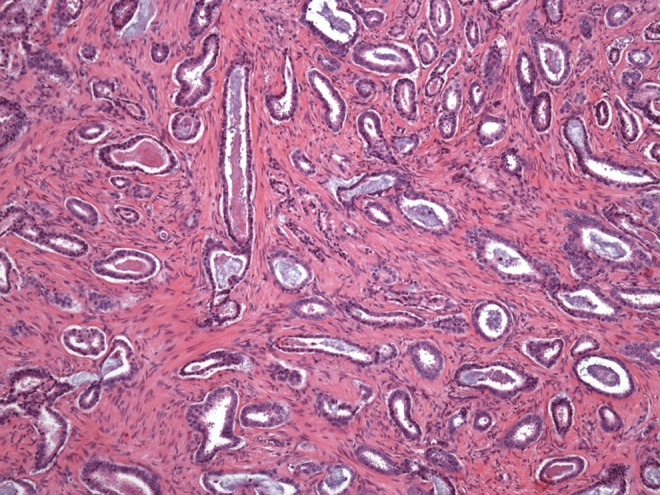


**PS-25-010**

**Metastases into renal cell carcinomas**

V. Caamaño Villaverde ^*^, M. Schiavo-Lena, A. Yagüe, L. Zaldumbide, R. Guarch, R. Tardanico, J. I. López

^*^Cruces University Hospital, Pathology, Barakaldo, Spain

**Objective:** To call the attention about the remote but feasible possibility of tumor-to-tumor metastasis in daily practice

**Method:** Case 1: 70 y/o woman with a chronic lymphocytic leukaemia presenting a left renal mass (1.9 cm). Case 2: 62 y/o woman with infiltrating duct carcinoma of the right breast treated 3 years before presenting a left renal mass (4.5 cm). Case 3: 65 y/o man with B-cell lymphoproliferative disease and a left renal mass (3.7 cm). Case 4: 75 y/o man with chronic lymphocytic leukaemia presenting a left renal mass (5.0 cm)

**Results:** Renal tumors were conventional clear cell renal cell carcinomas in cases 1, 2 and 4, Fuhrman’s grades 1 and 2 respectively, and chromophobe renal cell carcinoma in case 3. Leukemia infiltrates were intermingled with the neoplastic clear cells sometimes resembling chronic inflammation. Immunohistochemistry revealed its neoplastic B-cell origin. Immunohistochemistry highlighted the independent nature of breast and renal tumor cells in the second case. Her2 amplification was detected by FISH in breast cancer cells

**Conclusion:** Only very few reports of tumor to tumor metastases have been documented in the literature. Renal carcinomas are one of the commonest hosts in this poorly known tumor relationship and hemolymphoid, lung and breast neoplasms appear among the most frequent guests.

**Breast tumor into renal tumor:** 
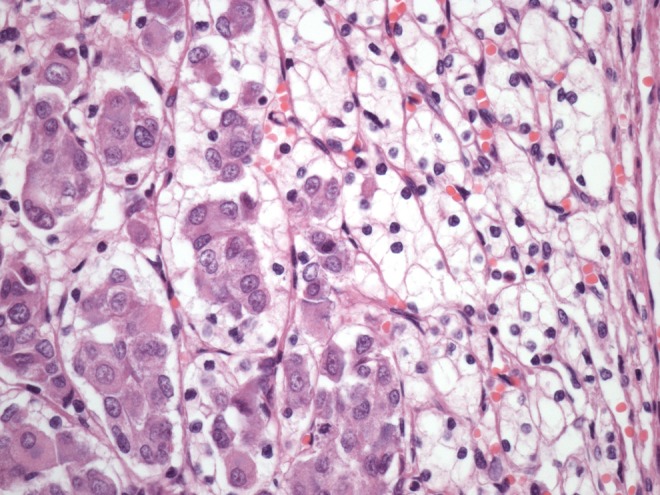


**PS-25-011**

**Prognostic utility of CD44, E-cadherin and EMA in non-clear cell renal cell cancer**

L. Moskvina ^*^, J. Andreeva, L. Zavalishina, P. Malkov, G. Frank

^*^Gertsen Research Oncology Inst., Dept. of Pathology, Moscow, Russia

**Objective:** Possible prognostic utility of CD44, E-cadherin and EMA was evaluated in non-clear cell renal cell carcinoma (ncRCC).

**Method:** 93 patients, undergoing partial or radical nephrectomy for RCC were retrospectively analyzed by immunohistochemical analysis for CD44, E-cadherin and EMA. Prognostic utility was established using Kaplan-Meier curves (log-rank test). Immunoreactivity for all markers was scored semiquantitatively (positive–strong expression in >40 % cells or moderate in >60 % cells).

**Results:** Patients with larger tumor size (pT) had significantly higher rate of CD44 and lower rate of E-cadherin expression (*χ*2, *p* < 0,05). We identified CD44 expression in 3/30 papillary RCC (pRCC) type 1, 18/24 pRCC type 2, 12/24 chromophobe RCC (chRCC) classic variant, 0/15 chRCC eosinophilic (*χ*2, *p* < 0,05), E-cadherin–10/30, 5/24, 19/24, 9/15 respectively (*χ*2, *p* < 0,01), EMA–15/30, 11/24, 13/24, 7/15 respectively (*χ*2, *p* < 0,05). pRCC type 2 showed highest CD44 expression and the lowest E-cadherin expression, that might result in the adverse clinical course. Kaplan-Meier survival analysis showed that CD44 expression only was strongly associated with decreased survival for patients with ncRCC (*p* = 0,018).

**Conclusion:** CD44 is very useful prognostic marker for ncRCC, prognostic significance of E-cadherin and EMA needs more research.

**PS-25-012**

**Adaptive processes of the great renal arteries in atherosclerosis**

D. Kozlov ^*^

^*^Institute of Pathology, Smolensk, Russia

**Objective:** The external renal arteries are functionally loaded not less than the arteries of the heart and brain. But clinically, atherosclerosis of renal arteries is more than that of arteries of heart and brain. Our goal was Study of adaptive processes of the great renal arteries in atherosclerosis.

**Method:** After autopsy we studied 825 renal arteries morphologically, from 194 women and 194 men (mean age is 60,15 years) for detection of adaptive changes.

**Results:** Ageing is accompanied by two types of changes in renal arteries. 1. Adaptive processes which are directed towards preservation of lumen and thickening of renal arteries: 2. Adaptive processes which help walls of renal arteries to fulfill their mechanical function. Atherosclerosis of renal arteries was detected in 33.2 % cases. In cases of atherosclerosis we distinguished three types of adaptive processes: 1. Adaptive processes which are directed towards strengthening the wall of the renal arteries. 2. Adaptive processes which save the lumen of renal arteries and also renal blood supply. 3. Adaptive processes which are connected with regression of atherosclerotic process in renal arteries.

**Conclusion:** Information about adaptive processes studied in renal arteries can be extrapolated to other arterial beds.

**PS-25-013**

**Frequency of ERG-positive prostate carcinoma in Poland: Preliminary results**

K. Okon ^*^, K. Kaczmarczyk, G. Dyduch, M. Bialas, S. Demczuk, T. Szpinski, P. Chlosta

^*^Jagiellonian University, Dept. of Pathomorphology, Krakow, Poland

**Objective:** Prostatic carcinoma (PC) is one of the most frequent cancers in men. Molecular pathogenesis of PC remains poorly understood, translocations involving ERG gene were found to be the single most frequent genetic event. A strong correlation exists between this translocation and ERG-positivity on immunohistochemistry. The rate of ERG-positivity and its relationship with other clinicopathologic parameters differs between populations; there are no data on ERG-positive PC in Eastern-Europeans.

**Method:** We constructed tissue microarrays of unselected radical prostatectomy cases and performed standard immunohistochemistry for ERG.

**Results:** The study group consisted of 42 cases; 18 were Gleason score 6; 15 Gleason score 7; 6 Gleason score 8 and 3 Gleason score 9. 19 cases were pT2 and 23 pT3. 20 cases were positive for ERG. Majority (59 %) ERG-negative cases were pT2 but majority (70 %) of ERG-positive cases were pT3. No relationship between Gleason score and ERG expression was found.

**Conclusion:** The frequency of ERG-positive PC in our series is similar to Western populations. Expansion of the study group is needed to show a consistent relationship between ERG expression, stage and grade of the tumor.

**PS-25-014**

**ERG, Ki-67, Gleason index and tumor volume correlations in transrectal core biopsies with prostate adenocarcinoma**

J. I. López^*^, N. Cerda, V. Caamaño, G. Larrinaga, A. Gaafar, C. Valenti, A. Gómez de Iturriaga, C. Bruno

^*^Cruces University Hospital, Dept. of Pathology, Barakaldo, Spain

**Objective:** To evaluate the ERG, Ki67, Gleason index (GI) and tumor volume (TV) statistical correlations in transrectal core biopsy material.

**Method:** Prospective series of 54 consecutive cases collected in the clinical practice of two hospitals in a 3 month period (Oct-Dec 2012). ERG (Dako, clone EP111) and Ki67 (Dako, clone MIB-1) were tested following routine methods. GI was lumped in three categories (low<7, intermediate = 7, high>7). TV was obtained by the total sum of millimetres of tumor in the biopsy.

**Results:** Average age of patients was 69.2 years. Low GI was found in 14 cases (25.9 %), intermediate in 24 (44.4 %) and high in 16 (29.7 %). Average TV was 20.9 mm (range 0.5–123). Ki67 index was <5 % in 50 cases (92.5 %) and ≥5 % in 8 (7.5 %). ERG was positive in 28 cases (51.8 %) and negative in 26 (48.2 %). GI correlated with TV (Spearman, *p* = 0.000) and with Ki67 (Spearman, *p* = 0.015). TV correlated with Ki67 (Spearman, *p* = 0.01). ERG did not show any significant correlation.

**Conclusion:** As expected, GI and TV statistically correlate with Ki67 index and support its impact in tumor prognosis. Conversely, ERG does not correlate with GI, TV or Ki67 index thus confirming previous disappointing data.

**KI67 immunostaining in prostate adenocarcinoma:** 
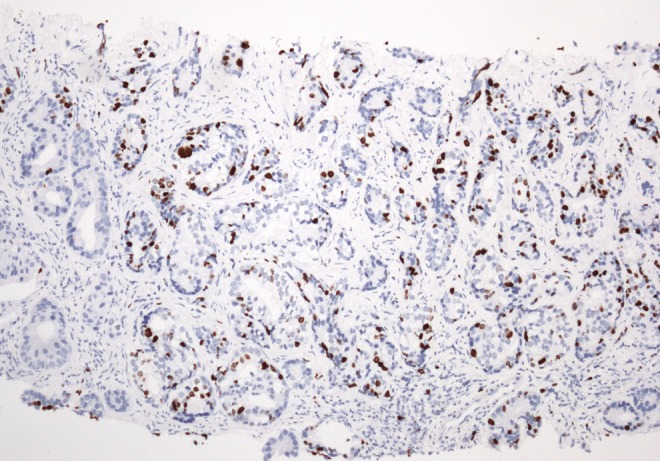


**PS-25-015**

**Immunohistochemical comparison of ERG and AMACR/HMWCK in difficult prostate needle biopsies diagnosing**

S. Bachurska-Yovcheva ^*^, D. Staykov, G. Ivanov, I. Bakardzhiev, V. Belovezhdov

^*^Medical University Plovdiv, General and Clinical Pathology, Bulgaria

**Objective:** The diagnosis of prostate adenocarcinoma is often challenging on the needle biopsy, because of the small amount of the tissue. Immunohistochemistry (IHC) could be helpful especially in these cases. The aim of the study is to compare the usefulness of anti-ERG and a combination of α-mathylacylcoenzym A recemase (AMACR) and high molecular cytokeratin 34βE12 (HMWCK) IHC staining on the needle prostate biopsies, presenting “atypical glands suspicious for cancer”.

**Method:** Biopsies from a retrospective cohort (*n* = 95) were IHC stained with anti-ERG, AMACR and HMWCK and antibodies

**Results:** Nuclear ERG staining were observed in 39 % (23/35) of the cancer biopsies. All adenocarcinoma cases (0/58) lacked HMWCK staining and 98 % (51/7) of these showed diffuse or focal cytoplasmic AMACR staining. 100 % (0/37) of the benign cases, including atrophy, adenosis, basal and clear cell hyperplasia, lacked ERG staining. Positive AMACR and HMWCK immunoreactivity were observed in 19 % (7/37) and respectively 98 % (36/37) of the benign biopsies.

**Conclusion:** Anti-ERG antibody is a new helpful tool for differentiation of the benign cases, since 100 % were ERG-negative. ERG/AMACR/HMWC cocktail could be used as a routine method in diagnosing of the challenging prostate needle biopsies.

**PS-25-016**

**Atrophic changes in the prostate gland and their differential diagnosis with carcinoma**

V. Kropelnytskyi ^*^, L. Zakhartseva, M. Dyatel, P. Yakovlev

^*^Kiev Clinical Oncology Center, Dept. of Pathology, Kyiv, Ukraine

**Objective:** To evaluate the incidence of atrophic changes in the prostate gland.

**Method:** During the period of 2010–2013 years 417 patient aged 18–86 years (median age 67) with suspicion of the prostate cancer were underwent core biopsy. Core number was 6–14 (median 10). Tissue sections stained with hematoxylin-eosin were performed. For immunostaining p63 (clone 4A4, DAKO) and Cytokeratin 5/6 (clone D5/16/B4, DAKO) antibodies with EnVision+ (DAKO) visualization system were used.

**Results:** Prostatic adenocarcinoma was diagnosed in 248 cases (59.5 %). Benign changes were diagnosed in 143 cases (34.3 %). In 26 cases ASAP foci were revealed (6.2 %). In 108 cases with unclear histology (26 %) immunostaining were used. Malignant process was diagnosed in 60 cases. Atrophic changes were diagnosed in 48 % cases. In 8 cases (16.6 %) in the atrophic regions focal basal cell markers disappearance were revealed. Overall atrophic changes were diagnosed in 234 cases: adjacent to the tumor in 49.5 % of carcinoma cases and in 65.6 % of cases with benign changes.

**Conclusion:** Atrophy is the most frequent benign pathology in prostate gland that can mimic carcinoma. For the differential diagnosis p63 and CK5/6 immunostaining can be used. But the use of immunostaining alone without accurate morphological data assessment could lead to the cancer misdiagnosis.

**PS-25-017**

**Unclassified testicular mixed sex-cord/stromal tumor in a young adult**

T. Bujas ^*^, J. Šoškic, M. Krajnc, R. Kavalar

^*^ University Medical Centre MB, Pathology, Maribor, Slovenia

**Objective:** Unclassified mixed sex-cord/stromal (UMSCS) tumors constitute about 4–6 % of adult testicular tumors. They originate from supporting tissues of the testis, including Leydig, Sertoli and granulosa cells.

**Method:** A 23 year old man with markedly enlarged left testis was referred to urological department. Ultrasound examination showed a mass, which has almost entirely replaced testicular parenchyma. The levels of tumor serum markers were normal.

**Results:** The testicle measured 6.4 × 4 cm and firm tumor measured 4.7 cm in greatest diameter. The tumor cut surface was yellow, without hemorrhagic and necrotic areas. Microscopically the tumor was composed from fascicles of spindle cells. Neither Sertoli cells nor granulosa cells were found. There was no nuclear pleomorphism, no necrosis and the mitotic figures were frequent (12/10HPF). Cells were immunohistochemically vimentin and sm-actin strongly positive, moderately positive for α-inhibin.

**Conclusion:** UMSCS tumors are composed of cellular elements which can’t be differentiated as Sertoli, Leydig or granulosa cells at light microscopy. We described UMSCS tumor of the testis made up predominantly of spindle cells, which is rare in post-puberty male. Tumor cell population was immunoreactive to vimentin, sm-actin and α-inhibin.

**PS-25-018**

**Renal clear cell carcinomas coexpressing p53 and mdm2: Preliminary analysis**

K. Okon ^*^, M. Hejnold, G. Dyduch, M. Bialas, S. Demczuk, J. Rys, P. Chlosta, T. Szopinski

^*^Jagiellonian University, Dept. of Pathomorphology, Krakow, Poland

**Objective:** Clear cell carcinoma (CCC) is the most frequent form of renal cancer; prognosis of this tumor remains uncertain, prompting search for new biomarkers. The prognostic role of TP53 mutation and p53 expression in CCC is controversial. It has been suggested that CCCs coexpressing p53 and mdm2 may represent subgroup with a more aggressive clinical course. The aim of the study was to test whether p53-mdm2 positive group differs from the rest of CCCs.

**Method:** Tissue microarrays containing unselected renal carcinomas were used for the study. Immunohistochemistry for p53, mdm2, CA-9, CD10, CK7, Ki67, VEGF-A, VEGF-C and VEGF-D was done by standard method and assessed semiquantitatively without knowledge of clinicopathologic data.

**Results:** The study group consisted of 485 cases; 126 cases (24.9 %) were positive for both mdm2 and p53. Positive cases showed higher expression of CK7 and Ki67 but lower expression of CD10, VEGF-D and VEGF-A. No difference in other markers expression was seen. Positive cases were smaller (5.5 vs. 6.4 cm) and of lower stage than negative ones.

**Conclusion:** Our results may support the opinion that mdm2 and p53 positive CCCs constitute a distinct subgroup. Multivariate survival analysis will be the next step to clarify the issue.

**PS-25-019**

**Clinical and morphological aspects of prostate cancer**

T. Pavlova ^*^, D. Bessmertniy, I. Pavlov, A. Komisov, U. Hoschenko, K. Prashchayeu, A. Nesterov

^*^BelSU, Dept. of Pathology, Belgorod, Russia

**Objective:** Every year more than half of million men all around the world get malignant prostate tumors.

**Method:** One thousand and two hundred cases of prostate cancer had been researched from 1999 to 2012. Morphological methods: light, transmission, probe, bitmap and scanning microscopies, with trace-element analysis, immunohistochemistry (PSA, p63, Ki-67).

**Results:** The location of tumor cells on endothelium of vessels, weak connection between them and presence of minor clones of tumor cells, which can contribute to metastasis are unfavorable prognostic features at prostate cancer. Minimal or absent expression of the high-molecular cytokeratin was revealed. Overgrowths of changed acinar structures, formed by light one-row epithelium with expression of PSA and weak expression of racemase were revealed in stroma among glands. The quantity of Ki67+ cells in tumors was 1–2 %. The content of separate macro- and microelements in the tumor node in patients with progression of prostate cancer was authentically higher than in cases without progression Thus, increase of content of oxygen, natrium, magnesium, phosphorus, (*р* < 0,05), what testifies about high metabolic activity of these cells. Pic.1

**Conclusion:** The conducted complex morpho-chemical analysis reflects diagnostic patterns of prostate cancer.

**Pic.1. Carcinoma of the prostate. A. Hematoxylin and eosin stain х 200. B. Scanning Electron Microscopy x 400. C. Expression of KI67 D. Atomic force microscopy.:** 
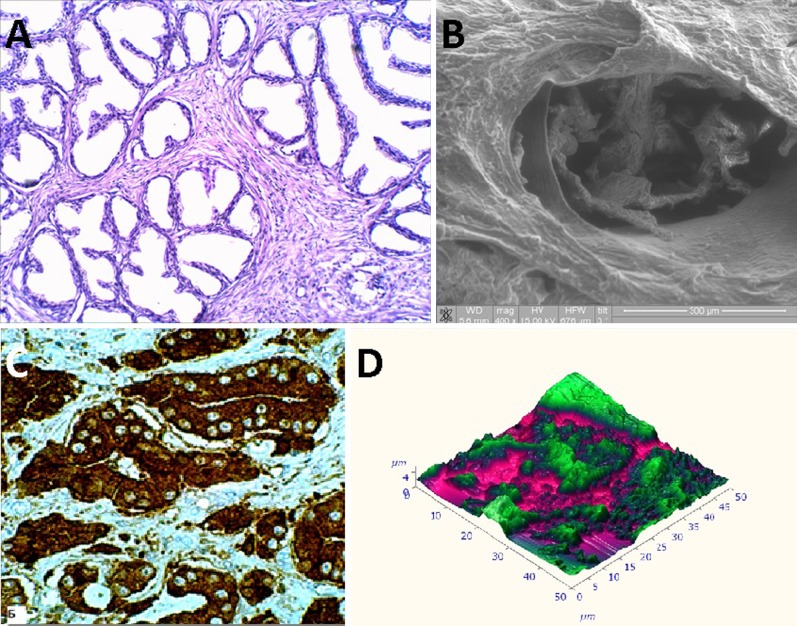


**PS-25-020**

**Macro and micro-elemental composition mapping of onco-urological tissues**

T. Pavlova ^*^, A. Komisov, I. Pavlov, D. Bessmertniy, D. Kolesnikov, E. Kudryavtsev

^*^BelSU, Dept. of Pathology, Belgorod, Russia

**Objective:** Development of the qualitative and quantitative methods of mapping of macro elemental composition of onco-urological tissues On the basis of quantitative analysis data to obtain isolines of concentration of different elements such as K, Mg, Ca, S etc.

**Method:** Prostate and bladder cancer tissues are studied with the help of Scanning (SEM), Transmitting (TEM) and Scanning Transmitting (STEM) Electron Microscopy and energy-dispersive X-ray spectroscopy (EDS).

**Results:** Obtained images and macro elemental composition mapping of IV stage prostate and bladder cancer tissues. The most efficient sample preparation protocols were approved.

**Conclusion:** Preliminary results showed good visualization of qualitative macro elemental distribution in cancer tissues. The further work on this problem will result in quantitative mapping of macro elements in cancer tissues.

**PS-25-021**

**The expression of phosphorylated mammalian Target Of Rapamycin (p-mTOR) correlates with increased lymphangiogenesis and lymph node metastasis in prostate adenocarcinoma: An immunohistochemical study**

K. Gyftopoulos^*^, I. Lilis, H. Kourea, H. Papadaki

^*^University of Patras, Dept. of Anatomy, Greece

**Objective:** To evaluate the expression of p-mTOR in prostate adenocarcinoma and examine possible correlations with tumor parameters, lymph node status and the lymphangiogenic cytokines Vascular Endothelial Growth Factor -C and A (VEGF-C and A).

**Method:** p-mTOR was assessed immunohistochemically in 24 radical prostatectomy specimens from patients with clinically localized disease who were found to have nodal metastasis (pN1) and was compared with 68 pN0 cases. Furthermore, mean lymph vessel density (LVD), VEGF-C and A expression, lymphatic invasion and clinicopathological parameters were examined in relation to p-mTOR expression

**Results:** Increased p-m TOR expression was present in N1 vs N0 cases (Mann–Whitney test, *p* = 0.009) and was associated with lymphatic invasion (Mann–Whitney test, *p* < 0.0001) and increased mean LVD (ANOVA, *p* = 0.003). VEGF-C and A expression were associated with increased p-mTOR immunostaining (Kruskal-Wallis, *p* = 0.008 and *p* = 0.045 respectively). Increased VEGF-A expression was associated with lymphatic invasion but not mean LVD in tumor specimens, while VEGF-C immunostaining was associated with both parameters. Gleason grade (but not T stage) was also associated with increased p-mTOR immunopositivity.

**Conclusion:** Our results indicate that activation of the mTOR pathway may facilititate nodal metastasis, possibly due to increased lymphangiogenesis and lymphatic vessel invasion via increased VEGF-C and VEGF-A expression.

**PS-25-022**

**Vascular endothelial growth factor and itscorrelation with angiogenesis in prostate cancer in untreated patients**

A. Salapura ^*^, L. Tadic Latinovic, Z. Eri, S. Usaj Knezevic

^*^Clinical Center Banja Luka, Dept. of Pathology, Bosnia and Herzegovina

**Objective:** Evaluation of vascular endothelial growth factor (VEGF) as a major inducer of angiogenesis, is useful in assessing the angiogenic phenotype in prostate carcinoma. The aim of the study was to investigate immunohistochemically the expression of VEGF and its correlation with microvessel density in prostate cancer.

**Method:** The study population included 61 patients who underwent radical prostatectomy (RP) for localized prostate carcinoma and did not receive chemo-, hormone, or radiation therapy before surgery. VEGF was examined by immunohistochemistry, and its tissue expression was compared with MVD evaluated by immunostaining the endothelial antigen CD31 and then correlated with tumor stage and grade.

**Results:** Normal prostate tissue generally showed no or only low VEGF expression, there was a significant increase in VEGF expression in all prostate cancer specimens, the intensity of the immunoreaction ranging from low to strong and being correlated with the tumor stage (p ˂ 0,0001), Gleason score (p ˂ 0,0001) and MVD (p ˂ 0,05) in prostate carcinoma.

**Conclusion:** Our immunohistochemical results indicate that VEGF seems to be an important, clinically relevant inducer of angiogenesis in prostate carcinoma. VEGF expression was shown to correlate positively with tumor stage, Gleason score and MVD.

**PS-25-023**

**Characterization of specific genetic aberrations in squamous bladder tumours**

N. Gaisa^*^, M. Molitor, R. Schmidt, E. Eltze, M. Toma, S. Siegert, K. Junker, R. Knuechel

^*^RWTH Aachen, Inst. für Pathologie, Germany

**Objective:** Little is known about specific genetic changes of non-Schistosoma associated squamous carcinoma of the bladder. Therefore, in this study we investigated squamous carcinomas, mixed tumours and urothelial cancers for structural genetic differences.

**Method:** Urovysion-FISH was performed on tissue microarray slides. Fifty nuclei of *n* = 25 pure squamous cancers (SCC), *n* = 31 mixed tumours (MIX) and *n* = 19 urothelial carcinoma (UC) could be successfully analysed. Additionally, comparative genomic hybridization of *n* = 35 SCCs, *n* = 40 MIXed and *n* = 22 UC samples was implemented.

**Results:** All tumours scored positive according to the Vysis FISH criteria. Overall means for each probe showed slight differences of chromosome numbers of squamous and mixed/urothelial carcinomas at chromosome 3 and 17. Mixed tumours presented highest levels of chromosome copies, SCCs showed lowest numbers of chromosome 3 and 17. Comparative genomic hybridization revealed significant differences between the three groups regarding chromosome 3p (*p* = 0,004), 5p (*p* = 0,020), 6q (*p* = 0,028), 11p (*p* = 0,023) and 21q (*p* = 0,018). Significant changes in pure squamous cell carcinomas comprised loss of genetic information at chromosome 3p and 5p.

**Conclusion:** SCCs showed less chromosome copy number changes than mixed and urothelial carcinomas, especially at chromosomes 3 and 17. Comparative genomic hybridization also showed fewest genetic aberrations in pure squamous tumours, and indicated losses at chromosome 3p and 5p as characteristic changes.

**PS-25-024**

**High risk pathways in urothelial carcinomas: Morphological correlation to p53, p21/p27 or p16 pathways and their effectors CDK4, pRb and E2F**

P. Stoemmer ^*^, P. Torres- Galea

^*^Forschungslabor für Pathologie, Augsburg, Germany

**Objective:** Urothelial carcinomas result via low risk FGFR3-pathway or via high risk pathways like p53 and p21 or via p16INK4a- pathway. IHC-protein-level study on these oncogenes/suppressors in relation to morphological behaviour of tumorcells

**Method:** FFPE archival invasive and non-invasive urothelial tumors, semiquantitatively analysed by IHC. Antibodies: p21WAF1Ab-5 HZ52 1:100; p27Kip1 SX53G8 1:150 E2F2 Polyclonal 1:25; p53 Ab8 DO-7 + BP53-12 1:250 (Medac, Germany). Cdk-4DCS-35 1:100; pRb IF8 1:100 (DCS, Germany). p16INK4a (mtm Heidelberg, Germany).

**Results:** p16INK4a: normal urothelial cells completely negative; in pTaG1 few, mostly basal tumor cells are reactive in cytoplasm and nucleus. Invasive carcinomas (pT1/2; G3) show intense reaction in nearly all tumorcells.

**Conclusion:** On proteinlevel only p16INK4a, p53 and p21 can be used as marker for high-risk-pathway in urothelium.

**Expression of p16INK4a in HG invasive urothelial carcinoma:** 
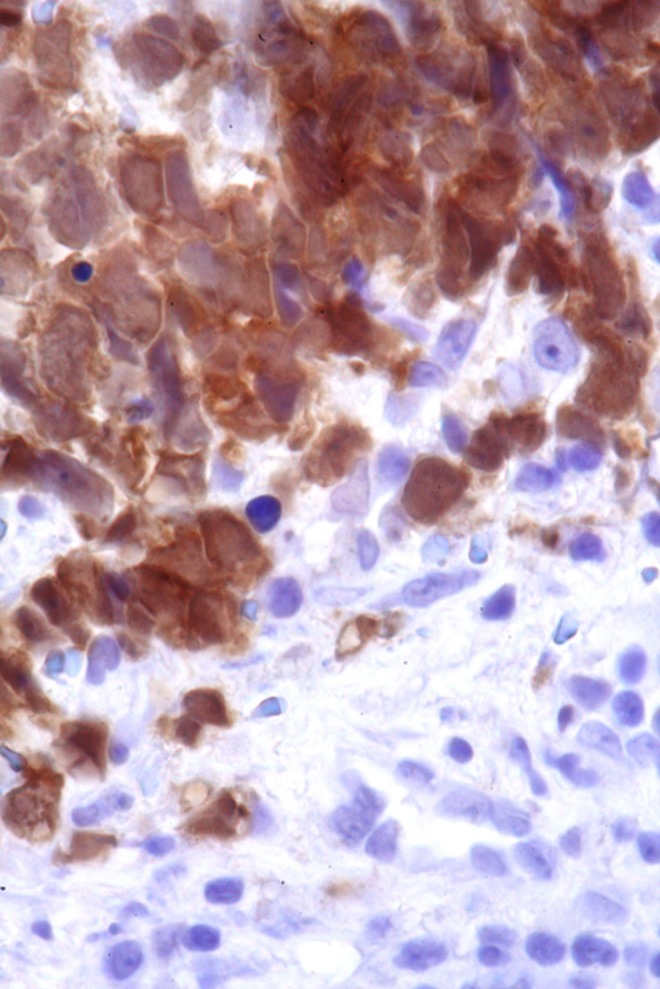


**PS-25-027**

**Basaloid squamous cell carcinoma in a 50-year-old woman with history of long term spinal cord injury**

R. Dias ^*^, F. Moreno, J. Preza, A. Duarte

^*^Hospital de Santo António, Porto, Portugal

**Objective:** Basaloid Squamous cell carcinoma (BSCC) was initially described by Wain et al., in a report of tumors of the tongue, hypopharynx and larynx. Since then, there were few reports of this condition arising in the bladder.

**Method:** We report a case of a 50-year-old woman, with history of long term spinal cord injury and a subsequent non-contractible neurogenic bladder.

**Results:** The patient was admitted to hospital September 2012 for a pyelonephritis. Ultrasonography revealed a suspicious bladder mass; citoscopy showed a tumor lesion, with fleshy appearance and the microscopic analysis of the transurethral resection showed an invasive carcinoma with squamous differentiation. The patient underwent radical cistectomy: on gross examination it was observed an ulcerative lesion with invasion through the anterior vaginal wall, and histopathology evaluation revealed an invasive BSBC. The bladder and uterus specimens showed cytological evidence of HPV infection and molecular study showed positivity for 18 serotype.

**Conclusion:** Some studies have reported a 2.5 %–10 % incidence of squamous cell carcinoma in the spinal cord-injured population. It has also been suggested an association between HPV infection and BSBC. The present case is of a woman with long term bladder trauma and infected with HPV witch are both probably a good territory to malignant transformation.

**PS-25-028**

**HOXB13 a new marker to distinguish prostate carcinoma from other origins**

E. Comperat ^*^, J. Varinot, O. Cussenot, L. Cheng

^*^Hopital La Pitié Salpatriere, Dept. of Pathology, Paris, France

**Objective:** HOXB13 gene is essential for prostate development. Few studies showed immunohistochemical expression of HOXB13 in the nuclear compartment of benign prostate luminal epithelium and prostate carcinoma (PCa) cells, but no expression in urothelium. The origin of carcinomas is sometimes difficult to establish in primitive or metastatic tumours, especially in bladder or prostate samples.

**Method:** Fourty-three cases of prostatic and vesical resections/biopsies, metastatic lymphnodes or pelvic masses were retrieved from our database. In all cases a doubt of prostatic versus urothelial origin persisted. All cases were stained for CK7, p63, p504s, PSA, CK20, and HOXB13. Chromogranine A, CD56, synaptophsin were used in case of suspicion of a neuroendocrine differentiation.

**Results:** HOXB13 staining was negative in all cases of urothelial origin. Seven doubtful cases (16 %) were retrospectively confirmed as PCa. Three of four cases with neuroendocrine differentiation, did not express HOXB13. The fourth case was positive; the patient had anterior history of PCa. One case with both types of carcinomas, HOXB13 was exclusively expressed in the prostatic part.

**Conclusion:** Our results demonstrate good sensitivity of HOXB13 in PCa, where the origin might be difficult to determine. HOXB13 IHC could be a sensitive marker for prostatic cells and a valuable diagnostic tool.

**PS-25-029**

**Immunohistochemical localization of interleukin-1 beta in benign prostatic hyperplasia and prostate cancer**

O. Zarnescu ^*^, P. Badea, A. Petrescu

^*^Bucharest University, Dept. of Animal Biology, Romania

**Objective:** Although a link between inflammation and cancer has been reported in many studies, there is limited knowledge about the source of interleukin-1 beta (IL-1 beta) in benign prostatic hyperplasia (BPH) and adenocarcinoma (ADK) of prostate. In this study, we compared the immunohistochemical expression of IL-1 beta in the BPH and ADK.

**Method:** This study was performed on 2 normal samples, 17 samples of BPH and 20 samples from patients with ADK. The patient’s ages varied between 55 and 85 years. IL-1 beta expression was assessed by immunohistochemistry.

**Results:** Normal and BPH samples showed heterogeneous immunostaining pattern, with prostate glands that express IL-1 beta, whereas others glands remained complete negative. In the ADK samples, with high Gleason grade, strong IL-1 beta staining was detected in stroma located between prostate glands.

**Conclusion:** We suggest that IL-1 beta should be taken in consideration as a potential marker for discriminating between BPH and ADK. Also, IL-1 beta can be used more effectively in combination with other tumor markers.

**IL-1 beta immunohistochemistry_prostate:** 
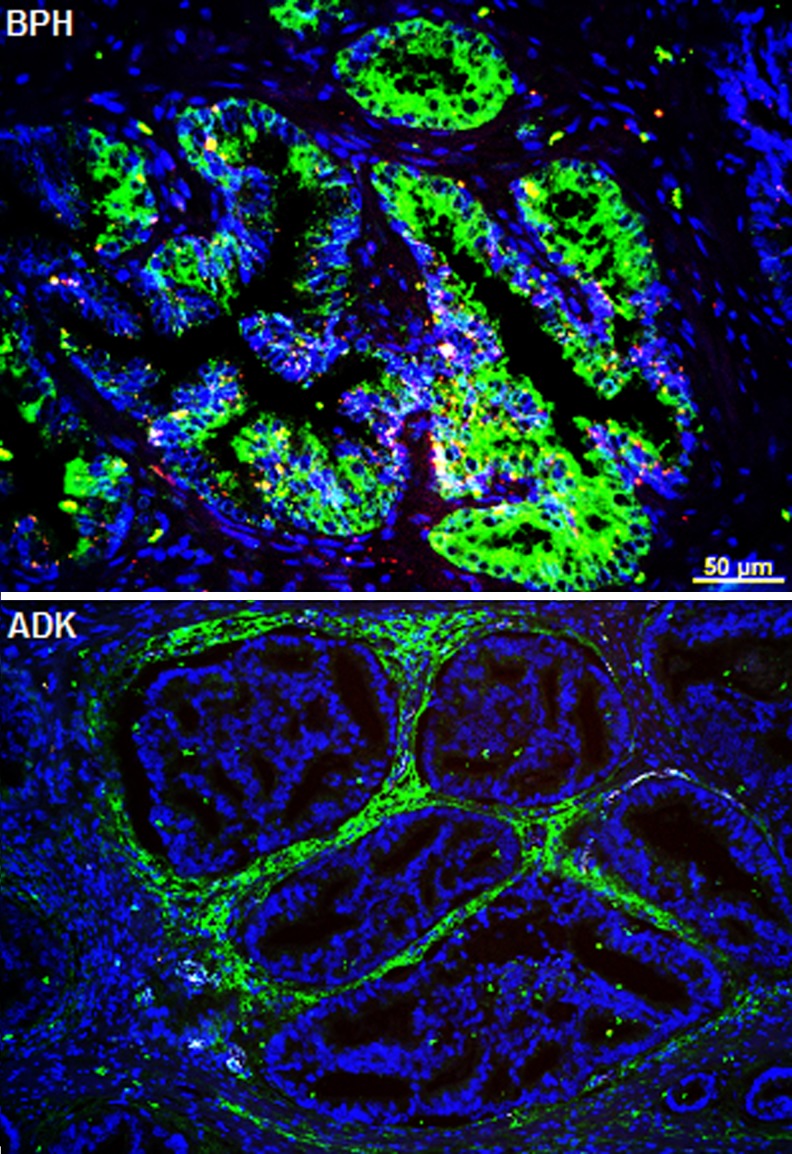


**PS-25-030**

**Tumor-infiltrating M2-type macrophages have the best prognostic value among a panel of hypoxia, angiogenesis and microenvironment related markers in 257 clear cell renal carcinomas**

J. Cros ^*^, E. Sbidian, K. Posseme, A. Letierce, C. Guettier, G. Benoit, S. Ferlicot

^*^Beaujon University Hospital, Dept. of Pathology, Clichy, France

**Objective:** Renal clear cell carcinomas (RCCC) is neoplasm with an unpredictable behavior. Despite adequate initial surgery, 20 to 30 % of patients will develop local recurrence or metastasis during follow-up. Usual clinical and pathological scores tend to class most patients in an intermediate prognostic group. We propose to assess the prognostic value of hypoxia, angiogenesis and microenvironment markers on metastatic diffusion and overall survival in RCCC.

**Method:** Tissue samples from 257 consecutive patients with RCCC were obtained retrospectively between 1993 and 2004. Expression of VEGFR3, HIF1α, HIF2α, CAIX, p-mTOR, nestin, CD44, SDF1/CXCR4, CD68 and CD163 was assessed by immunochemistry on TMA. Factors associated with metastasis and death were analyzed in uni and multivariate analyses.

**Results:** 257 patients were analyzed. Progression free survival (PFS) was associated with SDF1 (*p* = 0.09) and CD44 (*p* = 0.03) in univariate analysis. Overall survival (OS) was associated with VEGFR3 (*p* = 0.05), HIF2α (*p* = 0.05), CXCR4 (*p* = 0.09) in univariate analysis. pT stage, Fuhrman grade, CD68+ and CD163+ M2 type tumor-infiltrating macrophages (M2-TAM) were independently associated with PFS and OS in multivariate analysis.

**Conclusion:** Hypoxia and angiogenesis markers seem to be less associated with the prognosis, especially for metastatic diffusion, than markers involved in stroma-tumor cell interactions such as SDF1/CXCR4 and M2-TAM.

**PS-25-031**

**Transitional cell carcinoma of the bladder mimicking lobular carcinoma of the breast**

B. Chelly^*^, H. Azzouz, A. Zehani, I. Chelly, H. Nfoussi, S. Haouet, N. Kchir

^*^Tunis, Tunisia

**Objective:** To describe a case of transitional cell carcinoma which show morphological features which mimic lobular carcinoma of the breast and diffuse carcinoma of the stomach.

**Method:** Discohesive variant of urothelial carcinoma is a rare neoplasm defined by the World Health Organization as a type of urothelial carcinoma. We report a transitional cell carcinoma which shows morphological features which mimic lobular carcinoma of the breast.

**Results:** We describe a case of a 76 years old men whose past surgical history was consistent for a nephroureterectmy for urothelium disease. Since then, he was followed for recurrent urothelial carcinoma and had received BCG therapy 4times. Recently, he presented with lower urinary symptoms. A G2, Pt2 bladder tumor was diagnosed and a cystoprostatectomy was performed. Histological study concluded to a G2 Pt2 urothelial carcinoma with a striking discohesive growth pattern which shows morphological features that mimicked infiltrating lobular carcinoma of the breast.

**Conclusion:** Discohesive variant of urothelial carcinoma is a poorly defined category which is important to recognize in order to ovoid misdiagnosis of metastatic lobular carcinoma of the breast or diffuse carcinoma of the stomach.

**PS-25-032**

**Correlation between spermatogenic phenotype and successful pregnancy in azoospermy**

E. Dubova ^*^, Y. Popova, K. Pavlov, R. Ovchinnikov, A. Shchegolev

^*^Research Center for Obstetrics, Dept. of Pathology, Moscow, Russia

**Objective:** Incidence of azoospermy in the infertile men reaches 40–50 %. As a result the role of testes biopsy with complex morphological study in assisted reproductive technology increases (ART). Our aim was to study spermatogenesis disturbances in testis biopsy specimens in azoospermy patients.

**Method:** We studied testes biopsy specimens from 97 infertile men. Nonobstructive azoospermy was diagnosed in 64 cases, obstructive–in 33 cases.

**Results:** In 16 cases we revealed normal spermatogenesis signs in biopsy specimens, but only in 7 cases (43.8 %) ART was successful with following pregnancy. In 32 cases hypospermatogenesis was diagnosed and ART was successful only in 10 (31.3 %) of this cases. Subtotal seminiferous tubules epithelial atrophy was diagnosed in 12 cases with 3 successful ART cases (25 %). Germ cell arrest at the round spermatid level was revealed in 5 men with only 1 successful ART (20 %). No successful ARTs were observed in the cases of germ cell arrest at the primary spermatocyte level (8 cases) and Sertoli cell only syndrome (24 cases).

**Conclusion:** Morphological study of the testis biopsy specimens is useful to reveal a type, stage and degree of spermatogenesis disturbance and gives a prognosis of ART successfulness.

**PS-25-033**

**The endothelin axis promotes Epithelial to Mesenchymal Transition (EMT) and lymph node metastasis in prostate adenocarcinoma**

K. Gyftopoulos^*^, S. Papanikolaou, H. Kourea, H. Papadaki

^*^University of Patras, Dept. of Anatomy, Greece

**Objective:** To investigate the activation of the endothelin axis in prostate adenocarcinoma and examine possible associations with EMT markers, lymph node metastasis and clinicopathological parameters.

**Method:** We immunohistochemically evaluated the expression of Endothelin 1 (ET-1) and its receptors A and B (ET-A, ET-B) in 64 N0 and 23 N1 prostate adenocarcinoma cases. EMT markers E-cadherin, N-cadherin, β-catenin and the transcriptional factor Snail were evaluated. We examined possible correlations of ET pathway members with EMT markers, lymph node status, Gleason grade and T stage.

**Results:** Increased expression of ET-1 and ET-A (but not ET-B) was present in prostate carcinoma; both ET-1 and ET-A were associated with lymph metastasis and T stage but not with Gleason grade. We observed E-cadherin and β-catenin decrease/relocalization and increased N-cadherin expression. Snail also showed increased expression in tumor tissue and was associated with lymph node metastasis ((Mann–Whitney test, *p* = 0.0032). Expression of ET-1 and ET-A correlated with Snail expression (Spearmann r, *p* = 0.0002 and *p* = 0.0176 respectively).

**Conclusion:** Our results indicate that activation of the ET pathway induces EMT via Snail activation and correlates with increased metastatic potential.

**PS-25-034**

**Gleason automatisation: The end of a dilemma?**

E. Comperat ^*^, H. Alsehhe, J. Varinot, G. Choquet, M. Soussaline, O. Cussenot

^*^Hopital La Pitié Salpatriere, Dept. of Pathology, Paris, France

**Objective:** In 2005 the Gleason grading (GG) was modified. Nevertheless important discordance in GG still exists. The aim was to find an automatic computerized way to permit standard GG.

**Method:** Nineteen standard prostate biopsies (PB) from 15 patients were evaluated by one senior and two junior pathologists. GG was reported. Then new slides of the same PB were double stained by immunofluorescence, green with Annexin-3, a marker of low grade GG and normal tissue, red with BCAR-1, a marker of high GG. Virtual capture was made of the most representative zones of each sample at ×100 magnification. Computer analysis (CA) was made by Cellmarker and MarkIndex.

**Results:** Modified 2005 GG was applied by pathologists. No GG 2 was given on PB. Gleason score (GS) from 6 to 9 was attributed to each PB. In 10 (58 %) cases total concordance between pathologists and CA was found. In 7 (41 %) cases GS evaluated by pathologists was one GG less than by CA, one case was evaluated one GG higher by pathologists.

**Conclusion:** This study was a preliminary study of feasibility of GG standardizing. CA seems to be a promising technique for standardizing GG and permitting homogenous groups for prostate cancer treatment.

**PS-25-035**

**Familial occurrence of renal cell carcinomas of different histologic types: Report of three cases**

H. Kourea ^*^, M. Gkermpesi, D. Batsoulis, M. Papadopoulou, H. Geropoulou, E. Liatsikos, C. Scopa

^*^Uniersity of Patras, Dept. of Pathology, Greece

**Objective:** Familial renal cell carcinoma (RCC) of different histologies is associated with Birt-Hogg-Dube syndrome (BHDs), an autosomal dominant genodermatosis,also characterized by fibrofolliculomas, lung cysts and pneumothorax.

**Method:** We report three RCCs occurring in a family, exhibiting differing histologies, without known history of BHDs.

**Results:** First, a 37 year old nurse had a 1.5 cm clear cell RCC (cRCC), Furhman grade (FG) 2 resected. Four years later, her father, aged 73 years, had a 4.5 cm RCC resected by partial right nephrectomy. The tumor displayed oncocytic papillary features with solid and papillary growth along with scattered neoplastic cells showing clear multivacuolated cytoplasm, suggesting cRCC. At age 75, the patient had a left nephrectomy for a 17 cm predominantly cystic tumor. The neoplasm was largely necrotic with residual foci of cRCC FG3 in transition to a clear cell spindle cell component without frank nuclear atypia to fulfill the criteria for sarcomatoid carcinoma. The adjacent parenchyma displayed numerous microscopic foci of papillary RCC type 1, type 2 and oncocytic type and cysts. The patient has radiologically liver cysts and a hemangioma and, both his daughter and himself, multiple melanocytic nevi some previously resected.

**Conclusion:** Ongoing further clinical evaluation and genetic analyses will assist to the understanding of these unusual cases.

**PS-25-036**

**Primary malignant melanoma of urethra**

S. Kalantarli ^*^, E. Gadimaliyev

^*^Central Customs Hospital, Dept. of Pathology, Baku, Azerbaidshan

**Objective:** Melanoma is a very rarely tumours of urethra. K. Sugaya et al. indicated that in the world literature was described 42 melanoma of urethra. In most cases, the women were aged 32 to 80 years.

**Method:** Patient was female of 56 years old, postmenopausal for at least 5 years in our observation. She was admitted to the department of urology with complaints of periodic bloody discharge from urethra during last months. Papillary bleeding structure with necrotic fields originating from posterior wall (5 to 7 o’clock) of urethral meatus was revealed. Clinical diagnosis was caruncul. Macroscopicaly there was ulcerative polypoid formation with hemorrhages and dark areas.

**Results:** Microscopically we revealed atypical melanocytes with strong pigmentation. Immunohistochemical observation demonstrated of over expression of S100; HMB 45; CD 56; CD99; CD34; Tyrosinase and high level of Ki 67 (>40 %) proliferative index.

**Conclusion:** Primary melanoma of urethra is rare. Clinical investigations demonstrated the masking of urethral melanoma like as caruncul. We presented this case for the rarity of urethra tumors with high vascular and proliferative potential.

**Melanoma:** 
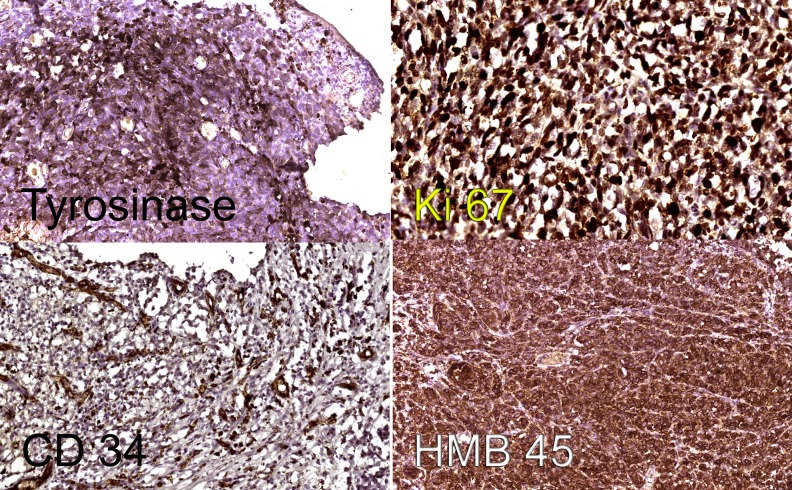


**PS-25-040**

**Clinicopathological features of primary diffuse large cell lymphoma of the testis: Report of a series of 9 cases diagnosed on orchiectomy specimens**

R. J. Luque ^*^, C. Martínez de Carvajal, M. Urdiales Viedma, F. Elósegui Martínez, A. López Beltran

^*^Complejo Hospitalario de Jaén, Dept. of Pathology, La Guardia de Jaén, Spain

**Objective:** Diffuse large B cell lymphoma (DLBCL) is the most frecuent primary lymphoma of the testis. The aim of this work is to determine the clinicopathological profile of a series of DLBCL diagnosed in orchiectomy specimens

**Method:** We have reviewed our orchives and selected a total of nine cases of DLBCL presenting primarily at the testis, after orchiectomy because of clinical diagnosis of testicular tumor. It has been recorded clinicopathological and immunohistochemical features as well as the behavior of disease.

**Results:** Nine cases of DLBCL were identified. Two of the cases were associated to immunodeficency. Isolated testicular mass was the clinical presentation in all the cases. Size of the mass ranged between 4 and 10 cm, and age between 37 and 78 years (when only non immunosupressed patients are considered the age range is 49–78 years). One case shwed high levels of LDH (2556 U/L, N: 240–480) without other serum markers anomaly. No extratesticular afectation was noted at diagnosis. Follow up (4 months-9 years) have shown relapse of the neoplasm in two cases (2 and 3 years after diagnosis).

**Conclusion:** Histopathological diagnosis of testicular DLBCL can be difficult if immunohistochemical markers are not performed. It is essential to have in mind this diagnosis in old patients as well as immunocompromised ones.

**PS-25-041**

**Combined low expression of cytokeratin 20 and high molecular weight cytokeratin defines a subset of non muscle invasive bladder carcinomas with good prognosis**

R. J. Luque ^*^, C. Martínez de Carvajal, A. M. Blanca Pedregosa, A. Quintero Cabello, J. Álvarez Kindelán, A. Lopez Beltran

^*^Complejo Hospitalario de Jaén, Dept. of Pathology, La Guardia de Jaén, Spain

**Objective:** Recent reports on genetic signatures of bladder cancer identify several phenotypes with diverse prognosis. The aim of this study is to assess if expression of cytokeratins (CK) 20 and high-molecular weight (HMWCK) has prognostic value in NMIBC.

**Method:** We assess expression of CK20 and HMWCK in 149 NMIBC and relate the combined expression of these markers (CK20-/HMWCK-, parietal-like, CK20-/HMWCK,+, squamous-like and CK20+/HMWCK+/−, basal-like) with recurrence, progression and specific survival intervals.

**Results:** The three types are not statistically associated to sex, gender or stage, but squamous-like phenotype is more frequent in high grade tumors (52.95 % vs. 13.83 % and 10.53 % for parietal and basal-like, respectively, *p* < 0.01) Recurrence rate showed non significative differences. Progression was 9.5 % vs 23.5–40.07 % (*p* = 0.016) and disease specific mortality 6.38 % vs 23.5–23.6 % respectively (*p* = 0.013). Survival analysis showed a significant better outcome for progression (*p* = 0.05) and disease-specific survival (*p* = 0.004) for parietal vs other types.

**Conclusion:** Our study identify a subset of NMIBC with low expression of CK20 and HMWCK, with better prognosis compared with other types. Further studies are needed to stablish the dynamic and molecular features of this subset and its clinical relevance.

**PS-25-042**

**Adenomatoid tumours: Report of three cases**

A. Kurt ^*^, I. Çalik, F. Erdogan, E. Sener

^*^Bölge Egitim ve Arastirma Hastesi, Dept. of Pathology, Erzurum, Turkey

**Objective:** In this study we present, adenomatoid tumour of epididimis.

**Method:** Adenomatoid tumours are regarded as distinctive benign mesothelial neoplasms of the paratesticular region.

**Results:** I-45-year-old male patient. The white solid mass was located in the lower pole of testis,just next to the the capsule and 3*2*1.5 cm sized. EMA −, CK 5/6 +, calretinin +, PAS −, PLAP −. CD 34 in the vessels +. Diagnosis: 1-Atrophic testis, 2-Adenomatoid tumor next to the testis. II-43-year-old male patient. 50 g in weight and 6*4 *3 Cm sized orchiectomy material. 3*2*1 cm sized, gray coloured nodular lesion which was adjacent to the testis was seen. EMA −, CK 5/6 +, calretinin +, PAS −, PLAP −. Diagnosis: 1-Testis which has hypospermatogenesis findings, 2-Adenomatoid tumor. III-71-year-old male patient. 60 g in weight bleeding gray material sent in pieces. EMA −, CK 5/6 +, calretinin +. Diagnosis: 1-Epididymal adenomatoid tumor, 2-Testicular tissue showing maturational arrest. 3-Severe bleeding and subacute inflammation.

**Conclusion:** Three adenomatoid tumor materials located in the scrotum that we have diagnosed in 2009–2013 years are presented. The tumor is out of the testicular tissue in each of the three materials. With this study we can say that adenomatoid tumor is a rare disease.

**PS-25-043**

**Examination of the effects of phenolic acid on rat testes which were exposed to lead poisoning, by capas 3 technique**

A. Kurt ^*^, K. A. Terim Kapakin, Y. S. Saglam, D. Saripinar

^*^Bölge Egitim ve Arastirma Hastesi, Dept. of Pathology, Erzurum, Turkey

**Method:** 20 rats were divided into 4 groups. The first group served as control. The second group was given 2,000 ppm of lead. In addition to lead the third group was given 1,050 μmol of phenolic. In addition to lead in the fourth group were given 2,100 μmol of phenolic. The testes of sacrificed rats were stained immunohistochemically (Tunnel and caspase 3) and histopathology (hematoxylin and eosin). They were examined microscopically.

**Results:** Testes of the rats in the control group were normal. In the 2nd group, disorganization was seen at the tubulus seminiferous and basement membranes. Hyaline appearance, intertubular edema and pyknosis at Sertoli cells were seen in some tubule lumens. Spermatozoa show disfigurement, or did not exist. Tubules degeneration and decreases at spermatozoa were seen at testes which were given phenolic (groups 3 and 4). The morphological appearance at groups 3 and 4, showed a decrease by the amount of the phenolic. Testes of rats exposed to lead, phenolics were found to be protective.

**Conclusion:** Tunnel staining results were made posters in the National Pathology Congress in 2011, Turkey. Here, the previously presented hematoxylin eosin, Tunnel staining and the results of the caspase3 staining which were made to the same material were discussed.

**PS-25-045**

**Incompletely differentiated (unclassified) sex cord/gonadal stromal tumor of the testis with a “pure” spindle cell component: report a case with diagnostic difficulties**

P. Constantinou ^*^, D. Riga, I. Themeli, M. Chantziara, G. Karagkounis, C. Vourlakou

^*^Athens, Greece

**Objective:** The group of incompletely differentiated (unclassified) sex cord/gonadal stromal tumor [IDSCGST] includes rare cases with predominant spindle cell morphology.

**Method:** We report a case of a “pure” spindle cell tumor of the testis with morphological and immunohistochemical features consistent with “IDSCGST” in the right testis of a 67-year-old man without hormonal abnormality.

**Results:** The testis was almost completely replaced by a solid, circumscribed, tan-colored tumor 13,5 cm in diameter, weighing 212 g. Histologically, a large portion of the tumor was composed of uniform spindle cells with eosinophilic cytoplasm, without nuclear pleomorphism or mitotic activity. Immunohistochemically tumor cells showed diffuse positivity for CK8.18, AE1/AE3, EMA, CD10 and Vimentin with focal expression of Melan-A, Calretinin, CD99, PR and Nestin and stained negatively for Inhibin-a, a-SMA, Desmin, PLAP, CD117 etc. Given the spindle cell morphology, the differential diagnosis with other benign and malignant spindle cell tumors is discussed and the literature regarding this entity is reviewed.

**Conclusion:** The rarity of the tumor and the presence of unusual features prompted us to report the case. The concurrent presence of some features of both Leyding and granulosa cell lines in the tumor supports its origin from a stromal stem cell, possibly capable of dual differentiation.

**PS-25-046**

**Plasmablastic lymphoma of the urinary bladder: Case report**

M. Vozmitel ^*^, T. I. Nabebina, A. Dubrovskij, A. Rolevitch, S. A. Krasny

^*^Minsk, Belarus

**Objective:** Plasmablastic lymphoma (PBL), firstly described as an aggressive lymphoma of the oral cavity of patients infected with the human immunodeficiency virus, may occur in other extra-nodal, mucosal-associated sites. We report a case of PBL of urinary bladder in a HIV-negative elderly person.

**Method:** Clinical data, histologic features of tumor. Formalin-fixed paraffin block for immunohistochemistry

**Results:** Patient was 60-year old woman with no evidence of a previous lymphoproliferative disorder, signs of multiple myeloma and no serum M-gradient. Except solitary bladder tumor 9,6 × 5,3 cm in size, MRI and CT investigations did not find mass lesions at other body locations. Histologically, surgically removed tumor was characterized by diffuse infiltration of bladder wall by large plasmablasts and immunoblasts with abundant basophilic cytoplasm, eccentric nuclei, and high mitotic rate. Starry sky pattern, numerous apoptotic bodies and small number of mature plasma cells and lymphocytes were seen. Tumor cells demonstrated the following immunoprofile: CD45-, CD20-, CD79A, PAX5-, CD3-, CD30-, ALK-, pan-CK-, S-100-, EBV-, CD138+ and MUM1+, Ki-67 >95 %. The patient received chemotherapy and alive 1/2 year after diagnosis.

**Conclusion:** PBL should be included in the differential diagnosis of urinary bladder lesions; careful morphology evaluation, awareness of existence of this uncommon lymphoma help lead to a correct diagnosis.

**PS-25-047**

**Neoplasm associated to end renal disease: Study of new entities**

M. Carme ^*^, A. Iban, M. Mireia

^*^Hospital Clinic, Anatomia Patològica, Barcelona, Spain

**Objective:** The association of neoplasms to terminal renal disease (TRD) is a well-known phenomenon. Papillary renal cell carcinoma has been reported as the most frequent but two new entities have been related to it: clear papillary renal cell carcinoma (CPRC) and “renal cell carcinoma related to end stage disease” (RCESRD), as well as oncocitic tubular changes. We studied their incidence in a 40 consecutive cases with neoplasms associated to TRD

**Method:** We revised 40 consecutives cases of nephrectomies because of neoplasms associated to TRD. Immunohistochemical and FISH studies were performed.

**Results:** In all cases we could identify oncocitic changes. Two cases had changes of CPRC and 6 had morphological characteristics of RCESRD. The other 33 cases corresponded with papillary changes, one hemangioma, 3 angiomyolipomas and 2 cases of classical clear cell carcinomas. Immunohistochemical nor FISH studies were not relevant.

**Conclusion:** Our results indicates that these new entities could be some kind of differentiation of renal cell carcinomas but are not the most frequent tumors associated to renal end disease. More studies had to be performed to exactly define the biological behavior relation of end renal disease of these new entities.

**PS-25-048**

**Usefulness of sentinel node strategy in invasive carcinoma of the urinary bladder: Initial study**

P. Czapiewski ^*^, W. Polom, M. Markuszewski, W. Cytawa, G. Romanowicz, M. Matuszewski, W. Biernat

^*^Medical University of Gdansk, Dept. of Pathology, Poland

**Objective:** Data concerning sentinel lymph node evaluation are very limited due to technical problems in maintaining lymphatic drainage in empty urinary bladder. After introducing own strategy that overcomes this difficulty we performed analysis of the usefulness of sentinel lymph node biopsy (SLNB) in patients with urinary bladder carcinoma.

**Method:** Nine patients with invasive carcinoma of the urinary bladder (with confirmed infiltration of the muscle layer in the biopsy), 6 males and 3 females, aged 56–66 years were enrolled into this study. During cystectomy 2 substances were used to detect SLN: radiotracer–technetium given day before surgery and fluorescence tracer–indocyanine green (ICG), given just before the surgery. ICG alone was given to 5 patients, both tracers to 4 patients.

**Results:** Tumor stage was 2b and 3a in one patient each, 3b and 4a in two patients. In 3 patients no invasive carcinoma was identified so it had been excised totally by electroresection. In 8/9 patients, SLN was identified. Metastases to the lymph nodes were observed in 5 patients, among them in 1 without detectable SLN. In each case, tumors with N+ lymph nodes contained SLN metastases (4/4, 100 %).

**Conclusion:** Our initial observations confirms value of identification and evaluation of SLN in cystectomy.

**PS-25-049**

**Melanoma of urethra**

I.-P. Efstratiou ^*^, E. Pazarli, S. Pervana

^*^Thessaloniki, Greece

**Objective:** We describe a case of a 72-year old woman presenting with hematuria.

**Method:** Physical examination and cysteoscopy showed a polypoid tumor in the urethra which diagnosed and managed as a caruncle and cauterize without histological examination. The woman after few months presented to the urological department of our hospital with the same symptoms. There was found a urethral mass which now excited.

**Results:** Histologicaly the tumor was composed by epithelioid cells with eosinophilic cytoplasm and abnormal vesicular nucleus with prominent nucleoli. Immunohistochemically tumour cells showed strong reactivity for vimentin, melan A and HMB-45 and were negative for keratins. So the diagnosis of malignant melanoma of the urethra was made.

**Conclusion:** Primary malignant melanoma of the urethra is an aggressive neoplasm associated with a poor prognosis and few cases have been published. The outcome is dependent on early diagnosis and surgical intervention. The diagnosis is often delayed because the tumor has clinically similarity with other urothelial neoplasm so we must have this diagnosis in our mind.

**PS-25-050**

**Eosinophilic cell renal cell carcinoma: Clinicopathologic and immunohistochemical profiling analysis of 22 cases**

F. Khanchel-Lakhoua ^*^, R. Dhouib, K. Mrad, I. Abbes, R. Doghri, L. Charfi, M. Driss, S. Sassi, H. Haouari, K. Ben Romdhane

^*^Salah Azaiez Institute, Pathology, Tunis, Tunisia

**Objective:** Renal cell carcinoma (RCC) is a heterogenous group with distinct histopathologic features, molecular characteristics and clinical outcome. Eosinophilic renal cell carcinoma include chromophobe RCC, epithelioid angiomyolipoma, juxtaglomerular cell tumor and clear cell RCC with eosinophilic component. The aim of our study was to investigate the morphological features and immunohistochemical phenotypes of eosinophilic cell RCC.

**Method:** We analyzed cases of of RCC with eosinophilic cells registered in the Department of Pathology Salah Azaiez Institut from 1992 to 2012.

**Results:** Were registred 22 cases. The histological spectrum included: chromophobe RCC (5/22), oncocytoma (3/22), clear cell RCC with eosinophilic cell component (11/22) and xp11.2 associated RCC (3/22). The mean age of patients was 65 years. Three cases of Xp11.2 translocation associated RCC are seen in young patients. The tumors lacked cytokeratin expression and were confirmed by nuclear reactivity to TFE3 protein. In the 11 cases of clear RCC the eosinophilic cell component was minority. Five tumors were classified as having Fuhrman grade 2 histologic features, five had grade 3 and one had grade 4.

**Conclusion:** The large eosinophilic renal cell carcinoma group of RCC contain morphological and molecular distinct tumor with different prognosis. Increases awareness and a high index of suspicion are key of the correct recognition of the entities.

**PS-25-052**

**Precancerous lesions of the prostate: Biological features and cancer risk**

V. Zakharava ^*^, T. Liatkouskaya, E. Cherstvoy

^*^Belarus State Medical University, Pathology, Minsk, Belarus

**Objective:** Prostatic intraepitelial neoplasia (PIN) and atypical small acinar proliferation (ASAP) has a high predictive value as a markers for prostate cancer (PCa).

**Method:** PCa-risk in patients with precancerous lesions has been assessed on biopsies material in patients having morphological suspicious to PCa. The expression of the АМАСR, high-weight molecular cytokeratine (HWC) (clone 34βE12), р63, cyclin D1,topoisomeraseIIa, TGFβ was estimated with the use of immunostaining. Suspicious foci were estimated with use of cocktail AMACR+HWC+p63. For the images analysis the set of software WCIF ImageJ and ImageScope was used.

**Results:** According to our results a finding of precancerous lesions in biopsies specimens has been associated with PCa on re-biopsy. Within the first-5-years the overall incidence of PCa on re-biopsy makes 27 % in the group of precancerous lesions. Life-time-without-PCa median made 5-years with no reliable difference between PIN and ASAP groups. Thus, the cumulative share of patients without PCa in the ASAP group decreased from 86 % to 60 % during these 5 years. Biological properties of precancerous processes were similar to the PCa and were characterized by increase of expression of AMACR and cell cycle markers (cyclin D1 and topoisomeraseIIa) along with decrease of antiproliferative action of TGFβ in comparison with hyperplasic processes (*p* < 0,05).

**PS-25-053**

**Laser microdissection coupled with RT-PCR identifies differential gene expression in glands and stroma of prostate cancer tissues**

M. Decaussin-Petruci ^*^, V. Cheynet, G. Oriol, A. Loghin, K. Castellano, A. Ruffion, P.-P. Bringuier, C. Rodriguez-Lafrasse, F. Mallet

^*^Lyon Sud Hospital, HCL, lyonI, Pathology, Pierre Benite Cedex, France

**Objective:** One feature of prostate cancer consists in the invasion of the underlying mesenchymal compartment by malignant glandular epithelial cells, such heterotypic cell-cell interaction contributing to tumor expansion. In order to systematically and architecturally identify gene expression patterns, we evaluate the usefulness of laser-based micro dissection (LMD) coupled with RT-PCR as a reference molecular process.

**Method:** Frozen tissue samples were obtained from radical prostatectomy. LMD was performed to isolate normal glands and adjacent stroma as well as tumor glands and adjacent stroma, from the same patient. RNAs isolated from all four compartments were qualified using comparative quantitative RT-PCR amplification of 10 genes.

**Results:** PSA and CDH1 qualified the glandular origin of mRNA as CNN1 and ACTA2 reflected stromal origin, independently of the differentiation state of the compartments. Conversely, DDP4 and p63 were down-regulated in the tumor glands whereas AMACR and PCA3 were up-regulated. In the tumor-associated stroma, CD90 and PENK were found up- and down-regulated, respectively.

**Conclusion:** LMD is a method of choice to sample cell populations of all four glandular and stromal compartments and carry out gene expression analysis. High throughput transcriptomic studies will be performed to decipher the interactions between these compartments and define a “gland plus stroma”-based molecular Gleason score.

**PS-25-054**

**Association of glutathione s-transferases M1 and T1 polymorphisms with clinicopathological parameters in prostate cancer**

U. Berber^*^, I. Yilmaz, O. Yilmaz, A. Haholu, Z. Kucukodaci, D. Demirel

^*^GATA Haydarpasha Training Hosp, Pathology, Istanbul, Turkey

**Objective:** We aimed to investigate the association between glutathione s-transferase (GST)-M1 and GSTT1 polymorphisms and histopathologic parameters in radical prostatectomy specimens.

**Method:** We developed a multiplex polymerase chain reaction and high resolution melting curve analysis method to screen GSTM1 and GSTT1 genotypes simultaneously in a single tube. Formallin fixed paraffin embedded archival tissues from 162 radical prostatectomy specimens were included in the study. Patient age, Gleason score, tumor volume, seminal vesicle invasion, perineural invasion, extraprostatic extension, and pathological tumor stages were analysed in different genotype groups.

**Results:** The frequencies of GSTM1 null genotypes were 50.6 % (82/162), and GSTT1 null genotypes were 20.4 % (33/162). When compared to GSTM1 present genotype, perineural invasion (75.6 % vs 65.0 %) and extraprostatic extension (9.8 % vs 6.3 %)were slightly more common in GSTM1 null genotype carriers, but did not reach the significancy level. No any differences were found between GSTT1 genotype groups and selected clinicopathological para meters.

**Conclusion:** The present study suggests that inherited absence of GSTM1 and GSTT1 genes are not associated with tumor characteristics in prostate cancer. However, further projects with exact genotyping are required to distinguish homozygote and heterozygote individuals to clarify their association with prognostic parameters in prostate cancer.

**PS-25-056**

**Proteomics in testicular lymphoma: Case report**

I. Poinareanu ^*^, M. Aschie, A. F. Mitroi, M. Enciu

^*^Ovidius University, Medicine–Pathology, Constanta, Romania

**Objective:** Primary non-Hodgkin’s lymphoma of the testis is an uncommon disease and that represent 1 %–2 % of all non-Hodgkin lymphomas. It accounts for about 9 % of testicular neoplasms. Histologically, 80 % to 90 % of primary testicular lymphomas are diffuse large-cell type with B cell phenotype, but isolated cases of other histological subtypes have been described such as Burkitt and burkitt’s-like types in 10–20 % of cases, mainly in HIV+ patients.

**Method:** In this paper we present a case report with primary non-Hodgkin testicular lymphoma in one 34 years old patient. The particularities of case are the age of patient and tumor morphological features witch can be confused with undifferentiated seminoma.

**Results:** Differential diagnosis is made by immunohistochemistry using specific antibodies for those situations: PLAP, AFP or OCT3/4 in embryonar carcinoma and CD19, CD20 and CD45RO in B cell lymphoma. Using immunohistochemistry we demonstrated tumor membership category lymphomas.

**Conclusion:** In conclusion, this paper demonstrates the usefulness of immunohistochemistry in pathology diagnoses especially in locating rare diseases at several levels.

**Undifferentiated testicular tumor:** 
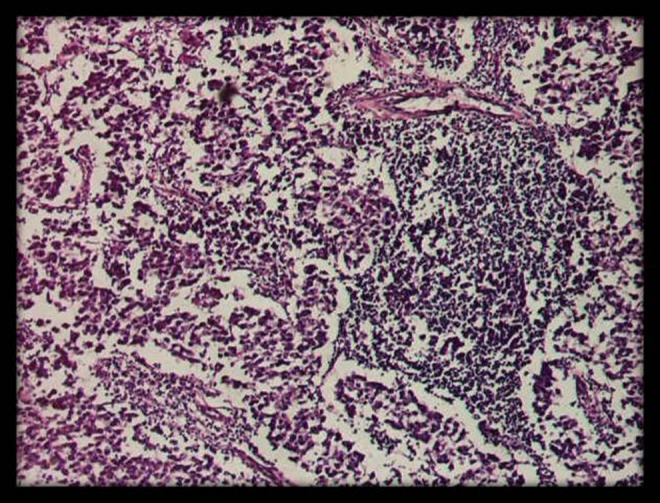


**PS-25-057**

**Low expression levels of miRNA 200b are related to pT3 and high Gleason score in prostate cancer**

B. Katz ^*^, S. Reis, N. Dip, N. Viana, D. Morais, I. Silva, M. Srougi, K. Ramos Moreira Leite

^*^Universidade de São Paulo, Brazil

**Objective:** The ZEB/miR200 loop plays a fundamental role in epitelial-to-mesenchymal transition. However, knowledge is scant about this loop in prostate cancer. Our objective is to assess the function of this loop in prostate cancer.

**Method:** Fifty-one fresh-frozen samples from patients with localized prostate submitted to radical prostatectomy were selected. Expression levels of E-cadherin, ZEB1, ZEB2, TGFβ1, miR-200b and miR-429 were assessed using qRT-PCR. We compared the genes and miRNAs expression with Gleason score (GS), pathological staging (pT), preoperative PSA and biochemical recurrence.

**Results:** miR200b, miR429, E-cadherin and ZEB2 were overexpressed in the majority of patients, while TGF-β1 and ZEB1 were predominantly underexpressed. miR-200b levels were significantly lower in pT3 patients comparing to pT2 (7.73 vs 23.86, *P* = 0.02), and in patients with GS ≥ 8 comparing to patients with GS ≤ 6 (9.94 vs 18.67, *P* = 0.035). No association was observed regarding miR-429 and the genes with the clinicopathological parameters.

**Conclusion:** This is the first study to show that patients with pT3 prostate cancer and high GS have lower levels of miR200b. Stage and GS are the most important predictive factors for cancer progression. Our results suggest that miR200b might contribute to this process, and could be a potential prognostic marker in prostate cancer.

**Expression profile of the genes and miRNA in 51 patients with prostate cancer:** 
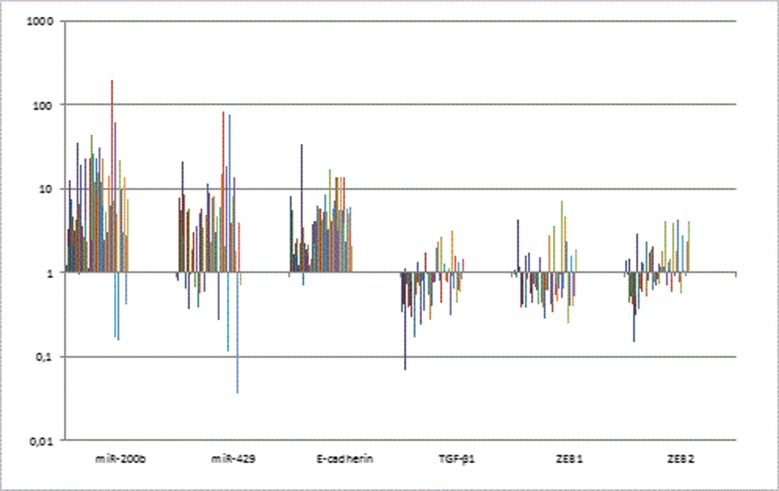


**PS-25-058**

**Bladder cancer in Tunisia: Epidemiological and clinicopathological features**

S. Hmissa ^*^, N. Missaoui, S. Korbi, H. Landolsi, M. Njima, L. Jaidaine, N. Ben Sorba, M. Mokni

^*^Farhet Hached Hospital, Dept. of Pathology, Sousse, Tunisia

**Objective:** The aim of this study was to survey for the first time the burden and characteristics of bladder cancer in Central Tunisia

**Method:** Characteristics of all bladder cancer cases diagnosed during a 15-year period were analyzed based on the data of the Cancer Registry of the Center of Tunisia

**Results:** A total of 1023 new cases of bladder cancer were recorded. The ASR was 15.1 per 100,000 for men and only 1.9 for women. The median age at diagnosis was 68 years (21–93). The tumor size was higher than 2 cm in 80.7 % of cases. Early stages (T1 and T2) represented 60.7 % followed by advanced stages (30.2 %). Papillary transitional cell carcinoma was the most frequent histological type (84.3 %) followed by transitional cell carcinoma, NOS (7.4 %). Grade II tumors were the most frequent tumors (43.2 %) followed by grade III (34.4 %)

**Conclusion:** Bladder cancer remains the most important urological cancer diagnosed among men in central Tunisia. These findings justify the need to plan and develop effective programs aiming at the control of the spread of cancer in Tunisia

**PS-25-059**

**Urothelial tumors of upper urinary tracts: A retrospective study of 66 cases**

S. Hmissa ^*^, N. Missaoui, S. Korbi, T. Tlili, M. Njima, N. Labaied, L. Jaidaine, M. Mokni

^*^Farhet Hached Hospital, Dept. of Pathology, Sousse, Tunisia

**Objective:** Urothelial tumors of upper urinary tracts were rare. Here, we analyzed their clinicopathological and prognostic factors.

**Method:** We used a retrospective study of 66 cases diagnosed between 1993 and 2008 and immunohistochemesty to analyze the expression of p53, p27, E-cadherin, and Ki-67.

**Results:** There were 48 men and 18 women (sex-ratio: 2.7). The median age was 68. All cases were of papillary transitional cell type. The majority of tumors was grade 2 (41 %) and grade 3 (29 %). The anatomoclinical stage was PT3 (35 %) and PT2 (29 %). The immunohistochemical staining was positive with p53 (36 %), Ki-67 (80 %), p27 (90 %) and E-cadherin (100 %). The age was a prognostic factor of survival for patients aged ≤60 years (77 %) and patients aged ≥65 years (50 %). Sex and tumor site did not influence survival. Tumor stage and grade remains always valid and reliable prognostic factors. Surgical limit was a good prognostic factor (*p* = 0.005): survival was 70 % for healthy surgical limits and only 22 % for tumoral surgical limits. The expression of Ki-67, p27 and E-cadherin was associated to invasive tumors (>PT1) and advanced tumor grade (2 and 3).

**Conclusion:** In addition to tumor stage and grade, the surgical limit may constitute a prognostic factor influencing the survival without second recurrence.

**PS-25-060**

**Inflammatory myofibroblastic tumor of scrotum and inguinal region: A case report**

C. I. Bassorgun*

^*^Akdeniz University, Pathology, Antalya, Turkey

**Objective:** Inflamatory myofibroblastic tumor (IMT), is an intermediate-grade neoplasm with potential for recurrence and rare metastasis. IMT of the scrotum and inguinal region is a rare lesion of unknown etiology.

**Method:** We report the case of a 78 year-old patient who presented clinically with a palpable scrotal and inguinal mass after a 6 month history of orchiectomy because of prostatic adenocarcinoma in 2012.

**Results:** He underwent urologic surgery and the following histopathologic, immunohistochemical and fluorescence in situ hybridization (FISH) evaluation revealed an IMT. The tumor was composed of predominantly myofibroblastic spindle cells embedded in a fibrous stroma with inflammatory cells. On immunohistochemical analysis, were positive for ALK and desmin, a finding that is consistent with a myofibroblastic origin. FISH with the use of break-apart probes showed ALK rearrangement.

**Conclusion:** We present the first case of IMT of the postorchiectomy scrotal and inguinal mass being reported in the literature

**PS-25-061**

**pT3b adenocarcinoma of the prostate: A clinico-pathological study of 100 cases**

F. Fraggetta ^*^, P. Pepe, V. Simeon, G. Improta, M. G. Tranchina, C. Emmanuele, F. Aragona, A. Galia, M. Colecchia

^*^AOE Cannizzaro, Pathology Unit, Catania, Italy

**Objective:** We retrospectively evaluated the clinico-pathological findings of 100 cases of adenocarcinoma of the prostate stage pT3bN0 (62) and pT3bN1 (38). Of the latter group 22/38 cases showed 1 or 2 positive nodes whereas in the remaining 16/38 cases there were more than 2 metastatic nodes.

**Method:** We evaluated which preoperative clinical and pathological findings were predictive of PSA nadir value >0.2 ng/ml (persistence of malignancy). Univariate analysis was conducted using the Fisher test. The following parameter were tested: preoperative PSA; bioptic Gleason score (GS); Greatest Percentage of Cancer (GPC); Total Percentage of cancer (TPC); initial vs repeat biopsy; T1c vs T2 clinical stage.

**Results:** A TPC equal or superior to 20 % the T2 clinical stage and a GS equal or superior to 9 were predictive (*p* < 0.01) of postsurgical persistence of malignancy. All the other parameters failed to forecast persistence of malignancy (*p* > 0.05). When post surgical pathological findings were considered the N1 and the GS equal or superior to 9 were predictive (*p* < 0.001) of persistence of malignancy. Positive surgical margins status was not related to the postsurgical PSA nadir value.

**Conclusion:** cT2, a GS equal or superior to 9 and a TPC equal or superior to 20 % strongly predict persistence of malignancy in the pT3 adenocarcinoma of the prostate.

**PS-25-062**

**CD44 correlates with MMP-9 in prostate carcinoma**

G. Dordevic ^*^, S. Stifter, I. Hadzisejdic, V. Mozetic, R. Oguic, E. Mustac

^*^School of Medicine, Pathology, Rijeka, Croatia

**Objective:** CD 44 is the adhesion molecule shown to be a useful prognostic marker of aggressive prostate cancer. Also, CD44s accumulates metalloproteinase-9 (MMP-9) on the cell membrane which allows angiogenesis and invasion. The aim of this study was to investigate the expression of CD44 in prostate cancer specimens obtained by prostatectomy and to correlate it with MMP-9 expression.

**Method:** Tissue microarrays (TMA) of 120 archival, formalin fixed paraffin embedded prostate carcinoma were immunohistochemically evaluated for CD44s and MMP-9 expression.

**Results:** Median CD44 expression was 52.1713 (SD ± 48.6701) with non significant correlation with clinicopathological parameters, stage, margin status or reccurence free survival. CD44 molecule shows loss of expression in tumor cells, discontinuous membrane staining and focal irregular distribution in tumors with higher Gleason score. CD44 and MMP9 showed inverse correlation (*p* = 0.0198).

**Conclusion:** Our results showed loss of expression of CD44 in poorly differentiated tumors, while correlation with MMP9 needs further evaluation.

**PS-25-063**

**A case report, pathogenesis and literature review of a borderline intratesticular papillary serous cystadenoma of ovarian type**

K. Quinlan^*^, M. Leader

^*^Booterstown, Dublin, Ireland

**Objective:** Clinical Background: Ovarian type epithelial tumours of the testis are extremely rare. Diagnosis is challenging as they can be confused with malignant tumours.

**Method:** In this case report, such a neoplasm in a male of 64 years is described and the literature is reviewed.

**Results:** The patient had a history of right orchidectomy for cryptorchidism at 25 years of age. He presented with a hydrocoele of the left hemiscrotum. After hydrocoele repair and left inguinal orchidectomy for a suspected cystic tumour, a borderline intratesticular papillary serous cystadenoma of ovarian type was confirmed.

**Conclusion:** This case is the first intratesticular serous tumour of ovarian type in which focal areas similar to endometrial surface syncytial change is seen. It is also the first in which atrophy of the seminiferous tubules is described. Immunohistochemical features point to a Mullerian epithelial origin.

**Borderline Intratesticular Papillary Serous Cystadenoma of Ovarian Type:** 
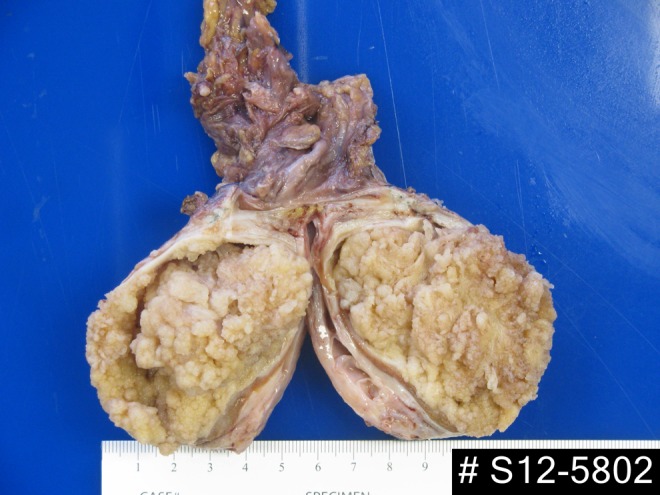


**PS-25-064**

**Micropapillary carcinoma of the urinary bladder: A rare and aggressive variant. A case presentation**

A. Loghin ^*^, C. Chibelean, O. Martha, A. Borda

^*^University of Medicine, Histology, Tirgu Mures, Romania

**Objective:** Micropapillary urothelial carcinoma is a rare variant of urothelial carcinoma with an aggressive clinical course. Usually it has an advanced stage at first presentation, with a high metastatic potential and a poor outcome. Its distinction from conventional urothelial carcinoma is crucial for a correct therapeutic decision.

**Method:** We report the case of a 66-year-old man who presented with haematuria. A transurethral resection was performed which showed an infiltrative pT1 urothelial carcinoma with a small component of micropapillary growth pattern. Radical cystoprostatectomy (RC) with lymph node dissection was performed subsequently.

**Results:** The pathological examination of the RC specimen revealed a conventional high grade papillary urothelial carcinoma, with a single small infiltrative component in the bladder neck. This infiltrative component had the architecture (nests and papillae surrounded by stromal reaction) and the morphology (abundant eosinophilic cytoplasm and nuclei with prominent nucleoli) of a micropapillary urothelial carcinoma. The same micropapillary pattern was detected in one of the ilio-obturator lymph node metastasis.

**Conclusion:** Micropapillary carcinoma is a highly aggressive variant of high grade urothelial carcinoma with specific morphological characteristics. Any amount of micropapillary component in the urothelial carcinoma should be reported and should alert urologists to apply an aggressive therapy.

**PS-25-065**

**Mixed Epithelial and Stromal Tumor of the kidney (MEST): 2 new cases diagnosed in the last 20 years of this poorly known and controversial entity**

C. Rivero Colmenarez ^*^, A. Rodríguez García, I. Gómez de la Riva, A. Serrano, M. L. Picazo García, P. González-Peramato Gutierrez

^*^Hospital Universitario La Paz, Dept. de Anatomia Patologica, Madrid, Spain

**Objective:** MEST, a rare and benign tumor with unknown origin that was first reported in 1998 and previously described under various nomenclatures. It is said that a hormonal pathogenic mechanism is involved, since most cases occur in middle-age/perimenopausal women or men with a history of hormone therapy. We report 2 new cases diagnosed in the last 20 years.

**Method:** The first case was a 53- year old woman with a 45 mm incidental renal mass. The second one was a 44- year old woman with a history of low back pain with a 15 mm right renal mass.

**Results:** Microscopic findings revealed a biphasic growth pattern comprising a mesenchymal element, consisting of spindle cells that mimic ovarian stroma and an epithelial element lining cysts with a particularly inmunohistochemical profile expression of estrogen and progesterone receptors. One case also expressed androgen receptors.

**Conclusion:** MEST clinical presentation is variable. Malignant sarcoma associated, local recurrence and a t(1;19) translocation, have been reported. Cystic nephroma is its most complex differential diagnosis and it is known that they could be a variant of the same kind of tumor called Renal Epithelial and Stromal Tumors (REST), however, further evidence is needed in order to classify this entity.

**Mixed epithelial and stromal tumor of the kidney:** 
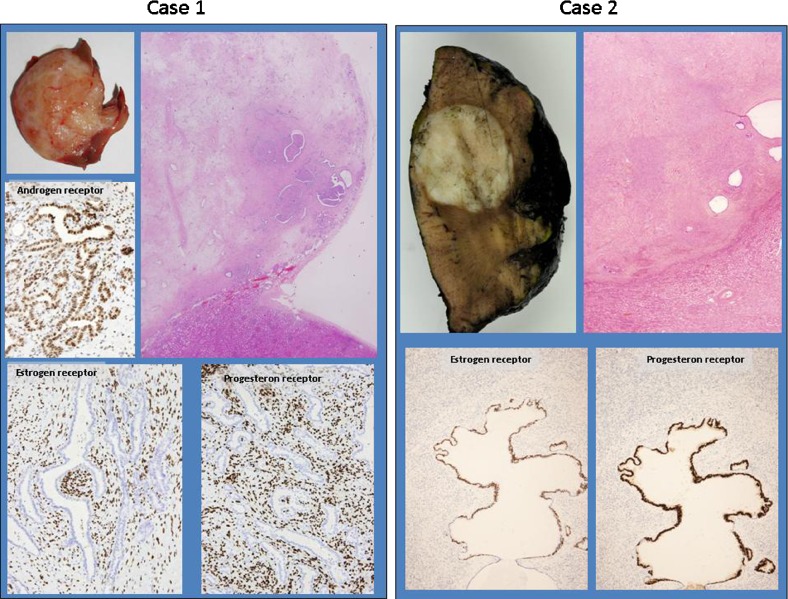


**PS-25-066**

**Paratesticular leiomyosarcoma: A case report in a young patient**

F. Costa ^*^, A. Coelho, J. R. Vizcaíno, O. Lima

^*^Centro Hospitalar do Porto, Braga, Portugal

**Objective:** Paratesticular leiomyosarcomas (PL) are rare lesions seen in all age groups, with a peak incidence in the sixth and seventh decades. It is often difficult to determine with certainty the exact site of origin of paratesticular tumours. However, it is thought that PL originates from testicular tunica, spermatic cord, epididymis, dartos muscle and subcutaneous tissue of scrotum.

**Method:** We report a case of a 18-year-old man with an unremarkable medical history who presented to our institution with left testicular pain and swelling. Ultrasound image revealed a highly vascularized neoplasm and the patient was subjected to radical orchiectomy.

**Results:** Histologically, the tumor was diagnosed as a grade 2 (Federation Nationale des Centres de Lutte Contre le Cancer), well-differentiated PL, with high mitotic activity, focal necrosis and mild or absent cytological atypia. The patient underwent adjuvant chemotherapy and radiotherapy. On follow-up, several pulmonary and retroperitoneal masses were detected and the patient died 16 months after initial diagnosis.

**Conclusion:** PL have an extremely variable prognosis, with a wide spectrum of behavior. Histologic grading, based on the evaluation of necrosis, mitotic activity and degree of differentiation, provides useful prognostic information. Although most examples are low-grade, high-grade lesions are aggressive and develop distant metastasis, albeit aggressive systemic therapy.

**PS-25-067**

**Osseous metaplasia of the urinary bladder**

C. Magkou ^*^, D. Riga, M. Chantziara, P. Constantinou, M.-A. Lianou, M. Strataki

^*^Evaggelismos General Hospital, Pathology, Cholargos, Athens, Greece

**Objective:** Bone formation outside the human skeleton is a well-known process; it is however rare in the urinary tract. Animal experiments have shown that osseous metaplasia of the urothelium may be due to ischemia, inflammation, necrosis, sclerosis and trauma. Even though the urothelium is known to have osteoinductive properties in animals, osseous metaplasia is seldom seen in humans.

**Method:** A 77 years old male patient admitted to our hospital with macroscopic hematuria. An exophytic lesion was found and excised during cystoscopy. A 0.6 cm specimen was sent to our department.

**Results:** Histopathologic examination revealed ulceration of the urothelial surface with inflammatory granulation tissue formation, as well as foci of metaplastic ossification embedded in the lamina propria. The reserved urothelium exhibited reactive atypia.

**Conclusion:** The changes observed in the bladder wall could be explained as the result of chronic inflammation. The latter leads to the release of cytokines and growth factors, and may induce the formation of metaplastic bone.

**PS-25-068**

**The effect of testosterone on angiogenesis in prepucial and urethral plate tissue in hypospadias**

C. Magkou ^*^, D. Violidaki, D. Riga, E.-A. Nikolaraki, M. Chantziara, M. Strataki

^*^Evaggelismos General Hospital, Pathology, Cholargos, Athens, Greece

**Objective:** To study the effect of testosterone in healing process after hypospadias surgery.

**Method:** Fifteen boys mean age 3.2 years old, with penoscrotal hypospadias underwent urethroplasty. In 8 pts (group A) testosterone was given i.m. preoperatively. The rest (group B) underwent hypospadias repair without androgens stimulation. Intraoperatively, tissues samples were taken from prepuce (PR) and urethral plate (UP). Immunohistochemistry was applied using anti-CD31, anti-VEGF, anti-MMP2 and anti-MMP9 antibodies.

**Results:** PR: Microvessel count was positively related to the intensity of immunoexpression of MMP-2 positive cells (*p* = 0.041) and MMP-9 positive vessels (*p* = 0.038) and negatively related to the stromal immunoexpression of VEGF (*p* = 0.016) and MMP-2 (*p* = 0.044), the number of MMP-9 positive cells (*p* = 0.027) and the intensity of immunoexpression of MMP-9 positive cells (*p* = 0.027). UP: Microvessel count was positively related to the number of MMP-9 positive cells (*p* = 0.005) and negatively related to the stromal immunoexpression of VEGF (*p* = 0.015). Regarding testosterone, the number of MMP-9 positive cells in PR samples was higher in group A than in group B (*p* = 0.010).

**Conclusion:** Ιt seems that angiogenesis induced by VEGF, MMP-2 and MMP-9 is more apparent in PR samples. Τestosterone, possibly via up regulating MMP-9, may affect this process.

**PS-25-069**

**Age influence in BBN induced urothelial lesions in ICR male mice**

C. Vasconcelos-Nóbrega^*^, C. Mega, R. Arantes, D. Talhada, C. Teixeira-Guedes, A. Faustino-Rocha, H. Vala, P. A. Oliveira

^*^Agrarian School of Viseu, Quinta da Alagoa, Est Nelas, Portugal

**Objective:** To study the influence of age on BBN induced urothelial lesions in ICR male mice.

**Method:** 42 ICR male mice were divided in four groups. Group I and III were subjected to BBN’s action (12 weeks) at different ages (I: 5 weeks old; III: 18 weeks old). Group II and IV (control groups), didn’t receive BBN (drunk only tap water).

**Results:** All animals subjected to BBN, developed urothelial lesions, in higher percentage in the younger group (I). The spectrum of urothelial lesions identified were: simple hyperplasia (I: 67 %; III: 50 %); nodular hyperplasia (I: 58.3 %; III: 30 %); dysplasia (I: 100 %; III: 70 %); carcinoma in situ (I: 3 %; III: 0 %), invasive carcinoma (I: 33 %; III: 20 %) and epidermoid meptaplasia (I: 67 %; III: 60 %).

**Conclusion:** Age-related decline in physiological functions, are likely to put older individuals at increased risk for tumorigenesis. This study suggests resistance to BBN in older animals, when the most frequent experimental conditions are applied.

**PS-25-070**

**Quantitative measurements of cell density and proliferative marker (Ki-67) by image analysis of renal cell carcinoma**

A. Kudaybergenova ^*^, N. Gorban

^*^RNCRCT, Pathology, St. Petersburg, Russia

**Objective:** We analyzed a total number of tumor cells in renal cell carcinoma and proliferative activity (% Ki67-positive cells) to establish absolute quantity of tumor cells per sq.mm per histological slide and relates this measure with proliferation.

**Method:** The study included 45 patients diagnosed with RCC. After whole slide scanning by the Mirax scanner (3DHistech, Budapest) of Ki67 stained slides, we located the areas within the tumors with maximal ki67 levels and calculated the precise quantity of tumor cells per sq.mm, which was the sum of negative and Ki67 positive cells. Morphometric analysis was performed using the Pannoramic Viewer software (3DHistech, Budapest). For each case we analyzed a total number of tumor cells in a 1 mm2 sample of Ki67 was evident in at least 10 fields of view.

**Results:** Mean tumor cells in 1 mm2 of histology slide was 4,389 +\\− 229 cells, only 11.62+/−1.65 % were positive for Ki67. There was no correlation between cell density and Ki67 *r* = 0,005 (*p* = 0,0032)

**Conclusion:** By analysis of RCC, was established the total quantity of tumor cells per mm2 and found no correlation between cell density and proliferation.

**PS-25-071**

**Ki-67 labeling index evaluation in urothelial bladder carcinoma**

E. Goupou ^*^, A. Nikolaidou, G. Moustakas, V. Theodorou, A. Giannouli, E. Velkopoulou, N. Dimasis

^*^Theagenion Cancer Hospital, Pathology, Thessaloniki, Greece

**Objective:** The purpose of this study is to evaluate ki-67 immunohistochemical overexpression in urothelial bladder carcinoma. Ki-67 protein is a cellular proliferation marker that is encoded by the MKI67 gene. It has been associated with histological grade and prognostic parameters as relapse, tumor progression, metastasis.

**Method:** 20 bladder biopsy and cystectomy specimens were included, viewing histological grade and pathological stage. The patients’ median age is 71, 16 (80 %) male and 4 (20 %) female. Among them 10 were high- and 10 low-grade and 10 pTa, 6 pT1, 3 pT2 and 1 pT4. Ki-67 was considered as positive when nuclear immunoreactivity exceeded 20 %.

**Results:** ki-67 positivity was observed in 8 (40 %) cases; 7 (35 %) high-grade and 1 (5 %) low-grade cases, while 12 (60 %) cases were negative; 3 (15 %) high-grade and 9 (45 %) low-grade. Concerning the pathological stage, positive were 1 pTa (5 %), 4 pT1 (20 %), 3 pT2 (all pT2) (15 %), while negative were 9 pTa cases (45 %), 2 pT1 (10 %) and the 1 pT4 (5 %).

**Conclusion:** The results of this study are challenging, corresponding to bibliographic data. Surveys’ outcomes are conflicting so far among series. Further and larger studies, together with patients’ follow-up, need to be conducted to evaluate ki-67 as a clinically useful biomarker.

**PS-25-072**

**P53 evaluation in urothelial bladder carcinoma**

E. Goupou ^*^, G. Moustakas, A. Nikolaidou, I. Michalopoulou Manoloutsiou, A. Klampatsas, G. Sarakapinas, N. Dimasis

^*^Theagenion Cancer Hospital, Pathology, Thessaloniki, Greece

**Objective:** The purpose of this study is to evaluate p53 immunohistochemical overexpression in urothelial bladder carcinoma. p53 protein is a cell cycle regulator that functions as a tumour suppressor and is encoded by the TP53 gene. It has been associated with histological grade and prognostic parameters as recurrence, tumor progression, metastasis-free interval.

**Method:** 20 bladder biopsy and cystectomy specimens were included, viewing histological grade and pathological stage. The patients’ median age is 71, 16 (80 %) male and 4 (20 %) female. Among them 10 were high- and 10 low-grade and 12 pTa, 6 pT1 and 2 pT2. p53 was considered as positive when 10 % of nuclear immunoreactivity was attained.

**Results:** p53 positivity was observed in 16 (80 %) cases; all 10 (50 %) high-grade and 6 (30 %) low-grade cases, while 4 (20 %) cases were negative (all 4 were low-grade). Concerning the pathological stage, positive were 8 pTa (40 %), 6 pT1 (all pT1) (30 %), 2 pT2 (all pT2) (10 %), while all 4 negative cases (20 %) were pTa.

**Conclusion:** There is a good correlation between p53 immunoreactivity and histological grade and an even better between p53 and pathological stage. Further and larger studies, together with patients’ follow-up, need to be conducted to evaluate p53 as a clinically useful prognostic biomarker.

**PS-25-074**

**Prostatic cystadenoma: A case report**

R. de Mora Féria ^*^, A. Furtado, M. C. Alves, P. Cardoso, S. Loureiro dos Santos

^*^Hospital Dr. Fernando Fonseca, Anatomia Patológica, Lisbon, Portugal

**Objective:** Prostatic cystadenoma is a rare and controversial entity, first described by Maluf et al. in 1991. Since then, 26 cases have been described.

**Method:** Case report: We present a case of a 49 year old man, with prostatic specific antigen (PSA) of 15 ng/mL and a retrovesical tumour with no connection to the prostate gland or bladder. It was a cystic multilocular tumour with 13,5 cm diameter. Microscopically, in a hypocellular stroma, there were some dilated hyperplastic glandular structures and cysts, both lined by two layers: internal of epithelial cubic to cylindrical cells, with basal nuclei, no atypia, PSA (+) and external of myoepithelial cells CK903 (+). There was focal low and high-grade prostatic intraepithelial neoplasia (PIN).

**Results:** The main differential diagnosis is ectopic prostate tissue with nodular hyperplasia, which is another rare entity, particularly in this localization. PIN and prostate adenocarcinoma have been described in both entities. In our case, the size, location and hypocellular stroma favours the diagnosis of cystadenoma.

**Conclusion:** Prostatic cystadenoma is a rare and benign but locally aggressive tumour. It is important to recognise it because it recurs if incompletely excised and may cause adhesions to other organs and structures.

**PS-25-075**

**Synchronous Large Cell Calcifying Sertoli Cell Tumor (LCCSCT) and malignant Leydig cell tumor of the testis: A case report**

D. Koumoundourou^*^, H. Geropoulou, M. Ghermpesi, C. Aletra, P. Ravazoula

^*^University Hospital of Patras, Pathology, Greece

**Objective:** LCCSCT is a very unusual variant of Sertoli cell tumor and usually occurs in young patients with Carney’s syndrome, or, less frequently in patients with Peutz-eghers syndrome. On the other hand, Leydig cell tumor is a rare neoplasm which in most of the cases has a benign clinical course.

**Method:** We report an uncommon case of testicular LCCSCT developing synchronously with a malignant Leydig cell tumor. A 25 years-old male patient presented with unilateral testicular enlargement. There were no other clinical symptoms or … findings. A right radical orchectomy was performed and the specimen was received in the pathology lab.

**Results:** Grossly, two separate tumors were recognized measuring 1 and 6 cm with a fleshy white appearance. Both tumors were partly encapsulated, while the small one had multiple calcifications. Microscopally the smaller tumor had the characteristics of Calcifying Sertoli Cell Tumor while the largest one was a malignant Leydig cell tumor.

**Conclusion:** Large Calcifying Sertoli Cell Tumor (LCCSCT) and malignant Leydig cell tumor is a peculiar neoplasm with no existing therapeutic and prognostic information

**PS-25-076**

**Sphingosine kinase-1 expression is associated with prostate cancer specific mortality: A potential new prognostic biomarker?**

U. Axcrona ^*^, L. Vlatkovic, T. Sæter, E. Servoll, G. Waaler, C. Mazerolles, S. Pitson, B. Malavaud, J. M. Nesland, O. Cuvillier

^*^Dept. of Pathology, The Norwegian Radium Hospital, Oslo, Norway

**Objective:** To examine the expression of the oncoenzymes sphingosine kinase-1 (Sphk1) and sphingosine1-phosphate lyase (SPL) in human prostate cancer (PCa) with correlation to clinicopathologic parameters and clinical outcome.

**Method:** Paraffin-embedded prostatectomy specimens from 133 PCa patients treated with radical prostatectomy were analysed by immunohistochemistry for SphK1 and SPL.

**Results:** SphK1 was demonstrated to varying degrees in both cytoplasm and nucleus of tumour cells as well as in tumour stroma. The expression of cytoplasmic SphK1 was significantly correlated to high Gleason score (GS) (*P* = 0.008) and locally advanced/pT3b PCa (*P* = 0.016). Kaplan-Meier analysis revealed a trend for increased biochemical and clinical failure rates at high levels of SphK1 and a significant negative prognostic impact on PCa specific survival (*P* = 0.036). Nuclear and stromal SphK1 were not found to correlate with any clinicopathologic parameters or prognosis. SPL was restricted to the cytoplasm and inversely correlated to SphK1 and GS.

**Conclusion:** We report the first finding of SphK1 as a possible prognostic biomarker for PCa specific mortality. Our findings demonstrate that increased levels of SphK1 together with low levels of SPL act as negative predictors for PCa outcome. One could speculate that SphK1 expression is associated with the terminal phase of epithelial mesenchymal transition.

**PS-25-077**

**PSMA expression in prostate carcinomas**

A. Dema ^*^, S. Taban, A. Borda, C. Lazureanu, E. Lazar, S. Ursoniu, D. Anderco, D. Herman, A. Loghin

^*^University of Medicine and Pha, Pathology, Timisoara, Romania

**Objective:** The aim of the present study was to investigate the PSMA expression in prostate carcinomas.

**Method:** PSMA expression was analyzed in 3 groups of prostate carcinoma: localized, locally advanced and metastatic. The Gleason score was used for grading and the tumors were subsequently classified in prognostic subgroups. For the IHC study we used the monoclonal antibody PSMA (clone SP29), EnVision system, DAB. It was assessed the diffuse or heterogeneous character (focal, regional) of the reaction, the pattern and the intensity of staining. There were considered cases with high expression of PSMA (PSMA overexpression), carcinomas with diffuse intense, intense regional or moderate diffuse staining. The statistical analysis was performed using STATA 9.2 program.

**Results:** Only 1/59 (1.7 %) of the investigated carcinomas was devoid of immunoreactivity for PSMA, another being lost during processing. 32 out of the 58 PSMA–positive carcinomas (55.2 %) showed high expression of the marker and 26 (44.8 %) demonstrated low expression. Our results highlight the existence of some correlations between high expression of PSMA and metastatic (*p* = 0.018) and poorly differentiated (*p* = 0.012) prostate tumors.

**Conclusion:** PSMA is a sensible marker for the diagnosis of prostate carcinoma but it could be considered a marker of high-risk prostate tumors.

**PS-25-078**

**Diagnostic and prognostic significance of claudin expression in renal cell carcinomas**

S. Erdogan ^*^, B. Hasbay Bicen, G. Gonlusen, Z. Tansug

^*^Cukurova University, Pathology, Adana, Turkey

**Objective:** We aimed to search the diagnostic impact of claudins on renal cell carcinoma types and oncocytoma and correlate with grade, stage and prognosis.

**Method:** 134 cases of renal cell tumors consisted of 72 clear cell, 26 chromophobe, 24 papillary renal cell carcinoma and 12 oncocytoma cases were evaluated. Claudin 1, 3, 4, 7 and 8 were studied immunohistochemically.

**Results:** By Claudin 1; % 73,1 of chromophobe renal cell carcinoma cases were stained membranous and % 91,7 of the oncocytomas were stained cytoplasmically positive. By Claudin 3; % 81,9 of the clear cell renal cell carcinoma were negative, % 100 of oncocytomas were stained cytoplasmically positive. By Claudin 4; % 95,8 of clear cell renal cell carcinoma, % 75 of papillary renal cell carcinoma, % 34,6 of chromophobe renal cell carcinoma were stained negative, % 100 of the oncocytoma cases were cytoplasmically positive. By Claudin 7; % 84,6 of chromophobe renal cell carcinoma were stained membranous positive, % 91,7 of oncocytoma cases were cytoplasmically positive.

**Conclusion:** As a result, we found out that claudin 1, 7, 8 can be use at the differential diagnosis of oncocytoma and chromophobe renal cell carcinoma, on the other hand claudin 3, 4 can be useful to differentiate oncocytoma from eosinophilic variant of clear cell renal cell carcinoma.

**PS-25-079**

**MUC1 in lymph node metastases independently predicts survival in advanced prostate cancer patients**

V. Genitsch ^*^, I. Zlobec, G. Thalmann, A. Fleischmann

^*^University of Bern, Institute of Pathology, Switzerland

**Objective:** MUC1 is an anti-adhesion molecule and a potential risk factor in prostate cancer. However its prognostic value in metastasizing prostate cancer is unknown.

**Method:** MUC1 expression was evaluated on tissue microarrays constructed from 119 nodal positive prostate cancer patients treated by radical prostatectomy and extended lymphadenectomy. MUC1 status was correlated with various tumor features and biochemical recurrence-free (bRFS), disease-specific (DSS) and overall (OS) survival.

**Results:** In primary tumors, high MUC1 expression was significantly correlated with higher tumor volume (*p* = 0.005) and T-stage (*p* = 0.009). Furthermore high MUC1 expression in lymph node metastases corresponded with greater total size of metastases (*p* < 0.001) and total number of metastases (*p* = 0.014). High MUC1 expression in lymph node metastases predicted independently an unfavorable outcome compared to patients with low MUC1 expression (5-year bRFS *p* = < 0.023, DSS and OS *p* ≤ 0.001), whereas in primary tumors only a tendency towards an adverse survival could be shown.

**Conclusion:** MUC1 in either primary tumor or lymph node metastases correlates significantly with more unfavorable clinico-pathological features. Its independent prognostic information is inherent only in lymph node metastases from prostate cancer. This indicates an important role of additional tumor sampling from metastases.

**PS-25-080**

**Immunophenotypic features of multilocular cystic renal cell carcinoma**

D. Pasechnik ^*^

^*^Rostov Regional Hospital 2, Pathological Anatomy, Rostov On Don, Russia

**Objective:** Multilocular cystic renal cell carcinoma (MCRCC) is a rare subtype of clear cell renal cell carcinoma with a good prognosis included to 2004 WHO classification. Microscopically, the cysts are lined with tumor cells with clear to occasionally slightly eosinophilic cytoplasm. The nuclear grade is usually low. However, immunohistochemical staining feature of MCRCC have not been well researched.

**Method:** 16 MCRCC were examinated immunohistochemically using antibodies for CD10, renal cell carcinoma (RCC) antigen, cytokeratin 7 (CK7), E-cadherin, α-methylacyl-CoA-racemase (AMARC), epithelial membrane antigen (EMA), smooth muscle actin, vimentin, Ki67.

**Results:** All MCRCC stained for RCC, vimentin. Other markers demonstrated the following results, respectively: CD10 (75 %), CK7 (62,5 %), E-cadherin (44 %), AMARC (25 %), EMA (87,5 %). Expression of Ki67 was observed in single cells. Smooth muscle actin showed myofibroblastic cells within the septa of MCRCC, the capillary vascular network and tumor capsule.

**Conclusion:** Our data confirmed that MCRCC immunophenotype is similar to clear cell RCC, but part of tumors shows coexpression proximal (CD10,RCC antigen) and distal (CK7,E-cadherin) nephron markers. Low proliferative activity allows them to be considered as neoplasm of low malignant potential.

**PS-25-081**

**Major adverse reactions after intravesical Bacille Calmette-Guerin (BCG) immunotherapy**

A. Yagüe Hernando ^*^, R. Guarch Troyas, A. Echegoyen Silanes, J. I. López, C. B. Marta Casanova, K. S. García Guevara, D. García García

^*^Complejo Hospitalario Navarra, Surgical Pathology, Pamplona, Spain

**Objective:** Intravesical instillation of bacille Calmette-Guerin effectively treats superficial urothelial cell carcinoma of the bladder. However, major adverse reactions have been described in approximately 5 % of patients. These include for example: pneumonitis (0.7 %), granulomatous orchiepididymitis (0.4 %), or renal abscess (0.1 %). We describe three cases in which intravesical infusion of BCG was followed by disseminated infection.

**Method:** Three patients aged 55, 61 and 64 years old underwent intravesical instillations of BCG for the treatment of biopsy proven superficial urothelial carcinoma. None of them received more than three instillations.

**Results:** Within 3 to 5 months after the first infusion, generalized symptoms such as fever, hemoptysis, testicular mass or several radiological granulomatous lesions in renal parenchyma appeared respectively in each patient. Bronchoscopic biopsy revealed interstitial pneumonitis with noncaseating micro granulomas. In the orchiectomy specimen, caseating granulomas with a polymorphous inflammatory infiltrate were found. The patients had negative tissue cultures for M. bovis and responded to antituberculous drugs.

**Conclusion:** After intravesical BCG immunotherapy in patients with superficial urothelial carcinoma, major adverse reactions may appear probably related with intravascular spread of the bacilli following urologic trauma, or with type IV hypersensitivity (cell mediated). Antituberculous treatment is required when generalized infection is suspected, adding glucocorticosteroids when features of hypersensitivity predominate.

**PS-25-082**

**Carcinosarcoma of the prostate: A case report**

B. Chelly ^*^, A. Zehani, I. Chelly, H. Azzouz, H. Nfoussi, K. Bellil, S. Haouet, N. Kchir

^*^Tunis, Tunisia

**Objective:** Carcinosarcoma of the prostate is an unusual malignancy characterized by adenocarcinoma intimately admixed with sarcoma. Our aim is to report a new case of this lesion and to describe its histological and immunohistochemical features.

**Method:** We report a case of carcinosarcoma of the prostate diagnosed in our Department of pathology, Rabta hospital, Tunis, Tunisia.

**Results:** A 72-year-old male presented with rapidly progressive urinary obstruction and pelvic pain 8 months after transurethral resection without histological examination. On physical examination, the patient had a rectal mass and hypogastric sensitivity. We decided a second transurethral resection and microscopic finding consisted of a prostate tumor showing predominantly high-grade spindle cell neoplasm with a small focus of adenocarcinoma Immunohistochemical staining revealed cytoplasmic reactivity for keratin in the adenocarcinoma component. The sarcoma component was positive for vimentin and actin.

**Conclusion:** Carcinosarcoma of the prostate is an aggressive malignancy, regardless of histologic type.

**PS-25-083**

**Ureteritis cystica presenting as a single polypoid mass: A case report**

M. Sollai^*^, R. Santi, N. Stomaci, M. Colecchia, G. Nesi

^*^University of Florence, Division of Pathology, Italy

**Objective:** Ureteritis cystica (UC) is a rare, benign lesion characterized by cystic transformation of von Brunn’s nests with multiple cystic spaces containing eosinophilic material and delineated by normal urothelium. These changes are common in patients with stone disease. Although usually microscopical in size, UC may rarely produce grossly visible fluid-filled cysts that elevate the urothelium.

**Method:** A 40-year-old woman underwent left nephroureterectomy for a 5 × 3.2 cm polypoid mass of the ureter that caused hydronephrosis.

**Results:** Histologically, the lesion consisted of cystic structures lined by benign-appearing urothelium with intact umbrella cells. Necrosis and significant mitotic activity were absent. These findings were consistent with UC. Renal parenchyma showed nephron atrophy, interstitial fibrosis and a dense chronic inflammatory infiltrate.

**Conclusion:** Obstructive UC may present as a large polypoid mass and cause chronic pyelonephritis with hydronephrosis. Therefore, UC should be kept in mind in the differential diagnosis of a ureteral mass even when exceeding several centimetres in diameter.

**PS-25-084**

**Signet ring adenocarcinoma of prostate: Two cases report**

E. Tastekin ^*^, O. Inci, N. Can, M. Azatcam, T. Ciftci, K. Özdedeli

^*^Trakya University, Pathology, Edirne, Turkey

**Objective:** Signet ring cell carcinoma (SRCC) of the prostate is rare. Tumoral cells are morphologically vacuolated and they may be present as singly or sheets. Their mucinous luminal secretions are generally mucicarmin negative. SRCC requires that 25 % or more tumors be composed of SRC.(some authors require 50 %).

**Method:** CASE-1: A 82 years old patient was admitted to urology department with hematuria and dysuria. On physical examination prostate gland was grown up, rigid-fixed (volume:80cc, total PSA:80,5 ng/ml. CASE-2: A 79 years old patient was admitted to urology department with hematuria. On physical examination prostate gland was grown up, rigid-fixed (volume: 43 cc, total PSA: 17 ng/ml) Ten quadrant needle biopsies were performed to both of two patients. Microscopically all biopsies were infiltrated by tumor in proportions between 60 and 100 %. Tumors which were seen in both biopsies had similar morphology. Tumor was infiltrated diffusely through the stroma. Tumor cells (more than %60) were shown distinctive nuclear displacement by clear cytoplasm (other areas Gleason pattern-4). SRCs had mucicarmine, PAS-AB negative luminal secrets. Immunohistochemically PSA, pankeratin and CEA were positive, CK7, CK20 and CDX2 were negative. We diagnosed these tumors as “Signet ring cell carcinoma”.

**Results:** Signet ring cell carcinoma is a rare entity for prostate gland. Thus, differential diagnosis from metastatic carcinomas is important.

**PS-25-085**

**Reactive stromal grading in needle biopsies predicts prostate cancer specific mortality**

U. Axcrona ^*^, L. Vlatkovic, T. Sæter, G. Waaler, E. Servoll, J. M. Nesland

^*^Dept. of Pathology, The Norwegian Radium Hospital, Oslo, Norway

**Objective:** Reactive tumour stroma is demonstrated to influence prostate cancer (PCa) tumourigenesis and progression. The correlation of reactive stromal grading (RSG) in diagnostic needle biopsies to clinicopathologic parameters and PCa specific mortality was investigated.

**Method:** A population-based cohort of 276 patients (1991 to 1999) with clinically localized PCa diagnosed by needle biopsies at a Norwegian county hospital was analysed. Primary treatments included prostatectomy (*n* = 38), radiotherapy (*n* = 34), watchful waiting (*n* = 104), hormone therapy (*n* = 97) and others (*n* = 3). RSG was evaluated semi-quantitatively on the basis of previously described criteria (Yanagisawa et al.). For statistical analyses Spearman rank-order correlation coefficients, Kaplan-Meier methods and multivariate Cox proportional hazards regression were used.

**Results:** The median follow-up time was 107 months. Sixty patients died from PCa. RSG correlated with PSA, clinical stage and Gleason score. The 10-year PCa specific survival rate for RSG 0, 1, 2, and 3 was 95 %, 81 %, 64 % and 63 %, respectively (*p*-value < 0.005). RSG remained an independent predictor of PCa specific mortality when adjusting for PSA, clinical stage, Gleason score and treatment modality.

**Conclusion:** Our study demonstrates RSG as an independent predictor of PCa specific mortality in needle biopsies. Thus, emphasizing the importance of the stromal compartment in PCa development.

**PS-25-086**

**Mixed acinar adenocarcinoma and small cell neuroendocrine carcinoma of the prostate gland. Report of a case**

P. Lazari^*^, V. Samaras, I. Nikolopoulos, H. Poulias, C. Barbatis

^*^Athens General Hospital, Dept. of Cytopathology, Greece

**Objective:** Mixed acinar adenocarcinoma and small cell neuroendocrine carcinoma (MASCC) is rarely encountered in the prostate gland. Approximately 50 % of all cases of small cell neuroendocrine carcinomas found within the prostate represent a component of a MASCC. We describe a rare case of MASCC diagnosed in prostatic biopsies.

**Method:** A 76-year-old man presented to our hospital with increased PSA serum levels. Clinical examination suggested a prostatic carcinoma and transrectal ultrasound-guided core biopsy was performed. The formalin-fixed tissue was studied with hematoxylin/eosin and immunohistochemistry.

**Results:** Biopsies from the left lobe were infiltrated by typical acinar adenocarcinoma of Gleason score 7 (3+4) (Androgen Rec +, PSMA +) in continuity or adjacent to a small cell neuroendocrine carcinoma (Androgen Rec -, PSMA +/30 % of cells, Chromogranin +, CD56 +, TTF-1 +/focally, Ki67 +/45 % of cells). A final diagnosis of MASCC was made as there was no evidence of other primary site.

**Conclusion:** According to the current WHO classification, although there is no difference in prognosis between patients with pure small cell carcinoma and those with MASCC, the emergence of a small cell component within acinar adenocarcinoma usually indicates an aggressive clinical course. MASCC should be differentially diagnosed from a metastatic small cell neuroendocrine carcinoma, especially from the lung.

**PS-25-087**

**Role of inmunohistochemistry in prostate biopsy**

M. E. Guerra ^*^, Y. Arce, F. Algaba

^*^Hospital Central de Asturias, Dept. of Pathology, Oviedo, Asturias, Spain

**Objective:** To assess the contribution of the inmunohistochemistry (IHC) in the diagnose of prostate biopsies.

**Method:** Retrospective and descriptive study of 3120 prostate biopsies were carried out in the period 2008–2012. Inmunohistochemical stainings for 34βE12 and P504s were performed.

**Results:** In our series, 88.5 % of cases were diagnosed with H&E stain. In this group, 36.4 % (*n* = 1,137/3,120) and 1.5 % (*n* = 46/3,120) were diagnosed of adenocarcinoma (ADC) and atypical glands (ASAP), respectively. Cases of doubtful diagnosis which required IHC were 11.5 % out of total (*n* = 359/3,120). After IHC 62.4 % (*n* = 224/359) was diagnosed of adenocarcinoma, 14.5 % (*n* = 52/359) of prostate intraepithelial neoplasm (PIN), 17 % (*n* = 63/359) of atypical glands and 5.6 % (*n* = 20/359) of prostate without neoplasic changes.

**Conclusion:** In cases of uncertain diagnosis in prostate biopsy, inmunohistochemistry is a helpful tool, since it allows us to increase the adenocarcinoma diagnosis in 7.2 %. Despite the use of inmunohistochemistry, 3.5 % (*n* = 109/3,120) of the whole biopsies are diagnosed as atypical glands.

**Table 1.:**

**Table:**
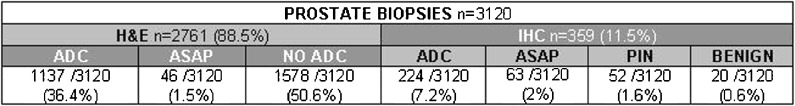

**PS-25-088**

**The minimal immunohistochemical diagnostic panel in a plasmacytoid urothelial bladder carcinoma**

A. Navarro ^*^, M. Aizpurua, R. Palhua, S. Landolfi, S. Ramón y Cajal, I. de Torres

^*^Vall d’Hebron Hospital, Pathology, Barcelona, Spain

**Objective:** The plasmacytoid urothelial carcinoma (PUC) is an aggressive and unusual variant of bladder carcinoma. Less than 50 cases have been described in the literature. The peculiar plasmacytoid-like or signet ring morphology with discohesive pattern makes difficult their diagnosis, which can be achieved using an appropriate inmunohistochemical panel. The purpose of this study was to define the minimum IHC profile for diagnosis of PUC.

**Method:** A serie of 7 pure and invasive PUC cases were identified from surgical pathology files of our Hospital. Immunohistochemical stains for AE1/AE3, CK7, EMA, CK20, 34BE12, CD138, CEA, E- Cadherin, p53, and К and λ light chains were performed in all cases.

**Results:** AE1/AE3, EMA and CK7 strong inmunoexpression was observed in 100 % of cases, whereas 85 % and 71 % showed CK20 and 34BE12 expression respectively. In all cases К and λ were negative. By contrast intense positivity for CD138 and CEA was showed in the 85 %. P53 nuclear expression was observed in 57 % but in only 27 % membranous E-Cadherin expression.

**Conclusion:** PUC can generally be suspected by careful morphologic evaluation. Immunohistochemistry is helpful for making a correct diagnosis. In our experience a minimum panel with CK7 and CD138 may be useful for the diagnosis of this unusual variant.

**PS-25-091**

**Spontaneous rupture of the bladder**

I.-D. Caruntu ^*^, A. Grigoras, B. Ioan, L. Knieling

^*^U.M.F., Morpho-functional Sciences, Iasi, Romania

**Objective:** We present a rare pathological condition, namely the spontaneous rupture of the bladder. The bladder rupture is usually related to urothelial tumors or pelvic radiotherapy. Since 1980, 177 cases have been reported in the mainstream, 25 of them directly related to alcohol abuse.

**Method:** A 65 aged woman was autopsied for clarifying the circumstances of her death at home, after the consumption of an important quantity of alcohol.

**Results:** The necropsy revealed, macroscopically, the rupture of the posterior wall of the bladder with haemoperitoneum; other associated lesions were cerebral edema, myocardial fibrosis, lung, kidneys and liver stasis and edema, pancreas and spleen with autolytic changes, and multiple ovarian cysts. The microscopic exam of the bladder showed hemorrhagic necrosis, with diffuse mucosal pattern, focally located in the muscular layer, and extended areas of hemorrhages in the entire wall.

**Conclusion:** The particularity of our case consists in the spontaneous character of the rupture, the traumatic, inflammatory, or tumoral lesions being excluded.

**PS-25-092**

**Malignant lymphoma of the testis: Clinical and pathological features–a case report**

L. Vasile ^*^, C. Simona

^*^University of Medicine, Histology-Citology, Timisoara, Romania

**Objective:** Primitive testicular lymphomas are very rare neoplasia between non-Hodgkin’s lymphomas, but seen autopticaly.

**Method:** We report a case of testis lymphoma of a 64-year-old man. Clinical course was asymptomatic for 1 year.

**Results:** The patient underwent surgery for a testicular tumor 8/8/5, 5 cm with infiltration of the spermatic cord and testicular vaginalis. In histologic investigation, a monotonous, monomorphic tumor cell proliferation was seen, also numerous mitotic figures and areas of necrosis in a conjunctive-vascular stroma. The first diagnosis was seminoma. A few days after the operation the patient dies. Autopsy was performed. To identify a retroperitoneal tumoral mass of 25/25 cm, infiltrating the ascending colon, also massive retroperitoneal lymphadenopathy. We found nonspecific lesions in organs and neoplastic cachexia. The Batson veins, a network of valveless veins can provide a route for the spread of testis metastases in our case.

**Conclusion:** The final diagnosis of testis lymphoma was made by histologic and immunohistochemical examination.

**PS-25-093**

**Giant retroperitoneal mature teratoma in an adult coexisting with classic seminoma of the testis: A case report**

R. Sampaio ^*^, J. Palla Garcia, R. Dias, A. Duarte

^*^Oporto’s Hospital Centre, Dept. of Surgical Pathology, Porto, Portugal

**Objective:** The origin of the extragonadal retroperitoneal germ cell tumors remains controversial whether they developed primarily in the retroperitoneum or whether they are metastases of a primary testicular tumor. Retroperitoneal teratomas often occur in infancy and childhood but they are a rare entity in adults.

**Method:** We present a case of a 23 years-old male patient, with accidental detection of an abdominal lesion, in the context of a gastrointestinal disorder study. The abdominal CT-scan revealed a bulky abdominal cystic lesion with 17 cm in its long axis, being proposed the diagnosis of a cystic lymphangioma. Resection of retroperitoneal lesion was performed.

**Results:** Grossly, it was a multi-loculated cyst tumor, 22 cm of greater dimension, locus of smooth lining and containing serous yellowish fluid and whitish pasty material. Histologically showed to be mature cystic teratoma. In this context, a testicular ultrasound was advised and a solid nodular hypoechoic lesion, measuring 1,3 cm was found. Histopathologic examination revealed a pure classic seminoma, and so the coexistence of two germinal tumors, seminomatous and nonseminomatous.

**Conclusion:** This case illustrates the importance of excluding a gonadal germ cell tumor when a extragonadal retroperitoneal germ cell tumor is found, a fact that is sometimes ignored by clinicians.

**PS-25-094**

**Nested variant of urothelial carcinoma: Report of 2 cases**

N. Cerda ^*^, A. Corominas-Cishek, G. Muñiz, V. Caamaño, A. Pérez, M. González, J. I. López

^*^Cruces University Hospital, Anatomia Patologica, Barakaldo, Spain

**Objective:** Nested urothelial carcinoma is characterized by highly aggressive behavior despite its bland histologic features. This variant is frequently underrecognized, estimated incidence is less than 0.3 % of invasive bladder tumors. We add two more cases to the literature.

**Method:** Case 1: 53 year-old man with smoking history and recent asyntomatic macroscopic hematuria. Sonography showed an exofitic bladder lesion. Patient underwent transurethral resection of the bladder tumor (TURBT), reporting invasive urothelial carcinoma (pT2). Two months later radical cistoprostatectomy (pT3b, pN1), radiotherapy and chemotherapy was perfomed. One year later patient died due to advanced metastatic disease. Case 2: 83 year-old woman with asyntomatic macroscopic hematuria who underwent TURBT, reporting invasive urothelial carcinoma (pT2). Surgical procedure was not done due to the advance age. Patient died 3 years later.

**Results:** Both tumors showed similar histologic features. Neoplastic cells were grouped in confluent smalls nests and abortive tubules composed of urothelial cells with mild nuclear atypia infiltrating deeply the wall bladder. In case 1, perivesical tissue, urethra and one lymph node were invaded. Immunohistochemistry profile demonstrated positivity for CK7, CK20, 34BE12 and P63.

**Conclusion:** It is importante to keep in mind this entity, specifically in superficial samples, to the correct recognition and distinction from benign mimics.

**PS-25-095**

**PDE5 inhibitors blunt inflammation in Benign Prostatic Hyperplasia (BPH): A potential mechanism of action for PDE5 inhibitors in Low Urinary Tract Symptoms (LUTS)**

R. Santi ^*^, L. Vignozzi, M. Gacci, M. Pepi, G. Baroni, M. Carini, M. Maggi, G. Nesi

^*^University of Florence, Pathology, Italy

**Objective:** Metabolic syndrome (MetS) and benign prostate hyperplasia (BPH)/low urinary tract symptoms (LUTS) are often comorbid. Chronic inflammation is one of the putative links between these diseases. Phosphodiesterases type 5 inhibitors (PDE5i) are recognized as an effective treatment of BPH-related LUTS. One proposed mechanism of action of PDE5 is the inhibition of intraprostatic inflammation. In this study we investigated whether PDE5i could blunt inflammation in the human prostate.

**Method:** We evaluated the effects of PDE5i on the secretion of IL-8 by human myofibroblast prostatic cells (hBPH) under different inflammatory stimuli. A clinicopathological study on 44 BPH patients treated with either vardenafil (10 mg/day, for 12 weeks) or placebo was also carried out.

**Results:** PDE5i reduced IL-8 secretion by hBPH in vitro. In BPH specimens, prostate inflammation showed a positive correlation with MetS severity (*p* = 0.002). In MetS patients, the intensity of the inflammatory infiltrate was lower in the vardenafil-arm than in the placebo-arm (*p* = 0.037).

**Conclusion:** Our data provide new insights into the mechanism of PDE5i action in alleviating LUTS in MetS patients. Histological assessment of inflammation may support clinical management of patients with MetS and BPH/LUTS.

**PS-25-096**

**Testicular lymphoma: A case report**

E. Tastekin ^*^, D. Inci, N. Can, A. Arslan

^*^Trakya University, Pathology, Edirne, Turkey

**Objective:** The majority of primary lymphomas of the male genital tract arise in the testes. Testicular Lymphomas constitute 2 % of all testicular neoplasms, 2 % of all high grade lymphomas and 5 % of all extranodal lymphomas in men.

**Method:** CASE-A 32 years old man admitted to the urologist with testicular swelling and unilateral enlargement of the scrotum. Radiologically coronal T2-weighted MRI was shown hypointense lesion in testis. Radical orchiectomy was performed. Macroscopically 6 × 6 × 4 cm sized, cut surface homogenous, yellow-white and necrotic lesion were seen. Microscopically diffuse lymphocytic infiltrate were involved all areas. Lymphocytes were with large and vesicular nuclei. Interstitial fibrosis, tubular hyalinization and loss of tubules were seen. Lymphoma infiltration was involved to the rete testis and extratesticular tissues. Immunohistochemically lymphocytes were stained diffuse positive with LCA, CD20, bcl6 but not stained with pankeratin and germ cell tumor specific antibodies. We diagnosed this lesion as “Diffuse lymphoma infiltration with B immunphenotype” and suggested to the hematologic examination. Systemic physical examination, laboratory and radiologic images were not compatible with systemic lymphoma. All of these findings supported to the “primary testicular lymphoma”

**Results:** Testicular lymphomas must take into account on evolution of testicular lesions. In order to testicular lymphoma diagnosis firstly systemic lymphomas should rule out.

**PS-25-097**

**Epididymal metastasis of pancreatic adenocarcinoma as a mimicker of primary epididymal carcinoma**

D. Kankaya ^*^, C. Ersoz, O. Ozkayar, D. Baydar, A. Kirmizi

^*^Ankara University Medical School, Pathology, Turkey

**Objective:** Metastastic tumours involving the epididymis are rare and most often found in patients with disseminated disease. Here we present a case of epididymal metastasis of pancreatic carcinoma.

**Method:** A 48 year old male underwent bilateral orchiectomy due to the epididymal masses detected bilaterally. Microscopic examination of the consultation case revealed an adenocarcinoma infiltrating between epididymal ducts and paratesticular soft tissues bilaterally. There was no infiltration of testis parenchyma. Tumor cells arranged in cords, trabeculas and tubules, showed vesicular round nuclei with prominent nucleoli and eosinophilic cytoplasm. Mitotic activity was very high and extensive lymphovascular invasion was detected. Tumor cells showed pancytokeratin, cytokeratin 7, MOC31, BerEp4, carcinoembryonic antigen, CA19.9, membranous β- catenin positivity, whereas inhibin, calretinin, cytokeratin 5/6, D2-40, Wilms tumor 1, cytokeratin 20, estrogen receptor, CDX2, prostate specific antigen and prostatic acid phosphatase were negative. Meanwhile, computerized tomography revealed a lobulated contoured mass, 3 cm in long diameter, in the tail of pancreas, infiltrating superior mesenteric artery.

**Results:** It was reported as adenocarcinoma infiltrating epididymis and paratesticular structures. Due to the clinical features, bilaterality of the tumor and CA19.9 positivity, it was accepted as a metastatic tumor of pancreatic origin.

**Conclusion:** Although metastatic cancer to epididymis is rare, it should always be considered as a possibility.

**PS-25-098**

**Oncocytic papillary renal cell carcinoma with different morphological features in a young patient**

D. Kankaya ^*^, D. Baydar, C. Ersoz, S. Kiremitci, A. Sertcelik

^*^Ankara University Medical School, Pathology, Turkey

**Objective:** Oncocytic renal neoplasms, some of which have been recently described, constitute a group of tumors with overlapping histopathologic features and differential diagnosis of them may be extremely challenging. Here, we report the case of oncocytic papillary renal cell carcinoma with different morphological features.

**Method:** A 23- year old woman undergone right nephrectomy due to the inferior pole mass. Macroscopic examination revealed a well circumscribed, solid creamy nodule, 5,5 cm in long diameter and limited in the kidney. Microscopically, the tumor displayed extensive areas of solid, alveolar and tubuler pattern of oncocytic cells mimicking oncocytoma. Papillary growth pattern with very rare foamy histiocytes were detected focally and there were hyalin globules accompanying them in a few areas. Mitotic activity was very low but atypical mitosis in two areas were determined. Tumor showed focal CD10 and alpha-methylacyl-coenzyme A racemase (AMACR) reactivity, whereas TFE3, Cathepsin K, Cytokeratin 7 were negative and Ki-67 was 3 %.

**Results:** Tumor was mimicking oncocytoma substantially but due to the papillary growth pattern with foamy histiocytes and focal CD10, AMACR positivity, it was reported as oncocytic papillary renal cell carcinoma.

**Conclusion:** Young patient age, the presence of hyalin globules and focality of AMACR and CD10 positivity were exceptional features for oncocytic papillary renal cell carcinoma.

**PS-25-099**

**Mixed congenital mesoblastic nephroma: A case report**

N. Abid^*^, N. Gouiaa, A. Lobna, E. Sameh, S. Makni, T. Boudawara

^*^Habib Bourguiba University Hospital, Pathology, Sfax, Tunisia

**Objective:** Congenital mesoblastic nephroma comprises 2 % of paediatric renal tumours. The mixed form (classic and cellular) represents only 10 % of cases. We present a case of mixed mesoblastic nephroma that occur in a 6 months-old- female baby. our aim is to review the clinical, morphologic, immunohistochemical and prognosis of Congenital mesoblastic nephroma with mixed faetures.

**Method:** We describe a case of a 6 months-old women with a kidney tumor measuring 10 cm of diameter; serial sections stained with hematoylin-eosin were examined and immunohistochemical staining was perfomed for vimentin, desmin, smooth muscle actin and CD34.

**Results:** The histologic study showed a interlacing fascicles of spindle-cell proliferation identique to low -grade fibroblastic sarcoma with foci of high cellular density, high mitotic rate and little foci of necrosis. Immunohistochemistry, tumours have features of fibroblasts and myofibroblasts.

**Conclusion:** Mixed Congenital mesoblastic nephroma has features of both classic and cellular mesoblastic nephroma within the same tumour; adjuvant chemotherapy is usually recommended, related to the cellular and atypical tumour component. A correct sampling of Congenital mesoblastic nephroma is necessary and the detection of atypical component in the tumor is not associated with poorly prognosis if adequatly treated.

**PS-25-101**

**Willms tumor with rhabdomyomatous differentiation: Two cases**

G. Tasova Yilmaz ^*^, B. Akkaya, S. S. Tuncer, O. A. Kupesiz, M. Melikoglu

^*^Akdeniz University, School of Medicine, Dept. of Pathology, Antalya, Turkey

**Objective:** Most renal tumors in children are classic nephroblastomas but there are variant histology pattern that are associated with distinct morphologic, biologic and clinical features. Some tumors have unusual histological appearance, like rhabdomyomatous differentiation (RD). We present two unusual case of Wilms tumor (WT) with RD.

**Method:** They are 3.5 and 4-year-old females. The first patient underwent nephrectomy; the other received prenephrectomy chemotherapy with previous biopsy. Histopathological examination revealed a triphasic nephroblastoma.

**Results:** A 3.5-year-old female presented with hematuria and abdominal mass, underwent nephrectomy. She was first misdiagnosed as neuroblastoma and finally diagnosed WT after surgery. Microscopically, the tumor was almost exclusively of epithelial component and foci of immature rhabdoid elements were present. Other tumor was predominantly composed of mesenchymal tissue such as skeletal muscle.

**Conclusion:** WT is a mixed tumor, with many histological patterns. As therapy becomes more effective, it is important to determine which characteristics are associated with aggresiveness and nonresponsiveness to therapy. Fetal Rhabdomyomatous Nephroblastoma (FRN) is not responsive to therapy, should be kept in mind. The distinction may be difficult between WT and FRN. FRN is a monophasic mesenchymal variant, it must contain >%30 of fetal rhabdomyomatous tissue. So, our patients represent WT with RD.

**PS-25-104**

**Spontaneous bilateral renal rupture with massive abdominal bleeding in Dravet syndrome: Two exceptional circumstances not reported in the literature**

A. Hens Perez ^*^, M. Añón, M. Moreno, G. Muñoz, J. L. Andrey, D. Martinez

^*^Hospital Puerto Real, UG A Patológica Bahía de Cádiz, Puerto Real (cádiz), Spain

**Objective:** Bilateral spontaneous renal rupture is an exceptional event, usually associated with acquired cystic kidney disease, tumours, vasculitis, amyloidosis, transplantation or a few other illnes. Dravet syndrome is a rare genetic progressive childhood neurodevelopmental disorder characterized by severe epilepsy referred to as a sodium channelopathy, whose complications include poor regulation of body temperature and increased susceptibility to infection, without relationship with visceral abdominal rupture. The present case includes this unique coincidence which etiopathogenic link provides an interesting field of investigation.

**Method:** A 17-year-old patient with Dravet syndrome, who was admitted to our hospital with high fever and decreased consciousness, in the context of a urinary tract infection. She had a bad outcome, appearing severe abdominal pain and signs of bilateral renal bleeding, proceeding to nephrectomy of the most affected kidney.

**Results:** Surgical specimen consisted of a 12,5 cm very softened kidney, with numerous surface continuity solutions and abundant hematic material. Histological sections showed hemorrhagic necrosis, edema and signs of acute tubular damage, without other significant lesions. The patient was able to stabilize and overcome this illness event.

**Conclusion:** Hereby, we report the unusual asociation of two rare conditions, whose possible pathogenic mechanisms relating both conditions, are discussed.
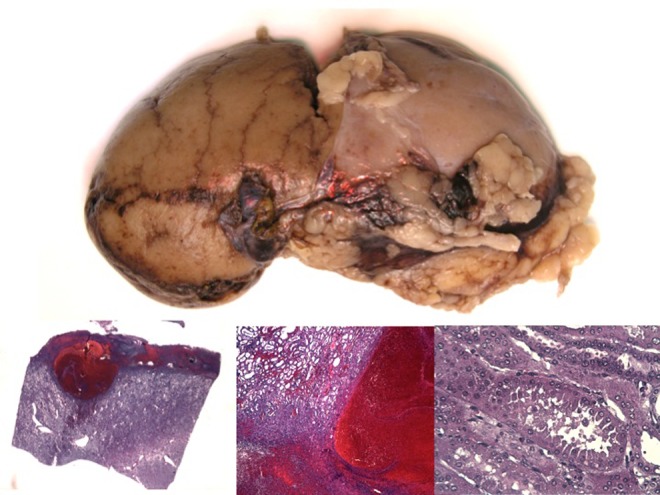


**PS-25-105**

**Primary intrarenal teratoma: Truly uncommon and easily dismissed**

C. Rivero Colmenarez ^*^, A. Berjón García, M. L. Picazo García, P. González-Peramato Gutierrez

^*^Hospital Universitario La Paz, Dept. de Anatomia Patologica, Madrid, Spain

**Objective:** We report a renal teratoma (RT) in an adult female as it is an extremely rare tumor of embryonal origin. It was first described in 1934, less than 30 cases have been reported since then, most of them in children.

**Method:** A 57-year-old female with an incidental right renal mass. The computarized tomography suggested RT. The patient underwent total nephrectomy with partial resection of the tumor with uneventful recovery.

**Results:** Gross examination showed a cystic mass of 21 cm, weighing 560 g. The surface was irregular and cut section revealed the presence of hair, teeth, jelly like material. The remaining kidney was unremarkable. Microscopic findings showed a cystic lesion partly lined by squamous epithelium, sebaceous glands and teeth. The renal parenchyma was normal, dysplastic features were absent.

**Conclusion:** Extragonadal germ cell tumors are predominantly located in the midline, retroperitoneum and mediastinum. The kidney is one of the least common sites of teratoma and other germs cell tumor location. Most of RT are dismissed as metastasis from a gonadal primary tumor, retroperitoned teratomas secondarily invading the kidney or Wilm’s tumor with teratoid features, which are the major differential diagnosis. However, RT must be considered as a diagnosis for abdominal masses.

**PS-25-106**

**Urachal tumour: Case report of an uncommon neoplasm**

R. Sampaio ^*^, J. Garcia, C. Peixoto

^*^Oporto’s Hospital Centre, Dept. of Surgical Pathology, Porto, Portugal

**Objective:** Urachal carcinoma has been estimated to comprise 0.17–0.34 % of all bladder cancers, it was first described in 1863 by Hue and Jacquin in a report translated, summarized by Sheldon and to date, fewer than 300 cases were reported in the literature.

**Method:** We present a case of 45-years-old caucasian woman without previous pathologic disease, with clinical complains of prolongued hematuria with 6 months of evolution. Surgical approach Pelvic exenteration was realized with anterior resection of the abdominal rectum and navel with pelvic lymphadenectomy and making ureteroileostomy of bricker.

**Results:** Pathologic Examination Macroscopy The complex surgical piece received, was composed by bladder, uterus, ovaries, navel, subcutaneous tissue and rectus abdominus muscle. Histology It was observed adenocarcinoma of intestinal type with poorly differentiated areas and areas of focal mucinous differentiation. Imunohistochemistry complementary study revealed cytokeratin 20 expression and absence of expression of cytokeratin 7 and 34bE12, which supports the hypothesis of adenocarcinoma originating from the urachus structures, in the absence of intestinal neoplasia.

**Conclusion:** The pathogenesis of urachal tumours is not fully understood. Surgery is the treatment of choice and the role of adjuvant treatment is not clearly defined. Pathologic factors valuable in predicting appear to be the tumour grade and the surgical margins status.

**PS-25-107**

**Endosalpingiosis in a leiomyoma of the pelvis “ƒ”: A case report**

T. Dzombeta ^*^, M. Ulamec, I. Grubisic, C. Bojan, B. Kruslin

^*^School of Medicine Zagreb, Dept. of Pathology, Croatia

**Objective:** Mullerianosis is a choristoma of embryonal origin, composed of normal appearing mullerian rests: endometrium, endosalpinx and endocervix. Pure endosalpingiosis is rare and is thought to result from metaplastic change of pluripotent peritoneal cells.

**Results:** Case report. A 59-year-old male was admitted to hospital due to history of dull pelvic pain accompanied by heaviness, pressure and bloating. The CT scan showed an intrapelvic lesion located between the bladder and rectum. Intraoperatively, a partly incapsulated, firm tumor, measuring 11 × 6 × 7 cm was received. The cut surface of the tumor was whorled, grey-white, with a central cavity. Histologically, it was composed of uniform smooth muscle bundles showing positive immunohistochemical reaction for smooth muscle actin and desmin, and a Ki67 measured proliferation index of up to 1 %. The central cavity contained dilated glandular formations lined by regular ciliated columnar epithelium showing positive reaction to CK7, estrogen and progesteron.

**Conclusion:** Tumors of retrovesical area usually represent direct invasion from malignancies arising in neighbouring organs, while primary ones, such as leiomyoma, are rare. The central glandular structures of endosalpingiosis found in this case, and the location of leiomyoma per se, demand a thorough investigation aiming to exclude more commonly found secondary neoplasms.

**PS-25-108**

**Small cell carcinoma of the urinary bladder: A clinicopathological study of 6 cases**

J. Pinto ^*^, T. Amaro, M. Honavar

^*^ULS Matosinhos, Dept. de Anatomia Patológica, Rio Tinto, Portugal

**Objective:** Small cell carcinoma (SCC) of the bladder is a rare, highly aggressive tumor, diagnosed frequently at an advanced stage. Six cases are described.

**Method:** Medical records and archived pathological studies of six patients with SCC of the urinary bladder, diagnosed between 2001 and 2012.

**Results:** Five males and one female, median age 81.5 years (80–96), presented with gross hematuria (*n* = 5/6) and a history of cigarette smoking (*n* = 4/6). The tumors were composed of small, uniform cells, with nuclear molding, scant cytoplasm and nuclei containing finely stippled chromatin and inconspicuous nucleoli. Mitoses were present and necrosis common. Two cases had areas of squamous differentiation. All cases were immunoreactive for at least one neuroendocrine marker. Four patients (pure or mixed) underwent transurethral resection and died of bladder cancer within 3.5 months. Both patients treated with radical cystoprostatectomy are alive, at 16 and 19 months of follow-up.

**Conclusion:** SCC of the bladder occurs more often in elderly males. Differential diagnoses include poorly differentiated urothelial carcinoma, lymphoma and sarcoma. The presence of any SCC within other urothelial carcinomas warrants the use of this diagnosis. Recognition of this rare entity should enable better management

**PS-25-109**

**Expression of metalloproteinase 2 and 9 in prostate carcinoma at the positive margin of radical prostatectomy specimens**

I. Hadzisejdic ^*^, G. Djordjevic, R. Oguic, V. Mozetic

^*^School of Medicine Rijeka, Dept. of Pathology, Croatia

**Objective:** The aim of this study was to evaluate and compare expression of MMP-2 and MMP-9 in prostate cancer in the main tumour mass and the tumour at the positive margin as well as the influence of these biomarkers on the biochemical recurrence of the disease in the prostatectomy patients.

**Method:** Tissue microarrays (TMA) of 120 archival, formalin fixed paraffin embedded prostate carcinoma obtained from patients treated by radical prostatectomy were immunohistochemically evaluated for MMP-2 and MMP-9 expression.

**Results:** MMP-2 showed statistically stronger expression in tumour cells at the positive margins than in the main tumour mass (*p* = 0.0301). Tumours with positive surgical margins showed statistically significant higher overall expression of MMP-9, comparing to a tumours with negative resection margins (*p* = 0.0121). MMP-9 expression was significantly elevated in tumours of patients who had biochemical recurrence (*p* = 0.0207). In group of patients with negative margins, increased MMP-9 expression above the cut-off value was significant for the reccurence (*p* = 0.065) while MMP-2 was not. Uni and multivariate analysis showed that MMP-9 is a good predictor of biochemical recurrence (OR = 8.75; *p* = 0.0078 and OR = 10.29; *p* = 0.0052).

**Conclusion:** These results indicate potential value of MMP-2 and MMP-9 in predicting invasive behaviour of tumours with positive as well as negative surgical margins.

**PS-25-110**

**Aquaporin 1 expression in fetal, normal and tumor renal tissues**

S. Cirovic ^*^, J. Vjestica, R. Jankovic, I. Simic, C. Müller, D. Djordjevic, J. Markovic-Lipkovski

^*^Institute of Pathology, Dept. of Nephropathology, Belgrade, Serbia

**Objective:** Aquaporin 1 (AQP1) is a water channel expressed in different epithelial tissues. It is also considered as a differentiation marker for proximal renal tubular cells, from which clear cell and papillary renal cell carcinoma (RCC) originate. We therefore studied AQP1 expression in various renal tissues.

**Method:** AQP1 expression in fetal, normal and RCC tissue was analyzed by immunofluorescence and immunohistochemistry. Additionally, the expression of neural cell adhesion molecule (NCAM) as renal progenitor and mesenchymal cell marker was determined.

**Results:** Fetal tissue at 22 week of gestation (wg) expressed AQP1 only in ureteric bud, but not in nephron precursors, whereas at 28 wg the expression of AQP1 was detected in early epithelial cells. Normal renal tissue showed AQP1 expression in glomeruli and proximal tubuli. Observing different tumor tissues, the expression of AQP1 was detected in clear cell and papillary, but not in chromophobe RCC or benign renal tumors. NCAM+ RCC cells did not display AQP1 positivity.

**Conclusion:** Fetal tissue in early gestation weeks does not express AQP1 in nephron progenitor cells. AQP1 shows RCC subtype-specific expression and could be useful diagnostic marker for clear cell and papillary RCC. The absence of NCAM and APQ1 coexpression suggests that NCAM+ and AQP1+ RCC cells belong to various cancer cell niches.

